# The impact of pension insurance types on the health of older adults in China: a study based on the 2018 CHARLS data

**DOI:** 10.3389/fpubh.2023.1180024

**Published:** 2023-06-02

**Authors:** Dongliang Yang, Zhichao Ren, Ge Zheng

**Affiliations:** ^1^Northeast Asian Research Center, Jilin University, Changchun, China; ^2^Department of Regional Economics, School of Northeast Asian, Jilin University, Changchun, China

**Keywords:** type of pension insurance, physical health, mental health, material consumption, non-material consumption

## Abstract

**Introduction:**

Pension insurance is an essential safeguard for the quality of life and health of older adults because it provides a stable and dependable source of income after retirement. China has constructed a multi-level social security system to accommodate the diverse needs of older adults, and offers various levels of pension insurance to maximize their interests.

**Methods:**

This study uses propensity score matching and ordinary least squares techniques to analyze 7,359 data from the 2018 China Health and Retirement Longitudinal Study (CHARLS) in order to explore the relationship between different pension insurance categories and the health of older individuals.

**Results:**

The research findings reveal that advanced insurances greatly benefit the health of older adults more than basic pension insurances, and the findings pass the robustness test. In addition, the effect was found to be heterogeneous, depending on the location of retirement and the marital status of older adults.Our findings suggest that both material and non-material consumption may be potential mechanisms by which pension insurance affects the health of older adults, providing new evidence for the causal mechanism between pension insurance and the health of older adults.

**Discussion:**

This study expands the scope of research on the health effects of pension insurance by covering a large representative sample across the country. The results show the important impact of the level of pension insurance on the health of older adults and can contribute to the development of social policies to promote the physical and mental health of older adults.

## 1. Introduction

Health issues are becoming more prevalent as the population of older adults grows. According to United Nations figures, the world’s aging population exceeded 1.04 billion in 2020 and is projected to reach 2.08 billion by 2050, with the share of older individuals increasing from 13.47 to 21.33% (1). Due to the one-child policy, a significant increase in life expectancy, and a drop in fertility, China’s population is aging disproportionately quickly. The number of older adults will increase from 249.776 million in 2020 to 485.489 million in 2050, a rise from 17.35 to 34.62%. China’s aged population is growing faster than Asia’s, which is projected to increase from 13.07% in 2020 to 24.40% in 2050. This will become a significant practical issue for China’s future economic and social development.

The probability of people developing age-related diseases (e.g., chronic diseases and dementia) continues to increase with age due to changes in physical and mental status, while the burden associated with these diseases also increases (2–4). For instance, according to data from the National Health Commission, there were over 190 million older adults in China with chronic diseases, and the number of dementia sufferers reached 15 million in 2021 (5). Because of global aging, the frequency of mental illness among older people has been rising in addition to physical sickness (6). Mental diseases have been identified as a leading cause of nonfatal health loss worldwide. According to the most recent statistics, the prevalence of depression and anxiety disorders in China was 2.1 and 4.98%, respectively (7). In particular, the COVID-19 pandemic affected people’s mental health severely, with a new report from the World Health Organization (WHO) estimating that the global prevalence of anxiety and depression increased by more than 25% in 2020 (8). In China, the issue of “unhealthy longevity” among older adults has become significant, and it is critical to encourage healthy aging.

In response to the global trend of population aging and to relieve the pressure on pension systems, scholars have increased their special attention to older adults. Some researchers argue for an employment perspective (9), suggesting that policymakers should encourage older workers to work longer and motivate employers to prolong the working life of their employees. For example, Mottaz found that older employees adapt well to their job and obtain more intrinsic rewards from it (10). This is because older workers have an enjoyable attitude toward their work, and they can derive a sense of satisfaction and accomplishment from the job they perform (11). Axelrad and Yirmiyahu further showed that working hours, additional benefits, and implementation of new work methods have a positive impact on older workers’ job satisfaction (12). A longer career is a desirable outcome for seniors themselves and for the pension system. These conditions and characteristics can be used by policy makers to implement policies and work practices accordingly. Therefore, China’s 14th Five-Year Plan, which was formulated in 2020, explicitly mentions the implementation of a gradual delay in the statutory retirement age and requires that the delayed retirement policy be effectively implemented by 2025.

China has established a nationwide, full-coverage, sustainable, and multi-level social security system in order to ensure the quality of life and health of older adults. This system currently consists of several pillars: the Urban–Rural Resident Social Pension (URRSP), the Enterprise Employee Basic Pension (EEBP), the Enterprise (Occupational) Annuity Pension (EAP or OAP), the Government and Institution Pension (GIP), the Individual Savings Pension (ISP), and the Commercial Pension (13). China uses a mixed pay-as-you-go and fund accumulation method for its unified account system. The first pillar is pay-as-you-go (PAYG), which includes URRSP, EEBP, and GIP. The second pillar is an individual account funded by employee contributions for EAP, OAP, ISP, and CP. The URRSP consists of a basic residents’ pension scheme that includes jobless urban and rural people; these two programs are not funded by employer payments, but rather by the central and local governments (14). The EEBP, EAP, and OAP are pension systems formed by companies for their employees, in which both employers and employees contribute to the pension fund, comparable to the 401 K pension plan introduced in the United States. The GIP, sometimes referred to as the public servants’ pension program, is designed for government employees. ISP and CP means that the insured person receives a pension from a certain age after paying a certain amount of premiums, which can effectively address both investment risk and longevity risk.

**Figure 1:**
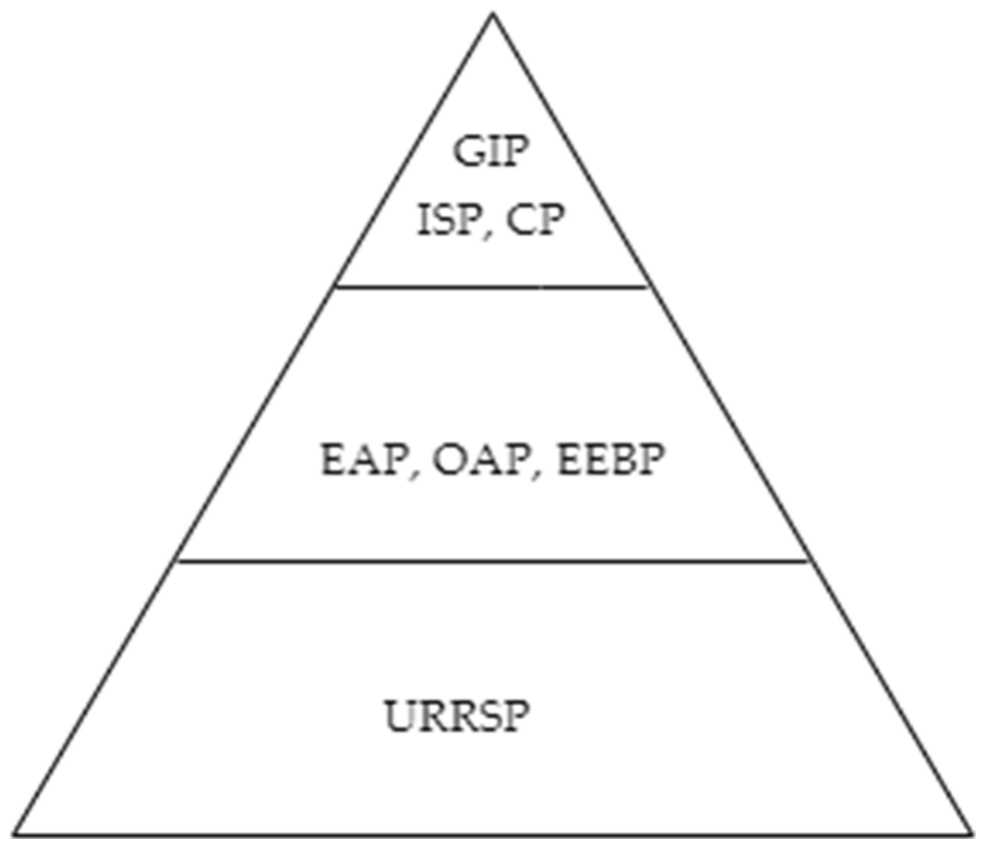
Pyramid-shaped pension system. GIP, Government and Institution Pension; ISP, Individual Savings Pension; CP, Commercial Pension; EAP, Enterprise Annuity Pension; OAP, Occupational Annuity Pension; EEBP, Enterprise Employee Basic Pension; URRSP, Urban–Rural Resident Social Pension.

In general, with the improvement of the level of pension insurance, the coverage and the degree of participation of the insured continue to improve. Zhu and Walker (15) describe the structure of China’s pension system as pyramidal. From the perspective of the insured’s income level, the pension benefits of the GIP are the most generous and are regarded as the top of the pyramid (16). In addition, the ISP and CP provide more flexible pension services and protect older adults living needs of the insured better; it is also regarded as the highest level of pension insurance. Employees covered by the EEBP and EAP (OAP) are in the middle of the pension benefit pyramid mentioned earlier (17). The URRSP forms the bottom of the pyramid. In a pyramid-shaped pension system like China (Figure 1), can advanced pension insurance better improve the health of older adults? Furthermore, what are the mechanisms behind the health effects of pension insurance? These remain crucial open questions that deserve further investigation (18).

This study aims to analyze the impact of several major types of pension plans. Using data from the China Health and Retirement Longitudinal Study (CHARLS), this paper divides pension insurance into basic pension insurance and advanced pension insurance, and then estimates the improvement effect of advanced pension insurance on the health of older adults. We focus on older adults in China for three reasons. First, there is a severe imbalance in China’s pension system. By the end of 2020, 542 million people were participating in the URRSP, and the accumulated fund was 975.9 billion yuan, which is obviously much weaker than the EEBP for employees with 456 million people insured and an accumulated fund of 4.83 trillion yuan. Therefore, clarifying the differences in the impact of different levels of pension insurance on health is crucial to alleviating the health problems of older adults. Second, the retirement location and residence status of older adults affect health, which will affect the impact of pension insurance on the health of older adults. Third, another reason that we focus on this cohort is that China is rapidly aging. Pension insurance will directly affect the daily life of older adults and will also affect the health of older adults through consumption channels, which is related to the process of aging and the quality of future development in China directly.

The novelty of this paper is four-fold. First, we gather and summarize the relevant research literature on the influence of pension insurance on the health of older adults. This implies that impact on the health of older adults varies widely by type of pension insurance. Secondly, it is different from studies that use a single indicator to measure the health of older adults. This paper selects multiple indicators from two aspects of physical and mental health to construct a comprehensive index that reflects the health of older adults. Third, the propensity score matching method is adopted to overcome the observational bias problem. The matched experimental group and the control group satisfy the balance assumption, to better estimate the causal effect of advanced pension insurance on the health of older adults. Finally, we address the potential mechanism of pension insurance affecting the health of older adults. An obvious mechanism is that pensions can improve the health of older adults through the material and non-material consumption. This provides new evidence for revealing the causal mechanism between pension and health.

Different types of pension insurance provide different levels of coverage and protection for older adults. Government-provided urban and rural pension insurance is inefficient relative to higher-rated commercial insurance, undermining the insureds’ incentive to invest in prevention, and in a situation of complete uncertainty, prudent decision makers were able to transfer these risks to an insurance company that is better capable of bearing them (19). Seniors will choose a higher level of pension insurance to protect their livelihood after retirement. The GIP, ISP, CP, and other types of pension insurance provide greater pension income than the URRSP, which has a direct impact on the health of the covered. The positive relationship between income and health has long been well documented (20–22). Hu et al. (23) employed data from the China Health and Retirement Longitudinal Study (CHARLS) and explored the role of pension on medical services for older adults. The study found that for every 100 yuan increase in monthly pension income, the preventive expenditure of older adults on medical services would increase by 8.72%; thus pension insurance helped older adults to maintain their health. Cheng et al. (24) noted that pension insurance supplied cash to covered older persons, which improved their nutritional intake, provided greater access to medical treatment, enhanced informal care, increased leisure activities, etc., and ultimately impacted older adults’ health. When pension income increases, the health of older adults continues to improve (25–28), indicating that advanced pension insurance has a more favorable influence on the health of older adults than basic pension insurance. Based on this deduction, we offer Hypothesis 1.

*Hypothesis 1 (H1)*: Advanced pension insurance can better improve the health of older adults.

One source of heterogeneity in the impact of pension insurance on health is the retirement location of older adults. A large body of evidence suggests that pension insurance’s effects on older adults’ health are heterogeneous across older adults’ retirement locations. Most of older adults have withdrawn from the labor market, and pensions have become their main source of income. However, due to the dual structure of urban and rural areas in China, there are significant differences in pensions between urban and rural areas. Lloyd-Sherlock et al. (29) studied the role of pension insurance in developing countries such as Ghana, Mexico and South Africa. They found that it did not significantly improve the health of older adults in rural areas because there were severe shortages of health education, health examinations and health services. Wang and Zheng (30) found that there were similar problems in rural China. The effect of pension income on improving the mental health of older adults in rural areas was limited, and there were noticeable urban and rural differences in the health of older adults. Abruquah et al. (31) found that although pension insurance improved the life satisfaction of older adults, urban older adults got higher benefits from it. Hence, Hypothesis 2 is proposed.

*Hypothesis 2 (H2)*: Urban older adults show a stronger response to advanced pension insurance compared to their rural counterparts.

The other source of heterogeneity is the residence status of older adults and their spouses. Almost every older adult will experience the loss of a spouse, which would cause a huge shock. Indeed, the loss of a spouse especially will lead to an increase in the probability of mental depression and worsening health status in older adults (32–35), longer lasting than other forms of marriage dissolution, such as divorce (36). Hughes and Waite (37) examined the effect of residence status on the physical health of older adults, and the results showed that widowed people had a worse health status than non-widowed people, and the longer the widowed time, the higher the probability of physical function limitation. Li et al. (38), based on the data of older adults in 1991 and 1994, found that widowhood increased the probability of depression in older adults in Wuhan. Zhang et al. (39) analyzed the effect of residence status on the cognitive status of older adults based on the CHARLS data in 2011 and 2013, and the results showed that being widowed for a long time led to a decline in memory ability in older adults. The appropriate residence status of older adults has an essential t impact on their health of older adults in their later years. Hence, Hypothesis 3 arises.

*Hypothesis 3 (H3)*: The health impact of advanced pension insurance on older adults living alone is weaker than that of older adults living with a spouse.

The implementation of pension insurance has changed the consumption pattern of older adults, which may be beneficial in improving the physical and mental health of older adults. Pensions improve older adults’ income and economic independence compared with traditional pension models supported by intergenerational economics (40, 41). Pensions relax older people’s budget constraints and change their consumption patterns, enabling them to buy and consume more nutrient-dense substances, which can affect older people’s physical health. For example, food is often associated with poor health, such as frailty, morbidity and mortality. In addition, with the increase in the time and amount of pension insurance participation, older adults reduce participation in physical labor, which has an adverse effect on health (42).

Among the few studies, Huang and Zhang (43) found that China’s new rural pension scheme significantly increased household income and food expenditure by 17.6 and 9.6%, respectively, and significantly reduced labor supply by 3.0 percentage points. In addition to material consumption, pension insurance improves the mental health of older adults by increasing non-material consumption expenditure. Cheng et al. (24) argued that pensioners respond to pension income in a variety of ways, such as with increased leisure activities, which serves as a channel for older adults to improve their health from pension income in turn. Zhang et al. (44) employed data from the China Health and Retirement Longitudinal Study. They found that pension income increased older adults’ non-material consumption such as fitness and beauty by 15.1% and improved their mental health. Based on the above analysis, we propose Hypothesis 4.

*Hypothesis 4a (H4a)*: Pension insurance improves the health of older adults through material consumption.

*Hypothesis 4a (H4b)*: Pension insurance improves the health of older adults through non-material consumption.

## 2. Data and variables

### 2.1. Data

The data comes from the 2018 China Health and Retirement Longitudinal Study (CHARLS). It is a national sample survey project hosted by the National Development Research Institute of Peking University. CHARLS data are collected every 2 years. The baseline survey conducted from 2011 to 2012 covered 28 provinces, 150 counties, and 450 villages (communities) in China, involving 17,708 people from 10,257 households. Three follow-up surveys were conducted in 2013, 2015, and 2018. The survey content mainly includes basic personal characteristics, health status and functions, pensions, household income and other information, which reflects the overall situation of middle-aged and older adults in China comprehensively (45). In order to ensure data completeness and validity, the number of individuals in the research sample obtained in this paper was7359.

### 2.2. Variable definitions

The dependent variable was the health of older adults. In this paper, all individuals over the age of 60 in the sample were used as the research object. Different from the practice of measuring the health status of older adults with a single indicator, following existing studies (46), this paper measured two aspects of physical and mental health. It introduced multiple indicators to measure the health of older adults. Specifically, we mainly used related indicators such as physical dysfunction and auxiliary help to measure physical health. Physical impairment is a relatively severe physiological health condition manifested in older adults’ inability to take care of themselves. Regarding the activities that older adults may encounter in their daily life, such as running, walking, bending over, bending the knees or squatting, etc., the CHARLS questionnaire covers the degree of difficulty encountered by the individual. The respondents answered, “I cannot do it,” “Yes, I have difficulty and need help,” “I have difficulty but can still do it,” or “No, I do not have any difficulty,” and the corresponding codes were1, 2, 3, and 4, respectively.

Mental health indicators include the frequency with which respondents act or feel the following 10 situations in the past week: (1) I am troubled by little things; (2) I have trouble concentrating when doing things; (3) I am depressed; (4) Everything is strenuous; (5) I am hopeful about the future; (6) I am afraid; (7) not sleeping well; (8) very pleasant; (9) feeling lonely; (10) feeling unable to go on with my life. Respondents used five scales to express the frequency of occurrence: “Most of the time (5–7 days),” “Sometimes or half of the time (3–4 days),” “Not much (1–2 days),” “Rarely or not at all (less than 1 day)”; these responses were assigned a value from 1 to 4, respectively. According to Dai and Gu (47), the Depression Scale designed by the Center for the Study of Depression Epidemiology (CES-D) has good reliability and validity in measuring mental health status. The depression scale design of the CHARLS questionnaire is based on the CES-D. To better reflect the health status of older adults, we used a comprehensive index to measure the health of the participants (48, 49). The higher the index score, the better the health status of older adults.

The independent variable was whether older adults participate in advanced pension insurance. The CHARLS questionnaire comprehensively investigates the participation of individuals in pension insurance: (1) the Urban–Rural Resident Social Pension (URRSP), (2) the Government and Institution Pension (GIP), (3) the Enterprise Employee Basic Pension (EEBP), (4) the Enterprise (Occupational) Annuity Pension (EAP or OAP), (5) the Individual Savings Pension (ISP) and the Commercial Pension (CP). According to the division standard of China’s pension insurance system and the existing literature, individuals who answered questions (2) to (7) in the affirmative were regarded as participating in high-level pension insurance and were regarded as the experimental group. Otherwise, those who answered question (1) in the affirmative were regarded as participating in the basic pension insurance individuals in the control group. We excluded individuals who did not participate in pension insurance and answered negatively to all questions. Finally, we obtained 5,125 individuals in the control group and 2,234 individuals in the experimental group.

The mediating variable was the material and spiritual entertainment consumption expenditure. Pension insurance increases the disposable income of older adults and reduces the uncertainty of the family’s future income and worries about retirement life, which improves survival consumption in areas such as lifestyle, and enables older adults to participate in more leisure and entertainment activities and obtain spiritual pleasure. The CHARLS questionnaire inquired about material consumption related to clothing, medical, health care and food. In addition, for the consumption of spiritual entertainment by older adults, we used “Long distance traveling expenses,” “Entertainment, including expenses for books, newspapers, VCCs, DVDs, cinema tickets and bars,” “Expenses for babysitters, housekeepers and servants” and other expenses in the questionnaire as the spiritual entertainment consumption of older adults.

The control variables were mainly the demographic characteristics of older adults. We tried to control the variables that jointly affected individuals’ choice of pension insurance types and health level and chose education level, residence status, retirement location, number of children, and family income level as control variables (see Table 1).

**Table 1:** Descriptive statistics of the key variables.

Variables	Definitions	N/Mean	%/Std
Health	General health status of older adults, including physical and mental health	−0.091	9.444
Physical health	Physical disability and assistance	0.116	4.934
Mental health	Mental state of older adults	−0.207	6.276
Type of pension	The value of participating in the advanced pension insurance is 1, and the value of participating in the basic pension insurance is 0	2,234	30.36
Age	Respondent’s age	68.108	6.223
Gender	1 for female, 0 for male	3,790	51.50
Education	1 for higher education, 0 for lower education	7,005	95.19
Residence status	The value of living alone is 1, and the value of living together is 0	6,067	82.44
Retirement location	The city value is 1, the rural value is 0	2,120	28.81
Family size	Number of people who usually live and eat together	3.112	1.862
Number of children	Respondent’s living children	0.019	0.216
Family income	The total household income of the respondents’ household members, such as wage income, agricultural income, livestock and aquatic product income, self-employment or starting a private enterprise, household public transfer payment income, etc. (in log)	8.792	1.808
Material consumption	The average monthly spending on health care, medical care, clothing and food of respondents’ households (in log)	6.821	1.196
Non-material consumption	The average monthly expenses of the respondents’ families in cultural entertainment, travel services, maid services, etc. (in log)	5.075	1.642

### 2.3. Descriptive statistics

We employed ordinary least squares to analyze pension insurance types on the health of older adults, as follows:


$$H_{i}=\alpha +\beta P_{i}+\gamma^{T}C_{i}+\varepsilon_{i}$$

$H_{i}$ represents the dependent variable (older adults’ health), where i refers to an older adult, $P_{i}$ represents the level of older adults participating in pension insurance, $C_{i}$ is a vector of observable determinants of older adults’ health, α is the constant term, ε_i_ is the disturbance term of the equation, ε_i_is not correlated with P_i_, and the estimated value of β parameter reflects the degree of impact of the pension insurance level on the health of older adults. γ is the vector of a parameter reflecting the other factors’ effect on older adults’ health.

**Figure 2:**
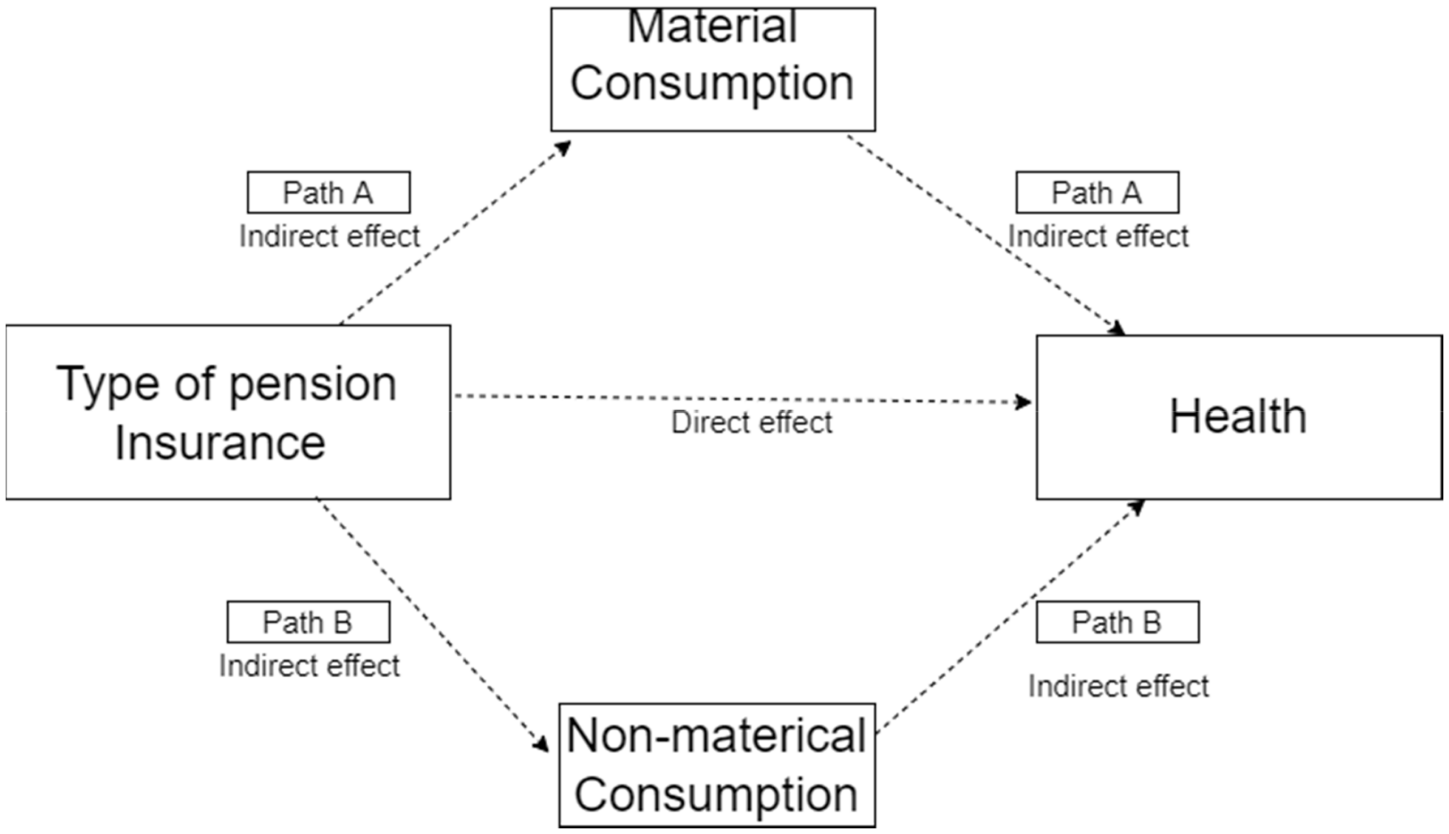
Framework of mediation effect analysis.

In addition, based on the mediation model to analyze the mechanism of pension affecting health (50, 51), we estimated the mediation effect by using two variables of material consumption and non-material consumption. Figure 2 represents the analysis framework. α is the constant term, εi is the disturbance term of the equation, εi is not correlated with Pi.

## 3. Results

### 3.1. Baseline regression results

When the multiple regression model is used to evaluate the causal relationship between the pension insurance level and the health of older adults, there may be a certain linear relationship between the explanatory variables, and the parameter β will not be effectively identified. Multicollinearity is generally diagnosed by variance inflation factor and tolerance, which are reciprocals of each other. Table 2 reports the VIF and tolerance of each variable. Since the maximum VIF is 1.24, less than the rule-of-thumb value of 10, it means that there is no severe collinearity problem between the explanatory variables.

**Table 2:** The variance inflation factor of each variable.

Variables	VIF	Tolerance
Type of pension	1.24	0.808
Gender	1.05	0.953
Age	1.13	0.894
Education	1.07	0.932
Retirement location	1.13	0.888
Pension location	1.19	0.839
Family size	1.12	0.889
Number of children	1.00	0.999
Family income	1.13	0.881

Table 3 shows the estimated results of the impact of pension insurance levels on the health of older adults. The dependent variables in columns (1), (2) and (3) are the general health status, physical health and mental health of older adults, respectively. In order to reduce the estimation bias caused by omitted variables, we control for the related factors such as personal characteristics and family characteristics of older adults. Furthermore, we chose the factors that can affect the health of older adults in the questionnaire as control variables to increase the explanatory power of older adults’ health as much as possible. However, besides other independent variables, the factors affecting the adjusted R-square also include systematic and random errors, which lead to the adjusted R-square appearing low in the OLS regression. As far as we are concerned, even if the adjusted R-square is small, the regression results are significant, which is acceptable. When controlling for a set of covariates in column (1), the OLS estimates show that the effect of pension insurance level on the overall health of older adults is 2.361 at the 1% level of significance. Column (2) shows that the effect of pension insurance level on the health of older adults is 0.972, which is slightly lower than the overall health level. Column (3) estimates that the impact of pension insurance level on the mental health of older adults is 1.389, which is higher than the impact of pension insurance on physical health. A possible reason for this result is that pension insurance can have a positive impact on the health by improving their economic conditions and further increasing their consumption expenditure on various aspects such as health care. The above estimation results show that the level of pension insurance affects the health of older adults significantly; that is, advanced pension insurance is more conducive to the health of older adults. Those results verify Hypothesis 1.

**Table 3:** OLS results of the effects of pension insurance types on the health of older adults.

Variables	Health	Physical Health	Mental Health
Type of pension	2.361^***^	0.972^***^	1.389^***^
	(0.318)	(0.163)	(0.223)
Gender	−4.194^***^	−2.040^***^	−2.154^***^
	(0.261)	(0.136)	(0.178)
Age	−0.178^***^	−0.173^***^	−0.004
	(0.024)	(0.013)	(0.016)
Education	1.587^**^	0.588	0.999^**^
	(0.694)	(0.361)	(0.475)
Residence status	1.699^***^	0.297	1.402^***^
	(0.384)	(0.199)	(0.262)
Retirement location	1.939^***^	0.673^***^	1.266^***^
	(0.345)	(0.175)	(0.245)
Family size	0.042	−0.005	0.047
	(0.068)	(0.035)	(0.048)
Number of children	−0.012	0.019	−0.032
	(0.607)	(0.279)	(0.383)
Family income	0.293^***^	0.142^***^	0.151^***^
	(0.073)	(0.036)	(0.051)
Constant	11.013^***^	12.393^***^	−1.380
	(2.055)	(1.097)	(1.384)
$R_{a}^{2}$	0.105	0.105	0.066

Robust standard errors in parentheses. ^***^ *p* < 0.01, ^**^ *p* < 0.05. $R_{a}^{2}$ means adjusted R-square. OLS represents ordinary least squares.

### 3.2. Endogeneity

Since the behavior of older adults participating in different types of insurance does not happen randomly, it is a rational decision to maximize utility based on its own characteristics. Even if the observable covariates are controlled, the traditional OLS method will not be able to obtain a consistent estimate of the parameters due to the existence of observation bias. To estimate the actual causal effect of advanced pension insurance on the health of older adults, drawing on the research of Rosenbaum and Rubin (52), we group the control group individuals with similar and well-matched characteristics of the experimental group by applying the propensity score matching (PSM) method to obtain the average causal effect of the experimental group.

**Figure 3:**
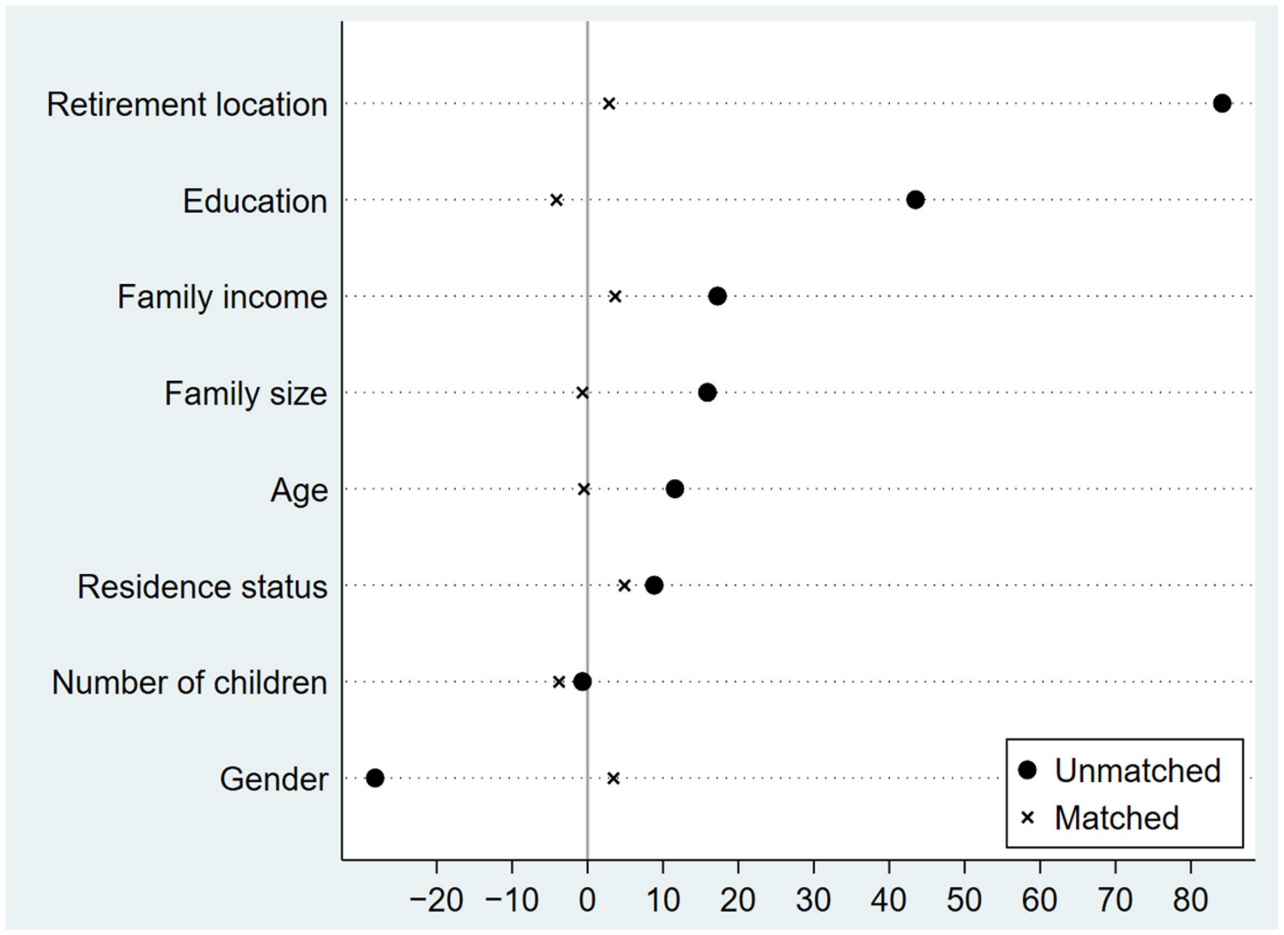
Standardized bias before and after matching.

The condition for propensity score matching requires that the covariates of the experimental and control groups are similar; that is, the experimental and control groups have similar structural characteristics. If the deviation of the two groups of covariates is too large, the propensity score matching method cannot be directly used for estimation. Figure 3 is a plot of the standardized deviation of covariates before and after matching. After matching, the standardized mean differences of covariates decrease significantly, and the standardized mean differences of each variable are concentrated within 5% of the deviation value and infinitely close to 0. Rosenbaum and Rubin (53) point out that the matching effect of the treatment group and the control group is acceptable when the standardized deviation of the covariates falls below 20 after matching. Figure 3 illustrates that propensity score matching achieves high balance and similarity of covariates between the experimental and control groups.

**Figure 4:**
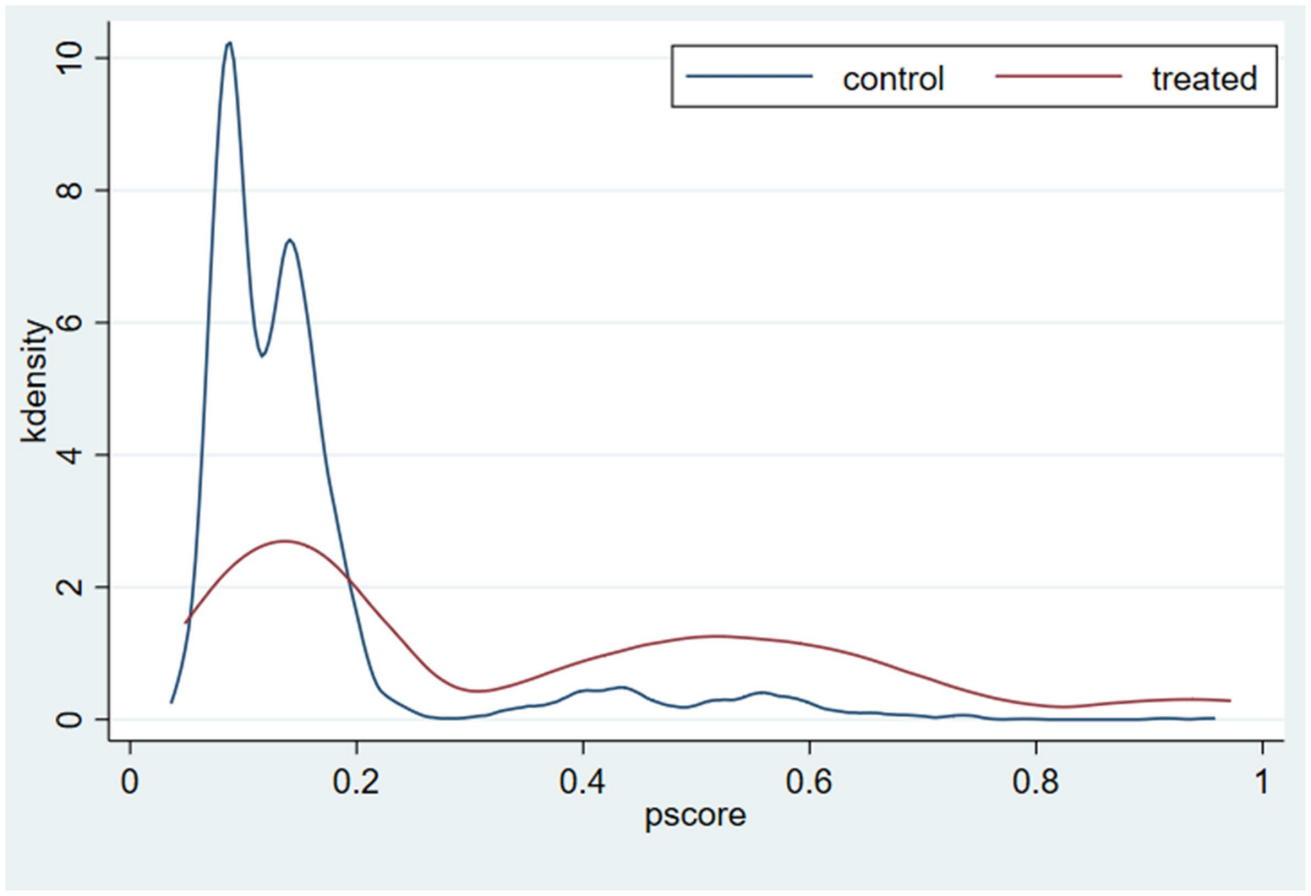
Density distribution of the propensity score.

Another important prerequisite for implementing matching is to meet the common interval requirement. This means that the value range of the individuals in the control group should cover the individuals in the experimental group; to ensure that the propensity scores of the two groups have the same value, so as to avoid unmatched situations. Figure 4 is the propensity index kernel density plot of the experimental group and the control group. The distribution of individual propensity indices in the control group and the experimental group overlapped, and the propensity index score range of the experimental group is included in the control group, which indicates that the matched two groups of individuals pass the common interval hypothesis test.

This paper presents different types of matching estimators to accurately identify the causal relationship between advanced pension insurance and health, including nearest neighbor matching (*k* = 4) (54), kernel matching, local linear regression matching, and Mahalanobis matching. It is worth noting that the k-nearest neighbor matching method involves the selection of k, which determines the number of individuals most like the matching object. We choose one-to-four nearest neighbor matching since one-to-four nearest neighbor matching minimizes the mean squared error. Additionally, c is the caliper set in the nearest neighbor matching process, which means searching for the individuals in the control group closest to the individuals in the experimental group within the range of the calipers.

Table 4 shows the estimated results of different matching strategies. Regardless of whether the dependent variable is the general health, physical health or mental health of older adults, the estimated value passes the 1% significance test, indicating that the level of pension insurance affects the health level of older adults significantly, and advanced pension insurance is more conducive to the health of older adults, which is consistent with the estimated results of OLS. The results show that the decision of older adults to participate in advanced pension insurance means that they have a good plan enter old age with a better mindset and pay more attention to daily health care.

**Table 4:** PSM analysis of the effects of pension insurance types on the health of older adults.

	Health	Physical Health	Mental Health
Nearest Neighbor Matching (*k* = 4)	2.647^***^	0.961^***^	1.686^***^
	(0.435)	(0.228)	(0.294)
Nearest Neighbor Matching (*k* = 4, c = 0.01)	2.749^***^	1.013^***^	1.736^***^
	(0.421)	(0.221)	(0.285)
Kernel matching	2.524^***^	0.948^***^	1.577^***^
	(0.406)	(0.212)	(0.273)
Local linear regression matching	2.533^***^	0.983^***^	1.550^***^
	(0.526)	(0.276)	(0.362)
Mahalanobis matching	2.413^***^	0.807^***^	1.605^***^
	(0.378)	(0.199)	(0.275)

Standard errors in parentheses. ^***^ *p* < 0.01. PSM represents propensity score matching.

The premise of the validity of propensity score matching estimation results is that the conditional independence assumption (CIA) or the conditional mean independence assumption (CMI) must be satisfied. Therefore, if the above estimation results have a causal effect explanation, the independence assumption needs to be further tested. Table 5 shows the estimated results of the independence test by the pseudo-intervention method. None of the estimated values passed the 1% significance test, and there was no significant difference in the health level between the “experimental group” and the “control group” of the pseudo-intervention, which further confirmed the significant effect of high-level pension insurance.

**Table 5:** Conditional independence assumptions test results for pseudo treatment.

	Health	Physical Health	Mental Health
Nearest Neighbor Matching (*k* = 4)	−0.448	−0.216	−0.232
	(0.325)	(0.171)	(0.217)
Nearest Neighbor Matching (*k* = 4, c = 0.01)	−0.450	−0.214	−0.236
	(0.325)	(0.170)	(0.217)
Kernel matching	−0.407	−0.192	−0.214
	(0.301)	(0.158)	(0.202)
Local linear regression matching	−0.366	−0.186	−0.179
	(0.386)	(0.204)	(0.257)
Mahalanobis matching	−0.323	−0.129	−0.192
	(0.317)	(0.166)	(0.213)

Standard errors in parentheses.

### 3.3. Robustness check

We conduct robustness checks to test the reliability of the impact of pension insurance types on health. In the first method, we use two-sided shrinkage treatment, which is a 5% two-sided abbreviated treatment of the explained variable. Table 6 shows that the level of pension insurance has a significant impact on the overall health, physical health and mental health of older adults, which are 2.213, 0.893 and 1.300, respectively, and pass the 1% significance test. The above estimation results show that the level of pension insurance affects the health level of older adults significantly; that is, advanced pension insurance is more conducive to the health of older adults, which is consistent with the results of the baseline regression.

**Table 6:** Two-sided shrinkage treatment.

Variables	Health	Physical Health	Mental Health
Type of pension	2.213^***^	0.893^***^	1.300^***^
	(0.298)	(0.143)	(0.214)
Control variable	YES	YES	YES
Constant	10.183^***^	10.962^***^	−1.102
	(1.882)	(0.943)	(1.299)
$R_{a}^{2}$	0.108	0.117	0.0652

Robust standard errors in parentheses. ^***^ *p* < 0.01. $R_{a}^{2}\;$ means adjusted *R*-square.

In the second robustness check method, we apply the ordinal logistic regression method to estimate the effect of the insurance level on older adults’ health. Ferraro and Farmer (55) believe that self-rated health is a comprehensive indicator that reflects an individual’s past and current health status compared to other health indicators. According to “How do you think your health is?” in the CHARLS questionnaire, the respondents’ options include “very bad,” “not good,” “average,” “good” and “very good,” and the corresponding numerical values are 1, 2, 3, 4 and 5; the higher the value, the better the health status. Table 7 shows that advanced pension insurance has a significant positive impact on health, with an estimated coefficient of 0.281. This paper calculates the probability changes of each explanatory variable to the five health states of the respondents, determining the marginal effect of the explanatory variable. This shows that the health of older adults who participate in advanced pension insurance will increase significantly, which is consistent with the results ich is consistent with the results.

**Table 7:** Ordinal logistic analysis of the effects of pension insurance types on the health of older adults.

Variables	Health	Very Bad	Not Good	Average	Good	Very Good
Type of pension	0.281^***^	−0.019^***^	−0.039^***^	0.013^***^	0.020^***^	0.026^***^
	(3.97)	(−3.91)	(−3.98)	(3.67)	(3.96)	(3.94)
Control variable	YES	YES	YES	YES	YES	YES

Standard errors in parentheses. ^***^ *p* < 0.01.

### 3.4. Heterogeneity

We further explore the heterogeneous effects of advanced pension insurance on health according to the differences in retirement locations and residence status. Specifically, the sample is split into two subsamples—urban and rural—by the respondent’s residential address. Columns (1) and (2) of Table 8 show the relevant estimation results. The impacts of advanced pension insurance on the health of older adults living in urban and rural areas are 3.421 and 1.941, respectively. The positive impact of advanced pension insurance on the health of urban older adults is stronger than that of rural older adults. Advanced pension insurance enhances the economic conditions of rural and urban older adults and enhances their sense of actual access, thus facilitating the function of pension insurance to protect the health status of rural and urban older adults. These results verify Hypothesis 2.

**Table 8:** Heterogeneous effects of pension locations and residence status.

Variables	Pension Location		Residence Status	
	Urban	Rural	Alone	Together
Type of pension	3.421^***^	1.941^***^	2.106***	3.843^***^
	(0.563)	(0.382)	(0.338)	(0.914)
Control variable	YES	YES	YES	YES
Constant	18.231^***^	9.690^***^	13.108^***^	12.267^***^
	(4.425)	(2.400)	(2.174)	(4.733)
$R_{a}^{2}$	0.0960	0.0950	0.0854	0.120

Robust standard errors in parentheses. ^***^ *p* < 0.01. $R_{a}^{2}\;$means adjusted *R*-square.

In addition, we divide the sample into two subsamples—living together and living alone—by the residence status of older adults and their spouses. Columns (3) and (4) of Table 8 show the results of the effect of pension insurance on the health of older adults under different living conditions. The causal effects of the two samples of living alone and living together are 2.106 and 3.843, respectively. The results show that the health impact of advanced pension insurance on older adults living alone is weaker than that of older adults living with a spouse. The possible reason is that pension insurance provides a stable and reliable financial source for older adults, improves their subjective welfare to a certain extent and improves the relationship between the family, which all contribute to their own mental health status, which has more direct implications for the choice of pension insurance for co-living older adults. These results verify Hypothesis 3.

### 3.5. Analysis of the mediating effect

To explore the mechanisms through which insurance level affect older adults’ health, two channels are studied in this section: material consumption and non-material consumption. We adopt the related expenditures on clothing, medical care, health care and food to measure the level of material consumption. Column (1) of Table 9 reports that the advanced pension insurance has a significant positive effect on the material consumption of older adults, which indicates that pension insurance has an impact on older adults’ health through its effects on material consumption. As the main source of income for older adults, pension insurance is a symbol of their social status. The higher the pension level, the higher the income of older adults, the more social services such as medical services older adults are willing to consume, the higher the quality of medical services they enjoy, and the healthier older adults will be. The results in Table 9 verify Hypothesis 4a.

**Table 9:** Material consumption and non-material consumption.

Variables	Material Consumption	Non-Material Consumption	Total Consumption	Health
Type of pension	0.398^***^	0.525^***^	0.478^***^	2.163^***^
	(0.039)	(0.132)	(0.032)	(0.328)
Total consumption				0.333^**^
				(0.150)
Control variable	YES	YES	YES	YES
Constant	5.407^***^	4.780^***^	5.539^***^	8.702^***^
	(0.246)	(0.908)	(0.204)	(2.222)
$R_{a}^{2}$	0.157	0.134	0.263	0.106

Robust standard errors in parentheses. ^***^ *p* < 0.01, ^**^ *p* < 0.05. $R_{a}^{2}$means adjusted *R*-square.

To test Hypothesis 4b, we take “Long distance traveling expenses,” “Entertainment, including expenses for books, newspapers, VCCs, DVDs, cinema tickets and bars,” “Expenses for babysitters, housekeepers and servants” and other expenses as the spiritual entertainment consumption of older adults in the questionnaire. Column (2) of Table 9 reports that the advanced pension insurance has a significant positive effect on the consumption of spiritual entertainment among older adults. This suggests that in addition to material consumption, pension insurance improves the mental health of older adults through increased spending on mental consumption. Older adults respond to the newly acquired pension income in a variety of ways, such as increased leisure activities, which is of great significance in increasing the mental health of older adults, including their sense of well-being. This indicates that pension insurance improves health through non-material consumption. The results in Table 9 verify Hypothesis 4b.

Finally, we use the total living expenses as a mediating variable to test the reliability of the estimated results. The results in columns (3) and (4) of Table 9 show that the advanced pension insurance affects the total consumption level; the coefficients are positive and significant statistically, and the total household consumption has a significant positive impact on the health of older adults, which indicates that advanced pension insurance improves health through their effects on consumption.

## 4. Discussion

A multi-level old-age insurance system has been built in China in response to the ongoing acceleration of aging, considerably ensuring the wellbeing of senior citizens. Although though a substantial body of research supports the usefulness of pension insurance, the majority of studies only compare the benefits of having pension insurance to those of not having it, omitting to include the differences between enrolling in advanced and basic pension insurance.

This study’s main goal is to find out how different types of pension insurance affect older people’s health in China. In order to assess the causal connection between pension insurance levels and older adults’ health, we first use a multiple regression model. The findings indicate that the level of pension insurance has a considerable impact on older persons’ physical and mental health, with more advanced pension insurance being better for their wellbeing. We use the PSM method to estimate the causal effect of pension type on the health effects of older adults in order to address the issue of endogeneity in the model. In addition, we test the robustness of the estimated results, which support the above conclusions. These results are consistent with previous studies (22). A recent study found a clear negative relationship between pension income and the risk of death in old age in the 1980s and 1990s, with mortality decreasing as pension income increased (56). These studies show that the rise in the level of pension insurance reflects the increasing scope and degree of protection available to insured persons, which means that the health of older adults will continue to improve.

Furthermore, our study shows that although the positive effect of pension insurance on the health of older adults has been recognized, the impact of pension insurance on health is heterogeneous, with urban older adults responding more favorably to advanced pension insurance than their rural counterparts. As well, the effects of pension insurance on health are different depending on living conditions. The health impact of advanced pension insurance on older adults living alone is weaker than that of older adults living with a spouse. These conclusions have more direct implications for the behavior of older adults in choosing their place of retirement and state of residence.

Finally, our findings provide new evidence for a causal mechanism between pension insurance and the health of older adults. Material consumption and non-material consumption are important mechanisms for pension insurance to affect the health of older adults. Studies on the health of older adults find that malnutrition is strongly associated with adverse health outcomes such as frailty, morbidity, cognitive impairment, depression and mortality (57, 58). Pension revenue relaxes budget constraints and leads to changes in food consumption by older adults. Moreover, the rise in pension income increases the likelihood of social and recreational activities, which are beneficial to the health of older adults (59).

In addition, this study expands the scope of research on the health effects of pension insurance by covering a large representative sample across the country. The results show the important impact of the level of pension insurance on the health of older adults and can contribute to the development of social policies to promote the physical and mental health of older adults.

However, there are some limitations in this study. First, the cross-sectional data used in this study cannot reflect the time-trend effect of pension insurance on the health of older adults. Second, the research objects of this paper were surviving respondents, and the deceased population was not considered; as a result, there may be a problem of sample selection bias, and the positive effect of advanced pension insurance is overestimated. Third, China’s pension insurance system is still in the development stage. There is a large time span between the choice of insurance and the acquisition of pensions, and the time and environment of respondents are different. It is necessary to overcome the influence of these objective factors and conduct more detailed and in-depth comparative studies or case studies.

Based on the conclusions, we propose the following policy recommendations. First, it is vital to raise the awareness of older adults to participate in advanced pension insurance. China should highlight the positive role of ISP, CP, EAP and OAP, promote the advantages of advanced pension insurance, and encourage eligible older adults to actively and reasonably enroll, i.e., convert some of the savings in their original plans into more health-friendly advanced pension insurance products. In addition, individuals should consider investment in human capital. Second, China should support the innovation of diversified pension insurance products and accelerate the launch of individual pension accounts. With the acceleration of aging, the ever-expanding demand for pension insurance products shows a diversified trend. It is necessary to learn from the development experience of the pension insurance market in developed countries to better meet the heterogeneous needs of older adults.

## 5. Conclusion

This study examines the causal effect of advanced pension insurance on the health of older adults using data from the 2018 China Health and Retirement Longitudinal Study. Compared to basic pension insurance, advanced pension insurance has a greater impact on the physical and mental health of older adults. The robustness test confirms the above conclusion. Further analysis of heterogeneous effects is conducted by splitting the sample into older adults’ retirement location and residence status. Urban older adults show a stronger response to advanced pension insurance compared to their rural counterparts. The health impact of advanced pension insurance on older adults living alone is weaker than that of older adults living with a spouse. Furthermore, we find that consumption is an important mechanism for pension insurance to affect the health of older adults, and pension insurance improves the health of older adults through material consumption and non-material consumption.

## Data availability statement

The original contributions presented in the study are included in the article/Supplementary material, further inquiries can be directed to the corresponding authors.

## Author contributions

DY conceived and designed the research, provided guidance throughout the entire research process, and responsible for all R&R works. ZR and GZ participated in data analysis, wrote and supplemented the English paper. GZ reviewed and edited the paper. All authors contributed to the article and approved the submitted version.

## Funding

The authors acknowledge funding support from the Project of Jilin Province Science and Technology Department (Grant number: 20230601017FG), Department of Education of Jilin Province (Grant number: JJKH20231095SK), Special Fund of Jilin University (Grant number: 2022JHJS08), Graduate Innovation Fund of Jilin University (Grant number: 2022019 & 2022158).

## Conflict of interest

The authors declare that the research was conducted in the absence of any commercial or financial relationships that could be construed as a potential conflict of interest.

## Publisher’s note

All claims expressed in this article are solely those of the authors and do not necessarily represent those of their affiliated organizations, or those of the publisher, the editors and the reviewers. Any product that may be evaluated in this article, or claim that may be made by its manufacturer, is not guaranteed or endorsed by the publisher.
