# Supplementary material for: The impact of pension insurance types on the health of older adults in China: a study based on the 2018 CHARLS data
Source: Front Public Health. 2023 Jun 2;11:1180024. doi: 10.3389/fpubh.2023.1180024 (PMC10272461; doi:10.3389/fpubh.2023.1180024)
Supplement: Supplementary file 1 [file Data_Sheet_1.ZIP › CHARLS_2018_Codebook.pdf]

China Health and Retirement Longitudinal Study

Wave 4 (2018)

CodeBook

VersionID: 20200914

Sep. 2020

National School of Development
Institute of Social Science Survey
Peking University

This page intentionally left blank

Contents

B	Demographic Backgrounds	1
C1	Family Information	17
C2	Family Transfer	259
D	Health Status and Functioning	389
DC	Cognition	513
DD	Insider	547
E	Health Care and Insurance	563
F	Work and Retirement	601
FN	Pension	647
G&H1	Individual Income	697
G&H2	Household Income	713
H&I	Housing	789
AUX1	Sample Information	967
AUX2	Sampling Weights	969

MODULE B

Demographic Backgrounds

ID: Individual ID

A String Variable	
Obs:	19,816

householdID: Household ID

A String Variable	
Obs:	19,816

communityID: Community ID

A String Variable	
Obs:	19,816

ba000_w2_3: Gender

	Freq.	%
1 Male	9,340	47.13
2 Female	10,476	52.87
Total	19,816	100.00

ba001: Chinese Zodiac

	Freq.	%
1 Rat	1,376	6.94
2 Ox	1,323	6.68
3 Tiger	1,648	8.32
4 Rabbit	1,799	9.08
5 Dragon	1,818	9.17
6 Snake	1,723	8.69
7 Horse	1,861	9.39
8 Goat	1,684	8.50

9 Monkey	1,745	8.81
10 Rooster	1,718	8.67
11 Dog	1,582	7.98
12 Pig	1,539	7.77
Total	19,816	100.00

ba004_w3: Date of Birth

	Freq.	%
1 Year Month Day	19,329	97.54
2 Not Have Hukou or ID Card	116	0.59
3 Refuse to Answer	371	1.87
Total	19,816	100.00

ba004_w3_1: Year of Birth

Mean	SD	Min	Max	Obs
1,956.26	10.33	1,900.00	2,000.00	19,494

ba004_w3_2: Month of Birth

Mean	SD	Min	Max	Obs
6.58	3.50	1.00	12.00	19,409

ba004_w3_3: Day of Birth

Mean	SD	Min	Max	Obs
14.55	8.54	1.00	31.00	19,392

ba005_w4: Actual Date of Birth and Recorded in Hukou or ID Card

	Freq.	%
1 Same	15,786	81.67
2 Different	3,543	18.33
Total	19,329	100.00

ba002_1: Actual Year of Birth

Mean	SD	Min	Max	Obs
1,956.37	10.93	1,900.00	2,018.00	4,030

ba002_2: Actual Month of Birth

Mean	SD	Min	Max	Obs
6.20	3.75	0.00	12.00	4,028

ba002_3: Actual Day of Birth

Mean	SD	Min	Max	Obs
13.73	9.13	0.00	31.00	4,030

ba003: Solar Calendar or Lunar Calendar

	Freq.	%
1 Solar Calendar	6,751	34.07
2 Lunar Calendar	13,065	65.93
Total	19,816	100.00

bb000_w3: Residential Address

	Freq.	%
1 Domestic	19,496	99.97
2 Abroad	5	0.03
Total	19,501	100.00

bb000_w3_1: Type of Residential Address

	Freq.	%
1 Family Housing	19,015	97.51
2 Nursing Home	30	0.15
3 Hospital	17	0.09
4 Other	439	2.25
Total	19,501	100.00

bb000_w3_2: Location of Residential Address

	Freq.	%
1 Central of City/Town	3,541	18.16
2 Urban-Rural Integration Zone	1,411	7.24
3 Rural	14,461	74.16
4 Special Zone	88	0.45
Total	19,501	100.00

bb001_w3: Residential Address Now

	Freq.	%
1 Address during Last Survey	17,510	88.36
2 Other Place	2,298	11.60
3 Abroad	8	0.04
Total	19,816	100.00

bb001_w3_1: Type of Residential Address

	Freq.	%
1 Family Housing	19,150	96.64
2 Nursing Home	52	0.26
3 Hospital	24	0.12

	Freq.	%
4 Other	590	2.98
Total	19,816	100.00

bb001_w3_2: Location of Residential Address

	Freq.	%
1 Central of City/Town	4,016	20.27
2 Urban-Rural Integration Zone	1,616	8.16
3 Rural	14,078	71.04
4 Special Zone	106	0.53
Total	19,816	100.00

bb001: Born Address

	Freq.	%
2 Township/Village/Neighborhood	169	53.65
3 Other	144	45.71
4 Abroad	2	0.63
Total	315	100.00

bb003: Year of First Live in Permanent Address

Mean	SD	Min	Max	Obs
1,989.20	21.76	1,900.00	2,018.00	308

bb004: Live in the Same Village/Community

	Freq.	%
1 Yes	221	70.16
2 No	94	29.84
Total	315	100.00

bb005: Year of First Live

Mean	SD	Min	Max	Obs
2,005.18	13.03	1,946.00	2,018.00	92

bb005_w3_1: Change Your Address

	Freq.	%
1 Yes	1,253	6.32
2 No	18,563	93.68
Total	19,816	100.00

bb005_w3_2: New Address

	Freq.	%
1 Not Sure	459	36.63
2 Another House in the Same Village	295	23.54

	Freq.	%
3 Other	499	39.82
Total	1,253	100.00

bc001_w3_1: Right or Not

	Freq.	%
1 Yes	18,649	96.65
2 No	646	3.35
Total	19,295	100.00

bc001_w3_2: Hukou Type in Last Interview

	Freq.	%
1 Agricultural Hukou	371	43.54
2 Non-agricultural Hukou	309	36.27
3 Unified Residence Hukou	170	19.95
4 Do not have Hukou	2	0.23
Total	852	100.00

bc001_w3_3: Address of Hukou

	Freq.	%
1 Address of HuKou	19,480	99.89
2 Do not have HuKou	21	0.11
Total	19,501	100.00

bc002_w3: Hukou Type and Hukou Location Changed

	Freq.	%
1 Only Hukou Type	146	0.75
2 Only Hukou Place	122	0.63
3 Both Hukou Type and Place	45	0.23
4 Both Haven't Changed	19,188	98.39
Total	19,501	100.00

bc002_w3_1: Current Hukou Type

	Freq.	%
1 Agricultural Hukou	211	41.70
2 Non-agricultural Hukou	178	35.18
3 Unified Residence Hukou	115	22.73
4 Do Not Have Hukou	2	0.40
Total	506	100.00

bc002_w3_2: Reason of HuKou Type Changed

	Freq.	%
1 Go to School	4	2.09
2 Marriage	2	1.05
3 Employment	5	2.62

	Freq.	%
4 Land is Acquired by the Government	29	15.18
5 Migration of the Whole Village	14	7.33
6 Other	137	71.73
Total	191	100.00

bc002_w3_3_1: Year of Type of Hukou Change

Mean	SD	Min	Max	Obs
2,012.99	11.35	1,900.00	2,018.00	188

bc002_w3_3_2: Month of Type of Hukou Change

Mean	SD	Min	Max	Obs
6.68	3.66	1.00	12.00	115

bc002_w3_4: Current Location of Hukou

	Freq.	%
1 Current Residential Address	295	61.20
2 Other	185	38.38
3 Do Not Have Hukou	2	0.41
Total	482	100.00

bc002_w3_5: Why did Your Location of Hukou Change

	Freq.	%
1 Go to School	6	3.59
2 Marriage	8	4.79
3 Employment	8	4.79
4 Land is Acquired by the Government	12	7.19
5 Migration of the Whole Village	9	5.39
6 Other	124	74.25
Total	167	100.00

bc002_w3_6_1: Year of Location of Hukou Change

Mean	SD	Min	Max	Obs
2,015.25	6.87	1,962.00	2,018.00	166

bc002_w3_6_2: Month of Location of Hukou Change

Mean	SD	Min	Max	Obs
6.44	3.04	1.00	12.00	141

bd001_w2_4: Education

	Freq.	%
1 No Formal Education (Illiterate)	4,496	22.69
2 Did not Finish Primary School	4,080	20.59

	Freq.	%
3 Sishu/Home School	47	0.24
4 Elementary School	4,357	21.99
5 Middle School	4,318	21.79
6 High School	1,613	8.14
7 Vocational School	475	2.40
8 Two-/Three-Year College/Associate Degree	262	1.32
9 Four-Year College/Bachelor's Degree	156	0.79
10 Master's Degree	11	0.06
11 Doctoral Degree/Ph.D.	1	0.01
Total	19,816	100.00

bd001_w3_1: Literate

	Freq.	%
1 Yes	3,088	35.81
2 No	5,535	64.19
Total	8,623	100.00

bd002_w3: Year of Education After Highest Level of Education

Mean	SD	Min	Max	Obs
0.30	0.99	0.00	24.00	11,192

bd006: Age of Finish Schooling

Mean	SD	Min	Max	Obs
12.13	8.10	0.00	117.00	19,816

bd007_w4_1_s1: None

	Freq.	%
0 No	187	0.96
1 None	19,314	99.04
Total	19,501	100.00

bd007_w4_1_s2: TV University

	Freq.	%
0 No	19,483	99.91
2 TV University	18	0.09
Total	19,501	100.00

bd007_w4_1_s3: Night School

	Freq.	%
0 No	19,448	99.73
3 Night School	53	0.27
Total	19,501	100.00

bd007_w4_1_s4: Zikao (Examinations for Self-Taught Students)

	Freq.	%
0 No	19,486	99.92
4 Zikao (Examinations for Self-Taught Students)	15	0.08
Total	19,501	100.00

bd007_w4_1_s5: Hanshou/Correspondence Course/Distance Learning

	Freq.	%
0 No	19,465	99.82
5 Hanshou/Correspondence Course/Distance Learning	36	0.18
Total	19,501	100.00

bd007_w4_1_s6: Literacy Course

	Freq.	%
0 No	19,464	99.81
6 Literacy Course	37	0.19
Total	19,501	100.00

bd007_w4_1_s7: Accelerated Education Course

	Freq.	%
0 No	19,495	99.97
7 Accelerated Education Course	6	0.03
Total	19,501	100.00

bd007_w4_1_s8: Other (Please Specify)

	Freq.	%
0 No	19,473	99.86
8 Other (Please Specify)	28	0.14
Total	19,501	100.00

bd008_w4_1: Year of Adult Education

Mean	SD	Min	Max	Obs
1.81	1.80	0.00	14.00	187

bd009_w4_1: Diploma or Degree

	Freq.	%
1 Yes	51	27.27
2 No	136	72.73
Total	187	100.00

bd011_w4: Highest Adult Schooling Degree

	Freq.	%
1 Vocational School	27	52.94
3 Four Year College / Bachelor's Degree	7	13.73
4 Others	17	33.33
Total	51	100.00

bd010_w4: When Receive the Diploma

Mean	SD	Min	Max	Obs
1,996.84	14.56	1,965.00	2,018.00	51

bd007_w2_1_s1: None

	Freq.	%
0 No	39	12.38
1 None	276	87.62
Total	315	100.00

bd007_w2_1_s2: TV University

	Freq.	%
0 No	309	98.10
2 TV University	6	1.90
Total	315	100.00

bd007_w2_1_s3: Night School

	Freq.	%
0 No	310	98.41
3 Night School	5	1.59
Total	315	100.00

bd007_w2_1_s4: Zikao (Examinations for Self-Taught Students)

	Freq.	%
0 No	308	97.78
4 Zikao (Examinations for Self-Taught Students)	7	2.22
Total	315	100.00

bd007_w2_1_s5: Hanshou/Correspondence Course/Distance Learning

	Freq.	%
0 No	299	94.92
5 Hanshou/Correspondence Course/Distance Learning	16	5.08
Total	315	100.00

bd007_w2_1_s6: Literacy Course

	Freq.	%
0 No	313	99.37
6 Literacy Course	2	0.63
Total	315	100.00

bd007_w2_1_s7: Accelerated Education Course

	Freq.	%
0 No	315	100.00
Total	315	100.00

bd007_w2_1_s8: Other (Please Specify)

	Freq.	%
0 No	310	98.41
8 Other (Please Specify)	5	1.59
Total	315	100.00

bd008_w2_1: Years of Adult Education

Mean	SD	Min	Max	Obs
2.43	1.48	0.00	6.00	39

bd009_w2_1: Diploma or Degree

	Freq.	%
1 Yes	30	76.92
2 No	9	23.08
Total	39	100.00

bd011: The Highest Adult Schooling Degree

	Freq.	%
1 Vocational School	7	23.33
2 Two/Three Year College / Associate Degree	11	36.67
3 Four Year College / Bachelor's Degree	10	33.33
4 Others	2	6.67
Total	30	100.00

bd010: Receive the Diploma

Mean	SD	Min	Max	Obs
1,998.63	9.45	1,980.00	2,018.00	30

bd012: Vocational and Technical Training

	Freq.	%

	Freq.	%
1 Yes	568	2.91
2 No	18,933	97.09
Total	19,501	100.00

bd013: Vocational Training Received

Mean	SD	Min	Max	Obs
3.14	5.55	0.00	72.00	568

bd014: Months Have Participated

Mean	SD	Min	Max	Obs
2.06	5.80	0.00	96.00	568

bd015_w4: Obtained Any Vocational Certificates

	Freq.	%
1 Yes	345	60.74
2 No	223	39.26
Total	568	100.00

bd012_w4: Vocational and Technical Training

	Freq.	%
1 Yes	53	16.83
2 No	262	83.17
Total	315	100.00

bd013_w4: Vocational Training

Mean	SD	Min	Max	Obs
6.96	9.79	1.00	54.00	53

bd014_w4: Months Have Participated

Mean	SD	Min	Max	Obs
6.91	12.15	0.00	81.00	53

bd017_w4: Obtained Any Vocational Certificates

	Freq.	%
1 Yes	38	71.70
2 No	15	28.30
Total	53	100.00

be001: Marital Status

	Freq.	%
1 Married with Spouse Present	15,530	78.37
2 Married But Not Living with Spouse Temporarily for Reasons Such as Work	1,334	6.73
3 Separated	72	0.36
4 Divorced	241	1.22
5 Widowed	2,521	12.72
6 Never Married	118	0.60
Total	19,816	100.00

be002: Have a Partner Living Together

	Freq.	%
1 Yes	12	0.41
2 No	2,940	99.59
Total	2,952	100.00

be003_w4: Months of Live with Spouse

Mean	SD	Min	Max	Obs
8.00	4.67	1.00	12.00	12

bf005_w4_1: Year of Live Apart

Mean	SD	Min	Max	Obs
2,009.56	11.02	1,971.00	2,018.00	34

bf005_w4_2: Month of Live Apart

Mean	SD	Min	Max	Obs
5.18	3.44	1.00	12.00	28

bf005_w4_3: Reason of Live Apart

	Freq.	%
1 Emotional Feud	19	55.88
2 Other	15	44.12
Total	34	100.00

bf006_w4_1: Year of Divorced

Mean	SD	Min	Max	Obs
2,009.75	16.50	1,900.00	2,018.00	52

bf006_w4_2: Month of Divorced

Mean	SD	Min	Max	Obs

6.90	3.11	1.00	12.00	39

bf006_w4_3: Reason of Divorced

	Freq.	%
1 Emotional Feud	33	62.26
2 Live in Different Places	2	3.77
3 In Order to Facilitate the Property Purchase Transactions	1	1.89
4 Other	17	32.08
Total	53	100.00

bf006_w4_4_1: You Obtained

Mean	SD	Min	Max	Obs
35,674.13	89,301.17	0.00	500,000.00	53

bf006_w4_4_2: Your Spouse Obtained

Mean	SD	Min	Max	Obs
22,208.25	61,908.60	0.00	300,000.00	53

bf006_w4_5: Have Any Children Under 18

	Freq.	%
1 Yes	15	28.30
2 No	38	71.70
Total	53	100.00

bf006_w4_5_1: The Number of Children Under 18

Mean	SD	Min	Max	Obs
1.93	1.53	1.00	7.00	15

bf006_w4_6_1: Number of Children You Raise

Mean	SD	Min	Max	Obs
1.47	1.64	0.00	7.00	15

bf006_w4_6_2: Number of Children Divorced Spouse Raise

Mean	SD	Min	Max	Obs
0.47	0.74	0.00	2.00	15

bf006_w4_7: Divorced Spouse Pays for Child-Rearing Expenses

Mean	SD	Min	Max	Obs
151.92	343.27	0.00	1,000.00	13

bf006_w4_8: You Pay for Child-Rearing Expenses

Mean	SD	Min	Max	Obs
400.00	894.43	0.00	2,000.00	5

bg001_w4: Nation

	Freq.	%
1 Han Nationality	18,235	92.02
2 Zhuang Nationality	192	0.97
3 Manchu	318	1.60
4 Hui Nationality	121	0.61
5 Miao Nationality	122	0.62
6 Uyghur Nationality	90	0.45
7 Tujia Nationality	28	0.14
8 Yi Nationality	104	0.52
9 Mongol Nationality	223	1.13
10 Tibetan Nationality	177	0.89
11 Other	206	1.04
Total	19,816	100.00

bg002_w4: Religious Beliefs

	Freq.	%
1 Yes	2,005	10.12
2 No	17,811	89.88
Total	19,816	100.00

bg003_w4: Religion

	Freq.	%
1 Buddhism	1,150	57.36
2 Taoism	31	1.55
3 Islam	98	4.89
4 Catholicism	89	4.44
5 Christianity	568	28.33
6 Other	69	3.44
Total	2,005	100.00

bg004_w4: Communist

	Freq.	%
1 Yes	1,903	9.60
2 No	17,913	90.40
Total	19,816	100.00

bg004_w4_1: The Year of Join The Communist Party

Mean	SD	Min	Max	Obs
1,983.23	16.75	1,900.00	2,018.00	1,906

bg005_w4: Ever Been an Educated Youth in a Poor Rural Area

	Freq.	%
1 Yes	504	2.54
2 No	19,312	97.46
Total	19,816	100.00

bg005_w4_1: Which Year

Mean	SD	Min	Max	Obs
1,970.52	5.29	1,950.00	1,993.00	512

bg006_w4: Return to the City

	Freq.	%
1 Yes	441	87.50
2 No	63	12.50
Total	504	100.00

bg006_w4_1: Which Year

Mean	SD	Min	Max	Obs
1,976.17	5.02	1,956.00	2,005.00	441

bf008: Respondents Seek Help from Others

	Freq.	%
1 Never	16,242	81.97
2 a Few Times	2,149	10.85
3 Always Ask for Help	311	1.57
4 Proxy Answers Questions	1,113	5.62
Total	19,815	100.00

xrtype: Respondent Type

	Freq.	%
1 RE Interview	19,501	98.41
2 New Interview	315	1.59
Total	19,816	100.00

xrgender: Generated Gender

	Freq.	%
1 Male	9,340	47.13
2 Female	10,476	52.87
Total	19,816	100.00

zfrgender: ZFRGender

Mean	SD	Min	Max	Obs
1.00	0.00	1.00	1.00	19,501

zfrzodiac: ZFRzodiac

Mean	SD	Min	Max	Obs
1.00	0.00	1.00	1.00	19,501

zfrbirth: ZFRbirth

Mean	SD	Min	Max	Obs
1.00	0.00	1.00	1.00	19,501

ziwtime: ZIWTime

A String Variable	
Obs:	19,501

zbc004: Record Hukou

	Freq.	%
1 Agricultural Hukou	15,298	79.28
2 Non-agricultural Hukou	3,954	20.49
3 Unified Residence Hukou	40	0.21
4 Do not have Hukou	3	0.02
Total	19,295	100.00

zfredu: ZFRedu

Mean	SD	Min	Max	Obs
1.00	0.00	1.00	1.00	19,501

versionID: Version ID

A String Variable	
Obs:	19,816

MODULE C1

Family Information

ID: Individual ID

A String Variable	
Obs:	11,628

householdID: Household ID

A String Variable	
Obs:	11,628

communityID: Community ID

A String Variable	
Obs:	11,628

cv009: Family Respondent

	Freq.	%
1 Main Respondent	4,991	57.37
2 Spouse	3,708	42.63
Total	8,699	100.00

ca000_w4_0_1_: Aware of Biological Father's Information

	Freq.	%
1 Yes	11,323	97.38
2 No	305	2.62
Total	11,628	100.00

ca000_w4_0_2_: Aware of Biological Father's Information

	Freq.	%
1 Yes	8,535	98.14
2 No	162	1.86
Total	8,697	100.00

ca000_w4_1_1_: Biological Father was Born in [ZparBirth[1]]

	Freq.	%
1 Right	5,376	76.34
2 Wrong	1,666	23.66
Total	7,042	100.00

ca000_w4_1_2_: Biological Father was Born in [ZparBirth[1]]

	Freq.	%
1 Right	3,902	78.04
2 Wrong	1,098	21.96
Total	5,000	100.00

ca000_w3_1_1_: When was Biological Father Born

Mean	SD	Min	Max	Obs
1,924.99	15.94	1,830.00	2,018.00	5,807

ca000_w3_1_2_: When was Biological Father Born

Mean	SD	Min	Max	Obs
1,927.89	15.31	1,800.00	2,018.00	4,472

ca000_w3_2_1_: Biological Father is Still Alive

	Freq.	%
1 Yes	1,451	12.82
2 No	9,869	87.18
Total	11,320	100.00

ca000_w3_2_2_: Biological Father is Still Alive

	Freq.	%
1 Yes	1,412	16.55
2 No	7,121	83.45
Total	8,533	100.00

ca000_w3_3_1_1_: When did Biological Father Die

Mean	SD	Min	Max	Obs
1,973.35	168.73	1.00	2,018.00	4,474

ca000_w3_3_1_2_: When did Biological Father Die

Mean	SD	Min	Max	Obs
1,983.28	130.65	2.00	2,018.00	3,470

ca000_w3_3_2_1_: The Age of Biological Father When He Died

Mean	SD	Min	Max	Obs
73.83	100.78	0.00	2,017.00	5,253

ca000_w3_3_2_2_: The Age of Biological Father When He Died

Mean	SD	Min	Max	Obs
73.17	99.58	0.00	2,008.00	3,434

ca001_w4_0_1_: Aware of Biological Mother's Information

	Freq.	%
1 Yes	11,347	97.61
2 No	278	2.39
Total	11,625	100.00

ca001_w4_0_2_: Aware of Biological Mother's Information

	Freq.	%
1 Yes	8,547	98.31
2 No	147	1.69
Total	8,694	100.00

ca001_w4_1_1_: Biological Mother was Born in [ZparBirth[2]]

	Freq.	%
1 Right	5,486	74.53
2 Wrong	1,875	25.47
Total	7,361	100.00

ca001_w4_1_2_: Biological Mother was Born in [ZparBirth[2]]

	Freq.	%
1 Right	4,045	76.93
2 Wrong	1,213	23.07
Total	5,258	100.00

ca001_w3_1_1_: When was Biological Mother Born

Mean	SD	Min	Max	Obs
1,927.97	15.24	1,830.00	2,018.00	5,737

ca001_w3_1_2_: When was Biological Mother Born

Mean	SD	Min	Max	Obs
1,930.95	14.53	1,800.00	2,018.00	4,369

ca001_w3_2_1_: Biological Mother is Still Alive

	Freq.	%
1 Yes	2,627	23.15
2 No	8,719	76.85
Total	11,346	100.00

ca001_w3_2_2_: Biological Mother is Still Alive

	Freq.	%
1 Yes	2,315	27.09
2 No	6,230	72.91
Total	8,545	100.00

ca001_w3_3_1_1_: When did Biological Mother Die

Mean	SD	Min	Max	Obs
1,983.52	133.60	0.00	2,018.00	4,033

ca001_w3_3_1_2_: When did Biological Mother Die

Mean	SD	Min	Max	Obs
1,987.82	121.03	0.00	2,018.00	3,084

ca001_w3_3_2_1_: The Age of Biological Mother When He Died

Mean	SD	Min	Max	Obs
75.67	104.69	0.00	2,016.00	4,439

ca001_w3_3_2_2_: The Age of Biological Mother When He Died

Mean	SD	Min	Max	Obs
79.76	143.29	0.00	2,015.00	2,919

ca002_w4_1_: Are Biological Father and Mother Still Married

	Freq.	%
1 Yes	880	98.88
2 No	10	1.12
Total	890	100.00

ca002_w4_2_: Are Biological Father and Mother Still Married

	Freq.	%
1 Yes	887	98.23
2 No	16	1.77
Total	903	100.00

ca006_w3_1_1_: Whether Having Foster Father

	Freq.	%
1 Yes	404	3.48
2 No	11,219	96.52
Total	11,623	100.00

ca006_w3_1_2_: Whether Having Foster Father

	Freq.	%
1 Yes	265	3.05
2 No	8,427	96.95
Total	8,692	100.00

ca006_w3_2_1_: Foster Father is Still Alive

	Freq.	%
1 Yes	40	9.90
2 No	364	90.10
Total	404	100.00

ca006_w3_2_2_: Foster Father is Still Alive

	Freq.	%
1 Yes	27	10.19
2 No	238	89.81
Total	265	100.00

ca006_w4_3_1_: Foster Father was Born in [ZparBirth[3]]

	Freq.	%
1 Right	4	100.00
Total	4	100.00

ca006_w4_3_2_: Foster Father was Born in [ZparBirth[3]]

	Freq.	%
1 Right	2	100.00
Total	2	100.00

ca006_w3_3_1_: When was Foster Father Born

Mean	SD	Min	Max	Obs
1,939.44	8.82	1,923.00	1,955.00	34

ca006_w3_3_2_: When was Foster Father Born

Mean	SD	Min	Max	Obs
1,939.80	18.23	1,910.00	2,018.00	25

ca007_w3_1_1_: Whether Having Foster Mother

	Freq.	%
1 Yes	345	2.97
2 No	11,278	97.03
Total	11,623	100.00

ca007_w3_1_2_: Whether Having Foster Mother

	Freq.	%
1 Yes	214	2.46
2 No	8,478	97.54
Total	8,692	100.00

ca007_w3_2_1_: Foster Mother is Still Alive

	Freq.	%
1 Yes	55	15.94
2 No	290	84.06
Total	345	100.00

ca007_w3_2_2_: Foster Mother is Still Alive

	Freq.	%
1 Yes	32	14.95
2 No	182	85.05
Total	214	100.00

ca007_w4_3_1_: Foster Mother was Born in [ZparBirth[4]]

	Freq.	%
1 Right	9	69.23
2 Wrong	4	30.77
Total	13	100.00

ca007_w4_3_2_: Foster Mother was Born in [ZparBirth[4]]

	Freq.	%
1 Right	7	70.00

	Freq.	%
2 Wrong	3	30.00
Total	10	100.00

ca007_w3_3_1_: When was Foster Mother Born

Mean	SD	Min	Max	Obs
1,937.74	9.92	1,917.00	1,958.00	42

ca007_w3_3_2_: When was Foster Mother Born

Mean	SD	Min	Max	Obs
1,939.04	11.41	1,900.00	1,957.00	25

ca007_w3_5_1_: Are Foster Father and Mother Still Married

	Freq.	%
1 Yes	7	100.00
Total	7	100.00

ca007_w3_5_2_: Are Foster Father and Mother Still Married

	Freq.	%
1 Yes	4	100.00
Total	4	100.00

ca009_w4_1_: [ParType[1]]'s Highest Degree is [ZParEdu[1]]

	Freq.	%
1 Right	6,953	85.64
2 Wrong	1,166	14.36
Total	8,119	100.00

ca009_w4_2_: [ParType[2]]'s Highest Degree is [ZParEdu[2]]

	Freq.	%
1 Right	7,959	93.32
2 Wrong	570	6.68
Total	8,529	100.00

ca009_w4_3_: [ParType[3]]'s Highest Degree is [ZParEdu[3]]

	Freq.	%
1 Right	4	66.67
2 Wrong	2	33.33
Total	6	100.00

ca009_w4_4_: [ParType[4]]'s Highest Degree is [ZParEdu[4]]

	Freq.	%
1 Right	14	100.00
Total	14	100.00

ca009_w4_5_: [ParType[5]]'s Highest Degree is [ZParEdu[5]]

	Freq.	%
1 Right	4,949	86.90
2 Wrong	746	13.10
Total	5,695	100.00

ca009_w4_6_: [ParType[6]]'s Highest Degree is [ZParEdu[6]]

	Freq.	%
1 Right	5,660	93.69
2 Wrong	381	6.31
Total	6,041	100.00

ca009_w4_7_: [ParType[7]]'s Highest Degree is [ZParEdu[7]]

	Freq.	%
1 Right	1	33.33
2 Wrong	2	66.67
Total	3	100.00

ca009_w4_8_: [ParType[8]]'s Highest Degree is [ZParEdu[8]]

	Freq.	%
1 Right	9	90.00
2 Wrong	1	10.00
Total	10	100.00

ca009_1_: [ParType[1]]'s Highest Degree

	Freq.	%
1 No Formal Education(Illiterate)	1,611	36.90
2 Did not Finish Primary School	861	19.72
3 Sishu/Home School	388	8.89
4 Elementary School	827	18.94
5 Middle School	401	9.18
6 High School	146	3.34
7 Vocational School	64	1.47
8 Two-/Three-Year College/Associate Degree	19	0.44
9 Four-Year College/Bachelor's Degree	47	1.08
10 Post-graduate, Master's Degree	1	0.02
11 Post-graduate, Doctoral Degree/Ph.D	1	0.02
Total	4,366	100.00

ca009_2_: [ParType[2]]'s Highest Degree

	Freq.	%
1 No Formal Education(Illiterate)	2,230	65.86
2 Did not Finish Primary School	453	13.38
3 Sishu/Home School	58	1.71
4 Elementary School	425	12.55
5 Middle School	142	4.19
6 High School	48	1.42
7 Vocational School	19	0.56
9 Four-Year College/Bachelor's Degree	10	0.30
11 Post-graduate, Doctoral Degree/Ph.D	1	0.03
Total	3,386	100.00

ca009_3_: [ParType[3]]'s Highest Degree

	Freq.	%
1 No Formal Education(Illiterate)	14	38.89
2 Did not Finish Primary School	5	13.89
3 Sishu/Home School	1	2.78
4 Elementary School	12	33.33
5 Middle School	3	8.33
9 Four-Year College/Bachelor's Degree	1	2.78
Total	36	100.00

ca009_4_: [ParType[4]]'s Highest Degree

	Freq.	%
1 No Formal Education(Illiterate)	24	58.54
2 Did not Finish Primary School	5	12.20
3 Sishu/Home School	1	2.44
4 Elementary School	6	14.63
5 Middle School	5	12.20
Total	41	100.00

ca009_5_: [ParType[5]]'s Highest Degree

	Freq.	%
1 No Formal Education(Illiterate)	1,389	38.79
2 Did not Finish Primary School	623	17.40
3 Sishu/Home School	207	5.78
4 Elementary School	758	21.17
5 Middle School	361	10.08
6 High School	138	3.85
7 Vocational School	56	1.56
8 Two-/Three-Year College/Associate Degree	19	0.53
9 Four-Year College/Bachelor's Degree	30	0.84
Total	3,581	100.00

ca009_6_: [ParType[6]]'s Highest Degree

	Freq.	%
1 No Formal Education(Illiterate)	1,901	65.89
2 Did not Finish Primary School	326	11.30

	Freq.	%
3 Sishu/Home School	43	1.49
4 Elementary School	420	14.56
5 Middle School	122	4.23
6 High School	43	1.49
7 Vocational School	21	0.73
8 Two-/Three-Year College/Associate Degree	8	0.28
9 Four-Year College/Bachelor's Degree	1	0.03
Total	2,885	100.00

ca009_7_: [ParType[7]]'s Highest Degree

	Freq.	%
1 No Formal Education(Illiterate)	18	69.23
2 Did not Finish Primary School	4	15.38
4 Elementary School	3	11.54
6 High School	1	3.85
Total	26	100.00

ca009_8_: [ParType[8]]'s Highest Degree

	Freq.	%
1 No Formal Education(Illiterate)	11	47.83
2 Did not Finish Primary School	4	17.39
4 Elementary School	6	26.09
5 Middle School	1	4.35
6 High School	1	4.35
Total	23	100.00

ca009_w3_1_: [ParType[1]] is Literate

	Freq.	%
1 Yes	1,587	55.49
2 No	1,273	44.51
Total	2,860	100.00

ca009_w3_2_: [ParType[2]] is Literate

	Freq.	%
1 Yes	585	21.34
2 No	2,156	78.66
Total	2,741	100.00

ca009_w3_3_: [ParType[3]] is Literate

	Freq.	%
1 Yes	4	20.00
2 No	16	80.00
Total	20	100.00

ca009_w3_4_: [ParType[4]] is Literate

	Freq.	%
1 Yes	4	13.33
2 No	26	86.67
Total	30	100.00

ca009_w3_5_: [ParType[5]] is Literate

	Freq.	%
1 Yes	1,059	47.72
2 No	1,160	52.28
Total	2,219	100.00

ca009_w3_6_: [ParType[6]] is Literate

	Freq.	%
1 Yes	444	19.56
2 No	1,826	80.44
Total	2,270	100.00

ca009_w3_7_: [ParType[7]] is Literate

	Freq.	%
1 Yes	5	22.73
2 No	17	77.27
Total	22	100.00

ca009_w3_8_: [ParType[8]] is Literate

	Freq.	%
1 Yes	4	26.67
2 No	11	73.33
Total	15	100.00

ca014_w4_1_: [ParType[1]]'s Highest Professional Position is [ZParOcc[1]]

	Freq.	%
1 Yes	879	85.67
2 No	147	14.33
Total	1,026	100.00

ca014_w4_2_: [ParType[2]]'s Highest Professional Position is [ZParOcc[2]]

	Freq.	%
1 Yes	1,797	91.64
2 No	164	8.36
Total	1,961	100.00

ca014_w4_3_: [ParType[3]]'s Highest Professional Position is [ZParOcc[3]]

	Freq.	%
1 Yes	5	100.00
Total	5	100.00

ca014_w4_4_: [ParType[4]]'s Highest Professional Position is [ZParOcc[4]]

	Freq.	%
1 Yes	12	85.71
2 No	2	14.29
Total	14	100.00

ca014_w4_5_: [ParType[5]]'s Highest Professional Position is [ZParOcc[5]]

	Freq.	%
1 Yes	804	84.99
2 No	142	15.01
Total	946	100.00

ca014_w4_6_: [ParType[6]]'s Highest Professional Position is [ZParOcc[6]]

	Freq.	%
1 Yes	1,547	92.58
2 No	124	7.42
Total	1,671	100.00

ca014_w4_7_: [ParType[7]]'s Highest Professional Position is [ZParOcc[7]]

	Freq.	%
1 Yes	4	100.00
Total	4	100.00

ca014_w4_8_: [ParType[8]]'s Highest Professional Position is [ZParOcc[8]]

	Freq.	%
1 Yes	8	100.00
Total	8	100.00

ca014_1_: [ParType[1]]'s Highest Professional Position

	Freq.	%
1 Managers	942	9.02
2 Professionals and Technicians	980	9.39
3 Clerks	419	4.01
4 Commercial and Service Workers	384	3.68
5 Agricultural, Forestry, Husbandry and Fishery Producers	6,635	63.55
6 Production and Transportation Workers	546	5.23
7 Can't be Specified	534	5.11
Total	10,440	100.00

ca014_2_: [ParType[2]]'s Highest Professional Position

	Freq.	%
1 Managers	101	1.06
2 Professionals and Technicians	174	1.82
3 Clerks	84	0.88
4 Commercial and Service Workers	171	1.79
5 Agricultural, Forestry, Husbandry and Fishery Producers	7,963	83.41
6 Production and Transportation Workers	219	2.29
7 Can't be Specified	835	8.75
Total	9,547	100.00

ca014_3_: [ParType[3]]'s Highest Professional Position

	Freq.	%
1 Managers	2	5.71
2 Professionals and Technicians	1	2.86
3 Clerks	1	2.86
5 Agricultural, Forestry, Husbandry and Fishery Producers	27	77.14
6 Production and Transportation Workers	4	11.43
Total	35	100.00

ca014_4_: [ParType[4]]'s Highest Professional Position

	Freq.	%
1 Managers	2	4.65
2 Professionals and Technicians	1	2.33
3 Clerks	1	2.33
5 Agricultural, Forestry, Husbandry and Fishery Producers	32	74.42
7 Can't be Specified	7	16.28
Total	43	100.00

ca014_5_: [ParType[5]]'s Highest Professional Position

	Freq.	%
1 Managers	649	8.40
2 Professionals and Technicians	651	8.43
3 Clerks	264	3.42
4 Commercial and Service Workers	245	3.17
5 Agricultural, Forestry, Husbandry and Fishery Producers	5,173	66.96
6 Production and Transportation Workers	370	4.79
7 Can't be Specified	374	4.84
Total	7,726	100.00

ca014_6_: [ParType[6]]'s Highest Professional Position

	Freq.	%
1 Managers	69	0.99
2 Professionals and Technicians	120	1.71
3 Clerks	72	1.03
4 Commercial and Service Workers	129	1.84
5 Agricultural, Forestry, Husbandry and Fishery Producers	5,915	84.52

	Freq.	%
6 Production and Transportation Workers	148	2.11
7 Can't be Specified	545	7.79
Total	6,998	100.00

ca014_7_: [ParType[7]]'s Highest Professional Position

	Freq.	%
2 Professionals and Technicians	2	8.70
5 Agricultural, Forestry, Husbandry and Fishery Producers	17	73.91
6 Production and Transportation Workers	2	8.70
7 Can't be Specified	2	8.70
Total	23	100.00

ca014_8_: [ParType[8]]'s Highest Professional Position

	Freq.	%
1 Managers	1	4.17
4 Commercial and Service Workers	2	8.33
5 Agricultural, Forestry, Husbandry and Fishery Producers	20	83.33
7 Can't be Specified	1	4.17
Total	24	100.00

ca023_w3_1_: [ParType[1]] is Communist Party Member

	Freq.	%
1 Yes	1,813	16.02
2 No	9,506	83.98
Total	11,319	100.00

ca023_w3_2_: [ParType[2]] is Communist Party Member

	Freq.	%
1 Yes	297	2.62
2 No	11,047	97.38
Total	11,344	100.00

ca023_w3_3_: [ParType[3]] is Communist Party Member

	Freq.	%
1 Yes	4	10.00
2 No	36	90.00
Total	40	100.00

ca023_w3_4_: [ParType[4]] is Communist Party Member

	Freq.	%
1 Yes	3	5.45
2 No	52	94.55
Total	55	100.00

ca023_w3_5_: [ParType[5]] is Communist Party Member

	Freq.	%
1 Yes	1,313	15.39
2 No	7,217	84.61
Total	8,530	100.00

ca023_w3_6_: [ParType[6]] is Communist Party Member

	Freq.	%
1 Yes	193	2.26
2 No	8,352	97.74
Total	8,545	100.00

ca023_w3_7_: [ParType[7]] is Communist Party Member

	Freq.	%
1 Yes	7	25.93
2 No	20	74.07
Total	27	100.00

ca023_w3_8_: [ParType[8]] is Communist Party Member

	Freq.	%
1 Yes	1	3.13
2 No	31	96.88
Total	32	100.00

ca016_1_: Where is [ParType[1]] Living Regularly

	Freq.	%
1 Livng with Respondent	134	9.25
2 Living in the Same or Nearby Courtyard House(Apartment) with Respondent	170	11.73
3 The Birthplace of Respondents' [ParType[1]]	755	52.10
4 Others	389	26.85
5 Abroad	1	0.07
Total	1,449	100.00

ca016_2_: Where is [ParType[2]] Living Regularly

	Freq.	%
1 Livng with Respondent	378	14.39
2 Living in the Same or Nearby Courtyard House(Apartment) with Respondent	386	14.69
3 The Birthplace of Respondents' [ParType[2]]	495	18.84
4 Others	1,364	51.92
5 Abroad	4	0.15
Total	2,627	100.00

ca016_3_: Where is [ParType[3]] Living Regularly

	Freq.	%
1 Livng with Respondent	5	12.50
2 Living in the Same or Nearby Courtyard House(Apartment) with Respondent	6	15.00
3 The Birthplace of Respondents' [ParType[3]]	14	35.00
4 Others	15	37.50
Total	40	100.00

ca016_4_: Where is [ParType[4]] Living Regularly

	Freq.	%
1 Livng with Respondent	5	9.09
2 Living in the Same or Nearby Courtyard House(Apartment) with Respondent	8	14.55
3 The Birthplace of Respondents' [ParType[4]]	7	12.73
4 Others	35	63.64
Total	55	100.00

ca016_5_: Where is [ParType[5]] Living Regularly

	Freq.	%
1 Livng with Respondent	110	7.79
2 Living in the Same or Nearby Courtyard House(Apartment) with Respondent	223	15.79
3 The Birthplace of Respondents' [ParType[5]]	731	51.77
4 Others	348	24.65
Total	1,412	100.00

ca016_6_: Where is [ParType[6]] Living Regularly

	Freq.	%
1 Livng with Respondent	238	10.28
2 Living in the Same or Nearby Courtyard House(Apartment) with Respondent	408	17.62
3 The Birthplace of Respondents' [ParType[6]]	506	21.86
4 Others	1,160	50.11
5 Abroad	3	0.13
Total	2,315	100.00

ca016_7_: Where is [ParType[7]] Living Regularly

	Freq.	%
2 Living in the Same or Nearby Courtyard House(Apartment) with Respondent	1	3.70
3 The Birthplace of Respondents' [ParType[7]]	16	59.26
4 Others	9	33.33
5 Abroad	1	3.70
Total	27	100.00

ca016_8_: Where is [ParType[8]] Living Regularly

	Freq.	%
1 Livng with Respondent	4	12.50
2 Living in the Same or Nearby Courtyard House(Apartment) with Respondent	2	6.25
3 The Birthplace of Respondents' [ParType[8]]	2	6.25
4 Others	24	75.00
Total	32	100.00

ca017_1_: Type of Living Place for [ParType[1]]

	Freq.	%
1 Center Area of City or Town	164	42.05
2 Urban-suburban-integration Area	45	11.54
3 Rural Area	177	45.38
4 Special Area	4	1.03
Total	390	100.00

ca017_2_: Type of Living Place for [ParType[2]]

	Freq.	%
1 Center Area of City or Town	306	22.37
2 Urban-suburban-integration Area	85	6.21
3 Rural Area	971	70.98
4 Special Area	6	0.44
Total	1,368	100.00

ca017_3_: Type of Living Place for [ParType[3]]

	Freq.	%
1 Center Area of City or Town	6	40.00
2 Urban-suburban-integration Area	1	6.67
3 Rural Area	8	53.33
Total	15	100.00

ca017_4_: Type of Living Place for [ParType[4]]

	Freq.	%
1 Center Area of City or Town	16	45.71
2 Urban-suburban-integration Area	3	8.57
3 Rural Area	15	42.86
4 Special Area	1	2.86
Total	35	100.00

ca017_5_: Type of Living Place for [ParType[5]]

	Freq.	%
1 Center Area of City or Town	132	37.93
2 Urban-suburban-integration Area	37	10.63
3 Rural Area	175	50.29
4 Special Area	4	1.15

Total	348	100.00

ca017_6_: Type of Living Place for [ParType[6]]

	Freq.	%
1 Center Area of City or Town	283	24.33
2 Urban-suburban-integration Area	81	6.96
3 Rural Area	792	68.10
4 Special Area	7	0.60
Total	1,163	100.00

ca017_7_: Type of Living Place for [ParType[7]]

	Freq.	%
1 Center Area of City or Town	4	40.00
3 Rural Area	6	60.00
Total	10	100.00

ca017_8_: Type of Living Place for [ParType[8]]

	Freq.	%
1 Center Area of City or Town	2	8.33
2 Urban-suburban-integration Area	5	20.83
3 Rural Area	17	70.83
Total	24	100.00

ca026_w3_1_: [ParType[1]] Has Self-care Ability

	Freq.	%
1 Yes	1,218	84.06
2 No	231	15.94
Total	1,449	100.00

ca026_w3_2_: [ParType[2]] Has Self-care Ability

	Freq.	%
1 Yes	2,128	81.00
2 No	499	19.00
Total	2,627	100.00

ca026_w3_3_: [ParType[3]] Has Self-care Ability

	Freq.	%
1 Yes	35	87.50
2 No	5	12.50
Total	40	100.00

ca026_w3_4_: [ParType[4]] Has Self-care Ability

	Freq.	%
1 Yes	48	87.27
2 No	7	12.73
Total	55	100.00

ca026_w3_5_: [ParType[5]] Has Self-care Ability

	Freq.	%
1 Yes	1,193	84.49
2 No	219	15.51
Total	1,412	100.00

ca026_w3_6_: [ParType[6]] Has Self-care Ability

	Freq.	%
1 Yes	1,919	82.89
2 No	396	17.11
Total	2,315	100.00

ca026_w3_7_: [ParType[7]] Has Self-care Ability

	Freq.	%
1 Yes	22	81.48
2 No	5	18.52
Total	27	100.00

ca026_w3_8_: [ParType[8]] Has Self-care Ability

	Freq.	%
1 Yes	27	84.38
2 No	5	15.63
Total	32	100.00

cb050_w3: Number of Children

Mean	SD	Min	Max	Obs
0.85	1.02	0.00	4.00	252

cb051_w3_1: How Many Children does FamilyR Have Except for [XChildName]

Mean	SD	Min	Max	Obs
0.03	0.25	0.00	5.00	11,364

cb039_1_1_: When did [XChildName[1]] Die

Mean	SD	Min	Max	Obs
2,001.32	19.09	1,900.00	2,018.00	249

cb039_1_2_: When did [XChildName[2]] Die

Mean	SD	Min	Max	Obs
1,999.72	15.88	1,900.00	2,018.00	226

cb039_1_3_: When did [XChildName[3]] Die

Mean	SD	Min	Max	Obs
1,992.67	19.95	1,900.00	2,018.00	311

cb039_1_4_: When did [XChildName[4]] Die

Mean	SD	Min	Max	Obs
1,988.68	18.90	1,900.00	2,018.00	273

cb039_1_5_: When did [XChildName[5]] Die

Mean	SD	Min	Max	Obs
1,985.56	22.47	1,900.00	2,018.00	222

cb039_1_6_: When did [XChildName[6]] Die

Mean	SD	Min	Max	Obs
1,982.02	22.73	1,900.00	2,018.00	164

cb039_1_7_: When did [XChildName[7]] Die

Mean	SD	Min	Max	Obs
1,977.24	24.07	1,900.00	2,018.00	88

cb039_1_8_: When did [XChildName[8]] Die

Mean	SD	Min	Max	Obs
1,973.02	23.92	1,900.00	2,009.00	41

cb039_1_9_: When did [XChildName[9]] Die

Mean	SD	Min	Max	Obs
1,974.88	25.23	1,900.00	2,015.00	16

cb039_1_10_: When did [XChildName[10]] Die

Mean	SD	Min	Max	Obs
1,978.33	14.96	1,957.00	1,999.00	6

cb039_1_11_: When did [XChildName[11]] Die

Mean	SD	Min	Max	Obs
1,990.33	8.02	1,982.00	1,998.00	3

cb039_2_1_: How Old was [XChildName[1]] When Died

Mean	SD	Min	Max	Obs
5.51	3.74	0.00	12.00	247

cb039_2_2_: How Old was [XChildName[2]] When Died

Mean	SD	Min	Max	Obs
5.43	3.86	0.00	12.00	219

cb039_2_3_: How Old was [XChildName[3]] When Died

Mean	SD	Min	Max	Obs
5.21	4.12	0.00	12.00	308

cb039_2_4_: How Old was [XChildName[4]] When Died

Mean	SD	Min	Max	Obs
4.73	4.00	0.00	12.00	263

cb039_2_5_: How Old was [XChildName[5]] When Died

Mean	SD	Min	Max	Obs
5.00	4.22	0.00	12.00	220

cb039_2_6_: How Old was [XChildName[6]] When Died

Mean	SD	Min	Max	Obs
3.92	3.78	0.00	12.00	161

cb039_2_7_: How Old was [XChildName[7]] When Died

Mean	SD	Min	Max	Obs
2.91	3.62	0.00	12.00	89

cb039_2_8_: How Old was [XChildName[8]] When Died

Mean	SD	Min	Max	Obs
2.90	4.14	0.00	12.00	40

cb039_2_9_: How Old was [XChildName[9]] When Died

Mean	SD	Min	Max	Obs
2.38	3.63	0.00	12.00	16

cb039_2_10_: How Old was [XChildName[10]] When Died

Mean	SD	Min	Max	Obs
3.33	4.46	0.00	9.00	6

cb039_2_11_: How Old was [XChildName[11]] When Died

Mean	SD	Min	Max	Obs
5.67	4.93	0.00	9.00	3

cb052_w4_1_: Is [XChildName[1]]'s Highest Degree Right

	Freq.	%
1 Right	8,508	84.27
2 Wrong	1,588	15.73
Total	10,096	100.00

cb052_w4_2_: Is [XChildName[2]]'s Highest Degree Right

	Freq.	%
1 Right	6,889	82.30
2 Wrong	1,482	17.70
Total	8,371	100.00

cb052_w4_3_: Is [XChildName[3]]'s Highest Degree Right

	Freq.	%
1 Right	3,997	84.88
2 Wrong	712	15.12
Total	4,709	100.00

cb052_w4_4_: Is [XChildName[4]]'s Highest Degree Right

	Freq.	%
1 Right	2,102	85.31
2 Wrong	362	14.69
Total	2,464	100.00

cb052_w4_5_: Is [XChildName[5]]'s Highest Degree Right

	Freq.	%
1 Right	978	83.23
2 Wrong	197	16.77
Total	1,175	100.00

cb052_w4_6_: Is [XChildName[6]]'s Highest Degree Right

	Freq.	%
1 Right	432	82.92
2 Wrong	89	17.08
Total	521	100.00

cb052_w4_7_: Is [XChildName[7]]'s Highest Degree Right

	Freq.	%
1 Right	206	84.43
2 Wrong	38	15.57
Total	244	100.00

cb052_w4_8_: Is [XChildName[8]]'s Highest Degree Right

	Freq.	%
1 Right	101	87.83
2 Wrong	14	12.17
Total	115	100.00

cb052_w4_9_: Is [XChildName[9]]'s Highest Degree Right

	Freq.	%
1 Right	42	93.33
2 Wrong	3	6.67
Total	45	100.00

cb052_w4_10_: Is [XChildName[10]]'s Highest Degree Right

	Freq.	%
1 Right	22	95.65
2 Wrong	1	4.35
Total	23	100.00

cb052_w4_11_: Is [XChildName[11]]'s Highest Degree Right

	Freq.	%
1 Right	7	77.78
2 Wrong	2	22.22
Total	9	100.00

cb052_w4_12_: Is [XChildName[12]]'s Highest Degree Right

	Freq.	%
1 Right	5	100.00
Total	5	100.00

cb052_w4_13_: Is [XChildName[13]]'s Highest Degree Right

	Freq.	%
1 Right	1	100.00
Total	1	100.00

cb052_w4_14_: Is [XChildName[14]]'s Highest Degree Right

	Freq.	%
1 Right	1	100.00
Total	1	100.00

cb052_w3_1_: [XChildName[1]]'s Highest Degree

	Freq.	%
1 No Formal Education(Illiterate)	55	2.21
2 Did not Finish Primary School	158	6.35
4 Elementary School	409	16.43
5 Middle School	593	23.82
6 High School	348	13.98
7 Vocational School	199	7.99
8 Two-/Three-Year College/Associate Degree	275	11.04
9 Four-Year College/Bachelor's Degree	380	15.26
10 Post-graduate, Master's Degree	54	2.17
11 Post-graduate, Doctoral Degree/Ph.D	4	0.16
997 Don't Know	11	0.44
999 Refuse to Answer	4	0.16
Total	2,490	100.00

cb052_w3_2_: [XChildName[2]]'s Highest Degree

	Freq.	%
1 No Formal Education(Illiterate)	75	3.38
2 Did not Finish Primary School	227	10.23
4 Elementary School	497	22.40
5 Middle School	618	27.85
6 High School	281	12.66
7 Vocational School	140	6.31
8 Two-/Three-Year College/Associate Degree	153	6.89
9 Four-Year College/Bachelor's Degree	184	8.29
10 Post-graduate, Master's Degree	23	1.04
11 Post-graduate, Doctoral Degree/Ph.D	7	0.32
997 Don't Know	8	0.36
999 Refuse to Answer	6	0.27
Total	2,219	100.00

cb052_w3_3_: [XChildName[3]]'s Highest Degree

	Freq.	%
1 No Formal Education(Illiterate)	76	6.68
2 Did not Finish Primary School	148	13.02
3 Sishu/Home School	1	0.09
4 Elementary School	269	23.66
5 Middle School	322	28.32

	Freq.	%
6 High School	120	10.55
7 Vocational School	48	4.22
8 Two-/Three-Year College/Associate Degree	57	5.01
9 Four-Year College/Bachelor's Degree	68	5.98
10 Post-graduate, Master's Degree	11	0.97
11 Post-graduate, Doctoral Degree/Ph.D	3	0.26
997 Don't Know	11	0.97
999 Refuse to Answer	3	0.26
Total	1,137	100.00

cb052_w3_4_: [XChildName[4]]'s Highest Degree

	Freq.	%
1 No Formal Education(Illiterate)	45	7.18
2 Did not Finish Primary School	103	16.43
4 Elementary School	181	28.87
5 Middle School	146	23.29
6 High School	42	6.70
7 Vocational School	36	5.74
8 Two-/Three-Year College/Associate Degree	26	4.15
9 Four-Year College/Bachelor's Degree	22	3.51
10 Post-graduate, Master's Degree	2	0.32
11 Post-graduate, Doctoral Degree/Ph.D	1	0.16
997 Don't Know	17	2.71
999 Refuse to Answer	6	0.96
Total	627	100.00

cb052_w3_5_: [XChildName[5]]'s Highest Degree

	Freq.	%
1 No Formal Education(Illiterate)	36	10.78
2 Did not Finish Primary School	69	20.66
4 Elementary School	85	25.45
5 Middle School	75	22.46
6 High School	24	7.19
7 Vocational School	8	2.40
8 Two-/Three-Year College/Associate Degree	15	4.49
9 Four-Year College/Bachelor's Degree	8	2.40
10 Post-graduate, Master's Degree	1	0.30
11 Post-graduate, Doctoral Degree/Ph.D	1	0.30
997 Don't Know	9	2.69
999 Refuse to Answer	3	0.90
Total	334	100.00

cb052_w3_6_: [XChildName[6]]'s Highest Degree

	Freq.	%
1 No Formal Education(Illiterate)	24	15.89
2 Did not Finish Primary School	29	19.21
4 Elementary School	38	25.17
5 Middle School	28	18.54
6 High School	10	6.62
7 Vocational School	6	3.97
8 Two-/Three-Year College/Associate Degree	1	0.66
9 Four-Year College/Bachelor's Degree	5	3.31

10 Post-graduate, Master's Degree	1	0.66
997 Don't Know	8	5.30
999 Refuse to Answer	1	0.66
Total	151	100.00

cb052_w3_7_: [XChildName[7]]'s Highest Degree

	Freq.	%
1 No Formal Education(Illiterate)	9	12.16
2 Did not Finish Primary School	14	18.92
4 Elementary School	17	22.97
5 Middle School	20	27.03
6 High School	3	4.05
7 Vocational School	1	1.35
8 Two-/Three-Year College/Associate Degree	3	4.05
9 Four-Year College/Bachelor's Degree	2	2.70
997 Don't Know	4	5.41
999 Refuse to Answer	1	1.35
Total	74	100.00

cb052_w3_8_: [XChildName[8]]'s Highest Degree

	Freq.	%
1 No Formal Education(Illiterate)	2	6.90
2 Did not Finish Primary School	6	20.69
4 Elementary School	8	27.59
5 Middle School	3	10.34
7 Vocational School	2	6.90
8 Two-/Three-Year College/Associate Degree	3	10.34
997 Don't Know	4	13.79
999 Refuse to Answer	1	3.45
Total	29	100.00

cb052_w3_9_: [XChildName[9]]'s Highest Degree

	Freq.	%
1 No Formal Education(Illiterate)	2	14.29
2 Did not Finish Primary School	2	14.29
4 Elementary School	5	35.71
5 Middle School	1	7.14
6 High School	1	7.14
9 Four-Year College/Bachelor's Degree	1	7.14
997 Don't Know	2	14.29
Total	14	100.00

cb052_w3_10_: [XChildName[10]]'s Highest Degree

	Freq.	%
2 Did not Finish Primary School	3	30.00
4 Elementary School	4	40.00
5 Middle School	2	20.00
997 Don't Know	1	10.00
Total	10	100.00

cb052_w3_11_: [XChildName[11]]'s Highest Degree

	Freq.	%
1 No Formal Education(Illiterate)	2	16.67
2 Did not Finish Primary School	4	33.33
4 Elementary School	2	16.67
5 Middle School	2	16.67
997 Don't Know	2	16.67
Total	12	100.00

cb052_w3_12_: [XChildName[12]]'s Highest Degree

	Freq.	%
1 No Formal Education(Illiterate)	1	20.00
4 Elementary School	1	20.00
8 Two-/Three-Year College/Associate Degree	1	20.00
997 Don't Know	2	40.00
Total	5	100.00

cb052_w3_13_: [XChildName[13]]'s Highest Degree

	Freq.	%
4 Elementary School	1	100.00
Total	1	100.00

cb052_w3_14_: [XChildName[14]]'s Highest Degree

	Freq.	%
2 Did not Finish Primary School	1	100.00
Total	1	100.00

cb052_w3_15_: [XChildName[15]]'s Highest Degree

	Freq.	%
5 Middle School	1	100.00
Total	1	100.00

cb052_w3_1_1_: Is [XChildName[1]] Literate

	Freq.	%
1 Yes	134	62.91
2 No	79	37.09
Total	213	100.00

cb052_w3_1_2_: Is [XChildName[2]] Literate

	Freq.	%
1 Yes	215	71.19
2 No	87	28.81

Total	302	100.00

cb052_w3_1_3_: Is [XChildName[3]] Literate

	Freq.	%
1 Yes	134	59.56
2 No	91	40.44
Total	225	100.00

cb052_w3_1_4_: Is [XChildName[4]] Literate

	Freq.	%
1 Yes	86	58.11
2 No	62	41.89
Total	148	100.00

cb052_w3_1_5_: Is [XChildName[5]] Literate

	Freq.	%
1 Yes	60	57.14
2 No	45	42.86
Total	105	100.00

cb052_w3_1_6_: Is [XChildName[6]] Literate

	Freq.	%
1 Yes	28	52.83
2 No	25	47.17
Total	53	100.00

cb052_w3_1_7_: Is [XChildName[7]] Literate

	Freq.	%
1 Yes	10	43.48
2 No	13	56.52
Total	23	100.00

cb052_w3_1_8_: Is [XChildName[8]] Literate

	Freq.	%
1 Yes	6	75.00
2 No	2	25.00
Total	8	100.00

cb052_w3_1_9_: Is [XChildName[9]] Literate

	Freq.	%
1 Yes	2	50.00
2 No	2	50.00

Total	4	100.00

cb052_w3_1_10_: Is [XChildName[10]] Literate

	Freq.	%
1 Yes	3	100.00
Total	3	100.00

cb052_w3_1_11_: Is [XChildName[11]] Literate

	Freq.	%
1 Yes	5	83.33
2 No	1	16.67
Total	6	100.00

cb052_w3_1_12_: Is [XChildName[12]] Literate

	Freq.	%
2 No	1	100.00
Total	1	100.00

cb052_w3_1_13_: Is [XChildName[13]] Literate

No Observations

cb052_w3_1_14_: Is [XChildName[14]] Literate

	Freq.	%
1 Yes	1	100.00
Total	1	100.00

cb053_1_: Where is [XChildName[1]] Living Regularly

	Freq.	%
1 Livng with Respondent	1,616	14.69
2 Livng with Respondent but Financially Independent	1,449	13.18
3 Living in the Same or Nearby Courtyard House(Apartment) with Respondent	1,021	9.28
4 Others	6,468	58.81
5 Abroad	48	0.44
997 Don't Know	376	3.42
999 Refuse to Answer	20	0.18
Total	10,998	100.00

cb053_2_: Where is [XChildName[2]] Living Regularly

	Freq.	%
1 Livng with Respondent	1,017	11.17
2 Livng with Respondent but Financially Independent	722	7.93

3 Living in the Same or Nearby Courtyard House(Apartment) with Respondent	940	10.32
4 Others	6,016	66.05
5 Abroad	40	0.44
997 Don't Know	351	3.85
999 Refuse to Answer	22	0.24
Total	9,108	100.00

cb053_3_: Where is [XChildName[3]] Living Regularly

	Freq.	%
1 Livng with Respondent	326	6.35
2 Livng with Respondent but Financially Independent	308	6.00
3 Living in the Same or Nearby Courtyard House(Apartment) with Respondent	557	10.85
4 Others	3,691	71.92
5 Abroad	16	0.31
997 Don't Know	220	4.29
999 Refuse to Answer	14	0.27
Total	5,132	100.00

cb053_4_: Where is [XChildName[4]] Living Regularly

	Freq.	%
1 Livng with Respondent	115	4.22
2 Livng with Respondent but Financially Independent	117	4.29
3 Living in the Same or Nearby Courtyard House(Apartment) with Respondent	320	11.73
4 Others	2,004	73.46
5 Abroad	9	0.33
997 Don't Know	154	5.65
999 Refuse to Answer	9	0.33
Total	2,728	100.00

cb053_5_: Where is [XChildName[5]] Living Regularly

	Freq.	%
1 Livng with Respondent	42	3.20
2 Livng with Respondent but Financially Independent	59	4.50
3 Living in the Same or Nearby Courtyard House(Apartment) with Respondent	138	10.52
4 Others	1,000	76.22
5 Abroad	2	0.15
997 Don't Know	66	5.03
999 Refuse to Answer	5	0.38
Total	1,312	100.00

cb053_6_: Where is [XChildName[6]] Living Regularly

	Freq.	%
1 Livng with Respondent	20	3.43
2 Livng with Respondent but Financially Independent	13	2.23
3 Living in the Same or Nearby Courtyard House(Apartment) with Respondent	66	11.32

	Freq.	%
4 Others	443	75.99
5 Abroad	2	0.34
997 Don't Know	36	6.17
999 Refuse to Answer	3	0.51
Total	583	100.00

cb053_7_: Where is [XChildName[7]] Living Regularly

	Freq.	%
1 Livng with Respondent	6	2.14
2 Livng with Respondent but Financially Independent	18	6.43
3 Living in the Same or Nearby Courtyard House(Apartment) with Respondent	30	10.71
4 Others	207	73.93
5 Abroad	1	0.36
997 Don't Know	17	6.07
999 Refuse to Answer	1	0.36
Total	280	100.00

cb053_8_: Where is [XChildName[8]] Living Regularly

	Freq.	%
1 Livng with Respondent	4	3.08
2 Livng with Respondent but Financially Independent	5	3.85
3 Living in the Same or Nearby Courtyard House(Apartment) with Respondent	11	8.46
4 Others	93	71.54
997 Don't Know	16	12.31
999 Refuse to Answer	1	0.77
Total	130	100.00

cb053_9_: Where is [XChildName[9]] Living Regularly

	Freq.	%
1 Livng with Respondent	1	1.79
2 Livng with Respondent but Financially Independent	1	1.79
3 Living in the Same or Nearby Courtyard House(Apartment) with Respondent	7	12.50
4 Others	38	67.86
997 Don't Know	8	14.29
999 Refuse to Answer	1	1.79
Total	56	100.00

cb053_10_: Where is [XChildName[10]] Living Regularly

	Freq.	%
1 Livng with Respondent	1	3.13
3 Living in the Same or Nearby Courtyard House(Apartment) with Respondent	8	25.00
4 Others	17	53.13
997 Don't Know	6	18.75
Total	32	100.00

cb053_11_: Where is [XChildName[11]] Living Regularly

	Freq.	%
1 Livng with Respondent	1	5.26
2 Livng with Respondent but Financially Independent	1	5.26
3 Living in the Same or Nearby Courtyard House(Apartment) with Respondent	2	10.53
4 Others	10	52.63
997 Don't Know	5	26.32
Total	19	100.00

cb053_12_: Where is [XChildName[12]] Living Regularly

	Freq.	%
3 Living in the Same or Nearby Courtyard House(Apartment) with Respondent	1	10.00
4 Others	5	50.00
997 Don't Know	4	40.00
Total	10	100.00

cb053_13_: Where is [XChildName[13]] Living Regularly

	Freq.	%
4 Others	2	100.00
Total	2	100.00

cb053_14_: Where is [XChildName[14]] Living Regularly

	Freq.	%
4 Others	2	100.00
Total	2	100.00

cb053_15_: Where is [XChildName[15]] Living Regularly

	Freq.	%
997 Don't Know	1	100.00
Total	1	100.00

cb054_1_: Type of Living Place for [XChildName[1]]

	Freq.	%
1 Center Area of City or Town	3,017	46.30
2 Urban-suburban-integration Area	887	13.61
3 Rural Area	2,099	32.21
4 Special Area	80	1.23
997 Don't Know	432	6.63
999 Refuse to Answer	1	0.02
Total	6,516	100.00

cb054_2_: Type of Living Place for [XChildName[2]]

	Freq.	%
1 Center Area of City or Town	2,423	40.01
2 Urban-suburban-integration Area	769	12.70
3 Rural Area	2,427	40.08
4 Special Area	84	1.39
997 Don't Know	352	5.81
999 Refuse to Answer	1	0.02
Total	6,056	100.00

cb054_3_: Type of Living Place for [XChildName[3]]

	Freq.	%
1 Center Area of City or Town	1,309	35.31
2 Urban-suburban-integration Area	393	10.60
3 Rural Area	1,773	47.83
4 Special Area	38	1.03
997 Don't Know	193	5.21
999 Refuse to Answer	1	0.03
Total	3,707	100.00

cb054_4_: Type of Living Place for [XChildName[4]]

	Freq.	%
1 Center Area of City or Town	676	33.58
2 Urban-suburban-integration Area	185	9.19
3 Rural Area	1,039	51.61
4 Special Area	18	0.89
997 Don't Know	95	4.72
Total	2,013	100.00

cb054_5_: Type of Living Place for [XChildName[5]]

	Freq.	%
1 Center Area of City or Town	296	29.54
2 Urban-suburban-integration Area	97	9.68
3 Rural Area	559	55.79
4 Special Area	13	1.30
997 Don't Know	37	3.69
Total	1,002	100.00

cb054_6_: Type of Living Place for [XChildName[6]]

	Freq.	%
1 Center Area of City or Town	118	26.52
2 Urban-suburban-integration Area	37	8.31
3 Rural Area	268	60.22
4 Special Area	3	0.67
997 Don't Know	19	4.27
Total	445	100.00

cb054_7_: Type of Living Place for [XChildName[7]]

	Freq.	%
1 Center Area of City or Town	57	27.40
2 Urban-suburban-integration Area	17	8.17
3 Rural Area	124	59.62
4 Special Area	2	0.96
997 Don't Know	8	3.85
Total	208	100.00

cb054_8_: Type of Living Place for [XChildName[8]]

	Freq.	%
1 Center Area of City or Town	20	21.51
2 Urban-suburban-integration Area	6	6.45
3 Rural Area	59	63.44
997 Don't Know	8	8.60
Total	93	100.00

cb054_9_: Type of Living Place for [XChildName[9]]

	Freq.	%
1 Center Area of City or Town	7	18.42
2 Urban-suburban-integration Area	3	7.89
3 Rural Area	23	60.53
4 Special Area	1	2.63
997 Don't Know	4	10.53
Total	38	100.00

cb054_10_: Type of Living Place for [XChildName[10]]

	Freq.	%
1 Center Area of City or Town	4	23.53
2 Urban-suburban-integration Area	3	17.65
3 Rural Area	9	52.94
997 Don't Know	1	5.88
Total	17	100.00

cb054_11_: Type of Living Place for [XChildName[11]]

	Freq.	%
1 Center Area of City or Town	4	40.00
2 Urban-suburban-integration Area	1	10.00
3 Rural Area	5	50.00
Total	10	100.00

cb054_12_: Type of Living Place for [XChildName[12]]

	Freq.	%
1 Center Area of City or Town	2	40.00
2 Urban-suburban-integration Area	2	40.00
3 Rural Area	1	20.00

Total	5	100.00

cb054_13_: Type of Living Place for [XChildName[13]]

	Freq.	%
1 Center Area of City or Town	1	50.00
3 Rural Area	1	50.00
Total	2	100.00

cb054_14_: Type of Living Place for [XChildName[14]]

	Freq.	%
1 Center Area of City or Town	1	50.00
3 Rural Area	1	50.00
Total	2	100.00

cb055_1_: Type of Hukou for [XChildName[1]]

	Freq.	%
1 Agriculture Hukou	7,837	71.26
2 Non-Agriculture Hukou	2,673	24.30
3 Unified Residency Hukou	348	3.16
4 Do not Have Hukou	8	0.07
997 Don't Know	128	1.16
999 Refuse to Answer	4	0.04
Total	10,998	100.00

cb055_2_: Type of Hukou for [XChildName[2]]

	Freq.	%
1 Agriculture Hukou	6,925	76.03
2 Non-Agriculture Hukou	1,768	19.41
3 Unified Residency Hukou	270	2.96
4 Do not Have Hukou	9	0.10
997 Don't Know	127	1.39
999 Refuse to Answer	9	0.10
Total	9,108	100.00

cb055_3_: Type of Hukou for [XChildName[3]]

	Freq.	%
1 Agriculture Hukou	3,967	77.30
2 Non-Agriculture Hukou	928	18.08
3 Unified Residency Hukou	133	2.59
4 Do not Have Hukou	1	0.02
997 Don't Know	98	1.91
999 Refuse to Answer	5	0.10
Total	5,132	100.00

cb055_4_: Type of Hukou for [XChildName[4]]

	Freq.	%
1 Agriculture Hukou	2,129	78.04
2 Non-Agriculture Hukou	460	16.86
3 Unified Residency Hukou	74	2.71
4 Do not Have Hukou	2	0.07
997 Don't Know	55	2.02
999 Refuse to Answer	8	0.29
Total	2,728	100.00

cb055_5_: Type of Hukou for [XChildName[5]]

	Freq.	%
1 Agriculture Hukou	1,054	80.34
2 Non-Agriculture Hukou	183	13.95
3 Unified Residency Hukou	30	2.29
997 Don't Know	42	3.20
999 Refuse to Answer	3	0.23
Total	1,312	100.00

cb055_6_: Type of Hukou for [XChildName[6]]

	Freq.	%
1 Agriculture Hukou	468	80.27
2 Non-Agriculture Hukou	81	13.89
3 Unified Residency Hukou	15	2.57
997 Don't Know	17	2.92
999 Refuse to Answer	2	0.34
Total	583	100.00

cb055_7_: Type of Hukou for [XChildName[7]]

	Freq.	%
1 Agriculture Hukou	224	80.00
2 Non-Agriculture Hukou	37	13.21
3 Unified Residency Hukou	6	2.14
997 Don't Know	12	4.29
999 Refuse to Answer	1	0.36
Total	280	100.00

cb055_8_: Type of Hukou for [XChildName[8]]

	Freq.	%
1 Agriculture Hukou	110	84.62
2 Non-Agriculture Hukou	14	10.77
3 Unified Residency Hukou	2	1.54
997 Don't Know	3	2.31
999 Refuse to Answer	1	0.77
Total	130	100.00

cb055_9_: Type of Hukou for [XChildName[9]]

	Freq.	%
1 Agriculture Hukou	42	75.00
2 Non-Agriculture Hukou	10	17.86
3 Unified Residency Hukou	2	3.57
997 Don't Know	2	3.57
Total	56	100.00

cb055_10_: Type of Hukou for [XChildName[10]]

	Freq.	%
1 Agriculture Hukou	25	78.13
2 Non-Agriculture Hukou	4	12.50
3 Unified Residency Hukou	1	3.13
997 Don't Know	2	6.25
Total	32	100.00

cb055_11_: Type of Hukou for [XChildName[11]]

	Freq.	%
1 Agriculture Hukou	13	68.42
2 Non-Agriculture Hukou	3	15.79
997 Don't Know	3	15.79
Total	19	100.00

cb055_12_: Type of Hukou for [XChildName[12]]

	Freq.	%
1 Agriculture Hukou	7	70.00
2 Non-Agriculture Hukou	2	20.00
997 Don't Know	1	10.00
Total	10	100.00

cb055_13_: Type of Hukou for [XChildName[13]]

	Freq.	%
1 Agriculture Hukou	1	50.00
2 Non-Agriculture Hukou	1	50.00
Total	2	100.00

cb055_14_: Type of Hukou for [XChildName[14]]

	Freq.	%
1 Agriculture Hukou	1	50.00
2 Non-Agriculture Hukou	1	50.00
Total	2	100.00

cb055_15_: Type of Hukou for [XChildName[15]]

	Freq.	%
1 Agriculture Hukou	1	100.00
Total	1	100.00

cb055_w2_1_1_: Type of Hukou before Getting Unified Hukou

	Freq.	%
1 Agriculture Hukou	207	59.48
2 Non-Agriculture Hukou	94	27.01
3 Do not Have Hukou	24	6.90
997 Don't Know	22	6.32
999 Refuse to Answer	1	0.29
Total	348	100.00

cb055_w2_1_2_: Type of Hukou before Getting Unified Hukou

	Freq.	%
1 Agriculture Hukou	195	72.22
2 Non-Agriculture Hukou	45	16.67
3 Do not Have Hukou	14	5.19
997 Don't Know	16	5.93
Total	270	100.00

cb055_w2_1_3_: Type of Hukou before Getting Unified Hukou

	Freq.	%
1 Agriculture Hukou	104	78.20
2 Non-Agriculture Hukou	18	13.53
3 Do not Have Hukou	4	3.01
997 Don't Know	7	5.26
Total	133	100.00

cb055_w2_1_4_: Type of Hukou before Getting Unified Hukou

	Freq.	%
1 Agriculture Hukou	60	81.08
2 Non-Agriculture Hukou	6	8.11
3 Do not Have Hukou	3	4.05
997 Don't Know	5	6.76
Total	74	100.00

cb055_w2_1_5_: Type of Hukou before Getting Unified Hukou

	Freq.	%
1 Agriculture Hukou	26	86.67
2 Non-Agriculture Hukou	2	6.67
3 Do not Have Hukou	2	6.67
Total	30	100.00

cb055_w2_1_6_: Type of Hukou before Getting Unified Hukou

	Freq.	%
1 Agriculture Hukou	11	73.33
2 Non-Agriculture Hukou	2	13.33
997 Don't Know	2	13.33
Total	15	100.00

cb055_w2_1_7_: Type of Hukou before Getting Unified Hukou

	Freq.	%
1 Agriculture Hukou	5	83.33
997 Don't Know	1	16.67
Total	6	100.00

cb055_w2_1_8_: Type of Hukou before Getting Unified Hukou

	Freq.	%
1 Agriculture Hukou	1	50.00
997 Don't Know	1	50.00
Total	2	100.00

cb055_w2_1_9_: Type of Hukou before Getting Unified Hukou

	Freq.	%
1 Agriculture Hukou	1	50.00
997 Don't Know	1	50.00
Total	2	100.00

cb055_w2_1_10_: Type of Hukou before Getting Unified Hukou

	Freq.	%
997 Don't Know	1	100.00
Total	1	100.00

cb055_w2_2_1_: When did [XChildName[1]] Get Unified Hukou

Mean	SD	Min	Max	Obs
1,997.32	15.34	1,900.00	2,018.00	335

cb055_w2_2_2_: When did [XChildName[2]] Get Unified Hukou

Mean	SD	Min	Max	Obs
1,999.80	14.33	1,960.00	2,018.00	257

cb055_w2_2_3_: When did [XChildName[3]] Get Unified Hukou

Mean	SD	Min	Max	Obs

1,998.46	19.35	1,900.00	2,018.00	125

cb055_w2_2_4_: When did [XChildName[4]] Get Unified Hukou

Mean	SD	Min	Max	Obs
1,998.72	15.20	1,956.00	2,017.00	68

cb055_w2_2_5_: When did [XChildName[5]] Get Unified Hukou

Mean	SD	Min	Max	Obs
1,992.42	22.79	1,900.00	2,016.00	24

cb055_w2_2_6_: When did [XChildName[6]] Get Unified Hukou

Mean	SD	Min	Max	Obs
2,001.85	12.67	1,978.00	2,018.00	13

cb055_w2_2_7_: When did [XChildName[7]] Get Unified Hukou

Mean	SD	Min	Max	Obs
2,003.17	12.22	1,988.00	2,015.00	6

cb055_w2_2_8_: When did [XChildName[8]] Get Unified Hukou

Mean	SD	Min	Max	Obs
2,003.00	14.14	1,993.00	2,013.00	2

cb055_w2_2_9_: When did [XChildName[9]] Get Unified Hukou

Mean	SD	Min	Max	Obs
2,014.50	2.12	2,013.00	2,016.00	2

cb055_w2_2_10_: When did [XChildName[10]] Get Unified Hukou

Mean	SD	Min	Max	Obs
2,013.00	.	2,013.00	2,013.00	1

cb057_1_: Current Location of [XChildName[1]]'s Hukou

	Freq.	%
1 His/Her Birthplace	6,113	55.62
2 His/Her Current Living Place	3,595	32.71
3 Others	1,159	10.55
997 Don't Know	117	1.06
999 Refuse to Answer	6	0.05
Total	10,990	100.00

cb057_2_: Current Location of [XChildName[2]]'s Hukou

	Freq.	%
1 His/Her Birthplace	4,639	50.98
2 His/Her Current Living Place	3,297	36.23
3 Others	1,007	11.07
997 Don't Know	145	1.59
999 Refuse to Answer	11	0.12
Total	9,099	100.00

cb057_3_: Current Location of [XChildName[3]]'s Hukou

	Freq.	%
1 His/Her Birthplace	2,211	43.09
2 His/Her Current Living Place	2,209	43.05
3 Others	611	11.91
997 Don't Know	92	1.79
999 Refuse to Answer	8	0.16
Total	5,131	100.00

cb057_4_: Current Location of [XChildName[4]]'s Hukou

	Freq.	%
1 His/Her Birthplace	1,013	37.16
2 His/Her Current Living Place	1,310	48.06
3 Others	324	11.89
997 Don't Know	70	2.57
999 Refuse to Answer	9	0.33
Total	2,726	100.00

cb057_5_: Current Location of [XChildName[5]]'s Hukou

	Freq.	%
1 His/Her Birthplace	451	34.38
2 His/Her Current Living Place	666	50.76
3 Others	161	12.27
997 Don't Know	30	2.29
999 Refuse to Answer	4	0.30
Total	1,312	100.00

cb057_6_: Current Location of [XChildName[6]]'s Hukou

	Freq.	%
1 His/Her Birthplace	181	31.05
2 His/Her Current Living Place	308	52.83
3 Others	76	13.04
997 Don't Know	16	2.74
999 Refuse to Answer	2	0.34
Total	583	100.00

cb057_7_: Current Location of [XChildName[7]]'s Hukou

	Freq.	%
1 His/Her Birthplace	80	28.57
2 His/Her Current Living Place	153	54.64
3 Others	35	12.50
997 Don't Know	11	3.93
999 Refuse to Answer	1	0.36
Total	280	100.00

cb057_8_: Current Location of [XChildName[8]]'s Hukou

	Freq.	%
1 His/Her Birthplace	38	29.23
2 His/Her Current Living Place	68	52.31
3 Others	16	12.31
997 Don't Know	7	5.38
999 Refuse to Answer	1	0.77
Total	130	100.00

cb057_9_: Current Location of [XChildName[9]]'s Hukou

	Freq.	%
1 His/Her Birthplace	15	26.79
2 His/Her Current Living Place	28	50.00
3 Others	8	14.29
997 Don't Know	4	7.14
999 Refuse to Answer	1	1.79
Total	56	100.00

cb057_10_: Current Location of [XChildName[10]]'s Hukou

	Freq.	%
1 His/Her Birthplace	7	21.88
2 His/Her Current Living Place	19	59.38
3 Others	4	12.50
997 Don't Know	2	6.25
Total	32	100.00

cb057_11_: Current Location of [XChildName[11]]'s Hukou

	Freq.	%
1 His/Her Birthplace	3	15.79
2 His/Her Current Living Place	12	63.16
997 Don't Know	4	21.05
Total	19	100.00

cb057_12_: Current Location of [XChildName[12]]'s Hukou

	Freq.	%
1 His/Her Birthplace	3	30.00
2 His/Her Current Living Place	6	60.00

	Freq.	%
997 Don't Know	1	10.00
Total	10	100.00

cb057_13_: Current Location of [XChildName[13]]'s Hukou

	Freq.	%
2 His/Her Current Living Place	2	100.00
Total	2	100.00

cb057_14_: Current Location of [XChildName[14]]'s Hukou

	Freq.	%
2 His/Her Current Living Place	2	100.00
Total	2	100.00

cb057_15_: Current Location of [XChildName[15]]'s Hukou

	Freq.	%
2 His/Her Current Living Place	1	100.00
Total	1	100.00

cb063_w3_2_1_: Is [XChildName[1]] Communist Party Member

	Freq.	%
1 Yes	1,113	10.12
2 No	9,662	87.86
997 Don't Know	219	1.99
999 Refuse to Answer	3	0.03
Total	10,997	100.00

cb063_w3_2_2_: Is [XChildName[2]] Communist Party Member

	Freq.	%
1 Yes	703	7.72
2 No	8,236	90.43
997 Don't Know	162	1.78
999 Refuse to Answer	7	0.08
Total	9,108	100.00

cb063_w3_2_3_: Is [XChildName[3]] Communist Party Member

	Freq.	%
1 Yes	359	7.00
2 No	4,672	91.04
997 Don't Know	96	1.87
999 Refuse to Answer	5	0.10
Total	5,132	100.00

cb063_w3_2_4_: Is [XChildName[4]] Communist Party Member

	Freq.	%
1 Yes	147	5.39
2 No	2,515	92.19
997 Don't Know	59	2.16
999 Refuse to Answer	7	0.26
Total	2,728	100.00

cb063_w3_2_5_: Is [XChildName[5]] Communist Party Member

	Freq.	%
1 Yes	70	5.34
2 No	1,214	92.53
997 Don't Know	25	1.91
999 Refuse to Answer	3	0.23
Total	1,312	100.00

cb063_w3_2_6_: Is [XChildName[6]] Communist Party Member

	Freq.	%
1 Yes	15	2.57
2 No	553	94.85
997 Don't Know	13	2.23
999 Refuse to Answer	2	0.34
Total	583	100.00

cb063_w3_2_7_: Is [XChildName[7]] Communist Party Member

	Freq.	%
1 Yes	6	2.14
2 No	263	93.93
997 Don't Know	10	3.57
999 Refuse to Answer	1	0.36
Total	280	100.00

cb063_w3_2_8_: Is [XChildName[8]] Communist Party Member

	Freq.	%
1 Yes	3	2.31
2 No	121	93.08
997 Don't Know	5	3.85
999 Refuse to Answer	1	0.77
Total	130	100.00

cb063_w3_2_9_: Is [XChildName[9]] Communist Party Member

	Freq.	%
1 Yes	3	5.36
2 No	51	91.07
997 Don't Know	1	1.79
999 Refuse to Answer	1	1.79

	Freq.	%
Total	56	100.00

cb063_w3_2_10_: Is [XChildName[10]] Communist Party Member

	Freq.	%
2 No	29	90.63
997 Don't Know	3	9.38
Total	32	100.00

cb063_w3_2_11_: Is [XChildName[11]] Communist Party Member

	Freq.	%
2 No	17	89.47
997 Don't Know	2	10.53
Total	19	100.00

cb063_w3_2_12_: Is [XChildName[12]] Communist Party Member

	Freq.	%
2 No	9	90.00
997 Don't Know	1	10.00
Total	10	100.00

cb063_w3_2_13_: Is [XChildName[13]] Communist Party Member

	Freq.	%
2 No	2	100.00
Total	2	100.00

cb063_w3_2_14_: Is [XChildName[14]] Communist Party Member

	Freq.	%
2 No	2	100.00
Total	2	100.00

cb063_w3_2_15_: Is [XChildName[15]] Communist Party Member

	Freq.	%
2 No	1	100.00
Total	1	100.00

cb063_w3_5_1_: [XChildName[1]] Has Religious Beliefs

	Freq.	%
1 Yes	589	5.36
2 No	10,246	93.17
997 Don't Know	159	1.45
999 Refuse to Answer	3	0.03
Total	10,997	100.00

cb063_w3_5_2_: [XChildName[2]] Has Religious Beliefs

	Freq.	%
1 Yes	454	4.98
2 No	8,519	93.53
997 Don't Know	128	1.41
999 Refuse to Answer	7	0.08
Total	9,108	100.00

cb063_w3_5_3_: [XChildName[3]] Has Religious Beliefs

	Freq.	%
1 Yes	293	5.71
2 No	4,733	92.23
997 Don't Know	98	1.91
999 Refuse to Answer	8	0.16
Total	5,132	100.00

cb063_w3_5_4_: [XChildName[4]] Has Religious Beliefs

	Freq.	%
1 Yes	169	6.20
2 No	2,496	91.50
997 Don't Know	56	2.05
999 Refuse to Answer	7	0.26
Total	2,728	100.00

cb063_w3_5_5_: [XChildName[5]] Has Religious Beliefs

	Freq.	%
1 Yes	106	8.08
2 No	1,175	89.56
997 Don't Know	28	2.13
999 Refuse to Answer	3	0.23
Total	1,312	100.00

cb063_w3_5_6_: [XChildName[6]] Has Religious Beliefs

	Freq.	%
1 Yes	43	7.38
2 No	526	90.22
997 Don't Know	12	2.06
999 Refuse to Answer	2	0.34
Total	583	100.00

cb063_w3_5_7_: [XChildName[7]] Has Religious Beliefs

	Freq.	%
1 Yes	15	5.36
2 No	255	91.07
997 Don't Know	9	3.21

999 Refuse to Answer	1	0.36
Total	280	100.00

cb063_w3_5_8_: [XChildName[8]] Has Religious Beliefs

	Freq.	%
1 Yes	8	6.15
2 No	117	90.00
997 Don't Know	4	3.08
999 Refuse to Answer	1	0.77
Total	130	100.00

cb063_w3_5_9_: [XChildName[9]] Has Religious Beliefs

	Freq.	%
1 Yes	4	7.14
2 No	50	89.29
997 Don't Know	1	1.79
999 Refuse to Answer	1	1.79
Total	56	100.00

cb063_w3_5_10_: [XChildName[10]] Has Religious Beliefs

	Freq.	%
1 Yes	1	3.13
2 No	29	90.63
997 Don't Know	2	6.25
Total	32	100.00

cb063_w3_5_11_: [XChildName[11]] Has Religious Beliefs

	Freq.	%
2 No	17	89.47
997 Don't Know	2	10.53
Total	19	100.00

cb063_w3_5_12_: [XChildName[12]] Has Religious Beliefs

	Freq.	%
2 No	9	90.00
997 Don't Know	1	10.00
Total	10	100.00

cb063_w3_5_13_: [XChildName[13]] Has Religious Beliefs

	Freq.	%
2 No	2	100.00
Total	2	100.00

cb063_w3_5_14_: [XChildName[14]] Has Religious Beliefs

	Freq.	%
2 No	2	100.00
Total	2	100.00

cb063_w3_5_15_: [XChildName[15]] Has Religious Beliefs

	Freq.	%
2 No	1	100.00
Total	1	100.00

cb063_w4_6_1_: Which Religious Belief

	Freq.	%
1 Buddhism	380	64.52
2 Taoism	8	1.36
3 Islamism	37	6.28
4 Catholicism	30	5.09
5 Christianity	101	17.15
6 Others	33	5.60
Total	589	100.00

cb063_w4_6_2_: Which Religious Belief

	Freq.	%
1 Buddhism	298	65.64
2 Taoism	5	1.10
3 Islamism	24	5.29
4 Catholicism	26	5.73
5 Christianity	71	15.64
6 Others	30	6.61
Total	454	100.00

cb063_w4_6_3_: Which Religious Belief

	Freq.	%
1 Buddhism	190	64.85
2 Taoism	4	1.37
3 Islamism	13	4.44
4 Catholicism	16	5.46
5 Christianity	46	15.70
6 Others	24	8.19
Total	293	100.00

cb063_w4_6_4_: Which Religious Belief

	Freq.	%
1 Buddhism	125	73.96
2 Taoism	1	0.59
3 Islamism	6	3.55

4 Catholicism	4	2.37
5 Christianity	22	13.02
6 Others	11	6.51
Total	169	100.00

cb063_w4_6_5_: Which Religious Belief

	Freq.	%
1 Buddhism	82	77.36
3 Islamism	5	4.72
4 Catholicism	4	3.77
5 Christianity	10	9.43
6 Others	5	4.72
Total	106	100.00

cb063_w4_6_6_: Which Religious Belief

	Freq.	%
1 Buddhism	35	81.40
3 Islamism	2	4.65
4 Catholicism	2	4.65
5 Christianity	2	4.65
6 Others	2	4.65
Total	43	100.00

cb063_w4_6_7_: Which Religious Belief

	Freq.	%
1 Buddhism	10	66.67
4 Catholicism	1	6.67
5 Christianity	3	20.00
6 Others	1	6.67
Total	15	100.00

cb063_w4_6_8_: Which Religious Belief

	Freq.	%
1 Buddhism	5	62.50
5 Christianity	1	12.50
6 Others	2	25.00
Total	8	100.00

cb063_w4_6_9_: Which Religious Belief

	Freq.	%
1 Buddhism	4	100.00
Total	4	100.00

cb063_w4_6_10_: Which Religious Belief

	Freq.	%
1 Buddhism	1	100.00
Total	1	100.00

cb070_w4_1_: Is [XChildName[1]] Working or at School

	Freq.	%
1 Working	9,262	84.22
2 At School	496	4.51
3 At School while Working	21	0.19
4 Neither at School nor working	1,162	10.57
997 Don't Know	43	0.39
999 Refuse to Answer	13	0.12
Total	10,997	100.00

cb070_w4_2_: Is [XChildName[2]] Working or at School

	Freq.	%
1 Working	7,214	79.21
2 At School	614	6.74
3 At School while Working	14	0.15
4 Neither at School nor working	1,220	13.39
997 Don't Know	34	0.37
999 Refuse to Answer	12	0.13
Total	9,108	100.00

cb070_w4_3_: Is [XChildName[3]] Working or at School

	Freq.	%
1 Working	4,173	81.31
2 At School	151	2.94
3 At School while Working	5	0.10
4 Neither at School nor working	753	14.67
997 Don't Know	39	0.76
999 Refuse to Answer	11	0.21
Total	5,132	100.00

cb070_w4_4_: Is [XChildName[4]] Working or at School

	Freq.	%
1 Working	2,224	81.52
2 At School	56	2.05
4 Neither at School nor working	412	15.10
997 Don't Know	29	1.06
999 Refuse to Answer	7	0.26
Total	2,728	100.00

cb070_w4_5_: Is [XChildName[5]] Working or at School

	Freq.	%

1 Working	1,078	82.16
2 At School	17	1.30
4 Neither at School nor working	206	15.70
997 Don't Know	6	0.46
999 Refuse to Answer	5	0.38
Total	1,312	100.00

cb070_w4_6_: Is [XChildName[6]] Working or at School

	Freq.	%
1 Working	472	80.96
2 At School	4	0.69
4 Neither at School nor working	95	16.30
997 Don't Know	10	1.72
999 Refuse to Answer	2	0.34
Total	583	100.00

cb070_w4_7_: Is [XChildName[7]] Working or at School

	Freq.	%
1 Working	228	81.43
2 At School	4	1.43
4 Neither at School nor working	42	15.00
997 Don't Know	4	1.43
999 Refuse to Answer	2	0.71
Total	280	100.00

cb070_w4_8_: Is [XChildName[8]] Working or at School

	Freq.	%
1 Working	100	76.92
2 At School	3	2.31
4 Neither at School nor working	22	16.92
997 Don't Know	4	3.08
999 Refuse to Answer	1	0.77
Total	130	100.00

cb070_w4_9_: Is [XChildName[9]] Working or at School

	Freq.	%
1 Working	44	78.57
4 Neither at School nor working	9	16.07
997 Don't Know	3	5.36
Total	56	100.00

cb070_w4_10_: Is [XChildName[10]] Working or at School

	Freq.	%
1 Working	28	87.50
4 Neither at School nor working	3	9.38
997 Don't Know	1	3.13
Total	32	100.00

cb070_w4_11_: Is [XChildName[11]] Working or at School

	Freq.	%
1 Working	13	68.42
4 Neither at School nor working	4	21.05
997 Don't Know	2	10.53
Total	19	100.00

cb070_w4_12_: Is [XChildName[12]] Working or at School

	Freq.	%
1 Working	8	80.00
997 Don't Know	2	20.00
Total	10	100.00

cb070_w4_13_: Is [XChildName[13]] Working or at School

	Freq.	%
1 Working	1	50.00
4 Neither at School nor working	1	50.00
Total	2	100.00

cb070_w4_14_: Is [XChildName[14]] Working or at School

	Freq.	%
4 Neither at School nor working	2	100.00
Total	2	100.00

cb070_w4_15_: Is [XChildName[15]] Working or at School

	Freq.	%
1 Working	1	100.00
Total	1	100.00

cb071_1_: [XChildName[1]]'s Main Job

	Freq.	%
1 Managers	422	4.55
2 Professionals and Technicians	1,786	19.24
3 Clerks	574	6.18
4 Commercial and Service Workers	2,256	24.31
5 Agricultural Forestry, Husbandry and Fishery Producers	1,445	15.57
6 Production and Transportation Workers	1,717	18.50
7 Others	1,082	11.66
Total	9,282	100.00

cb071_2_: [XChildName[2]]'s Main Job

	Freq.	%
1 Managers	277	3.83

2 Professionals and Technicians	1,188	16.44
3 Clerks	373	5.16
4 Commercial and Service Workers	1,925	26.63
5 Agricultural Forestry, Husbandry and Fishery Producers	1,358	18.79
6 Production and Transportation Workers	1,261	17.45
7 Others	846	11.70
Total	7,228	100.00

cb071_3_: [XChildName[3]]'s Main Job

	Freq.	%
1 Managers	120	2.87
2 Professionals and Technicians	599	14.34
3 Clerks	171	4.09
4 Commercial and Service Workers	1,052	25.18
5 Agricultural Forestry, Husbandry and Fishery Producers	1,018	24.37
6 Production and Transportation Workers	706	16.90
7 Others	512	12.25
Total	4,178	100.00

cb071_4_: [XChildName[4]]'s Main Job

	Freq.	%
1 Managers	65	2.92
2 Professionals and Technicians	253	11.38
3 Clerks	87	3.91
4 Commercial and Service Workers	535	24.06
5 Agricultural Forestry, Husbandry and Fishery Producers	644	28.96
6 Production and Transportation Workers	345	15.51
7 Others	295	13.26
Total	2,224	100.00

cb071_5_: [XChildName[5]]'s Main Job

	Freq.	%
1 Managers	22	2.04
2 Professionals and Technicians	105	9.74
3 Clerks	38	3.53
4 Commercial and Service Workers	229	21.24
5 Agricultural Forestry, Husbandry and Fishery Producers	408	37.85
6 Production and Transportation Workers	142	13.17
7 Others	134	12.43
Total	1,078	100.00

cb071_6_: [XChildName[6]]'s Main Job

	Freq.	%
1 Managers	7	1.48
2 Professionals and Technicians	47	9.96
3 Clerks	10	2.12
4 Commercial and Service Workers	91	19.28
5 Agricultural Forestry, Husbandry and Fishery Producers	182	38.56
6 Production and Transportation Workers	73	15.47

7 Others	62	13.14
Total	472	100.00

cb071_7_: [XChildName[7]]'s Main Job

	Freq.	%
1 Managers	6	2.63
2 Professionals and Technicians	24	10.53
3 Clerks	4	1.75
4 Commercial and Service Workers	42	18.42
5 Agricultural Forestry, Husbandry and Fishery Producers	93	40.79
6 Production and Transportation Workers	29	12.72
7 Others	30	13.16
Total	228	100.00

cb071_8_: [XChildName[8]]'s Main Job

	Freq.	%
2 Professionals and Technicians	10	10.00
3 Clerks	4	4.00
4 Commercial and Service Workers	16	16.00
5 Agricultural Forestry, Husbandry and Fishery Producers	36	36.00
6 Production and Transportation Workers	20	20.00
7 Others	14	14.00
Total	100	100.00

cb071_9_: [XChildName[9]]'s Main Job

	Freq.	%
1 Managers	1	2.27
3 Clerks	2	4.55
4 Commercial and Service Workers	9	20.45
5 Agricultural Forestry, Husbandry and Fishery Producers	22	50.00
6 Production and Transportation Workers	5	11.36
7 Others	5	11.36
Total	44	100.00

cb071_10_: [XChildName[10]]'s Main Job

	Freq.	%
2 Professionals and Technicians	2	7.14
3 Clerks	1	3.57
4 Commercial and Service Workers	2	7.14
5 Agricultural Forestry, Husbandry and Fishery Producers	15	53.57
6 Production and Transportation Workers	7	25.00
7 Others	1	3.57
Total	28	100.00

cb071_11_: [XChildName[11]]'s Main Job

	Freq.	%
2 Professionals and Technicians	1	7.69

	Freq.	%
4 Commercial and Service Workers	3	23.08
5 Agricultural Forestry, Husbandry and Fishery Producers	8	61.54
6 Production and Transportation Workers	1	7.69
Total	13	100.00

cb071_12_: [XChildName[12]]'s Main Job

	Freq.	%
2 Professionals and Technicians	1	12.50
4 Commercial and Service Workers	2	25.00
5 Agricultural Forestry, Husbandry and Fishery Producers	3	37.50
7 Others	2	25.00
Total	8	100.00

cb071_13_: [XChildName[13]]'s Main Job

	Freq.	%
5 Agricultural Forestry, Husbandry and Fishery Producers	1	100.00
Total	1	100.00

cb071_14_: [XChildName[14]]'s Main Job

No Observations

cb071_15_: [XChildName[15]]'s Main Job

	Freq.	%
7 Others	1	100.00
Total	1	100.00

cb059_w4_1_: Level of Schooling [XChildName[1]] Currently Enrolled in

	Freq.	%
1 Preschool Education	4	0.77
2 Primary School	32	6.19
3 Middle School	58	11.22
4 High School	79	15.28
5 College	251	48.55
6 Master's Degree	32	6.19
7 Doctoral Degree/Ph.D. Degree	5	0.97
8 Others	56	10.83
Total	517	100.00

cb059_w4_2_: Level of Schooling [XChildName[2]] Currently Enrolled in

	Freq.	%
1 Preschool Education	8	1.27
2 Primary School	112	17.83
3 Middle School	126	20.06
4 High School	155	24.68
5 College	147	23.41

	Freq.	%
6 Master's Degree	12	1.91
7 Doctoral Degree/Ph.D. Degree	5	0.80
8 Others	63	10.03
Total	628	100.00

cb059_w4_3_: Level of Schooling [XChildName[3]] Currently Enrolled in

	Freq.	%
1 Preschool Education	4	2.56
2 Primary School	37	23.72
3 Middle School	34	21.79
4 High School	37	23.72
5 College	29	18.59
6 Master's Degree	4	2.56
7 Doctoral Degree/Ph.D. Degree	1	0.64
8 Others	10	6.41
Total	156	100.00

cb059_w4_4_: Level of Schooling [XChildName[4]] Currently Enrolled in

	Freq.	%
1 Preschool Education	1	1.79
2 Primary School	14	25.00
3 Middle School	10	17.86
4 High School	14	25.00
5 College	8	14.29
6 Master's Degree	1	1.79
8 Others	8	14.29
Total	56	100.00

cb059_w4_5_: Level of Schooling [XChildName[5]] Currently Enrolled in

	Freq.	%
3 Middle School	4	23.53
4 High School	5	29.41
5 College	1	5.88
7 Doctoral Degree/Ph.D. Degree	1	5.88
8 Others	6	35.29
Total	17	100.00

cb059_w4_6_: Level of Schooling [XChildName[6]] Currently Enrolled in

	Freq.	%
3 Middle School	2	50.00
5 College	1	25.00
8 Others	1	25.00
Total	4	100.00

cb059_w4_7_: Level of Schooling [XChildName[7]] Currently Enrolled in

	Freq.	%
1 Preschool Education	1	25.00

2 Primary School	1	25.00
4 High School	1	25.00
8 Others	1	25.00
Total	4	100.00

cb059_w4_8_: Level of Schooling [XChildName[8]] Currently Enrolled in

	Freq.	%
1 Preschool Education	1	33.33
2 Primary School	2	66.67
Total	3	100.00

cb063_1_: [XChildName[1]]'s Marital Status

	Freq.	%
1 Married with Spouse Present	7,512	68.95
2 Married but not Living with Spouse Temporarily for Reasons Such as Work	702	6.44
3 Separated	48	0.44
4 Divorced	388	3.56
5 Widowed	101	0.93
6 Never Married	2,091	19.19
997 Don't Know	44	0.40
999 Refuse to Answer	9	0.08
Total	10,895	100.00

cb063_2_: [XChildName[2]]'s Marital Status

	Freq.	%
1 Married with Spouse Present	6,344	71.84
2 Married but not Living with Spouse Temporarily for Reasons Such as Work	591	6.69
3 Separated	33	0.37
4 Divorced	300	3.40
5 Widowed	92	1.04
6 Never Married	1,428	16.17
997 Don't Know	30	0.34
999 Refuse to Answer	13	0.15
Total	8,831	100.00

cb063_3_: [XChildName[3]]'s Marital Status

	Freq.	%
1 Married with Spouse Present	3,946	78.29
2 Married but not Living with Spouse Temporarily for Reasons Such as Work	316	6.27
3 Separated	20	0.40
4 Divorced	160	3.17
5 Widowed	68	1.35
6 Never Married	491	9.74
997 Don't Know	29	0.58
999 Refuse to Answer	10	0.20
Total	5,040	100.00

cb063_4_: [XChildName[4]]'s Marital Status

	Freq.	%
1 Married with Spouse Present	2,161	80.13
2 Married but not Living with Spouse Temporarily for Reasons Such as Work	167	6.19
3 Separated	6	0.22
4 Divorced	69	2.56
5 Widowed	54	2.00
6 Never Married	194	7.19
997 Don't Know	36	1.33
999 Refuse to Answer	10	0.37
Total	2,697	100.00

cb063_5_: [XChildName[5]]'s Marital Status

	Freq.	%
1 Married with Spouse Present	1,050	80.46
2 Married but not Living with Spouse Temporarily for Reasons Such as Work	112	8.58
3 Separated	2	0.15
4 Divorced	36	2.76
5 Widowed	26	1.99
6 Never Married	68	5.21
997 Don't Know	7	0.54
999 Refuse to Answer	4	0.31
Total	1,305	100.00

cb063_6_: [XChildName[6]]'s Marital Status

	Freq.	%
1 Married with Spouse Present	463	80.10
2 Married but not Living with Spouse Temporarily for Reasons Such as Work	37	6.40
3 Separated	8	1.38
4 Divorced	18	3.11
5 Widowed	19	3.29
6 Never Married	23	3.98
997 Don't Know	8	1.38
999 Refuse to Answer	2	0.35
Total	578	100.00

cb063_7_: [XChildName[7]]'s Marital Status

	Freq.	%
1 Married with Spouse Present	217	78.91
2 Married but not Living with Spouse Temporarily for Reasons Such as Work	17	6.18
4 Divorced	10	3.64
5 Widowed	9	3.27
6 Never Married	16	5.82
997 Don't Know	4	1.45
999 Refuse to Answer	2	0.73
Total	275	100.00

cb063_8_: [XChildName[8]]'s Marital Status

	Freq.	%
1 Married with Spouse Present	101	81.45
2 Married but not Living with Spouse Temporarily for Reasons Such as Work	7	5.65
4 Divorced	3	2.42
5 Widowed	3	2.42
6 Never Married	6	4.84
997 Don't Know	3	2.42
999 Refuse to Answer	1	0.81
Total	124	100.00

cb063_9_: [XChildName[9]]'s Marital Status

	Freq.	%
1 Married with Spouse Present	42	75.00
2 Married but not Living with Spouse Temporarily for Reasons Such as Work	6	10.71
4 Divorced	1	1.79
5 Widowed	2	3.57
6 Never Married	2	3.57
997 Don't Know	2	3.57
999 Refuse to Answer	1	1.79
Total	56	100.00

cb063_10_: [XChildName[10]]'s Marital Status

	Freq.	%
1 Married with Spouse Present	24	77.42
2 Married but not Living with Spouse Temporarily for Reasons Such as Work	2	6.45
4 Divorced	2	6.45
5 Widowed	1	3.23
997 Don't Know	2	6.45
Total	31	100.00

cb063_11_: [XChildName[11]]'s Marital Status

	Freq.	%
1 Married with Spouse Present	13	72.22
2 Married but not Living with Spouse Temporarily for Reasons Such as Work	2	11.11
997 Don't Know	3	16.67
Total	18	100.00

cb063_12_: [XChildName[12]]'s Marital Status

	Freq.	%
1 Married with Spouse Present	7	70.00
2 Married but not Living with Spouse Temporarily for Reasons Such as Work	1	10.00
997 Don't Know	2	20.00

Total	10	100.00

cb063_13_: [XChildName[13]]'s Marital Status

	Freq.	%
1 Married with Spouse Present	2	100.00
Total	2	100.00

cb063_14_: [XChildName[14]]'s Marital Status

	Freq.	%
1 Married with Spouse Present	2	100.00
Total	2	100.00

cb063_15_: [XChildName[15]]'s Marital Status

	Freq.	%
1 Married with Spouse Present	1	100.00
Total	1	100.00

cb063_w4_1_: [XChildName[1]] Have Cohabiting Partner

	Freq.	%
1 Yes	103	3.92
2 No	2,422	92.16
997 Don't Know	102	3.88
999 Refuse to Answer	1	0.04
Total	2,628	100.00

cb063_w4_2_: [XChildName[2]] Have Cohabiting Partner

	Freq.	%
1 Yes	64	3.45
2 No	1,715	92.55
997 Don't Know	74	3.99
Total	1,853	100.00

cb063_w4_3_: [XChildName[3]] Have Cohabiting Partner

	Freq.	%
1 Yes	26	3.52
2 No	678	91.75
997 Don't Know	35	4.74
Total	739	100.00

cb063_w4_4_: [XChildName[4]] Have Cohabiting Partner

	Freq.	%
1 Yes	15	4.64

	Freq.	%
2 No	292	90.40
997 Don't Know	16	4.95
Total	323	100.00

cb063_w4_5_: [XChildName[5]] Have Cohabiting Partner

	Freq.	%
1 Yes	5	3.79
2 No	118	89.39
997 Don't Know	9	6.82
Total	132	100.00

cb063_w4_6_: [XChildName[6]] Have Cohabiting Partner

	Freq.	%
1 Yes	2	2.94
2 No	66	97.06
Total	68	100.00

cb063_w4_7_: [XChildName[7]] Have Cohabiting Partner

	Freq.	%
1 Yes	3	8.57
2 No	31	88.57
997 Don't Know	1	2.86
Total	35	100.00

cb063_w4_8_: [XChildName[8]] Have Cohabiting Partner

	Freq.	%
2 No	12	100.00
Total	12	100.00

cb063_w4_9_: [XChildName[9]] Have Cohabiting Partner

	Freq.	%
2 No	4	80.00
997 Don't Know	1	20.00
Total	5	100.00

cb063_w4_10_: [XChildName[10]] Have Cohabiting Partner

	Freq.	%
2 No	2	66.67
997 Don't Know	1	33.33
Total	3	100.00

cb090_w4_1_: Living Place of [XChildName[1]]'s Spouse

	Freq.	%
1 Living with [XChildName[1]]	7,553	90.81
2 Others	663	7.97
997 Don't Know	96	1.15
999 Refuse to Answer	5	0.06
Total	8,317	100.00

cb090_w4_2_: Living Place of [XChildName[2]]'s Spouse

	Freq.	%
1 Living with [XChildName[2]]	6,365	90.94
2 Others	548	7.83
997 Don't Know	85	1.21
999 Refuse to Answer	1	0.01
Total	6,999	100.00

cb090_w4_3_: Living Place of [XChildName[3]]'s Spouse

	Freq.	%
1 Living with [XChildName[3]]	3,943	91.95
2 Others	294	6.86
997 Don't Know	49	1.14
999 Refuse to Answer	2	0.05
Total	4,288	100.00

cb090_w4_4_: Living Place of [XChildName[4]]'s Spouse

	Freq.	%
1 Living with [XChildName[4]]	2,149	91.72
2 Others	162	6.91
997 Don't Know	31	1.32
999 Refuse to Answer	1	0.04
Total	2,343	100.00

cb090_w4_5_: Living Place of [XChildName[5]]'s Spouse

	Freq.	%
1 Living with [XChildName[5]]	1,051	90.06
2 Others	98	8.40
997 Don't Know	18	1.54
Total	1,167	100.00

cb090_w4_6_: Living Place of [XChildName[6]]'s Spouse

	Freq.	%
1 Living with [XChildName[6]]	460	91.63
2 Others	32	6.37
997 Don't Know	10	1.99
Total	502	100.00

cb090_w4_7_: Living Place of [XChildName[7]]'s Spouse

	Freq.	%
1 Living with [XChildName[7]]	218	91.98
2 Others	14	5.91
997 Don't Know	5	2.11
Total	237	100.00

cb090_w4_8_: Living Place of [XChildName[8]]'s Spouse

	Freq.	%
1 Living with [XChildName[8]]	102	94.44
2 Others	3	2.78
997 Don't Know	3	2.78
Total	108	100.00

cb090_w4_9_: Living Place of [XChildName[9]]'s Spouse

	Freq.	%
1 Living with [XChildName[9]]	42	87.50
2 Others	3	6.25
997 Don't Know	3	6.25
Total	48	100.00

cb090_w4_10_: Living Place of [XChildName[10]]'s Spouse

	Freq.	%
1 Living with [XChildName[10]]	24	92.31
997 Don't Know	2	7.69
Total	26	100.00

cb090_w4_11_: Living Place of [XChildName[11]]'s Spouse

	Freq.	%
1 Living with [XChildName[11]]	12	80.00
2 Others	1	6.67
997 Don't Know	2	13.33
Total	15	100.00

cb090_w4_12_: Living Place of [XChildName[12]]'s Spouse

	Freq.	%
1 Living with [XChildName[12]]	7	87.50
997 Don't Know	1	12.50
Total	8	100.00

cb090_w4_13_: Living Place of [XChildName[13]]'s Spouse

	Freq.	%

	Freq.	%
1 Living with [XChildName[13]]	2	100.00
Total	2	100.00

cb090_w4_14_: Living Place of [XChildName[14]]'s Spouse

	Freq.	%
1 Living with [XChildName[14]]	2	100.00
Total	2	100.00

cb090_w4_15_: Living Place of [XChildName[15]]'s Spouse

	Freq.	%
1 Living with [XChildName[15]]	1	100.00
Total	1	100.00

cb091_w4_1_: [XChildName[1]]'s Spouse's Highest Degree

	Freq.	%
1 No Formal Education(Illiterate)	430	5.17
2 Did not Finish Primary School	474	5.70
3 Sishu/Home School	1	0.01
4 Elementary School	1,312	15.78
5 Middle School	2,659	31.98
6 High School	819	9.85
7 Vocational School	432	5.20
8 Two-/Three-Year College/Associate Degree	544	6.54
9 Four-Year College/Bachelor's Degree	759	9.13
10 Post-graduate, Master's Degree	87	1.05
11 Post-graduate, Doctoral Degree/Ph.D	7	0.08
997 Don't Know	788	9.48
999 Refuse to Answer	3	0.04
Total	8,315	100.00

cb091_w4_2_: [XChildName[2]]'s Spouse's Highest Degree

	Freq.	%
1 No Formal Education(Illiterate)	379	5.42
2 Did not Finish Primary School	443	6.33
3 Sishu/Home School	1	0.01
4 Elementary School	1,151	16.45
5 Middle School	2,299	32.85
6 High School	678	9.69
7 Vocational School	306	4.37
8 Two-/Three-Year College/Associate Degree	354	5.06
9 Four-Year College/Bachelor's Degree	520	7.43
10 Post-graduate, Master's Degree	36	0.51
11 Post-graduate, Doctoral Degree/Ph.D	9	0.13
997 Don't Know	821	11.73
999 Refuse to Answer	2	0.03
Total	6,999	100.00

cb091_w4_3_: [XChildName[3]]'s Spouse's Highest Degree

	Freq.	%
1 No Formal Education(Illiterate)	258	6.02
2 Did not Finish Primary School	282	6.58
4 Elementary School	832	19.40
5 Middle School	1,399	32.63
6 High School	403	9.40
7 Vocational School	126	2.94
8 Two-/Three-Year College/Associate Degree	144	3.36
9 Four-Year College/Bachelor's Degree	231	5.39
10 Post-graduate, Master's Degree	22	0.51
11 Post-graduate, Doctoral Degree/Ph.D	6	0.14
997 Don't Know	581	13.55
999 Refuse to Answer	4	0.09
Total	4,288	100.00

cb091_w4_4_: [XChildName[4]]'s Spouse's Highest Degree

	Freq.	%
1 No Formal Education(Illiterate)	164	7.00
2 Did not Finish Primary School	178	7.60
4 Elementary School	477	20.36
5 Middle School	734	31.33
6 High School	212	9.05
7 Vocational School	63	2.69
8 Two-/Three-Year College/Associate Degree	52	2.22
9 Four-Year College/Bachelor's Degree	98	4.18
10 Post-graduate, Master's Degree	9	0.38
11 Post-graduate, Doctoral Degree/Ph.D	6	0.26
997 Don't Know	349	14.90
999 Refuse to Answer	1	0.04
Total	2,343	100.00

cb091_w4_5_: [XChildName[5]]'s Spouse's Highest Degree

	Freq.	%
1 No Formal Education(Illiterate)	91	7.80
2 Did not Finish Primary School	103	8.83
3 Sishu/Home School	1	0.09
4 Elementary School	263	22.54
5 Middle School	337	28.88
6 High School	102	8.74
7 Vocational School	26	2.23
8 Two-/Three-Year College/Associate Degree	20	1.71
9 Four-Year College/Bachelor's Degree	29	2.49
10 Post-graduate, Master's Degree	3	0.26
11 Post-graduate, Doctoral Degree/Ph.D	1	0.09
997 Don't Know	191	16.37
Total	1,167	100.00

cb091_w4_6_: [XChildName[6]]'s Spouse's Highest Degree

	Freq.	%
1 No Formal Education(Illiterate)	53	10.56
2 Did not Finish Primary School	42	8.37

	Freq.	%
4 Elementary School	105	20.92
5 Middle School	140	27.89
6 High School	47	9.36
7 Vocational School	7	1.39
8 Two-/Three-Year College/Associate Degree	5	1.00
9 Four-Year College/Bachelor's Degree	13	2.59
10 Post-graduate, Master's Degree	3	0.60
11 Post-graduate, Doctoral Degree/Ph.D	1	0.20
997 Don't Know	86	17.13
Total	502	100.00

cb091_w4_7_: [XChildName[7]]'s Spouse's Highest Degree

	Freq.	%
1 No Formal Education(Illiterate)	29	12.24
2 Did not Finish Primary School	21	8.86
4 Elementary School	47	19.83
5 Middle School	57	24.05
6 High School	24	10.13
7 Vocational School	5	2.11
8 Two-/Three-Year College/Associate Degree	3	1.27
9 Four-Year College/Bachelor's Degree	6	2.53
11 Post-graduate, Doctoral Degree/Ph.D	1	0.42
997 Don't Know	44	18.57
Total	237	100.00

cb091_w4_8_: [XChildName[8]]'s Spouse's Highest Degree

	Freq.	%
1 No Formal Education(Illiterate)	12	11.11
2 Did not Finish Primary School	10	9.26
4 Elementary School	30	27.78
5 Middle School	18	16.67
6 High School	9	8.33
8 Two-/Three-Year College/Associate Degree	3	2.78
9 Four-Year College/Bachelor's Degree	3	2.78
997 Don't Know	23	21.30
Total	108	100.00

cb091_w4_9_: [XChildName[9]]'s Spouse's Highest Degree

	Freq.	%
1 No Formal Education(Illiterate)	2	4.17
2 Did not Finish Primary School	5	10.42
4 Elementary School	12	25.00
5 Middle School	11	22.92
6 High School	4	8.33
7 Vocational School	2	4.17
9 Four-Year College/Bachelor's Degree	3	6.25
997 Don't Know	9	18.75
Total	48	100.00

cb091_w4_10_: [XChildName[10]]'s Spouse's Highest Degree

	Freq.	%
1 No Formal Education(Illiterate)	3	11.54
2 Did not Finish Primary School	3	11.54
4 Elementary School	9	34.62
5 Middle School	6	23.08
7 Vocational School	1	3.85
8 Two-/Three-Year College/Associate Degree	1	3.85
997 Don't Know	3	11.54
Total	26	100.00

cb091_w4_11_: [XChildName[11]]'s Spouse's Highest Degree

	Freq.	%
1 No Formal Education(Illiterate)	2	13.33
2 Did not Finish Primary School	4	26.67
4 Elementary School	3	20.00
5 Middle School	3	20.00
6 High School	1	6.67
7 Vocational School	1	6.67
997 Don't Know	1	6.67
Total	15	100.00

cb091_w4_12_: [XChildName[12]]'s Spouse's Highest Degree

	Freq.	%
1 No Formal Education(Illiterate)	1	12.50
2 Did not Finish Primary School	2	25.00
5 Middle School	3	37.50
997 Don't Know	2	25.00
Total	8	100.00

cb091_w4_13_: [XChildName[13]]'s Spouse's Highest Degree

	Freq.	%
2 Did not Finish Primary School	1	50.00
8 Two-/Three-Year College/Associate Degree	1	50.00
Total	2	100.00

cb091_w4_14_: [XChildName[14]]'s Spouse's Highest Degree

	Freq.	%
2 Did not Finish Primary School	1	50.00
6 High School	1	50.00
Total	2	100.00

cb091_w4_15_: [XChildName[15]]'s Spouse's Highest Degree

	Freq.	%
2 Did not Finish Primary School	1	100.00
Total	1	100.00

cb092_w4_1_: Is [XChildName[1]]'s Spouse Working Now

	Freq.	%
1 Yes	6,708	80.67
2 No	1,532	18.42
997 Don't Know	71	0.85
999 Refuse to Answer	4	0.05
Total	8,315	100.00

cb092_w4_2_: Is [XChildName[2]]'s Spouse Working Now

	Freq.	%
1 Yes	5,806	82.95
2 No	1,133	16.19
997 Don't Know	59	0.84
999 Refuse to Answer	1	0.01
Total	6,999	100.00

cb092_w4_3_: Is [XChildName[3]]'s Spouse Working Now

	Freq.	%
1 Yes	3,671	85.61
2 No	568	13.25
997 Don't Know	46	1.07
999 Refuse to Answer	3	0.07
Total	4,288	100.00

cb092_w4_4_: Is [XChildName[4]]'s Spouse Working Now

	Freq.	%
1 Yes	2,004	85.53
2 No	310	13.23
997 Don't Know	29	1.24
Total	2,343	100.00

cb092_w4_5_: Is [XChildName[5]]'s Spouse Working Now

	Freq.	%
1 Yes	1,010	86.55
2 No	149	12.77
997 Don't Know	8	0.69
Total	1,167	100.00

cb092_w4_6_: Is [XChildName[6]]'s Spouse Working Now

	Freq.	%
1 Yes	428	85.26
2 No	61	12.15
997 Don't Know	12	2.39
999 Refuse to Answer	1	0.20
Total	502	100.00

cb092_w4_7_: Is [XChildName[7]]'s Spouse Working Now

	Freq.	%
1 Yes	193	81.43
2 No	37	15.61
997 Don't Know	7	2.95
Total	237	100.00

cb092_w4_8_: Is [XChildName[8]]'s Spouse Working Now

	Freq.	%
1 Yes	85	78.70
2 No	21	19.44
997 Don't Know	2	1.85
Total	108	100.00

cb092_w4_9_: Is [XChildName[9]]'s Spouse Working Now

	Freq.	%
1 Yes	43	89.58
2 No	4	8.33
997 Don't Know	1	2.08
Total	48	100.00

cb092_w4_10_: Is [XChildName[10]]'s Spouse Working Now

	Freq.	%
1 Yes	24	92.31
2 No	1	3.85
997 Don't Know	1	3.85
Total	26	100.00

cb092_w4_11_: Is [XChildName[11]]'s Spouse Working Now

	Freq.	%
1 Yes	13	86.67
2 No	2	13.33
Total	15	100.00

cb092_w4_12_: Is [XChildName[12]]'s Spouse Working Now

	Freq.	%
1 Yes	7	87.50
2 No	1	12.50
Total	8	100.00

cb092_w4_13_: Is [XChildName[13]]'s Spouse Working Now

	Freq.	%
1 Yes	2	100.00

	Freq.	%
Total	2	100.00

cb092_w4_14_: Is [XChildName[14]]'s Spouse Working Now

	Freq.	%
1 Yes	1	50.00
2 No	1	50.00
Total	2	100.00

cb092_w4_15_: Is [XChildName[15]]'s Spouse Working Now

	Freq.	%
1 Yes	1	100.00
Total	1	100.00

cb093_w4_1_: [XChildName[1]]'s Spouse's Main Job

	Freq.	%
1 Managers	259	3.86
2 Professionals and Technicians	1,096	16.34
3 Clerks	396	5.90
4 Commercial and Service Workers	1,824	27.19
5 Agricultural Forestry, Husbandry and Fishery Producers	1,384	20.63
6 Production and Transportation Workers	1,014	15.12
7 Others	735	10.96
Total	6,708	100.00

cb093_w4_2_: [XChildName[2]]'s Spouse's Main Job

	Freq.	%
1 Managers	227	3.91
2 Professionals and Technicians	834	14.36
3 Clerks	332	5.72
4 Commercial and Service Workers	1,426	24.56
5 Agricultural Forestry, Husbandry and Fishery Producers	1,294	22.29
6 Production and Transportation Workers	996	17.15
7 Others	697	12.00
Total	5,806	100.00

cb093_w4_3_: [XChildName[3]]'s Spouse's Main Job

	Freq.	%
1 Managers	120	3.27
2 Professionals and Technicians	539	14.68
3 Clerks	153	4.17
4 Commercial and Service Workers	821	22.36
5 Agricultural Forestry, Husbandry and Fishery Producers	971	26.45
6 Production and Transportation Workers	630	17.16
7 Others	437	11.90
Total	3,671	100.00

cb093_w4_4_: [XChildName[4]]'s Spouse's Main Job

	Freq.	%
1 Managers	69	3.44
2 Professionals and Technicians	256	12.77
3 Clerks	70	3.49
4 Commercial and Service Workers	432	21.56
5 Agricultural Forestry, Husbandry and Fishery Producers	567	28.29
6 Production and Transportation Workers	329	16.42
7 Others	281	14.02
Total	2,004	100.00

cb093_w4_5_: [XChildName[5]]'s Spouse's Main Job

	Freq.	%
1 Managers	21	2.08
2 Professionals and Technicians	137	13.56
3 Clerks	34	3.37
4 Commercial and Service Workers	178	17.62
5 Agricultural Forestry, Husbandry and Fishery Producers	358	35.45
6 Production and Transportation Workers	162	16.04
7 Others	120	11.88
Total	1,010	100.00

cb093_w4_6_: [XChildName[6]]'s Spouse's Main Job

	Freq.	%
1 Managers	12	2.80
2 Professionals and Technicians	52	12.15
3 Clerks	11	2.57
4 Commercial and Service Workers	90	21.03
5 Agricultural Forestry, Husbandry and Fishery Producers	153	35.75
6 Production and Transportation Workers	71	16.59
7 Others	39	9.11
Total	428	100.00

cb093_w4_7_: [XChildName[7]]'s Spouse's Main Job

	Freq.	%
1 Managers	6	3.11
2 Professionals and Technicians	23	11.92
3 Clerks	4	2.07
4 Commercial and Service Workers	34	17.62
5 Agricultural Forestry, Husbandry and Fishery Producers	79	40.93
6 Production and Transportation Workers	29	15.03
7 Others	18	9.33
Total	193	100.00

cb093_w4_8_: [XChildName[8]]'s Spouse's Main Job

	Freq.	%
1 Managers	1	1.18
2 Professionals and Technicians	7	8.24

	Freq.	%
3 Clerks	2	2.35
4 Commercial and Service Workers	12	14.12
5 Agricultural Forestry, Husbandry and Fishery Producers	34	40.00
6 Production and Transportation Workers	17	20.00
7 Others	12	14.12
Total	85	100.00

cb093_w4_9_: [XChildName[9]]'s Spouse's Main Job

	Freq.	%
1 Managers	2	4.65
2 Professionals and Technicians	3	6.98
3 Clerks	2	4.65
4 Commercial and Service Workers	6	13.95
5 Agricultural Forestry, Husbandry and Fishery Producers	24	55.81
6 Production and Transportation Workers	4	9.30
7 Others	2	4.65
Total	43	100.00

cb093_w4_10_: [XChildName[10]]'s Spouse's Main Job

	Freq.	%
2 Professionals and Technicians	3	12.50
4 Commercial and Service Workers	3	12.50
5 Agricultural Forestry, Husbandry and Fishery Producers	13	54.17
6 Production and Transportation Workers	4	16.67
7 Others	1	4.17
Total	24	100.00

cb093_w4_11_: [XChildName[11]]'s Spouse's Main Job

	Freq.	%
2 Professionals and Technicians	2	15.38
5 Agricultural Forestry, Husbandry and Fishery Producers	7	53.85
6 Production and Transportation Workers	3	23.08
7 Others	1	7.69
Total	13	100.00

cb093_w4_12_: [XChildName[12]]'s Spouse's Main Job

	Freq.	%
4 Commercial and Service Workers	2	28.57
5 Agricultural Forestry, Husbandry and Fishery Producers	1	14.29
6 Production and Transportation Workers	2	28.57
7 Others	2	28.57
Total	7	100.00

cb093_w4_13_: [XChildName[13]]'s Spouse's Main Job

	Freq.	%
5 Agricultural Forestry, Husbandry and Fishery Producers	1	50.00
7 Others	1	50.00

Total	2	100.00

cb093_w4_14_: [XChildName[14]]'s Spouse's Main Job

	Freq.	%
5 Agricultural Forestry, Husbandry and Fishery Producers	1	100.00
Total	1	100.00

cb093_w4_15_: [XChildName[15]]'s Spouse's Main Job

	Freq.	%
4 Commercial and Service Workers	1	100.00
Total	1	100.00

cb065_1_: Number of [XChildName[1]]'s Children

Mean	SD	Min	Max	Obs
1.28	0.96	0.00	7.00	10,866

cb065_2_: Number of [XChildName[2]]'s Children

Mean	SD	Min	Max	Obs
1.35	0.93	0.00	9.00	8,804

cb065_3_: Number of [XChildName[3]]'s Children

Mean	SD	Min	Max	Obs
1.55	0.89	0.00	6.00	5,021

cb065_4_: Number of [XChildName[4]]'s Children

Mean	SD	Min	Max	Obs
1.67	0.91	0.00	8.00	2,671

cb065_5_: Number of [XChildName[5]]'s Children

Mean	SD	Min	Max	Obs
1.70	0.87	0.00	6.00	1,295

cb065_6_: Number of [XChildName[6]]'s Children

Mean	SD	Min	Max	Obs
1.83	0.98	0.00	6.00	568

cb065_7_: Number of [XChildName[7]]'s Children

Mean	SD	Min	Max	Obs
1.80	0.96	0.00	6.00	269

cb065_8_: Number of [XChildName[8]]'s Children

Mean	SD	Min	Max	Obs
1.84	0.99	0.00	5.00	121

cb065_9_: Number of [XChildName[9]]'s Children

Mean	SD	Min	Max	Obs
1.74	0.76	0.00	3.00	54

cb065_10_: Number of [XChildName[10]]'s Children

Mean	SD	Min	Max	Obs
1.97	0.87	1.00	4.00	29

cb065_11_: Number of [XChildName[11]]'s Children

Mean	SD	Min	Max	Obs
1.88	0.96	0.00	3.00	16

cb065_12_: Number of [XChildName[12]]'s Children

Mean	SD	Min	Max	Obs
2.13	0.64	1.00	3.00	8

cb065_13_: Number of [XChildName[13]]'s Children

Mean	SD	Min	Max	Obs
2.00	1.41	1.00	3.00	2

cb065_14_: Number of [XChildName[14]]'s Children

Mean	SD	Min	Max	Obs
1.00	0.00	1.00	1.00	2

cb065_15_: Number of [XChildName[15]]'s Children

Mean	SD	Min	Max	Obs
1.00	.	1.00	1.00	1

cb066_1_: Number of [XChildName[1]]'s Children below 16 Years Old

Mean	SD	Min	Max	Obs
1.04	1.21	0.00	33.00	8,259

cb066_2_: Number of [XChildName[2]]'s Children below 16 Years Old

Mean	SD	Min	Max	Obs
0.99	1.16	0.00	24.00	7,056

cb066_3_: Number of [XChildName[3]]'s Children below 16 Years Old

Mean	SD	Min	Max	Obs
0.95	1.25	0.00	30.00	4,402

cb066_4_: Number of [XChildName[4]]'s Children below 16 Years Old

Mean	SD	Min	Max	Obs
0.83	1.29	0.00	27.00	2,430

cb066_5_: Number of [XChildName[5]]'s Children below 16 Years Old

Mean	SD	Min	Max	Obs
0.77	1.16	0.00	19.00	1,202

cb066_6_: Number of [XChildName[6]]'s Children below 16 Years Old

Mean	SD	Min	Max	Obs
0.75	1.18	0.00	12.00	529

cb066_7_: Number of [XChildName[7]]'s Children below 16 Years Old

Mean	SD	Min	Max	Obs
0.83	1.04	0.00	6.00	247

cb066_8_: Number of [XChildName[8]]'s Children below 16 Years Old

Mean	SD	Min	Max	Obs
0.75	1.03	0.00	4.00	110

cb066_9_: Number of [XChildName[9]]'s Children below 16 Years Old

Mean	SD	Min	Max	Obs
0.77	1.06	0.00	5.00	52

cb066_10_: Number of [XChildName[10]]'s Children below 16 Years Old

Mean	SD	Min	Max	Obs
0.86	1.06	0.00	4.00	29

cb066_11_: Number of [XChildName[11]]'s Children below 16 Years Old

Mean	SD	Min	Max	Obs
0.67	0.98	0.00	3.00	15

cb066_12_: Number of [XChildName[12]]'s Children below 16 Years Old

Mean	SD	Min	Max	Obs
1.13	0.99	0.00	2.00	8

cb066_13_: Number of [XChildName[13]]'s Children below 16 Years Old

Mean	SD	Min	Max	Obs
0.50	0.71	0.00	1.00	2

cb066_14_: Number of [XChildName[14]]'s Children below 16 Years Old

Mean	SD	Min	Max	Obs
0.50	0.71	0.00	1.00	2

cb066_15_: Number of [XChildName[15]]'s Children below 16 Years Old

Mean	SD	Min	Max	Obs
0.00	.	0.00	0.00	1

cb067_1_: Number of [XChildName[1]]'s Grandchildren

Mean	SD	Min	Max	Obs
0.29	0.85	0.00	11.00	8,261

cb067_2_: Number of [XChildName[2]]'s Grandchildren

Mean	SD	Min	Max	Obs
0.30	0.85	0.00	10.00	7,057

cb067_3_: Number of [XChildName[3]]'s Grandchildren

Mean	SD	Min	Max	Obs
0.33	0.89	0.00	18.00	4,404

cb067_4_: Number of [XChildName[4]]'s Grandchildren

Mean	SD	Min	Max	Obs
0.40	0.95	0.00	8.00	2,428

cb067_5_: Number of [XChildName[5]]'s Grandchildren

Mean	SD	Min	Max	Obs
0.49	1.07	0.00	10.00	1,203

cb067_6_: Number of [XChildName[6]]'s Grandchildren

Mean	SD	Min	Max	Obs
0.54	1.17	0.00	9.00	527

cb067_7_: Number of [XChildName[7]]'s Grandchildren

Mean	SD	Min	Max	Obs
0.54	1.15	0.00	7.00	248

cb067_8_: Number of [XChildName[8]]'s Grandchildren

Mean	SD	Min	Max	Obs
0.71	1.51	0.00	10.00	109

cb067_9_: Number of [XChildName[9]]'s Grandchildren

Mean	SD	Min	Max	Obs
0.53	1.16	0.00	6.00	51

cb067_10_: Number of [XChildName[10]]'s Grandchildren

Mean	SD	Min	Max	Obs
0.55	1.06	0.00	4.00	29

cb067_11_: Number of [XChildName[11]]'s Grandchildren

Mean	SD	Min	Max	Obs
0.56	1.31	0.00	5.00	16

cb067_12_: Number of [XChildName[12]]'s Grandchildren

Mean	SD	Min	Max	Obs
0.11	0.33	0.00	1.00	9

cb067_13_: Number of [XChildName[13]]'s Grandchildren

Mean	SD	Min	Max	Obs
0.50	0.71	0.00	1.00	2

cb067_14_: Number of [XChildName[14]]'s Grandchildren

Mean	SD	Min	Max	Obs
0.00	0.00	0.00	0.00	2

cb067_15_: Number of [XChildName[15]]'s Grandchildren

Mean	SD	Min	Max	Obs
0.00	.	0.00	0.00	1

cb068_1_: Number of [XChildName[1]]'s Grandchildren below 16 Years Old

Mean	SD	Min	Max	Obs
1.65	1.31	0.00	11.00	1,221

cb068_2_: Number of [XChildName[2]]'s Grandchildren below 16 Years Old

Mean	SD	Min	Max	Obs
1.67	1.27	0.00	9.00	1,092

cb068_3_: Number of [XChildName[3]]'s Grandchildren below 16 Years Old

Mean	SD	Min	Max	Obs
1.67	1.23	0.00	14.00	774

cb068_4_: Number of [XChildName[4]]'s Grandchildren below 16 Years Old

Mean	SD	Min	Max	Obs
1.61	1.22	0.00	8.00	530

cb068_5_: Number of [XChildName[5]]'s Grandchildren below 16 Years Old

Mean	SD	Min	Max	Obs
1.74	1.17	0.00	7.00	293

cb068_6_: Number of [XChildName[6]]'s Grandchildren below 16 Years Old

Mean	SD	Min	Max	Obs
1.63	1.35	0.00	9.00	138

cb068_7_: Number of [XChildName[7]]'s Grandchildren below 16 Years Old

Mean	SD	Min	Max	Obs
1.92	1.35	0.00	6.00	61

cb068_8_: Number of [XChildName[8]]'s Grandchildren below 16 Years Old

Mean	SD	Min	Max	Obs
1.77	1.33	0.00	6.00	31

cb068_9_: Number of [XChildName[9]]'s Grandchildren below 16 Years Old

Mean	SD	Min	Max	Obs
2.00	1.53	0.00	6.00	13

cb068_10_: Number of [XChildName[10]]'s Grandchildren below 16 Years Old

Mean	SD	Min	Max	Obs
2.00	1.07	1.00	4.00	8

cb068_11_: Number of [XChildName[11]]'s Grandchildren below 16 Years Old

Mean	SD	Min	Max	Obs
2.25	1.89	1.00	5.00	4

cb068_12_: Number of [XChildName[12]]'s Grandchildren below 16 Years Old

Mean	SD	Min	Max	Obs
1.00	.	1.00	1.00	1

cb068_13_: Number of [XChildName[13]]'s Grandchildren below 16 Years Old

Mean	SD	Min	Max	Obs
1.00	.	1.00	1.00	1

cb069_1_: The Range of [XChildName[1]] and Spouse's Total Income

	Freq.	%
1 0 No Income	859	7.89
2 Less Than 2000 Yuan	104	0.95
3 2000-5000 Yuan	251	2.30
4 5000-10000 Yuan	436	4.00
5 10000-20000 Yuan	1,141	10.47
6 20000-30000 Yuan	1,342	12.32
7 30000-50000 Yuan	1,769	16.24
8 50000-100000 Yuan	1,718	15.77
9 100000-150000 Yuan	540	4.96
10 150000-200000 Yuan	130	1.19
11 200000-300000 Yuan	97	0.89

	Freq.	%
12 More Than 300000 Yuan	81	0.74
997 Don't Know	2,385	21.89
999 Refuse to Answer	40	0.37
Total	10,893	100.00

cb069_2_: The Range of [XChildName[2]] and Spouse's Total Income

	Freq.	%
1 0 No Income	693	7.85
2 Less Than 2000 Yuan	73	0.83
3 2000-5000 Yuan	188	2.13
4 5000-10000 Yuan	339	3.84
5 10000-20000 Yuan	875	9.91
6 20000-30000 Yuan	1,112	12.59
7 30000-50000 Yuan	1,387	15.71
8 50000-100000 Yuan	1,281	14.51
9 100000-150000 Yuan	396	4.48
10 150000-200000 Yuan	113	1.28
11 200000-300000 Yuan	74	0.84
12 More Than 300000 Yuan	52	0.59
997 Don't Know	2,221	25.15
999 Refuse to Answer	27	0.31
Total	8,831	100.00

cb069_3_: The Range of [XChildName[3]] and Spouse's Total Income

	Freq.	%
1 0 No Income	227	4.50
2 Less Than 2000 Yuan	42	0.83
3 2000-5000 Yuan	129	2.56
4 5000-10000 Yuan	206	4.09
5 10000-20000 Yuan	498	9.88
6 20000-30000 Yuan	578	11.47
7 30000-50000 Yuan	731	14.50
8 50000-100000 Yuan	712	14.13
9 100000-150000 Yuan	225	4.46
10 150000-200000 Yuan	49	0.97
11 200000-300000 Yuan	27	0.54
12 More Than 300000 Yuan	39	0.77
997 Don't Know	1,559	30.93
999 Refuse to Answer	18	0.36
Total	5,040	100.00

cb069_4_: The Range of [XChildName[4]] and Spouse's Total Income

	Freq.	%
1 0 No Income	122	4.52
2 Less Than 2000 Yuan	24	0.89
3 2000-5000 Yuan	84	3.11
4 5000-10000 Yuan	89	3.30
5 10000-20000 Yuan	257	9.53
6 20000-30000 Yuan	298	11.05
7 30000-50000 Yuan	377	13.98
8 50000-100000 Yuan	330	12.24
9 100000-150000 Yuan	121	4.49

10 150000-200000 Yuan	22	0.82
11 200000-300000 Yuan	22	0.82
12 More Than 300000 Yuan	22	0.82
997 Don't Know	912	33.82
999 Refuse to Answer	17	0.63
Total	2,697	100.00

cb069_5_: The Range of [XChildName[5]] and Spouse's Total Income

	Freq.	%
1 0 No Income	59	4.52
2 Less Than 2000 Yuan	10	0.77
3 2000-5000 Yuan	38	2.91
4 5000-10000 Yuan	53	4.06
5 10000-20000 Yuan	125	9.58
6 20000-30000 Yuan	152	11.65
7 30000-50000 Yuan	176	13.49
8 50000-100000 Yuan	150	11.49
9 100000-150000 Yuan	51	3.91
10 150000-200000 Yuan	9	0.69
11 200000-300000 Yuan	8	0.61
12 More Than 300000 Yuan	5	0.38
997 Don't Know	462	35.40
999 Refuse to Answer	7	0.54
Total	1,305	100.00

cb069_6_: The Range of [XChildName[6]] and Spouse's Total Income

	Freq.	%
1 0 No Income	29	5.02
2 Less Than 2000 Yuan	8	1.38
3 2000-5000 Yuan	22	3.81
4 5000-10000 Yuan	30	5.19
5 10000-20000 Yuan	64	11.07
6 20000-30000 Yuan	47	8.13
7 30000-50000 Yuan	60	10.38
8 50000-100000 Yuan	64	11.07
9 100000-150000 Yuan	28	4.84
10 150000-200000 Yuan	6	1.04
11 200000-300000 Yuan	3	0.52
12 More Than 300000 Yuan	2	0.35
997 Don't Know	213	36.85
999 Refuse to Answer	2	0.35
Total	578	100.00

cb069_7_: The Range of [XChildName[7]] and Spouse's Total Income

	Freq.	%
1 0 No Income	12	4.36
2 Less Than 2000 Yuan	5	1.82
3 2000-5000 Yuan	9	3.27
4 5000-10000 Yuan	15	5.45
5 10000-20000 Yuan	35	12.73
6 20000-30000 Yuan	26	9.45
7 30000-50000 Yuan	32	11.64

	Freq.	%
8 50000-100000 Yuan	23	8.36
9 100000-150000 Yuan	12	4.36
11 200000-300000 Yuan	4	1.45
12 More Than 300000 Yuan	2	0.73
997 Don't Know	98	35.64
999 Refuse to Answer	2	0.73
Total	275	100.00

cb069_8_: The Range of [XChildName[8]] and Spouse's Total Income

	Freq.	%
1 0 No Income	8	6.45
2 Less Than 2000 Yuan	1	0.81
3 2000-5000 Yuan	4	3.23
4 5000-10000 Yuan	4	3.23
5 10000-20000 Yuan	16	12.90
6 20000-30000 Yuan	11	8.87
7 30000-50000 Yuan	10	8.06
8 50000-100000 Yuan	17	13.71
9 100000-150000 Yuan	4	3.23
10 150000-200000 Yuan	3	2.42
997 Don't Know	45	36.29
999 Refuse to Answer	1	0.81
Total	124	100.00

cb069_9_: The Range of [XChildName[9]] and Spouse's Total Income

	Freq.	%
1 0 No Income	2	3.57
3 2000-5000 Yuan	3	5.36
5 10000-20000 Yuan	9	16.07
6 20000-30000 Yuan	10	17.86
7 30000-50000 Yuan	5	8.93
8 50000-100000 Yuan	5	8.93
10 150000-200000 Yuan	1	1.79
997 Don't Know	20	35.71
999 Refuse to Answer	1	1.79
Total	56	100.00

cb069_10_: The Range of [XChildName[10]] and Spouse's Total Income

	Freq.	%
1 0 No Income	1	3.23
3 2000-5000 Yuan	1	3.23
4 5000-10000 Yuan	1	3.23
5 10000-20000 Yuan	3	9.68
6 20000-30000 Yuan	4	12.90
7 30000-50000 Yuan	6	19.35
8 50000-100000 Yuan	2	6.45
997 Don't Know	13	41.94
Total	31	100.00

cb069_11_: The Range of [XChildName[11]] and Spouse's Total Income

	Freq.	%
1 0 No Income	2	11.11
4 5000-10000 Yuan	1	5.56
5 10000-20000 Yuan	3	16.67
6 20000-30000 Yuan	2	11.11
7 30000-50000 Yuan	3	16.67
8 50000-100000 Yuan	1	5.56
9 100000-150000 Yuan	1	5.56
997 Don't Know	5	27.78
Total	18	100.00

cb069_12_: The Range of [XChildName[12]] and Spouse's Total Income

	Freq.	%
5 10000-20000 Yuan	1	10.00
6 20000-30000 Yuan	2	20.00
7 30000-50000 Yuan	2	20.00
997 Don't Know	5	50.00
Total	10	100.00

cb069_13_: The Range of [XChildName[13]] and Spouse's Total Income

	Freq.	%
5 10000-20000 Yuan	1	50.00
8 50000-100000 Yuan	1	50.00
Total	2	100.00

cb069_14_: The Range of [XChildName[14]] and Spouse's Total Income

	Freq.	%
5 10000-20000 Yuan	1	50.00
997 Don't Know	1	50.00
Total	2	100.00

cb069_15_: The Range of [XChildName[15]] and Spouse's Total Income

	Freq.	%
6 20000-30000 Yuan	1	100.00
Total	1	100.00

cb071_w3_1_: [XChildName[1]] Has House Property

	Freq.	%
1 Yes	5,539	50.85
2 No	5,169	47.45
997 Don't Know	171	1.57
999 Refuse to Answer	14	0.13
Total	10,893	100.00

cb071_w3_2_: [XChildName[2]] Has House Property

	Freq.	%
1 Yes	4,915	55.66
2 No	3,723	42.16
997 Don't Know	178	2.02
999 Refuse to Answer	15	0.17
Total	8,831	100.00

cb071_w3_3_: [XChildName[3]] Has House Property

	Freq.	%
1 Yes	3,148	62.46
2 No	1,758	34.88
997 Don't Know	126	2.50
999 Refuse to Answer	8	0.16
Total	5,040	100.00

cb071_w3_4_: [XChildName[4]] Has House Property

	Freq.	%
1 Yes	1,843	68.34
2 No	770	28.55
997 Don't Know	73	2.71
999 Refuse to Answer	11	0.41
Total	2,697	100.00

cb071_w3_5_: [XChildName[5]] Has House Property

	Freq.	%
1 Yes	919	70.42
2 No	345	26.44
997 Don't Know	37	2.84
999 Refuse to Answer	4	0.31
Total	1,305	100.00

cb071_w3_6_: [XChildName[6]] Has House Property

	Freq.	%
1 Yes	415	71.80
2 No	140	24.22
997 Don't Know	21	3.63
999 Refuse to Answer	2	0.35
Total	578	100.00

cb071_w3_7_: [XChildName[7]] Has House Property

	Freq.	%
1 Yes	197	71.64
2 No	69	25.09
997 Don't Know	8	2.91
999 Refuse to Answer	1	0.36

	Freq.	%
Total	275	100.00

cb071_w3_8_: [XChildName[8]] Has House Property

	Freq.	%
1 Yes	85	68.55
2 No	30	24.19
997 Don't Know	8	6.45
999 Refuse to Answer	1	0.81
Total	124	100.00

cb071_w3_9_: [XChildName[9]] Has House Property

	Freq.	%
1 Yes	42	75.00
2 No	10	17.86
997 Don't Know	3	5.36
999 Refuse to Answer	1	1.79
Total	56	100.00

cb071_w3_10_: [XChildName[10]] Has House Property

	Freq.	%
1 Yes	25	80.65
2 No	5	16.13
997 Don't Know	1	3.23
Total	31	100.00

cb071_w3_11_: [XChildName[11]] Has House Property

	Freq.	%
1 Yes	15	83.33
997 Don't Know	3	16.67
Total	18	100.00

cb071_w3_12_: [XChildName[12]] Has House Property

	Freq.	%
1 Yes	6	60.00
2 No	1	10.00
997 Don't Know	3	30.00
Total	10	100.00

cb071_w3_13_: [XChildName[13]] Has House Property

	Freq.	%
1 Yes	2	100.00
Total	2	100.00

cb071_w3_14_: [XChildName[14]] Has House Property

	Freq.	%
1 Yes	2	100.00
Total	2	100.00

cb071_w3_15_: [XChildName[15]] Has House Property

	Freq.	%
1 Yes	1	100.00
Total	1	100.00

cb072_w3_1_: Value of [XChildName[1]]'s House Property

Mean	SD	Min	Max	Obs
11,505.07	125,619.79	0.00	4,000,000.00	4,255

cb072_w3_2_: Value of [XChildName[2]]'s House Property

Mean	SD	Min	Max	Obs
9,339.05	87,102.57	0.00	2,000,000.00	3,709

cb072_w3_3_: Value of [XChildName[3]]'s House Property

Mean	SD	Min	Max	Obs
12,803.29	139,025.12	0.00	4,500,000.00	2,285

cb072_w3_4_: Value of [XChildName[4]]'s House Property

Mean	SD	Min	Max	Obs
19,152.62	371,727.45	0.00	13000000.00	1,280

cb072_w3_5_: Value of [XChildName[5]]'s House Property

Mean	SD	Min	Max	Obs
18,756.61	203,946.33	0.00	4,000,000.00	617

cb072_w3_6_: Value of [XChildName[6]]'s House Property

Mean	SD	Min	Max	Obs
12,382.41	89,077.05	0.00	1,000,000.00	283

cb072_w3_7_: Value of [XChildName[7]]'s House Property

Mean	SD	Min	Max	Obs
8,067.49	66,680.10	0.00	700,000.00	130

cb072_w3_8_: Value of [XChildName[8]]'s House Property

Mean	SD	Min	Max	Obs
9,566.47	48,187.83	0.04	300,000.00	55

cb072_w3_9_: Value of [XChildName[9]]'s House Property

Mean	SD	Min	Max	Obs
21.40	41.82	0.70	225.00	27

cb072_w3_10_: Value of [XChildName[10]]'s House Property

Mean	SD	Min	Max	Obs
11.99	8.34	0.00	30.00	20

cb072_w3_11_: Value of [XChildName[11]]'s House Property

Mean	SD	Min	Max	Obs
3,090.37	11,089.97	0.30	40,000.00	13

cb072_w3_12_: Value of [XChildName[12]]'s House Property

Mean	SD	Min	Max	Obs
10.50	7.71	0.00	20.00	6

cb072_w3_13_: Value of [XChildName[13]]'s House Property

Mean	SD	Min	Max	Obs
6.00	7.07	1.00	11.00	2

cb072_w3_14_: Value of [XChildName[14]]'s House Property

Mean	SD	Min	Max	Obs
6.00	5.66	2.00	10.00	2

cb072_w3_15_: Value of [XChildName[15]]'s House Property

Mean	SD	Min	Max	Obs
1.00	.	1.00	1.00	1

cb063_w3_1_1_: [XChildName[1]]'s Health Status Now

	Freq.	%
1 Very Good	3,528	32.09
2 Good	2,662	24.21
3 Fair	3,915	35.61

	Freq.	%
4 Poor	657	5.98
5 Very Poor	141	1.28
997 Don't Know	82	0.75
999 Refuse to Answer	9	0.08
Total	10,994	100.00

cb063_w3_1_2_: [XChildName[2]]'s Health Status Now

	Freq.	%
1 Very Good	2,750	30.19
2 Good	2,254	24.75
3 Fair	3,319	36.44
4 Poor	589	6.47
5 Very Poor	116	1.27
997 Don't Know	68	0.75
999 Refuse to Answer	12	0.13
Total	9,108	100.00

cb063_w3_1_3_: [XChildName[3]]'s Health Status Now

	Freq.	%
1 Very Good	1,298	25.29
2 Good	1,289	25.12
3 Fair	2,012	39.20
4 Poor	404	7.87
5 Very Poor	66	1.29
997 Don't Know	55	1.07
999 Refuse to Answer	8	0.16
Total	5,132	100.00

cb063_w3_1_4_: [XChildName[4]]'s Health Status Now

	Freq.	%
1 Very Good	621	22.76
2 Good	685	25.11
3 Fair	1,094	40.10
4 Poor	232	8.50
5 Very Poor	48	1.76
997 Don't Know	37	1.36
999 Refuse to Answer	11	0.40
Total	2,728	100.00

cb063_w3_1_5_: [XChildName[5]]'s Health Status Now

	Freq.	%
1 Very Good	259	19.74
2 Good	339	25.84
3 Fair	523	39.86
4 Poor	139	10.59
5 Very Poor	28	2.13
997 Don't Know	18	1.37
999 Refuse to Answer	6	0.46
Total	1,312	100.00

cb063_w3_1_6_: [XChildName[6]]'s Health Status Now

	Freq.	%
1 Very Good	108	18.52
2 Good	148	25.39
3 Fair	238	40.82
4 Poor	62	10.63
5 Very Poor	12	2.06
997 Don't Know	13	2.23
999 Refuse to Answer	2	0.34
Total	583	100.00

cb063_w3_1_7_: [XChildName[7]]'s Health Status Now

	Freq.	%
1 Very Good	49	17.50
2 Good	79	28.21
3 Fair	107	38.21
4 Poor	33	11.79
5 Very Poor	3	1.07
997 Don't Know	8	2.86
999 Refuse to Answer	1	0.36
Total	280	100.00

cb063_w3_1_8_: [XChildName[8]]'s Health Status Now

	Freq.	%
1 Very Good	18	13.85
2 Good	29	22.31
3 Fair	64	49.23
4 Poor	12	9.23
5 Very Poor	2	1.54
997 Don't Know	4	3.08
999 Refuse to Answer	1	0.77
Total	130	100.00

cb063_w3_1_9_: [XChildName[9]]'s Health Status Now

	Freq.	%
1 Very Good	4	7.14
2 Good	16	28.57
3 Fair	25	44.64
4 Poor	4	7.14
5 Very Poor	3	5.36
997 Don't Know	3	5.36
999 Refuse to Answer	1	1.79
Total	56	100.00

cb063_w3_1_10_: [XChildName[10]]'s Health Status Now

	Freq.	%
1 Very Good	5	15.63
2 Good	7	21.88

	Freq.	%
3 Fair	16	50.00
4 Poor	1	3.13
5 Very Poor	1	3.13
997 Don't Know	2	6.25
Total	32	100.00

cb063_w3_1_11_: [XChildName[11]]'s Health Status Now

	Freq.	%
1 Very Good	2	10.53
2 Good	6	31.58
3 Fair	7	36.84
4 Poor	1	5.26
997 Don't Know	3	15.79
Total	19	100.00

cb063_w3_1_12_: [XChildName[12]]'s Health Status Now

	Freq.	%
1 Very Good	1	10.00
2 Good	1	10.00
3 Fair	5	50.00
4 Poor	1	10.00
997 Don't Know	2	20.00
Total	10	100.00

cb063_w3_1_13_: [XChildName[13]]'s Health Status Now

	Freq.	%
1 Very Good	1	50.00
3 Fair	1	50.00
Total	2	100.00

cb063_w3_1_14_: [XChildName[14]]'s Health Status Now

	Freq.	%
3 Fair	1	50.00
4 Poor	1	50.00
Total	2	100.00

cb063_w3_1_15_: [XChildName[15]]'s Health Status Now

	Freq.	%
1 Very Good	1	100.00
Total	1	100.00

cb063_w3_6_1_: [XChildName[1]] Has Self-care Ability

	Freq.	%
1 Yes	354	97.52

2 No	9	2.48
Total	363	100.00

cb063_w3_6_2_: [XChildName[2]] Has Self-care Ability

	Freq.	%
1 Yes	253	96.56
2 No	9	3.44
Total	262	100.00

cb063_w3_6_3_: [XChildName[3]] Has Self-care Ability

	Freq.	%
1 Yes	129	96.27
2 No	5	3.73
Total	134	100.00

cb063_w3_6_4_: [XChildName[4]] Has Self-care Ability

	Freq.	%
1 Yes	104	98.11
2 No	2	1.89
Total	106	100.00

cb063_w3_6_5_: [XChildName[5]] Has Self-care Ability

	Freq.	%
1 Yes	62	95.38
2 No	3	4.62
Total	65	100.00

cb063_w3_6_6_: [XChildName[6]] Has Self-care Ability

	Freq.	%
1 Yes	32	94.12
2 No	2	5.88
Total	34	100.00

cb063_w3_6_7_: [XChildName[7]] Has Self-care Ability

	Freq.	%
1 Yes	15	93.75
2 No	1	6.25
Total	16	100.00

cb063_w3_6_8_: [XChildName[8]] Has Self-care Ability

	Freq.	%

1 Yes	10	100.00
Total	10	100.00

cb063_w3_6_9_: [XChildName[9]] Has Self-care Ability

	Freq.	%
1 Yes	2	50.00
2 No	2	50.00
Total	4	100.00

cb063_w3_6_10_: [XChildName[10]] Has Self-care Ability

	Freq.	%
1 Yes	1	100.00
Total	1	100.00

cc000_w4_1: Number of FamilyR's Siblings

Mean	SD	Min	Max	Obs
3.15	1.78	0.00	9.00	96

cc000_w4_1_s: Number of Spouse's Siblings

Mean	SD	Min	Max	Obs
2.95	1.92	0.00	8.00	276

cc002_w4_1: Number of FamilyR's Other Siblings

Mean	SD	Min	Max	Obs
0.15	0.74	0.00	12.00	11,512

cc003_w4_1_1_: When did Respondent's [ZSibName[1]] Die

	Freq.	%
1 Year	1,567	52.16
2 Age	1,437	47.84
Total	3,004	100.00

cc003_w4_1_2_: When did Respondent's [ZSibName[2]] Die

	Freq.	%
1 Year	1,160	53.33
2 Age	1,015	46.67
Total	2,175	100.00

cc003_w4_1_3_: When did Respondent's [ZSibName[3]] Die

	Freq.	%
1 Year	770	52.56
2 Age	695	47.44
Total	1,465	100.00

cc003_w4_1_4_: When did Respondent's [ZSibName[4]] Die

	Freq.	%
1 Year	452	49.08
2 Age	469	50.92
Total	921	100.00

cc003_w4_1_5_: When did Respondent's [ZSibName[5]] Die

	Freq.	%
1 Year	249	47.43
2 Age	276	52.57
Total	525	100.00

cc003_w4_1_6_: When did Respondent's [ZSibName[6]] Die

	Freq.	%
1 Year	133	47.16
2 Age	149	52.84
Total	282	100.00

cc003_w4_1_7_: When did Respondent's [ZSibName[7]] Die

	Freq.	%
1 Year	51	43.59
2 Age	66	56.41
Total	117	100.00

cc003_w4_1_8_: When did Respondent's [ZSibName[8]] Die

	Freq.	%
1 Year	21	42.86
2 Age	28	57.14
Total	49	100.00

cc003_w4_1_9_: When did Respondent's [ZSibName[9]] Die

	Freq.	%
1 Year	7	33.33
2 Age	14	66.67
Total	21	100.00

cc003_w4_1_10_: When did Respondent's [ZSibName[10]] Die

	Freq.	%
1 Year	2	33.33
2 Age	4	66.67
Total	6	100.00

cc003_w4_1_11_: When did Respondent's [ZSibName[11]] Die

	Freq.	%
1 Year	3	33.33
2 Age	6	66.67
Total	9	100.00

cc003_w4_1_12_: When did Respondent's [ZSibName[12]] Die

	Freq.	%
1 Year	1	33.33
2 Age	2	66.67
Total	3	100.00

cc003_w4_1_13_: When did Respondent's [ZSibName[13]] Die

	Freq.	%
1 Year	1	100.00
Total	1	100.00

cc003_w4_1_s_1_: When did Respondent's [ZSibName[1]] Die

	Freq.	%
1 Year	1,008	54.57
2 Age	839	45.43
Total	1,847	100.00

cc003_w4_1_s_2_: When did Respondent's [ZSibName[2]] Die

	Freq.	%
1 Year	709	56.63
2 Age	543	43.37
Total	1,252	100.00

cc003_w4_1_s_3_: When did Respondent's [ZSibName[3]] Die

	Freq.	%
1 Year	471	53.83
2 Age	404	46.17
Total	875	100.00

cc003_w4_1_s_4_: When did Respondent's [ZSibName[4]] Die

	Freq.	%
1 Year	282	51.55
2 Age	265	48.45
Total	547	100.00

cc003_w4_1_s_5_: When did Respondent's [ZSibName[5]] Die

	Freq.	%
1 Year	150	49.67
2 Age	152	50.33
Total	302	100.00

cc003_w4_1_s_6_: When did Respondent's [ZSibName[6]] Die

	Freq.	%
1 Year	100	53.76
2 Age	86	46.24
Total	186	100.00

cc003_w4_1_s_7_: When did Respondent's [ZSibName[7]] Die

	Freq.	%
1 Year	33	47.83
2 Age	36	52.17
Total	69	100.00

cc003_w4_1_s_8_: When did Respondent's [ZSibName[8]] Die

	Freq.	%
1 Year	8	30.77
2 Age	18	69.23
Total	26	100.00

cc003_w4_1_s_9_: When did Respondent's [ZSibName[9]] Die

	Freq.	%
1 Year	9	39.13
2 Age	14	60.87
Total	23	100.00

cc003_w4_1_s_10_: When did Respondent's [ZSibName[10]] Die

	Freq.	%
1 Year	5	62.50
2 Age	3	37.50
Total	8	100.00

cc003_w4_1_s_11_: When did Respondent's [ZSibName[11]] Die

	Freq.	%
2 Age	1	100.00
Total	1	100.00

cc003_w4_1_s_12_: When did Respondent's [ZSibName[12]] Die

	Freq.	%
2 Age	1	100.00
Total	1	100.00

cc003_w4_1_s_13_: When did Respondent's [ZSibName[13]] Die

No Observations

cc003_w4_1_s_14_: When did Respondent's [ZSibName[14]] Die

	Freq.	%
2 Age	1	100.00
Total	1	100.00

cc003_w4_1_1_1_: When did Respondent's [ZSibName[1]] Die: Year

Mean	SD	Min	Max	Obs
1,966.97	260.31	0.00	2,047.00	1,528

cc003_w4_1_1_2_: When did Respondent's [ZSibName[2]] Die: Year

Mean	SD	Min	Max	Obs
1,976.21	226.48	0.00	2,018.00	1,128

cc003_w4_1_1_3_: When did Respondent's [ZSibName[3]] Die: Year

Mean	SD	Min	Max	Obs
1,997.41	698.94	0.00	19,995.00	752

cc003_w4_1_1_4_: When did Respondent's [ZSibName[4]] Die: Year

Mean	SD	Min	Max	Obs
1,973.78	486.47	0.00	9,999.00	437

cc003_w4_1_1_5_: When did Respondent's [ZSibName[5]] Die: Year

Mean	SD	Min	Max	Obs
1,960.84	286.23	0.00	2,018.00	240

cc003_w4_1_1_6_: When did Respondent's [ZSibName[6]] Die: Year

Mean	SD	Min	Max	Obs
1,905.33	427.26	0.00	2,018.00	126

cc003_w4_1_1_7_: When did Respondent's [ZSibName[7]] Die: Year

Mean	SD	Min	Max	Obs
1,959.34	292.84	0.00	2,018.00	47

cc003_w4_1_1_8_: When did Respondent's [ZSibName[8]] Die: Year

Mean	SD	Min	Max	Obs
1,904.30	448.45	0.00	2,018.00	20

cc003_w4_1_1_9_: When did Respondent's [ZSibName[9]] Die: Year

Mean	SD	Min	Max	Obs
1,721.29	759.05	0.00	2,016.00	7

cc003_w4_1_1_10_: When did Respondent's [ZSibName[10]] Die: Year

Mean	SD	Min	Max	Obs
1,006.50	1,423.41	0.00	2,013.00	2

cc003_w4_1_1_11_: When did Respondent's [ZSibName[11]] Die: Year

Mean	SD	Min	Max	Obs
1,328.33	1,150.49	0.00	2,009.00	3

cc003_w4_1_1_12_: When did Respondent's [ZSibName[12]] Die: Year

Mean	SD	Min	Max	Obs
0.00	.	0.00	0.00	1

cc003_w4_1_1_13_: When did Respondent's [ZSibName[13]] Die: Year

Mean	SD	Min	Max	Obs
0.00	.	0.00	0.00	1

cc003_w4_1_1_s_1_: When did Spouse's [ZSibName[1]] Die: Year

Mean	SD	Min	Max	Obs
1,970.01	254.85	0.00	2,018.00	986

cc003_w4_1_1_s_2_: When did Spouse's [ZSibName[2]] Die: Year

Mean	SD	Min	Max	Obs
1,974.86	244.05	0.00	2,108.00	700

cc003_w4_1_1_s_3_: When did Spouse's [ZSibName[3]] Die: Year

Mean	SD	Min	Max	Obs
1,988.64	184.31	2.00	2,018.00	462

cc003_w4_1_1_s_4_: When did Spouse's [ZSibName[4]] Die: Year

Mean	SD	Min	Max	Obs
1,989.56	168.03	0.00	2,018.00	277

cc003_w4_1_1_s_5_: When did Spouse's [ZSibName[5]] Die: Year

Mean	SD	Min	Max	Obs
1,988.16	158.64	71.00	2,018.00	150

cc003_w4_1_1_s_6_: When did Spouse's [ZSibName[6]] Die: Year

Mean	SD	Min	Max	Obs
1,959.96	282.28	0.00	2,018.00	97

cc003_w4_1_1_s_7_: When did Spouse's [ZSibName[7]] Die: Year

Mean	SD	Min	Max	Obs
1,935.90	359.65	0.00	2,018.00	31

cc003_w4_1_1_s_8_: When did Spouse's [ZSibName[8]] Die: Year

Mean	SD	Min	Max	Obs
1,707.29	753.13	0.00	2,014.00	7

cc003_w4_1_1_s_9_: When did Spouse's [ZSibName[9]] Die: Year

Mean	SD	Min	Max	Obs
1,780.33	667.77	0.00	2,018.00	9

cc003_w4_1_1_s_10_: When did Spouse's [ZSibName[10]] Die: Year

Mean	SD	Min	Max	Obs
1,995.40	31.65	1,940.00	2,014.00	5

cc003_w4_1_2_1_: When did Respondent's [ZSibName[1]] Die: Age

Mean	SD	Min	Max	Obs
65.53	138.53	0.00	2,017.00	1,413

cc003_w4_1_2_2_: When did Respondent's [ZSibName[2]] Die: Age

Mean	SD	Min	Max	Obs
83.48	245.80	0.00	2,017.00	1,000

cc003_w4_1_2_3_: When did Respondent's [ZSibName[3]] Die: Age

Mean	SD	Min	Max	Obs
78.85	247.25	0.00	2,017.00	682

cc003_w4_1_2_4_: When did Respondent's [ZSibName[4]] Die: Age

Mean	SD	Min	Max	Obs
69.67	223.19	0.00	2,013.00	461

cc003_w4_1_2_5_: When did Respondent's [ZSibName[5]] Die: Age

Mean	SD	Min	Max	Obs
91.83	314.13	0.00	2,016.00	270

cc003_w4_1_2_6_: When did Respondent's [ZSibName[6]] Die: Age

Mean	SD	Min	Max	Obs
64.81	230.80	0.00	2,013.00	147

cc003_w4_1_2_7_: When did Respondent's [ZSibName[7]] Die: Age

Mean	SD	Min	Max	Obs
33.88	26.33	0.00	85.00	65

cc003_w4_1_2_8_: When did Respondent's [ZSibName[8]] Die: Age

Mean	SD	Min	Max	Obs
28.26	25.98	0.00	82.00	27

cc003_w4_1_2_9_: When did Respondent's [ZSibName[9]] Die: Age

Mean	SD	Min	Max	Obs
18.01	26.04	0.00	80.00	13

cc003_w4_1_2_10_: When did Respondent's [ZSibName[10]] Die: Age

Mean	SD	Min	Max	Obs
17.50	33.01	0.00	67.00	4

cc003_w4_1_2_11_: When did Respondent's [ZSibName[11]] Die: Age

Mean	SD	Min	Max	Obs
11.17	17.15	0.00	43.00	6

cc003_w4_1_2_12_: When did Respondent's [ZSibName[12]] Die: Age

Mean	SD	Min	Max	Obs
4.00	4.24	1.00	7.00	2

cc003_w4_1_2_s_1_: When did Spouse's [ZSibName[1]] Die: Age

Mean	SD	Min	Max	Obs
77.90	212.04	0.00	2,015.00	830

cc003_w4_1_2_s_2_: When did Spouse's [ZSibName[2]] Die: Age

Mean	SD	Min	Max	Obs
60.47	147.02	0.00	2,016.00	530

cc003_w4_1_2_s_3_: When did Spouse's [ZSibName[3]] Die: Age

Mean	SD	Min	Max	Obs
63.30	194.69	0.00	2,014.00	402

cc003_w4_1_2_s_4_: When did Spouse's [ZSibName[4]] Die: Age

Mean	SD	Min	Max	Obs
78.98	269.62	0.00	2,017.00	260

cc003_w4_1_2_s_5_: When did Spouse's [ZSibName[5]] Die: Age

Mean	SD	Min	Max	Obs
63.25	221.45	0.00	2,002.00	152

cc003_w4_1_2_s_6_: When did Spouse's [ZSibName[6]] Die: Age

Mean	SD	Min	Max	Obs
85.06	302.88	0.00	2,013.00	81

cc003_w4_1_2_s_7_: When did Spouse's [ZSibName[7]] Die: Age

Mean	SD	Min	Max	Obs
83.96	313.20	0.00	1,933.00	37

cc003_w4_1_2_s_8_: When did Spouse's [ZSibName[8]] Die: Age

Mean	SD	Min	Max	Obs
144.28	447.41	5.00	1,935.00	18

cc003_w4_1_2_s_9_: When did Spouse's [ZSibName[9]] Die: Age

Mean	SD	Min	Max	Obs
178.14	524.31	0.00	1,998.00	14

cc003_w4_1_2_s_10_: When did Spouse's [ZSibName[10]] Die: Age

Mean	SD	Min	Max	Obs
9.67	10.02	0.00	20.00	3

cc003_w4_1_2_s_11_: When did Spouse's [ZSibName[11]] Die: Age

Mean	SD	Min	Max	Obs
1,942.00	.	1,942.00	1,942.00	1

cc003_w4_1_2_s_12_: When did Spouse's [ZSibName[12]] Die: Age

Mean	SD	Min	Max	Obs
1,945.00	.	1,945.00	1,945.00	1

cc003_w4_1_2_s_13_: When did Spouse's [ZSibName[13]] Die: Age

No Observations

cc003_w4_1_2_s_14_: When did Spouse's [ZSibName[14]] Die: Age

Mean	SD	Min	Max	Obs
3.00	.	3.00	3.00	1

cc003_w4_5_1_: Is FamilyR's [ZSibName[1]]'s Highest Degree Right

	Freq.	%
1 Yes	6,862	89.71
2 No	787	10.29
Total	7,649	100.00

cc003_w4_5_2_: Is FamilyR's [ZSibName[2]]'s Highest Degree Right

	Freq.	%
1 Yes	6,850	90.08
2 No	754	9.92
Total	7,604	100.00

cc003_w4_5_3_: Is FamilyR's [ZSibName[3]]'s Highest Degree Right

	Freq.	%
1 Yes	5,990	90.43
2 No	634	9.57
Total	6,624	100.00

cc003_w4_5_4_: Is FamilyR's [ZSibName[4]]'s Highest Degree Right

	Freq.	%
1 Yes	4,447	89.69
2 No	511	10.31
Total	4,958	100.00

cc003_w4_5_5_: Is FamilyR's [ZSibName[5]]'s Highest Degree Right

	Freq.	%
1 Yes	2,800	89.51
2 No	328	10.49
Total	3,128	100.00

cc003_w4_5_6_: Is FamilyR's [ZSibName[6]]'s Highest Degree Right

	Freq.	%
1 Yes	1,422	90.06
2 No	157	9.94
Total	1,579	100.00

cc003_w4_5_7_: Is FamilyR's [ZSibName[7]]'s Highest Degree Right

	Freq.	%
1 Yes	581	88.43
2 No	76	11.57
Total	657	100.00

cc003_w4_5_8_: Is FamilyR's [ZSibName[8]]'s Highest Degree Right

	Freq.	%
1 Yes	201	88.94
2 No	25	11.06
Total	226	100.00

cc003_w4_5_9_: Is FamilyR's [ZSibName[9]]'s Highest Degree Right

	Freq.	%
1 Yes	76	88.37
2 No	10	11.63
Total	86	100.00

cc003_w4_5_10_: Is FamilyR's [ZSibName[10]]'s Highest Degree Right

	Freq.	%
1 Yes	23	79.31
2 No	6	20.69
Total	29	100.00

cc003_w4_5_11_: Is FamilyR's [ZSibName[11]]'s Highest Degree Right

	Freq.	%
1 Yes	11	78.57
2 No	3	21.43
Total	14	100.00

cc003_w4_5_12_: Is FamilyR's [ZSibName[12]]'s Highest Degree Right

	Freq.	%
1 Yes	4	80.00
2 No	1	20.00
Total	5	100.00

cc003_w4_5_13_: Is FamilyR's [ZSibName[13]]'s Highest Degree Right

	Freq.	%
1 Yes	1	50.00
2 No	1	50.00
Total	2	100.00

cc003_w4_5_14_: Is FamilyR's [ZSibName[14]]'s Highest Degree Right

	Freq.	%
1 Yes	1	100.00
Total	1	100.00

cc003_w4_5_s_1_: Is Spouse's [ZSibName[1]]'s Highest Degree Right

	Freq.	%
1 Yes	5,266	90.48
2 No	554	9.52
Total	5,820	100.00

cc003_w4_5_s_2_: Is Spouse's [ZSibName[2]]'s Highest Degree Right

	Freq.	%
1 Yes	5,313	90.53
2 No	556	9.47
Total	5,869	100.00

cc003_w4_5_s_3_: Is Spouse's [ZSibName[3]]'s Highest Degree Right

	Freq.	%
1 Yes	4,602	90.25
2 No	497	9.75
Total	5,099	100.00

cc003_w4_5_s_4_: Is Spouse's [ZSibName[4]]'s Highest Degree Right

	Freq.	%
1 Yes	3,328	89.82
2 No	377	10.18
Total	3,705	100.00

cc003_w4_5_s_5_: Is Spouse's [ZSibName[5]]'s Highest Degree Right

	Freq.	%
1 Yes	2,094	90.57
2 No	218	9.43
Total	2,312	100.00

cc003_w4_5_s_6_: Is Spouse's [ZSibName[6]]'s Highest Degree Right

	Freq.	%
1 Yes	1,038	89.79
2 No	118	10.21
Total	1,156	100.00

cc003_w4_5_s_7_: Is Spouse's [ZSibName[7]]'s Highest Degree Right

	Freq.	%
1 Yes	490	91.42
2 No	46	8.58
Total	536	100.00

cc003_w4_5_s_8_: Is Spouse's [ZSibName[8]]'s Highest Degree Right

	Freq.	%
1 Yes	181	88.29
2 No	24	11.71
Total	205	100.00

cc003_w4_5_s_9_: Is Spouse's [ZSibName[9]]'s Highest Degree Right

	Freq.	%
1 Yes	55	93.22
2 No	4	6.78
Total	59	100.00

cc003_w4_5_s_10_: Is Spouse's [ZSibName[10]]'s Highest Degree Right

	Freq.	%
1 Yes	14	82.35
2 No	3	17.65
Total	17	100.00

cc003_w4_5_s_11_: Is Spouse's [ZSibName[11]]'s Highest Degree Right

	Freq.	%
1 Yes	8	100.00
Total	8	100.00

cc003_w4_5_s_12_: Is Spouse's [ZSibName[12]]'s Highest Degree Right

	Freq.	%
1 Yes	4	100.00
Total	4	100.00

cc003_w4_5_s_13_: Is Spouse's [ZSibName[13]]'s Highest Degree Right

	Freq.	%
1 Yes	1	100.00
Total	1	100.00

cc003_w3_1_: FamilyR's [ZSibName[1]]'s Highest Degree

	Freq.	%
1 No Formal Education(Illiterate)	1,314	30.59
2 Did not Finish Primary School	589	13.71
3 Sishu/Home School	62	1.44
4 Elementary School	855	19.91
5 Middle School	683	15.90
6 High School	314	7.31
7 Vocational School	63	1.47
8 Two-/Three-Year College/Associate Degree	42	0.98
9 Four-Year College/Bachelor's Degree	58	1.35
10 Post-graduate, Master's Degree	2	0.05
11 Post-graduate, Doctoral Degree/Ph.D	2	0.05
997 Don't Know	298	6.94
999 Refuse to Answer	13	0.30
Total	4,295	100.00

cc003_w3_2_: FamilyR's [ZSibName[2]]'s Highest Degree

	Freq.	%
1 No Formal Education(Illiterate)	1,000	28.11
2 Did not Finish Primary School	503	14.14
3 Sishu/Home School	24	0.67
4 Elementary School	807	22.68
5 Middle School	628	17.65
6 High School	232	6.52
7 Vocational School	67	1.88
8 Two-/Three-Year College/Associate Degree	34	0.96
9 Four-Year College/Bachelor's Degree	49	1.38
10 Post-graduate, Master's Degree	5	0.14
997 Don't Know	196	5.51
999 Refuse to Answer	13	0.37
Total	3,558	100.00

cc003_w3_3_: FamilyR's [ZSibName[3]]'s Highest Degree

	Freq.	%
1 No Formal Education(Illiterate)	687	25.02
2 Did not Finish Primary School	407	14.82
3 Sishu/Home School	16	0.58
4 Elementary School	651	23.71
5 Middle School	520	18.94
6 High School	213	7.76
7 Vocational School	41	1.49
8 Two-/Three-Year College/Associate Degree	16	0.58
9 Four-Year College/Bachelor's Degree	36	1.31
10 Post-graduate, Master's Degree	3	0.11
11 Post-graduate, Doctoral Degree/Ph.D	1	0.04
997 Don't Know	143	5.21
999 Refuse to Answer	12	0.44
Total	2,746	100.00

cc003_w3_4_: FamilyR's [ZSibName[4]]'s Highest Degree

	Freq.	%
1 No Formal Education(Illiterate)	483	24.71
2 Did not Finish Primary School	301	15.40
3 Sishu/Home School	9	0.46
4 Elementary School	432	22.10
5 Middle School	387	19.80
6 High School	140	7.16
7 Vocational School	35	1.79
8 Two-/Three-Year College/Associate Degree	16	0.82
9 Four-Year College/Bachelor's Degree	22	1.13
10 Post-graduate, Master's Degree	1	0.05
997 Don't Know	121	6.19
999 Refuse to Answer	8	0.41
Total	1,955	100.00

cc003_w3_5_: FamilyR's [ZSibName[5]]'s Highest Degree

	Freq.	%
1 No Formal Education(Illiterate)	273	22.23

2 Did not Finish Primary School	204	16.61
3 Sishu/Home School	5	0.41
4 Elementary School	269	21.91
5 Middle School	267	21.74
6 High School	100	8.14
7 Vocational School	20	1.63
8 Two-/Three-Year College/Associate Degree	4	0.33
9 Four-Year College/Bachelor's Degree	15	1.22
10 Post-graduate, Master's Degree	1	0.08
997 Don't Know	64	5.21
999 Refuse to Answer	6	0.49
Total	1,228	100.00

cc003_w3_6_: FamilyR's [ZSibName[6]]'s Highest Degree

	Freq.	%
1 No Formal Education(Illiterate)	140	23.49
2 Did not Finish Primary School	93	15.60
3 Sishu/Home School	1	0.17
4 Elementary School	136	22.82
5 Middle School	113	18.96
6 High School	44	7.38
7 Vocational School	9	1.51
8 Two-/Three-Year College/Associate Degree	5	0.84
9 Four-Year College/Bachelor's Degree	4	0.67
997 Don't Know	48	8.05
999 Refuse to Answer	3	0.50
Total	596	100.00

cc003_w3_7_: FamilyR's [ZSibName[7]]'s Highest Degree

	Freq.	%
1 No Formal Education(Illiterate)	71	25.27
2 Did not Finish Primary School	41	14.59
3 Sishu/Home School	1	0.36
4 Elementary School	73	25.98
5 Middle School	44	15.66
6 High School	23	8.19
7 Vocational School	3	1.07
9 Four-Year College/Bachelor's Degree	1	0.36
10 Post-graduate, Master's Degree	1	0.36
11 Post-graduate, Doctoral Degree/Ph.D	1	0.36
997 Don't Know	20	7.12
999 Refuse to Answer	2	0.71
Total	281	100.00

cc003_w3_8_: FamilyR's [ZSibName[8]]'s Highest Degree

	Freq.	%
1 No Formal Education(Illiterate)	34	28.81
2 Did not Finish Primary School	16	13.56
4 Elementary School	23	19.49
5 Middle School	18	15.25
6 High School	10	8.47
8 Two-/Three-Year College/Associate Degree	3	2.54

9 Four-Year College/Bachelor's Degree	1	0.85
997 Don't Know	11	9.32
999 Refuse to Answer	2	1.69
Total	118	100.00

cc003_w3_9_: FamilyR's [ZSibName[9]]'s Highest Degree

	Freq.	%
1 No Formal Education(Illiterate)	21	34.43
2 Did not Finish Primary School	7	11.48
4 Elementary School	11	18.03
5 Middle School	10	16.39
6 High School	3	4.92
8 Two-/Three-Year College/Associate Degree	2	3.28
997 Don't Know	5	8.20
999 Refuse to Answer	2	3.28
Total	61	100.00

cc003_w3_10_: FamilyR's [ZSibName[10]]'s Highest Degree

	Freq.	%
1 No Formal Education(Illiterate)	6	30.00
2 Did not Finish Primary School	3	15.00
4 Elementary School	5	25.00
5 Middle School	3	15.00
7 Vocational School	1	5.00
997 Don't Know	2	10.00
Total	20	100.00

cc003_w3_11_: FamilyR's [ZSibName[11]]'s Highest Degree

	Freq.	%
1 No Formal Education(Illiterate)	6	37.50
2 Did not Finish Primary School	1	6.25
4 Elementary School	3	18.75
5 Middle School	3	18.75
7 Vocational School	1	6.25
997 Don't Know	2	12.50
Total	16	100.00

cc003_w3_12_: FamilyR's [ZSibName[12]]'s Highest Degree

	Freq.	%
1 No Formal Education(Illiterate)	2	50.00
5 Middle School	1	25.00
997 Don't Know	1	25.00
Total	4	100.00

cc003_w3_13_: FamilyR's [ZSibName[13]]'s Highest Degree

	Freq.	%
5 Middle School	1	50.00

997 Don't Know	1	50.00
Total	2	100.00

cc003_w3_s_1_: Spouse's [ZSibName[1]]'s Highest Degree

	Freq.	%
1 No Formal Education(Illiterate)	861	27.50
2 Did not Finish Primary School	374	11.95
3 Sishu/Home School	18	0.57
4 Elementary School	666	21.27
5 Middle School	529	16.90
6 High School	235	7.51
7 Vocational School	28	0.89
8 Two-/Three-Year College/Associate Degree	25	0.80
9 Four-Year College/Bachelor's Degree	51	1.63
10 Post-graduate, Master's Degree	2	0.06
11 Post-graduate, Doctoral Degree/Ph.D	2	0.06
997 Don't Know	333	10.64
999 Refuse to Answer	7	0.22
Total	3,131	100.00

cc003_w3_s_2_: Spouse's [ZSibName[2]]'s Highest Degree

	Freq.	%
1 No Formal Education(Illiterate)	598	23.77
2 Did not Finish Primary School	351	13.95
3 Sishu/Home School	11	0.44
4 Elementary School	577	22.93
5 Middle School	469	18.64
6 High School	174	6.92
7 Vocational School	35	1.39
8 Two-/Three-Year College/Associate Degree	27	1.07
9 Four-Year College/Bachelor's Degree	25	0.99
10 Post-graduate, Master's Degree	3	0.12
997 Don't Know	233	9.26
999 Refuse to Answer	13	0.52
Total	2,516	100.00

cc003_w3_s_3_: Spouse's [ZSibName[3]]'s Highest Degree

	Freq.	%
1 No Formal Education(Illiterate)	430	21.38
2 Did not Finish Primary School	254	12.63
3 Sishu/Home School	1	0.05
4 Elementary School	479	23.82
5 Middle School	431	21.43
6 High School	168	8.35
7 Vocational School	29	1.44
8 Two-/Three-Year College/Associate Degree	23	1.14
9 Four-Year College/Bachelor's Degree	28	1.39
10 Post-graduate, Master's Degree	3	0.15
11 Post-graduate, Doctoral Degree/Ph.D	2	0.10
997 Don't Know	157	7.81
999 Refuse to Answer	6	0.30
Total	2,011	100.00

cc003_w3_s_4_: Spouse's [ZSibName[4]]'s Highest Degree

	Freq.	%
1 No Formal Education(Illiterate)	277	19.30
2 Did not Finish Primary School	199	13.87
3 Sishu/Home School	1	0.07
4 Elementary School	362	25.23
5 Middle School	341	23.76
6 High School	93	6.48
7 Vocational School	17	1.18
8 Two-/Three-Year College/Associate Degree	14	0.98
9 Four-Year College/Bachelor's Degree	14	0.98
10 Post-graduate, Master's Degree	1	0.07
11 Post-graduate, Doctoral Degree/Ph.D	1	0.07
997 Don't Know	106	7.39
999 Refuse to Answer	9	0.63
Total	1,435	100.00

cc003_w3_s_5_: Spouse's [ZSibName[5]]'s Highest Degree

	Freq.	%
1 No Formal Education(Illiterate)	167	19.51
2 Did not Finish Primary School	101	11.80
4 Elementary School	221	25.82
5 Middle School	208	24.30
6 High School	50	5.84
7 Vocational School	14	1.64
8 Two-/Three-Year College/Associate Degree	6	0.70
9 Four-Year College/Bachelor's Degree	9	1.05
10 Post-graduate, Master's Degree	4	0.47
997 Don't Know	68	7.94
999 Refuse to Answer	8	0.93
Total	856	100.00

cc003_w3_s_6_: Spouse's [ZSibName[6]]'s Highest Degree

	Freq.	%
1 No Formal Education(Illiterate)	89	19.22
2 Did not Finish Primary School	51	11.02
4 Elementary School	122	26.35
5 Middle School	118	25.49
6 High School	29	6.26
7 Vocational School	6	1.30
8 Two-/Three-Year College/Associate Degree	4	0.86
9 Four-Year College/Bachelor's Degree	3	0.65
997 Don't Know	37	7.99
999 Refuse to Answer	4	0.86
Total	463	100.00

cc003_w3_s_7_: Spouse's [ZSibName[7]]'s Highest Degree

	Freq.	%
1 No Formal Education(Illiterate)	33	17.10
2 Did not Finish Primary School	26	13.47

4 Elementary School	52	26.94
5 Middle School	47	24.35
6 High School	9	4.66
7 Vocational School	1	0.52
8 Two-/Three-Year College/Associate Degree	2	1.04
9 Four-Year College/Bachelor's Degree	1	0.52
997 Don't Know	19	9.84
999 Refuse to Answer	3	1.55
Total	193	100.00

cc003_w3_s_8_: Spouse's [ZSibName[8]]'s Highest Degree

	Freq.	%
1 No Formal Education(Illiterate)	16	18.39
2 Did not Finish Primary School	10	11.49
4 Elementary School	20	22.99
5 Middle School	18	20.69
6 High School	6	6.90
7 Vocational School	1	1.15
8 Two-/Three-Year College/Associate Degree	1	1.15
997 Don't Know	11	12.64
999 Refuse to Answer	4	4.60
Total	87	100.00

cc003_w3_s_9_: Spouse's [ZSibName[9]]'s Highest Degree

	Freq.	%
1 No Formal Education(Illiterate)	11	30.56
2 Did not Finish Primary School	2	5.56
4 Elementary School	5	13.89
5 Middle School	6	16.67
6 High School	1	2.78
7 Vocational School	1	2.78
9 Four-Year College/Bachelor's Degree	1	2.78
997 Don't Know	7	19.44
999 Refuse to Answer	2	5.56
Total	36	100.00

cc003_w3_s_10_: Spouse's [ZSibName[10]]'s Highest Degree

	Freq.	%
1 No Formal Education(Illiterate)	2	14.29
2 Did not Finish Primary School	3	21.43
4 Elementary School	3	21.43
5 Middle School	2	14.29
6 High School	1	7.14
997 Don't Know	1	7.14
999 Refuse to Answer	2	14.29
Total	14	100.00

cc003_w3_s_11_: Spouse's [ZSibName[11]]'s Highest Degree

	Freq.	%

	Freq.	%
5 Middle School	1	25.00
997 Don't Know	2	50.00
999 Refuse to Answer	1	25.00
Total	4	100.00

cc003_w3_s_12_: Spouse's [ZSibName[12]]'s Highest Degree

	Freq.	%
997 Don't Know	2	66.67
999 Refuse to Answer	1	33.33
Total	3	100.00

cc003_w3_s_13_: Spouse's [ZSibName[13]]'s Highest Degree

	Freq.	%
999 Refuse to Answer	1	100.00
Total	1	100.00

cc003_w3_s_14_: Spouse's [ZSibName[14]]'s Highest Degree

	Freq.	%
997 Don't Know	1	50.00
999 Refuse to Answer	1	50.00
Total	2	100.00

cc003_w3_s_15_: Spouse's [ZSibName[15]]'s Highest Degree

	Freq.	%
1 No Formal Education(Illiterate)	1	50.00
999 Refuse to Answer	1	50.00
Total	2	100.00

cc003_w3_0_1_: Is Respondent's [ZSibName[1]] Literate

	Freq.	%
1 Yes	645	32.82
2 No	1,320	67.18
Total	1,965	100.00

cc003_w3_0_2_: Is Respondent's [ZSibName[2]] Literate

	Freq.	%
1 Yes	500	32.74
2 No	1,027	67.26
Total	1,527	100.00

cc003_w3_0_3_: Is Respondent's [ZSibName[3]] Literate

	Freq.	%

	Freq.	%
1 Yes	390	35.14
2 No	720	64.86
Total	1,110	100.00

cc003_w3_0_4_: Is Respondent's [ZSibName[4]] Literate

	Freq.	%
1 Yes	285	35.94
2 No	508	64.06
Total	793	100.00

cc003_w3_0_5_: Is Respondent's [ZSibName[5]] Literate

	Freq.	%
1 Yes	183	37.97
2 No	299	62.03
Total	482	100.00

cc003_w3_0_6_: Is Respondent's [ZSibName[6]] Literate

	Freq.	%
1 Yes	83	35.47
2 No	151	64.53
Total	234	100.00

cc003_w3_0_7_: Is Respondent's [ZSibName[7]] Literate

	Freq.	%
1 Yes	37	32.74
2 No	76	67.26
Total	113	100.00

cc003_w3_0_8_: Is Respondent's [ZSibName[8]] Literate

	Freq.	%
1 Yes	15	30.00
2 No	35	70.00
Total	50	100.00

cc003_w3_0_9_: Is Respondent's [ZSibName[9]] Literate

	Freq.	%
1 Yes	6	21.43
2 No	22	78.57
Total	28	100.00

cc003_w3_0_10_: Is Respondent's [ZSibName[10]] Literate

	Freq.	%

	Freq.	%
1 Yes	3	33.33
2 No	6	66.67
Total	9	100.00

cc003_w3_0_11_: Is Respondent's [ZSibName[11]] Literate

	Freq.	%
1 Yes	2	28.57
2 No	5	71.43
Total	7	100.00

cc003_w3_0_12_: Is Respondent's [ZSibName[12]] Literate

	Freq.	%
2 No	2	100.00
Total	2	100.00

cc003_w3_0_s_1_: Is Respondent's [ZSibName[1]] Literate

	Freq.	%
1 Yes	382	30.49
2 No	871	69.51
Total	1,253	100.00

cc003_w3_0_s_2_: Is Respondent's [ZSibName[2]] Literate

	Freq.	%
1 Yes	329	34.27
2 No	631	65.73
Total	960	100.00

cc003_w3_0_s_3_: Is Respondent's [ZSibName[3]] Literate

	Freq.	%
1 Yes	225	32.85
2 No	460	67.15
Total	685	100.00

cc003_w3_0_s_4_: Is Respondent's [ZSibName[4]] Literate

	Freq.	%
1 Yes	177	37.11
2 No	300	62.89
Total	477	100.00

cc003_w3_0_s_5_: Is Respondent's [ZSibName[5]] Literate

	Freq.	%
1 Yes	97	36.19

	Freq.	%
2 No	171	63.81
Total	268	100.00

cc003_w3_0_s_6_: Is Respondent's [ZSibName[6]] Literate

	Freq.	%
1 Yes	46	32.86
2 No	94	67.14
Total	140	100.00

cc003_w3_0_s_7_: Is Respondent's [ZSibName[7]] Literate

	Freq.	%
1 Yes	26	44.07
2 No	33	55.93
Total	59	100.00

cc003_w3_0_s_8_: Is Respondent's [ZSibName[8]] Literate

	Freq.	%
1 Yes	9	34.62
2 No	17	65.38
Total	26	100.00

cc003_w3_0_s_9_: Is Respondent's [ZSibName[9]] Literate

	Freq.	%
1 Yes	3	23.08
2 No	10	76.92
Total	13	100.00

cc003_w3_0_s_10_: Is Respondent's [ZSibName[10]] Literate

	Freq.	%
1 Yes	3	60.00
2 No	2	40.00
Total	5	100.00

cc003_w3_0_s_11_: Is Respondent's [ZSibName[11]] Literate

No Observations

cc003_w3_0_s_12_: Is Respondent's [ZSibName[12]] Literate

No Observations

cc015_w3_1_: FamilyR's [ZSibName[1]]'s Highest Professional Position

	Freq.	%
1 Managers	609	5.46
2 Professionals and Technicians	923	8.27
3 Clerks	369	3.31
4 Commercial and Service Workers	626	5.61
5 Agricultural Forestry, Husbandry and Fishery Producers	6,991	62.66
6 Production and Transportation Workers	797	7.14
7 Others	842	7.55
Total	11,157	100.00

cc015_w3_2_: FamilyR's [ZSibName[2]]'s Highest Professional Position

	Freq.	%
1 Managers	483	4.64
2 Professionals and Technicians	771	7.41
3 Clerks	300	2.88
4 Commercial and Service Workers	642	6.17
5 Agricultural Forestry, Husbandry and Fishery Producers	6,589	63.31
6 Production and Transportation Workers	840	8.07
7 Others	783	7.52
Total	10,408	100.00

cc015_w3_3_: FamilyR's [ZSibName[3]]'s Highest Professional Position

	Freq.	%
1 Managers	344	3.94
2 Professionals and Technicians	638	7.30
3 Clerks	237	2.71
4 Commercial and Service Workers	648	7.42
5 Agricultural Forestry, Husbandry and Fishery Producers	5,507	63.04
6 Production and Transportation Workers	693	7.93
7 Others	669	7.66
Total	8,736	100.00

cc015_w3_4_: FamilyR's [ZSibName[4]]'s Highest Professional Position

	Freq.	%
1 Managers	238	3.72
2 Professionals and Technicians	435	6.79
3 Clerks	149	2.33
4 Commercial and Service Workers	501	7.83
5 Agricultural Forestry, Husbandry and Fishery Producers	4,038	63.07
6 Production and Transportation Workers	550	8.59
7 Others	491	7.67
Total	6,402	100.00

cc015_w3_5_: FamilyR's [ZSibName[5]]'s Highest Professional Position

	Freq.	%
1 Managers	141	3.50
2 Professionals and Technicians	252	6.26
3 Clerks	86	2.14

4 Commercial and Service Workers	303	7.52
5 Agricultural Forestry, Husbandry and Fishery Producers	2,579	64.03
6 Production and Transportation Workers	351	8.71
7 Others	316	7.85
Total	4,028	100.00

cc015_w3_6_: FamilyR's [ZSibName[6]]'s Highest Professional Position

	Freq.	%
1 Managers	77	3.82
2 Professionals and Technicians	124	6.14
3 Clerks	48	2.38
4 Commercial and Service Workers	165	8.18
5 Agricultural Forestry, Husbandry and Fishery Producers	1,277	63.28
6 Production and Transportation Workers	147	7.28
7 Others	180	8.92
Total	2,018	100.00

cc015_w3_7_: FamilyR's [ZSibName[7]]'s Highest Professional Position

	Freq.	%
1 Managers	31	3.60
2 Professionals and Technicians	42	4.87
3 Clerks	24	2.78
4 Commercial and Service Workers	63	7.31
5 Agricultural Forestry, Husbandry and Fishery Producers	561	65.08
6 Production and Transportation Workers	65	7.54
7 Others	76	8.82
Total	862	100.00

cc015_w3_8_: FamilyR's [ZSibName[8]]'s Highest Professional Position

	Freq.	%
1 Managers	12	3.76
2 Professionals and Technicians	17	5.33
3 Clerks	4	1.25
4 Commercial and Service Workers	15	4.70
5 Agricultural Forestry, Husbandry and Fishery Producers	208	65.20
6 Production and Transportation Workers	31	9.72
7 Others	32	10.03
Total	319	100.00

cc015_w3_9_: FamilyR's [ZSibName[9]]'s Highest Professional Position

	Freq.	%
1 Managers	6	4.38
2 Professionals and Technicians	10	7.30
3 Clerks	2	1.46
4 Commercial and Service Workers	7	5.11
5 Agricultural Forestry, Husbandry and Fishery Producers	82	59.85
6 Production and Transportation Workers	16	11.68
7 Others	14	10.22
Total	137	100.00

cc015_w3_10_: FamilyR's [ZSibName[10]]'s Highest Professional Position

	Freq.	%
1 Managers	2	4.65
3 Clerks	1	2.33
4 Commercial and Service Workers	2	4.65
5 Agricultural Forestry, Husbandry and Fishery Producers	31	72.09
6 Production and Transportation Workers	3	6.98
7 Others	4	9.30
Total	43	100.00

cc015_w3_11_: FamilyR's [ZSibName[11]]'s Highest Professional Position

	Freq.	%
1 Managers	1	3.70
2 Professionals and Technicians	2	7.41
3 Clerks	1	3.70
4 Commercial and Service Workers	1	3.70
5 Agricultural Forestry, Husbandry and Fishery Producers	14	51.85
6 Production and Transportation Workers	2	7.41
7 Others	6	22.22
Total	27	100.00

cc015_w3_12_: FamilyR's [ZSibName[12]]'s Highest Professional Position

	Freq.	%
5 Agricultural Forestry, Husbandry and Fishery Producers	6	75.00
7 Others	2	25.00
Total	8	100.00

cc015_w3_13_: FamilyR's [ZSibName[13]]'s Highest Professional Position

	Freq.	%
5 Agricultural Forestry, Husbandry and Fishery Producers	1	33.33
6 Production and Transportation Workers	2	66.67
Total	3	100.00

cc015_w3_14_: FamilyR's [ZSibName[14]]'s Highest Professional Position

	Freq.	%
6 Production and Transportation Workers	1	100.00
Total	1	100.00

cc015_w3_s_1_: Spouse's [ZSibName[1]]'s Highest Professional Position

	Freq.	%
1 Managers	392	4.67
2 Professionals and Technicians	635	7.56
3 Clerks	271	3.23
4 Commercial and Service Workers	401	4.78
5 Agricultural Forestry, Husbandry and Fishery Producers	5,522	65.76

	Freq.	%
6 Production and Transportation Workers	637	7.59
7 Others	539	6.42
Total	8,397	100.00

cc015_w3_s_2_: Spouse's [ZSibName[2]]'s Highest Professional Position

	Freq.	%
1 Managers	324	4.14
2 Professionals and Technicians	538	6.87
3 Clerks	214	2.73
4 Commercial and Service Workers	462	5.90
5 Agricultural Forestry, Husbandry and Fishery Producers	5,119	65.39
6 Production and Transportation Workers	643	8.21
7 Others	529	6.76
Total	7,829	100.00

cc015_w3_s_3_: Spouse's [ZSibName[3]]'s Highest Professional Position

	Freq.	%
1 Managers	227	3.43
2 Professionals and Technicians	464	7.02
3 Clerks	163	2.46
4 Commercial and Service Workers	453	6.85
5 Agricultural Forestry, Husbandry and Fishery Producers	4,333	65.52
6 Production and Transportation Workers	528	7.98
7 Others	445	6.73
Total	6,613	100.00

cc015_w3_s_4_: Spouse's [ZSibName[4]]'s Highest Professional Position

	Freq.	%
1 Managers	170	3.57
2 Professionals and Technicians	306	6.42
3 Clerks	106	2.23
4 Commercial and Service Workers	319	6.70
5 Agricultural Forestry, Husbandry and Fishery Producers	3,155	66.24
6 Production and Transportation Workers	391	8.21
7 Others	316	6.63
Total	4,763	100.00

cc015_w3_s_5_: Spouse's [ZSibName[5]]'s Highest Professional Position

	Freq.	%
1 Managers	90	3.05
2 Professionals and Technicians	179	6.07
3 Clerks	61	2.07
4 Commercial and Service Workers	218	7.39
5 Agricultural Forestry, Husbandry and Fishery Producers	1,978	67.05
6 Production and Transportation Workers	209	7.08
7 Others	215	7.29
Total	2,950	100.00

cc015_w3_s_6_: Spouse's [ZSibName[6]]'s Highest Professional Position

	Freq.	%
1 Managers	36	2.40
2 Professionals and Technicians	81	5.40
3 Clerks	31	2.07
4 Commercial and Service Workers	129	8.59
5 Agricultural Forestry, Husbandry and Fishery Producers	1,020	67.95
6 Production and Transportation Workers	98	6.53
7 Others	106	7.06
Total	1,501	100.00

cc015_w3_s_7_: Spouse's [ZSibName[7]]'s Highest Professional Position

	Freq.	%
1 Managers	12	1.76
2 Professionals and Technicians	39	5.71
3 Clerks	11	1.61
4 Commercial and Service Workers	47	6.88
5 Agricultural Forestry, Husbandry and Fishery Producers	452	66.18
6 Production and Transportation Workers	61	8.93
7 Others	61	8.93
Total	683	100.00

cc015_w3_s_8_: Spouse's [ZSibName[8]]'s Highest Professional Position

	Freq.	%
1 Managers	2	0.75
2 Professionals and Technicians	21	7.84
3 Clerks	6	2.24
4 Commercial and Service Workers	19	7.09
5 Agricultural Forestry, Husbandry and Fishery Producers	170	63.43
6 Production and Transportation Workers	23	8.58
7 Others	27	10.07
Total	268	100.00

cc015_w3_s_9_: Spouse's [ZSibName[9]]'s Highest Professional Position

	Freq.	%
1 Managers	3	3.30
2 Professionals and Technicians	5	5.49
3 Clerks	4	4.40
4 Commercial and Service Workers	7	7.69
5 Agricultural Forestry, Husbandry and Fishery Producers	56	61.54
6 Production and Transportation Workers	8	8.79
7 Others	8	8.79
Total	91	100.00

cc015_w3_s_10_: Spouse's [ZSibName[10]]'s Highest Professional Position

	Freq.	%
2 Professionals and Technicians	2	7.14

4 Commercial and Service Workers	1	3.57
5 Agricultural Forestry, Husbandry and Fishery Producers	17	60.71
6 Production and Transportation Workers	3	10.71
7 Others	5	17.86
Total	28	100.00

cc015_w3_s_11_: Spouse's [ZSibName[11]]'s Highest Professional Position

	Freq.	%
5 Agricultural Forestry, Husbandry and Fishery Producers	7	58.33
6 Production and Transportation Workers	2	16.67
7 Others	3	25.00
Total	12	100.00

cc015_w3_s_12_: Spouse's [ZSibName[12]]'s Highest Professional Position

	Freq.	%
5 Agricultural Forestry, Husbandry and Fishery Producers	4	57.14
6 Production and Transportation Workers	1	14.29
7 Others	2	28.57
Total	7	100.00

cc015_w3_s_13_: Spouse's [ZSibName[13]]'s Highest Professional Position

	Freq.	%
5 Agricultural Forestry, Husbandry and Fishery Producers	1	50.00
6 Production and Transportation Workers	1	50.00
Total	2	100.00

cc015_w3_s_14_: Spouse's [ZSibName[14]]'s Highest Professional Position

	Freq.	%
6 Production and Transportation Workers	1	50.00
7 Others	1	50.00
Total	2	100.00

cc015_w3_s_15_: Spouse's [ZSibName[15]]'s Highest Professional Position

	Freq.	%
5 Agricultural Forestry, Husbandry and Fishery Producers	1	50.00
6 Production and Transportation Workers	1	50.00
Total	2	100.00

cc004_w3_1_: Is FamilyR's [ZSibName[1]] Communist Party Member

	Freq.	%
1 Yes	1,023	9.17
2 No	9,611	86.14
997 Don't Know	513	4.60
999 Refuse to Answer	10	0.09

Total	11,157	100.00

cc004_w3_2_: Is FamilyR's [ZSibName[2]] Communist Party Member

	Freq.	%
1 Yes	773	7.43
2 No	9,233	88.71
997 Don't Know	387	3.72
999 Refuse to Answer	15	0.14
Total	10,408	100.00

cc004_w3_3_: Is FamilyR's [ZSibName[3]] Communist Party Member

	Freq.	%
1 Yes	530	6.07
2 No	7,839	89.73
997 Don't Know	355	4.06
999 Refuse to Answer	12	0.14
Total	8,736	100.00

cc004_w3_4_: Is FamilyR's [ZSibName[4]] Communist Party Member

	Freq.	%
1 Yes	385	6.01
2 No	5,766	90.08
997 Don't Know	238	3.72
999 Refuse to Answer	12	0.19
Total	6,401	100.00

cc004_w3_5_: Is FamilyR's [ZSibName[5]] Communist Party Member

	Freq.	%
1 Yes	230	5.71
2 No	3,648	90.57
997 Don't Know	142	3.53
999 Refuse to Answer	8	0.20
Total	4,028	100.00

cc004_w3_6_: Is FamilyR's [ZSibName[6]] Communist Party Member

	Freq.	%
1 Yes	91	4.51
2 No	1,835	90.93
997 Don't Know	88	4.36
999 Refuse to Answer	4	0.20
Total	2,018	100.00

cc004_w3_7_: Is FamilyR's [ZSibName[7]] Communist Party Member

	Freq.	%

1 Yes	36	4.18
2 No	782	90.72
997 Don't Know	41	4.76
999 Refuse to Answer	3	0.35
Total	862	100.00

cc004_w3_8_: Is FamilyR's [ZSibName[8]] Communist Party Member

	Freq.	%
1 Yes	14	4.39
2 No	285	89.34
997 Don't Know	18	5.64
999 Refuse to Answer	2	0.63
Total	319	100.00

cc004_w3_9_: Is FamilyR's [ZSibName[9]] Communist Party Member

	Freq.	%
1 Yes	7	5.11
2 No	120	87.59
997 Don't Know	7	5.11
999 Refuse to Answer	3	2.19
Total	137	100.00

cc004_w3_10_: Is FamilyR's [ZSibName[10]] Communist Party Member

	Freq.	%
1 Yes	3	6.98
2 No	38	88.37
997 Don't Know	2	4.65
Total	43	100.00

cc004_w3_11_: Is FamilyR's [ZSibName[11]] Communist Party Member

	Freq.	%
1 Yes	1	3.70
2 No	23	85.19
997 Don't Know	3	11.11
Total	27	100.00

cc004_w3_12_: Is FamilyR's [ZSibName[12]] Communist Party Member

	Freq.	%
2 No	7	87.50
997 Don't Know	1	12.50
Total	8	100.00

cc004_w3_13_: Is FamilyR's [ZSibName[13]] Communist Party Member

	Freq.	%

	Freq.	%
2 No	2	66.67
997 Don't Know	1	33.33
Total	3	100.00

cc004_w3_14_: Is FamilyR's [ZSibName[14]] Communist Party Member

	Freq.	%
2 No	1	100.00
Total	1	100.00

cc004_w3_s_1_: Is Spouse's [ZSibName[1]] Communist Party Member

	Freq.	%
1 Yes	690	8.22
2 No	7,308	87.03
997 Don't Know	389	4.63
999 Refuse to Answer	10	0.12
Total	8,397	100.00

cc004_w3_s_2_: Is Spouse's [ZSibName[2]] Communist Party Member

	Freq.	%
1 Yes	518	6.62
2 No	6,971	89.04
997 Don't Know	326	4.16
999 Refuse to Answer	14	0.18
Total	7,829	100.00

cc004_w3_s_3_: Is Spouse's [ZSibName[3]] Communist Party Member

	Freq.	%
1 Yes	356	5.38
2 No	5,996	90.67
997 Don't Know	253	3.83
999 Refuse to Answer	8	0.12
Total	6,613	100.00

cc004_w3_s_4_: Is Spouse's [ZSibName[4]] Communist Party Member

	Freq.	%
1 Yes	221	4.64
2 No	4,372	91.79
997 Don't Know	159	3.34
999 Refuse to Answer	11	0.23
Total	4,763	100.00

cc004_w3_s_5_: Is Spouse's [ZSibName[5]] Communist Party Member

	Freq.	%
1 Yes	133	4.51

	Freq.	%
2 No	2,703	91.63
997 Don't Know	104	3.53
999 Refuse to Answer	10	0.34
Total	2,950	100.00

cc004_w3_s_6_: Is Spouse's [ZSibName[6]] Communist Party Member

	Freq.	%
1 Yes	50	3.33
2 No	1,395	92.94
997 Don't Know	51	3.40
999 Refuse to Answer	5	0.33
Total	1,501	100.00

cc004_w3_s_7_: Is Spouse's [ZSibName[7]] Communist Party Member

	Freq.	%
1 Yes	32	4.69
2 No	626	91.65
997 Don't Know	22	3.22
999 Refuse to Answer	3	0.44
Total	683	100.00

cc004_w3_s_8_: Is Spouse's [ZSibName[8]] Communist Party Member

	Freq.	%
1 Yes	8	2.99
2 No	244	91.04
997 Don't Know	12	4.48
999 Refuse to Answer	4	1.49
Total	268	100.00

cc004_w3_s_9_: Is Spouse's [ZSibName[9]] Communist Party Member

	Freq.	%
1 Yes	4	4.40
2 No	77	84.62
997 Don't Know	8	8.79
999 Refuse to Answer	2	2.20
Total	91	100.00

cc004_w3_s_10_: Is Spouse's [ZSibName[10]] Communist Party Member

	Freq.	%
2 No	23	82.14
997 Don't Know	3	10.71
999 Refuse to Answer	2	7.14
Total	28	100.00

cc004_w3_s_11_: Is Spouse's [ZSibName[11]] Communist Party Member

	Freq.	%
2 No	9	75.00
997 Don't Know	2	16.67
999 Refuse to Answer	1	8.33
Total	12	100.00

cc004_w3_s_12_: Is Spouse's [ZSibName[12]] Communist Party Member

	Freq.	%
2 No	4	57.14
997 Don't Know	2	28.57
999 Refuse to Answer	1	14.29
Total	7	100.00

cc004_w3_s_13_: Is Spouse's [ZSibName[13]] Communist Party Member

	Freq.	%
2 No	1	50.00
999 Refuse to Answer	1	50.00
Total	2	100.00

cc004_w3_s_14_: Is Spouse's [ZSibName[14]] Communist Party Member

	Freq.	%
2 No	1	50.00
999 Refuse to Answer	1	50.00
Total	2	100.00

cc004_w3_s_15_: Is Spouse's [ZSibName[15]] Communist Party Member

	Freq.	%
2 No	1	50.00
999 Refuse to Answer	1	50.00
Total	2	100.00

cc004_w3_1_1_: Which Year

Mean	SD	Min	Max	Obs
1,957.38	179.97	0.00	2,018.00	805

cc004_w3_1_2_: Which Year

Mean	SD	Min	Max	Obs
1,973.53	75.25	198.00	2,018.00	630

cc004_w3_1_3_: Which Year

Mean	SD	Min	Max	Obs
1,954.34	231.92	0.00	2,018.00	436

cc004_w3_1_4_: Which Year

Mean	SD	Min	Max	Obs
1,966.56	162.54	0.00	2,018.00	300

cc004_w3_1_5_: Which Year

Mean	SD	Min	Max	Obs
1,946.32	256.78	19.00	2,018.00	186

cc004_w3_1_6_: Which Year

Mean	SD	Min	Max	Obs
1,929.29	313.04	0.00	2,018.00	72

cc004_w3_1_7_: Which Year

Mean	SD	Min	Max	Obs
1,983.87	30.55	1,900.00	2,018.00	23

cc004_w3_1_8_: Which Year

Mean	SD	Min	Max	Obs
1,976.42	38.55	1,900.00	2,016.00	12

cc004_w3_1_9_: Which Year

Mean	SD	Min	Max	Obs
1,978.67	42.24	1,900.00	2,018.00	6

cc004_w3_1_10_: Which Year

Mean	SD	Min	Max	Obs
1,987.00	2.83	1,985.00	1,989.00	2

cc004_w3_1_11_: Which Year

No Observations

cc004_w3_1_s_1_: Which Year

Mean	SD	Min	Max	Obs

1,951.96	205.65	0.00	2,018.00	533

cc004_w3_1_s_2_: Which Year

Mean	SD	Min	Max	Obs
1,968.20	140.52	0.00	2,018.00	410

cc004_w3_1_s_3_: Which Year

Mean	SD	Min	Max	Obs
1,957.79	202.93	0.00	2,018.00	285

cc004_w3_1_s_4_: Which Year

Mean	SD	Min	Max	Obs
1,955.46	210.40	0.00	2,018.00	179

cc004_w3_1_s_5_: Which Year

Mean	SD	Min	Max	Obs
1,981.38	27.12	1,900.00	2,018.00	103

cc004_w3_1_s_6_: Which Year

Mean	SD	Min	Max	Obs
1,979.00	33.43	1,900.00	2,018.00	33

cc004_w3_1_s_7_: Which Year

Mean	SD	Min	Max	Obs
1,983.68	29.77	1,900.00	2,016.00	25

cc004_w3_1_s_8_: Which Year

Mean	SD	Min	Max	Obs
1,994.88	12.22	1,975.00	2,016.00	8

cc004_w3_1_s_9_: Which Year

Mean	SD	Min	Max	Obs
1,994.00	16.57	1,976.00	2,014.00	4

cc016_w3_1_: FamilyR's [ZSibName[1]] Has Ever Become as Educated Youth

	Freq.	%
1 Yes	295	2.64

2 No	10,622	95.20
997 Don't Know	226	2.03
999 Refuse to Answer	14	0.13
Total	11,157	100.00

cc016_w3_2_: FamilyR's [ZSibName[2]] Has Ever Become as Educated Youth

	Freq.	%
1 Yes	241	2.32
2 No	9,987	95.96
997 Don't Know	164	1.58
999 Refuse to Answer	16	0.15
Total	10,408	100.00

cc016_w3_3_: FamilyR's [ZSibName[3]] Has Ever Become as Educated Youth

	Freq.	%
1 Yes	179	2.05
2 No	8,435	96.55
997 Don't Know	112	1.28
999 Refuse to Answer	10	0.11
Total	8,736	100.00

cc016_w3_4_: FamilyR's [ZSibName[4]] Has Ever Become as Educated Youth

	Freq.	%
1 Yes	108	1.69
2 No	6,181	96.56
997 Don't Know	98	1.53
999 Refuse to Answer	14	0.22
Total	6,401	100.00

cc016_w3_5_: FamilyR's [ZSibName[5]] Has Ever Become as Educated Youth

	Freq.	%
1 Yes	54	1.34
2 No	3,909	97.05
997 Don't Know	57	1.42
999 Refuse to Answer	8	0.20
Total	4,028	100.00

cc016_w3_6_: FamilyR's [ZSibName[6]] Has Ever Become as Educated Youth

	Freq.	%
1 Yes	20	0.99
2 No	1,961	97.18
997 Don't Know	33	1.64
999 Refuse to Answer	4	0.20
Total	2,018	100.00

cc016_w3_7_: FamilyR's [ZSibName[7]] Has Ever Become as Educated Youth

	Freq.	%
1 Yes	9	1.04
2 No	829	96.17
997 Don't Know	21	2.44
999 Refuse to Answer	3	0.35
Total	862	100.00

cc016_w3_8_: FamilyR's [ZSibName[8]] Has Ever Become as Educated Youth

	Freq.	%
1 Yes	2	0.63
2 No	305	95.61
997 Don't Know	10	3.13
999 Refuse to Answer	2	0.63
Total	319	100.00

cc016_w3_9_: FamilyR's [ZSibName[9]] Has Ever Become as Educated Youth

	Freq.	%
2 No	131	95.62
997 Don't Know	3	2.19
999 Refuse to Answer	3	2.19
Total	137	100.00

cc016_w3_10_: FamilyR's [ZSibName[10]] Has Ever Become as Educated Youth

	Freq.	%
2 No	42	97.67
997 Don't Know	1	2.33
Total	43	100.00

cc016_w3_11_: FamilyR's [ZSibName[11]] Has Ever Become as Educated Youth

	Freq.	%
2 No	26	96.30
997 Don't Know	1	3.70
Total	27	100.00

cc016_w3_12_: FamilyR's [ZSibName[12]] Has Ever Become as Educated Youth

	Freq.	%
2 No	7	87.50
997 Don't Know	1	12.50
Total	8	100.00

cc016_w3_13_: FamilyR's [ZSibName[13]] Has Ever Become as Educated Youth

	Freq.	%
2 No	2	66.67

	Freq.	%
997 Don't Know	1	33.33
Total	3	100.00

cc016_w3_14_: FamilyR's [ZSibName[14]] Has Ever Become as Educated Youth

	Freq.	%
2 No	1	100.00
Total	1	100.00

cc016_w3_s_1_: Spouse's [ZSibName[1]] Has Ever Become as Educated Youth

	Freq.	%
1 Yes	164	1.95
2 No	8,053	95.90
997 Don't Know	170	2.02
999 Refuse to Answer	10	0.12
Total	8,397	100.00

cc016_w3_s_2_: Spouse's [ZSibName[2]] Has Ever Become as Educated Youth

	Freq.	%
1 Yes	132	1.69
2 No	7,549	96.42
997 Don't Know	134	1.71
999 Refuse to Answer	14	0.18
Total	7,829	100.00

cc016_w3_s_3_: Spouse's [ZSibName[3]] Has Ever Become as Educated Youth

	Freq.	%
1 Yes	101	1.53
2 No	6,404	96.84
997 Don't Know	98	1.48
999 Refuse to Answer	10	0.15
Total	6,613	100.00

cc016_w3_s_4_: Spouse's [ZSibName[4]] Has Ever Become as Educated Youth

	Freq.	%
1 Yes	60	1.26
2 No	4,625	97.10
997 Don't Know	67	1.41
999 Refuse to Answer	11	0.23
Total	4,763	100.00

cc016_w3_s_5_: Spouse's [ZSibName[5]] Has Ever Become as Educated Youth

	Freq.	%
1 Yes	20	0.68
2 No	2,882	97.69

	Freq.	%
997 Don't Know	38	1.29
999 Refuse to Answer	10	0.34
Total	2,950	100.00

cc016_w3_s_6_: Spouse's [ZSibName[6]] Has Ever Become as Educated Youth

	Freq.	%
1 Yes	12	0.80
2 No	1,467	97.73
997 Don't Know	16	1.07
999 Refuse to Answer	6	0.40
Total	1,501	100.00

cc016_w3_s_7_: Spouse's [ZSibName[7]] Has Ever Become as Educated Youth

	Freq.	%
1 Yes	3	0.44
2 No	663	97.07
997 Don't Know	14	2.05
999 Refuse to Answer	3	0.44
Total	683	100.00

cc016_w3_s_8_: Spouse's [ZSibName[8]] Has Ever Become as Educated Youth

	Freq.	%
1 Yes	1	0.37
2 No	255	95.15
997 Don't Know	8	2.99
999 Refuse to Answer	4	1.49
Total	268	100.00

cc016_w3_s_9_: Spouse's [ZSibName[9]] Has Ever Become as Educated Youth

	Freq.	%
1 Yes	1	1.10
2 No	83	91.21
997 Don't Know	5	5.49
999 Refuse to Answer	2	2.20
Total	91	100.00

cc016_w3_s_10_: Spouse's [ZSibName[10]] Has Ever Become as Educated Youth

	Freq.	%
2 No	24	85.71
997 Don't Know	2	7.14
999 Refuse to Answer	2	7.14
Total	28	100.00

cc016_w3_s_11_: Spouse's [ZSibName[11]] Has Ever Become as Educated Youth

	Freq.	%
1 Yes	1	8.33
2 No	8	66.67
997 Don't Know	2	16.67
999 Refuse to Answer	1	8.33
Total	12	100.00

cc016_w3_s_12_: Spouse's [ZSibName[12]] Has Ever Become as Educated Youth

	Freq.	%
2 No	3	42.86
997 Don't Know	3	42.86
999 Refuse to Answer	1	14.29
Total	7	100.00

cc016_w3_s_13_: Spouse's [ZSibName[13]] Has Ever Become as Educated Youth

	Freq.	%
2 No	1	50.00
999 Refuse to Answer	1	50.00
Total	2	100.00

cc016_w3_s_14_: Spouse's [ZSibName[14]] Has Ever Become as Educated Youth

	Freq.	%
2 No	1	50.00
999 Refuse to Answer	1	50.00
Total	2	100.00

cc016_w3_s_15_: Spouse's [ZSibName[15]] Has Ever Become as Educated Youth

	Freq.	%
2 No	1	50.00
999 Refuse to Answer	1	50.00
Total	2	100.00

cc016_w3_1_1_: Which Year

Mean	SD	Min	Max	Obs
1,948.40	200.36	19.00	2,000.00	283

cc016_w3_1_2_: Which Year

Mean	SD	Min	Max	Obs
1,970.22	9.40	1,900.00	2,018.00	227

cc016_w3_1_3_: Which Year

Mean	SD	Min	Max	Obs
1,969.90	13.53	1,900.00	2,018.00	172

cc016_w3_1_4_: Which Year

Mean	SD	Min	Max	Obs
1,952.86	196.26	1.00	2,000.00	101

cc016_w3_1_5_: Which Year

Mean	SD	Min	Max	Obs
1,971.84	5.78	1,958.00	1,997.00	51

cc016_w3_1_6_: Which Year

Mean	SD	Min	Max	Obs
1,848.19	487.02	22.00	1,977.00	16

cc016_w3_1_7_: Which Year

Mean	SD	Min	Max	Obs
1,972.14	5.58	1,965.00	1,982.00	7

cc016_w3_1_8_: Which Year

Mean	SD	Min	Max	Obs
1,970.50	3.54	1,968.00	1,973.00	2

cc016_w3_1_s_1_: Which Year

Mean	SD	Min	Max	Obs
1,945.95	209.99	20.00	2,018.00	157

cc016_w3_1_s_2_: Which Year

Mean	SD	Min	Max	Obs
1,938.66	247.08	1.00	2,010.00	125

cc016_w3_1_s_3_: Which Year

Mean	SD	Min	Max	Obs
1,952.02	195.61	26.00	2,000.00	99

cc016_w3_1_s_4_: Which Year

Mean	SD	Min	Max	Obs
1,940.52	251.61	25.00	2,013.00	60

cc016_w3_1_s_5_: Which Year

Mean	SD	Min	Max	Obs
1,879.29	426.04	20.00	1,978.00	21

cc016_w3_1_s_6_: Which Year

Mean	SD	Min	Max	Obs
1,975.00	16.23	1,951.00	2,018.00	11

cc016_w3_1_s_7_: Which Year

Mean	SD	Min	Max	Obs
1,970.00	6.93	1,962.00	1,974.00	3

cc016_w3_1_s_8_: Which Year

No Observations

cc016_w3_1_s_9_: Which Year

No Observations

cc016_w3_1_s_10_: Which Year

No Observations

cc016_w3_1_s_11_: Which Year

No Observations

cc016_w4_1_: Did FamilyR's [ZSibName[1]] Return to Urban

	Freq.	%
1 Yes	227	76.95
2 No	44	14.92
997 Don't Know	24	8.14
Total	295	100.00

cc016_w4_2_: Did FamilyR's [ZSibName[2]] Return to Urban

	Freq.	%
1 Yes	200	82.99

	Freq.	%
2 No	27	11.20
997 Don't Know	14	5.81
Total	241	100.00

cc016_w4_3_: Did FamilyR's [ZSibName[3]] Return to Urban

	Freq.	%
1 Yes	147	82.12
2 No	21	11.73
997 Don't Know	11	6.15
Total	179	100.00

cc016_w4_4_: Did FamilyR's [ZSibName[4]] Return to Urban

	Freq.	%
1 Yes	85	78.70
2 No	14	12.96
997 Don't Know	9	8.33
Total	108	100.00

cc016_w4_5_: Did FamilyR's [ZSibName[5]] Return to Urban

	Freq.	%
1 Yes	42	77.78
2 No	8	14.81
997 Don't Know	4	7.41
Total	54	100.00

cc016_w4_6_: Did FamilyR's [ZSibName[6]] Return to Urban

	Freq.	%
1 Yes	15	75.00
2 No	4	20.00
997 Don't Know	1	5.00
Total	20	100.00

cc016_w4_7_: Did FamilyR's [ZSibName[7]] Return to Urban

	Freq.	%
1 Yes	7	77.78
2 No	1	11.11
997 Don't Know	1	11.11
Total	9	100.00

cc016_w4_8_: Did FamilyR's [ZSibName[8]] Return to Urban

	Freq.	%
1 Yes	1	50.00
2 No	1	50.00
Total	2	100.00

cc016_w4_s_1_: Did Spouse's [ZSibName[1]] Return to Urban

	Freq.	%
1 Yes	142	86.59
2 No	16	9.76
997 Don't Know	6	3.66
Total	164	100.00

cc016_w4_s_2_: Did Spouse's [ZSibName[2]] Return to Urban

	Freq.	%
1 Yes	111	84.09
2 No	13	9.85
997 Don't Know	8	6.06
Total	132	100.00

cc016_w4_s_3_: Did Spouse's [ZSibName[3]] Return to Urban

	Freq.	%
1 Yes	91	90.10
2 No	8	7.92
997 Don't Know	2	1.98
Total	101	100.00

cc016_w4_s_4_: Did Spouse's [ZSibName[4]] Return to Urban

	Freq.	%
1 Yes	46	76.67
2 No	9	15.00
997 Don't Know	5	8.33
Total	60	100.00

cc016_w4_s_5_: Did Spouse's [ZSibName[5]] Return to Urban

	Freq.	%
1 Yes	17	85.00
2 No	2	10.00
997 Don't Know	1	5.00
Total	20	100.00

cc016_w4_s_6_: Did Spouse's [ZSibName[6]] Return to Urban

	Freq.	%
1 Yes	9	75.00
2 No	2	16.67
997 Don't Know	1	8.33
Total	12	100.00

cc016_w4_s_7_: Did Spouse's [ZSibName[7]] Return to Urban

	Freq.	%
1 Yes	2	66.67
997 Don't Know	1	33.33
Total	3	100.00

cc016_w4_s_8_: Did Spouse's [ZSibName[8]] Return to Urban

	Freq.	%
1 Yes	1	100.00
Total	1	100.00

cc016_w4_s_9_: Did Spouse's [ZSibName[9]] Return to Urban

	Freq.	%
1 Yes	1	100.00
Total	1	100.00

cc016_w4_s_10_: Did Spouse's [ZSibName[10]] Return to Urban

No Observations

cc016_w4_s_11_: Did Spouse's [ZSibName[11]] Return to Urban

	Freq.	%
1 Yes	1	100.00
Total	1	100.00

cc016_w4_1_1_: Which Year

Mean	SD	Min	Max	Obs
1,964.67	136.07	19.00	1,999.00	208

cc016_w4_1_2_: Which Year

Mean	SD	Min	Max	Obs
1,974.21	14.02	1,900.00	2,018.00	185

cc016_w4_1_3_: Which Year

Mean	SD	Min	Max	Obs
1,975.52	14.49	1,900.00	2,018.00	141

cc016_w4_1_4_: Which Year

Mean	SD	Min	Max	Obs
1,953.89	215.07	19.00	2,018.00	83

cc016_w4_1_5_: Which Year

Mean	SD	Min	Max	Obs
1,977.13	5.26	1,966.00	2,000.00	39

cc016_w4_1_6_: Which Year

Mean	SD	Min	Max	Obs
1,976.62	5.92	1,966.00	1,990.00	13

cc016_w4_1_7_: Which Year

Mean	SD	Min	Max	Obs
1,977.17	5.49	1,969.00	1,986.00	6

cc016_w4_1_8_: Which Year

Mean	SD	Min	Max	Obs
1,973.00	.	1,973.00	1,973.00	1

cc016_w4_1_s_1_: Which Year

Mean	SD	Min	Max	Obs
1,960.89	170.62	19.00	2,018.00	132

cc016_w4_1_s_2_: Which Year

Mean	SD	Min	Max	Obs
1,938.93	269.00	19.00	2,018.00	105

cc016_w4_1_s_3_: Which Year

Mean	SD	Min	Max	Obs
1,977.66	6.60	1,962.00	2,018.00	88

cc016_w4_1_s_4_: Which Year

Mean	SD	Min	Max	Obs
1,936.34	285.81	19.00	2,006.00	47

cc016_w4_1_s_5_: Which Year

Mean	SD	Min	Max	Obs
1,977.82	3.05	1,971.00	1,982.00	17

cc016_w4_1_s_6_: Which Year

Mean	SD	Min	Max	Obs
1,982.56	13.42	1,975.00	2,018.00	9

cc016_w4_1_s_7_: Which Year

Mean	SD	Min	Max	Obs
1,978.00	2.83	1,976.00	1,980.00	2

cc016_w4_1_s_8_: Which Year

No Observations

cc016_w4_1_s_9_: Which Year

No Observations

cc016_w4_1_s_10_: Which Year

No Observations

cc016_w4_1_s_11_: Which Year

No Observations

cc011_w4_1_: [ZSibName[1]]'s Age When Initially Getting Married

	Freq.	%
1 At [CC011_W4_1[1]] Years Old	7,883	70.67
2 Never Get Married	404	3.62
997 Don't Know	2,847	25.52
999 Refuse to Answer	21	0.19
Total	11,155	100.00

cc011_w4_2_: [ZSibName[2]]'s Age When Initially Getting Married

	Freq.	%
1 At [CC011_W4_1[2]] Years Old	7,472	71.79
2 Never Get Married	428	4.11
997 Don't Know	2,486	23.89
999 Refuse to Answer	22	0.21
Total	10,408	100.00

cc011_w4_3_: [ZSibName[3]]'s Age When Initially Getting Married

	Freq.	%
1 At [CC011_W4_1[3]] Years Old	6,356	72.76
2 Never Get Married	357	4.09
997 Don't Know	2,011	23.02

999 Refuse to Answer	12	0.14
Total	8,736	100.00

cc011_w4_4_: [ZSibName[4]]'s Age When Initially Getting Married

	Freq.	%
1 At [CC011_W4_1[4]] Years Old	4,641	72.50
2 Never Get Married	283	4.42
997 Don't Know	1,464	22.87
999 Refuse to Answer	13	0.20
Total	6,401	100.00

cc011_w4_5_: [ZSibName[5]]'s Age When Initially Getting Married

	Freq.	%
1 At [CC011_W4_1[5]] Years Old	2,858	70.95
2 Never Get Married	205	5.09
997 Don't Know	953	23.66
999 Refuse to Answer	12	0.30
Total	4,028	100.00

cc011_w4_6_: [ZSibName[6]]'s Age When Initially Getting Married

	Freq.	%
1 At [CC011_W4_1[6]] Years Old	1,434	71.06
2 Never Get Married	98	4.86
997 Don't Know	481	23.84
999 Refuse to Answer	5	0.25
Total	2,018	100.00

cc011_w4_7_: [ZSibName[7]]'s Age When Initially Getting Married

	Freq.	%
1 At [CC011_W4_1[7]] Years Old	610	70.77
2 Never Get Married	50	5.80
997 Don't Know	199	23.09
999 Refuse to Answer	3	0.35
Total	862	100.00

cc011_w4_8_: [ZSibName[8]]'s Age When Initially Getting Married

	Freq.	%
1 At [CC011_W4_1[8]] Years Old	214	67.08
2 Never Get Married	23	7.21
997 Don't Know	79	24.76
999 Refuse to Answer	3	0.94
Total	319	100.00

cc011_w4_9_: [ZSibName[9]]'s Age When Initially Getting Married

	Freq.	%
1 At [CC011_W4_1[9]] Years Old	87	63.50
2 Never Get Married	13	9.49
997 Don't Know	34	24.82
999 Refuse to Answer	3	2.19
Total	137	100.00

cc011_w4_10_: [ZSibName[10]]'s Age When Initially Getting Married

	Freq.	%
1 At [CC011_W4_1[10]] Years Old	25	58.14
2 Never Get Married	4	9.30
997 Don't Know	14	32.56
Total	43	100.00

cc011_w4_11_: [ZSibName[11]]'s Age When Initially Getting Married

	Freq.	%
1 At [CC011_W4_1[11]] Years Old	12	44.44
2 Never Get Married	7	25.93
997 Don't Know	8	29.63
Total	27	100.00

cc011_w4_12_: [ZSibName[12]]'s Age When Initially Getting Married

	Freq.	%
1 At [CC011_W4_1[12]] Years Old	4	50.00
2 Never Get Married	3	37.50
997 Don't Know	1	12.50
Total	8	100.00

cc011_w4_13_: [ZSibName[13]]'s Age When Initially Getting Married

	Freq.	%
1 At [CC011_W4_1[13]] Years Old	1	33.33
997 Don't Know	2	66.67
Total	3	100.00

cc011_w4_14_: [ZSibName[14]]'s Age When Initially Getting Married

	Freq.	%
1 At [CC011_W4_1[14]] Years Old	1	100.00
Total	1	100.00

cc011_w4_s_1_: [ZSibName[1]]'s Age When Initially Getting Married

	Freq.	%
1 At [CC011_W4_1[1]] Years Old	5,409	64.42
2 Never Get Married	311	3.70

	Freq.	%
997 Don't Know	2,660	31.68
999 Refuse to Answer	17	0.20
Total	8,397	100.00

cc011_w4_s_2_: [ZSibName[2]]'s Age When Initially Getting Married

	Freq.	%
1 At [CC011_W4_1[2]] Years Old	5,295	67.63
2 Never Get Married	253	3.23
997 Don't Know	2,262	28.89
999 Refuse to Answer	19	0.24
Total	7,829	100.00

cc011_w4_s_3_: [ZSibName[3]]'s Age When Initially Getting Married

	Freq.	%
1 At [CC011_W4_1[3]] Years Old	4,609	69.70
2 Never Get Married	252	3.81
997 Don't Know	1,741	26.33
999 Refuse to Answer	11	0.17
Total	6,613	100.00

cc011_w4_s_4_: [ZSibName[4]]'s Age When Initially Getting Married

	Freq.	%
1 At [CC011_W4_1[4]] Years Old	3,337	70.06
2 Never Get Married	202	4.24
997 Don't Know	1,211	25.43
999 Refuse to Answer	13	0.27
Total	4,763	100.00

cc011_w4_s_5_: [ZSibName[5]]'s Age When Initially Getting Married

	Freq.	%
1 At [CC011_W4_1[5]] Years Old	2,048	69.42
2 Never Get Married	136	4.61
997 Don't Know	751	25.46
999 Refuse to Answer	15	0.51
Total	2,950	100.00

cc011_w4_s_6_: [ZSibName[6]]'s Age When Initially Getting Married

	Freq.	%
1 At [CC011_W4_1[6]] Years Old	1,035	68.95
2 Never Get Married	72	4.80
997 Don't Know	388	25.85
999 Refuse to Answer	6	0.40
Total	1,501	100.00

cc011_w4_s_7_: [ZSibName[7]]'s Age When Initially Getting Married

	Freq.	%
1 At [CC011_W4_1[7]] Years Old	464	67.94
2 Never Get Married	38	5.56
997 Don't Know	178	26.06
999 Refuse to Answer	3	0.44
Total	683	100.00

cc011_w4_s_8_: [ZSibName[8]]'s Age When Initially Getting Married

	Freq.	%
1 At [CC011_W4_1[8]] Years Old	176	65.67
2 Never Get Married	13	4.85
997 Don't Know	75	27.99
999 Refuse to Answer	4	1.49
Total	268	100.00

cc011_w4_s_9_: [ZSibName[9]]'s Age When Initially Getting Married

	Freq.	%
1 At [CC011_W4_1[9]] Years Old	51	56.04
2 Never Get Married	7	7.69
997 Don't Know	30	32.97
999 Refuse to Answer	3	3.30
Total	91	100.00

cc011_w4_s_10_: [ZSibName[10]]'s Age When Initially Getting Married

	Freq.	%
1 At [CC011_W4_1[10]] Years Old	12	42.86
2 Never Get Married	3	10.71
997 Don't Know	11	39.29
999 Refuse to Answer	2	7.14
Total	28	100.00

cc011_w4_s_11_: [ZSibName[11]]'s Age When Initially Getting Married

	Freq.	%
1 At [CC011_W4_1[11]] Years Old	4	33.33
2 Never Get Married	1	8.33
997 Don't Know	6	50.00
999 Refuse to Answer	1	8.33
Total	12	100.00

cc011_w4_s_12_: [ZSibName[12]]'s Age When Initially Getting Married

	Freq.	%
1 At [CC011_W4_1[12]] Years Old	2	28.57
2 Never Get Married	1	14.29
997 Don't Know	3	42.86
999 Refuse to Answer	1	14.29

	Freq.	%
Total	7	100.00

cc011_w4_s_13_: [ZSibName[13]]'s Age When Initially Getting Married

	Freq.	%
1 At [CC011_W4_1[13]] Years Old	1	50.00
999 Refuse to Answer	1	50.00
Total	2	100.00

cc011_w4_s_14_: [ZSibName[14]]'s Age When Initially Getting Married

	Freq.	%
2 Never Get Married	1	50.00
999 Refuse to Answer	1	50.00
Total	2	100.00

cc011_w4_s_15_: [ZSibName[15]]'s Age When Initially Getting Married

	Freq.	%
2 Never Get Married	1	50.00
999 Refuse to Answer	1	50.00
Total	2	100.00

cc011_w4_a_1_: [ZSibName[1]]'s Age When Initially Getting Married: Years Old

Mean	SD	Min	Max	Obs
23.37	31.53	0.00	2,011.00	7,853

cc011_w4_a_2_: [ZSibName[2]]'s Age When Initially Getting Married: Years Old

Mean	SD	Min	Max	Obs
23.42	22.75	0.00	1,958.00	7,452

cc011_w4_a_3_: [ZSibName[3]]'s Age When Initially Getting Married: Years Old

Mean	SD	Min	Max	Obs
23.17	3.63	0.00	75.00	6,338

cc011_w4_a_4_: [ZSibName[4]]'s Age When Initially Getting Married: Years Old

Mean	SD	Min	Max	Obs
23.23	3.65	0.00	56.00	4,630

cc011_w4_a_5_: [ZSibName[5]]'s Age When Initially Getting Married: Years Old

Mean	SD	Min	Max	Obs
23.12	3.56	0.00	70.00	2,850

cc011_w4_a_6_: [ZSibName[6]]'s Age When Initially Getting Married: Years Old

Mean	SD	Min	Max	Obs
23.00	3.48	0.00	45.00	1,434

cc011_w4_a_7_: [ZSibName[7]]'s Age When Initially Getting Married: Years Old

Mean	SD	Min	Max	Obs
23.01	3.35	0.00	46.00	606

cc011_w4_a_8_: [ZSibName[8]]'s Age When Initially Getting Married: Years Old

Mean	SD	Min	Max	Obs
23.16	3.60	0.00	41.00	212

cc011_w4_a_9_: [ZSibName[9]]'s Age When Initially Getting Married: Years Old

Mean	SD	Min	Max	Obs
23.10	4.90	0.00	42.00	86

cc011_w4_a_10_: [ZSibName[10]]'s Age When Initially Getting Married: Years Old

Mean	SD	Min	Max	Obs
22.72	2.56	18.00	29.00	25

cc011_w4_a_11_: [ZSibName[11]]'s Age When Initially Getting Married: Years Old

Mean	SD	Min	Max	Obs
23.50	4.36	16.00	30.00	12

cc011_w4_a_12_: [ZSibName[12]]'s Age When Initially Getting Married: Years Old

Mean	SD	Min	Max	Obs
22.75	3.77	18.00	27.00	4

cc011_w4_a_13_: [ZSibName[13]]'s Age When Initially Getting Married: Years Old

Mean	SD	Min	Max	Obs
20.00	.	20.00	20.00	1

cc011_w4_a_14_: [ZSibName[14]]'s Age When Initially Getting Married: Years Old

Mean	SD	Min	Max	Obs
20.00	.	20.00	20.00	1

cc011_w4_a_s_1_: [ZSibName[1]]'s Age When Initially Getting Married: Years Old

Mean	SD	Min	Max	Obs
23.24	26.68	0.00	1,954.00	5,366

cc011_w4_a_s_2_: [ZSibName[2]]'s Age When Initially Getting Married: Years Old

Mean	SD	Min	Max	Obs
23.55	27.11	0.00	1,970.00	5,257

cc011_w4_a_s_3_: [ZSibName[3]]'s Age When Initially Getting Married: Years Old

Mean	SD	Min	Max	Obs
23.16	3.58	0.00	86.00	4,587

cc011_w4_a_s_4_: [ZSibName[4]]'s Age When Initially Getting Married: Years Old

Mean	SD	Min	Max	Obs
23.14	3.65	0.00	89.00	3,316

cc011_w4_a_s_5_: [ZSibName[5]]'s Age When Initially Getting Married: Years Old

Mean	SD	Min	Max	Obs
23.02	3.66	0.00	85.00	2,038

cc011_w4_a_s_6_: [ZSibName[6]]'s Age When Initially Getting Married: Years Old

Mean	SD	Min	Max	Obs
22.93	3.62	0.00	71.00	1,027

cc011_w4_a_s_7_: [ZSibName[7]]'s Age When Initially Getting Married: Years Old

Mean	SD	Min	Max	Obs
23.07	5.43	15.00	119.00	460

cc011_w4_a_s_8_: [ZSibName[8]]'s Age When Initially Getting Married: Years Old

Mean	SD	Min	Max	Obs
23.16	3.35	17.00	40.00	173

cc011_w4_a_s_9_: [ZSibName[9]]'s Age When Initially Getting Married: Years Old

Mean	SD	Min	Max	Obs
23.67	4.27	17.00	42.00	49

cc011_w4_a_s_10_: [ZSibName[10]]'s Age When Initially Getting Married: Years Old

Mean	SD	Min	Max	Obs
22.62	3.23	17.00	27.00	13

cc011_w4_a_s_11_: [ZSibName[11]]'s Age When Initially Getting Married: Years Old

Mean	SD	Min	Max	Obs
22.75	3.20	18.00	25.00	4

cc011_w4_a_s_12_: [ZSibName[12]]'s Age When Initially Getting Married: Years Old

Mean	SD	Min	Max	Obs
21.50	4.95	18.00	25.00	2

cc011_w4_a_s_13_: [ZSibName[13]]'s Age When Initially Getting Married: Years Old

Mean	SD	Min	Max	Obs
18.00	.	18.00	18.00	1

cc011_w3_1_: [ZSibName[1]]'s Marital Status

	Freq.	%
1 Married with Spouse Present	6,433	78.92
2 Married but not Living with Spouse Temporarily	113	1.39
3 Separated	21	0.26
4 Divorced	152	1.86
5 Widowed	1,213	14.88
6 Never Married	123	1.51
997 Don't Know	79	0.97
999 Refuse to Answer	17	0.21
Total	8,151	100.00

cc011_w3_2_: [ZSibName[2]]'s Marital Status

	Freq.	%
1 Married with Spouse Present	6,831	82.96
2 Married but not Living with Spouse Temporarily	111	1.35
3 Separated	23	0.28
4 Divorced	157	1.91
5 Widowed	890	10.81
6 Never Married	153	1.86
997 Don't Know	57	0.69
999 Refuse to Answer	12	0.15
Total	8,234	100.00

cc011_w3_3_: [ZSibName[3]]'s Marital Status

	Freq.	%
1 Married with Spouse Present	6,107	83.99
2 Married but not Living with Spouse Temporarily	115	1.58
3 Separated	23	0.32
4 Divorced	165	2.27
5 Widowed	661	9.09
6 Never Married	131	1.80
997 Don't Know	61	0.84
999 Refuse to Answer	8	0.11
Total	7,271	100.00

cc011_w3_4_: [ZSibName[4]]'s Marital Status

	Freq.	%
1 Married with Spouse Present	4,709	85.93
2 Married but not Living with Spouse Temporarily	93	1.70
3 Separated	14	0.26
4 Divorced	113	2.06
5 Widowed	383	6.99
6 Never Married	110	2.01
997 Don't Know	46	0.84
999 Refuse to Answer	12	0.22
Total	5,480	100.00

cc011_w3_5_: [ZSibName[5]]'s Marital Status

	Freq.	%
1 Married with Spouse Present	3,039	86.75
2 Married but not Living with Spouse Temporarily	51	1.46
3 Separated	7	0.20
4 Divorced	92	2.63
5 Widowed	206	5.88
6 Never Married	71	2.03
997 Don't Know	32	0.91
999 Refuse to Answer	5	0.14
Total	3,503	100.00

cc011_w3_6_: [ZSibName[6]]'s Marital Status

	Freq.	%
1 Married with Spouse Present	1,530	88.13
2 Married but not Living with Spouse Temporarily	26	1.50
3 Separated	7	0.40
4 Divorced	41	2.36
5 Widowed	78	4.49
6 Never Married	26	1.50
997 Don't Know	26	1.50
999 Refuse to Answer	2	0.12
Total	1,736	100.00

cc011_w3_7_: [ZSibName[7]]'s Marital Status

	Freq.	%
1 Married with Spouse Present	641	86.04
2 Married but not Living with Spouse Temporarily	7	0.94
3 Separated	2	0.27
4 Divorced	20	2.68
5 Widowed	42	5.64
6 Never Married	17	2.28
997 Don't Know	14	1.88
999 Refuse to Answer	2	0.27
Total	745	100.00

cc011_w3_8_: [ZSibName[8]]'s Marital Status

	Freq.	%
1 Married with Spouse Present	228	84.44
2 Married but not Living with Spouse Temporarily	5	1.85
4 Divorced	5	1.85
5 Widowed	16	5.93
6 Never Married	4	1.48
997 Don't Know	9	3.33
999 Refuse to Answer	3	1.11
Total	270	100.00

cc011_w3_9_: [ZSibName[9]]'s Marital Status

	Freq.	%
1 Married with Spouse Present	98	84.48
2 Married but not Living with Spouse Temporarily	2	1.72
4 Divorced	1	0.86
5 Widowed	7	6.03
6 Never Married	2	1.72
997 Don't Know	3	2.59
999 Refuse to Answer	3	2.59
Total	116	100.00

cc011_w3_10_: [ZSibName[10]]'s Marital Status

	Freq.	%
1 Married with Spouse Present	33	89.19
4 Divorced	1	2.70
5 Widowed	2	5.41
997 Don't Know	1	2.70
Total	37	100.00

cc011_w3_11_: [ZSibName[11]]'s Marital Status

	Freq.	%
1 Married with Spouse Present	18	100.00
Total	18	100.00

cc011_w3_12_: [ZSibName[12]]'s Marital Status

	Freq.	%
1 Married with Spouse Present	4	80.00
6 Never Married	1	20.00
Total	5	100.00

cc011_w3_13_: [ZSibName[13]]'s Marital Status

	Freq.	%
1 Married with Spouse Present	2	100.00
Total	2	100.00

cc011_w3_14_: [ZSibName[14]]'s Marital Status

	Freq.	%
1 Married with Spouse Present	1	100.00
Total	1	100.00

cc011_w3_s_1_: [ZSibName[1]]'s Marital Status

	Freq.	%
1 Married with Spouse Present	5,364	81.89
2 Married but not Living with Spouse Temporarily	73	1.11
3 Separated	10	0.15
4 Divorced	117	1.79
5 Widowed	818	12.49
6 Never Married	102	1.56
997 Don't Know	57	0.87
999 Refuse to Answer	9	0.14
Total	6,550	100.00

cc011_w3_s_2_: [ZSibName[2]]'s Marital Status

	Freq.	%
1 Married with Spouse Present	5,592	85.02
2 Married but not Living with Spouse Temporarily	87	1.32
3 Separated	15	0.23
4 Divorced	130	1.98
5 Widowed	584	8.88
6 Never Married	96	1.46
997 Don't Know	63	0.96
999 Refuse to Answer	10	0.15
Total	6,577	100.00

cc011_w3_s_3_: [ZSibName[3]]'s Marital Status

	Freq.	%
1 Married with Spouse Present	4,949	86.25
2 Married but not Living with Spouse Temporarily	83	1.45
3 Separated	11	0.19
4 Divorced	114	1.99

	Freq.	%
5 Widowed	410	7.15
6 Never Married	107	1.86
997 Don't Know	55	0.96
999 Refuse to Answer	9	0.16
Total	5,738	100.00

cc011_w3_s_4_: [ZSibName[4]]'s Marital Status

	Freq.	%
1 Married with Spouse Present	3,679	87.26
2 Married but not Living with Spouse Temporarily	53	1.26
3 Separated	10	0.24
4 Divorced	95	2.25
5 Widowed	244	5.79
6 Never Married	82	1.94
997 Don't Know	43	1.02
999 Refuse to Answer	10	0.24
Total	4,216	100.00

cc011_w3_s_5_: [ZSibName[5]]'s Marital Status

	Freq.	%
1 Married with Spouse Present	2,326	87.84
2 Married but not Living with Spouse Temporarily	39	1.47
3 Separated	4	0.15
4 Divorced	55	2.08
5 Widowed	132	4.98
6 Never Married	54	2.04
997 Don't Know	29	1.10
999 Refuse to Answer	9	0.34
Total	2,648	100.00

cc011_w3_s_6_: [ZSibName[6]]'s Marital Status

	Freq.	%
1 Married with Spouse Present	1,145	87.07
2 Married but not Living with Spouse Temporarily	22	1.67
3 Separated	4	0.30
4 Divorced	27	2.05
5 Widowed	71	5.40
6 Never Married	33	2.51
997 Don't Know	10	0.76
999 Refuse to Answer	3	0.23
Total	1,315	100.00

cc011_w3_s_7_: [ZSibName[7]]'s Marital Status

	Freq.	%
1 Married with Spouse Present	538	87.62
2 Married but not Living with Spouse Temporarily	7	1.14
3 Separated	2	0.33
4 Divorced	14	2.28
5 Widowed	28	4.56

6 Never Married	12	1.95
997 Don't Know	10	1.63
999 Refuse to Answer	3	0.49
Total	614	100.00

cc011_w3_s_8_: [ZSibName[8]]'s Marital Status

	Freq.	%
1 Married with Spouse Present	208	85.95
2 Married but not Living with Spouse Temporarily	3	1.24
3 Separated	1	0.41
4 Divorced	9	3.72
5 Widowed	10	4.13
6 Never Married	4	1.65
997 Don't Know	4	1.65
999 Refuse to Answer	3	1.24
Total	242	100.00

cc011_w3_s_9_: [ZSibName[9]]'s Marital Status

	Freq.	%
1 Married with Spouse Present	56	82.35
2 Married but not Living with Spouse Temporarily	1	1.47
3 Separated	1	1.47
4 Divorced	2	2.94
5 Widowed	3	4.41
6 Never Married	1	1.47
997 Don't Know	2	2.94
999 Refuse to Answer	2	2.94
Total	68	100.00

cc011_w3_s_10_: [ZSibName[10]]'s Marital Status

	Freq.	%
1 Married with Spouse Present	15	75.00
5 Widowed	2	10.00
997 Don't Know	1	5.00
999 Refuse to Answer	2	10.00
Total	20	100.00

cc011_w3_s_11_: [ZSibName[11]]'s Marital Status

	Freq.	%
1 Married with Spouse Present	8	72.73
6 Never Married	1	9.09
997 Don't Know	1	9.09
999 Refuse to Answer	1	9.09
Total	11	100.00

cc011_w3_s_12_: [ZSibName[12]]'s Marital Status

	Freq.	%

	Freq.	%
1 Married with Spouse Present	3	50.00
6 Never Married	1	16.67
997 Don't Know	1	16.67
999 Refuse to Answer	1	16.67
Total	6	100.00

cc011_w3_s_13_: [ZSibName[13]]'s Marital Status

	Freq.	%
1 Married with Spouse Present	1	50.00
999 Refuse to Answer	1	50.00
Total	2	100.00

cc011_w3_s_14_: [ZSibName[14]]'s Marital Status

	Freq.	%
999 Refuse to Answer	1	100.00
Total	1	100.00

cc011_w3_s_15_: [ZSibName[15]]'s Marital Status

	Freq.	%
1 Married with Spouse Present	1	50.00
999 Refuse to Answer	1	50.00
Total	2	100.00

cc011_w4_1_1_: Does [ZSibName[1]] Have Cohabiting Partner

	Freq.	%
1 Yes	103	6.42
2 No	1,408	87.73
997 Don't Know	80	4.98
999 Refuse to Answer	14	0.87
Total	1,605	100.00

cc011_w4_1_2_: Does [ZSibName[2]] Have Cohabiting Partner

	Freq.	%
1 Yes	72	5.57
2 No	1,138	88.08
997 Don't Know	70	5.42
999 Refuse to Answer	12	0.93
Total	1,292	100.00

cc011_w4_1_3_: Does [ZSibName[3]] Have Cohabiting Partner

	Freq.	%
1 Yes	87	8.29
2 No	891	84.94
997 Don't Know	62	5.91

	Freq.	%
999 Refuse to Answer	9	0.86
Total	1,049	100.00

cc011_w4_1_4_: Does [ZSibName[4]] Have Cohabiting Partner

	Freq.	%
1 Yes	61	9.00
2 No	552	81.42
997 Don't Know	55	8.11
999 Refuse to Answer	10	1.47
Total	678	100.00

cc011_w4_1_5_: Does [ZSibName[5]] Have Cohabiting Partner

	Freq.	%
1 Yes	30	7.26
2 No	342	82.81
997 Don't Know	37	8.96
999 Refuse to Answer	4	0.97
Total	413	100.00

cc011_w4_1_6_: Does [ZSibName[6]] Have Cohabiting Partner

	Freq.	%
1 Yes	4	2.22
2 No	144	80.00
997 Don't Know	30	16.67
999 Refuse to Answer	2	1.11
Total	180	100.00

cc011_w4_1_7_: Does [ZSibName[7]] Have Cohabiting Partner

	Freq.	%
1 Yes	7	7.22
2 No	75	77.32
997 Don't Know	13	13.40
999 Refuse to Answer	2	2.06
Total	97	100.00

cc011_w4_1_8_: Does [ZSibName[8]] Have Cohabiting Partner

	Freq.	%
1 Yes	2	5.41
2 No	24	64.86
997 Don't Know	8	21.62
999 Refuse to Answer	3	8.11
Total	37	100.00

cc011_w4_1_9_: Does [ZSibName[9]] Have Cohabiting Partner

	Freq.	%
2 No	10	62.50
997 Don't Know	3	18.75
999 Refuse to Answer	3	18.75
Total	16	100.00

cc011_w4_1_10_: Does [ZSibName[10]] Have Cohabiting Partner

	Freq.	%
2 No	3	75.00
997 Don't Know	1	25.00
Total	4	100.00

cc011_w4_1_11_: Does [ZSibName[11]] Have Cohabiting Partner

No Observations

cc011_w4_1_12_: Does [ZSibName[12]] Have Cohabiting Partner

	Freq.	%
2 No	1	100.00
Total	1	100.00

cc011_w4_1_s_1_: Does [ZSibName[1]] Have Cohabiting Partner

	Freq.	%
1 Yes	63	5.66
2 No	976	87.69
997 Don't Know	64	5.75
999 Refuse to Answer	10	0.90
Total	1,113	100.00

cc011_w4_1_s_2_: Does [ZSibName[2]] Have Cohabiting Partner

	Freq.	%
1 Yes	55	6.12
2 No	773	86.08
997 Don't Know	60	6.68
999 Refuse to Answer	10	1.11
Total	898	100.00

cc011_w4_1_s_3_: Does [ZSibName[3]] Have Cohabiting Partner

	Freq.	%
1 Yes	57	8.07
2 No	572	81.02
997 Don't Know	67	9.49
999 Refuse to Answer	10	1.42
Total	706	100.00

cc011_w4_1_s_4_: Does [ZSibName[4]] Have Cohabiting Partner

	Freq.	%
1 Yes	44	9.09
2 No	377	77.89
997 Don't Know	52	10.74
999 Refuse to Answer	11	2.27
Total	484	100.00

cc011_w4_1_s_5_: Does [ZSibName[5]] Have Cohabiting Partner

	Freq.	%
1 Yes	22	7.77
2 No	219	77.39
997 Don't Know	33	11.66
999 Refuse to Answer	9	3.18
Total	283	100.00

cc011_w4_1_s_6_: Does [ZSibName[6]] Have Cohabiting Partner

	Freq.	%
1 Yes	18	12.16
2 No	114	77.03
997 Don't Know	13	8.78
999 Refuse to Answer	3	2.03
Total	148	100.00

cc011_w4_1_s_7_: Does [ZSibName[7]] Have Cohabiting Partner

	Freq.	%
1 Yes	2	2.90
2 No	51	73.91
997 Don't Know	13	18.84
999 Refuse to Answer	3	4.35
Total	69	100.00

cc011_w4_1_s_8_: Does [ZSibName[8]] Have Cohabiting Partner

	Freq.	%
1 Yes	2	6.45
2 No	20	64.52
997 Don't Know	5	16.13
999 Refuse to Answer	4	12.90
Total	31	100.00

cc011_w4_1_s_9_: Does [ZSibName[9]] Have Cohabiting Partner

	Freq.	%
1 Yes	1	9.09
2 No	5	45.45
997 Don't Know	3	27.27

	Freq.	%
999 Refuse to Answer	2	18.18
Total	11	100.00

cc011_w4_1_s_10_: Does [ZSibName[10]] Have Cohabiting Partner

	Freq.	%
2 No	2	40.00
997 Don't Know	1	20.00
999 Refuse to Answer	2	40.00
Total	5	100.00

cc011_w4_1_s_11_: Does [ZSibName[11]] Have Cohabiting Partner

	Freq.	%
2 No	1	33.33
997 Don't Know	1	33.33
999 Refuse to Answer	1	33.33
Total	3	100.00

cc011_w4_1_s_12_: Does [ZSibName[12]] Have Cohabiting Partner

	Freq.	%
2 No	1	33.33
997 Don't Know	1	33.33
999 Refuse to Answer	1	33.33
Total	3	100.00

cc011_w4_1_s_13_: Does [ZSibName[13]] Have Cohabiting Partner

	Freq.	%
999 Refuse to Answer	1	100.00
Total	1	100.00

cc011_w4_1_s_14_: Does [ZSibName[14]] Have Cohabiting Partner

	Freq.	%
999 Refuse to Answer	1	100.00
Total	1	100.00

cc011_w4_1_s_15_: Does [ZSibName[15]] Have Cohabiting Partner

	Freq.	%
999 Refuse to Answer	1	100.00
Total	1	100.00

cc012_w3_1_: [ZSibName[1]]'s Health Status Now

	Freq.	%
1 Very Good	1,465	17.97

2 Good	1,504	18.45
3 Fair	3,418	41.93
4 Poor	1,307	16.03
5 Very Poor	293	3.59
997 Don't Know	147	1.80
999 Refuse to Answer	17	0.21
Total	8,151	100.00

cc012_w3_2_: [ZSibName[2]]'s Health Status Now

	Freq.	%
1 Very Good	1,612	19.58
2 Good	1,770	21.50
3 Fair	3,440	41.78
4 Poor	1,057	12.84
5 Very Poor	234	2.84
997 Don't Know	103	1.25
999 Refuse to Answer	18	0.22
Total	8,234	100.00

cc012_w3_3_: [ZSibName[3]]'s Health Status Now

	Freq.	%
1 Very Good	1,605	22.07
2 Good	1,708	23.49
3 Fair	2,882	39.64
4 Poor	824	11.33
5 Very Poor	152	2.09
997 Don't Know	88	1.21
999 Refuse to Answer	12	0.17
Total	7,271	100.00

cc012_w3_4_: [ZSibName[4]]'s Health Status Now

	Freq.	%
1 Very Good	1,266	23.10
2 Good	1,342	24.49
3 Fair	2,163	39.47
4 Poor	524	9.56
5 Very Poor	109	1.99
997 Don't Know	64	1.17
999 Refuse to Answer	12	0.22
Total	5,480	100.00

cc012_w3_5_: [ZSibName[5]]'s Health Status Now

	Freq.	%
1 Very Good	860	24.55
2 Good	884	25.24
3 Fair	1,358	38.77
4 Poor	279	7.96
5 Very Poor	62	1.77
997 Don't Know	55	1.57

999 Refuse to Answer	5	0.14
Total	3,503	100.00

cc012_w3_6_: [ZSibName[6]]'s Health Status Now

	Freq.	%
1 Very Good	424	24.42
2 Good	430	24.77
3 Fair	683	39.34
4 Poor	134	7.72
5 Very Poor	29	1.67
997 Don't Know	34	1.96
999 Refuse to Answer	2	0.12
Total	1,736	100.00

cc012_w3_7_: [ZSibName[7]]'s Health Status Now

	Freq.	%
1 Very Good	202	27.11
2 Good	173	23.22
3 Fair	272	36.51
4 Poor	62	8.32
5 Very Poor	11	1.48
997 Don't Know	21	2.82
999 Refuse to Answer	4	0.54
Total	745	100.00

cc012_w3_8_: [ZSibName[8]]'s Health Status Now

	Freq.	%
1 Very Good	71	26.30
2 Good	67	24.81
3 Fair	93	34.44
4 Poor	17	6.30
5 Very Poor	5	1.85
997 Don't Know	14	5.19
999 Refuse to Answer	3	1.11
Total	270	100.00

cc012_w3_9_: [ZSibName[9]]'s Health Status Now

	Freq.	%
1 Very Good	27	23.28
2 Good	29	25.00
3 Fair	40	34.48
4 Poor	11	9.48
5 Very Poor	2	1.72
997 Don't Know	4	3.45
999 Refuse to Answer	3	2.59
Total	116	100.00

cc012_w3_10_: [ZSibName[10]]'s Health Status Now

	Freq.	%
1 Very Good	8	21.62
2 Good	9	24.32
3 Fair	13	35.14
4 Poor	4	10.81
5 Very Poor	2	5.41
997 Don't Know	1	2.70
Total	37	100.00

cc012_w3_11_: [ZSibName[11]]'s Health Status Now

	Freq.	%
1 Very Good	6	33.33
2 Good	4	22.22
3 Fair	8	44.44
Total	18	100.00

cc012_w3_12_: [ZSibName[12]]'s Health Status Now

	Freq.	%
1 Very Good	1	20.00
2 Good	2	40.00
3 Fair	1	20.00
5 Very Poor	1	20.00
Total	5	100.00

cc012_w3_13_: [ZSibName[13]]'s Health Status Now

	Freq.	%
2 Good	1	50.00
3 Fair	1	50.00
Total	2	100.00

cc012_w3_14_: [ZSibName[14]]'s Health Status Now

	Freq.	%
3 Fair	1	100.00
Total	1	100.00

cc012_w3_s_1_: [ZSibName[1]]'s Health Status Now

	Freq.	%
1 Very Good	1,400	21.37
2 Good	1,387	21.18
3 Fair	2,685	40.99
4 Poor	779	11.89
5 Very Poor	179	2.73
997 Don't Know	109	1.66
999 Refuse to Answer	11	0.17
Total	6,550	100.00

cc012_w3_s_2_: [ZSibName[2]]'s Health Status Now

	Freq.	%
1 Very Good	1,512	22.99
2 Good	1,526	23.20
3 Fair	2,671	40.61
4 Poor	623	9.47
5 Very Poor	156	2.37
997 Don't Know	74	1.13
999 Refuse to Answer	15	0.23
Total	6,577	100.00

cc012_w3_s_3_: [ZSibName[3]]'s Health Status Now

	Freq.	%
1 Very Good	1,445	25.18
2 Good	1,417	24.70
3 Fair	2,205	38.43
4 Poor	465	8.10
5 Very Poor	109	1.90
997 Don't Know	85	1.48
999 Refuse to Answer	12	0.21
Total	5,738	100.00

cc012_w3_s_4_: [ZSibName[4]]'s Health Status Now

	Freq.	%
1 Very Good	1,081	25.65
2 Good	1,098	26.05
3 Fair	1,590	37.72
4 Poor	311	7.38
5 Very Poor	68	1.61
997 Don't Know	55	1.30
999 Refuse to Answer	12	0.28
Total	4,215	100.00

cc012_w3_s_5_: [ZSibName[5]]'s Health Status Now

	Freq.	%
1 Very Good	738	27.87
2 Good	680	25.68
3 Fair	973	36.74
4 Poor	163	6.16
5 Very Poor	42	1.59
997 Don't Know	42	1.59
999 Refuse to Answer	10	0.38
Total	2,648	100.00

cc012_w3_s_6_: [ZSibName[6]]'s Health Status Now

	Freq.	%
1 Very Good	385	29.28
2 Good	357	27.15

3 Fair	473	35.97
4 Poor	67	5.10
5 Very Poor	17	1.29
997 Don't Know	12	0.91
999 Refuse to Answer	4	0.30
Total	1,315	100.00

cc012_w3_s_7_: [ZSibName[7]]'s Health Status Now

	Freq.	%
1 Very Good	183	29.80
2 Good	166	27.04
3 Fair	216	35.18
4 Poor	28	4.56
5 Very Poor	9	1.47
997 Don't Know	9	1.47
999 Refuse to Answer	3	0.49
Total	614	100.00

cc012_w3_s_8_: [ZSibName[8]]'s Health Status Now

	Freq.	%
1 Very Good	87	35.95
2 Good	63	26.03
3 Fair	68	28.10
4 Poor	13	5.37
5 Very Poor	4	1.65
997 Don't Know	4	1.65
999 Refuse to Answer	3	1.24
Total	242	100.00

cc012_w3_s_9_: [ZSibName[9]]'s Health Status Now

	Freq.	%
1 Very Good	22	32.35
2 Good	23	33.82
3 Fair	14	20.59
4 Poor	2	2.94
5 Very Poor	2	2.94
997 Don't Know	3	4.41
999 Refuse to Answer	2	2.94
Total	68	100.00

cc012_w3_s_10_: [ZSibName[10]]'s Health Status Now

	Freq.	%
1 Very Good	10	50.00
2 Good	1	5.00
3 Fair	5	25.00
4 Poor	1	5.00
997 Don't Know	1	5.00
999 Refuse to Answer	2	10.00
Total	20	100.00

cc012_w3_s_11_: [ZSibName[11]]'s Health Status Now

	Freq.	%
1 Very Good	4	36.36
3 Fair	4	36.36
997 Don't Know	2	18.18
999 Refuse to Answer	1	9.09
Total	11	100.00

cc012_w3_s_12_: [ZSibName[12]]'s Health Status Now

	Freq.	%
1 Very Good	2	33.33
3 Fair	1	16.67
4 Poor	1	16.67
997 Don't Know	1	16.67
999 Refuse to Answer	1	16.67
Total	6	100.00

cc012_w3_s_13_: [ZSibName[13]]'s Health Status Now

	Freq.	%
3 Fair	1	50.00
999 Refuse to Answer	1	50.00
Total	2	100.00

cc012_w3_s_14_: [ZSibName[14]]'s Health Status Now

	Freq.	%
999 Refuse to Answer	1	100.00
Total	1	100.00

cc012_w3_s_15_: [ZSibName[15]]'s Health Status Now

	Freq.	%
3 Fair	1	50.00
999 Refuse to Answer	1	50.00
Total	2	100.00

cc006_w3_2_1_: [ZSibName[1]] Have Self-care Ability

	Freq.	%
1 Yes	7,612	93.39
2 No	424	5.20
997 Don't Know	98	1.20
999 Refuse to Answer	17	0.21
Total	8,151	100.00

cc006_w3_2_2_: [ZSibName[2]] Have Self-care Ability

	Freq.	%
1 Yes	7,826	95.04
2 No	321	3.90
997 Don't Know	76	0.92
999 Refuse to Answer	11	0.13
Total	8,234	100.00

cc006_w3_2_3_: [ZSibName[3]] Have Self-care Ability

	Freq.	%
1 Yes	6,969	95.85
2 No	218	3.00
997 Don't Know	74	1.02
999 Refuse to Answer	10	0.14
Total	7,271	100.00

cc006_w3_2_4_: [ZSibName[4]] Have Self-care Ability

	Freq.	%
1 Yes	5,228	95.40
2 No	179	3.27
997 Don't Know	64	1.17
999 Refuse to Answer	9	0.16
Total	5,480	100.00

cc006_w3_2_5_: [ZSibName[5]] Have Self-care Ability

	Freq.	%
1 Yes	3,380	96.49
2 No	84	2.40
997 Don't Know	34	0.97
999 Refuse to Answer	5	0.14
Total	3,503	100.00

cc006_w3_2_6_: [ZSibName[6]] Have Self-care Ability

	Freq.	%
1 Yes	1,664	95.85
2 No	40	2.30
997 Don't Know	28	1.61
999 Refuse to Answer	4	0.23
Total	1,736	100.00

cc006_w3_2_7_: [ZSibName[7]] Have Self-care Ability

	Freq.	%
1 Yes	713	95.70
2 No	14	1.88
997 Don't Know	15	2.01
999 Refuse to Answer	3	0.40

	Freq.	%
Total	745	100.00

cc006_w3_2_8_: [ZSibName[8]] Have Self-care Ability

	Freq.	%
1 Yes	247	91.48
2 No	8	2.96
997 Don't Know	12	4.44
999 Refuse to Answer	3	1.11
Total	270	100.00

cc006_w3_2_9_: [ZSibName[9]] Have Self-care Ability

	Freq.	%
1 Yes	105	90.52
2 No	4	3.45
997 Don't Know	4	3.45
999 Refuse to Answer	3	2.59
Total	116	100.00

cc006_w3_2_10_: [ZSibName[10]] Have Self-care Ability

	Freq.	%
1 Yes	36	97.30
997 Don't Know	1	2.70
Total	37	100.00

cc006_w3_2_11_: [ZSibName[11]] Have Self-care Ability

	Freq.	%
1 Yes	17	94.44
2 No	1	5.56
Total	18	100.00

cc006_w3_2_12_: [ZSibName[12]] Have Self-care Ability

	Freq.	%
1 Yes	4	80.00
2 No	1	20.00
Total	5	100.00

cc006_w3_2_13_: [ZSibName[13]] Have Self-care Ability

	Freq.	%
1 Yes	2	100.00
Total	2	100.00

cc006_w3_2_14_: [ZSibName[14]] Have Self-care Ability

	Freq.	%
1 Yes	1	100.00
Total	1	100.00

cc006_w3_2_s_1_: [ZSibName[1]] Have Self-care Ability

	Freq.	%
1 Yes	6,179	94.34
2 No	289	4.41
997 Don't Know	72	1.10
999 Refuse to Answer	10	0.15
Total	6,550	100.00

cc006_w3_2_s_2_: [ZSibName[2]] Have Self-care Ability

	Freq.	%
1 Yes	6,321	96.11
2 No	190	2.89
997 Don't Know	55	0.84
999 Refuse to Answer	11	0.17
Total	6,577	100.00

cc006_w3_2_s_3_: [ZSibName[3]] Have Self-care Ability

	Freq.	%
1 Yes	5,524	96.27
2 No	145	2.53
997 Don't Know	59	1.03
999 Refuse to Answer	10	0.17
Total	5,738	100.00

cc006_w3_2_s_4_: [ZSibName[4]] Have Self-care Ability

	Freq.	%
1 Yes	4,079	96.77
2 No	86	2.04
997 Don't Know	39	0.93
999 Refuse to Answer	11	0.26
Total	4,215	100.00

cc006_w3_2_s_5_: [ZSibName[5]] Have Self-care Ability

	Freq.	%
1 Yes	2,559	96.64
2 No	47	1.77
997 Don't Know	33	1.25
999 Refuse to Answer	9	0.34
Total	2,648	100.00

cc006_w3_2_s_6_: [ZSibName[6]] Have Self-care Ability

	Freq.	%
1 Yes	1,277	97.11
2 No	24	1.83
997 Don't Know	11	0.84
999 Refuse to Answer	3	0.23
Total	1,315	100.00

cc006_w3_2_s_7_: [ZSibName[7]] Have Self-care Ability

	Freq.	%
1 Yes	597	97.23
2 No	7	1.14
997 Don't Know	7	1.14
999 Refuse to Answer	3	0.49
Total	614	100.00

cc006_w3_2_s_8_: [ZSibName[8]] Have Self-care Ability

	Freq.	%
1 Yes	229	94.63
2 No	6	2.48
997 Don't Know	4	1.65
999 Refuse to Answer	3	1.24
Total	242	100.00

cc006_w3_2_s_9_: [ZSibName[9]] Have Self-care Ability

	Freq.	%
1 Yes	63	92.65
997 Don't Know	3	4.41
999 Refuse to Answer	2	2.94
Total	68	100.00

cc006_w3_2_s_10_: [ZSibName[10]] Have Self-care Ability

	Freq.	%
1 Yes	17	85.00
2 No	1	5.00
999 Refuse to Answer	2	10.00
Total	20	100.00

cc006_w3_2_s_11_: [ZSibName[11]] Have Self-care Ability

	Freq.	%
1 Yes	9	81.82
997 Don't Know	1	9.09
999 Refuse to Answer	1	9.09
Total	11	100.00

cc006_w3_2_s_12_: [ZSibName[12]] Have Self-care Ability

	Freq.	%
1 Yes	4	66.67
997 Don't Know	1	16.67
999 Refuse to Answer	1	16.67
Total	6	100.00

cc006_w3_2_s_13_: [ZSibName[13]] Have Self-care Ability

	Freq.	%
1 Yes	1	50.00
999 Refuse to Answer	1	50.00
Total	2	100.00

cc006_w3_2_s_14_: [ZSibName[14]] Have Self-care Ability

	Freq.	%
999 Refuse to Answer	1	100.00
Total	1	100.00

cc006_w3_2_s_15_: [ZSibName[15]] Have Self-care Ability

	Freq.	%
1 Yes	1	50.00
999 Refuse to Answer	1	50.00
Total	2	100.00

a001_w4_s1: Select Household Member from List

Mean	SD	Min	Max	Obs
1.00	0.00	1.00	1.00	118

a001_w4_s2: Select Household Member from List

Mean	SD	Min	Max	Obs
2.00	0.00	2.00	2.00	307

a001_w4_s3: Select Household Member from List

Mean	SD	Min	Max	Obs
3.00	0.00	3.00	3.00	4

a001_w4_s4: Select Household Member from List

Mean	SD	Min	Max	Obs
4.00	0.00	4.00	4.00	5

a001_w4_s5: Select Household Member from List

Mean	SD	Min	Max	Obs
5.00	0.00	5.00	5.00	105

a001_w4_s6: Select Household Member from List

Mean	SD	Min	Max	Obs
6.00	0.00	6.00	6.00	195

a001_w4_s7: Select Household Member from List

Mean	SD	Min	Max	Obs
7.00	0.00	7.00	7.00	2

a001_w4_s8: Select Household Member from List

Mean	SD	Min	Max	Obs
8.00	0.00	8.00	8.00	4

a001_w4_s9: Select Household Member from List

Mean	SD	Min	Max	Obs
9.00	0.00	9.00	9.00	2,703

a001_w4_s10: Select Household Member from List

Mean	SD	Min	Max	Obs
10.00	0.00	10.00	10.00	1,541

a001_w4_s11: Select Household Member from List

Mean	SD	Min	Max	Obs
11.00	0.00	11.00	11.00	541

a001_w4_s12: Select Household Member from List

Mean	SD	Min	Max	Obs
12.00	0.00	12.00	12.00	206

a001_w4_s13: Select Household Member from List

Mean	SD	Min	Max	Obs
13.00	0.00	13.00	13.00	90

a001_w4_s14: Select Household Member from List

Mean	SD	Min	Max	Obs
14.00	0.00	14.00	14.00	24

a001_w4_s15: Select Household Member from List

Mean	SD	Min	Max	Obs
15.00	0.00	15.00	15.00	15

a001_w4_s16: Select Household Member from List

Mean	SD	Min	Max	Obs
16.00	0.00	16.00	16.00	8

a001_w4_s17: Select Household Member from List

Mean	SD	Min	Max	Obs
17.00	0.00	17.00	17.00	2

a001_w4_s18: Select Household Member from List

No Observations

a001_w4_s19: Select Household Member from List

No Observations

a001_w4_s20: Select Household Member from List

No Observations

a001_w4_s21: Select Household Member from List

No Observations

a001_w4_s22: Select Household Member from List

No Observations

a001_w4_s23: Select Household Member from List

No Observations

a001_w4_s24: Select Household Member from List

No Observations

a001_w4_s25: Select Household Member from List

No Observations

a001_w4_s26: Select Household Member from List

No Observations

a001_w4_s27: Select Household Member from List

No Observations

a001_w4_s28: Select Household Member from List

No Observations

a001_w4_s29: Select Household Member from List

No Observations

a001_w4_s30: Select Household Member from List

No Observations

a001_w4_s31: Select Household Member from List

No Observations

a001_w4_s32: Select Household Member from List

No Observations

a001_w4_s33: Select Household Member from List

No Observations

a001_w4_s34: Select Household Member from List

Mean	SD	Min	Max	Obs
34.00	0.00	34.00	34.00	35

a001_w4_s35: Select Household Member from List

Mean	SD	Min	Max	Obs
35.00	0.00	35.00	35.00	22

a001_w4_s36: Select Household Member from List

Mean	SD	Min	Max	Obs
36.00	0.00	36.00	36.00	16

a001_w4_s37: Select Household Member from List

Mean	SD	Min	Max	Obs
37.00	0.00	37.00	37.00	13

a001_w4_s38: Select Household Member from List

Mean	SD	Min	Max	Obs
38.00	0.00	38.00	38.00	2

a001_w4_s39: Select Household Member from List

Mean	SD	Min	Max	Obs
39.00	0.00	39.00	39.00	4

a001_w4_s40: Select Household Member from List

Mean	SD	Min	Max	Obs
40.00	0.00	40.00	40.00	3

a001_w4_s41: Select Household Member from List

Mean	SD	Min	Max	Obs
41.00	0.00	41.00	41.00	2

a001_w4_s42: Select Household Member from List

Mean	SD	Min	Max	Obs
42.00	.	42.00	42.00	1

a001_w4_s43: Select Household Member from List

No Observations

a001_w4_s44: Select Household Member from List

No Observations

a001_w4_s45: Select Household Member from List

No Observations

a001_w4_s46: Select Household Member from List

No Observations

a001_w4_s47: Select Household Member from List

No Observations

a001_w4_s48: Select Household Member from List

No Observations

a001_w4_s49: Select Household Member from List

Mean	SD	Min	Max	Obs
49.00	0.00	49.00	49.00	14

a001_w4_s50: Select Household Member from List

Mean	SD	Min	Max	Obs
50.00	0.00	50.00	50.00	8

a001_w4_s51: Select Household Member from List

Mean	SD	Min	Max	Obs
51.00	0.00	51.00	51.00	6

a001_w4_s52: Select Household Member from List

Mean	SD	Min	Max	Obs
52.00	0.00	52.00	52.00	3

a001_w4_s53: Select Household Member from List

Mean	SD	Min	Max	Obs
53.00	.	53.00	53.00	1

a001_w4_s54: Select Household Member from List

Mean	SD	Min	Max	Obs
54.00	.	54.00	54.00	1

a005_w3_1_: HHMember[1]'s Gender

	Freq.	%
1 Male	741	29.05
2 Female	1,810	70.95

Total	2,551	100.00

a005_w3_2_: HHMember[2]'s Gender

	Freq.	%
1 Male	856	51.44
2 Female	808	48.56
Total	1,664	100.00

a005_w3_3_: HHMember[3]'s Gender

	Freq.	%
1 Male	408	47.11
2 Female	458	52.89
Total	866	100.00

a005_w3_4_: HHMember[4]'s Gender

	Freq.	%
1 Male	132	55.23
2 Female	107	44.77
Total	239	100.00

a005_w3_5_: HHMember[5]'s Gender

	Freq.	%
1 Male	44	46.32
2 Female	51	53.68
Total	95	100.00

a005_w3_6_: HHMember[6]'s Gender

	Freq.	%
1 Male	18	47.37
2 Female	20	52.63
Total	38	100.00

a005_w3_7_: HHMember[7]'s Gender

	Freq.	%
1 Male	9	47.37
2 Female	10	52.63
Total	19	100.00

a005_w3_8_: HHMember[8]'s Gender

	Freq.	%
1 Male	4	50.00
2 Female	4	50.00

Total	8	100.00

a005_w3_9_: HHMember[9]'s Gender

	Freq.	%
1 Male	2	66.67
2 Female	1	33.33
Total	3	100.00

a005_w3_10_: HHMember[10]'s Gender

	Freq.	%
1 Male	1	50.00
2 Female	1	50.00
Total	2	100.00

a005_w4_1_: HHMember[1]'s Age

Mean	SD	Min	Max	Obs
27.37	15.97	0.00	105.00	2,551

a005_w4_2_: HHMember[2]'s Age

Mean	SD	Min	Max	Obs
12.06	10.33	0.00	85.00	1,664

a005_w4_3_: HHMember[3]'s Age

Mean	SD	Min	Max	Obs
9.97	9.61	0.00	87.00	866

a005_w4_4_: HHMember[4]'s Age

Mean	SD	Min	Max	Obs
9.51	9.59	0.00	49.00	239

a005_w4_5_: HHMember[5]'s Age

Mean	SD	Min	Max	Obs
8.27	9.83	0.00	39.00	95

a005_w4_6_: HHMember[6]'s Age

Mean	SD	Min	Max	Obs
9.18	11.76	0.00	55.00	38

a005_w4_7_: HHMember[7]'s Age

Mean	SD	Min	Max	Obs
5.63	5.72	0.00	25.00	19

a005_w4_8_: HHMember[8]'s Age

Mean	SD	Min	Max	Obs
4.13	2.90	1.00	9.00	8

a005_w4_9_: HHMember[9]'s Age

Mean	SD	Min	Max	Obs
6.67	6.11	0.00	12.00	3

a005_w4_10_: HHMember[10]'s Age

Mean	SD	Min	Max	Obs
7.50	2.12	6.00	9.00	2

a006_1_: Relationship

	Freq.	%
1 Spouse of Child	1,529	59.94
2 Grandson or Granddaughter	861	33.75
3 Brother-in-law, sister-in-law	8	0.31
5 Mother	1	0.04
6 Mother-in-law	9	0.35
7 Father-in-law	7	0.27
8 Children	61	2.39
9 Sibling	4	0.16
10 Nanny or Driver	4	0.16
11 Other Relatives	67	2.63
Total	2,551	100.00

a006_2_: Relationship

	Freq.	%
1 Spouse of Child	115	6.91
2 Grandson or Granddaughter	1,455	87.44
3 Brother-in-law, sister-in-law	5	0.30
6 Mother-in-law	3	0.18
8 Children	20	1.20
11 Other Relatives	66	3.97
Total	1,664	100.00

a006_3_: Relationship

	Freq.	%
1 Spouse of Child	41	4.73
2 Grandson or Granddaughter	755	87.18

	Freq.	%
3 Brother-in-law, sister-in-law	1	0.12
6 Mother-in-law	2	0.23
8 Children	3	0.35
11 Other Relatives	64	7.39
Total	866	100.00

a006_4_: Relationship

	Freq.	%
1 Spouse of Child	11	4.60
2 Grandson or Granddaughter	173	72.38
8 Children	3	1.26
10 Nanny or Driver	1	0.42
11 Other Relatives	51	21.34
Total	239	100.00

a006_5_: Relationship

	Freq.	%
1 Spouse of Child	6	6.32
2 Grandson or Granddaughter	64	67.37
8 Children	2	2.11
11 Other Relatives	23	24.21
Total	95	100.00

a006_6_: Relationship

	Freq.	%
1 Spouse of Child	2	5.26
2 Grandson or Granddaughter	28	73.68
8 Children	1	2.63
11 Other Relatives	7	18.42
Total	38	100.00

a006_7_: Relationship

	Freq.	%
1 Spouse of Child	1	5.26
2 Grandson or Granddaughter	15	78.95
11 Other Relatives	3	15.79
Total	19	100.00

a006_8_: Relationship

	Freq.	%
2 Grandson or Granddaughter	5	62.50
11 Other Relatives	3	37.50
Total	8	100.00

a006_9_: Relationship

	Freq.	%
2 Grandson or Granddaughter	3	100.00
Total	3	100.00

a006_10_: Relationship

	Freq.	%
2 Grandson or Granddaughter	2	100.00
Total	2	100.00

a007_w4_1_: Which Child's Spouse is [HHMember[1]]

	Freq.	%
1 XChildName[1]	970	63.44
2 XChildName[2]	345	22.56
3 XChildName[3]	129	8.44
4 XChildName[4]	49	3.20
5 XChildName[5]	19	1.24
6 XChildName[6]	10	0.65
7 XChildName[7]	2	0.13
8 XChildName[8]	1	0.07
9 XChildName[9]	1	0.07
99 Other	3	0.20
Total	1,529	100.00

a007_w4_2_: Which Child's Spouse is [HHMember[2]]

	Freq.	%
1 XChildName[1]	45	39.13
2 XChildName[2]	43	37.39
3 XChildName[3]	15	13.04
4 XChildName[4]	4	3.48
5 XChildName[5]	3	2.61
6 XChildName[6]	2	1.74
7 XChildName[7]	2	1.74
99 Other	1	0.87
Total	115	100.00

a007_w4_3_: Which Child's Spouse is [HHMember[3]]

	Freq.	%
1 XChildName[1]	25	60.98
2 XChildName[2]	12	29.27
3 XChildName[3]	3	7.32
5 XChildName[5]	1	2.44
Total	41	100.00

a007_w4_4_: Which Child's Spouse is [HHMember[4]]

	Freq.	%

	Freq.	%
1 XChildName[1]	4	36.36
2 XChildName[2]	6	54.55
3 XChildName[3]	1	9.09
Total	11	100.00

a007_w4_5_: Which Child's Spouse is [HHMember[5]]

	Freq.	%
1 XChildName[1]	4	66.67
2 XChildName[2]	1	16.67
5 XChildName[5]	1	16.67
Total	6	100.00

a007_w4_6_: Which Child's Spouse is [HHMember[6]]

	Freq.	%
2 XChildName[2]	1	50.00
3 XChildName[3]	1	50.00
Total	2	100.00

a007_w4_7_: Which Child's Spouse is [HHMember[7]]

	Freq.	%
2 XChildName[2]	1	100.00
Total	1	100.00

a008_w4_1_: Which Child's Children is [HHMember[1]]

	Freq.	%
1 XChildName[1]	505	58.65
2 XChildName[2]	219	25.44
3 XChildName[3]	81	9.41
4 XChildName[4]	31	3.60
5 XChildName[5]	13	1.51
6 XChildName[6]	2	0.23
7 XChildName[7]	6	0.70
8 XChildName[8]	1	0.12
9 XChildName[9]	1	0.12
99 Other	2	0.23
Total	861	100.00

a008_w4_2_: Which Child's Children is [HHMember[2]]

	Freq.	%
1 XChildName[1]	918	63.09
2 XChildName[2]	339	23.30
3 XChildName[3]	120	8.25
4 XChildName[4]	44	3.02
5 XChildName[5]	18	1.24
6 XChildName[6]	10	0.69
7 XChildName[7]	3	0.21
8 XChildName[8]	2	0.14

	Freq.	%
99 Other	1	0.07
Total	1,455	100.00

a008_w4_3_: Which Child's Children is [HHMember[3]]

	Freq.	%
1 XChildName[1]	455	60.26
2 XChildName[2]	191	25.30
3 XChildName[3]	61	8.08
4 XChildName[4]	29	3.84
5 XChildName[5]	8	1.06
6 XChildName[6]	7	0.93
7 XChildName[7]	1	0.13
8 XChildName[8]	1	0.13
99 Other	2	0.26
Total	755	100.00

a008_w4_4_: Which Child's Children is [HHMember[4]]

	Freq.	%
1 XChildName[1]	94	54.34
2 XChildName[2]	45	26.01
3 XChildName[3]	15	8.67
4 XChildName[4]	8	4.62
5 XChildName[5]	6	3.47
6 XChildName[6]	2	1.16
7 XChildName[7]	1	0.58
8 XChildName[8]	1	0.58
99 Other	1	0.58
Total	173	100.00

a008_w4_5_: Which Child's Children is [HHMember[5]]

	Freq.	%
1 XChildName[1]	27	42.19
2 XChildName[2]	22	34.38
3 XChildName[3]	8	12.50
4 XChildName[4]	2	3.13
5 XChildName[5]	2	3.13
6 XChildName[6]	1	1.56
7 XChildName[7]	1	1.56
8 XChildName[8]	1	1.56
Total	64	100.00

a008_w4_6_: Which Child's Children is [HHMember[6]]

	Freq.	%
1 XChildName[1]	4	14.29
2 XChildName[2]	15	53.57
3 XChildName[3]	5	17.86
4 XChildName[4]	2	7.14
5 XChildName[5]	1	3.57
6 XChildName[6]	1	3.57

Total	28	100.00

a008_w4_7_: Which Child's Children is [HHMember[7]]

	Freq.	%
1 XChildName[1]	2	13.33
2 XChildName[2]	8	53.33
3 XChildName[3]	5	33.33
Total	15	100.00

a008_w4_8_: Which Child's Children is [HHMember[8]]

	Freq.	%
2 XChildName[2]	4	80.00
4 XChildName[4]	1	20.00
Total	5	100.00

a008_w4_9_: Which Child's Children is [HHMember[9]]

	Freq.	%
2 XChildName[2]	2	66.67
3 XChildName[3]	1	33.33
Total	3	100.00

a008_w4_10_: Which Child's Children is [HHMember[10]]

	Freq.	%
2 XChildName[2]	1	50.00
3 XChildName[3]	1	50.00
Total	2	100.00

xparexist_1_: Whether Parent[1] Exists

	Freq.	%
0 No	305	2.62
1 Yes	11,322	97.38
Total	11,627	100.00

xparexist_2_: Whether Parent[2] Exists

	Freq.	%
0 No	278	2.39
1 Yes	11,346	97.61
Total	11,624	100.00

xparexist_3_: Whether Parent[3] Exists

	Freq.	%
0 No	11,219	96.52

	Freq.	%
1 Yes	404	3.48
Total	11,623	100.00

xparexist_4_: Whether Parent[4] Exists

	Freq.	%
0 No	11,278	97.03
1 Yes	345	2.97
Total	11,623	100.00

xparexist_5_: Whether Parent[5] Exists

	Freq.	%
0 No	162	1.86
1 Yes	8,535	98.14
Total	8,697	100.00

xparexist_6_: Whether Parent[6] Exists

	Freq.	%
0 No	147	1.69
1 Yes	8,547	98.31
Total	8,694	100.00

xparexist_7_: Whether Parent[7] Exists

	Freq.	%
0 No	8,427	96.95
1 Yes	265	3.05
Total	8,692	100.00

xparexist_8_: Whether Parent[8] Exists

	Freq.	%
0 No	8,478	97.54
1 Yes	214	2.46
Total	8,692	100.00

xparbirth_1_: Parent[1]'s Birth Year

Mean	SD	Min	Max	Obs
1,924.99	15.94	1,830.00	2,018.00	5,807

xparbirth_2_: Parent[2]'s Birth Year

Mean	SD	Min	Max	Obs
1,927.97	15.24	1,830.00	2,018.00	5,737

xparbirth_3_: Parent[3]'s Birth Year

Mean	SD	Min	Max	Obs
1,938.34	10.61	1,900.00	1,955.00	38

xparbirth_4_: Parent[4]'s Birth Year

Mean	SD	Min	Max	Obs
1,937.22	10.85	1,900.00	1,958.00	51

xparbirth_5_: Parent[5]'s Birth Year

Mean	SD	Min	Max	Obs
1,927.89	15.31	1,800.00	2,018.00	4,472

xparbirth_6_: Parent[6]'s Birth Year

Mean	SD	Min	Max	Obs
1,930.95	14.53	1,800.00	2,018.00	4,369

xparbirth_7_: Parent[7]'s Birth Year

Mean	SD	Min	Max	Obs
1,939.93	17.53	1,910.00	2,018.00	27

xparbirth_8_: Parent[8]'s Birth Year

Mean	SD	Min	Max	Obs
1,940.66	12.44	1,900.00	1,979.00	32

xparalive_1_: Whether Parent[1] is Alive

	Freq.	%
1 Yes	1,451	12.82
2 No	9,869	87.18
Total	11,320	100.00

xparalive_2_: Whether Parent[2] is Alive

	Freq.	%
1 Yes	2,627	23.15
2 No	8,719	76.85
Total	11,346	100.00

xparalive_3_: Whether Parent[3] is Alive

	Freq.	%
1 Yes	40	9.90
2 No	364	90.10
Total	404	100.00

xparalive_4_: Whether Parent[4] is Alive

	Freq.	%
1 Yes	55	15.94
2 No	290	84.06
Total	345	100.00

xparalive_5_: Whether Parent[5] is Alive

	Freq.	%
1 Yes	1,412	16.55
2 No	7,121	83.45
Total	8,533	100.00

xparalive_6_: Whether Parent[6] is Alive

	Freq.	%
1 Yes	2,315	27.09
2 No	6,230	72.91
Total	8,545	100.00

xparalive_7_: Whether Parent[7] is Alive

	Freq.	%
1 Yes	27	10.19
2 No	238	89.81
Total	265	100.00

xparalive_8_: Whether Parent[8] is Alive

	Freq.	%
1 Yes	32	14.95
2 No	182	85.05
Total	214	100.00

xchildgender_1_: XChildGender[1]

	Freq.	%
1 Male	7,424	64.67
2 Female	4,055	35.33
Total	11,479	100.00

xchildgender_2_: XChildGender[2]

	Freq.	%
1 Male	5,021	51.98
2 Female	4,638	48.02
Total	9,659	100.00

xchildgender_3_: XChildGender[3]

	Freq.	%
1 Male	2,578	44.88
2 Female	3,166	55.12
Total	5,744	100.00

xchildgender_4_: XChildGender[4]

	Freq.	%
1 Male	1,352	41.19
2 Female	1,930	58.81
Total	3,282	100.00

xchildgender_5_: XChildGender[5]

	Freq.	%
1 Male	709	39.81
2 Female	1,072	60.19
Total	1,781	100.00

xchildgender_6_: XChildGender[6]

	Freq.	%
1 Male	378	40.30
2 Female	560	59.70
Total	938	100.00

xchildgender_7_: XChildGender[7]

	Freq.	%
1 Male	213	43.74
2 Female	274	56.26
Total	487	100.00

xchildgender_8_: XChildGender[8]

	Freq.	%
1 Male	96	40.68
2 Female	140	59.32
Total	236	100.00

xchildgender_9_: XChildGender[9]

	Freq.	%
1 Male	43	41.75
2 Female	60	58.25
Total	103	100.00

xchildgender_10_: XChildGender[10]

	Freq.	%
1 Male	27	45.76
2 Female	32	54.24
Total	59	100.00

xchildgender_11_: XChildGender[11]

	Freq.	%
1 Male	15	50.00
2 Female	15	50.00
Total	30	100.00

xchildgender_12_: XChildGender[12]

	Freq.	%
1 Male	8	50.00
2 Female	8	50.00
Total	16	100.00

xchildgender_13_: XChildGender[13]

	Freq.	%
1 Male	4	80.00
2 Female	1	20.00
Total	5	100.00

xchildgender_14_: XChildGender[14]

	Freq.	%
2 Female	3	100.00
Total	3	100.00

xchildgender_15_: XChildGender[15]

	Freq.	%
2 Female	2	100.00
Total	2	100.00

xchildgender_16_: XChildGender[16]

	Freq.	%
2 Female	1	100.00
Total	1	100.00

xchildbirth_1_: XChildBirth[1]

Mean	SD	Min	Max	Obs
1,980.73	11.92	1,900.00	2,018.00	11,470

xchildbirth_2_: XChildBirth[2]

Mean	SD	Min	Max	Obs
1,980.61	12.94	1,900.00	2,018.00	9,645

xchildbirth_3_: XChildBirth[3]

Mean	SD	Min	Max	Obs
1,977.83	12.51	1,900.00	2,018.00	5,737

xchildbirth_4_: XChildBirth[4]

Mean	SD	Min	Max	Obs
1,975.74	12.67	1,900.00	2,018.00	3,266

xchildbirth_5_: XChildBirth[5]

Mean	SD	Min	Max	Obs
1,974.29	13.36	1,900.00	2,018.00	1,763

xchildbirth_6_: XChildBirth[6]

Mean	SD	Min	Max	Obs
1,973.32	14.70	1,900.00	2,018.00	920

xchildbirth_7_: XChildBirth[7]

Mean	SD	Min	Max	Obs
1,973.31	14.84	1,900.00	2,018.00	477

xchildbirth_8_: XChildBirth[8]

Mean	SD	Min	Max	Obs
1,973.97	15.79	1,900.00	2,018.00	230

xchildbirth_9_: XChildBirth[9]

Mean	SD	Min	Max	Obs
1,973.95	15.18	1,900.00	2,013.00	100

xchildbirth_10_: XChildBirth[10]

Mean	SD	Min	Max	Obs
1,973.38	17.27	1,900.00	2,018.00	56

xchildbirth_11_: XChildBirth[11]

Mean	SD	Min	Max	Obs
1,976.18	12.13	1,955.00	2,015.00	28

xchildbirth_12_: XChildBirth[12]

Mean	SD	Min	Max	Obs
1,972.07	21.81	1,900.00	1,999.00	15

xchildbirth_13_: XChildBirth[13]

Mean	SD	Min	Max	Obs
1,972.80	8.32	1,966.00	1,987.00	5

xchildbirth_14_: XChildBirth[14]

Mean	SD	Min	Max	Obs
1,972.33	3.21	1,970.00	1,976.00	3

xchildbirth_15_: XChildBirth[15]

Mean	SD	Min	Max	Obs
1,967.50	7.78	1,962.00	1,973.00	2

xchildbirth_16_: XChildBirth[16]

Mean	SD	Min	Max	Obs
1,956.00	.	1,956.00	1,956.00	1

xchildtype_1_: XChildType[1]

	Freq.	%
1 [FamilyR] and [Spouse]'s Own	5,741	50.01
2 [FamilyR] or [Spouse]'s Adopted	65	0.57
3 [FamilyR]'s Own	5,354	46.64
4 [Spouse]'s Own	90	0.78
5 Not my Child	229	1.99

Total	11,479	100.00

xchildtype_2_: XChildType[2]

	Freq.	%
1 [FamilyR] and [Spouse]'s Own	4,596	47.58
2 [FamilyR] or [Spouse]'s Adopted	54	0.56
3 [FamilyR]'s Own	4,593	47.55
4 [Spouse]'s Own	96	0.99
5 Not my Child	320	3.31
Total	9,659	100.00

xchildtype_3_: XChildType[3]

	Freq.	%
1 [FamilyR] and [Spouse]'s Own	2,307	40.16
2 [FamilyR] or [Spouse]'s Adopted	52	0.91
3 [FamilyR]'s Own	3,014	52.47
4 [Spouse]'s Own	79	1.38
5 Not my Child	292	5.08
Total	5,744	100.00

xchildtype_4_: XChildType[4]

	Freq.	%
1 [FamilyR] and [Spouse]'s Own	1,046	31.87
2 [FamilyR] or [Spouse]'s Adopted	43	1.31
3 [FamilyR]'s Own	1,848	56.31
4 [Spouse]'s Own	72	2.19
5 Not my Child	273	8.32
Total	3,282	100.00

xchildtype_5_: XChildType[5]

	Freq.	%
1 [FamilyR] and [Spouse]'s Own	457	25.66
2 [FamilyR] or [Spouse]'s Adopted	29	1.63
3 [FamilyR]'s Own	1,009	56.65
4 [Spouse]'s Own	50	2.81
5 Not my Child	236	13.25
Total	1,781	100.00

xchildtype_6_: XChildType[6]

	Freq.	%
1 [FamilyR] and [Spouse]'s Own	186	19.83
2 [FamilyR] or [Spouse]'s Adopted	12	1.28
3 [FamilyR]'s Own	533	56.82
4 [Spouse]'s Own	29	3.09
5 Not my Child	178	18.98
Total	938	100.00

xchildtype_7_: XChildType[7]

	Freq.	%
1 [FamilyR] and [Spouse]'s Own	79	16.22
2 [FamilyR] or [Spouse]'s Adopted	11	2.26
3 [FamilyR]'s Own	269	55.24
4 [Spouse]'s Own	19	3.90
5 Not my Child	109	22.38
Total	487	100.00

xchildtype_8_: XChildType[8]

	Freq.	%
1 [FamilyR] and [Spouse]'s Own	41	17.37
2 [FamilyR] or [Spouse]'s Adopted	6	2.54
3 [FamilyR]'s Own	120	50.85
4 [Spouse]'s Own	10	4.24
5 Not my Child	59	25.00
Total	236	100.00

xchildtype_9_: XChildType[9]

	Freq.	%
1 [FamilyR] and [Spouse]'s Own	15	14.56
2 [FamilyR] or [Spouse]'s Adopted	3	2.91
3 [FamilyR]'s Own	50	48.54
4 [Spouse]'s Own	6	5.83
5 Not my Child	29	28.16
Total	103	100.00

xchildtype_10_: XChildType[10]

	Freq.	%
1 [FamilyR] and [Spouse]'s Own	9	15.25
2 [FamilyR] or [Spouse]'s Adopted	2	3.39
3 [FamilyR]'s Own	24	40.68
4 [Spouse]'s Own	5	8.47
5 Not my Child	19	32.20
Total	59	100.00

xchildtype_11_: XChildType[11]

	Freq.	%
1 [FamilyR] and [Spouse]'s Own	4	13.33
2 [FamilyR] or [Spouse]'s Adopted	1	3.33
3 [FamilyR]'s Own	17	56.67
4 [Spouse]'s Own	1	3.33
5 Not my Child	7	23.33
Total	30	100.00

xchildtype_12_: XChildType[12]

	Freq.	%
2 [FamilyR] or [Spouse]'s Adopted	1	6.25
3 [FamilyR]'s Own	7	43.75
4 [Spouse]'s Own	2	12.50
5 Not my Child	6	37.50
Total	16	100.00

xchildtype_13_: XChildType[13]

	Freq.	%
3 [FamilyR]'s Own	1	20.00
4 [Spouse]'s Own	1	20.00
5 Not my Child	3	60.00
Total	5	100.00

xchildtype_14_: XChildType[14]

	Freq.	%
3 [FamilyR]'s Own	1	33.33
4 [Spouse]'s Own	1	33.33
5 Not my Child	1	33.33
Total	3	100.00

xchildtype_15_: XChildType[15]

	Freq.	%
4 [Spouse]'s Own	1	50.00
5 Not my Child	1	50.00
Total	2	100.00

xchildtype_16_: XChildType[16]

	Freq.	%
5 Not my Child	1	100.00
Total	1	100.00

xchildalive_1_: XChildAlive[1]

	Freq.	%
1 Alive	11,058	96.33
2 Dead	421	3.67
Total	11,479	100.00

xchildalive_2_: XChildAlive[2]

	Freq.	%
1 Alive	9,210	95.35
2 Dead	449	4.65

Total	9,659	100.00

xchildalive_3_: XChildAlive[3]

	Freq.	%
1 Alive	5,230	91.05
2 Dead	514	8.95
Total	5,744	100.00

xchildalive_4_: XChildAlive[4]

	Freq.	%
1 Alive	2,837	86.44
2 Dead	445	13.56
Total	3,282	100.00

xchildalive_5_: XChildAlive[5]

	Freq.	%
1 Alive	1,395	78.33
2 Dead	386	21.67
Total	1,781	100.00

xchildalive_6_: XChildAlive[6]

	Freq.	%
1 Alive	658	70.15
2 Dead	280	29.85
Total	938	100.00

xchildalive_7_: XChildAlive[7]

	Freq.	%
1 Alive	320	65.71
2 Dead	167	34.29
Total	487	100.00

xchildalive_8_: XChildAlive[8]

	Freq.	%
1 Alive	156	66.10
2 Dead	80	33.90
Total	236	100.00

xchildalive_9_: XChildAlive[9]

	Freq.	%
1 Alive	63	61.17
2 Dead	40	38.83

Total	103	100.00

xchildalive_10_: XChildAlive[10]

	Freq.	%
1 Alive	38	64.41
2 Dead	21	35.59
Total	59	100.00

xchildalive_11_: XChildAlive[11]

	Freq.	%
1 Alive	22	73.33
2 Dead	8	26.67
Total	30	100.00

xchildalive_12_: XChildAlive[12]

	Freq.	%
1 Alive	12	75.00
2 Dead	4	25.00
Total	16	100.00

xchildalive_13_: XChildAlive[13]

	Freq.	%
1 Alive	3	60.00
2 Dead	2	40.00
Total	5	100.00

xchildalive_14_: XChildAlive[14]

	Freq.	%
1 Alive	3	100.00
Total	3	100.00

xchildalive_15_: XChildAlive[15]

	Freq.	%
1 Alive	2	100.00
Total	2	100.00

xchildalive_16_: XChildAlive[16]

	Freq.	%
1 Alive	1	100.00
Total	1	100.00

xsibgender_1_: XSibGender[1]

	Freq.	%
1 Male	6,004	53.30
2 Female	5,260	46.70
Total	11,264	100.00

xsibgender_2_: XSibGender[2]

	Freq.	%
1 Male	5,564	52.91
2 Female	4,951	47.09
Total	10,515	100.00

xsibgender_3_: XSibGender[3]

	Freq.	%
1 Male	4,559	51.52
2 Female	4,290	48.48
Total	8,849	100.00

xsibgender_4_: XSibGender[4]

	Freq.	%
1 Male	3,352	51.19
2 Female	3,196	48.81
Total	6,548	100.00

xsibgender_5_: XSibGender[5]

	Freq.	%
1 Male	2,073	49.88
2 Female	2,083	50.12
Total	4,156	100.00

xsibgender_6_: XSibGender[6]

	Freq.	%
1 Male	1,031	48.34
2 Female	1,102	51.66
Total	2,133	100.00

xsibgender_7_: XSibGender[7]

	Freq.	%
1 Male	479	50.05
2 Female	478	49.95
Total	957	100.00

xsibgender_8_: XSibGender[8]

	Freq.	%
1 Male	200	51.81
2 Female	186	48.19
Total	386	100.00

xsibgender_9_: XSibGender[9]

	Freq.	%
1 Male	76	44.19
2 Female	96	55.81
Total	172	100.00

xsibgender_10_: XSibGender[10]

	Freq.	%
1 Male	29	41.43
2 Female	41	58.57
Total	70	100.00

xsibgender_11_: XSibGender[11]

	Freq.	%
1 Male	21	52.50
2 Female	19	47.50
Total	40	100.00

xsibgender_12_: XSibGender[12]

	Freq.	%
1 Male	8	44.44
2 Female	10	55.56
Total	18	100.00

xsibgender_13_: XSibGender[13]

	Freq.	%
1 Male	3	42.86
2 Female	4	57.14
Total	7	100.00

xsibgender_14_: XSibGender[14]

	Freq.	%
1 Male	3	75.00
2 Female	1	25.00
Total	4	100.00

xsibgender_s_1_: XSibGender_S[1]

	Freq.	%
1 Male	4,542	53.73
2 Female	3,911	46.27
Total	8,453	100.00

xsibgender_s_2_: XSibGender_S[2]

	Freq.	%
1 Male	4,159	52.64
2 Female	3,742	47.36
Total	7,901	100.00

xsibgender_s_3_: XSibGender_S[3]

	Freq.	%
1 Male	3,431	51.43
2 Female	3,240	48.57
Total	6,671	100.00

xsibgender_s_4_: XSibGender_S[4]

	Freq.	%
1 Male	2,462	50.89
2 Female	2,376	49.11
Total	4,838	100.00

xsibgender_s_5_: XSibGender_S[5]

	Freq.	%
1 Male	1,436	47.68
2 Female	1,576	52.32
Total	3,012	100.00

xsibgender_s_6_: XSibGender_S[6]

	Freq.	%
1 Male	756	48.62
2 Female	799	51.38
Total	1,555	100.00

xsibgender_s_7_: XSibGender_S[7]

	Freq.	%
1 Male	362	49.25
2 Female	373	50.75
Total	735	100.00

xsibgender_s_8_: XSibGender_S[8]

	Freq.	%
1 Male	144	48.32
2 Female	154	51.68
Total	298	100.00

xsibgender_s_9_: XSibGender_S[9]

	Freq.	%
1 Male	51	45.54
2 Female	61	54.46
Total	112	100.00

xsibgender_s_10_: XSibGender_S[10]

	Freq.	%
1 Male	22	59.46
2 Female	15	40.54
Total	37	100.00

xsibgender_s_11_: XSibGender_S[11]

	Freq.	%
1 Male	9	64.29
2 Female	5	35.71
Total	14	100.00

xsibgender_s_12_: XSibGender_S[12]

	Freq.	%
1 Male	5	62.50
2 Female	3	37.50
Total	8	100.00

xsibgender_s_13_: XSibGender_S[13]

	Freq.	%
1 Male	2	66.67
2 Female	1	33.33
Total	3	100.00

xsibgender_s_14_: XSibGender_S[14]

	Freq.	%
1 Male	1	33.33
2 Female	2	66.67
Total	3	100.00

xsibgender_s_15_: XSibGender_S[15]

	Freq.	%
1 Male	1	50.00
2 Female	1	50.00
Total	2	100.00

xsibbirth_1_: XSibBirth[1]

Mean	SD	Min	Max	Obs
1,949.92	13.37	1,863.00	2,018.00	11,150

xsibbirth_2_: XSibBirth[2]

Mean	SD	Min	Max	Obs
1,954.26	13.29	1,888.00	2,018.00	10,421

xsibbirth_3_: XSibBirth[3]

Mean	SD	Min	Max	Obs
1,957.28	13.17	1,877.00	2,018.00	8,754

xsibbirth_4_: XSibBirth[4]

Mean	SD	Min	Max	Obs
1,959.43	13.29	1,873.00	2,018.00	6,463

xsibbirth_5_: XSibBirth[5]

Mean	SD	Min	Max	Obs
1,960.65	13.67	1,900.00	2,018.00	4,086

xsibbirth_6_: XSibBirth[6]

Mean	SD	Min	Max	Obs
1,961.26	15.05	1,899.00	2,018.00	2,095

xsibbirth_7_: XSibBirth[7]

Mean	SD	Min	Max	Obs
1,961.45	16.04	1,900.00	2,018.00	928

xsibbirth_8_: XSibBirth[8]

Mean	SD	Min	Max	Obs
1,961.12	18.80	1,900.00	2,018.00	377

xsibbirth_9_: XSibBirth[9]

Mean	SD	Min	Max	Obs
1,961.51	17.31	1,900.00	2,018.00	166

xsibbirth_10_: XSibBirth[10]

Mean	SD	Min	Max	Obs
1,960.74	22.31	1,900.00	2,015.00	70

xsibbirth_11_: XSibBirth[11]

Mean	SD	Min	Max	Obs
1,966.22	16.57	1,938.00	2,014.00	40

xsibbirth_12_: XSibBirth[12]

Mean	SD	Min	Max	Obs
1,970.61	17.09	1,938.00	2,014.00	18

xsibbirth_13_: XSibBirth[13]

Mean	SD	Min	Max	Obs
1,982.00	19.24	1,963.00	2,014.00	7

xsibbirth_14_: XSibBirth[14]

Mean	SD	Min	Max	Obs
1,964.75	44.78	1,900.00	2,000.00	4

xsibbirth_s_1_: XSibBirth_S[1]

Mean	SD	Min	Max	Obs
1,952.32	12.52	1,900.00	2,018.00	8,353

xsibbirth_s_2_: XSibBirth_S[2]

Mean	SD	Min	Max	Obs
1,956.55	12.48	1,854.00	2,018.00	7,788

xsibbirth_s_3_: XSibBirth_S[3]

Mean	SD	Min	Max	Obs
1,959.50	12.17	1,900.00	2,018.00	6,582

xsibbirth_s_4_: XSibBirth_S[4]

Mean	SD	Min	Max	Obs
1,961.28	12.71	1,900.00	2,018.00	4,768

xsibbirth_s_5_: XSibBirth_S[5]

Mean	SD	Min	Max	Obs
1,962.65	12.18	1,900.00	2,018.00	2,970

xsibbirth_s_6_: XSibBirth_S[6]

Mean	SD	Min	Max	Obs
1,963.04	12.58	1,900.00	2,018.00	1,524

xsibbirth_s_7_: XSibBirth_S[7]

Mean	SD	Min	Max	Obs
1,964.06	13.51	1,900.00	2,018.00	712

xsibbirth_s_8_: XSibBirth_S[8]

Mean	SD	Min	Max	Obs
1,963.62	15.55	1,900.00	2,014.00	284

xsibbirth_s_9_: XSibBirth_S[9]

Mean	SD	Min	Max	Obs
1,960.01	20.68	1,900.00	2,000.00	108

xsibbirth_s_10_: XSibBirth_S[10]

Mean	SD	Min	Max	Obs
1,961.17	21.09	1,900.00	2,000.00	35

xsibbirth_s_11_: XSibBirth_S[11]

Mean	SD	Min	Max	Obs
1,956.46	25.38	1,900.00	2,000.00	13

xsibbirth_s_12_: XSibBirth_S[12]

Mean	SD	Min	Max	Obs
1,951.50	25.89	1,900.00	1,982.00	8

xsibbirth_s_13_: XSibBirth_S[13]

Mean	SD	Min	Max	Obs
1,960.00	19.08	1,938.00	1,972.00	3

xsibbirth_s_14_: XSibBirth_S[14]

Mean	SD	Min	Max	Obs
1,962.67	19.86	1,940.00	1,977.00	3

xsibbirth_s_15_: XSibBirth_S[15]

Mean	SD	Min	Max	Obs
1,957.00	21.21	1,942.00	1,972.00	2

xsibtype_1_: XSibType[1]

	Freq.	%
1 The Same Parents	10,606	94.16
2 The Same Father but Diff. Mothers	230	2.04
3 The Same Mother but Diff. Fathers	209	1.86
4 Dff. Fathers and Diff. Mothers	119	1.06
5 Not My Sibling	100	0.89
Total	11,264	100.00

xsibtype_2_: XSibType[2]

	Freq.	%
1 The Same Parents	9,956	94.68
2 The Same Father but Diff. Mothers	169	1.61
3 The Same Mother but Diff. Fathers	212	2.02
4 Dff. Fathers and Diff. Mothers	81	0.77
5 Not My Sibling	97	0.92
Total	10,515	100.00

xsibtype_3_: XSibType[3]

	Freq.	%
1 The Same Parents	8,368	94.56
2 The Same Father but Diff. Mothers	129	1.46
3 The Same Mother but Diff. Fathers	183	2.07
4 Dff. Fathers and Diff. Mothers	65	0.73
5 Not My Sibling	104	1.18
Total	8,849	100.00

xsibtype_4_: XSibType[4]

	Freq.	%
1 The Same Parents	6,140	93.77
2 The Same Father but Diff. Mothers	94	1.44
3 The Same Mother but Diff. Fathers	117	1.79

4 Dff. Fathers and Diff. Mothers	58	0.89
5 Not My Sibling	139	2.12
Total	6,548	100.00

xsibtype_5_: XSibType[5]

	Freq.	%
1 The Same Parents	3,825	92.04
2 The Same Father but Diff. Mothers	67	1.61
3 The Same Mother but Diff. Fathers	87	2.09
4 Dff. Fathers and Diff. Mothers	53	1.28
5 Not My Sibling	124	2.98
Total	4,156	100.00

xsibtype_6_: XSibType[6]

	Freq.	%
1 The Same Parents	1,890	88.61
2 The Same Father but Diff. Mothers	46	2.16
3 The Same Mother but Diff. Fathers	46	2.16
4 Dff. Fathers and Diff. Mothers	38	1.78
5 Not My Sibling	113	5.30
Total	2,133	100.00

xsibtype_7_: XSibType[7]

	Freq.	%
1 The Same Parents	790	82.55
2 The Same Father but Diff. Mothers	24	2.51
3 The Same Mother but Diff. Fathers	22	2.30
4 Dff. Fathers and Diff. Mothers	27	2.82
5 Not My Sibling	94	9.82
Total	957	100.00

xsibtype_8_: XSibType[8]

	Freq.	%
1 The Same Parents	282	73.06
2 The Same Father but Diff. Mothers	13	3.37
3 The Same Mother but Diff. Fathers	6	1.55
4 Dff. Fathers and Diff. Mothers	19	4.92
5 Not My Sibling	66	17.10
Total	386	100.00

xsibtype_9_: XSibType[9]

	Freq.	%
1 The Same Parents	113	65.70
2 The Same Father but Diff. Mothers	7	4.07
3 The Same Mother but Diff. Fathers	1	0.58
4 Dff. Fathers and Diff. Mothers	16	9.30

5 Not My Sibling	35	20.35
Total	172	100.00

xsibtype_10_: XSibType[10]

	Freq.	%
1 The Same Parents	28	40.00
2 The Same Father but Diff. Mothers	5	7.14
3 The Same Mother but Diff. Fathers	2	2.86
4 Dff. Fathers and Diff. Mothers	8	11.43
5 Not My Sibling	27	38.57
Total	70	100.00

xsibtype_11_: XSibType[11]

	Freq.	%
1 The Same Parents	17	42.50
2 The Same Father but Diff. Mothers	4	10.00
4 Dff. Fathers and Diff. Mothers	6	15.00
5 Not My Sibling	13	32.50
Total	40	100.00

xsibtype_12_: XSibType[12]

	Freq.	%
1 The Same Parents	5	27.78
2 The Same Father but Diff. Mothers	1	5.56
4 Dff. Fathers and Diff. Mothers	2	11.11
5 Not My Sibling	10	55.56
Total	18	100.00

xsibtype_13_: XSibType[13]

	Freq.	%
1 The Same Parents	2	28.57
4 Dff. Fathers and Diff. Mothers	1	14.29
5 Not My Sibling	4	57.14
Total	7	100.00

xsibtype_14_: XSibType[14]

	Freq.	%
1 The Same Parents	1	25.00
5 Not My Sibling	3	75.00
Total	4	100.00

xsibtype_s_1_: XSibType_S[1]

	Freq.	%
1 The Same Parents	7,953	94.08

2 The Same Father but Diff. Mothers	165	1.95
3 The Same Mother but Diff. Fathers	184	2.18
4 Dff. Fathers and Diff. Mothers	100	1.18
5 Not My Sibling	51	0.60
Total	8,453	100.00

xsibtype_s_2_: XSibType_S[2]

	Freq.	%
1 The Same Parents	7,527	95.27
2 The Same Father but Diff. Mothers	110	1.39
3 The Same Mother but Diff. Fathers	153	1.94
4 Dff. Fathers and Diff. Mothers	44	0.56
5 Not My Sibling	67	0.85
Total	7,901	100.00

xsibtype_s_3_: XSibType_S[3]

	Freq.	%
1 The Same Parents	6,343	95.08
2 The Same Father but Diff. Mothers	106	1.59
3 The Same Mother but Diff. Fathers	127	1.90
4 Dff. Fathers and Diff. Mothers	42	0.63
5 Not My Sibling	53	0.79
Total	6,671	100.00

xsibtype_s_4_: XSibType_S[4]

	Freq.	%
1 The Same Parents	4,549	94.03
2 The Same Father but Diff. Mothers	72	1.49
3 The Same Mother but Diff. Fathers	103	2.13
4 Dff. Fathers and Diff. Mothers	42	0.87
5 Not My Sibling	72	1.49
Total	4,838	100.00

xsibtype_s_5_: XSibType_S[5]

	Freq.	%
1 The Same Parents	2,817	93.53
2 The Same Father but Diff. Mothers	48	1.59
3 The Same Mother but Diff. Fathers	55	1.83
4 Dff. Fathers and Diff. Mothers	31	1.03
5 Not My Sibling	61	2.03
Total	3,012	100.00

xsibtype_s_6_: XSibType_S[6]

	Freq.	%
1 The Same Parents	1,418	91.19
2 The Same Father but Diff. Mothers	29	1.86

	Freq.	%
3 The Same Mother but Diff. Fathers	29	1.86
4 Dff. Fathers and Diff. Mothers	25	1.61
5 Not My Sibling	54	3.47
Total	1,555	100.00

xsibtype_s_7_: XSibType_S[7]

	Freq.	%
1 The Same Parents	633	86.12
2 The Same Father but Diff. Mothers	16	2.18
3 The Same Mother but Diff. Fathers	15	2.04
4 Dff. Fathers and Diff. Mothers	19	2.59
5 Not My Sibling	52	7.07
Total	735	100.00

xsibtype_s_8_: XSibType_S[8]

	Freq.	%
1 The Same Parents	234	78.52
2 The Same Father but Diff. Mothers	14	4.70
3 The Same Mother but Diff. Fathers	7	2.35
4 Dff. Fathers and Diff. Mothers	13	4.36
5 Not My Sibling	30	10.07
Total	298	100.00

xsibtype_s_9_: XSibType_S[9]

	Freq.	%
1 The Same Parents	75	66.96
2 The Same Father but Diff. Mothers	6	5.36
3 The Same Mother but Diff. Fathers	3	2.68
4 Dff. Fathers and Diff. Mothers	7	6.25
5 Not My Sibling	21	18.75
Total	112	100.00

xsibtype_s_10_: XSibType_S[10]

	Freq.	%
1 The Same Parents	23	62.16
2 The Same Father but Diff. Mothers	3	8.11
3 The Same Mother but Diff. Fathers	1	2.70
4 Dff. Fathers and Diff. Mothers	1	2.70
5 Not My Sibling	9	24.32
Total	37	100.00

xsibtype_s_11_: XSibType_S[11]

	Freq.	%
1 The Same Parents	9	64.29
2 The Same Father but Diff. Mothers	3	21.43
5 Not My Sibling	2	14.29

Total	14	100.00

xsibtype_s_12_: XSibType_S[12]

	Freq.	%
1 The Same Parents	5	62.50
2 The Same Father but Diff. Mothers	2	25.00
5 Not My Sibling	1	12.50
Total	8	100.00

xsibtype_s_13_: XSibType_S[13]

	Freq.	%
1 The Same Parents	1	33.33
2 The Same Father but Diff. Mothers	1	33.33
5 Not My Sibling	1	33.33
Total	3	100.00

xsibtype_s_14_: XSibType_S[14]

	Freq.	%
1 The Same Parents	1	33.33
2 The Same Father but Diff. Mothers	1	33.33
5 Not My Sibling	1	33.33
Total	3	100.00

xsibtype_s_15_: XSibType_S[15]

	Freq.	%
1 The Same Parents	1	50.00
2 The Same Father but Diff. Mothers	1	50.00
Total	2	100.00

xsibalive_1_: XSibAlive[1]

	Freq.	%
1 Alive	8,233	73.09
2 Dead	3,031	26.91
Total	11,264	100.00

xsibalive_2_: XSibAlive[2]

	Freq.	%
1 Alive	8,322	79.14
2 Dead	2,193	20.86
Total	10,515	100.00

xsibalive_3_: XSibAlive[3]

	Freq.	%
1 Alive	7,358	83.15
2 Dead	1,491	16.85
Total	8,849	100.00

xsibalive_4_: XSibAlive[4]

	Freq.	%
1 Alive	5,596	85.46
2 Dead	952	14.54
Total	6,548	100.00

xsibalive_5_: XSibAlive[5]

	Freq.	%
1 Alive	3,614	86.96
2 Dead	542	13.04
Total	4,156	100.00

xsibalive_6_: XSibAlive[6]

	Freq.	%
1 Alive	1,829	85.75
2 Dead	304	14.25
Total	2,133	100.00

xsibalive_7_: XSibAlive[7]

	Freq.	%
1 Alive	831	86.83
2 Dead	126	13.17
Total	957	100.00

xsibalive_8_: XSibAlive[8]

	Freq.	%
1 Alive	332	86.01
2 Dead	54	13.99
Total	386	100.00

xsibalive_9_: XSibAlive[9]

	Freq.	%
1 Alive	150	87.21
2 Dead	22	12.79
Total	172	100.00

xsibalive_10_: XSibAlive[10]

	Freq.	%
1 Alive	63	90.00
2 Dead	7	10.00
Total	70	100.00

xsibalive_11_: XSibAlive[11]

	Freq.	%
1 Alive	31	77.50
2 Dead	9	22.50
Total	40	100.00

xsibalive_12_: XSibAlive[12]

	Freq.	%
1 Alive	15	83.33
2 Dead	3	16.67
Total	18	100.00

xsibalive_13_: XSibAlive[13]

	Freq.	%
1 Alive	6	85.71
2 Dead	1	14.29
Total	7	100.00

xsibalive_14_: XSibAlive[14]

	Freq.	%
1 Alive	4	100.00
Total	4	100.00

xsibalive_s_1_: XSibAlive_S[1]

	Freq.	%
1 Alive	6,591	77.97
2 Dead	1,862	22.03
Total	8,453	100.00

xsibalive_s_2_: XSibAlive_S[2]

	Freq.	%
1 Alive	6,640	84.04
2 Dead	1,261	15.96
Total	7,901	100.00

xsibalive_s_3_: XSibAlive_S[3]

	Freq.	%
1 Alive	5,785	86.72
2 Dead	886	13.28
Total	6,671	100.00

xsibalive_s_4_: XSibAlive_S[4]

	Freq.	%
1 Alive	4,276	88.38
2 Dead	562	11.62
Total	4,838	100.00

xsibalive_s_5_: XSibAlive_S[5]

	Freq.	%
1 Alive	2,704	89.77
2 Dead	308	10.23
Total	3,012	100.00

xsibalive_s_6_: XSibAlive_S[6]

	Freq.	%
1 Alive	1,366	87.85
2 Dead	189	12.15
Total	1,555	100.00

xsibalive_s_7_: XSibAlive_S[7]

	Freq.	%
1 Alive	664	90.34
2 Dead	71	9.66
Total	735	100.00

xsibalive_s_8_: XSibAlive_S[8]

	Freq.	%
1 Alive	270	90.60
2 Dead	28	9.40
Total	298	100.00

xsibalive_s_9_: XSibAlive_S[9]

	Freq.	%
1 Alive	88	78.57
2 Dead	24	21.43
Total	112	100.00

xsibalive_s_10_: XSibAlive_S[10]

	Freq.	%
1 Alive	28	75.68
2 Dead	9	24.32
Total	37	100.00

xsibalive_s_11_: XSibAlive_S[11]

	Freq.	%
1 Alive	13	92.86
2 Dead	1	7.14
Total	14	100.00

xsibalive_s_12_: XSibAlive_S[12]

	Freq.	%
1 Alive	7	87.50
2 Dead	1	12.50
Total	8	100.00

xsibalive_s_13_: XSibAlive_S[13]

	Freq.	%
1 Alive	3	100.00
Total	3	100.00

xsibalive_s_14_: XSibAlive_S[14]

	Freq.	%
1 Alive	2	66.67
2 Dead	1	33.33
Total	3	100.00

xsibalive_s_15_: XSibAlive_S[15]

	Freq.	%
1 Alive	2	100.00
Total	2	100.00

zparbirth_1_: ZParBirth[1]

Mean	SD	Min	Max	Obs
1,921.19	13.62	1,870.00	1,990.00	7,149

zpardyear_1_: ZParDYear[1]

Mean	SD	Min	Max	Obs
2,006.12	14.40	1,936.00	2,014.00	223

zparbirth_2_: ZParBirth[2]

Mean	SD	Min	Max	Obs
1,924.16	13.57	1,870.00	1,993.00	7,466

zpardyear_2_: ZParDYear[2]

Mean	SD	Min	Max	Obs
2,009.93	10.57	1,939.00	2,014.00	290

zparbirth_3_: ZParBirth[3]

Mean	SD	Min	Max	Obs
1,923.07	14.84	1,896.00	1,960.00	96

zparbirth_4_: ZParBirth[4]

Mean	SD	Min	Max	Obs
1,924.72	14.54	1,897.00	1,960.00	99

zparedu_1_: ZParEdu[1]

	Freq.	%
1 No Formal Education(Illiterate)	5,039	60.55
2 Did not Finish Primary School	1,035	12.44
3 Sishu/Home School	675	8.11
4 Elementary School	913	10.97
5 Middle School	348	4.18
6 High School	152	1.83
7 Vocational School	84	1.01
8 Two-/Three-Year College/Associate Degree	30	0.36
9 Four-Year College/Bachelor's Degree	46	0.55
Total	8,322	100.00

zparedu_2_: ZParEdu[2]

	Freq.	%
1 No Formal Education(Illiterate)	7,569	86.79
2 Did not Finish Primary School	440	5.05
3 Sishu/Home School	84	0.96
4 Elementary School	409	4.69
5 Middle School	129	1.48
6 High School	48	0.55
7 Vocational School	27	0.31
8 Two-/Three-Year College/Associate Degree	6	0.07
9 Four-Year College/Bachelor's Degree	9	0.10
Total	8,721	100.00

zparedu_3_: ZParEdu[3]

	Freq.	%
1 No Formal Education(Illiterate)	80	68.97
2 Did not Finish Primary School	15	12.93
3 Sishu/Home School	3	2.59
4 Elementary School	12	10.34
5 Middle School	4	3.45
6 High School	1	0.86
7 Vocational School	1	0.86
Total	116	100.00

zparedu_4_: ZParEdu[4]

	Freq.	%
1 No Formal Education(Illiterate)	109	88.62
2 Did not Finish Primary School	5	4.07
3 Sishu/Home School	2	1.63
4 Elementary School	3	2.44
5 Middle School	3	2.44
6 High School	1	0.81
Total	123	100.00

zparedu_5_: ZParEdu[5]

	Freq.	%
1 No Formal Education(Illiterate)	3,485	60.37
2 Did not Finish Primary School	700	12.13
3 Sishu/Home School	409	7.08
4 Elementary School	671	11.62
5 Middle School	289	5.01
6 High School	102	1.77
7 Vocational School	69	1.20
8 Two-/Three-Year College/Associate Degree	21	0.36
9 Four-Year College/Bachelor's Degree	27	0.47
Total	5,773	100.00

zparedu_6_: ZParEdu[6]

	Freq.	%
1 No Formal Education(Illiterate)	5,300	86.66
2 Did not Finish Primary School	292	4.77
3 Sishu/Home School	59	0.96
4 Elementary School	323	5.28
5 Middle School	86	1.41
6 High School	32	0.52
7 Vocational School	20	0.33
8 Two-/Three-Year College/Associate Degree	1	0.02
9 Four-Year College/Bachelor's Degree	3	0.05
Total	6,116	100.00

zparedu_7_: ZParEdu[7]

	Freq.	%

	Freq.	%
1 No Formal Education(Illiterate)	36	49.32
2 Did not Finish Primary School	12	16.44
3 Sishu/Home School	6	8.22
4 Elementary School	6	8.22
5 Middle School	6	8.22
6 High School	4	5.48
8 Two-/Three-Year College/Associate Degree	2	2.74
9 Four-Year College/Bachelor's Degree	1	1.37
Total	73	100.00

zparedu_8_: ZParEdu[8]

	Freq.	%
1 No Formal Education(Illiterate)	72	84.71
2 Did not Finish Primary School	3	3.53
4 Elementary School	8	9.41
5 Middle School	2	2.35
Total	85	100.00

zparocc_1_: ZParOcc_1_

	Freq.	%
1 Managers	120	11.67
2 Professionals and Technicians	108	10.51
3 Clerks	52	5.06
4 Commercial and Service Workers	21	2.04
5 Agricultural, Forestry, Husbandry and Fishery Producers	626	60.89
6 Production and Transportation Workers	68	6.61
7 Can't be Specified	33	3.21
Total	1,028	100.00

zparocc_2_: ZParOcc_2_

	Freq.	%
1 Managers	35	1.78
2 Professionals and Technicians	48	2.44
3 Clerks	25	1.27
4 Commercial and Service Workers	40	2.04
5 Agricultural, Forestry, Husbandry and Fishery Producers	1,604	81.67
6 Production and Transportation Workers	79	4.02
7 Can't be Specified	133	6.77
Total	1,964	100.00

zparocc_3_: ZParOcc_3_

	Freq.	%
1 Managers	2	7.14
3 Clerks	2	7.14
5 Agricultural, Forestry, Husbandry and Fishery Producers	22	78.57
6 Production and Transportation Workers	1	3.57
7 Can't be Specified	1	3.57
Total	28	100.00

zparocc_4_: ZParOcc_4_

	Freq.	%
1 Managers	1	2.00
2 Professionals and Technicians	2	4.00
5 Agricultural, Forestry, Husbandry and Fishery Producers	44	88.00
6 Production and Transportation Workers	2	4.00
7 Can't be Specified	1	2.00
Total	50	100.00

zparocc_5_: ZParOcc_5_

	Freq.	%
1 Managers	104	10.96
2 Professionals and Technicians	75	7.90
3 Clerks	49	5.16
4 Commercial and Service Workers	13	1.37
5 Agricultural, Forestry, Husbandry and Fishery Producers	637	67.12
6 Production and Transportation Workers	44	4.64
7 Can't be Specified	27	2.85
Total	949	100.00

zparocc_6_: ZParOcc_6_

	Freq.	%
1 Managers	28	1.67
2 Professionals and Technicians	40	2.39
3 Clerks	18	1.07
4 Commercial and Service Workers	24	1.43
5 Agricultural, Forestry, Husbandry and Fishery Producers	1,422	84.79
6 Production and Transportation Workers	47	2.80
7 Can't be Specified	98	5.84
Total	1,677	100.00

zparocc_7_: ZParOcc_7_

	Freq.	%
1 Managers	7	21.21
2 Professionals and Technicians	3	9.09
5 Agricultural, Forestry, Husbandry and Fishery Producers	22	66.67
7 Can't be Specified	1	3.03
Total	33	100.00

zparocc_8_: ZParOcc_8_

	Freq.	%
2 Professionals and Technicians	1	2.08
5 Agricultural, Forestry, Husbandry and Fishery Producers	46	95.83
7 Can't be Specified	1	2.08
Total	48	100.00

zchildnum: ZChildNum

Mean	SD	Min	Max	Obs
2.87	1.72	0.00	16.00	11,625

zchildedu_1_: ZChildEdu_1_

	Freq.	%
1 No Formal Education(Illiterate)	290	2.84
2 Did not Finish Primary School	865	8.48
4 Elementary School	1,943	19.05
5 Middle School	3,396	33.30
6 High School	1,312	12.86
7 Vocational School	646	6.33
8 Two-/Three-Year College/Associate Degree	767	7.52
9 Four-Year College/Bachelor's Degree	874	8.57
10 Post-graduate, Master's Degree	94	0.92
11 Post-graduate, Doctoral Degree/Ph.D	12	0.12
Total	10,199	100.00

zchildedu_2_: ZChildEdu_2_

	Freq.	%
1 No Formal Education(Illiterate)	355	4.20
2 Did not Finish Primary School	980	11.59
3 Sishu/Home School	1	0.01
4 Elementary School	1,882	22.25
5 Middle School	2,849	33.69
6 High School	799	9.45
7 Vocational School	488	5.77
8 Two-/Three-Year College/Associate Degree	495	5.85
9 Four-Year College/Bachelor's Degree	556	6.57
10 Post-graduate, Master's Degree	44	0.52
11 Post-graduate, Doctoral Degree/Ph.D	8	0.09
Total	8,457	100.00

zchildedu_3_: ZChildEdu_3_

	Freq.	%
1 No Formal Education(Illiterate)	318	6.64
2 Did not Finish Primary School	651	13.59
4 Elementary School	1,213	25.32
5 Middle School	1,563	32.62
6 High School	399	8.33
7 Vocational School	215	4.49
8 Two-/Three-Year College/Associate Degree	194	4.05
9 Four-Year College/Bachelor's Degree	215	4.49
10 Post-graduate, Master's Degree	19	0.40
11 Post-graduate, Doctoral Degree/Ph.D	4	0.08
Total	4,791	100.00

zchildedu_4_: ZChildEdu_4_

	Freq.	%

1 No Formal Education(Illiterate)	226	8.89
2 Did not Finish Primary School	415	16.33
4 Elementary School	695	27.35
5 Middle School	770	30.30
6 High School	176	6.93
7 Vocational School	95	3.74
8 Two-/Three-Year College/Associate Degree	70	2.75
9 Four-Year College/Bachelor's Degree	87	3.42
10 Post-graduate, Master's Degree	6	0.24
11 Post-graduate, Doctoral Degree/Ph.D	1	0.04
Total	2,541	100.00

zchildedu_5_: ZChildEdu_5_

	Freq.	%
1 No Formal Education(Illiterate)	176	14.37
2 Did not Finish Primary School	212	17.31
3 Sishu/Home School	1	0.08
4 Elementary School	333	27.18
5 Middle School	335	27.35
6 High School	72	5.88
7 Vocational School	40	3.27
8 Two-/Three-Year College/Associate Degree	13	1.06
9 Four-Year College/Bachelor's Degree	39	3.18
10 Post-graduate, Master's Degree	3	0.24
11 Post-graduate, Doctoral Degree/Ph.D	1	0.08
Total	1,225	100.00

zchildedu_6_: ZChildEdu_6_

	Freq.	%
1 No Formal Education(Illiterate)	81	14.36
2 Did not Finish Primary School	115	20.39
4 Elementary School	150	26.60
5 Middle School	151	26.77
6 High School	33	5.85
7 Vocational School	12	2.13
8 Two-/Three-Year College/Associate Degree	5	0.89
9 Four-Year College/Bachelor's Degree	16	2.84
10 Post-graduate, Master's Degree	1	0.18
Total	564	100.00

zchildedu_7_: ZChildEdu_7_

	Freq.	%
1 No Formal Education(Illiterate)	50	18.59
2 Did not Finish Primary School	56	20.82
4 Elementary School	62	23.05
5 Middle School	66	24.54
6 High School	15	5.58
7 Vocational School	9	3.35
8 Two-/Three-Year College/Associate Degree	2	0.74
9 Four-Year College/Bachelor's Degree	8	2.97
10 Post-graduate, Master's Degree	1	0.37
Total	269	100.00

zchildedu_8_: ZChildEdu_8_

	Freq.	%
1 No Formal Education(Illiterate)	28	21.88
2 Did not Finish Primary School	24	18.75
4 Elementary School	26	20.31
5 Middle School	33	25.78
6 High School	6	4.69
7 Vocational School	5	3.91
8 Two-/Three-Year College/Associate Degree	4	3.13
9 Four-Year College/Bachelor's Degree	2	1.56
Total	128	100.00

zchildedu_9_: ZChildEdu_9_

	Freq.	%
1 No Formal Education(Illiterate)	8	15.69
2 Did not Finish Primary School	12	23.53
4 Elementary School	10	19.61
5 Middle School	13	25.49
6 High School	2	3.92
7 Vocational School	2	3.92
8 Two-/Three-Year College/Associate Degree	3	5.88
9 Four-Year College/Bachelor's Degree	1	1.96
Total	51	100.00

zchildedu_10_: ZChildEdu_10_

	Freq.	%
1 No Formal Education(Illiterate)	7	25.00
2 Did not Finish Primary School	4	14.29
4 Elementary School	7	25.00
5 Middle School	6	21.43
6 High School	1	3.57
7 Vocational School	1	3.57
8 Two-/Three-Year College/Associate Degree	2	7.14
Total	28	100.00

zchildedu_11_: ZChildEdu_11_

	Freq.	%
1 No Formal Education(Illiterate)	2	16.67
2 Did not Finish Primary School	3	25.00
4 Elementary School	1	8.33
5 Middle School	5	41.67
6 High School	1	8.33
Total	12	100.00

zchildedu_12_: ZChildEdu_12_

	Freq.	%
1 No Formal Education(Illiterate)	2	28.57
2 Did not Finish Primary School	2	28.57

4 Elementary School	1	14.29
5 Middle School	2	28.57
Total	7	100.00

zchildedu_13_: ZChildEdu_13_

	Freq.	%
1 No Formal Education(Illiterate)	1	33.33
4 Elementary School	1	33.33
5 Middle School	1	33.33
Total	3	100.00

zchildedu_14_: ZChildEdu_14_

	Freq.	%
7 Vocational School	1	100.00
Total	1	100.00

zsibgender_1_: ZSibGender[1]

	Freq.	%
1 Male	5,766	53.45
2 Female	5,022	46.55
Total	10,788	100.00

zsibgender_2_: ZSibGender[2]

	Freq.	%
1 Male	5,291	52.95
2 Female	4,701	47.05
Total	9,992	100.00

zsibgender_3_: ZSibGender[3]

	Freq.	%
1 Male	4,300	51.58
2 Female	4,036	48.42
Total	8,336	100.00

zsibgender_4_: ZSibGender[4]

	Freq.	%
1 Male	3,117	50.95
2 Female	3,001	49.05
Total	6,118	100.00

zsibgender_5_: ZSibGender[5]

	Freq.	%
1 Male	1,879	48.81
2 Female	1,971	51.19
Total	3,850	100.00

zsibgender_6_: ZSibGender[6]

	Freq.	%
1 Male	934	48.05
2 Female	1,010	51.95
Total	1,944	100.00

zsibgender_7_: ZSibGender[7]

	Freq.	%
1 Male	430	50.47
2 Female	422	49.53
Total	852	100.00

zsibgender_8_: ZSibGender[8]

	Freq.	%
1 Male	162	50.31
2 Female	160	49.69
Total	322	100.00

zsibgender_9_: ZSibGender[9]

	Freq.	%
1 Male	55	40.15
2 Female	82	59.85
Total	137	100.00

zsibgender_10_: ZSibGender[10]

	Freq.	%
1 Male	24	40.00
2 Female	36	60.00
Total	60	100.00

zsibgender_11_: ZSibGender[11]

	Freq.	%
1 Male	16	45.71
2 Female	19	54.29
Total	35	100.00

zsibgender_12_: ZSibGender[12]

	Freq.	%
1 Male	7	43.75
2 Female	9	56.25
Total	16	100.00

zsibgender_13_: ZSibGender[13]

	Freq.	%
1 Male	2	33.33
2 Female	4	66.67
Total	6	100.00

zsibgender_14_: ZSibGender[14]

	Freq.	%
1 Male	2	66.67
2 Female	1	33.33
Total	3	100.00

zsibgender_s_1_: ZSibGender_S[1]

	Freq.	%
1 Male	4,236	53.71
2 Female	3,651	46.29
Total	7,887	100.00

zsibgender_s_2_: ZSibGender_S[2]

	Freq.	%
1 Male	3,853	52.54
2 Female	3,481	47.46
Total	7,334	100.00

zsibgender_s_3_: ZSibGender_S[3]

	Freq.	%
1 Male	3,154	51.02
2 Female	3,028	48.98
Total	6,182	100.00

zsibgender_s_4_: ZSibGender_S[4]

	Freq.	%
1 Male	2,253	50.67
2 Female	2,193	49.33
Total	4,446	100.00

zsibgender_s_5_: ZSibGender_S[5]

	Freq.	%
1 Male	1,316	47.61
2 Female	1,448	52.39
Total	2,764	100.00

zsibgender_s_6_: ZSibGender_S[6]

	Freq.	%
1 Male	701	49.23
2 Female	723	50.77
Total	1,424	100.00

zsibgender_s_7_: ZSibGender_S[7]

	Freq.	%
1 Male	323	48.64
2 Female	341	51.36
Total	664	100.00

zsibgender_s_8_: ZSibGender_S[8]

	Freq.	%
1 Male	121	46.72
2 Female	138	53.28
Total	259	100.00

zsibgender_s_9_: ZSibGender_S[9]

	Freq.	%
1 Male	40	43.48
2 Female	52	56.52
Total	92	100.00

zsibgender_s_10_: ZSibGender_S[10]

	Freq.	%
1 Male	14	51.85
2 Female	13	48.15
Total	27	100.00

zsibgender_s_11_: ZSibGender_S[11]

	Freq.	%
1 Male	5	50.00
2 Female	5	50.00
Total	10	100.00

zsibgender_s_12_: ZSibGender_S[12]

	Freq.	%
1 Male	2	33.33
2 Female	4	66.67
Total	6	100.00

zsibgender_s_13_: ZSibGender_S[13]

	Freq.	%
1 Male	1	50.00
2 Female	1	50.00
Total	2	100.00

zsibgender_s_14_: ZSibGender_S[14]

	Freq.	%
2 Female	2	100.00
Total	2	100.00

zsibgender_s_15_: ZSibGender_S[15]

	Freq.	%
2 Female	1	100.00
Total	1	100.00

zsibbirth_1_: ZSibBirth[1]

Mean	SD	Min	Max	Obs
1,950.20	12.80	1,863.00	2,015.00	9,958

zsibbirth_2_: ZSibBirth[2]

Mean	SD	Min	Max	Obs
1,954.62	12.55	1,862.00	2,015.00	9,167

zsibbirth_3_: ZSibBirth[3]

Mean	SD	Min	Max	Obs
1,957.68	12.07	1,900.00	2,015.00	7,597

zsibbirth_4_: ZSibBirth[4]

Mean	SD	Min	Max	Obs
1,959.98	11.84	1,873.00	2,015.00	5,544

zsibbirth_5_: ZSibBirth[5]

Mean	SD	Min	Max	Obs
1,961.50	11.66	1,868.00	2,015.00	3,435

zsibbirth_6_: ZSibBirth[6]

Mean	SD	Min	Max	Obs
1,962.51	11.89	1,900.00	2,015.00	1,710

zsibbirth_7_: ZSibBirth[7]

Mean	SD	Min	Max	Obs
1,962.78	12.23	1,900.00	2,015.00	729

zsibbirth_8_: ZSibBirth[8]

Mean	SD	Min	Max	Obs
1,962.42	13.14	1,900.00	2,015.00	267

zsibbirth_9_: ZSibBirth[9]

Mean	SD	Min	Max	Obs
1,962.19	13.48	1,900.00	2,014.00	111

zsibbirth_10_: ZSibBirth[10]

Mean	SD	Min	Max	Obs
1,964.89	15.21	1,930.00	2,015.00	47

zsibbirth_11_: ZSibBirth[11]

Mean	SD	Min	Max	Obs
1,963.55	18.29	1,900.00	2,014.00	31

zsibbirth_12_: ZSibBirth[12]

Mean	SD	Min	Max	Obs
1,965.54	23.89	1,900.00	2,014.00	13

zsibbirth_13_: ZSibBirth[13]

Mean	SD	Min	Max	Obs
1,977.60	20.53	1,966.00	2,014.00	5

zsibbirth_14_: ZSibBirth[14]

Mean	SD	Min	Max	Obs
1,933.00	46.67	1,900.00	1,966.00	2

zsibbirth_s_1_: ZSibBirth_S[1]

Mean	SD	Min	Max	Obs
1,952.38	11.88	1,900.00	2,015.00	7,209

zsibbirth_s_2_: ZSibBirth_S[2]

Mean	SD	Min	Max	Obs
1,956.61	11.73	1,854.00	2,015.00	6,628

zsibbirth_s_3_: ZSibBirth_S[3]

Mean	SD	Min	Max	Obs
1,959.66	11.48	1,871.00	2,015.00	5,566

zsibbirth_s_4_: ZSibBirth_S[4]

Mean	SD	Min	Max	Obs
1,961.74	11.16	1,900.00	2,015.00	3,967

zsibbirth_s_5_: ZSibBirth_S[5]

Mean	SD	Min	Max	Obs
1,963.05	10.52	1,900.00	2,015.00	2,426

zsibbirth_s_6_: ZSibBirth_S[6]

Mean	SD	Min	Max	Obs
1,963.74	10.94	1,900.00	2,014.00	1,243

zsibbirth_s_7_: ZSibBirth_S[7]

Mean	SD	Min	Max	Obs
1,965.31	10.20	1,900.00	2,014.00	571

zsibbirth_s_8_: ZSibBirth_S[8]

Mean	SD	Min	Max	Obs
1,965.83	10.57	1,900.00	2,014.00	215

zsibbirth_s_9_: ZSibBirth_S[9]

Mean	SD	Min	Max	Obs
1,964.12	13.84	1,900.00	1,990.00	78

zsibbirth_s_10_: ZSibBirth_S[10]

Mean	SD	Min	Max	Obs
1,962.55	19.19	1,900.00	1,983.00	20

zsibbirth_s_11_: ZSibBirth_S[11]

Mean	SD	Min	Max	Obs
1,960.63	17.65	1,934.00	1,980.00	8

zsibbirth_s_12_: ZSibBirth_S[12]

Mean	SD	Min	Max	Obs
1,958.00	19.05	1,936.00	1,969.00	3

zsibbirth_s_13_: ZSibBirth_S[13]

Mean	SD	Min	Max	Obs
1,960.00	19.08	1,938.00	1,972.00	3

zsibbirth_s_14_: ZSibBirth_S[14]

Mean	SD	Min	Max	Obs
1,962.67	19.86	1,940.00	1,977.00	3

zsibbirth_s_15_: ZSibBirth_S[15]

Mean	SD	Min	Max	Obs
1,957.00	21.21	1,942.00	1,972.00	2

zsibalive_1_: ZSibAlive[1]

	Freq.	%
0 Dead	1,916	17.62
1 Alive	8,958	82.38
Total	10,874	100.00

zsibalive_2_: ZSibAlive[2]

	Freq.	%
0 Dead	1,322	13.11
1 Alive	8,764	86.89
Total	10,086	100.00

zsibalive_3_: ZSibAlive[3]

	Freq.	%
0 Dead	879	10.41
1 Alive	7,568	89.59
Total	8,447	100.00

zsibalive_4_: ZSibAlive[4]

	Freq.	%
0 Dead	544	8.75
1 Alive	5,671	91.25
Total	6,215	100.00

zsibalive_5_: ZSibAlive[5]

	Freq.	%
0 Dead	304	7.74
1 Alive	3,626	92.26
Total	3,930	100.00

zsibalive_6_: ZSibAlive[6]

	Freq.	%
0 Dead	141	7.04
1 Alive	1,861	92.96
Total	2,002	100.00

zsibalive_7_: ZSibAlive[7]

	Freq.	%
0 Dead	52	5.88
1 Alive	833	94.12
Total	885	100.00

zsibalive_8_: ZSibAlive[8]

	Freq.	%
0 Dead	17	4.89
1 Alive	331	95.11
Total	348	100.00

zsibalive_9_: ZSibAlive[9]

	Freq.	%
0 Dead	8	5.37
1 Alive	141	94.63
Total	149	100.00

zsibalive_10_: ZSibAlive[10]

	Freq.	%
0 Dead	1	1.56
1 Alive	63	98.44
Total	64	100.00

zsibalive_11_: ZSibAlive[11]

	Freq.	%
0 Dead	2	5.56
1 Alive	34	94.44
Total	36	100.00

zsibalive_12_: ZSibAlive[12]

	Freq.	%
1 Alive	17	100.00
Total	17	100.00

zsibalive_13_: ZSibAlive[13]

	Freq.	%
1 Alive	7	100.00
Total	7	100.00

zsibalive_14_: ZSibAlive[14]

	Freq.	%
1 Alive	4	100.00
Total	4	100.00

zsibalive_s_1_: ZSibAlive_S[1]

	Freq.	%
0 Dead	1,159	14.57
1 Alive	6,797	85.43
Total	7,956	100.00

zsibalive_s_2_: ZSibAlive_S[2]

	Freq.	%
0 Dead	762	10.28
1 Alive	6,653	89.72
Total	7,415	100.00

zsibalive_s_3_: ZSibAlive_S[3]

	Freq.	%
0 Dead	527	8.43
1 Alive	5,725	91.57
Total	6,252	100.00

zsibalive_s_4_: ZSibAlive_S[4]

	Freq.	%
0 Dead	324	7.19
1 Alive	4,185	92.81
Total	4,509	100.00

zsibalive_s_5_: ZSibAlive_S[5]

	Freq.	%
0 Dead	175	6.23
1 Alive	2,633	93.77
Total	2,808	100.00

zsibalive_s_6_: ZSibAlive_S[6]

	Freq.	%
0 Dead	101	6.98
1 Alive	1,346	93.02
Total	1,447	100.00

zsibalive_s_7_: ZSibAlive_S[7]

	Freq.	%
0 Dead	32	4.67
1 Alive	653	95.33
Total	685	100.00

zsibalive_s_8_: ZSibAlive_S[8]

	Freq.	%
0 Dead	13	4.71
1 Alive	263	95.29
Total	276	100.00

zsibalive_s_9_: ZSibAlive_S[9]

	Freq.	%
0 Dead	9	8.82
1 Alive	93	91.18
Total	102	100.00

zsibalive_s_10_: ZSibAlive_S[10]

	Freq.	%
0 Dead	2	6.25
1 Alive	30	93.75
Total	32	100.00

zsibalive_s_11_: ZSibAlive_S[11]

	Freq.	%
1 Alive	12	100.00
Total	12	100.00

zsibalive_s_12_: ZSibAlive_S[12]

	Freq.	%
1 Alive	7	100.00
Total	7	100.00

zsibalive_s_13_: ZSibAlive_S[13]

	Freq.	%
1 Alive	3	100.00
Total	3	100.00

zsibalive_s_14_: ZSibAlive_S[14]

	Freq.	%
1 Alive	3	100.00
Total	3	100.00

zsibalive_s_15_: ZSibAlive_S[15]

	Freq.	%
1 Alive	2	100.00
Total	2	100.00

zsibtype_1_: ZSibType[1]

	Freq.	%
1 The Same Parents	10,262	95.51
2 The Same Father but Diff. Mothers	201	1.87
3 The Same Mother but Diff. Fathers	168	1.56
4 Dff. Fathers and Diff. Mothers	113	1.05
Total	10,744	100.00

zsibtype_2_: ZSibType[2]

	Freq.	%

	Freq.	%
1 The Same Parents	9,568	96.15
2 The Same Father but Diff. Mothers	142	1.43
3 The Same Mother but Diff. Fathers	168	1.69
4 Dff. Fathers and Diff. Mothers	73	0.73
Total	9,951	100.00

zsibtype_3_: ZSibType[3]

	Freq.	%
1 The Same Parents	7,989	96.07
2 The Same Father but Diff. Mothers	116	1.39
3 The Same Mother but Diff. Fathers	153	1.84
4 Dff. Fathers and Diff. Mothers	58	0.70
Total	8,316	100.00

zsibtype_4_: ZSibType[4]

	Freq.	%
1 The Same Parents	5,863	96.15
2 The Same Father but Diff. Mothers	75	1.23
3 The Same Mother but Diff. Fathers	106	1.74
4 Dff. Fathers and Diff. Mothers	54	0.89
Total	6,098	100.00

zsibtype_5_: ZSibType[5]

	Freq.	%
1 The Same Parents	3,674	95.48
2 The Same Father but Diff. Mothers	54	1.40
3 The Same Mother but Diff. Fathers	76	1.98
4 Dff. Fathers and Diff. Mothers	44	1.14
Total	3,848	100.00

zsibtype_6_: ZSibType[6]

	Freq.	%
1 The Same Parents	1,844	94.52
2 The Same Father but Diff. Mothers	37	1.90
3 The Same Mother but Diff. Fathers	41	2.10
4 Dff. Fathers and Diff. Mothers	29	1.49
Total	1,951	100.00

zsibtype_7_: ZSibType[7]

	Freq.	%
1 The Same Parents	791	92.95
2 The Same Father but Diff. Mothers	22	2.59
3 The Same Mother but Diff. Fathers	19	2.23
4 Dff. Fathers and Diff. Mothers	19	2.23
Total	851	100.00

zsibtype_8_: ZSibType[8]

	Freq.	%
1 The Same Parents	295	90.77
2 The Same Father but Diff. Mothers	13	4.00
3 The Same Mother but Diff. Fathers	5	1.54
4 Dff. Fathers and Diff. Mothers	12	3.69
Total	325	100.00

zsibtype_9_: ZSibType[9]

	Freq.	%
1 The Same Parents	118	88.06
2 The Same Father but Diff. Mothers	7	5.22
3 The Same Mother but Diff. Fathers	1	0.75
4 Dff. Fathers and Diff. Mothers	8	5.97
Total	134	100.00

zsibtype_10_: ZSibType[10]

	Freq.	%
1 The Same Parents	47	82.46
2 The Same Father but Diff. Mothers	3	5.26
3 The Same Mother but Diff. Fathers	2	3.51
4 Dff. Fathers and Diff. Mothers	5	8.77
Total	57	100.00

zsibtype_11_: ZSibType[11]

	Freq.	%
1 The Same Parents	29	85.29
2 The Same Father but Diff. Mothers	3	8.82
4 Dff. Fathers and Diff. Mothers	2	5.88
Total	34	100.00

zsibtype_12_: ZSibType[12]

	Freq.	%
1 The Same Parents	13	86.67
2 The Same Father but Diff. Mothers	1	6.67
4 Dff. Fathers and Diff. Mothers	1	6.67
Total	15	100.00

zsibtype_13_: ZSibType[13]

	Freq.	%
1 The Same Parents	5	83.33
4 Dff. Fathers and Diff. Mothers	1	16.67
Total	6	100.00

zsibtype_14_: ZSibType[14]

	Freq.	%
1 The Same Parents	3	100.00
Total	3	100.00

zsibtype_s_1_: ZSibType_S[1]

	Freq.	%
1 The Same Parents	7,490	95.30
2 The Same Father but Diff. Mothers	139	1.77
3 The Same Mother but Diff. Fathers	150	1.91
4 Dff. Fathers and Diff. Mothers	80	1.02
Total	7,859	100.00

zsibtype_s_2_: ZSibType_S[2]

	Freq.	%
1 The Same Parents	7,056	96.59
2 The Same Father but Diff. Mothers	87	1.19
3 The Same Mother but Diff. Fathers	126	1.72
4 Dff. Fathers and Diff. Mothers	36	0.49
Total	7,305	100.00

zsibtype_s_3_: ZSibType_S[3]

	Freq.	%
1 The Same Parents	5,932	96.28
2 The Same Father but Diff. Mothers	87	1.41
3 The Same Mother but Diff. Fathers	108	1.75
4 Dff. Fathers and Diff. Mothers	34	0.55
Total	6,161	100.00

zsibtype_s_4_: ZSibType_S[4]

	Freq.	%
1 The Same Parents	4,242	95.95
2 The Same Father but Diff. Mothers	65	1.47
3 The Same Mother but Diff. Fathers	89	2.01
4 Dff. Fathers and Diff. Mothers	25	0.57
Total	4,421	100.00

zsibtype_s_5_: ZSibType_S[5]

	Freq.	%
1 The Same Parents	2,643	95.83
2 The Same Father but Diff. Mothers	43	1.56
3 The Same Mother but Diff. Fathers	54	1.96
4 Dff. Fathers and Diff. Mothers	18	0.65
Total	2,758	100.00

zsibtype_s_6_: ZSibType_S[6]

	Freq.	%
1 The Same Parents	1,341	94.84
2 The Same Father but Diff. Mothers	23	1.63
3 The Same Mother but Diff. Fathers	29	2.05
4 Dff. Fathers and Diff. Mothers	21	1.49
Total	1,414	100.00

zsibtype_s_7_: ZSibType_S[7]

	Freq.	%
1 The Same Parents	615	93.47
2 The Same Father but Diff. Mothers	13	1.98
3 The Same Mother but Diff. Fathers	14	2.13
4 Dff. Fathers and Diff. Mothers	16	2.43
Total	658	100.00

zsibtype_s_8_: ZSibType_S[8]

	Freq.	%
1 The Same Parents	230	88.80
2 The Same Father but Diff. Mothers	12	4.63
3 The Same Mother but Diff. Fathers	5	1.93
4 Dff. Fathers and Diff. Mothers	12	4.63
Total	259	100.00

zsibtype_s_9_: ZSibType_S[9]

	Freq.	%
1 The Same Parents	78	85.71
2 The Same Father but Diff. Mothers	6	6.59
3 The Same Mother but Diff. Fathers	1	1.10
4 Dff. Fathers and Diff. Mothers	6	6.59
Total	91	100.00

zsibtype_s_10_: ZSibType_S[10]

	Freq.	%
1 The Same Parents	23	88.46
2 The Same Father but Diff. Mothers	2	7.69
4 Dff. Fathers and Diff. Mothers	1	3.85
Total	26	100.00

zsibtype_s_11_: ZSibType_S[11]

	Freq.	%
1 The Same Parents	9	81.82
2 The Same Father but Diff. Mothers	2	18.18
Total	11	100.00

zsibtype_s_12_: ZSibType_S[12]

	Freq.	%
1 The Same Parents	5	71.43
2 The Same Father but Diff. Mothers	2	28.57
Total	7	100.00

zsibtype_s_13_: ZSibType_S[13]

	Freq.	%
1 The Same Parents	2	66.67
2 The Same Father but Diff. Mothers	1	33.33
Total	3	100.00

zsibtype_s_14_: ZSibType_S[14]

	Freq.	%
1 The Same Parents	2	66.67
2 The Same Father but Diff. Mothers	1	33.33
Total	3	100.00

zsibtype_s_15_: ZSibType_S[15]

	Freq.	%
1 The Same Parents	1	50.00
2 The Same Father but Diff. Mothers	1	50.00
Total	2	100.00

zsibnum: ZSibNum

Mean	SD	Min	Max	Obs
3.71	2.00	0.00	14.00	11,624

zsibnum_s: ZSibNum_S

Mean	SD	Min	Max	Obs
2.71	2.36	0.00	15.00	11,618

zsibedu_1_: ZSibEdu[1]

	Freq.	%
1 No Formal Education(Illiterate)	1,705	22.19
2 Did not Finish Primary School	1,234	16.06
3 Sishu/Home School	17	0.22
4 Elementary School	1,815	23.62
5 Middle School	1,743	22.68
6 High School	754	9.81
7 Vocational School	150	1.95
8 Two-/Three-Year College/Associate Degree	108	1.41

	Freq.	%
9 Four-Year College/Bachelor's Degree	142	1.85
10 Post-graduate, Master's Degree	8	0.10
11 Post-graduate, Doctoral Degree/Ph.D	9	0.12
Total	7,685	100.00

zsibedu_2_: ZSibEdu[2]

	Freq.	%
1 No Formal Education(Illiterate)	1,504	19.66
2 Did not Finish Primary School	1,220	15.95
3 Sishu/Home School	8	0.10
4 Elementary School	1,906	24.92
5 Middle School	1,918	25.08
6 High School	711	9.30
7 Vocational School	143	1.87
8 Two-/Three-Year College/Associate Degree	111	1.45
9 Four-Year College/Bachelor's Degree	113	1.48
10 Post-graduate, Master's Degree	9	0.12
11 Post-graduate, Doctoral Degree/Ph.D	6	0.08
Total	7,649	100.00

zsibedu_3_: ZSibEdu[3]

	Freq.	%
1 No Formal Education(Illiterate)	1,102	16.56
2 Did not Finish Primary School	1,022	15.35
3 Sishu/Home School	10	0.15
4 Elementary School	1,700	25.54
5 Middle School	1,881	28.26
6 High School	630	9.47
7 Vocational School	108	1.62
8 Two-/Three-Year College/Associate Degree	92	1.38
9 Four-Year College/Bachelor's Degree	101	1.52
10 Post-graduate, Master's Degree	7	0.11
11 Post-graduate, Doctoral Degree/Ph.D	3	0.05
Total	6,656	100.00

zsibedu_4_: ZSibEdu[4]

	Freq.	%
1 No Formal Education(Illiterate)	775	15.51
2 Did not Finish Primary School	762	15.25
3 Sishu/Home School	5	0.10
4 Elementary School	1,251	25.03
5 Middle School	1,490	29.81
6 High School	497	9.94
7 Vocational School	62	1.24
8 Two-/Three-Year College/Associate Degree	66	1.32
9 Four-Year College/Bachelor's Degree	84	1.68
10 Post-graduate, Master's Degree	3	0.06
11 Post-graduate, Doctoral Degree/Ph.D	3	0.06
Total	4,998	100.00

zsibedu_5_: ZSibEdu[5]

	Freq.	%
1 No Formal Education(Illiterate)	481	15.14
2 Did not Finish Primary School	477	15.02
3 Sishu/Home School	2	0.06
4 Elementary School	857	26.98
5 Middle School	929	29.25
6 High School	298	9.38
7 Vocational School	45	1.42
8 Two-/Three-Year College/Associate Degree	31	0.98
9 Four-Year College/Bachelor's Degree	51	1.61
10 Post-graduate, Master's Degree	1	0.03
11 Post-graduate, Doctoral Degree/Ph.D	4	0.13
Total	3,176	100.00

zsibedu_6_: ZSibEdu[6]

	Freq.	%
1 No Formal Education(Illiterate)	241	14.86
2 Did not Finish Primary School	249	15.35
4 Elementary School	437	26.94
5 Middle School	477	29.41
6 High School	152	9.37
7 Vocational School	24	1.48
8 Two-/Three-Year College/Associate Degree	20	1.23
9 Four-Year College/Bachelor's Degree	20	1.23
10 Post-graduate, Master's Degree	2	0.12
Total	1,622	100.00

zsibedu_7_: ZSibEdu[7]

	Freq.	%
1 No Formal Education(Illiterate)	116	16.62
2 Did not Finish Primary School	89	12.75
3 Sishu/Home School	2	0.29
4 Elementary School	180	25.79
5 Middle School	212	30.37
6 High School	67	9.60
7 Vocational School	11	1.58
8 Two-/Three-Year College/Associate Degree	9	1.29
9 Four-Year College/Bachelor's Degree	12	1.72
Total	698	100.00

zsibedu_8_: ZSibEdu[8]

	Freq.	%
1 No Formal Education(Illiterate)	52	20.47
2 Did not Finish Primary School	33	12.99
3 Sishu/Home School	2	0.79
4 Elementary School	59	23.23
5 Middle School	68	26.77
6 High School	29	11.42
7 Vocational School	3	1.18
8 Two-/Three-Year College/Associate Degree	5	1.97
9 Four-Year College/Bachelor's Degree	3	1.18

Total	254	100.00

zsibedu_9_: ZSibEdu[9]

	Freq.	%
1 No Formal Education(Illiterate)	21	20.79
2 Did not Finish Primary School	11	10.89
3 Sishu/Home School	2	1.98
4 Elementary School	29	28.71
5 Middle School	23	22.77
6 High School	11	10.89
7 Vocational School	2	1.98
9 Four-Year College/Bachelor's Degree	2	1.98
Total	101	100.00

zsibedu_10_: ZSibEdu[10]

	Freq.	%
1 No Formal Education(Illiterate)	3	7.32
2 Did not Finish Primary School	4	9.76
4 Elementary School	15	36.59
5 Middle School	12	29.27
6 High School	6	14.63
8 Two-/Three-Year College/Associate Degree	1	2.44
Total	41	100.00

zsibedu_11_: ZSibEdu[11]

	Freq.	%
1 No Formal Education(Illiterate)	2	10.00
2 Did not Finish Primary School	3	15.00
4 Elementary School	7	35.00
5 Middle School	6	30.00
6 High School	2	10.00
Total	20	100.00

zsibedu_12_: ZSibEdu[12]

	Freq.	%
4 Elementary School	3	33.33
5 Middle School	3	33.33
6 High School	3	33.33
Total	9	100.00

zsibedu_13_: ZSibEdu[13]

	Freq.	%
1 No Formal Education(Illiterate)	1	25.00
4 Elementary School	1	25.00
7 Vocational School	1	25.00
9 Four-Year College/Bachelor's Degree	1	25.00
Total	4	100.00

zsibedu_14_: ZSibEdu[14]

	Freq.	%
4 Elementary School	1	50.00
6 High School	1	50.00
Total	2	100.00

zsibedu_s_1_: ZSibEdu_S[1]

	Freq.	%
1 No Formal Education(Illiterate)	1,263	21.65
2 Did not Finish Primary School	867	14.86
3 Sishu/Home School	11	0.19
4 Elementary School	1,415	24.25
5 Middle School	1,411	24.18
6 High School	585	10.03
7 Vocational School	115	1.97
8 Two-/Three-Year College/Associate Degree	69	1.18
9 Four-Year College/Bachelor's Degree	90	1.54
10 Post-graduate, Master's Degree	7	0.12
11 Post-graduate, Doctoral Degree/Ph.D	2	0.03
Total	5,835	100.00

zsibedu_s_2_: ZSibEdu_S[2]

	Freq.	%
1 No Formal Education(Illiterate)	1,081	18.34
2 Did not Finish Primary School	877	14.88
3 Sishu/Home School	6	0.10
4 Elementary School	1,488	25.24
5 Middle School	1,614	27.38
6 High School	577	9.79
7 Vocational School	91	1.54
8 Two-/Three-Year College/Associate Degree	63	1.07
9 Four-Year College/Bachelor's Degree	92	1.56
10 Post-graduate, Master's Degree	5	0.08
11 Post-graduate, Doctoral Degree/Ph.D	1	0.02
Total	5,895	100.00

zsibedu_s_3_: ZSibEdu_S[3]

	Freq.	%
1 No Formal Education(Illiterate)	820	16.01
2 Did not Finish Primary School	748	14.60
3 Sishu/Home School	2	0.04
4 Elementary School	1,288	25.15
5 Middle School	1,560	30.46
6 High School	521	10.17
7 Vocational School	75	1.46
8 Two-/Three-Year College/Associate Degree	42	0.82
9 Four-Year College/Bachelor's Degree	57	1.11
10 Post-graduate, Master's Degree	8	0.16
11 Post-graduate, Doctoral Degree/Ph.D	1	0.02
Total	5,122	100.00

zsibedu_s_4_: ZSibEdu_S[4]

	Freq.	%
1 No Formal Education(Illiterate)	527	14.11
2 Did not Finish Primary School	532	14.25
4 Elementary School	999	26.75
5 Middle School	1,162	31.12
6 High School	361	9.67
7 Vocational School	62	1.66
8 Two-/Three-Year College/Associate Degree	40	1.07
9 Four-Year College/Bachelor's Degree	49	1.31
10 Post-graduate, Master's Degree	2	0.05
Total	3,734	100.00

zsibedu_s_5_: ZSibEdu_S[5]

	Freq.	%
1 No Formal Education(Illiterate)	320	13.69
2 Did not Finish Primary School	340	14.55
3 Sishu/Home School	1	0.04
4 Elementary School	664	28.41
5 Middle School	705	30.17
6 High School	218	9.33
7 Vocational School	38	1.63
8 Two-/Three-Year College/Associate Degree	21	0.90
9 Four-Year College/Bachelor's Degree	28	1.20
10 Post-graduate, Master's Degree	1	0.04
11 Post-graduate, Doctoral Degree/Ph.D	1	0.04
Total	2,337	100.00

zsibedu_s_6_: ZSibEdu_S[6]

	Freq.	%
1 No Formal Education(Illiterate)	155	13.12
2 Did not Finish Primary School	150	12.70
4 Elementary School	348	29.47
5 Middle School	383	32.43
6 High School	108	9.14
7 Vocational School	15	1.27
8 Two-/Three-Year College/Associate Degree	9	0.76
9 Four-Year College/Bachelor's Degree	12	1.02
10 Post-graduate, Master's Degree	1	0.08
Total	1,181	100.00

zsibedu_s_7_: ZSibEdu_S[7]

	Freq.	%
1 No Formal Education(Illiterate)	75	13.35
2 Did not Finish Primary School	70	12.46
4 Elementary School	162	28.83
5 Middle School	182	32.38
6 High School	51	9.07
7 Vocational School	8	1.42
8 Two-/Three-Year College/Associate Degree	5	0.89

	Freq.	%
9 Four-Year College/Bachelor's Degree	8	1.42
10 Post-graduate, Master's Degree	1	0.18
Total	562	100.00

zsibedu_s_8_: ZSibEdu_S[8]

	Freq.	%
1 No Formal Education(Illiterate)	31	14.03
2 Did not Finish Primary School	28	12.67
4 Elementary School	72	32.58
5 Middle School	70	31.67
6 High School	14	6.33
7 Vocational School	2	0.90
8 Two-/Three-Year College/Associate Degree	2	0.90
9 Four-Year College/Bachelor's Degree	2	0.90
Total	221	100.00

zsibedu_s_9_: ZSibEdu_S[9]

	Freq.	%
1 No Formal Education(Illiterate)	3	4.23
2 Did not Finish Primary School	14	19.72
4 Elementary School	24	33.80
5 Middle School	18	25.35
6 High School	8	11.27
7 Vocational School	1	1.41
8 Two-/Three-Year College/Associate Degree	2	2.82
11 Post-graduate, Doctoral Degree/Ph.D	1	1.41
Total	71	100.00

zsibedu_s_10_: ZSibEdu_S[10]

	Freq.	%
1 No Formal Education(Illiterate)	3	14.29
2 Did not Finish Primary School	3	14.29
4 Elementary School	10	47.62
5 Middle School	4	19.05
8 Two-/Three-Year College/Associate Degree	1	4.76
Total	21	100.00

zsibedu_s_11_: ZSibEdu_S[11]

	Freq.	%
1 No Formal Education(Illiterate)	2	22.22
4 Elementary School	2	22.22
5 Middle School	4	44.44
8 Two-/Three-Year College/Associate Degree	1	11.11
Total	9	100.00

zsibedu_s_12_: ZSibEdu_S[12]

	Freq.	%

	Freq.	%
1 No Formal Education(Illiterate)	1	20.00
2 Did not Finish Primary School	1	20.00
4 Elementary School	1	20.00
5 Middle School	1	20.00
6 High School	1	20.00
Total	5	100.00

zsibedu_s_13_: ZSibEdu_S[13]

	Freq.	%
1 No Formal Education(Illiterate)	1	50.00
5 Middle School	1	50.00
Total	2	100.00

zsibedu_s_14_: ZSibEdu_S[14]

	Freq.	%
5 Middle School	1	100.00
Total	1	100.00

hhmembernumber: HHMemberNumber

Mean	SD	Min	Max	Obs
0.99	1.43	0.00	12.00	11,598

versionID: Version ID

A String Variable	
Obs:	11,628

MODULE **C2**

Family Transfer

ID: Individual ID

A String Variable	
Obs:	11,568

householdID: Household ID

A String Variable	
Obs:	11,568

communityID: Community ID

A String Variable	
Obs:	11,568

cd001_w4_1_: Who Live with XConParName[1]

	Freq.	%
1 Alone	523	36.37
2 His/Her Spouse	255	17.73
3 Respondents	129	8.97
4 Siblings	477	33.17
6 Children	5	0.35
7 Siblings' Children	25	1.74
9 Other Relatives	4	0.28
10 Nursing home	11	0.76
11 Others	9	0.63
Total	1,438	100.00

cd001_w4_2_: Who Live with XConParName[2]

	Freq.	%
1 Alone	544	31.35

	Freq.	%
2 His/Her Spouse	35	2.02
3 Respondents	274	15.79
4 Siblings	796	45.88
5 Spouse's Siblings	1	0.06
6 Children	10	0.58
7 Siblings' Children	36	2.07
9 Other Relatives	14	0.81
10 Nursing home	18	1.04
11 Others	7	0.40
Total	1,735	100.00

cd001_w4_3_: Who Live with XConParName[3]

	Freq.	%
1 Alone	15	37.50
2 His/Her Spouse	11	27.50
3 Respondents	4	10.00
4 Siblings	7	17.50
7 Siblings' Children	1	2.50
9 Other Relatives	1	2.50
11 Others	1	2.50
Total	40	100.00

cd001_w4_4_: Who Live with XConParName[4]

	Freq.	%
1 Alone	10	20.83
2 His/Her Spouse	11	22.92
3 Respondents	4	8.33
4 Siblings	17	35.42
6 Children	1	2.08
7 Siblings' Children	2	4.17
9 Other Relatives	1	2.08
10 Nursing home	1	2.08
11 Others	1	2.08
Total	48	100.00

cd001_w4_5_: Who Live with XConParName[5]

	Freq.	%
1 Alone	580	41.40
2 His/Her Spouse	258	18.42
3 Respondents	124	8.85
4 Siblings	1	0.07
5 Spouse's Siblings	397	28.34
6 Children	1	0.07
8 Siblings' Children of Spouse	22	1.57
9 Other Relatives	3	0.21
10 Nursing home	10	0.71
11 Others	5	0.36
Total	1,401	100.00

cd001_w4_6_: Who Live with XConParName[6]

	Freq.	%
1 Alone	468	32.93
2 His/Her Spouse	24	1.69
3 Respondents	181	12.74
4 Siblings	5	0.35
5 Spouse's Siblings	663	46.66
6 Children	2	0.14
8 Siblings' Children of Spouse	50	3.52
9 Other Relatives	7	0.49
10 Nursing home	13	0.91
11 Others	8	0.56
Total	1,421	100.00

cd001_w4_7_: Who Live with XConParName[7]

	Freq.	%
1 Alone	5	18.52
2 His/Her Spouse	11	40.74
3 Respondents	2	7.41
5 Spouse's Siblings	7	25.93
9 Other Relatives	1	3.70
11 Others	1	3.70
Total	27	100.00

cd001_w4_8_: Who Live with XConParName[8]

	Freq.	%
1 Alone	6	21.43
2 His/Her Spouse	4	14.29
3 Respondents	4	14.29
5 Spouse's Siblings	12	42.86
9 Other Relatives	1	3.57
11 Others	1	3.57
Total	28	100.00

cd001_w3_1_1__s1: Member[1] of Respondent's Siblings[1]

	Freq.	%
0 No	343	71.91
1 Yes	134	28.09
Total	477	100.00

cd001_w3_1_1__s2: Member[2] of Respondent's Siblings[1]

	Freq.	%
0 No	344	72.12
2 Yes	133	27.88
Total	477	100.00

cd001_w3_1_1__s3: Member[3] of Respondent's Siblings[1]

	Freq.	%
0 No	354	74.21
3 Yes	123	25.79
Total	477	100.00

cd001_w3_1_1__s4: Member[4] of Respondent's Siblings[1]

	Freq.	%
0 No	400	83.86
4 Yes	77	16.14
Total	477	100.00

cd001_w3_1_1__s5: Member[5] of Respondent's Siblings[1]

	Freq.	%
0 No	432	90.57
5 Yes	45	9.43
Total	477	100.00

cd001_w3_1_1__s6: Member[6] of Respondent's Siblings[1]

	Freq.	%
0 No	460	96.44
6 Yes	17	3.56
Total	477	100.00

cd001_w3_1_1__s7: Member[7] of Respondent's Siblings[1]

	Freq.	%
0 No	473	99.16
7 Yes	4	0.84
Total	477	100.00

cd001_w3_1_1__s8: Member[8] of Respondent's Siblings[1]

	Freq.	%
0 No	471	98.74
8 Yes	6	1.26
Total	477	100.00

cd001_w3_1_1__s9: Member[9] of Respondent's Siblings[1]

	Freq.	%
0 No	475	99.58
9 Yes	2	0.42
Total	477	100.00

cd001_w3_1_1__s10: Member[10] of Respondent's Siblings[1]

	Freq.	%
0 No	476	99.79
10 Yes	1	0.21
Total	477	100.00

cd001_w3_1_1__s11: Member[11] of Respondent's Siblings[1]

	Freq.	%
0 No	476	99.79
11 Yes	1	0.21
Total	477	100.00

cd001_w3_1_2__s1: Member[1] of Respondent's Siblings[2]

	Freq.	%
0 No	601	76.56
1 Yes	184	23.44
Total	785	100.00

cd001_w3_1_2__s2: Member[2] of Respondent's Siblings[2]

	Freq.	%
0 No	566	72.10
2 Yes	219	27.90
Total	785	100.00

cd001_w3_1_2__s3: Member[3] of Respondent's Siblings[2]

	Freq.	%
0 No	601	76.56
3 Yes	184	23.44
Total	785	100.00

cd001_w3_1_2__s4: Member[4] of Respondent's Siblings[2]

	Freq.	%
0 No	653	83.18
4 Yes	132	16.82
Total	785	100.00

cd001_w3_1_2__s5: Member[5] of Respondent's Siblings[2]

	Freq.	%
0 No	673	85.73
5 Yes	112	14.27
Total	785	100.00

cd001_w3_1_2__s6: Member[6] of Respondent's Siblings[2]

	Freq.	%
0 No	733	93.38
6 Yes	52	6.62
Total	785	100.00

cd001_w3_1_2__s7: Member[7] of Respondent's Siblings[2]

	Freq.	%
0 No	767	97.71
7 Yes	18	2.29
Total	785	100.00

cd001_w3_1_2__s8: Member[8] of Respondent's Siblings[2]

	Freq.	%
0 No	781	99.49
8 Yes	4	0.51
Total	785	100.00

cd001_w3_1_2__s11: Member[11] of Respondent's Siblings[2]

	Freq.	%
0 No	784	99.87
11 Yes	1	0.13
Total	785	100.00

cd001_w3_1_3_: Respondent's Siblings[3]

Mean	SD	Min	Max	Obs
2.57	1.72	1.00	6.00	7

cd001_w3_1_4_: Respondent's Siblings[4]

Mean	SD	Min	Max	Obs
3.00	1.73	1.00	7.00	13

cd001_w3_1_5_: Respondent's Siblings[5]

Mean	SD	Min	Max	Obs
1.00	.	1.00	1.00	1

cd001_w3_1_6_: Respondent's Siblings[6]

Mean	SD	Min	Max	Obs
2.80	1.30	1.00	4.00	5

cd001_w3_2_2_: RSpouse's Siblings[2]

Mean	SD	Min	Max	Obs
1.00	.	1.00	1.00	1

cd001_w3_2_5__s1: Member[1] of RSpouse's Siblings[5]

	Freq.	%
0 No	290	73.98
1 Yes	102	26.02
Total	392	100.00

cd001_w3_2_5__s2: Member[2] of RSpouse's Siblings[5]

	Freq.	%
0 No	258	65.82
2 Yes	134	34.18
Total	392	100.00

cd001_w3_2_5__s3: Member[3] of RSpouse's Siblings[5]

	Freq.	%
0 No	299	76.28
3 Yes	93	23.72
Total	392	100.00

cd001_w3_2_5__s4: Member[4] of RSpouse's Siblings[5]

	Freq.	%
0 No	331	84.44
4 Yes	61	15.56
Total	392	100.00

cd001_w3_2_5__s5: Member[5] of RSpouse's Siblings[5]

	Freq.	%
0 No	370	94.39
5 Yes	22	5.61
Total	392	100.00

cd001_w3_2_5__s6: Member[6] of RSpouse's Siblings[5]

	Freq.	%
0 No	378	96.43
6 Yes	14	3.57
Total	392	100.00

cd001_w3_2_5__s7: Member[7] of RSpouse's Siblings[5]

	Freq.	%
0 No	384	97.96
7 Yes	8	2.04
Total	392	100.00

cd001_w3_2_5__s8: Member[8] of RSpouse's Siblings[5]

	Freq.	%
0 No	390	99.49
8 Yes	2	0.51
Total	392	100.00

cd001_w3_2_6__s1: Member[1] of RSpouse's Siblings[6]

	Freq.	%
0 No	486	75.12
1 Yes	161	24.88
Total	647	100.00

cd001_w3_2_6__s2: Member[2] of RSpouse's Siblings[6]

	Freq.	%
0 No	474	73.26
2 Yes	173	26.74
Total	647	100.00

cd001_w3_2_6__s3: Member[3] of RSpouse's Siblings[6]

	Freq.	%
0 No	481	74.34
3 Yes	166	25.66
Total	647	100.00

cd001_w3_2_6__s4: Member[4] of RSpouse's Siblings[6]

	Freq.	%
0 No	536	82.84
4 Yes	111	17.16
Total	647	100.00

cd001_w3_2_6__s5: Member[5] of RSpouse's Siblings[6]

	Freq.	%
0 No	583	90.11
5 Yes	64	9.89
Total	647	100.00

cd001_w3_2_6__s6: Member[6] of RSpouse's Siblings[6]

	Freq.	%
0 No	613	94.74
6 Yes	34	5.26
Total	647	100.00

cd001_w3_2_6__s7: Member[7] of RSpouse's Siblings[6]

	Freq.	%
0 No	636	98.30
7 Yes	11	1.70
Total	647	100.00

cd001_w3_2_6__s8: Member[8] of RSpouse's Siblings[6]

	Freq.	%
0 No	642	99.23
8 Yes	5	0.77
Total	647	100.00

cd001_w3_2_7__s1: Member[1] of RSpouse's Siblings[7]

	Freq.	%
0 No	3	50.00
1 Yes	3	50.00
Total	6	100.00

cd001_w3_2_7__s2: Member[2] of RSpouse's Siblings[7]

	Freq.	%
0 No	4	66.67
2 Yes	2	33.33
Total	6	100.00

cd001_w3_2_7__s3: Member[3] of RSpouse's Siblings[7]

	Freq.	%
0 No	3	50.00
3 Yes	3	50.00
Total	6	100.00

cd001_w3_2_7__s4: Member[4] of RSpouse's Siblings[7]

	Freq.	%
0 No	5	83.33
4 Yes	1	16.67
Total	6	100.00

cd001_w3_2_8__s1: Member[1] of RSpouse's Siblings[8]

	Freq.	%
0 No	9	81.82
1 Yes	2	18.18
Total	11	100.00

cd001_w3_2_8__s2: Member[2] of RSpouse's Siblings[8]

	Freq.	%
0 No	8	72.73
2 Yes	3	27.27
Total	11	100.00

cd001_w3_2_8__s3: Member[3] of RSpouse's Siblings[8]

	Freq.	%
0 No	7	63.64
3 Yes	4	36.36
Total	11	100.00

cd001_w3_2_8__s4: Member[4] of RSpouse's Siblings[8]

	Freq.	%
0 No	9	81.82
4 Yes	2	18.18
Total	11	100.00

cd001_w3_2_8__s6: Member[6] of RSpouse's Siblings[8]

	Freq.	%
0 No	10	90.91
6 Yes	1	9.09
Total	11	100.00

cd001_w3_3_1__s1: Member[1] of Child Family[1]

	Freq.	%
0 No	3	60.00
1 Yes	2	40.00
Total	5	100.00

cd001_w3_3_1__s2: Member[2] of Child Family[1]

	Freq.	%
0 No	4	80.00
2 Yes	1	20.00
Total	5	100.00

cd001_w3_3_1__s3: Member[3] of Child Family[1]

	Freq.	%
0 No	3	60.00
3 Yes	2	40.00
Total	5	100.00

cd001_w3_3_1__s4: Member[4] of Child Family[1]

	Freq.	%
0 No	4	80.00
4 Yes	1	20.00
Total	5	100.00

cd001_w3_3_2_: Child Family[2]

Mean	SD	Min	Max	Obs
1.40	0.97	1.00	4.00	10

cd001_w3_3_4_: Child Family[4]

No Observations

cd001_w3_3_5_: Child Family[5]

Mean	SD	Min	Max	Obs
1.00	.	1.00	1.00	1

cd001_w3_3_6_: Child Family[6]

Mean	SD	Min	Max	Obs
1.00	.	1.00	1.00	1

cd001_w3_4_1__s1: Member[1] of Child Family[1] of Siblings

	Freq.	%
0 No	16	69.57
1 Yes	7	30.43
Total	23	100.00

cd001_w3_4_1__s2: Member[2] of Child Family[1] of Siblings

	Freq.	%
0 No	15	65.22
2 Yes	8	34.78
Total	23	100.00

cd001_w3_4_1__s3: Member[3] of Child Family[1] of Siblings

	Freq.	%
0 No	21	91.30
3 Yes	2	8.70
Total	23	100.00

cd001_w3_4_1__s4: Member[4] of Child Family[1] of Siblings

	Freq.	%
0 No	19	82.61
4 Yes	4	17.39
Total	23	100.00

cd001_w3_4_1__s5: Member[5] of Child Family[1] of Siblings

	Freq.	%
0 No	21	91.30
5 Yes	2	8.70
Total	23	100.00

cd001_w3_4_1__s6: Member[6] of Child Family[1] of Siblings

	Freq.	%
0 No	21	91.30
6 Yes	2	8.70
Total	23	100.00

cd001_w3_4_2__s1: Member[1] of Child Family[2] of Siblings

	Freq.	%
0 No	20	62.50
1 Yes	12	37.50
Total	32	100.00

cd001_w3_4_2__s2: Member[2] of Child Family[2] of Siblings

	Freq.	%
0 No	23	71.88
2 Yes	9	28.13
Total	32	100.00

cd001_w3_4_2__s3: Member[3] of Child Family[2] of Siblings

	Freq.	%
0 No	26	81.25
3 Yes	6	18.75
Total	32	100.00

cd001_w3_4_2__s4: Member[4] of Child Family[2] of Siblings

	Freq.	%
0 No	28	87.50
4 Yes	4	12.50
Total	32	100.00

cd001_w3_4_2__s5: Member[5] of Child Family[2] of Siblings

	Freq.	%
0 No	30	93.75
5 Yes	2	6.25
Total	32	100.00

cd001_w3_4_2__s6: Member[6] of Child Family[2] of Siblings

	Freq.	%
0 No	30	93.75
6 Yes	2	6.25
Total	32	100.00

cd001_w3_4_2__s7: Member[7] of Child Family[2] of Siblings

	Freq.	%
0 No	31	96.88
7 Yes	1	3.13
Total	32	100.00

cd001_w3_4_3_: Child Family[3] of Siblings

Mean	SD	Min	Max	Obs
2.00	.	2.00	2.00	1

cd001_w3_4_4__s2: Member[2] of Child Family[4] of Siblings

	Freq.	%
2 Yes	1	100.00
Total	1	100.00

cd001_w3_4_4__s3: Member[3] of Child Family[4] of Siblings

	Freq.	%
3 Yes	1	100.00
Total	1	100.00

cd001_w3_4_4__s4: Member[4] of Child Family[4] of Siblings

	Freq.	%
4 Yes	1	100.00

	Freq.	%
Total	1	100.00

cd001_w3_5_5_: Child Family[5] of Spouse' Siblings

Mean	SD	Min	Max	Obs
2.95	1.79	1.00	7.00	20

cd001_w3_5_6__s1: Member[1] of Child Family[6] of Siblings

	Freq.	%
0 No	33	66.00
1 Yes	17	34.00
Total	50	100.00

cd001_w3_5_6__s2: Member[2] of Child Family[6] of Siblings

	Freq.	%
0 No	45	90.00
2 Yes	5	10.00
Total	50	100.00

cd001_w3_5_6__s3: Member[3] of Child Family[6] of Siblings

	Freq.	%
0 No	41	82.00
3 Yes	9	18.00
Total	50	100.00

cd001_w3_5_6__s4: Member[4] of Child Family[6] of Siblings

	Freq.	%
0 No	45	90.00
4 Yes	5	10.00
Total	50	100.00

cd001_w3_5_6__s5: Member[5] of Child Family[6] of Siblings

	Freq.	%
0 No	44	88.00
5 Yes	6	12.00
Total	50	100.00

cd001_w3_5_6__s6: Member[6] of Child Family[6] of Siblings

	Freq.	%
0 No	48	96.00
6 Yes	2	4.00
Total	50	100.00

cd001_w3_5_6__s7: Member[7] of Child Family[6] of Siblings

	Freq.	%
0 No	48	96.00
7 Yes	2	4.00
Total	50	100.00

cd001_w3_5_6__s8: Member[8] of Child Family[6] of Siblings

	Freq.	%
0 No	49	98.00
8 Yes	1	2.00
Total	50	100.00

cd002_w4_1_: Frequency Visiting XConParName[1]

	Freq.	%
1 Almost Every Day	412	28.67
2 2-3 Times a Week	166	11.55
3 Once a Week	165	11.48
4 Every Two Weeks	168	11.69
5 Once a Month	194	13.50
6 Once Every Three Months	115	8.00
7 Once Every Six Months	78	5.43
8 Once a Year	71	4.94
9 Almost Never	31	2.16
10 Other	37	2.57
Total	1,437	100.00

cd002_w4_2_: Frequency Visiting XConParName[2]

	Freq.	%
1 Almost Every Day	602	34.72
2 2-3 Times a Week	162	9.34
3 Once a Week	182	10.50
4 Every Two Weeks	167	9.63
5 Once a Month	226	13.03
6 Once Every Three Months	117	6.75
7 Once Every Six Months	95	5.48
8 Once a Year	90	5.19
9 Almost Never	47	2.71
10 Other	46	2.65
Total	1,734	100.00

cd002_w4_3_: Frequency Visiting XConParName[3]

	Freq.	%
1 Almost Every Day	7	17.95
2 2-3 Times a Week	4	10.26
3 Once a Week	3	7.69
4 Every Two Weeks	5	12.82
5 Once a Month	5	12.82
6 Once Every Three Months	2	5.13

	Freq.	%
7 Once Every Six Months	3	7.69
8 Once a Year	4	10.26
9 Almost Never	4	10.26
10 Other	2	5.13
Total	39	100.00

cd002_w4_4_: Frequency Visiting XConParName[4]

	Freq.	%
1 Almost Every Day	10	20.83
2 2-3 Times a Week	1	2.08
3 Once a Week	1	2.08
4 Every Two Weeks	3	6.25
5 Once a Month	3	6.25
6 Once Every Three Months	8	16.67
7 Once Every Six Months	4	8.33
8 Once a Year	7	14.58
9 Almost Never	7	14.58
10 Other	4	8.33
Total	48	100.00

cd002_w4_5_: Frequency Visiting XConParName[5]

	Freq.	%
1 Almost Every Day	436	31.12
2 2-3 Times a Week	134	9.56
3 Once a Week	172	12.28
4 Every Two Weeks	130	9.28
5 Once a Month	227	16.20
6 Once Every Three Months	88	6.28
7 Once Every Six Months	79	5.64
8 Once a Year	80	5.71
9 Almost Never	26	1.86
10 Other	29	2.07
Total	1,401	100.00

cd002_w4_6_: Frequency Visiting XConParName[6]

	Freq.	%
1 Almost Every Day	446	31.39
2 2-3 Times a Week	109	7.67
3 Once a Week	155	10.91
4 Every Two Weeks	149	10.49
5 Once a Month	203	14.29
6 Once Every Three Months	125	8.80
7 Once Every Six Months	80	5.63
8 Once a Year	70	4.93
9 Almost Never	37	2.60
10 Other	47	3.31
Total	1,421	100.00

cd002_w4_7_: Frequency Visiting XConParName[7]

	Freq.	%

	Freq.	%
1 Almost Every Day	5	18.52
2 2-3 Times a Week	1	3.70
3 Once a Week	2	7.41
4 Every Two Weeks	3	11.11
5 Once a Month	3	11.11
6 Once Every Three Months	6	22.22
7 Once Every Six Months	1	3.70
8 Once a Year	3	11.11
9 Almost Never	1	3.70
10 Other	2	7.41
Total	27	100.00

cd002_w4_8_: Frequency Visiting XConParName[8]

	Freq.	%
1 Almost Every Day	9	32.14
3 Once a Week	1	3.57
4 Every Two Weeks	1	3.57
5 Once a Month	4	14.29
6 Once Every Three Months	1	3.57
7 Once Every Six Months	5	17.86
8 Once a Year	2	7.14
9 Almost Never	4	14.29
10 Other	1	3.57
Total	28	100.00

cd003_1_: Frequency Visiting XChildName[1]

	Freq.	%
1 Almost Every Day	1,167	14.27
2 2-3 Times a Week	444	5.43
3 Once a week	754	9.22
4 Every Two Weeks	632	7.73
5 Once a Month	950	11.62
6 Once Every Three Months	934	11.42
7 Once Every Six Months	1,074	13.13
8 Once a Year	1,413	17.28
9 Almost Never	588	7.19
10 Other	223	2.73
Total	8,179	100.00

cd003_2_: Frequency Visiting XChildName[2]

	Freq.	%
1 Almost Every Day	961	12.78
2 2-3 Times a Week	441	5.86
3 Once a week	733	9.75
4 Every Two Weeks	671	8.92
5 Once a Month	932	12.39
6 Once Every Three Months	837	11.13
7 Once Every Six Months	907	12.06
8 Once a Year	1,308	17.39
9 Almost Never	519	6.90
10 Other	212	2.82
Total	7,521	100.00

cd003_3_: Frequency Visiting XChildName[3]

	Freq.	%
1 Almost Every Day	556	12.25
2 2-3 Times a Week	264	5.81
3 Once a week	445	9.80
4 Every Two Weeks	396	8.72
5 Once a Month	555	12.22
6 Once Every Three Months	502	11.06
7 Once Every Six Months	504	11.10
8 Once a Year	847	18.66
9 Almost Never	317	6.98
10 Other	154	3.39
Total	4,540	100.00

cd003_4_: Frequency Visiting XChildName[4]

	Freq.	%
1 Almost Every Day	272	10.93
2 2-3 Times a Week	152	6.11
3 Once a week	214	8.60
4 Every Two Weeks	212	8.52
5 Once a Month	320	12.86
6 Once Every Three Months	291	11.69
7 Once Every Six Months	277	11.13
8 Once a Year	463	18.60
9 Almost Never	197	7.91
10 Other	91	3.66
Total	2,489	100.00

cd003_5_: Frequency Visiting XChildName[5]

	Freq.	%
1 Almost Every Day	120	9.84
2 2-3 Times a Week	87	7.13
3 Once a week	88	7.21
4 Every Two Weeks	113	9.26
5 Once a Month	169	13.85
6 Once Every Three Months	155	12.70
7 Once Every Six Months	165	13.52
8 Once a Year	193	15.82
9 Almost Never	88	7.21
10 Other	42	3.44
Total	1,220	100.00

cd003_6_: Frequency Visiting XChildName[6]

	Freq.	%
1 Almost Every Day	63	11.54
2 2-3 Times a Week	39	7.14
3 Once a week	35	6.41
4 Every Two Weeks	42	7.69
5 Once a Month	67	12.27
6 Once Every Three Months	64	11.72

	Freq.	%
7 Once Every Six Months	66	12.09
8 Once a Year	99	18.13
9 Almost Never	44	8.06
10 Other	27	4.95
Total	546	100.00

cd003_7_: Frequency Visiting XChildName[7]

	Freq.	%
1 Almost Every Day	22	8.40
2 2-3 Times a Week	10	3.82
3 Once a week	16	6.11
4 Every Two Weeks	23	8.78
5 Once a Month	42	16.03
6 Once Every Three Months	40	15.27
7 Once Every Six Months	30	11.45
8 Once a Year	50	19.08
9 Almost Never	19	7.25
10 Other	10	3.82
Total	262	100.00

cd003_8_: Frequency Visiting XChildName[8]

	Freq.	%
1 Almost Every Day	11	9.32
2 2-3 Times a Week	6	5.08
3 Once a week	14	11.86
4 Every Two Weeks	4	3.39
5 Once a Month	19	16.10
6 Once Every Three Months	11	9.32
7 Once Every Six Months	10	8.47
8 Once a Year	23	19.49
9 Almost Never	15	12.71
10 Other	5	4.24
Total	118	100.00

cd003_9_: Frequency Visiting XChildName[9]

	Freq.	%
1 Almost Every Day	5	9.62
2 2-3 Times a Week	3	5.77
3 Once a week	3	5.77
4 Every Two Weeks	2	3.85
5 Once a Month	6	11.54
6 Once Every Three Months	9	17.31
7 Once Every Six Months	5	9.62
8 Once a Year	11	21.15
9 Almost Never	5	9.62
10 Other	3	5.77
Total	52	100.00

cd003_10_: Frequency Visiting XChildName[10]

	Freq.	%

	Freq.	%
1 Almost Every Day	2	6.67
2 2-3 Times a Week	3	10.00
4 Every Two Weeks	3	10.00
5 Once a Month	2	6.67
6 Once Every Three Months	2	6.67
7 Once Every Six Months	3	10.00
8 Once a Year	9	30.00
9 Almost Never	5	16.67
10 Other	1	3.33
Total	30	100.00

cd003_11_: Frequency Visiting XChildName[11]

	Freq.	%
1 Almost Every Day	1	5.56
2 2-3 Times a Week	1	5.56
3 Once a week	3	16.67
4 Every Two Weeks	2	11.11
5 Once a Month	4	22.22
6 Once Every Three Months	2	11.11
7 Once Every Six Months	1	5.56
9 Almost Never	3	16.67
10 Other	1	5.56
Total	18	100.00

cd003_12_: Frequency Visiting XChildName[12]

	Freq.	%
2 2-3 Times a Week	1	11.11
3 Once a week	1	11.11
7 Once Every Six Months	1	11.11
8 Once a Year	2	22.22
9 Almost Never	4	44.44
Total	9	100.00

cd003_13_: Frequency Visiting XChildName[13]

	Freq.	%
3 Once a week	1	50.00
4 Every Two Weeks	1	50.00
Total	2	100.00

cd003_14_: Frequency Visiting XChildName[14]

	Freq.	%
3 Once a week	1	50.00
5 Once a Month	1	50.00
Total	2	100.00

cd003_15_: Frequency Visiting XChildName[15]

	Freq.	%

8 Once a Year	1	100.00
Total	1	100.00

cd003_w4_1_: How Long Live with XChildName[1]

Mean	SD	Min	Max	Obs
3.62	5.11	0.00	12.00	10,935

cd003_w4_2_: How Long Live with XChildName[2]

Mean	SD	Min	Max	Obs
2.52	4.51	0.00	12.00	9,059

cd003_w4_3_: How Long Live with XChildName[3]

Mean	SD	Min	Max	Obs
1.67	3.84	0.00	12.00	5,103

cd003_w4_4_: How Long Live with XChildName[4]

Mean	SD	Min	Max	Obs
1.29	3.43	0.00	12.00	2,715

cd003_w4_5_: How Long Live with XChildName[5]

Mean	SD	Min	Max	Obs
1.03	3.09	0.00	12.00	1,308

cd003_w4_6_: How Long Live with XChildName[6]

Mean	SD	Min	Max	Obs
0.98	2.97	0.00	12.00	579

cd003_w4_7_: How Long Live with XChildName[7]

Mean	SD	Min	Max	Obs
1.04	3.05	0.00	12.00	279

cd003_w4_8_: How Long Live with XChildName[8]

Mean	SD	Min	Max	Obs
1.19	3.39	0.00	12.00	129

cd003_w4_9_: How Long Live with XChildName[9]

Mean	SD	Min	Max	Obs

0.85	2.84	0.00	12.00	55

cd003_w4_10_: How Long Live with XChildName[10]

Mean	SD	Min	Max	Obs
0.78	2.95	0.00	12.00	32

cd003_w4_11_: How Long Live with XChildName[11]

Mean	SD	Min	Max	Obs
1.00	3.00	0.00	12.00	19

cd003_w4_12_: How Long Live with XChildName[12]

Mean	SD	Min	Max	Obs
1.20	3.79	0.00	12.00	10

cd003_w4_13_: How Long Live with XChildName[13]

Mean	SD	Min	Max	Obs
0.00	0.00	0.00	0.00	2

cd003_w4_14_: How Long Live with XChildName[14]

Mean	SD	Min	Max	Obs
0.00	0.00	0.00	0.00	2

cd003_w4_15_: How Long Live with XChildName[15]

Mean	SD	Min	Max	Obs
0.00	.	0.00	0.00	1

cd004_1_: Frequency Contacting XChildName[1]

	Freq.	%
1 Almost Every Day	852	14.66
2 2-3 Times a Week	1,164	20.02
3 Once a week	1,197	20.59
4 Every Two Weeks	820	14.11
5 Once a Month	709	12.20
6 Once Every Three Months	216	3.72
7 Once Every Six Months	89	1.53
8 Once a Year	51	0.88
9 Almost Never	559	9.62
10 Other	156	2.68
Total	5,813	100.00

cd004_2_: Frequency Contacting XChildName[2]

	Freq.	%
1 Almost Every Day	597	11.09
2 2-3 Times a Week	940	17.46
3 Once a week	1,106	20.54
4 Every Two Weeks	860	15.97
5 Once a Month	738	13.70
6 Once Every Three Months	237	4.40
7 Once Every Six Months	104	1.93
8 Once a Year	35	0.65
9 Almost Never	606	11.25
10 Other	162	3.01
Total	5,385	100.00

cd004_3_: Frequency Contacting XChildName[3]

	Freq.	%
1 Almost Every Day	277	8.46
2 2-3 Times a Week	417	12.73
3 Once a week	594	18.14
4 Every Two Weeks	539	16.46
5 Once a Month	572	17.47
6 Once Every Three Months	157	4.79
7 Once Every Six Months	74	2.26
8 Once a Year	33	1.01
9 Almost Never	481	14.69
10 Other	131	4.00
Total	3,275	100.00

cd004_4_: Frequency Contacting XChildName[4]

	Freq.	%
1 Almost Every Day	93	5.02
2 2-3 Times a Week	202	10.91
3 Once a week	303	16.37
4 Every Two Weeks	297	16.05
5 Once a Month	309	16.69
6 Once Every Three Months	106	5.73
7 Once Every Six Months	42	2.27
8 Once a Year	20	1.08
9 Almost Never	389	21.02
10 Other	90	4.86
Total	1,851	100.00

cd004_5_: Frequency Contacting XChildName[5]

	Freq.	%
1 Almost Every Day	52	5.62
2 2-3 Times a Week	93	10.05
3 Once a week	103	11.14
4 Every Two Weeks	146	15.78
5 Once a Month	151	16.32
6 Once Every Three Months	56	6.05
7 Once Every Six Months	29	3.14
8 Once a Year	9	0.97

	Freq.	%
9 Almost Never	244	26.38
10 Other	42	4.54
Total	925	100.00

cd004_6_: Frequency Contacting XChildName[6]

	Freq.	%
1 Almost Every Day	19	4.65
2 2-3 Times a Week	34	8.31
3 Once a week	45	11.00
4 Every Two Weeks	48	11.74
5 Once a Month	69	16.87
6 Once Every Three Months	21	5.13
7 Once Every Six Months	19	4.65
8 Once a Year	8	1.96
9 Almost Never	126	30.81
10 Other	20	4.89
Total	409	100.00

cd004_7_: Frequency Contacting XChildName[7]

	Freq.	%
1 Almost Every Day	14	6.54
2 2-3 Times a Week	19	8.88
3 Once a week	18	8.41
4 Every Two Weeks	34	15.89
5 Once a Month	39	18.22
6 Once Every Three Months	12	5.61
7 Once Every Six Months	3	1.40
8 Once a Year	2	0.93
9 Almost Never	62	28.97
10 Other	11	5.14
Total	214	100.00

cd004_8_: Frequency Contacting XChildName[8]

	Freq.	%
1 Almost Every Day	3	3.45
2 2-3 Times a Week	5	5.75
3 Once a week	6	6.90
4 Every Two Weeks	16	18.39
5 Once a Month	16	18.39
6 Once Every Three Months	5	5.75
7 Once Every Six Months	1	1.15
8 Once a Year	2	2.30
9 Almost Never	28	32.18
10 Other	5	5.75
Total	87	100.00

cd004_9_: Frequency Contacting XChildName[9]

	Freq.	%
1 Almost Every Day	1	2.44

2 2-3 Times a Week	1	2.44
3 Once a week	5	12.20
4 Every Two Weeks	6	14.63
5 Once a Month	7	17.07
6 Once Every Three Months	2	4.88
7 Once Every Six Months	1	2.44
8 Once a Year	1	2.44
9 Almost Never	16	39.02
10 Other	1	2.44
Total	41	100.00

ce002_1_1_: Total Money Support from XConParName[1]

Mean	SD	Min	Max	Obs
435.73	2,932.51	0.00	50,000.00	1,430

ce002_1_2_: Total Money Support from XConParName[2]

Mean	SD	Min	Max	Obs
222.74	2,927.20	0.00	100,000.00	1,728

ce002_1_3_: Total Money Support from XConParName[3]

Mean	SD	Min	Max	Obs
20.51	80.06	0.00	400.00	39

ce002_1_4_: Total Money Support from XConParName[4]

Mean	SD	Min	Max	Obs
0.00	0.00	0.00	0.00	48

ce002_1_5_: Total Money Support from XConParName[5]

Mean	SD	Min	Max	Obs
411.90	3,171.62	0.00	60,000.00	1,396

ce002_1_6_: Total Money Support from XConParName[6]

Mean	SD	Min	Max	Obs
310.66	6,254.88	0.00	200,000.00	1,410

ce002_1_7_: Total Money Support from XConParName[7]

Mean	SD	Min	Max	Obs
79.63	384.87	0.00	2,000.00	27

ce002_1_8_: Total Money Support from XConParName[8]

Mean	SD	Min	Max	Obs
185.19	962.25	0.00	5,000.00	27

ce002_2_1_: Regular Money Support from XConParName[1]

Mean	SD	Min	Max	Obs
69.42	1,148.89	0.00	36,000.00	1,432

ce002_2_2_: Regular Money Support from XConParName[2]

Mean	SD	Min	Max	Obs
38.47	807.43	0.00	30,000.00	1,729

ce002_2_3_: Regular Money Support from XConParName[3]

Mean	SD	Min	Max	Obs
11.15	64.15	0.00	400.00	39

ce002_2_4_: Regular Money Support from XConParName[4]

Mean	SD	Min	Max	Obs
0.00	0.00	0.00	0.00	48

ce002_2_5_: Regular Money Support from XConParName[5]

Mean	SD	Min	Max	Obs
86.39	2,184.09	0.00	80,000.00	1,396

ce002_2_6_: Regular Money Support from XConParName[6]

Mean	SD	Min	Max	Obs
95.49	3,196.01	0.00	120,000.00	1,415

ce002_2_7_: Regular Money Support from XConParName[7]

Mean	SD	Min	Max	Obs
0.00	0.00	0.00	0.00	27

ce002_2_8_: Regular Money Support from XConParName[8]

Mean	SD	Min	Max	Obs
0.00	0.00	0.00	0.00	27

ce002_3_1_: Total In_kind Support from XConParName[1]

Mean	SD	Min	Max	Obs
70.49	471.81	0.00	12,000.00	1,423

ce002_3_2_: Total In_kind Support from XConParName[2]

Mean	SD	Min	Max	Obs
37.88	273.57	0.00	5,000.00	1,718

ce002_3_3_: Total In_kind Support from XConParName[3]

Mean	SD	Min	Max	Obs
62.05	224.44	0.00	1,000.00	39

ce002_3_4_: Total In_kind Support from XConParName[4]

Mean	SD	Min	Max	Obs
21.00	144.32	0.00	1,000.00	48

ce002_3_5_: Total In_kind Support from XConParName[5]

Mean	SD	Min	Max	Obs
78.17	486.55	0.00	10,000.00	1,395

ce002_3_6_: Total In_kind Support from XConParName[6]

Mean	SD	Min	Max	Obs
26.55	228.79	0.00	5,000.00	1,411

ce002_3_7_: Total In_kind Support from XConParName[7]

Mean	SD	Min	Max	Obs
0.00	0.00	0.00	0.00	27

ce002_3_8_: Total In_kind Support from XConParName[8]

Mean	SD	Min	Max	Obs
9.63	50.04	0.00	260.00	27

ce002_4_1_: Regular In_kind Support from XConParName[1]

Mean	SD	Min	Max	Obs
7.08	122.09	0.00	4,000.00	1,430

ce002_4_2_: Regular In_kind Support from XConParName[2]

Mean	SD	Min	Max	Obs
2.21	39.23	0.00	1,000.00	1,724

ce002_4_3_: Regular In_kind Support from XConParName[3]

Mean	SD	Min	Max	Obs
0.00	0.00	0.00	0.00	39

ce002_4_4_: Regular In_kind Support from XConParName[4]

Mean	SD	Min	Max	Obs
0.00	0.00	0.00	0.00	48

ce002_4_5_: Regular In_kind Support from XConParName[5]

Mean	SD	Min	Max	Obs
11.22	237.11	0.00	8,000.00	1,396

ce002_4_6_: Regular In_kind Support from XConParName[6]

Mean	SD	Min	Max	Obs
0.30	6.41	0.00	200.00	1,414

ce002_4_7_: Regular In_kind Support from XConParName[7]

Mean	SD	Min	Max	Obs
0.00	0.00	0.00	0.00	27

ce002_4_8_: Regular In_kind Support from XConParName[8]

Mean	SD	Min	Max	Obs
0.00	0.00	0.00	0.00	27

ce002_1_1__min: Min Bracket of ce002_1_1

Mean	SD	Min	Max	Obs
1,000.00	848.53	400.00	1,600.00	2

ce002_1_1__max: Max Bracket of ce002_1_1

Mean	SD	Min	Max	Obs
400.00	.	400.00	400.00	1

ce002_1_2__min: Min Bracket of ce002_1_2

Mean	SD	Min	Max	Obs
1,600.00	.	1,600.00	1,600.00	1

ce002_1_2__max: Max Bracket of ce002_1_2

Mean	SD	Min	Max	Obs
1,600.00	.	1,600.00	1,600.00	1

ce002_1_5__min: Min Bracket of ce002_1_5

No Observations

ce002_1_5__max: Max Bracket of ce002_1_5

No Observations

ce002_1_6__min: Min Bracket of ce002_1_6

Mean	SD	Min	Max	Obs
950.00	754.98	200.00	1,600.00	4

ce002_1_6__max: Max Bracket of ce002_1_6

Mean	SD	Min	Max	Obs
875.00	838.15	100.00	1,600.00	4

ce002_1_8__min: Min Bracket of ce002_1_8

No Observations

ce002_1_8__max: Max Bracket of ce002_1_8

No Observations

ce002_2_1__min: Min Bracket of ce002_2_1

No Observations

ce002_2_1__max: Max Bracket of ce002_2_1

No Observations

ce002_2_2__min: Min Bracket of ce002_2_2

No Observations

ce002_2_2__max: Max Bracket of ce002_2_2

No Observations

ce002_2_5__min: Min Bracket of ce002_2_5

No Observations

ce002_2_5__max: Max Bracket of ce002_2_5

No Observations

ce002_2_6__min: Min Bracket of ce002_2_6

No Observations

ce002_2_6__max: Max Bracket of ce002_2_6

No Observations

ce002_2_8__min: Min Bracket of ce002_2_8

No Observations

ce002_2_8__max: Max Bracket of ce002_2_8

No Observations

ce002_3_1__min: Min Bracket of ce002_3_1

Mean	SD	Min	Max	Obs
1,114.29	630.95	200.00	1,600.00	7

ce002_3_1__max: Max Bracket of ce002_3_1

Mean	SD	Min	Max	Obs
700.00	600.00	100.00	1,600.00	5

ce002_3_2__min: Min Bracket of ce002_3_2

Mean	SD	Min	Max	Obs
442.86	522.36	100.00	1,600.00	7

ce002_3_2__max: Max Bracket of ce002_3_2

Mean	SD	Min	Max	Obs
233.33	136.63	100.00	400.00	6

ce002_3_5__min: Min Bracket of ce002_3_5

Mean	SD	Min	Max	Obs
200.00	.	200.00	200.00	1

ce002_3_5__max: Max Bracket of ce002_3_5

Mean	SD	Min	Max	Obs
200.00	.	200.00	200.00	1

ce002_3_6__min: Min Bracket of ce002_3_6

Mean	SD	Min	Max	Obs
850.00	1,060.66	100.00	1,600.00	2

ce002_3_6__max: Max Bracket of ce002_3_6

Mean	SD	Min	Max	Obs
850.00	1,060.66	100.00	1,600.00	2

ce002_3_8__min: Min Bracket of ce002_3_8

No Observations

ce002_3_8__max: Max Bracket of ce002_3_8

No Observations

ce002_4_1__min: Min Bracket of ce002_4_1

Mean	SD	Min	Max	Obs
100.00	.	100.00	100.00	1

ce002_4_1__max: Max Bracket of ce002_4_1

Mean	SD	Min	Max	Obs
100.00	0.00	100.00	100.00	2

ce002_4_2__min: Min Bracket of ce002_4_2

Mean	SD	Min	Max	Obs
400.00	.	400.00	400.00	1

ce002_4_2__max: Max Bracket of ce002_4_2

Mean	SD	Min	Max	Obs

400.00	.	400.00	400.00	1

ce002_4_5__min: Min Bracket of ce002_4_5

No Observations

ce002_4_5__max: Max Bracket of ce002_4_5

No Observations

ce002_4_6__min: Min Bracket of ce002_4_6

No Observations

ce002_4_6__max: Max Bracket of ce002_4_6

No Observations

ce002_4_8__min: Min Bracket of ce002_4_8

No Observations

ce002_4_8__max: Max Bracket of ce002_4_8

No Observations

ce009_1_1_: Total Money Support from XChildName[1]

Mean	SD	Min	Max	Obs
1,920.48	7,111.01	0.00	240,000.00	8,069

ce009_1_2_: Total Money Support from XChildName[2]

Mean	SD	Min	Max	Obs
1,448.03	4,744.45	0.00	150,000.00	7,431

ce009_1_3_: Total Money Support from XChildName[3]

Mean	SD	Min	Max	Obs
1,280.10	6,842.08	0.00	380,000.00	4,485

ce009_1_4_: Total Money Support from XChildName[4]

Mean	SD	Min	Max	Obs
974.49	3,362.64	0.00	100,000.00	2,450

ce009_1_5_: Total Money Support from XChildName[5]

Mean	SD	Min	Max	Obs
723.17	2,043.35	0.00	50,000.00	1,199

ce009_1_6_: Total Money Support from XChildName[6]

Mean	SD	Min	Max	Obs
562.03	1,154.71	0.00	10,000.00	533

ce009_1_7_: Total Money Support from XChildName[7]

Mean	SD	Min	Max	Obs
773.62	3,656.44	0.00	55,000.00	257

ce009_1_8_: Total Money Support from XChildName[8]

Mean	SD	Min	Max	Obs
1,259.05	5,484.24	0.00	50,000.00	116

ce009_1_9_: Total Money Support from XChildName[9]

Mean	SD	Min	Max	Obs
230.77	369.70	0.00	2,000.00	52

ce009_1_10_: Total Money Support from XChildName[10]

Mean	SD	Min	Max	Obs
194.83	342.87	0.00	1,400.00	29

ce009_1_11_: Total Money Support from XChildName[11]

Mean	SD	Min	Max	Obs
300.00	536.19	0.00	2,000.00	17

ce009_1_12_: Total Money Support from XChildName[12]

Mean	SD	Min	Max	Obs
183.33	297.91	0.00	800.00	9

ce009_1_13_: Total Money Support from XChildName[13]

Mean	SD	Min	Max	Obs
0.00	0.00	0.00	0.00	2

ce009_1_14_: Total Money Support from XChildName[14]

Mean	SD	Min	Max	Obs
0.00	0.00	0.00	0.00	2

ce009_1_15_: Total Money Support from XChildName[15]

Mean	SD	Min	Max	Obs
0.00	.	0.00	0.00	1

ce009_2_1_: Regular Money Support from XChildName[1]

Mean	SD	Min	Max	Obs
283.75	1,801.31	0.00	50,000.00	8,113

ce009_2_2_: Regular Money Support from XChildName[2]

Mean	SD	Min	Max	Obs
261.04	1,630.22	0.00	50,000.00	7,468

ce009_2_3_: Regular Money Support from XChildName[3]

Mean	SD	Min	Max	Obs
181.61	995.96	0.00	24,000.00	4,508

ce009_2_4_: Regular Money Support from XChildName[4]

Mean	SD	Min	Max	Obs
241.99	2,404.11	0.00	100,000.00	2,460

ce009_2_5_: Regular Money Support from XChildName[5]

Mean	SD	Min	Max	Obs
131.10	627.94	0.00	12,000.00	1,202

ce009_2_6_: Regular Money Support from XChildName[6]

Mean	SD	Min	Max	Obs
103.56	381.61	0.00	4,800.00	537

ce009_2_7_: Regular Money Support from XChildName[7]

Mean	SD	Min	Max	Obs
139.46	916.08	0.00	13,000.00	258

ce009_2_8_: Regular Money Support from XChildName[8]

Mean	SD	Min	Max	Obs
85.90	366.65	0.00	2,500.00	117

ce009_2_9_: Regular Money Support from XChildName[9]

Mean	SD	Min	Max	Obs
21.15	72.32	0.00	400.00	52

ce009_2_10_: Regular Money Support from XChildName[10]

Mean	SD	Min	Max	Obs
13.33	50.74	0.00	200.00	30

ce009_2_11_: Regular Money Support from XChildName[11]

Mean	SD	Min	Max	Obs
0.00	0.00	0.00	0.00	18

ce009_2_12_: Regular Money Support from XChildName[12]

Mean	SD	Min	Max	Obs
0.00	0.00	0.00	0.00	9

ce009_2_13_: Regular Money Support from XChildName[13]

Mean	SD	Min	Max	Obs
0.00	0.00	0.00	0.00	2

ce009_2_14_: Regular Money Support from XChildName[14]

Mean	SD	Min	Max	Obs
0.00	0.00	0.00	0.00	2

ce009_2_15_: Regular Money Support from XChildName[15]

Mean	SD	Min	Max	Obs
0.00	.	0.00	0.00	1

ce009_3_1_: Total In_kind Support from XChildName[1]

Mean	SD	Min	Max	Obs
873.48	2,477.99	0.00	80,000.00	7,840

ce009_3_2_: Total In_kind Support from XChildName[2]

Mean	SD	Min	Max	Obs
671.81	1,529.17	0.00	40,000.00	7,236

ce009_3_3_: Total In_kind Support from XChildName[3]

Mean	SD	Min	Max	Obs
640.81	3,251.66	0.00	150,000.00	4,354

ce009_3_4_: Total In_kind Support from XChildName[4]

Mean	SD	Min	Max	Obs
508.27	2,314.57	0.00	100,000.00	2,384

ce009_3_5_: Total In_kind Support from XChildName[5]

Mean	SD	Min	Max	Obs
462.84	1,428.45	0.00	30,000.00	1,159

ce009_3_6_: Total In_kind Support from XChildName[6]

Mean	SD	Min	Max	Obs
414.86	1,134.72	0.00	10,000.00	519

ce009_3_7_: Total In_kind Support from XChildName[7]

Mean	SD	Min	Max	Obs
244.84	506.72	0.00	4,000.00	248

ce009_3_8_: Total In_kind Support from XChildName[8]

Mean	SD	Min	Max	Obs
364.02	1,212.09	0.00	10,000.00	109

ce009_3_9_: Total In_kind Support from XChildName[9]

Mean	SD	Min	Max	Obs
155.50	209.85	0.00	1,000.00	50

ce009_3_10_: Total In_kind Support from XChildName[10]

Mean	SD	Min	Max	Obs
297.14	939.33	0.00	5,000.00	28

ce009_3_11_: Total In_kind Support from XChildName[11]

Mean	SD	Min	Max	Obs
132.35	219.33	0.00	750.00	17

ce009_3_12_: Total In_kind Support from XChildName[12]

Mean	SD	Min	Max	Obs
161.11	259.54	0.00	800.00	9

ce009_3_13_: Total In_kind Support from XChildName[13]

Mean	SD	Min	Max	Obs
100.00	141.42	0.00	200.00	2

ce009_3_14_: Total In_kind Support from XChildName[14]

Mean	SD	Min	Max	Obs
1,150.00	1,202.08	300.00	2,000.00	2

ce009_3_15_: Total In_kind Support from XChildName[15]

Mean	SD	Min	Max	Obs
1,000.00	.	1,000.00	1,000.00	1

ce009_4_1_: Regular In_kind Support from XChildName[1]

Mean	SD	Min	Max	Obs
82.05	634.94	0.00	20,000.00	8,051

ce009_4_2_: Regular In_kind Support from XChildName[2]

Mean	SD	Min	Max	Obs
67.69	507.66	0.00	20,000.00	7,426

ce009_4_3_: Regular In_kind Support from XChildName[3]

Mean	SD	Min	Max	Obs
59.77	402.31	0.00	10,000.00	4,481

ce009_4_4_: Regular In_kind Support from XChildName[4]

Mean	SD	Min	Max	Obs
50.99	354.49	0.00	10,000.00	2,446

ce009_4_5_: Regular In_kind Support from XChildName[5]

Mean	SD	Min	Max	Obs
51.49	364.91	0.00	8,000.00	1,192

ce009_4_6_: Regular In_kind Support from XChildName[6]

Mean	SD	Min	Max	Obs
45.09	277.94	0.00	5,000.00	530

ce009_4_7_: Regular In_kind Support from XChildName[7]

Mean	SD	Min	Max	Obs
8.66	47.69	0.00	500.00	254

ce009_4_8_: Regular In_kind Support from XChildName[8]

Mean	SD	Min	Max	Obs
3.56	21.38	0.00	200.00	117

ce009_4_9_: Regular In_kind Support from XChildName[9]

Mean	SD	Min	Max	Obs
0.54	3.48	0.00	25.00	52

ce009_4_10_: Regular In_kind Support from XChildName[10]

Mean	SD	Min	Max	Obs
0.10	0.56	0.00	3.00	29

ce009_4_11_: Regular In_kind Support from XChildName[11]

Mean	SD	Min	Max	Obs
0.00	0.00	0.00	0.00	18

ce009_4_12_: Regular In_kind Support from XChildName[12]

Mean	SD	Min	Max	Obs
0.00	0.00	0.00	0.00	9

ce009_4_13_: Regular In_kind Support from XChildName[13]

Mean	SD	Min	Max	Obs
0.00	0.00	0.00	0.00	2

ce009_4_14_: Regular In_kind Support from XChildName[14]

Mean	SD	Min	Max	Obs
0.00	0.00	0.00	0.00	2

ce009_4_15_: Regular In_kind Support from XChildName[15]

Mean	SD	Min	Max	Obs
0.00	.	0.00	0.00	1

ce009_1_1__min: Min Bracket of ce009_1_1_

Mean	SD	Min	Max	Obs
954.72	583.61	100.00	1,600.00	53

ce009_1_1__max: Max Bracket of ce009_1_1_

Mean	SD	Min	Max	Obs
725.64	566.48	100.00	1,600.00	39

ce009_1_2__min: Min Bracket of ce009_1_2_

Mean	SD	Min	Max	Obs
851.22	604.62	100.00	1,600.00	41

ce009_1_2__max: Max Bracket of ce009_1_2_

Mean	SD	Min	Max	Obs
806.90	581.21	100.00	1,600.00	29

ce009_1_3__min: Min Bracket of ce009_1_3_

Mean	SD	Min	Max	Obs
861.11	585.25	100.00	1,600.00	18

ce009_1_3__max: Max Bracket of ce009_1_3_

Mean	SD	Min	Max	Obs
708.33	496.27	100.00	1,600.00	12

ce009_1_4__min: Min Bracket of ce009_1_4_

Mean	SD	Min	Max	Obs
791.67	618.59	100.00	1,600.00	12

ce009_1_4__max: Max Bracket of ce009_1_4_

Mean	SD	Min	Max	Obs
611.11	453.99	100.00	1,600.00	9

ce009_1_5__min: Min Bracket of ce009_1_5_

Mean	SD	Min	Max	Obs
583.33	552.87	100.00	1,600.00	6

ce009_1_5__max: Max Bracket of ce009_1_5_

Mean	SD	Min	Max	Obs
625.00	665.21	100.00	1,600.00	4

ce009_1_6__min: Min Bracket of ce009_1_6_

Mean	SD	Min	Max	Obs
933.33	611.01	400.00	1,600.00	3

ce009_1_6__max: Max Bracket of ce009_1_6_

Mean	SD	Min	Max	Obs
1,200.00	692.82	400.00	1,600.00	3

ce009_1_7__min: Min Bracket of ce009_1_7_

Mean	SD	Min	Max	Obs
400.00	0.00	400.00	400.00	2

ce009_1_7__max: Max Bracket of ce009_1_7_

Mean	SD	Min	Max	Obs
800.00	.	800.00	800.00	1

ce009_1_8__min: Min Bracket of ce009_1_8_

Mean	SD	Min	Max	Obs
400.00	.	400.00	400.00	1

ce009_1_8__max: Max Bracket of ce009_1_8_

No Observations

ce009_1_10__min: Min Bracket of ce009_1_10_

Mean	SD	Min	Max	Obs

200.00	.	200.00	200.00	1

ce009_1_10__max: Max Bracket of ce009_1_10_

Mean	SD	Min	Max	Obs
200.00	.	200.00	200.00	1

ce009_1_11__min: Min Bracket of ce009_1_11_

No Observations

ce009_1_11__max: Max Bracket of ce009_1_11_

No Observations

ce009_2_1__min: Min Bracket of ce009_2_1_

Mean	SD	Min	Max	Obs
800.00	509.90	200.00	1,600.00	13

ce009_2_1__max: Max Bracket of ce009_2_1_

Mean	SD	Min	Max	Obs
613.64	604.98	100.00	1,600.00	22

ce009_2_2__min: Min Bracket of ce009_2_2_

Mean	SD	Min	Max	Obs
616.67	530.72	100.00	1,600.00	6

ce009_2_2__max: Max Bracket of ce009_2_2_

Mean	SD	Min	Max	Obs
333.33	453.94	100.00	1,600.00	12

ce009_2_3__min: Min Bracket of ce009_2_3_

Mean	SD	Min	Max	Obs
700.00	793.73	100.00	1,600.00	3

ce009_2_3__max: Max Bracket of ce009_2_3_

Mean	SD	Min	Max	Obs
540.00	665.58	100.00	1,600.00	5

ce009_2_4__min: Min Bracket of ce009_2_4_

Mean	SD	Min	Max	Obs
266.67	115.47	200.00	400.00	3

ce009_2_4__max: Max Bracket of ce009_2_4_

Mean	SD	Min	Max	Obs
283.33	278.69	100.00	800.00	6

ce009_2_5__min: Min Bracket of ce009_2_5_

Mean	SD	Min	Max	Obs
200.00	0.00	200.00	200.00	2

ce009_2_5__max: Max Bracket of ce009_2_5_

Mean	SD	Min	Max	Obs
166.67	57.74	100.00	200.00	3

ce009_2_6__min: Min Bracket of ce009_2_6_

No Observations

ce009_2_6__max: Max Bracket of ce009_2_6_

No Observations

ce009_2_7__min: Min Bracket of ce009_2_7_

No Observations

ce009_2_7__max: Max Bracket of ce009_2_7_

No Observations

ce009_2_8__min: Min Bracket of ce009_2_8_

No Observations

ce009_2_8__max: Max Bracket of ce009_2_8_

No Observations

ce009_2_10__min: Min Bracket of ce009_2_10_

No Observations

ce009_2_10__max: Max Bracket of ce009_2_10_

No Observations

ce009_2_11__min: Min Bracket of ce009_2_11_

No Observations

ce009_2_11__max: Max Bracket of ce009_2_11_

No Observations

ce009_3_1__min: Min Bracket of ce009_3_1_

Mean	SD	Min	Max	Obs
722.69	537.68	100.00	1,600.00	238

ce009_3_1__max: Max Bracket of ce009_3_1_

Mean	SD	Min	Max	Obs
703.08	508.17	100.00	1,600.00	195

ce009_3_2__min: Min Bracket of ce009_3_2_

Mean	SD	Min	Max	Obs
698.24	529.09	100.00	1,600.00	170

ce009_3_2__max: Max Bracket of ce009_3_2_

Mean	SD	Min	Max	Obs
681.41	499.01	100.00	1,600.00	156

ce009_3_3__min: Min Bracket of ce009_3_3_

Mean	SD	Min	Max	Obs
670.80	515.42	100.00	1,600.00	113

ce009_3_3__max: Max Bracket of ce009_3_3_

Mean	SD	Min	Max	Obs
639.60	500.62	100.00	1,600.00	101

ce009_3_4__min: Min Bracket of ce009_3_4_

Mean	SD	Min	Max	Obs
541.67	478.40	100.00	1,600.00	48

ce009_3_4__max: Max Bracket of ce009_3_4_

Mean	SD	Min	Max	Obs
491.30	420.49	100.00	1,600.00	46

ce009_3_5__min: Min Bracket of ce009_3_5_

Mean	SD	Min	Max	Obs
692.86	547.00	100.00	1,600.00	28

ce009_3_5__max: Max Bracket of ce009_3_5_

Mean	SD	Min	Max	Obs
610.71	547.95	100.00	1,600.00	28

ce009_3_6__min: Min Bracket of ce009_3_6_

Mean	SD	Min	Max	Obs
372.73	237.03	100.00	800.00	11

ce009_3_6__max: Max Bracket of ce009_3_6_

Mean	SD	Min	Max	Obs
409.09	270.02	100.00	800.00	11

ce009_3_7__min: Min Bracket of ce009_3_7_

Mean	SD	Min	Max	Obs
700.00	600.00	400.00	1,600.00	4

ce009_3_7__max: Max Bracket of ce009_3_7_

Mean	SD	Min	Max	Obs
500.00	200.00	400.00	800.00	4

ce009_3_8__min: Min Bracket of ce009_3_8_

Mean	SD	Min	Max	Obs
400.00	.	400.00	400.00	1

ce009_3_8__max: Max Bracket of ce009_3_8_

Mean	SD	Min	Max	Obs
400.00	.	400.00	400.00	1

ce009_3_10__min: Min Bracket of ce009_3_10_

Mean	SD	Min	Max	Obs
800.00	.	800.00	800.00	1

ce009_3_10__max: Max Bracket of ce009_3_10_

Mean	SD	Min	Max	Obs
1,600.00	.	1,600.00	1,600.00	1

ce009_3_11__min: Min Bracket of ce009_3_11_

No Observations

ce009_3_11__max: Max Bracket of ce009_3_11_

No Observations

ce009_4_1__min: Min Bracket of ce009_4_1_

Mean	SD	Min	Max	Obs
511.36	457.61	100.00	1,600.00	44

ce009_4_1__max: Max Bracket of ce009_4_1_

Mean	SD	Min	Max	Obs
408.20	435.62	100.00	1,600.00	61

ce009_4_2__min: Min Bracket of ce009_4_2_

Mean	SD	Min	Max	Obs
454.17	436.37	100.00	1,600.00	24

ce009_4_2__max: Max Bracket of ce009_4_2_

Mean	SD	Min	Max	Obs
394.74	434.93	100.00	1,600.00	38

ce009_4_3__min: Min Bracket of ce009_4_3_

Mean	SD	Min	Max	Obs
500.00	499.23	100.00	1,600.00	14

ce009_4_3__max: Max Bracket of ce009_4_3_

Mean	SD	Min	Max	Obs
352.00	427.32	100.00	1,600.00	25

ce009_4_4__min: Min Bracket of ce009_4_4_

Mean	SD	Min	Max	Obs
475.00	281.58	200.00	800.00	8

ce009_4_4__max: Max Bracket of ce009_4_4_

Mean	SD	Min	Max	Obs
306.25	269.49	100.00	800.00	16

ce009_4_5__min: Min Bracket of ce009_4_5_

Mean	SD	Min	Max	Obs
914.29	671.88	200.00	1,600.00	7

ce009_4_5__max: Max Bracket of ce009_4_5_

Mean	SD	Min	Max	Obs
488.89	637.27	100.00	1,600.00	9

ce009_4_6__min: Min Bracket of ce009_4_6_

Mean	SD	Min	Max	Obs
200.00	173.21	100.00	400.00	3

ce009_4_6__max: Max Bracket of ce009_4_6_

Mean	SD	Min	Max	Obs
160.00	134.16	100.00	400.00	5

ce009_4_7__min: Min Bracket of ce009_4_7_

No Observations

ce009_4_7__max: Max Bracket of ce009_4_7_

No Observations

ce009_4_8__min: Min Bracket of ce009_4_8_

No Observations

ce009_4_8__max: Max Bracket of ce009_4_8_

No Observations

ce009_4_10__min: Min Bracket of ce009_4_10_

No Observations

ce009_4_10__max: Max Bracket of ce009_4_10_

No Observations

ce009_4_11__min: Min Bracket of ce009_4_11_

No Observations

ce009_4_11__max: Max Bracket of ce009_4_11_

No Observations

ce016: Economic Supports from Relatives

Mean	SD	Min	Max	Obs
206.85	1,756.41	0.00	65,000.00	11,471

ce016_min: Min Bracket of ce016

Mean	SD	Min	Max	Obs
712.12	557.20	100.00	1,600.00	33

ce016_max: Max Bracket of ce016

Mean	SD	Min	Max	Obs
613.79	524.22	100.00	1,600.00	29

ce016_w3: Cash Gift from Relatives

Mean	SD	Min	Max	Obs
1,199.86	7,448.06	0.00	240,000.00	11,451

ce016_w3_min: Max Bracket of ce016_w3

Mean	SD	Min	Max	Obs
3,964.91	3,580.53	500.00	10,000.00	57

ce016_w3_max: Min Bracket of ce016_w3

Mean	SD	Min	Max	Obs
3,867.92	3,429.44	500.00	10,000.00	53

ce016_w4: Expenditure

Mean	SD	Min	Max	Obs
1,621.13	9,047.51	0.00	250,000.00	11,540

ce016_w4_min: Min Bracket of ce016_w4

Mean	SD	Min	Max	Obs
4,940.48	3,738.73	500.00	10,000.00	42

ce016_w4_max: Max Bracket of ce016_w4

Mean	SD	Min	Max	Obs
3,818.18	2,946.97	500.00	10,000.00	33

ce022_1_1_: Total Money Support for XConParName[1]

Mean	SD	Min	Max	Obs
1,054.98	2,770.68	0.00	50,000.00	1,425

ce022_2_1_: Regular Money Support for XConParName[1]

Mean	SD	Min	Max	Obs
170.76	857.13	0.00	12,000.00	1,430

ce022_3_1_: Total In_kind Support for XConParName[1]

Mean	SD	Min	Max	Obs
706.40	1,418.33	0.00	20,000.00	1,399

ce022_4_1_: Regular In_kind Support for XConParName[1]

Mean	SD	Min	Max	Obs
76.06	490.76	0.00	10,000.00	1,422

ce022_1_2_: Total Money Support for XConParName[2]

Mean	SD	Min	Max	Obs
857.43	2,019.88	0.00	25,000.00	1,715

ce022_2_2_: Regular Money Support for XConParName[2]

Mean	SD	Min	Max	Obs
154.17	889.62	0.00	20,000.00	1,724

ce022_3_2_: Total In_kind Support for XConParName[2]

Mean	SD	Min	Max	Obs
686.16	1,647.34	0.00	30,000.00	1,696

ce022_4_2_: Regular In_kind Support for XConParName[2]

Mean	SD	Min	Max	Obs
82.75	503.75	0.00	10,000.00	1,716

ce022_1_3_: Total Money Support for XConParName[3]

Mean	SD	Min	Max	Obs
235.90	393.02	0.00	1,200.00	39

ce022_2_3_: Regular Money Support for XConParName[3]

Mean	SD	Min	Max	Obs
12.82	80.06	0.00	500.00	39

ce022_3_3_: Total In_kind Support for XConParName[3]

Mean	SD	Min	Max	Obs
234.74	540.36	0.00	3,000.00	38

ce022_4_3_: Regular In_kind Support for XConParName[3]

Mean	SD	Min	Max	Obs
23.08	101.21	0.00	500.00	39

ce022_1_4_: Total Money Support for XConParName[4]

Mean	SD	Min	Max	Obs
259.38	606.06	0.00	3,000.00	48

ce022_2_4_: Regular Money Support for XConParName[4]

Mean	SD	Min	Max	Obs
164.58	838.05	0.00	5,500.00	48

ce022_3_4_: Total In_kind Support for XConParName[4]

Mean	SD	Min	Max	Obs
231.67	460.51	0.00	2,000.00	48

ce022_4_4_: Regular In_kind Support for XConParName[4]

Mean	SD	Min	Max	Obs
48.54	288.72	0.00	2,000.00	48

ce022_1_5_: Total Money Support for XConParName[5]

Mean	SD	Min	Max	Obs
1,224.32	4,964.66	0.00	150,000.00	1,385

ce022_2_5_: Regular Money Support for XConParName[5]

Mean	SD	Min	Max	Obs
213.90	1,106.99	0.00	20,000.00	1,389

ce022_3_5_: Total In_kind Support for XConParName[5]

Mean	SD	Min	Max	Obs
719.20	1,344.97	0.00	20,000.00	1,368

ce022_4_5_: Regular In_kind Support for XConParName[5]

Mean	SD	Min	Max	Obs
62.11	414.31	0.00	10,000.00	1,391

ce022_1_6_: Total Money Support for XConParName[6]

Mean	SD	Min	Max	Obs
969.40	3,881.64	0.00	120,000.00	1,401

ce022_2_6_: Regular Money Support for XConParName[6]

Mean	SD	Min	Max	Obs
161.63	1,111.91	0.00	36,000.00	1,414

ce022_3_6_: Total In_kind Support for XConParName[6]

Mean	SD	Min	Max	Obs
634.49	1,379.61	0.00	20,000.00	1,380

ce022_4_6_: Regular In_kind Support for XConParName[6]

Mean	SD	Min	Max	Obs
74.79	498.42	0.00	10,000.00	1,407

ce022_1_7_: Total Money Support for XConParName[7]

Mean	SD	Min	Max	Obs
805.56	2,101.21	0.00	10,000.00	27

ce022_2_7_: Regular Money Support for XConParName[7]

Mean	SD	Min	Max	Obs
168.89	769.60	0.00	4,000.00	27

ce022_3_7_: Total In_kind Support for XConParName[7]

Mean	SD	Min	Max	Obs
490.74	1,316.47	0.00	5,000.00	27

ce022_4_7_: Regular In_kind Support for XConParName[7]

Mean	SD	Min	Max	Obs
22.22	97.40	0.00	500.00	27

ce022_1_8_: Total Money Support for XConParName[8]

Mean	SD	Min	Max	Obs
427.78	719.55	0.00	3,000.00	27

ce022_2_8_: Regular Money Support for XConParName[8]

Mean	SD	Min	Max	Obs
166.67	313.79	0.00	1,200.00	27

ce022_3_8_: Total In_kind Support for XConParName[8]

Mean	SD	Min	Max	Obs
286.22	540.44	0.00	2,000.00	27

ce022_4_8_: Regular In_kind Support for XConParName[8]

Mean	SD	Min	Max	Obs
65.11	212.79	0.00	1,000.00	27

ce022_1_1__min: Min Bracket of ce022_1_1_

Mean	SD	Min	Max	Obs
1,000.00	692.82	400.00	1,600.00	4

ce022_1_1__max: Max Bracket of ce022_1_1_

Mean	SD	Min	Max	Obs
800.00	692.82	400.00	1,600.00	3

ce022_1_2__min: Min Bracket of ce022_1_2_

Mean	SD	Min	Max	Obs
940.00	596.66	200.00	1,600.00	10

ce022_1_2__max: Max Bracket of ce022_1_2_

Mean	SD	Min	Max	Obs
957.14	657.92	100.00	1,600.00	7

ce022_1_5__min: Min Bracket of ce022_1_5_

Mean	SD	Min	Max	Obs
900.00	503.32	400.00	1,600.00	4

ce022_1_5__max: Max Bracket of ce022_1_5_

Mean	SD	Min	Max	Obs
900.00	685.57	100.00	1,600.00	5

ce022_1_6__min: Min Bracket of ce022_1_6_

Mean	SD	Min	Max	Obs
1,054.55	544.73	400.00	1,600.00	11

ce022_1_6__max: Max Bracket of ce022_1_6_

Mean	SD	Min	Max	Obs
720.00	521.54	400.00	1,600.00	5

ce022_1_8__min: Min Bracket of ce022_1_8_

No Observations

ce022_1_8__max: Max Bracket of ce022_1_8_

No Observations

ce022_2_1__min: Min Bracket of ce022_2_1_

No Observations

ce022_2_1__max: Max Bracket of ce022_2_1_

No Observations

ce022_2_2__min: Min Bracket of ce022_2_2_

Mean	SD	Min	Max	Obs
400.00	.	400.00	400.00	1

ce022_2_2__max: Max Bracket of ce022_2_2_

Mean	SD	Min	Max	Obs
250.00	212.13	100.00	400.00	2

ce022_2_5__min: Min Bracket of ce022_2_5_

Mean	SD	Min	Max	Obs
333.33	115.47	200.00	400.00	3

ce022_2_5__max: Max Bracket of ce022_2_5_

Mean	SD	Min	Max	Obs
400.00	0.00	400.00	400.00	2

ce022_2_6__min: Min Bracket of ce022_2_6_

No Observations

ce022_2_6__max: Max Bracket of ce022_2_6_

No Observations

ce022_2_8__min: Min Bracket of ce022_2_8_

No Observations

ce022_2_8__max: Max Bracket of ce022_2_8_

No Observations

ce022_3_1__min: Min Bracket of ce022_3_1_

Mean	SD	Min	Max	Obs
762.50	547.57	100.00	1,600.00	24

ce022_3_1__max: Max Bracket of ce022_3_1_

Mean	SD	Min	Max	Obs
715.79	545.96	100.00	1,600.00	19

ce022_3_2__min: Min Bracket of ce022_3_2_

Mean	SD	Min	Max	Obs
616.67	502.75	100.00	1,600.00	24

ce022_3_2__max: Max Bracket of ce022_3_2_

Mean	SD	Min	Max	Obs
685.00	537.32	100.00	1,600.00	20

ce022_3_5__min: Min Bracket of ce022_3_5_

Mean	SD	Min	Max	Obs
640.00	466.11	200.00	1,600.00	20

ce022_3_5__max: Max Bracket of ce022_3_5_

Mean	SD	Min	Max	Obs
594.44	343.81	100.00	1,600.00	18

ce022_3_6__min: Min Bracket of ce022_3_6_

Mean	SD	Min	Max	Obs
895.24	546.33	200.00	1,600.00	21

ce022_3_6__max: Max Bracket of ce022_3_6_

Mean	SD	Min	Max	Obs
789.47	554.67	100.00	1,600.00	19

ce022_3_8__min: Min Bracket of ce022_3_8_

No Observations

ce022_3_8__max: Max Bracket of ce022_3_8_

No Observations

ce022_4_1__min: Min Bracket of ce022_4_1_

Mean	SD	Min	Max	Obs
300.00	336.65	100.00	800.00	4

ce022_4_1__max: Max Bracket of ce022_4_1_

Mean	SD	Min	Max	Obs
116.67	40.82	100.00	200.00	6

ce022_4_2__min: Min Bracket of ce022_4_2_

Mean	SD	Min	Max	Obs
400.00	282.84	200.00	800.00	4

ce022_4_2__max: Max Bracket of ce022_4_2_

Mean	SD	Min	Max	Obs
466.67	571.55	100.00	1,600.00	6

ce022_4_5__min: Min Bracket of ce022_4_5_

Mean	SD	Min	Max	Obs
800.00	.	800.00	800.00	1

ce022_4_5__max: Max Bracket of ce022_4_5_

Mean	SD	Min	Max	Obs
450.00	494.97	100.00	800.00	2

ce022_4_6__min: Min Bracket of ce022_4_6_

Mean	SD	Min	Max	Obs
300.00	141.42	200.00	400.00	2

ce022_4_6__max: Max Bracket of ce022_4_6_

Mean	SD	Min	Max	Obs
433.33	351.19	100.00	800.00	3

ce022_4_8__min: Min Bracket of ce022_4_8_

No Observations

ce022_4_8__max: Max Bracket of ce022_4_8_

No Observations

ce029_1_1_: Total Money Support for XChildName[1]

Mean	SD	Min	Max	Obs

3,910.73	30,231.64	0.00	1,200,000.00	8,106

ce029_2_1_: Regular Money Support for XChildName[1]

Mean	SD	Min	Max	Obs
557.52	9,040.44	0.00	672,000.00	8,134

ce029_3_1_: Total In_kind Support for XChildName[1]

Mean	SD	Min	Max	Obs
352.23	2,362.93	0.00	100,000.00	8,044

ce029_4_1_: Regular In_kind Support for XChildName[1]

Mean	SD	Min	Max	Obs
36.86	579.12	0.00	30,000.00	8,119

ce029_1_2_: Total Money Support for XChildName[2]

Mean	SD	Min	Max	Obs
1,648.38	11,457.06	0.00	600,000.00	7,471

ce029_2_2_: Regular Money Support for XChildName[2]

Mean	SD	Min	Max	Obs
271.89	2,064.71	0.00	40,000.00	7,490

ce029_3_2_: Total In_kind Support for XChildName[2]

Mean	SD	Min	Max	Obs
242.01	5,897.64	0.00	500,000.00	7,449

ce029_4_2_: Regular In_kind Support for XChildName[2]

Mean	SD	Min	Max	Obs
21.91	406.29	0.00	20,000.00	7,492

ce029_1_3_: Total Money Support for XChildName[3]

Mean	SD	Min	Max	Obs
934.47	15,841.07	0.00	1,000,000.00	4,515

ce029_2_3_: Regular Money Support for XChildName[3]

Mean	SD	Min	Max	Obs

141.97	1,750.54	0.00	50,000.00	4,526

ce029_3_3_: Total In_kind Support for XChildName[3]

Mean	SD	Min	Max	Obs
64.72	358.21	0.00	10,000.00	4,503

ce029_4_3_: Regular In_kind Support for XChildName[3]

Mean	SD	Min	Max	Obs
5.91	97.59	0.00	4,500.00	4,522

ce029_1_4_: Total Money Support for XChildName[4]

Mean	SD	Min	Max	Obs
291.17	2,345.29	0.00	70,000.00	2,470

ce029_2_4_: Regular Money Support for XChildName[4]

Mean	SD	Min	Max	Obs
42.99	620.81	0.00	20,000.00	2,476

ce029_3_4_: Total In_kind Support for XChildName[4]

Mean	SD	Min	Max	Obs
57.09	491.64	0.00	15,000.00	2,466

ce029_4_4_: Regular In_kind Support for XChildName[4]

Mean	SD	Min	Max	Obs
8.08	135.52	0.00	5,000.00	2,474

ce029_1_5_: Total Money Support for XChildName[5]

Mean	SD	Min	Max	Obs
378.92	3,466.74	0.00	80,000.00	1,213

ce029_2_5_: Regular Money Support for XChildName[5]

Mean	SD	Min	Max	Obs
89.11	1,580.77	0.00	50,000.00	1,213

ce029_3_5_: Total In_kind Support for XChildName[5]

Mean	SD	Min	Max	Obs

36.69	256.88	0.00	5,000.00	1,206

ce029_4_5_: Regular In_kind Support for XChildName[5]

Mean	SD	Min	Max	Obs
7.69	123.86	0.00	3,500.00	1,212

ce029_1_6_: Total Money Support for XChildName[6]

Mean	SD	Min	Max	Obs
362.16	2,779.39	0.00	40,000.00	541

ce029_2_6_: Regular Money Support for XChildName[6]

Mean	SD	Min	Max	Obs
83.20	1,253.96	0.00	25,000.00	541

ce029_3_6_: Total In_kind Support for XChildName[6]

Mean	SD	Min	Max	Obs
32.29	194.84	0.00	2,500.00	540

ce029_4_6_: Regular In_kind Support for XChildName[6]

Mean	SD	Min	Max	Obs
3.40	46.20	0.00	1,000.00	541

ce029_1_7_: Total Money Support for XChildName[7]

Mean	SD	Min	Max	Obs
469.46	4,030.18	0.00	50,000.00	261

ce029_2_7_: Regular Money Support for XChildName[7]

Mean	SD	Min	Max	Obs
214.75	3,109.01	0.00	50,000.00	261

ce029_3_7_: Total In_kind Support for XChildName[7]

Mean	SD	Min	Max	Obs
21.92	123.31	0.00	1,200.00	260

ce029_4_7_: Regular In_kind Support for XChildName[7]

Mean	SD	Min	Max	Obs

4.42	62.37	0.00	1,000.00	260

ce029_1_8_: Total Money Support for XChildName[8]

Mean	SD	Min	Max	Obs
26.50	113.06	0.00	1,000.00	117

ce029_2_8_: Regular Money Support for XChildName[8]

Mean	SD	Min	Max	Obs
2.14	19.02	0.00	200.00	117

ce029_3_8_: Total In_kind Support for XChildName[8]

Mean	SD	Min	Max	Obs
8.70	36.71	0.00	200.00	117

ce029_4_8_: Regular In_kind Support for XChildName[8]

Mean	SD	Min	Max	Obs
0.43	4.62	0.00	50.00	117

ce029_1_9_: Total Money Support for XChildName[9]

Mean	SD	Min	Max	Obs
55.77	281.74	0.00	2,000.00	52

ce029_2_9_: Regular Money Support for XChildName[9]

Mean	SD	Min	Max	Obs
0.00	0.00	0.00	0.00	52

ce029_3_9_: Total In_kind Support for XChildName[9]

Mean	SD	Min	Max	Obs
19.46	68.66	0.00	400.00	52

ce029_4_9_: Regular In_kind Support for XChildName[9]

Mean	SD	Min	Max	Obs
0.00	0.00	0.00	0.00	52

ce029_1_10_: Total Money Support for XChildName[10]

Mean	SD	Min	Max	Obs

70.00	205.36	0.00	1,000.00	30

ce029_2_10_: Regular Money Support for XChildName[10]

Mean	SD	Min	Max	Obs
0.00	0.00	0.00	0.00	30

ce029_3_10_: Total In_kind Support for XChildName[10]

Mean	SD	Min	Max	Obs
20.00	92.48	0.00	500.00	30

ce029_4_10_: Regular In_kind Support for XChildName[10]

Mean	SD	Min	Max	Obs
0.10	0.55	0.00	3.00	30

ce029_1_11_: Total Money Support for XChildName[11]

Mean	SD	Min	Max	Obs
33.33	118.82	0.00	500.00	18

ce029_2_11_: Regular Money Support for XChildName[11]

Mean	SD	Min	Max	Obs
0.00	0.00	0.00	0.00	18

ce029_3_11_: Total In_kind Support for XChildName[11]

Mean	SD	Min	Max	Obs
0.00	0.00	0.00	0.00	18

ce029_4_11_: Regular In_kind Support for XChildName[11]

Mean	SD	Min	Max	Obs
0.00	0.00	0.00	0.00	18

ce029_1_12_: Total Money Support for XChildName[12]

Mean	SD	Min	Max	Obs
55.56	166.67	0.00	500.00	9

ce029_2_12_: Regular Money Support for XChildName[12]

Mean	SD	Min	Max	Obs

0.00	0.00	0.00	0.00	9

ce029_3_12_: Total In_kind Support for XChildName[12]

Mean	SD	Min	Max	Obs
0.00	0.00	0.00	0.00	9

ce029_4_12_: Regular In_kind Support for XChildName[12]

Mean	SD	Min	Max	Obs
0.00	0.00	0.00	0.00	9

ce029_1_13_: Total Money Support for XChildName[13]

Mean	SD	Min	Max	Obs
0.00	0.00	0.00	0.00	2

ce029_2_13_: Regular Money Support for XChildName[13]

Mean	SD	Min	Max	Obs
0.00	0.00	0.00	0.00	2

ce029_3_13_: Total In_kind Support for XChildName[13]

Mean	SD	Min	Max	Obs
0.00	0.00	0.00	0.00	2

ce029_4_13_: Regular In_kind Support for XChildName[13]

Mean	SD	Min	Max	Obs
0.00	0.00	0.00	0.00	2

ce029_1_14_: Total Money Support for XChildName[14]

Mean	SD	Min	Max	Obs
0.00	0.00	0.00	0.00	2

ce029_2_14_: Regular Money Support for XChildName[14]

Mean	SD	Min	Max	Obs
0.00	0.00	0.00	0.00	2

ce029_3_14_: Total In_kind Support for XChildName[14]

Mean	SD	Min	Max	Obs

0.00	0.00	0.00	0.00	2

ce029_4_14_: Regular In_kind Support for XChildName[14]

Mean	SD	Min	Max	Obs
0.00	0.00	0.00	0.00	2

ce029_1_15_: Total Money Support for XChildName[15]

Mean	SD	Min	Max	Obs
0.00	.	0.00	0.00	1

ce029_2_15_: Regular Money Support for XChildName[15]

Mean	SD	Min	Max	Obs
0.00	.	0.00	0.00	1

ce029_3_15_: Total In_kind Support for XChildName[15]

Mean	SD	Min	Max	Obs
0.00	.	0.00	0.00	1

ce029_4_15_: Regular In_kind Support for XChildName[15]

Mean	SD	Min	Max	Obs
0.00	.	0.00	0.00	1

ce029_1_1__min: Min Bracket of ce029_1_1_

Mean	SD	Min	Max	Obs
1,064.29	611.14	200.00	1,600.00	28

ce029_1_1__max: Max Bracket of ce029_1_1_

Mean	SD	Min	Max	Obs
593.33	478.79	100.00	1,600.00	15

ce029_1_2__min: Min Bracket of ce029_1_2_

Mean	SD	Min	Max	Obs
1,028.57	616.98	200.00	1,600.00	14

ce029_1_2__max: Max Bracket of ce029_1_2_

Mean	SD	Min	Max	Obs

660.00	658.62	100.00	1,600.00	10

ce029_1_3__min: Min Bracket of ce029_1_3_

Mean	SD	Min	Max	Obs
550.00	300.00	200.00	800.00	4

ce029_1_3__max: Max Bracket of ce029_1_3_

Mean	SD	Min	Max	Obs
475.00	377.49	100.00	800.00	4

ce029_1_4__min: Min Bracket of ce029_1_4_

Mean	SD	Min	Max	Obs
933.33	611.01	400.00	1,600.00	3

ce029_1_4__max: Max Bracket of ce029_1_4_

Mean	SD	Min	Max	Obs
433.33	351.19	100.00	800.00	3

ce029_1_5__min: Min Bracket of ce029_1_5_

Mean	SD	Min	Max	Obs
800.00	.	800.00	800.00	1

ce029_1_5__max: Max Bracket of ce029_1_5_

Mean	SD	Min	Max	Obs
1,600.00	.	1,600.00	1,600.00	1

ce029_1_6__min: Min Bracket of ce029_1_6_

Mean	SD	Min	Max	Obs
200.00	.	200.00	200.00	1

ce029_1_6__max: Max Bracket of ce029_1_6_

Mean	SD	Min	Max	Obs
200.00	.	200.00	200.00	1

ce029_1_7__min: Min Bracket of ce029_1_7_

No Observations

ce029_1_7__max: Max Bracket of ce029_1_7_

No Observations

ce029_1_8__min: Min Bracket of ce029_1_8_

No Observations

ce029_1_8__max: Max Bracket of ce029_1_8_

No Observations

ce029_2_1__min: Min Bracket of ce029_2_1_

Mean	SD	Min	Max	Obs
1,600.00	.	1,600.00	1,600.00	1

ce029_2_1__max: Max Bracket of ce029_2_1_

Mean	SD	Min	Max	Obs
100.00	0.00	100.00	100.00	7

ce029_2_2__min: Min Bracket of ce029_2_2_

Mean	SD	Min	Max	Obs
560.00	589.92	200.00	1,600.00	5

ce029_2_2__max: Max Bracket of ce029_2_2_

Mean	SD	Min	Max	Obs
583.33	552.87	100.00	1,600.00	6

ce029_2_3__min: Min Bracket of ce029_2_3_

No Observations

ce029_2_3__max: Max Bracket of ce029_2_3_

No Observations

ce029_2_4__min: Min Bracket of ce029_2_4_

No Observations

ce029_2_4__max: Max Bracket of ce029_2_4_

Mean	SD	Min	Max	Obs

100.00	0.00	100.00	100.00	2

ce029_2_5__min: Min Bracket of ce029_2_5_

Mean	SD	Min	Max	Obs
800.00	.	800.00	800.00	1

ce029_2_5__max: Max Bracket of ce029_2_5_

Mean	SD	Min	Max	Obs
1,600.00	.	1,600.00	1,600.00	1

ce029_2_6__min: Min Bracket of ce029_2_6_

Mean	SD	Min	Max	Obs
200.00	.	200.00	200.00	1

ce029_2_6__max: Max Bracket of ce029_2_6_

Mean	SD	Min	Max	Obs
200.00	.	200.00	200.00	1

ce029_2_7__min: Min Bracket of ce029_2_7_

No Observations

ce029_2_7__max: Max Bracket of ce029_2_7_

No Observations

ce029_2_8__min: Min Bracket of ce029_2_8_

No Observations

ce029_2_8__max: Max Bracket of ce029_2_8_

No Observations

ce029_3_1__min: Min Bracket of ce029_3_1_

Mean	SD	Min	Max	Obs
842.65	613.36	100.00	1,600.00	68

ce029_3_1__max: Max Bracket of ce029_3_1_

Mean	SD	Min	Max	Obs

586.21	527.64	100.00	1,600.00	58

ce029_3_2__min: Min Bracket of ce029_3_2_

Mean	SD	Min	Max	Obs
853.57	569.91	100.00	1,600.00	28

ce029_3_2__max: Max Bracket of ce029_3_2_

Mean	SD	Min	Max	Obs
715.00	568.72	100.00	1,600.00	20

ce029_3_3__min: Min Bracket of ce029_3_3_

Mean	SD	Min	Max	Obs
576.92	500.26	100.00	1,600.00	13

ce029_3_3__max: Max Bracket of ce029_3_3_

Mean	SD	Min	Max	Obs
560.00	483.74	100.00	1,600.00	15

ce029_3_4__min: Min Bracket of ce029_3_4_

Mean	SD	Min	Max	Obs
680.00	558.57	200.00	1,600.00	5

ce029_3_4__max: Max Bracket of ce029_3_4_

Mean	SD	Min	Max	Obs
400.00	328.63	100.00	800.00	6

ce029_3_5__min: Min Bracket of ce029_3_5_

Mean	SD	Min	Max	Obs
400.00	0.00	400.00	400.00	2

ce029_3_5__max: Max Bracket of ce029_3_5_

Mean	SD	Min	Max	Obs
400.00	0.00	400.00	400.00	2

ce029_3_6__min: Min Bracket of ce029_3_6_

Mean	SD	Min	Max	Obs
100.00	0.00	100.00	100.00	2

ce029_3_6__max: Max Bracket of ce029_3_6_

Mean	SD	Min	Max	Obs
100.00	0.00	100.00	100.00	2

ce029_3_7__min: Min Bracket of ce029_3_7_

No Observations

ce029_3_7__max: Max Bracket of ce029_3_7_

No Observations

ce029_3_8__min: Min Bracket of ce029_3_8_

No Observations

ce029_3_8__max: Max Bracket of ce029_3_8_

No Observations

ce029_4_1__min: Min Bracket of ce029_4_1_

Mean	SD	Min	Max	Obs
400.00	498.57	100.00	1,600.00	8

ce029_4_1__max: Max Bracket of ce029_4_1_

Mean	SD	Min	Max	Obs
177.78	183.29	100.00	800.00	18

ce029_4_2__min: Min Bracket of ce029_4_2_

Mean	SD	Min	Max	Obs
900.00	989.95	200.00	1,600.00	2

ce029_4_2__max: Max Bracket of ce029_4_2_

Mean	SD	Min	Max	Obs
560.00	650.38	100.00	1,600.00	5

ce029_4_3__min: Min Bracket of ce029_4_3_

Mean	SD	Min	Max	Obs
733.33	757.19	200.00	1,600.00	3

ce029_4_3__max: Max Bracket of ce029_4_3_

Mean	SD	Min	Max	Obs
575.00	694.62	100.00	1,600.00	4

ce029_4_4__min: Min Bracket of ce029_4_4_

Mean	SD	Min	Max	Obs
1,600.00	.	1,600.00	1,600.00	1

ce029_4_4__max: Max Bracket of ce029_4_4_

Mean	SD	Min	Max	Obs
600.00	866.03	100.00	1,600.00	3

ce029_4_5__min: Min Bracket of ce029_4_5_

Mean	SD	Min	Max	Obs
100.00	.	100.00	100.00	1

ce029_4_5__max: Max Bracket of ce029_4_5_

Mean	SD	Min	Max	Obs
100.00	.	100.00	100.00	1

ce029_4_6__min: Min Bracket of ce029_4_6_

Mean	SD	Min	Max	Obs
400.00	.	400.00	400.00	1

ce029_4_6__max: Max Bracket of ce029_4_6_

Mean	SD	Min	Max	Obs
400.00	.	400.00	400.00	1

ce029_4_7__min: Min Bracket of ce029_4_7_

No Observations

ce029_4_7__max: Max Bracket of ce029_4_7_

No Observations

ce029_4_8__min: Min Bracket of ce029_4_8_

No Observations

ce029_4_8__max: Max Bracket of ce029_4_8_

No Observations

ce036: Economic Supports for Relatives

Mean	SD	Min	Max	Obs
246.09	1,715.55	0.00	60,000.00	11,479

ce036_min: Min Bracket of ce036

Mean	SD	Min	Max	Obs
870.97	554.49	100.00	1,600.00	31

ce036_max: Max Bracket of ce036

Mean	SD	Min	Max	Obs
783.33	577.27	100.00	1,600.00	18

ce036_w3: Cash Gift for Relatives

Mean	SD	Min	Max	Obs
1,866.45	6,995.37	0.00	600,000.00	11,324

ce036_w3_min: Min Bracket of ce036_w3

Mean	SD	Min	Max	Obs
997.30	590.85	100.00	1,600.00	148

ce036_w3_max: Max Bracket of ce036_w3

Mean	SD	Min	Max	Obs
790.70	542.03	100.00	1,600.00	86

ce066_w2_1_1_: Which Year XChildName[1] got Married

Mean	SD	Min	Max	Obs
1,996.05	260.32	-9,999.00	2,018.00	8,516

ce066_w2_2_1_: Which Month XChildName[1] got Married

Mean	SD	Min	Max	Obs
6.04	4.44	0.00	12.00	8,411

ce066_w2_1_2_: Which Year XChildName[2] got Married

Mean	SD	Min	Max	Obs
1,997.40	202.07	-9,999.00	2,018.00	7,078

ce066_w2_2_2_: Which Month XChildName[2] got Married

Mean	SD	Min	Max	Obs
5.79	4.54	0.00	12.00	6,991

ce066_w2_1_3_: Which Year XChildName[3] got Married

Mean	SD	Min	Max	Obs
1,998.57	12.71	1,900.00	2,018.00	4,267

ce066_w2_2_3_: Which Month XChildName[3] got Married

Mean	SD	Min	Max	Obs
5.36	4.67	0.00	12.00	4,204

ce066_w2_1_4_: Which Year XChildName[4] got Married

Mean	SD	Min	Max	Obs
1,995.94	13.70	1,900.00	2,018.00	2,282

ce066_w2_2_4_: Which Month XChildName[4] got Married

Mean	SD	Min	Max	Obs
5.03	4.71	0.00	12.00	2,248

ce066_w2_1_5_: Which Year XChildName[5] got Married

Mean	SD	Min	Max	Obs
1,994.96	14.08	1,900.00	2,018.00	1,127

ce066_w2_2_5_: Which Month XChildName[5] got Married

Mean	SD	Min	Max	Obs
4.87	4.83	0.00	12.00	1,109

ce066_w2_1_6_: Which Year XChildName[6] got Married

Mean	SD	Min	Max	Obs
1,994.12	13.95	1,900.00	2,018.00	498

ce066_w2_2_6_: Which Month XChildName[6] got Married

Mean	SD	Min	Max	Obs
4.34	4.68	0.00	12.00	490

ce066_w2_1_7_: Which Year XChildName[7] got Married

Mean	SD	Min	Max	Obs
1,995.44	10.21	1,965.00	2,018.00	225

ce066_w2_2_7_: Which Month XChildName[7] got Married

Mean	SD	Min	Max	Obs
4.48	4.57	0.00	12.00	223

ce066_w2_1_8_: Which Year XChildName[8] got Married

Mean	SD	Min	Max	Obs
1,994.50	14.98	1,900.00	2,018.00	96

ce066_w2_2_8_: Which Month XChildName[8] got Married

Mean	SD	Min	Max	Obs
3.71	4.60	0.00	12.00	98

ce066_w2_1_9_: Which Year XChildName[9] got Married

Mean	SD	Min	Max	Obs
1,994.24	10.64	1,970.00	2,018.00	45

ce066_w2_2_9_: Which Month XChildName[9] got Married

Mean	SD	Min	Max	Obs
2.91	4.16	0.00	12.00	43

ce066_w2_1_10_: Which Year XChildName[10] got Married

Mean	SD	Min	Max	Obs
1,991.96	10.85	1,970.00	2,012.00	26

ce066_w2_2_10_: Which Month XChildName[10] got Married

Mean	SD	Min	Max	Obs
4.42	4.78	0.00	12.00	26

ce066_w2_1_11_: Which Year XChildName[11] got Married

Mean	SD	Min	Max	Obs
1,991.00	12.92	1,963.00	2,009.00	15

ce066_w2_2_11_: Which Month XChildName[11] got Married

Mean	SD	Min	Max	Obs
4.64	5.00	0.00	12.00	14

ce066_w2_1_12_: Which Year XChildName[12] got Married

Mean	SD	Min	Max	Obs
1,992.38	13.05	1,970.00	2,010.00	8

ce066_w2_2_12_: Which Month XChildName[12] got Married

Mean	SD	Min	Max	Obs
3.38	5.37	0.00	12.00	8

ce066_w2_1_13_: Which Year XChildName[13] got Married

Mean	SD	Min	Max	Obs
1,989.50	0.71	1,989.00	1,990.00	2

ce066_w2_2_13_: Which Month XChildName[13] got Married

Mean	SD	Min	Max	Obs
8.00	5.66	4.00	12.00	2

ce066_w2_1_14_: Which Year XChildName[14] got Married

Mean	SD	Min	Max	Obs
1,991.00	2.83	1,989.00	1,993.00	2

ce066_w2_2_14_: Which Month XChildName[14] got Married

Mean	SD	Min	Max	Obs
5.50	0.71	5.00	6.00	2

ce066_w2_1_15_: Which Year XChildName[15] got Married

Mean	SD	Min	Max	Obs
1,994.00	.	1,994.00	1,994.00	1

ce066_w2_2_15_: Which Month XChildName[15] got Married

Mean	SD	Min	Max	Obs
10.00	.	10.00	10.00	1

ce067_w2_1_1_: Betrothal Gifts for XChildName[1]

	Freq.	%
1 Yes	5,335	61.36
2 No	3,360	38.64
Total	8,695	100.00

ce067_w2_1_2_: Betrothal Gifts for XChildName[2]

	Freq.	%
1 Yes	4,008	54.75
2 No	3,312	45.25
Total	7,320	100.00

ce067_w2_1_3_: Betrothal Gifts for XChildName[3]

	Freq.	%
1 Yes	2,159	48.16
2 No	2,324	51.84
Total	4,483	100.00

ce067_w2_1_4_: Betrothal Gifts for XChildName[4]

	Freq.	%
1 Yes	1,093	44.69
2 No	1,353	55.31
Total	2,446	100.00

ce067_w2_1_5_: Betrothal Gifts for XChildName[5]

	Freq.	%
1 Yes	482	39.41
2 No	741	60.59
Total	1,223	100.00

ce067_w2_1_6_: Betrothal Gifts for XChildName[6]

	Freq.	%
1 Yes	205	37.82
2 No	337	62.18
Total	542	100.00

ce067_w2_1_7_: Betrothal Gifts for XChildName[7]

	Freq.	%
1 Yes	90	35.71
2 No	162	64.29
Total	252	100.00

ce067_w2_1_8_: Betrothal Gifts for XChildName[8]

	Freq.	%
1 Yes	48	42.48
2 No	65	57.52
Total	113	100.00

ce067_w2_1_9_: Betrothal Gifts for XChildName[9]

	Freq.	%
1 Yes	20	40.00
2 No	30	60.00
Total	50	100.00

ce067_w2_1_10_: Betrothal Gifts for XChildName[10]

	Freq.	%
1 Yes	12	41.38
2 No	17	58.62
Total	29	100.00

ce067_w2_1_11_: Betrothal Gifts for XChildName[11]

	Freq.	%
1 Yes	9	60.00
2 No	6	40.00
Total	15	100.00

ce067_w2_1_12_: Betrothal Gifts for XChildName[12]

	Freq.	%
1 Yes	3	37.50
2 No	5	62.50
Total	8	100.00

ce067_w2_1_13_: Betrothal Gifts for XChildName[13]

	Freq.	%
1 Yes	2	100.00
Total	2	100.00

ce067_w2_1_14_: Betrothal Gifts for XChildName[14]

	Freq.	%
1 Yes	2	100.00
Total	2	100.00

ce067_w2_1_15_: Betrothal Gifts for XChildName[15]

	Freq.	%
1 Yes	1	100.00
Total	1	100.00

ce068_w2_1_1_: Total Value of Betrothal Gift

Mean	SD	Min	Max	Obs
21,610.31	39,193.03	0.00	600,000.00	5,174

ce068_w2_1_2_: Total Value of Betrothal Gift

Mean	SD	Min	Max	Obs
15,410.88	28,161.71	0.00	500,000.00	3,877

ce068_w2_1_3_: Total Value of Betrothal Gift

Mean	SD	Min	Max	Obs
9,933.79	20,537.92	0.00	250,000.00	2,070

ce068_w2_1_4_: Total Value of Betrothal Gift

Mean	SD	Min	Max	Obs
7,221.54	15,148.46	0.00	160,000.00	1,022

ce068_w2_1_5_: Total Value of Betrothal Gift

Mean	SD	Min	Max	Obs
4,731.18	9,344.78	0.00	100,000.00	450

ce068_w2_1_6_: Total Value of Betrothal Gift

Mean	SD	Min	Max	Obs
5,624.93	12,719.47	0.00	100,000.00	189

ce068_w2_1_7_: Total Value of Betrothal Gift

Mean	SD	Min	Max	Obs
9,128.47	25,830.72	0.00	160,000.00	79

ce068_w2_1_8_: Total Value of Betrothal Gift

Mean	SD	Min	Max	Obs
5,800.51	9,456.82	0.00	40,000.00	41

ce068_w2_1_9_: Total Value of Betrothal Gift

Mean	SD	Min	Max	Obs
5,158.82	7,567.95	0.00	24,000.00	17

ce068_w2_1_10_: Total Value of Betrothal Gift

Mean	SD	Min	Max	Obs
6,421.91	14,300.67	0.00	48,666.00	11

ce068_w2_1_11_: Total Value of Betrothal Gift

Mean	SD	Min	Max	Obs
4,594.44	6,560.60	0.00	20,000.00	9

ce068_w2_1_12_: Total Value of Betrothal Gift

Mean	SD	Min	Max	Obs
2,833.33	763.76	2,000.00	3,500.00	3

ce068_w2_1_13_: Total Value of Betrothal Gift

Mean	SD	Min	Max	Obs
3,000.00	0.00	3,000.00	3,000.00	2

ce068_w2_1_14_: Total Value of Betrothal Gift

Mean	SD	Min	Max	Obs
2,000.00	2,121.32	500.00	3,500.00	2

ce068_w2_1_15_: Total Value of Betrothal Gift

Mean	SD	Min	Max	Obs
600.00	.	600.00	600.00	1

ce069_w2_1_1_: Buy House for XChildName[1]

	Freq.	%
1 Yes	889	10.22
2 No	7,806	89.78
Total	8,695	100.00

ce069_w2_1_2_: Buy House for XChildName[2]

	Freq.	%
1 Yes	508	6.94
2 No	6,811	93.06
Total	7,319	100.00

ce069_w2_1_3_: Buy House for XChildName[3]

	Freq.	%
1 Yes	214	4.77
2 No	4,269	95.23
Total	4,483	100.00

ce069_w2_1_4_: Buy House for XChildName[4]

	Freq.	%
1 Yes	105	4.29
2 No	2,341	95.71
Total	2,446	100.00

ce069_w2_1_5_: Buy House for XChildName[5]

	Freq.	%
1 Yes	35	2.86
2 No	1,188	97.14
Total	1,223	100.00

ce069_w2_1_6_: Buy House for XChildName[6]

	Freq.	%
1 Yes	25	4.61
2 No	517	95.39
Total	542	100.00

ce069_w2_1_7_: Buy House for XChildName[7]

	Freq.	%
1 Yes	7	2.78
2 No	245	97.22
Total	252	100.00

ce069_w2_1_8_: Buy House for XChildName[8]

	Freq.	%
1 Yes	3	2.65
2 No	110	97.35
Total	113	100.00

ce069_w2_1_9_: Buy House for XChildName[9]

	Freq.	%
1 Yes	1	2.00
2 No	49	98.00
Total	50	100.00

ce069_w2_1_10_: Buy House for XChildName[10]

	Freq.	%
2 No	29	100.00
Total	29	100.00

ce069_w2_1_11_: Buy House for XChildName[11]

	Freq.	%
2 No	15	100.00
Total	15	100.00

ce069_w2_1_12_: Buy House for XChildName[12]

	Freq.	%
2 No	8	100.00
Total	8	100.00

ce069_w2_1_13_: Buy House for XChildName[13]

	Freq.	%
2 No	2	100.00
Total	2	100.00

ce069_w2_1_14_: Buy House for XChildName[14]

	Freq.	%
2 No	2	100.00
Total	2	100.00

ce069_w2_1_15_: Buy House for XChildName[15]

	Freq.	%
2 No	1	100.00
Total	1	100.00

ce070_w2_1_1_: Total Value of House for XChildName[1]

Mean	SD	Min	Max	Obs
11,904.54	74,983.34	0.00	850,000.00	852

ce070_w2_1_2_: Total Value of House for XChildName[2]

Mean	SD	Min	Max	Obs
7,210.03	40,412.01	0.00	500,000.00	481

ce070_w2_1_3_: Total Value of House for XChildName[3]

Mean	SD	Min	Max	Obs
9,317.99	47,586.39	0.00	420,000.00	198

ce070_w2_1_4_: Total Value of House for XChildName[4]

Mean	SD	Min	Max	Obs
1,671.28	12,223.47	0.00	110,000.00	91

ce070_w2_1_5_: Total Value of House for XChildName[5]

Mean	SD	Min	Max	Obs
3,135.60	12,810.85	0.02	70,000.00	31

ce070_w2_1_6_: Total Value of House for XChildName[6]

Mean	SD	Min	Max	Obs
4,602.30	20,421.38	0.02	100,000.00	24

ce070_w2_1_7_: Total Value of House for XChildName[7]

Mean	SD	Min	Max	Obs
0.73	1.02	0.03	3.00	7

ce070_w2_1_8_: Total Value of House for XChildName[8]

Mean	SD	Min	Max	Obs
1.88	2.72	0.03	5.00	3

ce070_w2_1_9_: Total Value of House for XChildName[9]

No Observations

ce072_w2_1_1_: Total Money Support from Siblings[1]

Mean	SD	Min	Max	Obs
877.50	4,455.54	0.00	100,000.00	677

ce072_w2_1_2_: Total Money Support from Siblings[2]

Mean	SD	Min	Max	Obs
555.25	1,390.17	0.00	20,000.00	711

ce072_w2_1_3_: Total Money Support from Siblings[3]

Mean	SD	Min	Max	Obs
718.82	3,226.73	0.00	50,000.00	624

ce072_w2_1_4_: Total Money Support from Siblings[4]

Mean	SD	Min	Max	Obs
614.13	2,225.31	0.00	30,000.00	466

ce072_w2_1_5_: Total Money Support from Siblings[5]

Mean	SD	Min	Max	Obs
555.21	3,112.26	0.00	48,000.00	290

ce072_w2_1_6_: Total Money Support from Siblings[6]

Mean	SD	Min	Max	Obs
1,291.62	8,643.86	0.00	100,000.00	139

ce072_w2_1_7_: Total Money Support from Siblings[7]

Mean	SD	Min	Max	Obs
550.60	1,301.38	0.00	7,000.00	67

ce072_w2_1_8_: Total Money Support from Siblings[8]

Mean	SD	Min	Max	Obs
338.89	653.62	0.00	2,000.00	18

ce072_w2_1_9_: Total Money Support from Siblings[9]

Mean	SD	Min	Max	Obs
50.00	100.00	0.00	200.00	4

ce072_w2_1_10_: Total Money Support from Siblings[10]

No Observations

ce072_w2_1_11_: Total Money Support from Siblings[11]

Mean	SD	Min	Max	Obs
200.00	.	200.00	200.00	1

ce072_w2_1_16_: Total Money Support from Siblings[16]

Mean	SD	Min	Max	Obs
763.97	5,349.95	0.00	100,000.00	407

ce072_w2_1_17_: Total Money Support from Siblings[17]

Mean	SD	Min	Max	Obs
532.17	2,868.63	0.00	50,000.00	405

ce072_w2_1_18_: Total Money Support from Siblings[18]

Mean	SD	Min	Max	Obs
573.58	2,449.49	0.00	30,000.00	377

ce072_w2_1_19_: Total Money Support from Siblings[19]

Mean	SD	Min	Max	Obs
540.26	2,220.35	0.00	30,000.00	274

ce072_w2_1_20_: Total Money Support from Siblings[20]

Mean	SD	Min	Max	Obs
370.75	1,148.61	0.00	10,000.00	173

ce072_w2_1_21_: Total Money Support from Siblings[21]

Mean	SD	Min	Max	Obs
393.00	1,348.71	0.00	10,000.00	90

ce072_w2_1_22_: Total Money Support from Siblings[22]

Mean	SD	Min	Max	Obs
818.11	3,292.77	0.00	20,000.00	37

ce072_w2_1_23_: Total Money Support from Siblings[23]

Mean	SD	Min	Max	Obs
134.38	179.55	0.00	500.00	16

ce072_w2_1_24_: Total Money Support from Siblings[24]

Mean	SD	Min	Max	Obs
175.00	125.83	0.00	300.00	4

ce072_w2_1_25_: Total Money Support from Siblings[25]

Mean	SD	Min	Max	Obs
100.00	141.42	0.00	200.00	2

ce072_w2_1_26_: Total Money Support from Siblings[26]

Mean	SD	Min	Max	Obs
100.00	141.42	0.00	200.00	2

ce072_w2_1_27_: Total Money Support from Siblings[27]

Mean	SD	Min	Max	Obs
200.00	.	200.00	200.00	1

ce072_w2_2_1_: Regular Money Support from Siblings[1]

Mean	SD	Min	Max	Obs
72.75	552.10	0.00	11,000.00	680

ce072_w2_2_2_: Regular Money Support from Siblings[2]

Mean	SD	Min	Max	Obs
28.22	187.54	0.00	4,000.00	713

ce072_w2_2_3_: Regular Money Support from Siblings[3]

Mean	SD	Min	Max	Obs
34.94	214.38	0.00	4,000.00	628

ce072_w2_2_4_: Regular Money Support from Siblings[4]

Mean	SD	Min	Max	Obs
74.16	944.52	0.00	20,000.00	468

ce072_w2_2_5_: Regular Money Support from Siblings[5]

Mean	SD	Min	Max	Obs
17.53	69.62	0.00	500.00	291

ce072_w2_2_6_: Regular Money Support from Siblings[6]

Mean	SD	Min	Max	Obs
32.86	152.50	0.00	1,500.00	140

ce072_w2_2_7_: Regular Money Support from Siblings[7]

Mean	SD	Min	Max	Obs
119.40	855.33	0.00	7,000.00	67

ce072_w2_2_8_: Regular Money Support from Siblings[8]

Mean	SD	Min	Max	Obs
0.00	0.00	0.00	0.00	18

ce072_w2_2_9_: Regular Money Support from Siblings[9]

Mean	SD	Min	Max	Obs
0.00	0.00	0.00	0.00	4

ce072_w2_2_10_: Regular Money Support from Siblings[10]

No Observations

ce072_w2_2_11_: Regular Money Support from Siblings[11]

Mean	SD	Min	Max	Obs
0.00	.	0.00	0.00	1

ce072_w2_2_16_: Regular Money Support from Siblings[16]

Mean	SD	Min	Max	Obs
39.56	314.89	0.00	5,000.00	411

ce072_w2_2_17_: Regular Money Support from Siblings[17]

Mean	SD	Min	Max	Obs
33.09	195.52	0.00	2,000.00	408

ce072_w2_2_18_: Regular Money Support from Siblings[18]

Mean	SD	Min	Max	Obs
119.55	1,425.83	0.00	25,000.00	379

ce072_w2_2_19_: Regular Money Support from Siblings[19]

Mean	SD	Min	Max	Obs
62.12	626.46	0.00	10,000.00	274

ce072_w2_2_20_: Regular Money Support from Siblings[20]

Mean	SD	Min	Max	Obs
10.69	52.42	0.00	500.00	173

ce072_w2_2_21_: Regular Money Support from Siblings[21]

Mean	SD	Min	Max	Obs
18.33	110.27	0.00	1,000.00	90

ce072_w2_2_22_: Regular Money Support from Siblings[22]

Mean	SD	Min	Max	Obs
17.57	85.16	0.00	500.00	37

ce072_w2_2_23_: Regular Money Support from Siblings[23]

Mean	SD	Min	Max	Obs
0.00	0.00	0.00	0.00	16

ce072_w2_2_24_: Regular Money Support from Siblings[24]

Mean	SD	Min	Max	Obs
0.00	0.00	0.00	0.00	4

ce072_w2_2_25_: Regular Money Support from Siblings[25]

Mean	SD	Min	Max	Obs
0.00	0.00	0.00	0.00	2

ce072_w2_2_26_: Regular Money Support from Siblings[26]

Mean	SD	Min	Max	Obs
0.00	0.00	0.00	0.00	2

ce072_w2_2_27_: Regular Money Support from Siblings[27]

Mean	SD	Min	Max	Obs
0.00	.	0.00	0.00	1

ce072_w2_3_1_: Total In_kind Support from Siblings[1]

Mean	SD	Min	Max	Obs
234.77	712.48	0.00	10,000.00	664

ce072_w2_3_2_: Total In_kind Support from Siblings[2]

Mean	SD	Min	Max	Obs
290.27	1,094.49	0.00	15,000.00	697

ce072_w2_3_3_: Total In_kind Support from Siblings[3]

Mean	SD	Min	Max	Obs
214.03	576.11	0.00	8,000.00	615

ce072_w2_3_4_: Total In_kind Support from Siblings[4]

Mean	SD	Min	Max	Obs
207.94	549.20	0.00	8,000.00	456

ce072_w2_3_5_: Total In_kind Support from Siblings[5]

Mean	SD	Min	Max	Obs
154.60	382.72	0.00	5,000.00	286

ce072_w2_3_6_: Total In_kind Support from Siblings[6]

Mean	SD	Min	Max	Obs
211.03	578.54	0.00	5,000.00	136

ce072_w2_3_7_: Total In_kind Support from Siblings[7]

Mean	SD	Min	Max	Obs
144.18	318.02	0.00	2,000.00	67

ce072_w2_3_8_: Total In_kind Support from Siblings[8]

Mean	SD	Min	Max	Obs
183.33	264.89	0.00	1,000.00	18

ce072_w2_3_9_: Total In_kind Support from Siblings[9]

Mean	SD	Min	Max	Obs
175.00	50.00	100.00	200.00	4

ce072_w2_3_10_: Total In_kind Support from Siblings[10]

No Observations

ce072_w2_3_11_: Total In_kind Support from Siblings[11]

Mean	SD	Min	Max	Obs
300.00	.	300.00	300.00	1

ce072_w2_3_16_: Total In_kind Support from Siblings[16]

Mean	SD	Min	Max	Obs
185.29	421.29	0.00	3,000.00	399

ce072_w2_3_17_: Total In_kind Support from Siblings[17]

Mean	SD	Min	Max	Obs
160.64	398.09	0.00	5,000.00	399

ce072_w2_3_18_: Total In_kind Support from Siblings[18]

Mean	SD	Min	Max	Obs
189.02	610.81	0.00	10,000.00	374

ce072_w2_3_19_: Total In_kind Support from Siblings[19]

Mean	SD	Min	Max	Obs
191.53	515.20	0.00	6,000.00	271

ce072_w2_3_20_: Total In_kind Support from Siblings[20]

Mean	SD	Min	Max	Obs
122.83	306.67	0.00	3,000.00	171

ce072_w2_3_21_: Total In_kind Support from Siblings[21]

Mean	SD	Min	Max	Obs
473.07	3,097.00	0.00	29,000.00	88

ce072_w2_3_22_: Total In_kind Support from Siblings[22]

Mean	SD	Min	Max	Obs
131.33	346.02	0.00	2,000.00	36

ce072_w2_3_23_: Total In_kind Support from Siblings[23]

Mean	SD	Min	Max	Obs
53.13	62.15	0.00	200.00	16

ce072_w2_3_24_: Total In_kind Support from Siblings[24]

Mean	SD	Min	Max	Obs
25.00	28.87	0.00	50.00	4

ce072_w2_3_25_: Total In_kind Support from Siblings[25]

Mean	SD	Min	Max	Obs
0.00	0.00	0.00	0.00	2

ce072_w2_3_26_: Total In_kind Support from Siblings[26]

Mean	SD	Min	Max	Obs
50.00	70.71	0.00	100.00	2

ce072_w2_3_27_: Total In_kind Support from Siblings[27]

Mean	SD	Min	Max	Obs
0.00	.	0.00	0.00	1

ce072_w2_4_1_: Regular In_kind Support from Siblings[1]

Mean	SD	Min	Max	Obs
25.19	141.06	0.00	2,000.00	678

ce072_w2_4_2_: Regular In_kind Support from Siblings[2]

Mean	SD	Min	Max	Obs
13.41	76.92	0.00	1,000.00	709

ce072_w2_4_3_: Regular In_kind Support from Siblings[3]

Mean	SD	Min	Max	Obs
14.34	82.16	0.00	1,200.00	624

ce072_w2_4_4_: Regular In_kind Support from Siblings[4]

Mean	SD	Min	Max	Obs
14.35	79.39	0.00	1,000.00	468

ce072_w2_4_5_: Regular In_kind Support from Siblings[5]

Mean	SD	Min	Max	Obs
12.17	63.57	0.00	800.00	290

ce072_w2_4_6_: Regular In_kind Support from Siblings[6]

Mean	SD	Min	Max	Obs
7.21	36.21	0.00	300.00	140

ce072_w2_4_7_: Regular In_kind Support from Siblings[7]

Mean	SD	Min	Max	Obs
5.67	37.18	0.00	300.00	67

ce072_w2_4_8_: Regular In_kind Support from Siblings[8]

Mean	SD	Min	Max	Obs
0.00	0.00	0.00	0.00	18

ce072_w2_4_9_: Regular In_kind Support from Siblings[9]

Mean	SD	Min	Max	Obs
0.00	0.00	0.00	0.00	4

ce072_w2_4_10_: Regular In_kind Support from Siblings[10]

No Observations

ce072_w2_4_11_: Regular In_kind Support from Siblings[11]

Mean	SD	Min	Max	Obs
0.00	.	0.00	0.00	1

ce072_w2_4_16_: Regular In_kind Support from Siblings[16]

Mean	SD	Min	Max	Obs
11.38	72.90	0.00	1,000.00	409

ce072_w2_4_17_: Regular In_kind Support from Siblings[17]

Mean	SD	Min	Max	Obs
13.00	73.95	0.00	1,000.00	406

ce072_w2_4_18_: Regular In_kind Support from Siblings[18]

Mean	SD	Min	Max	Obs
37.77	518.74	0.00	10,000.00	377

ce072_w2_4_19_: Regular In_kind Support from Siblings[19]

Mean	SD	Min	Max	Obs
32.60	369.33	0.00	6,000.00	273

ce072_w2_4_20_: Regular In_kind Support from Siblings[20]

Mean	SD	Min	Max	Obs
6.10	32.99	0.00	300.00	172

ce072_w2_4_21_: Regular In_kind Support from Siblings[21]

Mean	SD	Min	Max	Obs
22.36	119.40	0.00	1,000.00	89

ce072_w2_4_22_: Regular In_kind Support from Siblings[22]

Mean	SD	Min	Max	Obs
2.78	11.62	0.00	50.00	36

ce072_w2_4_23_: Regular In_kind Support from Siblings[23]

Mean	SD	Min	Max	Obs
6.25	25.00	0.00	100.00	16

ce072_w2_4_24_: Regular In_kind Support from Siblings[24]

Mean	SD	Min	Max	Obs
0.00	0.00	0.00	0.00	4

ce072_w2_4_25_: Regular In_kind Support from Siblings[25]

Mean	SD	Min	Max	Obs
0.00	0.00	0.00	0.00	2

ce072_w2_4_26_: Regular In_kind Support from Siblings[26]

Mean	SD	Min	Max	Obs
0.00	0.00	0.00	0.00	2

ce072_w2_4_27_: Regular In_kind Support from Siblings[27]

Mean	SD	Min	Max	Obs
0.00	.	0.00	0.00	1

ce072_w2_1_1__min: Min Bracket of ce072_w2_1_1_'

Mean	SD	Min	Max	Obs
1,000.00	692.82	400.00	1,600.00	4

ce072_w2_1_1__max: Max Bracket of ce072_w2_1_1_

Mean	SD	Min	Max	Obs
1,066.67	461.88	800.00	1,600.00	3

ce072_w2_1_2__min: Min Bracket of ce072_w2_1_2_'

Mean	SD	Min	Max	Obs
1,000.00	848.53	400.00	1,600.00	2

ce072_w2_1_2__max: Max Bracket of ce072_w2_1_2_

Mean	SD	Min	Max	Obs
400.00	.	400.00	400.00	1

ce072_w2_1_3__min: Min Bracket of ce072_w2_1_3_'

Mean	SD	Min	Max	Obs
1,000.00	848.53	400.00	1,600.00	2

ce072_w2_1_3__max: Max Bracket of ce072_w2_1_3_

No Observations

ce072_w2_1_4__min: Min Bracket of ce072_w2_1_4_'

No Observations

ce072_w2_1_4__max: Max Bracket of ce072_w2_1_4_

Mean	SD	Min	Max	Obs
100.00	.	100.00	100.00	1

ce072_w2_1_5__min: Min Bracket of ce072_w2_1_5_'

No Observations

ce072_w2_1_5__max: Max Bracket of ce072_w2_1_5_

No Observations

ce072_w2_1_6__min: Min Bracket of ce072_w2_1_6_'

No Observations

ce072_w2_1_6__max: Max Bracket of ce072_w2_1_6_

No Observations

ce072_w2_1_7__min: Min Bracket of ce072_w2_1_7_'

No Observations

ce072_w2_1_7__max: Max Bracket of ce072_w2_1_7_

No Observations

ce072_w2_1_8__min: Min Bracket of ce072_w2_1_8_'

No Observations

ce072_w2_1_8__max: Max Bracket of ce072_w2_1_8_

No Observations

ce072_w2_1_9__min: Min Bracket of ce072_w2_1_9_'

No Observations

ce072_w2_1_9__max: Max Bracket of ce072_w2_1_9_

No Observations

ce072_w2_1_10__min: Min Bracket of ce072_w2_1_10_'

No Observations

ce072_w2_1_10__max: Max Bracket of ce072_w2_1_10_

No Observations

ce072_w2_1_16__min: Min Bracket of ce072_w2_1_16_'

Mean	SD	Min	Max	Obs

400.00	.	400.00	400.00	1

ce072_w2_1_16__max: Max Bracket of ce072_w2_1_16_

Mean	SD	Min	Max	Obs
250.00	212.13	100.00	400.00	2

ce072_w2_1_17__min: Min Bracket of ce072_w2_1_17_'

Mean	SD	Min	Max	Obs
500.00	424.26	200.00	800.00	2

ce072_w2_1_17__max: Max Bracket of ce072_w2_1_17_

Mean	SD	Min	Max	Obs
600.00	282.84	400.00	800.00	2

ce072_w2_1_18__min: Min Bracket of ce072_w2_1_18_'

Mean	SD	Min	Max	Obs
400.00	.	400.00	400.00	1

ce072_w2_1_18__max: Max Bracket of ce072_w2_1_18_

Mean	SD	Min	Max	Obs
400.00	.	400.00	400.00	1

ce072_w2_2_1__min: Min Bracket of ce072_w2_2_1_'

No Observations

ce072_w2_2_1__max: Max Bracket of ce072_w2_2_1_

Mean	SD	Min	Max	Obs
100.00	.	100.00	100.00	1

ce072_w2_2_2__min: Min Bracket of ce072_w2_2_2_'

No Observations

ce072_w2_2_2__max: Max Bracket of ce072_w2_2_2_

Mean	SD	Min	Max	Obs
100.00	.	100.00	100.00	1

ce072_w2_2_3__min: Min Bracket of ce072_w2_2_3_'

No Observations

ce072_w2_2_3__max: Max Bracket of ce072_w2_2_3_

No Observations

ce072_w2_2_4__min: Min Bracket of ce072_w2_2_4_'

No Observations

ce072_w2_2_4__max: Max Bracket of ce072_w2_2_4_

Mean	SD	Min	Max	Obs
100.00	.	100.00	100.00	1

ce072_w2_2_5__min: Min Bracket of ce072_w2_2_5_'

No Observations

ce072_w2_2_5__max: Max Bracket of ce072_w2_2_5_

No Observations

ce072_w2_2_6__min: Min Bracket of ce072_w2_2_6_'

No Observations

ce072_w2_2_6__max: Max Bracket of ce072_w2_2_6_

No Observations

ce072_w2_2_7__min: Min Bracket of ce072_w2_2_7_'

No Observations

ce072_w2_2_7__max: Max Bracket of ce072_w2_2_7_

No Observations

ce072_w2_2_8__min: Min Bracket of ce072_w2_2_8_'

No Observations

ce072_w2_2_8__max: Max Bracket of ce072_w2_2_8_

No Observations

ce072_w2_2_9__min: Min Bracket of ce072_w2_2_9_'

No Observations

ce072_w2_2_9__max: Max Bracket of ce072_w2_2_9_

No Observations

ce072_w2_2_10__min: Min Bracket of ce072_w2_2_10_'

No Observations

ce072_w2_2_10__max: Max Bracket of ce072_w2_2_10_

No Observations

ce072_w2_2_16__min: Min Bracket of ce072_w2_2_16_'

No Observations

ce072_w2_2_16__max: Max Bracket of ce072_w2_2_16_

No Observations

ce072_w2_2_17__min: Min Bracket of ce072_w2_2_17_'

No Observations

ce072_w2_2_17__max: Max Bracket of ce072_w2_2_17_

No Observations

ce072_w2_2_18__min: Min Bracket of ce072_w2_2_18_'

No Observations

ce072_w2_2_18__max: Max Bracket of ce072_w2_2_18_

No Observations

ce072_w2_3_1__min: Min Bracket of ce072_w2_3_1_'

Mean	SD	Min	Max	Obs
255.56	142.40	100.00	400.00	9

ce072_w2_3_1__max: Max Bracket of ce072_w2_3_1_

Mean	SD	Min	Max	Obs
300.00	277.75	100.00	800.00	15

ce072_w2_3_2__min: Min Bracket of ce072_w2_3_2_'

Mean	SD	Min	Max	Obs
228.57	125.36	100.00	400.00	7

ce072_w2_3_2__max: Max Bracket of ce072_w2_3_2_

Mean	SD	Min	Max	Obs
262.50	118.77	100.00	400.00	8

ce072_w2_3_3__min: Min Bracket of ce072_w2_3_3_'

Mean	SD	Min	Max	Obs
362.50	213.39	100.00	800.00	8

ce072_w2_3_3__max: Max Bracket of ce072_w2_3_3_

Mean	SD	Min	Max	Obs
425.00	249.28	200.00	800.00	8

ce072_w2_3_4__min: Min Bracket of ce072_w2_3_4_'

Mean	SD	Min	Max	Obs
625.00	665.21	100.00	1,600.00	4

ce072_w2_3_4__max: Max Bracket of ce072_w2_3_4_

Mean	SD	Min	Max	Obs
516.67	545.59	100.00	1,600.00	6

ce072_w2_3_5__min: Min Bracket of ce072_w2_3_5_'

Mean	SD	Min	Max	Obs
250.00	212.13	100.00	400.00	2

ce072_w2_3_5__max: Max Bracket of ce072_w2_3_5_

Mean	SD	Min	Max	Obs
300.00	141.42	200.00	400.00	2

ce072_w2_3_6__min: Min Bracket of ce072_w2_3_6_'

Mean	SD	Min	Max	Obs
700.00	793.73	100.00	1,600.00	3

ce072_w2_3_6__max: Max Bracket of ce072_w2_3_6_

Mean	SD	Min	Max	Obs
700.00	793.73	100.00	1,600.00	3

ce072_w2_3_7__min: Min Bracket of ce072_w2_3_7_'

No Observations

ce072_w2_3_7__max: Max Bracket of ce072_w2_3_7_

No Observations

ce072_w2_3_8__min: Min Bracket of ce072_w2_3_8_'

No Observations

ce072_w2_3_8__max: Max Bracket of ce072_w2_3_8_

No Observations

ce072_w2_3_9__min: Min Bracket of ce072_w2_3_9_'

No Observations

ce072_w2_3_9__max: Max Bracket of ce072_w2_3_9_

No Observations

ce072_w2_3_10__min: Min Bracket of ce072_w2_3_10_'

No Observations

ce072_w2_3_10__max: Max Bracket of ce072_w2_3_10_

No Observations

ce072_w2_3_16__min: Min Bracket of ce072_w2_3_16_'

Mean	SD	Min	Max	Obs
300.00	238.05	100.00	800.00	7

ce072_w2_3_16__max: Max Bracket of ce072_w2_3_16_

Mean	SD	Min	Max	Obs
350.00	226.78	100.00	800.00	8

ce072_w2_3_17__min: Min Bracket of ce072_w2_3_17_'

Mean	SD	Min	Max	Obs
360.00	260.77	200.00	800.00	5

ce072_w2_3_17__max: Max Bracket of ce072_w2_3_17_

Mean	SD	Min	Max	Obs
220.00	109.54	100.00	400.00	5

ce072_w2_3_18__min: Min Bracket of ce072_w2_3_18_'

Mean	SD	Min	Max	Obs
1,000.00	848.53	400.00	1,600.00	2

ce072_w2_3_18__max: Max Bracket of ce072_w2_3_18_

Mean	SD	Min	Max	Obs
833.33	750.56	100.00	1,600.00	3

ce072_w2_4_1__min: Min Bracket of ce072_w2_4_1_'

Mean	SD	Min	Max	Obs
100.00	.	100.00	100.00	1

ce072_w2_4_1__max: Max Bracket of ce072_w2_4_1_

Mean	SD	Min	Max	Obs
100.00	0.00	100.00	100.00	4

ce072_w2_4_2__min: Min Bracket of ce072_w2_4_2_'

Mean	SD	Min	Max	Obs
100.00	.	100.00	100.00	1

ce072_w2_4_2__max: Max Bracket of ce072_w2_4_2_

Mean	SD	Min	Max	Obs
100.00	0.00	100.00	100.00	3

ce072_w2_4_3__min: Min Bracket of ce072_w2_4_3_'

Mean	SD	Min	Max	Obs
300.00	141.42	200.00	400.00	2

ce072_w2_4_3__max: Max Bracket of ce072_w2_4_3_

Mean	SD	Min	Max	Obs
250.00	212.13	100.00	400.00	2

ce072_w2_4_4__min: Min Bracket of ce072_w2_4_4_'

No Observations

ce072_w2_4_4__max: Max Bracket of ce072_w2_4_4_

No Observations

ce072_w2_4_5__min: Min Bracket of ce072_w2_4_5_'

Mean	SD	Min	Max	Obs
400.00	.	400.00	400.00	1

ce072_w2_4_5__max: Max Bracket of ce072_w2_4_5_

Mean	SD	Min	Max	Obs
400.00	.	400.00	400.00	1

ce072_w2_4_6__min: Min Bracket of ce072_w2_4_6_'

No Observations

ce072_w2_4_6__max: Max Bracket of ce072_w2_4_6_

No Observations

ce072_w2_4_7__min: Min Bracket of ce072_w2_4_7_'

No Observations

ce072_w2_4_7__max: Max Bracket of ce072_w2_4_7_

No Observations

ce072_w2_4_8__min: Min Bracket of ce072_w2_4_8_'

No Observations

ce072_w2_4_8__max: Max Bracket of ce072_w2_4_8_

No Observations

ce072_w2_4_9__min: Min Bracket of ce072_w2_4_9_'

No Observations

ce072_w2_4_9__max: Max Bracket of ce072_w2_4_9_

No Observations

ce072_w2_4_10__min: Min Bracket of ce072_w2_4_10_'

No Observations

ce072_w2_4_10__max: Max Bracket of ce072_w2_4_10_

No Observations

ce072_w2_4_16__min: Min Bracket of ce072_w2_4_16_'

No Observations

ce072_w2_4_16__max: Max Bracket of ce072_w2_4_16_

Mean	SD	Min	Max	Obs
100.00	.	100.00	100.00	1

ce072_w2_4_17__min: Min Bracket of ce072_w2_4_17_'

No Observations

ce072_w2_4_17__max: Max Bracket of ce072_w2_4_17_

Mean	SD	Min	Max	Obs
100.00	.	100.00	100.00	1

ce072_w2_4_18__min: Min Bracket of ce072_w2_4_18_'

No Observations

ce072_w2_4_18__max: Max Bracket of ce072_w2_4_18_

Mean	SD	Min	Max	Obs
100.00	.	100.00	100.00	1

ce074_w2_1_1_: Total Money Support for Siblings[1]

Mean	SD	Min	Max	Obs
554.86	3,290.94	0.00	60,000.00	680

ce074_w2_1_2_: Total Money Support for Siblings[2]

Mean	SD	Min	Max	Obs
273.02	959.63	0.00	15,000.00	713

ce074_w2_1_3_: Total Money Support for Siblings[3]

Mean	SD	Min	Max	Obs
456.75	2,363.35	0.00	30,000.00	626

ce074_w2_1_4_: Total Money Support for Siblings[4]

Mean	SD	Min	Max	Obs
307.28	1,291.77	0.00	20,000.00	469

ce074_w2_1_5_: Total Money Support for Siblings[5]

Mean	SD	Min	Max	Obs
255.82	858.64	0.00	10,000.00	291

ce074_w2_1_6_: Total Money Support for Siblings[6]

Mean	SD	Min	Max	Obs
190.54	634.02	0.00	5,000.00	139

ce074_w2_1_7_: Total Money Support for Siblings[7]

Mean	SD	Min	Max	Obs
292.79	1,046.98	0.00	8,000.00	68

ce074_w2_1_8_: Total Money Support for Siblings[8]

Mean	SD	Min	Max	Obs
136.84	457.30	0.00	2,000.00	19

ce074_w2_1_9_: Total Money Support for Siblings[9]

Mean	SD	Min	Max	Obs
160.00	167.33	0.00	400.00	5

ce074_w2_1_10_: Total Money Support for Siblings[10]

Mean	SD	Min	Max	Obs
0.00	.	0.00	0.00	1

ce074_w2_1_11_: Total Money Support for Siblings[11]

Mean	SD	Min	Max	Obs
0.00	.	0.00	0.00	1

ce074_w2_1_16_: Total Money Support for Siblings[16]

Mean	SD	Min	Max	Obs
613.33	3,528.98	0.00	60,000.00	408

ce074_w2_1_17_: Total Money Support for Siblings[17]

Mean	SD	Min	Max	Obs
283.71	752.49	0.00	8,000.00	407

ce074_w2_1_18_: Total Money Support for Siblings[18]

Mean	SD	Min	Max	Obs
456.63	2,074.02	0.00	30,000.00	377

ce074_w2_1_19_: Total Money Support for Siblings[19]

Mean	SD	Min	Max	Obs
282.01	972.30	0.00	10,000.00	274

ce074_w2_1_20_: Total Money Support for Siblings[20]

Mean	SD	Min	Max	Obs
275.70	1,148.28	0.00	10,500.00	172

ce074_w2_1_21_: Total Money Support for Siblings[21]

Mean	SD	Min	Max	Obs
132.33	375.04	0.00	3,000.00	90

ce074_w2_1_22_: Total Money Support for Siblings[22]

Mean	SD	Min	Max	Obs
428.92	1,693.34	0.00	10,000.00	37

ce074_w2_1_23_: Total Money Support for Siblings[23]

Mean	SD	Min	Max	Obs
75.00	148.32	0.00	500.00	16

ce074_w2_1_24_: Total Money Support for Siblings[24]

Mean	SD	Min	Max	Obs
50.00	100.00	0.00	200.00	4

ce074_w2_1_25_: Total Money Support for Siblings[25]

Mean	SD	Min	Max	Obs
100.00	141.42	0.00	200.00	2

ce074_w2_1_26_: Total Money Support for Siblings[26]

Mean	SD	Min	Max	Obs
600.00	565.69	200.00	1,000.00	2

ce074_w2_1_27_: Total Money Support for Siblings[27]

Mean	SD	Min	Max	Obs
200.00	.	200.00	200.00	1

ce074_w2_2_1_: Regular Money Support for Siblings[1]

Mean	SD	Min	Max	Obs
43.06	280.76	0.00	4,000.00	684

ce074_w2_2_2_: Regular Money Support for Siblings[2]

Mean	SD	Min	Max	Obs
18.64	142.87	0.00	2,400.00	716

ce074_w2_2_3_: Regular Money Support for Siblings[3]

Mean	SD	Min	Max	Obs
18.48	176.98	0.00	4,000.00	628

ce074_w2_2_4_: Regular Money Support for Siblings[4]

Mean	SD	Min	Max	Obs
35.06	469.12	0.00	10,000.00	470

ce074_w2_2_5_: Regular Money Support for Siblings[5]

Mean	SD	Min	Max	Obs
28.73	207.73	0.00	2,000.00	291

ce074_w2_2_6_: Regular Money Support for Siblings[6]

Mean	SD	Min	Max	Obs
18.63	133.88	0.00	1,500.00	140

ce074_w2_2_7_: Regular Money Support for Siblings[7]

Mean	SD	Min	Max	Obs
26.47	142.07	0.00	1,000.00	68

ce074_w2_2_8_: Regular Money Support for Siblings[8]

Mean	SD	Min	Max	Obs
52.63	229.42	0.00	1,000.00	19

ce074_w2_2_9_: Regular Money Support for Siblings[9]

Mean	SD	Min	Max	Obs
0.00	0.00	0.00	0.00	5

ce074_w2_2_10_: Regular Money Support for Siblings[10]

Mean	SD	Min	Max	Obs
0.00	.	0.00	0.00	1

ce074_w2_2_11_: Regular Money Support for Siblings[11]

Mean	SD	Min	Max	Obs
0.00	.	0.00	0.00	1

ce074_w2_2_16_: Regular Money Support for Siblings[16]

Mean	SD	Min	Max	Obs
60.52	799.49	0.00	16,000.00	412

ce074_w2_2_17_: Regular Money Support for Siblings[17]

Mean	SD	Min	Max	Obs
44.25	380.35	0.00	6,000.00	409

ce074_w2_2_18_: Regular Money Support for Siblings[18]

Mean	SD	Min	Max	Obs
50.02	376.35	0.00	5,000.00	378

ce074_w2_2_19_: Regular Money Support for Siblings[19]

Mean	SD	Min	Max	Obs
43.43	363.58	0.00	5,000.00	274

ce074_w2_2_20_: Regular Money Support for Siblings[20]

Mean	SD	Min	Max	Obs
6.65	39.97	0.00	300.00	173

ce074_w2_2_21_: Regular Money Support for Siblings[21]

Mean	SD	Min	Max	Obs
13.00	83.11	0.00	750.00	90

ce074_w2_2_22_: Regular Money Support for Siblings[22]

Mean	SD	Min	Max	Obs
4.05	24.66	0.00	150.00	37

ce074_w2_2_23_: Regular Money Support for Siblings[23]

Mean	SD	Min	Max	Obs
0.00	0.00	0.00	0.00	16

ce074_w2_2_24_: Regular Money Support for Siblings[24]

Mean	SD	Min	Max	Obs
0.00	0.00	0.00	0.00	4

ce074_w2_2_25_: Regular Money Support for Siblings[25]

Mean	SD	Min	Max	Obs
0.00	0.00	0.00	0.00	2

ce074_w2_2_26_: Regular Money Support for Siblings[26]

Mean	SD	Min	Max	Obs
0.00	0.00	0.00	0.00	2

ce074_w2_2_27_: Regular Money Support for Siblings[27]

Mean	SD	Min	Max	Obs
0.00	.	0.00	0.00	1

ce074_w2_3_1_: Total In_kind Support for Siblings[1]

Mean	SD	Min	Max	Obs
135.04	356.54	0.00	5,000.00	671

ce074_w2_3_2_: Total In_kind Support for Siblings[2]

Mean	SD	Min	Max	Obs
141.52	633.26	0.00	15,000.00	707

ce074_w2_3_3_: Total In_kind Support for Siblings[3]

Mean	SD	Min	Max	Obs
115.60	307.77	0.00	3,500.00	618

ce074_w2_3_4_: Total In_kind Support for Siblings[4]

Mean	SD	Min	Max	Obs
190.19	1,436.15	0.00	30,000.00	462

ce074_w2_3_5_: Total In_kind Support for Siblings[5]

Mean	SD	Min	Max	Obs
116.84	273.93	0.00	2,000.00	287

ce074_w2_3_6_: Total In_kind Support for Siblings[6]

Mean	SD	Min	Max	Obs
137.73	620.80	0.00	6,000.00	139

ce074_w2_3_7_: Total In_kind Support for Siblings[7]

Mean	SD	Min	Max	Obs
137.65	480.32	0.00	3,000.00	68

ce074_w2_3_8_: Total In_kind Support for Siblings[8]

Mean	SD	Min	Max	Obs
81.58	143.15	0.00	500.00	19

ce074_w2_3_9_: Total In_kind Support for Siblings[9]

Mean	SD	Min	Max	Obs
100.00	223.61	0.00	500.00	5

ce074_w2_3_10_: Total In_kind Support for Siblings[10]

Mean	SD	Min	Max	Obs
0.00	.	0.00	0.00	1

ce074_w2_3_11_: Total In_kind Support for Siblings[11]

Mean	SD	Min	Max	Obs
100.00	.	100.00	100.00	1

ce074_w2_3_16_: Total In_kind Support for Siblings[16]

Mean	SD	Min	Max	Obs
222.23	1,097.28	0.00	20,000.00	396

ce074_w2_3_17_: Total In_kind Support for Siblings[17]

Mean	SD	Min	Max	Obs
175.48	618.57	0.00	10,000.00	401

ce074_w2_3_18_: Total In_kind Support for Siblings[18]

Mean	SD	Min	Max	Obs
136.43	351.48	0.00	4,000.00	375

ce074_w2_3_19_: Total In_kind Support for Siblings[19]

Mean	SD	Min	Max	Obs
131.86	324.41	0.00	3,000.00	271

ce074_w2_3_20_: Total In_kind Support for Siblings[20]

Mean	SD	Min	Max	Obs
117.07	305.43	0.00	3,000.00	169

ce074_w2_3_21_: Total In_kind Support for Siblings[21]

Mean	SD	Min	Max	Obs
134.10	398.41	0.00	3,000.00	89

ce074_w2_3_22_: Total In_kind Support for Siblings[22]

Mean	SD	Min	Max	Obs
119.72	356.25	0.00	2,000.00	36

ce074_w2_3_23_: Total In_kind Support for Siblings[23]

Mean	SD	Min	Max	Obs
35.00	55.38	0.00	160.00	16

ce074_w2_3_24_: Total In_kind Support for Siblings[24]

Mean	SD	Min	Max	Obs
12.50	25.00	0.00	50.00	4

ce074_w2_3_25_: Total In_kind Support for Siblings[25]

Mean	SD	Min	Max	Obs
0.00	0.00	0.00	0.00	2

ce074_w2_3_26_: Total In_kind Support for Siblings[26]

Mean	SD	Min	Max	Obs
0.00	0.00	0.00	0.00	2

ce074_w2_3_27_: Total In_kind Support for Siblings[27]

Mean	SD	Min	Max	Obs
0.00	.	0.00	0.00	1

ce074_w2_4_1_: Regular In_kind Support for Siblings[1]

Mean	SD	Min	Max	Obs
24.21	225.38	0.00	5,000.00	681

ce074_w2_4_2_: Regular In_kind Support for Siblings[2]

Mean	SD	Min	Max	Obs
15.86	134.09	0.00	2,500.00	715

ce074_w2_4_3_: Regular In_kind Support for Siblings[3]

Mean	SD	Min	Max	Obs
10.53	60.25	0.00	800.00	627

ce074_w2_4_4_: Regular In_kind Support for Siblings[4]

Mean	SD	Min	Max	Obs
16.50	92.03	0.00	1,000.00	468

ce074_w2_4_5_: Regular In_kind Support for Siblings[5]

Mean	SD	Min	Max	Obs
23.99	144.09	0.00	2,000.00	291

ce074_w2_4_6_: Regular In_kind Support for Siblings[6]

Mean	SD	Min	Max	Obs
8.14	40.88	0.00	350.00	140

ce074_w2_4_7_: Regular In_kind Support for Siblings[7]

Mean	SD	Min	Max	Obs
3.09	16.32	0.00	120.00	68

ce074_w2_4_8_: Regular In_kind Support for Siblings[8]

Mean	SD	Min	Max	Obs
0.00	0.00	0.00	0.00	19

ce074_w2_4_9_: Regular In_kind Support for Siblings[9]

Mean	SD	Min	Max	Obs
0.00	0.00	0.00	0.00	5

ce074_w2_4_10_: Regular In_kind Support for Siblings[10]

Mean	SD	Min	Max	Obs
0.00	.	0.00	0.00	1

ce074_w2_4_11_: Regular In_kind Support for Siblings[11]

Mean	SD	Min	Max	Obs
0.00	.	0.00	0.00	1

ce074_w2_4_16_: Regular In_kind Support for Siblings[16]

Mean	SD	Min	Max	Obs
15.89	155.04	0.00	3,000.00	410

ce074_w2_4_17_: Regular In_kind Support for Siblings[17]

Mean	SD	Min	Max	Obs
11.27	67.70	0.00	900.00	407

ce074_w2_4_18_: Regular In_kind Support for Siblings[18]

Mean	SD	Min	Max	Obs
13.46	91.85	0.00	1,500.00	376

ce074_w2_4_19_: Regular In_kind Support for Siblings[19]

Mean	SD	Min	Max	Obs
51.01	728.36	0.00	12,000.00	272

ce074_w2_4_20_: Regular In_kind Support for Siblings[20]

Mean	SD	Min	Max	Obs
5.38	25.60	0.00	200.00	171

ce074_w2_4_21_: Regular In_kind Support for Siblings[21]

Mean	SD	Min	Max	Obs
3.93	20.26	0.00	150.00	89

ce074_w2_4_22_: Regular In_kind Support for Siblings[22]

Mean	SD	Min	Max	Obs
5.83	27.71	0.00	160.00	36

ce074_w2_4_23_: Regular In_kind Support for Siblings[23]

Mean	SD	Min	Max	Obs
0.00	0.00	0.00	0.00	16

ce074_w2_4_24_: Regular In_kind Support for Siblings[24]

Mean	SD	Min	Max	Obs
0.00	0.00	0.00	0.00	4

ce074_w2_4_25_: Regular In_kind Support for Siblings[25]

Mean	SD	Min	Max	Obs
0.00	0.00	0.00	0.00	2

ce074_w2_4_26_: Regular In_kind Support for Siblings[26]

Mean	SD	Min	Max	Obs
0.00	0.00	0.00	0.00	2

ce074_w2_4_27_: Regular In_kind Support for Siblings[27]

Mean	SD	Min	Max	Obs
0.00	.	0.00	0.00	1

ce074_w2_1_1__min: Min Bracket of ce074_w2_1_1_

Mean	SD	Min	Max	Obs
200.00	.	200.00	200.00	1

ce074_w2_1_1__max: Max Bracket of ce074_w2_1_1_

Mean	SD	Min	Max	Obs
400.00	.	400.00	400.00	1

ce074_w2_1_2__min: Min Bracket of ce074_w2_1_2_

Mean	SD	Min	Max	Obs
400.00	.	400.00	400.00	1

ce074_w2_1_2__max: Max Bracket of ce074_w2_1_2_

Mean	SD	Min	Max	Obs
800.00	.	800.00	800.00	1

ce074_w2_1_3__min: Min Bracket of ce074_w2_1_3_

No Observations

ce074_w2_1_3__max: Max Bracket of ce074_w2_1_3_

No Observations

ce074_w2_1_4__min: Min Bracket of ce074_w2_1_4_

No Observations

ce074_w2_1_4__max: Max Bracket of ce074_w2_1_4_

No Observations

ce074_w2_1_5__min: Min Bracket of ce074_w2_1_5_

No Observations

ce074_w2_1_5__max: Max Bracket of ce074_w2_1_5_

No Observations

ce074_w2_1_6__min: Min Bracket of ce074_w2_1_6_

No Observations

ce074_w2_1_6__max: Max Bracket of ce074_w2_1_6_

No Observations

ce074_w2_1_7__min: Min Bracket of ce074_w2_1_7_

No Observations

ce074_w2_1_7__max: Max Bracket of ce074_w2_1_7_

No Observations

ce074_w2_1_16__min: Min Bracket of ce074_w2_1_16_

Mean	SD	Min	Max	Obs
466.67	305.51	200.00	800.00	3

ce074_w2_1_16__max: Max Bracket of ce074_w2_1_16_

Mean	SD	Min	Max	Obs
533.33	230.94	400.00	800.00	3

ce074_w2_1_17__min: Min Bracket of ce074_w2_1_17_

Mean	SD	Min	Max	Obs
400.00	.	400.00	400.00	1

ce074_w2_1_17__max: Max Bracket of ce074_w2_1_17_

Mean	SD	Min	Max	Obs
400.00	.	400.00	400.00	1

ce074_w2_1_18__min: Min Bracket of ce074_w2_1_18_

Mean	SD	Min	Max	Obs

400.00	.	400.00	400.00	1

ce074_w2_1_18__max: Max Bracket of ce074_w2_1_18_

Mean	SD	Min	Max	Obs
400.00	.	400.00	400.00	1

ce074_w2_1_20__min: Min Bracket of ce074_w2_1_20_

Mean	SD	Min	Max	Obs
400.00	.	400.00	400.00	1

ce074_w2_1_20__max: Max Bracket of ce074_w2_1_20_

Mean	SD	Min	Max	Obs
400.00	.	400.00	400.00	1

ce074_w2_2_1__min: Min Bracket of ce074_w2_2_1_

No Observations

ce074_w2_2_1__max: Max Bracket of ce074_w2_2_1_

No Observations

ce074_w2_2_2__min: Min Bracket of ce074_w2_2_2_

No Observations

ce074_w2_2_2__max: Max Bracket of ce074_w2_2_2_

No Observations

ce074_w2_2_3__min: Min Bracket of ce074_w2_2_3_

No Observations

ce074_w2_2_3__max: Max Bracket of ce074_w2_2_3_

No Observations

ce074_w2_2_4__min: Min Bracket of ce074_w2_2_4_

No Observations

ce074_w2_2_4__max: Max Bracket of ce074_w2_2_4_

No Observations

ce074_w2_2_5__min: Min Bracket of ce074_w2_2_5_

No Observations

ce074_w2_2_5__max: Max Bracket of ce074_w2_2_5_

No Observations

ce074_w2_2_6__min: Min Bracket of ce074_w2_2_6_

No Observations

ce074_w2_2_6__max: Max Bracket of ce074_w2_2_6_

No Observations

ce074_w2_2_7__min: Min Bracket of ce074_w2_2_7_

No Observations

ce074_w2_2_7__max: Max Bracket of ce074_w2_2_7_

No Observations

ce074_w2_2_16__min: Min Bracket of ce074_w2_2_16_

No Observations

ce074_w2_2_16__max: Max Bracket of ce074_w2_2_16_

No Observations

ce074_w2_2_17__min: Min Bracket of ce074_w2_2_17_

No Observations

ce074_w2_2_17__max: Max Bracket of ce074_w2_2_17_

No Observations

ce074_w2_2_18__min: Min Bracket of ce074_w2_2_18_

Mean	SD	Min	Max	Obs
200.00	.	200.00	200.00	1

ce074_w2_2_18__max: Max Bracket of ce074_w2_2_18_

Mean	SD	Min	Max	Obs
200.00	.	200.00	200.00	1

ce074_w2_2_20__min: Min Bracket of ce074_w2_2_20_

No Observations

ce074_w2_2_20__max: Max Bracket of ce074_w2_2_20_

No Observations

ce074_w2_3_1__min: Min Bracket of ce074_w2_3_1_

Mean	SD	Min	Max	Obs
233.33	136.63	100.00	400.00	6

ce074_w2_3_1__max: Max Bracket of ce074_w2_3_1_

Mean	SD	Min	Max	Obs
228.57	125.36	100.00	400.00	7

ce074_w2_3_2__min: Min Bracket of ce074_w2_3_2_

Mean	SD	Min	Max	Obs
200.00	109.54	100.00	400.00	6

ce074_w2_3_2__max: Max Bracket of ce074_w2_3_2_

Mean	SD	Min	Max	Obs
250.00	122.47	100.00	400.00	6

ce074_w2_3_3__min: Min Bracket of ce074_w2_3_3_

Mean	SD	Min	Max	Obs
180.00	44.72	100.00	200.00	5

ce074_w2_3_3__max: Max Bracket of ce074_w2_3_3_

Mean	SD	Min	Max	Obs
200.00	0.00	200.00	200.00	5

ce074_w2_3_4__min: Min Bracket of ce074_w2_3_4_

Mean	SD	Min	Max	Obs

260.00	134.16	100.00	400.00	5

ce074_w2_3_4__max: Max Bracket of ce074_w2_3_4_

Mean	SD	Min	Max	Obs
400.00	244.95	200.00	800.00	5

ce074_w2_3_5__min: Min Bracket of ce074_w2_3_5_

Mean	SD	Min	Max	Obs
250.00	212.13	100.00	400.00	2

ce074_w2_3_5__max: Max Bracket of ce074_w2_3_5_

Mean	SD	Min	Max	Obs
300.00	141.42	200.00	400.00	2

ce074_w2_3_6__min: Min Bracket of ce074_w2_3_6_

No Observations

ce074_w2_3_6__max: Max Bracket of ce074_w2_3_6_

No Observations

ce074_w2_3_7__min: Min Bracket of ce074_w2_3_7_

No Observations

ce074_w2_3_7__max: Max Bracket of ce074_w2_3_7_

No Observations

ce074_w2_3_16__min: Min Bracket of ce074_w2_3_16_

Mean	SD	Min	Max	Obs
240.00	84.33	200.00	400.00	10

ce074_w2_3_16__max: Max Bracket of ce074_w2_3_16_

Mean	SD	Min	Max	Obs
276.92	187.77	100.00	800.00	13

ce074_w2_3_17__min: Min Bracket of ce074_w2_3_17_

Mean	SD	Min	Max	Obs

340.00	279.28	100.00	800.00	5

ce074_w2_3_17__max: Max Bracket of ce074_w2_3_17_

Mean	SD	Min	Max	Obs
300.00	268.33	100.00	800.00	6

ce074_w2_3_18__min: Min Bracket of ce074_w2_3_18_

Mean	SD	Min	Max	Obs
200.00	.	200.00	200.00	1

ce074_w2_3_18__max: Max Bracket of ce074_w2_3_18_

Mean	SD	Min	Max	Obs
150.00	70.71	100.00	200.00	2

ce074_w2_3_20__min: Min Bracket of ce074_w2_3_20_

Mean	SD	Min	Max	Obs
233.33	152.75	100.00	400.00	3

ce074_w2_3_20__max: Max Bracket of ce074_w2_3_20_

Mean	SD	Min	Max	Obs
250.00	173.21	100.00	400.00	4

ce074_w2_4_1__min: Min Bracket of ce074_w2_4_1_

No Observations

ce074_w2_4_1__max: Max Bracket of ce074_w2_4_1_

Mean	SD	Min	Max	Obs
1,600.00	.	1,600.00	1,600.00	1

ce074_w2_4_2__min: Min Bracket of ce074_w2_4_2_

No Observations

ce074_w2_4_2__max: Max Bracket of ce074_w2_4_2_

No Observations

ce074_w2_4_3__min: Min Bracket of ce074_w2_4_3_

No Observations

ce074_w2_4_3__max: Max Bracket of ce074_w2_4_3_

No Observations

ce074_w2_4_4__min: Min Bracket of ce074_w2_4_4_

No Observations

ce074_w2_4_4__max: Max Bracket of ce074_w2_4_4_

Mean	SD	Min	Max	Obs
100.00	.	100.00	100.00	1

ce074_w2_4_5__min: Min Bracket of ce074_w2_4_5_

No Observations

ce074_w2_4_5__max: Max Bracket of ce074_w2_4_5_

No Observations

ce074_w2_4_6__min: Min Bracket of ce074_w2_4_6_

No Observations

ce074_w2_4_6__max: Max Bracket of ce074_w2_4_6_

No Observations

ce074_w2_4_7__min: Min Bracket of ce074_w2_4_7_

No Observations

ce074_w2_4_7__max: Max Bracket of ce074_w2_4_7_

No Observations

ce074_w2_4_16__min: Min Bracket of ce074_w2_4_16_

No Observations

ce074_w2_4_16__max: Max Bracket of ce074_w2_4_16_

Mean	SD	Min	Max	Obs

100.00	.	100.00	100.00	1

ce074_w2_4_17__min: Min Bracket of ce074_w2_4_17_

No Observations

ce074_w2_4_17__max: Max Bracket of ce074_w2_4_17_

Mean	SD	Min	Max	Obs
100.00	.	100.00	100.00	1

ce074_w2_4_18__min: Min Bracket of ce074_w2_4_18_

Mean	SD	Min	Max	Obs
100.00	.	100.00	100.00	1

ce074_w2_4_18__max: Max Bracket of ce074_w2_4_18_

Mean	SD	Min	Max	Obs
100.00	0.00	100.00	100.00	2

ce074_w2_4_20__min: Min Bracket of ce074_w2_4_20_

No Observations

ce074_w2_4_20__max: Max Bracket of ce074_w2_4_20_

Mean	SD	Min	Max	Obs
100.00	.	100.00	100.00	1

cf001: Take Care of GrandChildren

	Freq.	%
1 Yes	4,466	38.71
2 No	5,161	44.74
3 There is No GrandChildren	1,909	16.55
Total	11,536	100.00

cf002_s1: Care XChildName[1]'s Children

	Freq.	%
0 No	1,585	35.49
1 Yes	2,881	64.51
Total	4,466	100.00

cf002_s2: Care XChildName[2]'s Children

	Freq.	%
0 No	2,818	63.10
2 Yes	1,648	36.90
Total	4,466	100.00

cf002_s3: Care XChildName[3]'s Children

	Freq.	%
0 No	3,879	86.86
3 Yes	587	13.14
Total	4,466	100.00

cf002_s4: Care XChildName[4]'s Children

	Freq.	%
0 No	4,250	95.16
4 Yes	216	4.84
Total	4,466	100.00

cf002_s5: Care XChildName[5]'s Children

	Freq.	%
0 No	4,393	98.37
5 Yes	73	1.63
Total	4,466	100.00

cf002_s6: Care XChildName[6]'s Children

	Freq.	%
0 No	4,439	99.40
6 Yes	27	0.60
Total	4,466	100.00

cf002_s7: Care XChildName[7]'s Children

	Freq.	%
0 No	4,454	99.73
7 Yes	12	0.27
Total	4,466	100.00

cf002_s8: Care XChildName[8]'s Children

	Freq.	%
0 No	4,457	99.80
8 Yes	9	0.20
Total	4,466	100.00

cf002_s9: Care XChildName[9]'s Children

	Freq.	%
0 No	4,463	99.93
9 Yes	3	0.07
Total	4,466	100.00

cf002_s10: Care XChildName[10]'s Children

	Freq.	%
0 No	4,465	99.98
10 Yes	1	0.02
Total	4,466	100.00

cf002_s11: Care XChildName[11]'s Children

	Freq.	%
0 No	4,465	99.98
11 Yes	1	0.02
Total	4,466	100.00

cf003_1_1_: Weeks for XChildName[1]

Mean	SD	Min	Max	Obs
36.64	19.80	0.00	52.00	2,881

cf003_1_2_: Weeks for XChildName[2]

Mean	SD	Min	Max	Obs
33.15	20.92	0.00	52.00	1,648

cf003_1_3_: Weeks for XChildName[3]

Mean	SD	Min	Max	Obs
30.90	21.53	0.00	52.00	587

cf003_1_4_: Weeks for XChildName[4]

Mean	SD	Min	Max	Obs
30.91	22.01	0.00	52.00	216

cf003_1_5_: Weeks for XChildName[5]

Mean	SD	Min	Max	Obs
33.52	21.16	0.00	52.00	73

cf003_1_6_: Weeks for XChildName[6]

Mean	SD	Min	Max	Obs
28.81	20.36	0.00	52.00	27

cf003_1_7_: Weeks for XChildName[7]

Mean	SD	Min	Max	Obs
32.50	19.74	4.00	52.00	12

cf003_1_8_: Weeks for XChildName[8]

Mean	SD	Min	Max	Obs
39.67	19.07	3.00	52.00	9

cf003_1_9_: Weeks for XChildName[9]

Mean	SD	Min	Max	Obs
35.67	28.29	3.00	52.00	3

cf003_1_10_: Weeks for XChildName[10]

Mean	SD	Min	Max	Obs
52.00	.	52.00	52.00	1

cf003_1_11_: Weeks for XChildName[11]

Mean	SD	Min	Max	Obs
12.00	.	12.00	12.00	1

cf003_2_1_: Hours for XChildName[1]

Mean	SD	Min	Max	Obs
48.23	37.29	0.00	140.00	2,881

cf003_2_2_: Hours for XChildName[2]

Mean	SD	Min	Max	Obs
46.29	37.57	0.00	140.00	1,648

cf003_2_3_: Hours for XChildName[3]

Mean	SD	Min	Max	Obs
47.72	39.11	0.00	140.00	587

cf003_2_4_: Hours for XChildName[4]

Mean	SD	Min	Max	Obs
39.95	33.28	0.00	126.00	216

cf003_2_5_: Hours for XChildName[5]

Mean	SD	Min	Max	Obs
46.72	38.50	0.00	140.00	73

cf003_2_6_: Hours for XChildName[6]

Mean	SD	Min	Max	Obs
45.89	36.19	0.00	100.00	27

cf003_2_7_: Hours for XChildName[7]

Mean	SD	Min	Max	Obs
65.25	32.64	1.00	112.00	12

cf003_2_8_: Hours for XChildName[8]

Mean	SD	Min	Max	Obs
40.78	30.93	7.00	84.00	9

cf003_2_9_: Hours for XChildName[9]

Mean	SD	Min	Max	Obs
45.83	21.05	31.50	70.00	3

cf003_2_10_: Hours for XChildName[10]

Mean	SD	Min	Max	Obs
70.00	.	70.00	70.00	1

cf003_2_11_: Hours for XChildName[11]

Mean	SD	Min	Max	Obs
8.00	.	8.00	8.00	1

cf003_3_1_: Weeks for XChildName[1] of Spouse

Mean	SD	Min	Max	Obs
27.05	23.18	0.00	52.00	2,881

cf003_3_2_: Weeks for XChildName[2] of Spouse

Mean	SD	Min	Max	Obs
24.26	22.88	0.00	52.00	1,648

cf003_3_3_: Weeks for XChildName[3] of Spouse

Mean	SD	Min	Max	Obs
21.64	23.00	0.00	52.00	587

cf003_3_4_: Weeks for XChildName[4] of Spouse

Mean	SD	Min	Max	Obs
21.64	23.44	0.00	52.00	216

cf003_3_5_: Weeks for XChildName[5] of Spouse

Mean	SD	Min	Max	Obs
19.16	23.26	0.00	52.00	73

cf003_3_6_: Weeks for XChildName[6] of Spouse

Mean	SD	Min	Max	Obs
15.89	21.90	0.00	52.00	27

cf003_3_7_: Weeks for XChildName[7] of Spouse

Mean	SD	Min	Max	Obs
16.42	21.91	0.00	52.00	12

cf003_3_8_: Weeks for XChildName[8] of Spouse

Mean	SD	Min	Max	Obs
18.56	24.41	0.00	52.00	9

cf003_3_9_: Weeks for XChildName[9] of Spouse

Mean	SD	Min	Max	Obs
18.33	29.19	0.00	52.00	3

cf003_3_10_: Weeks for XChildName[10] of Spouse

Mean	SD	Min	Max	Obs
52.00	.	52.00	52.00	1

cf003_3_11_: Weeks for XChildName[11] of Spouse

Mean	SD	Min	Max	Obs
0.00	.	0.00	0.00	1

cf003_4_1_: Hours for XChildName[1] of Spouse

Mean	SD	Min	Max	Obs
36.10	38.47	0.00	140.00	2,881

cf003_4_2_: Hours for XChildName[2] of Spouse

Mean	SD	Min	Max	Obs
33.71	37.69	0.00	140.00	1,648

cf003_4_3_: Hours for XChildName[3] of Spouse

Mean	SD	Min	Max	Obs
35.10	41.23	0.00	140.00	587

cf003_4_4_: Hours for XChildName[4] of Spouse

Mean	SD	Min	Max	Obs
29.70	35.10	0.00	126.00	216

cf003_4_5_: Hours for XChildName[5] of Spouse

Mean	SD	Min	Max	Obs
23.73	35.85	0.00	140.00	73

cf003_4_6_: Hours for XChildName[6] of Spouse

Mean	SD	Min	Max	Obs
29.41	39.34	0.00	100.00	27

cf003_4_7_: Hours for XChildName[7] of Spouse

Mean	SD	Min	Max	Obs
28.25	39.81	0.00	100.00	12

cf003_4_8_: Hours for XChildName[8] of Spouse

Mean	SD	Min	Max	Obs
17.44	28.18	0.00	84.00	9

cf003_4_9_: Hours for XChildName[9] of Spouse

Mean	SD	Min	Max	Obs
35.33	35.00	0.00	70.00	3

cf003_4_10_: Hours for XChildName[10] of Spouse

Mean	SD	Min	Max	Obs
70.00	.	70.00	70.00	1

cf003_4_11_: Hours for XChildName[11] of Spouse

Mean	SD	Min	Max	Obs
0.00	.	0.00	0.00	1

cf004_w4_1_: Take care of XConParName[1]

	Freq.	%
1 Yes	685	47.84
2 No	747	52.16
Total	1,432	100.00

cf004_w4_2_: Take care of XConParName[2]

	Freq.	%
1 Yes	814	47.08
2 No	915	52.92
Total	1,729	100.00

cf004_w4_3_: Take care of XConParName[3]

	Freq.	%
1 Yes	7	17.95
2 No	32	82.05
Total	39	100.00

cf004_w4_4_: Take care of XConParName[4]

	Freq.	%
1 Yes	10	20.83
2 No	38	79.17
Total	48	100.00

cf004_w4_5_: Take care of XConParName[5]

	Freq.	%
1 Yes	601	43.02
2 No	796	56.98
Total	1,397	100.00

cf004_w4_6_: Take care of XConParName[6]

	Freq.	%
1 Yes	606	42.68
2 No	814	57.32
Total	1,420	100.00

cf004_w4_7_: Take care of XConParName[7]

	Freq.	%
1 Yes	7	25.93
2 No	20	74.07
Total	27	100.00

cf004_w4_8_: Take care of XConParName[8]

	Freq.	%
1 Yes	8	28.57
2 No	20	71.43
Total	28	100.00

cf005_w4_1_1_: Weeks for XConParName[1]

Mean	SD	Min	Max	Obs
27.79	21.77	0.00	52.00	91

cf005_w4_1_2_: Weeks for XConParName[2]

Mean	SD	Min	Max	Obs
28.45	22.18	0.00	52.00	130

cf005_w4_1_3_: Weeks for XConParName[3]

Mean	SD	Min	Max	Obs
20.00	27.87	1.00	52.00	3

cf005_w4_1_4_: Weeks for XConParName[4]

Mean	SD	Min	Max	Obs
17.67	29.74	0.00	52.00	3

cf005_w4_1_5_: Weeks for XConParName[5]

Mean	SD	Min	Max	Obs
26.89	22.20	0.00	52.00	35

cf005_w4_1_6_: Weeks for XConParName[6]

Mean	SD	Min	Max	Obs
25.49	21.99	0.00	52.00	44

cf005_w4_1_7_: Weeks for XConParName[7]

Mean	SD	Min	Max	Obs
2.00	.	2.00	2.00	1

cf005_w4_2_1_: Hours for XConParName[1]

Mean	SD	Min	Max	Obs
24.75	30.35	0.00	128.00	91

cf005_w4_2_2_: Hours for XConParName[2]

Mean	SD	Min	Max	Obs
27.99	34.49	0.00	140.00	130

cf005_w4_2_3_: Hours for XConParName[3]

Mean	SD	Min	Max	Obs
47.00	42.23	1.00	84.00	3

cf005_w4_2_4_: Hours for XConParName[4]

Mean	SD	Min	Max	Obs
28.33	48.21	0.00	84.00	3

cf005_w4_2_5_: Hours for XConParName[5]

Mean	SD	Min	Max	Obs
18.56	32.97	0.00	126.00	35

cf005_w4_2_6_: Hours for XConParName[6]

Mean	SD	Min	Max	Obs
23.16	33.38	0.00	140.00	44

cf005_w4_2_7_: Hours for XConParName[7]

Mean	SD	Min	Max	Obs
84.00	.	84.00	84.00	1

cf006_w4_1_1_: Weeks for XConParName[1] of Spouse

Mean	SD	Min	Max	Obs
16.64	20.91	0.00	52.00	633

cf006_w4_1_2_: Weeks for XConParName[2] of Spouse

Mean	SD	Min	Max	Obs
18.71	21.86	0.00	52.00	714

cf006_w4_1_3_: Weeks for XConParName[3] of Spouse

Mean	SD	Min	Max	Obs
13.00	26.00	0.00	52.00	4

cf006_w4_1_4_: Weeks for XConParName[4] of Spouse

Mean	SD	Min	Max	Obs
22.00	25.66	0.00	52.00	7

cf006_w4_1_5_: Weeks for XConParName[5] of Spouse

Mean	SD	Min	Max	Obs
19.86	21.20	0.00	52.00	601

cf006_w4_1_6_: Weeks for XConParName[6] of Spouse

Mean	SD	Min	Max	Obs
21.58	21.49	0.00	52.00	606

cf006_w4_1_7_: Weeks for XConParName[7] of Spouse

Mean	SD	Min	Max	Obs
23.00	27.14	0.00	52.00	7

cf006_w4_1_8_: Weeks for XConParName[8] of Spouse

Mean	SD	Min	Max	Obs
18.88	24.71	0.00	50.00	8

cf006_w4_2_1_: Hours for XConParName[1] of Spouse

Mean	SD	Min	Max	Obs
16.52	26.59	0.00	140.00	633

cf006_w4_2_2_: Hours for XConParName[2] of Spouse

Mean	SD	Min	Max	Obs
18.49	30.54	0.00	140.00	714

cf006_w4_2_3_: Hours for XConParName[3] of Spouse

Mean	SD	Min	Max	Obs
5.00	10.00	0.00	20.00	4

cf006_w4_2_4_: Hours for XConParName[4] of Spouse

Mean	SD	Min	Max	Obs
38.80	42.47	0.00	84.00	7

cf006_w4_2_5_: Hours for XConParName[5] of Spouse

Mean	SD	Min	Max	Obs
18.31	26.39	0.00	140.00	601

cf006_w4_2_6_: Hours for XConParName[6] of Spouse

Mean	SD	Min	Max	Obs
27.05	35.19	0.00	140.00	606

cf006_w4_2_7_: Hours for XConParName[7] of Spouse

Mean	SD	Min	Max	Obs
19.43	29.32	0.00	84.00	7

cf006_w4_2_8_: Hours for XConParName[8] of Spouse

Mean	SD	Min	Max	Obs
11.50	12.98	0.00	32.00	8

versionID: Version ID

A String Variable	
Obs:	11,568

MODULE D

Health Status and Functioning

ID: Individual ID

A String Variable	
Obs:	19,752

householdID: Household ID

A String Variable	
Obs:	19,752

communityID: Community ID

A String Variable	
Obs:	19,752

da002: Self-Reported Health Status

	Freq.	%
1 Very Good	2,229	12.19
2 Good	2,348	12.85
3 Fair	8,938	48.90
4 Poor	3,687	20.17
5 Very Poor	1,076	5.89
Total	18,278	100.00

da002_w2_1: Health Status Compared with ZIWTime

	Freq.	%
1 Better	1,536	7.91
2 About The Same	8,195	42.21
3 Worse	9,683	49.88
Total	19,414	100.00

da005_1_: Physical Disabilities

	Freq.	%
1 Yes	574	3.14
2 No	17,723	96.86
Total	18,297	100.00

da005_2_: Brain Damage/Mental Retardation

	Freq.	%
1 Yes	630	3.39
2 No	17,934	96.61
Total	18,564	100.00

da005_3_: Vision Problem

	Freq.	%
1 Yes	734	4.15
2 No	16,947	95.85
Total	17,681	100.00

da005_4_: Hearing Problem

	Freq.	%
1 Yes	1,001	5.96
2 No	15,791	94.04
Total	16,792	100.00

da005_5_: Speech Impediment

	Freq.	%
1 Yes	131	0.67
2 No	19,424	99.33
Total	19,555	100.00

da006_1_: Year Had Physical Disabilities

Mean	SD	Min	Max	Obs
2,003.82	16.95	1,940.00	2,018.00	570

da006_2_: Year Had Brain Damage/Mental Retardation

Mean	SD	Min	Max	Obs
2,007.17	15.70	1,900.00	2,018.00	625

da006_3_: Year Had Vision Problem

Mean	SD	Min	Max	Obs

2,010.71	13.06	1,900.00	2,018.00	731

da006_4_: Year Had Hearing Problem

Mean	SD	Min	Max	Obs
2,011.73	10.84	1,946.00	2,018.00	992

da006_5_: Year Had Speech Impediment

Mean	SD	Min	Max	Obs
2,005.51	20.05	1,943.00	2,018.00	131

da006_w4_1_: Had Physical Disabilities

	Freq.	%
1 Yes	28	59.57
99 Never had it	19	40.43
Total	47	100.00

da006_w4_2_: Had Brain Damage/Mental Retardation

	Freq.	%
1 Yes	46	65.71
99 Never Had It	24	34.29
Total	70	100.00

da006_w4_3_: Had Vision Problem

	Freq.	%
1 Yes	40	55.56
99 Never Had It	32	44.44
Total	72	100.00

da006_w4_4_: Had Hearing Problem

	Freq.	%
1 Yes	83	73.45
99 Never Had It	30	26.55
Total	113	100.00

da006_w4_5_: Had Speech Impediment

	Freq.	%
1 Yes	10	52.63
99 Never Had It	9	47.37
Total	19	100.00

da006_w4_1_1_: Year Had Physical Disabilities

Mean	SD	Min	Max	Obs
1,848.04	522.63	-1.00	2,016.00	28

da006_w4_1_2_: Year Had Brain Damage/Mental Retardation

Mean	SD	Min	Max	Obs
1,905.77	406.94	-1.00	2,017.00	47

da006_w4_1_3_: Year Had Vision Problem

Mean	SD	Min	Max	Obs
1,864.12	505.56	-1.00	2,018.00	42

da006_w4_1_4_: Year Had Hearing Problem

Mean	SD	Min	Max	Obs
1,948.27	309.11	-1.00	2,018.00	83

da006_w4_1_5_: Year Had Speech Impediment

Mean	SD	Min	Max	Obs
1,776.00	624.74	-1.00	2,018.00	10

da007_1_: Diagnosed with Hypertension by a Doctor

	Freq.	%
1 Yes	2,187	15.07
2 No	12,322	84.93
Total	14,509	100.00

da007_2_: Diagnosed with Dyslipidemia by a Doctor

	Freq.	%
1 Yes	1,907	11.01
2 No	15,408	88.99
Total	17,315	100.00

da007_3_: Diagnosed with Diabetes by a Doctor

	Freq.	%
1 Yes	1,053	5.77
2 No	17,210	94.23
Total	18,263	100.00

da007_4_: Diagnosed with Cancer by a Doctor

	Freq.	%
1 Yes	259	1.32
2 No	19,298	98.68
Total	19,557	100.00

da007_5_: Diagnosed with Chronic Lung Diseases by a Doctor

	Freq.	%
1 Yes	994	5.54
2 No	16,947	94.46
Total	17,941	100.00

da007_6_: Diagnosed with Liver Disease by a Doctor

	Freq.	%
1 Yes	635	3.34
2 No	18,373	96.66
Total	19,008	100.00

da007_7_: Diagnosed with Heart Attack by a Doctor

	Freq.	%
1 Yes	1,371	7.96
2 No	15,848	92.04
Total	17,219	100.00

da007_8_: Diagnosed with Stroke by a Doctor

	Freq.	%
1 Yes	974	5.05
2 No	18,322	94.95
Total	19,296	100.00

da007_9_: Diagnosed with Kidney disease by a Doctor

	Freq.	%
1 Yes	762	4.11
2 No	17,781	95.89
Total	18,543	100.00

da007_10_: Diagnosed with Stomach Disease by a Doctor

	Freq.	%
1 Yes	1,551	10.11
2 No	13,795	89.89
Total	15,346	100.00

da007_11_: Diagnosed with Emotional Problems by a Doctor

	Freq.	%
1 Yes	229	1.18
2 No	19,248	98.82
Total	19,477	100.00

da007_12_: Diagnosed with Memory-Related Disease by a Doctor

	Freq.	%
1 Yes	445	2.29
2 No	18,951	97.71
Total	19,396	100.00

da007_13_: Diagnosed with Arthritis by a Doctor

	Freq.	%
1 Yes	1,517	11.17
2 No	12,059	88.83
Total	13,576	100.00

da007_14_: Diagnosed with Asthma by a Doctor

	Freq.	%
1 Yes	409	2.15
2 No	18,577	97.85
Total	18,986	100.00

da008_1_: Know Having Hypertension

	Freq.	%
1 Yes	129	1.06
2 No	10,813	88.73
3 Don't Know	1,244	10.21
Total	12,186	100.00

da008_5_: Know Having Chronic Lung Disease

	Freq.	%
1 Yes	160	0.96
2 No	13,891	83.22
3 Don't Know	2,641	15.82
Total	16,692	100.00

da008_11_: Know Having Emotional Problems

	Freq.	%
1 Yes	143	0.75
2 No	16,206	84.86
3 Don't Know	2,749	14.39
Total	19,098	100.00

da008_w2_1_1_: How Did You Know You Had Hypertension

	Freq.	%
1 Physical Examination after Had It	771	33.29
2 Physical Examination after Had Ill	571	24.65
3 Physical Examination Organized by Work Unit	136	5.87
4 Physical Examination Organized by Community	422	18.22
5 Charls Physical Examination	66	2.85
6 Other	350	15.11
Total	2,316	100.00

da008_w2_1_2_: How Did You Know You Had Dyslipidemia

	Freq.	%
1 Physical Examination after Had It	338	17.72
2 Physical Examination after Had Ill	601	31.52
3 Physical Examination Organized by Work Unit	170	8.91
4 Physical Examination Organized by Community	423	22.18
5 Charls Physical Examination	81	4.25
6 Other	294	15.42
Total	1,907	100.00

da008_w2_1_3_: How Did You Know You Had Diabetes

	Freq.	%
1 Physical Examination after Had It	186	17.66
2 Physical Examination after Had Ill	342	32.48
3 Physical Examination Organized by Work Unit	72	6.84
4 Physical Examination Organized by Community	233	22.13
5 Charls Physical Examination	55	5.22
6 Other	165	15.67
Total	1,053	100.00

da008_w2_1_4_: How Did You Know You Had Cancer

	Freq.	%
1 Physical Examination after Had It	158	61.00
2 Physical Examination after Had Ill	69	26.64
3 Physical Examination Organized by Work Unit	6	2.32
4 Physical Examination Organized by Community	7	2.70
6 Other	19	7.34
Total	259	100.00

da008_w2_1_5_: How Did You Know You Had Chronic Lung Diseases

	Freq.	%
1 Physical Examination after Had It	597	51.73
2 Physical Examination after Had Ill	317	27.47
3 Physical Examination Organized by Work Unit	23	1.99
4 Physical Examination Organized by Community	45	3.90
5 Charls Physical Examination	4	0.35
6 Other	168	14.56

Total	1,154	100.00

da008_w2_1_6_: How Did You Know You Had Liver disease

	Freq.	%
1 Physical Examination after Had It	202	31.81
2 Physical Examination after Had Ill	195	30.71
3 Physical Examination Organized by Work Unit	61	9.61
4 Physical Examination Organized by Community	94	14.80
5 Charls Physical Examination	2	0.31
6 Other	81	12.76
Total	635	100.00

da008_w2_1_7_: How Did You Know You Had Heart Attack

	Freq.	%
1 Physical Examination after Had It	634	46.24
2 Physical Examination after Had Ill	420	30.63
3 Physical Examination Organized by Work Unit	51	3.72
4 Physical Examination Organized by Community	149	10.87
5 Charls Physical Examination	3	0.22
6 Other	114	8.32
Total	1,371	100.00

da008_w2_1_8_: How Did You Know You Had Stroke

	Freq.	%
1 Physical Examination after Had It	740	75.98
2 Physical Examination after Had Ill	181	18.58
3 Physical Examination Organized by Work Unit	6	0.62
4 Physical Examination Organized by Community	15	1.54
5 Charls Physical Examination	2	0.21
6 Other	30	3.08
Total	974	100.00

da008_w2_1_9_: How Did You Know You Had Kidney Disease

	Freq.	%
1 Physical Examination after Had It	338	44.36
2 Physical Examination after Had Ill	225	29.53
3 Physical Examination Organized by Work Unit	47	6.17
4 Physical Examination Organized by Community	80	10.50
5 Charls Physical Examination	3	0.39
6 Other	69	9.06
Total	762	100.00

da008_w2_1_10_: How Did You Know You Had Stomach Disease

	Freq.	%
1 Physical Examination after Had It	1,111	71.63
2 Physical Examination after Had Ill	291	18.76
3 Physical Examination Organized by Work Unit	17	1.10

	Freq.	%
4 Physical Examination Organized by Community	40	2.58
5 Charls Physical Examination	4	0.26
6 Other	88	5.67
Total	1,551	100.00

da008_w2_1_11_: How Did You Know You Had Emotional Problems

	Freq.	%
1 Physical Examination after Had It	157	42.20
2 Physical Examination after Had Ill	77	20.70
3 Physical Examination Organized by Work Unit	3	0.81
4 Physical Examination Organized by Community	9	2.42
5 Charls Physical Examination	1	0.27
6 Other	125	33.60
Total	372	100.00

da008_w2_1_12_: How Did You Know You Had Memory-Related Disease

	Freq.	%
1 Physical Examination after Had It	172	38.65
2 Physical Examination after Had Ill	206	46.29
3 Physical Examination Organized by Work Unit	5	1.12
4 Physical Examination Organized by Community	18	4.04
5 Charls Physical Examination	3	0.67
6 Other	41	9.21
Total	445	100.00

da008_w2_1_13_: How Did You Know You Had Arthritis

	Freq.	%
1 Physical Examination after Had It	1,214	80.03
2 Physical Examination after Had Ill	171	11.27
3 Physical Examination Organized by Work Unit	18	1.19
4 Physical Examination Organized by Community	27	1.78
5 Charls Physical Examination	1	0.07
6 Other	86	5.67
Total	1,517	100.00

da008_w2_1_14_: How Did You Know You Had Asthma

	Freq.	%
1 Physical Examination after Had It	247	60.39
2 Physical Examination after Had Ill	135	33.01
3 Physical Examination Organized by Work Unit	2	0.49
4 Physical Examination Organized by Community	9	2.20
5 Charls Physical Examination	1	0.24
6 Other	15	3.67
Total	409	100.00

da009_1_: When Hypertension First Diagnosed or Known

	Freq.	%

	Freq.	%
1 Year	2,159	90.18
2 Age	210	8.77
99 Never Had It	25	1.04
Total	2,394	100.00

da009_1_1_: Year

Mean	SD	Min	Max	Obs
2,014.39	5.51	1,900.00	2,018.00	2,154

da009_2_1_: Age

Mean	SD	Min	Max	Obs
131.23	388.25	4.00	2,017.00	221

da009_2_: When Dyslipidemia First Diagnosed or Known

	Freq.	%
1 Year	1,776	90.80
2 Age	163	8.33
99 Never Had It	17	0.87
Total	1,956	100.00

da009_1_2_: Year

Mean	SD	Min	Max	Obs
2,010.86	82.10	20.00	2,018.00	1,775

da009_2_2_: Age

Mean	SD	Min	Max	Obs
97.90	298.35	0.00	2,018.00	170

da009_3_: When Diabetes First Diagnosed or Known

	Freq.	%
1 Year	1,002	93.21
2 Age	62	5.77
99 Never Had It	11	1.02
Total	1,075	100.00

da009_1_3_: Year

Mean	SD	Min	Max	Obs
2,015.02	4.56	1,969.00	2,018.00	1,000

da009_2_3_: Age

Mean	SD	Min	Max	Obs
114.03	344.52	0.00	2,017.00	64

da009_4_: When Cancer First Diagnosed or Known

	Freq.	%
1 Year	242	90.64
2 Age	21	7.87
99 Never Had It	4	1.50
Total	267	100.00

da009_1_4_: Year

Mean	SD	Min	Max	Obs
2,012.83	8.11	1,960.00	2,018.00	241

da009_2_4_: Age

Mean	SD	Min	Max	Obs
137.04	410.05	20.00	2,017.00	23

da009_5_: When Chronic Lung Diseases First Diagnosed or Known

	Freq.	%
1 Year	985	80.54
2 Age	203	16.60
99 Never Had It	35	2.86
Total	1,223	100.00

da009_1_5_: Year

Mean	SD	Min	Max	Obs
2,010.45	64.86	4.00	2,018.00	978

da009_2_5_: Age

Mean	SD	Min	Max	Obs
81.21	303.33	0.00	2,017.00	210

da009_6_: When Liver Disease First Diagnosed or Known

	Freq.	%
1 Year	558	86.38
2 Age	83	12.85
99 Never Had It	5	0.77
Total	646	100.00

da009_1_6_: Year

Mean	SD	Min	Max	Obs
2,004.91	114.06	22.00	2,018.00	560

da009_2_6_: Age

Mean	SD	Min	Max	Obs
61.93	216.67	11.00	2,018.00	84

da009_7_: When Heart Disease First Diagnosed or Known

	Freq.	%
1 Year	1,279	90.90
2 Age	114	8.10
99 Never Had It	14	1.00
Total	1,407	100.00

da009_1_7_: Year

Mean	SD	Min	Max	Obs
2,012.41	54.76	74.00	2,018.00	1,277

da009_2_7_: Age

Mean	SD	Min	Max	Obs
81.11	256.56	0.00	2,015.00	117

da009_8_: When Stroke First Diagnosed or Known

	Freq.	%
1 Year	887	90.05
2 Age	91	9.24
99 Never Had It	7	0.71
Total	985	100.00

da009_1_8_: Year

Mean	SD	Min	Max	Obs
2,008.62	94.75	20.00	2,018.00	889

da009_2_8_: Age

Mean	SD	Min	Max	Obs
117.90	345.80	18.00	2,014.00	94

da009_9_: When Kidney Disease First Diagnosed or Known

	Freq.	%
1 Year	697	89.13
2 Age	77	9.85
99 Never Had It	8	1.02
Total	782	100.00

da009_1_9_: Year

Mean	SD	Min	Max	Obs
2,010.59	76.03	16.00	2,018.00	696

da009_2_9_: Age

Mean	SD	Min	Max	Obs
140.68	429.96	0.00	2,018.00	81

da009_10_: When Digestive Disease First Diagnosed or Known

	Freq.	%
1 Year	1,359	81.18
2 Age	271	16.19
99 Never Had It	44	2.63
Total	1,674	100.00

da009_1_10_: Year

Mean	SD	Min	Max	Obs
2,004.34	117.84	20.00	2,018.00	1,354

da009_2_10_: Age

Mean	SD	Min	Max	Obs
58.94	205.25	5.00	2,016.00	275

da009_11_: When Emotional Problems First Diagnosed or Known

	Freq.	%
1 Year	322	79.12
2 Age	56	13.76
99 Never Had It	29	7.13
Total	407	100.00

da009_1_11_: Year

Mean	SD	Min	Max	Obs
2,010.60	10.09	1,963.00	2,018.00	317

da009_2_11_: Age

Mean	SD	Min	Max	Obs
36.02	19.05	0.00	81.00	56

da009_12_: When Memory-Related Disease First Diagnosed or Known

	Freq.	%
1 Year	417	91.05
2 Age	30	6.55
99 Never Had It	11	2.40
Total	458	100.00

da009_1_12_: Year

Mean	SD	Min	Max	Obs
2,013.86	7.50	1,900.00	2,018.00	416

da009_2_12_: Age

Mean	SD	Min	Max	Obs
119.32	352.29	0.00	2,015.00	31

da009_13_: When Arthritis First Diagnosed or Known

	Freq.	%
1 Year	1,393	82.18
2 Age	255	15.04
99 Never Had It	47	2.77
Total	1,695	100.00

da009_1_13_: Year

Mean	SD	Min	Max	Obs
2,006.98	92.63	2.00	2,018.00	1,386

da009_2_13_: Age

Mean	SD	Min	Max	Obs
79.19	270.70	0.00	2,016.00	262

da009_14_: When Arthritis First Diagnosed or Known

	Freq.	%
1 Year	349	78.25
2 Age	89	19.96
99 Never Had It	8	1.79
Total	446	100.00

da009_1_14_: Year

Mean	SD	Min	Max	Obs
2,006.43	105.34	43.00	2,018.00	351

da009_2_14_: Age

Mean	SD	Min	Max	Obs
56.59	210.04	0.00	2,017.00	90

da010_w4_2__s1: Treatments to Condition in DA007[1]

	Freq.	%
0 No	3,730	85.92
1 Taking Chinese Traditional Medicine	611	14.08
Total	4,341	100.00

da010_w4_2__s2: Treatments to Condition in DA007[2]

	Freq.	%
0 No	2,128	49.02
2 Taking Western Modern Medicine	2,213	50.98
Total	4,341	100.00

da010_w4_2__s3: Treatments to Condition in DA007[3]

	Freq.	%
0 No	3,836	88.37
3 Other Treatments, Please Specify	505	11.63
Total	4,341	100.00

da010_w4_2__s4: Treatments to Condition in DA007[4]

	Freq.	%
0 No	2,937	67.66
4 None of the Above	1,404	32.34
Total	4,341	100.00

da010_w4_2__s99: Treatments to Condition in DA007[99]

	Freq.	%
0 No	4,068	93.71
99 Never Had the Disease	273	6.29
Total	4,341	100.00

da010_w4_5__s1: Treatments to Condition in DA007[1]

	Freq.	%

	Freq.	%
0 No	2,588	80.50
1 Taking Chinese Traditional Medicine	627	19.50
Total	3,215	100.00

da010_w4_5__s2: Treatments to Condition in DA007[2]

	Freq.	%
0 No	1,712	53.25
2 Taking Western Modern Medicine	1,503	46.75
Total	3,215	100.00

da010_w4_5__s3: Treatments to Condition in DA007[3]

	Freq.	%
0 No	2,865	89.11
3 Other Treatments, Please Specify	350	10.89
Total	3,215	100.00

da010_w4_5__s4: Treatments to Condition in DA007[4]

	Freq.	%
0 No	2,207	68.65
4 None of the Above	1,008	31.35
Total	3,215	100.00

da010_w4_5__s99: Treatments to Condition in DA007[99]

	Freq.	%
0 No	2,798	87.03
99 Never Had the Disease	417	12.97
Total	3,215	100.00

da010_w4_6__s1: Treatments to Condition in DA007[1]

	Freq.	%
0 No	1,153	83.92
1 Taking Chinese Traditional Medicine	221	16.08
Total	1,374	100.00

da010_w4_6__s2: Treatments to Condition in DA007[2]

	Freq.	%
0 No	958	69.72
2 Taking Western Modern Medicine	416	30.28
Total	1,374	100.00

da010_w4_6__s3: Treatments to Condition in DA007[3]

	Freq.	%

	Freq.	%
0 No	1,273	92.65
3 Other Treatments, Please Specify	101	7.35
Total	1,374	100.00

da010_w4_6__s4: Treatments to Condition in DA007[4]

	Freq.	%
0 No	702	51.09
4 None of the Above	672	48.91
Total	1,374	100.00

da010_w4_6__s99: Treatments to Condition in DA007[99]

	Freq.	%
0 No	1,244	90.54
99 Never Had the Disease	130	9.46
Total	1,374	100.00

da010_w4_7__s1: Treatments to Condition in DA007[1]

	Freq.	%
0 No	3,016	77.35
1 Taking Chinese Traditional Medicine	883	22.65
Total	3,899	100.00

da010_w4_7__s2: Treatments to Condition in DA007[2]

	Freq.	%
0 No	1,738	44.58
2 Taking Western Modern Medicine	2,161	55.42
Total	3,899	100.00

da010_w4_7__s3: Treatments to Condition in DA007[3]

	Freq.	%
0 No	3,583	91.90
3 Other Treatments, Please Specify	316	8.10
Total	3,899	100.00

da010_w4_7__s4: Treatments to Condition in DA007[4]

	Freq.	%
0 No	2,868	73.56
4 None of the Above	1,031	26.44
Total	3,899	100.00

da010_w4_7__s99: Treatments to Condition in DA007[99]

	Freq.	%

0 No	3,668	94.08
99 Never Had the Disease	231	5.92
Total	3,899	100.00

da010_w4_9__s1: Treatments to Condition in DA007[1]

	Freq.	%
0 No	1,582	80.51
1 Taking Chinese Traditional Medicine	383	19.49
Total	1,965	100.00

da010_w4_9__s2: Treatments to Condition in DA007[2]

	Freq.	%
0 No	1,307	66.51
2 Taking Western Modern Medicine	658	33.49
Total	1,965	100.00

da010_w4_9__s3: Treatments to Condition in DA007[3]

	Freq.	%
0 No	1,774	90.28
3 Other Treatments, Please Specify	191	9.72
Total	1,965	100.00

da010_w4_9__s4: Treatments to Condition in DA007[4]

	Freq.	%
0 No	1,116	56.79
4 None of the Above	849	43.21
Total	1,965	100.00

da010_w4_9__s99: Treatments to Condition in DA007[99]

	Freq.	%
0 No	1,778	90.48
99 Never Had the Disease	187	9.52
Total	1,965	100.00

da010_w4_10__s1: Treatments to Condition in DA007[1]

	Freq.	%
0 No	4,779	80.32
1 Taking Chinese Traditional Medicine	1,171	19.68
Total	5,950	100.00

da010_w4_10__s2: Treatments to Condition in DA007[2]

	Freq.	%

	Freq.	%
0 No	2,958	49.71
2 Taking Western Modern Medicine	2,992	50.29
Total	5,950	100.00

da010_w4_10__s3: Treatments to Condition in DA007[3]

	Freq.	%
0 No	5,548	93.24
3 Other Treatments, Please Specify	402	6.76
Total	5,950	100.00

da010_w4_10__s4: Treatments to Condition in DA007[4]

	Freq.	%
0 No	3,943	66.27
4 None of the Above	2,007	33.73
Total	5,950	100.00

da010_w4_10__s99: Treatments to Condition in DA007[99]

	Freq.	%
0 No	5,647	94.91
99 Never Had the Disease	303	5.09
Total	5,950	100.00

da010_w4_12__s1: Treatments to Condition in DA007[1]

	Freq.	%
0 No	689	86.78
1 Taking Chinese Traditional Medicine	105	13.22
Total	794	100.00

da010_w4_12__s2: Treatments to Condition in DA007[2]

	Freq.	%
0 No	478	60.20
2 Taking Western Modern Medicine	316	39.80
Total	794	100.00

da010_w4_12__s3: Treatments to Condition in DA007[3]

	Freq.	%
0 No	737	92.82
3 Other Treatments, Please Specify	57	7.18
Total	794	100.00

da010_w4_12__s4: Treatments to Condition in DA007[4]

	Freq.	%

	Freq.	%
0 No	456	57.43
4 None of the Above	338	42.57
Total	794	100.00

da010_w4_12__s99: Treatments to Condition in DA007[99]

	Freq.	%
0 No	712	89.67
99 Never Had the Disease	82	10.33
Total	794	100.00

da010_w4_13__s1: Treatments to Condition in DA007[1]

	Freq.	%
0 No	6,140	79.89
1 Taking Chinese Traditional Medicine	1,546	20.11
Total	7,686	100.00

da010_w4_13__s2: Treatments to Condition in DA007[2]

	Freq.	%
0 No	4,755	61.87
2 Taking Western Modern Medicine	2,931	38.13
Total	7,686	100.00

da010_w4_13__s3: Treatments to Condition in DA007[3]

	Freq.	%
0 No	5,558	72.31
3 Other Treatments, Please Specify	2,128	27.69
Total	7,686	100.00

da010_w4_13__s4: Treatments to Condition in DA007[4]

	Freq.	%
0 No	5,124	66.67
4 None of the Above	2,562	33.33
Total	7,686	100.00

da010_w4_13__s99: Treatments to Condition in DA007[99]

	Freq.	%
0 No	7,205	93.74
99 Never Had the Disease	481	6.26
Total	7,686	100.00

da010_w2_1_1_: Blood Pressure Generally under Control

	Freq.	%

	Freq.	%
1 Yes	5,978	77.72
2 No	1,048	13.62
3 Don't Know	321	4.17
99 Never Had the Disease	345	4.49
Total	7,692	100.00

da010_w2_1_3_: Sugar Generally under Control

	Freq.	%
1 Yes	1,711	67.42
2 No	452	17.81
3 Don't Know	215	8.47
99 Never Had the Disease	160	6.30
Total	2,538	100.00

da010_w2_2_1_: Hypertension Compared to ZIWTime

	Freq.	%
1 Better	1,213	21.84
2 Worse	1,267	22.81
3 Same	2,727	49.09
99 Never Had the Disease	348	6.26
Total	5,555	100.00

da010_w2_2_2_: Dyslipidemia Compared to ZIWTime

	Freq.	%
1 Better	460	18.90
2 Worse	586	24.08
3 Same	1,136	46.67
99 Never Had the Disease	252	10.35
Total	2,434	100.00

da010_w2_2_3_: Diabetes Compared to ZIWTime

	Freq.	%
1 Better	277	18.65
2 Worse	408	27.47
3 Same	645	43.43
99 Never Had the Disease	155	10.44
Total	1,485	100.00

da010_w2_2_4_: Cancer Compared to ZIWTime

	Freq.	%
1 Better	81	42.63
2 Worse	17	8.95
3 Same	38	20.00
99 Never Had the Disease	54	28.42
Total	190	100.00

da010_w2_2_5_: Chronic Lung Diseases Compared to ZIWTime

	Freq.	%
1 Better	488	22.65
2 Worse	584	27.10
3 Same	675	31.32
99 Never Had the Disease	408	18.93
Total	2,155	100.00

da010_w2_2_6_: Liver Disease Compared to ZIWTime

	Freq.	%
1 Better	174	23.55
2 Worse	146	19.76
3 Same	294	39.78
99 Never Had the Disease	125	16.91
Total	739	100.00

da010_w2_2_7_: Heart Problems Compared to ZIWTime

	Freq.	%
1 Better	584	23.10
2 Worse	730	28.88
3 Same	992	39.24
99 Never Had the Disease	222	8.78
Total	2,528	100.00

da010_w2_2_8_: Stroke Compared to ZIWTime

	Freq.	%
1 Better	134	29.71
2 Worse	123	27.27
3 Same	141	31.26
99 Never Had the Disease	53	11.75
Total	451	100.00

da010_w2_2_9_: Kidney Disease Compared to ZIWTime

	Freq.	%
1 Better	244	20.28
2 Worse	332	27.60
3 Same	440	36.58
99 Never Had the Disease	187	15.54
Total	1,203	100.00

da010_w2_2_10_: Digestive Disease Compared to ZIWTime

	Freq.	%
1 Better	1,378	31.33
2 Worse	1,052	23.91
3 Same	1,700	38.65

	Freq.	%
99 Never Had the Disease	269	6.12
Total	4,399	100.00

da010_w2_2_11_: Emotional Problems Compared to ZIWTime

	Freq.	%
1 Better	74	17.09
2 Worse	111	25.64
3 Same	100	23.09
99 Never Had the Disease	148	34.18
Total	433	100.00

da010_w2_2_12_: Memory-Related Disease Compared to ZIWTime

	Freq.	%
1 Better	30	8.60
2 Worse	159	45.56
3 Same	83	23.78
99 Never Had the Disease	77	22.06
Total	349	100.00

da010_w2_2_13_: Arthritis Compared to ZIWTime

	Freq.	%
1 Better	1,086	17.60
2 Worse	2,592	42.02
3 Same	2,021	32.76
99 Never Had the Disease	470	7.62
Total	6,169	100.00

da010_w2_2_14_: Asthma Compared to ZIWTime

	Freq.	%
1 Better	209	27.54
2 Worse	239	31.49
3 Same	218	28.72
99 Never Had the Disease	93	12.25
Total	759	100.00

da011_w4_s1: Treatments to Hypertension

	Freq.	%
0 No	6,596	89.93
1 Taking Chinese Traditional Medicine	739	10.07
Total	7,335	100.00

da011_w4_s2: Treatments to Hypertension

	Freq.	%
0 No	2,023	27.58

	Freq.	%
2 Taking Western Modern Medicine	5,312	72.42
Total	7,335	100.00

da011_w4_s3: Treatments to Hypertension

	Freq.	%
0 No	6,862	93.55
3 Other Treatments	473	6.45
Total	7,335	100.00

da011_w4_s4: Treatments to Hypertension

	Freq.	%
0 No	5,856	79.84
4 None of the Above	1,479	20.16
Total	7,335	100.00

da011_w2_1: Ever Had Blood Pressure Checked

	Freq.	%
1 Yes	55	82.09
2 No	12	17.91
Total	67	100.00

da011_w4_1: Since ZIWTime Had Blood Pressure Checked

	Freq.	%
1 Yes	5,713	78.60
2 No	1,555	21.40
Total	7,268	100.00

da011_w2_2_1: Year

Mean	SD	Min	Max	Obs
2,017.78	1.68	1,900.00	2,018.00	5,751

da011_w2_2_2: Month

Mean	SD	Min	Max	Obs
5.73	2.18	0.00	12.00	5,724

da012: Times had Blood Pressure Checked during Last Year

Mean	SD	Min	Max	Obs
11.96	40.38	0.00	1,000.00	5,768

da012_w4: Blood Pressure Checked Regularly during Last Year

	Freq.	%
1 Yes	2,475	42.91
2 No	3,293	57.09
Total	5,768	100.00

da012_w3_1: How Often Had Blood Pressure Checked

	Freq.	%
1 Once a Week	230	9.29
2 Once Half a Month	296	11.96
3 Once a Month	630	25.45
4 Once Every Two Months	286	11.56
5 Once Every Three Months	329	13.29
6 Once Half a Year	365	14.75
7 Once a Year	339	13.70
Total	2,475	100.00

da012_w3_2: Pay for Blood Pressure Examination

	Freq.	%
1 Yes	87	3.52
2 No	2,388	96.48
Total	2,475	100.00

da013_s1: Care Providers Given Advice for Hypertension

	Freq.	%
0 No	5,047	68.81
1 Weight Control	2,288	31.19
Total	7,335	100.00

da013_s2: Care Providers Given Advice for Hypertension

	Freq.	%
0 No	4,304	58.68
2 Exercise	3,031	41.32
Total	7,335	100.00

da013_s3: Care Providers Given Advice for Hypertension

	Freq.	%
0 No	3,851	52.50
3 Diet	3,484	47.50
Total	7,335	100.00

da013_s4: Care Providers Given Advice for Hypertension

	Freq.	%

	Freq.	%
0 No	5,407	73.72
4 Smoking Control	1,928	26.28
Total	7,335	100.00

da013_s5: Care Providers Given Advice for Hypertension

	Freq.	%
0 No	4,222	57.56
5 None of the Above	3,113	42.44
Total	7,335	100.00

da014_w4_s1: Taking Treatments for Diabetes

	Freq.	%
0 No	2,117	89.32
1 Taking Chinese Traditional Medicine	253	10.68
Total	2,370	100.00

da014_w4_s2: Taking Treatments for Diabetes

	Freq.	%
0 No	1,001	42.24
2 Taking Western Modern Medicine	1,369	57.76
Total	2,370	100.00

da014_w4_s3: Taking Treatments for Diabetes

	Freq.	%
0 No	1,978	83.46
3 Taking Insulin Injections	392	16.54
Total	2,370	100.00

da014_w4_s4: Taking Treatments for Diabetes

	Freq.	%
0 No	2,187	92.28
4 Other Treatments, Please Specify	183	7.72
Total	2,370	100.00

da014_w4_s5: Taking Treatments for Diabetes

	Freq.	%
0 No	1,739	73.38
5 None of the Above	631	26.62
Total	2,370	100.00

da015_s1: Tests during Last Year

	Freq.	%
0 No	536	22.64
1 Blood Glucose Test	1,832	77.36
Total	2,368	100.00

da015_s2: Tests during Last Year

	Freq.	%
0 No	1,332	56.25
2 Urine Glucose Test	1,036	43.75
Total	2,368	100.00

da015_s3: Tests during Last Year

	Freq.	%
0 No	1,754	74.07
3 Fundus Examination	614	25.93
Total	2,368	100.00

da015_s4: Tests during Last Year

	Freq.	%
0 No	1,814	76.60
4 Micro-Albuminuria Test	554	23.40
Total	2,368	100.00

da015_s5: Tests during Last Year

	Freq.	%
0 No	1,888	79.73
5 None of the Above	480	20.27
Total	2,368	100.00

da015_1: Times Had Blood Glucose Test

Mean	SD	Min	Max	Obs
21.30	63.72	0.00	999.00	1,847

da015_2: Times Had Urine Glucose Test

Mean	SD	Min	Max	Obs
5.01	29.87	0.00	730.00	1,067

da015_3: Times Had Fundus Examination

Mean	SD	Min	Max	Obs
2.19	5.07	0.00	60.00	644

da015_4: Times Had Micro-Albuminuria Test

Mean	SD	Min	Max	Obs
2.39	5.40	0.00	100.00	576

da016_w4: Had Diabetes Examination Regularly

	Freq.	%
1 Yes	529	28.02
2 No	1,359	71.98
Total	1,888	100.00

da016_w3_1: How often Had Diabetes Examination

	Freq.	%
1 Once a Week	37	6.99
2 Once Half a Month	39	7.37
3 Once a Month	88	16.64
4 Once Every Two Months	51	9.64
5 Once Every Three Months	97	18.34
6 Once Half a Year	84	15.88
7 Once a Year	133	25.14
Total	529	100.00

da016_w3_2: Pay for Diabetes Examination

	Freq.	%
1 Yes	114	21.55
2 No	415	78.45
Total	529	100.00

da016_s1: Care Providers Given Advice for Diabetes Control

	Freq.	%
0 No	1,443	60.94
1 Weight Control	925	39.06
Total	2,368	100.00

da016_s2: Care Providers Given Advice for Diabetes Control

	Freq.	%
0 No	1,140	48.14
2 Exercise	1,228	51.86
Total	2,368	100.00

da016_s3: Care Providers Given Advice for Diabetes Control

	Freq.	%
0 No	863	36.44
3 Diet	1,505	63.56

	Freq.	%
Total	2,368	100.00

da016_s4: Care Providers Given Advice for Diabetes Control

	Freq.	%
0 No	1,683	71.07
4 Smoking Control	685	28.93
Total	2,368	100.00

da016_s5: Care Providers Given Advice for Diabetes Control

	Freq.	%
0 No	1,846	77.96
5 Foot Self-care	522	22.04
Total	2,368	100.00

da016_s6: Care Providers Given Advice for Diabetes Control

	Freq.	%
0 No	1,630	68.83
6 None of the Above	738	31.17
Total	2,368	100.00

da007_w2_5: Had Heart Attack since Last ZIWTime

	Freq.	%
1 Yes	885	38.39
2 No	1,420	61.61
Total	2,305	100.00

da007_w2_6: When Was the Most Recent Heart Attack

	Freq.	%
1 Year	876	99.10
2 Age	8	0.90
Total	884	100.00

da007_w2_6_1: Year

Mean	SD	Min	Max	Obs
2,017.59	0.71	2,012.00	2,018.00	874

da007_w2_6_2: Age

Mean	SD	Min	Max	Obs
549.83	885.21	2.00	2,018.00	12

da017_s1: Organ or Body Part have Cancer

	Freq.	%
0 No	377	95.44
1 Brain	18	4.56
Total	395	100.00

da017_s2: Organ or Body Part have Cancer

	Freq.	%
0 No	391	98.99
2 Oral cavity	4	1.01
Total	395	100.00

da017_s3: Organ or Body Part have Cancer

	Freq.	%
0 No	385	97.47
3 Larynx	10	2.53
Total	395	100.00

da017_s4: Organ or Body Part have Cancer

	Freq.	%
0 No	389	98.48
4 Other Pharynx	6	1.52
Total	395	100.00

da017_s5: Organ or Body Part have Cancer

	Freq.	%
0 No	376	95.19
5 Thyroid	19	4.81
Total	395	100.00

da017_s6: Organ or Body Part have Cancer

	Freq.	%
0 No	354	89.62
6 Lung	41	10.38
Total	395	100.00

da017_s7: Organ or Body Part have Cancer

	Freq.	%
0 No	343	86.84
7 Breast	52	13.16
Total	395	100.00

da017_s8: Organ or Body Part have Cancer

	Freq.	%
0 No	380	96.20
8 Oesophagus	15	3.80
Total	395	100.00

da017_s9: Organ or Body Part have Cancer

	Freq.	%
0 No	361	91.39
9 Stomach	34	8.61
Total	395	100.00

da017_s10: Organ or Body Part have Cancer

	Freq.	%
0 No	369	93.42
10 Liver	26	6.58
Total	395	100.00

da017_s11: Organ or Body Part have Cancer

	Freq.	%
0 No	392	99.24
11 Pancreas	3	0.76
Total	395	100.00

da017_s12: Organ or Body Part have Cancer

	Freq.	%
0 No	382	96.71
12 Kidney	13	3.29
Total	395	100.00

da017_s13: Organ or Body Part have Cancer

	Freq.	%
0 No	390	98.73
13 Prostate	5	1.27
Total	395	100.00

da017_s14: Organ or Body Part have Cancer

	Freq.	%
0 No	395	100.00
Total	395	100.00

da017_s15: Organ or Body Part have Cancer

	Freq.	%
0 No	382	96.71
15 Ovary	13	3.29
Total	395	100.00

da017_s16: Organ or Body Part have Cancer

	Freq.	%
0 No	345	87.34
16 Cervix	50	12.66
Total	395	100.00

da017_s17: Organ or Body Part have Cancer

	Freq.	%
0 No	361	91.39
17 Endometrium	34	8.61
Total	395	100.00

da017_s18: Organ or Body Part have Cancer

	Freq.	%
0 No	362	91.65
18 Colon or Rectum	33	8.35
Total	395	100.00

da017_s19: Organ or Body Part have Cancer

	Freq.	%
0 No	384	97.22
19 Bladder	11	2.78
Total	395	100.00

da017_s20: Organ or Body Part have Cancer

	Freq.	%
0 No	393	99.49
20 Skin	2	0.51
Total	395	100.00

da017_s21: Organ or Body Part have Cancer

	Freq.	%
0 No	387	97.97
21 Non-Hodgkin lymphoma	8	2.03
Total	395	100.00

da017_s22: Organ or Body Part have Cancer

	Freq.	%
0 No	390	98.73
22 Leukemia	5	1.27
Total	395	100.00

da017_s23: Organ or Body Part have Cancer

	Freq.	%
0 No	340	86.08
23 Other organ	55	13.92
Total	395	100.00

da018_w4_s1: Treatments for Cancer in the Past Two Years

	Freq.	%
0 No	313	79.24
1 Taking Chinese Traditional Medicine	82	20.76
Total	395	100.00

da018_w4_s2: Treatments for Cancer in the Past Two Years

	Freq.	%
0 No	244	61.77
2 Taking Western Modern Medicine	151	38.23
Total	395	100.00

da018_w4_s3: Treatments for Cancer in the Past Two Years

	Freq.	%
0 No	313	79.24
3 Chemotherapy	82	20.76
Total	395	100.00

da018_w4_s4: Treatments for Cancer in the Past Two Years

	Freq.	%
0 No	258	65.32
4 Surgery	137	34.68
Total	395	100.00

da018_w4_s5: Treatments for Cancer in the Past Two Years

	Freq.	%
0 No	346	87.59
5 Radiation Therapy	49	12.41
Total	395	100.00

da018_w4_s6: Treatments for Cancer in the Past Two Years

	Freq.	%
0 No	371	93.92
6 Other Treatments, Please Specify	24	6.08
Total	395	100.00

da018_w4_s7: Treatments for Cancer in the Past Two Years

	Freq.	%
0 No	250	63.29
7 None of the Above	145	36.71
Total	395	100.00

da019_w4_s1: Treatments for Stroke

	Freq.	%
0 No	1,079	78.64
1 Taking Chinese Traditional Medicine	293	21.36
Total	1,372	100.00

da019_w4_s2: Treatments for Stroke

	Freq.	%
0 No	582	42.42
2 Taking Western Modern Medicine	790	57.58
Total	1,372	100.00

da019_w4_s3: Treatments for Stroke

	Freq.	%
0 No	1,307	95.26
3 Physical Therapy	65	4.74
Total	1,372	100.00

da019_w4_s4: Treatments for Stroke

	Freq.	%
0 No	1,259	91.76
4 Acupuncture and Moxibustion	113	8.24
Total	1,372	100.00

da019_w4_s5: Treatments for Stroke

	Freq.	%
0 No	1,301	94.83
5 Occupational Therapy	71	5.17
Total	1,372	100.00

da019_w4_s6: Treatments for Stroke

	Freq.	%
0 No	1,251	91.18
6 Other Treatments, Please Specify	121	8.82
Total	1,372	100.00

da019_w4_s7: Treatments for Stroke

	Freq.	%
0 No	964	70.26
7 None of the Above	408	29.74
Total	1,372	100.00

da019_w2_1: Since Last ZIWTime Been Told Having Another Stroke

	Freq.	%
1 Yes	65	16.33
2 No	333	83.67
Total	398	100.00

da019_w2_2: When Was the Most Recent Stroke

	Freq.	%
1 Year	64	98.46
2 Age	1	1.54
Total	65	100.00

da019_w2_2_1: Year

Mean	SD	Min	Max	Obs
1,985.59	252.14	0.00	2,018.00	64

da019_w2_2_2: Age

Mean	SD	Min	Max	Obs
0.00	.	0.00	0.00	1

da020_w4_s1: Treatments for Emotional Problems

	Freq.	%
0 No	585	91.12
1 Receiving Psychiatric or Psychological Treatment	57	8.88
Total	642	100.00

da020_w4_s2: Treatments for Emotional Problems

	Freq.	%
0 No	541	84.27

	Freq.	%
2 Taking Anti Depressants	101	15.73
Total	642	100.00

da020_w4_s3: Treatments for Emotional Problems

	Freq.	%
0 No	508	79.13
3 Taking Tranquilizers or Sleeping Pills	134	20.87
Total	642	100.00

da020_w4_s4: Treatments for Emotional Problems

	Freq.	%
0 No	583	90.81
4 Other Treatments, Please Specify	59	9.19
Total	642	100.00

da020_w4_s5: Treatments for Emotional Problems

	Freq.	%
0 No	253	39.41
5 None of the Above	389	60.59
Total	642	100.00

da021: Traffic Accident/Injury and Received Medical Treatment

	Freq.	%
1 Yes	24	7.77
2 No	285	92.23
Total	309	100.00

da022: Traffic Accident/Injury Limit Daily Activities

	Freq.	%
1 Yes	366	46.62
2 No	419	53.38
Total	785	100.00

da023: Fallen Down

	Freq.	%
1 Yes	89	28.80
2 No	220	71.20
Total	309	100.00

da023_w4: Fallen Down since ZIWTime

	Freq.	%
1 Yes	3,805	19.58

	Freq.	%
2 No	15,627	80.42
Total	19,432	100.00

da024: Times Fallen Down Needing Medical Treatment

Mean	SD	Min	Max	Obs
0.64	3.06	0.00	150.00	3,894

da025: Fractured Hip

	Freq.	%
1 Yes	3	0.97
2 No	306	99.03
Total	309	100.00

da025_w4: Fractured Hip since ZIWTime?

	Freq.	%
1 Yes	219	1.13
2 No	19,213	98.87
Total	19,432	100.00

da026: Time Began Menarche

	Freq.	%
1 Year	10	6.17
2 Age	149	91.98
99 Never Had It	3	1.85
Total	162	100.00

da026_1: Year

Mean	SD	Min	Max	Obs
719.00	1,094.80	11.00	1,980.00	3

da026_2: Age

Mean	SD	Min	Max	Obs
14.68	3.88	0.00	25.00	150

da027: Started Menopause

	Freq.	%
1 Yes	1,800	47.66
2 No	1,909	50.54
997 Don't Know	68	1.80
Total	3,777	100.00

da028: Time Began Menopause

	Freq.	%
1 Year	762	42.33
2 Age	1,038	57.67
Total	1,800	100.00

da028_1: Year

Mean	SD	Min	Max	Obs
1,991.44	214.44	5.00	2,018.00	755

da028_2: Age

Mean	SD	Min	Max	Obs
52.41	86.30	0.00	2,015.00	1,042

da029: Diagnosed with a Prostate Illness

	Freq.	%
1 Yes	12	8.22
2 No	134	91.78
Total	146	100.00

da029_w4: Diagnosed with a Prostate Illness since ZIWTime

	Freq.	%
1 Yes	1,128	12.32
2 No	8,027	87.68
Total	9,155	100.00

da029_w2_1: Know having a Prostate Illness

	Freq.	%
1 Yes	415	5.09
2 No	6,113	74.91
3 Don't know	1,633	20.01
Total	8,161	100.00

da030: Time Diagnosed with a Prostate Illness

	Freq.	%
1 Year	778	92.51
2 Age	63	7.49
Total	841	100.00

da030_1: Year

Mean	SD	Min	Max	Obs
2,009.61	96.67	20.00	2,018.00	778

da030_2: Age

Mean	SD	Min	Max	Obs
112.52	341.97	5.00	2,018.00	65

da031: Taking Medication for Prostate Illness

	Freq.	%
1 Yes	526	33.83
2 No	1,029	66.17
Total	1,555	100.00

da032: Usually Wear Glasses

	Freq.	%
1 Yes	2,111	10.69
2 Legally Blind	55	0.28
3 No	14,594	73.93
4 Sometimes	2,980	15.10
Total	19,740	100.00

da033: Eyesight at a Distance

	Freq.	%
1 Excellent	435	2.21
2 Very Good	2,346	11.92
3 Good	2,880	14.63
4 Fair	9,931	50.45
5 Poor	4,037	20.51
997 Don't Know	56	0.28
Total	19,685	100.00

da034: Eyesight up Close

	Freq.	%
1 Excellent	362	1.84
2 Very Good	2,214	11.25
3 Good	3,312	16.82
4 Fair	9,859	50.08
5 Poor	3,881	19.72
997 Don't Know	57	0.29
Total	19,685	100.00

da035: Cataract Surgery

	Freq.	%

	Freq.	%
1 Yes	4	1.30
2 No	304	98.70
Total	308	100.00

da035_w2_1: Another Cataract Surgery since ZIWTime

	Freq.	%
1 Yes	53	22.46
2 No	183	77.54
Total	236	100.00

da035_w2_2: Had Cataract Surgery since ZIWTime

	Freq.	%
1 Yes	346	1.83
2 No	18,530	98.17
Total	18,876	100.00

da036: Had Cataract Surgery on Both Eyes or Just One

	Freq.	%
1 Yes	234	66.86
2 No	116	33.14
Total	350	100.00

da037: Treated for Glaucoma

	Freq.	%
1 Yes	249	1.29
2 No	19,063	98.71
Total	19,312	100.00

da037_w2: Has Glaucoma Relapsed since ZIWTime

	Freq.	%
1 Yes	74	19.84
2 No	201	53.89
3 Never Had Glaucoma	98	26.27
Total	373	100.00

da038_w4: Wear a Hearing Aid

	Freq.	%
1 Yes	132	0.67
2 No	19,607	99.33
Total	19,739	100.00

da039: Hearing Status

	Freq.	%
1 Excellent	414	2.10
2 Very Good	2,892	14.65
3 Good	3,601	18.24
4 Fair	10,021	50.77
5 Poor	2,769	14.03
997 Don't Know	42	0.21
Total	19,739	100.00

da040: Lost All Teeth

	Freq.	%
1 Yes	920	5.27
2 No	16,536	94.73
Total	17,456	100.00

da040_w4_1: Wear Denture

	Freq.	%
1 Yes	5,190	29.73
2 No	12,266	70.27
Total	17,456	100.00

da040_w4_2: Difficulty Chewing Hard Food

	Freq.	%
1 Yes	6,684	38.29
2 No	10,675	61.15
3 Not Applicable	97	0.56
Total	17,456	100.00

da041_w4: Troubled with Body Pain

	Freq.	%
1 None	7,820	39.62
2 A little	6,048	30.64
3 Somewhat	2,334	11.83
4 Quite a Bit	1,940	9.83
5 Very	1,595	8.08
Total	19,737	100.00

da042_s1: Body Parts Feeling Pain

	Freq.	%
0 No	6,922	58.09
1 Head (Headache)	4,995	41.91
Total	11,917	100.00

da042_s2: Body Parts Feeling Pain

	Freq.	%
0 No	6,716	56.36
2 Shoulder	5,201	43.64
Total	11,917	100.00

da042_s3: Body Parts Feeling Pain

	Freq.	%
0 No	7,953	66.74
3 Arm	3,964	33.26
Total	11,917	100.00

da042_s4: Body Parts Feeling Pain

	Freq.	%
0 No	9,240	77.54
4 Wrist	2,677	22.46
Total	11,917	100.00

da042_s5: Body Parts Feeling Pain

	Freq.	%
0 No	9,072	76.13
5 Fingers	2,845	23.87
Total	11,917	100.00

da042_s6: Body Parts Feeling Pain

	Freq.	%
0 No	9,838	82.55
6 Chest	2,079	17.45
Total	11,917	100.00

da042_s7: Body Parts Feeling Pain

	Freq.	%
0 No	8,611	72.26
7 Stomach (Stomachache)	3,306	27.74
Total	11,917	100.00

da042_s8: Body Parts Feeling Pain

	Freq.	%
0 No	8,143	68.33
8 Back	3,774	31.67
Total	11,917	100.00

da042_s9: Body Parts Feeling Pain

	Freq.	%
0 No	4,575	38.39
9 Waist	7,342	61.61
Total	11,917	100.00

da042_s10: Body Parts Feeling Pain

	Freq.	%
0 No	10,102	84.77
10 Buttocks	1,815	15.23
Total	11,917	100.00

da042_s11: Body Parts Feeling Pain

	Freq.	%
0 No	6,891	57.82
11 Leg	5,026	42.18
Total	11,917	100.00

da042_s12: Body Parts Feeling Pain

	Freq.	%
0 No	6,248	52.43
12 Knees	5,669	47.57
Total	11,917	100.00

da042_s13: Body Parts Feeling Pain

	Freq.	%
0 No	9,285	77.91
13 Ankle	2,632	22.09
Total	11,917	100.00

da042_s14: Body Parts Feeling Pain

	Freq.	%
0 No	10,112	84.85
14 Toes	1,805	15.15
Total	11,917	100.00

da042_s15: Body Parts Feeling Pain

	Freq.	%
0 No	8,324	69.85
15 Neck	3,593	30.15
Total	11,917	100.00

da042_s16: Body Parts Feeling Pain

	Freq.	%
0 No	10,564	88.65
16 Other, Please Specify	1,353	11.35
Total	11,917	100.00

da042_w2_1_s1: Measures to Reduce Pain

	Freq.	%
0 No	9,673	81.17
1 Taking Chinese Traditional Medicine	2,244	18.83
Total	11,917	100.00

da042_w2_1_s2: Measures to Reduce Pain

	Freq.	%
0 No	6,321	53.04
2 Taking Western Modern Medicine	5,596	46.96
Total	11,917	100.00

da042_w2_1_s3: Measures to Reduce Pain

	Freq.	%
0 No	10,536	88.41
3 Acupuncture Treatment	1,381	11.59
Total	11,917	100.00

da042_w2_1_s4: Measures to Reduce Pain

	Freq.	%
0 No	10,548	88.51
4 Professional Massage Therapy	1,369	11.49
Total	11,917	100.00

da042_w2_1_s5: Measures to Reduce Pain

	Freq.	%
0 No	9,681	81.24
5 Other, Please Specify	2,236	18.76
Total	11,917	100.00

da042_w2_1_s6: Measures to Reduce Pain

	Freq.	%
0 No	8,173	68.58
6 None	3,744	31.42
Total	11,917	100.00

da045: Other Medical Diseases or Conditions

	Freq.	%
1 Yes	1,358	6.88
2 No	18,379	93.12
Total	19,737	100.00

da048: Evaluate Health during Childhood

	Freq.	%
1 Excellent	2,282	12.34
2 Very Good	7,433	40.18
3 Good	3,711	20.06
4 Fair	3,864	20.89
5 Poor	1,019	5.51
997 Don't Know	191	1.03
Total	18,500	100.00

da049: During Last Month Average Hours of Actual Sleep

Mean	SD	Min	Max	Obs
6.24	2.02	0.00	24.00	19,735

da050: During Last Month Time for a Nap after Lunch

Mean	SD	Min	Max	Obs
41.41	50.28	0.00	999.00	19,733

da051_1_: Intensive Physical Activity More than 10 Mins Each Time

	Freq.	%
1 Yes	6,221	31.53
2 No	13,511	68.47
Total	19,732	100.00

da051_2_: Moderate Physical Activity More than 10 Mins Each Time

	Freq.	%
1 Yes	9,625	48.78
2 No	10,107	51.22
Total	19,732	100.00

da051_3_: Light Physical Activity More than 10 Mins Each Time

	Freq.	%
1 Yes	16,285	82.53
2 No	3,447	17.47
Total	19,732	100.00

da052_1_: Days with Intensive Physical Activity

Mean	SD	Min	Max	Obs
5.36	2.13	1.00	7.00	6,221

da052_2_: Days with Moderate Physical Activity

Mean	SD	Min	Max	Obs
5.61	2.06	1.00	7.00	9,625

da052_3_: Days with Light Physical Activity

Mean	SD	Min	Max	Obs
6.47	1.39	1.00	7.00	16,285

da053_1_: Time with Intensive Physical Activity

	Freq.	%
1 <2 Hours	1,374	22.09
2 >=2 Hours	4,847	77.91
Total	6,221	100.00

da053_2_: Time with Moderate Physical Activity

	Freq.	%
1 <2 Hours	5,165	53.66
2 >=2 Hours	4,460	46.34
Total	9,625	100.00

da053_3_: Time with Light Physical Activity

	Freq.	%
1 <2 Hours	9,989	61.34
2 >=2 Hours	6,296	38.66
Total	16,285	100.00

da054_1_: Time with Intensive Physical Activity

	Freq.	%
1 <30 Minutes	196	14.26
2 >=30 Minutes	1,178	85.74
Total	1,374	100.00

da054_2_: Time with Moderate Physical Activity

	Freq.	%
1 <30 Minutes	1,197	23.18
2 >=30 Minutes	3,968	76.82
Total	5,165	100.00

da054_3_: Time with Light Physical Activity

	Freq.	%
1 <30 Minutes	1,924	19.26
2 >=30 Minutes	8,065	80.74
Total	9,989	100.00

da055_1_: Time with Intensive Physical Activity

	Freq.	%
1 <4 Hours	1,406	29.01
2 >=4 Hours	3,441	70.99
Total	4,847	100.00

da055_2_: Time with Moderate Physical Activity

	Freq.	%
1 <4 Hours	2,195	49.22
2 >=4 Hours	2,265	50.78
Total	4,460	100.00

da055_3_: Time with Light Physical Activity

	Freq.	%
1 <4 Hours	3,640	57.81
2 >=4 Hours	2,656	42.19
Total	6,296	100.00

da051_1_1_: Purpose for Intensive Physical Activity

	Freq.	%
1 Job Demands	5,161	82.96
2 Entertainment	108	1.74
3 Exercise	640	10.29
4 Other	312	5.02
Total	6,221	100.00

da051_1_2_: Purpose for Moderate Physical Activity

	Freq.	%
1 Job Demands	5,225	54.29
2 Entertainment	255	2.65
3 Exercise	1,572	16.33
4 Other	2,573	26.73
Total	9,625	100.00

da051_1_3_: Purpose for Light Physical Activity

	Freq.	%

	Freq.	%
1 Job Demands	6,825	41.91
2 Entertainment	1,477	9.07
3 Exercise	6,031	37.03
4 Other	1,952	11.99
Total	16,285	100.00

da056_s1: Activities in Last Month

	Freq.	%
0 No	13,072	66.25
1 Interacted With Friends	6,658	33.75
Total	19,730	100.00

da056_s2: Activities in Last Month

	Freq.	%
0 No	16,485	83.55
2 Played Ma-jong, Played Chess, Played Cards, or Went to Community Club	3,245	16.45
Total	19,730	100.00

da056_s3: Activities in Last Month

	Freq.	%
0 No	16,980	86.06
3 Provided Help to Family, Friends, or Neighbors Who Do Not Live With You	2,750	13.94
Total	19,730	100.00

da056_s4: Activities in Last Month

	Freq.	%
0 No	18,425	93.39
4 Went to a Sport, Social, or Other Kind of Club	1,305	6.61
Total	19,730	100.00

da056_s5: Activities in Last Month

	Freq.	%
0 No	19,251	97.57
5 Took Part in a Community-Related Organization	479	2.43
Total	19,730	100.00

da056_s6: Activities in Last Month

	Freq.	%
0 No	19,393	98.29
6 Done Voluntary or Charity work	337	1.71
Total	19,730	100.00

da056_s7: Activities in Last Month

	Freq.	%
0 No	19,154	97.08
7 Cared for a Sick or Disabled Adult Who Does Not Live With You	576	2.92
Total	19,730	100.00

da056_s8: Activities in Last Month

	Freq.	%
0 No	19,562	99.15
8 Attended an Educational or Training Course	168	0.85
Total	19,730	100.00

da056_s9: Activities in Last Month

	Freq.	%
0 No	19,618	99.43
9 Stock Investment	112	0.57
Total	19,730	100.00

da056_s10: Activities in Last Month

	Freq.	%
0 No	17,077	86.55
10 Used the Internet	2,653	13.45
Total	19,730	100.00

da056_s11: Activities in Last Month

	Freq.	%
0 No	19,457	98.62
11 Other	273	1.38
Total	19,730	100.00

da056_s12: Activities in Last Month

	Freq.	%
0 No	10,494	53.19
12 None of These	9,236	46.81
Total	19,730	100.00

da056_w3_s1: How to Use Internet

	Freq.	%
0 No	2,143	80.78
1 Desktop Computer	510	19.22
Total	2,653	100.00

da056_w3_s2: How to Use Internet

	Freq.	%
0 No	2,497	94.12
2 Laptop Computer	156	5.88
Total	2,653	100.00

da056_w3_s3: How to Use Internet

	Freq.	%
0 No	2,490	93.86
3 Tablet Computer	163	6.14
Total	2,653	100.00

da056_w3_s4: How to Use Internet

	Freq.	%
0 No	129	4.86
4 Cellphone	2,524	95.14
Total	2,653	100.00

da056_w3_s5: How to Use Internet

	Freq.	%
0 No	2,646	99.74
5 Other Devices	7	0.26
Total	2,653	100.00

da057_1_: Frequency of Activities[1]

	Freq.	%
1 Almost Daily	2,509	37.68
2 Almost Every Week	1,570	23.58
3 Not Regularly	2,579	38.74
Total	6,658	100.00

da057_2_: Frequency of Activities[2]

	Freq.	%
1 Almost Daily	926	28.54
2 Almost Every Week	1,028	31.68
3 Not Regularly	1,291	39.78
Total	3,245	100.00

da057_3_: Frequency of Activities[3]

	Freq.	%
1 Almost Daily	268	9.75
2 Almost Every Week	559	20.33

3 Not Regularly	1,923	69.93
Total	2,750	100.00

da057_4_: Frequency of Activities[4]

	Freq.	%
1 Almost Daily	829	63.52
2 Almost Every Week	256	19.62
3 Not Regularly	220	16.86
Total	1,305	100.00

da057_5_: Frequency of Activities[5]

	Freq.	%
1 Almost Daily	62	12.94
2 Almost Every Week	130	27.14
3 Not Regularly	287	59.92
Total	479	100.00

da057_6_: Frequency of Activities[6]

	Freq.	%
1 Almost Daily	16	4.75
2 Almost Every Week	50	14.84
3 Not Regularly	271	80.42
Total	337	100.00

da057_7_: Frequency of Activities[7]

	Freq.	%
1 Almost Daily	138	23.96
2 Almost Every Week	126	21.88
3 Not Regularly	312	54.17
Total	576	100.00

da057_8_: Frequency of Activities[8]

	Freq.	%
1 Almost Daily	19	11.31
2 Almost Every Week	39	23.21
3 Not Regularly	110	65.48
Total	168	100.00

da057_9_: Frequency of Activities[9]

	Freq.	%
1 Almost Daily	57	50.89
2 Almost Every Week	25	22.32
3 Not Regularly	30	26.79
Total	112	100.00

da057_10_: Frequency of Activities[10]

	Freq.	%
1 Almost Daily	2,207	83.19
2 Almost Every Week	199	7.50
3 Not Regularly	247	9.31
Total	2,653	100.00

da057_11_: Frequency of Activities[11]

	Freq.	%
1 Almost Daily	76	27.84
2 Almost Every Week	88	32.23
3 Not Regularly	109	39.93
Total	273	100.00

da056_w4_1_s1: Activities with Internet

	Freq.	%
0 No	923	34.79
1 Chatting	1,730	65.21
Total	2,653	100.00

da056_w4_1_s2: Activities with Internet

	Freq.	%
0 No	567	21.37
2 Reading News	2,086	78.63
Total	2,653	100.00

da056_w4_1_s3: Activities with Internet

	Freq.	%
0 No	942	35.51
3 Watching Videos	1,711	64.49
Total	2,653	100.00

da056_w4_1_s4: Activities with Internet

	Freq.	%
0 No	2,066	77.87
4 Playing Games	587	22.13
Total	2,653	100.00

da056_w4_1_s5: Activities with Internet

	Freq.	%
0 No	2,481	93.52
5 Money Management	172	6.48

	Freq.	%
Total	2,653	100.00

da056_w4_1_s6: Activities with Internet

	Freq.	%
0 No	2,227	83.94
6 Others, Please Specify	426	16.06
Total	2,653	100.00

da056_w4_2: Payment with Mobile Phone

	Freq.	%
1 Yes	1,600	60.31
2 No	1,053	39.69
Total	2,653	100.00

da056_w4_3: Use Wechat

	Freq.	%
1 Yes	2,516	94.84
2 No	137	5.16
Total	2,653	100.00

da056_w4_4: Use Wechat Moments

	Freq.	%
1 Yes	1,896	75.36
2 No	620	24.64
Total	2,516	100.00

da059: Ever Smoked

	Freq.	%
1 Yes	911	7.38
2 No	11,439	92.62
Total	12,350	100.00

da061: Still Smoke or already Quit

	Freq.	%
1 Still smoke	465	51.04
2 Quit	446	48.96
Total	911	100.00

da061_w4: Still Smoke or already Quit

	Freq.	%
1 Still Smoke	4,801	65.06
2 Quit	2,378	32.23

	Freq.	%
3 Never Smoked	200	2.71
Total	7,379	100.00

da060: Products Normally Use

	Freq.	%
1 Smoking a Pipe	563	6.96
2 Smoking Self-Rolled Cigarettes	727	8.99
3 Filtered Cigarette	6,039	74.65
4 Unfiltered Cigarette	515	6.37
5 Cigar	3	0.04
6 Water Cigarettes	243	3.00
Total	8,090	100.00

da062: Time Totally Quit Smoking

	Freq.	%
1 Year	2,300	81.44
2 Age	524	18.56
Total	2,824	100.00

da062_1: Year

Mean	SD	Min	Max	Obs
2,003.25	116.07	2.00	2,018.00	2,305

da062_2: Age

Mean	SD	Min	Max	Obs
91.74	302.45	0.00	2,018.00	539

da063: Cigarettes in One Day

Mean	SD	Min	Max	Obs
18.91	19.55	0.00	1,200.00	6,554

da065: Time Started Smoking on a Regular Basis

	Freq.	%
1 Year	68	7.29
2 Age	865	92.71
Total	933	100.00

da065_1: Year

Mean	SD	Min	Max	Obs
1,474.15	871.41	2.00	2,018.00	88

da065_2: Age

Mean	SD	Min	Max	Obs
25.77	95.45	0.00	2,017.00	867

da067: Frequency Drank Alcoholic Beverages in the Past Year

	Freq.	%
1 Drink More than Once a Month	5,147	26.09
2 Drink But Less than Once a Month	1,490	7.55
3 None of These	13,092	66.36
Total	19,729	100.00

da068_s1: Alcoholic Beverages

	Freq.	%
0 No	1,418	27.55
1 Liquor, Including White Liquor, Whisky, and Others	3,729	72.45
Total	5,147	100.00

da068_s2: Alcoholic Beverages

	Freq.	%
0 No	2,939	57.10
2 Beer	2,208	42.90
Total	5,147	100.00

da068_s3: Alcoholic Beverages

	Freq.	%
0 No	4,248	82.53
3 Wine or Rice Wine	899	17.47
Total	5,147	100.00

da069: Whether Drank Alcoholic Beverages in the Past and Frequency

	Freq.	%
1 I Never Had a Drink	1,114	50.07
2 I Used to Drink Less than Once a Month	353	15.87
3 I Used to Drink More Than Once a Month	758	34.07
Total	2,225	100.00

da070: Time Quit or Reduce Drinking

	Freq.	%
1 Year	691	85.73
2 Age	115	14.27
Total	806	100.00

da070_1: Year

Mean	SD	Min	Max	Obs
2,004.56	132.59	-1.00	2,018.00	692

da070_2: Age

Mean	SD	Min	Max	Obs
81.96	255.13	0.00	2,016.00	118

da071: Time Started Drinking

	Freq.	%
1 Year	291	17.48
2 Age	1,374	82.52
Total	1,665	100.00

da071_1: Year

Mean	SD	Min	Max	Obs
1,832.81	556.88	0.00	2,018.00	291

da071_2: Age

Mean	SD	Min	Max	Obs
29.39	106.47	0.00	2,017.00	1,379

da072: Frequncy Drinking Liquor Per Month in the Last Year

	Freq.	%
1 Once a Month	314	8.42
2 Two or Three Times a Month	661	17.73
3 Once a Week	293	7.86
4 Two or Three Times a Week	497	13.33
5 Four to six times a Week	178	4.77
6 Once a Day	1,087	29.15
7 Twice a Day	521	13.97
8 More than Twice a Day	178	4.77
Total	3,729	100.00

da073: Liang of Liquor Drank Last Time

Mean	SD	Min	Max	Obs
37.80	298.25	0.00	10,950.00	3,729

da074: Frequncy Drinking Beer per Month in the Last Year

	Freq.	%

	Freq.	%
1 Once a Month	315	14.27
2 Two or Three Times a Month	464	21.02
3 Once a Week	269	12.19
4 Two or Three Times a Week	366	16.58
5 Four to Six Times a Week	106	4.80
6 Once a Day	469	21.25
7 Twice a Day	185	8.38
8 More than Twice a Day	33	1.50
Total	2,207	100.00

da075: Amount of Beer Drank Last Time

	Freq.	%
1 Bottle	1,959	88.76
2 Mug	248	11.24
Total	2,207	100.00

da075_1: Bottle

Mean	SD	Min	Max	Obs
5.53	56.96	0.00	1,800.00	1,966

da075_2: Mug

Mean	SD	Min	Max	Obs
2.07	7.93	0.20	90.00	252

da076: Frequncy Drinking Wine per Month in the Last Year

	Freq.	%
1 Once a Month	220	24.47
2 Two or Three Times a Month	138	15.35
3 Once a Week	81	9.01
4 Two or Three Times a Week	91	10.12
5 Four to Six Times a Week	35	3.89
6 Once a Day	189	21.02
7 Twice a Day	84	9.34
8 More Than Twice a Day	61	6.79
Total	899	100.00

da077: Liang of Wine Drank Last Time

Mean	SD	Min	Max	Obs
24.25	222.86	0.00	4,380.00	899

da081: Life Expectancy

	Freq.	%
1 Almost Impossible	2,357	14.82
2 Not Very Likely	2,858	17.96

	Freq.	%
3 Maybe	6,136	38.57
4 Very Likely	1,913	12.02
5 Almost Certain	2,645	16.63
Total	15,909	100.00

db001: Difficulty with Running or Jogging about 1Km

	Freq.	%
1 Don't Have Any Difficulty	9,102	46.78
2 Have Difficulty But Can Still Do It	2,085	10.72
3 Have Difficulty and Need Help	289	1.49
4 Can Not Do It	7,981	41.02
Total	19,457	100.00

db002: Difficulty with Walking 1Km

	Freq.	%
1 Don't Have Any Difficulty	6,631	64.04
2 Have Difficulty But Can Still Do It	1,517	14.65
3 Have Difficulty and Need Help	195	1.88
4 Can Not Do It	2,011	19.42
Total	10,354	100.00

db003: Difficulty with Walking 100 Metres

	Freq.	%
1 Don't Have Any Difficulty	2,062	55.39
2 Have Difficulty But Can Still Do It	782	21.00
3 Have Difficulty and Need Help	127	3.41
4 Can Not Do It	752	20.20
Total	3,723	100.00

db004: Difficulty with Getting up from a Chair

	Freq.	%
1 Don't Have Any Difficulty	13,532	69.55
2 Have Difficulty But Can Still Do It	5,236	26.91
3 Have Difficulty and Need Help	397	2.04
4 Can Not Do It	291	1.50
Total	19,456	100.00

db005: Difficulty with Climbing Several Flights of Stairs Without Resting

	Freq.	%
1 Don't Have Any Difficulty	10,502	53.98
2 Have Difficulty But Can Still Do It	5,674	29.16
3 Have Difficulty and Need Help	535	2.75
4 Can Not Do It	2,744	14.10
Total	19,455	100.00

db006: Difficulty with Stooping, Kneeling, or Crouching

	Freq.	%
1 Don't Have Any Difficulty	12,372	63.59
2 Have Difficulty But Can Still Do It	4,403	22.63
3 Have Difficulty and Need Help	434	2.23
4 Can Not Do It	2,246	11.54
Total	19,455	100.00

db007: Difficulty with Reaching or Extending Your arms

	Freq.	%
1 Don't Have Any Difficulty	16,796	86.33
2 Have Difficulty But Can Still Do It	1,107	5.69
3 Have Difficulty and Need Help	136	0.70
4 Can Not Do It	1,416	7.28
Total	19,455	100.00

db008: Difficulty with Lifting or Carrying Weights over 10 Jin

	Freq.	%
1 Don't Have Any Difficulty	15,987	82.17
2 Have Difficulty But Can Still Do It	1,226	6.30
3 Have Difficulty and Need Help	310	1.59
4 Can Not Do It	1,932	9.93
Total	19,455	100.00

db009: Difficulty with Picking up a Small Coin

	Freq.	%
1 Don't Have Any Difficulty	18,408	94.62
2 Have Difficulty But Can Still Do It	455	2.34
3 Have Difficulty and Need Help	83	0.43
4 Can Not Do It	509	2.62
Total	19,455	100.00

db010: Difficulty with Dressing

	Freq.	%
1 Don't Have Any Difficulty	12,263	89.89
2 Have Difficulty But Can Still Do It	880	6.45
3 Have Difficulty and Need Help	345	2.53
4 Can Not Do It	154	1.13
Total	13,642	100.00

db010_w2: Anyone Ever Helped

	Freq.	%
1 Yes	628	45.54
2 No	751	54.46
Total	1,379	100.00

db011: Difficulty with Bathing or Showering

	Freq.	%
1 Don't Have Any Difficulty	11,966	87.72
2 Have Difficulty But Can Still Do It	719	5.27
3 Have Difficulty and Need Help	637	4.67
4 Can Not Do It	319	2.34
Total	13,641	100.00

db011_w2: Anyone Ever Helped

	Freq.	%
1 Yes	976	58.27
2 No	699	41.73
Total	1,675	100.00

db012: Difficulty with Eating

	Freq.	%
1 Don't Have Any Difficulty	13,099	96.03
2 Have Difficulty But Can Still Do It	343	2.51
3 Have Difficulty and Need Help	109	0.80
4 Can Not Do It	90	0.66
Total	13,641	100.00

db012_w2: Anyone Ever Helped

	Freq.	%
1 Yes	258	47.60
2 No	284	52.40
Total	542	100.00

db013: Difficulty with Getting into or out of Bed

	Freq.	%
1 Don't Have Any Difficulty	12,230	89.66
2 Have Difficulty But Can Still Do It	1,046	7.67
3 Have Difficulty and Need Help	236	1.73
4 Can Not Do It	129	0.95
Total	13,641	100.00

db013_w2: Anyone Ever Helped

	Freq.	%
1 Yes	459	32.53
2 No	952	67.47
Total	1,411	100.00

db014: Difficulty with Using the Toilet

	Freq.	%
1 Don't Have Any Difficulty	11,392	83.51
2 Have Difficulty But Can Still Do It	1,687	12.37
3 Have Difficulty and Need Help	312	2.29
4 Can Not Do It	250	1.83
Total	13,641	100.00

db014_w2: Anyone Ever Helped

	Freq.	%
1 Yes	422	18.76
2 No	1,827	81.24
Total	2,249	100.00

db015: Difficulty with Controlling Urination and Defecation

	Freq.	%
1 Don't Have Any Difficulty	12,733	93.34
2 Have Difficulty But Can Still Do It	585	4.29
3 Have Difficulty and Need Help	135	0.99
4 Can Not Do It	188	1.38
Total	13,641	100.00

db016: Difficulty with Household Chores

	Freq.	%
1 Don't Have Any Difficulty	16,814	85.26
2 Have Difficulty But Can Still Do It	1,242	6.30
3 Have Difficulty and Need Help	592	3.00
4 Can Not Do It	1,073	5.44
Total	19,721	100.00

db016_w2: Anyone Ever Helped

	Freq.	%
1 Yes	1,763	60.65
2 No	1,144	39.35
Total	2,907	100.00

db017: Difficulty with Preparing Hot Meals

	Freq.	%
1 Don't Have Any Difficulty	17,467	88.57
2 Have Difficulty But Can Still Do It	734	3.72
3 Have Difficulty and Need Help	412	2.09
4 Can Not Do It	1,108	5.62
Total	19,721	100.00

db017_w2: Anyone Ever Helped

	Freq.	%
1 Yes	1,601	71.03
2 No	653	28.97
Total	2,254	100.00

db018: Difficulty with Shopping for Groceries

	Freq.	%
1 Don't Have Any Difficulty	17,825	90.39
2 Have Difficulty But Can Still Do It	460	2.33
3 Have Difficulty and Need Help	358	1.82
4 Can Not Do It	1,078	5.47
Total	19,721	100.00

db018_w2: Anyone Ever Helped

	Freq.	%
1 Yes	1,513	79.80
2 No	383	20.20
Total	1,896	100.00

db035: Difficulty with Making Phone Calls

	Freq.	%
1 Don't Have Any Difficulty	16,480	83.57
2 Have Difficulty But Can Still Do It	373	1.89
3 Have Difficulty and Need Help	495	2.51
4 Can Not Do It	1,226	6.22
5 Not Relevant To Me (No Phone)	1,147	5.82
Total	19,721	100.00

db035_w2: Anyone Ever Helped

	Freq.	%
1 Yes	1,485	70.92
2 No	609	29.08
Total	2,094	100.00

db020: Difficulty with Taking Medications

	Freq.	%
1 Don't Have Any Difficulty	18,567	94.15
2 Have Difficulty But Can Still Do It	373	1.89
3 Have Difficulty and Need Help	518	2.63
4 Can Not Do It	263	1.33
Total	19,721	100.00

db020_w2: Anyone Ever Helped

	Freq.	%
1 Yes	785	68.02
2 No	369	31.98
Total	1,154	100.00

db019: Difficulty with Managing Money

	Freq.	%
1 Don't Have Any Difficulty	17,417	88.32
2 Have Difficulty But Can Still Do It	532	2.70
3 Have Difficulty and Need Help	561	2.84
4 Can Not Do It	1,211	6.14
Total	19,721	100.00

db019_w2: Anyone Ever Helped

	Freq.	%
1 Yes	1,597	69.31
2 No	707	30.69
Total	2,304	100.00

db022_w3_1_s1: Helper[1]

	Freq.	%
0 No	1,595	39.99
1 Spouse	2,393	60.01
Total	3,988	100.00

db022_w3_1_s2: Helper[2]

	Freq.	%
0 No	3,963	99.37
2 Father, Mother, Father-in-Law, Mother-in-Law	25	0.63
Total	3,988	100.00

db022_w3_1_s3: Helper[3]

	Freq.	%
0 No	1,920	48.14
3 Children, Children's Spouse, Grandson, Granddaughter	2,068	51.86
Total	3,988	100.00

db022_w3_1_s4: Helper[4]

	Freq.	%
0 No	3,866	96.94
4 Sibling, Brother-in-Law, Sister-in-Law, Sibling of Spouse, Etc	122	3.06
Total	3,988	100.00

db022_w3_1_s5: Helper[5]

	Freq.	%
0 No	3,944	98.90
5 Other Relative	44	1.10
Total	3,988	100.00

db022_w3_1_s6: Helper[6]

	Freq.	%
0 No	3,950	99.05
6 Paid Helper (Such as Nanny)	38	0.95
Total	3,988	100.00

db022_w3_1_s7: Helper[7]

	Freq.	%
0 No	3,985	99.92
7 Volunteer or Employee of Facility	3	0.08
Total	3,988	100.00

db022_w3_1_s8: Helper[8]

	Freq.	%
0 No	3,968	99.50
8 Nursing Home	20	0.50
Total	3,988	100.00

db022_w3_1_s9: Helper[9]

	Freq.	%
0 No	3,975	99.67
9 Help from the Community	13	0.33
Total	3,988	100.00

db022_w3_1_s10: Helper[10]

	Freq.	%
0 No	3,774	94.63
10 Other, Please Specify	214	5.37
Total	3,988	100.00

db022_w3_1_1: Number of Paid Helpers

Mean	SD	Min	Max	Obs
1.00	0.33	0.00	2.00	38

db023_w3_1_s1: Who Help the Most

	Freq.	%
0 No	20	80.00
1 Father	5	20.00
Total	25	100.00

db023_w3_1_s2: Who Help the Most

	Freq.	%
0 No	9	36.00
2 Mother	16	64.00
Total	25	100.00

db023_w3_1_s3: Who Help the Most

	Freq.	%
0 No	25	100.00
Total	25	100.00

db023_w3_1_s4: Who Help the Most

	Freq.	%
0 No	17	68.00
4 Mother-in-Law	8	32.00
Total	25	100.00

db023_w3_2_s1: Which Child's Family Helped

	Freq.	%
0 No	774	37.41
1 Preload XChildName[1]	1,295	62.59
Total	2,069	100.00

db023_w3_2_s2: Which Child's Family Helped

	Freq.	%
0 No	1,178	56.94
2 Preload XChildName[2]	891	43.06
Total	2,069	100.00

db023_w3_2_s3: Which Child's Family Helped

	Freq.	%
0 No	1,514	73.18
3 Preload XChildName[3]	555	26.82
Total	2,069	100.00

db023_w3_2_s4: Which Child's Family Helped

	Freq.	%
0 No	1,726	83.42
4 Preload XChildName[4]	343	16.58
Total	2,069	100.00

db023_w3_2_s5: Which Child's Family Helped

	Freq.	%
0 No	1,896	91.64
5 Preload XChildName[5]	173	8.36
Total	2,069	100.00

db023_w3_2_s6: Which Child's Family Helped

	Freq.	%
0 No	2,000	96.67
6 Preload XChildName[6]	69	3.33
Total	2,069	100.00

db023_w3_2_s7: Which Child's Family Helped

	Freq.	%
0 No	2,033	98.26
7 Preload XChildName[7]	36	1.74
Total	2,069	100.00

db023_w3_2_s8: Which Child's Family Helped

	Freq.	%
0 No	2,055	99.32
8 Preload XChildName[8]	14	0.68
Total	2,069	100.00

db023_w3_2_s9: Which Child's Family Helped

	Freq.	%
0 No	2,063	99.71
9 Preload XChildName[9]	6	0.29
Total	2,069	100.00

db023_w3_2_s10: Which Child's Family Helped

	Freq.	%
0 No	2,068	99.95
10 Preload XChildName[10]	1	0.05
Total	2,069	100.00

db023_w3_2_s11: Which Child's Family Helped

	Freq.	%
0 No	2,066	99.86
11 Preload XChildName[11]	3	0.14
Total	2,069	100.00

db023_w3_2_s12: Which Child's Family Helped

	Freq.	%
0 No	2,069	100.00
Total	2,069	100.00

db023_w3_2_s13: Which Child's Family Helped

	Freq.	%
0 No	2,069	100.00
Total	2,069	100.00

db023_w3_2_s14: Which Child's Family Helped

	Freq.	%
0 No	2,069	100.00
Total	2,069	100.00

db023_w3_2_s15: Which Child's Family Helped

	Freq.	%
0 No	2,069	100.00
Total	2,069	100.00

db023_w3_2_s16: Which Child's Family Helped

	Freq.	%
0 No	2,069	100.00
Total	2,069	100.00

db023_w3_2_s17: Which Child's Family Helped

	Freq.	%
0 No	2,069	100.00
Total	2,069	100.00

db023_w3_2_s18: Which Child's Family Helped

	Freq.	%
0 No	2,069	100.00
Total	2,069	100.00

db023_w3_2_s19: Which Child's Family Helped

	Freq.	%
0 No	2,069	100.00
Total	2,069	100.00

db023_w3_2_s20: Which Child's Family Helped

	Freq.	%
0 No	2,069	100.00
Total	2,069	100.00

db023_w3_2_s21: Which Child's Family Helped

	Freq.	%
0 No	2,069	100.00
Total	2,069	100.00

db023_w3_2_s22: Which Child's Family Helped

	Freq.	%
0 No	2,069	100.00
Total	2,069	100.00

db023_w3_2_s23: Which Child's Family Helped

	Freq.	%
0 No	2,069	100.00
Total	2,069	100.00

db023_w3_2_s24: Which Child's Family Helped

	Freq.	%
0 No	2,069	100.00
Total	2,069	100.00

db023_w3_2_s25: Which Child's Family Helped

	Freq.	%
0 No	2,069	100.00
Total	2,069	100.00

db023_w3_2_s26: Which Child's Family Helped

	Freq.	%
0 No	2,069	100.00
Total	2,069	100.00

db023_w3_3_1__s1: Who in Child[1]'s Family Helped

	Freq.	%
0 No	234	18.07
1 Himself/herself	1,061	81.93
Total	1,295	100.00

db023_w3_3_1__s2: Who in Child[1]'s Family Helped

	Freq.	%
0 No	623	48.11
2 His/her Spouse	672	51.89
Total	1,295	100.00

db023_w3_3_1__s3: Who in Child[1]'s Family Helped

	Freq.	%
0 No	842	65.02
3 His/her Children	453	34.98
Total	1,295	100.00

db023_w3_3_2__s1: Who in Child[2]'s Family Helped

	Freq.	%
0 No	116	13.02
1 Himself/herself	775	86.98
Total	891	100.00

db023_w3_3_2__s2: Who in Child[2]'s Family Helped

	Freq.	%
0 No	435	48.82
2 His/her Spouse	456	51.18
Total	891	100.00

db023_w3_3_2__s3: Who in Child[2]'s Family Helped

	Freq.	%
0 No	631	70.82
3 His/her Children	260	29.18
Total	891	100.00

db023_w3_3_3__s1: Who in Child[3]'s Family Helped

	Freq.	%
0 No	55	9.91
1 Himself/herself	500	90.09
Total	555	100.00

db023_w3_3_3__s2: Who in Child[3]'s Family Helped

	Freq.	%
0 No	250	45.05
2 His/her Spouse	305	54.95
Total	555	100.00

db023_w3_3_3__s3: Who in Child[3]'s Family Helped

	Freq.	%
0 No	388	69.91
3 His/her Children	167	30.09
Total	555	100.00

db023_w3_3_4__s1: Who in Child[4]'s Family Helped

	Freq.	%
0 No	47	13.70
1 Himself/herself	296	86.30
Total	343	100.00

db023_w3_3_4__s2: Who in Child[4]'s Family Helped

	Freq.	%
0 No	159	46.36
2 His/her Spouse	184	53.64
Total	343	100.00

db023_w3_3_4__s3: Who in Child[4]'s Family Helped

	Freq.	%
0 No	235	68.51
3 His/her Children	108	31.49
Total	343	100.00

db023_w3_3_5__s1: Who in Child[5]'s Family Helped

	Freq.	%
0 No	25	14.45
1 Himself/herself	148	85.55
Total	173	100.00

db023_w3_3_5__s2: Who in Child[5]'s Family Helped

	Freq.	%
0 No	84	48.55
2 His/her Spouse	89	51.45
Total	173	100.00

db023_w3_3_5__s3: Who in Child[5]'s Family Helped

	Freq.	%
0 No	124	71.68
3 His/her Children	49	28.32
Total	173	100.00

db023_w3_3_6__s1: Who in Child[6]'s Family Helped

	Freq.	%
0 No	5	7.25
1 Himself/herself	64	92.75
Total	69	100.00

db023_w3_3_6__s2: Who in Child[6]'s Family Helped

	Freq.	%
0 No	28	40.58
2 His/her Spouse	41	59.42
Total	69	100.00

db023_w3_3_6__s3: Who in Child[6]'s Family Helped

	Freq.	%
0 No	49	71.01
3 His/her Children	20	28.99
Total	69	100.00

db023_w3_3_7__s1: Who in Child[7]'s Family Helped

	Freq.	%
0 No	4	11.11
1 Himself/herself	32	88.89
Total	36	100.00

db023_w3_3_7__s2: Who in Child[7]'s Family Helped

	Freq.	%
0 No	16	44.44
2 His/her Spouse	20	55.56
Total	36	100.00

db023_w3_3_7__s3: Who in Child[7]'s Family Helped

	Freq.	%
0 No	26	72.22
3 His/her Children	10	27.78
Total	36	100.00

db023_w3_3_8__s1: Who in Child[8]'s Family Helped

	Freq.	%
0 No	2	14.29
1 Himself/herself	12	85.71
Total	14	100.00

db023_w3_3_8__s2: Who in Child[8]'s Family Helped

	Freq.	%
0 No	4	28.57
2 His/her Spouse	10	71.43
Total	14	100.00

db023_w3_3_8__s3: Who in Child[8]'s Family Helped

	Freq.	%
0 No	11	78.57
3 His/her Children	3	21.43
Total	14	100.00

db023_w3_3_9__s1: Who in Child[9]'s Family Helped

	Freq.	%
1 Himself/herself	6	100.00
Total	6	100.00

db023_w3_3_9__s2: Who in Child[9]'s Family Helped

	Freq.	%
0 No	2	33.33
2 His/her Spouse	4	66.67
Total	6	100.00

db023_w3_3_9__s3: Who in Child[9]'s Family Helped

	Freq.	%
0 No	6	100.00
Total	6	100.00

db023_w3_3_10__s1: Who in Child[10]'s Family Helped

	Freq.	%
1 Himself/herself	1	100.00
Total	1	100.00

db023_w3_3_10__s2: Who in Child[10]'s Family Helped

	Freq.	%
2 His/her Spouse	1	100.00
Total	1	100.00

db023_w3_3_10__s3: Who in Child[10]'s Family Helped

	Freq.	%
0 No	1	100.00
Total	1	100.00

db023_w3_3_11__s1: Who in Child[11]'s Family Helped

	Freq.	%
1 Himself/herself	3	100.00
Total	3	100.00

db023_w3_3_11__s2: Who in Child[11]'s Family Helped

	Freq.	%
0 No	1	33.33
2 His/her Spouse	2	66.67
Total	3	100.00

db023_w3_3_11__s3: Who in Child[11]'s Family Helped

	Freq.	%
0 No	3	100.00
Total	3	100.00

db023_w3_3_1_1_: Number of Children Helped

Mean	SD	Min	Max	Obs
1.67	0.91	0.00	13.00	470

db023_w3_3_1_2_: Number of Children Helped

Mean	SD	Min	Max	Obs
1.53	0.70	0.00	4.00	274

db023_w3_3_1_3_: Number of Children Helped

Mean	SD	Min	Max	Obs
1.57	0.68	1.00	4.00	172

db023_w3_3_1_4_: Number of Children Helped

Mean	SD	Min	Max	Obs
1.65	0.74	1.00	4.00	109

db023_w3_3_1_5_: Number of Children Helped

Mean	SD	Min	Max	Obs
1.73	0.72	1.00	4.00	51

db023_w3_3_1_6_: Number of Children Helped

Mean	SD	Min	Max	Obs
1.71	0.78	1.00	4.00	21

db023_w3_3_1_7_: Number of Children Helped

Mean	SD	Min	Max	Obs
1.40	0.52	1.00	2.00	10

db023_w3_3_1_8_: Number of Children Helped

Mean	SD	Min	Max	Obs
1.75	0.50	1.00	2.00	4

db023_w3_4_s1: Which Sibling's Family Helps

	Freq.	%
0 No	87	71.31
1 Preload XSibName[1]	35	28.69
Total	122	100.00

db023_w3_4_s2: Which Sibling's Family Helps

	Freq.	%
0 No	82	67.21
2 Preload XSibName[2]	40	32.79
Total	122	100.00

db023_w3_4_s3: Which Sibling's Family Helps

	Freq.	%
0 No	88	72.13
3 Preload XSibName[3]	34	27.87
Total	122	100.00

db023_w3_4_s4: Which Sibling's Family Helps

	Freq.	%
0 No	100	81.97
4 Preload XSibName[4]	22	18.03
Total	122	100.00

db023_w3_4_s5: Which Sibling's Family Helps

	Freq.	%
0 No	108	88.52
5 Preload XSibName[5]	14	11.48
Total	122	100.00

db023_w3_4_s6: Which Sibling's Family Helps

	Freq.	%
0 No	111	90.98
6 Preload XSibName[6]	11	9.02
Total	122	100.00

db023_w3_4_s7: Which Sibling's Family Helps

	Freq.	%
0 No	116	95.08
7 Preload XSibName[7]	6	4.92
Total	122	100.00

db023_w3_4_s8: Which Sibling's Family Helps

	Freq.	%
0 No	119	97.54
8 Preload XSibName[8]	3	2.46
Total	122	100.00

db023_w3_4_s9: Which Sibling's Family Helps

	Freq.	%
0 No	121	99.18
9 Preload XSibName[9]	1	0.82
Total	122	100.00

db023_w3_4_s10: Which Sibling's Family Helps

	Freq.	%
0 No	122	100.00
Total	122	100.00

db023_w3_4_s11: Which Sibling's Family Helps

	Freq.	%
0 No	122	100.00
Total	122	100.00

db023_w3_4_s12: Which Sibling's Family Helps

	Freq.	%
0 No	122	100.00
Total	122	100.00

db023_w3_4_s13: Which Sibling's Family Helps

	Freq.	%
0 No	122	100.00
Total	122	100.00

db023_w3_4_s14: Which Sibling's Family Helps

	Freq.	%
0 No	122	100.00
Total	122	100.00

db023_w3_4_s15: Which Sibling's Family Helps

	Freq.	%
0 No	122	100.00
Total	122	100.00

db023_w3_4_s16: Which Sibling's Family Helps

	Freq.	%
0 No	103	84.43
16 Preload XSibName[16]	19	15.57
Total	122	100.00

db023_w3_4_s17: Which Sibling's Family Helps

	Freq.	%
0 No	101	82.79
17 Preload XSibName[17]	21	17.21
Total	122	100.00

db023_w3_4_s18: Which Sibling's Family Helps

	Freq.	%
0 No	106	86.89
18 Preload XSibName[18]	16	13.11

Total	122	100.00

db023_w3_4_s19: Which Sibling's Family Helps

	Freq.	%
0 No	106	86.89
19 Preload XSibName[19]	16	13.11
Total	122	100.00

db023_w3_4_s20: Which Sibling's Family Helps

	Freq.	%
0 No	113	92.62
20 Preload XSibName[20]	9	7.38
Total	122	100.00

db023_w3_4_s21: Which Sibling's Family Helps

	Freq.	%
0 No	119	97.54
21 Preload XSibName[21]	3	2.46
Total	122	100.00

db023_w3_4_s22: Which Sibling's Family Helps

	Freq.	%
0 No	118	96.72
22 Preload XSibName[22]	4	3.28
Total	122	100.00

db023_w3_4_s23: Which Sibling's Family Helps

	Freq.	%
0 No	121	99.18
23 Preload XSibName[23]	1	0.82
Total	122	100.00

db023_w3_4_s24: Which Sibling's Family Helps

	Freq.	%
0 No	122	100.00
Total	122	100.00

db023_w3_4_s25: Which Sibling's Family Helps

	Freq.	%
0 No	122	100.00
Total	122	100.00

db023_w3_4_s26: Which Sibling's Family Helps

	Freq.	%
0 No	122	100.00
Total	122	100.00

db023_w3_4_s27: Which Sibling's Family Helps

	Freq.	%
0 No	122	100.00
Total	122	100.00

db023_w3_4_s28: Which Sibling's Family Helps

	Freq.	%
0 No	122	100.00
Total	122	100.00

db023_w3_4_s29: Which Sibling's Family Helps

	Freq.	%
0 No	122	100.00
Total	122	100.00

db023_w3_4_s30: Which Sibling's Family Helps

	Freq.	%
0 No	122	100.00
Total	122	100.00

db023_w3_4_s99: Which Sibling's Family Helps

	Freq.	%
0 No	101	82.79
99 Preload XSibName[99]	21	17.21
Total	122	100.00

db023_w3_5_1__s1: Who Help in Person from Sibling[1]'s Family

	Freq.	%
0 No	9	25.71
1 Himself/herself	26	74.29
Total	35	100.00

db023_w3_5_1__s2: Who Help in Person from Sibling[1]'s Family

	Freq.	%
0 No	19	54.29

	Freq.	%
2 His/her Spouse	16	45.71
Total	35	100.00

db023_w3_5_1__s3: Who Help in Person from Sibling[1]'s Family

	Freq.	%
0 No	16	45.71
3 Their Children	19	54.29
Total	35	100.00

db023_w3_5_2__s1: Who Help in Person from Sibling[2]'s Family

	Freq.	%
0 No	4	10.00
1 Himself/herself	36	90.00
Total	40	100.00

db023_w3_5_2__s2: Who Help in Person from Sibling[2]'s Family

	Freq.	%
0 No	21	52.50
2 His/her Spouse	19	47.50
Total	40	100.00

db023_w3_5_2__s3: Who Help in Person from Sibling[2]'s Family

	Freq.	%
0 No	20	50.00
3 Their Children	20	50.00
Total	40	100.00

db023_w3_5_3__s1: Who Help in Person from Sibling[3]'s Family

	Freq.	%
0 No	5	14.71
1 Himself/herself	29	85.29
Total	34	100.00

db023_w3_5_3__s2: Who Help in Person from Sibling[3]'s Family

	Freq.	%
0 No	18	52.94
2 His/her Spouse	16	47.06
Total	34	100.00

db023_w3_5_3__s3: Who Help in Person from Sibling[3]'s Family

	Freq.	%

	Freq.	%
0 No	21	61.76
3 Their Children	13	38.24
Total	34	100.00

db023_w3_5_4__s1: Who Help in Person from Sibling[4]'s Family

	Freq.	%
0 No	2	9.09
1 Himself/herself	20	90.91
Total	22	100.00

db023_w3_5_4__s2: Who Help in Person from Sibling[4]'s Family

	Freq.	%
0 No	11	50.00
2 His/her Spouse	11	50.00
Total	22	100.00

db023_w3_5_4__s3: Who Help in Person from Sibling[4]'s Family

	Freq.	%
0 No	13	59.09
3 Their Children	9	40.91
Total	22	100.00

db023_w3_5_5__s1: Who Help in Person from Sibling[5]'s Family

	Freq.	%
0 No	2	14.29
1 Himself/herself	12	85.71
Total	14	100.00

db023_w3_5_5__s2: Who Help in Person from Sibling[5]'s Family

	Freq.	%
0 No	8	57.14
2 His/her Spouse	6	42.86
Total	14	100.00

db023_w3_5_5__s3: Who Help in Person from Sibling[5]'s Family

	Freq.	%
0 No	9	64.29
3 Their Children	5	35.71
Total	14	100.00

db023_w3_5_6__s1: Who Help in Person from Sibling[6]'s Family

	Freq.	%
1 Himself/herself	11	100.00
Total	11	100.00

db023_w3_5_6__s2: Who Help in Person from Sibling[6]'s Family

	Freq.	%
0 No	6	54.55
2 His/her Spouse	5	45.45
Total	11	100.00

db023_w3_5_6__s3: Who Help in Person from Sibling[6]'s Family

	Freq.	%
0 No	8	72.73
3 Their Children	3	27.27
Total	11	100.00

db023_w3_5_7__s1: Who Help in Person from Sibling[7]'s Family

	Freq.	%
1 Himself/herself	6	100.00
Total	6	100.00

db023_w3_5_7__s2: Who Help in Person from Sibling[7]'s Family

	Freq.	%
0 No	4	66.67
2 His/her Spouse	2	33.33
Total	6	100.00

db023_w3_5_7__s3: Who Help in Person from Sibling[7]'s Family

	Freq.	%
0 No	5	83.33
3 Their Children	1	16.67
Total	6	100.00

db023_w3_5_8__s1: Who Help in Person from Sibling[8]'s Family

	Freq.	%
0 No	19,749	99.98
1 Himself/herself	3	0.02
Total	19,752	100.00

db023_w3_5_8__s2: Who Help in Person from Sibling[8]'s Family

	Freq.	%
0 No	19,752	100.00
Total	19,752	100.00

db023_w3_5_8__s3: Who Help in Person from Sibling[8]'s Family

	Freq.	%
0 No	19,752	100.00
Total	19,752	100.00

db023_w3_5_9__s1: Who Help in Person from Sibling[9]'s Family

	Freq.	%
1 Himself/herself	1	100.00
Total	1	100.00

db023_w3_5_9__s2: Who Help in Person from Sibling[9]'s Family

	Freq.	%
2 His/her Spouse	1	100.00
Total	1	100.00

db023_w3_5_9__s3: Who Help in Person from Sibling[9]'s Family

	Freq.	%
0 No	1	100.00
Total	1	100.00

db023_w3_5_16__s1: Who Help in Person from Sibling[16]'s Family

	Freq.	%
0 No	2	10.53
1 Himself/herself	17	89.47
Total	19	100.00

db023_w3_5_16__s2: Who Help in Person from Sibling[16]'s Family

	Freq.	%
0 No	8	42.11
2 His/her Spouse	11	57.89
Total	19	100.00

db023_w3_5_16__s3: Who Help in Person from Sibling[16]'s Family

	Freq.	%
0 No	9	47.37
3 Their Children	10	52.63

Total	19	100.00

db023_w3_5_17__s1: Who Help in Person from Sibling[17]'s Family

	Freq.	%
0 No	2	9.52
1 Himself/herself	19	90.48
Total	21	100.00

db023_w3_5_17__s2: Who Help in Person from Sibling[17]'s Family

	Freq.	%
0 No	8	38.10
2 His/her Spouse	13	61.90
Total	21	100.00

db023_w3_5_17__s3: Who Help in Person from Sibling[17]'s Family

	Freq.	%
0 No	12	57.14
3 Their Children	9	42.86
Total	21	100.00

db023_w3_5_18__s1: Who Help in Person from Sibling[18]'s Family

	Freq.	%
0 No	2	12.50
1 Himself/herself	14	87.50
Total	16	100.00

db023_w3_5_18__s2: Who Help in Person from Sibling[18]'s Family

	Freq.	%
0 No	8	50.00
2 His/her Spouse	8	50.00
Total	16	100.00

db023_w3_5_18__s3: Who Help in Person from Sibling[18]'s Family

	Freq.	%
0 No	9	56.25
3 Their Children	7	43.75
Total	16	100.00

db023_w3_5_19__s1: Who Help in Person from Sibling[19]'s Family

	Freq.	%
0 No	1	6.25

	Freq.	%
1 Himself/herself	15	93.75
Total	16	100.00

db023_w3_5_19__s2: Who Help in Person from Sibling[19]'s Family

	Freq.	%
0 No	6	37.50
2 His/her Spouse	10	62.50
Total	16	100.00

db023_w3_5_19__s3: Who Help in Person from Sibling[19]'s Family

	Freq.	%
0 No	9	56.25
3 Their Children	7	43.75
Total	16	100.00

db023_w3_5_20__s1: Who Help in Person from Sibling[20]'s Family

	Freq.	%
1 Himself/herself	9	100.00
Total	9	100.00

db023_w3_5_20__s2: Who Help in Person from Sibling[20]'s Family

	Freq.	%
0 No	3	33.33
2 His/her Spouse	6	66.67
Total	9	100.00

db023_w3_5_20__s3: Who Help in Person from Sibling[20]'s Family

	Freq.	%
0 No	3	33.33
3 Their Children	6	66.67
Total	9	100.00

db023_w3_5_21__s1: Who Help in Person from Sibling[21]'s Family

	Freq.	%
1 Himself/herself	3	100.00
Total	3	100.00

db023_w3_5_21__s2: Who Help in Person from Sibling[21]'s Family

	Freq.	%
2 His/her Spouse	3	100.00
Total	3	100.00

db023_w3_5_21__s3: Who Help in Person from Sibling[21]'s Family

	Freq.	%
3 Their Children	3	100.00
Total	3	100.00

db023_w3_5_22__s1: Who Help in Person from Sibling[22]'s Family

	Freq.	%
1 Himself/herself	4	100.00
Total	4	100.00

db023_w3_5_22__s2: Who Help in Person from Sibling[22]'s Family

	Freq.	%
0 No	1	25.00
2 His/her Spouse	3	75.00
Total	4	100.00

db023_w3_5_22__s3: Who Help in Person from Sibling[22]'s Family

	Freq.	%
0 No	2	50.00
3 Their Children	2	50.00
Total	4	100.00

db023_w3_5_23__s1: Who Help in Person from Sibling[23]'s Family

	Freq.	%
0 No	19,751	99.99
1 Himself/herself	1	0.01
Total	19,752	100.00

db023_w3_5_23__s2: Who Help in Person from Sibling[23]'s Family

	Freq.	%
0 No	19,752	100.00
Total	19,752	100.00

db023_w3_5_23__s3: Who Help in Person from Sibling[23]'s Family

	Freq.	%
0 No	19,752	100.00
Total	19,752	100.00

db023_w3_5_1_1_: Number of Children Help

Mean	SD	Min	Max	Obs
2.47	1.02	1.00	4.00	19

db023_w3_5_1_2_: Number of Children Help

Mean	SD	Min	Max	Obs
2.25	1.21	1.00	4.00	20

db023_w3_5_1_3_: Number of Children Help

Mean	SD	Min	Max	Obs
2.38	1.04	1.00	4.00	13

db023_w3_5_1_4_: Number of Children Help

Mean	SD	Min	Max	Obs
2.33	1.00	1.00	4.00	9

db023_w3_5_1_5_: Number of Children Help

Mean	SD	Min	Max	Obs
1.20	0.45	1.00	2.00	5

db023_w3_5_1_6_: Number of Children Help

Mean	SD	Min	Max	Obs
2.67	0.58	2.00	3.00	3

db023_w3_5_1_7_: Number of Children Help

Mean	SD	Min	Max	Obs
1.00	.	1.00	1.00	1

db023_w3_5_1_16_: Number of Children Help

Mean	SD	Min	Max	Obs
2.10	1.29	1.00	5.00	10

db023_w3_5_1_17_: Number of Children Help

Mean	SD	Min	Max	Obs
2.44	0.73	1.00	3.00	9

db023_w3_5_1_18_: Number of Children Help

Mean	SD	Min	Max	Obs
2.43	0.53	2.00	3.00	7

db023_w3_5_1_19_: Number of Children Help

Mean	SD	Min	Max	Obs
2.00	0.82	1.00	3.00	7

db023_w3_5_1_20_: Number of Children Help

Mean	SD	Min	Max	Obs
1.67	0.52	1.00	2.00	6

db023_w3_5_1_21_: Number of Children Help

Mean	SD	Min	Max	Obs
1.67	0.58	1.00	2.00	3

db023_w3_5_1_22_: Number of Children Help

Mean	SD	Min	Max	Obs
1.00	0.00	1.00	1.00	2

db023_w3_6: Number of Relatives Help in Person

Mean	SD	Min	Max	Obs
2.32	1.94	1.00	10.00	44

db023_w3_7: Number of Others Who Help in Person

Mean	SD	Min	Max	Obs
2.47	2.20	1.00	15.00	214

db023_w3_9_s1: Most Important 7 Helpers

	Freq.	%
0 No	22	13.02
1 Helper[1]	147	86.98
Total	169	100.00

db023_w3_9_s2: Most Important 7 Helpers

	Freq.	%
0 No	37	21.89
2 Helper[2]	132	78.11

	Freq.	%
Total	169	100.00

db023_w3_9_s3: Most Important 7 Helpers

	Freq.	%
0 No	53	31.36
3 Helper[3]	116	68.64
Total	169	100.00

db023_w3_9_s4: Most Important 7 Helpers

	Freq.	%
0 No	41	24.26
4 Helper[4]	128	75.74
Total	169	100.00

db023_w3_9_s5: Most Important 7 Helpers

	Freq.	%
0 No	47	27.81
5 Helper[5]	122	72.19
Total	169	100.00

db023_w3_9_s6: Most Important 7 Helpers

	Freq.	%
0 No	58	34.32
6 Helper[6]	111	65.68
Total	169	100.00

db023_w3_9_s7: Most Important 7 Helpers

	Freq.	%
0 No	57	33.73
7 Helper[7]	112	66.27
Total	169	100.00

db023_w3_9_s8: Most Important 7 Helpers

	Freq.	%
0 No	63	37.28
8 Helper[8]	106	62.72
Total	169	100.00

db023_w3_9_s9: Most Important 7 Helpers

	Freq.	%
0 No	107	63.31
9 Helper[9]	62	36.69

Total	169	100.00

db023_w3_9_s10: Most Important 7 Helpers

	Freq.	%
0 No	126	74.56
10 Helper[10]	43	25.44
Total	169	100.00

db023_w3_9_s11: Most Important 7 Helpers

	Freq.	%
0 No	138	81.66
11 Helper[11]	31	18.34
Total	169	100.00

db023_w3_9_s12: Most Important 7 Helpers

	Freq.	%
0 No	152	89.94
12 Helper[12]	17	10.06
Total	169	100.00

db023_w3_9_s13: Most Important 7 Helpers

	Freq.	%
0 No	147	86.98
13 Helper[13]	22	13.02
Total	169	100.00

db023_w3_9_s14: Most Important 7 Helpers

	Freq.	%
0 No	158	93.49
14 Helper[14]	11	6.51
Total	169	100.00

db023_w3_9_s15: Most Important 7 Helpers

	Freq.	%
0 No	163	96.45
15 Helper[15]	6	3.55
Total	169	100.00

db023_w3_9_s16: Most Important 7 Helpers

	Freq.	%
0 No	164	97.04
16 Helper[16]	5	2.96

Total	169	100.00

db023_w3_9_s17: Most Important 7 Helpers

	Freq.	%
0 No	167	98.82
17 Helper[17]	2	1.18
Total	169	100.00

db023_w3_9_s18: Most Important 7 Helpers

	Freq.	%
0 No	166	98.22
18 Helper[18]	3	1.78
Total	169	100.00

db023_w3_9_s19: Most Important 7 Helpers

	Freq.	%
0 No	168	99.41
19 Helper[19]	1	0.59
Total	169	100.00

db023_w3_9_s20: Most Important 7 Helpers

	Freq.	%
0 No	168	99.41
20 Helper[20]	1	0.59
Total	169	100.00

db023_w3_9_s21: Most Important 7 Helpers

	Freq.	%
0 No	168	99.41
21 Helper[21]	1	0.59
Total	169	100.00

db023_w3_9_s22: Most Important 7 Helpers

	Freq.	%
0 No	168	99.41
22 Helper[22]	1	0.59
Total	169	100.00

db023_w3_9_s23: Most Important 7 Helpers

	Freq.	%
0 No	168	99.41
23 Helper[23]	1	0.59

Total	169	100.00

db023_w3_9_s24: Most Important 7 Helpers

	Freq.	%
0 No	169	100.00
Total	169	100.00

db023_w3_9_s25: Most Important 7 Helpers

	Freq.	%
0 No	169	100.00
Total	169	100.00

db023_w3_9_s26: Most Important 7 Helpers

	Freq.	%
0 No	169	100.00
Total	169	100.00

db023_w3_9_s27: Most Important 7 Helpers

	Freq.	%
0 No	169	100.00
Total	169	100.00

db023_w3_9_s28: Most Important 7 Helpers

	Freq.	%
0 No	168	99.41
28 Helper[28]	1	0.59
Total	169	100.00

db023_w3_9_s29: Most Important 7 Helpers

	Freq.	%
0 No	169	100.00
Total	169	100.00

db023_w3_9_s30: Most Important 7 Helpers

	Freq.	%
0 No	169	100.00
Total	169	100.00

db023_w3_9_s31: Most Important 7 Helpers

	Freq.	%
0 No	169	100.00
Total	169	100.00

db023_w3_9_s32: Most Important 7 Helpers

	Freq.	%
0 No	169	100.00
Total	169	100.00

db023_w3_9_s33: Most Important 7 Helpers

	Freq.	%
0 No	169	100.00
Total	169	100.00

db023_w3_9_s34: Most Important 7 Helpers

	Freq.	%
0 No	168	99.41
34 Helper[34]	1	0.59
Total	169	100.00

db023_1_: Days Helper[1] Helped

Mean	SD	Min	Max	Obs
20.89	12.49	0.00	31.00	3,917

db023_2_: Days Helper[2] Helped

Mean	SD	Min	Max	Obs
14.80	13.10	0.00	31.00	1,540

db023_3_: Days Helper[3] Helped

Mean	SD	Min	Max	Obs
11.89	12.64	0.00	31.00	925

db023_4_: Days Helper[4] Helped

Mean	SD	Min	Max	Obs
10.40	12.10	0.00	31.00	555

db023_5_: Days Helper[5] Helped

Mean	SD	Min	Max	Obs

8.89	11.19	0.00	31.00	362

db023_6_: Days Helper[6] Helped

Mean	SD	Min	Max	Obs
7.71	10.41	0.00	31.00	248

db023_7_: Days Helper[7] Helped

Mean	SD	Min	Max	Obs
6.38	9.14	0.00	31.00	173

db023_8_: Days Helper[8] Helped

Mean	SD	Min	Max	Obs
9.34	11.16	0.00	31.00	106

db023_9_: Days Helper[9] Helped

Mean	SD	Min	Max	Obs
6.71	9.75	0.00	31.00	62

db023_10_: Days Helper[10] Helped

Mean	SD	Min	Max	Obs
6.84	9.33	0.00	30.00	43

db023_11_: Days Helper[11] Helped

Mean	SD	Min	Max	Obs
6.52	9.82	0.00	30.00	31

db023_12_: Days Helper[12] Helped

Mean	SD	Min	Max	Obs
6.53	10.03	0.00	30.00	17

db023_13_: Days Helper[13] Helped

Mean	SD	Min	Max	Obs
3.59	4.57	0.00	15.00	22

db023_14_: Days Helper[14] Helped

Mean	SD	Min	Max	Obs
5.09	6.77	0.00	15.00	11

db023_15_: Days Helper[15] Helped

Mean	SD	Min	Max	Obs
4.00	5.59	0.00	15.00	6

db023_16_: Days Helper[16] Helped

Mean	SD	Min	Max	Obs
2.80	2.59	0.00	5.00	5

db023_17_: Days Helper[17] Helped

Mean	SD	Min	Max	Obs
3.50	0.71	3.00	4.00	2

db023_18_: Days Helper[18] Helped

Mean	SD	Min	Max	Obs
10.67	16.77	0.00	30.00	3

db023_19_: Days Helper[19] Helped

Mean	SD	Min	Max	Obs
0.00	.	0.00	0.00	1

db023_20_: Days Helper[20] Helped

Mean	SD	Min	Max	Obs
0.00	.	0.00	0.00	1

db023_21_: Days Helper[21] Helped

Mean	SD	Min	Max	Obs
0.00	.	0.00	0.00	1

db023_22_: Days Helper[22] Helped

Mean	SD	Min	Max	Obs
10.00	.	10.00	10.00	1

db023_23_: Days Helper[23] Helped

Mean	SD	Min	Max	Obs
0.00	.	0.00	0.00	1

db023_24_: Days Helper[24] Helped

No Observations

db023_25_: Days Helper[25] Helped

No Observations

db023_26_: Days Helper[26] Helped

No Observations

db023_27_: Days Helper[27] Helped

No Observations

db023_28_: Days Helper[28] Helped

Mean	SD	Min	Max	Obs
10.00	.	10.00	10.00	1

db023_29_: Days Helper[29] Helped

No Observations

db023_30_: Days Helper[30] Helped

No Observations

db023_31_: Days Helper[31] Helped

No Observations

db023_32_: Days Helper[32] Helped

No Observations

db023_33_: Days Helper[33] Helped

No Observations

db023_34_: Days Helper[34] Helped

Mean	SD	Min	Max	Obs
10.00	.	10.00	10.00	1

db024_1_: Hours Helper[1] Helped

Mean	SD	Min	Max	Obs
4.60	6.60	1.00	24.00	3,917

db024_2_: Hours Helper[2] Helped

Mean	SD	Min	Max	Obs
3.63	5.25	1.00	24.00	1,540

db024_3_: Hours Helper[3] Helped

Mean	SD	Min	Max	Obs
3.46	5.02	1.00	24.00	925

db024_4_: Hours Helper[4] Helped

Mean	SD	Min	Max	Obs
3.51	5.18	1.00	24.00	555

db024_5_: Hours Helper[5] Helped

Mean	SD	Min	Max	Obs
3.90	5.65	1.00	24.00	362

db024_6_: Hours Helper[6] Helped

Mean	SD	Min	Max	Obs
3.30	4.90	1.00	24.00	248

db024_7_: Hours Helper[7] Helped

Mean	SD	Min	Max	Obs
3.62	5.35	1.00	24.00	173

db024_8_: Hours Helper[8] Helped

Mean	SD	Min	Max	Obs
4.25	6.67	1.00	24.00	106

db024_9_: Hours Helper[9] Helped

Mean	SD	Min	Max	Obs
3.50	5.80	1.00	24.00	62

db024_10_: Hours Helper[10] Helped

Mean	SD	Min	Max	Obs
5.03	7.31	1.00	24.00	43

db024_11_: Hours Helper[11] Helped

Mean	SD	Min	Max	Obs
3.60	5.81	1.00	24.00	31

db024_12_: Hours Helper[12] Helped

Mean	SD	Min	Max	Obs
1.88	1.50	1.00	6.00	17

db024_13_: Hours Helper[13] Helped

Mean	SD	Min	Max	Obs
2.05	2.13	1.00	9.00	22

db024_14_: Hours Helper[14] Helped

Mean	SD	Min	Max	Obs
2.82	3.37	1.00	12.00	11

db024_15_: Hours Helper[15] Helped

Mean	SD	Min	Max	Obs
1.67	1.21	1.00	4.00	6

db024_16_: Hours Helper[16] Helped

Mean	SD	Min	Max	Obs
3.30	2.28	1.50	7.00	5

db024_17_: Hours Helper[17] Helped

Mean	SD	Min	Max	Obs
5.00	4.24	2.00	8.00	2

db024_18_: Hours Helper[18] Helped

Mean	SD	Min	Max	Obs
2.33	1.53	1.00	4.00	3

db024_19_: Hours Helper[19] Helped

Mean	SD	Min	Max	Obs
2.50	.	2.50	2.50	1

db024_20_: Hours Helper[20] Helped

Mean	SD	Min	Max	Obs
1.00	.	1.00	1.00	1

db024_21_: Hours Helper[21] Helped

Mean	SD	Min	Max	Obs
1.00	.	1.00	1.00	1

db024_22_: Hours Helper[22] Helped

Mean	SD	Min	Max	Obs
2.00	.	2.00	2.00	1

db024_23_: Hours Helper[23] Helped

Mean	SD	Min	Max	Obs
1.00	.	1.00	1.00	1

db024_24_: Hours Helper[24] Helped

No Observations

db024_25_: Hours Helper[25] Helped

No Observations

db024_26_: Hours Helper[26] Helped

No Observations

db024_27_: Hours Helper[27] Helped

No Observations

db024_28_: Hours Helper[28] Helped

Mean	SD	Min	Max	Obs
2.00	.	2.00	2.00	1

db024_29_: Hours Helper[29] Helped

No Observations

db024_30_: Hours Helper[30] Helped

No Observations

db024_31_: Hours Helper[31] Helped

No Observations

db024_32_: Hours Helper[32] Helped

No Observations

db024_33_: Hours Helper[33] Helped

No Observations

db024_34_: Hours Helper[34] Helped

Mean	SD	Min	Max	Obs
2.00	.	2.00	2.00	1

db025_1_: Helper[1] Living in Your Home

	Freq.	%
1 Yes	3,333	85.09
2 No	584	14.91
Total	3,917	100.00

db025_2_: Helper[2] Living in Your Home

	Freq.	%
1 Yes	1,005	65.26
2 No	535	34.74
Total	1,540	100.00

db025_3_: Helper[3] Living in Your Home

	Freq.	%
1 Yes	543	58.70
2 No	382	41.30
Total	925	100.00

db025_4_: Helper[4] Living in Your Home

	Freq.	%

1 Yes	286	51.53
2 No	269	48.47
Total	555	100.00

db025_5_: Helper[5] Living in Your Home

	Freq.	%
1 Yes	160	44.20
2 No	202	55.80
Total	362	100.00

db025_6_: Helper[6] Living in Your Home

	Freq.	%
1 Yes	99	39.92
2 No	149	60.08
Total	248	100.00

db025_7_: Helper[7] Living in Your Home

	Freq.	%
1 Yes	61	35.26
2 No	112	64.74
Total	173	100.00

db025_8_: Helper[8] Living in Your Home

	Freq.	%
1 Yes	42	39.62
2 No	64	60.38
Total	106	100.00

db025_9_: Helper[9] Living in Your Home

	Freq.	%
1 Yes	22	35.48
2 No	40	64.52
Total	62	100.00

db025_10_: Helper[10] Living in Your Home

	Freq.	%
1 Yes	17	39.53
2 No	26	60.47
Total	43	100.00

db025_11_: Helper[11] Living in Your Home

	Freq.	%

	Freq.	%
1 Yes	13	41.94
2 No	18	58.06
Total	31	100.00

db025_12_: Helper[12] Living in Your Home

	Freq.	%
1 Yes	3	17.65
2 No	14	82.35
Total	17	100.00

db025_13_: Helper[13] Living in Your Home

	Freq.	%
1 Yes	1	4.55
2 No	21	95.45
Total	22	100.00

db025_14_: Helper[14] Living in Your Home

	Freq.	%
1 Yes	3	27.27
2 No	8	72.73
Total	11	100.00

db025_15_: Helper[15] Living in Your Home

	Freq.	%
1 Yes	1	16.67
2 No	5	83.33
Total	6	100.00

db025_16_: Helper[16] Living in Your Home

	Freq.	%
1 Yes	1	20.00
2 No	4	80.00
Total	5	100.00

db025_17_: Helper[17] Living in Your Home

	Freq.	%
2 No	2	100.00
Total	2	100.00

db025_18_: Helper[18] Living in Your Home

	Freq.	%
2 No	3	100.00

Total	3	100.00

db025_19_: Helper[19] Living in Your Home

	Freq.	%
2 No	1	100.00
Total	1	100.00

db025_20_: Helper[20] Living in Your Home

	Freq.	%
2 No	1	100.00
Total	1	100.00

db025_21_: Helper[21] Living in Your Home

	Freq.	%
2 No	1	100.00
Total	1	100.00

db025_22_: Helper[22] Living in Your Home

	Freq.	%
2 No	1	100.00
Total	1	100.00

db025_23_: Helper[23] Living in Your Home

	Freq.	%
2 No	1	100.00
Total	1	100.00

db025_24_: Helper[24] Living in Your Home

No Observations

db025_25_: Helper[25] Living in Your Home

No Observations

db025_26_: Helper[26] Living in Your Home

No Observations

db025_27_: Helper[27] Living in Your Home

No Observations

db025_28_: Helper[28] Living in Your Home

	Freq.	%
2 No	1	100.00
Total	1	100.00

db025_29_: Helper[29] Living in Your Home

No Observations

db025_30_: Helper[30] Living in Your Home

No Observations

db025_31_: Helper[31] Living in Your Home

No Observations

db025_32_: Helper[32] Living in Your Home

No Observations

db025_33_: Helper[33] Living in Your Home

No Observations

db025_34_: Helper[34] Living in Your Home

	Freq.	%
2 No	1	100.00
Total	1	100.00

db029_s1: Use the Following Auxiliary

	Freq.	%
0 No	18,476	93.69
1 Walking Stick	1,245	6.31
Total	19,721	100.00

db029_s2: Use the Following Auxiliary

	Freq.	%
0 No	19,651	99.65
2 Travel Device	70	0.35
Total	19,721	100.00

db029_s3: Use the Following Auxiliary

	Freq.	%
0 No	19,483	98.79
3 Manual Wheelchair	238	1.21
Total	19,721	100.00

db029_s4: Use the Following Auxiliary

	Freq.	%
0 No	19,697	99.88
4 Electric Wheelchair	24	0.12
Total	19,721	100.00

db029_s5: Use the Following Auxiliary

	Freq.	%
0 No	19,659	99.69
5 Catheter, Urine Collection Bag	62	0.31
Total	19,721	100.00

db029_s6: Use the Following Auxiliary

	Freq.	%
0 No	19,359	98.16
6 Toilet Series	362	1.84
Total	19,721	100.00

db029_s7: Use the Following Auxiliary

	Freq.	%
0 No	1,581	8.02
7 None of the Above	18,140	91.98
Total	19,721	100.00

db030: Any Possible Helper in the Future

	Freq.	%
1 Yes	14,238	72.20
2 No	5,483	27.80
Total	19,721	100.00

db031_s1: Relationship to You

	Freq.	%
0 No	7,024	49.33
1 Spouse	7,214	50.67
Total	14,238	100.00

db031_s2: Relationship to You

	Freq.	%
0 No	14,169	99.52
2 Father, Mother, Father-in-Law, Mother-in-Law	69	0.48
Total	14,238	100.00

db031_s3: Relationship to You

	Freq.	%
0 No	2,532	17.78
3 Children, Children's Spouse, Grandson, Granddaughter	11,706	82.22
Total	14,238	100.00

db031_s4: Relationship to You

	Freq.	%
0 No	13,649	95.86
4 Sibling, Brother-in-Law, Sister-in-Law, Sibling of Spouse, Etc	589	4.14
Total	14,238	100.00

db031_s5: Relationship to You

	Freq.	%
0 No	14,148	99.37
5 Other Relative	90	0.63
Total	14,238	100.00

db031_s6: Relationship to You

	Freq.	%
0 No	13,972	98.13
6 Paid Helper (Such as Nanny)	266	1.87
Total	14,238	100.00

db031_s7: Relationship to You

	Freq.	%
0 No	14,170	99.52
7 Volunteer or Employee of Facility	68	0.48
Total	14,238	100.00

db031_s8: Relationship to You

	Freq.	%
0 No	13,954	98.01
8 Nursing Home	284	1.99
Total	14,238	100.00

db031_s9: Relationship to You

	Freq.	%
0 No	14,158	99.44
9 Help from the Community	80	0.56
Total	14,238	100.00

db031_s10: Relationship to You

	Freq.	%
0 No	14,180	99.59
10 Other, Please Specify	58	0.41
Total	14,238	100.00

db031_w3_1_s1: Who Will Help You in Future

	Freq.	%
0 No	31	44.93
1 Father	38	55.07
Total	69	100.00

db031_w3_1_s2: Who Will Help You in Future

	Freq.	%
0 No	10	14.49
2 Mother	59	85.51
Total	69	100.00

db031_w3_1_s3: Who Will Help You in Future

	Freq.	%
0 No	49	71.01
3 Father-in-Law	20	28.99
Total	69	100.00

db031_w3_1_s4: Who Will Help You in Future

	Freq.	%
0 No	43	62.32
4 Mother-in-Law	26	37.68
Total	69	100.00

db031_w3_2_s1: Which Child's Family Will Help You in Future

	Freq.	%
0 No	1,470	12.56
1 Preload XChildName[1]	10,236	87.44
Total	11,706	100.00

db031_w3_2_s2: Which Child's Family Will Help You in Future

	Freq.	%
0 No	3,704	31.64
2 Preload XChildName[2]	8,002	68.36
Total	11,706	100.00

db031_w3_2_s3: Which Child's Family Will Help You in Future

	Freq.	%
0 No	7,684	65.64
3 Preload XChildName[3]	4,022	34.36
Total	11,706	100.00

db031_w3_2_s4: Which Child's Family Will Help You in Future

	Freq.	%
0 No	9,835	84.02
4 Preload XChildName[4]	1,871	15.98
Total	11,706	100.00

db031_w3_2_s5: Which Child's Family Will Help You in Future

	Freq.	%
0 No	10,876	92.91
5 Preload XChildName[5]	830	7.09
Total	11,706	100.00

db031_w3_2_s6: Which Child's Family Will Help You in Future

	Freq.	%
0 No	11,362	97.06
6 Preload XChildName[6]	344	2.94
Total	11,706	100.00

db031_w3_2_s7: Which Child's Family Will Help You in Future

	Freq.	%
0 No	11,566	98.80
7 Preload XChildName[7]	140	1.20
Total	11,706	100.00

db031_w3_2_s8: Which Child's Family Will Help You in Future

	Freq.	%
0 No	11,645	99.48
8 Preload XChildName[8]	61	0.52
Total	11,706	100.00

db031_w3_2_s9: Which Child's Family Will Help You in Future

	Freq.	%
0 No	11,682	99.79
9 Preload XChildName[9]	24	0.21
Total	11,706	100.00

db031_w3_2_s10: Which Child's Family Will Help You in Future

	Freq.	%
0 No	11,698	99.93
10 Preload XChildName[10]	8	0.07
Total	11,706	100.00

db031_w3_2_s11: Which Child's Family Will Help You in Future

	Freq.	%
0 No	11,698	99.93
11 Preload XChildName[11]	8	0.07
Total	11,706	100.00

db031_w3_2_s12: Which Child's Family Will Help You in Future

	Freq.	%
0 No	11,705	99.99
12 Preload XChildName[12]	1	0.01
Total	11,706	100.00

db031_w3_2_s13: Which Child's Family Will Help You in Future

	Freq.	%
0 No	11,705	99.99
13 Preload XChildName[13]	1	0.01
Total	11,706	100.00

db031_w3_2_s14: Which Child's Family Will Help You in Future

	Freq.	%
0 No	11,706	100.00
Total	11,706	100.00

db031_w3_2_s15: Which Child's Family Will Help You in Future

	Freq.	%
0 No	11,705	99.99
15 Preload XChildName[15]	1	0.01
Total	11,706	100.00

db031_w3_2_s16: Which Child's Family Will Help You in Future

	Freq.	%
0 No	11,706	100.00
Total	11,706	100.00

db031_w3_2_s17: Which Child's Family Will Help You in Future

	Freq.	%
0 No	11,706	100.00
Total	11,706	100.00

db031_w3_2_s18: Which Child's Family Will Help You in Future

	Freq.	%
0 No	11,706	100.00
Total	11,706	100.00

db031_w3_2_s19: Which Child's Family Will Help You in Future

	Freq.	%
0 No	11,706	100.00
Total	11,706	100.00

db031_w3_2_s20: Which Child's Family Will Help You in Future

	Freq.	%
0 No	11,706	100.00
Total	11,706	100.00

db031_w3_2_s21: Which Child's Family Will Help You in Future

	Freq.	%
0 No	11,706	100.00
Total	11,706	100.00

db031_w3_2_s22: Which Child's Family Will Help You in Future

	Freq.	%
0 No	11,706	100.00
Total	11,706	100.00

db031_w3_2_s23: Which Child's Family Will Help You in Future

	Freq.	%
0 No	11,706	100.00
Total	11,706	100.00

db031_w3_2_s24: Which Child's Family Will Help You in Future

	Freq.	%
0 No	11,706	100.00
Total	11,706	100.00

db031_w3_2_s25: Which Child's Family Will Help You in Future

	Freq.	%
0 No	11,706	100.00
Total	11,706	100.00

db031_w3_2_s26: Which Child's Family Will Help You in Future

	Freq.	%
0 No	11,706	100.00
Total	11,706	100.00

db031_w3_4_s1: Which Sibling's Family Will Help You

	Freq.	%
0 No	254	43.12
1 Preload XSibName[1]	335	56.88
Total	589	100.00

db031_w3_4_s2: Which Sibling's Family Will Help You

	Freq.	%
0 No	263	44.65
2 Preload XSibName[2]	326	55.35
Total	589	100.00

db031_w3_4_s3: Which Sibling's Family Will Help You

	Freq.	%
0 No	309	52.46
3 Preload XSibName[3]	280	47.54
Total	589	100.00

db031_w3_4_s4: Which Sibling's Family Will Help You

	Freq.	%
0 No	393	66.72
4 Preload XSibName[4]	196	33.28
Total	589	100.00

db031_w3_4_s5: Which Sibling's Family Will Help You

	Freq.	%

	Freq.	%
0 No	472	80.14
5 Preload XSibName[5]	117	19.86
Total	589	100.00

db031_w3_4_s6: Which Sibling's Family Will Help You

	Freq.	%
0 No	523	88.79
6 Preload XSibName[6]	66	11.21
Total	589	100.00

db031_w3_4_s7: Which Sibling's Family Will Help You

	Freq.	%
0 No	563	95.59
7 Preload XSibName[7]	26	4.41
Total	589	100.00

db031_w3_4_s8: Which Sibling's Family Will Help You

	Freq.	%
0 No	578	98.13
8 Preload XSibName[8]	11	1.87
Total	589	100.00

db031_w3_4_s9: Which Sibling's Family Will Help You

	Freq.	%
0 No	584	99.15
9 Preload XSibName[9]	5	0.85
Total	589	100.00

db031_w3_4_s10: Which Sibling's Family Will Help You

	Freq.	%
0 No	587	99.66
10 Preload XSibName[10]	2	0.34
Total	589	100.00

db031_w3_4_s11: Which Sibling's Family Will Help You

	Freq.	%
0 No	588	99.83
11 Preload XSibName[11]	1	0.17
Total	589	100.00

db031_w3_4_s12: Which Sibling's Family Will Help You

	Freq.	%

	Freq.	%
0 No	589	100.00
Total	589	100.00

db031_w3_4_s13: Which Sibling's Family Will Help You

	Freq.	%
0 No	589	100.00
Total	589	100.00

db031_w3_4_s14: Which Sibling's Family Will Help You

	Freq.	%
0 No	589	100.00
Total	589	100.00

db031_w3_4_s15: Which Sibling's Family Will Help You

	Freq.	%
0 No	589	100.00
Total	589	100.00

db031_w3_4_s16: Which Sibling's Family Will Help You

	Freq.	%
0 No	286	48.56
16 Preload XSibName[16]	303	51.44
Total	589	100.00

db031_w3_4_s17: Which Sibling's Family Will Help You

	Freq.	%
0 No	299	50.76
17 Preload XSibName[17]	290	49.24
Total	589	100.00

db031_w3_4_s18: Which Sibling's Family Will Help You

	Freq.	%
0 No	346	58.74
18 Preload XSibName[18]	243	41.26
Total	589	100.00

db031_w3_4_s19: Which Sibling's Family Will Help You

	Freq.	%
0 No	434	73.68
19 Preload XSibName[19]	155	26.32
Total	589	100.00

db031_w3_4_s20: Which Sibling's Family Will Help You

	Freq.	%
0 No	480	81.49
20 Preload XSibName[20]	109	18.51
Total	589	100.00

db031_w3_4_s21: Which Sibling's Family Will Help You

	Freq.	%
0 No	527	89.47
21 Preload XSibName[21]	62	10.53
Total	589	100.00

db031_w3_4_s22: Which Sibling's Family Will Help You

	Freq.	%
0 No	562	95.42
22 Preload XSibName[22]	27	4.58
Total	589	100.00

db031_w3_4_s23: Which Sibling's Family Will Help You

	Freq.	%
0 No	577	97.96
23 Preload XSibName[23]	12	2.04
Total	589	100.00

db031_w3_4_s24: Which Sibling's Family Will Help You

	Freq.	%
0 No	586	99.49
24 Preload XSibName[24]	3	0.51
Total	589	100.00

db031_w3_4_s25: Which Sibling's Family Will Help You

	Freq.	%
0 No	587	99.66
25 Preload XSibName[25]	2	0.34
Total	589	100.00

db031_w3_4_s26: Which Sibling's Family Will Help You

	Freq.	%
0 No	589	100.00
Total	589	100.00

db031_w3_4_s27: Which Sibling's Family Will Help You

	Freq.	%
0 No	589	100.00
Total	589	100.00

db031_w3_4_s28: Which Sibling's Family Will Help You

	Freq.	%
0 No	589	100.00
Total	589	100.00

db031_w3_4_s29: Which Sibling's Family Will Help You

	Freq.	%
0 No	589	100.00
Total	589	100.00

db031_w3_4_s30: Which Sibling's Family Will Help You

	Freq.	%
0 No	589	100.00
Total	589	100.00

db031_w3_4_s99: Which Sibling's Family Will Help You

	Freq.	%
0 No	555	94.23
99 Preload XSibName[99]	34	5.77
Total	589	100.00

db031_w3_6: Number of other Relatives Who Will Help

Mean	SD	Min	Max	Obs
4.74	3.85	1.00	15.00	90

db031_w3_7: Number of Others Who will Help

Mean	SD	Min	Max	Obs
3.14	2.71	1.00	10.00	58

db036_w4: Can't Work Because of Disability or Health

	Freq.	%
1 I Can't Work at All	2,704	13.71
2 I Can't Work for Long Time	4,440	22.51
3 I Have No Problem Working	12,577	63.77
Total	19,721	100.00

db037_w4: Can't Do Household Work Because of Disability or Health

	Freq.	%
1 I Can't Do at All	178	1.05
2 I Can't Do for Long Time	2,915	17.13
3 I Have No Problem	13,924	81.82
Total	17,017	100.00

db032: How often the Respondent Receive Assistance

	Freq.	%
1 Never	16,201	82.15
2 A Few Times	2,105	10.67
3 Most or All of the Time	453	2.30
4 The Section Was Completed by a Proxy Respondent	962	4.88
Total	19,721	100.00

db033: Relationship to R

	Freq.	%
1 Spouse	536	55.78
2 Mother	9	0.94
3 Father	3	0.31
4 Mother-in-Law	6	0.62
5 Father-in-Law	6	0.62
6 Sibling	20	2.08
7 Brother-in-Law, sister-in-Law	13	1.35
8 Child	214	22.27
9 Spouse of Child	61	6.35
10 Grandchild	39	4.06
11 Other Relative	30	3.12
12 Helper or Other Non-Relative	24	2.50
Total	961	100.00

db034: Main Reason for Proxy

	Freq.	%
1 The Respondent Has Serious Physical Handicaps	332	34.55
2 The Respondent Has Serious Mental Handicaps	121	12.59
3 The Respondent Has Rejected this Interview	241	25.08
4 Other	267	27.78
Total	961	100.00

xrtype: Respondent Type

	Freq.	%
1 RE Interview	19,443	98.44
2 New Interview	309	1.56
Total	19,752	100.00

xrgender: Generated Gender

	Freq.	%
1 Male	9,308	47.12
2 Female	10,444	52.88
Total	19,752	100.00

zdisability_1_: Had Disability[1] Listed in DA005 at ZIWTime

	Freq.	%
1 Yes	1,452	100.00
Total	1,452	100.00

zdisability_2_: Had Disability[2] Listed in DA005 at ZIWTime

	Freq.	%
1 Yes	1,185	100.00
Total	1,185	100.00

zdisability_3_: Had Disability[3] Listed in DA005 at ZIWTime

	Freq.	%
1 Yes	2,068	100.00
Total	2,068	100.00

zdisability_4_: Had Disability[4] Listed in DA005 at ZIWTime

	Freq.	%
1 Yes	2,957	100.00
Total	2,957	100.00

zdisability_5_: Had Disability[5] Listed in DA005 at ZIWTime

	Freq.	%
1 Yes	194	100.00
Total	194	100.00

ziwtime: R's LAST IW Time

A String Variable	
Obs:	19,443

zda006_1_: Reported Disabled Time at ZIWTime

	Freq.	%
1 Yes	1,413	100.00
Total	1,413	100.00

zda006_2_: Reported Disabled Time at ZIWTime

	Freq.	%
1 Yes	1,125	100.00
Total	1,125	100.00

zda006_3_: Reported Disabled Time at ZIWTime

	Freq.	%
1 Yes	2,005	100.00
Total	2,005	100.00

zda006_4_: Reported Disabled Time at ZIWTime

	Freq.	%
1 Yes	2,850	100.00
Total	2,850	100.00

zda006_5_: Reported Disabled Time at ZIWTime

	Freq.	%
1 Yes	179	100.00
Total	179	100.00

zdiagnosed_1_: Had Doctor Diagnosed Disease[1] at ZIWTime

	Freq.	%
1 Yes	5,240	100.00
Total	5,240	100.00

zdiagnosed_2_: Had Doctor Diagnosed Disease[2] at ZIWTime

	Freq.	%
1 Yes	2,434	100.00
Total	2,434	100.00

zdiagnosed_3_: Had Doctor Diagnosed Disease[3] at ZIWTime

	Freq.	%
1 Yes	1,485	100.00
Total	1,485	100.00

zdiagnosed_4_: Had Doctor Diagnosed Disease[4] at ZIWTime

	Freq.	%
1 Yes	190	100.00
Total	190	100.00

zdiagnosed_5_: Had Doctor Diagnosed Disease[5] at ZIWTime

	Freq.	%
1 Yes	1,806	100.00
Total	1,806	100.00

zdiagnosed_6_: Had Doctor Diagnosed Disease[6] at ZIWTime

	Freq.	%
1 Yes	739	100.00
Total	739	100.00

zdiagnosed_7_: Had Doctor Diagnosed Disease[7] at ZIWTime

	Freq.	%
1 Yes	2,528	100.00
Total	2,528	100.00

zdiagnosed_8_: Had Doctor Diagnosed Disease[8] at ZIWTime

	Freq.	%
1 Yes	451	100.00
Total	451	100.00

zdiagnosed_9_: Had Doctor Diagnosed Disease[9] at ZIWTime

	Freq.	%
1 Yes	1,203	100.00
Total	1,203	100.00

zdiagnosed_10_: Had Doctor Diagnosed Disease[10] at ZIWTime

	Freq.	%
1 Yes	4,400	100.00
Total	4,400	100.00

zdiagnosed_11_: Had Doctor Diagnosed Disease[11] at ZIWTime

	Freq.	%
1 Yes	268	100.00
Total	268	100.00

zdiagnosed_12_: Had Doctor Diagnosed Disease[12] at ZIWTime

	Freq.	%
1 Yes	349	100.00
Total	349	100.00

zdiagnosed_13_: Had Doctor Diagnosed Disease[13] at ZIWTime

	Freq.	%
1 Yes	6,170	100.00
Total	6,170	100.00

zdiagnosed_14_: Had Doctor Diagnosed Disease[14] at ZIWTime

	Freq.	%
1 Yes	759	100.00
Total	759	100.00

zdisease_1_: Had Disease[1] at ZIWTime

	Freq.	%
1 Yes	5,555	100.00
Total	5,555	100.00

zdisease_2_: Had Disease[2] at ZIWTime

	Freq.	%
1 Yes	2,434	100.00
Total	2,434	100.00

zdisease_3_: Had Disease[3] at ZIWTime

	Freq.	%
1 Yes	1,485	100.00
Total	1,485	100.00

zdisease_4_: Had Disease[4] at ZIWTime

	Freq.	%
1 Yes	190	100.00
Total	190	100.00

zdisease_5_: Had Disease[5] at ZIWTime

	Freq.	%
1 Yes	2,155	100.00
Total	2,155	100.00

zdisease_6_: Had Disease[6] at ZIWTime

	Freq.	%
1 Yes	739	100.00
Total	739	100.00

zdisease_7_: Had Disease[7] at ZIWTime

	Freq.	%
1 Yes	2,528	100.00
Total	2,528	100.00

zdisease_8_: Had Disease[8] at ZIWTime

	Freq.	%
1 Yes	451	100.00
Total	451	100.00

zdisease_9_: Had Disease[9] at ZIWTime

	Freq.	%
1 Yes	1,203	100.00
Total	1,203	100.00

zdisease_10_: Had Disease[10] at ZIWTime

	Freq.	%
1 Yes	4,400	100.00
Total	4,400	100.00

zdisease_11_: Had Disease[11] at ZIWTime

	Freq.	%
1 Yes	433	100.00
Total	433	100.00

zdisease_12_: Had Disease[12] at ZIWTime

	Freq.	%
1 Yes	349	100.00
Total	349	100.00

zdisease_13_: Had Disease[13] at ZIWTime

	Freq.	%
1 Yes	6,170	100.00
Total	6,170	100.00

zdisease_14_: Had Disease[14] at ZIWTime

	Freq.	%
1 Yes	759	100.00
Total	759	100.00

zda008_1_: Known Having Disease[1] at ZIWTime

	Freq.	%
1 Yes	315	100.00
Total	315	100.00

zda008_5_: Known Having Disease[5] at ZIWTime

	Freq.	%
1 Yes	349	100.00
Total	349	100.00

zda008_11_: Known Having Disease[11] at ZIWTime

	Freq.	%
1 Yes	165	100.00
Total	165	100.00

zda009_1_: Answered When was the Condition[1] First Diagnosed or Known

	Freq.	%
1 Yes	6,017	100.00
Total	6,017	100.00

zda009_2_: Answered When was the Condition[2] First Diagnosed or Known

	Freq.	%
1 Yes	3,009	100.00
Total	3,009	100.00

zda009_3_: Answered When was the Condition[3] First Diagnosed or Known

	Freq.	%
1 Yes	1,783	100.00
Total	1,783	100.00

zda009_4_: Answered When was the Condition[4] First Diagnosed or Known

	Freq.	%
1 Yes	292	100.00
Total	292	100.00

zda009_5_: Answered When was the Condition[5] First Diagnosed or Known

	Freq.	%
1 Yes	2,717	100.00
Total	2,717	100.00

zda009_6_: Answered When was the Condition[6] First Diagnosed or Known

	Freq.	%
1 Yes	1,146	100.00
Total	1,146	100.00

zda009_7_: Answered When was the Condition[7] First Diagnosed or Known

	Freq.	%
1 Yes	3,016	100.00
Total	3,016	100.00

zda009_8_: Answered When was the Condition[8] First Diagnosed or Known

	Freq.	%
1 Yes	624	100.00
Total	624	100.00

zda009_9_: Answered When was the Condition[9] First Diagnosed or Known

	Freq.	%
1 Yes	1,732	100.00
Total	1,732	100.00

zda009_10_: Answered When was the Condition[10] First Diagnosed or Known

	Freq.	%
1 Yes	5,418	100.00
Total	5,418	100.00

zda009_11_: Answered When was the Condition[11] First Diagnosed or Known

	Freq.	%
1 Yes	566	100.00
Total	566	100.00

zda009_12_: Answered When was the Condition[12] First Diagnosed or Known

	Freq.	%
1 Yes	497	100.00
Total	497	100.00

zda009_13_: Answered When was the Condition[13] First Diagnosed or Known

	Freq.	%
1 Yes	7,386	100.00
Total	7,386	100.00

zda009_14_: Answered When was the Condition[14] First Diagnosed or Known

	Freq.	%
1 Yes	918	100.00
Total	918	100.00

zmenopause: Started Menopause at Last Interview

	Freq.	%
1 Yes	6,683	100.00
Total	6,683	100.00

zda030: Answered When First Diagnosed

	Freq.	%
1 Yes	1,109	100.00
Total	1,109	100.00

zcataract: Had Cataract Surgery at ZIWTime

	Freq.	%
1 Yes	504	100.00
Total	504	100.00

zcataractnum: Had Cataract Surgery for One or Two Eyes at ZIWTime

	Freq.	%
1 One Eye	238	57.49
2 Two Eyes	176	42.51
Total	414	100.00

zglaucoma: Had Glaucoma at ZIWTime

	Freq.	%
1 Yes	380	100.00
Total	380	100.00

ztooth: Lost All Teeth at ZIWTime

	Freq.	%
1 Yes	2,283	100.00
Total	2,283	100.00

zsmoke: Ever Smoked at ZIWTime

	Freq.	%
1 Yes	7,384	100.00
Total	7,384	100.00

zda065: Answered Age Started to Smoke on a Regular Basis

	Freq.	%
1 Yes	7,567	100.00
Total	7,567	100.00

zda069: The Frequency of Ever Drank Alcoholic Beverages

	Freq.	%
1 Never	10,435	77.15
2 Less than Once a Month	1,512	11.18
3 More than Once a Month	1,579	11.67
Total	13,526	100.00

zda070: Answered When Quit or Reduced Drinking

	Freq.	%
1 Yes	1,501	100.00
Total	1,501	100.00

zda071: Answered When Start Drinking

	Freq.	%
1 Yes	6,090	100.00
Total	6,090	100.00

versionID: Version ID

A String Variable	
Obs:	19,752

MODULE DC

Cognition

ID: Individual ID

A String Variable	
Obs:	19,744

householdID: Household ID

A String Variable	
Obs:	19,744

communityID: Community ID

A String Variable	
Obs:	19,744

dc001_w4: Checking Year

	Freq.	%
1 Correct	13,459	74.11
5 Error	3,620	19.93
97 Not Assessed	1,081	5.95
Total	18,160	100.00

dc002_w4: Checking Season

	Freq.	%
1 Correct	13,123	72.27
5 Error	4,090	22.52
97 Not Assessed	945	5.20
Total	18,158	100.00

dc003_w4: Checking Date

	Freq.	%
1 Correct	11,485	63.25
5 Error	5,488	30.22
97 Not Assessed	1,185	6.53
Total	18,158	100.00

dc005_w4: Checking Day

	Freq.	%
1 Correct	10,986	60.50
5 Error	5,689	31.33
97 Not Assessed	1,483	8.17
Total	18,158	100.00

dc006_w4: Checking Month

	Freq.	%
1 Correct	16,047	88.37
5 Error	1,784	9.82
97 Not Assessed	327	1.80
Total	18,158	100.00

dc007_w4: Checking State

	Freq.	%
1 Correct	8,115	81.24
5 Error	1,351	13.52
97 Not Assessed	523	5.24
Total	9,989	100.00

dc008_w4: Checking County

	Freq.	%
1 Correct	8,484	84.93
5 Error	1,130	11.31
97 Not Assessed	375	3.75
Total	9,989	100.00

dc009_w4: Checking City

	Freq.	%
1 Correct	9,159	91.69
5 Error	626	6.27
97 Not Assessed	204	2.04
Total	9,989	100.00

dc010_w4: Checking Floor

	Freq.	%

	Freq.	%
1 Correct	8,496	85.05
5 Error	904	9.05
97 Not Assessed	589	5.90
Total	9,989	100.00

dc012_w4: Checking Address

	Freq.	%
1 Correct	9,147	91.57
5 Error	537	5.38
97 Not Assessed	305	3.05
Total	9,989	100.00

dc004: Self-Rated Memory

	Freq.	%
1 Excellent	126	0.69
2 Very Good	893	4.92
3 Good	1,419	7.82
4 Fair	9,893	54.49
5 Poor	5,826	32.09
Total	18,157	100.00

dc013_w4_1_s1: Repeated Time 1: Ball

	Freq.	%
0 No	2,089	20.91
1 Ball	7,900	79.09
Total	9,989	100.00

dc013_w4_1_s2: Repeated Time 1: Flag

	Freq.	%
0 No	3,298	33.02
2 Flag	6,691	66.98
Total	9,989	100.00

dc013_w4_1_s3: Repeated Time 1: Tree

	Freq.	%
0 No	2,894	28.97
3 Tree	7,095	71.03
Total	9,989	100.00

dc013_w4_1_s4: Repeated Time 1: None

	Freq.	%
0 No	9,351	93.61
4 None	638	6.39
Total	9,989	100.00

dc013_w4_1_s97: Repeated Time 1: Not Assessed

	Freq.	%
0 No	9,293	93.03
97 Not Assessed	696	6.97
Total	9,989	100.00

dc013_w4_2_s1: Repeated Time 2: Ball

	Freq.	%
0 No	1,204	26.44
1 Ball	3,350	73.56
Total	4,554	100.00

dc013_w4_2_s2: Repeated Time 2: Flag

	Freq.	%
0 No	1,600	35.13
2 Flag	2,954	64.87
Total	4,554	100.00

dc013_w4_2_s3: Repeated Time 2: Tree

	Freq.	%
0 No	1,427	31.34
3 Tree	3,127	68.66
Total	4,554	100.00

dc013_w4_2_s4: Repeated Time 2: None

	Freq.	%
0 No	4,259	93.52
4 None	295	6.48
Total	4,554	100.00

dc013_w4_2_s97: Repeated Time 2: Not Assessed

	Freq.	%
0 No	4,011	88.08
97 Not Assessed	543	11.92
Total	4,554	100.00

dc013_w4_3_s1: Repeated Time 3: Ball

	Freq.	%
0 No	916	42.66
1 Ball	1,231	57.34
Total	2,147	100.00

dc013_w4_3_s2: Repeated Time 3: Flag

	Freq.	%
0 No	1,089	50.72
2 Flag	1,058	49.28
Total	2,147	100.00

dc013_w4_3_s3: Repeated Time 3: Tree

	Freq.	%
0 No	1,003	46.72
3 Tree	1,144	53.28
Total	2,147	100.00

dc013_w4_3_s4: Repeated Time 3: None

	Freq.	%
0 No	1,922	89.52
4 None	225	10.48
Total	2,147	100.00

dc013_w4_3_s97: Repeated Time 3: Not Assessed

	Freq.	%
0 No	1,654	77.04
97 Not Assessed	493	22.96
Total	2,147	100.00

dc013_w4_4_s1: Repeated Time 4: Ball

	Freq.	%
0 No	779	56.70
1 Ball	595	43.30
Total	1,374	100.00

dc013_w4_4_s2: Repeated Time 4: Flag

	Freq.	%
0 No	894	65.07
2 Flag	480	34.93
Total	1,374	100.00

dc013_w4_4_s3: Repeated Time 4: Tree

	Freq.	%
0 No	827	60.19
3 Tree	547	39.81
Total	1,374	100.00

dc013_w4_4_s4: Repeated Time 4: None

	Freq.	%
0 No	1,193	86.83
4 None	181	13.17
Total	1,374	100.00

dc013_w4_4_s97: Repeated Time 4: Not Assessed

	Freq.	%
0 No	882	64.19
97 Not Assessed	492	35.81
Total	1,374	100.00

dc013_w4_5_s1: Repeated Time 5: Ball

	Freq.	%
0 No	732	70.72
1 Ball	303	29.28
Total	1,035	100.00

dc013_w4_5_s2: Repeated Time 5: Flag

	Freq.	%
0 No	790	76.33
2 Flag	245	23.67
Total	1,035	100.00

dc013_w4_5_s3: Repeated Time 5: Tree

	Freq.	%
0 No	750	72.46
3 Tree	285	27.54
Total	1,035	100.00

dc013_w4_5_s4: Repeated Time 5: None

	Freq.	%
0 No	851	82.22
4 None	184	17.78
Total	1,035	100.00

dc013_w4_5_s97: Repeated Time 5: Not Assessed

	Freq.	%
0 No	569	54.98
97 Not Assessed	466	45.02
Total	1,035	100.00

dc014_w4_1: Reason for Missing from 100-7

	Freq.	%
97 Don't Know	2,775	61.08
98 Refused	1,768	38.92
Total	4,543	100.00

dc014_w4_1_1: Specific Result from 100-7

Mean	SD	Min	Max	Obs
717,306.93	80465572.32	1.00	9.39e+09	13,614

dc014_w4_2: Reason for Missing from dc014_w4_1-7

	Freq.	%
97 Don't Know	1,273	80.83
98 Refused	302	19.17
Total	1,575	100.00

dc014_w4_2_1: Specific Result from dc014_w4_2-7

Mean	SD	Min	Max	Obs
85.08	12.25	-4.00	886.00	12,040

dc014_w4_3: Reason for Missing from dc014_w4_2-7

	Freq.	%
97 Don't Know	485	76.98
98 Refused	145	23.02
Total	630	100.00

dc014_w4_3_1: Specific Result from dc014_w4_3-7

Mean	SD	Min	Max	Obs
77.24	12.45	-11.00	979.00	11,411

dc014_w4_4: Reason for Missing from dc014_w4_3-7

	Freq.	%
97 Don't Know	310	78.48
98 Refused	85	21.52
Total	395	100.00

dc014_w4_4_1: Specific Result from dc014_w4_4-7

Mean	SD	Min	Max	Obs
69.53	11.84	-26.00	974.00	11,016

dc014_w4_5: Reason for Missing from dc014_w4_4-7

	Freq.	%
97 Don't Know	299	81.25
98 Refused	69	18.75
Total	368	100.00

dc014_w4_5_1: Specific Result from dc014_w4_5-7

Mean	SD	Min	Max	Obs
62.54	8.58	-89.00	180.00	10,648

dc024: Use Pen Paper or Other Instruments for Mathmatics

	Freq.	%
1 Used	723	6.56
2 Not Used	10,293	93.44
Total	11,016	100.00

dc015_w4_s1: Delayed Recall: Ball Flag Tree

	Freq.	%
0 No	2,277	25.35
1 Ball	6,707	74.65
Total	8,984	100.00

dc015_w4_s2: Delayed Recall: Ball Flag Tree

	Freq.	%
0 No	3,254	36.22
2 Flag	5,730	63.78
Total	8,984	100.00

dc015_w4_s3: Delayed Recall: Ball Flag Tree

	Freq.	%
0 No	3,281	36.52
3 Tree	5,703	63.48
Total	8,984	100.00

dc015_w4_s4: Delayed Recall: Ball Flag Tree

	Freq.	%
0 No	8,058	89.69
4 None	926	10.31
Total	8,984	100.00

dc015_w4_s97: Delayed Recall: Ball Flag Tree

	Freq.	%
0 No	8,729	97.16
97 Not Assessed	255	2.84
Total	8,984	100.00

dc016_w4: Watch Correct

	Freq.	%
1 Correct	8,788	87.99
5 Error	1,058	10.59
97 Not Assessed	142	1.42
Total	9,988	100.00

dc017_w4: Pencil Correct

	Freq.	%
1 Correct	8,925	89.36
5 Error	922	9.23
97 Not Assessed	141	1.41
Total	9,988	100.00

dc018_w4: Repeat Correct

	Freq.	%
1 Correct	3,576	35.80
5 Error	4,743	47.49
97 Not Assessed	1,669	16.71
Total	9,988	100.00

dc019_w4: Read Correct

	Freq.	%
1 Correct, Respondent Closed Eyes	5,250	52.77
2 Correct, Respondent Closed Eyes After Been Read out Loudly	2,285	22.97
5 Error	1,619	16.27
97 Not Assessed	795	7.99
Total	9,949	100.00

dc020_w4: Hand Correct

	Freq.	%
1 Correct	6,972	69.80
5 Error	2,534	25.37
97 Not Assessed	482	4.83
Total	9,988	100.00

dc021_w4: Folds Correct

	Freq.	%

	Freq.	%
1 Correct	6,528	65.36
5 Error	2,820	28.23
97 Not Assessed	640	6.41
Total	9,988	100.00

dc022_w4: Leg Correct

	Freq.	%
1 Correct	6,482	64.90
5 Error	2,801	28.04
97 Not Assessed	705	7.06
Total	9,988	100.00

dc023_w4: Sentence Correct

	Freq.	%
1 Correct	3,168	31.72
5 Error	1,783	17.85
97 Not Assessed	5,037	50.43
Total	9,988	100.00

dc024_w4: Draw Correct

	Freq.	%
1 Correct	9,509	52.38
5 Error	6,033	33.23
97 Not Assessed	2,613	14.39
Total	18,155	100.00

dc025_w4: Scissors Correct

	Freq.	%
1 Correct	8,587	85.98
5 Error	478	4.79
8 Don't Know	851	8.52
97 Refused	71	0.71
Total	9,987	100.00

dc026_w4: Cactus Correct

	Freq.	%
1 Correct	1,552	15.54
5 Error	1,951	19.54
8 Don't Know	6,074	60.82
97 Refused	74	0.74
99 Other	336	3.36
Total	9,987	100.00

dc026_w4_revised: Cactus Correct: Revised

	Freq.	%
1 Correct	1,634	16.36
5 Error	2,183	21.86
8 Don't Know	6,096	61.04
97 Refused	74	0.74
Total	9,987	100.00

dc027_w4: President Correct

	Freq.	%
1 Correct	8,312	83.23
5 Error	517	5.18
8 Don't Know	1,107	11.08
97 Refused	51	0.51
Total	9,987	100.00

wr101_intro: Understand and Accept WR Test

	Freq.	%
1 Yes	16,343	90.02
2 No	1,811	9.98
Total	18,154	100.00

dc028_w4_1: Reasons for Rufused Word Recall Test

	Freq.	%
1 Refused	1,185	65.43
3 Dumb at Old Age	9	0.50
4 Deaf	156	8.61
5 Other	461	25.46
Total	1,811	100.00

dc028_w4_s1: Word Recall 1st: Butter

	Freq.	%
0 No	9,898	60.56
1 Butter	6,445	39.44
Total	16,343	100.00

dc028_w4_s2: Word Recall 1st: Arm

	Freq.	%
0 No	11,960	73.18
2 Arm	4,383	26.82
Total	16,343	100.00

dc028_w4_s3: Word Recall 1st: Shore

	Freq.	%

	Freq.	%
0 No	14,582	89.22
3 Shore	1,761	10.78
Total	16,343	100.00

dc028_w4_s4: Word Recall 1st: Letter

	Freq.	%
0 No	15,594	95.42
4 Letter	749	4.58
Total	16,343	100.00

dc028_w4_s5: Word Recall 1st: Queen

	Freq.	%
0 No	14,742	90.20
5 Queen	1,601	9.80
Total	16,343	100.00

dc028_w4_s6: Word Recall 1st: Cabin

	Freq.	%
0 No	11,216	68.63
6 Cabin	5,127	31.37
Total	16,343	100.00

dc028_w4_s7: Word Recall 1st: Pole

	Freq.	%
0 No	9,740	59.60
7 Pole	6,603	40.40
Total	16,343	100.00

dc028_w4_s8: Word Recall 1st: Ticket

	Freq.	%
0 No	11,298	69.13
8 Ticket	5,045	30.87
Total	16,343	100.00

dc028_w4_s9: Word Recall 1st: Grass

	Freq.	%
0 No	7,638	46.74
9 Grass	8,705	53.26
Total	16,343	100.00

dc028_w4_s10: Word Recall 1st: Engine

	Freq.	%

	Freq.	%
0 No	5,121	31.33
10 Engine	11,222	68.67
Total	16,343	100.00

dc028_w4_s11: Word Recall 1st: None

	Freq.	%
0 No	14,466	88.51
11 None	1,877	11.49
Total	16,343	100.00

dc028_w4_s12: Word Recall 1st: Refused to Recall

	Freq.	%
0 No	15,690	96.00
12 Refused to Recall	653	4.00
Total	16,343	100.00

dc029_w4_s1: Word Recall 2nd: Pole

	Freq.	%
0 No	7,503	47.82
1 Pole	8,187	52.18
Total	15,690	100.00

dc029_w4_s2: Word Recall 2nd: Letter

	Freq.	%
0 No	12,882	82.10
2 Letter	2,808	17.90
Total	15,690	100.00

dc029_w4_s3: Word Recall 2nd: Butter

	Freq.	%
0 No	6,345	40.44
3 Butter	9,345	59.56
Total	15,690	100.00

dc029_w4_s4: Word Recall 2nd: Queen

	Freq.	%
0 No	11,216	71.49
4 Queen	4,474	28.51
Total	15,690	100.00

dc029_w4_s5: Word Recall 2nd: Arm

	Freq.	%

	Freq.	%
0 No	7,424	47.32
5 Arm	8,266	52.68
Total	15,690	100.00

dc029_w4_s6: Word Recall 2nd: Shore

	Freq.	%
0 No	10,462	66.68
6 Shore	5,228	33.32
Total	15,690	100.00

dc029_w4_s7: Word Recall 2nd: Grass

	Freq.	%
0 No	6,116	38.98
7 Grass	9,574	61.02
Total	15,690	100.00

dc029_w4_s8: Word Recall 2nd: Cabin

	Freq.	%
0 No	5,969	38.04
8 Cabin	9,721	61.96
Total	15,690	100.00

dc029_w4_s9: Word Recall 2nd: Ticket

	Freq.	%
0 No	8,406	53.58
9 Ticket	7,284	46.42
Total	15,690	100.00

dc029_w4_s10: Word Recall 2nd: Engine

	Freq.	%
0 No	3,261	20.78
10 Engine	12,429	79.22
Total	15,690	100.00

dc029_w4_s11: Word Recall 2nd: None

	Freq.	%
0 No	14,862	94.72
11 None	828	5.28
Total	15,690	100.00

dc029_w4_s12: Word Recall 2nd: Refused to Recall

	Freq.	%

0 No	15,111	96.31
12 Refused to Recall	579	3.69
Total	15,690	100.00

dc030_w4_s1: Word Recall 3rd: Shore

	Freq.	%
0 No	7,986	52.85
1 Shore	7,124	47.15
Total	15,110	100.00

dc030_w4_s2: Word Recall 3rd: Letter

	Freq.	%
0 No	10,596	70.13
2 Letter	4,514	29.87
Total	15,110	100.00

dc030_w4_s3: Word Recall 3rd: Arm

	Freq.	%
0 No	6,152	40.71
3 Arm	8,958	59.29
Total	15,110	100.00

dc030_w4_s4: Word Recall 3rd: Cabin

	Freq.	%
0 No	5,894	39.01
4 Cabin	9,216	60.99
Total	15,110	100.00

dc030_w4_s5: Word Recall 3rd: Pole

	Freq.	%
0 No	5,595	37.03
5 Pole	9,515	62.97
Total	15,110	100.00

dc030_w4_s6: Word Recall 3rd: Ticket

	Freq.	%
0 No	9,631	63.74
6 Ticket	5,479	36.26
Total	15,110	100.00

dc030_w4_s7: Word Recall 3rd: Engine

	Freq.	%

	Freq.	%
0 No	4,656	30.81
7 Engine	10,454	69.19
Total	15,110	100.00

dc030_w4_s8: Word Recall 3rd: Grass

	Freq.	%
0 No	4,544	30.07
8 Grass	10,566	69.93
Total	15,110	100.00

dc030_w4_s9: Word Recall 3rd: Butter

	Freq.	%
0 No	3,587	23.74
9 Butter	11,523	76.26
Total	15,110	100.00

dc030_w4_s10: Word Recall 3rd: Queen

	Freq.	%
0 No	4,042	26.75
10 Queen	11,068	73.25
Total	15,110	100.00

dc030_w4_s11: Word Recall 3rd: None

	Freq.	%
0 No	14,493	95.92
11 None	617	4.08
Total	15,110	100.00

dc030_w4_s12: Word Recall 3rd: Refused to Recall

	Freq.	%
0 No	14,609	96.68
12 Refused to Recall	501	3.32
Total	15,110	100.00

dc031_w4_s1: Whether any of the Following Apply: Administered Verbally

	Freq.	%
0 No	10,205	62.45
1 Administered Verbally	6,137	37.55
Total	16,342	100.00

dc031_w4_s2: Whether any of the Following Apply: Interruption Occured during Administration

	Freq.	%
0 No	15,780	96.56
2 Interruption Occured during Administration	562	3.44
Total	16,342	100.00

dc031_w4_s3: Whether any of the Following Apply: Respondent Had Difficulty Reading the Words

	Freq.	%
0 No	14,149	86.58
3 Respondent Had Difficulty Reading the Words	2,193	13.42
Total	16,342	100.00

dc031_w4_s4: Whether any of the Following Apply: None of the above

	Freq.	%
0 No	7,978	48.82
4 None of the above	8,364	51.18
Total	16,342	100.00

rf101_intro: Understand and Accept Animal Naming Test

	Freq.	%
1 Yes	8,324	83.36
2 No	1,662	16.64
Total	9,986	100.00

dc039_w4_1: Reasons for Rufused Animal Naming

	Freq.	%
1 Refused	899	54.09
3 Dumb at Old Age	13	0.78
4 Deaf	132	7.94
5 Other	618	37.18
Total	1,662	100.00

dc032_w4: Total Number during 0-15 Seconds

Mean	SD	Min	Max	Obs
6.52	6.57	0.00	544.00	8,324

dc033_w4: Correct Number during 0-15 Seconds

Mean	SD	Min	Max	Obs
6.23	2.92	0.00	62.00	8,324

dc034_w4: Total Number during 16-30 Seconds

Mean	SD	Min	Max	Obs
2.94	2.01	0.00	13.00	8,324

dc035_w4: Correct Number during 16-30 Seconds

Mean	SD	Min	Max	Obs
4.99	219.18	0.00	19,999.00	8,324

dc036_w4: Total Number during 31-45 Seconds

Mean	SD	Min	Max	Obs
2.07	1.86	0.00	14.00	8,324

dc037_w4: Correct Number during 31-45 Seconds

Mean	SD	Min	Max	Obs
1.67	1.64	0.00	13.00	8,324

dc038_w4: Total Number during 46-60 Seconds

Mean	SD	Min	Max	Obs
1.60	1.77	0.00	20.00	8,324

dc039_w4: Correct Number during 46-60 Seconds

Mean	SD	Min	Max	Obs
1.28	1.55	0.00	18.00	8,324

dc042_w4: Elbow Correct

	Freq.	%
1 Correct	8,525	85.37
5 Error	1,258	12.60
97 Cannot Answer	203	2.03
Total	9,986	100.00

dc043_w4: Hammer Correct

	Freq.	%
1 Correct	8,807	88.19
5 Error	850	8.51
97 Cannot Answer	329	3.29
Total	9,986	100.00

dc044_w4: Store Correct

	Freq.	%
1 Clearly Indicate the Route	3,246	32.51
2 Address of Market	2,326	23.29
3 Name of Market	1,303	13.05
4 A Vague Answer	2,368	23.71
5 Don't Know	631	6.32
97 Cannot Answer	112	1.12
Total	9,986	100.00

dc045_w4: Point Correct

	Freq.	%
1 Point to the Window and Then the Door	7,241	72.51
2 Ponit to Window Only-No Door Available	57	0.57
3 Point to Door Only-No Window Availabel	383	3.84
4 Not Applicable	421	4.22
5 Incorrect Action	1,433	14.35
97 Cannot Answer	451	4.52
Total	9,986	100.00

dc009: Bothered by Things

	Freq.	%
1 Rarely or None (<1 Day)	8,824	48.61
2 Some or A Little (1-2 Days)	3,696	20.36
3 Occasionally or Moderate Amount of Time (3-4 Days)	2,609	14.37
4 Most of the Time (5-7 Days)	2,474	13.63
8 Don't Know	355	1.96
9 Refused	195	1.07
Total	18,153	100.00

dc010: Had Trouble Keeping Mind

	Freq.	%
1 Rarely or None (<1 Day)	8,743	48.16
2 Some or A Little (1-2 Days)	3,518	19.38
3 Occasionally or Moderate Amount of Time (3-4 Days)	2,705	14.90
4 Most of the Time (5-7 Days)	2,300	12.67
8 Don't Know	671	3.70
9 Refused	216	1.19
Total	18,153	100.00

dc011: Felt Depressed

	Freq.	%
1 Rarely or None (<1 Day)	8,792	48.44
2 Some or A Little (1-2 Days)	3,725	20.52
3 Occasionally or Moderate Amount of Time (3-4 Days)	2,803	15.44
4 Most of the Time (5-7 Days)	2,287	12.60
8 Don't Know	370	2.04
9 Refused	175	0.96
Total	18,152	100.00

dc012: I Felt Everything I Did Was An Effort

	Freq.	%
1 Rarely or None (<1 Day)	9,008	49.63
2 Some or A Little (1-2 Days)	3,097	17.06
3 Occasionally or Moderate Amount of Time (3-4 Days)	2,450	13.50
4 Most of the Time (5-7 Days)	3,031	16.70
8 Don't Know	387	2.13
9 Refused	179	0.99
Total	18,152	100.00

dc013: I Felt Hopeful about the Future

	Freq.	%
1 Rarely or None (<1 Day)	4,997	27.53
2 Some or A Little (1-2 Days)	2,309	12.72
3 Occasionally or Moderate Amount of Time (3-4 Days)	2,778	15.30
4 Most of the Time (5-7 Days)	6,893	37.97
8 Don't Know	950	5.23
9 Refused	225	1.24
Total	18,152	100.00

dc014: I Felt Fearful

	Freq.	%
1 Rarely or None (<1 Day)	13,938	76.78
2 Some or A Little (1-2 Days)	1,761	9.70
3 Occasionally or Moderate Amount of Time (3-4 Days)	1,144	6.30
4 Most of the Time (5-7 Days)	982	5.41
8 Don't Know	175	0.96
9 Refused	152	0.84
Total	18,152	100.00

dc015: My Sleep Was Restless

	Freq.	%
1 Rarely or None (<1 Day)	8,255	45.48
2 Some or A Little (1-2 Days)	2,922	16.10
3 Occasionally or Moderate Amount of Time (3-4 Days)	2,713	14.95
4 Most of the Time (5-7 Days)	3,969	21.87
8 Don't Know	152	0.84
9 Refused	141	0.78
Total	18,152	100.00

dc016: I Was Happy

	Freq.	%
1 Rarely or None (<1 Day)	3,544	19.52
2 Some or A Little (1-2 Days)	2,300	12.67
3 Occasionally or Moderate Amount of Time (3-4 Days)	3,408	18.77
4 Most of the Time (5-7 Days)	8,488	46.76
8 Don't Know	245	1.35
9 Refused	167	0.92

	Freq.	%
Total	18,152	100.00

dc017: I Felt Lonely

	Freq.	%
1 Rarely or None (<1 Day)	12,252	67.50
2 Some or A Little (1-2 Days)	2,111	11.63
3 Occasionally or Moderate Amount of Time (3-4 Days)	1,541	8.49
4 Most of the Time (5-7 Days)	1,814	9.99
8 Don't Know	269	1.48
9 Refused	165	0.91
Total	18,152	100.00

dc018: I Could Not Get on

	Freq.	%
1 Rarely or None (<1 Day)	13,657	75.24
2 Some or A Little (1-2 Days)	1,534	8.45
3 Occasionally or Moderate Amount of Time (3-4 Days)	1,205	6.64
4 Most of the Time (5-7 Days)	1,155	6.36
8 Don't Know	377	2.08
9 Refused	224	1.23
Total	18,152	100.00

dc028: Life Satisfication

	Freq.	%
1 Completely Satisfied	929	5.12
2 Very Satisfied	5,303	29.21
3 Somewhat Satistied	9,801	53.99
4 Not Very Satistied	1,542	8.49
5 Not at All Satisfied	577	3.18
Total	18,152	100.00

dc042_w3: Health Satisfication

	Freq.	%
1 Completely Satisfied	667	3.67
2 Very Satisfied	4,057	22.35
3 Somewhat Satistied	8,446	46.53
4 Not Very Satistied	3,627	19.98
5 Not at All Satisfied	1,355	7.46
Total	18,152	100.00

dc043_w3: Marriage Satisfication

	Freq.	%
1 Completely Satisfied	1,154	6.36
2 Very Satisfied	6,426	35.40
3 Somewhat Satistied	7,205	39.69
4 Not Very Satistied	1,044	5.75
5 Not at All Satisfied	470	2.59

6 No Spouse	1,853	10.21
Total	18,152	100.00

dc044_w3: Chidren Satisfication

	Freq.	%
1 Completely Satisfied	1,628	8.97
2 Very Satisfied	8,871	48.87
3 Somewhat Satistied	6,684	36.82
4 Not Very Satistied	615	3.39
5 Not at All Satisfied	192	1.06
6 No Child	162	0.89
Total	18,152	100.00

dc046_w4: Air Quality Satisfication

	Freq.	%
1 Completely Satisfied	881	4.85
2 Very Satisfied	4,797	26.43
3 Somewhat Satistied	9,424	51.92
4 Not Very Satistied	2,442	13.45
5 Not at All Satisfied	608	3.35
Total	18,152	100.00

dc047_w4_s1: Delayed Recall: Shore

	Freq.	%
0 No	9,534	60.77
1 Shore	6,154	39.23
Total	15,688	100.00

dc047_w4_s2: Delayed Recall: Letter

	Freq.	%
0 No	11,976	76.34
2 Letter	3,712	23.66
Total	15,688	100.00

dc047_w4_s3: Delayed Recall: Arm

	Freq.	%
0 No	7,816	49.82
3 Arm	7,872	50.18
Total	15,688	100.00

dc047_w4_s4: Delayed Recall: Cabin

	Freq.	%
0 No	7,480	47.68
4 Cabin	8,208	52.32

	Freq.	%
Total	15,688	100.00

dc047_w4_s5: Delayed Recall: Pole

	Freq.	%
0 No	7,661	48.83
5 Pole	8,027	51.17
Total	15,688	100.00

dc047_w4_s6: Delayed Recall: Ticket

	Freq.	%
0 No	11,301	72.04
6 Ticket	4,387	27.96
Total	15,688	100.00

dc047_w4_s7: Delayed Recall: Engine

	Freq.	%
0 No	8,391	53.49
7 Engine	7,297	46.51
Total	15,688	100.00

dc047_w4_s8: Delayed Recall: Grass

	Freq.	%
0 No	5,751	36.66
8 Grass	9,937	63.34
Total	15,688	100.00

dc047_w4_s9: Delayed Recall: Butter

	Freq.	%
0 No	7,597	48.43
9 Butter	8,091	51.57
Total	15,688	100.00

dc047_w4_s10: Delayed Recall: Queen

	Freq.	%
0 No	10,276	65.50
10 Queen	5,412	34.50
Total	15,688	100.00

dc047_w4_s11: Delayed Recall: None

	Freq.	%
0 No	13,354	85.12
11 None	2,334	14.88

	Freq.	%
Total	15,688	100.00

dc047_w4_s12: Delayed Recall: Refused to Recall

	Freq.	%
0 No	15,183	96.78
12 Refused to Recall	505	3.22
Total	15,688	100.00

dc029_w3_1: Answer of Example 1

Mean	SD	Min	Max	Obs
6.10	7.89	0.00	888.00	14,579

dc029_w4_1: Reason for No Response of Example 1

	Freq.	%
6 R Cannot Understand	920	25.76
7 R Cannot Read or Write	446	12.49
8 Don't Know	1,600	44.79
9 Refused	606	16.97
Total	3,572	100.00

dc030_w3_1: Answer of Example 2

Mean	SD	Min	Max	Obs
5.11	1.37	0.00	72.00	14,992

dc030_w4_1: Reason for No Response of Example 2

	Freq.	%
6 R Cannot Understand	698	25.74
8 Don't Know	1,415	52.18
9 Refused	599	22.09
Total	2,712	100.00

dc031_w4_0: Understand This Test or Not

	Freq.	%
1 Continue	12,932	73.05
5 R Seems Confused or Cannot Understand	2,836	16.02
8 Don't Know	1,321	7.46
9 Refused	615	3.47
Total	17,704	100.00

dc031_w3_1: Answer of Starting Block 1

Mean	SD	Min	Max	Obs

8.96	0.63	0.00	50.00	12,828

dc031_w4_1: Reason for No Response of Starting Block 1

	Freq.	%
7 R Unable to Do	20	19.23
8 Don't Know	81	77.88
9 Refused	3	2.88
Total	104	100.00

dc031_w3_2: Answer of Starting Block 2

Mean	SD	Min	Max	Obs
9.78	1.33	1.00	34.00	12,343

dc031_w4_2: Reason for No Response of Starting Block 2

	Freq.	%
8 Don't Know	555	97.54
9 Refused	14	2.46
Total	569	100.00

dc031_w3_3: Answer of Starting Block 3

Mean	SD	Min	Max	Obs
4.94	2.61	0.00	100.00	9,570

dc031_w4_3: Reason for No Response of Starting Block 3

	Freq.	%
8 Don't Know	3,300	98.74
9 Refused	42	1.26
Total	3,342	100.00

dc032_w3_1: Answer of 0 Correct Block 1

Mean	SD	Min	Max	Obs
4.42	1.82	0.00	20.00	226

dc032_w4_1: Reason for No Response of 0 Correct Block 1

	Freq.	%
8 Don't Know	35	79.55
9 Refused	9	20.45
Total	44	100.00

dc032_w3_2: Answer of 0 Correct Block 2

Mean	SD	Min	Max	Obs
5.46	4.69	0.00	60.00	217

dc032_w4_2: Reason for No Response of 0 Correct Block 2

	Freq.	%
8 Don't Know	42	79.25
9 Refused	11	20.75
Total	53	100.00

dc032_w3_3: Answer of 0 Correct Block 3

Mean	SD	Min	Max	Obs
11.29	12.12	1.00	125.00	192

dc032_w4_3: Reason for No Response of 0 Correct Block 3

	Freq.	%
8 Don't Know	64	82.05
9 Refused	14	17.95
Total	78	100.00

dc033_w3_1: Answer of 1 Correct Block 1

Mean	SD	Min	Max	Obs
4.42	1.05	0.00	14.00	3,518

dc033_w4_1: Reason for No Response of 1 Correct Block 1

	Freq.	%
8 Don't Know	75	84.27
9 Refused	14	15.73
Total	89	100.00

dc033_w3_2: Answer of 1 Correct Block 2

Mean	SD	Min	Max	Obs
10.45	2.31	0.00	28.00	3,105

dc033_w4_2: Reason for No Response of 1 Correct Block 2

	Freq.	%
8 Don't Know	487	97.01
9 Refused	15	2.99
Total	502	100.00

dc033_w3_3: Answer of 1 Correct Block 3

Mean	SD	Min	Max	Obs
40.77	2,147.41	0.00	123,456.00	3,305

dc033_w4_3: Reason for No Response of 1 Correct Block 3

	Freq.	%
8 Don't Know	282	93.38
9 Refused	20	6.62
Total	302	100.00

dc034_w3_1: Answer of 2 Correct Block 1

Mean	SD	Min	Max	Obs
7.80	2.97	0.00	100.00	4,811

dc034_w4_1: Reason for No Response of 2 Correct Block 1

	Freq.	%
8 Don't Know	1,843	99.30
9 Refused	13	0.70
Total	1,856	100.00

dc034_w3_2: Answer of 2 Correct Block 2

Mean	SD	Min	Max	Obs
5.36	9.72	0.00	700.00	5,262

dc034_w4_2: Reason for No Response of 2 Correct Block 2

	Freq.	%
8 Don't Know	1,379	98.15
9 Refused	26	1.85
Total	1,405	100.00

dc034_w3_3: Answer of 2 Correct Block 3

Mean	SD	Min	Max	Obs
13.76	3.91	0.00	75.00	4,983

dc034_w4_3: Reason for No Response of 2 Correct Block 3

	Freq.	%
8 Don't Know	1,642	97.51
9 Refused	42	2.49
Total	1,684	100.00

dc035_w3_1: Answer of 3 Correct Block 1

Mean	SD	Min	Max	Obs
6.55	1.57	0.00	21.00	2,150

dc035_w4_1: Reason for No Response of 3 Correct Block 1

	Freq.	%
8 Don't Know	217	100.00
Total	217	100.00

dc035_w3_2: Answer of 3 Correct Block 2

Mean	SD	Min	Max	Obs
11.59	2.23	1.00	18.00	2,146

dc035_w4_2: Reason for No Response of 3 Correct Block 2

	Freq.	%
8 Don't Know	218	98.64
9 Refused	3	1.36
Total	221	100.00

dc035_w3_3_1: Answer of 3 Correct Block 3_1

Mean	SD	Min	Max	Obs
7.88	1.63	1.00	38.00	2,169

dc035_w3_3_2: Answer of 3 Correct Block 3_2

Mean	SD	Min	Max	Obs
8.00	1.81	0.00	39.00	2,169

dc035_w4_3: Reason for No Response of 3 Correct Block 3

	Freq.	%
8 Don't Know	195	98.48
9 Refused	3	1.52
Total	198	100.00

wre_intro: Understand and Accept WRE Test

	Freq.	%
1 Yes	7,801	95.39
2 No	377	4.61
Total	8,178	100.00

dc048_w4: Word Recognition: Church

	Freq.	%
1 Yes	1,347	17.27
5 No	6,148	78.81
8 Don't Know	306	3.92
Total	7,801	100.00

dc049_w4: Word Recognition: Coffee

	Freq.	%
1 Yes	850	10.90
5 No	6,707	85.98
8 Don't Know	244	3.13
Total	7,801	100.00

dc050_w4: Word Recognition: Butter

	Freq.	%
1 Yes	7,250	92.94
5 No	457	5.86
8 Don't Know	94	1.20
Total	7,801	100.00

dc051_w4: Word Recognition: Dollar

	Freq.	%
1 Yes	1,042	13.36
5 No	6,579	84.34
8 Don't Know	180	2.31
Total	7,801	100.00

dc052_w4: Word Recognition: Arm

	Freq.	%
1 Yes	7,010	89.86
5 No	660	8.46
8 Don't Know	131	1.68
Total	7,801	100.00

dc053_w4: Word Recognition: Shore

	Freq.	%
1 Yes	6,842	87.71
5 No	823	10.55
8 Don't Know	136	1.74
Total	7,801	100.00

dc054_w4: Word Recognition: Five

	Freq.	%
1 Yes	1,188	15.23
5 No	6,472	82.96
8 Don't Know	141	1.81
Total	7,801	100.00

dc055_w4: Word Recognition: Letter

	Freq.	%
1 Yes	5,808	74.45
5 No	1,778	22.79
8 Don't Know	215	2.76
Total	7,801	100.00

dc056_w4: Word Recognition: Hotel

	Freq.	%
1 Yes	2,483	31.83
5 No	5,124	65.68
8 Don't Know	194	2.49
Total	7,801	100.00

dc057_w4: Word Recognition: Mountain

	Freq.	%
1 Yes	773	9.91
5 No	6,907	88.54
8 Don't Know	121	1.55
Total	7,801	100.00

dc058_w4: Word Recognition: Queen

	Freq.	%
1 Yes	6,738	86.37
5 No	923	11.83
8 Don't Know	140	1.79
Total	7,801	100.00

dc059_w4: Word Recognition: Cabin

	Freq.	%
1 Yes	6,893	88.36
5 No	754	9.67
8 Don't Know	154	1.97
Total	7,801	100.00

dc060_w4: Word Recognition: Slipper

	Freq.	%

1 Yes	696	8.92
5 No	6,951	89.10
8 Don't Know	154	1.97
Total	7,801	100.00

dc061_w4: Word Recognition: Pole

	Freq.	%
1 Yes	6,925	88.77
5 No	765	9.81
8 Don't Know	111	1.42
Total	7,801	100.00

dc062_w4: Word Recognition: Village

	Freq.	%
1 Yes	877	11.24
5 No	6,765	86.72
8 Don't Know	159	2.04
Total	7,801	100.00

dc063_w4: Word Recognition: String

	Freq.	%
1 Yes	650	8.33
5 No	7,016	89.94
8 Don't Know	135	1.73
Total	7,801	100.00

dc064_w4: Word Recognition: Ticket

	Freq.	%
1 Yes	6,208	79.58
5 No	1,451	18.60
8 Don't Know	142	1.82
Total	7,801	100.00

dc065_w4: Word Recognition: Troops

	Freq.	%
1 Yes	617	7.91
5 No	7,030	90.12
8 Don't Know	154	1.97
Total	7,801	100.00

dc066_w4: Word Recognition: Grass

	Freq.	%
1 Yes	6,962	89.24
5 No	723	9.27

	Freq.	%
8 Don't Know	116	1.49
Total	7,801	100.00

dc067_w4: Word Recognition: Engine

	Freq.	%
1 Yes	7,193	92.21
5 No	492	6.31
8 Don't Know	116	1.49
Total	7,801	100.00

dc068_w4_s1: Things Happened during Respondent Interview 1

	Freq.	%
0 No	17,633	97.16
1 R Had Poor Eysight	515	2.84
Total	18,148	100.00

dc068_w4_s2: Things Happened during Respondent Interview 2

	Freq.	%
0 No	17,567	96.80
2 R Had Poor Hearing and Didn't Wear Hearing Aid	581	3.20
Total	18,148	100.00

dc068_w4_s3: Things Happened during Respondent Interview 3

	Freq.	%
0 No	18,117	99.83
3 R Wore Hearing Aid	31	0.17
Total	18,148	100.00

dc068_w4_s4: Things Happened during Respondent Interview 4

	Freq.	%
0 No	17,951	98.91
4 R Had Tremor Hand, Which Interfered Some Tests	197	1.09
Total	18,148	100.00

dc068_w4_s5: Things Happened during Respondent Interview 5

	Freq.	%
0 No	16,898	93.11
5 Interview Was Interfered by Other Affairs or Noisy	1,250	6.89
Total	18,148	100.00

dc068_w4_s6: Things Happened during Respondent Interview 6

	Freq.	%

0 No	17,162	94.57
6 Quality Not Good for Emotion Problems of R	986	5.43
Total	18,148	100.00

dc068_w4_s7: Things Happened during Respondent Interview 7

	Freq.	%
0 No	16,911	93.18
7 Other	1,237	6.82
Total	18,148	100.00

dc068_w4_s8: Things Happened during Respondent Interview 8

	Freq.	%
0 No	3,827	21.09
8 None of the above	14,321	78.91
Total	18,148	100.00

dc069_w4: Laguage Used by Interviweres

	Freq.	%
1 Mandarin	9,511	52.42
2 Local Dialect	8,236	45.39
3 Other Dialect	398	2.19
Total	18,145	100.00

dc070_w4: Using Translator during Interview

	Freq.	%
1 Yes	1,324	7.30
2 No	16,820	92.70
Total	18,144	100.00

versionID: Version ID

A String Variable	
Obs:	19,744

MODULE DD

Insider

ID: Individual ID

A String Variable	
Obs:	11,011

householdID: Household ID

A String Variable	
Obs:	11,011

communityID: Community ID

A String Variable	
Obs:	11,011

intro_infselect: Informant Nominate

	Freq.	%
1 Continue	10,323	93.85
2 Respondent Doesn't Have Any Informant	676	6.15
Total	10,999	100.00

intro_infselect_1: Approval Code for Giving up Respondent Interview

A String Variable	
Obs:	164

inf1_rtr: Relationship of 1st Informant

	Freq.	%
1 Spouse/Partner	5,493	53.43

	Freq.	%
2 Child	1,873	18.22
3 Grandchild	389	3.78
4 Sibling	159	1.55
5 Parent	22	0.21
6 Friend	312	3.03
7 Guardian	6	0.06
8 Neighbor	1,000	9.73
9 Other	1,027	9.99
Total	10,281	100.00

notice1: Have 2nd Informant Nominate

	Freq.	%
1 Yes	554	5.39
2 No	9,726	94.61
Total	10,280	100.00

inf2_rtr: Relationship of 2st Informant

	Freq.	%
1 Spouse/Partner	51	9.21
2 Child	263	47.47
3 Grandchild	39	7.04
4 Sibling	26	4.69
6 Friend	41	7.40
8 Neighbor	44	7.94
9 Other	90	16.25
Total	554	100.00

notice2: Have 3rd Informant Nominate

	Freq.	%
1 Yes	217	39.17
2 No	337	60.83
Total	554	100.00

inf3_rtr: Relationship of 3rd Informant

	Freq.	%
1 Spouse/Partner	13	5.99
2 Child	109	50.23
3 Grandchild	22	10.14
4 Sibling	11	5.07
6 Friend	10	4.61
8 Neighbor	15	6.91
9 Other	37	17.05
Total	217	100.00

informant_selection: Informant Selection

	Freq.	%
1 The 1st Informant	10,068	97.96

	Freq.	%
2 The 2nd Informant	156	1.52
3 The 3rd Informant	30	0.29
97 Other Informant	24	0.23
Total	10,278	100.00

inf1_noiwreason: Why Didn't Choose the 1st Informant

	Freq.	%
1 Refused	16	8.60
2 No Contact	76	40.86
3 Health Reasons	21	11.29
4 Not Knowledgeable about R	7	3.76
5 Other Reason	66	35.48
Total	186	100.00

inf2_noiwreason: Why Didn't Choose the 2nd Informant

	Freq.	%
1 Refused	4	13.33
2 No Contact	16	53.33
3 Health Reasons	2	6.67
4 Not Knowledgeable about R	2	6.67
5 Other Reason	6	20.00
Total	30	100.00

infother_reason: Why Choose the Other Informant

	Freq.	%
1 Other Reason	12	50.00
2 None of the Norminated Informant Worked out	12	50.00
Total	24	100.00

dd001_w4: Age of Informant

Mean	SD	Min	Max	Obs
58.17	14.95	18.00	96.00	10,163

dd002_w4: Gender of Informant

	Freq.	%
1 Man	4,863	47.85
2 Woman	5,299	52.15
Total	10,162	100.00

dd003_w4: Education of Informant

	Freq.	%
1 No Formal Education (Illiterate)	1,952	19.21
2 Capable of Reading/Writing	1,432	14.09
3 Sichu/Home School	9	0.09

	Freq.	%
4 Elementary School	2,206	21.71
5 Middle School	2,502	24.62
6 High School	945	9.30
7 Vocational School	466	4.59
8 Two/Three Year College/Associate Degree	359	3.53
9 Four-year College/Bachelor's Degree	269	2.65
10 Master's Degree	16	0.16
11 Doctoral Degree	5	0.05
Total	10,161	100.00

dd004_w4: Relationship with R

	Freq.	%
1 Spouse/Partner	5,445	53.59
2 Child	1,783	17.55
3 Grandchild	363	3.57
4 Sibling	162	1.59
5 Parent	48	0.47
6 Friend	287	2.82
7 Guardian	5	0.05
8 Neighbor	1,010	9.94
9 Other	1,057	10.40
Total	10,160	100.00

dd005_w4: How Many Years Have Kown R

Mean	SD	Min	Max	Obs
39.90	14.33	0.17	88.00	8,164

dd006_w4: Meet Frequency

	Freq.	%
1 Lives with R	6,410	63.12
2 Daily	1,809	17.81
3 Several Times/Week	427	4.20
4 Once A Week	359	3.53
5 1-3 Times A Month	484	4.77
6 Less Than Once A Month	472	4.65
7 Never	26	0.26
8 Other	169	1.66
Total	10,156	100.00

dd007_w4: Caregiver

	Freq.	%
1 Yes	6,865	67.60
2 No	3,291	32.40
Total	10,156	100.00

dd008_w4: R Been Diagnose with Stroke

	Freq.	%

	Freq.	%
1 Yes	819	8.06
2 No	9,125	89.86
9 Don't Know	211	2.08
Total	10,155	100.00

dd009_w4: R Been Diagnose with Parkinson's Disease

	Freq.	%
1 Yes	189	1.86
2 No	9,592	94.47
9 Don't Know	373	3.67
Total	10,154	100.00

dd010_w4: R Been Diagnose with Alzheimer's Disease

	Freq.	%
1 Yes	210	2.07
2 No	9,627	94.81
9 Don't Know	317	3.12
Total	10,154	100.00

dd011_w4: R Been Diagnose with Memory Problems

	Freq.	%
1 Yes	656	6.46
2 No	9,122	89.85
9 Don't Know	374	3.68
Total	10,152	100.00

dd012_w4: Recognize Faces of Families and Friends

	Freq.	%
1 Much Better	180	1.77
2 Improved A Little	230	2.27
3 Not Much Changed	6,836	67.34
4 A little Worse	1,593	15.69
5 Much Worse	1,212	11.94
6 Not Applicable	16	0.16
8 Don't Know	76	0.75
9 Refused to Answer	8	0.08
Total	10,151	100.00

dd013_w4: Remember Names of Families and Friends

	Freq.	%
1 Much Better	129	1.27
2 Improved A Little	167	1.65
3 Not Much Changed	7,272	71.64
4 A little Worse	1,500	14.78
5 Much Worse	957	9.43
6 Not Applicable	14	0.14
8 Don't Know	107	1.05

9 Refused to Answer	5	0.05
Total	10,151	100.00

dd014_w4: Remember Occupations Etc

	Freq.	%
1 Much Better	105	1.03
2 Improved A Little	199	1.96
3 Not Much Changed	6,364	62.69
4 A little Worse	1,793	17.66
5 Much Worse	1,361	13.41
6 Not Applicable	80	0.79
8 Don't Know	244	2.40
9 Refused to Answer	5	0.05
Total	10,151	100.00

dd015_w4: Remember Things Happened Recently

	Freq.	%
1 Much Better	105	1.03
2 Improved A Little	176	1.73
3 Not Much Changed	6,090	59.99
4 A little Worse	2,071	20.40
5 Much Worse	1,476	14.54
6 Not Applicable	32	0.32
8 Don't Know	192	1.89
9 Refused to Answer	9	0.09
Total	10,151	100.00

dd016_w4: Recall Conversations A Few Days before

	Freq.	%
1 Much Better	91	0.90
2 Improved A Little	137	1.35
3 Not Much Changed	5,371	52.91
4 A little Worse	2,454	24.17
5 Much Worse	1,740	17.14
6 Not Applicable	38	0.37
8 Don't Know	310	3.05
9 Refused to Answer	10	0.10
Total	10,151	100.00

dd017_w4: Forget What to Say When Talk

	Freq.	%
1 Much Better	50	0.49
2 Improved A Little	94	0.93
3 Not Much Changed	4,739	46.69
4 A little Worse	2,908	28.65
5 Much Worse	1,945	19.16
6 Not Applicable	217	2.14
8 Don't Know	186	1.83
9 Refused to Answer	12	0.12

Total	10,151	100.00

dd018_w4: Remember Address and Telephone Number

	Freq.	%
1 Much Better	72	0.71
2 Improved A Little	130	1.28
3 Not Much Changed	6,338	62.45
4 A little Worse	1,646	16.22
5 Much Worse	1,512	14.90
6 Not Applicable	275	2.71
8 Don't Know	163	1.61
9 Refused to Answer	13	0.13
Total	10,149	100.00

dd019_w4: Remember What Day and Month It is

	Freq.	%
1 Much Better	74	0.73
2 Improved A Little	126	1.24
3 Not Much Changed	5,920	58.33
4 A little Worse	1,885	18.57
5 Much Worse	1,667	16.43
6 Not Applicable	232	2.29
8 Don't Know	228	2.25
9 Refused to Answer	17	0.17
Total	10,149	100.00

dd020_w4: Remember Where Things Are Usually Kept

	Freq.	%
1 Much Better	64	0.63
2 Improved A Little	66	0.65
3 Not Much Changed	4,177	41.16
4 A little Worse	3,212	31.65
5 Much Worse	2,344	23.10
6 Not Applicable	31	0.31
8 Don't Know	244	2.40
9 Refused to Answer	10	0.10
Total	10,148	100.00

dd021_w4: Able to Find Things Which Have Been Put in Different Place

	Freq.	%
1 Much Better	62	0.61
2 Improved A Little	98	0.97
3 Not Much Changed	4,529	44.64
4 A little Worse	2,897	28.55
5 Much Worse	2,245	22.13
6 Not Applicable	54	0.53
8 Don't Know	250	2.46
9 Refused to Answer	11	0.11
Total	10,146	100.00

dd022_w4: Able to Adapt Changes in Daily Life

	Freq.	%
1 Much Better	227	2.24
2 Improved A Little	428	4.22
3 Not Much Changed	5,651	55.70
4 A little Worse	1,907	18.80
5 Much Worse	1,524	15.02
6 Not Applicable	180	1.77
8 Don't Know	214	2.11
9 Refused to Answer	15	0.15
Total	10,146	100.00

dd023_w4: Ability to Use Regular Machines Around House Such as TV

	Freq.	%
1 Much Better	197	1.94
2 Improved A Little	339	3.34
3 Not Much Changed	5,747	56.64
4 A little Worse	1,857	18.30
5 Much Worse	1,641	16.17
6 Not Applicable	200	1.97
8 Don't Know	151	1.49
9 Refused to Answer	14	0.14
Total	10,146	100.00

dd024_w4: Ability to Use New Machines Around House

	Freq.	%
1 Much Better	213	2.10
2 Improved A Little	511	5.04
3 Not Much Changed	4,804	47.35
4 A little Worse	1,993	19.64
5 Much Worse	1,864	18.37
6 Not Applicable	531	5.23
8 Don't Know	216	2.13
9 Refused to Answer	14	0.14
Total	10,146	100.00

dd025_w4: Ability to Learn New Things

	Freq.	%
1 Much Better	172	1.70
2 Improved A Little	421	4.15
3 Not Much Changed	4,163	41.03
4 A little Worse	2,339	23.05
5 Much Worse	2,288	22.55
6 Not Applicable	507	5.00
8 Don't Know	240	2.37
9 Refused to Answer	16	0.16
Total	10,146	100.00

dd026_w4: Ability to Remember Past Things Happened at Young or Childhood

	Freq.	%
1 Much Better	75	0.74
2 Improved A Little	169	1.67
3 Not Much Changed	5,052	49.80
4 A little Worse	2,143	21.13
5 Much Worse	2,068	20.39
6 Not Applicable	58	0.57
8 Don't Know	563	5.55
9 Refused to Answer	16	0.16
Total	10,144	100.00

dd027_w4: Ability to Remember Things Learned at Yong

	Freq.	%
1 Much Better	82	0.81
2 Improved A Little	132	1.30
3 Not Much Changed	4,733	46.66
4 A little Worse	2,260	22.28
5 Much Worse	2,228	21.97
6 Not Applicable	128	1.26
8 Don't Know	561	5.53
9 Refused to Answer	19	0.19
Total	10,143	100.00

dd028_w4: Understand Uncommon Used Words

	Freq.	%
1 Much Better	69	0.68
2 Improved A Little	219	2.16
3 Not Much Changed	3,836	37.82
4 A little Worse	1,600	15.77
5 Much Worse	1,478	14.57
6 Not Applicable	2,438	24.04
8 Don't Know	484	4.77
9 Refused to Answer	19	0.19
Total	10,143	100.00

dd029_w4: Understand Aticles in Journals or Papers

	Freq.	%
1 Much Better	64	0.63
2 Improved A Little	141	1.39
3 Not Much Changed	3,873	38.18
4 A little Worse	1,032	10.17
5 Much Worse	1,104	10.88
6 Not Applicable	3,639	35.88
8 Don't Know	268	2.64
9 Refused to Answer	22	0.22
Total	10,143	100.00

dd030_w4: Understand Storis in Books or TV

	Freq.	%
1 Much Better	130	1.28
2 Improved A Little	292	2.88
3 Not Much Changed	5,320	52.46
4 A little Worse	1,448	14.28
5 Much Worse	1,377	13.58
6 Not Applicable	1,203	11.86
8 Don't Know	350	3.45
9 Refused to Answer	22	0.22
Total	10,142	100.00

dd031_w4: Ability to Write Letters

	Freq.	%
1 Much Better	39	0.38
2 Improved A Little	87	0.86
3 Not Much Changed	3,317	32.71
4 A little Worse	1,007	9.93
5 Much Worse	1,083	10.68
6 Not Applicable	4,298	42.38
8 Don't Know	289	2.85
9 Refused to Answer	22	0.22
Total	10,142	100.00

dd032_w4: Known Important Historical Event

	Freq.	%
1 Much Better	102	1.01
2 Improved A Little	191	1.88
3 Not Much Changed	4,967	48.97
4 A little Worse	1,551	15.29
5 Much Worse	1,681	16.57
6 Not Applicable	1,015	10.01
8 Don't Know	607	5.99
9 Refused to Answer	28	0.28
Total	10,142	100.00

dd033_w4: Make Decisions on Everyday Matters

	Freq.	%
1 Much Better	138	1.36
2 Improved A Little	244	2.41
3 Not Much Changed	6,047	59.62
4 A little Worse	1,676	16.53
5 Much Worse	1,663	16.40
6 Not Applicable	133	1.31
8 Don't Know	216	2.13
9 Refused to Answer	25	0.25
Total	10,142	100.00

dd034_w4: Handling Money for Shopping

	Freq.	%
1 Much Better	185	1.82
2 Improved A Little	300	2.96
3 Not Much Changed	6,782	66.87
4 A little Worse	1,192	11.75
5 Much Worse	1,310	12.92
6 Not Applicable	257	2.53
8 Don't Know	97	0.96
9 Refused to Answer	19	0.19
Total	10,142	100.00

dd035_w4: Handling Finacial Matters

	Freq.	%
1 Much Better	177	1.75
2 Improved A Little	244	2.41
3 Not Much Changed	6,009	59.25
4 A little Worse	1,305	12.87
5 Much Worse	1,516	14.95
6 Not Applicable	634	6.25
8 Don't Know	233	2.30
9 Refused to Answer	23	0.23
Total	10,141	100.00

dd036_w4: Handling Everyday Arithmatic Problems

	Freq.	%
1 Much Better	124	1.22
2 Improved A Little	204	2.01
3 Not Much Changed	5,945	58.62
4 A little Worse	1,746	17.22
5 Much Worse	1,641	16.18
6 Not Applicable	237	2.34
8 Don't Know	220	2.17
9 Refused to Answer	25	0.25
Total	10,142	100.00

dd037_w4: Understand What's Going on and to Reason Things through

	Freq.	%
1 Much Better	137	1.35
2 Improved A Little	230	2.27
3 Not Much Changed	5,455	53.79
4 A little Worse	1,953	19.26
5 Much Worse	1,895	18.68
6 Not Applicable	175	1.73
8 Don't Know	268	2.64
9 Refused to Answer	29	0.29
Total	10,142	100.00

dd039_w4: Eating with Help

	Freq.	%
1 Without Help	9,708	95.75
2 Need A Little Help to Do	231	2.28
3 Need Much Help to Do	86	0.85
4 Unable to Do	80	0.79
8 Don't Know	16	0.16
9 Refused to Answer	18	0.18
Total	10,139	100.00

dd040_w4: Using Toilet with Help

	Freq.	%
1 Without Help	9,756	96.23
2 Need A Little Help to Do	160	1.58
3 Need Much Help to Do	93	0.92
4 Unable to Do	75	0.74
8 Don't Know	37	0.36
9 Refused to Answer	17	0.17
Total	10,138	100.00

dd041_w4: Dressing with Help

	Freq.	%
1 Without Help	9,554	94.24
2 Need A Little Help to Do	308	3.04
3 Need Much Help to Do	121	1.19
4 Unable to Do	119	1.17
8 Don't Know	19	0.19
9 Refused to Answer	17	0.17
Total	10,138	100.00

dd042_w4: Ability to Handle Affairs or Speak Become Much Worse

	Freq.	%
1 No	6,143	60.59
2 Yes	3,897	38.44
8 Don't Know	74	0.73
9 Refused to Answer	25	0.25
Total	10,139	100.00

dd043_w4: Thinking or Understanding Ability Become Much Worse

	Freq.	%
1 No	5,188	51.17
2 Yes	4,762	46.97
8 Don't Know	166	1.64
9 Refused to Answer	23	0.23
Total	10,139	100.00

dd044_w4: Always Forgot Where Things to be Placed

	Freq.	%
1 No	3,822	37.70
2 Yes	5,995	59.13
8 Don't Know	295	2.91
9 Refused to Answer	26	0.26
Total	10,138	100.00

dd045_w4: Forgot What Happened Day before Today

	Freq.	%
1 No	5,555	54.81
2 Yes	4,153	40.98
8 Don't Know	397	3.92
9 Refused to Answer	30	0.30
Total	10,135	100.00

dd046_w4: Forgot Where He is

	Freq.	%
1 No	8,244	81.35
2 Yes	1,643	16.21
8 Don't Know	224	2.21
9 Refused to Answer	23	0.23
Total	10,134	100.00

dd047_w4: Have Difficulty in Dressing

	Freq.	%
1 No	9,179	90.59
2 Yes	886	8.74
8 Don't Know	45	0.44
9 Refused to Answer	23	0.23
Total	10,133	100.00

dd048_w4_s1: Situations Happened during Interview 1

	Freq.	%
0 No	9,651	95.28
1 Informant Was Hesitate to Repond Because of the Presence of R	478	4.72
Total	10,129	100.00

dd048_w4_s2: Situations Happened during Interview 2

	Freq.	%
0 No	9,760	96.36
2 Quality Not Good for Cognition Problems of Informant	369	3.64
Total	10,129	100.00

dd048_w4_s3: Situations Happened during Interview 3

	Freq.	%
0 No	9,817	96.92
3 Quality Not Good for Emotion Problems of Informant	312	3.08
Total	10,129	100.00

dd048_w4_s4: Situations Happened during Interview 4

	Freq.	%
0 No	9,755	96.31
4 Interfered by Other Affairs or Noisy	374	3.69
Total	10,129	100.00

dd048_w4_s5: Situations Happened during Interview 5

	Freq.	%
0 No	9,916	97.90
5 Other	213	2.10
Total	10,129	100.00

dd048_w4_s6: Situations Happened during Interview 6

	Freq.	%
0 No	1,397	13.79
6 None of the above	8,732	86.21
Total	10,129	100.00

dd049_w4: Laguage Used by Interviwers

	Freq.	%
1 Mandarin	5,791	57.17
2 Local Dialect	4,202	41.48
3 Other Dialect	136	1.34
Total	10,129	100.00

dd050_w4: Using Translator during Interview

	Freq.	%
1 Yes	320	3.16
2 No	9,809	96.84
Total	10,129	100.00

dd051_w4: Interviewed by Telephone

	Freq.	%
1 Yes	277	14.59
2 No	1,621	85.41
Total	1,898	100.00

versionID: Version ID

A String Variable	
Obs:	11,011

This page intentionally left blank

MODULE E

Health Care and Insurance

ID: Individual ID

A String Variable	
Obs:	19,776

householdID: Household ID

A String Variable	
Obs:	19,776

communityID: Community ID

A String Variable	
Obs:	19,776

ea001_w4_s1: Urban Employee Medical Insurance

	Freq.	%
0 No	16,924	85.60
1 Yes	2,848	14.40
Total	19,772	100.00

ea001_w4_s2: Urban and Rural Resident Medical Insurance

	Freq.	%
0 No	17,375	87.88
2 Yes	2,397	12.12
Total	19,772	100.00

ea001_w4_s3: Urban Resident Medical Insurance

	Freq.	%

	Freq.	%
0 No	18,933	95.76
3 Yes	839	4.24
Total	19,772	100.00

ea001_w4_s4: New Rural Cooperative Medical Insurance

	Freq.	%
0 No	7,021	35.51
4 Yes	12,751	64.49
Total	19,772	100.00

ea001_w4_s5: Government Medical Insurance

	Freq.	%
0 No	19,545	98.85
5 Yes	227	1.15
Total	19,772	100.00

ea001_w4_s6: Medical Aid

	Freq.	%
0 No	19,709	99.68
6 Yes	63	0.32
Total	19,772	100.00

ea001_w4_s7: Private Medical Insurance, Work Unit

	Freq.	%
0 No	19,706	99.67
7 Yes	66	0.33
Total	19,772	100.00

ea001_w4_s8: Private Mdical Insurance, Individual

	Freq.	%
0 No	19,107	96.64
8 Yes	665	3.36
Total	19,772	100.00

ea001_w4_s9: Urban Non-employed Persons's Medical Insurance

	Freq.	%
0 No	19,739	99.83
9 Yes	33	0.17
Total	19,772	100.00

ea001_w4_s10: Long-term Care Insurance

	Freq.	%

	Freq.	%
0 No	19,752	99.90
10 Yes	20	0.10
Total	19,772	100.00

ea001_w4_s11: Other Medical Insurance

	Freq.	%
0 No	19,479	98.52
11 Yes	293	1.48
Total	19,772	100.00

ea001_w4_s12: No Insurance

	Freq.	%
0 No	19,173	96.97
12 Yes	599	3.03
Total	19,772	100.00

ea002: Supplemental Insurance

	Freq.	%
1 Yes	1,016	5.51
2 No	17,431	94.49
Total	18,447	100.00

ea003_w4_1_: Place of MI

	Freq.	%
1 This County	2,113	74.19
2 Place of Hukou	262	9.20
3 Other Place	473	16.61
Total	2,848	100.00

ea003_w4_2_: Place of MI

	Freq.	%
1 This County	2,195	91.57
2 Place of Hukou	173	7.22
3 Other Place	29	1.21
Total	2,397	100.00

ea003_w4_3_: Place of MI

	Freq.	%
1 This County	737	87.84
2 Place of Hukou	80	9.54
3 Other Place	22	2.62
Total	839	100.00

ea003_w4_4_: Place of MI

	Freq.	%
1 This County	11,206	87.89
2 Place of Hukou	1,426	11.18
3 Other Place	118	0.93
Total	12,750	100.00

ea003_w4_5_: Place of MI

	Freq.	%
1 This County	154	67.84
2 Place of Hukou	30	13.22
3 Other Place	43	18.94
Total	227	100.00

ea003_w4_6_: Place of MI

	Freq.	%
1 This County	54	85.71
2 Place of Hukou	4	6.35
3 Other Place	5	7.94
Total	63	100.00

ea003_w4_7_: Place of MI

	Freq.	%
1 This County	43	65.15
2 Place of Hukou	8	12.12
3 Other Place	15	22.73
Total	66	100.00

ea003_w4_8_: Place of MI

	Freq.	%
1 This County	484	72.78
2 Place of Hukou	53	7.97
3 Other Place	128	19.25
Total	665	100.00

ea003_w4_9_: Place of MI

	Freq.	%
1 This County	28	84.85
2 Place of Hukou	5	15.15
Total	33	100.00

ea003_w4_10_: Place of MI

	Freq.	%
1 This County	19	95.00

2 Place of Hukou	1	5.00
Total	20	100.00

ea003_w4_11_: Place of MI

	Freq.	%
1 This County	231	78.84
2 Place of Hukou	31	10.58
3 Other Place	31	10.58
Total	293	100.00

ea008_1_1_: Year

Mean	SD	Min	Max	Obs
1,996.23	14.79	1,900.00	2,018.00	2,801

ea008_2_1_: Month

Mean	SD	Min	Max	Obs
3.64	4.19	0.00	12.00	2,769

ea008_1_2_: Year

Mean	SD	Min	Max	Obs
2,010.64	6.82	1,900.00	2,018.00	2,343

ea008_2_2_: Month

Mean	SD	Min	Max	Obs
3.58	4.33	0.00	12.00	2,317

ea008_1_3_: Year

Mean	SD	Min	Max	Obs
2,008.79	6.66	1,925.00	2,018.00	823

ea008_2_3_: Month

Mean	SD	Min	Max	Obs
4.17	4.35	0.00	12.00	800

ea008_1_4_: Year

Mean	SD	Min	Max	Obs
2,008.06	5.58	1,900.00	2,018.00	12,496

ea008_2_4_: Month

Mean	SD	Min	Max	Obs
3.44	4.46	0.00	12.00	12,333

ea008_1_5_: Year

Mean	SD	Min	Max	Obs
1,984.81	17.97	1,947.00	2,018.00	223

ea008_2_5_: Month

Mean	SD	Min	Max	Obs
4.72	4.37	0.00	12.00	215

ea008_1_6_: Year

Mean	SD	Min	Max	Obs
2,007.30	16.31	1,900.00	2,018.00	61

ea008_2_6_: Month

Mean	SD	Min	Max	Obs
3.03	3.78	0.00	12.00	60

ea008_1_7_: Year

Mean	SD	Min	Max	Obs
2,006.59	10.34	1,972.00	2,018.00	66

ea008_2_7_: Month

Mean	SD	Min	Max	Obs
3.30	3.36	0.00	11.00	66

ea008_1_8_: Year

Mean	SD	Min	Max	Obs
2,009.85	8.83	1,900.00	2,018.00	663

ea008_2_8_: Month

Mean	SD	Min	Max	Obs
4.65	3.80	0.00	12.00	658

ea008_1_9_: Year

Mean	SD	Min	Max	Obs
2,009.50	10.02	1,966.00	2,017.00	32

ea008_2_9_: Month

Mean	SD	Min	Max	Obs
3.73	4.34	0.00	12.00	33

ea008_1_10_: Year

Mean	SD	Min	Max	Obs
2,014.68	6.05	1,998.00	2,018.00	19

ea008_2_10_: Month

Mean	SD	Min	Max	Obs
4.21	5.43	0.00	12.00	19

ea008_1_11_: Year

Mean	SD	Min	Max	Obs
2,011.25	8.28	1,969.00	2,018.00	283

ea008_2_11_: Month

Mean	SD	Min	Max	Obs
4.22	4.10	0.00	12.00	278

ea009_s1: Don't Need

	Freq.	%
0 No	517	86.45
1 Yes	81	13.55
Total	598	100.00

ea009_s2: Cannot Afford

	Freq.	%
0 No	407	68.06
2 Yes	191	31.94
Total	598	100.00

ea009_s3: Don't Know Where to Get

	Freq.	%
0 No	537	89.80

	Freq.	%
3 Yes	61	10.20
Total	598	100.00

ea009_s4: Don’t Trust the Insititutions

	Freq.	%
0 No	584	97.66
4 Yes	14	2.34
Total	598	100.00

ea009_s5: Don’t have Suitable Programs

	Freq.	%
0 No	591	98.83
5 Yes	7	1.17
Total	598	100.00

ea009_s6: Don’t Know or never Thought of It

	Freq.	%
0 No	551	92.14
6 Yes	47	7.86
Total	598	100.00

ea009_s7: Others

	Freq.	%
0 No	364	60.87
7 Yes	234	39.13
Total	598	100.00

eb001_w4_s1: Urban Emplyee Medical Insurance

	Freq.	%
0 No	590	98.66
1 Yes	8	1.34
Total	598	100.00

eb001_w4_s2: Urban and Rural Resident Medical Insurance

	Freq.	%
0 No	579	96.82
2 Yes	19	3.18
Total	598	100.00

eb001_w4_s3: Urban Resident Medical Insurance

	Freq.	%
0 No	592	99.00

	Freq.	%
3 Yes	6	1.00
Total	598	100.00

eb001_w4_s4: New Rural Cooperative Medical Insurance

	Freq.	%
0 No	449	75.08
4 Yes	149	24.92
Total	598	100.00

eb001_w4_s5: Government Medical Insurance

	Freq.	%
0 No	598	100.00
Total	598	100.00

eb001_w4_s6: Medical Aid

	Freq.	%
0 No	598	100.00
Total	598	100.00

eb001_w4_s7: Private Medical Insurance, Worker Union

	Freq.	%
0 No	597	99.83
7 Yes	1	0.17
Total	598	100.00

eb001_w4_s8: Private Medical Insurance, Individual

	Freq.	%
0 No	595	99.50
8 Yes	3	0.50
Total	598	100.00

eb001_w4_s9: Urban Non-employed Persons's Medical Insurance

	Freq.	%
0 No	598	100.00
Total	598	100.00

eb001_w4_s10: Long-term Care Insurance

	Freq.	%
0 No	598	100.00
Total	598	100.00

eb001_w4_s11: Other Medical Insurance

	Freq.	%
0 No	597	99.83
11 Yes	1	0.17
Total	598	100.00

eb001_w4_s12: No Insurance

	Freq.	%
0 No	183	30.60
12 Yes	415	69.40
Total	598	100.00

eb003_1_1_: Year of Stop UEMI

Mean	SD	Min	Max	Obs
2,005.88	8.97	1,994.00	2,018.00	8

eb003_1_2_: Year of Stop URRMI

Mean	SD	Min	Max	Obs
2,016.42	2.43	2,008.00	2,018.00	19

eb003_1_3_: Year of Stop URMI

Mean	SD	Min	Max	Obs
2,013.00	4.29	2,008.00	2,018.00	6

eb003_1_4_: Year of Stop NRCMI

Mean	SD	Min	Max	Obs
2,015.01	4.16	1,998.00	2,018.00	148

eb003_1_7_: Year of Stop PMI Union

Mean	SD	Min	Max	Obs
2,004.00	.	2,004.00	2,004.00	1

eb003_1_8_: Year of Stop PMI Ind

Mean	SD	Min	Max	Obs
2,010.00	5.00	2,005.00	2,015.00	3

eb003_1_11_: Year of Stop Other MI

Mean	SD	Min	Max	Obs
2,016.00	.	2,016.00	2,016.00	1

eb003_2_1_: Month of Stop UEMI

Mean	SD	Min	Max	Obs
3.00	4.28	0.00	10.00	7

eb003_2_2_: Month of Stop URRMI

Mean	SD	Min	Max	Obs
1.00	2.03	0.00	9.00	19

eb003_2_3_: Month of Stop URMI

Mean	SD	Min	Max	Obs
1.67	3.61	0.00	9.00	6

eb003_2_4_: Month of Stop NRCMI

Mean	SD	Min	Max	Obs
3.17	4.38	0.00	12.00	145

eb003_2_7_: Month of Stop PMI Union

Mean	SD	Min	Max	Obs
10.00	.	10.00	10.00	1

eb003_2_8_: Month of Stop PMI Ind

Mean	SD	Min	Max	Obs
2.33	4.04	0.00	7.00	3

eb003_2_11_: Month of Stop Other MI

Mean	SD	Min	Max	Obs
1.00	.	1.00	1.00	1

eb004_w4_1_: Reason of Stop UEMI

	Freq.	%
1 Worker Union No Longer Exist	2	25.00
3 Fired from Union	2	25.00
4 Don't Participat Myself	1	12.50
6 Too Expensive	1	12.50

	Freq.	%
7 Other Reasons	2	25.00
Total	8	100.00

eb004_w4_2_: Reason of Stop URRMI

	Freq.	%
2 Insurance No Longer Exist	2	10.53
4 Don't Participat Myself	8	42.11
6 Too Expensive	2	10.53
7 Other Reasons	7	36.84
Total	19	100.00

eb004_w4_3_: Reason of Stop URMI

	Freq.	%
4 Don't Participat Myself	1	16.67
6 Too Expensive	1	16.67
7 Other Reasons	4	66.67
Total	6	100.00

eb004_w4_4_: Reason of Stop NRCMI

	Freq.	%
2 Insurance No Longer Exist	7	4.70
4 Don't Participat Myself	28	18.79
5 Don't Participat Family	4	2.68
6 Too Expensive	56	37.58
7 Other Reasons	54	36.24
Total	149	100.00

eb004_w4_7_: Reason of Stop PMI Union

	Freq.	%
1 Worker Union No Longer Exist	1	100.00
Total	1	100.00

eb004_w4_8_: Reason of Stop PMI Ind

	Freq.	%
4 Don't Participat Myself	2	66.67
7 Other Reasons	1	33.33
Total	3	100.00

eb004_w4_11_: Reason of Stop Other MI

	Freq.	%
7 Other Reasons	1	100.00
Total	1	100.00

ec001: Time of Taking Last Physical Examination

	Freq.	%
1 Year and Month	194	62.58
2 Didn't Ever Take	116	37.42
Total	310	100.00

ec001_1: Year

Mean	SD	Min	Max	Obs
2,016.02	8.78	1,900.00	2,018.00	199

ec001_2: Month

Mean	SD	Min	Max	Obs
5.76	3.23	0.00	12.00	196

ec001_w4: Time of Last Physical Examination

	Freq.	%
1 Year and Month	9,253	47.56
2 Didn't Take	10,203	52.44
Total	19,456	100.00

ec001_w4_1: Year

Mean	SD	Min	Max	Obs
2,017.55	8.22	2,000.00	2,816.00	9,515

ec001_w4_2: Month

Mean	SD	Min	Max	Obs
5.05	2.80	0.00	12.00	9,450

ec001_w3_1_s1: Physical Examination

	Freq.	%
0 No	3,126	33.09
1 Yes	6,321	66.91
Total	9,447	100.00

ec001_w3_1_s2: Routine Blood Test

	Freq.	%
0 No	1,031	10.91
2 Yes	8,416	89.09
Total	9,447	100.00

ec001_w3_1_s3: Routine Urine Test

	Freq.	%
0 No	2,631	27.85
3 Yes	6,816	72.15
Total	9,447	100.00

ec001_w3_1_s4: Liver Function Test

	Freq.	%
0 No	3,517	37.23
4 Yes	5,930	62.77
Total	9,447	100.00

ec001_w3_1_s5: Kidney Function Test

	Freq.	%
0 No	4,065	43.03
5 Yes	5,382	56.97
Total	9,447	100.00

ec001_w3_1_s6: Lipids Profile Test

	Freq.	%
0 No	2,622	27.75
6 Yes	6,825	72.25
Total	9,447	100.00

ec001_w3_1_s7: Blood Glucose Test

	Freq.	%
0 No	2,498	26.44
7 Yes	6,949	73.56
Total	9,447	100.00

ec001_w3_1_s8: Surgical

	Freq.	%
0 No	5,714	60.48
8 Yes	3,733	39.52
Total	9,447	100.00

ec001_w3_1_s9: Internal Medicine

	Freq.	%
0 No	4,941	52.30
9 Yes	4,506	47.70
Total	9,447	100.00

ec001_w3_1_s10: Five Sense Organ Test

	Freq.	%
0 No	5,587	59.14
10 Yes	3,860	40.86
Total	9,447	100.00

ec001_w3_1_s11: Electrocardiogram

	Freq.	%
0 No	2,290	24.24
11 Yes	7,157	75.76
Total	9,447	100.00

ec001_w3_1_s12: B-type Ultrasonic

	Freq.	%
0 No	2,895	30.64
12 Yes	6,552	69.36
Total	9,447	100.00

ec001_w3_1_s13: Chest Fluoroscopy

	Freq.	%
0 No	4,471	47.33
13 Yes	4,976	52.67
Total	9,447	100.00

ec001_w3_1_s14: Male or Female Specialist

	Freq.	%
0 No	6,647	70.36
14 Yes	2,800	29.64
Total	9,447	100.00

ec001_w3_1_s15: Other

	Freq.	%
0 No	8,932	94.55
15 Yes	515	5.45
Total	9,447	100.00

ed001: Accept Outpatient Care

	Freq.	%
1 Yes	3,255	16.47
2 No	16,509	83.53
Total	19,764	100.00

ed004_w4_s1: General Hospital

	Freq.	%
0 No	1,957	60.12
1 Yes	1,298	39.88
Total	3,255	100.00

ed004_w4_s2: Specialized Hospital

	Freq.	%
0 No	3,108	95.48
2 Yes	147	4.52
Total	3,255	100.00

ed004_w4_s3: Chinese Medicine Hospital

	Freq.	%
0 No	3,017	92.69
3 Yes	238	7.31
Total	3,255	100.00

ed004_w4_s4: Community Healthcare Center

	Freq.	%
0 No	3,063	94.10
4 Yes	192	5.90
Total	3,255	100.00

ed004_w4_s5: Township Hospital

	Freq.	%
0 No	2,525	77.57
5 Yes	730	22.43
Total	3,255	100.00

ed004_w4_s6: Health Care Post

	Freq.	%
0 No	3,157	96.99
6 Yes	98	3.01
Total	3,255	100.00

ed004_w4_s7: Village Clinic/Private Clinic

	Freq.	%
0 No	2,532	77.79
7 Yes	723	22.21
Total	3,255	100.00

ed004_w4_s8: Institution

	Freq.	%
0 No	3,252	99.91
8 Yes	3	0.09
Total	3,255	100.00

ed004_w4_s9: Other

	Freq.	%
0 No	3,197	98.22
9 Yes	58	1.78
Total	3,255	100.00

ed005_1_: Times Outp GH

Mean	SD	Min	Max	Obs
1.78	1.93	1.00	30.00	1,298

ed005_2_: Times Outp SH

Mean	SD	Min	Max	Obs
2.07	3.05	1.00	30.00	147

ed005_3_: Times Outp CMH

Mean	SD	Min	Max	Obs
1.76	1.73	1.00	20.00	238

ed005_4_: Times Outp Comm

Mean	SD	Min	Max	Obs
2.24	3.35	1.00	31.00	192

ed005_5_: Times Outp Towship

Mean	SD	Min	Max	Obs
2.23	2.56	1.00	30.00	730

ed005_6_: Times Outp HCP

Mean	SD	Min	Max	Obs
1.94	2.15	1.00	20.00	98

ed005_7_: Times Outp VC

Mean	SD	Min	Max	Obs

2.60	3.27	1.00	31.00	723

ed005_8_: Times Outp Ins

Mean	SD	Min	Max	Obs
13.33	8.33	4.00	20.00	3

ed005_w4_1_: Same Condition GH

	Freq.	%
1 Yes	394	79.76
2 No	100	20.24
Total	494	100.00

ed005_w4_2_: Same Condition SH

	Freq.	%
1 Yes	53	86.89
2 No	8	13.11
Total	61	100.00

ed005_w4_3_: Same Condition CMH

	Freq.	%
1 Yes	80	87.91
2 No	11	12.09
Total	91	100.00

ed005_w4_4_: Same Condition Comm

	Freq.	%
1 Yes	73	81.11
2 No	17	18.89
Total	90	100.00

ed005_w4_5_: Same Condition Towship

	Freq.	%
1 Yes	260	74.93
2 No	87	25.07
Total	347	100.00

ed005_w4_6_: Same Condition HCP

	Freq.	%
1 Yes	32	76.19
2 No	10	23.81
Total	42	100.00

ed005_w4_7_: Same Condition VC

	Freq.	%
1 Yes	318	72.27
2 No	122	27.73
Total	440	100.00

ed005_w4_8_: Same Condition Ins

	Freq.	%
1 Yes	3	100.00
Total	3	100.00

ed006_w4: Outp Exp

	Freq.	%
1 Total Cost	1,454	93.38
2 Don't Know	103	6.62
Total	1,557	100.00

ed006_w4_1: Outp Exp

Mean	SD	Min	Max	Obs
1,712.31	6,030.36	0.00	130,000.00	1,467

ed006_w4_max: Max Bracket of ed006_w4

Mean	SD	Min	Max	Obs
432.14	314.70	50.00	1,000.00	56

ed006_w4_min: Min Bracket of ed006_w4

Mean	SD	Min	Max	Obs
552.47	386.64	50.00	1,000.00	81

ed007: OOP Outp

	Freq.	%
1 OOP Outp	1,459	93.71
2 Did not Pay Anything	52	3.34
3 Don't Know	46	2.95
Total	1,557	100.00

ed007_1: OOP Outp

Mean	SD	Min	Max	Obs
1,253.01	4,541.04	0.00	120,000.00	1,464

ed007_max: Max Bracket of ed007

Mean	SD	Min	Max	Obs
252.38	243.14	50.00	1,000.00	21

ed007_min: Min Bracket of ed007

Mean	SD	Min	Max	Obs
441.38	405.34	50.00	1,000.00	29

ed008_w4: Health Care Ins

	Freq.	%
1 General Hospital	1,231	37.82
2 Specialized Hospital	132	4.06
3 Chinese Medicine Hospital	220	6.76
4 Community Healthcare Center	170	5.22
5 Township Hospital	676	20.77
6 Health Care Post	91	2.80
7 Village Clinic or Private Clinic	678	20.83
8 Institution	3	0.09
9 Other	54	1.66
Total	3,255	100.00

ed009: Public or Private

	Freq.	%
1 Public	2,438	74.90
2 Private	817	25.10
Total	3,255	100.00

ed010: Level of Facility

	Freq.	%
1 County/District	1,154	72.90
2 Regional/City	254	16.05
3 Provincial/Ministry	96	6.06
4 Military	11	0.69
5 Others	52	3.28
6 Not Applicable	16	1.01
Total	1,583	100.00

ed012: The Provider Visit at Home

	Freq.	%
1 Yes	266	8.17
2 No	2,989	91.83
Total	3,255	100.00

ed013: Distance

Mean	SD	Min	Max	Obs
21.25	107.37	0.00	4,200.00	2,974

ed017_s1: Purpose of Visit, Immunization

	Freq.	%
0 No	3,246	99.72
1 Yes	9	0.28
Total	3,255	100.00

ed017_s2: Purpose of Visit, Consultation

	Freq.	%
0 No	3,211	98.65
2 Yes	44	1.35
Total	3,255	100.00

ed017_s3: Purpose of Visit, Medical check-up

	Freq.	%
0 No	3,103	95.33
3 Yes	152	4.67
Total	3,255	100.00

ed017_s4: Purpose of Visit, Treatment of illness

	Freq.	%
0 No	264	8.11
4 Yes	2,991	91.89
Total	3,255	100.00

ed017_s5: Purpose of Visit, Other

	Freq.	%
0 No	3,106	95.42
5 Yes	149	4.58
Total	3,255	100.00

ed019: A First or a Follow-up Visit

	Freq.	%
1 First	1,196	39.99
2 Follow-up	1,795	60.01
Total	2,991	100.00

ed020: Ordinary Outpatient or Emergency

	Freq.	%
1 Ordinary	2,848	95.22
2 Emergency	143	4.78
Total	2,991	100.00

ed023_w4: Total Cost of This Visit

	Freq.	%
1 Total Cost	3,075	94.47
2 Don't Know	180	5.53
Total	3,255	100.00

ed023_w4_1: Total Cost of This Visit

Mean	SD	Min	Max	Obs
1,398.39	7,460.28	0.00	280,000.00	3,095

ed023_w4_max: Max Bracket of ed023_w4

Mean	SD	Min	Max	Obs
380.20	431.04	25.00	1,200.00	102

ed023_w4_min: Min Bracket of ed023_w4

Mean	SD	Min	Max	Obs
424.72	446.85	25.00	1,200.00	109

ed024: OOP of This Visit

	Freq.	%
1 OOP	2,965	91.09
2 Don't Pay Anything	216	6.64
3 Don't Know	74	2.27
Total	3,255	100.00

ed024_1: OOP of This Visit

Mean	SD	Min	Max	Obs
893.75	3,990.02	0.00	120,000.00	2,998

ed024_max: Max Bracket of ed024

Mean	SD	Min	Max	Obs
263.48	332.78	15.00	1,000.00	33

ed024_min: Min Bracket of ed024

Mean	SD	Min	Max	Obs
254.09	335.75	15.00	1,000.00	33

ed028_w4_s1: Reimbursement_UEMI

	Freq.	%
0 No	3,010	92.47
1 Yes	245	7.53
Total	3,255	100.00

ed028_w4_s2: Reimbursement_URRMI

	Freq.	%
0 No	3,099	95.21
2 Yes	156	4.79
Total	3,255	100.00

ed028_w4_s3: Reimbursement_URMI

	Freq.	%
0 No	3,227	99.14
3 Yes	28	0.86
Total	3,255	100.00

ed028_w4_s4: Reimbursement_NRCMI

	Freq.	%
0 No	2,467	75.79
4 Yes	788	24.21
Total	3,255	100.00

ed028_w4_s5: Reimbursement_GMI

	Freq.	%
0 No	3,226	99.11
5 Yes	29	0.89
Total	3,255	100.00

ed028_w4_s6: Reimbursement_MA

	Freq.	%
0 No	3,250	99.85
6 Yes	5	0.15
Total	3,255	100.00

ed028_w4_s7: Reimbursement_PMI_Union

	Freq.	%
0 No	3,254	99.97
7 Yes	1	0.03
Total	3,255	100.00

ed028_w4_s8: Reimbursement_PMI_Indi

	Freq.	%
0 No	3,246	99.72
8 Yes	9	0.28
Total	3,255	100.00

ed028_w4_s9: Reimbursement_Urban Non-employed

	Freq.	%
0 No	3,255	100.00
Total	3,255	100.00

ed028_w4_s10: Reimbursement_Long-term

	Freq.	%
0 No	3,255	100.00
Total	3,255	100.00

ed028_w4_s11: Reimbursement_Other MI

	Freq.	%
0 No	3,240	99.54
11 Yes	15	0.46
Total	3,255	100.00

ed028_w4_s12: Reimbursement_Union

	Freq.	%
0 No	3,246	99.72
12 Yes	9	0.28
Total	3,255	100.00

ed028_w4_s13: Reimbursement_Noinsurance

	Freq.	%
0 No	3,067	94.22
13 Yes	188	5.78
Total	3,255	100.00

ed028_w4_s14: Not revelent

	Freq.	%
0 No	1,469	45.13
14 Yes	1,786	54.87
Total	3,255	100.00

ed029: Red Envelopes to the Doctor

	Freq.	%
1 Yes	12	0.37
2 No	3,243	99.63
Total	3,255	100.00

ed030_w4: Reimbursement

	Freq.	%
4 None of Above	3	100.00
Total	3	100.00

ee003: Received Inpatient Care

	Freq.	%
1 Yes	3,327	16.83
2 No	16,437	83.17
Total	19,764	100.00

ee004: Times have Received Inpatient Care

Mean	SD	Min	Max	Obs
1.67	2.96	1.00	150.00	3,327

ee004_w4: Same Reason

	Freq.	%
1 Yes	781	70.68
2 No	324	29.32
Total	1,105	100.00

ee005_w4: Total Medical Cost of Inpatient

	Freq.	%
1 Total Medical Cost of Inpatient	987	89.32
2 Don't Know	118	10.68
Total	1,105	100.00

ee005_w4_1: Total Cost

Mean	SD	Min	Max	Obs

27,486.51	61,724.42	0.00	1,400,000.00	1,003

ee005_w4_max: Max Bracket of ee005_w4

Mean	SD	Min	Max	Obs
13,648.65	9,523.85	1,500.00	30,000.00	74

ee005_w4_min: Min Bracket of ee005_w4

Mean	SD	Min	Max	Obs
12,603.45	9,566.82	1,500.00	30,000.00	87

ee006: OOP of Inpatient

	Freq.	%
1 OOP of Inpatient	1,007	91.13
2 Didn't Pay Anything	20	1.81
3 Don't Know	78	7.06
Total	1,105	100.00

ee006_1: Self-paid Part

Mean	SD	Min	Max	Obs
14,405.69	39,536.99	0.00	1,000,000.00	1,007

ee006_max: Max Bracket of ee006

Mean	SD	Min	Max	Obs
6,662.79	5,345.58	600.00	18,000.00	43

ee006_min: Min Bracket of ee006

Mean	SD	Min	Max	Obs
6,171.74	5,311.48	600.00	18,000.00	46

ee007: Same Facility as Outpatient

	Freq.	%
1 Yes	496	55.05
2 No	405	44.95
Total	901	100.00

ee008_w4: Type of Health Servie Facility

	Freq.	%
1 General Hospital	1,798	63.53
2 Specialized Hospital	278	9.82

	Freq.	%
3 Chinese Medicine Hospital	286	10.11
4 Community Healthcare Center	37	1.31
5 Township Hospital	387	13.67
6 Health Care Post	7	0.25
7 Nursing Agency	3	0.11
8 Other	34	1.20
Total	2,830	100.00

ee009: Facility Public or Private

	Freq.	%
1 Public	2,585	91.34
2 Private	245	8.66
Total	2,830	100.00

ee010: Level of Facility

	Freq.	%
1 County/District	1,679	71.08
2 Regional/City	450	19.05
3 Provincial/Ministry	155	6.56
4 Military	17	0.72
5 Others	61	2.58
Total	2,362	100.00

ee012_w4: Location of Facility

	Freq.	%
1 This County	1,824	64.45
2 Other	1,006	35.55
Total	2,830	100.00

ee013: Distance

Mean	SD	Min	Max	Obs
49.96	201.22	0.00	6,000.00	2,799

ee016: Nights were you Hospitalized

Mean	SD	Min	Max	Obs
12.44	19.22	0.00	365.00	3,324

ee017_1: Starting Year of Hospital Stay

Mean	SD	Min	Max	Obs
2,017.54	0.65	2,000.00	2,018.00	3,325

ee017_2: Starting Month of Hospital Stay

Mean	SD	Min	Max	Obs
5.78	3.42	0.00	12.00	3,310

ee017_3: Starting Day of Hospital Stay

Mean	SD	Min	Max	Obs
7.66	9.38	0.00	31.00	3,269

ee018: Data of Exit

	Freq.	%
1 Year Month Day	2,958	95.11
2 Still There	152	4.89
Total	3,110	100.00

ee018_1: Year of Exit

Mean	SD	Min	Max	Obs
2,017.60	0.59	2,000.00	2,018.00	3,263

ee018_2: Month of Exit

Mean	SD	Min	Max	Obs
5.73	3.51	0.00	12.00	3,253

ee018_3: Day of Exit

Mean	SD	Min	Max	Obs
9.06	10.22	0.00	31.00	3,209

ee019: Reason of Hospitalized

	Freq.	%
1 Sickness	2,991	89.98
2 Accident	253	7.61
4 Other	80	2.41
Total	3,324	100.00

ee024_w4: Total Medical Cost of Hospitalization

	Freq.	%
1 Total Medical Cost of Hospitalization	3,015	90.70
2 Don't Know	309	9.30
Total	3,324	100.00

ee024_w4_1: Total Cost

Mean	SD	Min	Max	Obs
11,966.24	34,144.46	0.00	1,400,000.00	3,054

ee024_w4_max: Max Bracket of ee024_w4

Mean	SD	Min	Max	Obs
6,234.41	4,714.78	700.00	15,000.00	186

ee024_w4_min: Min Bracket of ee024_w4

Mean	SD	Min	Max	Obs
5,831.88	4,748.73	700.00	15,000.00	207

ee027: OOP of Hospitalization

	Freq.	%
1 OOP of Hospitalization	2,989	89.95
2 Didn't Pay Anything	102	3.07
3 Don't Know	232	6.98
Total	3,323	100.00

ee027_1: Self-paid Part

Mean	SD	Min	Max	Obs
6,431.57	22,593.47	0.00	1,000,000.00	3,011

ee027_max: Max Bracket of ee027

Mean	SD	Min	Max	Obs
3,541.82	2,635.17	400.00	10,000.00	110

ee027_min: Min Bracket of ee027

Mean	SD	Min	Max	Obs
3,445.76	3,323.41	400.00	10,000.00	118

ee031_w4_s1: Reimbursement_UEMI

	Freq.	%
0 No	2,791	83.99
1 Yes	532	16.01
Total	3,323	100.00

ee031_w4_s2: Reimbursement_URRMI

	Freq.	%

	Freq.	%
0 No	2,979	89.65
2 Yes	344	10.35
Total	3,323	100.00

ee031_w4_s3: Reimbursement_URMI

	Freq.	%
0 No	3,179	95.67
3 Yes	144	4.33
Total	3,323	100.00

ee031_w4_s4: Reimbursement_NRCMI

	Freq.	%
0 No	1,460	43.94
4 Yes	1,863	56.06
Total	3,323	100.00

ee031_w4_s5: Reimbursement_GMI

	Freq.	%
0 No	3,277	98.62
5 Yes	46	1.38
Total	3,323	100.00

ee031_w4_s6: Reimbursement_MA

	Freq.	%
0 No	3,317	99.82
6 Yes	6	0.18
Total	3,323	100.00

ee031_w4_s7: Reimbursement_PMI_Union

	Freq.	%
0 No	3,322	99.97
7 Yes	1	0.03
Total	3,323	100.00

ee031_w4_s8: Reimbursement_PMI_indi

	Freq.	%
0 No	3,300	99.31
8 Yes	23	0.69
Total	3,323	100.00

ee031_w4_s9: Reimbursement_Urban Non-employed

	Freq.	%

	Freq.	%
0 No	3,322	99.97
9 Yes	1	0.03
Total	3,323	100.00

ee031_w4_s10: Reimbursement_Long-term

	Freq.	%
0 No	3,323	100.00
Total	3,323	100.00

ee031_w4_s11: Reimbursement_Other MI

	Freq.	%
0 No	3,297	99.22
11 Yes	26	0.78
Total	3,323	100.00

ee031_w4_s12: Reimbursement_Union

	Freq.	%
0 No	3,301	99.34
12 Yes	22	0.66
Total	3,323	100.00

ee031_w4_s13: Reimbursement_Noinsurance

	Freq.	%
0 No	3,265	98.25
13 Yes	58	1.75
Total	3,323	100.00

ee031_w4_s14: Not Revelent

	Freq.	%
0 No	3,035	91.33
14 Yes	288	8.67
Total	3,323	100.00

ee032: Pay Red Envelopes

	Freq.	%
1 Yes	48	1.44
2 No	3,275	98.56
Total	3,323	100.00

ee033_w4: Reimbursement

	Freq.	%
3 Nursing and Recovery Fee	1	33.33

4 None	2	66.67
Total	3	100.00

ef001_w4: Take Any Purchased Medicine

	Freq.	%
1 Yes	11,382	57.60
2 No	8,378	42.40
Total	19,760	100.00

ef002_w4: Cost for Purchased Medicine

	Freq.	%
1 Total Cost	10,968	96.37
2 Don't Know	413	3.63
Total	11,381	100.00

ef002_w4_1: Total Cost

Mean	SD	Min	Max	Obs
283.19	749.78	0.00	40,000.00	10,985

ef002_w4_max: Max Bracket of ef002_w4

Mean	SD	Min	Max	Obs
146.46	87.71	10.00	300.00	254

ef002_w4_min: Min Bracket of ef002_w4

Mean	SD	Min	Max	Obs
148.87	103.96	10.00	300.00	309

ef003: OOP for Purchased Medicine

	Freq.	%
1 OOP	10,535	93.45
2 Didn't pay anything	510	4.52
3 Don't Know	228	2.02
Total	11,273	100.00

ef003_1: OOP

Mean	SD	Min	Max	Obs
263.84	702.45	0.00	40,000.00	10,636

ef003_max: Max Bracket of ef003

Mean	SD	Min	Max	Obs
148.66	101.03	10.00	300.00	97

ef003_min: Min Bracket of ef003

Mean	SD	Min	Max	Obs
151.82	109.08	10.00	300.00	110

ef005_w4_s1: Reimbursement_UEMI

	Freq.	%
0 No	10,646	94.45
1 Yes	626	5.55
Total	11,272	100.00

ef005_w4_s2: Reimbursement_URRMI

	Freq.	%
0 No	10,863	96.37
2 Yes	409	3.63
Total	11,272	100.00

ef005_w4_s3: Reimbursement_URMI

	Freq.	%
0 No	11,195	99.32
3 Yes	77	0.68
Total	11,272	100.00

ef005_w4_s4: Reimbursement_NRCMI

	Freq.	%
0 No	9,653	85.64
4 Yes	1,619	14.36
Total	11,272	100.00

ef005_w4_s5: Reimbursement_GMI

	Freq.	%
0 No	11,197	99.33
5 Yes	75	0.67
Total	11,272	100.00

ef005_w4_s6: Reimbursement_MA

	Freq.	%
0 No	11,265	99.94

	Freq.	%
6 Yes	7	0.06
Total	11,272	100.00

ef005_w4_s7: Reimbursement_PMI_Union

	Freq.	%
0 No	11,266	99.95
7 Yes	6	0.05
Total	11,272	100.00

ef005_w4_s8: Reimbursement_PMI_Indi

	Freq.	%
0 No	11,256	99.86
8 Yes	16	0.14
Total	11,272	100.00

ef005_w4_s9: Reimbursement_Urban Non-employed

	Freq.	%
0 No	11,269	99.97
9 Yes	3	0.03
Total	11,272	100.00

ef005_w4_s10: Reimbursement_Long-term

	Freq.	%
0 No	11,272	100.00
Total	11,272	100.00

ef005_w4_s11: Reimbursement_Other MI

	Freq.	%
0 No	11,243	99.74
11 Yes	29	0.26
Total	11,272	100.00

ef005_w4_s12: Reimbursement_Union

	Freq.	%
0 No	11,247	99.78
12 Yes	25	0.22
Total	11,272	100.00

ef005_w4_s13: Reimbursement_Noinsurance

	Freq.	%
0 No	10,504	93.19
13 Yes	768	6.81

	Freq.	%
Total	11,272	100.00

ef005_w4_s14: Not Revelent

	Freq.	%
0 No	3,614	32.06
14 Yes	7,658	67.94
Total	11,272	100.00

eh001_w4: Have Long term Care Insurance

	Freq.	%
1 Yes	12	0.06
2 No	19,746	99.94
Total	19,758	100.00

eh002_w4_s1: Basic Life Care

	Freq.	%
0 No	8	66.67
1 Yes	4	33.33
Total	12	100.00

eh002_w4_s2: Common Clinical Care

	Freq.	%
0 No	7	58.33
2 Yes	5	41.67
Total	12	100.00

eh002_w4_s3: Risk Prevention Guidance

	Freq.	%
0 No	10	83.33
3 Yes	2	16.67
Total	12	100.00

eh002_w4_s4: Functional Maintenance or Rehabilitation Training

	Freq.	%
0 No	9	75.00
4 Yes	3	25.00
Total	12	100.00

eh002_w4_s5: Other

	Freq.	%
0 No	7	58.33
5 Yes	5	41.67

Total	12	100.00

eh003_w4_1: Expense for Long-term Care Service

Mean	SD	Min	Max	Obs
165.27	366.04	0.00	1,000.00	11

eh003_w4_2: Expense Only for Long-term Care Insurance

Mean	SD	Min	Max	Obs
258.17	384.58	0.00	1,000.00	12

eh004_w4: Long-term Care Insurance Ielp

	Freq.	%
1 Completely Helpful	2	16.67
2 Very Helpful	3	25.00
3 Somewhat Helpful	3	25.00
4 Not Very Helpful	1	8.33
5 Not at All Helpful	3	25.00
Total	12	100.00

eh005_w4_s1: Aged Care Service Centers

	Freq.	%
0 No	10,956	99.41
1 Yes	65	0.59
Total	11,021	100.00

eh005_w4_s2: Regular Medical Examination

	Freq.	%
0 No	9,219	83.65
2 Yes	1,802	16.35
Total	11,021	100.00

eh005_w4_s3: Home Visit

	Freq.	%
0 No	10,650	96.63
3 Yes	371	3.37
Total	11,021	100.00

eh005_w4_s4: Family Bed

	Freq.	%
0 No	11,004	99.85
4 Yes	17	0.15

	Freq.	%
Total	11,021	100.00

eh005_w4_s5: Community Care

	Freq.	%
0 No	10,964	99.48
5 Yes	57	0.52
Total	11,021	100.00

eh005_w4_s6: Health Management

	Freq.	%
0 No	10,846	98.41
6 Yes	175	1.59
Total	11,021	100.00

eh005_w4_s7: Entertainment

	Freq.	%
0 No	10,765	97.68
7 Yes	256	2.32
Total	11,021	100.00

eh005_w4_s8: Other

	Freq.	%
0 No	10,988	99.70
8 Yes	33	0.30
Total	11,021	100.00

eh005_w4_s9: None of the Above

	Freq.	%
0 No	2,177	19.75
9 Yes	8,844	80.25
Total	11,021	100.00

eh007_w3: Satisfied with Local Health Care Services

	Freq.	%
1 Very Satisfied	2,778	14.62
2 Somewhat Satisfied	4,369	23.00
3 Neutral	8,757	46.09
4 Somewhat dissatisfied	1,401	7.37
5 Very Dissatisfied	1,694	8.92
Total	18,999	100.00

eh008_w4: Family Doctor Signing Service

	Freq.	%
1 Yes	812	4.11
2 No	18,943	95.89
Total	19,755	100.00

ef006: Receive Assistance in Answering

	Freq.	%
1 Never	15,006	75.96
2 A Few Times	3,176	16.08
3 Most or All of the Time	571	2.89
4 The Section was Completed by a Proxy Respondent	1,002	5.07
Total	19,755	100.00

ef007: The relationship to R

	Freq.	%
1 Spouse	553	55.19
2 Mother	10	1.00
3 Father	3	0.30
4 Mother in Law	7	0.70
5 Father in Law	8	0.80
6 Sibling	19	1.90
7 Brother in Law or sister in Law	13	1.30
8 Child	226	22.55
9 Spouse of Child	68	6.79
10 Grandchild	38	3.79
11 Other Relative	29	2.89
12 Helper or Other Non-relative"	28	2.79
Total	1,002	100.00

ef008: Main reason for Proxy

	Freq.	%
1 Has Serious Physical Handicaps	365	36.43
2 Has Serious Mental Handicaps	119	11.88
3 Has Rejected This Interview	241	24.05
4 Other	277	27.64
Total	1,002	100.00

versionID: Version ID

A String Variable	
Obs:	19,776

MODULE F

Work and Retirement

ID: Individual ID

A String Variable	
Obs:	19,725

householdID: Household ID

A String Variable	
Obs:	19,725

communityID: Community ID

A String Variable	
Obs:	19,725

fa002_w4: Nonfarm Work (for at Least One Hour Last Month) or Not

	Freq.	%
1 Yes	5,597	28.38
2 No	14,123	71.62
Total	19,720	100.00

fa003: Temporarily Laid off, on Sick or Other Leave, On-the-job Training, or Not

	Freq.	%
1 Yes	256	1.81
2 No	13,867	98.19
Total	14,123	100.00

fa004_1: Year (Beginning of the Leave)

Mean	SD	Min	Max	Obs
2,017.41	3.31	1,983.00	2,018.00	254

fa004_2: Month (Beginning of the Leave)

Mean	SD	Min	Max	Obs
6.21	2.00	0.00	12.00	256

fa005: Expect to Return to the Job or Not

	Freq.	%
1 Yes	201	78.52
2 No	55	21.48
Total	256	100.00

fa006: Still Get Paid on the Job or Not

	Freq.	%
1 Yes	12	21.82
2 No	43	78.18
Total	55	100.00

fa006_w3_1: Agricultural Work in the LastIW or Not

	Freq.	%
1 Yes	1,536	79.96
2 No	385	20.04
Total	1,921	100.00

fa006_w3_2_s1: Family Agricultural Work in the LastIW or Not

	Freq.	%
0 No	11	0.72
1 Household Agricultural Work	1,525	99.28
Total	1,536	100.00

fa006_w3_2_s2: Employed Agricultural Work in the LastIW or Not

	Freq.	%
0 No	1,430	93.10
2 Farm Employed	106	6.90
Total	1,536	100.00

fa006_w3_3: Non-agricultural Work in the LastIW or Not

	Freq.	%
1 Yes	728	37.90

	Freq.	%
2 No	1,193	62.10
Total	1,921	100.00

fa006_w3_4: Type of Non-agricultural Work in the LastIW

	Freq.	%
1 Employed	431	59.20
2 Self-employed	238	32.69
3 Unpaid Family Business	59	8.10
Total	728	100.00

fa007: Ever Worked for Three Months or Not

	Freq.	%
1 Yes	565	88.01
2 No	77	11.99
Total	642	100.00

fa008: Never Worked for Three Months or Not

	Freq.	%
1 Yes, Never Worked Before	56	72.73
2 No, Ever Worked	21	27.27
Total	77	100.00

fa009: Reason for Never Having Worked Before

	Freq.	%
1 Disabled (Physical or Psychological)	12	21.43
2 Homemaker	28	50.00
3 My Family Is So Rich That I Don't Need to Work	1	1.79
5 Other	15	26.79
Total	56	100.00

fa010_w4_1: Since When Started to Work After the LastIW

	Freq.	%
1 Correct Recall: Not Working in the LastIW	476	39.67
2 Wrong Recall: Working in the LastIW	724	60.33
Total	1,200	100.00

fa010_w4_1_1: Year (When Started to Work since the LastIW)

Mean	SD	Min	Max	Obs
1,977.32	275.98	-1.00	2,018.00	525

fa010_w4_1_2: Month (When Started to Work since the LastIW)

Mean	SD	Min	Max	Obs

5.49	3.14	-1.00	12.00	487

fa010_w4_3: Reason to Start to Work

	Freq.	%
1 More Income	230	48.32
2 Health Problem	34	7.14
3 Family Care	32	6.72
4 Personal Development	3	0.63
5 Other	177	37.18
Total	476	100.00

fa010_w4_4: Type of the Job When Started to Work

	Freq.	%
1 Nonfarm Employed	184	38.66
2 Nonfarm Self-employed	41	8.61
3 Unpaid Family Business	33	6.93
4 Farm Employed	38	7.98
5 Other	180	37.82
Total	476	100.00

fa011_w4_1: Since When Stopped Working After the LastIW

	Freq.	%
1 Correct Recall: Working in the LastIW	1,550	68.64
2 Wrong Recall: Not Working in the LastIW	708	31.36
Total	2,258	100.00

fa011_w4_1_1: Year (Since When Stopped Working)

Mean	SD	Min	Max	Obs
2,011.24	100.94	-1.00	2,018.00	1,595

fa011_w4_1_1_1: Year (Since When Stopped Working if not Worked in the LastIW)

Mean	SD	Min	Max	Obs
1,965.29	289.70	-1.00	2,018.00	708

fa011_w4_1_1_2: Month (Since When Stopped Working if not Worked in the LastIW)

Mean	SD	Min	Max	Obs
5.18	3.86	-1.00	12.00	513

fa011_w4_1_2: Month (Since When Stopped Working)

Mean	SD	Min	Max	Obs

5.89	3.61	-1.00	12.00	1,447

fa011_w4_2: Type of the Last Job

	Freq.	%
1 Nonfarm Employed	766	33.92
2 Nonfarm Self-employed	161	7.13
3 Unpaid Family Business	64	2.83
4 Farm Employed	86	3.81
5 Other	1,181	52.30
Total	2,258	100.00

fa011_w4_3: Reason to Stop Working

	Freq.	%
1 Forced to Leave	132	5.85
2 Health Problem	1,155	51.15
3 Family Care	392	17.36
4 Retirement	217	9.61
5 Other	362	16.03
Total	2,258	100.00

fa011_w4_4: Benefits When Leaving the Last Job

	Freq.	%
1 Yes	44	1.95
2 No	2,214	98.05
Total	2,258	100.00

fa011_w4_5_1: Benefits (in Yuan)

Mean	SD	Min	Max	Obs
32,298.86	61,259.75	200.00	240,000.00	42

fa011_w4_max: Max Bracket of FA011_W4_5_1

Mean	SD	Min	Max	Obs
10,000.00	.	10,000.00	10,000.00	1

fa011_w4_min: Min Bracket of FA011_W4_5_1

Mean	SD	Min	Max	Obs
12,500.00	10,606.60	5,000.00	20,000.00	2

fa011_w4_5_3: Years of Employment to Compute Retirement Benefits When Forced to Leave

Mean	SD	Min	Max	Obs
9.84	14.75	0.00	50.00	37

fa012_w4_1: Since When at Work Other than Self-Employed Farming after the LastIW

	Freq.	%
1 Correct Recall: At Work Other Than Self-employed Farming in the LastIW	645	64.44
2 Wrong Recall: Only Self-employed Farming in the LastIW	356	35.56
Total	1,001	100.00

fa012_w4_1_1: Year (Since When at Work Other than Self-Employed Farming)

Mean	SD	Min	Max	Obs
1,975.80	282.22	-1.00	2,018.00	702

fa012_w4_1_2: Month (Since When at Work Other than Self-Employed Farming)

Mean	SD	Min	Max	Obs
5.23	3.17	-1.00	12.00	666

fa012_w4_2_1: Year (Start of the Current Job)

Mean	SD	Min	Max	Obs
1,960.32	332.85	-1.00	2,018.00	644

fa012_w4_2_2: Month (Start of the Current Job)

Mean	SD	Min	Max	Obs
5.14	3.20	-1.00	12.00	625

fa012_w4_3: Reason to Have a Job Other than Self-Employed Farming

	Freq.	%
1 Forced to Change	14	2.17
2 More Income	366	56.83
3 Health Problem	50	7.76
4 Family Care	52	8.07
5 Residence Change	8	1.24
6 Other	154	23.91
Total	644	100.00

fa013_w4_1: Since When Stopped Working on Jobs Other than Self-Employed Farming

	Freq.	%
1 Correct Recall: Only Self-employed Farming in the LastIW	667	58.51
2 Wrong Recall: At Work Other Than Self-employed Farming in the LastIW	473	41.49
Total	1,140	100.00

fa013_w4_1_1: Year (Since When Left Jobs Other than Self-Employed Farming)

Mean	SD	Min	Max	Obs
2,001.77	172.38	-1.00	2,018.00	681

fa013_w4_1_2: Month (Since When Left Jobs Other than Self-Employed Farming)

Mean	SD	Min	Max	Obs
5.59	3.41	-1.00	12.00	634

fa013_w4_2_1: Year (Since When Only at Work of Self-Employed Farming)

Mean	SD	Min	Max	Obs
1,986.50	245.39	-1.00	2,018.00	667

fa013_w4_2_2: Month (Since When Only at Work of Self-Employed Farming)

Mean	SD	Min	Max	Obs
5.24	3.44	-1.00	12.00	633

fa013_w4_3: Reason to Only Have a Job of Self-Employed Farming

	Freq.	%
1 Forced to Change	72	10.79
2 More Income	14	2.10
3 Health Problem	273	40.93
4 Family Care	121	18.14
5 Residence Change	2	0.30
6 Retirement	31	4.65
7 Other	154	23.09
Total	667	100.00

fa014_w4_1: Since When Left the Employed Nonfarm Work Reported in the LastIW

	Freq.	%
1 Correct Recall: Employed in the LastIW	101	53.72
2 Wrong Recall: Self-employed in the LastIW	87	46.28
Total	188	100.00

fa014_w4_1_1: Year (Since When Left Employed Jobs)

Mean	SD	Min	Max	Obs
2,016.48	1.11	2,012.00	2,018.00	104

fa014_w4_1_2: Month (Since When Left Employed Jobs)

Mean	SD	Min	Max	Obs

5.50	3.74	1.00	12.00	92

fa014_w4_2_1: Year (Since When at Work of the Current Self-Employed Job)

Mean	SD	Min	Max	Obs
1,936.61	395.44	-1.00	2,018.00	101

fa014_w4_2_2: Month (Since When at Work of the Current Self-Employed Job)

Mean	SD	Min	Max	Obs
5.03	3.55	-1.00	12.00	92

fa014_w4_3: Reason to Switch from Employed to Self-Employed Jobs

	Freq.	%
1 Inheritance or Bestowal From Relatives	1	0.99
2 Forced to Change	9	8.91
3 More Income	34	33.66
4 Health Problem	21	20.79
5 Family Care	7	6.93
6 Retirement	5	4.95
7 Other	24	23.76
Total	101	100.00

fa014_w4_4: From Which Relative to Take Over the Self-Employed Business

	Freq.	%
6 Other Relative	1	100.00
Total	1	100.00

fa015_w4_1: Since When Left the Self-Employed Nonfarm Work Reported in the LastIW

	Freq.	%
1 Correct Recall: Self-employed in the LastIW	185	52.86
2 Wrong Recall: Employed in the LastIW	165	47.14
Total	350	100.00

fa015_w4_1_1: Year (Since When Left Self-Employed Jobs)

Mean	SD	Min	Max	Obs
1,985.58	248.32	-1.00	2,018.00	196

fa015_w4_1_2: Month (Since When Left Self-Employed Jobs)

Mean	SD	Min	Max	Obs
6.09	3.31	-1.00	12.00	182

fa015_w4_2_1: Year (Since When at Work of the Current Employed Job)

Mean	SD	Min	Max	Obs
1,896.53	478.40	-1.00	2,018.00	185

fa015_w4_2_2: Month (Since When at Work of the Current Employed Job)

Mean	SD	Min	Max	Obs
5.21	3.54	-1.00	12.00	180

fa015_w4_3: Reason to Switch from Self-Employed to Employed Jobs

	Freq.	%
1 Inherited or Gifted to Relatives	2	1.08
2 Forced to Change	24	12.97
3 More Income	71	38.38
4 Health Problem	31	16.76
5 Family Care	14	7.57
6 Lost Due to Family Dispute	1	0.54
7 Other	42	22.70
Total	185	100.00

fa015_w4_4: To Which Relative to Give Away the Self-Employed Business

	Freq.	%
3 Spouse	1	50.00
7 Grandchild	1	50.00
Total	2	100.00

fb011_w4: Completed Retirement Procedures or Internal Retirement

	Freq.	%
1 Yes	559	3.35
2 No	16,131	96.65
Total	16,690	100.00

fb012: Completed Receding Position Procedures

	Freq.	%
1 Yes	52	0.32
2 No	16,079	99.68
Total	16,131	100.00

fc001: Farm Employed or Not

	Freq.	%
1 Yes	868	4.40
2 No	18,853	95.60
Total	19,721	100.00

fc008: Self-Employed (Household) Farm Work (for at Least One Month Last Year) or Not

	Freq.	%
1 Yes	8,971	45.49
2 No	10,751	54.51
Total	19,722	100.00

fc009: Months for Household Agricultural Work

Mean	SD	Min	Max	Obs
6.19	4.25	0.03	12.00	8,893

fc010: Days for Household Agricultural Work

Mean	SD	Min	Max	Obs
5.10	2.15	0.00	7.00	8,894

fc011: Hours for Household Agricultural Work

Mean	SD	Min	Max	Obs
5.77	3.09	0.05	24.00	8,922

fc012: Location of the Most Frequent Workplace

	Freq.	%
1 Same as the Residence	8,508	94.85
2 Another Neighborhood but Same Province-city as the Residence	248	2.76
3 Other Domestic Location	214	2.39
Total	8,970	100.00

fc012_w3: Satisfaction of the Household Agricultural Work

	Freq.	%
1 Completely Satisfied	380	4.24
2 Very Satisfied	2,327	25.94
3 Somewhat Satisfied	4,939	55.06
4 Not Very Satisfied	976	10.88
5 Not at All Satisfied	348	3.88
Total	8,970	100.00

fc013: Days Not Working due to Health Problems

Mean	SD	Min	Max	Obs
16.77	44.63	0.00	365.00	8,970

fc019_w4_b: More than One Employed Agricultural Job or Not

	Freq.	%
1 Yes	47	9.22
2 No	463	90.78
Total	510	100.00

fc019_w4_c: More than One Non-Agricultural Job or Not

	Freq.	%
1 Yes	52	2.45
2 No	2,070	97.55
Total	2,122	100.00

fc019_w4_e: More than One Employed Agricultural Job or Not

	Freq.	%
1 Yes	4	3.54
2 No	109	96.46
Total	113	100.00

fc019_w4_f: More than One Non-Agricultural Job or Not

	Freq.	%
1 Yes	105	3.05
2 No	3,337	96.95
Total	3,442	100.00

fc020_w4_a: Main Job Employed or Self Employed

	Freq.	%
1 Employed Nonfarm	132	65.67
2 Self-employed Nonfarm	27	13.43
3 Unpaid Family Business Nonfarm	5	2.49
4 Employed Farming	37	18.41
Total	201	100.00

fc020_w4_c: Main Job Employed or Self Employed

	Freq.	%
1 Employed Nonfarm	39	75.00
2 Self-employed Nonfarm	13	25.00
Total	52	100.00

fc020_w4_d: Main Job Employed or Self Employed

	Freq.	%
1 Employed Nonfarm	32	72.73
2 Self-employed Nonfarm	4	9.09
3 Unpaid Family Business Nonfarm	3	6.82

4 Employed Farming	5	11.36
Total	44	100.00

fc020_w4_f: Main Job Employed or Self Employed

	Freq.	%
1 Employed Nonfarm	66	62.86
2 Self-employed Nonfarm	35	33.33
3 Unpaid Family Business Nonfarm	4	3.81
Total	105	100.00

fc021_w4_c: The Job Being Employed or Self-Employed

	Freq.	%
1 Employed Nonfarm	1,589	76.76
2 Self-employed Nonfarm	388	18.74
3 Unpaid Family Business Nonfarm	93	4.49
Total	2,070	100.00

fc021_w4_f: The Job Being Employed or Self-Employed

	Freq.	%
1 Employed Nonfarm	2,320	69.52
2 Self-employed Nonfarm	858	25.71
3 Unpaid Family Business Nonfarm	159	4.76
Total	3,337	100.00

fc023_w4: Occupation (Type) of the Family Farm Work

	Freq.	%
1 Manual Labor	8,382	93.44
2 Management	202	2.25
3 Machine/Vehicle Operator	325	3.62
4 Other	61	0.68
Total	8,970	100.00

fc024_w4: Number of Employees Hired by the Family Farm Business

Mean	SD	Min	Max	Obs
0.50	20.08	0.00	1,888.00	8,970

fd001: Receive Wage from the Workplace or Dispatch Company

	Freq.	%
1 Workplace	2,147	44.33
2 Dispatch Company	190	3.92
3 Individual	2,506	51.74
Total	4,843	100.00

fd002: Type of the Employer

	Freq.	%
1 Government	396	8.18
2 Institutions	383	7.91
3 NGO	30	0.62
4 Firm	1,125	23.23
5 Individual Firm	1,982	40.93
6 Farmer	585	12.08
7 Individual Household	162	3.35
8 Other	180	3.72
Total	4,843	100.00

fd003_w4_1: Same Employer as in the LastIW

	Freq.	%
1 Yes	1,124	61.02
2 No	718	38.98
Total	1,842	100.00

fd003_w4_2: Same Employer as in the LastIW if Employer Missing in the Record

	Freq.	%
1 Yes	897	44.25
2 No	1,130	55.75
Total	2,027	100.00

fd003_w4_3: Reason for the Employer Change

	Freq.	%
1 Forced to Change	243	21.50
2 Better Paid	192	16.99
3 Health	123	10.88
4 Other Personal	80	7.08
5 Residence Location Consideration	51	4.51
6 Retirement	35	3.10
7 Other	406	35.93
Total	1,130	100.00

fd004: Location of the Workplace

	Freq.	%
1 Same as the Residence	2,946	60.84
2 Another Neighborhood but Same Province-city as the Residence	1,160	23.96
3 Other Domestic Location	723	14.93
4 Abroad	13	0.27
Total	4,842	100.00

fd006: Civil Servant or Not

	Freq.	%
1 Yes	71	17.93
2 No	325	82.07
Total	396	100.00

fd007: Formal Employee or Not

	Freq.	%
1 Yes	159	40.15
2 No	237	59.85
Total	396	100.00

fd009: Formal Employee or Not

	Freq.	%
1 Yes	225	58.75
2 No	158	41.25
Total	383	100.00

fd010: Ownership Type of Employer

	Freq.	%
1 State-owned Firm	186	16.53
2 State-controlled Firm	48	4.27
3 Collective-owned Firm	40	3.56
4 Collective-controlled Firm	27	2.40
5 Private Firm	542	48.18
6 Private-controlled Firm	183	16.27
7 Foreign-owned Firm	9	0.80
8 Joint Venture	11	0.98
9 Other Joint Ownership	3	0.27
10 Other	76	6.76
Total	1,125	100.00

fd011_1: When Did You Start Working for This Employer(Year)

Mean	SD	Min	Max	Obs
2,010.86	10.43	1,900.00	2,018.00	4,826

fd011_2: When Did You Start Working for This Employer(Month)

Mean	SD	Min	Max	Obs
4.88	3.36	0.00	12.00	4,805

fd013: Current Position

	Freq.	%
1 Clerk/Worker	4,021	83.04
2 Team Head	119	2.46

	Freq.	%
3 Section Head	53	1.09
4 Division Head	9	0.19
5 Bureau Head or More Senior	8	0.17
6 Village Official	86	1.78
7 Township Official	8	0.17
8 Division Manager	41	0.85
9 Chief Executive	8	0.17
10 Other	489	10.10
Total	4,842	100.00

fd014: Current Professional Level

	Freq.	%
1 Technician	71	1.47
2 Primary Level	85	1.76
3 Intermediate Level	177	3.66
4 Advanced Level	154	3.18
5 No Professional/Technical Level	4,355	89.94
Total	4,842	100.00

fd015: Supervise Anyone or Not

	Freq.	%
1 Yes	521	10.76
2 No	4,321	89.24
Total	4,842	100.00

fd016: How Many People under Your Supervision

	Freq.	%
1 1 5 People	148	28.41
2 6 10 People	102	19.58
3 11 15 People	72	13.82
4 16 30 People	48	9.21
5 31 99 People	58	11.13
6 More Than 100 People	93	17.85
Total	521	100.00

fd020: Any Written Labor Contract with the Current Employer or Not

	Freq.	%
1 Yes	1,087	22.45
2 No	3,755	77.55
Total	4,842	100.00

fd021: Labor Contract Period

	Freq.	%
1 Defined Preiod	621	57.13
2 Not Defined	415	38.18
3 Same as the Term of the Project	51	4.69

	Freq.	%
Total	1,087	100.00

fd021_1: Years

Mean	SD	Min	Max	Obs
243.23	654.19	0.00	2,046.00	629

fd021_2: Months

Mean	SD	Min	Max	Obs
1.11	2.91	0.00	12.00	629

fd022: Current Employment Contract Ever Been Renewed or Not

	Freq.	%
1 Yes	447	66.52
2 No	225	33.48
Total	672	100.00

fd023: Times The Contract Ever Been Renewed

Mean	SD	Min	Max	Obs
5.69	5.37	1.00	35.00	427

fd024: Expected Time to Continue Work at the Current Workplace

	Freq.	%
1 Less Than One Year	1,762	36.39
2 One to Two Years	827	17.08
3 Two to Three Years	452	9.33
4 More Than Three Years	1,801	37.20
Total	4,842	100.00

fd025: Why Do You Expect So

	Freq.	%
1 Because the Predefined Contract Period Will Expire	45	1.74
2 Because Typically the Contract Expires	54	2.09
3 Because I Was Hired Under the Condition That I Would Resign Upon the Request of My Employer	97	3.75
4 Because the Current Job Will Be Completed	644	24.87
5 Because the Person I Am Substituting/Replacing Will Return to Work	4	0.15
6 Because I Can Only Work During Certain Seasons	72	2.78
7 Because I Plan to Find Another Job That Better Suits My Job Aptitude, Abilities, and Preferences	145	5.60
8 Because I Will Reach Retirement Age as Set By Regualtions	217	8.38
9 Because of Family Care Responsibilites, Poor Health, Etc.	691	26.69
10 Other	620	23.95
Total	2,589	100.00

fd029: Days of Paid Vacations

Mean	SD	Min	Max	Obs
4.27	18.56	0.00	365.00	4,833

fd030: Days of Sick Leaves

Mean	SD	Min	Max	Obs
3.79	19.56	0.00	365.00	4,842

fd031: Days of Paid Sick Leaves

Mean	SD	Min	Max	Obs
5.28	24.37	0.00	365.00	833

fd032_w3: Have Occupational Accident Insurance or Not

	Freq.	%
1 Yes	748	15.45
2 No	4,094	84.55
Total	4,842	100.00

fd033_w3: Maximum Amount Payable Upon Work-Related Injuries

Mean	SD	Min	Max	Obs
378,308.27	4,742,660.23	0.00	99999999.00	445

fd034_w4: Commercial Medical Insurance Provided by the Employer

	Freq.	%
1 Yes	105	2.17
2 No	4,737	97.83
Total	4,842	100.00

fe001: How Many Months Did You Work in The Past Year

Mean	SD	Min	Max	Obs
7.59	4.33	1.00	12.00	4,790

fe002: How Many Days Did You Work per Week

Mean	SD	Min	Max	Obs
5.69	1.63	1.00	7.00	4,773

fe003: How Many Hours Did You Work per Day

Mean	SD	Min	Max	Obs
8.80	2.92	1.00	24.00	4,811

ff001: Periodic Pattern of Wage Payment

	Freq.	%
1 Yearly Pay	364	7.52
2 Monthly Pay	2,502	51.67
3 Weekly Pay	17	0.35
4 Daily Pay	889	18.36
5 Hourly Pay	95	1.96
6 Contract-based	309	6.38
7 Performance-based	375	7.74
8 Other	291	6.01
Total	4,842	100.00

ff002_w4_1: Annual Salary Received After Tax or Not

	Freq.	%
1 Yes	26	7.14
2 No	318	87.36
997 Do Not Know	20	5.49
Total	364	100.00

ff002_w4_2_1: Amount of Tax (and Others) Deducted or Should be Deducted from the Annual Salary

Mean	SD	Min	Max	Obs
530.48	5,838.89	0.00	100,000.00	321

ff002_w4_2_max: Max Bracket of FF002_W4_2_1

Mean	SD	Min	Max	Obs
11,818.18	8,528.03	10,000.00	50,000.00	22

ff002_w4_2_min: Min Bracket of FF002_W4_2_1

Mean	SD	Min	Max	Obs
50,000.00	.	50,000.00	50,000.00	1

ff002_w4_a: Salary Last Year (in Yuan)

Mean	SD	Min	Max	Obs
25,604.23	43,276.33	0.00	500,000.00	361

ff002_w4_max: Max Bracket of FF002_W4_A

Mean	SD	Min	Max	Obs
7,000.00	9,013.88	5,000.00	50,000.00	25

ff002_w4_min: Min Bracket of FF002_W4_A

Mean	SD	Min	Max	Obs
12,142.86	16,797.11	5,000.00	50,000.00	7

ff002_w4_b: Bonus Included in the Salary Last Year (in Yuan)

Mean	SD	Min	Max	Obs
481.16	3,887.85	0.00	60,000.00	359

ff004_w4_1: Monthly Salary Received After Tax or Not

	Freq.	%
1 Yes	759	30.34
2 No	1,645	65.75
997 Do Not Know	98	3.92
Total	2,502	100.00

ff004_w4_2_1: Amount of Tax Deducted or Should be Deducted from the Monthly Salary

Mean	SD	Min	Max	Obs
299.11	1,088.66	0.00	26,400.00	2,164

ff004_w4_2_max: Max Bracket of FF004_W4_2_1

Mean	SD	Min	Max	Obs
288.30	306.86	200.00	2,500.00	94

ff004_w4_2_min: Min Bracket of FF004_W4_2_1

Mean	SD	Min	Max	Obs
666.67	599.60	200.00	2,500.00	15

ff004_w4_a: Salary Last Month (in Yuan)

Mean	SD	Min	Max	Obs
3,086.74	4,854.51	0.00	210,000.00	2,469

ff004_w4_max: Max Bracket of FF004_W4_A

Mean	SD	Min	Max	Obs

856.52	1,025.48	500.00	5,000.00	115

ff004_w4_min: Min Bracket of FF004_W4_A

Mean	SD	Min	Max	Obs
1,960.00	1,383.84	500.00	5,000.00	25

ff004_w4_b: Bonus Included in the Salary Last Month (in Yuan)

Mean	SD	Min	Max	Obs
120.86	680.55	0.00	16,000.00	2,436

ff006_w4: Salary Last Week (in Yuan)

Mean	SD	Min	Max	Obs
440.63	560.46	0.00	2,000.00	16

ff006_w4_max: Max Bracket of FF006_W4

Mean	SD	Min	Max	Obs
50.00	73.48	20.00	200.00	6

ff006_w4_min: Min Bracket of FF006_W4

Mean	SD	Min	Max	Obs
200.00	.	200.00	200.00	1

ff008: Daily Wage (in Yuan)

Mean	SD	Min	Max	Obs
118.87	61.75	8.00	600.00	887

ff008_max: Max Bracket of FF008

Mean	SD	Min	Max	Obs
100.00	0.00	100.00	100.00	2

ff008_min: Min Bracket of FF008

Mean	SD	Min	Max	Obs
100.00	0.00	100.00	100.00	2

ff010: Hourly Wage (in Yuan)

Mean	SD	Min	Max	Obs

17.22	26.54	5.00	220.00	90

ff010_max: Max Bracket of FF010

Mean	SD	Min	Max	Obs
23.33	23.09	10.00	50.00	3

ff010_min: Min Bracket of FF010

Mean	SD	Min	Max	Obs
23.33	23.09	10.00	50.00	3

ff012_w4_1: Monthly Salary Received After Tax or Not

	Freq.	%
1 Yes	159	8.05
2 No	1,711	86.59
997 Do Not Know	106	5.36
Total	1,976	100.00

ff012_w4_2_1: Amount of Tax Deducted or Should be Deducted from the Monthly Salary

Mean	SD	Min	Max	Obs
26.64	238.06	0.00	6,000.00	1,832

ff012_w4_2_max: Max Bracket of FF012_W4_2_1

Mean	SD	Min	Max	Obs
23.07	25.90	20.00	300.00	127

ff012_w4_2_min: Min Bracket of FF012_W4_2_1

Mean	SD	Min	Max	Obs
117.50	126.06	20.00	300.00	4

ff012_w4_a: Total Salary Last Month (in Yuan) if Not Paid Monthly

Mean	SD	Min	Max	Obs
1,859.55	3,115.56	0.00	90,000.00	1,937

ff012_w4_max: Max Bracket of FF012_W4_A

Mean	SD	Min	Max	Obs
715.48	833.73	500.00	10,000.00	420

ff012_w4_min: Min Bracket of FF012_W4_A

Mean	SD	Min	Max	Obs
1,619.05	1,125.15	500.00	5,000.00	42

ff012_w4_b: Bonus Included in Total Salary Last Month (in Yuan) if Not Paid Monthly

Mean	SD	Min	Max	Obs
12.59	252.31	0.00	10,000.00	1,935

ff014: All Other Bonuses Last Year

Mean	SD	Min	Max	Obs
3,002.60	11,607.00	0.00	250,000.00	4,744

ff014_max: Max Bracket of FF014

Mean	SD	Min	Max	Obs
1,179.10	1,515.84	1,000.00	20,000.00	201

ff014_min: Min Bracket of FF014

Mean	SD	Min	Max	Obs
4,666.67	4,412.10	1,000.00	10,000.00	6

fg001_s1: Free Lunch

	Freq.	%
0 No	3,458	71.42
1 Free Lunch	1,384	28.58
Total	4,842	100.00

fg001_s2: Free Breakfast

	Freq.	%
0 No	4,039	83.42
2 Free Breakfast	803	16.58
Total	4,842	100.00

fg001_s3: Free Dinner

	Freq.	%
0 No	4,003	82.67
3 Free Dinner	839	17.33
Total	4,842	100.00

fg001_s4: Meal Cash Allowance

	Freq.	%
0 No	4,542	93.80
4 Meal Cash Allowance	300	6.20
Total	4,842	100.00

fg001_s5: Transportation Allowance

	Freq.	%
0 No	4,643	95.89
5 Transportation Allowance	199	4.11
Total	4,842	100.00

fg001_s6: Free Housing

	Freq.	%
0 No	3,974	82.07
6 Free Housing	868	17.93
Total	4,842	100.00

fg001_s7: Housing Allowance

	Freq.	%
0 No	4,772	98.55
7 Housing Allowance	70	1.45
Total	4,842	100.00

fg001_s8: Company Car

	Freq.	%
0 No	4,788	98.88
8 Company Car	54	1.12
Total	4,842	100.00

fg001_s9: Free Employee Shuttle

	Freq.	%
0 No	4,714	97.36
9 Free Employee Shuttle	128	2.64
Total	4,842	100.00

fg001_s10: Other Benefits not Mentioned Above

	Freq.	%
0 No	4,617	95.35
10 Other Benefits Not Mentioned Above	225	4.65
Total	4,842	100.00

fg001_s11: None

	Freq.	%
0 No	2,224	45.93
11 None	2,618	54.07
Total	4,842	100.00

fg002_10_: Monetary Value of the Itemized Benefits per Month Corresponding to FG001

Mean	SD	Min	Max	Obs
149.25	429.13	0.00	5,500.00	214

fg002_1_: Monetary Value of the Itemized Benefits per Month Corresponding to FG001

Mean	SD	Min	Max	Obs
205.33	183.66	0.00	2,000.00	1,346

fg002_2_: Monetary Value of the Itemized Benefits per Month Corresponding to FG001

Mean	SD	Min	Max	Obs
122.72	138.38	0.00	2,000.00	774

fg002_3_: Monetary Value of the Itemized Benefits per Month Corresponding to FG001

Mean	SD	Min	Max	Obs
196.16	192.67	0.00	2,000.00	813

fg002_4_: Monetary Value of the Itemized Benefits per Month Corresponding to FG001

Mean	SD	Min	Max	Obs
270.38	302.72	0.00	3,360.00	286

fg002_5_: Monetary Value of the Itemized Benefits per Month Corresponding to FG001

Mean	SD	Min	Max	Obs
344.19	322.46	0.00	1,800.00	186

fg002_6_: Monetary Value of the Itemized Benefits per Month Corresponding to FG001

Mean	SD	Min	Max	Obs
468.69	1,428.41	0.00	36,000.00	766

fg002_7_: Monetary Value of the Itemized Benefits per Month Corresponding to FG001

Mean	SD	Min	Max	Obs
421.35	532.59	0.00	3,000.00	65

fg002_8_: Monetary Value of the Itemized Benefits per Month Corresponding to FG001

Mean	SD	Min	Max	Obs
1,548.87	4,576.52	0.00	30,000.00	46

fg002_9_: Monetary Value of the Itemized Benefits per Month Corresponding to FG001

Mean	SD	Min	Max	Obs
224.05	272.36	0.00	1,500.00	111

fg010_w4: On What Basis Does Monthly Social Insurance Contributions Calculated

	Freq.	%
1 Specific Amount of Salary Base	451	52.02
2 Minimum Contribution Requirement	58	6.69
997 Do Not Know	358	41.29
Total	867	100.00

fg010_w4_1: Self-Contributed Social Insurance (Yuan per Month)

Mean	SD	Min	Max	Obs
1,979.71	2,440.52	0.00	21,000.00	462

fh001: How Many Months Did You Work in The Past Year

Mean	SD	Min	Max	Obs
9.82	3.45	1.00	12.00	1,577

fh002: How Many Days Did You Work per Week

Mean	SD	Min	Max	Obs
6.04	1.68	1.00	7.00	1,575

fh003: How Many Hours Did You Work per Day

Mean	SD	Min	Max	Obs
8.20	3.89	1.00	24.00	1,574

fh004: Days of Sick Leave

Mean	SD	Min	Max	Obs
9.18	31.15	0.00	365.00	1,589

fh005_w4_1: Same Self Employment Business as in the LastIW

	Freq.	%
1 Yes	439	91.08
2 No	43	8.92
Total	482	100.00

fh005_w4_2: Same Self Employment Business as in the LastIW if Missing in the Record

	Freq.	%
1 Yes	425	90.23
2 No	46	9.77
Total	471	100.00

fh005_w4_3: Reason for the Business Change

	Freq.	%
1 New Business Is Better	22	47.83
2 Old Business Failed	3	6.52
3 Forced To	7	15.22
4 Health	5	10.87
5 Other Personal	2	4.35
6 Residence Location Consideration	1	2.17
7 Other	6	13.04
Total	46	100.00

fh006: Location of the Self Employment Business

	Freq.	%
1 Same as the Residence	1,098	82.87
2 Another Neighborhood but Same Province-city as the Residence	157	11.85
3 Other Domestic Location	69	5.21
4 Abroad	1	0.08
Total	1,325	100.00

fh008_1: When Started the Business (Year)

Mean	SD	Min	Max	Obs
2,004.53	10.70	1,956.00	2,018.00	1,323

fh008_2: When Started the Business (Month)

Mean	SD	Min	Max	Obs
4.16	3.79	0.00	12.00	1,306

fh009_w4: Number of Employees

Mean	SD	Min	Max	Obs
6.81	87.98	0.00	3,000.00	1,324

fh012_w4_1: Same Family Business as in the LastIW

	Freq.	%
1 Yes	23	85.19
2 No	4	14.81
Total	27	100.00

fh012_w4_2: Same Family Business as in the LastIW if Missing in the Record

	Freq.	%
1 Yes	62	93.94
2 No	4	6.06
Total	66	100.00

fh012_w4_3: Reason for the Business Change

	Freq.	%
2 Old Business Failed	1	25.00
3 Forced To	1	25.00
7 Other	2	50.00
Total	4	100.00

fh013: Location of the Family Business

	Freq.	%
1 Same as the Residence	219	82.95
2 Another Neighborhood but Same Province-city as the Residence	28	10.61
3 Other Domestic Location	17	6.44
Total	264	100.00

fh018_1: When Did You Start Working at the Family Business (Year)

Mean	SD	Min	Max	Obs
2,009.35	9.28	1,973.00	2,018.00	263

fh018_2: When Did You Start Working at the Family Business (Month)

Mean	SD	Min	Max	Obs

4.57	3.83	0.00	12.00	260

fj001_w4: Number of Side Jobs

Mean	SD	Min	Max	Obs
1.15	0.52	1.00	5.00	453

fj002_w4: Working Hours on Side Jobs per Week

Mean	SD	Min	Max	Obs
25.54	25.36	0.00	100.00	453

fj003_w4: Monthly Income from Side Jobs (in Yuan)

Mean	SD	Min	Max	Obs
1,792.80	2,629.15	0.00	30,000.00	432

fj003_w4_max: Max Bracket of FJ003_W4

Mean	SD	Min	Max	Obs
1,533.33	1,552.26	500.00	5,000.00	15

fj003_w4_min: Min Bracket of FJ003_W4

Mean	SD	Min	Max	Obs
1,944.44	1,827.64	500.00	5,000.00	9

fk000_w3_1: Satisfaction of Non-agricultural Work

	Freq.	%
1 Completely Satisfied	193	3.00
2 Very Satisfied	1,164	18.10
3 Somewhat Satisfied	3,933	61.17
4 Not Very Satisfied	917	14.26
5 Not at All Satisfied	223	3.47
Total	6,430	100.00

fk000_w3_2: Expected Age When to Stop Working

Mean	SD	Min	Max	Obs
66.27	69.34	1.00	2,026.00	4,854

fk002: Did You Search for a Job Last Month

	Freq.	%
1 Yes	88	1.23
2 No	7,060	98.77

Total	7,148	100.00

fl001: Type of the Last Job (if Currently Not Working And New)

	Freq.	%
1 Employed	45	65.22
2 Self-employed Nonfarm	10	14.49
3 Unpaid Family Business Nonfarm	1	1.45
4 Self-employed Farming	13	18.84
Total	69	100.00

fl002_1: When Did You Start Working at the Last Job (Year)

Mean	SD	Min	Max	Obs
1,989.30	20.54	1,900.00	2,018.00	67

fl002_2: When Did You Start Working at the Last Job (Month)

Mean	SD	Min	Max	Obs
4.35	4.28	0.00	12.00	66

fl003_1: When Did You Stop Working at the Last Job (Year)

Mean	SD	Min	Max	Obs
2,010.48	7.58	1,991.00	2,018.00	67

fl003_2: When Did You Stop Working at the Last Job (Month)

Mean	SD	Min	Max	Obs
4.32	3.71	0.00	12.00	65

fl004: Location of the Last Job

	Freq.	%
1 Same as the Residence	39	56.52
2 Another Neighborhood but Same Province-city as the Residence	13	18.84
3 Other Domestic Location	17	24.64
Total	69	100.00

fl004_w4_2: Occupation of the Farm Work

	Freq.	%
1 Manual Labor	12	92.31
3 Machine/Vehicle Operator	1	7.69
Total	13	100.00

fl007: This Employer Still Exists or Not

	Freq.	%
1 Yes	34	60.71
2 No	22	39.29
Total	56	100.00

fl008: How Many Hours Did You Work per Week

Mean	SD	Min	Max	Obs
40.71	21.97	0.00	112.00	56

fl009: Monthly Wage before You Stopped Working at This Job

Mean	SD	Min	Max	Obs
2,624.67	4,603.10	0.00	30,000.00	43

fl009_max: Max Bracket of FL009

Mean	SD	Min	Max	Obs
5,000.00	.	5,000.00	5,000.00	1

fl009_min: Min Bracket of FL009

Mean	SD	Min	Max	Obs
5,000.00	.	5,000.00	5,000.00	1

fl011: Other Bonuses per Year

Mean	SD	Min	Max	Obs
3,711.90	16,735.86	0.00	100,000.00	42

fl011_max: Max Bracket of FL011

Mean	SD	Min	Max	Obs
839.29	1,795.33	500.00	10,000.00	28

fl011_min: Min Bracket of FL011

Mean	SD	Min	Max	Obs
3,666.67	5,484.83	500.00	10,000.00	3

fl012_w4: Number of Employees

Mean	SD	Min	Max	Obs
219.70	313.75	0.00	1,000.00	10

fl013: Formal Employee, Contracted, or Temporary

	Freq.	%
1 Formal Worker	22	48.89
2 Contracted Worker	6	13.33
3 Temporarily-hired Worker	17	37.78
Total	45	100.00

fl014: Type of Employer

	Freq.	%
1 Government	5	11.11
2 Institutions	4	8.89
4 Firm	19	42.22
5 Individual Firm	14	31.11
7 Individual Household	1	2.22
8 Other	2	4.44
Total	45	100.00

fl015: Were You a Civil Servant

	Freq.	%
1 Yes	4	80.00
2 No	1	20.00
Total	5	100.00

fl016: Ownership Type of Employer

	Freq.	%
1 State-owned Firm	11	57.89
2 State-controlled Firm	1	5.26
3 Collective-owned Firm	1	5.26
5 Private Firm	4	21.05
6 Private-controlled Firm	1	5.26
8 Joint Venture	1	5.26
Total	19	100.00

fl020_w4: Reason to Leave the Last Job and Stop Working

	Freq.	%
1 Forced to Leave	11	15.94
2 Health Problem	8	11.59
3 Family Care	12	17.39
4 Retirement	19	27.54
5 Other	19	27.54
Total	69	100.00

fl021: Did You Receive Any Severance Pay upon Leaving the Last Job

	Freq.	%
1 Yes	2	2.90

	Freq.	%
2 No	67	97.10
Total	69	100.00

fl022_w4_1: Benefits (in Yuan)

Mean	SD	Min	Max	Obs
6,250.00	353.55	6,000.00	6,500.00	2

fl022_w4_3: Years of Employment to Compute Retirement Benefits When Forced to Leave

Mean	SD	Min	Max	Obs
0.00	.	0.00	0.00	1

fm000_w4: Retirement Processed in the LastIW

	Freq.	%
1 Correct Recall: Retired in the LastIW	1,131	67.44
2 Wrong Recall: Not Retired in the LastIW	546	32.56
Total	1,677	100.00

fm001: The Office Where Retirement Processed Belongs to the Current Employer or Others

	Freq.	%
1 Current Employer	34	5.56
2 Last Employer	106	17.35
3 None of the Above	471	77.09
Total	611	100.00

fm003: Type of the Office That Processed Retirement/Receding Position

	Freq.	%
1 Government	134	28.45
2 Institutions	65	13.80
3 NGO	5	1.06
4 Firm	218	46.28
5 Individual Firm	19	4.03
6 Farmer	8	1.70
7 Individual Household	3	0.64
8 Other	19	4.03
Total	471	100.00

fm004: Location of the Retirement Processing Office

	Freq.	%
1 Same as the Residence	909	46.98
2 Another Neighborhood but Same Province-city as the Residence	632	32.66
3 Other Domestic Location	388	20.05

4 Abroad	6	0.31
Total	1,935	100.00

fm005_1: When Did You Receded from the Position (Year)

Mean	SD	Min	Max	Obs
2,007.20	10.34	1,960.00	2,018.00	111

fm005_2: When Did You Receded from the Position (Month)

Mean	SD	Min	Max	Obs
5.34	4.27	0.00	12.00	111

fm006: Reason You Receded from the Position

	Freq.	%
1 Due to Poor Health, I Couldn't Continue My Work Any More	7	13.46
4 I Receded From My Position Voluntary	18	34.62
5 Reach Retirement Age but Not Eligible Working Age	2	3.85
6 Other	25	48.08
Total	52	100.00

fm007: Pre-receding Total Salary

Mean	SD	Min	Max	Obs
1,598.35	1,733.69	0.00	7,000.00	101

fm007_max: Max Bracket of FM007

Mean	SD	Min	Max	Obs
1,000.00	774.60	500.00	2,000.00	6

fm007_min: Min Bracket of FM007

Mean	SD	Min	Max	Obs
1,500.00	866.03	500.00	2,000.00	3

fm008: Did You Receive Any Payments from Leaving the Job

	Freq.	%
1 Yes	22	42.31
2 No	30	57.69
Total	52	100.00

fm009: How Much Was The Compensation

Mean	SD	Min	Max	Obs

46,832.25	90,625.63	145.00	300,000.00	20

fm009_max: Max Bracket of FM009

Mean	SD	Min	Max	Obs
500.00	.	500.00	500.00	1

fm009_min: Min Bracket of FM009

Mean	SD	Min	Max	Obs
5,000.00	.	5,000.00	5,000.00	1

fm011: Type of Retirement

	Freq.	%
1 Formal Retirement	483	86.40
2 Early Retirement	40	7.16
3 Internal Retirement First Then Formal Retirement	25	4.47
4 Internal Retirement but Not Yet Formal Retirement	11	1.97
Total	559	100.00

fm012: Did You Retire as A Worker or A Cadre

	Freq.	%
1 Worker	454	81.22
2 Cadre	105	18.78
Total	559	100.00

fm014_1: When Did You Process Retirement(Year)

Mean	SD	Min	Max	Obs
2,008.13	11.97	1,900.00	2,018.00	957

fm014_2: When Did You Process Retirement(Month)

Mean	SD	Min	Max	Obs
6.03	3.97	0.00	12.00	956

fm015: Reason You Processed Early Retirement

	Freq.	%
1 I Have 30 Years Job Experience Which Is Enough for Early Retirement	1	2.50
2 My Work Unit Belonged to the Category of High-risk and Hard Manual Labor	7	17.50
3 My Work Unit Was Restructuring/Bankrupt	8	20.00
4 Due to Poor Health	11	27.50
5 Due to Family Reason	4	10.00
6 Other	9	22.50

Total	40	100.00

fm016: Pre-retirement Total Salary

Mean	SD	Min	Max	Obs
1,738.37	2,784.86	0.00	45,000.00	860

fm016_max: Max Bracket of FM016

Mean	SD	Min	Max	Obs
946.28	934.97	500.00	5,000.00	121

fm016_min: Min Bracket of FM016

Mean	SD	Min	Max	Obs
1,500.00	1,203.63	500.00	5,000.00	40

fm025_1: When Did You Take Internal Retirement(Year)

Mean	SD	Min	Max	Obs
2,003.43	17.94	1,900.00	2,018.00	42

fm025_2: When Did You Take Internal Retirement(Month)

Mean	SD	Min	Max	Obs
5.08	3.94	0.00	12.00	40

fm026: Reason You Processed Internal Retirement

	Freq.	%
1 5 Years Less Than the Legal Retirement Age	5	13.89
2 My Work Unit Was Already Restructuring/Bankrupt	11	30.56
3 Due to Poor Health	6	16.67
4 Due to Family Reason	2	5.56
5 Other	12	33.33
Total	36	100.00

fm027: Pre-internal Retirement Total Salary

Mean	SD	Min	Max	Obs
1,431.00	1,039.90	200.00	4,000.00	30

fm027_max: Max Bracket of FM027

Mean	SD	Min	Max	Obs
2,100.00	1,387.44	500.00	3,500.00	5

fm027_min: Min Bracket of FM027

Mean	SD	Min	Max	Obs
1,875.00	1,181.45	1,000.00	3,500.00	4

fm028: Total Salary When Internal Retirement Processed

Mean	SD	Min	Max	Obs
1,182.06	992.43	0.00	4,500.00	33

fm028_max: Max Bracket of FM028

Mean	SD	Min	Max	Obs
2,250.00	1,767.77	1,000.00	3,500.00	2

fm028_min: Min Bracket of FM028

Mean	SD	Min	Max	Obs
3,500.00	.	3,500.00	3,500.00	1

fm030_1: When Did You Process Formal Retirement(Year)

Mean	SD	Min	Max	Obs
2,012.46	4.84	2,000.00	2,018.00	28

fm030_2: When Did You Process Formal Retirement(Month)

Mean	SD	Min	Max	Obs
5.79	3.17	0.00	12.00	28

fm036: Years of Eligible Work for Pension Calculation When Formal Retirement Processed

Mean	SD	Min	Max	Obs
25.77	12.83	0.00	50.00	551

fm037_1: When Are You Going to Process Formal Retirement(Year)

Mean	SD	Min	Max	Obs
2,010.27	36.67	1,900.00	2,026.00	11

fm037_2: When Are You Going to Process Formal Retirement(Month)

Mean	SD	Min	Max	Obs
6.09	3.70	0.00	12.00	11

fm037_w2: Have Proceeded with Formal Retirement or Not

	Freq.	%
1 Yes	3	50.00
2 No	3	50.00
Total	6	100.00

fm040: Years of Eligible Work for Pension Calculation at The Time of Retirement

Mean	SD	Min	Max	Obs
29.45	16.56	0.00	45.00	11

fm041: Years of Eligible Work Currently Have

Mean	SD	Min	Max	Obs
26.45	14.53	0.00	41.00	11

fm042: Did You Have A Spouse When Processing You Retirement

	Freq.	%
1 Yes	573	93.32
2 No	41	6.68
Total	614	100.00

fm043: Health Status at The Time of Your Retirement

	Freq.	%
1 Excellent	40	6.51
2 Very Good	165	26.87
3 Good	153	24.92
4 Fair	211	34.36
5 Poor	45	7.33
Total	614	100.00

fm044: Had Your Spouse Already Processed Retirement When You Processed Retirement

	Freq.	%
1 Yes	204	35.60
2 No	369	64.40
Total	573	100.00

fm045: What Kind of Work Was Your Spouse Engaged in When You Processed Retirement

	Freq.	%
1 Employed	250	43.63
2 Self Employed	53	9.25
3 Unemployed and Still Searching for a Job	8	1.40
4 Unemployed and Not Searching for a Job	165	28.80

	Freq.	%
5 Farming	97	16.93
Total	573	100.00

fm046: How Was Your Spouse's Health at The Time of Your Retirement

	Freq.	%
1 Excellent	23	4.01
2 Very Good	154	26.88
3 Good	130	22.69
4 Fair	214	37.35
5 Poor	52	9.08
Total	573	100.00

fm047: Was Your Father Alive When You Processed Retirement

	Freq.	%
1 Yes	144	23.45
2 No	470	76.55
Total	614	100.00

fm048: How Was Your Father's Health at The Time of Your Retirement

	Freq.	%
1 Excellent	4	2.78
2 Very Good	27	18.75
3 Good	28	19.44
4 Fair	50	34.72
5 Poor	35	24.31
Total	144	100.00

fm049: Was Your Mother Alive When You Processed Retirement

	Freq.	%
1 Yes	243	39.58
2 No	371	60.42
Total	614	100.00

fm050: How Was Your Mother's Health at The Time of Your Retirement

	Freq.	%
1 Excellent	6	2.47
2 Very Good	44	18.11
3 Good	38	15.64
4 Fair	94	38.68
5 Poor	61	25.10
Total	243	100.00

fm051: How Many Grandchildren Below Age 6 Did You Have at The Time of Your Retirement

Mean	SD	Min	Max	Obs
0.80	1.20	0.00	8.00	614

fm052: Did You Work After You Processed Retirement

	Freq.	%
1 Yes	290	48.49
2 No	308	51.51
Total	598	100.00

fm053: How Long Did You Start to Work Again After Retirement

Mean	SD	Min	Max	Obs
0.83	2.65	0.00	20.00	306

fm054: Engaged in Recreational Work With Insignificant Income or Not

	Freq.	%
1 Yes	168	2.35
2 No	6,980	97.65
Total	7,148	100.00

fm056_1: When Did You Start This Work (Year)

Mean	SD	Min	Max	Obs
2,008.00	16.05	1,900.00	2,018.00	168

fm056_2: When Did You Start This Work (Month)

Mean	SD	Min	Max	Obs
3.65	3.51	0.00	12.00	168

fm057: How Many Days Do You Participate in Recreational Work per Week

Mean	SD	Min	Max	Obs
4.13	2.67	0.00	7.00	168

fm058: How Many Hours Do You Participate in Recreational Work per Day

Mean	SD	Min	Max	Obs
13.91	19.14	0.00	84.00	168

fm059: Monthly Income of Recreational Work

Mean	SD	Min	Max	Obs

773.94	2,945.05	0.00	35,000.00	162

fm059_max: Max Bracket of FM059

Mean	SD	Min	Max	Obs
1,750.00	1,993.74	500.00	5,000.00	6

fm059_min: Min Bracket of FM059

Mean	SD	Min	Max	Obs
4,250.00	1,060.66	3,500.00	5,000.00	2

xrtype: Respondent Type

	Freq.	%
1 RE Interview	19,418	98.44
2 New Interview	307	1.56
Total	19,725	100.00

xf1: Ever Worked, but Currently Not Working

	Freq.	%
1 Currently Not Working	7,093	35.97
2 Never Worked	56	0.28
3 Working	12,569	63.74
Total	19,718	100.00

xf11: Employed or Self-Employed

	Freq.	%
1 Employed	4,843	75.30
2 Self-employed Nonfarm	1,589	24.70
Total	6,432	100.00

xf4: Self-Employed Farm Work or Not

	Freq.	%
1 Yes	8,970	100.00
Total	8,970	100.00

xf5: Only Participating in Self-Employed Farm Work or Having Other Jobs

	Freq.	%
1 Yes	6,137	48.83
2 No	6,432	51.17
Total	12,569	100.00

xf7: Type of the Main Job

	Freq.	%
1 Nonfarm Employed	4,178	64.96
2 Nonfarm Self-employed	1,325	20.60
3 Unpaid Family Business	264	4.10
4 Farm Employed	665	10.34
Total	6,432	100.00

xzf1: Updated ZF1

	Freq.	%
1 Currently Not Working	5,036	27.36
2 Never Worked	204	1.11
3 Working	13,169	71.54
Total	18,409	100.00

xzf11: Updated ZF11

	Freq.	%
1 Employed	4,442	68.45
2 Self-employed Nonfarm	2,047	31.55
Total	6,489	100.00

xzf13: Updated ZF13

	Freq.	%
0 No	19,411	100.00
Total	19,411	100.00

xzf17: Updated ZF17

	Freq.	%
0 No	36	4.30
1 Yes	802	95.70
Total	838	100.00

xzf18: Updated ZF18

	Freq.	%
0 No	9	21.95
1 Yes	32	78.05
Total	41	100.00

xzf19: Updated ZF19

	Freq.	%
0 No	25	16.34
1 Yes	128	83.66
Total	153	100.00

xzf20: Updated ZF20

	Freq.	%
0 No	9	60.00
1 Yes	6	40.00
Total	15	100.00

xzf21: Updated ZF21

Mean	SD	Min	Max	Obs
1.00	0.00	1.00	1.00	7,671

xzf4: Updated ZF4

	Freq.	%
1 Yes	9,435	100.00
Total	9,435	100.00

xzf5: Updated ZF5

	Freq.	%
1 Yes	6,600	49.92
2 No	6,620	50.08
Total	13,220	100.00

xzf7: Updated ZF7

	Freq.	%
1 Nonfarm Employed	4,336	66.82
2 Nonfarm Self-employed	1,519	23.41
3 Unpaid Family Business	519	8.00
4 Farm Employed	115	1.77
Total	6,489	100.00

zf1: (Preloaded from the LastIW) Ever Worked, but Currently Not Working

	Freq.	%
1 Currently Not Working	5,411	29.42
2 Never Worked	206	1.12
3 Working	12,777	69.46
Total	18,394	100.00

zf11: (Preloaded from the LastIW) Employed or Self-Employed

	Freq.	%
1 Employed	4,020	67.97
2 Self-employed Nonfarm	1,894	32.03
Total	5,914	100.00

zf13: (Preloaded from the LastIW) Missing Work Status

	Freq.	%
1 Yes	95	100.00
Total	95	100.00

zf17: (Preloaded from the LastIW) Retirement Processed

	Freq.	%
1 Yes	1,837	100.00
Total	1,837	100.00

zf18: (Preloaded from the LastIW) Internal Retirement Processed

	Freq.	%
1 Yes	99	100.00
Total	99	100.00

zf19: (Preloaded from the LastIW) Position Receding Processed

	Freq.	%
1 Yes	317	100.00
Total	317	100.00

zf20: (Preloaded from the LastIW) Internal Retirement Processed, but Retirement Not

	Freq.	%
1 Yes	17	100.00
Total	17	100.00

zf21: (Preloaded from the LastIW) No (Internal) Retirement or Receding Processed

	Freq.	%
1 Yes	17,460	100.00
Total	17,460	100.00

zf25_1: Preloaded LastIW Retirement Information

	Freq.	%
1 Missing Address of Company	1,983	100.00
Total	1,983	100.00

zf25_11: Preloaded LastIW Retirement Information

	Freq.	%
1 Missing Salary at Internal Retirement	1	100.00

Total	1	100.00

zf25_2: Preloaded LastIW Retirement Information

	Freq.	%
1 Missing Time of Retirement	87	100.00
Total	87	100.00

zf25_3: Preloaded LastIW Retirement Information

	Freq.	%
1 Missing Salary	80	100.00
Total	80	100.00

zf25_4: Preloaded LastIW Retirement Information

	Freq.	%
1 Formal Retirement or Early Retirement	1,837	100.00
Total	1,837	100.00

zf25_5: Preloaded LastIW Retirement Information

	Freq.	%
1 Missing Time in Formal Retirement or Early Retirement	456	100.00
Total	456	100.00

zf25_6: Preloaded LastIW Retirement Information

	Freq.	%
1 Missing Salary in Formal Retirement or Early Retirement	417	100.00
Total	417	100.00

zf25_8: Preloaded LastIW Retirement Information

	Freq.	%
1 Completed Internal Retirement	99	100.00
Total	99	100.00

zf25_9: Preloaded LastIW Retirement Information

	Freq.	%
1 Missing Time in Internal Retirement	8	100.00
Total	8	100.00

zf4: (Preloaded from the LastIW) Self-Employed Farm Work or Not

	Freq.	%

	Freq.	%
1 Yes	8,696	100.00
Total	8,696	100.00

zf5: (Preloaded from the LastIW) Only in Self-Employed Farm Work or Having Other Jobs

	Freq.	%
1 Yes	5,911	47.31
2 No	6,582	52.69
Total	12,493	100.00

zf7: (Preloaded from the LastIW) Type of the Main Job

	Freq.	%
1 Nonfarm Employed	4,013	67.86
2 Nonfarm Self-employed	1,399	23.66
3 Unpaid Family Business	495	8.37
4 Farm Employed	7	0.12
Total	5,914	100.00

versionID: Version ID

A String Variable	
Obs:	19,725

This page intentionally left blank

MODULE FN

Pension

ID: Individual ID

A String Variable	
Obs:	19,718

householdID: Household ID

A String Variable	
Obs:	19,718

communityID: Community ID

A String Variable	
Obs:	19,718

fn002_w4: Receive/Participate Government/Institutions/Firm Pension

	Freq.	%
1 Yes	3,878	19.67
2 No	15,835	80.33
Total	19,713	100.00

fn002_w4_a_s1: Government Pension

	Freq.	%
0 No	3,633	93.68
1 Government Pension	245	6.32
Total	3,878	100.00

fn002_w4_a_s2: Institutions Pension

	Freq.	%
0 No	3,295	84.97
2 Institutions Pension	583	15.03
Total	3,878	100.00

fn002_w4_a_s3: Firm Pension

	Freq.	%
0 No	946	24.39
3 Firm Pension	2,932	75.61
Total	3,878	100.00

fn002_w4_a_s4: Don't Know the Type

	Freq.	%
0 No	3,752	96.75
4 Don't Know the Type	126	3.25
Total	3,878	100.00

fn002_w4_b_1_: Receive Government Pension

	Freq.	%
1 Yes	179	73.06
2 No	66	26.94
Total	245	100.00

fn002_w4_b_2_: Receive Institutions Pension

	Freq.	%
1 Yes	422	72.38
2 No	161	27.62
Total	583	100.00

fn002_w4_b_3_: Receive Firm Pension

	Freq.	%
1 Yes	2,028	69.17
2 No	904	30.83
Total	2,932	100.00

fn002_w4_b_4_: Receive Government/Institutions/Firm Pension

	Freq.	%
1 Yes	74	58.73
2 No	52	41.27
Total	126	100.00

fn003_w2_1_1_: Year of Receive Government Pension

Mean	SD	Min	Max	Obs
2,004.43	8.85	1,967.00	2,018.00	178

fn003_w2_1_2_: Year of Receive Institutions Pension

Mean	SD	Min	Max	Obs
2,004.98	8.97	1,969.00	2,018.00	422

fn003_w2_1_3_: Year of Receive Firm Pension

Mean	SD	Min	Max	Obs
2,006.09	9.00	1,953.00	2,018.00	2,024

fn003_w2_1_4_: Year of Receive Government/Institutions/Firm Pension

Mean	SD	Min	Max	Obs
2,005.89	9.02	1,981.00	2,018.00	73

fn005_w2_1_: Monthly Pension of Government Pension

Mean	SD	Min	Max	Obs
3,970.53	1,868.84	0.00	10,700.00	179

fn005_w2_2_: Monthly Pension of Institutions Pension

Mean	SD	Min	Max	Obs
3,805.70	1,744.01	68.00	11,000.00	422

fn005_w2_3_: Monthly Pension of Firm Pension

Mean	SD	Min	Max	Obs
2,496.73	1,152.12	0.00	23,000.00	2,019

fn005_w2_4_: Monthly Pension of Government/Institutions/Firm Pension

Mean	SD	Min	Max	Obs
1,914.27	1,532.90	50.00	9,800.00	74

fn005_w2_1__min: Min Bracket of fn005_w2_1_

No Observations

fn005_w2_1__max: Max Bracket of fn005_w2_1_

No Observations

fn005_w2_3__min: Min Bracket of fn005_w2_3_

Mean	SD	Min	Max	Obs
2,750.00	1,443.38	1,500.00	4,000.00	4

fn005_w2_3__max: Max Bracket of fn005_w2_3_

Mean	SD	Min	Max	Obs
2,000.00	1,000.00	1,500.00	4,000.00	6

fn006_w4_1_: Address of Receive Pension

	Freq.	%
1 Current Residence	111	62.01
2 Other Town/Village	34	18.99
3 Other Place	28	15.64
997 Don't Know	6	3.35
Total	179	100.00

fn006_w4_2_: Address of Receive Pension

	Freq.	%
1 Current Residence	240	56.87
2 Other Town/Village	92	21.80
3 Other Place	80	18.96
997 Don't Know	10	2.37
Total	422	100.00

fn006_w4_3_: Address of Receive Pension

	Freq.	%
1 Current Residence	1,308	64.50
2 Other Town/Village	389	19.18
3 Other Place	272	13.41
997 Don't Know	59	2.91
Total	2,028	100.00

fn006_w4_4_: Address of Receive Pension

	Freq.	%
1 Current Residence	39	52.70
2 Other Town/Village	19	25.68
3 Other Place	9	12.16
997 Don't Know	7	9.46
Total	74	100.00

fn007_w4_1_: Address of Participate Pension

	Freq.	%
1 Current Residence	38	57.58

	Freq.	%
2 Other Town/Village	16	24.24
3 Other Place	10	15.15
997 Don't Know	2	3.03
Total	66	100.00

fn007_w4_2_: Address of Participate Pension

	Freq.	%
1 Current Residence	68	42.24
2 Other Town/Village	64	39.75
3 Other Place	23	14.29
997 Don't Know	6	3.73
Total	161	100.00

fn007_w4_3_: Address of Participate Pension

	Freq.	%
1 Current Residence	431	47.68
2 Other Town/Village	225	24.89
3 Other Place	216	23.89
997 Don't Know	32	3.54
Total	904	100.00

fn007_w4_4_: Address of Participate Pension

	Freq.	%
1 Current Residence	27	51.92
2 Other Town/Village	10	19.23
3 Other Place	3	5.77
997 Don't Know	12	23.08
Total	52	100.00

fn008_w4_1_: Participate Pension by Yourself or through Company

	Freq.	%
1 Myself	3	4.55
2 Through Company	63	95.45
Total	66	100.00

fn008_w4_2_: Participate Pension by Yourself or through Company

	Freq.	%
1 Myself	13	8.07
2 Through Company	148	91.93
Total	161	100.00

fn008_w4_3_: Participate Pension by Yourself or through Company

	Freq.	%
1 Myself	279	30.86

	Freq.	%
2 Through Company	625	69.14
Total	904	100.00

fn008_w4_4_: Participate Pension by Yourself or through Company

	Freq.	%
1 Myself	30	57.69
2 Through Company	22	42.31
Total	52	100.00

fn104_w4: 4050 Subsidy

	Freq.	%
1 Yes	31	9.54
2 No	286	88.00
997 Don't Know	8	2.46
Total	325	100.00

fn105_w4_1: Year of Receive 4050 Subsidy

Mean	SD	Min	Max	Obs
2,014.13	4.27	1,995.00	2,018.00	31

fn105_w4_2: Month of Receive 4050 Subsidy

Mean	SD	Min	Max	Obs
4.65	4.56	0.00	12.00	31

fn106_w4_1: Subsidy Ratio of Pension Insurance

Mean	SD	Min	Max	Obs
29.30	27.75	-1.00	70.00	20

fn106_w4_2: Subsidy Yuan/Yue of Pension Insurance

Mean	SD	Min	Max	Obs
85.92	106.35	-1.00	300.00	18

fn107_w4_1: Subsidy Ratio of Medical Insurance

Mean	SD	Min	Max	Obs
14.26	25.47	-1.00	70.00	19

fn107_w4_2: Subsidy Yuan/Yue of Medical Insurance

Mean	SD	Min	Max	Obs

74.99	335.42	-1.00	1,500.00	20

fn012_w2_1_: Need to Pay the Premium by Yourself/Work Unit

	Freq.	%
1 Yes	61	92.42
2 No	5	7.58
Total	66	100.00

fn012_w2_2_: Need to Pay the Premium by Yourself/Work Unit

	Freq.	%
1 Yes	150	93.17
2 No	11	6.83
Total	161	100.00

fn012_w2_3_: Need to Pay the Premium by Yourself/Work Unit

	Freq.	%
1 Yes	834	92.26
2 No	70	7.74
Total	904	100.00

fn012_w2_4_: Need to Pay the Premium by Yourself/Work Unit

	Freq.	%
1 Yes	35	67.31
2 No	17	32.69
Total	52	100.00

fn013_w2_1_: Yuan/Yue or Ratio of Salary

	Freq.	%
1 Yuan/Yue	55	90.16
2 Ratio of Salary	6	9.84
Total	61	100.00

fn013_w2_1_1_: Yuan/Yue

Mean	SD	Min	Max	Obs
354.44	423.28	-1.00	2,000.00	55

fn013_w2_2_1_: Ratio of Salary

Mean	SD	Min	Max	Obs
6.67	3.50	0.00	10.00	6

fn013_w2_2_: Yuan/Yue or Ratio of Salary

	Freq.	%
1 Yuan/Yue	121	80.67
2 Ratio of Salary	29	19.33
Total	150	100.00

fn013_w2_1_2_: Yuan/Yue

Mean	SD	Min	Max	Obs
312.78	430.57	-1.00	1,932.00	123

fn013_w2_2_2_: Ratio of Salary

Mean	SD	Min	Max	Obs
9.29	6.71	-1.00	28.00	31

fn013_w2_3_: Yuan/Yue or Ratio of Salary

	Freq.	%
1 Yuan/Yue	750	89.93
2 Ratio of Salary	84	10.07
Total	834	100.00

fn013_w2_1_3_: Yuan/Yue

Mean	SD	Min	Max	Obs
1,336.97	23,460.47	-1.00	643,000.00	754

fn013_w2_2_3_: Ratio of Salary

Mean	SD	Min	Max	Obs
17.36	19.76	-1.00	100.00	92

fn013_w2_4_: Yuan/Yue or Ratio of Salary

	Freq.	%
1 Yuan/Yue	33	94.29
2 Ratio of Salary	2	5.71
Total	35	100.00

fn013_w2_1_4_: Yuan/Yue

Mean	SD	Min	Max	Obs
290.73	371.67	-1.00	1,340.00	33

fn013_w2_2_4_: Ratio of Salary

Mean	SD	Min	Max	Obs
8.40	12.99	-1.00	25.00	5

fn013_w2_1__min: Min Bracket of fn013_w2_1_

Mean	SD	Min	Max	Obs
462.50	266.93	50.00	750.00	8

fn013_w2_1__max: Max Bracket of fn013_w2_1_

Mean	SD	Min	Max	Obs
437.50	242.81	200.00	750.00	4

fn013_w2_2__min: Min Bracket of fn013_w2_2_

Mean	SD	Min	Max	Obs
433.33	213.08	50.00	750.00	30

fn013_w2_2__max: Max Bracket of fn013_w2_2_

Mean	SD	Min	Max	Obs
424.00	186.59	200.00	750.00	25

fn013_w2_3__min: Min Bracket of fn013_w2_3_

Mean	SD	Min	Max	Obs
363.70	210.53	50.00	750.00	73

fn013_w2_3__max: Max Bracket of fn013_w2_3_

Mean	SD	Min	Max	Obs
405.19	209.41	50.00	750.00	77

fn013_w2_4__min: Min Bracket of fn013_w2_4_

Mean	SD	Min	Max	Obs
400.00	141.42	300.00	500.00	2

fn013_w2_4__max: Max Bracket of fn013_w2_4_

Mean	SD	Min	Max	Obs
500.00	0.00	500.00	500.00	2

fn014_w2_1_: Yuan/Month or Ratio of Salary

	Freq.	%
1 Yuan/Month	54	91.53
2 Ratio of Salary	5	8.47
Total	59	100.00

fn014_w2_1_1_: Yuan/Month

Mean	SD	Min	Max	Obs
119.49	317.94	-1.00	1,698.00	54

fn014_w2_2_1_: Ratio of Salary

Mean	SD	Min	Max	Obs
12.67	9.75	-1.00	22.00	6

fn014_w2_2_: Yuan/Month or Ratio of Salary

	Freq.	%
1 Yuan/Month	114	82.61
2 Ratio of Salary	24	17.39
Total	138	100.00

fn014_w2_1_2_: Yuan/Month

Mean	SD	Min	Max	Obs
252.72	469.85	-1.00	2,000.00	115

fn014_w2_2_2_: Ratio of Salary

Mean	SD	Min	Max	Obs
17.18	19.53	-1.00	80.00	28

fn014_w2_3_: Yuan/Month or Ratio of Salary

	Freq.	%
1 Yuan/Month	505	86.77
2 Ratio of Salary	77	13.23
Total	582	100.00

fn014_w2_1_3_: Yuan/Month

Mean	SD	Min	Max	Obs
297.23	470.21	-1.00	4,000.00	506

fn014_w2_2_3_: Ratio of Salary

Mean	SD	Min	Max	Obs
24.40	20.94	-1.00	80.00	79

fn014_w2_4_: Yuan/Month or Ratio of Salary

	Freq.	%
1 Yuan/Month	18	90.00
2 Ratio of Salary	2	10.00
Total	20	100.00

fn014_w2_1_4_: Yuan/Month

Mean	SD	Min	Max	Obs
169.66	262.62	-1.00	700.00	18

fn014_w2_2_4_: Ratio of Salary

Mean	SD	Min	Max	Obs
52.50	38.89	25.00	80.00	2

fn014_w2_1__min: Min Bracket of fn014_w2_1_

Mean	SD	Min	Max	Obs
347.06	226.71	50.00	750.00	17

fn014_w2_1__max: Max Bracket of fn014_w2_1_

Mean	SD	Min	Max	Obs
376.92	238.59	50.00	750.00	13

fn014_w2_2__min: Min Bracket of fn014_w2_2_

Mean	SD	Min	Max	Obs
460.61	283.33	50.00	750.00	33

fn014_w2_2__max: Max Bracket of fn014_w2_2_

Mean	SD	Min	Max	Obs
339.47	220.21	50.00	750.00	19

fn014_w2_3__min: Min Bracket of fn014_w2_3_

Mean	SD	Min	Max	Obs
444.29	252.33	50.00	750.00	105

fn014_w2_3__max: Max Bracket of fn014_w2_3_

Mean	SD	Min	Max	Obs
457.59	241.67	50.00	750.00	79

fn014_w2_4__min: Min Bracket of fn014_w2_4_

Mean	SD	Min	Max	Obs
283.33	404.15	50.00	750.00	3

fn014_w2_4__max: Max Bracket of fn014_w2_4_

Mean	SD	Min	Max	Obs
750.00	.	750.00	750.00	1

fn017_w2_1_: Years of Included in This Program at Local Province

Mean	SD	Min	Max	Obs
26.23	11.76	0.00	47.00	66

fn017_w3_1_: Years of Included in This Program in Other Province

Mean	SD	Min	Max	Obs
4.03	9.84	0.00	44.00	66

fn017_w2_2_: Years of Included in This Program at Local Province

Mean	SD	Min	Max	Obs
26.28	9.93	0.00	43.00	161

fn017_w3_2_: Years of Included in This Program in Other Province

Mean	SD	Min	Max	Obs
3.06	8.59	0.00	41.00	161

fn017_w2_3_: Years of Included in This Program at Local Province

Mean	SD	Min	Max	Obs
19.00	11.18	0.00	45.00	904

fn017_w3_3_: Years of Included in This Program in Other Province

Mean	SD	Min	Max	Obs
2.78	7.79	0.00	60.00	904

fn017_w2_4_: Years of Included in This Program at Local Province

Mean	SD	Min	Max	Obs
12.04	12.16	0.00	42.00	52

fn017_w3_4_: Years of Included in This Program in Other Province

Mean	SD	Min	Max	Obs
3.39	8.35	0.00	37.00	52

fn019_w4_1_: Receive Pension in the Future

	Freq.	%
1 Yes	65	98.48
2 No	1	1.52
Total	66	100.00

fn020_w4_1_: Age of Receive Pension

Mean	SD	Min	Max	Obs
59.09	3.02	45.00	63.00	65

fn019_w4_2_: Receive Pension in the Future

	Freq.	%
1 Yes	161	100.00
Total	161	100.00

fn020_w4_2_: Age of Receive Pension

Mean	SD	Min	Max	Obs
58.83	3.07	50.00	65.00	161

fn019_w4_3_: Receive Pension in the Future

	Freq.	%
1 Yes	867	95.91
2 No	11	1.22
997 Don't Know	26	2.88
Total	904	100.00

fn020_w4_3_: Age of Receive Pension

Mean	SD	Min	Max	Obs
58.02	4.07	45.00	70.00	867

fn019_w4_4_: Receive Pension in the Future

	Freq.	%
1 Yes	33	63.46
2 No	8	15.38
997 Don't Know	11	21.15
Total	52	100.00

fn020_w4_4_: Age of Receive Pension

Mean	SD	Min	Max	Obs
58.03	3.34	50.00	63.00	33

fn021_w2_1_: Yuan/Month or Percentage of Final Pay

	Freq.	%
1 Yuan/Month	63	96.92
2 Percentage of Final Pay	2	3.08
Total	65	100.00

fn021_w2_1_1_: Yuan/Month

Mean	SD	Min	Max	Obs
3,743.53	1,886.16	0.00	7,500.00	51

fn021_w2_2_: Yuan/Month or Percentage of Final Pay

	Freq.	%
1 Yuan/Month	153	95.03
2 Percentage of Final Pay	8	4.97
Total	161	100.00

fn021_w2_1_2_: Yuan/Month

Mean	SD	Min	Max	Obs
4,157.69	1,772.21	0.00	10,000.00	118

fn021_w2_3_: Yuan/Month or Percentage of Final Pay

	Freq.	%
1 Yuan/Month	853	98.39
2 Percentage of Final Pay	14	1.61
Total	867	100.00

fn021_w2_1_3_: Yuan/Month

Mean	SD	Min	Max	Obs
2,241.28	1,487.25	0.00	10,000.00	566

fn021_w2_4_: Yuan/Month or Percentage of Final Pay

	Freq.	%
1 Yuan/Month	33	100.00
Total	33	100.00

fn021_w2_1_4_: Yuan/Month

Mean	SD	Min	Max	Obs
1,961.40	1,919.14	20.00	6,000.00	25

fn021_w2_2_1_: Percentage of Final Pay

Mean	SD	Min	Max	Obs
82.50	3.54	80.00	85.00	2

fn021_w2_2_2_: Percentage of Final Pay

Mean	SD	Min	Max	Obs
74.13	27.47	8.00	90.00	8

fn021_w2_2_3_: Percentage of Final Pay

Mean	SD	Min	Max	Obs
52.76	36.59	-1.00	100.00	17

fn021_w2_1__min: Min Bracket of fn021_w2_1_

Mean	SD	Min	Max	Obs
3,590.91	1,136.18	1,500.00	4,500.00	11

fn021_w2_1__max: Max Bracket of fn021_w2_1_

Mean	SD	Min	Max	Obs
3,900.00	821.58	3,000.00	4,500.00	5

fn021_w2_2__min: Min Bracket of fn021_w2_2_

Mean	SD	Min	Max	Obs
2,666.67	1,090.14	1,000.00	4,500.00	24

fn021_w2_2__max: Max Bracket of fn021_w2_2_

Mean	SD	Min	Max	Obs
2,657.89	1,236.62	1,000.00	4,500.00	19

fn021_w2_3__min: Min Bracket of fn021_w2_3_

Mean	SD	Min	Max	Obs
2,324.02	1,162.88	1,000.00	4,500.00	179

fn021_w2_3__max: Max Bracket of fn021_w2_3_

Mean	SD	Min	Max	Obs
2,091.89	1,026.65	1,000.00	4,500.00	185

fn021_w2_4__min: Min Bracket of fn021_w2_4_

Mean	SD	Min	Max	Obs
2,100.00	1,596.87	1,000.00	4,500.00	5

fn021_w2_4__max: Max Bracket of fn021_w2_4_

Mean	SD	Min	Max	Obs
2,750.00	1,917.03	1,000.00	4,500.00	6

fn014_w4_a_1_: Extra Payment

	Freq.	%
1 Yes	9	3.83
2 No	226	96.17
Total	235	100.00

fn014_w4_a_2_: Extra Payment

	Freq.	%
1 Yes	62	10.82
2 No	511	89.18
Total	573	100.00

fn014_w4_a_3_: Extra Payment

	Freq.	%
1 Yes	669	23.30
2 No	2,202	76.70
Total	2,871	100.00

fn014_w4_a_4_: Extra Payment

	Freq.	%
1 Yes	24	21.24
2 No	89	78.76
Total	113	100.00

fn014_w4_b_1_: Payment

	Freq.	%
1 Yuan	7	77.78
2 Ten Thousand Yuan	2	22.22
Total	9	100.00

fn014_w4_b_1_1_: Yuan

Mean	SD	Min	Max	Obs
8,028.14	12,807.83	-1.00	36,000.00	7

fn014_w4_b_2_1_: Ten Thousand Yuan

Mean	SD	Min	Max	Obs
1.65	0.64	1.20	2.10	2

fn014_w4_b_2_: Payment

	Freq.	%
1 Yuan	37	59.68
2 Ten Thousand Yuan	25	40.32
Total	62	100.00

fn014_w4_b_1_2_: Yuan

Mean	SD	Min	Max	Obs
14,411.32	19,808.33	-1.00	95,000.00	37

fn014_w4_b_2_2_: Ten Thousand Yuan

Mean	SD	Min	Max	Obs
2,122.89	7,736.10	0.68	35,000.00	25

fn014_w4_b_3_: Payment

	Freq.	%
1 Yuan	303	45.29
2 Ten Thousand Yuan	366	54.71
Total	669	100.00

fn014_w4_b_1_3_: Yuan

Mean	SD	Min	Max	Obs
14,454.86	17,417.66	-1.00	94,800.00	303

fn014_w4_b_2_3_: Ten Thousand Yuan

Mean	SD	Min	Max	Obs
2,910.71	11,666.97	-1.00	94,000.00	369

fn014_w4_b_4_: Payment

	Freq.	%
1 Yuan	6	25.00
2 Ten Thousand Yuan	18	75.00
Total	24	100.00

fn014_w4_b_1_4_: Yuan

Mean	SD	Min	Max	Obs
18,316.67	24,946.86	1,500.00	68,000.00	6

fn014_w4_b_2_4_: Ten Thousand Yuan

Mean	SD	Min	Max	Obs
4,724.82	13,922.41	1.00	49,000.00	18

fn014_w4_b_1__min: Min Bracket of fn014_w4_b_1_

Mean	SD	Min	Max	Obs
9,000.00	1,414.21	8,000.00	10,000.00	2

fn014_w4_b_1__max: Max Bracket of fn014_w4_b_1_

Mean	SD	Min	Max	Obs
8,000.00	.	8,000.00	8,000.00	1

fn014_w4_b_2__min: Min Bracket of fn014_w4_b_2_

Mean	SD	Min	Max	Obs
5,500.00	3,535.53	3,000.00	8,000.00	2

fn014_w4_b_2__max: Max Bracket of fn014_w4_b_2_

Mean	SD	Min	Max	Obs
6,500.00	4,949.75	3,000.00	10,000.00	2

fn014_w4_b_3__min: Min Bracket of fn014_w4_b_3_

Mean	SD	Min	Max	Obs
6,062.50	3,732.18	1,000.00	10,000.00	16

fn014_w4_b_3__max: Max Bracket of fn014_w4_b_3_

Mean	SD	Min	Max	Obs
5,909.09	3,448.32	1,000.00	10,000.00	11

fn014_w4_c_1_1_: Year

Mean	SD	Min	Max	Obs
2,010.14	6.72	1,997.00	2,015.00	7

fn014_w4_c_2_1_: Month

Mean	SD	Min	Max	Obs
4.50	4.04	1.00	10.00	6

fn014_w4_c_1_2_: Year

Mean	SD	Min	Max	Obs
2,008.07	6.93	1,987.00	2,018.00	57

fn014_w4_c_2_2_: Month

Mean	SD	Min	Max	Obs
5.59	4.16	0.00	12.00	58

fn014_w4_c_1_3_: Year

Mean	SD	Min	Max	Obs
2,008.99	6.41	1,977.00	2,018.00	653

fn014_w4_c_2_3_: Month

Mean	SD	Min	Max	Obs
5.05	4.19	0.00	12.00	655

fn014_w4_c_1_4_: Year

Mean	SD	Min	Max	Obs
2,010.00	5.07	1,998.00	2,018.00	24

fn014_w4_c_2_4_: Month

Mean	SD	Min	Max	Obs
4.57	4.53	0.00	12.00	23

fn030_w4: Receive/Particate Supplemental Pension

	Freq.	%
1 Yes	126	0.64
2 No	19,587	99.36
Total	19,713	100.00

fn030_w4_a: Supplemental Pension

	Freq.	%
1 Government Supplemental Pension	17	13.49
2 Institution Supplemental Pension	40	31.75
3 Firm Supplemental Pension	61	48.41
4 Don't Know the Type	8	6.35
Total	126	100.00

fn031_w4: Currently Receiving the Supplemental Pension

	Freq.	%
1 Yes	47	37.30
2 No	79	62.70
Total	126	100.00

fn032_w4: Type of Supplemental Pension

	Freq.	%
1 Defined Benefit (DB) Retirement Pension	24	19.05
2 Defined Contribution (DC) Retirement Pension	57	45.24
997 Don't Know the Type	45	35.71
Total	126	100.00

fn034_w4: Address of Participate in This Pension

	Freq.	%
1 Currently Residence	32	40.51
2 Other Township/Village/Neighborhood	24	30.38
3 Other Place	21	26.58
997 Don't Know	2	2.53
Total	79	100.00

fn033_w2: Years of Included in This Pension

Mean	SD	Min	Max	Obs
8.29	8.20	0.00	40.00	72

fn035_w4_a: Pay the Premium by Yourself or by Work Unit

	Freq.	%
1 Yes	71	89.87
2 No	8	10.13

Total	79	100.00

fn035_w4_b: Unit Paid

	Freq.	%
1 Yuan/Month	52	73.24
2 Percent	19	26.76
Total	71	100.00

fn035_w4_b_1: Yuan/Month

Mean	SD	Min	Max	Obs
161.25	265.60	-1.00	1,100.00	53

fn035_w4_b_2: Percent

Mean	SD	Min	Max	Obs
12.67	20.23	-1.00	80.00	21

fn035_w4_b_min: Min Bracket of fn035_w4_b

Mean	SD	Min	Max	Obs
1,500.00	963.62	500.00	3,500.00	8

fn035_w4_b_max: Max Bracket of fn035_w4_b

Mean	SD	Min	Max	Obs
1,375.00	932.37	500.00	3,500.00	12

fn035_w4_c: You Paid

	Freq.	%
1 Yuan/Month	54	76.06
2 Percent	17	23.94
Total	71	100.00

fn035_w4_c_1: Yuan/Month

Mean	SD	Min	Max	Obs
152.19	223.30	-1.00	1,000.00	54

fn035_w4_c_2: Percent

Mean	SD	Min	Max	Obs
6.02	4.49	1.00	20.00	17

fn035_w4_c_min: Min Bracket of fn035_w4_c

Mean	SD	Min	Max	Obs
1,166.67	1,169.05	500.00	3,500.00	6

fn035_w4_c_max: Max Bracket of fn035_w4_c

Mean	SD	Min	Max	Obs
1,000.00	898.72	500.00	3,500.00	14

fn038_w2: Age of Receive Pension

Mean	SD	Min	Max	Obs
58.65	3.84	45.00	70.00	79

fn039_w4: Expect Benefits

	Freq.	%
1 Yuan/Month	77	97.47
2 Percent of Final Pay	2	2.53
Total	79	100.00

fn039_w4_1: Yuan/Month

Mean	SD	Min	Max	Obs
3,858.77	22,782.62	-1.00	200,000.00	77

fn039_w4_2: Percent of Final Pay

Mean	SD	Min	Max	Obs
10.67	18.50	-1.00	32.00	3

fn039_w4_min: Min Bracket of fn039_w4

Mean	SD	Min	Max	Obs
1,766.67	1,437.59	500.00	5,000.00	15

fn039_w4_max: Max Bracket of fn039_w4

Mean	SD	Min	Max	Obs
1,710.53	1,326.19	500.00	5,000.00	19

fn041_w2_1: Year of Receive Pension

Mean	SD	Min	Max	Obs
2,007.07	12.09	1,954.00	2,018.00	45

fn041_w2_2: Month of Receive Pension

Mean	SD	Min	Max	Obs
4.98	4.29	0.00	12.00	45

fn042_w2: Monthly Benefits

Mean	SD	Min	Max	Obs
6,449.13	31,384.37	-1.00	216,000.00	47

fn042_w2_min: Min Bracket of fn042_w2_min

Mean	SD	Min	Max	Obs
1,000.00	.	1,000.00	1,000.00	1

fn042_w2_max: Min Bracket of fn042_w2_max

Mean	SD	Min	Max	Obs
750.00	353.55	500.00	1,000.00	2

fn042_w4: Address of Receive Pension

	Freq.	%
1 Currently Residence	27	57.45
2 Other Township/Village/Neighborhood	8	17.02
3 Other Place	10	21.28
997 Don't Know	2	4.26
Total	47	100.00

fn037_w2: Checked Account Balance

	Freq.	%
1 Yes	15	14.71
2 No	87	85.29
Total	102	100.00

fn037_w2_1: Yuan

Mean	SD	Min	Max	Obs
17,587.36	21,159.89	0.00	65,000.00	14

fn037_w2_2: Year

Mean	SD	Min	Max	Obs
2,017.93	0.27	2,017.00	2,018.00	14

fn037_w2_3: Month

Mean	SD	Min	Max	Obs
5.21	2.08	2.00	7.00	14

fn058_w4: Residents/Newrural/Urban Residents Pension

	Freq.	%
1 Yes	13,127	66.59
2 No	6,586	33.41
Total	19,713	100.00

fn069_w4: Eligible to Receive or Participate in Social Endownment Insurance

	Freq.	%
1 Yes,	1,340	20.35
2 No	4,057	61.60
997 I Do Not Know	1,189	18.05
Total	6,586	100.00

fn069_w4_a: Reason of Not Eligible

	Freq.	%
1 Not Available in My Area	236	5.82
2 Have Participated in Government/Institution/Firm pension	3,034	74.78
3 Other	787	19.40
Total	4,057	100.00

fn057_w4_4: Old Enough to Receive Pension

	Freq.	%
1 Yes,	1,148	45.39
2 No	1,381	54.61
Total	2,529	100.00

fn057_w4_5: Reason of Not Receive

	Freq.	%
1 Did Not Pay the Insurance Premium	690	60.10
2 I am Not in My Hukou Place	8	0.70
3 Do Not Know Where to Receive	113	9.84
4 Poor Health, Cannot Go and Receive	8	0.70
5 Other	329	28.66
Total	1,148	100.00

fn057_w3_6: Reason of Not Pay the Premium

	Freq.	%
1 No Money	593	42.94
2 Future Payments Are Too Small	34	2.46
3 It Won't Make a Big Difference to My Life	21	1.52

4 It Is Not Convenient to Apply and to Pay	28	2.03
5 The Design of the Payments Is Not Reasonable	7	0.51
6 I Do Not Have the Local HuKou	12	0.87
7 I Have Already Participated in Other Pension Plans	221	16.00
8 Others, Please Specify	465	33.67
Total	1,381	100.00

fn058_w4_a: Which Pension

	Freq.	%
1 Social Endownment Insurance for Urban and Rural Residents	2,254	17.17
2 New Social Endowment Insurance for Rural Residents	9,674	73.70
3 Social Endowment Insurance for Non-working Urban Residents	434	3.31
4 I Do Not Know the Type	765	5.83
Total	13,127	100.00

fn058_w4_b: Currently Receiving the Pension

	Freq.	%
1 Yes,	6,999	53.32
2 No	6,128	46.68
Total	13,127	100.00

fn061_w4: Years of Particated or Contributed to the Pension

Mean	SD	Min	Max	Obs
7.42	4.80	0.00	100.00	6,127

fn059_w2: Address of Participate in the Pension

	Freq.	%
1 Currently Residence	5,038	82.21
2 Other Township/Village/Neighborhood	301	4.91
3 Other Place	778	12.70
997 Don't Know	11	0.18
Total	6,128	100.00

fn062_w4: Pay the Premium

	Freq.	%
1 Yes,	5,714	93.24
2 No	414	6.76
Total	6,128	100.00

fn062_w4_a: Government Subsidise

	Freq.	%
1 Yuan/Month	3,592	62.86
2 Yuan/Year	2,122	37.14
Total	5,714	100.00

fn062_w4_a_1: Yuan/Month

Mean	SD	Min	Max	Obs
57,941.44	2,353,708.36	-1.00	100000000.00	3,627

fn062_w4_a_2: Yuan/Year

Mean	SD	Min	Max	Obs
197.40	3,099.28	-1.00	99,999.00	2,143

fn062_w4_a_min: Min Bracket of fn062_w4_a

Mean	SD	Min	Max	Obs
18.10	9.23	5.00	30.00	105

fn062_w4_a_max: Max Bracket of fn062_w4_a

Mean	SD	Min	Max	Obs
11.15	8.04	5.00	30.00	148

fn062_w4_b: Premium You Paid

	Freq.	%
1 Yuan/Month	321	5.62
2 Yuan/Year	5,393	94.38
Total	5,714	100.00

fn062_w4_b_1: Yuan/Month

Mean	SD	Min	Max	Obs
328.00	1,828.38	-1.00	34,200.00	355

fn062_w4_b_2: Yuan/Year

Mean	SD	Min	Max	Obs
385.62	1,755.64	-1.00	60,000.00	5,397

fn062_w4_b_min: Min Bracket of fn062_w4_b

Mean	SD	Min	Max	Obs
93.53	106.08	10.00	500.00	34

fn062_w4_b_max: Max Bracket of fn062_w4_b

Mean	SD	Min	Max	Obs
41.05	92.17	10.00	500.00	133

fn065_w2: When do You Expect to Receive the Pension

	Freq.	%
1 Age	5,918	96.57
2 Year	210	3.43
Total	6,128	100.00

fn065_w2_1: Age

Mean	SD	Min	Max	Obs
60.06	2.02	50.00	100.00	5,927

fn065_w2_2: Year

Mean	SD	Min	Max	Obs
19.21	24.80	0.00	100.00	226

fn066_w2: Expect Benefits

	Freq.	%
1 Yuan/Month	6,054	98.79
2 Lump Sum Amount	74	1.21
Total	6,128	100.00

fn066_w2_1: Yuan/Month

Mean	SD	Min	Max	Obs
1,773.96	128,489.37	-1.00	9,999,999.00	6,057

fn066_w2_2: Lump Sum Amount

Mean	SD	Min	Max	Obs
933.84	2,340.76	-1.00	15,000.00	79

fn066_w2_min: Min Bracket of fn066_w2

Mean	SD	Min	Max	Obs
108.65	92.46	55.00	400.00	1,184

fn066_w2_max: Max Bracket of fn066_w2

Mean	SD	Min	Max	Obs
137.10	125.15	55.00	400.00	1,176

fn063_w4: Checked Account Balance

	Freq.	%
1 Yes	88	1.44
2 No	6,040	98.56
Total	6,128	100.00

fn063_w4_1: Yuan

Mean	SD	Min	Max	Obs
2,855.24	7,770.80	0.00	65,000.00	87

fn063_w4_2: Year

Mean	SD	Min	Max	Obs
2,017.31	2.08	2,000.00	2,018.00	89

fn063_w4_3: Month

Mean	SD	Min	Max	Obs
5.14	3.03	0.00	12.00	87

fn067_w2_1: Year of Receive Pension

Mean	SD	Min	Max	Obs
2,011.97	5.44	1,900.00	2,018.00	6,941

fn067_w2_2: Month of Receive Pension

Mean	SD	Min	Max	Obs
4.21	4.30	0.00	12.00	6,855

fn068_w2: Monthly Pension

Mean	SD	Min	Max	Obs
189.59	368.65	-1.00	8,640.00	6,999

fn068_w2_min: Min Bracket of fn068_w2

Mean	SD	Min	Max	Obs
79.31	14.17	55.00	100.00	144

fn068_w2_max: Max Bracket of fn068_w2

Mean	SD	Min	Max	Obs
78.91	15.12	55.00	100.00	138

fn070_w4: Address of Receive Pension

	Freq.	%
1 Currently Residence	5,176	73.95
2 Other Township/Village/Neighborhood	1,140	16.29
3 Other Place	596	8.52
997 Don't Know	87	1.24
Total	6,999	100.00

fn072_w3: Extra Payment When Particiated in the Pension

	Freq.	%
1 Yes	1,834	13.97
2 No	10,672	81.30
997 Don't Know	620	4.72
999 Refuse to Answer	1	0.01
Total	13,127	100.00

fn072_w3_1: Extra Payment

	Freq.	%
1 Yuan	1,479	80.64
2 Ten Thousand Yuan	355	19.36
Total	1,834	100.00

fn072_w3_1_1: Yuan

Mean	SD	Min	Max	Obs
4,048.54	9,433.77	-1.00	97,460.00	1,480

fn072_w3_1_2: Ten Thousand Yuan

Mean	SD	Min	Max	Obs
2,479.24	10,009.36	-1.00	97,000.00	357

fn072_w3_1_min: Min Bracket of fn072_w3_1

Mean	SD	Min	Max	Obs
4,148.15	3,230.90	1,000.00	10,000.00	27

fn072_w3_1_max: Max Bracket of fn072_w3_1

Mean	SD	Min	Max	Obs
2,925.00	2,525.64	1,000.00	10,000.00	40

fn072_w4_b_1: Year

Mean	SD	Min	Max	Obs

2,011.76	6.89	1,900.00	2,018.00	1,752

fn072_w4_b_2: Month

Mean	SD	Min	Max	Obs
4.69	4.23	0.00	12.00	1,758

fn103_w4: Participated in the Old Rural Pension

	Freq.	%
1 Yes	691	3.51
2 No	19,022	96.49
Total	19,713	100.00

fn079_w2_3: Particate in Land Expropriation Pension Insurance

	Freq.	%
1 Yes, But I don't Receive It	98	0.50
2 Yes, I Receive It	313	1.59
3 No	19,302	97.92
Total	19,713	100.00

fn079_w4: Address of Participate in the Pension

	Freq.	%
1 Currently Residence	83	84.69
2 Other Township/Village/Neighborhood	7	7.14
3 Other Place	8	8.16
Total	98	100.00

fn079_w2_4: Pay the Premium by Yourself

	Freq.	%
1 Yes	59	60.20
2 No	39	39.80
Total	98	100.00

fn079_w2_6: How Much Need to Pay

	Freq.	%
1 Yuan/Month	20	33.90
2 Yuan/Year	39	66.10
Total	59	100.00

fn079_w2_6_1: Yuan/Month

Mean	SD	Min	Max	Obs
423.40	339.95	-1.00	1,040.00	20

fn079_w2_6_2: Yuan/Year

Mean	SD	Min	Max	Obs
13,342.54	17,684.06	-1.00	80,000.00	39

fn079_w2_6_min: Min Bracket of fn079_w2_6

Mean	SD	Min	Max	Obs
750.00	353.55	500.00	1,000.00	2

fn079_w2_6_max: Max Bracket of fn079_w2_6

Mean	SD	Min	Max	Obs
533.33	450.92	100.00	1,000.00	3

fn079_w2_8: When Expect to Receive the Pension

	Freq.	%
1 Age	90	91.84
2 Year	8	8.16
Total	98	100.00

fn079_w2_8_1: Age

Mean	SD	Min	Max	Obs
59.49	5.23	50.00	90.00	90

fn079_w2_8_2: Year

Mean	SD	Min	Max	Obs
1.33	0.83	0.50	3.00	9

fn079_w2_9: Expect Benefits

	Freq.	%
1 Yuan/Month	92	93.88
2 Lump Sum Amount	6	6.12
Total	98	100.00

fn079_w2_9_1: Yuan/Month

Mean	SD	Min	Max	Obs
109,524.92	1,042,486.65	-1.00	9,999,999.00	92

fn079_w2_9_2: Lump Sum Amount

Mean	SD	Min	Max	Obs
15,233.17	37,118.15	-1.00	91,000.00	6

fn079_w2_9_min: Min Bracket of fn079_w2_9

Mean	SD	Min	Max	Obs
800.00	461.88	100.00	1,500.00	7

fn079_w2_9_max: Max Bracket of fn079_w2_9

Mean	SD	Min	Max	Obs
837.50	555.33	100.00	1,500.00	8

fn079_w2_10_1: Year of Receive Pension

Mean	SD	Min	Max	Obs
2,012.33	7.54	1,900.00	2,018.00	313

fn079_w2_10_2: Month of Receive Pension

Mean	SD	Min	Max	Obs
5.01	4.17	0.00	12.00	307

fn079_w2_11: Monthly Pension

Mean	SD	Min	Max	Obs
938.90	597.96	-1.00	3,000.00	313

fn079_w2_11_min: Min Bracket of fn079_w2_11

Mean	SD	Min	Max	Obs
500.00	.	500.00	500.00	1

fn079_w2_11_max: Max Bracket of fn079_w2_11

Mean	SD	Min	Max	Obs
1,000.00	.	1,000.00	1,000.00	1

fn080_w4: Address of Participate in the Pension

	Freq.	%
1 Currently Residence	208	66.45
2 Other Township/Village/Neighborhood	62	19.81
3 Other Place	37	11.82
997 Don't Know	6	1.92

	Freq.	%
Total	313	100.00

fn073_w4: Receive/Participate in Life Insurance

	Freq.	%
1 Yes	766	3.89
2 No	18,947	96.11
Total	19,713	100.00

fn056_w2_3: Kind of Life Insurance

	Freq.	%
1 Term Life Insurance	221	28.85
2 Whole Life Insurance	258	33.68
3 Survivorship Insurance	62	8.09
4 Endowment Insurance	82	10.70
5 Other, Please Specify	143	18.67
Total	766	100.00

fn056_w2_7: Receive Reimbursement

	Freq.	%
1 Yes	74	9.66
2 No	692	90.34
Total	766	100.00

fn074_w4: Address of Participate in Pension

	Freq.	%
1 Currently Residence	388	56.07
2 Other Township/Village/Neighborhood	114	16.47
3 Other Place	162	23.41
997 Don't Know	28	4.05
Total	692	100.00

fn075_w4: Years of Participate in Pension

Mean	SD	Min	Max	Obs
10.04	6.89	0.00	50.00	687

fn056_w2_5: Pay Insurance Premium

	Freq.	%
1 Monthly	21	3.03
2 Quaterly	4	0.58
3 Half a Year	3	0.43
4 Annually	664	95.95
Total	692	100.00

fn056_w2_6: Payment Amount

	Freq.	%
1 Pay It Monthly	18	2.60
2 Pay It Quarterly	4	0.58
3 Pay It Every Six Months	3	0.43
4 Pay It Annually	667	96.39
Total	692	100.00

fn056_w2_6_1: Pay It Monthly

Mean	SD	Min	Max	Obs
207.10	354.91	1.00	1,200.00	15

fn056_w2_6_2: Pay It Quarterly

Mean	SD	Min	Max	Obs
1,805.00	2,173.70	300.00	5,000.00	4

fn056_w2_6_3: Pay It Every Six Months

Mean	SD	Min	Max	Obs
472.50	180.31	345.00	600.00	2

fn056_w2_6_4: Pay It Annually

Mean	SD	Min	Max	Obs
4,014.97	10,165.16	0.00	200,000.00	658

fn076_w4: Receive Pension in the Future

	Freq.	%
1 Yes	302	47.94
2 No	328	52.06
Total	630	100.00

fn076_w4_a: When do You Expect to Receive the Pension

	Freq.	%
1 Age	213	70.53
2 Year	89	29.47
Total	302	100.00

fn076_w4_a_1: Age

Mean	SD	Min	Max	Obs
64.14	9.16	50.00	120.00	214

fn076_w4_a_2: Years

Mean	SD	Min	Max	Obs
9.85	11.60	0.00	65.00	91

fn056_w2_9: Expect Receive Pension

	Freq.	%
1 Yuan/Month	155	51.32
2 Yuan/Lump sum amount	147	48.68
Total	302	100.00

fn056_w2_9_1: Yuan/Month

Mean	SD	Min	Max	Obs
591.08	2,510.93	-1.00	30,000.00	157

fn056_w2_9_2: Yuan/Lump sum amount

Mean	SD	Min	Max	Obs
58,231.37	100,568.18	-1.00	1,000,000.00	147

fn056_w2_9_min: Min Bracket of fn056_w2_9

Mean	SD	Min	Max	Obs
4,333.33	3,925.78	1,000.00	10,000.00	18

fn056_w2_9_max: Max Bracket of fn056_w2_9

Mean	SD	Min	Max	Obs
2,129.03	2,473.08	1,000.00	10,000.00	31

fn077_w4_1: Year of Receive Pension

Mean	SD	Min	Max	Obs
2,010.32	6.90	1,990.00	2,018.00	73

fn077_w4_2: Month of Receive Pension

Mean	SD	Min	Max	Obs
4.53	4.20	0.00	12.00	73

fn056_w2_8: How Much did You Get

Mean	SD	Min	Max	Obs
7,007.92	12,556.22	-1.00	66,000.00	74

fn056_w2_8_min: Min Bracket of fn056_w2_8

Mean	SD	Min	Max	Obs
3,000.00	2,828.43	1,000.00	5,000.00	2

fn056_w2_8_max: Max Bracket of fn056_w2_8

Mean	SD	Min	Max	Obs
2,750.00	3,500.00	1,000.00	8,000.00	4

fn078_w4: Address of Receive Pension

	Freq.	%
1 Currently Residence	43	58.11
2 Other Township/Village/Neighborhood	12	16.22
3 Other Place	13	17.57
997 Don't Know	6	8.11
Total	74	100.00

fn043_w4: Receive/Particate in Commercial Pension

	Freq.	%
1 Yes, I Participate	175	0.89
2 Yes, I Receive it	35	0.18
No	19,503	98.93
Total	19,713	100.00

fn044_w2: Address of Participate

	Freq.	%
1 Currently Residence	92	52.57
2 Other Township/Village/Neighborhood	26	14.86
3 Other Place	44	25.14
997 Don't Know	13	7.43
Total	175	100.00

fn047_w2: Contribute to Commercial Pension

	Freq.	%
1 Annual Payment	153	87.43
2 Lump Sum Amount	22	12.57
Total	175	100.00

fn045_w4: Years of Participated in the Pension

Mean	SD	Min	Max	Obs
7.45	5.47	0.00	20.00	151

fn048_w2: Contribute to Pension

Mean	SD	Min	Max	Obs
6,852.82	12,670.15	-1.00	100,000.00	153

fn048_w2_min: Min Bracket of fn048_w2

No Observations

fn048_w2_max: Max Bracket of fn048_w2

No Observations

fn049_w2: Years of Need to Pay

Mean	SD	Min	Max	Obs
11.42	6.58	0.00	30.00	151

fn046_w4_1: Year of Started to Pay

Mean	SD	Min	Max	Obs
2,005.14	11.45	1,977.00	2,018.00	22

fn046_w4_2: Month of Started to Pay

Mean	SD	Min	Max	Obs
4.50	4.06	0.00	12.00	22

fn050_w2: Premium in Total

Mean	SD	Min	Max	Obs
33,477.23	34,026.49	-1.00	107,000.00	22

fn050_w2_min: Min Bracket of fn050_w2

No Observations

fn050_w2_max: Max Bracket of fn050_w2

No Observations

fn051_w4: Expect to Receive Pension

	Freq.	%
1 Age	127	72.57
2 Year	48	27.43
Total	175	100.00

fn051_w4_1: Age

Mean	SD	Min	Max	Obs
62.52	8.50	45.00	100.00	127

fn051_w4_2: Years

Mean	SD	Min	Max	Obs
8.04	6.13	0.00	20.00	50

fn051_w2: Receive the Pension

	Freq.	%
1 Lump Sum Amount	72	41.14
2 Yearly	48	27.43
Monthly	55	31.43
Total	175	100.00

fn052_w4: Expect to Receive After Your Retirement

Mean	SD	Min	Max	Obs
5,102.44	8,209.00	-1.00	30,000.00	48

fn052_w4_min: Min Bracket of fn052_w4

Mean	SD	Min	Max	Obs
4,320.00	4,769.91	1,200.00	12,000.00	5

fn052_w4_max: Max Bracket of fn052_w4

Mean	SD	Min	Max	Obs
7,950.00	7,880.36	1,200.00	24,000.00	8

fn053_w2: Expect to Receive in the Future

Mean	SD	Min	Max	Obs
995.42	1,428.38	-1.00	7,300.00	55

fn053_w2_min: Min Bracket of fn053_w2

Mean	SD	Min	Max	Obs
785.71	267.26	500.00	1,000.00	7

fn053_w2_max: Max Bracket of fn053_w2

Mean	SD	Min	Max	Obs

1,000.00	433.01	500.00	2,000.00	9

fn054_w4: Expect to Receive in Total

Mean	SD	Min	Max	Obs
37,166.49	55,111.67	-1.00	300,000.00	72

fn054_w4_min: Min Bracket of fn054_w4

Mean	SD	Min	Max	Obs
45,666.67	28,133.61	1,000.00	100,000.00	9

fn054_w4_max: Max Bracket of fn054_w4

Mean	SD	Min	Max	Obs
45,818.18	41,123.76	1,000.00	100,000.00	11

fn055_w2_1: The Year of You Started to Receive Pension

Mean	SD	Min	Max	Obs
2,012.34	6.93	1,982.00	2,018.00	35

fn055_w2_2: The Month of You Started to Receive Pension

Mean	SD	Min	Max	Obs
5.71	4.43	0.00	12.00	35

fn056_w2: Monthly Benefit

Mean	SD	Min	Max	Obs
1,395.49	3,321.63	-1.00	20,000.00	35

fn056_w2_max: Max Bracket of fn056_w2

Mean	SD	Min	Max	Obs
500.00	.	500.00	500.00	1

fn056_w4: Where did You Receive Pension

	Freq.	%
1 Currently Residence	20	57.14
2 Other Township/Village/Neighborhood	11	31.43
3 Other Place	3	8.57
997 Don't Know	1	2.86
Total	35	100.00

fn083_w2: Other Pension Program

	Freq.	%
1 Yes Participate in	82	0.42
2 Yes Receive	124	0.63
No	19,507	98.96
Total	19,713	100.00

fn084_w4: Receive Pension

Mean	SD	Min	Max	Obs
1.34	0.47	1.00	2.00	206

fn085_w2: Address of Participate Pension

	Freq.	%
1 Currently Residence	34	48.57
2 Other Township/Village/Neighborhood	8	11.43
3 Other Place	27	38.57
997 Don't Know	1	1.43
Total	70	100.00

fn087_w2: Need to Pay the Premium

	Freq.	%
1 Yes	59	84.29
2 No	11	15.71
Total	70	100.00

fn089_w2: Contribute to Pension

	Freq.	%
1 Annual/Monthly Payment	48	81.36
2 Lump Sum Amount	11	18.64
Total	59	100.00

fn090_w4: Years of Participate in Pension

Mean	SD	Min	Max	Obs
7.92	6.75	1.00	35.00	48

fn091_w2: Years of Need to Pay

Mean	SD	Min	Max	Obs
11.79	7.40	0.00	42.00	47

fn090_w2: Need to Contribute

Mean	SD	Min	Max	Obs
3,910.60	4,051.62	3.00	20,000.00	48

fn092_w4: Premium Need to Pay in Total

Mean	SD	Min	Max	Obs
11,336.36	27,285.76	300.00	93,000.00	11

fn093_w2: When do You Expect to Receive Pension

	Freq.	%
1 Age	63	90.00
2 Year	7	10.00
Total	70	100.00

fn093_w2_1: Age

Mean	SD	Min	Max	Obs
59.79	4.23	50.00	77.00	63

fn093_w2_2: Year

Mean	SD	Min	Max	Obs
14.81	18.77	0.50	60.00	8

fn094_w2: Expect Pension

	Freq.	%
1 Yuan/month	63	90.00
2 Yuan/Lump sum amount	7	10.00
Total	70	100.00

fn094_w2_1: Yuan/month

Mean	SD	Min	Max	Obs
541.91	768.31	0.00	3,780.00	54

fn094_w2_2: Yuan/Lump sum amount

Mean	SD	Min	Max	Obs
28,750.00	48,023.43	0.00	100,000.00	4

fn095_w2_1: The Year of Receive Pension

Mean	SD	Min	Max	Obs

2,011.09	6.40	1,962.00	2,018.00	133

fn095_w2_2: The Month of Receive Pension

Mean	SD	Min	Max	Obs
4.26	4.33	0.00	12.00	128

fn096_w2: Monthly Pension

Mean	SD	Min	Max	Obs
854.49	1,368.17	-1.00	11,000.00	136

fn096_w2_min: Min Bracket of fn096_w2

Mean	SD	Min	Max	Obs
750.00	353.55	500.00	1,000.00	2

fn096_w2_max: Max Bracket of fn096_w2

Mean	SD	Min	Max	Obs
750.00	353.55	500.00	1,000.00	2

fn096_w4: Where did You Receive Pension

	Freq.	%
1 Currently Residence	85	62.50
2 Other Township/Village/Neighborhood	30	22.06
3 Other Place	18	13.24
997 Don't Know	3	2.21
Total	136	100.00

fn099_w4: Receive/Particate in Any of the Following Pensions

	Freq.	%
14 Have No Pension	2,081	94.72
997 Don't Know	102	4.64
999 Refused to Answer	14	0.64
Total	2,197	100.00

fn100_w4_s1: If Ever Had the Pension

	Freq.	%
0 No	2,197	100.00
Total	2,197	100.00

fn100_w4_s2: If Ever Had the Pension

	Freq.	%
0 No	2,196	99.95
2 Institutions Pension	1	0.05
Total	2,197	100.00

fn100_w4_s3: If Ever Had the Pension

	Freq.	%
0 No	2,170	98.77
3 Firm Pension	27	1.23
Total	2,197	100.00

fn100_w4_s4: If Ever Had the Pension

	Freq.	%
0 No	2,197	100.00
Total	2,197	100.00

fn100_w4_s5: If Ever Had the Pension

	Freq.	%
0 No	2,197	100.00
Total	2,197	100.00

fn100_w4_s6: If Ever Had the Pension

	Freq.	%
0 No	2,197	100.00
Total	2,197	100.00

fn100_w4_s7: If Ever Had the Pension

	Freq.	%
0 No	2,188	99.59
7 Urban and Rural Residents Pension	9	0.41
Total	2,197	100.00

fn100_w4_s8: If Ever Had the Pension

	Freq.	%
0 No	2,161	98.36
8 Newrural Pension	36	1.64
Total	2,197	100.00

fn100_w4_s9: If Ever Had the Pension

	Freq.	%

	Freq.	%
0 No	2,193	99.82
9 Urban Residents Pension	4	0.18
Total	2,197	100.00

fn100_w4_s10: If Ever Had the Pension

	Freq.	%
0 No	2,197	100.00
Total	2,197	100.00

fn100_w4_s11: If Ever Had the Pension

	Freq.	%
0 No	2,188	99.59
11 Life Pension	9	0.41
Total	2,197	100.00

fn100_w4_s12: If Ever Had the Pension

	Freq.	%
0 No	2,193	99.82
12 Commercial Pension	4	0.18
Total	2,197	100.00

fn100_w4_s13: If Ever Had the Pension

	Freq.	%
0 No	2,167	98.63
13 Other Pension	30	1.37
Total	2,197	100.00

fn100_w4_s14: If Ever Had the Pension

	Freq.	%
0 No	2,192	99.77
14 Have No Pension	5	0.23
Total	2,197	100.00

fn100_w4_s15: Never Received or Participated in Any Pension

	Freq.	%
0 No	234	10.65
15 Never Received or Participated in Any Pension Insurance	1,963	89.35
Total	2,197	100.00

fn101_w4_1_2_: The Year of Stop This Pension

Mean	SD	Min	Max	Obs

2,011.00	.	2,011.00	2,011.00	1

fn101_w4_2_2_: The Month of Stop This Pension

Mean	SD	Min	Max	Obs
7.00	.	7.00	7.00	1

fn101_w4_1_3_: The Year of Stop This Pension

Mean	SD	Min	Max	Obs
2,005.85	7.14	1,992.00	2,017.00	27

fn101_w4_2_3_: The Month of Stop This Pension

Mean	SD	Min	Max	Obs
4.08	4.01	0.00	12.00	26

fn101_w4_1_7_: The Year of Stop This Pension

Mean	SD	Min	Max	Obs
2,015.00	1.80	2,012.00	2,017.00	9

fn101_w4_2_7_: The Month of Stop This Pension

Mean	SD	Min	Max	Obs
6.33	3.57	1.00	11.00	9

fn101_w4_1_8_: The Year of Stop This Pension

Mean	SD	Min	Max	Obs
2,012.69	4.40	2,000.00	2,018.00	35

fn101_w4_2_8_: The Month of Stop This Pension

Mean	SD	Min	Max	Obs
4.03	4.72	0.00	12.00	35

fn101_w4_1_9_: The Year of Stop This Pension

Mean	SD	Min	Max	Obs
2,014.75	1.26	2,013.00	2,016.00	4

fn101_w4_2_9_: The Month of Stop This Pension

Mean	SD	Min	Max	Obs

6.50	4.93	0.00	12.00	4

fn101_w4_1_11_: The Year of Stop This Pension

Mean	SD	Min	Max	Obs
2,014.22	3.63	2,008.00	2,018.00	9

fn101_w4_2_11_: The Month of Stop This Pension

Mean	SD	Min	Max	Obs
1.88	2.47	0.00	6.00	8

fn101_w4_1_12_: The Year of Stop This Pension

Mean	SD	Min	Max	Obs
2,010.75	6.50	2,002.00	2,017.00	4

fn101_w4_2_12_: The Month of Stop This Pension

Mean	SD	Min	Max	Obs
4.75	5.85	0.00	12.00	4

fn101_w4_1_13_: The Year of Stop This Pension

Mean	SD	Min	Max	Obs
2,006.41	22.61	1,900.00	2,018.00	27

fn101_w4_2_13_: The Month of Stop This Pension

Mean	SD	Min	Max	Obs
4.92	4.48	0.00	12.00	26

fn101_w4_1_14_: The Year of Stop This Pension

Mean	SD	Min	Max	Obs
2,010.40	9.86	1,993.00	2,016.00	5

fn101_w4_2_14_: The Month of Stop This Pension

Mean	SD	Min	Max	Obs
7.00	4.97	0.00	11.00	4

fn097_w2: What You Can Rely on Financially for Old-Age Support

	Freq.	%

1 Children	11,178	56.70
2 Savings	732	3.71
3 Pension or Retirement Salary	6,369	32.31
4 Commercial Pension Insurance	65	0.33
5 Other	1,369	6.94
Total	19,713	100.00

fn098_w2_s1: 1th Child

	Freq.	%
0 No	1,531	13.70
1th Child	9,647	86.30
Total	11,178	100.00

fn098_w2_s2: 2th Child

	Freq.	%
0 No	3,228	28.88
2th Child	7,950	71.12
Total	11,178	100.00

fn098_w2_s3: 3th Child

	Freq.	%
0 No	6,982	62.46
3th Child	4,196	37.54
Total	11,178	100.00

fn098_w2_s4: 4th Child

	Freq.	%
0 No	9,150	81.86
4th Child	2,028	18.14
Total	11,178	100.00

fn098_w2_s5: 5th Child

	Freq.	%
0 No	10,279	91.96
5th Child	899	8.04
Total	11,178	100.00

fn098_w2_s6: 6th Child

	Freq.	%
0 No	10,800	96.62
6th Child	378	3.38
Total	11,178	100.00

fn098_w2_s7: 7th Child

	Freq.	%
0 No	11,010	98.50
7th Child	168	1.50
Total	11,178	100.00

fn098_w2_s8: 8th Child

	Freq.	%
0 No	11,112	99.41
8th Child	66	0.59
Total	11,178	100.00

fn098_w2_s9: 9th Child

	Freq.	%
0 No	11,154	99.79
9th Child	24	0.21
Total	11,178	100.00

fn098_w2_s10: 10th Child

	Freq.	%
0 No	11,167	99.90
10th Child	11	0.10
Total	11,178	100.00

fn098_w2_s11: 11th Child

	Freq.	%
0 No	11,171	99.94
11th Child	7	0.06
Total	11,178	100.00

fn098_w2_s12: 12th Child

	Freq.	%
0 No	11,175	99.97
12th Child	3	0.03
Total	11,178	100.00

fn098_w2_s13: 13th Child

	Freq.	%
0 No	11,177	99.99
13th Child	1	0.01
Total	11,178	100.00

fn098_w2_s14: 14th Child

	Freq.	%
0 No	11,177	99.99
14th Child	1	0.01
Total	11,178	100.00

fn098_w2_s15: 15th Child

	Freq.	%
0 No	11,177	99.99
15th Child	1	0.01
Total	11,178	100.00

fn098_w2_s16: 16th Child

	Freq.	%
0 No	11,178	100.00
Total	11,178	100.00

fn098_w2_s17: 17th Child

	Freq.	%
0 No	11,178	100.00
Total	11,178	100.00

fn098_w2_s18: 18th Child

	Freq.	%
0 No	11,178	100.00
Total	11,178	100.00

fn098_w2_s19: 19th Child

	Freq.	%
0 No	11,178	100.00
Total	11,178	100.00

fn098_w2_s20: 20th Child

	Freq.	%
0 No	11,178	100.00
Total	11,178	100.00

fn098_w2_s21: 21th Child

	Freq.	%
0 No	11,178	100.00
Total	11,178	100.00

fn098_w2_s22: 22th Child

	Freq.	%
0 No	11,178	100.00
Total	11,178	100.00

fn098_w2_s23: 23th Child

	Freq.	%
0 No	11,178	100.00
Total	11,178	100.00

fn098_w2_s24: 24th Child

	Freq.	%
0 No	11,178	100.00
Total	11,178	100.00

fn098_w2_s25: 25th Child

	Freq.	%
0 No	11,178	100.00
Total	11,178	100.00

fn098_w2_s99: Don't Know

	Freq.	%
0 No	11,142	99.68
99 Don't Know	36	0.32
Total	11,178	100.00

versionID: Version ID

A String Variable	
Obs:	19,718

MODULE **G&H1**

Individual Income

ID: Individual ID

A String Variable	
Obs:	19,755

householdID: Household ID

A String Variable	
Obs:	19,755

communityID: Community ID

A String Variable	
Obs:	19,755

ga001: Receive Wage and Bonus Income

	Freq.	%
1 Yes	4,947	25.05
2 No	14,804	74.95
Total	19,751	100.00

ga002: How Much Receive

Mean	SD	Min	Max	Obs
28,199.30	92,512.09	0.00	6,000,000.00	4,849

ga002_min: Min Bracket of ga002

Mean	SD	Min	Max	Obs
26,610.17	23,588.44	5,000.00	100,000.00	59

ga002_max: Max Bracket of ga002

Mean	SD	Min	Max	Obs
26,582.28	26,584.94	5,000.00	100,000.00	79

ga002_w2_1: Exclude Insurance and Some Others

	Freq.	%
1 Yes	1,120	22.64
2 No	3,665	74.10
997 Don't Know	149	3.01
999 Refused to Answer	12	0.24
Total	4,946	100.00

ga002_w2_2: Total Excluded Amount

	Freq.	%
1 Yuan/Year	462	9.36
2 Yuan/Month	463	9.38
3 Percent of Wage	69	1.40
4 No	3,446	69.84
997 Don't Know	486	9.85
999 Refused to Answer	8	0.16
Total	4,934	100.00

ga002_w2_2a: Yuan/Year

Mean	SD	Min	Max	Obs
5,476.22	12,445.06	0.00	100,000.00	506

ga002_w2_2b: Yuan/Month

Mean	SD	Min	Max	Obs
958.98	992.92	0.00	7,500.00	468

ga002_w2_2c: Percent of Wage

Mean	SD	Min	Max	Obs
14.55	15.88	0.00	108.00	75

ga002_w2_2_min: Min Bracket of ga002_w2_2

Mean	SD	Min	Max	Obs
600.00	624.50	300.00	2,000.00	7

ga002_w2_2_max: Max Bracket of ga002_w2_2

Mean	SD	Min	Max	Obs

314.63	130.16	300.00	2,000.00	205

ga003_w4_s1: Pension

	Freq.	%
0 No	10,376	52.54
1 Yes	9,374	47.46
Total	19,750	100.00

ga003_w4_s2: Unemployment Compensation

	Freq.	%
0 No	19,711	99.80
2 Yes	39	0.20
Total	19,750	100.00

ga003_w4_s3: Pension Voucher

	Freq.	%
0 No	19,632	99.40
3 Yes	118	0.60
Total	19,750	100.00

ga003_w4_s4: Pension Subsidy for the Oldest Old

	Freq.	%
0 No	18,790	95.14
4 Yes	960	4.86
Total	19,750	100.00

ga003_w4_s5: Workers' Industrial Accident Compensation

	Freq.	%
0 No	19,699	99.74
5 Yes	51	0.26
Total	19,750	100.00

ga003_w4_s6: Elderly Family Planning Subsidy

	Freq.	%
0 No	19,174	97.08
6 Yes	576	2.92
Total	19,750	100.00

ga003_w4_s7: Medical Aid

	Freq.	%
0 No	19,674	99.62

	Freq.	%
7 Yes	76	0.38
Total	19,750	100.00

ga003_w4_s8: Other Government Subsidy

	Freq.	%
0 No	18,765	95.01
8 Yes	985	4.99
Total	19,750	100.00

ga003_w4_s9: Other Income Source

	Freq.	%
0 No	19,678	99.64
9 Yes	72	0.36
Total	19,750	100.00

ga003_w4_s10: None of the Above

	Freq.	%
0 No	10,556	53.45
10 Yes	9,194	46.55
Total	19,750	100.00

ga003_w4_1: Pension Amount

Mean	SD	Min	Max	Obs
10,798.60	18,989.82	0.00	600,000.00	9,292

ga003_w4_2: Unemployment Compensation Amount

Mean	SD	Min	Max	Obs
7,595.48	26,738.21	0.00	170,012.00	40

ga003_w4_3: Pension Voucher Amount

Mean	SD	Min	Max	Obs
1,561.72	3,215.41	0.00	28,000.00	130

ga003_w4_4: Pension Subsidy for the Oldest Old Amount

Mean	SD	Min	Max	Obs
944.33	1,035.22	0.00	15,600.00	954

ga003_w4_5: Workers' Industrial Accident Compensation Amount

Mean	SD	Min	Max	Obs

2,200.42	4,975.80	0.00	25,000.00	52

ga003_w4_6: Elderly Family Planning Subsidy Amount

Mean	SD	Min	Max	Obs
1,069.53	1,454.03	0.00	21,024.00	573

ga003_w4_7: Medical Aid Amount

Mean	SD	Min	Max	Obs
3,417.66	9,941.83	0.00	80,000.00	79

ga003_w4_8: Other Government Subsidy Amount

Mean	SD	Min	Max	Obs
3,077.41	6,396.72	0.00	100,000.00	977

ga003_w4_9: Other Income Source Amount

Mean	SD	Min	Max	Obs
2,398.59	3,891.99	0.00	20,268.00	81

ga004_w4_3_1_s1: Life Care

	Freq.	%
0 No	81	68.64
1 Yes	37	31.36
Total	118	100.00

ga004_w4_3_1_s2: Domestic Service

	Freq.	%
0 No	117	99.15
2 Yes	1	0.85
Total	118	100.00

ga004_w4_3_1_s3: Rehabilitation Service

	Freq.	%
0 No	118	100.00
Total	118	100.00

ga004_w4_3_1_s4: Buy Food

	Freq.	%
0 No	117	99.15
4 Yes	1	0.85

	Freq.	%
Total	118	100.00

ga004_w4_3_1_s5: Buy Medicine

	Freq.	%
0 No	59	50.00
5 Yes	59	50.00
Total	118	100.00

ga004_w4_3_1_s6: Buy Life Items

	Freq.	%
0 No	77	65.25
6 Yes	41	34.75
Total	118	100.00

ga004_w4_3_1_s7: Emergence Medical Service

	Freq.	%
0 No	118	100.00
Total	118	100.00

ga004_w4_3_1_s8: Other

	Freq.	%
0 No	83	70.34
8 Yes	35	29.66
Total	118	100.00

ga004_w4_1_min: Min Bracket of ga003_w4_1

Mean	SD	Min	Max	Obs
1,505.56	3,214.22	400.00	20,000.00	72

ga004_w4_1_max: Max Bracket of ga003_w4_1

Mean	SD	Min	Max	Obs
2,490.24	4,593.62	400.00	20,000.00	82

ga009_w4_1: Inherited

	Freq.	%
1 Yes	99	0.51
2 No	19,320	99.38
999 Refused to Answer	22	0.11
Total	19,441	100.00

ga009_w4_1_1: Times

Mean	SD	Min	Max	Obs
0.98	0.19	0.00	2.00	113

ga009_w4_2_1: Amount Inherited

Mean	SD	Min	Max	Obs
96,064.90	317,052.52	0.00	3,000,000.00	96

ga009_w4_2_2: Year Inherited

Mean	SD	Min	Max	Obs
2,014.34	6.79	1,970.00	2,018.00	97

ga009_w4_3: From Whom Inherited

	Freq.	%
1 Parents	64	65.31
2 Parents-in-Law	5	5.10
4 Relatives	4	4.08
5 Others	25	25.51
Total	98	100.00

ga009_w4_4_1: Amount Inherited

Mean	SD	Min	Max	Obs
600,000.00	.	600,000.00	600,000.00	1

ga009_w4_4_2: Amount Inherited

Mean	SD	Min	Max	Obs
400,000.00	.	400,000.00	400,000.00	1

ga009_w4_4_3: Year Inherited

Mean	SD	Min	Max	Obs
2,010.00	.	2,010.00	2,010.00	1

ga009_w4_5: From Whom Inherited

	Freq.	%
1 Parents	1	100.00
Total	1	100.00

ga011_w4_1: Inherited

	Freq.	%
1 Yes	5	1.62
2 No	303	98.38
Total	308	100.00

ga011_w4_1_1: Times

Mean	SD	Min	Max	Obs
1.17	0.98	0.00	3.00	6

ga011_w4_2_1: Amount Inherited

Mean	SD	Min	Max	Obs
90,250.00	99,967.91	5,000.00	200,000.00	4

ga011_w4_2_2: Year Inherited

Mean	SD	Min	Max	Obs
2,004.00	5.48	1,997.00	2,010.00	4

ga011_w4_3: From Whom Inherited

	Freq.	%
1 Parents	3	75.00
2 Parents-in-Law	1	25.00
Total	4	100.00

ga011_w4_4_1: Amount Inherited

Mean	SD	Min	Max	Obs
20,000.00	.	20,000.00	20,000.00	1

ga011_w4_4_2: Amount Inherited

Mean	SD	Min	Max	Obs
20,000.00	.	20,000.00	20,000.00	1

ga011_w4_4_3: Year Inherited

Mean	SD	Min	Max	Obs
1,993.00	.	1,993.00	1,993.00	1

ga011_w4_5: From Whom Inherited

	Freq.	%

4 Relatives	1	100.00
Total	1	100.00

hc001: Cash at Home

Mean	SD	Min	Max	Obs
2,412.58	10,866.10	0.00	500,000.00	19,116

hc002_min: Min Bracket of hc002

Mean	SD	Min	Max	Obs
1,812.18	2,040.46	500.00	10,000.00	1,962

hc002_max: Max Bracket of hc002

Mean	SD	Min	Max	Obs
1,336.72	1,768.41	500.00	10,000.00	3,785

hc003_w4: Electronic Money

Mean	SD	Min	Max	Obs
393.35	5,173.69	0.00	500,000.00	19,669

hc004_w4_min: Min Bracket of hc004_w4

Mean	SD	Min	Max	Obs
1,610.94	2,019.61	50.00	5,000.00	32

hc004_w4_max: Max Bracket of hc004_w4

Mean	SD	Min	Max	Obs
1,367.04	1,807.12	10.00	5,000.00	27

hc005: Deposit

Mean	SD	Min	Max	Obs
20,467.28	730,620.24	0.00	100000000.00	19,054

hc006_min: Min Bracket of hc006

Mean	SD	Min	Max	Obs
16,193.05	43,077.30	2,000.00	500,000.00	2,476

hc006_max: Max Bracket of hc006

Mean	SD	Min	Max	Obs

9,366.93	40,620.42	2,000.00	500,000.00	11,133

hc007: Own Bond

	Freq.	%
1 Yes	75	0.38
2 No	19,614	99.34
999 Refused to Answer	55	0.28
Total	19,744	100.00

hc008: Bond

Mean	SD	Min	Max	Obs
49,574.78	132,216.32	0.00	1,000,000.00	69

hc009_min: Min Bracket of hc009

No Observations

hc009_max: Max Bracket of hc009

Mean	SD	Min	Max	Obs
10,000.00	0.00	10,000.00	10,000.00	5

hc010: Own Stock

	Freq.	%
1 Yes	209	1.06
2 No	19,496	98.74
999 Refused to Answer	39	0.20
Total	19,744	100.00

hc013: Stock

Mean	SD	Min	Max	Obs
88,024.38	218,773.47	0.00	2,500,000.00	201

hc014_min: Min Bracket of hc014

Mean	SD	Min	Max	Obs
52,000.00	31,937.44	10,000.00	100,000.00	5

hc014_max: Max Bracket of hc014

Mean	SD	Min	Max	Obs
30,000.00	32,732.68	10,000.00	100,000.00	15

hc015: Own Mutual Fund

	Freq.	%
1 Yes	124	0.63
2 No	19,576	99.15
999 Refused to Answer	44	0.22
Total	19,744	100.00

hc018: Mutual Fund

Mean	SD	Min	Max	Obs
91,358.47	259,031.54	0.00	2,500,000.00	118

hc019_min: Min Bracket of hc019

Mean	SD	Min	Max	Obs
275,000.00	318,198.05	50,000.00	500,000.00	2

hc019_max: Max Bracket of hc019

Mean	SD	Min	Max	Obs
32,500.00	45,000.00	10,000.00	100,000.00	4

hc016_w4: Own Other Financial Products

	Freq.	%
1 Yes	256	1.30
2 No	19,448	98.50
999 Refused to Answer	40	0.20
Total	19,744	100.00

hc017_w4: Other Financial Products

Mean	SD	Min	Max	Obs
123,511.62	302,373.95	0.00	3,000,000.00	241

hc017_w4_min: Min Bracket of hc017_w4

Mean	SD	Min	Max	Obs
157,500.00	153,133.93	10,000.00	500,000.00	8

hc017_w4_max: Max Bracket of hc017_w4

Mean	SD	Min	Max	Obs
113,636.36	147,802.08	10,000.00	500,000.00	11

hc020: Percent of Financial Products Controlled by You Not Your Spouse

Mean	SD	Min	Max	Obs
72.25	36.72	0.00	100.00	8,538

hc021: Any Financial Products Under Other Person's Name

	Freq.	%
1 Yes	189	0.96
2 No	19,502	98.77
999 Refused to Answer	53	0.27
Total	19,744	100.00

hc022: Value of Financial Products Under Other Person's Name

Mean	SD	Min	Max	Obs
52,437.32	121,578.93	0.00	1,200,000.00	179

hc027: Own Public Housing Funding

	Freq.	%
1 Yes	650	3.29
2 No	19,066	96.57
999 Refused to Answer	28	0.14
Total	19,744	100.00

hc028: Public Housing Funding

Mean	SD	Min	Max	Obs
46,748.44	54,405.79	0.00	280,000.00	550

hc029_min: Min Bracket of hc029

Mean	SD	Min	Max	Obs
29,538.46	30,435.94	5,000.00	100,000.00	65

hc029_max: Max Bracket of hc029

Mean	SD	Min	Max	Obs
36,559.63	45,839.38	5,000.00	200,000.00	109

hc030: Own Jizikuan

	Freq.	%
1 Yes	196	0.99
2 No	19,514	98.84
999 Refused to Answer	34	0.17
Total	19,744	100.00

hc031: Jizikuan

Mean	SD	Min	Max	Obs
76,841.40	202,553.08	0.00	1,500,000.00	188

hc032_min: Min Bracket of hc032

Mean	SD	Min	Max	Obs
53,333.33	45,092.50	10,000.00	100,000.00	3

hc032_max: Max Bracket of hc032

Mean	SD	Min	Max	Obs
51,666.67	42,504.90	5,000.00	100,000.00	6

hc033: Have Unpaid Salary

	Freq.	%
1 Yes	960	4.86
2 No	18,761	95.02
999 Refused to Answer	23	0.12
Total	19,744	100.00

hc034: Unpaid Salary

Mean	SD	Min	Max	Obs
39,547.87	352,631.05	0.00	10000000.00	949

hc035_min: Min Bracket of hc035

Mean	SD	Min	Max	Obs
25,000.00	22,912.88	5,000.00	50,000.00	5

hc035_max: Max Bracket of hc035

Mean	SD	Min	Max	Obs
20,000.00	22,156.47	5,000.00	50,000.00	12

hc039_w3: Lend Money to Others

	Freq.	%
1 Yes	1,856	9.40
2 No	17,833	90.32
999 Refused to Answer	55	0.28
Total	19,744	100.00

hc040_w3: Money Lent to Others

Mean	SD	Min	Max	Obs
77,500.03	369,345.00	0.00	7,000,000.00	1,828

hc041_w3_min: Min Bracket of hc041_w3

Mean	SD	Min	Max	Obs
52,631.58	61,629.91	10,000.00	200,000.00	19

hc041_w3_max: Max Bracket of hc041_w3

Mean	SD	Min	Max	Obs
50,526.32	51,096.17	5,000.00	200,000.00	19

hd001: Loan

Mean	SD	Min	Max	Obs
5,955.84	56,043.95	0.00	3,000,000.00	19,689

hd002_min: Min Bracket of hd002

Mean	SD	Min	Max	Obs
47,894.74	36,867.47	5,000.00	100,000.00	19

hd002_max: Max Bracket of hd002

Mean	SD	Min	Max	Obs
78,800.00	132,329.14	5,000.00	500,000.00	25

hd003: Credit Card Balance

Mean	SD	Min	Max	Obs
231.85	4,230.27	0.00	200,000.00	19,712

hd004_min: Min Bracket of hd004

Mean	SD	Min	Max	Obs
6,642.86	4,422.62	500.00	10,000.00	7

hd004_max: Max Bracket of hd004

Mean	SD	Min	Max	Obs
11,318.18	19,478.54	500.00	50,000.00	11

hd004_w3: Other Borrowing

Mean	SD	Min	Max	Obs
7,995.72	72,719.09	0.00	5,000,000.00	19,682

hd004_w3_1_min: Min Bracket of hd004_w3_1

Mean	SD	Min	Max	Obs
18,578.95	19,571.28	1,000.00	50,000.00	19

hd004_w3_1_max: Max Bracket of hd004_w3_1

Mean	SD	Min	Max	Obs
13,638.89	20,299.82	500.00	50,000.00	18

hd006_w4: Any Fraud

	Freq.	%
1 Yes	1,456	7.38
2 No	18,269	92.54
999 Refused to Answer	17	0.09
Total	19,742	100.00

hd007_w4: Fraud Type

	Freq.	%
1 Telephone Fraud	943	64.77
2 Fund Raising Fraud	52	3.57
3 Pyramid Sale	45	3.09
4 Sale Fraud	250	17.17
5 Other	166	11.40
Total	1,456	100.00

hd008_w4: Lost Amount

Mean	SD	Min	Max	Obs
5,436.60	65,939.55	0.00	2,000,000.00	1,453

hd005_w4: Any Fraud

	Freq.	%
1 Yes	1,686	8.54
2 No	18,021	91.28
999 Refused to Answer	35	0.18
Total	19,742	100.00

hd005_w4_0: Times

Mean	SD	Min	Max	Obs

8.34	153.35	0.00	6,000.00	1,811

hd005_w4_1: Lost Amount

Mean	SD	Min	Max	Obs
20,879.31	130,671.63	0.00	3,000,000.00	1,678

hd005_w4_2: Year

Mean	SD	Min	Max	Obs
2,008.15	9.47	1,900.00	2,018.00	1,644

hd012: Respondent Receive Assistance

	Freq.	%
1 Never	16,610	84.14
2 A Few Time	2,340	11.85
3 Most or All of Time	792	4.01
Total	19,742	100.00

xrtype: Respondent Type

	Freq.	%
1 RE Interview	19,446	98.44
2 New Interview	309	1.56
Total	19,755	100.00

versionID: Version ID

A String Variable	
Obs:	19,755

MODULE G&H2

Household Income

householdID: Household ID

A String Variable	
Obs:	11,544

communityID: Community ID

A String Variable	
Obs:	11,544

ga000_w4: Financial Respondent

	Freq.	%
1 HHMemberName[1]	524	4.54
2 HHMemberName[2]	161	1.40
3 HHMemberName[3]	29	0.25
4 HHMemberName[4]	5	0.04
5 HHMemberName[5]	1	0.01
97 HHMemberName[97]	9,229	79.99
98 HHMemberName[98]	1,588	13.76
Total	11,537	100.00

ga005_1_: Wage and Bonus Income HHMemberName[1] Receive

	Freq.	%
1 Yes	1,811	33.81
2 No	3,453	64.47
997 Don't Know	82	1.53
999 Refused to Answer	10	0.19
Total	5,356	100.00

ga005_2_: Wage and Bonus Income HHMemberName[2] Receive

	Freq.	%
1 Yes	834	28.60
2 No	2,038	69.89
997 Don't Know	38	1.30
999 Refused to Answer	6	0.21
Total	2,916	100.00

ga005_3_: Wage and Bonus Income HHMemberName[3] Receive

	Freq.	%
1 Yes	204	12.08
2 No	1,462	86.56
997 Don't Know	19	1.12
999 Refused to Answer	4	0.24
Total	1,689	100.00

ga005_4_: Wage and Bonus Income HHMemberName[4] Receive

	Freq.	%
1 Yes	74	8.33
2 No	802	90.32
997 Don't Know	8	0.90
999 Refused to Answer	4	0.45
Total	888	100.00

ga005_5_: Wage and Bonus Income HHMemberName[5] Receive

	Freq.	%
1 Yes	16	5.37
2 No	278	93.29
997 Don't Know	3	1.01
999 Refused to Answer	1	0.34
Total	298	100.00

ga005_6_: Wage and Bonus Income HHMemberName[6] Receive

	Freq.	%
1 Yes	11	8.21
2 No	120	89.55
997 Don't Know	2	1.49
999 Refused to Answer	1	0.75
Total	134	100.00

ga005_7_: Wage and Bonus Income HHMemberName[7] Receive

	Freq.	%
1 Yes	3	4.55
2 No	62	93.94
997 Don't Know	1	1.52
Total	66	100.00

ga005_8_: Wage and Bonus Income HHMemberName[8] Receive

	Freq.	%
1 Yes	2	6.90
2 No	26	89.66
997 Don't Know	1	3.45
Total	29	100.00

ga005_9_: Wage and Bonus Income HHMemberName[9] Receive

	Freq.	%
2 No	18	94.74
997 Don't Know	1	5.26
Total	19	100.00

ga005_10_: Wage and Bonus Income HHMemberName[10] Receive

	Freq.	%
2 No	8	100.00
Total	8	100.00

ga005_11_: Wage and Bonus Income HHMemberName[11] Receive

	Freq.	%
2 No	3	100.00
Total	3	100.00

ga005_12_: Wage and Bonus Income HHMemberName[12] Receive

	Freq.	%
2 No	1	100.00
Total	1	100.00

ga006_w4_1_: Amount HHMemberName[1] Receive

Mean	SD	Min	Max	Obs
33,767.00	38,415.84	0.00	1,000,000.00	1,499

ga006_w4_2_: Amount HHMemberName[2] Receive

Mean	SD	Min	Max	Obs
31,151.04	34,934.19	0.00	700,000.00	704

ga006_w4_3_: Amount HHMemberName[3] Receive

Mean	SD	Min	Max	Obs
24,982.66	17,019.74	500.00	80,000.00	173

ga006_w4_4_: Amount HHMemberName[4] Receive

Mean	SD	Min	Max	Obs
27,685.93	26,473.84	500.00	150,000.00	54

ga006_w4_5_: Amount HHMemberName[5] Receive

Mean	SD	Min	Max	Obs
21,572.73	14,968.11	3,500.00	50,000.00	11

ga006_w4_6_: Amount HHMemberName[6] Receive

Mean	SD	Min	Max	Obs
23,277.78	17,126.57	2,500.00	50,000.00	9

ga006_w4_7_: Amount HHMemberName[7] Receive

Mean	SD	Min	Max	Obs
8,000.00	2,828.43	6,000.00	10,000.00	2

ga006_w4_8_: Amount HHMemberName[8] Receive

Mean	SD	Min	Max	Obs
18,000.00	.	18,000.00	18,000.00	1

ga006_w4_1__min: Min Bracket of ga006_w4_1_

Mean	SD	Min	Max	Obs
27,051.28	22,225.36	5,000.00	100,000.00	195

ga006_w4_1__max: Max Bracket of ga006_w4_1_

Mean	SD	Min	Max	Obs
34,133.66	24,517.57	5,000.00	100,000.00	202

ga006_w4_2__min: Min Bracket of ga006_w4_2_

Mean	SD	Min	Max	Obs
26,884.06	23,950.03	5,000.00	100,000.00	69

ga006_w4_2__max: Max Bracket of ga006_w4_2_

Mean	SD	Min	Max	Obs
34,452.05	29,054.09	5,000.00	100,000.00	73

ga006_w4_3__min: Min Bracket of ga006_w4_3_

Mean	SD	Min	Max	Obs
21,428.57	24,684.83	5,000.00	100,000.00	14

ga006_w4_3__max: Max Bracket of ga006_w4_3_

Mean	SD	Min	Max	Obs
28,125.00	24,824.38	10,000.00	100,000.00	16

ga006_w4_4__min: Min Bracket of ga006_w4_4_

Mean	SD	Min	Max	Obs
13,500.00	8,834.91	5,000.00	30,000.00	10

ga006_w4_4__max: Max Bracket of ga006_w4_4_

Mean	SD	Min	Max	Obs
22,777.78	10,929.06	5,000.00	30,000.00	9

ga006_w4_5__min: Min Bracket of ga006_w4_5_

Mean	SD	Min	Max	Obs
20,000.00	14,142.14	10,000.00	30,000.00	2

ga006_w4_5__max: Max Bracket of ga006_w4_5_

Mean	SD	Min	Max	Obs
15,000.00	13,228.76	5,000.00	30,000.00	3

ga006_w4_6__min: Min Bracket of ga006_w4_6_

No Observations

ga006_w4_6__max: Max Bracket of ga006_w4_6_

No Observations

ga006_w4_7__min: Min Bracket of ga006_w4_7_

No Observations

ga006_w4_7__max: Max Bracket of ga006_w4_7_

No Observations

ga006_w4_8__min: Min Bracket of ga006_w4_8_

No Observations

ga006_w4_8__max: Max Bracket of ga006_w4_8_

No Observations

ga006_w4_1_1_: Exclude Insurance and Some Others for HHMemberName[1]

	Freq.	%
1 Yes	573	31.64
2 No	922	50.91
997 Don't Know	305	16.84
999 Refused to Answer	11	0.61
Total	1,811	100.00

ga006_w4_1_2_: Exclude Insurance and Some Others for HHMemberName[2]

	Freq.	%
1 Yes	266	31.89
2 No	426	51.08
997 Don't Know	137	16.43
999 Refused to Answer	5	0.60
Total	834	100.00

ga006_w4_1_3_: Exclude Insurance and Some Others for HHMemberName[3]

	Freq.	%
1 Yes	57	27.94
2 No	114	55.88
997 Don't Know	31	15.20
999 Refused to Answer	2	0.98
Total	204	100.00

ga006_w4_1_4_: Exclude Insurance and Some Others for HHMemberName[4]

	Freq.	%
1 Yes	19	25.68
2 No	34	45.95
997 Don't Know	21	28.38
Total	74	100.00

ga006_w4_1_5_: Exclude Insurance and Some Others for HHMemberName[5]

	Freq.	%
1 Yes	3	18.75
2 No	12	75.00
997 Don't Know	1	6.25
Total	16	100.00

ga006_w4_1_6_: Exclude Insurance and Some Others for HHMemberName[6]

	Freq.	%
1 Yes	1	9.09
2 No	8	72.73
997 Don't Know	2	18.18
Total	11	100.00

ga006_w4_1_7_: Exclude Insurance and Some Others for HHMemberName[7]

	Freq.	%
2 No	2	66.67
997 Don't Know	1	33.33
Total	3	100.00

ga006_w4_1_8_: Exclude Insurance and Some Others for HHMemberName[8]

	Freq.	%
1 Yes	1	50.00
999 Refused to Answer	1	50.00
Total	2	100.00

ga006_w4_2_1_: Total Excluded Amount for HHMemberName[1]

	Freq.	%
1 Yuan/Year	122	6.78
2 Yuan/Month	181	10.06
3 Percent of Wage	35	1.94
4 No	807	44.83
997 Don't Know	647	35.94
999 Refused to Answer	8	0.44
Total	1,800	100.00

ga006_w4_2_2_: Total Excluded Amount for HHMemberName[2]

	Freq.	%
1 Yuan/Year	52	6.27
2 Yuan/Month	88	10.62
3 Percent of Wage	18	2.17
4 No	374	45.11
997 Don't Know	296	35.71
999 Refused to Answer	1	0.12
Total	829	100.00

ga006_w4_2_3_: Total Excluded Amount for HHMemberName[3]

	Freq.	%
1 Yuan/Year	21	10.40
2 Yuan/Month	19	9.41
3 Percent of Wage	3	1.49
4 No	94	46.53

	Freq.	%
997 Don't Know	65	32.18
Total	202	100.00

ga006_w4_2_4_: Total Excluded Amount for HHMemberName[4]

	Freq.	%
1 Yuan/Year	3	4.05
2 Yuan/Month	8	10.81
4 No	32	43.24
997 Don't Know	31	41.89
Total	74	100.00

ga006_w4_2_5_: Total Excluded Amount for HHMemberName[5]

	Freq.	%
2 Yuan/Month	1	6.25
4 No	12	75.00
997 Don't Know	3	18.75
Total	16	100.00

ga006_w4_2_6_: Total Excluded Amount for HHMemberName[6]

	Freq.	%
2 Yuan/Month	1	9.09
4 No	8	72.73
997 Don't Know	2	18.18
Total	11	100.00

ga006_w4_2_7_: Total Excluded Amount for HHMemberName[7]

	Freq.	%
4 No	2	66.67
997 Don't Know	1	33.33
Total	3	100.00

ga006_w4_2_8_: Total Excluded Amount for HHMemberName[8]

	Freq.	%
2 Yuan/Month	1	100.00
Total	1	100.00

ga006_w4_2a_1_: Yuan/Year for HHMemberName[1]

Mean	SD	Min	Max	Obs
5,884.15	11,466.96	0.00	70,000.00	135

ga006_w4_2a_2_: Yuan/Year for HHMemberName[2]

Mean	SD	Min	Max	Obs
8,530.18	27,437.89	0.00	200,000.00	55

ga006_w4_2a_3_: Yuan/Year for HHMemberName[3]

Mean	SD	Min	Max	Obs
7,971.43	10,821.56	0.00	40,000.00	21

ga006_w4_2a_4_: Yuan/Year for HHMemberName[4]

Mean	SD	Min	Max	Obs
2,333.33	4,041.45	0.00	7,000.00	3

ga006_w4_2a_5_: Yuan/Year for HHMemberName[5]

Mean	SD	Min	Max	Obs
0.00	.	0.00	0.00	1

ga006_w4_2b_1_: Yuan/Month for HHMemberName[1]

Mean	SD	Min	Max	Obs
754.61	761.70	0.00	5,000.00	179

ga006_w4_2b_2_: Yuan/Month for HHMemberName[2]

Mean	SD	Min	Max	Obs
784.73	748.41	0.00	4,000.00	88

ga006_w4_2b_3_: Yuan/Month for HHMemberName[3]

Mean	SD	Min	Max	Obs
878.95	745.60	200.00	3,000.00	19

ga006_w4_2b_4_: Yuan/Month for HHMemberName[4]

Mean	SD	Min	Max	Obs
475.00	236.04	300.00	1,000.00	8

ga006_w4_2b_5_: Yuan/Month for HHMemberName[5]

Mean	SD	Min	Max	Obs
400.00	.	400.00	400.00	1

ga006_w4_2b_6_: Yuan/Month for HHMemberName[6]

Mean	SD	Min	Max	Obs
500.00	.	500.00	500.00	1

ga006_w4_2b_8_: Yuan/Month for HHMemberName[8]

Mean	SD	Min	Max	Obs
300.00	.	300.00	300.00	1

ga006_w4_2c_1_: Percent of Wage for HHMemberName[1]

Mean	SD	Min	Max	Obs
15.91	13.89	0.00	60.00	37

ga006_w4_2c_2_: Percent of Wage for HHMemberName[2]

Mean	SD	Min	Max	Obs
18.51	21.34	3.00	100.00	18

ga006_w4_2c_3_: Percent of Wage for HHMemberName[3]

Mean	SD	Min	Max	Obs
12.67	4.04	8.00	15.00	3

ga006_w4_2_1__min: Min Bracket of ga006_w4_2_1_

Mean	SD	Min	Max	Obs
916.00	694.89	300.00	3,000.00	250

ga006_w4_2_1__max: Max Bracket of ga006_w4_2_1_

Mean	SD	Min	Max	Obs
829.57	688.23	300.00	3,000.00	345

ga006_w4_2_2__min: Min Bracket of ga006_w4_2_2_

Mean	SD	Min	Max	Obs
1,000.00	750.18	300.00	3,000.00	95

ga006_w4_2_2__max: Max Bracket of ga006_w4_2_2_

Mean	SD	Min	Max	Obs
859.86	690.57	300.00	3,000.00	142

ga006_w4_2_3__min: Min Bracket of ga006_w4_2_3_

Mean	SD	Min	Max	Obs
1,022.22	881.55	300.00	3,000.00	18

ga006_w4_2_3__max: Max Bracket of ga006_w4_2_3_

Mean	SD	Min	Max	Obs
950.00	929.16	300.00	3,000.00	28

ga006_w4_2_4__min: Min Bracket of ga006_w4_2_4_

Mean	SD	Min	Max	Obs
612.50	331.39	300.00	1,000.00	8

ga006_w4_2_4__max: Max Bracket of ga006_w4_2_4_

Mean	SD	Min	Max	Obs
608.33	508.94	300.00	2,000.00	12

ga006_w4_2_5__min: Min Bracket of ga006_w4_2_5_

Mean	SD	Min	Max	Obs
500.00	.	500.00	500.00	1

ga006_w4_2_5__max: Max Bracket of ga006_w4_2_5_

Mean	SD	Min	Max	Obs
400.00	141.42	300.00	500.00	2

ga006_w4_2_6__min: Min Bracket of ga006_w4_2_6_

No Observations

ga006_w4_2_6__max: Max Bracket of ga006_w4_2_6_

No Observations

ga006_w4_2_7__min: Min Bracket of ga006_w4_2_7_

No Observations

ga006_w4_2_7__max: Max Bracket of ga006_w4_2_7_

No Observations

ga006_w4_2_8__min: Min Bracket of ga006_w4_2_8_

No Observations

ga006_w4_2_8__max: Max Bracket of ga006_w4_2_8_

No Observations

ga007_w4_1__s1: Pension HHMemberName[1] Receive

	Freq.	%
0 No	4,664	87.10
1 Yes	691	12.90
Total	5,355	100.00

ga007_w4_2__s1: Pension HHMemberName[2] Receive

	Freq.	%
0 No	2,777	95.23
1 Yes	139	4.77
Total	2,916	100.00

ga007_w4_3__s1: Pension HHMemberName[3] Receive

	Freq.	%
0 No	1,670	98.88
1 Yes	19	1.12
Total	1,689	100.00

ga007_w4_4__s1: Pension HHMemberName[4] Receive

	Freq.	%
0 No	885	99.66
1 Yes	3	0.34
Total	888	100.00

ga007_w4_5__s1: Pension HHMemberName[5] Receive

	Freq.	%
0 No	11,543	99.99
1 Yes	1	0.01
Total	11,544	100.00

ga007_w4_6__s1: Pension HHMemberName[6] Receive

	Freq.	%
0 No	11,543	99.99
1 Yes	1	0.01
Total	11,544	100.00

ga007_w4_7__s1: Pension HHMemberName[7] Receive

	Freq.	%
0 No	11,543	99.99
1 Yes	1	0.01
Total	11,544	100.00

ga007_w4_8__s1: Pension HHMemberName[8] Receive

	Freq.	%
0 No	11,544	100.00
Total	11,544	100.00

ga007_w4_9__s1: Pension HHMemberName[9] Receive

	Freq.	%
0 No	11,544	100.00
Total	11,544	100.00

ga007_w4_10__s1: Pension HHMemberName[10] Receive

	Freq.	%
0 No	11,544	100.00
Total	11,544	100.00

ga007_w4_11__s1: Pension HHMemberName[11] Receive

	Freq.	%
0 No	11,544	100.00
Total	11,544	100.00

ga007_w4_12__s1: Pension HHMemberName[12] Receive

	Freq.	%
0 No	11,544	100.00
Total	11,544	100.00

ga007_w4_1__s2: Unemployment Compensation HHMemberName[1] Receive

	Freq.	%
0 No	5,336	99.65
2 Yes	19	0.35
Total	5,355	100.00

ga007_w4_2__s2: Unemployment Compensation HHMemberName[2] Receive

	Freq.	%

	Freq.	%
0 No	2,912	99.86
2 Yes	4	0.14
Total	2,916	100.00

ga007_w4_3__s2: Unemployment Compensation HHMemberName[3] Receive

	Freq.	%
0 No	1,688	99.94
2 Yes	1	0.06
Total	1,689	100.00

ga007_w4_4__s2: Unemployment Compensation HHMemberName[4] Receive

	Freq.	%
0 No	888	100.00
Total	888	100.00

ga007_w4_5__s2: Unemployment Compensation HHMemberName[5] Receive

	Freq.	%
0 No	11,544	100.00
Total	11,544	100.00

ga007_w4_6__s2: Unemployment Compensation HHMemberName[6] Receive

	Freq.	%
0 No	11,544	100.00
Total	11,544	100.00

ga007_w4_7__s2: Unemployment Compensation HHMemberName[7] Receive

	Freq.	%
0 No	11,544	100.00
Total	11,544	100.00

ga007_w4_8__s2: Unemployment Compensation HHMemberName[8] Receive

	Freq.	%
0 No	11,544	100.00
Total	11,544	100.00

ga007_w4_9__s2: Unemployment Compensation HHMemberName[9] Receive

	Freq.	%
0 No	11,544	100.00
Total	11,544	100.00

ga007_w4_10__s2: Unemployment Compensation HHMemberName[10] Receive

	Freq.	%
0 No	11,544	100.00
Total	11,544	100.00

ga007_w4_11__s2: Unemployment Compensation HHMemberName[11] Receive

	Freq.	%
0 No	11,544	100.00
Total	11,544	100.00

ga007_w4_12__s2: Unemployment Compensation HHMemberName[12] Receive

	Freq.	%
0 No	11,544	100.00
Total	11,544	100.00

ga007_w4_1__s3: Pension Voucher HHMemberName[1] Receive

	Freq.	%
0 No	5,329	99.51
3 Yes	26	0.49
Total	5,355	100.00

ga007_w4_2__s3: Pension Voucher HHMemberName[2] Receive

	Freq.	%
0 No	2,914	99.93
3 Yes	2	0.07
Total	2,916	100.00

ga007_w4_3__s3: Pension Voucher HHMemberName[3] Receive

	Freq.	%
0 No	1,689	100.00
Total	1,689	100.00

ga007_w4_4__s3: Pension Voucher HHMemberName[4] Receive

	Freq.	%
0 No	888	100.00
Total	888	100.00

ga007_w4_5__s3: Pension Voucher HHMemberName[5] Receive

	Freq.	%
0 No	11,544	100.00
Total	11,544	100.00

ga007_w4_6__s3: Pension Voucher HHMemberName[6] Receive

	Freq.	%
0 No	11,544	100.00
Total	11,544	100.00

ga007_w4_7__s3: Pension Voucher HHMemberName[7] Receive

	Freq.	%
0 No	11,544	100.00
Total	11,544	100.00

ga007_w4_8__s3: Pension Voucher HHMemberName[8] Receive

	Freq.	%
0 No	11,544	100.00
Total	11,544	100.00

ga007_w4_9__s3: Pension Voucher HHMemberName[9] Receive

	Freq.	%
0 No	11,544	100.00
Total	11,544	100.00

ga007_w4_10__s3: Pension Voucher HHMemberName[10] Receive

	Freq.	%
0 No	11,544	100.00
Total	11,544	100.00

ga007_w4_11__s3: Pension Voucher HHMemberName[11] Receive

	Freq.	%
0 No	11,544	100.00
Total	11,544	100.00

ga007_w4_12__s3: Pension Voucher HHMemberName[12] Receive

	Freq.	%
0 No	11,544	100.00
Total	11,544	100.00

ga007_w4_1__s4: Pension Subsidy for the Oldest Old HHMemberName[1] Receive

	Freq.	%
0 No	5,147	96.12
4 Yes	208	3.88

Total	5,355	100.00

ga007_w4_2__s4: Pension Subsidy for the Oldest Old HHMemberName[2] Receive

	Freq.	%
0 No	2,897	99.35
4 Yes	19	0.65
Total	2,916	100.00

ga007_w4_3__s4: Pension Subsidy for the Oldest Old HHMemberName[3] Receive

	Freq.	%
0 No	1,687	99.88
4 Yes	2	0.12
Total	1,689	100.00

ga007_w4_4__s4: Pension Subsidy for the Oldest Old HHMemberName[4] Receive

	Freq.	%
0 No	887	99.89
4 Yes	1	0.11
Total	888	100.00

ga007_w4_5__s4: Pension Subsidy for the Oldest Old HHMemberName[5] Receive

	Freq.	%
0 No	11,544	100.00
Total	11,544	100.00

ga007_w4_6__s4: Pension Subsidy for the Oldest Old HHMemberName[6] Receive

	Freq.	%
0 No	11,544	100.00
Total	11,544	100.00

ga007_w4_7__s4: Pension Subsidy for the Oldest Old HHMemberName[7] Receive

	Freq.	%
0 No	11,544	100.00
Total	11,544	100.00

ga007_w4_8__s4: Pension Subsidy for the Oldest Old HHMemberName[8] Receive

	Freq.	%
0 No	11,544	100.00
Total	11,544	100.00

ga007_w4_9__s4: Pension Subsidy for the Oldest Old HHMemberName[9] Receive

	Freq.	%
0 No	11,544	100.00
Total	11,544	100.00

ga007_w4_10__s4: Pension Subsidy for the Oldest Old HHMemberName[10] Receive

	Freq.	%
0 No	11,544	100.00
Total	11,544	100.00

ga007_w4_11__s4: Pension Subsidy for the Oldest Old HHMemberName[11] Receive

	Freq.	%
0 No	11,544	100.00
Total	11,544	100.00

ga007_w4_12__s4: Pension Subsidy for the Oldest Old HHMemberName[12] Receive

	Freq.	%
0 No	11,544	100.00
Total	11,544	100.00

ga007_w4_1__s5: Workers' Industrial Accident Compensation HHMemberName[1] Receive

	Freq.	%
0 No	5,332	99.57
5 Yes	23	0.43
Total	5,355	100.00

ga007_w4_2__s5: Workers' Industrial Accident Compensation HHMemberName[2] Receive

	Freq.	%
0 No	2,911	99.83
5 Yes	5	0.17
Total	2,916	100.00

ga007_w4_3__s5: Workers' Industrial Accident Compensation HHMemberName[3] Receive

	Freq.	%
0 No	1,688	99.94
5 Yes	1	0.06
Total	1,689	100.00

ga007_w4_4__s5: Workers' Industrial Accident Compensation HHMemberName[4] Receive

	Freq.	%
0 No	886	99.77
5 Yes	2	0.23
Total	888	100.00

ga007_w4_5__s5: Workers' Industrial Accident Compensation HHMemberName[5] Receive

	Freq.	%
0 No	11,544	100.00
Total	11,544	100.00

ga007_w4_6__s5: Workers' Industrial Accident Compensation HHMemberName[6] Receive

	Freq.	%
0 No	11,544	100.00
Total	11,544	100.00

ga007_w4_7__s5: Workers' Industrial Accident Compensation HHMemberName[7] Receive

	Freq.	%
0 No	11,544	100.00
Total	11,544	100.00

ga007_w4_8__s5: Workers' Industrial Accident Compensation HHMemberName[8] Receive

	Freq.	%
0 No	11,544	100.00
Total	11,544	100.00

ga007_w4_9__s5: Workers' Industrial Accident Compensation HHMemberName[9] Receive

	Freq.	%
0 No	11,544	100.00
Total	11,544	100.00

ga007_w4_10__s5: Workers' Industrial Accident Compensation HHMemberName[10] Receive

	Freq.	%
0 No	11,544	100.00
Total	11,544	100.00

ga007_w4_11__s5: Workers' Industrial Accident Compensation HHMemberName[11] Receive

	Freq.	%
0 No	11,544	100.00
Total	11,544	100.00

ga007_w4_12__s5: Workers' Industrial Accident Compensation HHMemberName[12] Receive

	Freq.	%
0 No	11,544	100.00
Total	11,544	100.00

ga007_w4_1__s6: Elderly Family Planning Subsidy HHMemberName[1] Receive

	Freq.	%
0 No	5,323	99.40
6 Yes	32	0.60
Total	5,355	100.00

ga007_w4_2__s6: Elderly Family Planning Subsidy HHMemberName[2] Receive

	Freq.	%
0 No	2,901	99.49
6 Yes	15	0.51
Total	2,916	100.00

ga007_w4_3__s6: Elderly Family Planning Subsidy HHMemberName[3] Receive

	Freq.	%
0 No	1,687	99.88
6 Yes	2	0.12
Total	1,689	100.00

ga007_w4_4__s6: Elderly Family Planning Subsidy HHMemberName[4] Receive

	Freq.	%
0 No	888	100.00
Total	888	100.00

ga007_w4_5__s6: Elderly Family Planning Subsidy HHMemberName[5] Receive

	Freq.	%
0 No	11,544	100.00
Total	11,544	100.00

ga007_w4_6__s6: Elderly Family Planning Subsidy HHMemberName[6] Receive

	Freq.	%
0 No	11,544	100.00
Total	11,544	100.00

ga007_w4_7__s6: Elderly Family Planning Subsidy HHMemberName[7] Receive

	Freq.	%
0 No	11,544	100.00
Total	11,544	100.00

ga007_w4_8__s6: Elderly Family Planning Subsidy HHMemberName[8] Receive

	Freq.	%
0 No	11,544	100.00
Total	11,544	100.00

ga007_w4_9__s6: Elderly Family Planning Subsidy HHMemberName[9] Receive

	Freq.	%
0 No	11,544	100.00
Total	11,544	100.00

ga007_w4_10__s6: Elderly Family Planning Subsidy HHMemberName[10] Receive

	Freq.	%
0 No	11,544	100.00
Total	11,544	100.00

ga007_w4_11__s6: Elderly Family Planning Subsidy HHMemberName[11] Receive

	Freq.	%
0 No	11,544	100.00
Total	11,544	100.00

ga007_w4_12__s6: Elderly Family Planning Subsidy HHMemberName[12] Receive

	Freq.	%
0 No	11,544	100.00

Total	11,544	100.00

ga007_w4_1__s7: Medical Aid HHMemberName[1] Receive

	Freq.	%
0 No	5,319	99.33
7 Yes	36	0.67
Total	5,355	100.00

ga007_w4_2__s7: Medical Aid HHMemberName[2] Receive

	Freq.	%
0 No	2,906	99.66
7 Yes	10	0.34
Total	2,916	100.00

ga007_w4_3__s7: Medical Aid HHMemberName[3] Receive

	Freq.	%
0 No	1,682	99.59
7 Yes	7	0.41
Total	1,689	100.00

ga007_w4_4__s7: Medical Aid HHMemberName[4] Receive

	Freq.	%
0 No	887	99.89
7 Yes	1	0.11
Total	888	100.00

ga007_w4_5__s7: Medical Aid HHMemberName[5] Receive

	Freq.	%
0 No	11,543	99.99
7 Yes	1	0.01
Total	11,544	100.00

ga007_w4_6__s7: Medical Aid HHMemberName[6] Receive

	Freq.	%
0 No	11,544	100.00
Total	11,544	100.00

ga007_w4_7__s7: Medical Aid HHMemberName[7] Receive

	Freq.	%
0 No	11,544	100.00
Total	11,544	100.00

ga007_w4_8__s7: Medical Aid HHMemberName[8] Receive

	Freq.	%
0 No	11,544	100.00
Total	11,544	100.00

ga007_w4_9__s7: Medical Aid HHMemberName[9] Receive

	Freq.	%
0 No	11,544	100.00
Total	11,544	100.00

ga007_w4_10__s7: Medical Aid HHMemberName[10] Receive

	Freq.	%
0 No	11,544	100.00
Total	11,544	100.00

ga007_w4_11__s7: Medical Aid HHMemberName[11] Receive

	Freq.	%
0 No	11,544	100.00
Total	11,544	100.00

ga007_w4_12__s7: Medical Aid HHMemberName[12] Receive

	Freq.	%
0 No	11,544	100.00
Total	11,544	100.00

ga007_w4_1__s8: Other Government Subsidy HHMemberName[1] Receive

	Freq.	%
0 No	5,015	93.65
8 Yes	340	6.35
Total	5,355	100.00

ga007_w4_2__s8: Other Government Subsidy HHMemberName[2] Receive

	Freq.	%
0 No	2,815	96.54
8 Yes	101	3.46
Total	2,916	100.00

ga007_w4_3__s8: Other Government Subsidy HHMemberName[3] Receive

	Freq.	%

	Freq.	%
0 No	1,639	97.04
8 Yes	50	2.96
Total	1,689	100.00

ga007_w4_4__s8: Other Government Subsidy HHMemberName[4] Receive

	Freq.	%
0 No	861	96.96
8 Yes	27	3.04
Total	888	100.00

ga007_w4_5__s8: Other Government Subsidy HHMemberName[5] Receive

	Freq.	%
0 No	11,536	99.93
8 Yes	8	0.07
Total	11,544	100.00

ga007_w4_6__s8: Other Government Subsidy HHMemberName[6] Receive

	Freq.	%
0 No	11,540	99.97
8 Yes	4	0.03
Total	11,544	100.00

ga007_w4_7__s8: Other Government Subsidy HHMemberName[7] Receive

	Freq.	%
0 No	11,544	100.00
Total	11,544	100.00

ga007_w4_8__s8: Other Government Subsidy HHMemberName[8] Receive

	Freq.	%
0 No	11,544	100.00
Total	11,544	100.00

ga007_w4_9__s8: Other Government Subsidy HHMemberName[9] Receive

	Freq.	%
0 No	11,544	100.00
Total	11,544	100.00

ga007_w4_10__s8: Other Government Subsidy HHMemberName[10] Receive

	Freq.	%
0 No	11,544	100.00
Total	11,544	100.00

ga007_w4_11__s8: Other Government Subsidy HHMemberName[11] Receive

	Freq.	%
0 No	11,544	100.00
Total	11,544	100.00

ga007_w4_12__s8: Other Government Subsidy HHMemberName[12] Receive

	Freq.	%
0 No	11,544	100.00
Total	11,544	100.00

ga007_w4_1__s9: Other Income Source HHMemberName[1] Receive

	Freq.	%
0 No	5,311	99.18
9 Yes	44	0.82
Total	5,355	100.00

ga007_w4_2__s9: Other Income Source HHMemberName[2] Receive

	Freq.	%
0 No	2,907	99.69
9 Yes	9	0.31
Total	2,916	100.00

ga007_w4_3__s9: Other Income Source HHMemberName[3] Receive

	Freq.	%
0 No	1,681	99.53
9 Yes	8	0.47
Total	1,689	100.00

ga007_w4_4__s9: Other Income Source HHMemberName[4] Receive

	Freq.	%
0 No	884	99.55
9 Yes	4	0.45
Total	888	100.00

ga007_w4_5__s9: Other Income Source HHMemberName[5] Receive

	Freq.	%
0 No	11,543	99.99
9 Yes	1	0.01
Total	11,544	100.00

ga007_w4_6__s9: Other Income Source HHMemberName[6] Receive

	Freq.	%
0 No	11,544	100.00
Total	11,544	100.00

ga007_w4_7__s9: Other Income Source HHMemberName[7] Receive

	Freq.	%
0 No	11,544	100.00
Total	11,544	100.00

ga007_w4_8__s9: Other Income Source HHMemberName[8] Receive

	Freq.	%
0 No	11,544	100.00
Total	11,544	100.00

ga007_w4_9__s9: Other Income Source HHMemberName[9] Receive

	Freq.	%
0 No	11,544	100.00
Total	11,544	100.00

ga007_w4_10__s9: Other Income Source HHMemberName[10] Receive

	Freq.	%
0 No	11,544	100.00
Total	11,544	100.00

ga007_w4_11__s9: Other Income Source HHMemberName[11] Receive

	Freq.	%
0 No	11,544	100.00
Total	11,544	100.00

ga007_w4_12__s9: Other Income Source HHMemberName[12] Receive

	Freq.	%
0 No	11,544	100.00
Total	11,544	100.00

ga007_w4_1__s10: None of the Above HHMemberName[1] Receive

	Freq.	%
0 No	1,124	20.99
10 Yes	4,231	79.01
Total	5,355	100.00

ga007_w4_2__s10: None of the Above HHMemberName[2] Receive

	Freq.	%
0 No	267	9.16
10 Yes	2,649	90.84
Total	2,916	100.00

ga007_w4_3__s10: None of the Above HHMemberName[3] Receive

	Freq.	%
0 No	83	4.91
10 Yes	1,606	95.09
Total	1,689	100.00

ga007_w4_4__s10: None of the Above HHMemberName[4] Receive

	Freq.	%
0 No	35	3.94
10 Yes	853	96.06
Total	888	100.00

ga007_w4_5__s10: None of the Above HHMemberName[5] Receive

	Freq.	%
0 No	11,257	97.51
10 Yes	287	2.49
Total	11,544	100.00

ga007_w4_6__s10: None of the Above HHMemberName[6] Receive

	Freq.	%
0 No	11,415	98.88
10 Yes	129	1.12
Total	11,544	100.00

ga007_w4_7__s10: None of the Above HHMemberName[7] Receive

	Freq.	%
0 No	11,479	99.44
10 Yes	65	0.56
Total	11,544	100.00

ga007_w4_8__s10: None of the Above HHMemberName[8] Receive

	Freq.	%
0 No	11,515	99.75
10 Yes	29	0.25
Total	11,544	100.00

ga007_w4_9__s10: None of the Above HHMemberName[9] Receive

	Freq.	%
0 No	11,525	99.84
10 Yes	19	0.16
Total	11,544	100.00

ga007_w4_10__s10: None of the Above HHMemberName[10] Receive

	Freq.	%
0 No	11,536	99.93
10 Yes	8	0.07
Total	11,544	100.00

ga007_w4_11__s10: None of the Above HHMemberName[11] Receive

	Freq.	%
0 No	11,541	99.97
10 Yes	3	0.03
Total	11,544	100.00

ga007_w4_12__s10: None of the Above HHMemberName[12] Receive

	Freq.	%
0 No	11,543	99.99
10 Yes	1	0.01
Total	11,544	100.00

ga007_w4_1_1_: Pension Amount HHMemberName[1] Receive

Mean	SD	Min	Max	Obs
9,360.95	21,660.06	0.00	400,003.00	678

ga007_w4_1_2_: Pension Amount HHMemberName[2] Receive

Mean	SD	Min	Max	Obs
8,368.27	11,594.15	0.00	44,000.00	132

ga007_w4_1_3_: Pension Amount HHMemberName[3] Receive

Mean	SD	Min	Max	Obs
3,788.13	6,592.06	600.00	24,000.00	16

ga007_w4_1_4_: Pension Amount HHMemberName[4] Receive

Mean	SD	Min	Max	Obs
866.67	577.35	200.00	1,200.00	3

ga007_w4_1_5_: Pension Amount HHMemberName[5] Receive

Mean	SD	Min	Max	Obs
1,200.00	.	1,200.00	1,200.00	1

ga007_w4_1_6_: Pension Amount HHMemberName[6] Receive

Mean	SD	Min	Max	Obs
1,200.00	.	1,200.00	1,200.00	1

ga007_w4_1_7_: Pension Amount HHMemberName[7] Receive

Mean	SD	Min	Max	Obs
1,200.00	.	1,200.00	1,200.00	1

ga007_w4_2_1_: Unemployment Compensation Amount HHMemberName[1] Receive

Mean	SD	Min	Max	Obs
5,226.15	8,027.95	0.00	24,000.00	13

ga007_w4_2_2_: Unemployment Compensation Amount HHMemberName[2] Receive

Mean	SD	Min	Max	Obs
2,880.00	3,711.06	0.00	8,400.00	5

ga007_w4_2_3_: Unemployment Compensation Amount HHMemberName[3] Receive

No Observations

ga007_w4_3_1_: Pension Voucher Amount HHMemberName[1] Receive

Mean	SD	Min	Max	Obs
958.96	1,086.64	0.00	4,000.00	23

ga007_w4_3_2_: Pension Voucher Amount HHMemberName[2] Receive

Mean	SD	Min	Max	Obs
50.00	86.60	0.00	150.00	3

ga007_w4_4_1_: Pension Subsidy for the Oldest Old Amount HHMemberName[1] Receive

Mean	SD	Min	Max	Obs
1,005.87	874.16	0.00	6,000.00	191

ga007_w4_4_2_: Pension Subsidy for the Oldest Old Amount HHMemberName[2] Receive

Mean	SD	Min	Max	Obs
1,088.89	1,369.28	0.00	6,000.00	18

ga007_w4_4_3_: Pension Subsidy for the Oldest Old Amount HHMemberName[3] Receive

Mean	SD	Min	Max	Obs
1,600.00	565.69	1,200.00	2,000.00	2

ga007_w4_4_4_: Pension Subsidy for the Oldest Old Amount HHMemberName[4] Receive

Mean	SD	Min	Max	Obs
150.00	.	150.00	150.00	1

ga007_w4_5_1_: Workers' Industrial Accident Compensation Amount HHMember-Name[1] Receive

Mean	SD	Min	Max	Obs
871.76	497.09	0.00	2,000.00	17

ga007_w4_5_2_: Workers' Industrial Accident Compensation Amount HHMember-Name[2] Receive

Mean	SD	Min	Max	Obs
2,412.00	4,699.65	0.00	10,800.00	5

ga007_w4_5_3_: Workers' Industrial Accident Compensation Amount HHMember-Name[3] Receive

Mean	SD	Min	Max	Obs
800.00	.	800.00	800.00	1

ga007_w4_5_4_: Workers' Industrial Accident Compensation Amount HHMember-Name[4] Receive

Mean	SD	Min	Max	Obs
690.00	42.43	660.00	720.00	2

ga007_w4_6_1_: Elderly Family Planning Subsidy Amount HHMemberName[1] Receive

Mean	SD	Min	Max	Obs
772.23	755.23	0.00	3,000.00	30

ga007_w4_6_2_: Elderly Family Planning Subsidy Amount HHMemberName[2] Receive

Mean	SD	Min	Max	Obs
882.60	2,019.10	0.00	8,000.00	15

ga007_w4_6_3_: Elderly Family Planning Subsidy Amount HHMemberName[3] Receive

Mean	SD	Min	Max	Obs
90.00	.	90.00	90.00	1

ga007_w4_7_1_: Medical Aid Amount HHMemberName[1] Receive

Mean	SD	Min	Max	Obs
3,327.93	5,086.54	0.00	20,000.00	29

ga007_w4_7_2_: Medical Aid Amount HHMemberName[2] Receive

Mean	SD	Min	Max	Obs
941.82	1,107.36	0.00	3,000.00	11

ga007_w4_7_3_: Medical Aid Amount HHMemberName[3] Receive

Mean	SD	Min	Max	Obs
4,254.29	6,392.70	0.00	15,000.00	7

ga007_w4_7_4_: Medical Aid Amount HHMemberName[4] Receive

Mean	SD	Min	Max	Obs
80,000.00	.	80,000.00	80,000.00	1

ga007_w4_7_5_: Medical Aid Amount HHMemberName[5] Receive

Mean	SD	Min	Max	Obs
2,000.00	.	2,000.00	2,000.00	1

ga007_w4_8_1_: Other Government Subsidy Amount HHMemberName[1] Receive

Mean	SD	Min	Max	Obs
2,588.19	4,510.55	0.00	48,000.00	331

ga007_w4_8_2_: Other Government Subsidy Amount HHMemberName[2] Receive

Mean	SD	Min	Max	Obs
1,809.28	2,552.40	0.00	20,000.00	97

ga007_w4_8_3_: Other Government Subsidy Amount HHMemberName[3] Receive

Mean	SD	Min	Max	Obs
3,492.63	16,692.41	0.00	120,000.00	51

ga007_w4_8_4_: Other Government Subsidy Amount HHMemberName[4] Receive

Mean	SD	Min	Max	Obs
894.44	947.31	50.00	5,000.00	27

ga007_w4_8_5_: Other Government Subsidy Amount HHMemberName[5] Receive

Mean	SD	Min	Max	Obs
697.50	531.89	180.00	1,800.00	8

ga007_w4_8_6_: Other Government Subsidy Amount HHMemberName[6] Receive

Mean	SD	Min	Max	Obs
800.00	355.90	500.00	1,200.00	4

ga007_w4_9_1_: Other Income Source Amount HHMemberName[1] Receive

Mean	SD	Min	Max	Obs
1,602.78	1,687.20	0.00	7,200.00	41

ga007_w4_9_2_: Other Income Source Amount HHMemberName[2] Receive

Mean	SD	Min	Max	Obs
625.88	678.90	0.00	2,100.00	8

ga007_w4_9_3_: Other Income Source Amount HHMemberName[3] Receive

Mean	SD	Min	Max	Obs
1,700.00	1,894.54	150.00	6,000.00	8

ga007_w4_9_4_: Other Income Source Amount HHMemberName[4] Receive

Mean	SD	Min	Max	Obs

1,125.00	629.15	500.00	2,000.00	4

ga007_w4_9_5_: Other Income Source Amount HHMemberName[5] Receive

Mean	SD	Min	Max	Obs
20.00	.	20.00	20.00	1

ga008_w4_1_1__min: Min Bracket of ga008_w4_1_1_

Mean	SD	Min	Max	Obs
1,385.71	889.13	400.00	3,000.00	14

ga008_w4_1_1__max: Max Bracket of ga008_w4_1_1_

Mean	SD	Min	Max	Obs
3,160.00	4,776.25	400.00	20,000.00	15

ga008_w4_1_2__min: Min Bracket of ga008_w4_1_2_

Mean	SD	Min	Max	Obs
866.67	230.94	600.00	1,000.00	3

ga008_w4_1_2__max: Max Bracket of ga008_w4_1_2_

Mean	SD	Min	Max	Obs
1,250.00	1,193.04	400.00	3,000.00	4

ga008_w4_1_3__min: Min Bracket of ga008_w4_1_3_

Mean	SD	Min	Max	Obs
2,000.00	1,414.21	1,000.00	3,000.00	2

ga008_w4_1_3__max: Max Bracket of ga008_w4_1_3_

Mean	SD	Min	Max	Obs
3,000.00	0.00	3,000.00	3,000.00	2

ga012_w4_1_1_: Inherited for HHMemberName[1]

	Freq.	%
1 Yes	102	1.90
2 No	5,181	96.73
997 Don't Know	65	1.21
999 Refused to Answer	8	0.15
Total	5,356	100.00

ga012_w4_1_2_: Inherited for HHMemberName[2]

	Freq.	%
1 Yes	10	0.34
2 No	2,855	97.91
997 Don't Know	43	1.47
999 Refused to Answer	8	0.27
Total	2,916	100.00

ga012_w4_1_3_: Inherited for HHMemberName[3]

	Freq.	%
1 Yes	1	0.06
2 No	1,675	99.17
997 Don't Know	9	0.53
999 Refused to Answer	4	0.24
Total	1,689	100.00

ga012_w4_1_4_: Inherited for HHMemberName[4]

	Freq.	%
2 No	875	98.54
997 Don't Know	9	1.01
999 Refused to Answer	4	0.45
Total	888	100.00

ga012_w4_1_5_: Inherited for HHMemberName[5]

	Freq.	%
2 No	293	98.32
997 Don't Know	5	1.68
Total	298	100.00

ga012_w4_1_6_: Inherited for HHMemberName[6]

	Freq.	%
2 No	133	99.25
997 Don't Know	1	0.75
Total	134	100.00

ga012_w4_1_7_: Inherited for HHMemberName[7]

	Freq.	%
2 No	65	98.48
997 Don't Know	1	1.52
Total	66	100.00

ga012_w4_1_8_: Inherited for HHMemberName[8]

	Freq.	%
2 No	28	96.55
997 Don't Know	1	3.45
Total	29	100.00

ga012_w4_1_9_: Inherited for HHMemberName[9]

	Freq.	%
2 No	18	94.74
997 Don't Know	1	5.26
Total	19	100.00

ga012_w4_1_10_: Inherited for HHMemberName[10]

	Freq.	%
2 No	8	100.00
Total	8	100.00

ga012_w4_1_11_: Inherited for HHMemberName[11]

	Freq.	%
2 No	3	100.00
Total	3	100.00

ga012_w4_1_12_: Inherited for HHMemberName[12]

	Freq.	%
2 No	1	100.00
Total	1	100.00

ga012_w4_1_1_1_: Times for HHMemberName[1]

Mean	SD	Min	Max	Obs
1.02	0.13	1.00	2.00	112

ga012_w4_1_1_2_: Times for HHMemberName[2]

Mean	SD	Min	Max	Obs
1.09	0.30	1.00	2.00	11

ga012_w4_1_1_3_: Times for HHMemberName[3]

Mean	SD	Min	Max	Obs
1.50	0.71	1.00	2.00	2

ga012_w4_2_1_1_: Amount Inherited for HHMemberName[1]

Mean	SD	Min	Max	Obs
75,781.41	196,865.71	0.00	1,500,000.00	93

ga012_w4_2_1_2_: Amount Inherited for HHMemberName[2]

Mean	SD	Min	Max	Obs
21,236.43	41,939.17	55.00	115,000.00	7

ga012_w4_2_1_3_: Amount Inherited for HHMemberName[3]

Mean	SD	Min	Max	Obs
200,000.00	.	200,000.00	200,000.00	1

ga012_w4_2_2_1_: Year Inherited for HHMemberName[1]

Mean	SD	Min	Max	Obs
2,003.23	16.25	1,940.00	2,018.00	98

ga012_w4_2_2_2_: Year Inherited for HHMemberName[2]

Mean	SD	Min	Max	Obs
2,008.75	14.32	1,975.00	2,016.00	8

ga012_w4_2_2_3_: Year Inherited for HHMemberName[3]

Mean	SD	Min	Max	Obs
2,016.00	.	2,016.00	2,016.00	1

ga012_w4_3_1_: From Whom Inherited for HHMemberName[1]

	Freq.	%
1 HHMemberName[1]'s Parents	86	86.00
2 HHMemberName[1]'s Parents in Law	5	5.00
4 HHMemberName[1]'s Relatives	2	2.00
5 Others	7	7.00
Total	100	100.00

ga012_w4_3_2_: From Whom Inherited for HHMemberName[2]

	Freq.	%
1 HHMemberName[2]'s Parents	5	55.56
2 HHMemberName[2]'s Parents in Law	1	11.11
4 HHMemberName[2]'s Relatives	2	22.22
5 Others	1	11.11
Total	9	100.00

ga012_w4_3_3_: From Whom Inherited for HHMemberName[3]

	Freq.	%
1 HHMemberName[3]'s Parents	1	100.00
Total	1	100.00

ga012_w4_4_1_1_: Amount Inherited for HHMemberName[1]

Mean	SD	Min	Max	Obs
13,000.00	7,071.07	8,000.00	18,000.00	2

ga012_w4_4_1_2_: Amount Inherited for HHMemberName[2]

Mean	SD	Min	Max	Obs
30,000.00	.	30,000.00	30,000.00	1

ga012_w4_4_2_1_: Amount Inherited for HHMemberName[1]

Mean	SD	Min	Max	Obs
8,000.00	1,414.21	7,000.00	9,000.00	2

ga012_w4_4_2_2_: Amount Inherited for HHMemberName[2]

Mean	SD	Min	Max	Obs
20,000.00	.	20,000.00	20,000.00	1

ga012_w4_4_3_1_: Year Inherited for HHMemberName[1]

Mean	SD	Min	Max	Obs
2,006.50	16.26	1,995.00	2,018.00	2

ga012_w4_4_3_2_: Year Inherited for HHMemberName[2]

Mean	SD	Min	Max	Obs
2,017.00	.	2,017.00	2,017.00	1

ga012_w4_5_1_: From Whom Inherited for HHMemberName[1]

	Freq.	%
4 HHMemberName[1]'s Relatives	1	50.00
5 Others	1	50.00
Total	2	100.00

ga012_w4_5_2_: From Whom Inherited for HHMemberName[2]

	Freq.	%

	Freq.	%
1 HHMemberName[2]'s Parents	1	100.00
Total	1	100.00

gb001_w4: Engage in Agricultural Work

	Freq.	%
1 Yes	5,740	49.79
2 No	5,788	50.21
Total	11,528	100.00

gb002_w4_s1: HHMemberName[1] Engaged in Agricultural Work

	Freq.	%
0 No	4,644	80.91
1 Yes	1,096	19.09
Total	5,740	100.00

gb002_w4_s2: HHMemberName[2] Engaged in Agricultural Work

	Freq.	%
0 No	5,158	89.86
2 Yes	582	10.14
Total	5,740	100.00

gb002_w4_s3: HHMemberName[3] Engaged in Agricultural Work

	Freq.	%
0 No	5,612	97.77
3 Yes	128	2.23
Total	5,740	100.00

gb002_w4_s4: HHMemberName[4] Engaged in Agricultural Work

	Freq.	%
0 No	5,679	98.94
4 Yes	61	1.06
Total	5,740	100.00

gb002_w4_s5: HHMemberName[5] Engaged in Agricultural Work

	Freq.	%
0 No	5,712	99.51
5 Yes	28	0.49
Total	5,740	100.00

gb002_w4_s6: HHMemberName[6] Engaged in Agricultural Work

	Freq.	%

	Freq.	%
0 No	5,727	99.77
6 Yes	13	0.23
Total	5,740	100.00

gb002_w4_s7: HHMemberName[7] Engaged in Agricultural Work

	Freq.	%
0 No	5,733	99.88
7 Yes	7	0.12
Total	5,740	100.00

gb002_w4_s8: HHMemberName[8] Engaged in Agricultural Work

	Freq.	%
0 No	5,738	99.97
8 Yes	2	0.03
Total	5,740	100.00

gb002_w4_s9: HHMemberName[9] Engaged in Agricultural Work

	Freq.	%
0 No	5,739	99.98
9 Yes	1	0.02
Total	5,740	100.00

gb002_w4_s97: HHMemberName[97] Engaged in Agricultural Work

	Freq.	%
0 No	835	14.55
97 Yes	4,905	85.45
Total	5,740	100.00

gb002_w4_s98: HHMemberName[98] Engaged in Agricultural Work

	Freq.	%
0 No	2,008	34.98
98 Yes	3,732	65.02
Total	5,740	100.00

gb003: Engage in Cropping or Forestry

	Freq.	%
1 Yes	5,309	92.49
2 No	431	7.51
Total	5,740	100.00

gb004_1: Recent Harvest Year

Mean	SD	Min	Max	Obs
2,017.59	2.87	1,900.00	2,018.00	5,296

gb004_2: Recent Harvest Month

Mean	SD	Min	Max	Obs
7.06	2.01	0.00	12.00	5,302

gb005_1: Total Value of All Crops and Forestry

Mean	SD	Min	Max	Obs
9,101.68	36,701.33	0.00	2,000,000.00	4,995

gb005_2: Home Consumed Yuan

Mean	SD	Min	Max	Obs
2,782.34	11,255.77	0.00	500,000.00	2,593

gb005_3: Home Consumed Percent

Mean	SD	Min	Max	Obs
71.40	43.08	0.00	1,000.00	2,688

gb005_min: Min Bracket of gb005_1

Mean	SD	Min	Max	Obs
3,852.35	3,093.97	1,000.00	10,000.00	149

gb005_max: Max Bracket of gb005_1

Mean	SD	Min	Max	Obs
2,389.31	2,108.45	1,000.00	10,000.00	262

gb005_w2_min: Min Bracket of gb005_2

Mean	SD	Min	Max	Obs
3,123.08	2,786.73	1,000.00	10,000.00	65

gb005_w2_max: Max Bracket of gb005_2

Mean	SD	Min	Max	Obs
1,437.16	1,324.23	1,000.00	10,000.00	366

gb006: Total Cost of Producing Crops and Forestry

Mean	SD	Min	Max	Obs
5,106.78	30,528.74	0.00	1,200,000.00	5,126

gb006_min: Min Bracket of gb006

Mean	SD	Min	Max	Obs
1,794.17	1,603.53	300.00	5,000.00	120

gb006_max: Max Bracket of gb006

Mean	SD	Min	Max	Obs
1,184.69	1,488.61	300.00	5,000.00	196

gb007: Grow Livestock or Aquatic Life

	Freq.	%
1 Yes	2,860	49.83
2 No	2,880	50.17
Total	5,740	100.00

gb008: Current Value of All Livestock or Aquatic Life

Mean	SD	Min	Max	Obs
12,523.81	90,123.89	0.00	3,000,000.00	2,694

gb008_min: Min Bracket of gb008

Mean	SD	Min	Max	Obs
2,730.77	3,009.53	500.00	9,000.00	91

gb008_max: Max Bracket of gb008

Mean	SD	Min	Max	Obs
1,386.74	1,776.28	500.00	9,000.00	181

gb009: Last Year Value of All Livestock or Aquatic Life

Mean	SD	Min	Max	Obs
11,274.35	57,777.97	0.00	2,000,000.00	2,720

gb009_min: Min Bracket of gb009

Mean	SD	Min	Max	Obs
2,500.00	2,412.23	500.00	8,000.00	70

gb009_max: Max Bracket of gb009

Mean	SD	Min	Max	Obs
866.44	1,056.47	500.00	8,000.00	292

gb010: Cost of New Livestock and Aquatic Life in the Past Year

Mean	SD	Min	Max	Obs
4,810.98	72,543.23	0.00	3,150,000.00	2,785

gb011_1: Value of All Livestock and Aquatic Life Sold or Consumed

Mean	SD	Min	Max	Obs
9,202.38	61,974.01	0.00	2,400,000.00	2,725

gb011_2: Self-Consumed Yuan

Mean	SD	Min	Max	Obs
2,671.47	24,844.54	0.00	900,000.00	1,481

gb011_3: Self-Consumed Percent

Mean	SD	Min	Max	Obs
87.40	323.32	0.00	12,000.00	1,378

gb011_min: Min Bracket of gb011_1

Mean	SD	Min	Max	Obs
1,395.33	1,483.71	200.00	5,000.00	107

gb011_max: Max Bracket of gb011_1

Mean	SD	Min	Max	Obs
418.75	645.81	200.00	5,000.00	512

gb011_w2_min: Min Bracket of gb011_2

Mean	SD	Min	Max	Obs
1,115.63	1,244.11	200.00	5,000.00	32

gb011_w2_max: Max Bracket of gb011_2

Mean	SD	Min	Max	Obs
375.69	574.62	200.00	5,000.00	181

gb012_1: Value of All Livestock Products Produced

Mean	SD	Min	Max	Obs
3,226.82	76,987.92	0.00	3,650,000.00	2,659

gb012_2: Self-Consumed Yuan

Mean	SD	Min	Max	Obs
392.32	2,181.95	0.00	50,000.00	1,484

gb012_3: Self-Consumed Percent

Mean	SD	Min	Max	Obs
82.21	36.61	0.00	100.00	1,370

gb012_min: Min Bracket of gb012_1

Mean	SD	Min	Max	Obs
298.14	214.03	100.00	1,000.00	161

gb012_max: Max Bracket of gb012_1

Mean	SD	Min	Max	Obs
136.15	121.05	100.00	1,000.00	910

gb012_w2_min: Min Bracket of gb012_2

Mean	SD	Min	Max	Obs
266.67	130.77	100.00	500.00	24

gb012_w2_max: Max Bracket of gb012_2

Mean	SD	Min	Max	Obs
160.87	156.16	100.00	1,000.00	92

gb013: Cost of Producing Livestock and Aquatic Life

Mean	SD	Min	Max	Obs
7,828.03	71,245.45	0.00	3,000,000.00	2,686

gc001: Engage in Self-employed Activities

	Freq.	%
1 Yes	1,061	9.20
2 No	10,467	90.80
Total	11,528	100.00

gc002: Number of Self-employed Activities

Mean	SD	Min	Max	Obs
1.09	0.32	1.00	4.00	1,061

gc003_1__s1: HHMemberName[1] Engage in Self-employed Activity[1]

	Freq.	%
0 No	728	68.61
1 Yes	333	31.39
Total	1,061	100.00

gc003_2__s1: HHMemberName[1] Engage in Self-employed Activity[2]

	Freq.	%
0 No	60	72.29
1 Yes	23	27.71
Total	83	100.00

gc003_3__s1: HHMemberName[1] Engage in Self-employed Activity[3]

	Freq.	%
0 No	4	57.14
1 Yes	3	42.86
Total	7	100.00

gc003_4__s1: HHMemberName[1] Engage in Self-employed Activity[4]

	Freq.	%
1 Yes	2	100.00
Total	2	100.00

gc003_1__s2: HHMemberName[2] Engage in Self-employed Activity[1]

	Freq.	%
0 No	910	85.77
2 Yes	151	14.23
Total	1,061	100.00

gc003_2__s2: HHMemberName[2] Engage in Self-employed Activity[2]

	Freq.	%
0 No	63	75.90
2 Yes	20	24.10
Total	83	100.00

gc003_3__s2: HHMemberName[2] Engage in Self-employed Activity[3]

	Freq.	%
0 No	6	85.71
2 Yes	1	14.29
Total	7	100.00

gc003_4__s2: HHMemberName[2] Engage in Self-employed Activity[4]

	Freq.	%
0 No	1	50.00
2 Yes	1	50.00
Total	2	100.00

gc003_1__s3: HHMemberName[3] Engage in Self-employed Activity[1]

	Freq.	%
0 No	1,039	97.93
3 Yes	22	2.07
Total	1,061	100.00

gc003_2__s3: HHMemberName[3] Engage in Self-employed Activity[2]

	Freq.	%
0 No	81	97.59
3 Yes	2	2.41
Total	83	100.00

gc003_3__s3: HHMemberName[3] Engage in Self-employed Activity[3]

	Freq.	%
0 No	7	100.00
Total	7	100.00

gc003_4__s3: HHMemberName[3] Engage in Self-employed Activity[4]

	Freq.	%
0 No	2	100.00
Total	2	100.00

gc003_1__s4: HHMemberName[4] Engage in Self-employed Activity[1]

	Freq.	%
0 No	1,052	99.15
4 Yes	9	0.85
Total	1,061	100.00

gc003_2__s4: HHMemberName[4] Engage in Self-employed Activity[2]

	Freq.	%
0 No	80	96.39
4 Yes	3	3.61
Total	83	100.00

gc003_3__s4: HHMemberName[4] Engage in Self-employed Activity[3]

	Freq.	%
0 No	6	85.71
4 Yes	1	14.29
Total	7	100.00

gc003_4__s4: HHMemberName[4] Engage in Self-employed Activity[4]

	Freq.	%
0 No	2	100.00
Total	2	100.00

gc003_1__s5: HHMemberName[5] Engage in Self-employed Activity[1]

	Freq.	%
0 No	1,059	99.81
5 Yes	2	0.19
Total	1,061	100.00

gc003_2__s5: HHMemberName[5] Engage in Self-employed Activity[2]

	Freq.	%
0 No	83	100.00
Total	83	100.00

gc003_3__s5: HHMemberName[5] Engage in Self-employed Activity[3]

	Freq.	%
0 No	7	100.00
Total	7	100.00

gc003_4__s5: HHMemberName[5] Engage in Self-employed Activity[4]

	Freq.	%
0 No	2	100.00
Total	2	100.00

gc003_1__s6: HHMemberName[6] Engage in Self-employed Activity[1]

	Freq.	%
0 No	1,059	99.81

	Freq.	%
6 Yes	2	0.19
Total	1,061	100.00

gc003_2__s6: HHMemberName[6] Engage in Self-employed Activity[2]

	Freq.	%
0 No	83	100.00
Total	83	100.00

gc003_3__s6: HHMemberName[6] Engage in Self-employed Activity[3]

	Freq.	%
0 No	7	100.00
Total	7	100.00

gc003_4__s6: HHMemberName[6] Engage in Self-employed Activity[4]

	Freq.	%
0 No	2	100.00
Total	2	100.00

gc003_1__s7: HHMemberName[7] Engage in Self-employed Activity[1]

	Freq.	%
0 No	1,061	100.00
Total	1,061	100.00

gc003_2__s7: HHMemberName[7] Engage in Self-employed Activity[2]

	Freq.	%
0 No	83	100.00
Total	83	100.00

gc003_3__s7: HHMemberName[7] Engage in Self-employed Activity[3]

	Freq.	%
0 No	7	100.00
Total	7	100.00

gc003_4__s7: HHMemberName[7] Engage in Self-employed Activity[4]

	Freq.	%
0 No	2	100.00
Total	2	100.00

gc003_1__s8: HHMemberName[8] Engage in Self-employed Activity[1]

	Freq.	%
0 No	1,061	100.00
Total	1,061	100.00

gc003_2__s8: HHMemberName[8] Engage in Self-employed Activity[2]

	Freq.	%
0 No	83	100.00
Total	83	100.00

gc003_3__s8: HHMemberName[8] Engage in Self-employed Activity[3]

	Freq.	%
0 No	7	100.00
Total	7	100.00

gc003_4__s8: HHMemberName[8] Engage in Self-employed Activity[4]

	Freq.	%
0 No	2	100.00
Total	2	100.00

gc003_1__s9: HHMemberName[9] Engage in Self-employed Activity[1]

	Freq.	%
0 No	1,060	99.91
9 Yes	1	0.09
Total	1,061	100.00

gc003_2__s9: HHMemberName[9] Engage in Self-employed Activity[2]

	Freq.	%
0 No	83	100.00
Total	83	100.00

gc003_3__s9: HHMemberName[9] Engage in Self-employed Activity[3]

	Freq.	%
0 No	7	100.00
Total	7	100.00

gc003_4__s9: HHMemberName[9] Engage in Self-employed Activity[4]

	Freq.	%
0 No	2	100.00
Total	2	100.00

gc003_1__s97: HHMemberName[97] Engage in Self-employed Activity[1]

	Freq.	%
0 No	400	37.70
97 Yes	661	62.30
Total	1,061	100.00

gc003_2__s97: HHMemberName[97] Engage in Self-employed Activity[2]

	Freq.	%
0 No	45	54.22
97 Yes	38	45.78
Total	83	100.00

gc003_3__s97: HHMemberName[97] Engage in Self-employed Activity[3]

	Freq.	%
0 No	3	42.86
97 Yes	4	57.14
Total	7	100.00

gc003_4__s97: HHMemberName[97] Engage in Self-employed Activity[4]

	Freq.	%
0 No	1	50.00
97 Yes	1	50.00
Total	2	100.00

gc003_1__s98: HHMemberName[98] Engage in Self-employed Activity[1]

	Freq.	%
0 No	560	52.78
98 Yes	501	47.22
Total	1,061	100.00

gc003_2__s98: HHMemberName[98] Engage in Self-employed Activity[2]

	Freq.	%
0 No	50	60.24
98 Yes	33	39.76
Total	83	100.00

gc003_3__s98: HHMemberName[98] Engage in Self-employed Activity[3]

	Freq.	%
0 No	5	71.43
98 Yes	2	28.57
Total	7	100.00

gc003_4__s98: HHMemberName[98] Engage in Self-employed Activity[4]

	Freq.	%
0 No	1	50.00
98 Yes	1	50.00
Total	2	100.00

gc004_1_: Activity[1] is Which Type

	Freq.	%
1 Services	310	29.22
2 Transportation	30	2.83
3 Construction	41	3.86
5 Processing Production	102	9.61
6 Business	516	48.63
7 Others	62	5.84
Total	1,061	100.00

gc004_2_: Activity[2] is Which Type

	Freq.	%
1 Services	23	27.71
2 Transportation	3	3.61
3 Construction	4	4.82
4 Mining	1	1.20
5 Processing Production	7	8.43
6 Business	36	43.37
7 Others	9	10.84
Total	83	100.00

gc004_3_: Activity[3] is Which Type

	Freq.	%
2 Transportation	1	14.29
3 Construction	1	14.29
5 Processing Production	1	14.29
6 Business	4	57.14
Total	7	100.00

gc004_4_: Activity[4] is Which Type

	Freq.	%
3 Construction	1	50.00
6 Business	1	50.00
Total	2	100.00

gc005_1_: Net Income from Activity[1]

Mean	SD	Min	Max	Obs
50,340.86	186,874.74	-100,000.00	5,000,000.00	923

gc005_2_: Net Income from Activity[2]

Mean	SD	Min	Max	Obs
60,241.62	119,832.67	0.00	800,000.00	73

gc005_3_: Net Income from Activity[3]

Mean	SD	Min	Max	Obs
21,733.33	39,156.44	0.00	100,000.00	6

gc005_4_: Net Income from Activity[4]

Mean	SD	Min	Max	Obs
1,000.00	1,414.21	0.00	2,000.00	2

gc005_1__min: Min Bracket of gc005_1_

Mean	SD	Min	Max	Obs
35,679.01	41,138.13	5,000.00	200,000.00	81

gc005_1__max: Max Bracket of gc005_1_

Mean	SD	Min	Max	Obs
31,268.66	35,792.25	5,000.00	100,000.00	134

gc005_2__min: Min Bracket of gc005_2_

Mean	SD	Min	Max	Obs
45,000.00	45,825.76	5,000.00	100,000.00	6

gc005_2__max: Max Bracket of gc005_2_

Mean	SD	Min	Max	Obs
44,545.45	63,970.87	5,000.00	200,000.00	11

gc005_3__min: Min Bracket of gc005_3_

Mean	SD	Min	Max	Obs
50,000.00	.	50,000.00	50,000.00	1

gc005_3__max: Max Bracket of gc005_3_

Mean	SD	Min	Max	Obs
27,500.00	31,819.81	5,000.00	50,000.00	2

gc005_4__min: Min Bracket of gc005_4_

No Observations

gc005_4__max: Max Bracket of gc005_4_

No Observations

gd001_w4_1: Dibao Household

	Freq.	%
1 Yes	959	8.32
2 No	10,555	91.56
999 Refused to Answer	14	0.12
Total	11,528	100.00

gd001_w4_2: Year to Be Dibao Household

Mean	SD	Min	Max	Obs
2,011.96	9.67	1,900.00	2,018.00	943

gd001_w4_3: Amount of Dibao Household Assistant

Mean	SD	Min	Max	Obs
2,795.52	3,248.35	0.00	27,000.00	928

gd003_w4_1: Jiandanglika Poor Household

	Freq.	%
1 Yes	874	7.58
2 No	10,419	90.39
997 Don't Know	227	1.97
999 Refused to Answer	7	0.06
Total	11,527	100.00

gd003_w4_2: Year to Be Jiandanglika Poor Household

Mean	SD	Min	Max	Obs
2,014.71	5.82	1,938.00	2,018.00	846

gd002_w4_s1: Receive Reforestation Subsidies

	Freq.	%
0 No	10,536	91.40
1 Yes	991	8.60
Total	11,527	100.00

gd002_w4_s2: Receive Agricultural Subsidies

	Freq.	%
0 No	7,449	64.62
2 Yes	4,078	35.38
Total	11,527	100.00

gd002_w4_s3: Receive Wubaohu Subsidies

	Freq.	%
0 No	11,352	98.48
3 Yes	175	1.52
Total	11,527	100.00

gd002_w4_s4: Receive Poor Household Subsidies

	Freq.	%
0 No	10,893	94.50
4 Yes	634	5.50
Total	11,527	100.00

gd002_w4_s5: Receive Work Injury Subsidies

	Freq.	%
0 No	11,499	99.76
5 Yes	28	0.24
Total	11,527	100.00

gd002_w4_s6: Receive Emergency or Disaster Relief

	Freq.	%
0 No	11,466	99.47
6 Yes	61	0.53
Total	11,527	100.00

gd002_w4_s7: Receive Social Subsidies

	Freq.	%
0 No	11,475	99.55
7 Yes	52	0.45
Total	11,527	100.00

gd002_w4_s8: Receive Compensation for Land Seizure

	Freq.	%
0 No	11,036	95.74
8 Yes	491	4.26
Total	11,527	100.00

gd002_w4_s9: Receive Compensation to Pull Down House or Apartment

	Freq.	%
0 No	11,407	98.96
9 Yes	120	1.04
Total	11,527	100.00

gd002_w4_s10: Receive Other Subsidies

	Freq.	%
0 No	11,020	95.60
10 Yes	507	4.40
Total	11,527	100.00

gd002_w4_s11: None of the Above

	Freq.	%
0 No	5,550	48.15
11 Yes	5,977	51.85
Total	11,527	100.00

gd002_w4_1: Amount of Reforestation Subsidies

Mean	SD	Min	Max	Obs
1,009.51	4,311.19	0.00	85,000.00	909

gd002_w4_2: Amount of Agricultural Subsidies

Mean	SD	Min	Max	Obs
916.48	7,735.51	0.00	400,000.00	3,862

gd002_w4_3: Amount of Wubaohu Subsidies

Mean	SD	Min	Max	Obs
3,246.24	3,609.64	0.00	36,000.00	167

gd002_w4_4: Amount of Poor Household Subsidies

Mean	SD	Min	Max	Obs
3,564.51	12,780.44	0.00	280,000.00	608

gd002_w4_5: Amount of Work Injury Subsidies

Mean	SD	Min	Max	Obs
2,748.89	2,799.94	0.00	12,000.00	27

gd002_w4_6: Amount of Emergency or Disaster Relief

Mean	SD	Min	Max	Obs
8,898.54	30,568.59	0.00	160,000.00	57

gd002_w4_7: Amount of Social Subsidies

Mean	SD	Min	Max	Obs
453.90	530.49	0.00	2,400.00	49

gd002_w4_8: Amount of Compensation for Land Seizure

Mean	SD	Min	Max	Obs
21,610.71	99,279.15	0.00	1,880,000.00	478

gd002_w4_9: Amount of Compensation to Pull Down House or Apartment

Mean	SD	Min	Max	Obs
191,210.67	567,437.02	0.00	5,100,000.00	117

gd002_w4_10: Amount of Other Subsidies

Mean	SD	Min	Max	Obs
81,527.39	1,731,940.35	0.00	39000500.00	507

gd004_w4: Productive Insurance Payment

Mean	SD	Min	Max	Obs
11.05	192.00	0.00	13,000.00	11,512

ge000_w4: Monthly Household Expenditure

Mean	SD	Min	Max	Obs
1,940.31	2,928.24	0.00	150,000.00	11,306

ge001: Primary Person Who Purchases Food

	Freq.	%
1 Yes	9,697	84.12
2 No	1,830	15.88
Total	11,527	100.00

ge002: Who is Primary Person Purchasing Food

	Freq.	%
1 HHMemberName[1]	228	12.46
2 HHMemberName[2]	149	8.14

3 HHMemberName[3]	13	0.71
4 HHMemberName[4]	3	0.16
5 HHMemberName[5]	3	0.16
6 HHMemberName[6]	1	0.05
26 HHMemberName[26]	103	5.63
27 HHMemberName[27]	61	3.33
28 HHMemberName[28]	41	2.24
29 HHMemberName[29]	33	1.80
30 HHMemberName[30]	11	0.60
31 HHMemberName[31]	7	0.38
32 HHMemberName[32]	4	0.22
33 HHMemberName[33]	4	0.22
36 HHMemberName[36]	1	0.05
51 HHMemberName[51]	8	0.44
52 HHMemberName[52]	1	0.05
53 HHMemberName[53]	207	11.31
97 HHMemberName[97]	214	11.69
98 HHMemberName[98]	738	40.33
Total	1,830	100.00

ge004: Number of People Ate Meals in the Past Week

Mean	SD	Min	Max	Obs
3.10	1.86	0.00	20.00	11,522

ge005: Number of Meals Provided to Guests

Mean	SD	Min	Max	Obs
1.64	8.11	0.00	500.00	11,506

ge006_w4: Food Expenditure, Excluding Somethings

Mean	SD	Min	Max	Obs
242.13	477.44	0.00	29,000.00	11,239

ge006_w2: Produce Agricultural Products Yourself

	Freq.	%
1 Yes	5,945	51.58
2 No	5,580	48.42
Total	11,525	100.00

ge006_w2_1: Market Value of Agricultural Products

Mean	SD	Min	Max	Obs
117.96	352.51	0.00	8,000.00	5,549

ge007_w4: Eating Out

Mean	SD	Min	Max	Obs

43.24	233.00	0.00	10,000.00	11,462

ge008: Alcohol, Cigarettes, Cigars and Tobacco

Mean	SD	Min	Max	Obs
56.94	210.22	0.00	10,000.00	11,390

ge009_1: Communication Fees

Mean	SD	Min	Max	Obs
180.01	3,914.73	0.00	400,150.00	10,993

ge009_2: Utilities

Mean	SD	Min	Max	Obs
149.73	298.72	0.00	15,025.00	11,146

ge009_3: Fuels

Mean	SD	Min	Max	Obs
63.70	214.89	0.00	16,067.00	11,219

ge009_4: Fees for Matron, Housekeepers and Servants

Mean	SD	Min	Max	Obs
17.72	299.63	0.00	15,000.00	11,515

ge009_5: Local Transportation

Mean	SD	Min	Max	Obs
79.45	313.23	0.00	13,000.00	11,327

ge009_6: Household Items and Personal Toiletries

Mean	SD	Min	Max	Obs
79.63	255.53	0.00	13,000.00	10,983

ge009_7: Entertainment

Mean	SD	Min	Max	Obs
10.89	70.17	0.00	2,100.00	11,369

ge009_w4_4_s1: Elderly in the Household

	Freq.	%

	Freq.	%
0 No	47	51.09
1 Yes	45	48.91
Total	92	100.00

ge009_w4_4_s2: Child in the Household

	Freq.	%
0 No	85	92.39
2 Yes	7	7.61
Total	92	100.00

ge009_w4_4_s3: Others

	Freq.	%
0 No	51	55.43
3 Yes	41	44.57
Total	92	100.00

ge010_1: Clothing and Bedding

Mean	SD	Min	Max	Obs
1,708.17	19,377.48	0.00	2,000,000.00	10,943

ge010_2: Traveling Expenses

Mean	SD	Min	Max	Obs
766.90	7,656.97	0.00	500,000.00	11,418

ge010_3: Heating

Mean	SD	Min	Max	Obs
286.82	1,077.70	0.00	60,000.00	11,448

ge010_4: Furniture, Durable Goods and Electronics

Mean	SD	Min	Max	Obs
1,008.15	6,289.27	0.00	250,000.00	11,438

ge010_5: Education and Training

Mean	SD	Min	Max	Obs
1,886.04	7,706.75	0.00	348,000.00	11,325

ge010_6: Medical Expenditure

Mean	SD	Min	Max	Obs

6,538.88	23,546.19	0.00	1,200,000.00	11,200

ge010_7: Fitness Expenditures

Mean	SD	Min	Max	Obs
276.45	2,322.73	0.00	100,000.00	11,440

ge010_8: Beauty

Mean	SD	Min	Max	Obs
175.87	1,078.49	0.00	50,000.00	11,349

ge010_9: Automobiles

Mean	SD	Min	Max	Obs
3,698.03	31,157.79	0.00	1,250,000.00	11,477

ge010_10: Purchase, Maintenance and Repair

Mean	SD	Min	Max	Obs
601.12	2,975.15	0.00	170,000.00	11,399

ge010_11: Property Management Fees

Mean	SD	Min	Max	Obs
153.68	744.28	0.00	40,000.00	11,413

ge010_12: Taxes and Fees

Mean	SD	Min	Max	Obs
198.65	5,842.48	0.00	500,000.00	11,341

ge010_13: Donations

Mean	SD	Min	Max	Obs
101.04	2,514.31	0.00	200,000.00	11,384

ge010_14: Rent

Mean	SD	Min	Max	Obs
576.72	3,878.91	0.00	120,000.00	11,382

ge010_15: Banquet and Wedding Expenditure

Mean	SD	Min	Max	Obs

1,362.99	7,785.06	0.00	200,000.00	11,463

ge011: Receive Assistance

	Freq.	%
1 Never	8,935	77.54
2 A Few Times	2,198	19.07
3 Most or All of the Time	390	3.38
Total	11,523	100.00

ha054_s1: Cultivated Land

	Freq.	%
0 No	4,716	40.93
1 Yes	6,807	59.07
Total	11,523	100.00

ha054_s2: Forest Land

	Freq.	%
0 No	10,132	87.93
2 Yes	1,391	12.07
Total	11,523	100.00

ha054_s3: Pasture

	Freq.	%
0 No	11,418	99.09
3 Yes	105	0.91
Total	11,523	100.00

ha054_s4: Pond

	Freq.	%
0 No	11,368	98.65
4 Yes	155	1.35
Total	11,523	100.00

ha054_s5: None of the Above

	Freq.	%
0 No	6,995	60.70
5 Yes	4,528	39.30
Total	11,523	100.00

ha070_w4_1_: Quequan (Issuance of Land Document) for Land[1]

	Freq.	%
1 Yes	4,151	60.98

	Freq.	%
2 No	2,080	30.56
997 Don't Know	576	8.46
Total	6,807	100.00

ha070_w4_2_: Quequan (Issuance of Land Document) for Land[2]

	Freq.	%
1 Yes	153	51.00
2 No	132	44.00
997 Don't Know	15	5.00
Total	300	100.00

ha070_w4_3_: Quequan (Issuance of Land Document) for Land[3]

	Freq.	%
1 Yes	24	53.33
2 No	20	44.44
997 Don't Know	1	2.22
Total	45	100.00

ha070_w4_4_: Quequan (Issuance of Land Document) for Land[4]

	Freq.	%
1 Yes	9	20.93
2 No	26	60.47
997 Don't Know	8	18.60
Total	43	100.00

ha071_w4_1_: Year of Issuance of Land Document for Land[1]

Mean	SD	Min	Max	Obs
2,010.51	13.14	1,900.00	2,018.00	3,936

ha071_w4_2_: Year of Issuance of Land Document for Land[2]

Mean	SD	Min	Max	Obs
2,007.18	12.80	1,958.00	2,018.00	146

ha071_w4_3_: Year of Issuance of Land Document for Land[3]

Mean	SD	Min	Max	Obs
2,003.30	11.40	1,985.00	2,018.00	23

ha071_w4_4_: Year of Issuance of Land Document for Land[4]

Mean	SD	Min	Max	Obs
2,010.67	9.85	1,988.00	2,018.00	9

ha055_1_: Land Size for Land[1]

Mean	SD	Min	Max	Obs
5.51	15.85	0.00	500.00	6,721

ha055_2_: Land Size for Land[2]

Mean	SD	Min	Max	Obs
7.25	22.13	0.00	300.00	290

ha055_3_: Land Size for Land[3]

Mean	SD	Min	Max	Obs
602.36	381.62	0.00	1,500.00	45

ha055_4_: Land Size for Land[4]

Mean	SD	Min	Max	Obs
4.48	8.56	0.00	50.00	42

ha056_1_: Irrigable Land Size for Land[1]

Mean	SD	Min	Max	Obs
2.91	11.14	0.00	500.00	6,691

ha056_2_: Irrigable Land Size for Land[2]

Mean	SD	Min	Max	Obs
0.83	2.57	0.00	20.00	290

ha056_3_: Irrigable Land Size for Land[3]

Mean	SD	Min	Max	Obs
69.13	209.81	0.00	900.00	45

ha056_4_: Irrigable Land Size for Land[4]

Mean	SD	Min	Max	Obs
3.87	8.83	0.00	50.00	40

ha057_1_: Rent Per Mu for Land[1]

Mean	SD	Min	Max	Obs
541.92	1,465.72	0.00	51,000.00	5,585

ha057_2_: Rent Per Mu for Land[2]

Mean	SD	Min	Max	Obs
340.93	713.32	0.00	5,000.00	212

ha057_3_: Rent Per Mu for Land[3]

Mean	SD	Min	Max	Obs
588.33	3,328.32	0.00	20,000.00	36

ha057_4_: Rent Per Mu for Land[4]

Mean	SD	Min	Max	Obs
2,405.17	7,777.96	0.00	43,000.00	30

ha057_1__min: Min Bracket of ha057_1_

Mean	SD	Min	Max	Obs
333.88	292.54	50.00	1,000.00	484

ha057_1__max: Max Bracket of ha057_1_

Mean	SD	Min	Max	Obs
268.84	283.92	50.00	1,000.00	727

ha057_2__min: Min Bracket of ha057_2_

Mean	SD	Min	Max	Obs
292.19	281.16	50.00	1,000.00	32

ha057_2__max: Max Bracket of ha057_2_

Mean	SD	Min	Max	Obs
235.19	276.20	50.00	1,000.00	54

ha057_3__min: Min Bracket of ha057_3_

Mean	SD	Min	Max	Obs
150.00	132.29	50.00	300.00	3

ha057_3__max: Max Bracket of ha057_3_

Mean	SD	Min	Max	Obs
216.67	144.34	50.00	300.00	3

ha057_4__min: Min Bracket of ha057_4_

Mean	SD	Min	Max	Obs
458.33	431.76	50.00	1,000.00	6

ha057_4__max: Max Bracket of ha057_4_

Mean	SD	Min	Max	Obs
314.29	361.38	50.00	1,000.00	7

ha057_w4_1_1_: Zero Rent's Reasons for Land[1]

	Freq.	%
1 Rent is Low	46	6.43
2 Nobody Want to Rent	610	85.31
3 Others	59	8.25
Total	715	100.00

ha057_w4_1_2_: Zero Rent's Reasons for Land[2]

	Freq.	%
1 Rent is Low	4	5.06
2 Nobody Want to Rent	72	91.14
3 Others	3	3.80
Total	79	100.00

ha057_w4_1_3_: Zero Rent's Reasons for Land[3]

	Freq.	%
1 Rent is Low	1	50.00
2 Nobody Want to Rent	1	50.00
Total	2	100.00

ha057_w4_1_4_: Zero Rent's Reasons for Land[4]

	Freq.	%
3 Others	2	100.00
Total	2	100.00

ha058_1_: Rent Out for Land[1]

	Freq.	%
1 Yes	1,749	25.70
2 No	5,057	74.30
Total	6,806	100.00

ha058_2_: Rent Out for Land[2]

	Freq.	%

	Freq.	%
1 Yes	19	6.33
2 No	281	93.67
Total	300	100.00

ha058_3_: Rent Out for Land[3]

	Freq.	%
1 Yes	4	8.89
2 No	41	91.11
Total	45	100.00

ha058_4_: Rent Out for Land[4]

	Freq.	%
1 Yes	6	13.95
2 No	37	86.05
Total	43	100.00

ha059_1_: Land Size Rent Out for Land[1]

Mean	SD	Min	Max	Obs
5.72	21.93	0.00	500.00	1,736

ha059_2_: Land Size Rent Out for Land[2]

Mean	SD	Min	Max	Obs
1.85	2.68	0.00	10.00	19

ha059_3_: Land Size Rent Out for Land[3]

Mean	SD	Min	Max	Obs
513.75	226.62	315.00	800.00	4

ha059_4_: Land Size Rent Out for Land[4]

Mean	SD	Min	Max	Obs
6.77	9.13	0.10	24.00	6

ha060_1_: Rent Earned for Land[1]

Mean	SD	Min	Max	Obs
1,921.09	5,109.66	0.00	100,000.00	1,742

ha060_2_: Rent Earned for Land[2]

Mean	SD	Min	Max	Obs
1,060.00	1,902.61	0.00	8,000.00	18

ha060_3_: Rent Earned for Land[3]

Mean	SD	Min	Max	Obs
6,250.00	1,500.00	5,000.00	8,000.00	4

ha060_4_: Rent Earned for Land[4]

Mean	SD	Min	Max	Obs
4,540.00	5,805.00	0.00	14,400.00	5

ha061_1_: Rent from Others for Land[1]

	Freq.	%
1 Yes	661	9.71
2 No	6,145	90.29
Total	6,806	100.00

ha061_2_: Rent from Others for Land[2]

	Freq.	%
1 Yes	18	6.00
2 No	282	94.00
Total	300	100.00

ha061_3_: Rent from Others for Land[3]

	Freq.	%
1 Yes	13	28.89
2 No	32	71.11
Total	45	100.00

ha061_4_: Rent from Others for Land[4]

	Freq.	%
1 Yes	13	30.23
2 No	30	69.77
Total	43	100.00

ha062_1_: Land Size You Rent for Land[1]

Mean	SD	Min	Max	Obs
19.56	77.21	0.00	1,000.00	656

ha062_2_: Land Size You Rent for Land[2]

Mean	SD	Min	Max	Obs
26.42	50.35	0.50	200.00	18

ha062_3_: Land Size You Rent for Land[3]

Mean	SD	Min	Max	Obs
2,085.77	5,692.91	55.00	21,000.00	13

ha062_4_: Land Size You Rent for Land[4]

Mean	SD	Min	Max	Obs
99.19	271.79	1.00	1,000.00	13

ha063_1_: Rent You Pay for Land[1]

Mean	SD	Min	Max	Obs
5,423.45	19,465.00	0.00	250,000.00	657

ha063_2_: Rent You Pay for Land[2]

Mean	SD	Min	Max	Obs
2,608.53	4,600.27	0.00	20,000.00	18

ha063_3_: Rent You Pay for Land[3]

Mean	SD	Min	Max	Obs
18,462.88	25,046.40	17.50	90,000.00	13

ha063_4_: Rent You Pay for Land[4]

Mean	SD	Min	Max	Obs
8,904.17	13,313.98	0.00	43,000.00	12

ha064: Rental Income from Other Household Assets

	Freq.	%
1 Yes	89	0.86
2 No	10,176	97.99
997 Don't Know	104	1.00
999 Refused to Answer	16	0.15
Total	10,385	100.00

ha064_1: Rental Income You Earn

Mean	SD	Min	Max	Obs
17,892.68	34,767.81	0.00	300,000.00	92

ha065_s1: Automobile

	Freq.	%
0 No	10,179	88.34
1 Yes	1,343	11.66
Total	11,522	100.00

ha065_s2: Electric Bicycle

	Freq.	%
0 No	7,013	60.87
2 Yes	4,509	39.13
Total	11,522	100.00

ha065_s3: Motorcycle

	Freq.	%
0 No	8,710	75.59
3 Yes	2,812	24.41
Total	11,522	100.00

ha065_s4: Refrigerator

	Freq.	%
0 No	2,716	23.57
4 Yes	8,806	76.43
Total	11,522	100.00

ha065_s5: Washing Machine

	Freq.	%
0 No	3,351	29.08
5 Yes	8,171	70.92
Total	11,522	100.00

ha065_s6: TV

	Freq.	%
0 No	1,736	15.07
6 Yes	9,786	84.93
Total	11,522	100.00

ha065_s7: Computer

	Freq.	%
0 No	9,393	81.52
7 Yes	2,129	18.48
Total	11,522	100.00

ha065_s8: Stereo System

	Freq.	%
0 No	10,947	95.01
8 Yes	575	4.99
Total	11,522	100.00

ha065_s9: Video Camera

	Freq.	%
0 No	11,418	99.10
9 Yes	104	0.90
Total	11,522	100.00

ha065_s10: Camera

	Freq.	%
0 No	11,196	97.17
10 Yes	326	2.83
Total	11,522	100.00

ha065_s11: Air Conditioner

	Freq.	%
0 No	6,994	60.70
11 Yes	4,528	39.30
Total	11,522	100.00

ha065_s12: Mobile Phone

	Freq.	%
0 No	1,315	11.41
12 Yes	10,207	88.59
Total	11,522	100.00

ha065_s13: Furniture

	Freq.	%
0 No	10,982	95.31
13 Yes	540	4.69
Total	11,522	100.00

ha065_s14: Music Instrument

	Freq.	%
0 No	11,425	99.16
14 Yes	97	0.84
Total	11,522	100.00

ha065_s15: Valuable Decorations, Ornaments

	Freq.	%
0 No	11,440	99.29
15 Yes	82	0.71
Total	11,522	100.00

ha065_s16: Treasures and Precious Metal

	Freq.	%
0 No	10,585	91.87
16 Yes	937	8.13
Total	11,522	100.00

ha065_s17: Artistic Work

	Freq.	%
0 No	11,443	99.31
17 Yes	79	0.69
Total	11,522	100.00

ha065_s18: Air Purifier

	Freq.	%
0 No	11,305	98.12
18 Yes	217	1.88
Total	11,522	100.00

ha065_s19: None of the Above

	Freq.	%
0 No	11,046	95.87
19 Yes	476	4.13
Total	11,522	100.00

ha065_1: Automobile Value

Mean	SD	Min	Max	Obs
69,470.51	166,549.94	0.00	5,000,000.00	1,339

ha065_2: Electric Bicycle Value

Mean	SD	Min	Max	Obs
1,592.35	2,288.05	0.00	50,000.00	4,490

ha065_3: Motorcycle Value

Mean	SD	Min	Max	Obs
1,749.52	2,248.23	0.00	37,000.00	2,791

ha065_4: Refrigerator Value

Mean	SD	Min	Max	Obs
1,042.43	1,631.67	0.00	70,000.00	8,644

ha065_5: Washing Machine Value

Mean	SD	Min	Max	Obs
575.70	1,012.72	0.00	60,000.00	8,015

ha065_6: TV Value

Mean	SD	Min	Max	Obs
883.37	1,472.40	0.00	40,000.00	9,551

ha065_7: Computer Value

Mean	SD	Min	Max	Obs
1,539.34	3,077.21	0.00	100,000.00	2,024

ha065_8: Stereo System Value

Mean	SD	Min	Max	Obs
536.99	1,627.50	0.00	20,000.00	545

ha065_9: Video Camera Value

Mean	SD	Min	Max	Obs
1,480.79	2,974.75	0.00	20,000.00	103

ha065_10: Camera Value

Mean	SD	Min	Max	Obs
1,743.33	4,522.86	0.00	50,000.00	308

ha065_11: Air Conditioner Value

Mean	SD	Min	Max	Obs
2,242.72	3,606.67	0.00	110,000.00	4,386

ha065_12: Mobile Phone Value

Mean	SD	Min	Max	Obs
780.34	1,568.28	0.00	40,000.00	9,985

ha065_13: Furniture Value

Mean	SD	Min	Max	Obs
10,649.19	40,506.96	0.00	600,000.00	533

ha065_14: Music Instrument Value

Mean	SD	Min	Max	Obs
4,102.38	6,275.87	0.00	30,000.00	99

ha065_15: Valuable Decorations, Ornaments Value

Mean	SD	Min	Max	Obs
5,382.82	10,234.59	0.00	60,000.00	87

ha065_16: Treasures and Precious Metal Value

Mean	SD	Min	Max	Obs
8,101.15	14,830.91	0.00	250,000.00	910

ha065_17: Artistic Work Value

Mean	SD	Min	Max	Obs
222,036.62	1,269,966.62	0.00	10000000.00	71

ha065_18: Air Purifier Value

Mean	SD	Min	Max	Obs
1,333.17	1,874.73	0.00	10,000.00	208

ha066_w4_s1: Tractor

	Freq.	%
0 No	10,522	91.32
1 Yes	1,000	8.68
Total	11,522	100.00

ha066_w4_s2: Thresher

	Freq.	%
0 No	11,163	96.88
2 Yes	359	3.12
Total	11,522	100.00

ha066_w4_s3: Harvester

	Freq.	%
0 No	11,463	99.49

3 Yes	59	0.51
Total	11,522	100.00

ha066_w4_s4: Water Pump

	Freq.	%
0 No	10,299	89.39
4 Yes	1,223	10.61
Total	11,522	100.00

ha066_w4_s5: Processing Equipment

	Freq.	%
0 No	11,194	97.15
5 Yes	328	2.85
Total	11,522	100.00

ha066_w4_s6: Seeder

	Freq.	%
0 No	11,251	97.65
6 Yes	271	2.35
Total	11,522	100.00

ha066_w4_s7: Agricultural Aircraft

	Freq.	%
0 No	11,517	99.96
7 Yes	5	0.04
Total	11,522	100.00

ha066_w4_s8: None of the Above

	Freq.	%
0 No	2,312	20.07
8 Yes	9,210	79.93
Total	11,522	100.00

ha066_w4_1: Tractor Value

Mean	SD	Min	Max	Obs
4,956.91	22,609.25	0.00	400,000.00	981

ha066_w4_2: Thresher Value

Mean	SD	Min	Max	Obs
1,593.51	11,637.72	0.00	200,000.00	350

ha066_w4_3: Harvester Value

Mean	SD	Min	Max	Obs
15,485.59	37,703.97	0.00	200,000.00	59

ha066_w4_4: Water Pump Value

Mean	SD	Min	Max	Obs
756.04	8,799.36	0.00	300,000.00	1,189

ha066_w4_5: Processing Equipment Value

Mean	SD	Min	Max	Obs
5,861.09	67,663.20	0.00	1,200,000.00	325

ha066_w4_6: Seeder Value

Mean	SD	Min	Max	Obs
1,093.74	3,235.12	0.00	40,000.00	265

ha066_w4_7: Agricultural Aircraft Value

Mean	SD	Min	Max	Obs
486.67	803.28	0.00	2,000.00	6

ha067_w4: Other Fixed Capital Assets

Mean	SD	Min	Max	Obs
15,780.36	321,100.49	0.00	20000000.00	11,494

ha068: Other Durable or Fixed Asset Worth 500 Yuan or More

	Freq.	%
1 Yes	598	5.19
2 No	10,776	93.53
997 Don't Know	132	1.15
999 Refused to Answer	16	0.14
Total	11,522	100.00

ha068_1: Worth

Mean	SD	Min	Max	Obs
14,747.16	71,454.83	0.00	1,000,000.00	598

ha076: Respondent Receive Assistance

	Freq.	%

1 Never	9,339	81.05
2 A Few Time	1,816	15.76
3 Most or All of Time	367	3.19
Total	11,522	100.00

versionID: Version ID

A String Variable	
Obs:	11,544

This page intentionally left blank

MODULE H&I

Housing

householdID: Household ID

A String Variable	
Obs:	11,535

communityID: Community ID

A String Variable	
Obs:	11,535

ha000_w4_0: Number of Houses

Mean	SD	Min	Max	Obs
1.11	1.23	0.00	11.00	90

ha000_w4_1_: Check House[1]

	Freq.	%
1 Know House[1]	3,378	42.00
2 Do Not know House[1]	4,665	58.00
Total	8,043	100.00

ha000_w4_2_: Check House[2]

	Freq.	%
1 Know House[2]	3,237	45.20
2 Do Not know House[2]	3,924	54.80
Total	7,161	100.00

ha000_w4_3_: Check House[3]

	Freq.	%

	Freq.	%
1 Know House[3]	1,849	37.47
2 Do Not know House[3]	3,085	62.53
Total	4,934	100.00

ha000_w4_4_: Check House[4]

	Freq.	%
1 Know House[4]	143	29.24
2 Do Not know House[4]	346	70.76
Total	489	100.00

ha000_w4_5_: Check House[5]

	Freq.	%
1 Know House[5]	23	30.26
2 Do Not know House[5]	53	69.74
Total	76	100.00

ha000_w4_6_: Check House[6]

	Freq.	%
1 Know House[6]	3	27.27
2 Do Not know House[6]	8	72.73
Total	11	100.00

ha000_w4_7_: Check House[7]

	Freq.	%
1 Know House[7]	1	50.00
2 Do Not know House[7]	1	50.00
Total	2	100.00

ha000_w4_1_1_: Check Ownership Structure of House[1]

	Freq.	%
1 Yes	1,109	77.55
2 No	319	22.31
999 Refused to Answer	2	0.14
Total	1,430	100.00

ha000_w4_1_2_: Check Ownership Structure of House[2]

	Freq.	%
1 Yes	1,495	79.06
2 No	382	20.20
999 Refused to Answer	14	0.74
Total	1,891	100.00

ha000_w4_1_3_: Check Ownership Structure of House[3]

	Freq.	%
1 Yes	894	80.61
2 No	209	18.85
999 Refused to Answer	6	0.54
Total	1,109	100.00

ha000_w4_1_4_: Check Ownership Structure of House[4]

	Freq.	%
1 Yes	71	82.56
2 No	15	17.44
Total	86	100.00

ha000_w4_1_5_: Check Ownership Structure of House[5]

	Freq.	%
1 Yes	8	72.73
2 No	3	27.27
Total	11	100.00

ha000_w4_1_6_: Check Ownership Structure of House[6]

	Freq.	%
1 Yes	2	100.00
Total	2	100.00

ha000_w4_1_7_: Check Ownership Structure of House[7]

	Freq.	%
2 No	1	100.00
Total	1	100.00

ha000_w4_2_1__s1: Household Head and Spouse Own House[1]

	Freq.	%
0 Do Not Own	626	27.66
1 Own	1,637	72.34
Total	2,263	100.00

ha000_w4_2_2__s1: Household Head and Spouse Own House[2]

	Freq.	%
0 Do Not Own	557	32.09
1 Own	1,179	67.91
Total	1,736	100.00

ha000_w4_2_3__s1: Household Head and Spouse Own House[3]

	Freq.	%
0 Do Not Own	288	30.22
1 Own	665	69.78
Total	953	100.00

ha000_w4_2_4__s1: Household Head and Spouse Own House[4]

	Freq.	%
0 Do Not Own	24	33.33
1 Own	48	66.67
Total	72	100.00

ha000_w4_2_5__s1: Household Head and Spouse Own House[5]

	Freq.	%
0 Do Not Own	3	20.00
1 Own	12	80.00
Total	15	100.00

ha000_w4_2_6__s1: Household Head and Spouse Own House[6]

	Freq.	%
1 Own	1	100.00
Total	1	100.00

ha000_w4_2_7__s1: Household Head and Spouse Own House[7]

	Freq.	%
1 Own	1	100.00
Total	1	100.00

ha000_w4_2_1__s2: Children and Children's Spouses Own House[1]

	Freq.	%
0 Do Not Own	1,741	76.93
2 Own	522	23.07
Total	2,263	100.00

ha000_w4_2_2__s2: Children and Children's Spouses Own House[2]

	Freq.	%
0 Do Not Own	1,219	70.22
2 Own	517	29.78
Total	1,736	100.00

ha000_w4_2_3__s2: Children and Children's Spouses Own House[3]

	Freq.	%
0 Do Not Own	694	72.82
2 Own	259	27.18
Total	953	100.00

ha000_w4_2_4__s2: Children and Children's Spouses Own House[4]

	Freq.	%
0 Do Not Own	56	77.78
2 Own	16	22.22
Total	72	100.00

ha000_w4_2_5__s2: Children and Children's Spouses Own House[5]

	Freq.	%
0 Do Not Own	12	80.00
2 Own	3	20.00
Total	15	100.00

ha000_w4_2_6__s2: Children and Children's Spouses Own House[6]

	Freq.	%
0 Do Not Own	1	100.00
Total	1	100.00

ha000_w4_2_7__s2: Children and Children's Spouses Own House[7]

	Freq.	%
0 Do Not Own	1	100.00
Total	1	100.00

ha000_w4_2_1__s3: Siblings Own House[1]

	Freq.	%
0 Do Not Own	2,241	99.03
3 Own	22	0.97
Total	2,263	100.00

ha000_w4_2_2__s3: Siblings Own House[2]

	Freq.	%
0 Do Not Own	1,717	98.91
3 Own	19	1.09
Total	1,736	100.00

ha000_w4_2_3__s3: Siblings Own House[3]

	Freq.	%
0 Do Not Own	939	98.53
3 Own	14	1.47
Total	953	100.00

ha000_w4_2_4__s3: Siblings Own House[4]

	Freq.	%
0 Do Not Own	72	100.00
Total	72	100.00

ha000_w4_2_5__s3: Siblings Own House[5]

	Freq.	%
0 Do Not Own	15	100.00
Total	15	100.00

ha000_w4_2_6__s3: Siblings Own House[6]

	Freq.	%
0 Do Not Own	1	100.00
Total	1	100.00

ha000_w4_2_7__s3: Siblings Own House[7]

	Freq.	%
0 Do Not Own	1	100.00
Total	1	100.00

ha000_w4_2_1__s4: Parents and Parents-in-Law Own House[1]

	Freq.	%
0 Do Not Own	2,235	98.76
4 Own	28	1.24
Total	2,263	100.00

ha000_w4_2_2__s4: Parents and Parents-in-Law Own House[2]

	Freq.	%
0 Do Not Own	1,722	99.19
4 Own	14	0.81
Total	1,736	100.00

ha000_w4_2_3__s4: Parents and Parents-in-Law Own House[3]

	Freq.	%
0 Do Not Own	946	99.27

	Freq.	%
4 Own	7	0.73
Total	953	100.00

ha000_w4_2_4__s4: Parents and Parents-in-Law Own House[4]

	Freq.	%
0 Do Not Own	72	100.00
Total	72	100.00

ha000_w4_2_5__s4: Parents and Parents-in-Law Own House[5]

	Freq.	%
0 Do Not Own	15	100.00
Total	15	100.00

ha000_w4_2_6__s4: Parents and Parents-in-Law Own House[6]

	Freq.	%
0 Do Not Own	1	100.00
Total	1	100.00

ha000_w4_2_7__s4: Parents and Parents-in-Law Own House[7]

	Freq.	%
0 Do Not Own	1	100.00
Total	1	100.00

ha000_w4_2_1__s5: Grandchildren Own House[1]

	Freq.	%
0 Do Not Own	2,244	99.16
5 Own	19	0.84
Total	2,263	100.00

ha000_w4_2_2__s5: Grandchildren Own House[2]

	Freq.	%
0 Do Not Own	1,707	98.33
5 Own	29	1.67
Total	1,736	100.00

ha000_w4_2_3__s5: Grandchildren Own House[3]

	Freq.	%
0 Do Not Own	937	98.32
5 Own	16	1.68
Total	953	100.00

ha000_w4_2_4__s5: Grandchildren Own House[4]

	Freq.	%
0 Do Not Own	72	100.00
Total	72	100.00

ha000_w4_2_5__s5: Grandchildren Own House[5]

	Freq.	%
0 Do Not Own	15	100.00
Total	15	100.00

ha000_w4_2_6__s5: Grandchildren Own House[6]

	Freq.	%
0 Do Not Own	1	100.00
Total	1	100.00

ha000_w4_2_7__s5: Grandchildren Own House[7]

	Freq.	%
0 Do Not Own	1	100.00
Total	1	100.00

ha000_w4_2_1__s6: Other Relatives Own House[1]

	Freq.	%
0 Do Not Own	2,256	99.69
6 Own	7	0.31
Total	2,263	100.00

ha000_w4_2_2__s6: Other Relatives Own House[2]

	Freq.	%
0 Do Not Own	1,731	99.71
6 Own	5	0.29
Total	1,736	100.00

ha000_w4_2_3__s6: Other Relatives Own House[3]

	Freq.	%
0 Do Not Own	951	99.79
6 Own	2	0.21
Total	953	100.00

ha000_w4_2_4__s6: Other Relatives Own House[4]

	Freq.	%

	Freq.	%
0 Do Not Own	72	100.00
Total	72	100.00

ha000_w4_2_5__s6: Other Relatives Own House[5]

	Freq.	%
0 Do Not Own	15	100.00
Total	15	100.00

ha000_w4_2_6__s6: Other Relatives Own House[6]

	Freq.	%
0 Do Not Own	1	100.00
Total	1	100.00

ha000_w4_2_7__s6: Other Relatives Own House[7]

	Freq.	%
0 Do Not Own	1	100.00
Total	1	100.00

ha000_w4_2_1__s7: Friends Own House[1]

	Freq.	%
0 Do Not Own	2,262	99.96
7 Own	1	0.04
Total	2,263	100.00

ha000_w4_2_2__s7: Friends Own House[2]

	Freq.	%
0 Do Not Own	1,736	100.00
Total	1,736	100.00

ha000_w4_2_3__s7: Friends Own House[3]

	Freq.	%
0 Do Not Own	953	100.00
Total	953	100.00

ha000_w4_2_4__s7: Friends Own House[4]

	Freq.	%
0 Do Not Own	72	100.00
Total	72	100.00

ha000_w4_2_5__s7: Friends Own House[5]

	Freq.	%
0 Do Not Own	15	100.00
Total	15	100.00

ha000_w4_2_6__s7: Friends Own House[6]

	Freq.	%
0 Do Not Own	1	100.00
Total	1	100.00

ha000_w4_2_7__s7: Friends Own House[7]

	Freq.	%
0 Do Not Own	1	100.00
Total	1	100.00

ha000_w4_2_1__s8: Others Own House[1]

	Freq.	%
0 Do Not Own	2,161	95.49
8 Own	102	4.51
Total	2,263	100.00

ha000_w4_2_2__s8: Others Own House[2]

	Freq.	%
0 Do Not Own	1,659	95.56
8 Own	77	4.44
Total	1,736	100.00

ha000_w4_2_3__s8: Others Own House[3]

	Freq.	%
0 Do Not Own	918	96.33
8 Own	35	3.67
Total	953	100.00

ha000_w4_2_4__s8: Others Own House[4]

	Freq.	%
0 Do Not Own	66	91.67
8 Own	6	8.33
Total	72	100.00

ha000_w4_2_5__s8: Others Own House[5]

	Freq.	%

	Freq.	%
0 Do Not Own	15	100.00
Total	15	100.00

ha000_w4_2_6__s8: Others Own House[6]

	Freq.	%
0 Do Not Own	1	100.00
Total	1	100.00

ha000_w4_2_7__s8: Others Own House[7]

	Freq.	%
0 Do Not Own	1	100.00
Total	1	100.00

ha000_w4_2_1__s9: Own House[1] But do Not Have Ownership Certificate

	Freq.	%
0 Do Not Own	2,163	95.58
9 Own	100	4.42
Total	2,263	100.00

ha000_w4_2_2__s9: Own House[2] But do Not Have Ownership Certificate

	Freq.	%
0 Do Not Own	1,667	96.03
9 Own	69	3.97
Total	1,736	100.00

ha000_w4_2_3__s9: Own House[3] But do Not Have Ownership Certificate

	Freq.	%
0 Do Not Own	916	96.12
9 Own	37	3.88
Total	953	100.00

ha000_w4_2_4__s9: Own House[4] But do Not Have Ownership Certificate

	Freq.	%
0 Do Not Own	69	95.83
9 Own	3	4.17
Total	72	100.00

ha000_w4_2_5__s9: Own House[5] But do Not Have Ownership Certificate

	Freq.	%
0 Do Not Own	14	93.33
9 Own	1	6.67

	Freq.	%
Total	15	100.00

ha000_w4_2_6__s9: Own House[6] But do Not Have Ownership Certificate

	Freq.	%
0 Do Not Own	1	100.00
Total	1	100.00

ha000_w4_2_7__s9: Own House[7] But do Not Have Ownership Certificate

	Freq.	%
0 Do Not Own	1	100.00
Total	1	100.00

ha000_w4_2_1__s999: Refused to Answer House[1] Ownership

	Freq.	%
0 No	2,257	99.73
999 Yes	6	0.27
Total	2,263	100.00

ha000_w4_2_2__s999: Refused to Answer House[2] Ownership

	Freq.	%
0 No	1,723	99.25
999 Yes	13	0.75
Total	1,736	100.00

ha000_w4_2_3__s999: Refused to Answer House[3] Ownership

	Freq.	%
0 No	940	98.64
999 Yes	13	1.36
Total	953	100.00

ha000_w4_2_4__s999: Refused to Answer House[4] Ownership

	Freq.	%
0 No	71	98.61
999 Yes	1	1.39
Total	72	100.00

ha000_w4_2_5__s999: Refused to Answer House[5] Ownership

	Freq.	%
0 No	15	100.00
Total	15	100.00

ha000_w4_2_6__s999: Refused to Answer House[6] Ownership

	Freq.	%
0 No	1	100.00
Total	1	100.00

ha000_w4_2_7__s999: Refused to Answer House[7] Ownership

	Freq.	%
0 No	1	100.00
Total	1	100.00

ha000_w4_2_1_1_: Percent Household Head and Spouse Own House[1]

Mean	SD	Min	Max	Obs
93.23	18.85	0.00	100.00	1,655

ha000_w4_2_1_2_: Percent Household Head and Spouse Own House[2]

Mean	SD	Min	Max	Obs
90.36	22.51	0.00	100.00	1,195

ha000_w4_2_1_3_: Percent Household Head and Spouse Own House[3]

Mean	SD	Min	Max	Obs
92.04	20.53	0.00	100.00	679

ha000_w4_2_1_4_: Percent Household Head and Spouse Own House[4]

Mean	SD	Min	Max	Obs
95.73	17.04	20.00	100.00	48

ha000_w4_2_1_5_: Percent Household Head and Spouse Own House[5]

Mean	SD	Min	Max	Obs
95.83	14.43	50.00	100.00	12

ha000_w4_2_1_6_: Percent Household Head and Spouse Own House[6]

Mean	SD	Min	Max	Obs
100.00	.	100.00	100.00	1

ha000_w4_2_1_7_: Percent Household Head and Spouse Own House[7]

Mean	SD	Min	Max	Obs
100.00	.	100.00	100.00	1

ha000_w4_2_3_1_: Percent Siblings Own House[1]

Mean	SD	Min	Max	Obs
67.51	25.16	20.00	100.00	22

ha000_w4_2_3_2_: Percent Siblings Own House[2]

Mean	SD	Min	Max	Obs
68.14	23.62	20.00	100.00	19

ha000_w4_2_3_3_: Percent Siblings Own House[3]

Mean	SD	Min	Max	Obs
71.36	29.44	20.00	100.00	14

ha000_w4_2_6_1_: Percent Other Relatives Own House[1]

Mean	SD	Min	Max	Obs
70.14	30.39	25.00	100.00	7

ha000_w4_2_6_2_: Percent Other Relatives Own House[2]

Mean	SD	Min	Max	Obs
85.00	22.36	50.00	100.00	5

ha000_w4_2_6_3_: Percent Other Relatives Own House[3]

Mean	SD	Min	Max	Obs
75.00	35.36	50.00	100.00	2

ha000_w4_2_7_1_: Percent Friends Own House[1]

Mean	SD	Min	Max	Obs
100.00	.	100.00	100.00	1

ha000_w4_2_8_1_: Percent Others Own House[1]

Mean	SD	Min	Max	Obs
85.77	32.41	0.00	100.00	106

ha000_w4_2_8_2_: Percent Others Own House[2]

Mean	SD	Min	Max	Obs
77.70	36.87	0.00	100.00	80

ha000_w4_2_8_3_: Percent Others Own House[3]

Mean	SD	Min	Max	Obs
82.31	35.09	0.00	100.00	36

ha000_w4_2_8_4_: Percent Others Own House[4]

Mean	SD	Min	Max	Obs
100.00	0.00	100.00	100.00	6

ha000_w4_3_1__s1: XChildName[1]/'s Spouse Owns House[1]

	Freq.	%
0 Do Not Own	217	41.57
1 Own	305	58.43
Total	522	100.00

ha000_w4_3_2__s1: XChildName[1]/'s Spouse Owns House[2]

	Freq.	%
0 Do Not Own	186	35.98
1 Own	331	64.02
Total	517	100.00

ha000_w4_3_3__s1: XChildName[1]/'s Spouse Owns House[3]

	Freq.	%
0 Do Not Own	97	37.45
1 Own	162	62.55
Total	259	100.00

ha000_w4_3_4__s1: XChildName[1]/'s Spouse Owns House[4]

	Freq.	%
0 Do Not Own	9	56.25
1 Own	7	43.75
Total	16	100.00

ha000_w4_3_5__s1: XChildName[1]/'s Spouse Owns House[5]

	Freq.	%
1 Own	3	100.00
Total	3	100.00

ha000_w4_3_1__s2: XChildName[2]/'s Spouse Owns House[1]

	Freq.	%
0 Do Not Own	364	69.73
2 Own	158	30.27

Total	522	100.00

ha000_w4_3_2__s2: XChildName[2]/'s Spouse Owns House[2]

	Freq.	%
0 Do Not Own	375	72.53
2 Own	142	27.47
Total	517	100.00

ha000_w4_3_3__s2: XChildName[2]/'s Spouse Owns House[3]

	Freq.	%
0 Do Not Own	199	76.83
2 Own	60	23.17
Total	259	100.00

ha000_w4_3_4__s2: XChildName[2]/'s Spouse Owns House[4]

	Freq.	%
0 Do Not Own	9	56.25
2 Own	7	43.75
Total	16	100.00

ha000_w4_3_5__s2: XChildName[2]/'s Spouse Owns House[5]

	Freq.	%
0 Do Not Own	2	66.67
2 Own	1	33.33
Total	3	100.00

ha000_w4_3_1__s3: XChildName[3]/'s Spouse Owns House[1]

	Freq.	%
0 Do Not Own	455	87.16
3 Own	67	12.84
Total	522	100.00

ha000_w4_3_2__s3: XChildName[3]/'s Spouse Owns House[2]

	Freq.	%
0 Do Not Own	454	87.81
3 Own	63	12.19
Total	517	100.00

ha000_w4_3_3__s3: XChildName[3]/'s Spouse Owns House[3]

	Freq.	%
0 Do Not Own	210	81.08
3 Own	49	18.92

	Freq.	%
Total	259	100.00

ha000_w4_3_4__s3: XChildName[3]/'s Spouse Owns House[4]

	Freq.	%
0 Do Not Own	12	75.00
3 Own	4	25.00
Total	16	100.00

ha000_w4_3_5__s3: XChildName[3]/'s Spouse Owns House[5]

	Freq.	%
0 Do Not Own	3	100.00
Total	3	100.00

ha000_w4_3_1__s4: XChildName[4]/'s Spouse Owns House[1]

	Freq.	%
0 Do Not Own	474	90.80
4 Own	48	9.20
Total	522	100.00

ha000_w4_3_2__s4: XChildName[4]/'s Spouse Owns House[2]

	Freq.	%
0 Do Not Own	485	93.81
4 Own	32	6.19
Total	517	100.00

ha000_w4_3_3__s4: XChildName[4]/'s Spouse Owns House[3]

	Freq.	%
0 Do Not Own	242	93.44
4 Own	17	6.56
Total	259	100.00

ha000_w4_3_4__s4: XChildName[4]/'s Spouse Owns House[4]

	Freq.	%
0 Do Not Own	15	93.75
4 Own	1	6.25
Total	16	100.00

ha000_w4_3_5__s4: XChildName[4]/'s Spouse Owns House[5]

	Freq.	%
0 Do Not Own	3	100.00
Total	3	100.00

ha000_w4_3_1__s5: XChildName[5]/'s Spouse Owns House[1]

	Freq.	%
0 Do Not Own	504	96.55
5 Own	18	3.45
Total	522	100.00

ha000_w4_3_2__s5: XChildName[5]/'s Spouse Owns House[2]

	Freq.	%
0 Do Not Own	503	97.29
5 Own	14	2.71
Total	517	100.00

ha000_w4_3_3__s5: XChildName[5]/'s Spouse Owns House[3]

	Freq.	%
0 Do Not Own	249	96.14
5 Own	10	3.86
Total	259	100.00

ha000_w4_3_4__s5: XChildName[5]/'s Spouse Owns House[4]

	Freq.	%
0 Do Not Own	16	100.00
Total	16	100.00

ha000_w4_3_5__s5: XChildName[5]/'s Spouse Owns House[5]

	Freq.	%
0 Do Not Own	3	100.00
Total	3	100.00

ha000_w4_3_1__s6: XChildName[6]/'s Spouse Owns House[1]

	Freq.	%
0 Do Not Own	514	98.47
6 Own	8	1.53
Total	522	100.00

ha000_w4_3_2__s6: XChildName[6]/'s Spouse Owns House[2]

	Freq.	%
0 Do Not Own	510	98.65
6 Own	7	1.35
Total	517	100.00

ha000_w4_3_3__s6: XChildName[6]/'s Spouse Owns House[3]

	Freq.	%
0 Do Not Own	256	98.84
6 Own	3	1.16
Total	259	100.00

ha000_w4_3_4__s6: XChildName[6]/'s Spouse Owns House[4]

	Freq.	%
0 Do Not Own	16	100.00
Total	16	100.00

ha000_w4_3_5__s6: XChildName[6]/'s Spouse Owns House[5]

	Freq.	%
0 Do Not Own	3	100.00
Total	3	100.00

ha000_w4_3_1__s7: XChildName[7]/'s Spouse Owns House[1]

	Freq.	%
0 Do Not Own	521	99.81
7 Own	1	0.19
Total	522	100.00

ha000_w4_3_2__s7: XChildName[7]/'s Spouse Owns House[2]

	Freq.	%
0 Do Not Own	515	99.61
7 Own	2	0.39
Total	517	100.00

ha000_w4_3_3__s7: XChildName[7]/'s Spouse Owns House[3]

	Freq.	%
0 Do Not Own	259	100.00
Total	259	100.00

ha000_w4_3_4__s7: XChildName[7]/'s Spouse Owns House[4]

	Freq.	%
0 Do Not Own	16	100.00
Total	16	100.00

ha000_w4_3_5__s7: XChildName[7]/'s Spouse Owns House[5]

	Freq.	%
0 Do Not Own	3	100.00

Total	3	100.00

ha000_w4_3_1__s8: XChildName[8]/'s Spouse Owns House[1]

	Freq.	%
0 Do Not Own	521	99.81
8 Own	1	0.19
Total	522	100.00

ha000_w4_3_2__s8: XChildName[8]/'s Spouse Owns House[2]

	Freq.	%
0 Do Not Own	517	100.00
Total	517	100.00

ha000_w4_3_3__s8: XChildName[8]/'s Spouse Owns House[3]

	Freq.	%
0 Do Not Own	259	100.00
Total	259	100.00

ha000_w4_3_4__s8: XChildName[8]/'s Spouse Owns House[4]

	Freq.	%
0 Do Not Own	15	93.75
8 Own	1	6.25
Total	16	100.00

ha000_w4_3_5__s8: XChildName[8]/'s Spouse Owns House[5]

	Freq.	%
0 Do Not Own	3	100.00
Total	3	100.00

ha000_w4_3_1__s9: XChildName[9]/'s Spouse Owns House[1]

	Freq.	%
0 Do Not Own	522	100.00
Total	522	100.00

ha000_w4_3_2__s9: XChildName[9]/'s Spouse Owns House[2]

	Freq.	%
0 Do Not Own	517	100.00
Total	517	100.00

ha000_w4_3_3__s9: XChildName[9]/'s Spouse Owns House[3]

	Freq.	%
0 Do Not Own	259	100.00
Total	259	100.00

ha000_w4_3_4__s9: XChildName[9]/'s Spouse Owns House[4]

	Freq.	%
0 Do Not Own	16	100.00
Total	16	100.00

ha000_w4_3_5__s9: XChildName[9]/'s Spouse Owns House[5]

	Freq.	%
0 Do Not Own	3	100.00
Total	3	100.00

ha000_w4_3_1__s10: XChildName[10]/'s Spouse Owns House[1]

	Freq.	%
0 Do Not Own	522	100.00
Total	522	100.00

ha000_w4_3_2__s10: XChildName[10]/'s Spouse Owns House[2]

	Freq.	%
0 Do Not Own	517	100.00
Total	517	100.00

ha000_w4_3_3__s10: XChildName[10]/'s Spouse Owns House[3]

	Freq.	%
0 Do Not Own	259	100.00
Total	259	100.00

ha000_w4_3_4__s10: XChildName[10]/'s Spouse Owns House[4]

	Freq.	%
0 Do Not Own	16	100.00
Total	16	100.00

ha000_w4_3_5__s10: XChildName[10]/'s Spouse Owns House[5]

	Freq.	%
0 Do Not Own	3	100.00
Total	3	100.00

ha000_w4_3_1__s11: XChildName[11]/'s Spouse Owns House[1]

	Freq.	%
0 Do Not Own	522	100.00
Total	522	100.00

ha000_w4_3_2__s11: XChildName[11]/'s Spouse Owns House[2]

	Freq.	%
0 Do Not Own	516	99.81
11 Own	1	0.19
Total	517	100.00

ha000_w4_3_3__s11: XChildName[11]/'s Spouse Owns House[3]

	Freq.	%
0 Do Not Own	258	99.61
11 Own	1	0.39
Total	259	100.00

ha000_w4_3_4__s11: XChildName[11]/'s Spouse Owns House[4]

	Freq.	%
0 Do Not Own	16	100.00
Total	16	100.00

ha000_w4_3_5__s11: XChildName[11]/'s Spouse Owns House[5]

	Freq.	%
0 Do Not Own	3	100.00
Total	3	100.00

ha000_w4_3_1__s12: XChildName[12]/'s Spouse Owns House[1]

	Freq.	%
0 Do Not Own	522	100.00
Total	522	100.00

ha000_w4_3_2__s12: XChildName[12]/'s Spouse Owns House[2]

	Freq.	%
0 Do Not Own	517	100.00
Total	517	100.00

ha000_w4_3_3__s12: XChildName[12]/'s Spouse Owns House[3]

	Freq.	%
0 Do Not Own	259	100.00
Total	259	100.00

ha000_w4_3_4__s12: XChildName[12]/'s Spouse Owns House[4]

	Freq.	%
0 Do Not Own	16	100.00
Total	16	100.00

ha000_w4_3_5__s12: XChildName[12]/'s Spouse Owns House[5]

	Freq.	%
0 Do Not Own	3	100.00
Total	3	100.00

ha000_w4_3_1__s13: XChildName[13]/'s Spouse Owns House[1]

	Freq.	%
0 Do Not Own	522	100.00
Total	522	100.00

ha000_w4_3_2__s13: XChildName[13]/'s Spouse Owns House[2]

	Freq.	%
0 Do Not Own	517	100.00
Total	517	100.00

ha000_w4_3_3__s13: XChildName[13]/'s Spouse Owns House[3]

	Freq.	%
0 Do Not Own	259	100.00
Total	259	100.00

ha000_w4_3_4__s13: XChildName[13]/'s Spouse Owns House[4]

	Freq.	%
0 Do Not Own	16	100.00
Total	16	100.00

ha000_w4_3_5__s13: XChildName[13]/'s Spouse Owns House[5]

	Freq.	%
0 Do Not Own	3	100.00
Total	3	100.00

ha000_w4_3_1__s14: XChildName[14]/'s Spouse Owns House[1]

	Freq.	%
0 Do Not Own	522	100.00
Total	522	100.00

ha000_w4_3_2__s14: XChildName[14]/'s Spouse Owns House[2]

	Freq.	%
0 Do Not Own	517	100.00
Total	517	100.00

ha000_w4_3_3__s14: XChildName[14]/'s Spouse Owns House[3]

	Freq.	%
0 Do Not Own	259	100.00
Total	259	100.00

ha000_w4_3_4__s14: XChildName[14]/'s Spouse Owns House[4]

	Freq.	%
0 Do Not Own	16	100.00
Total	16	100.00

ha000_w4_3_5__s14: XChildName[14]/'s Spouse Owns House[5]

	Freq.	%
0 Do Not Own	3	100.00
Total	3	100.00

ha000_w4_3_1__s15: XChildName[15]/'s Spouse Owns House[1]

	Freq.	%
0 Do Not Own	522	100.00
Total	522	100.00

ha000_w4_3_2__s15: XChildName[15]/'s Spouse Owns House[2]

	Freq.	%
0 Do Not Own	517	100.00
Total	517	100.00

ha000_w4_3_3__s15: XChildName[15]/'s Spouse Owns House[3]

	Freq.	%
0 Do Not Own	259	100.00
Total	259	100.00

ha000_w4_3_4__s15: XChildName[15]/'s Spouse Owns House[4]

	Freq.	%
0 Do Not Own	16	100.00
Total	16	100.00

ha000_w4_3_5__s15: XChildName[15]/'s Spouse Owns House[5]

	Freq.	%
0 Do Not Own	3	100.00
Total	3	100.00

ha000_w4_3_1__s16: XChildName[16]/'s Spouse Owns House[1]

	Freq.	%
0 Do Not Own	522	100.00
Total	522	100.00

ha000_w4_3_2__s16: XChildName[16]/'s Spouse Owns House[2]

	Freq.	%
0 Do Not Own	517	100.00
Total	517	100.00

ha000_w4_3_3__s16: XChildName[16]/'s Spouse Owns House[3]

	Freq.	%
0 Do Not Own	259	100.00
Total	259	100.00

ha000_w4_3_4__s16: XChildName[16]/'s Spouse Owns House[4]

	Freq.	%
0 Do Not Own	16	100.00
Total	16	100.00

ha000_w4_3_5__s16: XChildName[16]/'s Spouse Owns House[5]

	Freq.	%
0 Do Not Own	3	100.00
Total	3	100.00

ha000_w4_3_1__s99: None of the Above Owns House[1]

	Freq.	%
0 No	515	98.66
99 Yes	7	1.34
Total	522	100.00

ha000_w4_3_2__s99: None of the Above Owns House[2]

	Freq.	%
0 No	512	99.03
99 Yes	5	0.97

Total	517	100.00

ha000_w4_3_3__s99: None of the Above Owns House[3]

	Freq.	%
0 No	252	97.30
99 Yes	7	2.70
Total	259	100.00

ha000_w4_3_4__s99: None of the Above Owns House[4]

	Freq.	%
0 No	16	100.00
Total	16	100.00

ha000_w4_3_5__s99: None of the Above Owns House[5]

	Freq.	%
0 No	3	100.00
Total	3	100.00

ha000_w4_4_1__1_: Percent XChildName[1]/'s Spouse Owns House[1]

Mean	SD	Min	Max	Obs
75.70	30.43	0.00	100.00	305

ha000_w4_4_2__1_: Percent XChildName[1]/'s Spouse Owns House[2]

Mean	SD	Min	Max	Obs
76.51	31.21	0.00	100.00	331

ha000_w4_4_3__1_: Percent XChildName[1]/'s Spouse Owns House[3]

Mean	SD	Min	Max	Obs
74.27	31.19	14.00	100.00	162

ha000_w4_4_4__1_: Percent XChildName[1]/'s Spouse Owns House[4]

Mean	SD	Min	Max	Obs
89.29	28.35	25.00	100.00	7

ha000_w4_4_5__1_: Percent XChildName[1]/'s Spouse Owns House[5]

Mean	SD	Min	Max	Obs
75.00	43.30	25.00	100.00	3

ha000_w4_4_1__2_: Percent XChildName[2]/'s Spouse Owns House[1]

Mean	SD	Min	Max	Obs
68.92	32.77	10.00	100.00	158

ha000_w4_4_2__2_: Percent XChildName[2]/'s Spouse Owns House[2]

Mean	SD	Min	Max	Obs
72.00	33.54	0.00	100.00	142

ha000_w4_4_3__2_: Percent XChildName[2]/'s Spouse Owns House[3]

Mean	SD	Min	Max	Obs
63.53	34.67	14.00	100.00	60

ha000_w4_4_4__2_: Percent XChildName[2]/'s Spouse Owns House[4]

Mean	SD	Min	Max	Obs
76.86	33.58	25.00	100.00	7

ha000_w4_4_5__2_: Percent XChildName[2]/'s Spouse Owns House[5]

Mean	SD	Min	Max	Obs
25.00	.	25.00	25.00	1

ha000_w4_4_1__3_: Percent XChildName[3]/'s Spouse Owns House[1]

Mean	SD	Min	Max	Obs
60.93	31.13	10.00	100.00	67

ha000_w4_4_2__3_: Percent XChildName[3]/'s Spouse Owns House[2]

Mean	SD	Min	Max	Obs
71.10	33.97	7.50	100.00	63

ha000_w4_4_3__3_: Percent XChildName[3]/'s Spouse Owns House[3]

Mean	SD	Min	Max	Obs
74.04	32.86	15.00	100.00	49

ha000_w4_4_4__3_: Percent XChildName[3]/'s Spouse Owns House[4]

Mean	SD	Min	Max	Obs
64.50	41.12	25.00	100.00	4

ha000_w4_4_1__4_: Percent XChildName[4]/'s Spouse Owns House[1]

Mean	SD	Min	Max	Obs
79.79	31.38	10.00	100.00	48

ha000_w4_4_2__4_: Percent XChildName[4]/'s Spouse Owns House[2]

Mean	SD	Min	Max	Obs
63.17	36.94	0.00	100.00	32

ha000_w4_4_3__4_: Percent XChildName[4]/'s Spouse Owns House[3]

Mean	SD	Min	Max	Obs
61.18	35.16	15.00	100.00	17

ha000_w4_4_4__4_: Percent XChildName[4]/'s Spouse Owns House[4]

Mean	SD	Min	Max	Obs
33.00	.	33.00	33.00	1

ha000_w4_4_1__5_: Percent XChildName[5]/'s Spouse Owns House[1]

Mean	SD	Min	Max	Obs
87.94	27.78	25.00	100.00	18

ha000_w4_4_2__5_: Percent XChildName[5]/'s Spouse Owns House[2]

Mean	SD	Min	Max	Obs
66.61	38.60	0.00	100.00	14

ha000_w4_4_3__5_: Percent XChildName[5]/'s Spouse Owns House[3]

Mean	SD	Min	Max	Obs
56.30	31.70	25.00	100.00	10

ha000_w4_4_1__6_: Percent XChildName[6]/'s Spouse Owns House[1]

Mean	SD	Min	Max	Obs
76.88	32.62	25.00	100.00	8

ha000_w4_4_2__6_: Percent XChildName[6]/'s Spouse Owns House[2]

Mean	SD	Min	Max	Obs
58.93	39.94	7.50	100.00	7

ha000_w4_4_3__6_: Percent XChildName[6]/'s Spouse Owns House[3]

Mean	SD	Min	Max	Obs
83.33	28.87	50.00	100.00	3

ha000_w4_4_1__7_: Percent XChildName[7]/'s Spouse Owns House[1]

Mean	SD	Min	Max	Obs
100.00	.	100.00	100.00	1

ha000_w4_4_2__7_: Percent XChildName[7]/'s Spouse Owns House[2]

Mean	SD	Min	Max	Obs
100.00	0.00	100.00	100.00	2

ha000_w4_4_1__8_: Percent XChildName[8]/'s Spouse Owns House[1]

Mean	SD	Min	Max	Obs
100.00	.	100.00	100.00	1

ha000_w4_4_4__8_: Percent XChildName[8]/'s Spouse Owns House[4]

Mean	SD	Min	Max	Obs
100.00	.	100.00	100.00	1

ha000_w4_4_2__11_: Percent XChildName[11]/'s Spouse Owns House[2]

Mean	SD	Min	Max	Obs
100.00	.	100.00	100.00	1

ha000_w4_4_3__11_: Percent XChildName[11]/'s Spouse Owns House[3]

Mean	SD	Min	Max	Obs
100.00	.	100.00	100.00	1

ha000_w4_5_1__s1: XConParName[1] Owns House[1]

	Freq.	%
0 Do Not Own	19	67.86
1 Own	9	32.14
Total	28	100.00

ha000_w4_5_2__s1: XConParName[1] Owns House[2]

	Freq.	%
0 Do Not Own	13	92.86
1 Own	1	7.14
Total	14	100.00

ha000_w4_5_3__s1: XConParName[1] Owns House[3]

	Freq.	%
0 Do Not Own	6	85.71
1 Own	1	14.29
Total	7	100.00

ha000_w4_5_1__s2: XConParName[2] Owns House[1]

	Freq.	%
0 Do Not Own	18	64.29
2 Own	10	35.71
Total	28	100.00

ha000_w4_5_2__s2: XConParName[2] Owns House[2]

	Freq.	%
0 Do Not Own	11	78.57
2 Own	3	21.43
Total	14	100.00

ha000_w4_5_3__s2: XConParName[2] Owns House[3]

	Freq.	%
0 Do Not Own	6	85.71
2 Own	1	14.29
Total	7	100.00

ha000_w4_5_1__s3: XConParName[3] Owns House[1]

	Freq.	%
0 Do Not Own	28	100.00
Total	28	100.00

ha000_w4_5_2__s3: XConParName[3] Owns House[2]

	Freq.	%
0 Do Not Own	14	100.00
Total	14	100.00

ha000_w4_5_3__s3: XConParName[3] Owns House[3]

	Freq.	%
0 Do Not Own	7	100.00
Total	7	100.00

ha000_w4_5_1__s4: XConParName[4] Owns House[1]

	Freq.	%
0 Do Not Own	28	100.00
Total	28	100.00

ha000_w4_5_2__s4: XConParName[4] Owns House[2]

	Freq.	%
0 Do Not Own	14	100.00
Total	14	100.00

ha000_w4_5_3__s4: XConParName[4] Owns House[3]

	Freq.	%
0 Do Not Own	7	100.00
Total	7	100.00

ha000_w4_5_1__s5: XConParName[5] Owns House[1]

	Freq.	%
0 Do Not Own	23	82.14
5 Own	5	17.86
Total	28	100.00

ha000_w4_5_2__s5: XConParName[5] Owns House[2]

	Freq.	%
0 Do Not Own	12	85.71
5 Own	2	14.29
Total	14	100.00

ha000_w4_5_3__s5: XConParName[5] Owns House[3]

	Freq.	%
0 Do Not Own	6	85.71
5 Own	1	14.29
Total	7	100.00

ha000_w4_5_1__s6: XConParName[6] Owns House[1]

	Freq.	%
0 Do Not Own	25	89.29
6 Own	3	10.71
Total	28	100.00

ha000_w4_5_2__s6: XConParName[6] Owns House[2]

	Freq.	%

	Freq.	%
0 Do Not Own	11	78.57
6 Own	3	21.43
Total	14	100.00

ha000_w4_5_3__s6: XConParName[6] Owns House[3]

	Freq.	%
0 Do Not Own	7	100.00
Total	7	100.00

ha000_w4_5_1__s7: XConParName[7] Owns House[1]

	Freq.	%
0 Do Not Own	28	100.00
Total	28	100.00

ha000_w4_5_2__s7: XConParName[7] Owns House[2]

	Freq.	%
0 Do Not Own	14	100.00
Total	14	100.00

ha000_w4_5_3__s7: XConParName[7] Owns House[3]

	Freq.	%
0 Do Not Own	7	100.00
Total	7	100.00

ha000_w4_5_1__s8: XConParName[8] Owns House[1]

	Freq.	%
0 Do Not Own	28	100.00
Total	28	100.00

ha000_w4_5_2__s8: XConParName[8] Owns House[2]

	Freq.	%
0 Do Not Own	13	92.86
8 Own	1	7.14
Total	14	100.00

ha000_w4_5_3__s8: XConParName[8] Owns House[3]

	Freq.	%
0 Do Not Own	7	100.00
Total	7	100.00

ha000_w4_5_1__s99: None of the Above Owns House[1]

	Freq.	%
0 No	19	67.86
99 Yes	9	32.14
Total	28	100.00

ha000_w4_5_2__s99: None of the Above Owns House[2]

	Freq.	%
0 No	7	50.00
99 Yes	7	50.00
Total	14	100.00

ha000_w4_5_3__s99: None of the Above Owns House[3]

	Freq.	%
0 No	3	42.86
99 Yes	4	57.14
Total	7	100.00

ha000_w4_6_1__1_: Percent XConParName[1] Owns House[1]

Mean	SD	Min	Max	Obs
56.39	35.47	12.50	100.00	9

ha000_w4_6_2__1_: Percent XConParName[1] Owns House[2]

Mean	SD	Min	Max	Obs
12.50	.	12.50	12.50	1

ha000_w4_6_3__1_: Percent XConParName[1] Owns House[3]

Mean	SD	Min	Max	Obs
100.00	.	100.00	100.00	1

ha000_w4_6_1__2_: Percent XConParName[2] Owns House[1]

Mean	SD	Min	Max	Obs
60.75	36.17	12.50	100.00	10

ha000_w4_6_2__2_: Percent XConParName[2] Owns House[2]

Mean	SD	Min	Max	Obs
59.50	44.11	12.50	100.00	3

ha000_w4_6_3__2_: Percent XConParName[2] Owns House[3]

Mean	SD	Min	Max	Obs
100.00	.	100.00	100.00	1

ha000_w4_6_1__5_: Percent XConParName[5] Owns House[1]

Mean	SD	Min	Max	Obs
70.00	27.39	50.00	100.00	5

ha000_w4_6_2__5_: Percent XConParName[5] Owns House[2]

Mean	SD	Min	Max	Obs
37.50	17.68	25.00	50.00	2

ha000_w4_6_3__5_: Percent XConParName[5] Owns House[3]

Mean	SD	Min	Max	Obs
100.00	.	100.00	100.00	1

ha000_w4_6_1__6_: Percent XConParName[6] Owns House[1]

Mean	SD	Min	Max	Obs
44.67	9.24	34.00	50.00	3

ha000_w4_6_2__6_: Percent XConParName[6] Owns House[2]

Mean	SD	Min	Max	Obs
58.33	38.19	25.00	100.00	3

ha000_w4_6_2__8_: Percent XConParName[8] Owns House[2]

Mean	SD	Min	Max	Obs
100.00	.	100.00	100.00	1

ha000_w4_7_1__s1: XChildName[1]'s Child(Grandchild) Owns House[1]

	Freq.	%
0 Do Not Own	7	36.84
1 Own	12	63.16
Total	19	100.00

ha000_w4_7_2__s1: XChildName[1]'s Child(Grandchild) Owns House[2]

	Freq.	%
0 Do Not Own	8	27.59
1 Own	21	72.41
Total	29	100.00

ha000_w4_7_3__s1: XChildName[1]'s Child(Grandchild) Owns House[3]

	Freq.	%
0 Do Not Own	2	12.50
1 Own	14	87.50
Total	16	100.00

ha000_w4_7_1__s2: XChildName[2]'s Child(Grandchild) Owns House[1]

	Freq.	%
0 Do Not Own	15	78.95
2 Own	4	21.05
Total	19	100.00

ha000_w4_7_2__s2: XChildName[2]'s Child(Grandchild) Owns House[2]

	Freq.	%
0 Do Not Own	26	89.66
2 Own	3	10.34
Total	29	100.00

ha000_w4_7_3__s2: XChildName[2]'s Child(Grandchild) Owns House[3]

	Freq.	%
0 Do Not Own	15	93.75
2 Own	1	6.25
Total	16	100.00

ha000_w4_7_1__s3: XChildName[3]'s Child(Grandchild) Owns House[1]

	Freq.	%
0 Do Not Own	17	89.47
3 Own	2	10.53
Total	19	100.00

ha000_w4_7_2__s3: XChildName[3]'s Child(Grandchild) Owns House[2]

	Freq.	%
0 Do Not Own	25	86.21
3 Own	4	13.79
Total	29	100.00

ha000_w4_7_3__s3: XChildName[3]'s Child(Grandchild) Owns House[3]

	Freq.	%
0 Do Not Own	15	93.75
3 Own	1	6.25
Total	16	100.00

ha000_w4_7_1__s4: XChildName[4]'s Child(Grandchild) Owns House[1]

	Freq.	%
0 Do Not Own	19	100.00
Total	19	100.00

ha000_w4_7_2__s4: XChildName[4]'s Child(Grandchild) Owns House[2]

	Freq.	%
0 Do Not Own	29	100.00
Total	29	100.00

ha000_w4_7_3__s4: XChildName[4]'s Child(Grandchild) Owns House[3]

	Freq.	%
0 Do Not Own	16	100.00
Total	16	100.00

ha000_w4_7_1__s5: XChildName[5]'s Child(Grandchild) Owns House[1]

	Freq.	%
0 Do Not Own	18	94.74
5 Own	1	5.26
Total	19	100.00

ha000_w4_7_2__s5: XChildName[5]'s Child(Grandchild) Owns House[2]

	Freq.	%
0 Do Not Own	29	100.00
Total	29	100.00

ha000_w4_7_3__s5: XChildName[5]'s Child(Grandchild) Owns House[3]

	Freq.	%
0 Do Not Own	15	93.75
5 Own	1	6.25
Total	16	100.00

ha000_w4_7_1__s6: XChildName[6]'s Child(Grandchild) Owns House[1]

	Freq.	%
0 Do Not Own	18	94.74
6 Own	1	5.26
Total	19	100.00

ha000_w4_7_2__s6: XChildName[6]'s Child(Grandchild) Owns House[2]

	Freq.	%

	Freq.	%
0 Do Not Own	27	93.10
6 Own	2	6.90
Total	29	100.00

ha000_w4_7_3__s6: XChildName[6]'s Child(Grandchild) Owns House[3]

	Freq.	%
0 Do Not Own	16	100.00
Total	16	100.00

ha000_w4_7_1__s7: XChildName[7]'s Child(Grandchild) Owns House[1]

	Freq.	%
0 Do Not Own	19	100.00
Total	19	100.00

ha000_w4_7_2__s7: XChildName[7]'s Child(Grandchild) Owns House[2]

	Freq.	%
0 Do Not Own	29	100.00
Total	29	100.00

ha000_w4_7_3__s7: XChildName[7]'s Child(Grandchild) Owns House[3]

	Freq.	%
0 Do Not Own	16	100.00
Total	16	100.00

ha000_w4_7_1__s8: XChildName[8]'s Child(Grandchild) Owns House[1]

	Freq.	%
0 Do Not Own	19	100.00
Total	19	100.00

ha000_w4_7_2__s8: XChildName[8]'s Child(Grandchild) Owns House[2]

	Freq.	%
0 Do Not Own	29	100.00
Total	29	100.00

ha000_w4_7_3__s8: XChildName[8]'s Child(Grandchild) Owns House[3]

	Freq.	%
0 Do Not Own	16	100.00
Total	16	100.00

ha000_w4_7_1__s9: XChildName[9]'s Child(Grandchild) Owns House[1]

	Freq.	%
0 Do Not Own	19	100.00
Total	19	100.00

ha000_w4_7_2__s9: XChildName[9]'s Child(Grandchild) Owns House[2]

	Freq.	%
0 Do Not Own	29	100.00
Total	29	100.00

ha000_w4_7_3__s9: XChildName[9]'s Child(Grandchild) Owns House[3]

	Freq.	%
0 Do Not Own	16	100.00
Total	16	100.00

ha000_w4_7_1__s10: XChildName[10]'s Child(Grandchild) Owns House[1]

	Freq.	%
0 Do Not Own	19	100.00
Total	19	100.00

ha000_w4_7_2__s10: XChildName[10]'s Child(Grandchild) Owns House[2]

	Freq.	%
0 Do Not Own	29	100.00
Total	29	100.00

ha000_w4_7_3__s10: XChildName[10]'s Child(Grandchild) Owns House[3]

	Freq.	%
0 Do Not Own	16	100.00
Total	16	100.00

ha000_w4_7_1__s11: XChildName[11]'s Child(Grandchild) Owns House[1]

	Freq.	%
0 Do Not Own	19	100.00
Total	19	100.00

ha000_w4_7_2__s11: XChildName[11]'s Child(Grandchild) Owns House[2]

	Freq.	%
0 Do Not Own	29	100.00
Total	29	100.00

ha000_w4_7_3__s11: XChildName[11]'s Child(Grandchild) Owns House[3]

	Freq.	%
0 Do Not Own	16	100.00
Total	16	100.00

ha000_w4_7_1__s12: XChildName[12]'s Child(Grandchild) Owns House[1]

	Freq.	%
0 Do Not Own	19	100.00
Total	19	100.00

ha000_w4_7_2__s12: XChildName[12]'s Child(Grandchild) Owns House[2]

	Freq.	%
0 Do Not Own	29	100.00
Total	29	100.00

ha000_w4_7_3__s12: XChildName[12]'s Child(Grandchild) Owns House[3]

	Freq.	%
0 Do Not Own	16	100.00
Total	16	100.00

ha000_w4_7_1__s13: XChildName[13]'s Child(Grandchild) Owns House[1]

	Freq.	%
0 Do Not Own	19	100.00
Total	19	100.00

ha000_w4_7_2__s13: XChildName[13]'s Child(Grandchild) Owns House[2]

	Freq.	%
0 Do Not Own	29	100.00
Total	29	100.00

ha000_w4_7_3__s13: XChildName[13]'s Child(Grandchild) Owns House[3]

	Freq.	%
0 Do Not Own	16	100.00
Total	16	100.00

ha000_w4_7_1__s14: XChildName[14]'s Child(Grandchild) Owns House[1]

	Freq.	%
0 Do Not Own	19	100.00
Total	19	100.00

ha000_w4_7_2__s14: XChildName[14]'s Child(Grandchild) Owns House[2]

	Freq.	%
0 Do Not Own	29	100.00
Total	29	100.00

ha000_w4_7_3__s14: XChildName[14]'s Child(Grandchild) Owns House[3]

	Freq.	%
0 Do Not Own	16	100.00
Total	16	100.00

ha000_w4_7_1__s15: XChildName[15]'s Child(Grandchild) Owns House[1]

	Freq.	%
0 Do Not Own	19	100.00
Total	19	100.00

ha000_w4_7_2__s15: XChildName[15]'s Child(Grandchild) Owns House[2]

	Freq.	%
0 Do Not Own	29	100.00
Total	29	100.00

ha000_w4_7_3__s15: XChildName[15]'s Child(Grandchild) Owns House[3]

	Freq.	%
0 Do Not Own	16	100.00
Total	16	100.00

ha000_w4_7_1__s16: XChildName[16]'s Child(Grandchild) Owns House[1]

	Freq.	%
0 Do Not Own	19	100.00
Total	19	100.00

ha000_w4_7_2__s16: XChildName[16]'s Child(Grandchild) Owns House[2]

	Freq.	%
0 Do Not Own	29	100.00
Total	29	100.00

ha000_w4_7_3__s16: XChildName[16]'s Child(Grandchild) Owns House[3]

	Freq.	%
0 Do Not Own	16	100.00
Total	16	100.00

ha000_w4_8_1__1_: Percent XChildName[1]'s Child(Grandchild) Owns House[1]

Mean	SD	Min	Max	Obs
39.36	29.17	15.00	100.00	12

ha000_w4_8_2__1_: Percent XChildName[1]'s Child(Grandchild) Owns House[2]

Mean	SD	Min	Max	Obs
30.76	17.80	10.00	100.00	21

ha000_w4_8_3__1_: Percent XChildName[1]'s Child(Grandchild) Owns House[3]

Mean	SD	Min	Max	Obs
40.39	27.31	12.50	100.00	14

ha000_w4_8_1__2_: Percent XChildName[2]'s Child(Grandchild) Owns House[1]

Mean	SD	Min	Max	Obs
48.32	35.44	20.00	100.00	4

ha000_w4_8_2__2_: Percent XChildName[2]'s Child(Grandchild) Owns House[2]

Mean	SD	Min	Max	Obs
25.00	15.00	10.00	40.00	3

ha000_w4_8_3__2_: Percent XChildName[2]'s Child(Grandchild) Owns House[3]

Mean	SD	Min	Max	Obs
12.50	.	12.50	12.50	1

ha000_w4_8_1__3_: Percent XChildName[3]'s Child(Grandchild) Owns House[1]

Mean	SD	Min	Max	Obs
100.00	0.00	100.00	100.00	2

ha000_w4_8_2__3_: Percent XChildName[3]'s Child(Grandchild) Owns House[2]

Mean	SD	Min	Max	Obs
75.00	28.87	50.00	100.00	4

ha000_w4_8_3__3_: Percent XChildName[3]'s Child(Grandchild) Owns House[3]

Mean	SD	Min	Max	Obs
40.00	.	40.00	40.00	1

ha000_w4_8_1__5_: Percent XChildName[5]'s Child(Grandchild) Owns House[1]

Mean	SD	Min	Max	Obs
100.00	.	100.00	100.00	1

ha000_w4_8_3__5_: Percent XChildName[5]'s Child(Grandchild) Owns House[3]

Mean	SD	Min	Max	Obs
100.00	.	100.00	100.00	1

ha000_w4_8_1__6_: Percent XChildName[6]'s Child(Grandchild) Owns House[1]

Mean	SD	Min	Max	Obs
20.00	.	20.00	20.00	1

ha000_w4_8_2__6_: Percent XChildName[6]'s Child(Grandchild) Owns House[2]

Mean	SD	Min	Max	Obs
30.00	14.14	20.00	40.00	2

ha001_w4_0_1_: 1th House Ownership Change

	Freq.	%
1 Changes	403	11.95
2 Unchanges	2,919	86.54
997 Don't Know	47	1.39
999 Refused to Answer	4	0.12
Total	3,373	100.00

ha001_w4_0_2_: 2th House Ownership Change

	Freq.	%
1 Changes	341	10.55
2 Unchanges	2,841	87.93
997 Don't Know	41	1.27
999 Refused to Answer	8	0.25
Total	3,231	100.00

ha001_w4_0_3_: 3th House Ownership Change

	Freq.	%
1 Changes	177	9.58
2 Unchanges	1,643	88.96
997 Don't Know	22	1.19
999 Refused to Answer	5	0.27
Total	1,847	100.00

ha001_w4_0_4_: 4th House Ownership Change

	Freq.	%
1 Changes	17	11.89
2 Unchanges	125	87.41
997 Don't Know	1	0.70
Total	143	100.00

ha001_w4_0_5_: 5th House Ownership Change

	Freq.	%
1 Changes	1	4.35
2 Unchanges	22	95.65
Total	23	100.00

ha001_w4_0_6_: 6th House Ownership Change

	Freq.	%
2 Unchanges	3	100.00
Total	3	100.00

ha001_w4_0_7_: 7th House Ownership Change

	Freq.	%
2 Unchanges	1	100.00
Total	1	100.00

ha001_w4_1_1__s1: Household Head and Spouse Own House[1]

	Freq.	%
0 Do Not Own	303	22.08
1 Own	1,069	77.92
Total	1,372	100.00

ha001_w4_1_2__s1: Household Head and Spouse Own House[2]

	Freq.	%
0 Do Not Own	260	15.38
1 Own	1,430	84.62
Total	1,690	100.00

ha001_w4_1_3__s1: Household Head and Spouse Own House[3]

	Freq.	%
0 Do Not Own	147	14.74
1 Own	850	85.26
Total	997	100.00

ha001_w4_1_4__s1: Household Head and Spouse Own House[4]

	Freq.	%
0 Do Not Own	9	11.11
1 Own	72	88.89
Total	81	100.00

ha001_w4_1_5__s1: Household Head and Spouse Own House[5]

	Freq.	%
0 Do Not Own	1	11.11
1 Own	8	88.89
Total	9	100.00

ha001_w4_1_6__s1: Household Head and Spouse Own House[6]

	Freq.	%
1 Own	2	100.00
Total	2	100.00

ha001_w4_1_1__s2: Children and Children's Spouses Own House[1]

	Freq.	%
0 Do Not Own	1,112	81.05
2 Own	260	18.95
Total	1,372	100.00

ha001_w4_1_2__s2: Children and Children's Spouses Own House[2]

	Freq.	%
0 Do Not Own	1,388	82.13
2 Own	302	17.87
Total	1,690	100.00

ha001_w4_1_3__s2: Children and Children's Spouses Own House[3]

	Freq.	%
0 Do Not Own	850	85.26
2 Own	147	14.74
Total	997	100.00

ha001_w4_1_4__s2: Children and Children's Spouses Own House[4]

	Freq.	%
0 Do Not Own	64	79.01
2 Own	17	20.99
Total	81	100.00

ha001_w4_1_5__s2: Children and Children's Spouses Own House[5]

	Freq.	%
0 Do Not Own	9	100.00
Total	9	100.00

ha001_w4_1_6__s2: Children and Children's Spouses Own House[6]

	Freq.	%
0 Do Not Own	1	50.00
2 Own	1	50.00
Total	2	100.00

ha001_w4_1_1__s3: Siblings Own House[1]

	Freq.	%
0 Do Not Own	1,363	99.34
3 Own	9	0.66
Total	1,372	100.00

ha001_w4_1_2__s3: Siblings Own House[2]

	Freq.	%
0 Do Not Own	1,681	99.47
3 Own	9	0.53
Total	1,690	100.00

ha001_w4_1_3__s3: Siblings Own House[3]

	Freq.	%
0 Do Not Own	993	99.60
3 Own	4	0.40
Total	997	100.00

ha001_w4_1_4__s3: Siblings Own House[4]

	Freq.	%
0 Do Not Own	81	100.00
Total	81	100.00

ha001_w4_1_5__s3: Siblings Own House[5]

	Freq.	%
0 Do Not Own	9	100.00
Total	9	100.00

ha001_w4_1_6__s3: Siblings Own House[6]

	Freq.	%

	Freq.	%
0 Do Not Own	2	100.00
Total	2	100.00

ha001_w4_1_1__s4: Parents and Parents-in-Law Own House[1]

	Freq.	%
0 Do Not Own	1,368	99.71
4 Own	4	0.29
Total	1,372	100.00

ha001_w4_1_2__s4: Parents and Parents-in-Law Own House[2]

	Freq.	%
0 Do Not Own	1,681	99.47
4 Own	9	0.53
Total	1,690	100.00

ha001_w4_1_3__s4: Parents and Parents-in-Law Own House[3]

	Freq.	%
0 Do Not Own	995	99.80
4 Own	2	0.20
Total	997	100.00

ha001_w4_1_4__s4: Parents and Parents-in-Law Own House[4]

	Freq.	%
0 Do Not Own	81	100.00
Total	81	100.00

ha001_w4_1_5__s4: Parents and Parents-in-Law Own House[5]

	Freq.	%
0 Do Not Own	9	100.00
Total	9	100.00

ha001_w4_1_6__s4: Parents and Parents-in-Law Own House[6]

	Freq.	%
0 Do Not Own	2	100.00
Total	2	100.00

ha001_w4_1_1__s5: Grandchildren Own House[1]

	Freq.	%
0 Do Not Own	1,343	97.89
5 Own	29	2.11
Total	1,372	100.00

ha001_w4_1_2__s5: Grandchildren Own House[2]

	Freq.	%
0 Do Not Own	1,665	98.52
5 Own	25	1.48
Total	1,690	100.00

ha001_w4_1_3__s5: Grandchildren Own House[3]

	Freq.	%
0 Do Not Own	986	98.90
5 Own	11	1.10
Total	997	100.00

ha001_w4_1_4__s5: Grandchildren Own House[4]

	Freq.	%
0 Do Not Own	79	97.53
5 Own	2	2.47
Total	81	100.00

ha001_w4_1_5__s5: Grandchildren Own House[5]

	Freq.	%
0 Do Not Own	9	100.00
Total	9	100.00

ha001_w4_1_6__s5: Grandchildren Own House[6]

	Freq.	%
0 Do Not Own	2	100.00
Total	2	100.00

ha001_w4_1_1__s6: Other Relatives Own House[1]

	Freq.	%
0 Do Not Own	1,367	99.64
6 Own	5	0.36
Total	1,372	100.00

ha001_w4_1_2__s6: Other Relatives Own House[2]

	Freq.	%
0 Do Not Own	1,688	99.88
6 Own	2	0.12
Total	1,690	100.00

ha001_w4_1_3__s6: Other Relatives Own House[3]

	Freq.	%
0 Do Not Own	995	99.80
6 Own	2	0.20
Total	997	100.00

ha001_w4_1_4__s6: Other Relatives Own House[4]

	Freq.	%
0 Do Not Own	81	100.00
Total	81	100.00

ha001_w4_1_5__s6: Other Relatives Own House[5]

	Freq.	%
0 Do Not Own	9	100.00
Total	9	100.00

ha001_w4_1_6__s6: Other Relatives Own House[6]

	Freq.	%
0 Do Not Own	2	100.00
Total	2	100.00

ha001_w4_1_1__s7: Friends Own House[1]

	Freq.	%
0 Do Not Own	1,371	99.93
7 Own	1	0.07
Total	1,372	100.00

ha001_w4_1_2__s7: Friends Own House[2]

	Freq.	%
0 Do Not Own	1,689	99.94
7 Own	1	0.06
Total	1,690	100.00

ha001_w4_1_3__s7: Friends Own House[3]

	Freq.	%
0 Do Not Own	997	100.00
Total	997	100.00

ha001_w4_1_4__s7: Friends Own House[4]

	Freq.	%
0 Do Not Own	81	100.00

Total	81	100.00

ha001_w4_1_5__s7: Friends Own House[5]

	Freq.	%
0 Do Not Own	9	100.00
Total	9	100.00

ha001_w4_1_6__s7: Friends Own House[6]

	Freq.	%
0 Do Not Own	2	100.00
Total	2	100.00

ha001_w4_1_1__s8: Others Own House[1]

	Freq.	%
0 Do Not Own	1,249	91.03
8 Own	123	8.97
Total	1,372	100.00

ha001_w4_1_2__s8: Others Own House[2]

	Freq.	%
0 Do Not Own	1,602	94.79
8 Own	88	5.21
Total	1,690	100.00

ha001_w4_1_3__s8: Others Own House[3]

	Freq.	%
0 Do Not Own	956	95.89
8 Own	41	4.11
Total	997	100.00

ha001_w4_1_4__s8: Others Own House[4]

	Freq.	%
0 Do Not Own	78	96.30
8 Own	3	3.70
Total	81	100.00

ha001_w4_1_5__s8: Others Own House[5]

	Freq.	%
0 Do Not Own	8	88.89
8 Own	1	11.11
Total	9	100.00

ha001_w4_1_6__s8: Others Own House[6]

	Freq.	%
0 Do Not Own	2	100.00
Total	2	100.00

ha001_w4_1_1__s9: Own House[1] But do Not Have Ownership Certificate

	Freq.	%
0 Do Not Own	1,355	98.76
9 Own	17	1.24
Total	1,372	100.00

ha001_w4_1_2__s9: Own House[2] But do Not Have Ownership Certificate

	Freq.	%
0 Do Not Own	1,669	98.76
9 Own	21	1.24
Total	1,690	100.00

ha001_w4_1_3__s9: Own House[3] But do Not Have Ownership Certificate

	Freq.	%
0 Do Not Own	982	98.50
9 Own	15	1.50
Total	997	100.00

ha001_w4_1_4__s9: Own House[4] But do Not Have Ownership Certificate

	Freq.	%
0 Do Not Own	81	100.00
Total	81	100.00

ha001_w4_1_5__s9: Own House[5] But do Not Have Ownership Certificate

	Freq.	%
0 Do Not Own	9	100.00
Total	9	100.00

ha001_w4_1_6__s9: Own House[6] But do Not Have Ownership Certificate

	Freq.	%
0 Do Not Own	2	100.00
Total	2	100.00

ha001_w4_1_1__s997: Do Not Know

	Freq.	%

0 Do Not Select 997	1,346	98.10
997 Do Not Know	26	1.90
Total	1,372	100.00

ha001_w4_1_2__s997: Do Not Know

	Freq.	%
0 Do Not Select 997	1,667	98.64
997 Do Not Know	23	1.36
Total	1,690	100.00

ha001_w4_1_3__s997: Do Not Know

	Freq.	%
0 Do Not Select 997	982	98.50
997 Do Not Know	15	1.50
Total	997	100.00

ha001_w4_1_4__s997: Do Not Know

	Freq.	%
0 Do Not Select 997	81	100.00
Total	81	100.00

ha001_w4_1_5__s997: Do Not Know

	Freq.	%
0 Do Not Select 997	9	100.00
Total	9	100.00

ha001_w4_1_6__s997: Do Not Know

	Freq.	%
0 Do Not Select 997	2	100.00
Total	2	100.00

ha001_w4_1_1__s999: Refused to Answer House[1] Ownership

	Freq.	%
0 No	1,366	99.56
999 Yes	6	0.44
Total	1,372	100.00

ha001_w4_1_2__s999: Refused to Answer House[2] Ownership

	Freq.	%
0 No	1,680	99.41
999 Yes	10	0.59

	Freq.	%
Total	1,690	100.00

ha001_w4_1_3__s999: Refused to Answer House[3] Ownership

	Freq.	%
0 No	991	99.40
999 Yes	6	0.60
Total	997	100.00

ha001_w4_1_4__s999: Refused to Answer House[4] Ownership

	Freq.	%
0 No	81	100.00
Total	81	100.00

ha001_w4_1_5__s999: Refused to Answer House[5] Ownership

	Freq.	%
0 No	9	100.00
Total	9	100.00

ha001_w4_1_6__s999: Refused to Answer House[6] Ownership

	Freq.	%
0 No	2	100.00
Total	2	100.00

ha001_w4_1_1_1_: Percent Household Head and Spouse Own House[1]

Mean	SD	Min	Max	Obs
89.82	22.39	0.00	100.00	1,077

ha001_w4_1_1_2_: Percent Household Head and Spouse Own House[2]

Mean	SD	Min	Max	Obs
91.51	20.22	0.00	100.00	1,439

ha001_w4_1_1_3_: Percent Household Head and Spouse Own House[3]

Mean	SD	Min	Max	Obs
93.26	18.31	0.00	100.00	854

ha001_w4_1_1_4_: Percent Household Head and Spouse Own House[4]

Mean	SD	Min	Max	Obs
88.22	23.96	10.00	100.00	72

ha001_w4_1_1_5_: Percent Household Head and Spouse Own House[5]

Mean	SD	Min	Max	Obs
100.00	0.00	100.00	100.00	8

ha001_w4_1_1_6_: Percent Household Head and Spouse Own House[6]

Mean	SD	Min	Max	Obs
75.00	35.36	50.00	100.00	2

ha001_w4_1_3_1_: Percent Siblings Own House[1]

Mean	SD	Min	Max	Obs
78.81	22.34	50.00	100.00	9

ha001_w4_1_3_2_: Percent Siblings Own House[2]

Mean	SD	Min	Max	Obs
62.15	27.19	25.00	100.00	11

ha001_w4_1_3_3_: Percent Siblings Own House[3]

Mean	SD	Min	Max	Obs
60.25	31.42	25.00	100.00	4

ha001_w4_1_6_1_: Percent Other Relatives Own House[1]

Mean	SD	Min	Max	Obs
59.80	25.32	33.00	100.00	5

ha001_w4_1_6_2_: Percent Other Relatives Own House[2]

Mean	SD	Min	Max	Obs
87.50	17.68	75.00	100.00	2

ha001_w4_1_6_3_: Percent Other Relatives Own House[3]

Mean	SD	Min	Max	Obs
55.00	63.64	10.00	100.00	2

ha001_w4_1_7_1_: Percent Friends Own House[1]

Mean	SD	Min	Max	Obs
100.00	.	100.00	100.00	1

ha001_w4_1_7_2_: Percent Friends Own House[2]

Mean	SD	Min	Max	Obs
100.00	.	100.00	100.00	1

ha001_w4_1_8_1_: Percent Others Own House[1]

Mean	SD	Min	Max	Obs
82.08	36.91	0.00	100.00	123

ha001_w4_1_8_2_: Percent Others Own House[2]

Mean	SD	Min	Max	Obs
81.59	37.21	0.00	100.00	88

ha001_w4_1_8_3_: Percent Others Own House[3]

Mean	SD	Min	Max	Obs
85.37	35.78	0.00	100.00	41

ha001_w4_1_8_4_: Percent Others Own House[4]

Mean	SD	Min	Max	Obs
100.00	0.00	100.00	100.00	3

ha001_w4_1_8_5_: Percent Others Own House[5]

Mean	SD	Min	Max	Obs
0.00	.	0.00	0.00	1

ha001_w4_2_1__s1: XChildName[1]/'s Spouse Owns House[1]

	Freq.	%
0 Do Not Own	89	34.23
1 Own	171	65.77
Total	260	100.00

ha001_w4_2_2__s1: XChildName[1]/'s Spouse Owns House[2]

	Freq.	%
0 Do Not Own	93	30.79
1 Own	209	69.21
Total	302	100.00

ha001_w4_2_3__s1: XChildName[1]/'s Spouse Owns House[3]

	Freq.	%
0 Do Not Own	50	34.01

	Freq.	%
1 Own	97	65.99
Total	147	100.00

ha001_w4_2_4__s1: XChildName[1]/'s Spouse Owns House[4]

	Freq.	%
0 Do Not Own	7	41.18
1 Own	10	58.82
Total	17	100.00

ha001_w4_2_6__s1: XChildName[1]/'s Spouse Owns House[6]

	Freq.	%
0 Do Not Own	1	100.00
Total	1	100.00

ha001_w4_2_1__s2: XChildName[2]/'s Spouse Owns House[1]

	Freq.	%
0 Do Not Own	173	66.54
2 Own	87	33.46
Total	260	100.00

ha001_w4_2_2__s2: XChildName[2]/'s Spouse Owns House[2]

	Freq.	%
0 Do Not Own	195	64.57
2 Own	107	35.43
Total	302	100.00

ha001_w4_2_3__s2: XChildName[2]/'s Spouse Owns House[3]

	Freq.	%
0 Do Not Own	99	67.35
2 Own	48	32.65
Total	147	100.00

ha001_w4_2_4__s2: XChildName[2]/'s Spouse Owns House[4]

	Freq.	%
0 Do Not Own	12	70.59
2 Own	5	29.41
Total	17	100.00

ha001_w4_2_6__s2: XChildName[2]/'s Spouse Owns House[6]

	Freq.	%
0 Do Not Own	1	100.00
Total	1	100.00

ha001_w4_2_1__s3: XChildName[3]/'s Spouse Owns House[1]

	Freq.	%
0 Do Not Own	222	85.38
3 Own	38	14.62
Total	260	100.00

ha001_w4_2_2__s3: XChildName[3]/'s Spouse Owns House[2]

	Freq.	%
0 Do Not Own	269	89.07
3 Own	33	10.93
Total	302	100.00

ha001_w4_2_3__s3: XChildName[3]/'s Spouse Owns House[3]

	Freq.	%
0 Do Not Own	125	85.03
3 Own	22	14.97
Total	147	100.00

ha001_w4_2_4__s3: XChildName[3]/'s Spouse Owns House[4]

	Freq.	%
0 Do Not Own	15	88.24
3 Own	2	11.76
Total	17	100.00

ha001_w4_2_6__s3: XChildName[3]/'s Spouse Owns House[6]

	Freq.	%
0 Do Not Own	1	100.00
Total	1	100.00

ha001_w4_2_1__s4: XChildName[4]/'s Spouse Owns House[1]

	Freq.	%
0 Do Not Own	247	95.00
4 Own	13	5.00
Total	260	100.00

ha001_w4_2_2__s4: XChildName[4]/'s Spouse Owns House[2]

	Freq.	%
0 Do Not Own	285	94.37
4 Own	17	5.63
Total	302	100.00

ha001_w4_2_3__s4: XChildName[4]/'s Spouse Owns House[3]

	Freq.	%
0 Do Not Own	138	93.88
4 Own	9	6.12
Total	147	100.00

ha001_w4_2_4__s4: XChildName[4]/'s Spouse Owns House[4]

	Freq.	%
0 Do Not Own	17	100.00
Total	17	100.00

ha001_w4_2_6__s4: XChildName[4]/'s Spouse Owns House[6]

	Freq.	%
0 Do Not Own	1	100.00
Total	1	100.00

ha001_w4_2_1__s5: XChildName[5]/'s Spouse Owns House[1]

	Freq.	%
0 Do Not Own	258	99.23
5 Own	2	0.77
Total	260	100.00

ha001_w4_2_2__s5: XChildName[5]/'s Spouse Owns House[2]

	Freq.	%
0 Do Not Own	293	97.02
5 Own	9	2.98
Total	302	100.00

ha001_w4_2_3__s5: XChildName[5]/'s Spouse Owns House[3]

	Freq.	%
0 Do Not Own	144	97.96
5 Own	3	2.04
Total	147	100.00

ha001_w4_2_4__s5: XChildName[5]/'s Spouse Owns House[4]

	Freq.	%
0 Do Not Own	16	94.12
5 Own	1	5.88
Total	17	100.00

ha001_w4_2_6__s5: XChildName[5]/'s Spouse Owns House[6]

	Freq.	%
0 Do Not Own	1	100.00
Total	1	100.00

ha001_w4_2_1__s6: XChildName[6]/'s Spouse Owns House[1]

	Freq.	%
0 Do Not Own	259	99.62
6 Own	1	0.38
Total	260	100.00

ha001_w4_2_2__s6: XChildName[6]/'s Spouse Owns House[2]

	Freq.	%
0 Do Not Own	300	99.34
6 Own	2	0.66
Total	302	100.00

ha001_w4_2_3__s6: XChildName[6]/'s Spouse Owns House[3]

	Freq.	%
0 Do Not Own	146	99.32
6 Own	1	0.68
Total	147	100.00

ha001_w4_2_4__s6: XChildName[6]/'s Spouse Owns House[4]

	Freq.	%
0 Do Not Own	17	100.00
Total	17	100.00

ha001_w4_2_6__s6: XChildName[6]/'s Spouse Owns House[6]

	Freq.	%
0 Do Not Own	1	100.00
Total	1	100.00

ha001_w4_2_1__s7: XChildName[7]/'s Spouse Owns House[1]

	Freq.	%
0 Do Not Own	259	99.62
7 Own	1	0.38
Total	260	100.00

ha001_w4_2_2__s7: XChildName[7]/'s Spouse Owns House[2]

	Freq.	%

	Freq.	%
0 Do Not Own	302	100.00
Total	302	100.00

ha001_w4_2_3__s7: XChildName[7]/'s Spouse Owns House[3]

	Freq.	%
0 Do Not Own	147	100.00
Total	147	100.00

ha001_w4_2_4__s7: XChildName[7]/'s Spouse Owns House[4]

	Freq.	%
0 Do Not Own	17	100.00
Total	17	100.00

ha001_w4_2_6__s7: XChildName[7]/'s Spouse Owns House[6]

	Freq.	%
0 Do Not Own	1	100.00
Total	1	100.00

ha001_w4_2_1__s8: XChildName[8]/'s Spouse Owns House[1]

	Freq.	%
0 Do Not Own	259	99.62
8 Own	1	0.38
Total	260	100.00

ha001_w4_2_2__s8: XChildName[8]/'s Spouse Owns House[2]

	Freq.	%
0 Do Not Own	302	100.00
Total	302	100.00

ha001_w4_2_3__s8: XChildName[8]/'s Spouse Owns House[3]

	Freq.	%
0 Do Not Own	146	99.32
8 Own	1	0.68
Total	147	100.00

ha001_w4_2_4__s8: XChildName[8]/'s Spouse Owns House[4]

	Freq.	%
0 Do Not Own	17	100.00
Total	17	100.00

ha001_w4_2_6__s8: XChildName[8]/'s Spouse Owns House[6]

	Freq.	%
0 Do Not Own	1	100.00
Total	1	100.00

ha001_w4_2_1__s9: XChildName[9]/'s Spouse Owns House[1]

	Freq.	%
0 Do Not Own	259	99.62
9 Own	1	0.38
Total	260	100.00

ha001_w4_2_2__s9: XChildName[9]/'s Spouse Owns House[2]

	Freq.	%
0 Do Not Own	302	100.00
Total	302	100.00

ha001_w4_2_3__s9: XChildName[9]/'s Spouse Owns House[3]

	Freq.	%
0 Do Not Own	147	100.00
Total	147	100.00

ha001_w4_2_4__s9: XChildName[9]/'s Spouse Owns House[4]

	Freq.	%
0 Do Not Own	17	100.00
Total	17	100.00

ha001_w4_2_6__s9: XChildName[9]/'s Spouse Owns House[6]

	Freq.	%
0 Do Not Own	1	100.00
Total	1	100.00

ha001_w4_2_1__s10: XChildName[10]/'s Spouse Owns House[1]

	Freq.	%
0 Do Not Own	260	100.00
Total	260	100.00

ha001_w4_2_2__s10: XChildName[10]/'s Spouse Owns House[2]

	Freq.	%
0 Do Not Own	302	100.00
Total	302	100.00

ha001_w4_2_3__s10: XChildName[10]/'s Spouse Owns House[3]

	Freq.	%
0 Do Not Own	146	99.32
10 Own	1	0.68
Total	147	100.00

ha001_w4_2_4__s10: XChildName[10]/'s Spouse Owns House[4]

	Freq.	%
0 Do Not Own	17	100.00
Total	17	100.00

ha001_w4_2_6__s10: XChildName[10]/'s Spouse Owns House[6]

	Freq.	%
0 Do Not Own	1	100.00
Total	1	100.00

ha001_w4_2_1__s11: XChildName[11]/'s Spouse Owns House[1]

	Freq.	%
0 Do Not Own	260	100.00
Total	260	100.00

ha001_w4_2_2__s11: XChildName[11]/'s Spouse Owns House[2]

	Freq.	%
0 Do Not Own	302	100.00
Total	302	100.00

ha001_w4_2_3__s11: XChildName[11]/'s Spouse Owns House[3]

	Freq.	%
0 Do Not Own	147	100.00
Total	147	100.00

ha001_w4_2_4__s11: XChildName[11]/'s Spouse Owns House[4]

	Freq.	%
0 Do Not Own	17	100.00
Total	17	100.00

ha001_w4_2_6__s11: XChildName[11]/'s Spouse Owns House[6]

	Freq.	%
0 Do Not Own	1	100.00
Total	1	100.00

ha001_w4_2_1__s12: XChildName[12]/'s Spouse Owns House[1]

	Freq.	%
0 Do Not Own	260	100.00
Total	260	100.00

ha001_w4_2_2__s12: XChildName[12]/'s Spouse Owns House[2]

	Freq.	%
0 Do Not Own	302	100.00
Total	302	100.00

ha001_w4_2_3__s12: XChildName[12]/'s Spouse Owns House[3]

	Freq.	%
0 Do Not Own	147	100.00
Total	147	100.00

ha001_w4_2_4__s12: XChildName[12]/'s Spouse Owns House[4]

	Freq.	%
0 Do Not Own	17	100.00
Total	17	100.00

ha001_w4_2_6__s12: XChildName[12]/'s Spouse Owns House[6]

	Freq.	%
0 Do Not Own	1	100.00
Total	1	100.00

ha001_w4_2_1__s13: XChildName[13]/'s Spouse Owns House[1]

	Freq.	%
0 Do Not Own	260	100.00
Total	260	100.00

ha001_w4_2_2__s13: XChildName[13]/'s Spouse Owns House[2]

	Freq.	%
0 Do Not Own	302	100.00
Total	302	100.00

ha001_w4_2_3__s13: XChildName[13]/'s Spouse Owns House[3]

	Freq.	%
0 Do Not Own	147	100.00
Total	147	100.00

ha001_w4_2_4__s13: XChildName[13]/'s Spouse Owns House[4]

	Freq.	%
0 Do Not Own	17	100.00
Total	17	100.00

ha001_w4_2_6__s13: XChildName[13]/'s Spouse Owns House[6]

	Freq.	%
0 Do Not Own	1	100.00
Total	1	100.00

ha001_w4_2_1__s14: XChildName[14]/'s Spouse Owns House[1]

	Freq.	%
0 Do Not Own	260	100.00
Total	260	100.00

ha001_w4_2_2__s14: XChildName[14]/'s Spouse Owns House[2]

	Freq.	%
0 Do Not Own	302	100.00
Total	302	100.00

ha001_w4_2_3__s14: XChildName[14]/'s Spouse Owns House[3]

	Freq.	%
0 Do Not Own	147	100.00
Total	147	100.00

ha001_w4_2_4__s14: XChildName[14]/'s Spouse Owns House[4]

	Freq.	%
0 Do Not Own	17	100.00
Total	17	100.00

ha001_w4_2_6__s14: XChildName[14]/'s Spouse Owns House[6]

	Freq.	%
0 Do Not Own	1	100.00
Total	1	100.00

ha001_w4_2_1__s15: XChildName[15]/'s Spouse Owns House[1]

	Freq.	%
0 Do Not Own	260	100.00
Total	260	100.00

ha001_w4_2_2__s15: XChildName[15]/'s Spouse Owns House[2]

	Freq.	%
0 Do Not Own	302	100.00
Total	302	100.00

ha001_w4_2_3__s15: XChildName[15]/'s Spouse Owns House[3]

	Freq.	%
0 Do Not Own	147	100.00
Total	147	100.00

ha001_w4_2_4__s15: XChildName[15]/'s Spouse Owns House[4]

	Freq.	%
0 Do Not Own	17	100.00
Total	17	100.00

ha001_w4_2_6__s15: XChildName[15]/'s Spouse Owns House[6]

	Freq.	%
0 Do Not Own	1	100.00
Total	1	100.00

ha001_w4_2_1__s16: XChildName[16]/'s Spouse Owns House[1]

	Freq.	%
0 Do Not Own	260	100.00
Total	260	100.00

ha001_w4_2_2__s16: XChildName[16]/'s Spouse Owns House[2]

	Freq.	%
0 Do Not Own	302	100.00
Total	302	100.00

ha001_w4_2_3__s16: XChildName[16]/'s Spouse Owns House[3]

	Freq.	%
0 Do Not Own	147	100.00
Total	147	100.00

ha001_w4_2_4__s16: XChildName[16]/'s Spouse Owns House[4]

	Freq.	%
0 Do Not Own	17	100.00
Total	17	100.00

ha001_w4_2_6__s16: XChildName[16]/'s Spouse Owns House[6]

	Freq.	%
0 Do Not Own	1	100.00
Total	1	100.00

ha001_w4_2_1__s99: None of the Above Owns House[1]

	Freq.	%
0 No	252	96.92
99 Yes	8	3.08
Total	260	100.00

ha001_w4_2_2__s99: None of the Above Owns House[2]

	Freq.	%
0 No	293	97.02
99 Yes	9	2.98
Total	302	100.00

ha001_w4_2_3__s99: None of the Above Owns House[3]

	Freq.	%
0 No	145	98.64
99 Yes	2	1.36
Total	147	100.00

ha001_w4_2_4__s99: None of the Above Owns House[4]

	Freq.	%
0 No	17	100.00
Total	17	100.00

ha001_w4_2_6__s99: None of the Above Owns House[6]

	Freq.	%
99 Yes	1	100.00
Total	1	100.00

ha001_w4_3_1__1_: Percent XChildName[1]/'s Spouse Owns House[1]

Mean	SD	Min	Max	Obs
58.69	32.06	0.25	100.00	171

ha001_w4_3_2__1_: Percent XChildName[1]/'s Spouse Owns House[2]

Mean	SD	Min	Max	Obs
51.06	31.57	0.00	100.00	209

ha001_w4_3_3__1_: Percent XChildName[1]/'s Spouse Owns House[3]

Mean	SD	Min	Max	Obs
58.63	31.31	12.50	100.00	97

ha001_w4_3_4__1_: Percent XChildName[1]/'s Spouse Owns House[4]

Mean	SD	Min	Max	Obs
57.40	32.01	16.00	100.00	10

ha001_w4_3_1__2_: Percent XChildName[2]/'s Spouse Owns House[1]

Mean	SD	Min	Max	Obs
52.11	30.15	0.25	100.00	87

ha001_w4_3_2__2_: Percent XChildName[2]/'s Spouse Owns House[2]

Mean	SD	Min	Max	Obs
48.57	32.06	10.00	100.00	107

ha001_w4_3_3__2_: Percent XChildName[2]/'s Spouse Owns House[3]

Mean	SD	Min	Max	Obs
56.00	31.72	12.50	100.00	48

ha001_w4_3_4__2_: Percent XChildName[2]/'s Spouse Owns House[4]

Mean	SD	Min	Max	Obs
67.20	33.54	33.00	100.00	5

ha001_w4_3_1__3_: Percent XChildName[3]/'s Spouse Owns House[1]

Mean	SD	Min	Max	Obs
60.85	31.79	18.75	100.00	38

ha001_w4_3_2__3_: Percent XChildName[3]/'s Spouse Owns House[2]

Mean	SD	Min	Max	Obs
44.88	33.91	0.00	100.00	33

ha001_w4_3_3__3_: Percent XChildName[3]/'s Spouse Owns House[3]

Mean	SD	Min	Max	Obs
54.70	33.43	12.50	100.00	22

ha001_w4_3_4__3_: Percent XChildName[3]/'s Spouse Owns House[4]

Mean	SD	Min	Max	Obs
70.00	28.28	50.00	90.00	2

ha001_w4_3_1__4_: Percent XChildName[4]/'s Spouse Owns House[1]

Mean	SD	Min	Max	Obs
63.08	36.45	18.75	100.00	13

ha001_w4_3_2__4_: Percent XChildName[4]/'s Spouse Owns House[2]

Mean	SD	Min	Max	Obs
43.41	32.27	0.00	100.00	17

ha001_w4_3_3__4_: Percent XChildName[4]/'s Spouse Owns House[3]

Mean	SD	Min	Max	Obs
49.50	31.51	12.50	100.00	9

ha001_w4_3_1__5_: Percent XChildName[5]/'s Spouse Owns House[1]

Mean	SD	Min	Max	Obs
50.00	0.00	50.00	50.00	2

ha001_w4_3_2__5_: Percent XChildName[5]/'s Spouse Owns House[2]

Mean	SD	Min	Max	Obs
55.59	35.07	17.00	100.00	9

ha001_w4_3_3__5_: Percent XChildName[5]/'s Spouse Owns House[3]

Mean	SD	Min	Max	Obs
21.67	2.89	20.00	25.00	3

ha001_w4_3_4__5_: Percent XChildName[5]/'s Spouse Owns House[4]

Mean	SD	Min	Max	Obs
100.00	.	100.00	100.00	1

ha001_w4_3_1__6_: Percent XChildName[6]/'s Spouse Owns House[1]

Mean	SD	Min	Max	Obs
25.00	.	25.00	25.00	1

ha001_w4_3_2__6_: Percent XChildName[6]/'s Spouse Owns House[2]

Mean	SD	Min	Max	Obs
33.50	23.33	17.00	50.00	2

ha001_w4_3_3__6_: Percent XChildName[6]/'s Spouse Owns House[3]

Mean	SD	Min	Max	Obs
20.00	.	20.00	20.00	1

ha001_w4_3_1__7_: Percent XChildName[7]/'s Spouse Owns House[1]

Mean	SD	Min	Max	Obs
100.00	.	100.00	100.00	1

ha001_w4_3_1__8_: Percent XChildName[8]/'s Spouse Owns House[1]

Mean	SD	Min	Max	Obs
100.00	.	100.00	100.00	1

ha001_w4_3_3__8_: Percent XChildName[8]/'s Spouse Owns House[3]

Mean	SD	Min	Max	Obs
40.00	.	40.00	40.00	1

ha001_w4_3_1__9_: Percent XChildName[9]/'s Spouse Owns House[1]

Mean	SD	Min	Max	Obs
25.00	.	25.00	25.00	1

ha001_w4_3_3__10_: Percent XChildName[10]/'s Spouse Owns House[3]

Mean	SD	Min	Max	Obs
40.00	.	40.00	40.00	1

ha001_w4_4_1__s1: XConParName[1] Owns House[1]

	Freq.	%
0 Do Not Own	3	75.00
1 Own	1	25.00
Total	4	100.00

ha001_w4_4_2__s1: XConParName[1] Owns House[2]

	Freq.	%
0 Do Not Own	6	66.67
1 Own	3	33.33
Total	9	100.00

ha001_w4_4_3__s1: XConParName[1] Owns House[3]

	Freq.	%
0 Do Not Own	2	100.00
Total	2	100.00

ha001_w4_4_1__s2: XConParName[2] Owns House[1]

	Freq.	%
0 Do Not Own	2	50.00
2 Own	2	50.00
Total	4	100.00

ha001_w4_4_2__s2: XConParName[2] Owns House[2]

	Freq.	%
0 Do Not Own	5	55.56
2 Own	4	44.44
Total	9	100.00

ha001_w4_4_3__s2: XConParName[2] Owns House[3]

	Freq.	%
0 Do Not Own	1	50.00
2 Own	1	50.00
Total	2	100.00

ha001_w4_4_1__s3: XConParName[3] Owns House[1]

	Freq.	%
0 Do Not Own	4	100.00
Total	4	100.00

ha001_w4_4_2__s3: XConParName[3] Owns House[2]

	Freq.	%
0 Do Not Own	9	100.00
Total	9	100.00

ha001_w4_4_3__s3: XConParName[3] Owns House[3]

	Freq.	%
0 Do Not Own	2	100.00
Total	2	100.00

ha001_w4_4_1__s4: XConParName[4] Owns House[1]

	Freq.	%

	Freq.	%
0 Do Not Own	4	100.00
Total	4	100.00

ha001_w4_4_2__s4: XConParName[4] Owns House[2]

	Freq.	%
0 Do Not Own	9	100.00
Total	9	100.00

ha001_w4_4_3__s4: XConParName[4] Owns House[3]

	Freq.	%
0 Do Not Own	2	100.00
Total	2	100.00

ha001_w4_4_1__s5: XConParName[5] Owns House[1]

	Freq.	%
0 Do Not Own	2	50.00
5 Own	2	50.00
Total	4	100.00

ha001_w4_4_2__s5: XConParName[5] Owns House[2]

	Freq.	%
0 Do Not Own	7	77.78
5 Own	2	22.22
Total	9	100.00

ha001_w4_4_3__s5: XConParName[5] Owns House[3]

	Freq.	%
0 Do Not Own	2	100.00
Total	2	100.00

ha001_w4_4_1__s6: XConParName[6] Owns House[1]

	Freq.	%
0 Do Not Own	2	50.00
6 Own	2	50.00
Total	4	100.00

ha001_w4_4_2__s6: XConParName[6] Owns House[2]

	Freq.	%
0 Do Not Own	7	77.78
6 Own	2	22.22
Total	9	100.00

ha001_w4_4_3__s6: XConParName[6] Owns House[3]

	Freq.	%
0 Do Not Own	2	100.00
Total	2	100.00

ha001_w4_4_1__s7: XConParName[7] Owns House[1]

	Freq.	%
0 Do Not Own	4	100.00
Total	4	100.00

ha001_w4_4_2__s7: XConParName[7] Owns House[2]

	Freq.	%
0 Do Not Own	9	100.00
Total	9	100.00

ha001_w4_4_3__s7: XConParName[7] Owns House[3]

	Freq.	%
0 Do Not Own	2	100.00
Total	2	100.00

ha001_w4_4_1__s8: XConParName[8] Owns House[1]

	Freq.	%
0 Do Not Own	4	100.00
Total	4	100.00

ha001_w4_4_2__s8: XConParName[8] Owns House[2]

	Freq.	%
0 Do Not Own	9	100.00
Total	9	100.00

ha001_w4_4_3__s8: XConParName[8] Owns House[3]

	Freq.	%
0 Do Not Own	2	100.00
Total	2	100.00

ha001_w4_4_1__s99: None of the Above Owns House[1]

	Freq.	%
0 No	4	100.00
Total	4	100.00

ha001_w4_4_2__s99: None of the Above Owns House[2]

	Freq.	%
0 No	7	77.78
99 Yes	2	22.22
Total	9	100.00

ha001_w4_4_3__s99: None of the Above Owns House[3]

	Freq.	%
0 No	1	50.00
99 Yes	1	50.00
Total	2	100.00

ha001_w4_5_1__1_: Percent XConParName[1] Owns House[1]

Mean	SD	Min	Max	Obs
20.00	.	20.00	20.00	1

ha001_w4_5_2__1_: Percent XConParName[1] Owns House[2]

Mean	SD	Min	Max	Obs
21.10	11.36	8.30	30.00	3

ha001_w4_5_1__2_: Percent XConParName[2] Owns House[1]

Mean	SD	Min	Max	Obs
22.50	3.54	20.00	25.00	2

ha001_w4_5_2__2_: Percent XConParName[2] Owns House[2]

Mean	SD	Min	Max	Obs
18.33	7.09	8.30	25.00	4

ha001_w4_5_3__2_: Percent XConParName[2] Owns House[3]

Mean	SD	Min	Max	Obs
20.00	.	20.00	20.00	1

ha001_w4_5_1__5_: Percent XConParName[5] Owns House[1]

Mean	SD	Min	Max	Obs
37.50	17.68	25.00	50.00	2

ha001_w4_5_2__5_: Percent XConParName[5] Owns House[2]

Mean	SD	Min	Max	Obs

20.00	0.00	20.00	20.00	2

ha001_w4_5_1__6_: Percent XConParName[6] Owns House[1]

Mean	SD	Min	Max	Obs
37.50	17.68	25.00	50.00	2

ha001_w4_5_2__6_: Percent XConParName[6] Owns House[2]

Mean	SD	Min	Max	Obs
20.00	0.00	20.00	20.00	2

ha001_w4_6_1__s1: XChildName[1]'s Child(Grandchild) Owns House[1]

	Freq.	%
0 Do Not Own	11	37.93
1 Own	18	62.07
Total	29	100.00

ha001_w4_6_2__s1: XChildName[1]'s Child(Grandchild) Owns House[2]

	Freq.	%
0 Do Not Own	3	12.00
1 Own	22	88.00
Total	25	100.00

ha001_w4_6_3__s1: XChildName[1]'s Child(Grandchild) Owns House[3]

	Freq.	%
0 Do Not Own	2	18.18
1 Own	9	81.82
Total	11	100.00

ha001_w4_6_4__s1: XChildName[1]'s Child(Grandchild) Owns House[4]

	Freq.	%
1 Own	2	100.00
Total	2	100.00

ha001_w4_6_1__s2: XChildName[2]'s Child(Grandchild) Owns House[1]

	Freq.	%
0 Do Not Own	24	82.76
2 Own	5	17.24
Total	29	100.00

ha001_w4_6_2__s2: XChildName[2]'s Child(Grandchild) Owns House[2]

	Freq.	%
0 Do Not Own	22	88.00
2 Own	3	12.00
Total	25	100.00

ha001_w4_6_3__s2: XChildName[2]'s Child(Grandchild) Owns House[3]

	Freq.	%
0 Do Not Own	11	100.00
Total	11	100.00

ha001_w4_6_4__s2: XChildName[2]'s Child(Grandchild) Owns House[4]

	Freq.	%
0 Do Not Own	2	100.00
Total	2	100.00

ha001_w4_6_1__s3: XChildName[3]'s Child(Grandchild) Owns House[1]

	Freq.	%
0 Do Not Own	26	89.66
3 Own	3	10.34
Total	29	100.00

ha001_w4_6_2__s3: XChildName[3]'s Child(Grandchild) Owns House[2]

	Freq.	%
0 Do Not Own	25	100.00
Total	25	100.00

ha001_w4_6_3__s3: XChildName[3]'s Child(Grandchild) Owns House[3]

	Freq.	%
0 Do Not Own	9	81.82
3 Own	2	18.18
Total	11	100.00

ha001_w4_6_4__s3: XChildName[3]'s Child(Grandchild) Owns House[4]

	Freq.	%
0 Do Not Own	2	100.00
Total	2	100.00

ha001_w4_6_1__s4: XChildName[4]'s Child(Grandchild) Owns House[1]

	Freq.	%
0 Do Not Own	26	89.66

	Freq.	%
4 Own	3	10.34
Total	29	100.00

ha001_w4_6_2__s4: XChildName[4]'s Child(Grandchild) Owns House[2]

	Freq.	%
0 Do Not Own	25	100.00
Total	25	100.00

ha001_w4_6_3__s4: XChildName[4]'s Child(Grandchild) Owns House[3]

	Freq.	%
0 Do Not Own	11	100.00
Total	11	100.00

ha001_w4_6_4__s4: XChildName[4]'s Child(Grandchild) Owns House[4]

	Freq.	%
0 Do Not Own	2	100.00
Total	2	100.00

ha001_w4_6_1__s5: XChildName[5]'s Child(Grandchild) Owns House[1]

	Freq.	%
0 Do Not Own	26	89.66
5 Own	3	10.34
Total	29	100.00

ha001_w4_6_2__s5: XChildName[5]'s Child(Grandchild) Owns House[2]

	Freq.	%
0 Do Not Own	25	100.00
Total	25	100.00

ha001_w4_6_3__s5: XChildName[5]'s Child(Grandchild) Owns House[3]

	Freq.	%
0 Do Not Own	11	100.00
Total	11	100.00

ha001_w4_6_4__s5: XChildName[5]'s Child(Grandchild) Owns House[4]

	Freq.	%
0 Do Not Own	2	100.00
Total	2	100.00

ha001_w4_6_1__s6: XChildName[6]'s Child(Grandchild) Owns House[1]

	Freq.	%
0 Do Not Own	29	100.00
Total	29	100.00

ha001_w4_6_2__s6: XChildName[6]'s Child(Grandchild) Owns House[2]

	Freq.	%
0 Do Not Own	25	100.00
Total	25	100.00

ha001_w4_6_3__s6: XChildName[6]'s Child(Grandchild) Owns House[3]

	Freq.	%
0 Do Not Own	11	100.00
Total	11	100.00

ha001_w4_6_4__s6: XChildName[6]'s Child(Grandchild) Owns House[4]

	Freq.	%
0 Do Not Own	2	100.00
Total	2	100.00

ha001_w4_6_1__s7: XChildName[7]'s Child(Grandchild) Owns House[1]

	Freq.	%
0 Do Not Own	29	100.00
Total	29	100.00

ha001_w4_6_2__s7: XChildName[7]'s Child(Grandchild) Owns House[2]

	Freq.	%
0 Do Not Own	25	100.00
Total	25	100.00

ha001_w4_6_3__s7: XChildName[7]'s Child(Grandchild) Owns House[3]

	Freq.	%
0 Do Not Own	11	100.00
Total	11	100.00

ha001_w4_6_4__s7: XChildName[7]'s Child(Grandchild) Owns House[4]

	Freq.	%
0 Do Not Own	2	100.00
Total	2	100.00

ha001_w4_6_1__s8: XChildName[8]'s Child(Grandchild) Owns House[1]

	Freq.	%
0 Do Not Own	29	100.00
Total	29	100.00

ha001_w4_6_2__s8: XChildName[8]'s Child(Grandchild) Owns House[2]

	Freq.	%
0 Do Not Own	25	100.00
Total	25	100.00

ha001_w4_6_3__s8: XChildName[8]'s Child(Grandchild) Owns House[3]

	Freq.	%
0 Do Not Own	11	100.00
Total	11	100.00

ha001_w4_6_4__s8: XChildName[8]'s Child(Grandchild) Owns House[4]

	Freq.	%
0 Do Not Own	2	100.00
Total	2	100.00

ha001_w4_6_1__s9: XChildName[9]'s Child(Grandchild) Owns House[1]

	Freq.	%
0 Do Not Own	29	100.00
Total	29	100.00

ha001_w4_6_2__s9: XChildName[9]'s Child(Grandchild) Owns House[2]

	Freq.	%
0 Do Not Own	25	100.00
Total	25	100.00

ha001_w4_6_3__s9: XChildName[9]'s Child(Grandchild) Owns House[3]

	Freq.	%
0 Do Not Own	11	100.00
Total	11	100.00

ha001_w4_6_4__s9: XChildName[9]'s Child(Grandchild) Owns House[4]

	Freq.	%
0 Do Not Own	2	100.00
Total	2	100.00

ha001_w4_6_1__s10: XChildName[10]'s Child(Grandchild) Owns House[1]

	Freq.	%
0 Do Not Own	29	100.00
Total	29	100.00

ha001_w4_6_2__s10: XChildName[10]'s Child(Grandchild) Owns House[2]

	Freq.	%
0 Do Not Own	25	100.00
Total	25	100.00

ha001_w4_6_3__s10: XChildName[10]'s Child(Grandchild) Owns House[3]

	Freq.	%
0 Do Not Own	11	100.00
Total	11	100.00

ha001_w4_6_4__s10: XChildName[10]'s Child(Grandchild) Owns House[4]

	Freq.	%
0 Do Not Own	2	100.00
Total	2	100.00

ha001_w4_6_1__s11: XChildName[11]'s Child(Grandchild) Owns House[1]

	Freq.	%
0 Do Not Own	29	100.00
Total	29	100.00

ha001_w4_6_2__s11: XChildName[11]'s Child(Grandchild) Owns House[2]

	Freq.	%
0 Do Not Own	25	100.00
Total	25	100.00

ha001_w4_6_3__s11: XChildName[11]'s Child(Grandchild) Owns House[3]

	Freq.	%
0 Do Not Own	11	100.00
Total	11	100.00

ha001_w4_6_4__s11: XChildName[11]'s Child(Grandchild) Owns House[4]

	Freq.	%
0 Do Not Own	2	100.00
Total	2	100.00

ha001_w4_6_1__s12: XChildName[12]'s Child(Grandchild) Owns House[1]

	Freq.	%
0 Do Not Own	29	100.00
Total	29	100.00

ha001_w4_6_2__s12: XChildName[12]'s Child(Grandchild) Owns House[2]

	Freq.	%
0 Do Not Own	25	100.00
Total	25	100.00

ha001_w4_6_3__s12: XChildName[12]'s Child(Grandchild) Owns House[3]

	Freq.	%
0 Do Not Own	11	100.00
Total	11	100.00

ha001_w4_6_4__s12: XChildName[12]'s Child(Grandchild) Owns House[4]

	Freq.	%
0 Do Not Own	2	100.00
Total	2	100.00

ha001_w4_6_1__s13: XChildName[13]'s Child(Grandchild) Owns House[1]

	Freq.	%
0 Do Not Own	29	100.00
Total	29	100.00

ha001_w4_6_2__s13: XChildName[13]'s Child(Grandchild) Owns House[2]

	Freq.	%
0 Do Not Own	25	100.00
Total	25	100.00

ha001_w4_6_3__s13: XChildName[13]'s Child(Grandchild) Owns House[3]

	Freq.	%
0 Do Not Own	11	100.00
Total	11	100.00

ha001_w4_6_4__s13: XChildName[13]'s Child(Grandchild) Owns House[4]

	Freq.	%
0 Do Not Own	2	100.00
Total	2	100.00

ha001_w4_6_1__s14: XChildName[14]'s Child(Grandchild) Owns House[1]

	Freq.	%
0 Do Not Own	29	100.00
Total	29	100.00

ha001_w4_6_2__s14: XChildName[14]'s Child(Grandchild) Owns House[2]

	Freq.	%
0 Do Not Own	25	100.00
Total	25	100.00

ha001_w4_6_3__s14: XChildName[14]'s Child(Grandchild) Owns House[3]

	Freq.	%
0 Do Not Own	11	100.00
Total	11	100.00

ha001_w4_6_4__s14: XChildName[14]'s Child(Grandchild) Owns House[4]

	Freq.	%
0 Do Not Own	2	100.00
Total	2	100.00

ha001_w4_6_1__s15: XChildName[15]'s Child(Grandchild) Owns House[1]

	Freq.	%
0 Do Not Own	29	100.00
Total	29	100.00

ha001_w4_6_2__s15: XChildName[15]'s Child(Grandchild) Owns House[2]

	Freq.	%
0 Do Not Own	25	100.00
Total	25	100.00

ha001_w4_6_3__s15: XChildName[15]'s Child(Grandchild) Owns House[3]

	Freq.	%
0 Do Not Own	11	100.00
Total	11	100.00

ha001_w4_6_4__s15: XChildName[15]'s Child(Grandchild) Owns House[4]

	Freq.	%
0 Do Not Own	2	100.00
Total	2	100.00

ha001_w4_6_1__s16: XChildName[16]'s Child(Grandchild) Owns House[1]

	Freq.	%
0 Do Not Own	29	100.00
Total	29	100.00

ha001_w4_6_2__s16: XChildName[16]'s Child(Grandchild) Owns House[2]

	Freq.	%
0 Do Not Own	25	100.00
Total	25	100.00

ha001_w4_6_3__s16: XChildName[16]'s Child(Grandchild) Owns House[3]

	Freq.	%
0 Do Not Own	11	100.00
Total	11	100.00

ha001_w4_6_4__s16: XChildName[16]'s Child(Grandchild) Owns House[4]

	Freq.	%
0 Do Not Own	2	100.00
Total	2	100.00

ha001_w4_7_1__1_: Percent XChildName[1]'s Child(Grandchild) Owns House[1]

Mean	SD	Min	Max	Obs
31.76	19.59	13.00	100.00	18

ha001_w4_7_2__1_: Percent XChildName[1]'s Child(Grandchild) Owns House[2]

Mean	SD	Min	Max	Obs
26.60	10.21	10.00	50.00	22

ha001_w4_7_3__1_: Percent XChildName[1]'s Child(Grandchild) Owns House[3]

Mean	SD	Min	Max	Obs
32.74	26.43	10.00	100.00	9

ha001_w4_7_4__1_: Percent XChildName[1]'s Child(Grandchild) Owns House[4]

Mean	SD	Min	Max	Obs
20.50	6.36	16.00	25.00	2

ha001_w4_7_1__2_: Percent XChildName[2]'s Child(Grandchild) Owns House[1]

Mean	SD	Min	Max	Obs
28.26	14.23	13.00	50.00	5

ha001_w4_7_2__2_: Percent XChildName[2]'s Child(Grandchild) Owns House[2]

Mean	SD	Min	Max	Obs
26.67	11.55	20.00	40.00	3

ha001_w4_7_1__3_: Percent XChildName[3]'s Child(Grandchild) Owns House[1]

Mean	SD	Min	Max	Obs
46.00	47.15	13.00	100.00	3

ha001_w4_7_3__3_: Percent XChildName[3]'s Child(Grandchild) Owns House[3]

Mean	SD	Min	Max	Obs
70.00	42.43	40.00	100.00	2

ha001_w4_7_1__4_: Percent XChildName[4]'s Child(Grandchild) Owns House[1]

Mean	SD	Min	Max	Obs
80.00	34.64	40.00	100.00	3

ha001_w4_7_1__5_: Percent XChildName[5]'s Child(Grandchild) Owns House[1]

Mean	SD	Min	Max	Obs
75.00	43.30	25.00	100.00	3

ha004_w4_1_: Total Value or Unit Value for House[1]

	Freq.	%
1 Total Value	2,541	96.36
2 Unit Value	96	3.64
Total	2,637	100.00

ha004_w4_2_: Total Value or Unit Value for House[2]

	Freq.	%
1 Total Value	2,475	96.23
2 Unit Value	97	3.77
Total	2,572	100.00

ha004_w4_3_: Total Value or Unit Value for House[3]

	Freq.	%
1 Total Value	1,446	96.14
2 Unit Value	58	3.86
Total	1,504	100.00

ha004_w4_4_: Total Value or Unit Value for House[4]

	Freq.	%
1 Total Value	110	94.02
2 Unit Value	7	5.98
Total	117	100.00

ha004_w4_5_: Total Value or Unit Value for House[5]

	Freq.	%
1 Total Value	19	95.00
2 Unit Value	1	5.00
Total	20	100.00

ha004_w4_6_: Total Value or Unit Value for House[6]

	Freq.	%
1 Total Value	3	100.00
Total	3	100.00

ha004_w4_7_: Total Value or Unit Value for House[7]

	Freq.	%
1 Total Value	1	100.00
Total	1	100.00

ha004_w4_1_1_: Total Value for House[1]

Mean	SD	Min	Max	Obs
13,818.28	235,288.81	0.00	10000000.00	2,147

ha004_w4_1_2_: Total Value for House[2]

Mean	SD	Min	Max	Obs
4,653.04	41,873.86	0.00	840,000.00	2,076

ha004_w4_1_3_: Total Value for House[3]

Mean	SD	Min	Max	Obs
5,029.09	36,090.31	0.00	500,000.00	1,230

ha004_w4_1_4_: Total Value for House[4]

Mean	SD	Min	Max	Obs
7,013.33	38,506.20	0.10	300,000.00	94

ha004_w4_1_5_: Total Value for House[5]

Mean	SD	Min	Max	Obs
37.37	51.35	0.10	200.00	15

ha004_w4_1_6_: Total Value for House[6]

Mean	SD	Min	Max	Obs
23.50	12.02	15.00	32.00	2

ha004_w4_1_7_: Total Value for House[7]

Mean	SD	Min	Max	Obs
27.00	.	27.00	27.00	1

ha004_w4_2_1_: Unit Value for House[1]

Mean	SD	Min	Max	Obs
1,460.86	2,792.76	0.00	13,000.00	77

ha004_w4_2_2_: Unit Value for House[2]

Mean	SD	Min	Max	Obs
1,419.35	2,963.29	0.05	15,000.00	83

ha004_w4_2_3_: Unit Value for House[3]

Mean	SD	Min	Max	Obs
4,041.15	19,329.73	0.00	130,000.00	45

ha004_w4_2_4_: Unit Value for House[4]

Mean	SD	Min	Max	Obs
3,785.21	7,476.82	0.40	15,000.00	4

ha004_w4_2_5_: Unit Value for House[5]

Mean	SD	Min	Max	Obs
0.40	.	0.40	0.40	1

ha004_w4_1__min: Min Bracket of ha004_w4_1_

Mean	SD	Min	Max	Obs
5,112.00	4,664.93	1,000.00	15,000.00	125

ha004_w4_1__max: Max Bracket of ha004_w4_1_

Mean	SD	Min	Max	Obs
2,782.01	3,253.88	1,000.00	15,000.00	289

ha004_w4_2__min: Min Bracket of ha004_w4_2_

Mean	SD	Min	Max	Obs
4,317.31	4,311.34	1,000.00	15,000.00	104

ha004_w4_2__max: Max Bracket of ha004_w4_2_

Mean	SD	Min	Max	Obs
2,617.33	3,080.70	1,000.00	15,000.00	277

ha004_w4_3__min: Min Bracket of ha004_w4_3_

Mean	SD	Min	Max	Obs
3,579.71	3,647.75	1,000.00	15,000.00	69

ha004_w4_3__max: Max Bracket of ha004_w4_3_

Mean	SD	Min	Max	Obs
2,525.00	2,627.74	1,000.00	15,000.00	160

ha004_w4_4__min: Min Bracket of ha004_w4_4_

Mean	SD	Min	Max	Obs
6,500.00	5,972.16	1,000.00	15,000.00	4

ha004_w4_4__max: Max Bracket of ha004_w4_4_

Mean	SD	Min	Max	Obs
2,888.89	2,571.21	1,000.00	8,000.00	9

ha004_w4_5__min: Min Bracket of ha004_w4_5_

Mean	SD	Min	Max	Obs
3,000.00	.	3,000.00	3,000.00	1

ha004_w4_5__max: Max Bracket of ha004_w4_5_

Mean	SD	Min	Max	Obs
6,333.33	7,571.88	1,000.00	15,000.00	3

ha004_w4_6__min: Min Bracket of ha004_w4_6_

No Observations

ha004_w4_6__max: Max Bracket of ha004_w4_6_

No Observations

ha005_w4_1_: House[1] Rent Out

	Freq.	%
1 Yes	122	4.63
2 No	2,510	95.18
999 Refused to Answer	5	0.19
Total	2,637	100.00

ha005_w4_2_: House[2] Rent Out

	Freq.	%
1 Yes	88	3.42
2 No	2,477	96.31
999 Refused to Answer	7	0.27
Total	2,572	100.00

ha005_w4_3_: House[3] Rent Out

	Freq.	%
1 Yes	53	3.52
2 No	1,445	96.08
999 Refused to Answer	6	0.40
Total	1,504	100.00

ha005_w4_4_: House[4] Rent Out

	Freq.	%
1 Yes	11	9.40
2 No	106	90.60
Total	117	100.00

ha005_w4_5_: House[5] Rent Out

	Freq.	%
1 Yes	2	10.00
2 No	18	90.00
Total	20	100.00

ha005_w4_6_: House[6] Rent Out

	Freq.	%
2 No	3	100.00

Total	3	100.00

ha005_w4_7_: House[7] Rent Out

	Freq.	%
1 Yes	1	100.00
Total	1	100.00

ha006_w4_1_: House[1]'s Rent

Mean	SD	Min	Max	Obs
1,151.38	1,614.59	-1.00	10,000.00	122

ha006_w4_2_: House[2]'s Rent

Mean	SD	Min	Max	Obs
1,017.19	1,249.22	-1.00	8,000.00	88

ha006_w4_3_: House[3]'s Rent

Mean	SD	Min	Max	Obs
1,198.96	1,812.59	-1.00	11,666.00	53

ha006_w4_4_: House[4]'s Rent

Mean	SD	Min	Max	Obs
1,100.82	1,529.98	-1.00	5,000.00	11

ha006_w4_5_: House[5]'s Rent

Mean	SD	Min	Max	Obs
275.00	247.49	100.00	450.00	2

ha006_w4_7_: House[7]'s Rent

Mean	SD	Min	Max	Obs
250.00	.	250.00	250.00	1

ha006_w4_1__min: Min Bracket of ha006_w4_1_

Mean	SD	Min	Max	Obs
750.00	353.55	500.00	1,000.00	2

ha006_w4_1__max: Max Bracket of ha006_w4_1_

Mean	SD	Min	Max	Obs

666.67	288.68	500.00	1,000.00	3

ha006_w4_2__min: Min Bracket of ha006_w4_2_

Mean	SD	Min	Max	Obs
500.00	.	500.00	500.00	1

ha006_w4_2__max: Max Bracket of ha006_w4_2_

Mean	SD	Min	Max	Obs
500.00	0.00	500.00	500.00	2

ha006_w4_3__min: Min Bracket of ha006_w4_3_

No Observations

ha006_w4_3__max: Max Bracket of ha006_w4_3_

Mean	SD	Min	Max	Obs
500.00	.	500.00	500.00	1

ha006_w4_4__min: Min Bracket of ha006_w4_4_

No Observations

ha006_w4_4__max: Max Bracket of ha006_w4_4_

No Observations

ha007_w4_1_: House[1]'s Market Rent

Mean	SD	Min	Max	Obs
746.56	1,666.70	0.00	20,000.00	1,798

ha007_w4_2_: House[2]'s Market Rent

Mean	SD	Min	Max	Obs
643.68	1,201.73	0.00	20,000.00	1,730

ha007_w4_3_: House[3]'s Market Rent

Mean	SD	Min	Max	Obs
671.68	1,271.68	0.00	20,000.00	987

ha007_w4_4_: House[4]'s Market Rent

Mean	SD	Min	Max	Obs
854.25	1,413.79	0.00	8,000.00	83

ha007_w4_5_: House[5]'s Market Rent

Mean	SD	Min	Max	Obs
512.50	507.12	0.00	2,000.00	16

ha007_w4_6_: House[6]'s Market Rent

Mean	SD	Min	Max	Obs
300.00	282.84	100.00	500.00	2

ha007_w4_7_: House[7]'s Market Rent

Mean	SD	Min	Max	Obs
250.00	.	250.00	250.00	1

ha007_w4_1__min: Min Bracket of ha007_w4_1_

Mean	SD	Min	Max	Obs
1,333.33	1,592.87	500.00	8,000.00	189

ha007_w4_1__max: Max Bracket of ha007_w4_1_

Mean	SD	Min	Max	Obs
790.89	973.54	500.00	8,000.00	801

ha007_w4_2__min: Min Bracket of ha007_w4_2_

Mean	SD	Min	Max	Obs
1,358.64	1,462.12	500.00	8,000.00	191

ha007_w4_2__max: Max Bracket of ha007_w4_2_

Mean	SD	Min	Max	Obs
830.80	1,046.61	500.00	8,000.00	789

ha007_w4_3__min: Min Bracket of ha007_w4_3_

Mean	SD	Min	Max	Obs
1,113.21	1,082.95	500.00	8,000.00	106

ha007_w4_3__max: Max Bracket of ha007_w4_3_

Mean	SD	Min	Max	Obs
789.30	1,033.80	500.00	8,000.00	458

ha007_w4_4__min: Min Bracket of ha007_w4_4_

Mean	SD	Min	Max	Obs
1,312.50	752.97	500.00	2,000.00	8

ha007_w4_4__max: Max Bracket of ha007_w4_4_

Mean	SD	Min	Max	Obs
982.14	976.41	500.00	5,000.00	28

ha010_w4_1_: Household Head and Spouse do Not Own House[1]

	Freq.	%
1 Sold	36	17.73
2 Demolished	53	26.11
3 Gift	76	37.44
4 Other	38	18.72
Total	203	100.00

ha010_w4_2_: Household Head and Spouse do Not Own House[2]

	Freq.	%
1 Sold	16	10.00
2 Demolished	40	25.00
3 Gift	75	46.88
4 Other	29	18.13
Total	160	100.00

ha010_w4_3_: Household Head and Spouse do Not Own House[3]

	Freq.	%
1 Sold	13	14.77
2 Demolished	19	21.59
3 Gift	42	47.73
4 Other	14	15.91
Total	88	100.00

ha010_w4_4_: Household Head and Spouse do Not Own House[4]

	Freq.	%
2 Demolished	2	28.57
3 Gift	5	71.43
Total	7	100.00

ha010_w4_5_: Household Head and Spouse do Not Own House[5]

	Freq.	%
2 Demolished	1	100.00
Total	1	100.00

ha011_w4_1_1_: Year for House[1] Sold

Mean	SD	Min	Max	Obs
2,013.94	5.19	1,991.00	2,018.00	36

ha011_w4_1_2_: Year for House[2] Sold

Mean	SD	Min	Max	Obs
2,015.06	3.70	2,005.00	2,018.00	16

ha011_w4_1_3_: Year for House[3] Sold

Mean	SD	Min	Max	Obs
2,016.23	1.09	2,015.00	2,018.00	13

ha011_w4_2_1_: Month for House[1] Sold

Mean	SD	Min	Max	Obs
6.00	3.55	1.00	12.00	29

ha011_w4_2_2_: Month for House[2] Sold

Mean	SD	Min	Max	Obs
7.47	3.52	3.00	12.00	15

ha011_w4_2_3_: Month for House[3] Sold

Mean	SD	Min	Max	Obs
6.75	3.67	1.00	12.00	12

ha012_w4_1_: Net Income for House[1] Sold

Mean	SD	Min	Max	Obs
9,948.99	39,355.44	0.00	230,000.00	36

ha012_w4_2_: Net Income for House[2] Sold

Mean	SD	Min	Max	Obs
900.16	3,493.42	0.80	14,000.00	16

ha012_w4_3_: Net Income for House[3] Sold

Mean	SD	Min	Max	Obs
13,908.35	49,904.39	2.00	180,000.00	13

ha013_w4_1_: Get Compensation for House[1] Demolished

	Freq.	%
1 Yes	24	45.28
2 No	29	54.72
Total	53	100.00

ha013_w4_2_: Get Compensation for House[2] Demolished

	Freq.	%
1 Yes	25	62.50
2 No	15	37.50
Total	40	100.00

ha013_w4_3_: Get Compensation for House[3] Demolished

	Freq.	%
1 Yes	10	52.63
2 No	9	47.37
Total	19	100.00

ha013_w4_4_: Get Compensation for House[4] Demolished

	Freq.	%
1 Yes	1	50.00
2 No	1	50.00
Total	2	100.00

ha013_w4_5_: Get Compensation for House[5] Demolished

	Freq.	%
1 Yes	1	100.00
Total	1	100.00

ha014_w4_1_1_: Year Get Compensation for House[1]

Mean	SD	Min	Max	Obs
2,015.33	1.55	2,011.00	2,018.00	24

ha014_w4_1_2_: Year Get Compensation for House[2]

Mean	SD	Min	Max	Obs
2,015.56	1.56	2,011.00	2,018.00	25

ha014_w4_1_3_: Year Get Compensation for House[3]

Mean	SD	Min	Max	Obs
2,015.60	0.84	2,015.00	2,017.00	10

ha014_w4_1_4_: Year Get Compensation for House[4]

Mean	SD	Min	Max	Obs
2,016.00	.	2,016.00	2,016.00	1

ha014_w4_1_5_: Year Get Compensation for House[5]

Mean	SD	Min	Max	Obs
2,014.00	.	2,014.00	2,014.00	1

ha014_w4_2_1_: Month Get Compensation for House[1]

Mean	SD	Min	Max	Obs
8.15	3.36	2.00	12.00	20

ha014_w4_2_2_: Month Get Compensation for House[2]

Mean	SD	Min	Max	Obs
8.64	3.14	2.00	12.00	22

ha014_w4_2_3_: Month Get Compensation for House[3]

Mean	SD	Min	Max	Obs
8.00	3.74	1.00	12.00	8

ha014_w4_2_4_: Month Get Compensation for House[4]

Mean	SD	Min	Max	Obs
6.00	.	6.00	6.00	1

ha014_w4_2_5_: Month Get Compensation for House[5]

No Observations

ha015_w4_1_: Total Compensation for House[1]

Mean	SD	Min	Max	Obs
10,928.02	50,971.03	1.20	250,000.00	24

ha015_w4_2_: Total Compensation for House[2]

Mean	SD	Min	Max	Obs
6,407.26	19,458.03	0.30	71,500.00	24

ha015_w4_3_: Total Compensation for House[3]

Mean	SD	Min	Max	Obs
62.57	52.52	0.20	130.00	10

ha015_w4_4_: Total Compensation for House[4]

Mean	SD	Min	Max	Obs
5.50	.	5.50	5.50	1

ha015_w4_5_: Total Compensation for House[5]

Mean	SD	Min	Max	Obs
120.00	.	120.00	120.00	1

ha015_w4_2__min: Min Bracket of ha015_w4_2_

Mean	SD	Min	Max	Obs
500,000.00	.	500,000.00	500,000.00	1

ha015_w4_2__max: Max Bracket of ha015_w4_2_

Mean	SD	Min	Max	Obs
500,000.00	.	500,000.00	500,000.00	1

ha016_w4_1__s1: Children/Children's Spouse House[1] Gifted To

	Freq.	%
0 No	5	6.58
1 Yes	71	93.42
Total	76	100.00

ha016_w4_2__s1: Children/Children's Spouse House[2] Gifted To

	Freq.	%
0 No	3	4.00
1 Yes	72	96.00
Total	75	100.00

ha016_w4_3__s1: Children/Children's Spouse House[3] Gifted To

	Freq.	%

	Freq.	%
0 No	3	7.14
1 Yes	39	92.86
Total	42	100.00

ha016_w4_4__s1: Children/Children's Spouse House[4] Gifted To

	Freq.	%
1 Yes	5	100.00
Total	5	100.00

ha016_w4_1__s2: Siblings House[1] Gifted To

	Freq.	%
0 No	74	97.37
2 Yes	2	2.63
Total	76	100.00

ha016_w4_2__s2: Siblings House[2] Gifted To

	Freq.	%
0 No	75	100.00
Total	75	100.00

ha016_w4_3__s2: Siblings House[3] Gifted To

	Freq.	%
0 No	41	97.62
2 Yes	1	2.38
Total	42	100.00

ha016_w4_4__s2: Siblings House[4] Gifted To

	Freq.	%
0 No	5	100.00
Total	5	100.00

ha016_w4_1__s3: Parents/Parents-in-Law House[1] Gifted To

	Freq.	%
0 No	76	100.00
Total	76	100.00

ha016_w4_2__s3: Parents/Parents-in-Law House[2] Gifted To

	Freq.	%
0 No	75	100.00
Total	75	100.00

ha016_w4_3__s3: Parents/Parents-in-Law House[3] Gifted To

	Freq.	%
0 No	42	100.00
Total	42	100.00

ha016_w4_4__s3: Parents/Parents-in-Law House[4] Gifted To

	Freq.	%
0 No	5	100.00
Total	5	100.00

ha016_w4_1__s4: Grandchildren House[1] Gifted To

	Freq.	%
0 No	73	96.05
4 Yes	3	3.95
Total	76	100.00

ha016_w4_2__s4: Grandchildren House[2] Gifted To

	Freq.	%
0 No	74	98.67
4 Yes	1	1.33
Total	75	100.00

ha016_w4_3__s4: Grandchildren House[3] Gifted To

	Freq.	%
0 No	40	95.24
4 Yes	2	4.76
Total	42	100.00

ha016_w4_4__s4: Grandchildren House[4] Gifted To

	Freq.	%
0 No	5	100.00
Total	5	100.00

ha016_w4_1__s5: Other Relatives House[1] Gifted To

	Freq.	%
0 No	76	100.00
Total	76	100.00

ha016_w4_2__s5: Other Relatives House[2] Gifted To

	Freq.	%

	Freq.	%
0 No	74	98.67
5 Yes	1	1.33
Total	75	100.00

ha016_w4_3__s5: Other Relatives House[3] Gifted To

	Freq.	%
0 No	42	100.00
Total	42	100.00

ha016_w4_4__s5: Other Relatives House[4] Gifted To

	Freq.	%
0 No	5	100.00
Total	5	100.00

ha016_w4_1__s6: Friends House[1] Gifted To

	Freq.	%
0 No	76	100.00
Total	76	100.00

ha016_w4_2__s6: Friends House[2] Gifted To

	Freq.	%
0 No	75	100.00
Total	75	100.00

ha016_w4_3__s6: Friends House[3] Gifted To

	Freq.	%
0 No	42	100.00
Total	42	100.00

ha016_w4_4__s6: Friends House[4] Gifted To

	Freq.	%
0 No	5	100.00
Total	5	100.00

ha016_w4_1__s7: Others House[1] Gifted To

	Freq.	%
0 No	75	98.68
7 Yes	1	1.32
Total	76	100.00

ha016_w4_2__s7: Others House[2] Gifted To

	Freq.	%
0 No	73	97.33
7 Yes	2	2.67
Total	75	100.00

ha016_w4_3__s7: Others House[3] Gifted To

	Freq.	%
0 No	41	97.62
7 Yes	1	2.38
Total	42	100.00

ha016_w4_4__s7: Others House[4] Gifted To

	Freq.	%
0 No	5	100.00
Total	5	100.00

ha016_w4_2_1__s1: XChildName[1]/'s Spouse House[1] Gifted To

	Freq.	%
0 No	34	47.89
1 Yes	37	52.11
Total	71	100.00

ha016_w4_2_2__s1: XChildName[1]/'s Spouse House[2] Gifted To

	Freq.	%
0 No	29	40.28
1 Yes	43	59.72
Total	72	100.00

ha016_w4_2_3__s1: XChildName[1]/'s Spouse House[3] Gifted To

	Freq.	%
0 No	17	43.59
1 Yes	22	56.41
Total	39	100.00

ha016_w4_2_4__s1: XChildName[1]/'s Spouse House[4] Gifted To

	Freq.	%
0 No	2	40.00
1 Yes	3	60.00
Total	5	100.00

ha016_w4_2_1__s2: XChildName[2]/'s Spouse House[1] Gifted To

	Freq.	%
0 No	49	69.01
2 Yes	22	30.99
Total	71	100.00

ha016_w4_2_2__s2: XChildName[2]/'s Spouse House[2] Gifted To

	Freq.	%
0 No	47	65.28
2 Yes	25	34.72
Total	72	100.00

ha016_w4_2_3__s2: XChildName[2]/'s Spouse House[3] Gifted To

	Freq.	%
0 No	27	69.23
2 Yes	12	30.77
Total	39	100.00

ha016_w4_2_4__s2: XChildName[2]/'s Spouse House[4] Gifted To

	Freq.	%
0 No	4	80.00
2 Yes	1	20.00
Total	5	100.00

ha016_w4_2_1__s3: XChildName[3]/'s Spouse House[1] Gifted To

	Freq.	%
0 No	57	80.28
3 Yes	14	19.72
Total	71	100.00

ha016_w4_2_2__s3: XChildName[3]/'s Spouse House[2] Gifted To

	Freq.	%
0 No	66	91.67
3 Yes	6	8.33
Total	72	100.00

ha016_w4_2_3__s3: XChildName[3]/'s Spouse House[3] Gifted To

	Freq.	%
0 No	32	82.05
3 Yes	7	17.95
Total	39	100.00

ha016_w4_2_4__s3: XChildName[3]/'s Spouse House[4] Gifted To

	Freq.	%
0 No	5	100.00
Total	5	100.00

ha016_w4_2_1__s4: XChildName[4]/'s Spouse House[1] Gifted To

	Freq.	%
0 No	64	90.14
4 Yes	7	9.86
Total	71	100.00

ha016_w4_2_2__s4: XChildName[4]/'s Spouse House[2] Gifted To

	Freq.	%
0 No	68	94.44
4 Yes	4	5.56
Total	72	100.00

ha016_w4_2_3__s4: XChildName[4]/'s Spouse House[3] Gifted To

	Freq.	%
0 No	39	100.00
Total	39	100.00

ha016_w4_2_4__s4: XChildName[4]/'s Spouse House[4] Gifted To

	Freq.	%
0 No	5	100.00
Total	5	100.00

ha016_w4_2_1__s5: XChildName[5]/'s Spouse House[1] Gifted To

	Freq.	%
0 No	69	97.18
5 Yes	2	2.82
Total	71	100.00

ha016_w4_2_2__s5: XChildName[5]/'s Spouse House[2] Gifted To

	Freq.	%
0 No	72	100.00
Total	72	100.00

ha016_w4_2_3__s5: XChildName[5]/'s Spouse House[3] Gifted To

	Freq.	%
0 No	39	100.00

	Freq.	%
Total	39	100.00

ha016_w4_2_4__s5: XChildName[5]/'s Spouse House[4] Gifted To

	Freq.	%
0 No	4	80.00
5 Yes	1	20.00
Total	5	100.00

ha016_w4_2_1__s6: XChildName[6]/'s Spouse House[1] Gifted To

	Freq.	%
0 No	71	100.00
Total	71	100.00

ha016_w4_2_2__s6: XChildName[6]/'s Spouse House[2] Gifted To

	Freq.	%
0 No	72	100.00
Total	72	100.00

ha016_w4_2_3__s6: XChildName[6]/'s Spouse House[3] Gifted To

	Freq.	%
0 No	39	100.00
Total	39	100.00

ha016_w4_2_4__s6: XChildName[6]/'s Spouse House[4] Gifted To

	Freq.	%
0 No	5	100.00
Total	5	100.00

ha016_w4_2_1__s7: XChildName[7]/'s Spouse House[1] Gifted To

	Freq.	%
0 No	71	100.00
Total	71	100.00

ha016_w4_2_2__s7: XChildName[7]/'s Spouse House[2] Gifted To

	Freq.	%
0 No	72	100.00
Total	72	100.00

ha016_w4_2_3__s7: XChildName[7]/'s Spouse House[3] Gifted To

	Freq.	%
0 No	39	100.00
Total	39	100.00

ha016_w4_2_4__s7: XChildName[7]/'s Spouse House[4] Gifted To

	Freq.	%
0 No	5	100.00
Total	5	100.00

ha016_w4_2_1__s8: XChildName[8]/'s Spouse House[1] Gifted To

	Freq.	%
0 No	70	98.59
8 Yes	1	1.41
Total	71	100.00

ha016_w4_2_2__s8: XChildName[8]/'s Spouse House[2] Gifted To

	Freq.	%
0 No	72	100.00
Total	72	100.00

ha016_w4_2_3__s8: XChildName[8]/'s Spouse House[3] Gifted To

	Freq.	%
0 No	39	100.00
Total	39	100.00

ha016_w4_2_4__s8: XChildName[8]/'s Spouse House[4] Gifted To

	Freq.	%
0 No	5	100.00
Total	5	100.00

ha016_w4_2_1__s9: XChildName[9]/'s Spouse House[1] Gifted To

	Freq.	%
0 No	71	100.00
Total	71	100.00

ha016_w4_2_2__s9: XChildName[9]/'s Spouse House[2] Gifted To

	Freq.	%
0 No	72	100.00
Total	72	100.00

ha016_w4_2_3__s9: XChildName[9]/'s Spouse House[3] Gifted To

	Freq.	%
0 No	39	100.00
Total	39	100.00

ha016_w4_2_4__s9: XChildName[9]/'s Spouse House[4] Gifted To

	Freq.	%
0 No	5	100.00
Total	5	100.00

ha016_w4_2_1__s10: XChildName[10]/'s Spouse House[1] Gifted To

	Freq.	%
0 No	71	100.00
Total	71	100.00

ha016_w4_2_2__s10: XChildName[10]/'s Spouse House[2] Gifted To

	Freq.	%
0 No	72	100.00
Total	72	100.00

ha016_w4_2_3__s10: XChildName[10]/'s Spouse House[3] Gifted To

	Freq.	%
0 No	39	100.00
Total	39	100.00

ha016_w4_2_4__s10: XChildName[10]/'s Spouse House[4] Gifted To

	Freq.	%
0 No	5	100.00
Total	5	100.00

ha016_w4_2_1__s11: XChildName[11]/'s Spouse House[1] Gifted To

	Freq.	%
0 No	71	100.00
Total	71	100.00

ha016_w4_2_2__s11: XChildName[11]/'s Spouse House[2] Gifted To

	Freq.	%
0 No	72	100.00
Total	72	100.00

ha016_w4_2_3__s11: XChildName[11]/'s Spouse House[3] Gifted To

	Freq.	%
0 No	39	100.00
Total	39	100.00

ha016_w4_2_4__s11: XChildName[11]/'s Spouse House[4] Gifted To

	Freq.	%
0 No	5	100.00
Total	5	100.00

ha016_w4_2_1__s12: XChildName[12]/'s Spouse House[1] Gifted To

	Freq.	%
0 No	71	100.00
Total	71	100.00

ha016_w4_2_2__s12: XChildName[12]/'s Spouse House[2] Gifted To

	Freq.	%
0 No	72	100.00
Total	72	100.00

ha016_w4_2_3__s12: XChildName[12]/'s Spouse House[3] Gifted To

	Freq.	%
0 No	39	100.00
Total	39	100.00

ha016_w4_2_4__s12: XChildName[12]/'s Spouse House[4] Gifted To

	Freq.	%
0 No	5	100.00
Total	5	100.00

ha016_w4_2_1__s13: XChildName[13]/'s Spouse House[1] Gifted To

	Freq.	%
0 No	71	100.00
Total	71	100.00

ha016_w4_2_2__s13: XChildName[13]/'s Spouse House[2] Gifted To

	Freq.	%
0 No	72	100.00
Total	72	100.00

ha016_w4_2_3__s13: XChildName[13]/'s Spouse House[3] Gifted To

	Freq.	%
0 No	39	100.00
Total	39	100.00

ha016_w4_2_4__s13: XChildName[13]/'s Spouse House[4] Gifted To

	Freq.	%
0 No	5	100.00
Total	5	100.00

ha016_w4_2_1__s14: XChildName[14]/'s Spouse House[1] Gifted To

	Freq.	%
0 No	71	100.00
Total	71	100.00

ha016_w4_2_2__s14: XChildName[14]/'s Spouse House[2] Gifted To

	Freq.	%
0 No	72	100.00
Total	72	100.00

ha016_w4_2_3__s14: XChildName[14]/'s Spouse House[3] Gifted To

	Freq.	%
0 No	39	100.00
Total	39	100.00

ha016_w4_2_4__s14: XChildName[14]/'s Spouse House[4] Gifted To

	Freq.	%
0 No	5	100.00
Total	5	100.00

ha016_w4_2_1__s15: XChildName[15]/'s Spouse House[1] Gifted To

	Freq.	%
0 No	71	100.00
Total	71	100.00

ha016_w4_2_2__s15: XChildName[15]/'s Spouse House[2] Gifted To

	Freq.	%
0 No	72	100.00
Total	72	100.00

ha016_w4_2_3__s15: XChildName[15]/'s Spouse House[3] Gifted To

	Freq.	%
0 No	39	100.00
Total	39	100.00

ha016_w4_2_4__s15: XChildName[15]/'s Spouse House[4] Gifted To

	Freq.	%
0 No	5	100.00
Total	5	100.00

ha016_w4_2_1__s16: XChildName[16]/'s Spouse House[1] Gifted To

	Freq.	%
0 No	71	100.00
Total	71	100.00

ha016_w4_2_2__s16: XChildName[16]/'s Spouse House[2] Gifted To

	Freq.	%
0 No	72	100.00
Total	72	100.00

ha016_w4_2_3__s16: XChildName[16]/'s Spouse House[3] Gifted To

	Freq.	%
0 No	39	100.00
Total	39	100.00

ha016_w4_2_4__s16: XChildName[16]/'s Spouse House[4] Gifted To

	Freq.	%
0 No	5	100.00
Total	5	100.00

ha016_w4_2_1__s99: None of the Above House[1] Gifted To

	Freq.	%
0 No	70	98.59
99 Yes	1	1.41
Total	71	100.00

ha016_w4_2_2__s99: None of the Above House[2] Gifted To

	Freq.	%
0 No	70	97.22
99 Yes	2	2.78

	Freq.	%
Total	72	100.00

ha016_w4_2_3__s99: None of the Above House[3] Gifted To

	Freq.	%
0 No	37	94.87
99 Yes	2	5.13
Total	39	100.00

ha016_w4_2_4__s99: None of the Above House[4] Gifted To

	Freq.	%
0 No	5	100.00
Total	5	100.00

ha016_w4_4_1__s1: XChildName[1]'s Child(Grandchild) House[1] Gifted To

	Freq.	%
0 No	3	100.00
Total	3	100.00

ha016_w4_4_2__s1: XChildName[1]'s Child(Grandchild) House[2] Gifted To

	Freq.	%
1 Yes	1	100.00
Total	1	100.00

ha016_w4_4_3__s1: XChildName[1]'s Child(Grandchild) House[3] Gifted To

	Freq.	%
0 No	1	50.00
1 Yes	1	50.00
Total	2	100.00

ha016_w4_4_1__s2: XChildName[2]'s Child(Grandchild) House[1] Gifted To

	Freq.	%
0 No	3	100.00
Total	3	100.00

ha016_w4_4_2__s2: XChildName[2]'s Child(Grandchild) House[2] Gifted To

	Freq.	%
0 No	1	100.00
Total	1	100.00

ha016_w4_4_3__s2: XChildName[2]'s Child(Grandchild) House[3] Gifted To

	Freq.	%
0 No	2	100.00
Total	2	100.00

ha016_w4_4_1__s3: XChildName[3]'s Child(Grandchild) House[1] Gifted To

	Freq.	%
0 No	2	66.67
3 Yes	1	33.33
Total	3	100.00

ha016_w4_4_2__s3: XChildName[3]'s Child(Grandchild) House[2] Gifted To

	Freq.	%
0 No	1	100.00
Total	1	100.00

ha016_w4_4_3__s3: XChildName[3]'s Child(Grandchild) House[3] Gifted To

	Freq.	%
0 No	1	50.00
3 Yes	1	50.00
Total	2	100.00

ha016_w4_4_1__s4: XChildName[4]'s Child(Grandchild) House[1] Gifted To

	Freq.	%
0 No	1	33.33
4 Yes	2	66.67
Total	3	100.00

ha016_w4_4_2__s4: XChildName[4]'s Child(Grandchild) House[2] Gifted To

	Freq.	%
0 No	1	100.00
Total	1	100.00

ha016_w4_4_3__s4: XChildName[4]'s Child(Grandchild) House[3] Gifted To

	Freq.	%
0 No	2	100.00
Total	2	100.00

ha016_w4_4_1__s5: XChildName[5]'s Child(Grandchild) House[1] Gifted To

	Freq.	%
0 No	3	100.00

Total	3	100.00

ha016_w4_4_2__s5: XChildName[5]'s Child(Grandchild) House[2] Gifted To

	Freq.	%
0 No	1	100.00
Total	1	100.00

ha016_w4_4_3__s5: XChildName[5]'s Child(Grandchild) House[3] Gifted To

	Freq.	%
0 No	2	100.00
Total	2	100.00

ha016_w4_4_1__s6: XChildName[6]'s Child(Grandchild) House[1] Gifted To

	Freq.	%
0 No	3	100.00
Total	3	100.00

ha016_w4_4_2__s6: XChildName[6]'s Child(Grandchild) House[2] Gifted To

	Freq.	%
0 No	1	100.00
Total	1	100.00

ha016_w4_4_3__s6: XChildName[6]'s Child(Grandchild) House[3] Gifted To

	Freq.	%
0 No	2	100.00
Total	2	100.00

ha016_w4_4_1__s7: XChildName[7]'s Child(Grandchild) House[1] Gifted To

	Freq.	%
0 No	3	100.00
Total	3	100.00

ha016_w4_4_2__s7: XChildName[7]'s Child(Grandchild) House[2] Gifted To

	Freq.	%
0 No	1	100.00
Total	1	100.00

ha016_w4_4_3__s7: XChildName[7]'s Child(Grandchild) House[3] Gifted To

	Freq.	%

	Freq.	%
0 No	2	100.00
Total	2	100.00

ha016_w4_4_1__s8: XChildName[8]'s Child(Grandchild) House[1] Gifted To

	Freq.	%
0 No	3	100.00
Total	3	100.00

ha016_w4_4_2__s8: XChildName[8]'s Child(Grandchild) House[2] Gifted To

	Freq.	%
0 No	1	100.00
Total	1	100.00

ha016_w4_4_3__s8: XChildName[8]'s Child(Grandchild) House[3] Gifted To

	Freq.	%
0 No	2	100.00
Total	2	100.00

ha016_w4_4_1__s9: XChildName[9]'s Child(Grandchild) House[1] Gifted To

	Freq.	%
0 No	3	100.00
Total	3	100.00

ha016_w4_4_2__s9: XChildName[9]'s Child(Grandchild) House[2] Gifted To

	Freq.	%
0 No	1	100.00
Total	1	100.00

ha016_w4_4_3__s9: XChildName[9]'s Child(Grandchild) House[3] Gifted To

	Freq.	%
0 No	2	100.00
Total	2	100.00

ha016_w4_4_1__s10: XChildName[10]'s Child(Grandchild) House[1] Gifted To

	Freq.	%
0 No	3	100.00
Total	3	100.00

ha016_w4_4_2__s10: XChildName[10]'s Child(Grandchild) House[2] Gifted To

	Freq.	%
0 No	1	100.00
Total	1	100.00

ha016_w4_4_3__s10: XChildName[10]'s Child(Grandchild) House[3] Gifted To

	Freq.	%
0 No	2	100.00
Total	2	100.00

ha016_w4_4_1__s11: XChildName[11]'s Child(Grandchild) House[1] Gifted To

	Freq.	%
0 No	3	100.00
Total	3	100.00

ha016_w4_4_2__s11: XChildName[11]'s Child(Grandchild) House[2] Gifted To

	Freq.	%
0 No	1	100.00
Total	1	100.00

ha016_w4_4_3__s11: XChildName[11]'s Child(Grandchild) House[3] Gifted To

	Freq.	%
0 No	2	100.00
Total	2	100.00

ha016_w4_4_1__s12: XChildName[12]'s Child(Grandchild) House[1] Gifted To

	Freq.	%
0 No	3	100.00
Total	3	100.00

ha016_w4_4_2__s12: XChildName[12]'s Child(Grandchild) House[2] Gifted To

	Freq.	%
0 No	1	100.00
Total	1	100.00

ha016_w4_4_3__s12: XChildName[12]'s Child(Grandchild) House[3] Gifted To

	Freq.	%
0 No	2	100.00
Total	2	100.00

ha016_w4_4_1__s13: XChildName[13]'s Child(Grandchild) House[1] Gifted To

	Freq.	%
0 No	3	100.00
Total	3	100.00

ha016_w4_4_2__s13: XChildName[13]'s Child(Grandchild) House[2] Gifted To

	Freq.	%
0 No	1	100.00
Total	1	100.00

ha016_w4_4_3__s13: XChildName[13]'s Child(Grandchild) House[3] Gifted To

	Freq.	%
0 No	2	100.00
Total	2	100.00

ha016_w4_4_1__s14: XChildName[14]'s Child(Grandchild) House[1] Gifted To

	Freq.	%
0 No	3	100.00
Total	3	100.00

ha016_w4_4_2__s14: XChildName[14]'s Child(Grandchild) House[2] Gifted To

	Freq.	%
0 No	1	100.00
Total	1	100.00

ha016_w4_4_3__s14: XChildName[14]'s Child(Grandchild) House[3] Gifted To

	Freq.	%
0 No	2	100.00
Total	2	100.00

ha016_w4_4_1__s15: XChildName[15]'s Child(Grandchild) House[1] Gifted To

	Freq.	%
0 No	3	100.00
Total	3	100.00

ha016_w4_4_2__s15: XChildName[15]'s Child(Grandchild) House[2] Gifted To

	Freq.	%
0 No	1	100.00
Total	1	100.00

ha016_w4_4_3__s15: XChildName[15]'s Child(Grandchild) House[3] Gifted To

	Freq.	%
0 No	2	100.00
Total	2	100.00

ha016_w4_4_1__s16: XChildName[16]'s Child(Grandchild) House[1] Gifted To

	Freq.	%
0 No	3	100.00
Total	3	100.00

ha016_w4_4_2__s16: XChildName[16]'s Child(Grandchild) House[2] Gifted To

	Freq.	%
0 No	1	100.00
Total	1	100.00

ha016_w4_4_3__s16: XChildName[16]'s Child(Grandchild) House[3] Gifted To

	Freq.	%
0 No	2	100.00
Total	2	100.00

ha017_w4_1_1_: Year for House[1] Gifted To

Mean	SD	Min	Max	Obs
2,013.15	7.67	1,979.00	2,018.00	74

ha017_w4_1_2_: Year for House[2] Gifted To

Mean	SD	Min	Max	Obs
2,012.28	7.77	1,988.00	2,018.00	74

ha017_w4_1_3_: Year for House[3] Gifted To

Mean	SD	Min	Max	Obs
2,014.98	5.04	1,988.00	2,018.00	41

ha017_w4_1_4_: Year for House[4] Gifted To

Mean	SD	Min	Max	Obs
2,014.20	5.76	2,004.00	2,018.00	5

ha017_w4_2_1_: Month for House[1] Gifted To

Mean	SD	Min	Max	Obs
5.63	3.54	1.00	12.00	57

ha017_w4_2_2_: Month for House[2] Gifted To

Mean	SD	Min	Max	Obs
5.68	3.58	1.00	12.00	59

ha017_w4_2_3_: Month for House[3] Gifted To

Mean	SD	Min	Max	Obs
4.87	3.56	1.00	12.00	31

ha017_w4_2_4_: Month for House[4] Gifted To

Mean	SD	Min	Max	Obs
5.25	3.50	1.00	9.00	4

ha018_w4_1_: Compensation In Kind and Cash for House[1]

	Freq.	%
1 Yes	1	1.32
2 No	73	96.05
999 Refused To Answer	2	2.63
Total	76	100.00

ha018_w4_2_: Compensation In Kind and Cash for House[2]

	Freq.	%
1 Yes	1	1.33
2 No	74	98.67
Total	75	100.00

ha018_w4_3_: Compensation In Kind and Cash for House[3]

	Freq.	%
1 Yes	1	2.38
2 No	40	95.24
999 Refused To Answer	1	2.38
Total	42	100.00

ha018_w4_4_: Compensation In Kind and Cash for House[4]

	Freq.	%
2 No	5	100.00
Total	5	100.00

ha019_w4_1_: Compensation Amount for House[1]

Mean	SD	Min	Max	Obs

0.50	.	0.50	0.50	1

ha019_w4_2_: Compensation Amount for House[2]

Mean	SD	Min	Max	Obs
0.00	.	0.00	0.00	1

ha019_w4_3_: Compensation Amount for House[3]

Mean	SD	Min	Max	Obs
150.00	.	150.00	150.00	1

ha021_w4_1_1_: Year for House[1] Disposed

Mean	SD	Min	Max	Obs
2,013.65	6.04	1,991.00	2,018.00	37

ha021_w4_1_2_: Year for House[2] Disposed

Mean	SD	Min	Max	Obs
2,014.59	6.58	1,988.00	2,018.00	27

ha021_w4_1_3_: Year for House[3] Disposed

Mean	SD	Min	Max	Obs
2,014.93	6.07	1,994.00	2,018.00	14

ha021_w4_2_1_: Month for House[1] Disposed

Mean	SD	Min	Max	Obs
5.84	3.18	1.00	12.00	25

ha021_w4_2_2_: Month for House[2] Disposed

Mean	SD	Min	Max	Obs
6.70	2.94	1.00	12.00	20

ha021_w4_2_3_: Month for House[3] Disposed

Mean	SD	Min	Max	Obs
7.00	3.35	1.00	12.00	11

ha022_w4_1_: Total Amount for House[1] Disposed

Mean	SD	Min	Max	Obs

109.60	657.37	0.00	4,000.00	37

ha022_w4_2_: Total Amount for House[2] Disposed

Mean	SD	Min	Max	Obs
0.04	0.20	0.00	1.00	25

ha022_w4_3_: Total Amount for House[3] Disposed

Mean	SD	Min	Max	Obs
0.43	1.44	0.00	5.00	12

ha022_w4_1__min: Min Bracket of ha022_w4_1_

No Observations

ha022_w4_1__max: Max Bracket of ha022_w4_1_

No Observations

ha022_w4_2__min: Min Bracket of ha022_w4_2_

Mean	SD	Min	Max	Obs
100,000.00	.	100,000.00	100,000.00	1

ha022_w4_2__max: Max Bracket of ha022_w4_2_

Mean	SD	Min	Max	Obs
60,000.00	56,568.54	20,000.00	100,000.00	2

ha022_w4_3__min: Min Bracket of ha022_w4_3_

No Observations

ha022_w4_3__max: Max Bracket of ha022_w4_3_

No Observations

ha023_w4: The Number of Other/New Houses

Mean	SD	Min	Max	Obs
0.39	0.59	0.00	7.00	11,441

ha025_w4_1_: Urban or Rural for House[1]

	Freq.	%

1 City/Town Central Area	833	20.88
2 Rural-Urban Fringe	343	8.60
3 Rural	2,795	70.05
4 Special Zone	19	0.48
Total	3,990	100.00

ha025_w4_2_: Urban or Rural for House[2]

	Freq.	%
1 City/Town Central Area	170	32.82
2 Rural-Urban Fringe	61	11.78
3 Rural	282	54.44
4 Special Zone	5	0.97
Total	518	100.00

ha025_w4_3_: Urban or Rural for House[3]

	Freq.	%
1 City/Town Central Area	24	48.00
2 Rural-Urban Fringe	4	8.00
3 Rural	22	44.00
Total	50	100.00

ha025_w4_4_: Urban or Rural for House[4]

	Freq.	%
1 City/Town Central Area	4	36.36
2 Rural-Urban Fringe	3	27.27
3 Rural	4	36.36
Total	11	100.00

ha025_w4_5_: Urban or Rural for House[5]

	Freq.	%
1 City/Town Central Area	1	50.00
2 Rural-Urban Fringe	1	50.00
Total	2	100.00

ha025_w4_6_: Urban or Rural for House[6]

	Freq.	%
1 City/Town Central Area	1	50.00
2 Rural-Urban Fringe	1	50.00
Total	2	100.00

ha025_w4_7_: Urban or Rural for House[7]

	Freq.	%
1 City/Town Central Area	1	50.00
2 Rural-Urban Fringe	1	50.00

	Freq.	%
Total	2	100.00

ha025_w4_8_: Urban or Rural for House[8]

	Freq.	%
2 Rural-Urban Fringe	1	100.00
Total	1	100.00

ha025_w4_9_: Urban or Rural for House[9]

	Freq.	%
2 Rural-Urban Fringe	1	100.00
Total	1	100.00

ha025_w4_10_: Urban or Rural for House[10]

	Freq.	%
2 Rural-Urban Fringe	1	100.00
Total	1	100.00

ha025_w4_11_: Urban or Rural for House[11]

	Freq.	%
2 Rural-Urban Fringe	1	100.00
Total	1	100.00

ha026_w4_1_: House Type for House[1]

	Freq.	%
1 Unit Building	964	24.16
2 Detached House	1,832	45.91
3 Attached House	334	8.37
4 Siheyuan	153	3.83
5 Compound Occupied by Many Households	180	4.51
6 Work Shed	3	0.08
7 Haphazard Building	95	2.38
8 Other	429	10.75
Total	3,990	100.00

ha026_w4_2_: House Type for House[2]

	Freq.	%
1 Unit Building	206	39.77
2 Detached House	160	30.89
3 Attached House	50	9.65
4 Siheyuan	26	5.02
5 Compound Occupied by Many Households	19	3.67
6 Work Shed	1	0.19
7 Haphazard Building	8	1.54
8 Other	48	9.27

Total	518	100.00

ha026_w4_3_: House Type for House[3]

	Freq.	%
1 Unit Building	24	48.00
2 Detached House	14	28.00
3 Attached House	5	10.00
4 Siheyuan	4	8.00
8 Other	3	6.00
Total	50	100.00

ha026_w4_4_: House Type for House[4]

	Freq.	%
1 Unit Building	6	54.55
2 Detached House	2	18.18
3 Attached House	2	18.18
4 Siheyuan	1	9.09
Total	11	100.00

ha026_w4_5_: House Type for House[5]

	Freq.	%
1 Unit Building	2	100.00
Total	2	100.00

ha026_w4_6_: House Type for House[6]

	Freq.	%
1 Unit Building	2	100.00
Total	2	100.00

ha026_w4_7_: House Type for House[7]

	Freq.	%
1 Unit Building	1	50.00
3 Attached House	1	50.00
Total	2	100.00

ha026_w4_8_: House Type for House[8]

	Freq.	%
1 Unit Building	1	100.00
Total	1	100.00

ha026_w4_9_: House Type for House[9]

	Freq.	%
4 Siheyuan	1	100.00
Total	1	100.00

ha026_w4_10_: House Type for House[10]

	Freq.	%
4 Siheyuan	1	100.00
Total	1	100.00

ha026_w4_11_: House Type for House[11]

	Freq.	%
1 Unit Building	1	100.00
Total	1	100.00

ha027_w4_1_: Household Head and Spouse Have All Ownership for House[1]

	Freq.	%
1 Yes	3,555	89.10
2 No	435	10.90
Total	3,990	100.00

ha027_w4_2_: Household Head and Spouse Have All Ownership for House[2]

	Freq.	%
1 Yes	465	89.77
2 No	53	10.23
Total	518	100.00

ha027_w4_3_: Household Head and Spouse Have All Ownership for House[3]

	Freq.	%
1 Yes	42	84.00
2 No	8	16.00
Total	50	100.00

ha027_w4_4_: Household Head and Spouse Have All Ownership for House[4]

	Freq.	%
1 Yes	11	100.00
Total	11	100.00

ha027_w4_5_: Household Head and Spouse Have All Ownership for House[5]

	Freq.	%
1 Yes	2	100.00

Total	2	100.00

ha027_w4_6_: Household Head and Spouse Have All Ownership for House[6]

	Freq.	%
1 Yes	2	100.00
Total	2	100.00

ha027_w4_7_: Household Head and Spouse Have All Ownership for House[7]

	Freq.	%
1 Yes	2	100.00
Total	2	100.00

ha027_w4_8_: Household Head and Spouse Have All Ownership for House[8]

	Freq.	%
1 Yes	1	100.00
Total	1	100.00

ha027_w4_9_: Household Head and Spouse Have All Ownership for House[9]

	Freq.	%
1 Yes	1	100.00
Total	1	100.00

ha027_w4_10_: Household Head and Spouse Have All Ownership for House[10]

	Freq.	%
1 Yes	1	100.00
Total	1	100.00

ha027_w4_11_: Household Head and Spouse Have All Ownership for House[11]

	Freq.	%
1 Yes	1	100.00
Total	1	100.00

ha028_w4_1__s1: Household Head and Spouse Own House[1]

	Freq.	%
0 Do Not Own	193	44.37
1 Own	242	55.63
Total	435	100.00

ha028_w4_2__s1: Household Head and Spouse Own House[2]

	Freq.	%
0 Do Not Own	35	66.04
1 Own	18	33.96
Total	53	100.00

ha028_w4_3__s1: Household Head and Spouse Own House[3]

	Freq.	%
0 Do Not Own	6	75.00
1 Own	2	25.00
Total	8	100.00

ha028_w4_1__s2: Children and Children's Spouses Own House[1]

	Freq.	%
0 Do Not Own	179	41.15
2 Own	256	58.85
Total	435	100.00

ha028_w4_2__s2: Children and Children's Spouses Own House[2]

	Freq.	%
0 Do Not Own	21	39.62
2 Own	32	60.38
Total	53	100.00

ha028_w4_3__s2: Children and Children's Spouses Own House[3]

	Freq.	%
0 Do Not Own	2	25.00
2 Own	6	75.00
Total	8	100.00

ha028_w4_1__s3: Siblings Own House[1]

	Freq.	%
0 Do Not Own	398	91.49
3 Own	37	8.51
Total	435	100.00

ha028_w4_2__s3: Siblings Own House[2]

	Freq.	%
0 Do Not Own	47	88.68
3 Own	6	11.32
Total	53	100.00

ha028_w4_3__s3: Siblings Own House[3]

	Freq.	%
0 Do Not Own	8	100.00
Total	8	100.00

ha028_w4_1__s4: Parents and Parents-in-Law Own House[1]

	Freq.	%
0 Do Not Own	418	96.09
4 Own	17	3.91
Total	435	100.00

ha028_w4_2__s4: Parents and Parents-in-Law Own House[2]

	Freq.	%
0 Do Not Own	51	96.23
4 Own	2	3.77
Total	53	100.00

ha028_w4_3__s4: Parents and Parents-in-Law Own House[3]

	Freq.	%
0 Do Not Own	8	100.00
Total	8	100.00

ha028_w4_1__s5: Grandchildren Own House[1]

	Freq.	%
0 Do Not Own	420	96.55
5 Own	15	3.45
Total	435	100.00

ha028_w4_2__s5: Grandchildren Own House[2]

	Freq.	%
0 Do Not Own	51	96.23
5 Own	2	3.77
Total	53	100.00

ha028_w4_3__s5: Grandchildren Own House[3]

	Freq.	%
0 Do Not Own	8	100.00
Total	8	100.00

ha028_w4_1__s6: Other Relatives Own House[1]

	Freq.	%

	Freq.	%
0 Do Not Own	426	97.93
6 Own	9	2.07
Total	435	100.00

ha028_w4_2__s6: Other Relatives Own House[2]

	Freq.	%
0 Do Not Own	52	98.11
6 Own	1	1.89
Total	53	100.00

ha028_w4_3__s6: Other Relatives Own House[3]

	Freq.	%
0 Do Not Own	8	100.00
Total	8	100.00

ha028_w4_1__s7: Friends Own House[1]

	Freq.	%
0 Do Not Own	434	99.77
7 Own	1	0.23
Total	435	100.00

ha028_w4_2__s7: Friends Own House[2]

	Freq.	%
0 Do Not Own	53	100.00
Total	53	100.00

ha028_w4_3__s7: Friends Own House[3]

	Freq.	%
0 Do Not Own	8	100.00
Total	8	100.00

ha028_w4_1__s8: Others Own House[1]

	Freq.	%
0 Do Not Own	388	89.20
8 Own	47	10.80
Total	435	100.00

ha028_w4_2__s8: Others Own House[2]

	Freq.	%
0 Do Not Own	47	88.68
8 Own	6	11.32
Total	53	100.00

ha028_w4_3__s8: Others Own House[3]

	Freq.	%
0 Do Not Own	6	75.00
8 Own	2	25.00
Total	8	100.00

ha028_w4_1__s9: Own House[1] But Do Not Have Ownership Certificate

	Freq.	%
0 Do Not Own	406	93.33
9 Own	29	6.67
Total	435	100.00

ha028_w4_2__s9: Own House[2] But Do Not Have Ownership Certificate

	Freq.	%
0 Do Not Own	49	92.45
9 Own	4	7.55
Total	53	100.00

ha028_w4_3__s9: Own House[3] But Do Not Have Ownership Certificate

	Freq.	%
0 Do Not Own	8	100.00
Total	8	100.00

ha028_w4_1__s999: Refused to Answer House[1] Ownership

	Freq.	%
0 No	431	99.08
999 Yes	4	0.92
Total	435	100.00

ha028_w4_2__s999: Refused to Answer House[2] Ownership

	Freq.	%
0 No	53	100.00
Total	53	100.00

ha028_w4_3__s999: Refused to Answer House[3] Ownership

	Freq.	%
0 No	8	100.00
Total	8	100.00

ha028_w4_1_1_: Percent Household Head and Spouse Own House[1]

Mean	SD	Min	Max	Obs
46.61	21.19	0.00	100.00	248

ha028_w4_1_2_: Percent Household Head and Spouse Own House[2]

Mean	SD	Min	Max	Obs
39.44	22.07	0.00	100.00	18

ha028_w4_1_3_: Percent Household Head and Spouse Own House[3]

Mean	SD	Min	Max	Obs
41.65	11.81	33.30	50.00	2

ha028_w4_3_1_: Percent Siblings Own House[1]

Mean	SD	Min	Max	Obs
58.31	17.60	8.33	80.00	37

ha028_w4_3_2_: Percent Siblings Own House[2]

Mean	SD	Min	Max	Obs
68.00	18.58	50.00	100.00	6

ha028_w4_6_1_: Percent Other Relatives Own House[1]

Mean	SD	Min	Max	Obs
63.60	27.05	10.00	100.00	9

ha028_w4_6_2_: Percent Other Relatives Own House[2]

Mean	SD	Min	Max	Obs
100.00	.	100.00	100.00	1

ha028_w4_7_1_: Percent Friends Own House[1]

Mean	SD	Min	Max	Obs
75.00	.	75.00	75.00	1

ha028_w4_8_1_: Percent Others Own House[1]

Mean	SD	Min	Max	Obs
72.56	27.70	0.00	100.00	48

ha028_w4_8_2_: Percent Others Own House[2]

Mean	SD	Min	Max	Obs
88.88	27.23	33.30	100.00	6

ha028_w4_8_3_: Percent Others Own House[3]

Mean	SD	Min	Max	Obs
83.35	23.55	66.70	100.00	2

ha029_w4_1__s1: XChildName[1]/'s Spouse Owns House[1]

	Freq.	%
0 Do Not Own	90	35.16
1 Own	166	64.84
Total	256	100.00

ha029_w4_2__s1: XChildName[1]/'s Spouse Owns House[2]

	Freq.	%
0 Do Not Own	13	40.63
1 Own	19	59.38
Total	32	100.00

ha029_w4_3__s1: XChildName[1]/'s Spouse Owns House[3]

	Freq.	%
0 Do Not Own	2	33.33
1 Own	4	66.67
Total	6	100.00

ha029_w4_1__s2: XChildName[2]/'s Spouse Owns House[1]

	Freq.	%
0 Do Not Own	170	66.41
2 Own	86	33.59
Total	256	100.00

ha029_w4_2__s2: XChildName[2]/'s Spouse Owns House[2]

	Freq.	%
0 Do Not Own	19	59.38
2 Own	13	40.63
Total	32	100.00

ha029_w4_3__s2: XChildName[2]/'s Spouse Owns House[3]

	Freq.	%
0 Do Not Own	5	83.33

	Freq.	%
2 Own	1	16.67
Total	6	100.00

ha029_w4_1__s3: XChildName[3]/'s Spouse Owns House[1]

	Freq.	%
0 Do Not Own	214	83.59
3 Own	42	16.41
Total	256	100.00

ha029_w4_2__s3: XChildName[3]/'s Spouse Owns House[2]

	Freq.	%
0 Do Not Own	30	93.75
3 Own	2	6.25
Total	32	100.00

ha029_w4_3__s3: XChildName[3]/'s Spouse Owns House[3]

	Freq.	%
0 Do Not Own	5	83.33
3 Own	1	16.67
Total	6	100.00

ha029_w4_1__s4: XChildName[4]/'s Spouse Owns House[1]

	Freq.	%
0 Do Not Own	242	94.53
4 Own	14	5.47
Total	256	100.00

ha029_w4_2__s4: XChildName[4]/'s Spouse Owns House[2]

	Freq.	%
0 Do Not Own	29	90.63
4 Own	3	9.38
Total	32	100.00

ha029_w4_3__s4: XChildName[4]/'s Spouse Owns House[3]

	Freq.	%
0 Do Not Own	6	100.00
Total	6	100.00

ha029_w4_1__s5: XChildName[5]/'s Spouse Owns House[1]

	Freq.	%
0 Do Not Own	251	98.05
5 Own	5	1.95

Total	256	100.00

ha029_w4_2__s5: XChildName[5]/'s Spouse Owns House[2]

	Freq.	%
0 Do Not Own	32	100.00
Total	32	100.00

ha029_w4_3__s5: XChildName[5]/'s Spouse Owns House[3]

	Freq.	%
0 Do Not Own	6	100.00
Total	6	100.00

ha029_w4_1__s6: XChildName[6]/'s Spouse Owns House[1]

	Freq.	%
0 Do Not Own	256	100.00
Total	256	100.00

ha029_w4_2__s6: XChildName[6]/'s Spouse Owns House[2]

	Freq.	%
0 Do Not Own	32	100.00
Total	32	100.00

ha029_w4_3__s6: XChildName[6]/'s Spouse Owns House[3]

	Freq.	%
0 Do Not Own	6	100.00
Total	6	100.00

ha029_w4_1__s7: XChildName[7]/'s Spouse Owns House[1]

	Freq.	%
0 Do Not Own	255	99.61
7 Own	1	0.39
Total	256	100.00

ha029_w4_2__s7: XChildName[7]/'s Spouse Owns House[2]

	Freq.	%
0 Do Not Own	32	100.00
Total	32	100.00

ha029_w4_3__s7: XChildName[7]/'s Spouse Owns House[3]

	Freq.	%
0 Do Not Own	6	100.00
Total	6	100.00

ha029_w4_1__s8: XChildName[8]/'s Spouse Owns House[1]

	Freq.	%
0 Do Not Own	255	99.61
8 Own	1	0.39
Total	256	100.00

ha029_w4_2__s8: XChildName[8]/'s Spouse Owns House[2]

	Freq.	%
0 Do Not Own	32	100.00
Total	32	100.00

ha029_w4_3__s8: XChildName[8]/'s Spouse Owns House[3]

	Freq.	%
0 Do Not Own	6	100.00
Total	6	100.00

ha029_w4_1__s9: XChildName[9]/'s Spouse Owns House[1]

	Freq.	%
0 Do Not Own	255	99.61
9 Own	1	0.39
Total	256	100.00

ha029_w4_2__s9: XChildName[9]/'s Spouse Owns House[2]

	Freq.	%
0 Do Not Own	32	100.00
Total	32	100.00

ha029_w4_3__s9: XChildName[9]/'s Spouse Owns House[3]

	Freq.	%
0 Do Not Own	6	100.00
Total	6	100.00

ha029_w4_1__s10: XChildName[10]/'s Spouse Owns House[1]

	Freq.	%
0 Do Not Own	256	100.00
Total	256	100.00

ha029_w4_2__s10: XChildName[10]/'s Spouse Owns House[2]

	Freq.	%
0 Do Not Own	32	100.00
Total	32	100.00

ha029_w4_3__s10: XChildName[10]/'s Spouse Owns House[3]

	Freq.	%
0 Do Not Own	6	100.00
Total	6	100.00

ha029_w4_1__s11: XChildName[11]/'s Spouse Owns House[1]

	Freq.	%
0 Do Not Own	255	99.61
11 Own	1	0.39
Total	256	100.00

ha029_w4_2__s11: XChildName[11]/'s Spouse Owns House[2]

	Freq.	%
0 Do Not Own	32	100.00
Total	32	100.00

ha029_w4_3__s11: XChildName[11]/'s Spouse Owns House[3]

	Freq.	%
0 Do Not Own	6	100.00
Total	6	100.00

ha029_w4_1__s12: XChildName[12]/'s Spouse Owns House[1]

	Freq.	%
0 Do Not Own	256	100.00
Total	256	100.00

ha029_w4_2__s12: XChildName[12]/'s Spouse Owns House[2]

	Freq.	%
0 Do Not Own	32	100.00
Total	32	100.00

ha029_w4_3__s12: XChildName[12]/'s Spouse Owns House[3]

	Freq.	%
0 Do Not Own	6	100.00
Total	6	100.00

ha029_w4_1__s13: XChildName[13]/'s Spouse Owns House[1]

	Freq.	%
0 Do Not Own	256	100.00
Total	256	100.00

ha029_w4_2__s13: XChildName[13]/'s Spouse Owns House[2]

	Freq.	%
0 Do Not Own	32	100.00
Total	32	100.00

ha029_w4_3__s13: XChildName[13]/'s Spouse Owns House[3]

	Freq.	%
0 Do Not Own	6	100.00
Total	6	100.00

ha029_w4_1__s14: XChildName[14]/'s Spouse Owns House[1]

	Freq.	%
0 Do Not Own	256	100.00
Total	256	100.00

ha029_w4_2__s14: XChildName[14]/'s Spouse Owns House[2]

	Freq.	%
0 Do Not Own	32	100.00
Total	32	100.00

ha029_w4_3__s14: XChildName[14]/'s Spouse Owns House[3]

	Freq.	%
0 Do Not Own	6	100.00
Total	6	100.00

ha029_w4_1__s15: XChildName[15]/'s Spouse Owns House[1]

	Freq.	%
0 Do Not Own	256	100.00
Total	256	100.00

ha029_w4_2__s15: XChildName[15]/'s Spouse Owns House[2]

	Freq.	%
0 Do Not Own	32	100.00
Total	32	100.00

ha029_w4_3__s15: XChildName[15]/'s Spouse Owns House[3]

	Freq.	%
0 Do Not Own	6	100.00
Total	6	100.00

ha029_w4_1__s16: XChildName[16]/'s Spouse Owns House[1]

	Freq.	%
0 Do Not Own	256	100.00
Total	256	100.00

ha029_w4_2__s16: XChildName[16]/'s Spouse Owns House[2]

	Freq.	%
0 Do Not Own	32	100.00
Total	32	100.00

ha029_w4_3__s16: XChildName[16]/'s Spouse Owns House[3]

	Freq.	%
0 Do Not Own	6	100.00
Total	6	100.00

ha029_w4_1__s99: None of the Above Owns House[1]

	Freq.	%
0 No	251	98.05
99 Yes	5	1.95
Total	256	100.00

ha029_w4_2__s99: None of the Above Owns House[2]

	Freq.	%
0 No	32	100.00
Total	32	100.00

ha029_w4_3__s99: None of the Above Owns House[3]

	Freq.	%
0 No	6	100.00
Total	6	100.00

ha030_w4_1__1_: Percent XChildName[1]/'s Spouse Owns House[1]

Mean	SD	Min	Max	Obs
59.54	31.33	0.00	100.00	166

ha030_w4_2__1_: Percent XChildName[1]/'s Spouse Owns House[2]

Mean	SD	Min	Max	Obs
78.07	30.51	25.00	100.00	19

ha030_w4_3__1_: Percent XChildName[1]/'s Spouse Owns House[3]

Mean	SD	Min	Max	Obs
87.50	25.00	50.00	100.00	4

ha030_w4_1__2_: Percent XChildName[2]/'s Spouse Owns House[1]

Mean	SD	Min	Max	Obs
52.70	30.48	0.00	100.00	86

ha030_w4_2__2_: Percent XChildName[2]/'s Spouse Owns House[2]

Mean	SD	Min	Max	Obs
67.79	31.86	25.00	100.00	13

ha030_w4_3__2_: Percent XChildName[2]/'s Spouse Owns House[3]

Mean	SD	Min	Max	Obs
100.00	.	100.00	100.00	1

ha030_w4_1__3_: Percent XChildName[3]/'s Spouse Owns House[1]

Mean	SD	Min	Max	Obs
54.76	32.89	12.50	100.00	42

ha030_w4_2__3_: Percent XChildName[3]/'s Spouse Owns House[2]

Mean	SD	Min	Max	Obs
66.50	47.38	33.00	100.00	2

ha030_w4_3__3_: Percent XChildName[3]/'s Spouse Owns House[3]

Mean	SD	Min	Max	Obs
100.00	.	100.00	100.00	1

ha030_w4_1__4_: Percent XChildName[4]/'s Spouse Owns House[1]

Mean	SD	Min	Max	Obs
52.15	33.53	12.50	100.00	14

ha030_w4_2__4_: Percent XChildName[4]/'s Spouse Owns House[2]

Mean	SD	Min	Max	Obs
61.10	34.71	33.30	100.00	3

ha030_w4_1__5_: Percent XChildName[5]/'s Spouse Owns House[1]

Mean	SD	Min	Max	Obs
59.80	38.62	16.00	100.00	5

ha030_w4_1__7_: Percent XChildName[7]/'s Spouse Owns House[1]

Mean	SD	Min	Max	Obs
100.00	.	100.00	100.00	1

ha030_w4_1__8_: Percent XChildName[8]/'s Spouse Owns House[1]

Mean	SD	Min	Max	Obs
50.00	.	50.00	50.00	1

ha030_w4_1__9_: Percent XChildName[9]/'s Spouse Owns House[1]

Mean	SD	Min	Max	Obs
100.00	.	100.00	100.00	1

ha030_w4_1__11_: Percent XChildName[11]/'s Spouse Owns House[1]

Mean	SD	Min	Max	Obs
100.00	.	100.00	100.00	1

ha031_w4_1__s1: XConParName[1] Owns House[1]

	Freq.	%
0 Do Not Own	10	58.82
1 Own	7	41.18
Total	17	100.00

ha031_w4_2__s1: XConParName[1] Owns House[2]

	Freq.	%
0 Do Not Own	2	100.00
Total	2	100.00

ha031_w4_1__s2: XConParName[2] Owns House[1]

	Freq.	%
0 Do Not Own	8	47.06
2 Own	9	52.94

	Freq.	%
Total	17	100.00

ha031_w4_2__s2: XConParName[2] Owns House[2]

	Freq.	%
0 Do Not Own	1	50.00
2 Own	1	50.00
Total	2	100.00

ha031_w4_1__s3: XConParName[3] Owns House[1]

	Freq.	%
0 Do Not Own	17	100.00
Total	17	100.00

ha031_w4_2__s3: XConParName[3] Owns House[2]

	Freq.	%
0 Do Not Own	2	100.00
Total	2	100.00

ha031_w4_1__s4: XConParName[4] Owns House[1]

	Freq.	%
0 Do Not Own	17	100.00
Total	17	100.00

ha031_w4_2__s4: XConParName[4] Owns House[2]

	Freq.	%
0 Do Not Own	2	100.00
Total	2	100.00

ha031_w4_1__s5: XConParName[5] Owns House[1]

	Freq.	%
0 Do Not Own	15	88.24
5 Own	2	11.76
Total	17	100.00

ha031_w4_2__s5: XConParName[5] Owns House[2]

	Freq.	%
0 Do Not Own	1	50.00
5 Own	1	50.00
Total	2	100.00

ha031_w4_1__s6: XConParName[6] Owns House[1]

	Freq.	%
0 Do Not Own	16	94.12
6 Own	1	5.88
Total	17	100.00

ha031_w4_2__s6: XConParName[6] Owns House[2]

	Freq.	%
0 Do Not Own	1	50.00
6 Own	1	50.00
Total	2	100.00

ha031_w4_1__s7: XConParName[7] Owns House[1]

	Freq.	%
0 Do Not Own	17	100.00
Total	17	100.00

ha031_w4_2__s7: XConParName[7] Owns House[2]

	Freq.	%
0 Do Not Own	2	100.00
Total	2	100.00

ha031_w4_1__s8: XConParName[8] Owns House[1]

	Freq.	%
0 Do Not Own	17	100.00
Total	17	100.00

ha031_w4_2__s8: XConParName[8] Owns House[2]

	Freq.	%
0 Do Not Own	2	100.00
Total	2	100.00

ha031_w4_1__s99: None of the Above Owns House[1]

	Freq.	%
0 No	16	94.12
99 Yes	1	5.88
Total	17	100.00

ha031_w4_2__s99: None of the Above Owns House[2]

	Freq.	%
0 No	2	100.00

Total	2	100.00

ha032_w4_1__1_: Percent XConParName[1] Owns House[1]

Mean	SD	Min	Max	Obs
57.14	31.34	25.00	100.00	7

ha032_w4_1__2_: Percent XConParName[2] Owns House[1]

Mean	SD	Min	Max	Obs
54.00	36.51	16.00	100.00	9

ha032_w4_2__2_: Percent XConParName[2] Owns House[2]

Mean	SD	Min	Max	Obs
20.00	.	20.00	20.00	1

ha032_w4_1__5_: Percent XConParName[5] Owns House[1]

Mean	SD	Min	Max	Obs
29.17	5.89	25.00	33.33	2

ha032_w4_2__5_: Percent XConParName[5] Owns House[2]

Mean	SD	Min	Max	Obs
50.00	.	50.00	50.00	1

ha032_w4_1__6_: Percent XConParName[6] Owns House[1]

Mean	SD	Min	Max	Obs
25.00	.	25.00	25.00	1

ha032_w4_2__6_: Percent XConParName[6] Owns House[2]

Mean	SD	Min	Max	Obs
50.00	.	50.00	50.00	1

ha033_w4_1__s1: XChildName[1]'s Child(Grandchild) Owns House[1]

	Freq.	%
0 Do Not Own	5	33.33
1 Own	10	66.67
Total	15	100.00

ha033_w4_2__s1: XChildName[1]'s Child(Grandchild) Owns House[2]

	Freq.	%
0 Do Not Own	1	50.00
1 Own	1	50.00
Total	2	100.00

ha033_w4_1__s2: XChildName[2]'s Child(Grandchild) Owns House[1]

	Freq.	%
0 Do Not Own	11	73.33
2 Own	4	26.67
Total	15	100.00

ha033_w4_2__s2: XChildName[2]'s Child(Grandchild) Owns House[2]

	Freq.	%
0 Do Not Own	1	50.00
2 Own	1	50.00
Total	2	100.00

ha033_w4_1__s3: XChildName[3]'s Child(Grandchild) Owns House[1]

	Freq.	%
0 Do Not Own	14	93.33
3 Own	1	6.67
Total	15	100.00

ha033_w4_2__s3: XChildName[3]'s Child(Grandchild) Owns House[2]

	Freq.	%
0 Do Not Own	2	100.00
Total	2	100.00

ha033_w4_1__s4: XChildName[4]'s Child(Grandchild) Owns House[1]

	Freq.	%
0 Do Not Own	15	100.00
Total	15	100.00

ha033_w4_2__s4: XChildName[4]'s Child(Grandchild) Owns House[2]

	Freq.	%
0 Do Not Own	2	100.00
Total	2	100.00

ha033_w4_1__s5: XChildName[5]'s Child(Grandchild) Owns House[1]

	Freq.	%

	Freq.	%
0 Do Not Own	15	100.00
Total	15	100.00

ha033_w4_2__s5: XChildName[5]'s Child(Grandchild) Owns House[2]

	Freq.	%
0 Do Not Own	2	100.00
Total	2	100.00

ha033_w4_1__s6: XChildName[6]'s Child(Grandchild) Owns House[1]

	Freq.	%
0 Do Not Own	15	100.00
Total	15	100.00

ha033_w4_2__s6: XChildName[6]'s Child(Grandchild) Owns House[2]

	Freq.	%
0 Do Not Own	2	100.00
Total	2	100.00

ha033_w4_1__s7: XChildName[7]'s Child(Grandchild) Owns House[1]

	Freq.	%
0 Do Not Own	15	100.00
Total	15	100.00

ha033_w4_2__s7: XChildName[7]'s Child(Grandchild) Owns House[2]

	Freq.	%
0 Do Not Own	2	100.00
Total	2	100.00

ha033_w4_1__s8: XChildName[8]'s Child(Grandchild) Owns House[1]

	Freq.	%
0 Do Not Own	15	100.00
Total	15	100.00

ha033_w4_2__s8: XChildName[8]'s Child(Grandchild) Owns House[2]

	Freq.	%
0 Do Not Own	2	100.00
Total	2	100.00

ha033_w4_1__s9: XChildName[9]'s Child(Grandchild) Owns House[1]

	Freq.	%
0 Do Not Own	15	100.00
Total	15	100.00

ha033_w4_2__s9: XChildName[9]'s Child(Grandchild) Owns House[2]

	Freq.	%
0 Do Not Own	2	100.00
Total	2	100.00

ha033_w4_1__s10: XChildName[10]'s Child(Grandchild) Owns House[1]

	Freq.	%
0 Do Not Own	15	100.00
Total	15	100.00

ha033_w4_2__s10: XChildName[10]'s Child(Grandchild) Owns House[2]

	Freq.	%
0 Do Not Own	2	100.00
Total	2	100.00

ha033_w4_1__s11: XChildName[11]'s Child(Grandchild) Owns House[1]

	Freq.	%
0 Do Not Own	15	100.00
Total	15	100.00

ha033_w4_2__s11: XChildName[11]'s Child(Grandchild) Owns House[2]

	Freq.	%
0 Do Not Own	2	100.00
Total	2	100.00

ha033_w4_1__s12: XChildName[12]'s Child(Grandchild) Owns House[1]

	Freq.	%
0 Do Not Own	15	100.00
Total	15	100.00

ha033_w4_2__s12: XChildName[12]'s Child(Grandchild) Owns House[2]

	Freq.	%
0 Do Not Own	2	100.00
Total	2	100.00

ha033_w4_1__s13: XChildName[13]'s Child(Grandchild) Owns House[1]

	Freq.	%
0 Do Not Own	15	100.00
Total	15	100.00

ha033_w4_2__s13: XChildName[13]'s Child(Grandchild) Owns House[2]

	Freq.	%
0 Do Not Own	2	100.00
Total	2	100.00

ha033_w4_1__s14: XChildName[14]'s Child(Grandchild) Owns House[1]

	Freq.	%
0 Do Not Own	15	100.00
Total	15	100.00

ha033_w4_2__s14: XChildName[14]'s Child(Grandchild) Owns House[2]

	Freq.	%
0 Do Not Own	2	100.00
Total	2	100.00

ha033_w4_1__s15: XChildName[15]'s Child(Grandchild) Owns House[1]

	Freq.	%
0 Do Not Own	15	100.00
Total	15	100.00

ha033_w4_2__s15: XChildName[15]'s Child(Grandchild) Owns House[2]

	Freq.	%
0 Do Not Own	2	100.00
Total	2	100.00

ha033_w4_1__s16: XChildName[16]'s Child(Grandchild) Owns House[1]

	Freq.	%
0 Do Not Own	15	100.00
Total	15	100.00

ha033_w4_2__s16: XChildName[16]'s Child(Grandchild) Owns House[2]

	Freq.	%
0 Do Not Own	2	100.00
Total	2	100.00

ha034_w4_1__1_: Percent XChildName[1]'s Child(Grandchild) Owns House[1]

Mean	SD	Min	Max	Obs
55.63	38.44	20.00	100.00	10

ha034_w4_2__1_: Percent XChildName[1]'s Child(Grandchild) Owns House[2]

Mean	SD	Min	Max	Obs
25.00	.	25.00	25.00	1

ha034_w4_1__2_: Percent XChildName[2]'s Child(Grandchild) Owns House[1]

Mean	SD	Min	Max	Obs
63.25	42.77	20.00	100.00	4

ha034_w4_2__2_: Percent XChildName[2]'s Child(Grandchild) Owns House[2]

Mean	SD	Min	Max	Obs
20.00	.	20.00	20.00	1

ha034_w4_1__3_: Percent XChildName[3]'s Child(Grandchild) Owns House[1]

Mean	SD	Min	Max	Obs
40.00	.	40.00	40.00	1

ha035_w4_1_: House Size for House[1]

Mean	SD	Min	Max	Obs
134.72	120.06	0.00	2,000.00	3,989

ha035_w4_2_: House Size for House[2]

Mean	SD	Min	Max	Obs
128.65	158.95	0.00	2,600.00	518

ha035_w4_3_: House Size for House[3]

Mean	SD	Min	Max	Obs
328.82	1,399.68	10.00	10,000.00	50

ha035_w4_4_: House Size for House[4]

Mean	SD	Min	Max	Obs
133.27	119.18	23.00	450.00	11

ha035_w4_5_: House Size for House[5]

Mean	SD	Min	Max	Obs
143.00	18.38	130.00	156.00	2

ha035_w4_6_: House Size for House[6]

Mean	SD	Min	Max	Obs
380.00	395.98	100.00	660.00	2

ha035_w4_7_: House Size for House[7]

Mean	SD	Min	Max	Obs
147.50	116.67	65.00	230.00	2

ha035_w4_8_: House Size for House[8]

Mean	SD	Min	Max	Obs
161.00	.	161.00	161.00	1

ha035_w4_9_: House Size for House[9]

Mean	SD	Min	Max	Obs
225.00	.	225.00	225.00	1

ha035_w4_10_: House Size for House[10]

Mean	SD	Min	Max	Obs
144.00	.	144.00	144.00	1

ha035_w4_11_: House Size for House[11]

Mean	SD	Min	Max	Obs
120.00	.	120.00	120.00	1

ha036_w4_1_: Total Value or Unit Value for House[1]

	Freq.	%
1 Total Value	3,797	95.21
2 Unit Value	191	4.79
Total	3,988	100.00

ha036_w4_2_: Total Value or Unit Value for House[2]

	Freq.	%
1 Total Value	475	91.70
2 Unit Value	43	8.30
Total	518	100.00

ha036_w4_3_: Total Value or Unit Value for House[3]

	Freq.	%
1 Total Value	46	92.00
2 Unit Value	4	8.00
Total	50	100.00

ha036_w4_4_: Total Value or Unit Value for House[4]

	Freq.	%
1 Total Value	10	90.91
2 Unit Value	1	9.09
Total	11	100.00

ha036_w4_5_: Total Value or Unit Value for House[5]

	Freq.	%
1 Total Value	1	50.00
2 Unit Value	1	50.00
Total	2	100.00

ha036_w4_6_: Total Value or Unit Value for House[6]

	Freq.	%
1 Total Value	2	100.00
Total	2	100.00

ha036_w4_7_: Total Value or Unit Value for House[7]

	Freq.	%
1 Total Value	1	50.00
2 Unit Value	1	50.00
Total	2	100.00

ha036_w4_8_: Total Value or Unit Value for House[8]

	Freq.	%
1 Total Value	1	100.00
Total	1	100.00

ha036_w4_9_: Total Value or Unit Value for House[9]

	Freq.	%
1 Total Value	1	100.00
Total	1	100.00

ha036_w4_10_: Total Value or Unit Value for House[10]

	Freq.	%
1 Total Value	1	100.00
Total	1	100.00

ha036_w4_11_: Total Value or Unit Value for House[11]

	Freq.	%
1 Total Value	1	100.00
Total	1	100.00

ha036_w4_1_1_: Total Value for House[1]

Mean	SD	Min	Max	Obs
8,638.95	78,584.79	0.00	2,400,000.00	3,101

ha036_w4_1_2_: Total Value for House[2]

Mean	SD	Min	Max	Obs
24,878.08	262,173.36	0.00	5,000,000.00	411

ha036_w4_1_3_: Total Value for House[3]

Mean	SD	Min	Max	Obs
73,252.50	468,508.20	0.05	3,000,000.00	41

ha036_w4_1_4_: Total Value for House[4]

Mean	SD	Min	Max	Obs
50,065.33	158,090.94	0.30	500,000.00	10

ha036_w4_1_5_: Total Value for House[5]

Mean	SD	Min	Max	Obs
70.00	.	70.00	70.00	1

ha036_w4_1_6_: Total Value for House[6]

Mean	SD	Min	Max	Obs
290.00	296.98	80.00	500.00	2

ha036_w4_1_7_: Total Value for House[7]

Mean	SD	Min	Max	Obs
26.00	.	26.00	26.00	1

ha036_w4_1_8_: Total Value for House[8]

Mean	SD	Min	Max	Obs
110.00	.	110.00	110.00	1

ha036_w4_1_9_: Total Value for House[9]

Mean	SD	Min	Max	Obs
200.00	.	200.00	200.00	1

ha036_w4_1_10_: Total Value for House[10]

Mean	SD	Min	Max	Obs
150.00	.	150.00	150.00	1

ha036_w4_1_11_: Total Value for House[11]

Mean	SD	Min	Max	Obs
98.00	.	98.00	98.00	1

ha036_w4_2_1_: Unit Value for House[1]

Mean	SD	Min	Max	Obs
2,484.23	8,650.41	0.00	100,000.00	165

ha036_w4_2_2_: Unit Value for House[2]

Mean	SD	Min	Max	Obs
2,867.16	5,060.55	1.60	25,000.00	37

ha036_w4_2_3_: Unit Value for House[3]

Mean	SD	Min	Max	Obs
10.00	6.53	2.00	18.00	4

ha036_w4_2_4_: Unit Value for House[4]

Mean	SD	Min	Max	Obs
10.00	.	10.00	10.00	1

ha036_w4_2_5_: Unit Value for House[5]

Mean	SD	Min	Max	Obs
24.00	.	24.00	24.00	1

ha036_w4_2_7_: Unit Value for House[7]

Mean	SD	Min	Max	Obs
25.00	.	25.00	25.00	1

ha036_w4_1__min: Min Bracket of ha036_w4_1_

Mean	SD	Min	Max	Obs
4,484.21	4,504.82	1,000.00	15,000.00	190

ha036_w4_1__max: Max Bracket of ha036_w4_1_

Mean	SD	Min	Max	Obs
3,031.91	3,083.56	1,000.00	15,000.00	376

ha036_w4_2__min: Min Bracket of ha036_w4_2_

Mean	SD	Min	Max	Obs
3,739.13	4,025.12	1,000.00	15,000.00	23

ha036_w4_2__max: Max Bracket of ha036_w4_2_

Mean	SD	Min	Max	Obs
2,825.00	2,890.19	1,000.00	15,000.00	40

ha036_w4_3__min: Min Bracket of ha036_w4_3_

Mean	SD	Min	Max	Obs
8,000.00	9,899.49	1,000.00	15,000.00	2

ha036_w4_3__max: Max Bracket of ha036_w4_3_

Mean	SD	Min	Max	Obs
1,000.00	0.00	1,000.00	1,000.00	2

ha037_w4_1_: Year Get House[1]

Mean	SD	Min	Max	Obs
1,998.96	17.07	1,900.00	2,018.00	3,922

ha037_w4_2_: Year Get House[2]

Mean	SD	Min	Max	Obs
2,001.72	17.37	1,900.00	2,018.00	508

ha037_w4_3_: Year Get House[3]

Mean	SD	Min	Max	Obs
2,006.29	12.52	1,950.00	2,018.00	49

ha037_w4_4_: Year Get House[4]

Mean	SD	Min	Max	Obs
2,012.45	4.48	2,003.00	2,018.00	11

ha037_w4_5_: Year Get House[5]

Mean	SD	Min	Max	Obs
2,011.50	0.71	2,011.00	2,012.00	2

ha037_w4_6_: Year Get House[6]

Mean	SD	Min	Max	Obs
2,013.00	4.24	2,010.00	2,016.00	2

ha037_w4_7_: Year Get House[7]

Mean	SD	Min	Max	Obs
2,008.00	1.41	2,007.00	2,009.00	2

ha037_w4_8_: Year Get House[8]

Mean	SD	Min	Max	Obs
2,018.00	.	2,018.00	2,018.00	1

ha037_w4_9_: Year Get House[9]

Mean	SD	Min	Max	Obs
1,998.00	.	1,998.00	1,998.00	1

ha037_w4_10_: Year Get House[10]

Mean	SD	Min	Max	Obs
1,988.00	.	1,988.00	1,988.00	1

ha037_w4_11_: Year Get House[11]

Mean	SD	Min	Max	Obs
2,017.00	.	2,017.00	2,017.00	1

ha038_w4_1_: Money Paid to Get House[1]

Mean	SD	Min	Max	Obs
5,437.33	40,851.29	0.00	1,000,000.00	3,752

ha038_w4_2_: Money Paid to Get House[2]

Mean	SD	Min	Max	Obs
11,604.26	101,967.03	0.00	2,000,000.00	484

ha038_w4_3_: Money Paid to Get House[3]

Mean	SD	Min	Max	Obs
33,942.30	221,138.69	0.00	1,500,000.00	46

ha038_w4_4_: Money Paid to Get House[4]

Mean	SD	Min	Max	Obs
18,213.83	60,291.66	0.10	200,000.00	11

ha038_w4_5_: Money Paid to Get House[5]

Mean	SD	Min	Max	Obs
58.50	44.55	27.00	90.00	2

ha038_w4_6_: Money Paid to Get House[6]

Mean	SD	Min	Max	Obs
213.25	264.10	26.50	400.00	2

ha038_w4_7_: Money Paid to Get House[7]

Mean	SD	Min	Max	Obs
106.00	132.94	12.00	200.00	2

ha038_w4_8_: Money Paid to Get House[8]

Mean	SD	Min	Max	Obs
110.00	.	110.00	110.00	1

ha038_w4_9_: Money Paid to Get House[9]

Mean	SD	Min	Max	Obs
5.50	.	5.50	5.50	1

ha038_w4_10_: Money Paid to Get House[10]

Mean	SD	Min	Max	Obs
0.30	.	0.30	0.30	1

ha038_w4_11_: Money Paid to Get House[11]

Mean	SD	Min	Max	Obs
98.00	.	98.00	98.00	1

ha039_w4_1_: House[1] Rent Out

	Freq.	%
1 Yes	190	4.76
2 No	3,797	95.21
999 Refused to Answer	1	0.03
Total	3,988	100.00

ha039_w4_2_: House[2] Rent Out

	Freq.	%
1 Yes	69	13.32
2 No	449	86.68
Total	518	100.00

ha039_w4_3_: House[3] Rent Out

	Freq.	%
1 Yes	15	30.00
2 No	35	70.00
Total	50	100.00

ha039_w4_4_: House[4] Rent Out

	Freq.	%
1 Yes	3	27.27
2 No	8	72.73
Total	11	100.00

ha039_w4_5_: House[5] Rent Out

	Freq.	%
2 No	2	100.00
Total	2	100.00

ha039_w4_6_: House[6] Rent Out

	Freq.	%
2 No	2	100.00

Total	2	100.00

ha039_w4_7_: House[7] Rent Out

	Freq.	%
2 No	2	100.00
Total	2	100.00

ha039_w4_8_: House[8] Rent Out

	Freq.	%
2 No	1	100.00
Total	1	100.00

ha039_w4_9_: House[9] Rent Out

	Freq.	%
1 Yes	1	100.00
Total	1	100.00

ha039_w4_10_: House[10] Rent Out

	Freq.	%
1 Yes	1	100.00
Total	1	100.00

ha039_w4_11_: House[11] Rent Out

	Freq.	%
2 No	1	100.00
Total	1	100.00

ha040_w4_1_: House[1]'s Rent

Mean	SD	Min	Max	Obs
1,720.62	3,088.92	0.00	30,000.00	175

ha040_w4_2_: House[2]'s Rent

Mean	SD	Min	Max	Obs
1,872.15	2,851.28	0.00	15,000.00	69

ha040_w4_3_: House[3]'s Rent

Mean	SD	Min	Max	Obs
2,565.33	4,924.92	200.00	20,000.00	15

ha040_w4_4_: House[4]'s Rent

Mean	SD	Min	Max	Obs
2,446.67	2,707.13	340.00	5,500.00	3

ha040_w4_9_: House[9]'s Rent

Mean	SD	Min	Max	Obs
8,333.00	.	8,333.00	8,333.00	1

ha040_w4_10_: House[10]'s Rent

Mean	SD	Min	Max	Obs
6,666.00	.	6,666.00	6,666.00	1

ha040_w4_1__min: Min Bracket of ha040_w4_1_

Mean	SD	Min	Max	Obs
1,357.14	801.78	500.00	2,000.00	7

ha040_w4_1__max: Max Bracket of ha040_w4_1_

Mean	SD	Min	Max	Obs
1,250.00	801.78	500.00	2,000.00	8

ha041_w4_1_: House[1]'s Market Rent

Mean	SD	Min	Max	Obs
1,021.08	2,497.81	0.00	50,000.00	2,633

ha041_w4_2_: House[2]'s Market Rent

Mean	SD	Min	Max	Obs
1,148.59	1,842.60	0.00	15,000.00	395

ha041_w4_3_: House[3]'s Market Rent

Mean	SD	Min	Max	Obs
2,031.54	3,221.72	0.00	20,000.00	39

ha041_w4_4_: House[4]'s Market Rent

Mean	SD	Min	Max	Obs
1,937.78	1,343.07	340.00	4,500.00	9

ha041_w4_5_: House[5]'s Market Rent

Mean	SD	Min	Max	Obs
1,600.00	565.69	1,200.00	2,000.00	2

ha041_w4_6_: House[6]'s Market Rent

Mean	SD	Min	Max	Obs
4,000.00	2,828.43	2,000.00	6,000.00	2

ha041_w4_7_: House[7]'s Market Rent

Mean	SD	Min	Max	Obs
2,900.00	2,969.85	800.00	5,000.00	2

ha041_w4_8_: House[8]'s Market Rent

Mean	SD	Min	Max	Obs
1,500.00	.	1,500.00	1,500.00	1

ha041_w4_9_: House[9]'s Market Rent

Mean	SD	Min	Max	Obs
8,333.00	.	8,333.00	8,333.00	1

ha041_w4_10_: House[10]'s Market Rent

Mean	SD	Min	Max	Obs
6,666.00	.	6,666.00	6,666.00	1

ha041_w4_11_: House[11]'s Market Rent

Mean	SD	Min	Max	Obs
1,500.00	.	1,500.00	1,500.00	1

ha041_w4_1__min: Min Bracket of ha041_w4_1_

Mean	SD	Min	Max	Obs
1,309.88	1,436.52	500.00	8,000.00	334

ha041_w4_1__max: Max Bracket of ha041_w4_1_

Mean	SD	Min	Max	Obs
952.46	1,262.62	500.00	8,000.00	1,136

ha041_w4_2__min: Min Bracket of ha041_w4_2_

Mean	SD	Min	Max	Obs
1,632.35	1,377.90	500.00	5,000.00	34

ha041_w4_2__max: Max Bracket of ha041_w4_2_

Mean	SD	Min	Max	Obs
1,129.81	1,403.87	500.00	8,000.00	104

ha041_w4_3__min: Min Bracket of ha041_w4_3_

Mean	SD	Min	Max	Obs
1,166.67	763.76	500.00	2,000.00	3

ha041_w4_3__max: Max Bracket of ha041_w4_3_

Mean	SD	Min	Max	Obs
1,000.00	774.60	500.00	2,000.00	6

ha041_w4_4__min: Min Bracket of ha041_w4_4_

Mean	SD	Min	Max	Obs
5,000.00	.	5,000.00	5,000.00	1

ha041_w4_4__max: Max Bracket of ha041_w4_4_

Mean	SD	Min	Max	Obs
5,000.00	.	5,000.00	5,000.00	1

ha054_w3: Bank Load to Purchase Houses

	Freq.	%
1 Yes	415	3.89
2 No	10,239	95.87
999 Refused to Answer	26	0.24
Total	10,680	100.00

ha055_w3: Amount of Bank Load

Mean	SD	Min	Max	Obs
3,541.54	18,262.30	0.00	214,000.00	404

ha056_w3: Unpaid Interest

Mean	SD	Min	Max	Obs
2,074.86	10,282.78	0.00	100,000.00	350

ha057_w3: Monthly Mortgage Payment

Mean	SD	Min	Max	Obs
161.69	893.25	0.00	9,000.00	387

i000_w4: Which House You Reside

	Freq.	%
1 House 1	4,043	35.07
2 House 2	2,305	19.99
3 House 3	1,632	14.16
4 House 4	965	8.37
5 House 5	103	0.89
6 House 6	23	0.20
7 House 7	5	0.04
99 Not Any Above	2,452	21.27
Total	11,528	100.00

i000_w4_2: Urban or Rural

	Freq.	%
1 City/Town Central Area	728	29.69
2 Rural-Urban Fringe	330	13.46
3 Rural	1,370	55.87
4 Special Zone	24	0.98
Total	2,452	100.00

i000_w4_3: House Type

	Freq.	%
1 Unit Building	797	32.50
2 Detached House	942	38.42
3 Attached House	191	7.79
4 Siheyuan	60	2.45
5 Compound Occupied by Many Households	114	4.65
6 Work Shed	48	1.96
7 Haphazard Building	58	2.37
8 Other	242	9.87
Total	2,452	100.00

i000_w4_4_s1: Household Head and Spouse Own the House

	Freq.	%
0 Do Not Own	2,341	95.47
1 Own	111	4.53
Total	2,452	100.00

i000_w4_4_s2: Children and Children's Spouses Own the House

	Freq.	%
0 Do Not Own	1,097	44.74

	Freq.	%
2 Own	1,355	55.26
Total	2,452	100.00

i000_w4_4_s3: Siblings Own the House

	Freq.	%
0 Do Not Own	2,402	97.96
3 Own	50	2.04
Total	2,452	100.00

i000_w4_4_s4: Parents and Parents-in-Law Own the House

	Freq.	%
0 Do Not Own	2,396	97.72
4 Own	56	2.28
Total	2,452	100.00

i000_w4_4_s5: Grandchildren Own the House

	Freq.	%
0 Do Not Own	2,416	98.53
5 Own	36	1.47
Total	2,452	100.00

i000_w4_4_s6: Other Relatives Own the House

	Freq.	%
0 Do Not Own	2,416	98.53
6 Own	36	1.47
Total	2,452	100.00

i000_w4_4_s7: riends Own the House

	Freq.	%
0 Do Not Own	2,396	97.72
7 Own	56	2.28
Total	2,452	100.00

i000_w4_4_s8: Others Own the House

	Freq.	%
0 Do Not Own	1,862	75.94
8 Own	590	24.06
Total	2,452	100.00

i000_w4_4_s9: Own the House But do Not Have Ownership Certificate

	Freq.	%
0 Do Not Own	2,276	92.82

9 Own	176	7.18
Total	2,452	100.00

i000_w4_4_s999: Refused to Answer the House Ownership

	Freq.	%
0 No	2,434	99.27
999 Yes	18	0.73
Total	2,452	100.00

i000_w4_4_1: Percent Household Head and Spouse Own the House

Mean	SD	Min	Max	Obs
72.80	42.54	0.00	100.00	123

i000_w4_4_3: Percent Siblings Own the House

Mean	SD	Min	Max	Obs
96.22	14.26	20.00	100.00	50

i000_w4_4_6: Percent Other Relatives Own the House

Mean	SD	Min	Max	Obs
89.49	28.29	0.00	100.00	37

i000_w4_4_7: Percent Friends Own the House

Mean	SD	Min	Max	Obs
100.00	0.00	100.00	100.00	56

i000_w4_4_8: Percent Others Own the House

Mean	SD	Min	Max	Obs
85.93	34.68	0.00	100.00	590

i000_w4_5_s1: XChildName[1]/'s Spouse Owns the House

	Freq.	%
0 Do Not Own	708	52.25
1 Own	647	47.75
Total	1,355	100.00

i000_w4_5_s2: XChildName[2]/'s Spouse Owns the House

	Freq.	%
0 Do Not Own	956	70.55
2 Own	399	29.45

Total	1,355	100.00

i000_w4_5_s3: XChildName[3]/'s Spouse Owns the House

	Freq.	%
0 Do Not Own	1,158	85.46
3 Own	197	14.54
Total	1,355	100.00

i000_w4_5_s4: XChildName[4]/'s Spouse Owns the House

	Freq.	%
0 Do Not Own	1,247	92.03
4 Own	108	7.97
Total	1,355	100.00

i000_w4_5_s5: XChildName[5]/'s Spouse Owns the House

	Freq.	%
0 Do Not Own	1,294	95.50
5 Own	61	4.50
Total	1,355	100.00

i000_w4_5_s6: XChildName[6]/'s Spouse Owns the House

	Freq.	%
0 Do Not Own	1,330	98.15
6 Own	25	1.85
Total	1,355	100.00

i000_w4_5_s7: XChildName[7]/'s Spouse Owns the House

	Freq.	%
0 Do Not Own	1,343	99.11
7 Own	12	0.89
Total	1,355	100.00

i000_w4_5_s8: XChildName[8]/'s Spouse Owns the House

	Freq.	%
0 Do Not Own	1,350	99.63
8 Own	5	0.37
Total	1,355	100.00

i000_w4_5_s9: XChildName[9]/'s Spouse Owns the House

	Freq.	%
0 Do Not Own	1,355	100.00

Total	1,355	100.00

i000_w4_5_s10: XChildName[10]/'s Spouse Owns the House

	Freq.	%
0 Do Not Own	1,354	99.93
10 Own	1	0.07
Total	1,355	100.00

i000_w4_5_s11: XChildName[11]/'s Spouse Owns the House

	Freq.	%
0 Do Not Own	1,354	99.93
11 Own	1	0.07
Total	1,355	100.00

i000_w4_5_s12: XChildName[12]/'s Spouse Owns the House

	Freq.	%
0 Do Not Own	1,355	100.00
Total	1,355	100.00

i000_w4_5_s13: XChildName[13]/'s Spouse Owns the House

	Freq.	%
0 Do Not Own	1,355	100.00
Total	1,355	100.00

i000_w4_5_s14: XChildName[14]/'s Spouse Owns the House

	Freq.	%
0 Do Not Own	1,355	100.00
Total	1,355	100.00

i000_w4_5_s15: XChildName[15]/'s Spouse Owns the House

	Freq.	%
0 Do Not Own	1,355	100.00
Total	1,355	100.00

i000_w4_5_s16: XChildName[16]/'s Spouse Owns the House

	Freq.	%
0 Do Not Own	1,355	100.00
Total	1,355	100.00

i000_w4_5_s99: None of the Above Owns the House

	Freq.	%
0 No	1,333	98.38
99 Yes	22	1.62
Total	1,355	100.00

i000_w4_6_1_: Percent XChildName[1]/'s Spouse Owns the House

Mean	SD	Min	Max	Obs
91.80	20.57	0.00	100.00	647

i000_w4_6_2_: Percent XChildName[2]/'s Spouse Owns the House

Mean	SD	Min	Max	Obs
88.64	22.97	0.00	100.00	399

i000_w4_6_3_: Percent XChildName[3]/'s Spouse Owns the House

Mean	SD	Min	Max	Obs
86.89	25.19	0.00	100.00	197

i000_w4_6_4_: Percent XChildName[4]/'s Spouse Owns the House

Mean	SD	Min	Max	Obs
87.23	27.54	0.00	100.00	108

i000_w4_6_5_: Percent XChildName[5]/'s Spouse Owns the House

Mean	SD	Min	Max	Obs
85.43	27.57	20.00	100.00	61

i000_w4_6_6_: Percent XChildName[6]/'s Spouse Owns the House

Mean	SD	Min	Max	Obs
78.26	31.91	0.00	100.00	25

i000_w4_6_7_: Percent XChildName[7]/'s Spouse Owns the House

Mean	SD	Min	Max	Obs
90.27	22.99	33.30	100.00	12

i000_w4_6_8_: Percent XChildName[8]/'s Spouse Owns the House

Mean	SD	Min	Max	Obs
76.66	32.50	33.30	100.00	5

i000_w4_6_10_: Percent XChildName[10]/'s Spouse Owns the House

Mean	SD	Min	Max	Obs
100.00	.	100.00	100.00	1

i000_w4_6_11_: Percent XChildName[11]/'s Spouse Owns the House

Mean	SD	Min	Max	Obs
70.00	.	70.00	70.00	1

i000_w4_7_s1: XConParName[1] Owns the House

	Freq.	%
0 Do Not Own	44	78.57
1 Own	12	21.43
Total	56	100.00

i000_w4_7_s2: XConParName[2] Owns the House

	Freq.	%
0 Do Not Own	44	78.57
2 Own	12	21.43
Total	56	100.00

i000_w4_7_s3: XConParName[3] Owns the House

	Freq.	%
0 Do Not Own	56	100.00
Total	56	100.00

i000_w4_7_s4: XConParName[4] Owns the House

	Freq.	%
0 Do Not Own	56	100.00
Total	56	100.00

i000_w4_7_s5: XConParName[5] Owns the House

	Freq.	%
0 Do Not Own	42	75.00
5 Own	14	25.00
Total	56	100.00

i000_w4_7_s6: XConParName[6] Owns the House

	Freq.	%
0 Do Not Own	47	83.93
6 Own	9	16.07

	Freq.	%
Total	56	100.00

i000_w4_7_s7: XConParName[7] Owns the House

	Freq.	%
0 Do Not Own	56	100.00
Total	56	100.00

i000_w4_7_s8: XConParName[8] Owns the House

	Freq.	%
0 Do Not Own	56	100.00
Total	56	100.00

i000_w4_7_s99: None of the Above Owns the House

	Freq.	%
0 No	38	67.86
99 Yes	18	32.14
Total	56	100.00

i000_w4_8_1_: Percent XConParName[1] Owns the House

Mean	SD	Min	Max	Obs
87.50	22.61	50.00	100.00	12

i000_w4_8_2_: Percent XConParName[2] Owns the House

Mean	SD	Min	Max	Obs
79.17	25.75	50.00	100.00	12

i000_w4_8_5_: Percent XConParName[5] Owns the House

Mean	SD	Min	Max	Obs
78.57	25.68	50.00	100.00	14

i000_w4_8_6_: Percent XConParName[6] Owns the House

Mean	SD	Min	Max	Obs
66.67	25.00	50.00	100.00	9

i000_w4_9_s1: XChildName[1]'s Child(Grandchild) Owns the House

	Freq.	%
0 Do Not Own	23	63.89
1 Own	13	36.11
Total	36	100.00

i000_w4_9_s2: XChildName[2]'s Child(Grandchild) Owns the House

	Freq.	%
0 Do Not Own	24	66.67
2 Own	12	33.33
Total	36	100.00

i000_w4_9_s3: XChildName[3]'s Child(Grandchild) Owns the House

	Freq.	%
0 Do Not Own	29	80.56
3 Own	7	19.44
Total	36	100.00

i000_w4_9_s4: XChildName[4]'s Child(Grandchild) Owns the House

	Freq.	%
0 Do Not Own	35	97.22
4 Own	1	2.78
Total	36	100.00

i000_w4_9_s5: XChildName[5]'s Child(Grandchild) Owns the House

	Freq.	%
0 Do Not Own	34	94.44
5 Own	2	5.56
Total	36	100.00

i000_w4_9_s6: XChildName[6]'s Child(Grandchild) Owns the House

	Freq.	%
0 Do Not Own	35	97.22
6 Own	1	2.78
Total	36	100.00

i000_w4_9_s7: XChildName[7]'s Child(Grandchild) Owns the House

	Freq.	%
0 Do Not Own	36	100.00
Total	36	100.00

i000_w4_9_s8: XChildName[8]'s Child(Grandchild) Owns the House

	Freq.	%
0 Do Not Own	36	100.00
Total	36	100.00

i000_w4_9_s9: XChildName[9]'s Child(Grandchild) Owns the House

	Freq.	%
0 Do Not Own	36	100.00
Total	36	100.00

i000_w4_9_s10: XChildName[10]'s Child(Grandchild) Owns the House

	Freq.	%
0 Do Not Own	36	100.00
Total	36	100.00

i000_w4_9_s11: XChildName[11]'s Child(Grandchild) Owns the House

	Freq.	%
0 Do Not Own	36	100.00
Total	36	100.00

i000_w4_9_s12: XChildName[12]'s Child(Grandchild) Owns the House

	Freq.	%
0 Do Not Own	36	100.00
Total	36	100.00

i000_w4_9_s13: XChildName[13]'s Child(Grandchild) Owns the House

	Freq.	%
0 Do Not Own	36	100.00
Total	36	100.00

i000_w4_9_s14: XChildName[14]'s Child(Grandchild) Owns the House

	Freq.	%
0 Do Not Own	36	100.00
Total	36	100.00

i000_w4_9_s15: XChildName[15]'s Child(Grandchild) Owns the House

	Freq.	%
0 Do Not Own	36	100.00
Total	36	100.00

i000_w4_9_s16: XChildName[16]'s Child(Grandchild) Owns the House

	Freq.	%
0 Do Not Own	36	100.00
Total	36	100.00

i000_w4_10_1_: Percent XChildName[1]'s Child(Grandchild) Owns the House

Mean	SD	Min	Max	Obs
92.31	18.78	50.00	100.00	13

i000_w4_10_2_: Percent XChildName[2]'s Child(Grandchild) Owns the House

Mean	SD	Min	Max	Obs
95.00	17.32	40.00	100.00	12

i000_w4_10_3_: Percent XChildName[3]'s Child(Grandchild) Owns the House

Mean	SD	Min	Max	Obs
100.00	0.00	100.00	100.00	7

i000_w4_10_4_: Percent XChildName[4]'s Child(Grandchild) Owns the House

Mean	SD	Min	Max	Obs
100.00	.	100.00	100.00	1

i000_w4_10_5_: Percent XChildName[5]'s Child(Grandchild) Owns the House

Mean	SD	Min	Max	Obs
100.00	0.00	100.00	100.00	2

i000_w4_10_6_: Percent XChildName[6]'s Child(Grandchild) Owns the House

Mean	SD	Min	Max	Obs
20.00	.	20.00	20.00	1

i000_w4_11: Usable House Size

Mean	SD	Min	Max	Obs
111.46	113.83	0.00	2,150.00	2,165

i000_w4_12: House Size

Mean	SD	Min	Max	Obs
122.84	215.34	0.00	3,000.00	287

i000_w4_15_1: Total Value for the House

Mean	SD	Min	Max	Obs
5,414.09	54,510.50	0.00	1,224,000.00	1,287

i000_w4_15_2: Unit Value for the House

Mean	SD	Min	Max	Obs
1,146.46	2,495.32	0.00	16,000.00	249

i000_w4_15_min: Min Bracket of i000_w4_15

Mean	SD	Min	Max	Obs
4,866.67	4,398.35	1,000.00	15,000.00	45

i000_w4_15_max: Max Bracket of i000_w4_15

Mean	SD	Min	Max	Obs
3,566.37	3,774.62	1,000.00	15,000.00	113

i000_w4_16: Year Get the House

Mean	SD	Min	Max	Obs
2,003.97	13.32	1,900.00	2,018.00	1,542

i000_w4_17: Money Paid to Get the House

Mean	SD	Min	Max	Obs
3,476.00	31,426.27	0.00	600,000.00	1,413

i000_w4_13: The House's Rent

Mean	SD	Min	Max	Obs
257.54	971.10	0.00	30,000.00	2,263

i000_w4_13_min: Min Bracket of i000_w4_13

Mean	SD	Min	Max	Obs
928.57	569.25	500.00	2,000.00	42

i000_w4_13_max: Max Bracket of i000_w4_13

Mean	SD	Min	Max	Obs
964.71	1,008.26	500.00	5,000.00	85

i000_w4_14: The House's Market Rent

Mean	SD	Min	Max	Obs
984.10	2,783.22	0.00	70,000.00	1,657

i000_w4_14_min: Min Bracket of i000_w4_14

Mean	SD	Min	Max	Obs
1,379.49	1,332.42	500.00	8,000.00	195

i000_w4_14_max: Max Bracket of i000_w4_14

Mean	SD	Min	Max	Obs
1,013.18	1,284.20	500.00	8,000.00	607

i002: The Housing Land Area

Mean	SD	Min	Max	Obs
254.30	9,373.84	0.00	999,999.00	11,527

i003: Resident Used for Business

	Freq.	%
1 Yes	905	7.85
2 No	10,622	92.15
Total	11,527	100.00

i004: Building Structure

	Freq.	%
1 Concrete and Steel/Bricks and Wood	9,980	86.58
2 Adobe	920	7.98
3 Wood/Thatched	47	0.41
4 Cave Dwelling	77	0.67
5 Mongolian Yurt/Woolen Felt/Tent	13	0.11
6 Stone	258	2.24
7 Other	232	2.01
Total	11,527	100.00

i005: Time House Built

Mean	SD	Min	Max	Obs
1,998.87	14.06	1,900.00	2,018.00	10,315

i005_1: Time Interval House Built

	Freq.	%
1 0-5 Years	93	7.67
2 5-10 Years	186	15.35
3 10-20 Years	330	27.23
4 20-30 Years	247	20.38
5 30-40 Years	143	11.80
6 More Than 40 Years	213	17.57
Total	1,212	100.00

i006: One Stroy or Multi-level Building

	Freq.	%
1 One-stroy Building	5,213	45.22
2 Common Multi-story Building	2,920	25.33
3 Self-contained Multi-story Building	3,394	29.44
Total	11,527	100.00

i007: The Stroy is Independent or Compound

	Freq.	%
1 Independent Stroy	4,706	90.27
2 Compound	507	9.73
Total	5,213	100.00

i008: Which Story

Mean	SD	Min	Max	Obs
3.90	4.08	0.00	33.00	2,911

i009: Elevator

	Freq.	%
1 Yes	465	8.37
2 No	5,092	91.63
Total	5,557	100.00

i010_w4: Handicapped Facilities

	Freq.	%
1 Yes	1,235	10.71
2 No	6,702	58.14
3 Do Not Need	3,590	31.14
Total	11,527	100.00

i011: Steps to Main Entrance of the House

	Freq.	%
1 0 Step	2,633	39.29
2 1-5 Steps	1,945	29.02
3 6-15 Steps	445	6.64
4 16-25 Steps	378	5.64
5 More Than 25 Steps	1,301	19.41
Total	6,702	100.00

i012_1: Bedrooms

Mean	SD	Min	Max	Obs
3.09	2.30	0.00	52.00	11,513

i012_2: Livingrooms

Mean	SD	Min	Max	Obs
1.17	0.84	0.00	17.00	11,515

i012_3: Toilets

Mean	SD	Min	Max	Obs
1.12	1.23	0.00	50.00	11,519

i012_4: Kitchens

Mean	SD	Min	Max	Obs
1.00	0.77	0.00	50.00	11,519

i012_5: Balcony

Mean	SD	Min	Max	Obs
0.53	0.81	0.00	20.00	11,520

i013: Distance from the Nearest Toilet to House

Mean	SD	Min	Max	Obs
4,268,251.80	206592164.70	0.00	1.00e+10	2,343

i014: Type of Toilet

	Freq.	%
1 Without a Seat	7,855	68.15
2 With a Seat	3,671	31.85
Total	11,526	100.00

i015: Flushable Toilet

	Freq.	%
1 Yes	3,595	45.77
2 No	4,260	54.23
Total	7,855	100.00

i016: Electricity

	Freq.	%
1 Yes	11,399	98.90
2 No	127	1.10
Total	11,526	100.00

i017: Running Water

	Freq.	%
1 Yes	9,392	81.49
2 No	2,134	18.51
Total	11,526	100.00

i018: In-house Shower

	Freq.	%
1 Hot Water Provided	218	1.89
2 Water Heater Installed by the Household	7,206	62.52
3 No	4,102	35.59
Total	11,526	100.00

i019: Coal Gas or Natural Gas Supply

	Freq.	%
1 Yes	2,666	23.13
2 No	8,860	76.87
Total	11,526	100.00

i020: Heating

	Freq.	%
1 Yes	1,622	14.07
2 No	9,904	85.93
Total	11,526	100.00

i021_w4: Heating Energy Source

	Freq.	%
1 Solar	6	0.37
2 Coal	554	34.16
3 Natural Gas	193	11.90
4 Liquefied Petroleum Gas	5	0.31
5 Electric	126	7.77
6 Crop Residue/Wood Burning	19	1.17
7 Other	36	2.22
8 Heating Provided	683	42.11
Total	1,622	100.00

i022_w4: Main Source of Cooking Fuel

	Freq.	%
1 Coal	548	4.75
2 Natural Gas	3,049	26.45
3 Marsh Gas	54	0.47
4 Liquefied Petroleum Gas	2,037	17.67
5 Electric	2,647	22.97
6 Crop Residue/Wood Burning	2,925	25.38
7 Other	177	1.54

8 Do Not Cook	89	0.77
Total	11,526	100.00

i023: Telephone

	Freq.	%
1 Yes	2,239	19.43
2 No	9,287	80.57
Total	11,526	100.00

i024: Broad-band Internet Connection

	Freq.	%
1 Yes	5,187	45.00
2 No	6,339	55.00
Total	11,526	100.00

i027_w3: Air Cleaner

	Freq.	%
1 Yes	296	2.57
2 No	11,230	97.43
Total	11,526	100.00

i025: Clear and Tidy in this Household

	Freq.	%
1 Excellent	1,343	11.65
2 Very Clear	2,350	20.39
3 Clear	3,318	28.79
4 Fair	2,846	24.69
5 Poor	736	6.39
6 Not Applicable	932	8.09
Total	11,525	100.00

i026: Temperature in this Household

	Freq.	%
1 Very Hot	105	0.91
2 Hot	1,294	11.23
3 Bearable	8,923	77.43
4 Cold	244	2.12
5 Very Cold	11	0.10
6 Not Applicable	947	8.22
Total	11,524	100.00

zsize_1_: House Size for Loaded House[1]

Mean	SD	Min	Max	Obs
122.51	80.56	1.00	1,400.00	7,973

zsize_2_: House Size for Loaded House[2]

Mean	SD	Min	Max	Obs
127.80	88.70	0.00	1,600.00	7,096

zsize_3_: House Size for Loaded House[3]

Mean	SD	Min	Max	Obs
130.58	90.06	0.00	1,500.00	4,918

zsize_4_: House Size for Loaded House[4]

Mean	SD	Min	Max	Obs
136.11	93.03	20.00	867.10	489

zsize_5_: House Size for Loaded House[5]

Mean	SD	Min	Max	Obs
130.46	96.36	30.00	520.00	76

zsize_6_: House Size for Loaded House[6]

Mean	SD	Min	Max	Obs
189.55	237.02	72.00	900.00	11

zsize_7_: House Size for Loaded House[7]

Mean	SD	Min	Max	Obs
75.00	21.21	60.00	90.00	2

ztimebuy_1_: Time Loaded House[1] Bought

Mean	SD	Min	Max	Obs
1,994.15	13.54	1,900.00	2,015.00	8,043

ztimebuy_2_: Time Loaded House[2] Bought

Mean	SD	Min	Max	Obs
1,995.35	12.95	1,900.00	2,015.00	7,161

ztimebuy_3_: Time Loaded House[3] Bought

Mean	SD	Min	Max	Obs
1,995.39	12.61	1,900.00	2,015.00	4,934

ztimebuy_4_: Time Loaded House[4] Bought

Mean	SD	Min	Max	Obs
1,998.28	10.76	1,943.00	2,015.00	489

ztimebuy_5_: Time Loaded House[5] Bought

Mean	SD	Min	Max	Obs
1,997.30	11.14	1,965.00	2,013.00	76

ztimebuy_6_: Time Loaded House[6] Bought

Mean	SD	Min	Max	Obs
1,997.36	10.17	1,981.00	2,012.00	11

ztimebuy_7_: Time Loaded House[7] Bought

Mean	SD	Min	Max	Obs
1,993.50	10.61	1,986.00	2,001.00	2

ztimeprice_1_: Price (10K Yuan) Loaded House[1] Bought

Mean	SD	Min	Max	Obs
9.42	39.04	0.00	800.00	3,112

ztimeprice_2_: Price (10K Yuan) Loaded House[2] Bought

Mean	SD	Min	Max	Obs
9.20	37.49	0.00	800.00	6,022

ztimeprice_3_: Price (10K Yuan) Loaded House[3] Bought

Mean	SD	Min	Max	Obs
8.35	32.22	0.00	800.00	4,244

ztimeprice_4_: Price (10K Yuan) Loaded House[4] Bought

Mean	SD	Min	Max	Obs
9.77	27.76	0.00	500.00	434

ztimeprice_5_: Price (10K Yuan) Loaded House[5] Bought

Mean	SD	Min	Max	Obs
8.84	19.51	0.00	150.00	69

ztimeprice_6_: Price (10K Yuan) Loaded House[6] Bought

Mean	SD	Min	Max	Obs
13.21	29.01	0.00	100.00	11

ztimeprice_7_: Price (10K Yuan) Loaded House[7] Bought

Mean	SD	Min	Max	Obs
3.30	3.82	0.60	6.00	2

zvaluelasttime_1_: Value (10K Yuan) at ZiwTime for Loaded House[1]

Mean	SD	Min	Max	Obs
14.42	26.10	0.00	720.00	6,943

zvaluelasttime_2_: Value (10K Yuan) at ZiwTime for Loaded House[2]

Mean	SD	Min	Max	Obs
18.81	33.94	0.01	600.00	5,298

zvaluelasttime_3_: Value (10K Yuan) at ZiwTime for Loaded House[3]

Mean	SD	Min	Max	Obs
19.29	36.90	0.01	653.00	3,684

zvaluelasttime_4_: Value (10K Yuan) at ZiwTime for Loaded House[4]

Mean	SD	Min	Max	Obs
28.68	77.30	0.05	1,260.00	391

zvaluelasttime_5_: Value (10K Yuan) at ZiwTime for Loaded House[5]

Mean	SD	Min	Max	Obs
25.74	48.96	0.03	360.00	68

zvaluelasttime_6_: Value (10K Yuan) at ZiwTime for Loaded House[6]

Mean	SD	Min	Max	Obs
24.90	26.95	1.00	80.00	10

zvaluelasttime_7_: Value (10K Yuan) at ZiwTime for Loaded House[7]

Mean	SD	Min	Max	Obs
22.50	10.61	15.00	30.00	2

zha000_w4_1_1_: Have Ownership Information at ZiwTime for Loaded House[1]

	Freq.	%
0 No	4,283	53.25
1 Yes	3,760	46.75
Total	8,043	100.00

zha000_w4_1_2_: Have Ownership Information at ZiwTime for Loaded House[2]

	Freq.	%
0 No	3,121	43.58
1 Yes	4,040	56.42
Total	7,161	100.00

zha000_w4_1_3_: Have Ownership Information at ZiwTime for Loaded House[3]

	Freq.	%
0 No	2,137	43.31
1 Yes	2,797	56.69
Total	4,934	100.00

zha000_w4_1_4_: Have Ownership Information at ZiwTime for Loaded House[4]

	Freq.	%
0 No	214	43.76
1 Yes	275	56.24
Total	489	100.00

zha000_w4_1_5_: Have Ownership Information at ZiwTime for Loaded House[5]

	Freq.	%
0 No	28	36.84
1 Yes	48	63.16
Total	76	100.00

zha000_w4_1_6_: Have Ownership Information at ZiwTime for Loaded House[6]

	Freq.	%
0 No	4	36.36
1 Yes	7	63.64
Total	11	100.00

zha000_w4_1_7_: Have Ownership Information at ZiwTime for Loaded House[7]

	Freq.	%
0 No	1	50.00
1 Yes	1	50.00
Total	2	100.00

zhousenum: Loaded House Number

Mean	SD	Min	Max	Obs
1.80	1.36	0.00	7.00	11,533

ziwtime: ZIWTime

A String Variable	
Obs:	11,445

versionID: Version ID

A String Variable	
Obs:	11,535

This page intentionally left blank

MODULE AUX1

Sample Information

ID: Individual ID

A String Variable	
Obs:	20,813

householdID: Household ID

A String Variable	
Obs:	20,813

communityID: Community ID

A String Variable	
Obs:	20,813

died: Whether Individual Died

	Freq.	%
0 Alive	19,816	95.21
1 Died	997	4.79
Total	20,813	100.00

crosssection: Whether Cross-Section Sample

	Freq.	%
0 No	2,843	13.66
1 Yes	17,970	86.34
Total	20,813	100.00

iyear: Interview Year

A String Variable	
Obs:	20,813

imonth: Interview Month

A String Variable	
Obs:	20,813

versionID: Version ID

A String Variable	
Obs:	20,813

MODULE AUX2

Sampling Weights

ID: Individual ID

A String Variable	
Obs:	17,970

householdID: Household ID

A String Variable	
Obs:	17,970

communityID: Community ID

A String Variable	
Obs:	17,970

HH_weight: Household Base Weight

Mean	SD	Min	Max	Obs
25,428.54	24,149.17	6,466.32	1,136,124.50	17,970

HH_weight_ad1: Household Weight with Household Response Adjustment

Mean	SD	Min	Max	Obs
28,787.69	40,618.57	6,774.24	2,434,552.50	17,970

INDV_weight: Individual Base Weight

Mean	SD	Min	Max	Obs
27,653.70	26,800.91	6,583.96	1,178,061.50	17,970

INDV_weight_ad2: Individual Weight with Household and Individual Response Ad-

justment

Mean	SD	Min	Max	Obs
32,437.31	33,738.78	7,370.20	1,521,872.08	17,970

versionID: Version ID

A String Variable	
Obs:	17,970
