# Supplementary material for: The impact of pension insurance types on the health of older adults in China: a study based on the 2018 CHARLS data
Source: Front Public Health. 2023 Jun 2;11:1180024. doi: 10.3389/fpubh.2023.1180024 (PMC10272461; doi:10.3389/fpubh.2023.1180024)
Supplement: Supplementary file 1 [file Data_Sheet_1.ZIP › CHARLS_2018_Household_Questionnaire.pdf]

China Health and Retirement Longitudinal Study Wave 4 (2018) Questionnaire

中国健康与养老追踪调查 2018 年追访问卷

VersionID: 20200914

National School of Development
Institute of Social Science Survey
Peking University

Contents 目 录

CV Cover Screen 过滤问卷 **1**

B Demographic Backgrounds 基本信息 **5**

C Family 家庭 **19**

C1 Parent, Children and Sibling Information 父母、子女以及兄弟姐妹信息 . 20

CA Parent Information 父母信息 20

CB Children Information 子女信息 30

CC Sibling Information 兄弟姐妹信息 41

A Household Member Information 家户成员信息 47

C2 Time Transfer and Transfers 家庭交往与经济帮助 49

CD Time Transfer 与父母、子女间的交往 49

CE Transfers 家庭得到及提供的经济帮助 53

CF Time Spent Providing Care 提供照料时间 59

D Health Status and Functioning 健康状况和功能 **63**

DA Health Status 健康状况 64

PART I General Health Status and Disease History 第一部分：一般健康状况和疾病史 64

PART II Lifestyle and Health Behaviors 第二部分：生活方式和健康行为 81

DB Functional Limitations and Helpers 身体功能障碍以及辅助者 89

DC Cognition and Depression 认知和抑郁 **101**

SECTION INTRO Introducing Respondent Interview 介绍受访者问卷 101

SECTION MMSE Mini Mental State Exam 简易精神状态检查 101

SECTION HT HRS Telephone Interview for Cognitive Status (TICS) 认知状况电话访问量表 107

SECTION WR Word Recall 字词回忆 108

SECTION RF Retrieval Fluency 口语流畅性：“动物类别” 111

SECTION CSI-D Community Screening Instrument for Dementia Interviewee Part 简明社区痴呆筛查量表-认知功能部分 114

SECTION CESD Depression 抑郁量表 114

SECTION SAT Satisfaction 满意度部分 117

SECTION DR Delayed Recall 延迟回忆 118

SECTION NS Number Series 数列题 . . . 119
SECTION WRE Wordlist Recognition 词组辨识 . . . 125
END_DC Interview Observation DC 部分访员观察 . . . 129

DD Informants Information 知情人信息收集 131

SECTION INTRO IF Introducing Informant Interview 介绍知情人问卷 . . . 133
SECTION DEMOGRAPHICS_IF Demographics of Informant 知情人基本信息 134
SECTION JORM IQCODE The Jorm Informant Quesionnaire on Cognitive Decline in the Elderly 老年人认知功能减退知情者问卷 . . . 135
SECTION BLESSED Blessed Dementia Scale Part II Blessed 痴呆量表第二部分 . . . 143
SECTION CSI-D Community Screening Instrument for Dementia Informant Part 简明社区痴呆筛查量表知情人部分 . . . 145
END_DD Interview Observation DD 部分访员观察 . . . 146

E Health Care and Insurance 医疗保健与保险 149

PART I Medical Insurance 医疗保险 . . . 149
PART II Health Care Costs and Utilization 医疗成本与使用情况 . . . 152

F Work and Retirement 工作和退休 165

FA Work Status 工作概况 . . . 167
Update Missing Work Status in the Last Visit 上轮工作状态补询子模块 . 173
Job Switch 两轮工作状态转换确认子模块 . . . 175
FC Self-Employed Agricultural Work 农业自雇 . . . 182
FD Employed 受雇 . . . 183
FE Questions About Labor Supply 劳动力供给 . . . 190
FF Questions About Wages 受雇工作的工资问题 . . . 191
FG Fringe Benefits 单位福利 . . . 194
FH Non-Farm Self-Employed and Unpaid Work for Family Business 非农自雇和为家庭经营活动帮工 . . . 195
FJ Side Job (Employed or Self-employed) 非主要职业（受雇或自雇） . . . 199
FK Unemployment and Job Search Activities 失业，求职经历 . . . 200
FL Last Job 最近一份工作 . . . 201
FM Retirement 退休与退职 . . . 206

FN Pension 养老金 217

PART 1 Pension for Public Servants, Public Institution Employees, and Basic Pension for Enterprise Employees 政府机关、事业单位养老保险（退休金）及职工基本养老保险 . . . 217
PART 2 Supplementary Pension Insurance (Annuity) 补充养老保险（年金） 222
PART 3 Urban and Rural Resident Pension, New Rural Resident Pension and Urban Resident Pension 城乡居民养老保险、新型农村养老保险及城镇居民养老保险 . . . 226
PART 4 Pension for Land-Expropriated Farmers 征地养老保险（失地农民养老保险/被征地农民养老保险） . . . 231
PART 5 Life Insurance 人寿保险 . . . 233
PART 6 Commercial Pension Insurance（Exclude Life Insurance）商业养老保险（人寿保险除外） . . . 235
PART 7 Other Pension 其他养老保险 . . . 238

G&H Income, Expenditures and Assets 收入、支出与资产 245

G2 Household Income and Expenditures 家户收入与支出 . . . 245
PART 1 Household Wage Income and Individual-based Transfers 家户工资收入和个人获得的转移收入 . . . 245
PART 2 Household Agricultural Income and Expenditure 家户农业收入与支出 . . . 253
PART 3 Self-Employed Activities 个体经营或开办私营企业 . . . 257
PART 4 Household Public Transfer Income 家户公共转移支付收入 . . . 258
PART 5 Household Living Expenditure 家户生活支出 . . . 260
HA Household Assets 家户资产 . . . 263
PART 1 Land 土地 . . . 263
PART 2 Equipments, Consumption Durables, and Valuables 家用设备、耐用消费品和其他贵重物品 . . . 266
HB Individual Assets 个人资产 . . . 268
PART 1 Financial Assets 金融资产 . . . 268
PART 2 Debts 债务 . . . 272

HA&I House Property and Housing Characteristics 房产和住房情况 275

HA House Property 房产 . . . 275
I Housing Characteristics 住房情况 . . . 291

This page intentionally left blank

CV Cover Screen 过滤问卷

CV_HType CAPI preloads type of respondent household CAPI 加载受访家户类型

1. Two-person household previously interviewed 双人已访家户 → CAPI generates XHType = 1 CAPI 生成 XHType = 1
2. One-person household previously interviewed 单人已访家户 → CAPI generates XHType = 1 CAPI 生成 XHType = 1
3. New household 备访家户 → CAPI generates XHType = 2 CAPI 生成 XHType = 2

[IWER: Respondent for cover screen must be [Name of Respondent 1] or [Name of Respondent 2]. In which case both respondents had died or can not answer questions due to deaf, mutism, mental illness, or dementia, another family member can answer 访员注意：回答过滤问卷的只能是 [受访者 1 姓名] 或 [受访者 2 姓名]，在他们均已去世或因聋哑、精神疾病或老年痴呆等原因无法接受访问的情况下，才可以让他们的家人代为回答过滤问卷]

CV001 Of people on the following list, who is answering the cover screen? 以下列表中，回答过滤问卷的是谁?

1. [Name of Respondent 1][受访者 1 姓名]
2. [Name of Respondent 2][受访者 2 姓名]
3. Informant 代理人，name 姓名 _____ (**CV001_1**)，relationship with [Name of respondent 1] 是 [受访者 1 姓名] 的什么人 _____ (**CV001_2**)

CV002 Is [Name of Respondent 1] still alive? [受访者 1 姓名] 是否健在?

[IWER: If [Name of Respondent 1] is who answers the cover screen, then do not ask, choose ”alive” directly 访员注意：如果回答过滤问卷的就是 [受访者 1 姓名]，无需提问该题，直接选择健在]

1. Alive 健在
2. Died 去世,Date of death was 去世时间是 _____ (**CV002_1**) Year 年 ___ (**CV002_2**) Month 月 ___ (**CV002_3**) Day 日

PROCEDURE 程序:

If CV_HType ≠ 1, Skip CV003 如果 CV_HType ≠ 1，跳过 CV003

CV003 Is [Name of Respondent 2] still alive? [受访者 2 姓名] 是否健在?

[IWER: If [Name of Respondent 2] is who answers the cover screen, then do not ask, choose “alive” directly 访员注意：如果回答过滤问卷的就是 [受访者 2 姓名]，无需提问该题，直接选择健在]

1. Alive 健在
2. Died 去世,Date of death was 去世时间是 _____ (**CV003_1**) Year 年 ___ (**CV003_2**) Month 月 ___ (**CV003_3**) Date 日

PROCEDURE 程序:

If CV_HType = 1, and CV002 = 1, and CV003 = 1, ask CV004 to CAPI Split Household 如果 CV_HType = 1，且 CV002 = 1，且 CV003 = 1，提问 CV004 至 CAPI 分户

If CV_HType = 1, and CV002 = 1, and CV003 = 2, Save [Name of Respondent 1] as [Main Respondent], Save XRType of [Main Respondent] as 1.REIW, Generates Exit and Death Cause Questionair for [Name of Respondent 2] and skip to CV006 then 如果 CV_HType = 1，且 CV002 = 1，且 CV003 = 2，将 [受访者 1 姓名] 存入 [主要受访者姓名]，[主要受访者姓名] 的 XRType存为 1.REIW，同时生成 [受访者 2 姓名] 的退出以及死因问卷，然后跳至 CV006

If CV_HType = 1, and CV002 = 2, and CV003 = 1, Save [Name of Respondent 2] as [Main Respondent], Save XRType of [Main Respondent] as 1. REIW, Generate Exit and Death Cause Questionaire for [Name of Respondent 1] and skip to CV006 then 如果 CV_HType = 1，且 CV002 = 2，且 CV003 = 1，将 [受访者 2 姓名] 存入 [主要受访者姓名]，[主要受访者姓名] 的 XRType 存为 1. REIW，同时生成 [受访者 1 姓名] 的退出以及死因问卷，然后跳至 CV006

If CV_HType = 1, and CV002 = 2, and CV003 = 2, Generate Exit and Death Cause Questionair for [Name of Respondent 1] and [Name of Respondent 2], and end Cover Screen then 如果 CV_HType = 1，且 CV002 = 2，且 CV003 = 2，生成 [受访者 1 姓名] 以及 [受访者 2 姓名] 的退出以及死因问卷，然后结束过滤问卷

If CV_HType = 2, and CV002 = 1, Save [Name of Respondent 2] as [Main Respondent], Save XRType of [Main Respondent] as 1. REIW, and skip to CV006 如果 CV_HType = 2，且 CV002 = 1，将 [受访者 1 姓名] 存入 [主要受访者姓名]，[主要受访者姓名] 的 XRTyp 存为 1. REIW，然后跳至 CV006

If CV_HType = 2, and CV002 = 2, Generate Exit and Death Cause Questionaire for [Name of Respondent 1], and end Cover Screen then 如果 CV_HType = 2，且 CV002 = 2，生成 [受访者 1 姓名] 的退出以及死因问卷，然后结束过滤问卷

If CV_HType = 3, and CV002 = 1, Save [Name of Respondent 2] as [Main Respondent], and skip to HH_MRBirth1 then 如果 CV_HType = 3，且 CV002 = 1，将 [受访者 1 姓名] 存入 [主要受访者姓名]，然后跳至 HH_MRBirth1

If CV_HType = 3, and CV002 = 2, end the interview 如果 CV_HType = 3，且 CV002 = 2，结束整个访问

CV004 Are [Name of Respondent 1] and [Name of Respondent 2] still in a marital relationship or living together as married? [受访者 1 姓名] 与 [受访者 2 姓名] 目前是否仍然是夫妻或同居关系？

1. [Name of Respondent 1] and [Name of Respondent 2] are still in marital relationship or living together as married [受访者 1 姓名] 与 [受访者 2 姓名] 当前仍是夫妻或同居关系 → CAPI save [Name of Respondent 1] as [Main Respondent], XRType of [Main Respondent] as 1. REIW, Save [Name of Respondent 2] as [MR’s spouse], XRType of [Main Respondent] as 1. REIW, skip to CAPI generates personal questionaire CAPI 将 [受访者 1 姓名] 存为 [主要受访者姓名]，[主要受访者姓名] 的 XRType 存为 1. REIW，将 [受访者 2 姓名] 存为 [配偶姓名]，[配偶姓名] 的 XRType 存为 1. REIW，然后跳至 CAPI 生成个人问卷
2. [Name of Respondent 1] and [Name of Respondent 2] have divorced [受访者 1 姓名]

与 [受访者 2 姓名] 已经离婚了

3. [Name of Respondent 1] and [Name of Respondent 2] is living apart for a while, would not live as a couple in a foreseeable future [受访者 1 姓名] 与 [受访者 2 姓名] 已经长期分居，且预期未来不会再作为配偶共同生活

PROCEDURE 程序:

If CV001 = 3, ask CV005 如果 CV001 = 3，提问 CV005

CV005 You are more familiar with [Name of Respondent 1] or [Name of Respondent 2]? 对于 [受访者 1 姓名] 和 [受访者 2 姓名]，您更熟悉谁的情况?

1. [Name of Respondent 1] [受访者 1 姓名]
2. [Name of Respondent 2] [受访者 2 姓名]

CAPI Split Household CAPI 分户：Split the divorced household into two households 将离婚家户拆分成两个家户

Respondent of the Cover Screen（if CV001 ≠ 3）or CV005（if CV001 = 3）take the original household ID, save the name of the respondent into [Main Respondent name], save the XRType of [Main Respondent name] as 为 1. REIW. Save the name of the other respondent as [Name of Divorced Spouse], and skip to CV006 过滤问卷受访者（如果 CV001 ≠ 3）或 CV005（如果 CV001 = 3）继承原来的家户 ID，其姓名存入 [主要受访者姓名]，[主要受访者姓名] 的 XRType 存为 1. REIW，另外一个人的姓名存为 [离异配偶姓名]，然后跳至 CV006

Generate a new household as a one-person household previously interviewed for this divorced respondent 另外一个人以单人已访家户的类型生成另外一个家户

HH_MRBirth1 According to the information of previously interviewed, the birthdate of the [Main Respondent] is [pre-loaded Birthdate of Respondent] 根据之前我们访问获得的信息，[主要受访者姓名] 的出生日期为 [加载备访受访者出生日期]，对吗?

1. Right 对 → Skip HH_MRBirth2 跳过 HH_MRBirth2
2. Not Right 不对

HH_MRBirth2 The birthdate of [Main Respondent name] is [主要受访者姓名] 的出生日期?______

(HH_MRBirth2_1) Year 年 ___ **(HH_MRBirth2_2)** Month 月 ___ **(HH_MRBirth2_3)** Date 日

CAPI Determine the respondent is age-eligible or not CAPI 确定是否适龄

CAPI Based on [pre-loaded Birthdate of Respondent] and the refreshed HH_MRBirth2, determine the respondent is age-eligible or not. Standard is whether the birthday is early or equal to 1972/7/1 CAPI 根据 [加载备访受访者出生日期] 以及可能的更新 HH_MRBirth2，确定 [主要受访者姓名] 是否适龄，判断标准为出生日期早于或等于 1972 年 7 月 1 日

If eligible, Save the Xrtype of [Main Respondent name] as 2. NEWIW, continue on ask CV006 如果适龄，[主要受访者姓名] 的 XRType 存为 2. NEWIW，继续询问 CV006

If not eligible, end the interview 如果不适龄，结束访问

CV006 What is [preload MR name]'s marital status? [主要受访者姓名] 当前的婚姻状态是?

1. Married with spouse present 已婚并与配偶共同居住 → Skip to CV008 跳至 CV008
2. Married but not living with spouse temporarily for reasons such as work 已婚，但因为工作等原因暂时没有跟配偶在一起居住 → Skip to CV008 跳至 CV008
3. Separated 分居，不再作为配偶共同生活
4. Divorced 离异
5. Widowed 丧偶
6. Never married 从未结婚

CV007 For [preload MR name] who is single, or separate in marital status, does [preload MR] have any partner living together as a spouse? [主要受访者姓名] 现在没有配偶，或者与已婚配偶分居且不再作为配偶共同生活，那么 [主要受访者姓名] 当前是否有伴侣以配偶身份共同生活?

1. Yes 有，Name 姓名 ______ **(CV007_1)** → Skip to CAPI Generates Spouse 跳至 CAPI 生成配偶
2. No 没有 → Skip to CAPI Generates Spouse 跳至 CAPI 生成配偶

CV008 What is the current spouse's name of [preload MR name]? [主要受访者姓名] 的配偶叫什么名字? ______

**CAPI Generates Spouse CAPI 生成配偶**
If CV006 = 1, 2, Save CV008 as [MR's spouse name], Save the XRType of [MR's Spouse name] as 2.NEWIW 如果 CV006 = 1, 2，将 CV008 存为 [配偶姓名]，[配偶姓名] 的 XRType 存为 2. NEWIW<br>If CV006 = 3, 4, 5, 6 and CV007 = 1, Save CV007_1 as [MR's spouse name], Save XRType of [MR's spouse name] as 2. NEWIW 如果 CV006 = 3, 4, 5, 6 且 CV007 = 1，将 CV007_1 存为 [配偶姓名]，[配偶姓名] 的 XRType 存为 2. NEWIW<br>Otherwise, Save [MR's spouse] as Null 否则，[配偶姓名] 为空

**CAPI Generates Personal Questionaire CAPI 生成个人问卷**
Generate Personal Questionaire for [preload MR's name], Generate MainR=1 for [preload MR's name] 生成 [主要受访者姓名] 的个人问卷，对 [主要受访者姓名] 生成变量 **MainR** = 1<br>If [MR's spouse's name] is not Null, generate Personal Questionaire for [MR's spouse's name] 如果 [配偶姓名] 不为空，生成 [配偶姓名] 的个人问卷

B Demographic Backgrounds 基本信息

CAPI: Ask [Name of Main Respondent] and [Name of Main Respondent's spouse] questions in this module. Write [Name of Respondent] in this questionnaire, unless ambiguity occurs 对 [主要受访者姓名] 以及 [配偶姓名] 询问该模块，在不引起歧义的情况下，问卷中统一写为 [受访者姓名]

PROCEDURE 程序:

If this is a reinterview, XRType = 1 and [ZFRgender] = 1, ask BA000_W2_3, then renew XRGender = BA000_W2_3 如果是回访受访者，即 XRType = 1，且 [ZFRgender] = 1，则询问 BA000_W2_3，然后更新 XRGender 为 BA000_W2_3

If this is a new interview, XRType = 2, ask BA000_W2_3, then renew XRGender = BA000_W2_3 如果是新受访者，即 XRType = 2，则询问 BA000_W2_3，然后更新 XRGender 为 BA000_W2_3

If not, XRGender=[ZRGender], then skip to the procedure before BA001 否则更新 XRGender 为 [ZRGender]，然后跳至 BA001 前的程序

BA000_W2_3 Interviewer record the Respondent's gender 访员记录受访者性别

1. Male 男
2. Female 女

PROCEDURE 程序:

If this is a reinterview, XRType = 1 and ZFRgender = 1, ask BA001 如果是回访受访者，即 XRType = 1，且 [ZFRzodiac] = 1，则询问 BA001

If this is a new interview, XRType = 2, ask BA001 如果是新受访者，即 XRType = 2，则询问 BA001

If not, skip to the procedure before BA004_W3 否则跳至 BA004_W3 前的程序

BA001 What is your Chinese Zodiac sign? 您的属相是?

1. Rat 鼠
2. Ox 牛
3. Tiger 虎
4. Rabbit 兔
5. Dragon 龙
6. Snake 蛇
7. Horse 马
8. Goat 羊
9. Monkey 猴
10. Rooster 鸡
11. Dog 狗
12. Pig 猪

PROCEDURE 程序:

If this is a reinterview, XRType = 1 and ZFRgender = = 1, ask BA004_W3 to BA003 如果是回访受访者，即 XRType = 1，且 [ZFRbirth] = 1，则询问 BA004_W3 至 BA003

If this is a new interview, XRType = 2, ask BA004_W3 to BA003 如果是新受访者，即 XRType = 2，则询问 BA004_W3 至 BA003

If not, skip to the procedure before BB000_W3. 否则，跳至 BB000_W3 前的程序

BA004_W3 What's your date of birth on ID card or Household register? 您身份证或户口本上登记的出生日期是?

1. _____ (**BA004_W3_1**) Year 年 ___ (**BA004_W3_2**) Month 月 ___ (**BA004_W3_3**) Day 日
2. Does not have Hukou or ID card 没有户口或者没有身份证 → Skip to BA002 跳至 BA002
3. Has Hukou or ID card, but refuses to answer the date of birth on ID card or Household register 有身份证或户口本，但是拒绝透露登记的出生日期 → Skip to BA002 跳至 BA002

[IWER: If the respondent doesn't remember the date of birth on ID card or Household register, remind him/her to find ID card or Household register to confirm the date. The year must be a number in the range [1900-2018]. Mark the year using four digits. Take down the month as its actual number. For example, write January as "1" not "01", December as "12" 访员注意：如果受访者不记得身份证或户口本上的出生日期，提醒他们找出证件以确定答案。年份填写范围 [1900-2018]，用 4 位数表示年，按照实际的月份填写月，例：1 月写作"1"，而不是"01"，12 月写作"12"]

BA005_W4 Is your actual date of birth the same as on ID card or Household register? 您的真实的出生日期是否与身份证或户口本上登记的出生日期一样?

1. Yes 一样 → Skip BA002 跳过 BA002
2. No 不一样

BA002 What's your actual date of birth? 您的真实的出生日期是? _____ (**BA002_1**) Year 年 _____ (**BA002_2**) Month 月 _____ (**BA002_3**) Day 日

[IWER: The year must be a number in the range [1900-2018]. Mark the year using four digits. Take down the month as its actual number. For example, write January as "1" not "01", December as "12". If does not remember month and day, fill "0" 访员注意：年份填写范围 [1900-2018]，用 4 位数表示年，按照实际的月份填写月，例：1 月写作"1"，而不是"01"，12 月写作"12"。如果记不住月份和日期，请填入"0"]

PROCEDURE 程序:

CAPI generates [actual date of birth] CAPI 生成 [真实出生日期]

If BA005_W4 = 1, then [actual date of birth] = BA004_W3_1 year BA004_W3_2 month BA004_W3_3 date 如果 BA005_W4 = 1，则 [真实出生日期] = BA004_W3_1 年 BA004_W3_2 月 BA004_W3_3 日

If BA005_W4 = 2 or BA004_W3 = 2, 3, then [actual date of birth]= BA002_1 year BA002_2 month BA002_3 date 如果 BA005_W4 = 2 或者 BA004_W3 = 2, 3，则 [真实出生日期] = BA002_1 年 BA002_2 月 BA002_3 日

BA003 Is your previous answer based on the solar or the lunar calendar? 您刚才回答的真实出生日期 [真实出生日期] 是公历（阳历）还是农历（阴历）?

1. Solar calendar 公历（阳历）
2. Lunar calendar 农历（阴历）

PROCEDURE 程序:

If this is a reinterview, XRType = 1 and [ZRpaddress] is missing, ask BB000_W3, then generate an updated address for the last wave [XRLpaddress] = BB000_W3 如果是回访受访者，即 XRType = 1，且上一期的居住地址信息 [ZRpaddress] 缺失，则询问 BB000_W3，之后生成更新过的上一期居住地址信息 [XRLpaddress] = BB000_W3

If this is a reinterview, XRType = 1, and [ZRpaddress] is not missing, generate an updated address for the last wave [XRLpaddress] = BB000_W3, then skip to BB001_W3 如果是回访受访者，即 XRType = 1，且上一期的居住地址信息 [ZRpaddress] 不缺失，生成更新过的上一期居住地址信息 [XRLpaddress] = BB000_W3，然后跳至 BB001_W3

If this is a new interview, XRType = 2, skip to BB001_W3 如果是新受访者，即 XRType = 2，则跳至 BB001_W3

BB000_W3 What's your address in [ZIWTime]? [ZIWTime] 时，您一般住在哪里?

[IWER: county/city includes conty-level administrative units/county-level city/disrtict 访员注意：县/市包括县级行政单位/县级市/区]

1. Domestic 国内:
   ______ (**BB000_W3_a_1**) province/city/county 省/市/县
   ______ (**BB000_W3_a_2**) township/village/neighborhood 乡/镇/街道/村/社区
2. Abroad 国外

BB000_W3_1 What was the type of your address BB000_W3 in [ZIWTime]? [ZIWTime] 时，您在居住地址 BB000_W3 居住的类型是?

1. Family housing 家庭住宅
2. Nursing home 养老院或其他养老机构
3. Hospital 医院
4. Other 其他，please specify 请注明 ______ （**BB000_W3_1_1**）

BB000_W3_2 Was your address, BB000_W3, in the village or city/town? [ZIWTime] 时，您在居住地址 BB000_W3 居住时主要生活在农村还是城市?

1. The center of city/town 城或镇中心区
2. Combination zone between urban and rural areas 城乡或镇乡结合区
3. Village 农村
4. Special area 特殊区域

BB001_W3 What's your address now? 您现在一般住在哪里?

[IWER: county/city includes conty-level administrative units/county-level city/disrtict 访员注意：县/市包括县级行政单位/县级市/区]

1. preload address in the last wave [XRLpaddress] 加载上期访问时的居住地址 [XRLpaddress]
2. Other 其他地方:
   ______ (**BB001_W3_a_1**) province/city/county 省/市/县
   ______ (**BB001_W3_a_2**) township/village/neighborhood 乡/镇/街道/村/社区
3. Abroad 国外

BB001_W3_1 What was the type of your address, BB001_W3? 您在居住地址 BB001_W3 居住的类型是?

1. Family housing 家庭住宅
2. Nursing home 养老院或其他养老机构
3. Hospital 医院
4. Other 其他，please specify 请注明 _____ （**BB001_W3_1_1**）

BB001_W3_2 Was your address BB001_W3 in the village or city/town? 您在居住地址 BB001_W3 居住时主要生活在农村还是城市?

1. The center of city/town 城或镇中心区
2. Combination zone between urban and rural areas 城乡或镇乡结合区
3. Village 农村
4. Special area 特殊区域

PROCEDURE 程序:

If this is a new interview, XRType = 2, ask BB001 to BB005 如果是新受访者，即 XRType = 2，则询问 BB001 至 BB005

If not, skip to BB005_W3_1 否则，跳至 BB005_W3_1

BB001 Where were you born? 您的出生地是哪里?

[IWER: county/city includes conty-level administrative units/county-level city/disrtict 访员注意：县/市包括县级行政单位/县级市/区]

1. preload current address BB001_W3 加载当前居住地址 BB001_W3
2. Another village/neighborhood in current address's (BB001_W3) county/city/district BB001_W3 所在县/市/区的其他村/社区
   _____ (**BB001_1**) township 乡/镇/街道
   _____ (**BB001_2**) village/neighborhood 村/社区
3. Other 其它:
   _____ (**BB001_3**) province/city/county/district 省/市/县
   _____ (**BB001_4**) township/village/neighborhood 乡/镇/街道/村/社区
4. Abroad 国外

BB003 When did you first move to this county/city/district where your current address located(BB001_W3) 您第一次搬到居住地 BB001_W3 所在县/市/区是在什么时候?_____ [1900..2018] Year 年

BB004 When you first moved to current address BB001_W3, did you live in the same village/community as you currently do? 您第一次搬到居住地 BB001_W3 所在县/市/区，就是住在当前村/社区吗?

1. Yes 是 → skip to BB005_W3_1 跳至 BB005_W3_1
2. No 否

BB005 In what year did you first live in current address BB001_W3's village/community 您第一次搬来居住地 BB001_W3 所在村/社区居住是在什么时候? _____ [1900..2018] Year 年

BB005_W3_1 Would you change your address in next two years? 接下来的 2 年里, 您会搬家吗?

1. Yes 是
2. No 否 → Skip to the procedure before BC001_W3_1 跳至 BC001_W3_1 前的程序

BB005_W3_2 Where would your new address? 您的新家地址会是?

1. I am not sure 我不知道
2. Another house in the same village/community 同一个村/社区, 不同的房子
3. Other 其他地方:
   ______ (**BB005_W3_2_1**) province/city/county 省/市/县
   ______ (**BB005_W3_2_2**) township/village/neighborhood 乡/镇/街道/村/社区

PROCEDURE 程序:

If this is a reinterview, XRType = 1, ask BC001_W3_1 to BC002_W3_6 如果是回访受访者, 即 XRType = 1, 则提问 BC001_W3_1 至 BC002_W3_6

If this is a new interview, XRType = 2, ask BC002_W3_1 and BC002_W3_4, then skip to the procedure before BD001_W2_4 如果是新受访者, 即 XRType = 2, 则提问 BC002_W3_1 以及 BC002_W3_4, 然后跳至 BD001_W2_4 前的程序

BC001_W3_1 We record your Hukou type was [ZBC004] in [ZIWTime], right? 我们记录 [ZIWTime] 访问您时您的户口类型是 [ZBC004], 对吗?

1. Yes 是 → Skip to BC001_W3_3 跳至 BC001_W3_3
2. No 否

BC001_W3_2 What was your Hukou type in [ZIWTime]? [ZIWTime] 时, 您的户口类型是什么?

1. Agricultural Hukou 农业
2. Non-agricultural Hukou 非农业
3. Unified Residence Hukou 统一居民户
4. Do not have Hukou 没有户口

BC001_W3_3 Where was your Hukou in [ZIWTime]? [ZIWTime] 时, 您的户口所在地是哪儿?

1. ______ (**BC001_W3_3_1**) province/city/county 省/市/县
   ______ (**BC001_W3_3_2**) township/village/neighborhood 乡/镇/街道/村/社区
2. Did not have Hukou 没有户口

BC002_W3 Since [ZIWTime], have you Hukou type and Hukou location changed? 自从 [ZIWTime] 以来, 您的户口类型和户口所在地有没有发生变化?

1. Only Hukou type has changed 户口类型变了
2. Only Hukou registration place has changed 户口所在地变了
3. Both Hukou type and registration place have changed 户口类型和户口所在地都变了
4. Neither of Hukou type nor place has changed 都没变

PROCEDURE 程序:

If BC002_W3 = 1, 3, ask BC002_W3_1 to BC002_W3_3 如果 BC002_W3 = 1, 3, 则提问 BC002_W3_1 至 BC002_W3_3

BC002_W3_1 What is your current Hukou type? 您现在的户口类型是?

1. Agricultural Hukou 农业
2. Non-agricultural Hukou 非农业
3. Unified Residence Hukou 统一居民户口
4. Do not have Hukou 没有户口

BC002_W3_2 Why did your Hukou type change? 您的户口类型发生变化的原因是什么?

1. Go to school 上学
2. Marriage 结婚
3. Employment 就业
4. Land is acquired by the government 土地被征用
5. Migration of the whole village 整村搬迁
6. Other 其他: _____ (**BC002_W3_2_1**)

BC002_W3_3 When did your type of Hukou change? 您这次户口类型发生变化的时间是?

_____ (**BC002_W3_3_1**) Year 年 ___ (**BC002_W3_3_2**) Month 月

PROCEDURE 程序:

If BC002_W3 = 2, 3, ask BC002_W3_4 to BC002_W3_6 如果 BC002_W3 = 2, 3, 则提问 BC002_W3_4 至 BC002_W3_6

BC002_W3_4 What's your current location of Hukou? 您现在的户口所在地是?

1. Preload current address BB001_W3 加载当前常住地址 BB001_W3
2. Other 其他:
   _____ (**BC002_W3_4_1**) province/city/county 省/市/县
   _____ (**BC002_W3_4_2**) township/village/neighborhood 乡/镇/街道/村/社区
3. Does not have Hukou 没有户口

BC002_W3_5 Why did your location of Hukou change? 您这次户口所在地发生变化的原因是什么?

1. Education 上学
2. Marriage 结婚
3. Employment 就业
4. Land is acquired by the government 土地被征用
5. Migration of the whole village 整村搬迁
6. Other 其他: _____ (**BC002_W3_5_1**)

BC002_W3_6 When did your location of Hukou change? 您这次户口所在地发生变化的时间是?

_____ (**BC002_W3_6_1**) Year 年 _____ (**BC002_W3_6_2**) Month 月

PROCEDURE 程序:

If this is a reinterview, XRType = 1 and [ZFRedu] = 1, ask BD001_W2_4 to BD010_W4, then skip to the procedure before BD012 如果是回访受访者，即 XRType = 1，且 [ZFRedu] = 1，则询问 BD001_W2_4 至 BD010_W4，然后跳至 BD012 前的程序

If this is a reinterview, XRType = 1 and [ZFRedu] = 2, ask BD007_W4_1 to BD010_W4, then skip to the procedure before BD012 如果是回访受访者，即 XRType = 1，且 [ZFRedu] = 2，则询问 BD007_W4_1 至 BD010_W4，然后跳至 BD012 前的程序

If this is a new interview, XRType = 2, ask BD001_W2_4 to BD006, then skip to BD007_W2_1 如果是新受访者，即 XRType = 2，则询问 BD001_W2_4 至 BD006，然后跳至 BD007_W2_1

BD001_W2_4 What's the highest level of education your have now (not including adult education)? 您现在获得的最高教育水平是什么（不包括成人教育）?

1. No formal education (illiterate) 未受过教育（文盲）
2. Did not finish primary school 未读完小学
3. Sishu/home school 私塾毕业
4. Elementary school 小学毕业
5. Middle school 初中毕业
6. High school 高中毕业
7. Vocational school 中专（包括中等师范、职高）毕业
8. Two-/Three-Year College/Associate degree 大专毕业
9. Four-Year College/Bachelor's degree 本科毕业
10. Master's degree 硕士毕业
11. Doctoral degree/Ph.D. 博士毕业

PROCEDURE 程序:

If BD001_W2_4 = 1, 2, 3, ask BD001_W3_1 如果 BD001_W2_4 = 1, 2, 3，则询问 BD001_W3_1

BD001_W3_1 Are you literate? 您是否识字?

1. Yes 是
2. No 否

PROCEDURE 程序:

If BD001_W2_4 $\neq$ 1, 2, 3, ask BD002_W3 如果 BD001_W2_4 $\neq$ 1, 2, 3，则询问 BD002_W3

BD002_W3 How many years have you spent in school after your highest level of education, BD001_W2_4? (If haven't attended school after highest level of education, take down 0) 您从最高学历 BD001_W2_4 毕业以后又读了几年书?（毕业后没再读书的填 0） ______ Year 年

BD006 How old were you when you finished your highest level of education? 您几岁读完书? ______ [0...120] years old 岁

[IWER: If haven't attended school, take down 0 访员注意：没有读过书请填 0]

BD007_W4_1 Since [ZIWTime], have you attended school for adult education? (Choose all that apply) 自从 [ZIWTime] 以来，您参加过成人教育（如电大，夜校，自考，函授，扫盲班，速成班）吗？（可多选）

1. None 没有 → Skip to the procedure before BD012 跳至 BD012 前的程序
2. TV University 电大
3. Night School 夜校
4. Zikao (examinations for self-taught students) 自考
5. Hanshou/Correspondence course/Distance learning 函授
6. Literacy course 扫盲班
7. Accelerated education course 速成班
8. Other 其他，please specify 请注明 _____ **(BD007_W4_1_1)**

BD008_W4_1 How many years have you spent in adult education since ZIWTime? 自从 [ZIWTime] 以来，您成人教育一共读了几年？ _____ year 年

BD009_W4_1 Have you gotten a diploma or degree from the adult education program you have attended since ZIWTime? 自从 [ZIWTime] 以来，您有从成人教育中获得了学位或文凭吗？

1. Yes 是
2. No 否 → Skip to the procedure before BD012 跳至 BD012 前的程序

BD011_W4 What is the highest adult schooling degree or diploma you have since [ZIWTime]? 自从 [ZIWTime] 以来，您获得最高的成人教育学位或文凭是什么？

1. Vocational school 中专
2. Two/Three Year College / Associate degree 大专
3. Four Year College / Bachelor's degree 本科
4. Others 其他，please specify 请注明 _____ **(BD011_W4_1)**

BD010_W4 When did you attain the degree or diploma? 您什么时候获得该学位或者文凭的？ _____ Year 年

BD007_W2_1 Have you attended school for adult education? (Choose all that apply) 您参加过成人教育（如电大，夜校，自考，函授，扫盲班，速成班）吗？（可多选）

1. None 没有 → Skip to the procedure before BD012 跳至 BD012 前的程序
2. TV University 电大
3. Night School 夜校
4. Zikao (examinations for self-taught students) 自考
5. Hanshou/Correspondence course/Distance learning 函授
6. Literacy course 扫盲班
7. Accelerated education course 速成班
8. Other 其他，Please specify 请注明 _____ **(BD007_W2_1_1)**

BD008_W2_1 How many years did you spend in adult education? 您成人教育一共读了几年？ _____ year 年

BD009_W2_1 Did you get a diploma or degree from the adult education program you attended? 您有从成人教育中获得了学位或文凭吗?

1. Yes 是
2. No 否 → Skip to the procedure before BD012 跳至 BD012 前的程序

BD011 What is the highest adult schooling degree or diploma you have? 您获得最高的成人教育学位或文凭是什么?

1. Vocational school 中专
2. Two/Three Year College / Associate degree 大专
3. Four Year College / Bachelor's degree 本科
4. Others 其他，Please specify 请注明 _____ **(BD011_1)**

BD010 When did you attain the degree or diploma? 您什么时候获得该学位或者文凭的? _____ [1900...2018] year 年

PROCEDURE 程序:

If this is a reinterview, XRType = 1, ask BD012 to BD016_W4, then skip to BE001 如果是回访受访者，即 XRType = 1，则询问 BD012 至 BD016_W4，然后跳至 BE001

If this is a new interview, XRType = 2, skip to BD012_W4 如果是新受访者，即 XRType = 2，则跳至 BD012_W4

BD012 Since [ZIWTime], have you participated in vocational and technical training? 自从 [ZIWTime] 以来，您参加过职业技术培训吗?

1. Yes 是
2. No 否 → Skip to BE001 跳至 BE001

BD013 Since [ZIWTime], how many times have you participated in vocational and technical training? 自从 [ZIWTime] 以来，您接收过几次职业培训? _____ Times 次

BD014 Since [ZIWTime], how many months have you spent participating in vocational and technical training? 自从 [ZIWTime] 以来, 您职业技术培训一共参加了几个月? _____ Months 月

[IWER: If respondent has participated in training more than once, please add up the traing time 访员注意：如果参加了多次培训，请将培训时间累加起来]

BD015_W4 Since [ZIWTime], have you attained vocational skill certificate by participating in vocational and technical training? 自从 [ZIWTime] 以来，您通过参加的职业技术培训获得过职业技术证书吗?

1. Yes 是，获得过
2. No 否，没有 → Skip to BE001 跳至 BE001

BD016_W4 Since [ZIWTime], what vocational skill certificates you have attained by participating in vocational and technical training? 自从 [ZIWTime] 以来，您通过参加的职业技术培训获得过哪些职业技术证书? _____

BD012_W4 Have you participated in vocational and technical training? 您参加过职业技术培训吗?

1. Yes 是
2. No 否 → Skip to BE001 跳至 BE001

BD013_W4 How many times have you participated in vocational and technical training? 您接收过几次职业培训? _____ Times 次

BD014_W4 How many months have you spent participating in vocational and technical training? 您职业技术培训一共参加了几个月? _____ Months 月

[IWER: If respondent has participated in training more than once, please add up the traing time 访员注意：如果参加了多次培训，请将培训时间累加起来]

BD017_W4 您通过参加的职业技术培训获得过职业技术证书吗?

1. Yes 是，获得过
2. No 否，没有 → Skip to BE001 跳至 BE001

BD018_W4 Have you attained vocational skill certificate by participating in vocational and technical training? 您通过参加的职业技术培训获得过哪些职业技术证书? _____

BE001 What is your marital status? 您目前的婚姻状态是?

[IWER: common-law marriage is considered as married 访员注意：即使没有领结婚证但自称结婚者也可视为已婚]

1. Married and live with spouse 已婚与配偶一同居住 → Skip BE002 跳过 BE002
2. Married but don't living with spouse temporarily for reasons such as work 已婚，但因为工作等原因暂时没有跟配偶在一起居住 → Skip BE002 跳过 BE002
3. Separated, don't live together as a couple anymore 分居（不再作为配偶共同生活）
4. Divorced 离异
5. Widowed 丧偶
6. Never married 从未结婚

BE002 Do you have a mate living with you as a couple (cohabit)? 您现在是否有伴侣以配偶身份共同生活（同居）?

1. 有
2. 没有 → Skip BE003_W4 跳过 BE003_W4

BE003_W4 How long have you been living with your spouse in the last year? 过去一年，您和您的配偶/伴侣共同居住了多长时间? _____ [0...12] Month 月

PROCEDURE 程序:

If this is a reinterview, XRType = 1, and the respondent had separated from his/her spouse for a long time, CV004 = 3, ask BF005_W4 and BF005_W4_3, then skip to BF008 如果是回访受访者，即 XRType = 1，且与上次访问时的配偶已经长期分居即 CV004 = 3，则询问 BF005_W4 以及 BF005_W4_3， 然后跳至 BF008

If this is a reinterview, XRType = 1. And the respondent had divorced, CV004 = 2, skip to BF006_W4 如果是回访受访者，即 XRType = 1，且与上次访问时的配偶已经离婚即 CV004 = 2，则跳至 BF006_W4

If not, skip to BG001_W4 否则， 跳至 BG001_W4

BF005_W4 When did you separate from your [name of ex-spouse]? 您什么时候和 [离异配偶姓名] 分居的? ______ 1900...2015 (**BF005_W4_1**) Year 年 ___ 1...12 (**BF005_W4_2**) Month 月

BF005_W4_3 Why did you separate from your [name of ex-spouse]? 您和 [离异配偶姓名] 分居的原因是?

1. Emotional feud 感情不和
2. Other 其他原因，please specify 请注明 ______ (**BF005_W4_3_1**)

BF006_W4 When did you divorce [name of ex-spouse]? 您什么时候和 [离异配偶姓名] 离婚的? ______ [2011...2018] (**BF006_W4_1**) Year 年 ___ 1...12 (**BF006_W4_2**) Month 月

BF006_W4_3 What's the main reason why you divorced your [name of ex-spouse]? 您和 [离异配偶姓名] 离婚的主要原因是?

1. Emotional feud 感情不和
2. Live in different places 两地分居
3. In order to facilitate the property purchase transactions 为了便利不动产等购买交易
4. Other 其他原因，please specify 请注明 ______ (**BF006_W4_3_1**)

BF006_W4_4 When you divorced [name of ex-spouse], regarding divided assets, how much is yours including real estate, car, etc calculated as currency 和 [离异配偶姓名] 离婚时，关于双方共同财产（包括不动产、汽车等）分割情况, 以货币价值计算, 您得到 ______ (**BF006_W4_4_1**) Yuan 元， How much is [name of ex-spouse] [离异配偶姓名] 得到 ______ (**BF006_W4_4_2**) Yuan 元

BF006_W4_5 When you divorced [name of ex-spouse], did you have any infancy children (under 18)? 您和 [离异配偶姓名] 离婚时， 你们有未成年（不满 18 岁）的孩子吗?

1. Yes 有， there is/are 有 ______ (**BF006_W4_5_1**) child/children 个
2. No 没有 → Skip to BG001_W4 跳至 BG001_W4

BF006_W4_6 When you divorced [name of ex-spouse], who brought up infancy child/children? 您和 [离异配偶姓名] 离婚时， 未成年的孩子归谁抚养?

I brought up 我抚养 ______ (**BF006_W4_6_1**) child/children 个, [name of ex-spouse] brought up [离异配偶姓名] 抚养 ______ (**BF006_W4_6_2**) child/children 个

[IWER: Take down 0 if one didn't bring up children. The total number of children here should match the number of children when they divorced 访员注意：没有抚养孩子的一方填写 0，双方抚养孩子的加总应当等于离婚时的未成年孩子数]

**PROCEDURE** 程序:
If BF006_W4_6_1 ≥ 1，ask BF006_W4_7 如果 BF006_W4_6_1 ≥ 1，则询问 BF006_W4_7

BF006_W4_7 How much does [name of ex-spouse] pay for you every month as child support payment? [离异配偶姓名] 需要每月给您多少子女抚养费？ ______ Yuan 元

**PROCEDURE** 程序:
If BF006_W4_6_2 ≥ 1，ask BF006_W4_8 如果 BF006_W4_6_2 ≥ 1，则询问 BF006_W4_8

BF006_W4_8 How much do you pay for [name of ex-spouse] every month as child support payment? 您需要每月给 [离异配偶姓名] 多少子女抚养费？ ______ Yuan 元

BG001_W4 Are you Han or Ethnic Minorities? 您是汉族还是少数民族？

1. Han 汉族
2. Zhuang 壮族
3. Man 满族
4. Hui 回族
5. Miao 苗族
6. Weiwuer 维吾尔族
7. Tujia 土家族
8. Yi 彝族
9. Mongol 蒙古族
10. Zang 藏族
11. Other 其他少数名族，please specify 请注明 ______ **(BG001_W4_1)**

BG002_W4 Do you have any religious belief? Such as Buddhism, Taosim, Christianity, etc. 您是否有宗教信仰？或者说是否信教，比如佛教、道教基督教等？

1. Yes 是
2. No 否 → Skip BG003_W4 跳过 BG003_W4

BG003_W4 Which religious belief do you believe in? 您信仰哪个宗教？

1. Buddhism 佛教
2. Taosim 道教
3. Islam 伊斯兰教
4. Catholicism 天主教
5. Christianity 基督教
6. Other 其他，please specify 请注明 ______ **(BG003_W4_1)**

BG004_W4 Are you the Communist party member? 您是不是共产党员？

1. Yes 是，When did you join the Cmmunist Party 哪一年入党的? ______ (**BG004_W4_1**) Year 年
2. No 否

BG005_W4 Did you participate in the Up to Mountains and Down to the Countryside Movement as an educated youth? 您有没有过上山下乡当知青的经历?

1. Yes 有，When did you participate in the Up to Mountains and Down to the Countryside Movement as an educated youth 哪一年上山下乡的? ______ (**BG005_W4_1**) Year 年
2. No 没有 → Skip BG006_W4 跳过 BG006_W4

BG006_W4 Were you back to the city after the Up to Mountains and Down to the Countryside Movement as an educated youth? 您上山下乡当知青后有没有回城?

1. Yes 有，When were you back to the city 哪一年回城的? ______ (**BG006_W4_1**) Year 年
2. No 没有

BF008 Interviewer records respondent behavior throughout the questionnaire: how often did [name of respondent] receive assistance in answering this module, Demographic backgrounds? 访员记录受访者回答基本信息时的应答行为，[受访者姓名] 在回答基本信息模块时是否有求助其他人?

1. Never 从未
2. A few times 偶尔几次
3. Most of time 大多数
4. Proxy by others 由其他人代理

C Family 家庭

PROCEDURE 程序:

If the value of [MR's spouse name] is not null, ask CV009, and save the name as [Name of the family respondent] and save name of the other respondent as [Name of spouse of the family respondent] 如果 [配偶姓名] 不为空，则询问 CV009，然后将其存为 [家庭受访者姓名]，将另外一个受访者的姓名存为 [家庭受访者的配偶姓名]

If the value of [MR's spouse name] is null, save [Main Respondent's name] as [Name of the family respondent] and save [Name of spouse of the family respondent] as null, and skip directly to PARENT, CHILDREN AND SIBLING INFORMATION 如果 [配偶姓名] 为空，则将 [主要受访者姓名] 存为 [家庭受访者姓名]，[家庭受访者的配偶姓名] 存为空，然后跳至 父母、子女以及兄弟姐妹信息

CV009 We would like to know the information about your family members, including children, parents and siblings of you, and your husband/wife, etc. Who has a better idea about such information, [Main Respondent's name] or [MR's spouse name]? 接下来的我们会问到一些关于您家庭成员的问题，包括子女、夫妻双方的父母及兄弟姐妹等等，请问 [主要受访者姓名] 和 [配偶姓名] 谁更了解这方面的情况?

1. [Main Respondent's name][主要受访者姓名]
2. [MR's spouse name][配偶姓名]

C1 Parent, Children and Sibling Information 父母、子女以及兄弟姐妹信息

CA Parent Information 父母信息

CAPI:

CA section is regarding parent information of [Name of the family respondent] and [Name of spouse of the family respondent]. Respondent for this part should be [Name of the family respondent], while it is allowed for [Name of spouse of the family respondent] to answer questions about his/her own parents during on-site interview. Answers to questions in this part will be saved under [Name of the family respondent]'s ID CA 部分询问 [家庭受访者姓名] 及 [家庭受访者的配偶姓名] 的父母信息。回答 CA 部分的受访者原则上是 [家庭受访者姓名]，在实地允许 [家庭受访者的配偶姓名] 回答自己的父母部分，CA 部分的信息记录在 [家庭受访者姓名] 的个人 ID 下

Codes for parents of [Name of the family respondent] are: 1 Biological father, 2 Biological mother, 3 Adoptive father, 4 Adoptive mother; and codes for parents of [Name of spouse of the family respondent] are: 5 Biological father, 6 Biological mother, 7 Adoptive father, 8 Adoptive mother [家庭受访者姓名] 父母编码为：1 亲生父亲，2 亲生母亲，3 养父，4 养母。[家庭受访者的配偶姓名] 父母编码为：5 亲生父亲，6 亲生母亲，7 养父，8 养母

The following intermediate variables (series) will be generated, with the codes for parents/parents-in-law as subscript: 生成下列中间变量（数组），下标为父母编码：

- XParExist denotes whether such parent exists or not 存储是否存在该类父母
- XParBirth denotes the date of birth of the parents 存储父母出生日期
- XParAlive denotes whether such parent still alives or not 存储父母是否健在

[INTRO: Firstly, here are a couple of questions about parents of [Name of the family respondent] 引语：首先我想问您一些关于 [家庭受访者姓名] 父母的问题]

CA000_W4_0 Does [Name of the family respondent] and [Name of spouse of the family respondent] know information about biological father of [Name of the family respondent]? [家庭受访者姓名] 和 [家庭受访者的配偶姓名] 是否了解 [家庭受访者姓名] 的亲生父亲的基本信息？ [IWER: The vast majority of people know information about their biological father, unless under certain circumstances where they were adopted by strangers upon birth, they may have no idea about their biological father. During on-site interview, there is no need to ask respondent this question and select "Yes, I know" directly. In the subsequent parts of the interview, if you've got to know that the respondent was given away to someone else for adoption or if he/she was abandoned upon birth, you should return to this question and select "No, I don't" after a telephone call for approval code and skip questions about biological father of [Name of the family respondent] 访员注意：绝大部分人肯定是了解自己亲生父亲的信息的，仅仅是那些出生后就被送养给陌生人等特殊情况才会完全不知道自己亲生父亲的信息。在访问中，无需询问该题，直接选择"是，了解"，在后续访问中，如果了解到受访者确实是属于出生后就被送养、遗弃等特殊情况，再返回该题，电话申请批准代码后再选择"否，不了解"从而跳过亲生父亲的相关问题]

1. Yes, I know 是，了解 → CAPI updates the value of XParExist[1] to 1 CAPI 更新 XParExist[1] 为 1
2. No, I don't. Please specify the reason why the respondent doesn't know information about his/her biological father 否，不了解。请输入不了解亲身父亲信息的具体原因 ______ (**CA000_W4_0_1**) input the approval code here 请输入批准代码 ______ (**CA000_W4_0_2**) → CAPI updates the value of XParExist[1] to 0 CAPI 更新 XParExist[1] 为 0

PROCEDURE 程序:

Ask the family respondent and his/her spouse (if applicable) questions from CA000_W4_1 to CA007_W3_5 . Automatically preload [Name of the family respondent] or [Name of spouse of the family respondent] in corresponding questions, choices, prefaces, and logics of procedures, with all variables postfixed and $i \in \{1,2\}$ as loop index 对家庭受访者及家庭受访者的配偶（如果存在）询问 CA000_W4_1 至 CA007_W3_5 。在题干、选项、引语及逻辑中加载 [家庭受访者姓名] 或 [家庭受访者的配偶姓名]，所有变量名加后缀，循环指标为 i, $i \in \{1,2\}$

PROCEDURE 程序:

If XParExist[1] = 1,go on with the next question, otherwise go to CA001_W4_0 如果 XParExist[1] = 1，则继续下一条逻辑，否则跳至 CA001_W4_0

If the value of [ZParBirth[1]] is not null, question CA000_W4_1 should be asked 如果 [ZParBirth[1]] 不为空，则询问 CA000_W4_1

CA000_W4_1 Was the biological father of [Name of the family respondent] born in [ZParBirth[1]]? [家庭受访者姓名] 的亲生父亲是 [ZParBirth[1]] 年出生的，对吗?

1. Yes 对 → CAPI updates XParBirth[1] to ZParBirth[1], and go directly to CAPI 更新 XParBirth[1] 为 ZParBirth[1]，然后跳至 CA000_W3_2
2. No 不对

CA000_W3_1 When was the biological father of [Name of the family respondent] born? [家庭受访者姓名] 的亲生父亲是哪一年出生的? ______ Year 年 [1800..1970]

[IWER: Mark the year using four digits. If the respondent does not remember the birth year of his/her biological father clearly, you could calculate it from information about his/her biological father's age now, his/her biological father's death year and the age of death, or age of his/her biological father when he/she was born 访员注意：用 4 位数表示年，如果受访者记不得亲生父亲的出生年份，可以通过其今年多大，或者哪年去世的，去世时多大以及受访者出生时亲生父亲多大等信息推算出生年份]

[CAPI: update XParBirth[1] to the value of CA000_W3_1 更新 XParBirth[1] 为 CA000_W3_1]

CA000_W3_2 Is [Name of the family respondent]'s biological father still alive? [家庭受访者姓名] 的亲生父亲还健在吗?

1. Yes 是 → CAPI updates XParAlive[1] to 1, and go to procedures before CA001_W4_1 CAPI 更新 XParAlive[1] 为 1，然后跳至 CA001_W4_1 之前的程序
2. No 否 → CAPI updates XParAlive[1] to 0, and go on with the next question CAPI 更新 XParAlive[1] 为 0，然后继续询问

PROCEDURE 程序:
If [ZParDYear[1]] is null, go on with question CA000_W3_3 如果 [ZParDYear[1]] 为空，则询问 CA000_W3_3

CA000_W3_3 When did [Name of the family respondent]'s biological father pass away? [家庭受访者姓名] 的亲生父亲是什么时候去世的? _____ (**CA000_W3_3_1**) Year 年 or how old was he when he died? 或去世时 _____ (**CA000_W3_3_2**) Age 岁

PROCEDURE 程序:
If [ZParDReason[1]] is null, go on with question CA000_W3_4 如果 [ZParDReason[1]] 为空，则询问 CA000_W3_4

CA000_W3_4 What's the leading cause of death for [Name of the family respondent]'s biological father? [家庭受访者姓名] 的亲生父亲去世的主要原因是什么? _____
[IWER: If the cause of death is illness, please specify the kind of illness. For example, if it is cancer, the specific type of cancer should be recorded (gastric cancer, lung cancer, etc.); if it is infectious disease, please specify the disease (tuberculosis, dysentery, etc.); or if it is an accident, please give information on the accident type (car accident, fire, intoxication, etc.) 访员注意：如果是因疾病死亡，请详细写明病因，例如因癌症去世，要注明是哪种类型的癌症（胃癌、肺癌等）；如果因传染病去世，要写明传染病的类型（结核病、痢疾）；如果是因为事故死亡，要写明事故类型，如车祸、火灾、意外中毒等]

CA001_W4_0 Does [Name of the family respondent] and [Name of spouse of the family respondent] know information about biological mother of [Name of the family respondent]? [家庭受访者姓名] 和 [家庭受访者的配偶姓名] 是否了解 [家庭受访者姓名] 的亲生母亲的基本信息?
[IWER: The vast majority of people know information about their biological mother, unless under certain circumstances where they were adopted by strangers upon birth, they may have no idea about their biological mother. During on-site interview, there is no need to ask respondent this question and select "Yes, I know" directly. In the subsequent parts of the interview, if you've got to know that the respondent was given away to someone else for adoption or if he/she was abandoned upon birth, you should return to this question and select "No, I don't" after a telephone call for approval code and skip questions about biological mother of [Name of the family respondent] 访员注意：绝大部分人肯定是了解自己亲生母亲的信息的，仅仅是那些出生后就被送养给陌生人等特殊情况才会完全不知道自己亲生母亲的信息。在访问中，无需询问该题，直接选择"是，了解"，在后续访问中，如果了解到受访者确实是属于出生后就被送养、遗弃等特殊情况，再返回该题，电话申请批准代码后再选择"否，不了解"从而跳过亲生母亲的相关问题]

1. Yes, I know 是，了解 → CAPI updates the value of XParExist[2] to 1 CAPI 更新 XParExist[2] 为 1
2. No, I don't 否，不了解 Please specify the reason why the respondent doesn't know information about his/her biological mother 请输入不了解亲生母亲信息的具体原因 _____ (**CA001_W4_0_1**) input the approval code here 请输入批准代码 _____ (**CA001_W4_0_2**) → CAPI updates the value of XParExist[2] to 0 CAPI 更新 XParExist[2] 为 0

PROCEDURE 程序:

If XParExist[2] = 1，go on with the next question, otherwise skip to CA006_W3_1 如果 XParExist[2] = 1，则继续下一条逻辑，否则跳至 CA006_W3_1

If the value of [ZParBirth[2]] is not null, question CA001_W4_1 should be asked 如果 [ZParBirth[2]] 不为空，则询问 CA001_W4_1

CA001_W4_1 Was the biological mother of [Name of the family respondent] born in [ZParBirth[2]]? [家庭受访者姓名] 的亲生母亲是 [ZParBirth[2]] 年出生的，对吗?

1. Yes 对 → CAPI updates XParBirth[2] to ZParBirth[2], and go directly to CAPI 更新 XParBirth[2] 为 ZParBirth[2]，然后跳至 CA001_W3_2
2. No 不对

CA001_W3_1 When was the biological mother of [Name of the family respondent] born? [家庭受访者姓名] 的亲生母亲是哪一年出生的? _____ Year 年 [1800..1970]

[IWER: Mark the year using four digits. If the respondent does not remember the birth year of his/her biological mother clearly, you could calculate it from information about his/her biological mother's age now, his/her biological mother's death year and the age of death, or age of his/her biological mother when he/she was born 访员注意：用 4 位数表示年，如果受访者记不得亲生母亲的出生年份，可以通过其今年多大，或者哪年去世的，去世时多大以及受访者出生时亲生母亲多大等信息推算出生年份]

[CAPI: Update XParBirth[2] to the value of CA001_W3_1 更新 XParBirth[2] 为 CA001_W3_1]

CA001_W3_2 Is [Name of the family respondent]'s biological mother still alive? [家庭受访者姓名] 的亲生母亲还健在吗?

1. Yes 是 → CAPI updates XParAlive[2] to 1, and go to procedures before CA002_W4 CAPI 更新 XParAlive[2] 为 1，然后跳至 CA002_W4 之前的程序
2. No 否 → CAPI updates XParAlive[2] to 0, and go on with the next question CAPI 更新 XParAlive[2] 为 0，然后继续询问

PROCEDURE 程序:

If [ZParDYear[2]] is null, go on with question CA001_W3_3 如果 [ZParDYear[2]] 为空，则询问 CA001_W3_3

CA001_W3_3 When did [Name of the family respondent]'s biological mother pass away? [家庭受访者姓名] 的亲生母亲是什么时候去世的? _____ (**CA001_W3_3_1**) Year 年 or how old was she when she died? 或去世时 _____ (**CA001_W3_3_2**) Age 岁

PROCEDURE 程序:

If [ZParDReason[2]] is null, go on with question CA001_W3_4 如果 [ZParDReason[2]] 为空，则询问 CA001_W3_4

CA001_W3_4 What's the leading cause of death for [Name of the family respondent]'s biological mother? [家庭受访者姓名] 的亲生母亲去世的主要原因是什么? ______

[IWER: If the cause of death is illness, please specify the kind of illness. For example, if it is cancer, the specific type of cancer should be recorded (gastric cancer, lung cancer, etc.); if it is infectious disease, please specify the disease (tuberculosis, dysentery, etc.); or if it is an accident, please give information on the accident type (car accident, fire, intoxication, etc.) 访员注意：如果是因疾病死亡，请详细写明病因，例如因癌症去世，要注明是哪种类型的癌症（胃癌、肺癌等）；如果因传染病去世，要写明传染病的类型（结核病、痢疾）；如果是因为事故死亡，要写明事故类型，如车祸、火灾、意外中毒等]

PROCEDURE 程序:

If ZParAlive[1] = 1 and ZParAlive[2] = 1, go on with question CA002_W4 如果 ZParAlive[1] = 1 且 ZParAlive[2] = 1，则询问 CA002_W4

CA002_W4 Are [Name of the family respondent]'s biological father and biological mother still spouse of each other?[家庭受访者姓名] 的亲生父母现在还是夫妻吗?

[IWER: Long-term separation due to lack of affection constitutes divorce in this context; and a couple remarrying each other after a divorce are not deemed as divorced 访员注意：因为感情问题长久分居也算离婚；离婚又复婚的不算离婚]

1. Yes 是
2. No 否

CA006_W3_1 Does [Name of the family respondent] have an adoptive father? [家庭受访者姓名] 有养父吗?

1. Yes 有 → CAPI updates XParExist[3] to 1, and go on with the next question CAPI 更新 XParExist[3] 为 1，然后继续询问
2. No 没有 → CAPI updates XParExist[3] to 0, and go to CAPI 更新 XParExist[3] 为 0，然后跳至 CA007_W3_1

CA006_W3_2 Does [Name of the family respondent]'s adoptive father still alive? [家庭受访者姓名] 的养父还健在吗?

[IWER: If more than one adoptive fathers exist, please ask about information on the last one that the respondent stay with 访员注意：如果 [家庭受访者姓名] 有两个及以上的养父，请询问最后一个]

1. Yes 是 → CAPI updates XParAlive[3] to 1, and go on with the next question CAPI 更新 XParAlive[3] 为 1，然后继续询问
2. No 否 → CAPI updates XParAlive[3] to 0, and go to CAPI 更新 XParAlive[3] 为 0，然后跳至 CA007_W3_1

PROCEDURE 程序:
If [ZParBirth[3]] is not null, go on with question CA006_W4_3 如果 [ZParBirth[3]] 不为空，则询问 CA006_W4_3

CA006_W4_3 Was the adoptive father of [Name of the family respondent] born in [ZParBirth[3]]? [家庭受访者姓名] 的养父是 [ZParBirth[3]] 年出生的，对吗?

1. Yes 对 → CAPI updates XParBirth[3] to ZParBirth[3], and go directly to CAPI 更新 XParBirth[3] 为 ZParBirth[3]，然后跳至 CA007_W3_1
2. No 不对

CA006_W3_3 When was the adoptive father of [Name of the family respondent] born? [家庭受访者姓名] 的养父是哪一年出生的? ______ Year 年 [1800..1970]
[IWER: Mark the year using four digits. If the respondent does not remember the birth year of his/her adoptive father clearly, you could calculate it from information about his/her adoptive father age now, his/her adoptive father's death year and the age of death, or age of his/her adoptive father when he/she was born 访员注意：用 4 位数表示年，如果受访者记不得养父的出生年份，可以通过其今年多大等信息推算出生年份]
[CAPI: Update XParBirth[3] to the value of CA006_W3_3] 更新 XParBirth[3] 为 CA006_W3_3]

CA007_W3_1 Does [Name of the family respondent] have an adoptive mother? [家庭受访者姓名] 有养母吗?

1. Yes 有 → CAPI updates XParExist[4] to 1, and go on with the next question CAPI 更新 XParExist[4] 为 1，然后继续询问
2. No 没有 → CAPI updates XParExist[4] to 0, and go to procedures before CA007_W3_5 directly CAPI 更新 XParExist[4] 为 0，然后跳至 CA007_W3_5 之前的程序

CA007_W3_2 Does [Name of the family respondent]'s adoptive mother still alive? [家庭受访者姓名] 的养母还健在吗?
[IWER: If more than one adoptive mothers exist, please ask about information on the last one that the respondent stay with 访员注意：如果 [家庭受访者姓名] 有两个及以上的养母，请询问最后一个]

1. Yes 是 → CAPI updates XParAlive[4] to 1, and go on with the next question CAPI 更新 XParAlive[4] 为 1，然后继续询问
2. No 否 → CAPI updates XParAlive[4] to 0, and go to procedures before CA007_W3_5 directly CAPI 更新 XParAlive[4] 为 0，然后跳至 CA007_W3_5 之前的程序

PROCEDURE 程序:
If [ZParBirth[4]] is not null, go on with question CA007_W4_3 如果 [ZParBirth[4]] 不为空，则询问 CA007_W4_3

CA007_W4_3 Was the adoptive mother of [Name of the family respondent] born in [ZParBirth[4]]? [家庭受访者姓名] 的养母是 [ZParBirth[4]] 年出生的，对吗?

1. Yes 对 → CAPI updates XParBirth[4] to ZParBirth[4], and go to procedures before CA007_W3_5 CAPI 更新 XParBirth[4] 为 ZParBirth[4]，然后跳至 CA007_W3_5 之前的程序
2. No 不对

CA007_W3_3 When was the adoptive mother of [Name of the family respondent] born? [家庭受访者姓名] 的养母是哪一年出生的? ______ Year 年 [1800..1970]
[IWER: Mark the year using four digits. If the respondent does not remember the birth year of his/her adoptive mother clearly, you could calculate it from information about his/her adoptive father age now, his/her adoptive mother's death year and the age of death, or age of his/her adoptive mother when he/she was born 访员注意：用 4 位数表示年，如果受访者记不得养母的出生年份，可以通过其今年多大等信息推算出生年份]
[CAPI: Update XParBirth[4] to the value of CA007_W3_3 更新 XParBirth[4] 为 CA007_W3_3]

PROCEDURE 程序:
If XParAlive[3] = 1 and XParAlive[4] = 1, go on with question CA007_W3_5 如果 XParAlive[3] = 1 并且 XParAlive[4] = 1, 则询问 CA007_W3_5

CA007_W3_5 Are [Name of the family respondent]'s adoptive father and adoptive mother still spouse of each other? [家庭受访者姓名] 的养父和养母目前是否是夫妻?
1. Yes 是
2. No 否

CAPI: Ask the respondent questions from CA009_W4_i to CA026_W3_i on his/her bilogical father, biological mother, adoptive father and adoptive mother sequentially with i as the loop index and following the rules below 针对 [家庭受访者姓名] 的亲生父亲、亲生母亲、养父、养母循环提问 CA009_W4_i 至 CA026_W3_i，循环指标为 i，规则如下：
Define such a series as [biological father, biological mother, adoptive father and adoptive mother,biological father, biological mother, adoptive father and adoptive mother] 定义数组 ParType 为 [亲生父亲、亲生母亲、养父、养母、亲生父亲、亲生母亲、养父、养母]
Questions will be asked only if XParExist[i] = 1 仅针对 XParExist[i] = 1 的父母进行提问

PROCEDURE 程序:
- For biological parents, i.e. $i = 1, 2$, questions CA009_W4_i to CA023_W3_i should be asked no matter the parent is alive or not; questions CA016_W3_i to CA026_W3_i are only applicable to biological parents who is still alive (i.e. XParAlive[i] = 1) 如果是亲生父母，即 $i = 1, 2$，则无论是否健在，均提问 CA009_W4_i 至 CA023_W3_i，然后仅针对健在的亲生父母（XParAlive[i] = 1）提问 CA016_W3_i 至 CA026_W3_i
- For other type of parents, i.e. $i = 3, 4$, questions CA009_W4_i to CA026_W3_i should only be asked for alive ones (i.e. XParAlive[i] = 1) 如果是其他父母, 即 $i = 3, 4$, 则仅对 XParAlive[i] = 1 的父母提问 CA009_W4_i 至 CA026_W3_i

PROCEDURE 程序:
If [ZParEdu[*i*]] is not null, go on with question CA009_W4_*i* 如果 [ZParEdu[*i*]] 不为空，则询问 CA009_W4_*i*

CA009_W4_*i* Is [Name of the family respondent]'s [ParType[*i*]]'s highest achieved education [ZParEdu[*i*]]? [家庭受访者姓名] 的 [ParType[*i*]] 的最高学历是 [ZParEdu[*i*]]，对吗?

1. Yes 对 → Skip to procedures before CA014_W4_*i* directly 跳至 CA014_W4_*i* 之前的程序
2. No 不对

CA009_*i* What's [Name of the family respondent]'s [ParType[*i*]]'s highest achieved education? [家庭受访者姓名] 的 [ParType[*i*]] 的最高学历是?

1. No formal education (illiterate) 未受过教育
2. Did not finish elementary school 未读完小学
3. Sishu/home school 私塾毕业
4. Elementary school 小学毕业
5. Middle school 初中毕业
6. High school 高中毕业
7. Vocational school 中专（包括中等师范、职高）毕业
8. Two-/Three-Year College / Associate degree 大专毕业
9. Four-Year College / Bachelor's degree 本科毕业
10. Post-graduate, Master's degree 硕士毕业
11. Post-graduate, Doctoral degree/Ph.D 博士毕业

PROCEDURE 程序:
If CA009_*i* = 1, 2, 3, go on with question CA009_W3_*i* 如果 CA009_*i* = 1, 2, 3, 询问 CA009_W3_*i*

CA009_W3_*i* Is [Name of the family respondent]'s [ParType[*i*]] literate? [家庭受访者姓名] 的 [ParType[*i*]] 是否识字?

1. Yes 是
2. No 否

PROCEDURE 程序:
If [ZParOcc[*i*]] is not null, go on with question CA014_W4_*i* 如果 [ZParOcc[*i*]] 不为空，则询问 CA014_W4_*i*

CA014_W4_*i* Is [ZParOcc[*i*]] [Name of the family respondent]'s [ParType[*i*]]'s highest achieved position in his/her career? [家庭受访者姓名] 的 [ParType[*i*]] 的职位最高的职业是 [ZParOcc[*i*]]，对吗?

1. Yes 对 → Skip to 跳至 CA023_W3_*i*
2. No 不对

CA014_*i* What's [Name of the family respondent]'s [ParType[*i*]]'s highest achieved position in his/her career? [家庭受访者姓名] 的 [ParType[*i*]] 的职位最高的职业是什么?

1. Managers in an enterprise or the persons in charge of general affairs in government agency/ organization of political party/ public service organization 国家机关、党群组织、企业、事业单位负责人
2. Professionals and technicians 专业技术人员
3. Clerks 办事人员和有关人员
4. Workers in business and services 商业、服务业人员
5. Workers in Agriculture, forestry, animal husbandry and fishery 农、林、牧、渔、水利业生产人员
6. Operating personnel of production/transportation equipment 生产、运输设备操作人员及有关人员
7. Others, please specify 不便分类的工作，请注明 ______ (**CA014_1_*i***)

CA023_W3_*i* Is [Name of the family respondent]'s [ParType[*i*]] a member of the Communist Party? [家庭受访者姓名] 的 [ParType[*i*]] 是共产党员吗?

1. Yes 是
2. No 否

CA016_W3_*i* Where was [Name of the family respondent]'s [ParType[*i*]] born? [家庭受访者姓名] 的 [ParType[*i*]] 是在哪里出生的?

______ (**CA016_W3_1_*i***) Province-city-county 省-市-县

______ (**CA016_W3_2_*i***) District-county/district-village/community 区县-乡镇-村社区

[CAPI: Drop-down lists are provided for all levels of address, of which the option of "other" is available for choice and corresponding manual inputs are allowed for address level of village 所有层级的地址都是下拉选择，其中村级包括其他选项，且可填写其他的具体名称]

CA016_*i* Where does [Name of the family respondent]'s [ParType[*i*]] live? [家庭受访者姓名] 的 [ParType[*i*]] 现在一般住在哪里?

1. Living with [Name of the family respondent] 与 [家庭受访者姓名] 同住
2. Living in houses in the same/neighboring courtyard(s) or flats in the same/neighboring building(s) as/of [Name of the family respondent]'s 与 [家庭受访者姓名] 住同一个院子（公寓）或者相邻的院子（公寓）
3. The place where he/she was born in [家庭受访者姓名] 的 [ParType[*i*]] 的出生地
4. Other 其它:______ (**CA016_1_*i***) Province-city-county 省-市-县,______ (**CA016_2_*i***) District-county/district-village/community 区县-乡镇-村社区
5. Living abroad 国外

[CAPI: Drop-down lists are provided for all levels of address, of which the option of "other" is available for choice and corresponding manual inputs are allowed for address level of village 所有层级的地址都是下拉选择，其中村级包括其他选项，且可填写其他的具体名称]

PROCEDURE 程序:

If CA016_*i* = 4, 5, go on with question CA017_*i* 如果 CA016_*i* = 4, 5, 则询问 CA017_*i*

CA017_i The place where [Name of the family respondent]'s [ParType[i]] lives in is [家庭受访者姓名] 的 [ParType[i]] 现在居住地的类型是?

1. Urban areas 城或镇中心区
2. Suburban areas 城乡或镇乡结合区
3. Rural areas 农村
4. Other special areas 特殊区域

CA026_W3_i Does [Name of the family respondent]'s [ParType[i]] have the ability to take care of him/herself? [家庭受访者姓名] 的 [ParType[i]] 现在是否能生活自理?

1. Yes 是
2. No 否

PROCEDURE 程序: 如果 [家庭受访者的配偶姓名] 不为空

If the value of [Name of spouse of the family respondent] is not null, questions CA009_W4_i to CA026_W3_i should then be asked to gather information on [Name of spouse of the family respondent]'s biological father, biological mother, adoptive father and adoptive mother, with $i \in \{5, 6, 7, 8\}$ being the loop index 同样的逻辑针对 [家庭受访者的配偶姓名] 的亲生父亲、亲生母亲、养父、养母循环提问 CA009_W4_i 至 CA026_W3_i，循环指标为 i，$i \in \{5, 6, 7, 8\}$

CAPI: CAPI: Generate a new series XParName = [Name of the family respondent]'s biological father, [Name of the family respondent]' biological mother, [Name of the family respondent]'s adoptive father, [Name of the family respondent]'s adoptive mother, [Name of spouse of the family respondent]'s biological father, [Name of spouse of the family respondent]' biological mother, [Name of spouse of the family respondent]'s adoptive father, [Name of spouse of the family respondent]'s adoptive mother. The newly defined series XParName, together with the previously defined XParExist and XParAlive will be used in subsequent sections to preload the names of the repondents' parents in relevant questions 生成数组 XParName = [[家庭受访者姓名] 的亲生父亲、[家庭受访者姓名] 的亲生母亲、[家庭受访者姓名] 的养父、[家庭受访者姓名] 的养母、[家庭受访者的配偶姓名] 的亲生父亲、[家庭受访者的配偶姓名] 的亲生母亲、[家庭受访者的配偶姓名] 的养父、[家庭受访者的配偶姓名] 的养母]，加上已有的 XParExist 以及 XParAlive 数组以方便后续模块调用父母姓名

CB Children Information 子女信息

CAPI:

CB section is regarding children's information of [Name of the family respondent]. Respondent for this part should be [Name of the family respondent], while it is allowed for [Name of spouse of the family respondent] to answer questions in this section during on-site interview. Answers to questions in this part will be saved under [Name of the family respondent]'s ID. All relationships are defined from the perspective of [Name of the family respondent] CB 部分询问 [家庭受访者姓名] 的子女信息。回答 CB 部分的受访者原则上是 [家庭受访者姓名]，在实地也允许 [家庭受访者的配偶姓名] 回答，CB 部分的信息记录在 [家庭受访者姓名] 的个人 ID 下，所有相对关系都是相对于 [家庭受访者姓名]

Codes for children of [Name of the family respondent] will follow rules as they are preloaded in CAPI. Any newly mentioned children will be coded after the preloaded ones. If the respondent is added to the survey for the first time, the codes for children will start from 1 [家庭受访者姓名] 的子女编码继承加载的编码，如果有新增加的子女，在原序号后增加，如果是新受访户，则从 1 开始编号

The following intermediate variables (series) will be generated, with the codes for children as subscript 生成下列中间变量（数组），下标为子女编码

- XChildName a string variable, saving names of children 字符型，存储子女姓名
- XChildGender drop-down list provided, with 1 denoting male and 2 denoting female, saving genders of children 下拉菜单，1 男性，2 女性，存储子女性别
- XChildBirth an integer of 4 digits, saving the birth year of children 四位整数型，存储子女出生年份
- XChildType drop-down list provided, with 1 denoting biological child of [Name of the family respondent] and [Name of spouse of the family respondent], 2 denoting adoptive child of [Name of the family respondent] and [Name of spouse of the family respondent], 3 denoting biological child of [Name of the family respondent], 4 denoting biological child of [Name of spouse of the family respondent], 5 denoting "not my children" ; if the value of [Name of spouse of the family respondent] is null, CAPI will not show options with [Name of spouse of the family respondent] 下拉菜单，1 [家庭受访者姓名] 和 [家庭受访者配偶姓名] 亲生，2 [家庭受访者姓名] 或 [家庭受访者配偶姓名] 领养，3 [家庭受访者姓名] 亲生，4 [家庭受访者配偶姓名] 亲生，5 不是我的孩子；如果 [家庭受访者配偶姓名] 为空，则不显示带有该加载的选项
- XChildEdu saving education of children; definition for this can be found in CB052_W3_*i* 存储子女教育，其定义见 CB052_W3_*i*

[INTRO: In the following section, we would like to ask some questions about your children 引语：下面我想问您一些关于您子女的问题]

PROCEDURE 程序:

If this household has been already included in our previous survey (XHType = 1), and ZChildNum ≠ 0, skip to CB050_W4_1 如果是回访受访户，即 XHType = 1，且 ZChildNum ≠ 0，则跳至 CB050_W4_1

If this household has been already included in our previous survey (XHType = 1), and ZChildNum = 0, skip to CB051_W3_1 如果是回访受访户，即 XHType = 1，且 ZChildNum = 0，则跳至 CB051_W3_1

If this household is added into the survey for the first time (XHType = 2), questions CB050_W3 and CB050_W4 should be asked and then skip to procedures before CB039_*i* 如果是新受访户，即 XHType = 2，则询问 CB050_W3 和 CB050_W4，然后跳至 CB039_*i* 前的 CAPI

CB050_W3 Including biological children, stepchildren, and adoptive children. How many children who are still alive does [Name of the family respondent] have? 包括亲生子女、继子女和养子女，[家庭受访者姓名] 一共有多少个健在的子女？ ______

PROCEDURE 程序:

If CB050_W3 ≥ 1, go on with question CB050_W4, otherwise skip all questions about children 如果 CB050_W3 ≥ 1，则提问 CB050_W4，否则跳过子女部分

CB050_W4 Please ask the respondent the following questions regarding his/her children in the order from the oldest to the youngest 访员请按照从大到小的顺序向受访者提问下表中的内容 [CAPI: CB050_W3 is the number of rows/children; the option 5 "not my children" should not be available for choice for this question 表中显示的需要提问的行数为 CB050_W3，此题子女类型不要显示选项 5 不是我的孩子]

子女序号<br>Serial number of children	姓名<br>(XChildName)	性别<br>(XChildGender)	出生年份<br>(XChildBirth)	子女类型<br>(XChildType)
1				
2				
...				
CB050_W3				

CB050_W4_1 Please confirm with the respondent the following information regarding his/her children in the order from the oldest to the youngest; If true, leave it as it is; if false, correct it accordingly in the following table. Please be noted that childern's education cannot be adjusted in this table 访员请按照从大到小的顺序向受访者确认下表中的内容，如果正确，无需改动，如果错误，直接在表中修改，注意子女教育在该表中不可修改

[IWER: Please don't skip to questions about another child before you finish asking/confirming all information about one particular child. Typos or other kinds of errors in children's names may occur due to misunderstanding of answers given in dialect; in such cases, please check with the respondent carefully before you conclude that this child does not exist 访员注意：请将某个子女的所有信息一起与受访者确认，其名字可能因为存在方言等问题，拼写不准确，此时不要轻易判定为不存在该子女]

[CAPI: In the following table, all information except for children education can be modified directly according to respondent's answers 姓名，性别，出生年份，是否健在，子女类型都是可以直接修改的，子女教育列仅用于提示，不可修改]

子女序号	姓名	性别	出生年份	是否健在	子女类型	子女教育
Serial number of children	(ZChildName)	(ZChildGender)	(ZChildBirth)	(ZChildAlive)	(ZChildType)	(ZChildEdu)
1						
2						
...						
ZChildNum						

[CAPI: Update the values of relevant Xchild series according to confirmation/ modification; don't generate variables for children classified as 5 "not my children" in ZChildType 请根据访员确认及修改后的结果更新相应的 XChild 变量，对于 ZChildType 确认为"不是我的孩子"的子女，不要生成对应的 XChild 变量]

CB051_W3_1 In addition to [XChildName], does [Name of the family respondent] have any other children who are still alive, including biological children, stepchildren and adoptive children? 除了 [XChildName]，[家庭受访者姓名] 还有几个其他健在的孩子？包括亲生子女、继子女和养子女。_____ Number 个

PROCEDURE 程序:

If CB051_W3_1 ≥ 1, go on with question CB051_W4 如果 CB051_W3_1 ≥ 1，则提问 CB051_W4

CB051_W4 Please give information about this/ these child(ren) mentioned in CB051_W3_1 in the order from the oldest to the youngest 请您按照年龄从大到小的顺序告诉我们 [家庭受访者姓名] 这 CB051_W3_1 个我们没有记录的子女的信息

[CAPI: The number of rows in the below table is CB051_W3_1; and the serial number for children starts from ZChildNum+1 表中显示的需要提问的行数为 CB051_W3_1；子女编号从 ZChildNum+1 开始]

子女序号	姓名	性别	出生年份	子女类型
	(XChildName)	(XChildGender)	(XChildBirth)	(XChildType)
ZChildNum+1				
ZChildNum+2				
...				
ZChildNum+CB051_W3_1				

CAPI: For each child who has been dead, i.e. XChildAlive= 0, questions CB039_*i* to CB040_W4_*i* should be asked iteratively, with *i* being the loop index 针对每个去世的子女，即 XChildAlive = 0，循环提问 CB039_*i* 至 CB040_W4_*i*，循环指标为 *i*

PROCEDURE 程序:

If ZChildDYear[*i*] is null, go on with question CB039_*i* 如果 ZChildDYear[*i*] 为空，则询问 CB039_*i*

CB039_*i* When did [XChildName[*i*]] pass away? [XChildName[*i*]] 是什么时候去世的? _____ (**CB039_1_*i***) Year 年 or his/her age when he/she died 或去世时 _____ (**CB039_2_*i***) Age 岁

> **PROCEDURE** 程序:
> If ZChildDReason[*i*] is null, go on with question CB040_W4_*i* 如果 ZChildDReason[*i*] 为空, 则询问 CB040_W4_*i*

CB040_W4_*i* What's the leading cause of death for [XChildName[*i*]]? [XChildName[*i*]] 去世的主要原因是什么? _____

[IWER: If the cause of death is illness, please specify the kind of illness. For example, if it is cancer, the specific type of cancer should be recorded (gastric cancer, lung cancer, etc.); if it is infectious disease, please specify the disease (tuberculosis, dysentery, etc.); or if it is an accident, please give information on the accident type (car accident, fire, intoxication, etc.) 访员注意：如果是因疾病死亡，请详细写明病因，例如因癌症去世，要注明是哪种类型的癌症（胃癌、肺癌等）；如果因传染病去世，要写明传染病的类型（结核病、痢疾）；如果是因为事故死亡，要写明事故类型，如车祸、火灾、意外中毒等]

> **CAPI**: For each child who is still alive, i.e. XChildAlive = = 1, questions CB052_W4_*i* to CB063_W3_6_*i* should be asked iteratively, with *i* being the loop index 针对每个健在的子女（XChildAlive = 1）循环提问 CB052_W4_*i* 至 CB063_W3_6_*i*，循环指标为 *i*

> **PROCEDURE** 程序:
> If ZChildEdu[*i*] is not null, go on with question CB052_W4_*i* 如果 ZChildEdu[*i*] 非空，则询问 CB052_W4_*i*

CB052_W4_*i* Without taking continuing education into account, is [ZChildEdu[*i*]] [XChildName[*i*]'s highest achieved education? 不包括成人教育,[XChildName[*i*]] 的最高学历是 [ZChildEdu[*i*]], 对吗?

1. Yes 对 → Skip to 跳至 CB053_*i*
2. No 不对

CB052_W3_*i* Without taking continuing education into account, what's [XChildName[*i*]'s highest achieved education? 不包括成人教育，[XChildName[*i*]] 的最高学历是?

1. No formal education (illiterate) 未受过正规教育
2. Did not finish elementary school 未读完小学
3. Sishu/home school 私塾
4. Elementary school 小学毕业
5. Middle school 初中毕业
6. High school 高中毕业
7. Vocational school 中专（包括中等师范、职高）毕业
8. Two-/Three-Year College/Associate degree 大专毕业
9. Four-Year College/Bachelor's degree 本科毕业
10. Master's degree 硕士毕业

11. Doctoral degree/Ph.D. 博士毕业
997. Have no idea 不知道
999. Refuse to answer this question 拒绝回答

PROCEDURE 程序:
If CB052_W3_*i* = 1, 2, 3, go on with question CB052_W3_1_*i* 如果 CB052_W3_*i* = 1, 2, 3, 则询问 CB052_W3_1_*i*

CB052_W3_1_*i* Is [XChildName[*i*]] literate? [XChildName[*i*]] 是否识字?
1. Yes 是
2. No 否

CB053_*i* Where does [XChildName[*i*]] live? [XChildName[*i*]] 现在一般住哪里?
1. Living with [Name of the family respondent] and not financially independent 与 [家庭受访者姓名] 同住且经济上不独立
2. Living with [Name of the family respondent] and financially independent 与 [家庭受访者姓名] 同住，但是经济上独立
3. Living in houses in the same/neighboring courtyard(s) or flats in the same/neighboring building(s) as/of [Name of the family respondent]'s 与 [家庭受访者姓名] 同一个院子（公寓）或者相邻的院子（公寓）
4. Other 其它:_____ (**CB053_1_*i***) Province-city-county/district 省-市-县,_____ (**CB053_2_*i***) county/district-village/community 区县-乡镇-村社区
5. Living abroad 国外
997. Have no idea 不知道
999. Refuse to answer this question 拒绝回答

[CAPI: Drop-down lists are provided for all levels of address, of which the option of "other" is available for choice and corresponding manual inputs are allowed for address level of village 所有层级的地址都是下拉选择，其中村级包括其他选项，且可填写其他的具体名称]

PROCEDURE 程序:
If CB053_*i* = 4, 5, go on with question CB054_*i* 如果 CB053_*i* = 4, 5, 则询问 CB054_*i*

CB054_*i* The place where [XChildName[*i*]] lives in is [XChildName[*i*]] 现在居住地的类型是?
1. Urban areas 城或镇中心区
2. Suburban areas 城乡或镇乡结合区
3. Rural areas 农村
4. Other special areas 特殊区域
997. Have no idea 不知道
999. Refuse to answer this question 拒绝回答

CB055_*i* What is [XChildName[*i*]]'s hukou type? [XChildName[*i*]] 目前的户口类型是什么?
[IWER: Unified Residency Hukou is a hukou type in some areas where Hukou system has

been reformed and it no longer differentiates between agricultural and non-agricultural residents. Instead, a single hukou type is used 访员注意："统一居民户口"指的是某些地方实行户口制度改革后，不再区分农业与非农业户口，而是统一为"居民户口"]

1. Agriculture Hukou 农业户口
2. Non-Agriculture Hukou 非农业户口
3. Unified Residency Hukou 统一居民户口
4. Do not have Hukou 没有户口 → Skip to CB063_W3_2_*i* 跳至 CB063_W3_2_*i*

997. Have no idea 不知道 → Skip to CB057_*i* 跳至 CB057_*i*
999. Refuse to answer this question 拒绝回答 → Skip to CB057_*i* 跳至 CB057_*i*

**PROCEDURE** 程序:
If CB055_*i* = 3, go on with questions CB055_W2_1_*i* and CB055_W2_2_*i* 如果 CB055_*i* = 3, 则询问 CB055_W2_1_*i* 和 CB055_W2_2_*i*

CB055_W2_1_*i* What was [XChildName[*i*]]'s hukou type before his/her Unified Residency Hukou? [XChildName[*i*]] 在获得统一居民户之前是什么户口?

1. Agriculture Hukou 农业户口
2. Non-Agriculture Hukou 非农业户口
3. Do not have Hukou 没有户口

997. Have no idea 不知道
999. Refuse to answer this question 拒绝回答

CB055_W2_2_*i* When did [XChildName[*i*]] get the Unified Residency Hukou? [XChildName[*i*]] 是什么时候获得的统一居民户口的? ______ Year 年

[CAPI: The answer could be "don't know" here 这里允许不知道]

CB057_*i* What's the registered location in [XChildName[*i*]] 's hukou? [XChildName[*i*]] 目前的户口所在地是?

1. The place where he/she was born in 他/她的出生地
2. The place where he/she lives in 他/她现在一般居住的地方
3. Other 其它:______ (**CB057_1_*i***) Province-city-county 省-市-县,______ (**CB057_2_*i***) county / district-village / community 区县-乡镇-村社区

997. Have no idea 不知道
999. Refuse to answer this question 拒绝回答

[CAPI: Drop-down lists are provided for all levels of address, of which the option of "other" is available for choice and corresponding manual inputs are allowed for address level of village 所有层级的地址都是下拉选择，其中村级包括其他选项，且可填写其他的具体名称]

CB063_W3_2_*i* Is [XChildName[*i*]] a member of the Communist Party? [XChildName[*i*]] 现在是不是共产党员?

1. Yes 是
2. No 否

997. Have no idea 不知道
999. Refuse to answer this question 拒绝回答

CB063_W3_5_*i* Does [XChildName[*i*]] have any religious belief? [XChildName[*i*]] 是否有宗教信仰?

1. Yes 是
2. No 否 → Skip CB063_W4_6_*i* 跳过 CB063_W4_6_*i*

997. Have no idea 不知道 → Skip CB063_W4_6_*i* 跳过 CB063_W4_6_*i*
999. Refuse to answer this question 拒绝回答 → Skip CB063_W4_6_*i* 跳过 CB063_W4_6_*i*

CB063_W4_6_*i* What's XChildName[*i*]]'s religious belief? [XChildName[*i*]] 信哪个宗教?

1. Buddhism 佛教
2. Taoism 道教
3. Islam 伊斯兰教
4. Catholicism 天主教
5. Christian 基督教
6. Other, Please specify 其他，请注明：_____ (**CB063_W4_6_1_*i***)

CB070_W4_*i* Is [XChildName[*i*]] a student or does [XChildName[*i*]] work? [XChildName[*i*]] 现在是在工作还是在上学?

[IWER: By definition here, "work" means being a farmer, doing work for salary, self-employed or assisting in family business without getting paid 访员注意：工作包括务农、挣工资的工作、从事个体、私营活动或不拿工资为家庭经营活动帮工等]

1. Working 工作
2. Student 上学
3. A student working part-timely or full-time worker while studying part-timely 边工作边上学
4. Neither a student nor does he work 既不工作也不上学 please specify what he has been doing 请注明现在主要做什么 _____ (**CB070_W4_1_*i***)

997. 不知道
999. 拒绝回答

PROCEDURE 程序:

If CB070_W4_*i* = 1, go on with question CB71_*i*, and then skip to procedures before CB063_*i* 如果 CB070_W4_*i* = 1，则询问 CB71_*i*，然后跳至 CB063_*i* 之前的程序

If CB070_W4_*i* = 2, skip to CB059_W4_*i* 如果 CB070_W4_*i* = 2，则跳至 CB059_W4_*i*

If CB070_W4_*i* = 3, go on with question CB71_*i* and CB059_W4_*i* 如果 CB070_W4_*i* = 3，则继续询问

If CB070_W4_*i* = 4, 997, 999, skip to procedures before CB063_*i* 如果 CB070_W4_*i* = 4, 997, 999，则跳至 CB063_*i* 之前的程序

CB071_*i* What does [XChildName[*i*]] do? [XChildName[*i*]] 现在主要做什么工作?

1. Managers in an enterprise or the persons in charge of general affairs in government agency/ organization of political party/ public service organization 国家机关、党群组织、企业、事业单位负责人
2. Professionals and technicians 专业技术人员
3. Clerks 办事人员和有关人员
4. Workers in business and services 商业、服务业人员
5. Workers in Agriculture, forestry, animal husbandry and fishery 农、林、牧、渔、水利业生产人员
6. Operating personnel of production/transportation equipment 生产、运输设备操作人员及有关人员
7. Others, please specify 其他，请注明：______ (**CB071_1_*i***)

CB059_W4_*i* What kind of education is [XChildName[*i*]] receiving now? [XChildName[*i*]] 现在在上什么学?

1. Preschool education 学前教育
2. Primary school 小学
3. Middle school 初中
4. High school 高中
5. College/ University 大学
6. Graduate school (Master's degree) 硕士研究生
7. Graduate school (Doctoral degree) 博士研究生
8. Others, please specify 其他，请注明：______ (**CB059_W4_1_*i***)

**PROCEDURE** 程序: If [XChildName[*i*]] >16, i.e. XChildBirth[i] $\leq$ 2002, go on with questions CB063_*i* to CB072_W3_*i*, otherwise skip to CB063_W3_1_*i* 如果 [XChildName[*i*]] 超过 16 岁，即 XChildBirth[i] $\leq$ 2002，则询问 CB063_*i* 至 CB072_W3_*i*，否则跳至 CB063_W3_1_*i*

CB063_*i* What's [XChildName[*i*]]'s current marital status? [XChildName[*i*]] 目前的婚姻状况是?

1. Married with spouse present 已婚并与配偶一同居住 → Skip CB063_W4_*i* 跳过 CB063_W4_*i*
2. Married but not living with spouse temporarily for reasons such as work 已婚，但因为工作等原因暂时没有跟配偶在一起居住 → Skip CB063_W4_*i* 跳过 CB063_W4_*i*
3. Separated 分居（不再作为配偶共同生活）
4. Divorced 离异
5. Widowed 丧偶
6. Never married 从未结婚

997. Have no idea 不知道 → Skip to CB065_*i* 跳至 CB065_*i*
999. Refuse to answer this question 拒绝回答 → Skip to CB065_*i* 跳至 CB065_*i*

CB063_W4_*i* Is there anyone who is living together with [XChildName[*i*]] as his/her partner? [XChildName[*i*]] 目前是否有同居伴侣以配偶身份共同生活?

1. Yes 有
2. No 没有

997. Have no idea 不知道
999. Refuse to answer this question 拒绝回答

PROCEDURE 程序:
If [XChildName[i]] has a partner currently, i.e. CB063_*i* = 1, 2 or CB063_W4_*i* = 1, go on with questions CB090_W4_*i* to CB093_W4_*i*, otherwise skip to CB065_*i* 如果 [XChildName[*i*]] 当前有配偶，即 CB063_*i* = 1, 2 或 CB063_W4_*i* = 1，则询问 CB090_W4_*i* 至 CB093_W4_*i*，否则跳至 CB065_*i*

CB090_W4_*i* Where does [XChildName[*i*]]'s partner live? [XChildName[*i*]] 的配偶现在一般住哪儿?
1. Living togher with [XChildName[*i*]] generally 和 [XChildName[*i*]] 一般住在一起
2. Other 其他: _____ (**CB090_W4_1**_*i*) Province 省, _____ (**CB090_W4_2**_*i*) City 市, _____ (**CB090_W4_3**_*i*) County 县, _____ (**CB090_W4_4**_*i*) District/Town 乡/镇/街道, _____ (**CB090_W4_5**_*i*) Village/Community 村/社区

997. Have no idea 不知道
999. Refuse to answer this question 拒绝回答

CB091_W4_*i* Without taking continuing education into account, what's [XChildName[*i*]]'s partner's highest achieved education? 不包括成人教育，[XChildName[*i*]] 的配偶的最高学历是?
1. No formal education (illiterate) 未受过正规教育
2. Did not finish primary school 未读完小学
3. Sishu/home school 私塾
4. Elementary school 小学毕业
5. Middle school 初中毕业
6. High school 高中毕业
7. Vocational school 中专（包括中等师范、职高）毕业
8. Two-/Three-Year College/Associate degree 大专毕业
9. Four-Year College/Bachelor's degree 本科毕业
10. Master's degree 硕士毕业
11. Doctoral degree/Ph.D. 博士毕业

997. Have no idea 不知道
999. Refuse to answer this question 拒绝回答

CB092_W4_*i* Does [XChildName[*i*]]'s partner work? [XChildName[*i*]] 的配偶现在是否工作?
[IWER: By definition here, "work" means being a farmer, doing work for salary, self-employed or assisting in family business without getting paid 访员注意：工作包括务农、挣工资的工作、从事个体、私营活动或不拿工资为家庭经营活动帮工等]
1. Yes 是
2. No 否 please specify what he/she has been doing 那她/他现在做什么?_____ (**CB092_W4_1**_*i*) → Skip CB093_W4_*i* 跳过 CB093_W4_*i*

997. Have no idea 不知道 → Skip CB093_W4_*i* 跳过 CB093_W4_*i*
999. Refuse to answer this question 拒绝回答 → Skip CB093_W4_*i* 跳过 CB093_W4_*i*

CB093_W4_*i* What does [XChildName[*i*]]'s partner do? [XChildName[*i*]] 的配偶现在主要做什么工作?

1. Managers in an enterprise or the persons in charge of general affairs in government agency/ organization of political party/ public service organization 国家机关、党群组织、企业、事业单位负责人
2. Professionals and technicians 专业技术人员
3. Clerks 办事人员和有关人员
4. Workers in business and services 商业、服务业人员
5. Workers in Agriculture, forestry, animal husbandry and fishery 农、林、牧、渔、水利业生产人员
6. Operating personnel of production/transportation equipment 生产、运输设备操作人员及有关人员
7. Others, please specify 其他，请注明：_____ (**CB093_W4_1_*i***)

CB065_*i* How many childern does [XChildName[*i*]] have? [XChildName[*i*]] 有多少个子女? _____ Number 个

[CAPI: The answer could be "don't know" here 这里允许不知道]

**PROCEDURE** 程序:<br>If CB065_*i* = 0, go directly to CB069_*i* 如果 CB065_*i* = 0，则跳至 CB069_*i*

CB066_*i* Of those children, how many are under age of 16? [XChildName[*i*]] 有多少个 16 岁以下的子女? _____ Number 个

[CAPI: The answer could be "don't know" here 这里允许不知道]

CB067_*i* How many grandchildren does [XChildName[*i*]] have? [XChildName[*i*]] 有多少个孙子女? _____ 个

[CAPI: The answer could be "don't know" here 这里允许不知道]

**PROCEDURE** 程序:<br>If CB067_*i* ≥ 1, go on with question CB068_*i* 如果 CB067_*i* ≥ 1, 则询问 CB068_*i*

CB068_*i* Of those grandchildren, how many are under age of 16? [XChildName[*i*]] 有多少个 16 岁以下的孙子女? _____ 个

[CAPI: The answer could be "don't know" here 这里允许不知道]

CB069_*i* To which of the following buckets, does [XChildName[*i*]] and his/her partner's aggregate annual income for last year belong? [XChildName[*i*]] 和他/她的配偶去年的总收入属于下面哪一类?

1. 0 没有收入
2. less than 2000 yuan 少于 2 千元
3. 2000-5000 yuan 2 千与 5 千之间
4. 5000-10000 yuan 5 千与 1 万之间

5. 10000-20000 yuan 1 万与 2 万之间
6. 20000-30000 yuan 2 万与 3 万之间
7. 30000-50000 yuan 3 万与 5 万之间
8. 50000-100000 yuan 5 万与 10 万之间
9. 100000-150000 yuan 10 万与 15 万之间
10. 150000-200000 yuan 15 万与 20 万之间
11. 200000-300000 yuan 20 万与 30 万之间
12. More than 300000 yuan 多于 30 万
997. Have no idea 不知道
999. Refuse to answer this question 拒绝回答

CB071_W3_*i* Does [XChildName[*i*]] have any real estate property under his/her name? [XChildName[*i*]] 有房产吗?

1. Yes 有
2. No 没有 → Skip CB072_W3_*i* 跳过 CB072_W3_*i*
997. Have no idea 不知道 → Skip CB072_W3_*i* 跳过 CB072_W3_*i*
999. Refuse to answer this question 拒绝回答 → Skip CB072_W3_*i* 跳过 CB072_W3_*i*

CB072_W3_*i* How much is this real eatate property worth now? [XChildName[*i*]] 的房产现在值多少钱? ______ (in 10000 yuan) 万元
[CAPI: The answer could be "don't know" here 这里允许不知道]

CB063_W3_1_*i* What would you say about [XChildName[*i*]]'s health status? 请问 [XChildName[*i*]] 现在的身体情况怎么样? 是很好，好，一般，不好还是很不好?

1. Very good 很好
2. Good 好
3. Fair 一般
4. Poor 不好
5. Very poor 很不好
997. Have no idea 不知道
999. Refuse to answer this question 拒绝回答

PROCEDURE 程序:
If [XChildName[*i*]] >=60, i.e. XChildBirth[*i*] $\leq$ 1958, go on with question CB063_W3_6_*i* 如果 [XChildName[*i*]] 超过 60 岁，即 XChildBirth[*i*] $\leq$ 1958，则询问 CB063_W3_6_*i*

CB063_W3_6_*i* Does [XChildName[*i*]] have the ability to take care of him/herself? [XChildName[*i*]] 现在是否能生活自理?

1. Yes 是
2. No 否
997. Have no idea 不知道
999. Refuse to answer this question 拒绝回答

CC Sibling Information 兄弟姐妹信息

CAPI:

CC section is regarding sibling information of [Name of the family respondent] and [Name of spouse of the family respondent] (If still alive). The family respondent and his/her spouse should answer questions regarding their own siblings. Answers to questions in this part will be saved under [Name of the family respondent]'s ID CC 部分询问 [家庭受访者姓名] 以及 [家庭受访者的配偶姓名]（如果其健在）的兄弟姐妹信息。原则上 [家庭受访者姓名] 和 [家庭受访者的配偶姓名] 各自回答自己的兄弟姐妹部分，CC 部分的信息记录在 [家庭受访者姓名] 的个人 ID 下

Codes for sibling of [Name of the family respondent] will follow rules as they are preloaded in CAPI. Any newly mentioned siblings will be coded after the preloaded ones. If the respondent is added to the survey for the first time, the codes for siblings will start from 1 [家庭受访者姓名] 的兄弟姐妹编码继承加载的编码，如果有新增加的兄弟姐妹，在原序号后增加，如果是新受访者，则从 1 开始编号

The following intermediate variables (series) will be generated, with the codes for siblings as subscript 生成下列中间变量（数组），下标为兄弟姐妹编码

- XSibName a string variable, saving names of siblings 字符型，存储兄弟姐妹姓名
- XSibGender drop-down list provided, with 1 denoting male and 2 denoting female, saving genders of siblings 下拉菜单，1 男性，2 女性，存储兄弟姐妹性别
- XSibBirth an integer of 4 digits, saving the birth year of siblings 四位整数型，存储兄弟姐妹出生年份
- XSibType drop-down list provided, with 1 denoting same-parents siblings of [Name of the family respondent], 2 denoting same-father half-blood siblings of [Name of the family respondent], 3 denoting same-mother half-blood siblings of [Name of the family respondent], 4 denoting siblings of [Name of the family respondent] with different father and different mother, 5 denoting "not siblings of [Name of the family respondent]" 下拉菜单，1 与 [家庭受访者姓名] 同父同母，2 与 [家庭受访者姓名] 同父异母，3 与 [家庭受访者姓名] 同母异父，4 与 [家庭受访者姓名] 既不同父也不同母，5 不是 [家庭受访者姓名] 的兄弟姐妹

[INTRO: In the following section, we would like to ask some questions about [Name of the family respondent]'s siblings 引语：下面我想问您一些关于 [家庭受访者姓名] 的兄弟姐妹的问题]

PROCEDURE 程序:

If this household has been already included in our previous survey (XRType = 1), and ZSibNum ≠ 0, skip to CC001_W4; 如果是回访受访者，即 XRType = 1，且 ZSibNum ≠ 0，则跳至 CC001_W4

If this household has been already included in our previous survey (XRType = 1), and ZSibNum = 0, skip to CC002_W4_1 如果是回访受访者，即 XRType = 1，且 ZSibNum = 0，则跳至 CC002_W4_1

If this household is added into the survey for the first time (XRType = 2), questions CC000_W4_1 and CC000_W4 should be asked and then skip to procedures before CC003_W4_1 如果是新受访者，即 XRType = 2，则询问 CC000_W4_1 和CC000_W4，然后跳至 CC003_W4_1 之前的 CAPI

CC000_W4_1 Including biological children and adoptive children of your parents/step-parent, How many siblings does [Name of the family respondent] have? 包括继兄弟姐妹和养兄弟姐妹，[家庭受访者姓名] 一共有多少个兄弟姐妹? ______

PROCEDURE 程序:

If CC000_W4_1 ≥ 1, go on with question CC000_W4, otherwise skip all questions about [Name of the family respondent]'s siblings 如果 CC000_W4_1 ≥ 1，则提问 CC000_W4，否则跳过 [家庭受访者姓名] 的兄弟姐妹部分

CC000_W4 Please ask the respondent the following questions regarding his/her siblings in the order from the oldest to the youngest 访员请按照从大到小的顺序向受访者提问下表中的内容

[CAPI: CC000_W4_1 is the number of rows/siblings; the option 5 "not siblings of [Name of the family respondent]" should not be available for choice for this question 表中显示的需要提问的行数为 CC000_W4_1；此处类型不显示选项 5 不是 [家庭受访者姓名] 的兄弟姐妹]

序号<br>Serial number of siblings	姓名<br>(XSibName)	性别<br>(XSibGender)	出生年份<br>(XSibBirth)	是否在世<br>(XSibAlive)	类型<br>(XSibType)
1					
2					
...					
CC000_W4_1					

CC001_W4 Please confirm with the respondent the following information regarding his/her siblings in the order from the oldest to the youngest; If true, leave it as it is; if false, correct it accordingly in the following table 访员请按照从大到小的顺序向受访者确认下表中的内容，如果正确，无需改动，如果错误，直接在表中修改

[IWER: Please don't skip to questions about another sibling before you finish asking/confirming all information of one particular sibling. Typos or other kinds of errors in sibling names may occur due to misunderstanding of answers given in dialect; in such cases, please check with the respondent carefully before you conclude that this sibling does not exist 访员注意：请将某个兄弟姐妹的所有信息一起与受访者确认，其名字可能因为存在方言等问题，拼写不准确，此时不要轻易判定为不存在该兄弟姐妹]

序号	姓名	性别	出生年份	是否健在	类型
	(ZSibName)	(ZSibGender)	(ZSibBirth)	(ZSibAlive)	(ZSibType)
1					
2					
...					
ZSibNum					

[CAPI: Update the values of relevant Xsib series according to confirmation/ modification; don't generate variables for siblings classified as 5 "not siblings of [Name of the family respondent]" in ZsibType 请根据访员确认及修改后的结果更新相应的 XSib 变量，对于 ZSibType 确认为 "5 不是 [家庭受访者姓名] 的兄弟姐妹" 的兄弟姐妹，不要生成对应的 XSib 变量]

CC002_W4_1 In addition to [XSibName], does [Name of the family respondent] have any other siblings, including biological children and adoptive children of his/her parents/ step-parent? 除了 [XSibName]，[家庭受访者姓名] 还有几个其他的兄弟姐妹？包括继兄弟姐妹和养兄弟姐妹。______ Number 个

**PROCEDURE** 程序:
If CC002_W4_1 ≥ 1, go on with question CC002_W4 如果 CC002_W4_1 ≥ 1, 则提问 CC002_W4

CC002_W4 Please give information about this/ these sibling(s) mentioned in CC002_W4_1 in the order from the oldest to the youngest 请您按照年龄从大到小的顺序告诉我们 [家庭受访者姓名] 这 CC002_W4_1个我们没有记录的兄弟姐妹的信息
[CAPI: The number of rows in the below table is CC002_W4_1; and the serial number for siblings starts from ZSibNum+1 表中显示的需要提问的行数为 CC002_W4_1；兄弟姐妹编号从 ZSibNum+1 开始]

序号	姓名	性别	出生年份	是否健在	类型
	(XSibName)	(XSibGender)	(XSibBirth)	(XSibAlive)	(XSibType)
ZSibNum+1					
ZSibNum+2					
...					
ZSib+CC002_W4_1					

**CAPI**: For each sibling who has been dead, i.e. XSibAlive = 0, questions CC003_W4_1_i to CC003_W4_2_i should be asked iteratively, with i being the loop index 针对每个去世的兄弟姐妹，即 XSibAlive = 0，循环提问 CC003_W4_1_i 至 CC003_W4_2_i，循环指标为 i

**PROCEDURE** 程序:
If ZSibDYear[i] is null, go on with question CC003_W4_2_i 如果 ZSibDYear[i] 为空，则询问 CC003_W4_2_i

CC003_W4_1_*i* When did [ZSibName[*i*]] pass away? [家庭受访者姓名] 的兄弟姐妹 [ZSibName[*i*]] 是什么时候去世的?

______ (**CC003_W4_1_1_*i***) Year 年 or his/her age when he/she died 或去世时 ______ (**CC003_W4_1_2_*i***) Age 岁

PROCEDURE 程序:

If ZSibDReason[*i*] is null, go on with question CC003_W4_2_*i* 如果 ZSibDReason[*i*] 为空，则询问 CC003_W4_2_*i*

CC003_W4_2_*i* What's the leading cause of death for [ZSibName[*i*]]? [家庭受访者姓名] 的兄弟姐妹 [ZSibName[*i*]] 去世的主要原因是什么? ______

[IWER: If the cause of death is illness, please specify the kind of illness. For example, if it is cancer, the specific type of cancer should be recorded (gastric cancer, lung cancer, etc.); if it is infectious disease, please specify the disease (tuberculosis, dysentery, etc.); or if it is an accident, please give information on the accident type (car accident, fire, intoxication, etc.) 访员注意：如果是因疾病死亡，请详细写明病因，例如因癌症去世，要注明是哪种类型的癌症（胃癌、肺癌等）；如果因传染病去世，要写明传染病的类型（结核病、痢疾）；如果是因为事故死亡，要写明事故类型，如车祸、火灾、意外中毒等]

CAPI: For each sibling (both the alive and the dead), questions CC003_W4_5_*i* to CC011_W4_*i* should be asked iteratively, with i being the loop index 针对每个兄弟姐妹（包括健在的和去世的）循环提问 CC003_W4_5_*i* 至 CC011_W4_*i*，循环指标为 *i*

PROCEDURE 程序:

If ZSibEdu[*i*] is not null, go on with question CC003_W4_5_*i* 如果 ZSibEdu[*i*] 非空，则询问 CC003_W4_5_*i*

CC003_W4_5_*i* Without taking continuing education into account, is [ZSibEdu[*i*]] [XSibName[*i*]'s highest achieved education? 不包括成人教育，[家庭受访者姓名] 的兄弟姐妹 [ZSibName[*i*]] 的最高学历是 [ZSibEdu[*i*]]，对吗?

1. Yes 对 → Skip to procedures before CC015_W3_*i* 跳至 CC015_W3_*i* 之前的程序
2. No 不对

CC003_W3_*i* Without taking continuing education into account, what's [ZSibName[*i*]]'s highest achieved education? 不包括成人教育，[家庭受访者姓名] 的兄弟姐妹 [ZSibName[*i*]] 的最高学历是?

1. No formal education (illiterate) 未受过正规教育
2. Did not finish elementary school 未读完小学
3. Sishu/home school 私塾
4. Elementary school 小学毕业
5. Middle school 初中毕业
6. High school 高中毕业
7. Vocational school 中专（包括中等师范、职高）毕业

8. Two-/Three-Year College/Associate degree 大专毕业
9. Four-Year College/Bachelor's degree 本科毕业
10. Master's degree 硕士毕业
11. Doctoral degree/Ph.D. 博士毕业
997. Have no idea 不知道
999. Refuse to answer this question 拒绝回答

PROCEDURE 程序:
If CC003_W3_*i* $= 1, 2, 3$, go on with question CC003_W3_0_*i* 如果 CC003_W3_*i* $= 1, 2, 3$, 则询问 CC003_W3_0_*i*

CC003_W3_0_*i* Is [ZSibName[*i*]] literate? [家庭受访者姓名] 的兄弟姐妹 [ZSibName[*i*]] 是否识字?
1. Yes 是
2. No 否

CC015_W3_*i* What's [ZSibName[*i*]]'s highest achieved position in his/her career? [家庭受访者姓名] 的兄弟姐妹 [ZSibName[*i*]] 做到的职位最高的职业是什么?
1. Managers in an enterprise or the persons in charge of general affairs in government agency/ organization of political party/ public service organization 国家机关、党群组织、企业、事业单位负责人
2. Professionals and technicians 专业技术人员
3. Clerks 办事人员和有关人员
4. Workers in business and services 商业、服务业人员
5. Workers in Agriculture, forestry, animal husbandry and fishery 农、林、牧、渔、水利业生产人员
6. Operating personnel of production/transportation equipment 生产、运输设备操作人员及有关人员
7. Others, please specify 其他，请注明: ______ (**CC015_W3_1_*i***)

CC004_W3_*i* Is [ZSibName[*i*]] a member of the Communist Party? [家庭受访者姓名] 的兄弟姐妹 [ZSibName[*i*]] 是不是共产党员?
1. Yes, Specify the year when he/she became a member of the Communist Party 是，哪一年入党的? ______ (**CC004_W3_1_*i***) Year 年
2. No 否
997. Have no idea 不知道
999. Refuse to answer this question 拒绝回答

[CAPI: An answer of "have no idea" is allowed for the year 入党年份允许不知道]

CC016_W3_*i* Did [ZSibName[*i*]] have the experience to go down to the coutryside as an educated urban youth? [家庭受访者姓名] 的兄弟姐妹 [ZSibName[*i*]] 有没有过上山下乡当知青的经历?
1. Yes, specify the year 有，哪一年上山下乡的? ______ (**CC016_W3_1_*i***) Year 年
2. No 没有 → Skip CC016_W4_*i* 跳过 CC016_W4_*i*

997. Have no idea 不知道 → Skip CC016_W4_*i* 跳过 CC016_W4_*i*
999. Refuse to answer this question 拒绝回答 → Skip CC016_W4_*i* 跳过 CC016_W4_*i*

CC016_W4_*i* Did [ZSibName[*i*]] return to his/her home city/other urban areas after that movement? [家庭受访者姓名] 的兄弟姐妹 [ZSibName[*i*]] 上山下乡当知青后有没有回城?
1. Yes, specify the year 有，哪一年回城的? ______ (**CC016_W4_1_*i***) Year 年
2. No 没有
997. Have no idea 不知道
999. Refuse to answer this question 拒绝回答

CC011_W4_*i* How old was [ZSibName[*i*]] when he/she first married? [家庭受访者姓名] 的兄弟姐妹 [ZSibName[*i*]] 是多大的时候第一次结婚的?
1. ______ (**CC011_W4_a_*i***) At the age of 岁第一次结婚的
2. Never married 从未结婚
997. Have no idea 不知道
999. Refuse to answer this question 拒绝回答

CAPI: For each sibling who is still alive, i.e. XSibAlive = 1, questions CC011_W3_*i* to CC006_W3_2_*i* should be asked iteratively, with *i* being the loop index 针对每个健在的兄弟姐妹（即 XSibAlive = 1）循环提问 CC011_W3_*i* 至 CC006_W3_2_*i*，循环指标为 *i*

CC011_W3_*i* What's [ZSibName[*i*]]'s current marital status? [家庭受访者姓名] 的兄弟姐妹 [ZSibName[*i*]] 目前的婚姻状况是?
1. Married with spouse present 已婚并与配偶一同居住 → Skip CC011_W4_1_*i* 跳过 CC011_W4_1_*i*
2. Married but not living with spouse temporarily for reasons such as work 已婚，但因为工作等原因暂时没有跟配偶在一起居住 → Skip CC011_W4_1_*i* 跳过 CC011_W4_1_*i*
3. Separated 分居（不再作为配偶共同生活）
4. Divorced 离异
5. Widowed 丧偶
6. Never married 从未结婚
997. Have no idea 不知道
999. Refuse to answer this question 拒绝回答

CC011_W4_1_*i* Is there anyone who is living together with [ZSibName[*i*]] as his/her partner? [家庭受访者姓名] 的兄弟姐妹 [ZSibName[*i*]] 目前是否有同居伴侣以配偶身份共同生活?
1. Yes 有
2. No 没有
997. Have no idea 不知道
999. Refuse to answer this question 拒绝回答

CC012_W3_*i* What would you say about [ZSibName[*i*]]'s health status? [家庭受访者姓名] 的兄弟姐妹 [ZSibName[*i*]] 现在的身体情况怎么样? 是很好，好，一般，不好还是很不好?
1. Very good 很好

2. Good 好
3. Fair 一般
4. Poor 不好
5. Very poor 很不好

997. Have no idea 不知道
999. Refuse to answer this question 拒绝回答

CC006_W3_2_*i* Does [ZSibName[*i*]] have the ability to take care of him/herself? [家庭受访者姓名] 的兄弟姐妹 [ZSibName[*i*]] 现在是否能生活自理?

1. Yes 是
2. No 否

997. Have no idea 不知道
999. Refuse to answer this question 拒绝回答

CAPI:
If [Name of spouse of the family respondent] is still alive, we would also like to gather information on [Name of spouse of the family respondent]'s siblings. The questions used are almost the same as those of [Name of the family respondent]'s siblings, except that we would replace [Name of the family respondent] with [Name of spouse of the family respondent] in corresponding questions, choices, prefaces, and logics of procedures, and all generated variables in this part are further subscripted by an "_s" 如果 [家庭受访者的配偶姓名] 健在，将 CC000_W4_1 至 CC006_W3_2_*i* 题干、选项、引语及逻辑中的 [家庭受访者姓名] 替换为 [家庭受访者的配偶姓名]，编写同样的一套题询问 [家庭受访者的配偶姓名] 的兄弟姐妹信息，所有变量名加后缀 s

A Household Member Information 家户成员信息

[INTRO: We would like to know some information on your household members. By definition here, a household member is someone who lives together with you, and with whom you would share family income and expenses 引语：我们想问问您除了您和您配偶外的其他家户成员的情况，家户成员是指和当前您一同居住，且共享生活收支的人]

A001_W4 Of all people listed here, which are your household members? 以下这些人哪些是您的家户成员?（可多选）

[IWER: By definition here, a household member is someone who lives together with you, and with whom you would share family income and expenses. During on-site interview, a particular difficulty is to determine whether a child is a household member of the family respondent or not. A trick that could be used during on-site interview is to inquire the respondent whether the child has been financially indenpendent from him/her 访员注意：家户成员是指和受访者共同居住且共享生活收支的人，在实地比较难以判断的是子女是否是受访者的家户成员，一般可以通过询问受访者是否与某个子女分家等方式来确定]

1-8. CAPI will automatically load all parents of [Name of the family respondent] and [Name of spouse of the family respondent] who are still alive CAPI 加载所有健在的父母姓名

9-33. CAPI will automatically load all children of [Name of the family respondent] and [Name of spouse of the family respondent] who are still alive CAPI 加载所有健在的子女姓名

34-48. CAPI will automatically load all siblings of [Name of the family respondent] and [Name of spouse of the family respondent] who are still alive CAPI 加载所有健在的兄弟姐妹姓名

99. None of people in above list 以上都不是

[CAPI: Updates the list of household members HHMember, in an order which is in accordance with the respondent's answers to A001_W4 更新家户成员列表 HHMember，按照选择的顺序依次加入选中的家户成员]

A002_W4_1 In addition to [HHMember], is there anyone else who is your household member? If any, please specify 除了 [HHMember]，还有哪些人是您的家户成员?

1. ______
2. ______

... ______

[CAPI: Updates the list of household members HHMember once more in accordance with the respondent's answers, adds any new ones sequentially 更新家户成员列表 HHMember，按照选择的顺序依次加入新增加的家户成员，同时标记这些新增加的家户成员]

PROCEDURE 程序:

For each household member added from A002_W4_1, questions A005_W3_*i* to A008_W4_*i* should be asked iteratively, with *i* being the loop index 对于家户成员列表 HHMember 中来自 A002_W4_1 中新增加的家户成员循环提问 A005_W3_*i* 至 A008_W4_*i*，循环变量为 *i*

A005_W3_*i* [HHMember[i]] is a [HHMember[*i*]] 的性别是?

1. Male 男性
2. Female 女性

A005_W4_*i* How old is [HHMember[*i*]] ? [HHMember[*i*]] 今年多大? ______ Age 岁

A006_*i* [HHMember[*i*]] is [Name of the family respondent]'s [HHMember[*i*]] 是 [家庭受访者姓名] 的什么人?

1. Daughter-in-law/ son-in-law 儿媳或女婿
2. Grandchild 孙子或孙女
3. Brother-in-law 姐夫妹夫或嫂子弟媳
4. Father 父亲
5. Mother 母亲
6. Mother-in-law 岳母/婆婆
7. Father-in-law 岳父/公公

8. Child 子女
9. Sibling 兄弟姐妹
10. Carer/driver of this household 保姆或司机
11. Other kind of relative, please specify 其他亲戚，请注明 ______ (**A006_1_*i***)

PROCEDURE 程序:
If A006_*i* = 1, go on with question A007_W4 如果 A006_*i* = 1，则询问 A007_W4

A007_W4_*i* Which child of yours has [HHMember[*i*]] been married with? [HHMember[*i*]] 是您哪个孩子的配偶?

1-25. CAPI will automatically load names of all children, both the alive and dead ones CAPI 加载所有的子女姓名（注意要包括去世的）

99. Other, please specify the name 其他，姓名 ______ (**A007_W4_1_*i***)

PROCEDURE 程序:
If A006_*i* = 2, go on with question A008_W4 如果 A006_*i* = 2，则询问 A008_W4

A008_W4_*i* Who is [HHMember[*i*]]'s parent? [HHMember[*i*]] 是您哪个孩子的孩子?

1-25. CAPI will automatically load names of all children, both the alive and dead ones CAPI 加载所有的子女姓名（注意要包括去世的）

99. Other, please specify the name 其他，姓名 ______ (**A008_W4_1_*i***)

C2 Time Transfer and Transfers 家庭交往与经济帮助

[INTRO: We would then like to ask some questions about interaction within your family, including contact between, financial support and care-giving provided to and received from your/your spouse's parents, children and siblings 引语：接下来我们将询问您与父母、子女间的交往以及家庭成员之间的经济帮助]

CD Time Transfer 与父母、子女间的交往

Contact with Parents 与父母的交往

PROCEDURE 程序:
All relationships are defined from the perspective of [Name of the family respondent] and [Name of spouse of the family respondent]. Questions CD001_W4 to CD002_W4 should only be asked for "parents" who are still alive and not household members. For "parents" who are a couple and at least one of which is household member, the couple is deemed as household members and questions CD001_W4 to CD002_W4 should be skipped 如果[家庭受访者姓名] 和 [家庭受访者的配偶姓名] 没有健在且不是家户成员的亲生父母/养父母，跳至 CD003_W4 之前的程序。如果父母亲两人目前是夫妻且一方是家户成员，则将双方作为一个整体，不做询问。

LOOP:

Loop: Questions CD001_W4 to CD002_W4 should be asked iteratively for “parents” who are still alive and not household members. For each couple: If father and mother are still spouse of each other, the two should be treated as a whole and CAPI preloads both their names in each question; If father and mother are both still alive but no longer spouse of each other, the following questions should be asked separately for each of them; If only one of the couple is alive, the following questions should be asked just for the alive one 循环提问 [家庭受访者姓名] 和 [家庭受访者的配偶姓名] 的健在且不是家户成员的亲生父母/养父母。如果父母亲两人仍然是夫妻，则将二人作为一个整体提问，在 [XConParName] 中调用数组 XParName 里两个人的姓名；如果父母亲健在但不再是夫妻，则对二人分别提问，在 [XConParName] 中调用数组 XParName 里单人的姓名；如果父母亲只有一人健在，则对单人提问，在 [XConParName] 中调用数组 XParName 里单人的姓名.

CD001_W4 With whom is [XConParName] living together? [XConParName] 现在和谁住在一起（可多选）?

1. Living by him/herself 自己单独住
2. Living with his/her spouse/ partner 和他的配偶/伴侣住
3. Living together with [Name of the family respondent] 和 [家庭受访者姓名] 一起住
4. Living with [Name of the family respondent]'s siblings [家庭受访者姓名] 的兄弟姐妹家
5. Living with [Name of spouse of the family respondent]'s siblings [家庭受访者的配偶姓名] 的兄弟姐妹家
6. Living with [Name of the family respondent]'s children [家庭受访者姓名] 的子女家
7. Living with children of [Name of the family respondent]'s sibling's [家庭受访者姓名] 的兄弟姐妹的子女家
8. Living with children of [Name of spouse of the family respondent]'s sibling's [家庭受访者的配偶姓名] 的兄弟姐妹的子女家
9. Living with other relatives 其它亲戚家
10. Livinng in nursing home 养老院
11. Living with someone who is not a relative, please specify 不是亲属关系的其他人，请注明 ______ **(CD001_W4_1)**

PROCEDURE 程序:

If CD001_W4 = 4, go on with question CD001_W3_1 如果 CD001_W4 = 4，即回答“[家庭受访者姓名] 的兄弟姐妹家”，则询问 CD001_W3_1

CD001_W3_1 Of [Name of the family respondent]'s siblings, with whom is [XConParName] living together? (CAPI preloads names of [Name of the family respondent]'s siblings) [XConParName] 现在住在 [家庭受访者姓名] 的哪些兄弟姐妹家?（加载所有的 [家庭受访者姓名] 的兄弟姐妹的名单）

PROCEDURE 程序:

If CD001_W4 = 5, go on with question CD001_W3_2 如果 CD001_W4 = 5，即“[家庭受访者的配偶姓名] 的兄弟姐妹家”，则询问 CD001_W3_2

CD001_W3_2 Of [Name of spouse of the family respondent]'s siblings, with whom is [XConParName] living together? (CAPI preloads names of [Name of spouse of the family respondent]'s siblings) [XConParName] 现在住在 [家庭受访者的配偶姓名] 的哪些兄弟姐妹家?（加载所有 [家庭受访者的配偶姓名] 的兄弟姐妹名单）

PROCEDURE 程序:
If CD001_W4 = 6, go on with question CD001_W3_3 如果 CD001_W4 = 6，即 "[家庭受访者的姓名] 的子女家"，则询问 CD001_W3_3

CD001_W3_3 Of [Name of the family respondent]'s children, with whom is [XConParName] living together? (CAPI preloads names of [Name of the family respondent]'s children) [XConParName] 现在住在 [家庭受访者姓名] 的哪些子女家?（加载所有 [家庭受访者的姓名] 的子女的信息）

PROCEDURE 程序:
If CD001_W4 = 7, go on with question CD001_W3_4 如果 CD001_W4 = 7，即 "[家庭受访者的姓名] 的兄弟姐妹的子女家"，则询问 CD001_W3_4

CD001_W3_4 Of [Name of the family respondent]'s siblings, with whose children, is [XConParName] living together? (CAPI preloads names of [Name of the family respondent]'s siblings) [XConParName] 现在住在 [家庭受访者的姓名] 的哪些兄弟姐妹的子女家?（加载所有 [家庭受访者的姓名] 的兄弟姐妹的名单）

PROCEDURE 程序:
If CD001_W4 = 8, go on with question CD001_W3_5 如果 CD001_W4 = 8，即 "[家庭受访者的配偶姓名] 的兄弟姐妹的子女家"，则询问 CD001_W3_5

CD001_W3_5 Of [Name of spouse of the family respondent]'s siblings, with whose children, is [XConParName] living together? (CAPI preloads names of [Name of spouse of the family respondent]'s siblings) [XConParName] 现在住在 [家庭受访者的配偶姓名] 的哪些兄弟姐妹的子女家?（加载所有 [家庭受访者的配偶姓名] 的兄弟姐妹的信息）

CD002_W4 When [XConParName] is not living with you, how often do you and/or your spouse go visit him/her? 不和您住在一起时，您或您的配偶多长时间去看望一次 [XConParName]?

1. Almost every day 差不多每天
2. 2-3 times a week 每周 2-3 次
3. Once a week 每周一次
4. Every two weeks 每半个月一次
5. Once a month 每月一次
6. Once every three months 每三个月一次
7. Once every six months 半年一次
8. Once a year 每年一次
9. Almost never 几乎从来没有
10. Other 其他

Contact with Children 与子女的交往

PROCEDURE 程序:
For each of [Name of the family respondent]'s children who are still alive, questions CD003_W4 to CD004 should be asked iteratively, with i being loop index 对 [家庭受访者姓名] 的所有健在子女，即 XChildAlive = 1，循环提问 CD003_W4 至 CD004，循环指标为 i

CD003_W4_i During last year, how long had [XChildName[i]] lived with you and your spouse? (in month) 过去一年，[XChildName[i]] 和您以及您的配偶一起居住了多长时间？______ Month 月

[IWER: A short visit does not constitute "living together"; input 0 here if not living together at all and 12 if always living together 访员注意：短暂的走亲戚不算一起居住；没有一起居住请填写 0；一直住在一起请填写 12]

PROCEDURE 程序:
If CD003_W4_i = 12, skip to the next child 如果 CD003_W4_i = 12，则跳至下一个孩子

CD003_i When [XChildName[i]] is not living with you, how often do you contact with him/her on phone/by message/ on wechat/ by mail/ by email? 您和 [XChildName[i]] 不在一起住的时候，您多长时间能见到 [XChildName[i]] 一次?

1. Almost every day 差不多每天
2. 2-3 times a week 每周 2-3 次
3. Once a week 每周一次
4. Every two weeks 每半个月一次
5. Once a month 每月一次
6. Once every three months 每三个月一次
7. Once every six months 半年一次
8. Once a year 每年一次
9. Almost never 几乎从来没有
10. Other 其他

PROCEDURE 程序:
If CD003_i = 1, 2, 3, skip to the next child 如果 CD003_i = 1, 2, 3，则跳至下一个孩子

CD004_i When [XChildName[i]] is not living with you, how often do you contact with him/her on phone/by message/ on wechat/ by mail/ by email? 您和 [XChildName[i]] 不在一起住的时候，您多长时间跟 [XChildName[i]] 通过电话、短信、微信、信件或者电子邮件联系一次?

1. Almost every day 差不多每天
2. 2-3 times a week 每周 2-3 次
3. Once a week 每周一次
4. Every two weeks 每半个月一次
5. Once a month 每月一次
6. Once every three months 每三个月一次
7. Once every six months 半年一次

8. Once a year 每年一次
9. Almost never 几乎从来没有
10. Other 其他

CE Transfers 家庭得到及提供的经济帮助

[INTRO: From time to time, family members will provide help or support each other in various forms, and each form of support matters. And in the following section, here are some questions about the financial support you and your spouse received and provided 引语：有时候，家庭之间会有多种形式的互相帮助，而每一种形式的帮助都很重要。所以，接下来我们想了解一下，您和您配偶有没有从其他人那里得到或者给其他人什么经济帮助]

Financial Support Received from and Provided to Parents 与父母间的经济支持

PROCEDURE 程序:

Questions CE002 to CE023 should be asked iteratively for parents in the list of [XConParName] 根据 [XConParName] 中的列表名单循环提问 CE002 到 CE023

CE002 During last year, what's the amount of financial support received from [XConParName] when he/she/they was/were not living with you? 过去一年，在 [XConParName] 不和您住在一起时，您或您的配偶从 [XConParName] 那里共收到过多少经济支持?

1. Money received in total 总共给钱_____ (**CE002_1**) Yuan 元,of which regular payment was 其中，定期给 _____ (**CE002_2**) Yuan 元 (Regular payment includes providing living expenses, paying for water, electricity or telephone bill, paying for mortgage/rent or other forms of regular expenses)（如定期提供生活费、支付每月水电费及电话费、支付房贷/房租费用或其他定期的费用）
2. In-kind payment received worth in total 总共给物 _____ (**CE002_3**) Yuan 元, of which regular payment was 其中，定期给物 _____ **CE002_4**) Yuan 元 (For example, buying food, clothes or other stuff regularly for you)（如定期提供粮食、买菜、买衣服或其他物品等）

[IWER: Regular payments are payments occurring monthly, quarterly, semi-annually, annually, in cash or in-kind, at some fixed points of time; If nothing received, input 0 here; if an answer of "have no idea" is given or no answer is provided, input "-1" here 访员注意：定期是指按月、按季度、按半年、按年给钱或东西，时间上大致固定；没有给钱或物，请填入"0"，不知道或拒绝回答请填入"-1"]

CE003 If the answer for CE002_1, CE002_2, CE002_3 or CE002_4 is "have no idea" or the respondent refused to answer those questions, please further ask about these questions by giving choices of number buckets (100, 200, 400, 800, 1600 yuan) 请在此处对 CE002_1, CE002_2, CE002_3以及 CE002_4 中回答不知道或拒绝回答的添加分级展开问题（100, 200, 400, 800, 1600 元）

CE022 During last year, what's the amount of financial support provided to [XConParName] when he/she/they was/were not living with you? 过去一年，在 [XConParName] 不和您住在一起时，您或您的配偶给 [XConParName] 多少经济支持?

1. Money received in total 总共给钱_____ (**CE022_1**) Yuan 元,of which regular payment was 其中，定期给 _____ (**CE022_2**) Yuan 元 (Regular payment includes providing living expenses, paying for water, electricity or telephone bill, paying for mortgage/ rent or other forms of regular expenses)（如定期提供生活费、支付每月水电费及电话费、支付房贷/房租费用或其他定期的费用）
2. In-kind payment provided worth in total 总共给物 _____ (**CE022_3**) Yuan 元, of which regular payment was 其中，定期给物 _____ (**CE022_4**) Yuan 元 (For example, buying food, clothes or other stuff regularly for you)（如定期提供粮食、买菜、买衣服或其他物品等）。

[IWER: Regular payments are payments occurring monthly, quarterly, semi-annually, annually, in cash or in-kind, at some fixed points of time; If nothing received, input 0 here; if an answer of "have no idea" is given or no answer is provided, input "-1" here 访员注意：定期是指按月、按季度、按半年、按年给钱或东西，时间上大致固定；没有给钱或物，请填入"0"，不知道或拒绝回答请填入"-1"]

CE023 If the answer for CE022_1, CE022_2, CE022_3 or CE022_4 is "have no idea" or the respondent refused to answer those questions, please further ask about these questions by giving choices of number buckets (100, 200, 400, 800, 1600 yuan) 请在此处对 CE022_1, CE022_2, CE022_3 以及 CE022_4 中回答不知道或拒绝回答的添加分级展开问题（100, 200, 400, 800, 1600 元）

Financial Support Received from and Provided to Children 与子女间的经济支持

**PROCEDURE** 程序: Questions CE009 to CE030 should be asked iteratively for children who are still alive and not household member of [Name of the family respondent] (i.e. XChildAlive = 1 and CD003_W4 < 12) 对 [家庭受访者姓名] 的所有健在且共同居住时间小于 12 个月的子女，即 XChildAlive = 1 且 CD003_W4 < 12，循环提问 CE009 到 CE030

CE009 During last year, what's the amount of financial support received from [XChildName] when he/she was not living with you? 过去一年，在 [XChildName] 不和您住在一起时，您或您的配偶从 [XChildName] 那里收到过多少经济支持?

[IWER: Financial support received from [XChildName]'s children should be included here 访员注意：包括从 [XChildName] 的孩子那里收到的经济支持]

1. Money received in total 总共给钱_____ (**CE009_1**) Yuan 元,of which regular payment was 其中，定期给 _____ (**CE009_2**) Yuan 元 (Regular payment includes providing living expenses, paying for water, electricity or telephone bill, paying for mortgage/ rent or other forms of regular expenses)（如定期提供生活费、支付每月水电费及电话费、支付房贷/房租费用或其他定期的费用）。
2. In-kind payment received worth in total 总共给物 _____ (**CE009_3**) Yuan 元, of which regular payment was 其中，定期给物 _____ (**CE009_4**) Yuan 元 (For example, buying food, clothes or other stuff regularly for you)（如定期提供粮食、买菜、买衣服或其他物品等）。

[IWER: Regular payments are payments occuring monthly, quarterly, semi-annully, annually, in cash or in kind, at some fixed points of time; If nothing received, input 0 here; if an answer of “have no idea” is given or no answer is provided, input “-1” here 访员注意：定期是指按月、按季度、按半年、按年给钱或东西，时间上大致固定；没有给钱或物，请填入“0”，不知道或拒绝回答请填入“-1”]

CE010 If the answer for CE009_1, CE009_2, CE009_3 or CE009_4 is “have no idea” or the respondent refused to answer those questions, please further ask about these questions by giving choices of number buckets (100, 200, 400, 800, 1600 yuan) 请在此处对 CE009_1, CE009_2, CE009_3 以及 CE009_4 中回答不知道或拒绝回答的添加分级展开问题（100, 200, 400, 800, 1600 元）。

CE029 During last year, what’s the amount of financial support provided to [XChildName] when he/she was not living with you? 过去一年，在 [XChildName] 不和您住在一起时，您或您的配偶给 [XChildName] 多少经济支持？

1. Money provided in total 总共给钱 _____ (**CE029_1**) Yuan 元，of which regular payment was 其中，定期给 _____ (**CE029_2**) Yuan 元 (Regular payment includes providing living expenses, paying for water, electricity or telephone bill, paying for mortgage/ rent or other forms of regular expenses)（如定期提供生活费、支付每月水电费及电话费、支付房贷/房租费用或其他定期的费用）。
2. In-kind payment provided worth in total 总共给物 _____ (**CE029_3**) Yuan 元, of which regular payment was 其中，定期给物 _____ (**CE029_4**) Yuan 元 (For example, buying food, clothes or other stuff regularly for you)（如定期提供粮食、买菜、买衣服或其他物品等）。

[IWER: Regular payments are payments occurring monthly, quarterly, semi-annually, annually, in cash or in-kind, at some fixed points of time; If nothing received, input 0 here; if an answer of “have no idea” is given or no answer is provided, input “-1” here] [访员注意：定期是指按月、按季度、按半年、按年给钱或东西，时间上大致固定；没有给钱或物，请填入“0”，不知道或拒绝回答请填入“-1”]

CE030 If the answer for CE029_1, CE029_2, CE029_3 or CE029_4 is “have no idea” or the respondent refused to answer those questions, please further ask about these questions by giving choices of number buckets (100, 200, 400, 800, 1600 yuan) 请在此处对 CE029_1, CE029_2, CE029_3 以及 CE029_4 中回答不知道或拒绝回答的添加分级展开问题（100, 200, 400, 800, 1600 元）。

Financial Support Received from and Provided to Siblings 与兄弟姐妹间的经济支持

CE072_W3 During last year, did you/your spouse ever receive any financial support from or provide financial support to any of your/ your spouse’s siblings? 过去一年，您或您的配偶从以下哪一些兄弟姐妹那里收到过或给过他们经济支持？

1. CAPI preloads names of [Name of the family respondent]’s and [Name of spouse of the family respondent]’s siblings who are still alive 加载 [家庭受访者姓名] 以及 [家庭受访者的配偶姓名] 的所有健在兄弟姐妹名单

99. None of the above 以上都没有 → Skip to CE016_W4 跳至 CE016_W4

PROCEDURE 程序:
Questions CE072_W2 to CE075_W2 should be asked iteratively for each sibling chosen in CE072_W3 对 CE072_W3 中选中的所有兄弟姐妹循环询问 CE072_W2 至 CE075_W2。

CE072_W2 During last year, what's the amount of financial support received from [name of sibling_i]? 过去一年，您或您的配偶从【兄弟姐妹姓名】那里收到过多少经济支持?

1. Money received in total 总共给钱 _____ (**CE072_W2_1**) Yuan 元 (Money received is cash gifts for marriage, funeral, migration to new home, child birth or children schooling, or financial support for illness/ difficulty, but any kind of borrowing should be excluded)（给钱是指婚丧嫁娶、搬迁新房、新生儿、子女升学等情况下的随礼，以及生病、生活困难等情况下的经济资助等，但不包括借钱），of which regular payment was 其中, 定期给 _____ (**CE072_W2_2**) Yuan 元 (Regular payment includes providing living expenses, paying for water, electricity or telephone bill, paying for mortgage/ rent or other forms of regular expenses)（如定期提供生活费、支付每月水电费及电话费、支付房贷/房租费用或其他定期的费用）
2. In-kind payment received worth in total 总共给物 _____ (**CE072_W2_3**) Yuan 元，of which regular payment was 其中，定期给物 _____ (**CE072_W2_4**) Yuan 元 (For example, buying food, clothes or other stuff regularly for you)（如定期提供粮食、买菜、买衣服或其他物品等）。

[IWER: Regular payments are payments occurring monthly, quarterly, semi-annually, annually, in cash or in-kind, at some fixed points of time; If nothing received, input 0 here; if an answer of "have no idea" is given or no answer is provided, input "-1" here 访员注意：定期是指按月、按季度、按半年、按年给钱或东西，时间上大致固定；没有给钱或物，请填入"0"，不知道或拒绝回答请填入"-1"]

CE073_W2 If the answer for CE072_W2_1, CE072_W2_2, CE072_W2_3 or CE072_W2_4 is "have no idea" or the respondent refused to answer those questions, please further ask about these questions by giving choices of number buckets (100, 200, 400, 800, 1600 yuan) 请在此处对 CE072_W2_1，CE072_W2_2，CE072_W2_3 以及 CE072_W2_4 中回答不知道或拒绝回答的添加分级展开问题（100, 200, 400, 800, 1600 元）

CE074_W2 During last year, what's the amount of financial support provided to [name of sibling_i]? 过去一年，您或您的配偶给【兄弟姐妹姓名】过多少经济支持?

1. Money provided in total 总共给钱 _____ (**CE074_W2_1**) Yuan 元 (Money received is cash gifts for marriage, funeral, migration to a new home, childbirth or children schooling, or financial support for illness/ difficulty, but any kind of borrowing should be excluded)（给钱是指婚丧嫁娶、搬迁新房、新生儿、子女升学等情况下的随礼，以及生病、生活困难等情况下的经济资助等，但不包括借钱），of which regular payment was 其中, 定期给 _____ (**CE074_W2_2**) Yuan 元 (Regular payment includes providing living expenses, paying for water, electricity or telephone bill, paying for mortgage/ rent or other forms of regular expenses)（如定期提供生活费、支付每月水电费及电话费、支付房贷/房租费用或其他定期的费用）

2. In-kind payment provided worth in total 总共给物 _____ **(CE074_W2_3)** Yuan 元, of which regular payment was 其中，定期给物 _____ **(CE074_W2_4)** Yuan 元 (For example, buying food, clothes or other stuff regularly for you)（如定期提供粮食、买菜、买衣服或其他物品等）。

[IWER: Regular payments are payments occurring monthly, quarterly, semi-annually, annually, in cash or in-kind, at some fixed points of time; If nothing received, input 0 here; if an answer of "have no idea" is given or no answer is provided, input "-1" here 访员注意：定期是指按月、按季度、按半年、按年给钱或东西，时间上大致固定；没有给钱或物，请填入"0"，不知道或拒绝回答请填入"-1"]

CE075_W2 If the answer for CE074_W2_1, CE074_W2_2, CE074_W2_3 or CE074_W2_4 is "have no idea" or the respondent refused to answer those questions, please further ask about these questions by giving choices of number buckets (100, 200, 400, 800, 1600 yuan) 请在此处对 CE074_W2_1，CE074_W2_2，CE074_W2_3 以及 CE074_W2_4 中回答不知道或拒绝回答的添加分级展开问题（100, 200, 400, 800, 1600 元）。

Financial Support Received from and Provided to Other Relatives and Friends 与其他亲戚朋友间的经济支持

CE016_W4 During last year, how much did you/ your spouse spend in inviting others to dinner for marriage, funeral, migration to new home, childbirth or children schooling? 过去一年，您或您的配偶因为婚丧嫁娶、搬迁新房、新生儿、子女升学等办酒席的花费有多少钱? _____ Yuan 元

[IWER: if no such expense, input 0 here; if the answer is "don't know" or the respondent refused to answer this question, input "-1" here 访员注意：没有办酒席请填入"0"，不知道或拒绝回答请填入"-1"]

CE017_W4 If the answer for CE016_W4 is "have no idea" or the respondent refused to answer this question, please further ask about this question by giving choices of number buckets (500, 1000, 2000, 5000, 10000 yuan) 请在此处对 CE016_W4 中回答不知道或拒绝回答的添加分级展开问题（500, 1000, 2000, 5000, 10000 元）。

CE016_W3 During last year, did you/your spouse ever receive cash gifts from any other relatives or friends except for parents/ children/ siblings? Or any in-kind giving? 过去一年，您或您的配偶从您没住在一起的其他不是父母、子女以及兄弟姐妹的亲戚朋友那里收到过多少礼金? 总共收到钱物 _____ Yuan 元 (gifts in cash or in-kind are gifts for marriage, funeral, migration to new home, childbirth or children schooling)（礼金是指婚丧嫁娶、搬迁新房、新生儿、子女升学等情况下的随礼）。

[IWER: if no such receiving, input 0 here; if the answer is "don't know" or the respondent refused to answer this question, input "-1" here 访员注意：没有收到礼金请填入"0"，不知道或拒绝回答请填入"-1"]

CE017_W3 If the answer for CE016_W3 is “have no idea” or the respondent refused to answer this question, please further ask about this question by giving choices of number buckets (500, 1000, 2000, 5000, 10000 yuan) 请在此处对 CE016_W3 中回答不知道或拒绝回答的添加分级展开问题（500, 1000, 2000, 5000, 10000 元）。

CE036_W3 During last year, did you/your spouse ever give cash gifts to any other relatives or friends except for parents/ children/ siblings? Or any in-kind giving? 过去一年，您或您的配偶给您没住在一起的其他不是父母、子女以及兄弟姐妹的亲戚朋友多少礼金？总共给钱物 ______ Yuan 元 (gifts in cash or in-kind are gifts for marriage, funeral, migration to new home, childbirth or children schooling)（礼金是指婚丧嫁娶、搬迁新房、新生儿、子女升学等情况下的随礼）。
[IWER: if no such receiving, input 0 here; if the answer is “don’t know” or the respondent refused to answer this question, input “-1” here 访员注意：没有给过礼金请填入“0”，不知道或拒绝回答请填入“-1”]

CE037_W3 If the answer for CE036_W3 is “have no idea” or the respondent refused to answer this question, please further ask about this question by giving choices of number buckets (100, 200, 400, 800, 1600 yuan) 请在此处对 CE036_W3 中回答不知道或拒绝回答的添加分级展开问题（100, 200, 400, 800, 1600 元）。

CE016 During last year, in addition to gifts received mentioned above, did you/your spouse ever receive other financial support from any other relatives or friends except for parents/ children/ siblings? Or any in-kind receiving? 过去一年，除了收到的礼金之外，您或您的配偶从您没住在一起的其他不是父母、子女以及兄弟姐妹的亲戚朋友那里收到过多少经济支持？总共给钱物 ______ Yuan 元 (financial support for illness/ difficulty, but any kind of borrowing should be excluded)（给钱包括生病、生活困难等情况下的经济资助等，但不包括借钱。）
[IWER: if no such financial support, input 0 here; if an answer of “have no idea” is given or no answer is provided, input “-1” here 访员注意：没有收到经济支持请填入“0”，不知道或拒绝回答请填入“-1”]

CE017 If the answer for CE016 is “have no idea” or the respondent refused to answer this question, please further ask this question by giving choices of number buckets (100, 200, 400, 800, 1600 yuan) 请在此处对 CE016 中回答不知道或拒绝回答的添加分级展开问题（100, 200, 400, 800, 1600 元）。

CE036 During last year, in addition to gifts given mentioned above, did you/your spouse ever give other financial support to any other relatives or friends except for parents/ children/ siblings? Or any in-kind giving? 过去一年，除了送出的礼金之外，您或您的配偶给您没住在一起的其他不是父母、子女以及兄弟姐妹的亲戚朋友过多少经济支持？总共给钱物 ______ Yuan 元 (financial support for illness/ difficulty, but any kind of lending should be excluded)（给钱包括生病、生活困难等情况下的经济资助等，但不包括借钱）。
[IWER: if no such financial support, input 0 here; if an answer of “have no idea” is given

or no answer is provided, input “-1” here 访员注意：没有给出经济支持请填入“0”，不知道或拒绝回答请填入“-1”]

CE037 If the answer for CE036 is “have no idea” or the respondent refused to answer this question, please further ask this question by giving choices of number buckets (100, 200, 400, 800, 1600 yuan) 请在此处对 CE036 中回答不知道或拒绝回答的添加分级展开问题（100, 200, 400, 800, 1600 元）。

Marriage Gifts 彩礼嫁妆

PROCEDURE:
Questions CE066_W2 to CE070_W2_1 should be asked iteratively for each children who are still alive and ever been married (i.e. XChildAlive = 1 and CB063 = 1, 2, 3, 4, 5) 对每个健在的曾经结过婚的子女，即 XChildAlive = 1 且 CB063 = 1, 2, 3, 4, 5，循环提问 CE066_W2 - CE070_W2_1

CE066_W2 When did [XChildName] first get married? [XChildName] 第一次结婚是什么时候?
_____ [1900...2018] (**CE066_W2_1**) Year 年 ___ [0...12] (**CE066_W2_2**) Month 月

CE067_W2_1 Did you give any marriage gifts when [XChildName] first get married? [XChildName] 第一次结婚时您给彩礼或嫁妆了吗?
1. Yes 是
2. No 否 → Skip to CE069_W2_1 跳至 CE069_W2_1

CE068_W2_1 What were these gifts worth at the price level at that time? 在当时的物价水平下，这些彩礼或嫁妆总共值多少钱? _____ Yuan 元

CE069_W2_1 Did you buy [XChildName] a home when he/she first get married? [XChildName] 第一次结婚时您给买房了吗?
1. Yes 是
2. No 否 → Skip to next child or skip to CF001 for the last child 跳至下一个孩子，如果是最后一个孩子，则跳至 CF001

CE070_W2_1 How much did the house/flat cost you at that time? 买房子花了多少钱? _____ (In 10000 yuan) 万元
[IWER: input the amount in 10000 访员注意：注意这里的金额单位是万元]

CF Time Spent Providing Care 提供照料时间

CF001 During last year, did you/ your spouse spend time in taking care of your grandchildren? 过去一年，您或您的配偶是否花时间照看了您的孙子女以及外孙子女?
1. Yes 是
2. No 否 → Skip to procedures before CF004_W4 跳至 CF004_W4 之前的程序

3. Have no grandchild 没有孙子女以及外孙子女 → Skip to procedures before CF004_W4 跳至 CF004_W4 之前的程序

CF002 During last year, whose children did you take care of? 过去一年，您或您配偶照看了哪些子女的孩子？

1. CAPI preloads names of all children of the respondent, both the alive and dead ones 加载所有子女的姓名，包括健在的和去世的

99. None of the above 以上都没有 → Skip to procedures before CF004_W4 跳至 CF004_W4 之前的程序

PROCEDURE 程序:

For each children name chosen, question CF003 should be asked iteratively 对 CF002 选中的子女名单循环提问 CF003

CF003 During last year, how many weeks did you spend in taking care of [XChildName]'s children? And on average, how many hours per week? 过去一年，您和您配偶大约花几周，每周花多少时间来照看 [XChildName] 的孩子？

I 我： _____ (**CF003_1**) Weeks 周； 一周 _____ (**CF003_2**) Hours per week 小时

My spouse 我爱人： _____ (**CF003_3**) Weeks 周； 一周 _____ (**CF003_4**) Hours per week 小时

[IWER: input 0 here if no time spending in taking care of grandchildren; and 1 if less than 1 week last year and 1 for less than 1 hour per week 访员注意：没有照料请填写"0"，不足一周请填写"1"，一周不足一小时请填写"1"]

[Hardcheck: For answers of greater than 52 weeks or 140 hours per week, please check for any error 如果答案大于 52 周或者大于 140 小时]

PROCEDURE 程序:

For each parent in the list of [XConParName], questions CF004_W4 to CF006_W4 should be asked iteratively 对 [XConParName] 列表名单循环询问 CF004_W4 到 CF006_W4

CF004_W4 During last year, did you/ your spouse ever help [XConParName] with dealing with everyday activities, such as doing household work, cooking, doing laundry, shopping or financial management? 过去一年，[家庭受访者姓名] 或 [家庭受访者的配偶姓名] 有没有在日常活动（或其他活动）方面给 [XConParName] 提供帮助（例如家务劳动，做饭，洗衣，外出，购物和财务管理）？

1. Yes 是
2. No 否 → Skip to the next parent 跳至下一个父母

CF005_W4 During last year, how many weeks did you ([Name of the Family Respondent]) spend in taking care of [XConParName]? And on average, how many hours per week? 过去一年，[家庭受访者姓名] 大约花几周，每周花多少时间来照看 [XConParName]？

_____ (**CF005_W4_1**) Weeks 周； 一周 _____ (**CF005_W4_2**) Hours per week 小时

[IWER: input 0 here if no time spending in taking care of parent; and 1 if less than 1 week

last year and 1 for less than 1 hour per week 访员注意：没有照料请填写“0”，不足一周请填写“1”，一周不足一小时请填写“1”]

[Hardcheck: For answers of greater than 52 weeks or 140 hours per week, please check for any error 如果答案大于 52 周或者大于 140 小时]

CF006_W4 During last year, how many weeks did your spouse ([Name of spouse of the Family Respondent]) spend in taking care of [XConParName]? And on average, how many hours per week? 过去一年，[家庭受访者的配偶姓名] 大约花几周，每周花多少时间来照看 [XConParName]?

______ (**CF006_W4_1**) Weeks 周；一周 ______ (**CF006_W4_2**) Hours per week 小时

[IWER: input 0 here if no time spending in taking care of parent; and 1 if less than 1 week last year and 1 for less than 1 hour per week 访员注意：没有照料请填写“0”，不足一周请填写“1”，一周不足一小时请填写“1”]

[Hardcheck: For answers of greater than 52 weeks or 140 hours per week, please check for any error 如果答案大于 52 周或者大于 140 小时]

CF007_W2 Has the respondent ever turned to others for help when answering the questions in this part? 受访者回答家庭信息部分问卷时是否求助?

[IWER: If the family respondent was assisted by others, please record the respondent's response to such answers; if the family respondent was away from home or couldn't answer questions for other reasons, select 4 here if questions were answered by family respondent's spouse 访员注意：如果是协助回答，请记录受访者的反应。如果家庭受访者不在家或者无法回答问题，而是由其配偶回答的，请选择“4”]

1. Never 从未
2. A few times 偶尔几次
3. Most or all of the time 大多数
4. Proxy by the spouse 由家庭受访者的配偶代理回答

This page intentionally left blank

D Health Status and Functioning 健康状况和功能

**Health Conditions Reported in Last Wave Interview** 上次访问时受访者报告的健康状况	
**Zdisability[*i*]**	If Zdisability[*i*] =1 Yes, R had kind of disabilities listed in DA005 at ZIWTime
**ZDA006[*i*]**	If ZDA006[*i*] =1 Yes, R reported disabled time at ZIWTime
**Zdiagnosed[*i*]**	If Zdiagnosed[*i*] =1 Yes, R had kind of doctor diagnosed disease at ZIWTime
**Zdisease[*i*]**	If Zdisease[*i*] =1 Yes, R had kind of disease at ZIWTime
**ZDA008[*i*]**	If ZDA008[*i*] =1 Yes, R had known R had kind of disease at ZIWTime
**ZDA009[*i*]**	If ZDA009[*i*] =1 Yes, R had answered "When was the condition first dignosed or known by yourself? " at ZIWTime
**Zmenopause**	If Zmenopause =1 Yes, R had started menopause at R's last interview
**ZDA030**	If ZDA030 =1 Yes, R had answered "When was the condition first diagnosed" at ZIWTime
**Zcataract**	If Zcataract =1 Yes, R had cataract surgery at ZIWTime
**ZcataractNUM**	If ZcataractNUM = 1, R had cataract surgery for one eye at ZIWTime
**Zglaucoma**	If Zglaucoma =1 Yes, R had Glaucoma at ZIWTime
**Ztooth**	If Ztooth =1 Yes, R had lost all teeth at ZIWTime
**Zsmoke**	If Zsmoke =1 Yes, R had ever smoked at R's last interview
**ZDA065**	If ZDA065 =1 Yes, R had answered "At what age did you start to smoke on a regular basis?" at ZIWTime
**ZDA069**	"Did you ever drink alcoholic beverages in the past" in last IW?
**ZDA070**	If ZDA070 =1 Yes, R had answered "When did you quit or reduce drinking?" at ZIWTime
**ZDA071**	If ZDA071 =1 Yes, R had answered "When did you start drinking?" at ZIWTime

DA Health Status 健康状况

PART I General Health Status and Disease History 第一部分：一般健康状况和疾病史

DA002 Next, I have some questions about your health. Would you say your health is very good, good, fair, poor or very poor? 下面我将问到一些关于您的健康状况的问题。您认为您的健康状况怎样？是很好，好，一般，不好，还是很不好？

[IWER: Interviewer should read all the following options 访员注意：必须读出所有的选项]

1. Very good 很好
2. Good 好
3. Fair 一般
4. Poor 不好
5. Very poor 很不好

997 Don't know 不知道

PROCEDURE 程序:

If XRType = REIW, ask DA002_W2_1 如果是回访受访者，询问 DA002_W2_1

DA002_W2_1 Compared with your health when we talked with you in R's LAST IW MONTH, YEAR, would you say that your health is better now, about the same, or worse? 与上一次访问时 [加载日期] 您的健康相比，您觉得您的健康状况变好了，差不多，还是变差了？

1. Better 变好
2. About the same 差不多
3. Worse 变差

997 Don't know 不知道

PROCEDURE 程序:

If XRType = REIW who reported disability and the year became disabled in last iw time (Zdisability[i] = 1) and (ZDA006[i] ≠ null), skip to DA007 如果是上期回答有此项残疾的回访受访者且告知了患病时间，开始下一个残疾循环或跳至 DA007

If XRType = REIW who had disability but did not report the year became disabled in last iw time (Zdisability[i] = 1) and (ZDA006[i] = null), skip to DA006_W4 如果是上期回答有此项残疾的回访受访者且没有告知患病时间，跳至 DA006_W4

If this is a new interview R or this is a reinterview R who did not report yes in last wave (Zdisability[i] ≠ 1), go to DA005 如果是新的受访者或上期回答没患有此项残疾的回访受访者询问 DA005

DA005 Do you have one of the following disabilities? 您是否有下列残疾问题？

DA005[1] Do you have Physical disabilities 您是否有躯体残疾？

1. Yes 是
2. No 否

DA005[2] Do you have Brain damage/intellectual disability 您是否有大脑受损/智力缺陷？

1. Yes 是
2. No 否

DA005[3] Do you have Vision problem 您是否有失明或半失明?
1. Yes 是
2. No 否

DA005[4] Do you have Hearing problem 您是否有聋或半聋?
1. Yes 是
2. No 否

DA005[5] Do you have Speech impediment 您是否有哑或严重口吃?
1. Yes 是
2. No 否

PROCEDURE 程序:
If DA005[i] = 1, ask DA006[i] 针对受访者回答患有的每一项残疾问题，依次询问 DA006[i]

DA006[*i*] In what year did you become disabled? 您是什么时候开始患有 DA005[*i*]? ______ Year 年

[IWER: Mark the year using four digits 访员注意：用 4 位数表示年]

→ Skip to procedure before DA007 跳至 DA007 前的程序

DA006_W4 Our records from your last interview show that you have had [Zdisability[i] disability name], In what year did you become disabled? 上一次的访问记录显示您患有 [Zdisability[i] 的残疾名称]，您是什么时候开始患有 [Zdisability[i] 的残疾名称]？

1. ______ (**DA006_W4_1**) Year 年

99. Have never had the disability 从未患有此项残疾

[IWER: Mark the year using four digits. 访员注意：用 4 位数表示年]

PROCEDURE 程序:
If XRType = NEIW or XRType = REIW and REIW R not diagnosed with chronic disease, ask DA007 如果是新的受访者，或者上次访问时未患有医生诊断慢性病的受访者 Zdiagnosed[i] = ., 询问 DA007

DA007 Have you been diagnosed with [conditions listed below, read one by one] by a doctor? 是否有医生曾经告诉过您有以下这些慢性病?

DA007_1_ Have you been diagnosed with Hypertension by a doctor? 是否有医生曾经告诉过您有高血压病?
1. Yes 是
2. No 否

DA007_2_ Have you been diagnosed with Dyslipidemia (elevation of low density lipoprotein, triglycerides (TGs),and total cholesterol, or a low high density lipoprotein level) by a doctor? 是否有医生曾经告诉过您有血脂异常（高血脂或低血脂)?
1. Yes 是
2. No 否

DA007_3_ Have you been diagnosed with Diabetes or high blood sugar by a doctor? 是否有医生曾经告诉过您有糖尿病或血糖升高（包括糖耐量异常和空腹血糖升高）?

1. Yes 是
2. No 否

DA007_4_ Have you been diagnosed with Cancer or malignant tumor (excluding minor skin cancers) by a doctor? 是否有医生曾经告诉过您有癌症等恶性肿瘤（不包括轻度皮肤癌）?

1. Yes 是
2. No 否

DA007_5_ Have you been diagnosed with Chronic lung diseases, such as chronic bronchitis , emphysema (excluding tumors, or cancer) by a doctor? 是否有医生曾经告诉过您有慢性肺部疾患如慢性支气管炎或肺气肿、肺心病（不包括肿瘤或癌）?

1. Yes 是
2. No 否

DA007_6_ Have you been diagnosed with Liver disease (except fatty liver, tumors, and cancer) by a doctor? 是否有医生曾经告诉过您有肝脏疾病（除外脂肪肝、肿瘤或癌）?

1. Yes 是
2. No 否

DA007_7_ Have you been diagnosed with Heart attack, coronary heart disease, angina, congestive heart failure, or other heart problems by a doctor? 是否有医生曾经告诉过您有心脏病（如心肌梗塞、冠心病、心绞痛、充血性心力衰竭和其他心脏疾病）?

1. Yes 是
2. No 否

DA007_8_ Have you been diagnosed with Stroke by a doctor? 是否有医生曾经告诉过您有中风（包括脑梗和脑出血）?

1. Yes 是
2. No 否

DA007_9_ Have you been diagnosed with Kidney disease (except for tumor or cancer) by a doctor? 是否有医生曾经告诉过您有肾脏疾病（不包括肿瘤或癌）?

1. Yes 是
2. No 否

DA007_10_ Have you been diagnosed with Stomach or other digestive diseases (except for tumor or cancer) by a doctor? 是否有医生曾经告诉过您有胃部疾病或消化系统疾病（不包括肿瘤或癌）?

1. Yes 是
2. No 否

DA007_11_ Have you been diagnosed with Emotional, nervous, or psychiatric problems by a doctor? 是否有医生曾经告诉过您有情感及精神方面问题?

1. Yes 是
2. No 否

DA007_12_ Have you been diagnosed with Memory-related disease (such as dementia, brain atrophy, and Parkinson’s disease) by a doctor? 是否有医生曾经告诉过您有与记忆相关

的疾病（如老年痴呆症、脑萎缩、帕金森症）？

1. Yes 是
2. No 否

DA007_13_ Have you been diagnosed with Arthritis or rheumatism by a doctor? 是否有医生曾经告诉过您有关节炎或风湿病？

1. Yes 是
2. No 否

DA007_14_ Have you been diagnosed with Asthma by a doctor? 是否有医生曾经告诉过您有哮喘？

1. Yes 是
2. No 否

PROCEDURE 程序：

If DA007[1,5,11] $\neq$ 1 and ZDA008 $\neq$ 1, ask DA008 对于每个 DA007 的第 1⍰5⍰11 选项中没有回答是且 ZDA008 $\neq$ 1 的疾病，依次询问 DA008

DA008 Do you know if you have [preload the current choice in DA007 [1,5,11]]? 您是否知道自己患有 [依次显示所有 DA007 中第 1，5，11 选项中回答否的选项的疾病名称]？

1. Yes, I know I have this disease 知道自己患有
2. No, I don't I have this disease 知道自己没有
3. I don't know if I have this disease 不知道有没有

PROCEDURE 程序：

If DA007[i] = 1 or DA008[i] = 1, ask DA008_W2_1 如果医生诊断或自我诊断患有慢性病，询问 DA008_W2_1

DA008_W2_1 How did you know that you had had [preload disease], through routine or charls physical examination, or any other? 您是通过何种方式知道自己患有 [加载慢性病] 的，是生病后体检，[加载慢性病] 发作后体检，常规体检，charls 体检还是其他方式？

1. Physical examination after had [preload disease] attack [加载慢性病] 发作后检查
2. Physical examination after had ill 生病后检查
3. Physical examination organized by work unit 单位组织的体检
4. Physical examination organized by community 社区组织的体检
5. CHARLS physical examination charls 体检
6. Other, please specify 其他方式，请注明 _____ (**DA008_W2_1_1**)

DA009 BRANCHPOINT:

If DA007[i] = 1 or DA008[i] = 1 or (Zdisease[i] = 1 and ZDA009[i] = null), ask DA009 如果医生诊断或自我诊断患有慢性病，但不知道第一次诊断出慢性病的时间 (DA007[i] = 1 或 DA008[i] = 1 或 (Zdisease[i] = 1 且 ZDA009[i] = null))，询问 DA009

DA009 When was the condition first diagnosed or known by yourself? 第一次诊断出或者自己知道患有 [加载慢性病] 在什么时候？

1. _____ (**DA009_1**) Year 年或 _____ (**DA009_2**) Age 岁

99. Have never had the disease 从未患有此项疾病

[IWER: Mark the year using four digits 访员注意: 用 4 位数记录年份]

PROCEDURE 程序:

If (DA007[2,5,6,7,9,10,12,13] = 1 or DA008[5] = 1 or Zdisease[2,5,6,7,9,10,12,13] = 1), ask DA010_W4 如果医生诊断或自己知道患有 DA007 中第 2, 5, 6, 7, 9, 10, 12, 13 项慢性病 (DA007[2,5,6,7,9,10,12,13] = 1 或者 DA008[5] = 1 或者 Zdisease[2,5,6,7,9,10,12,13] = 1), 询问 DA010_W4

DA010_W4 Are you now taking any of the following treatments to treat [preload health condition] or its complications (Check all that apply)? Taking Chinese traditional medicine, taking Western modern medicine, other treatments? 您目前有没有正在采用以下方式来治疗 [加载慢性病] 及其并发症 (选择所有采用的方式)? 服用中药, 服用西药, 吃药以外的其他治疗方法?(可多选)

[IWER: Read one by one 访问员注意:请逐项读出以下答案, 并让受访者逐一回答]

1. Taking Chinese traditional medicine 服用中药
2. Taking Western modern medicine 服用西药
3. Other treatments, please specify 吃药以外的其他治疗方法,请注明 ______ **(DA010_W4_1)**
4. None of the above 以上都没有
99. Have never had the disease 从未患有此项疾病

PROCEDURE 程序:

Ask DA010_W2_1 if you have hypertension or diabetes 如果医生诊断或自己知道患有高血压 (DA007[1] = 1 或 DA008[1] = 1 或 Zdisease[1] = 1) 或糖尿病 (DA007[3] = 1 或 Zdisease[3] = 1), 询问 DA010_W2_1

DA010_W2_1 Is your [Blood pressure/ sugar] generally under control? 您的 [血压/血糖] 现在是否控制住了?

1. Yes 是
2. No 否
3. Don't know 不知道
99. Have never had the disease 从未患有此项疾病

PROCEDURE 程序:

If XRType = REIW that had chronic diesease in last wave (Zdisease[i] = 1), ask DA010_W2_2 如果是回访受访者, 上次访问时的确患有慢性病 (Zdisease[i] = 1) , 询问 DA010_W2_2

DA010_W2_2 Compared to when we interviewed you in [ZIWTime], is your [preload heatlh condition] better, about the same as it was then or worse? 与上次访问相比 (加载上期访问时间), 您的 [加载慢性病] 好一些了, 跟原来差不多还是更差?

1. Better 更好
2. Worse 更差

3. About the same 与原来差不多
99. Have never had the disease 从未患有此项疾病

PROCEDURE 程序:
If respondents have hypertension, then ask DA011_W4 - DA013 如果医生诊断或自我诊断患有高血压（DA007[1] = 1 或 DA008[1] = 1 或 Zdisease[1] = 1），询问 DA011_W4 - DA013
If DA010_W2_1[1] = 99 or DA010_W2_2[1] = 99, skip DA011_W4 - DA013 如果 DA010_W2_1[1] 或者 DA010_W2_2[1] 选择 99, 即从未患有此项疾病, 跳过 DA011_W4 - DA013

DA011_W4 Are you now taking any of the following treatments to treat or control your hypertension?(Check all that apply) Taking Chinese traditional medicine, taking Western modern medicine? 您目前有没有正在采用以下方式来治疗控制高血压？服用中药，服用西药？（可多选）
[IWER: Read one by one and let R answer 访问员注意：请逐项读出以下答案，并让受访者逐一回答]
1. Taking Chinese traditional medicine 服用中药
2. Taking Western modern medicine 服用西药
3. Other treatments, please specify 吃药以外的其他治疗方法,请注明 _____ **(DA011_W4_a)**
4. None of the above 以上都没有

[Soft check: If DA011_W4 = 4 "None of the above" and DA010_W2_1[1] = 1, remind IWER "You just said your hypertension is under control. Please confirm whether you are taking any treatments. Or revise the response to DA010_W2_1" 如果DA011_W4 选了"4 以上都没有"，但 DA010_W2_1[1] = 1，提示访员"前面您回答了高血压已经控制住了，请核实是否采用治疗措施，或返回修改 DA010_W2_1 的答案"]

PROCEDURE 程序:
If XRType = NEWIW, ask DA011_W2_1 如果是新增受访者，询问 DA011_W2_1
If XRType = REIW, ask DA011_W4_1 如果是回访受访者，询问 DA011_W4_1

DA011_W2_1 Have you ever had your blood pressure checked by a doctor or nurse? 是否曾有医生或护士给您检查血压？
1. Yes 是
2. No 否 → Skip to DA013 跳至 DA013

DA011_W4_1 Since [ZIWTime], have you had your blood pressure checked by a doctor or nurse? 自上次访问以来 [加载上次访问日期]，是否曾有医生或护士给您检查血压？
1. Yes 是
2. No 否 → Skip to DA013 跳至 DA013

DA011_W2_2 When did you last have it checked? 最近一次检查是什么时候？
_____ **(DA011_w2_2_1)** Year 年 _____ **(DA011_w2_2_2)** Month 月

DA012 During last year (last 12 months), how many times have you had blood pressure examination? 在过去一年（12 个月）中，医生护士给您检查过几次血压？ _____ [0 ... 999] Times 次

DA012_W4 During the last year (last 12 months), have you had blood pressure examination by community/village doctors regularly? 过去一年是否有社区医生/村医经常为您检查血压？

1. Yes 是
2. No 否 → Skip to DA013 跳至 DA013

DA012_W3_1 How often did you have blood pressure examination by community/village doctors? 社区医生/村医多长时间为您检查一次血压？

1. Once a week 一周一次
2. Once half a month 半月一次
3. Once a month 一月一次
4. Once every two months 两月一次
5. Once every three months 一季度一次
6. Once half a year 半年一次
7. Once a year 一年一次

DA012_W3_2 Do you have to pay for the blood pressure examination by community/village doctors? 社区医生/村医为您检查血压需要付费吗？

1. Yes 需要
2. No 不需要

DA013 Have your care providers ever given you health education/advice on the following (check all that apply)? Weight control, exercise, diet and/or smoking control? 有没有医生建议您注意以下问题来控制高血压？（可多选）控制体重，身体锻炼，饮食调理，控烟？
[IWER: Read one by one and let R answer 访问员注意：请逐项读出以下答案，并让受访者逐一回答]

1. Weight control 控制体重
2. Exercise 身体锻炼
3. Diet 饮食调理
4. Smoking control 控烟
5. None of the above 以上都没有

PROCEDURE 程序：

If respondents have diabetes, then ask DA014_W4 - DA016 如果受访者患有糖尿病（DA007[3] = 1 或 Zdisease[3] = 1），询问 DA014_W4 - DA016
if DA010_W2_1[2] = 99 or DA010_W2_2[2] = 99, skip DA014_W4 - DA016 如果 DA010_W2_1[2] 或者 DA010_W2_2[2] 选择 99，即从未患有此项疾病，跳过 DA014_W4 - DA016

DA014_W4 Are you now taking any of the following treatments to treat or control your diabetes? (check all that apply) 您目前有没有正在采用以下方式来治疗控制糖尿病？服用中药，服用西药，注射胰岛素？（可多选）
[IWER: Read one by one 访问员注意：请逐项读出以下答案，并让受访者逐一回答]

1. Taking Chinese traditional medicine 服用中药
2. Taking Western modern medicine 服用西药
3. Taking insulin injections 注射胰岛素
4. Other treatments, please specify 其他治疗方法，请注明 _____ **(DA014_W4_1)**
5. None of the above 以上都没有

[Soft check: If DA014_W4 = 5 "None of the above" and DA010_W2_1[3] = 1, remind IWER "You just said your diabetes is under control. Please confirm whether you are taking any treatments. Or revise the response to DA010_W2_1" 如果 DA014_W4 选了"5 以上都没有"，但 DA010_W2_1[3] = 1，提示访员"前面您回答了糖尿病已经控制住了，请核实是否采用治疗措施，或返回修改 DA010_W2_1 的答案"]

DA015 During last year (last 12 months), how many times have you had the following? 过去一年（12 个月）中，下列各种检测您分别做了几次?

1. Blood glucose test 血糖检测 _____ **(DA015_1**) 0...999 Times 次
2. Urine glucose test 尿糖检测 _____ **(DA015_2**) 0...999 Times 次
3. Fundus examination 眼底检查 _____ **(DA015_3**) 0...999 Times 次
4. Micro-albuminuria test 微蛋白尿检查 _____ **(DA015_4**) 0...999 Times 次
5. None of the above 以上都没有 → Skip to DA016 跳至 DA016

DA016_W4 During the last year (last 12 months), have you had diabetes examination by community/village doctors regularly? 过去一年是否有社区医生/村医经常为您做糖尿病检查?

1. Yes 是
2. No 否 → Skip DA016_W3_1 and DA016_W3_2 跳过 DA016_W3_1和 DA016_W3_2

DA016_W3_1 How often did you have diabetes examination by community/village doctors? 社区医生/村医多长时间为您做一次糖尿病的检查?

1. Once a week 一周一次
2. Once half a month 半月一次
3. Once a month 一月一次
4. Once every two months 两月一次
5. Once every three months 一季度一次
6. Once half a year 半年一次
7. Once a year 一年一次

DA016_W3_2 Do you have to pay for diabetes examination done by community/village doctors? 社区医生/村医为您做糖尿病检查需要付费吗?

1. Yes 需要
2. No 不需要

DA016 Have your care providers ever given you health education/advice on the following? (check all that apply) 有没有医生建议您注意以下问题来控制糖尿病? 控制体重，身体锻炼，饮食调理，控烟，足部自我护理?（可多选）

[IWER: Read one by one and let R answer 访问员注意：请逐项读出以下答案，并让受访者逐一回答]

1. Weight control 控制体重
2. Exercise 身体锻炼
3. Diet 饮食调理
4. Smoking control 控烟
5. Foot self-care 足部自我护理
6. None of the above 以上都没有

PROCEDURE 程序:

If reinterview respondents have heart attack (Zdisease[7] = 1), then ask DA007_W2_5 如果回访受访者患有心脏病（Zdisease[7]=1），回答DA007_W2_5

If DA010_W2_2[7] = 99, skip DA007_W2_5 - DA007_W2_6 如果 DA010_W2_2[7] 选择 99，即从未患此项疾病，跳过 DA007_W2_5 - DA007_W2_6

DA007_W2_5 Since [ZIWTime], have you had a heart attack? 自 [加载上次访问时间] 以来，您是否发作过心脏病?

1. Yes 是
2. No 否 → Skip DA007_W2_6 跳过 DA007_W2_6

DA007_W2_6 When was the (most recent) heart attack? 最近一次发病是什么时候?

_____ (**DA007_W2_6_1**) Year 年 _____ (**DA007_W2_6_2**) Age 岁

PROCEDURE 程序:

If respondents have cancer or maligant tumor (excluding minor skin cancers) (DA007[4] = 1 or Zdisease[4] = 1), ask DA017 and DA018_W4 如果受访者患有癌症或恶性肿瘤（不包括轻度皮肤癌）（DA007[4] = 1 或 Zdisease[4] = 1），询问 DA017 及 DA018_W4

If DA010_W2_2[4] = 99, skip DA017 and DA018_W4 如果 DA010_W2_2[4] 选择 99 从未患此项疾病，跳过 DA017 及 DA018_W4

DA017 In which organ or part of your body do you have cancer? Including the origins and metastasis of tumor.（check all that apply）身体的哪个器官或部位患有或曾经患有癌症？包括原发和已转移的肿瘤。（可多选）

[IWER: Read one by one. We should still ask R even if he/she has already been cured 访员注意：依次读出所有选项，若受访者所患癌症已痊愈，仍需记录]

1. Brain 大脑
2. Oral cavity 口腔
3. Larynx 喉
4. Other pharynx 咽
5. Thyroid 甲状腺
6. Lung 肺
7. Breast 乳房
8. Oesophagus 食管
9. Stomach 胃
10. Liver 肝脏

11. Pancreas 胰腺
12. Kidney 肾脏
13. Prostate 前列腺
14. Testicle 睾丸
15. Ovary 卵巢
16. Cervix 子宫颈
17. Endometrium 子宫内膜
18. Colon or rectum 结肠或直肠
19. Bladder 膀胱
20. Skin 皮肤
21. Non-Hodgkin lymphoma 非何杰金淋巴瘤（非霍奇金淋巴瘤）
22. Leukemia 白血病
23. Other organ 其他器官 ______ **(DA017_1)**

DA018_W4 Have you taken any of the following treatments to treat your cancer or relieve its/their symptoms (e.g., pain, nausea, etc.) in the past two years? (Check all that apply) Taking Chinese traditional medicine, taking Western modern medicine, chemotherapy, surgery, radiation therapy? 在过去的两年中，您有没有采用以下方式来治疗肿瘤或缓解肿瘤所引起的疼痛、恶心等症状（可多选）? 服用中药，服用西药，化学疗法，手术治疗，放射疗法? [IWER: Read one by one and let R answer 访问员注意：请逐项读出以下答案，并让受访者逐一回答]

1. Taking Chinese traditional medicine 服用中药
2. Taking Western morden medicine 服用西药
3. Chemotherapy 化学疗法
4. Surgery 手术治疗
5. Radiation therapy 放射疗法
6. Other treatments, please spcify 其他治疗方法，请注明 ______ **(DA018_W4_1)**
7. None of the above 以上都没有

[F1：(1)“化学疗法”指用化学合成药物治疗疾病的方法。化学药物治疗（简称化疗）是目前治疗肿瘤及某些身免疫性疾病的主要手段之一。
(2)“手术治疗”是最早应用的治疗癌症的方法，也是目前许多早期癌症治疗的首选疗法。
(3)“放射疗法”是用各种不同能量的射线照射肿瘤，以抑制和杀灭癌细胞的一种治疗方法]

PROCEDURE 程序：

If respondents have stroke (DA007[8] = 1 or Zdisease[8] = 1), then ask DA019_W4 如果受访者患有中风（DA007[8] = 1 或 Zdisease[8] = 1），询问 DA019_W4

If DA010_W2_2[8] = 99, skip DA019_W4 - DA019_W2_2 如果 DA010_W2_2[8] 选择 99 从未患此项疾病，跳过 DA019_W4 - DA019_W2_2

DA019_W4 Are you now taking any of the following treatments because of your stroke?(Check all that apply) Taking Chinese traditional medicine, taking Western modern medicine, physical therapy, acupuncture and moxibustion, occupational therapy? 您目前有没有正

在采用以下方式来治疗或控制由于中风引起的痉挛或者其它并发症？服用中药，服用西药，物理治疗，针灸治疗，康复治疗？（可多选）

[IWER: Read one by one and let R answer 访问员注意：请逐项读出以下答案，并让受访者逐一回答]

1. Taking Chinese traditional medicine 服用中药
2. Taking Western morden medicine 服用西药
3. Physical therapy 物理治疗
4. Acupuncture and moxibustion 针灸治疗
5. Occupational therapy 康复治疗
6. Other treatments, please spcify 其他治疗方法，请注明_____ **(DA019_W4_1)**
7. None of the above 以上都没有

[F1：（1）"物理治疗"指主要借着自然界中的物理因子（声光水冷电热力）、运用人体生理学原理法则等，针对人体局部或全身性的功能障碍或病变，施予适当的非侵入性、非药物性治疗来处理患者身体不适和病痛治疗方式，使其尽可能地恢复其原有的生理功能。

（2）"针灸治疗"指针法和灸法的合称。针法是把毫针按一定穴位刺入患者体内，运用捻转与提插等针刺手法来治疗疾病。灸法是把燃烧着的艾绒按一定穴位熏灼皮肤，利用热的刺激来治疗疾病。

（3）"康复治疗"指透过悉心选择而适用于病者的康复活动，帮助在精神、情绪、体能或智能上有障碍的人恢复/增强身体及心智功能，阻止/减轻伤残所带来的影向，使他们能适应工作，社会及家庭上的要求，达至正常和独立的生活]

PROCEDURE 程序：

If reinterview respondents have stroke, then ask DA019_W2_1 and DA019_W2_2 如果回访受访者上次访问的确患有中风（Zdisease[8] = 1），询问 DA019_W2_1 和 DA019_W2_2

DA019_W2_1 Since R's LAST IW MONTH, YEAR, has a doctor told you that you had another stroke? [加载上次访问日期] 自上次访问以来，是否有医生诊断您中风复发？

1. Yes 是
2. No 否 → Skip DA019_W2_2 跳过 DA019_W2_2

DA019_W2_2 When was your most recent stroke? 最近一次中风的诊断时间是？

_____ **(DA019_W2_2_1)** Year 年_____ **(DA019_W2_2_2)** Age 岁

PROCEDURE 程序：

If respondents have emotional, nervous, or psychiatric problems (DA007[11] = 1 or DA008[11] = 1 or Zdisease[11] = 1), then ask DA020_W4 如果受访者患有精神、心理和情感方面的疾病（DA007[11] = 1 或 DA008[11] = 1 或 Zdisease[11] = 1），询问 DA020_W4

If DA010_W2_2[11] = 99 never have this disease, skip DA020_W4 如果 DA010_W2_2[11] 选择 99 从未患此项疾病，跳过 DA020_W4

DA020_W4 Are you now taking any of the following treatments for your emotional, nervous, or psychiatric problems?(Check all that apply) Receiving psychiatric or psychological treatment, taking anti depressants, taking tranquilizers or sleeping pills? 您目前有没有正

在采用以下方式来治疗精神、心理和情感方面的疾病?（可多选）
[IWER: Read one by one and let R answer 访问员注意：请逐项读出以下答案，并让受访者逐一回答]

1. Receiving psychiatric or psychological treatment 接受精神科治疗或心理治疗
2. Taking anti depressants 服用抗抑郁药物
3. Taking tranquilizers or sleeping pills 服用镇静或安眠药物
4. Other treatments, please specify 其他治疗方法，请注明 ______ **(DA020_W4_1)**
5. None of the above 以上都没有

PROCEDURE 程序:
If XRType = NEWIW, ask DA021 如果是新的受访者，询问 DA021
If XRType = REIW, ask DA021_W4 如果是回访受访者，询问 DA021_W4

DA021 Have you ever been in a traffic accident or any other kind of major accidental injury and received medical treatment? 您是否经历过交通事故，或任何的重大意外伤害，并接受了治疗?

1. Yes 是
2. No 否 → Skip to procedure before DA023 跳至 DA023 前的程序

DA021_W4 Have you ever been in a traffic accident or any other kind of major accidental injury and received medical treatment [since R's LAST IW MONTH, YEAR/ in the last two years]? 自上次访问以来 (加载上次访问日期), 您是否经历过交通事故，或任何的重大意外伤害，并接受了治疗?

1. Yes 是
2. No 否 → Skip to procedure before DA023 跳至 DA023 前的程序

DA022 Does your injury caused by the accident limit your daily activities? 事故导致的伤害是否影响您现在的日常活动?

1. Yes 是
2. No 否

PROCEDURE 程序:
If XRType = NEWIW, ask DA023 如果是新的受访者，询问 DA023
If XRType = REIW, ask DA023_W4 如果是回访受访者，询问 DA023_W4

DA023 Have you fallen down? 您有没有摔倒过?

1. Yes 是
2. No 否 → Skip to procedure before DA025 跳至 DA025 前的程序

DA023_W4 Have you fallen down since [ZIWTime]? 自上次访问以来 [加载上次访问日期]，您有没有摔倒过?

1. Yes 是
2. No 否 → Skip to procedure before DA025 跳至 DA025 前的程序

DA024 How many times have you fallen down seriously enough to need medical treatment? 有多少次摔倒受伤严重到需要接受治疗? _____ times 次

PROCEDURE 程序:
If XRType = NEWIW, ask DA025 如果是新的受访者，询问 DA025
If XRType = REIW, ask DA025_W4 如果是回访受访者，询问 DA025_W4

DA025 Have you ever fractured your hip? 您有没有过髋骨骨折?
1. Yes 是
2. No 没有

[F1:“髋骨”指人体腰部的骨骼，共左右两块。幼年时，髋骨分为髂骨,坐骨和耻骨以及软骨连接。成年后，它们之间的软骨会骨化，成为一个整体，即髋骨]

DA025_W4 Have you fractured your hip since we talked in [ZIWTime]? 自上次访问以来 [加载上次访问日期]，您有没有过髋骨骨折?
1. Yes 是
2. No 没有

[F1:“髋骨”指人体腰部的骨骼，共左右两块。幼年时，髋骨分为髂骨,坐骨和耻骨以及软骨连接。成年后，它们之间的软骨会骨化，成为一个整体，即髋骨]

PROCEDURE 程序:
IF R is male, skip to procedure before DA029 男性跳至 DA029 前的程序

DA026 BRANCHPOINT:
If XRType = REIW reported in previous wave that has not started menopause, skip to DA027 如果是回访受访者并且没有绝经 Zmenopause $\neq 1$，跳至 DA027
If XRType = REIW reported in previous wave that has started menopause, skip to DA032 如果回访受访者已绝经 Zmenopause = 1，跳至 DA032
If XRType = NEWIW, ask DA026 - DA028 如果是新的受访者，询问 DA026 - DA028

DA026 When did you begin the menarche? 您什么时候开始来月经的?
1. _____ 1900...2018 (**DA026_1**) Year 年 Or 或 Age 年龄 _____ 1...120 (**DA026_2**) Years 岁
99 Never have menstrual periods 从未来过月经

[IWER: Mark the year using four digits 访员注意: 用 4 位数表示年]

DA027 Have you started menopause? 您绝经了吗?
1. Yes 是
2. No 没有 → Skip to DA032 跳至 DA032
3. Don’t Know 不知道 → Skip to DA032 跳至 DA032

DA028 When did you begin the menopause? 您什么时候开始绝经的?
_____ (**DA028_1**) Year 年 Or 或 Age 年龄 _____ (**DA028_2**) Years 岁 [IWER: Mark the year using four digits 访员注意: 用 4 位数表示年]

PROCEDURE 程序：
If XRType = NEWIW and R is male, ask DA029 如果是新的男性受访者，询问 DA029
If XRType = REIW and R is male, ask DA029_W4 如果是回访男性受访者，询问 DA029_W4

DA029 Have you ever been diagnosed with a prostate illness, such as prostate hyperplasia (excluding prostatic cancer)？有没有医生诊断您得了前列腺疾病如前列腺增生（排除前列腺癌）?
1. Yes 是 → Skip to procedure before DA030 跳至 DA030 前的程序
2. No 没有

DA029_W4 Have you ever been diagnosed with a prostate illness, such as prostate hyperplasia（excluding prostatic cancer）since [ZIWTime]? 上一次访问之后（加载上一次访问时间），有没有医生诊断您得了前列腺疾病如前列腺增生（排除前列腺癌）?
1. Yes 是 → Skip to procedure before DA030 跳至 DA030 前的程序
2. No 没有

[F1：“前列腺增生”主要症状有排尿困难，轻者夜里起床小便次数增多，有尿不净或尿完后还有少量排出的现象；严重者出现尿流变细，甚或排不出的现象；同时常伴有腰酸腰痛、四肢无力、遗精等症状]

DA029_W2_1 Do you know if you had a prostate illness, such as prostate hyperplasia (excluding prostatic cancer)？您是否知道自己患有前列腺疾病如前列腺增生（排除前列腺癌）?
1. Yes 知道自己患有
2. No 知道自己没有 → Skip to DA032 跳至 DA032
3. Don't know 不知道有没有 → Skip to DA032 跳至 DA032

PROCEDURE 程序：
If ZDA030 is not missing, skip DA030 如果 ZDA030 不缺失，跳过 DA030

DA030 When was the condition first diagnosed? 第一次诊断出或您自己知道患有前列腺疾病是在什么时候?
______ (**DA030_1**) Year 年 Or 或 Age 年龄 ______ (**DA030_2**) Years 岁
[IWER: Mark the year using four digits 访员注意: 用 4 位数表示年]

DA031 Are you now taking medication or other treatment for your prostate illness? 您目前是否正在服药或以其他方式治疗前列腺疾病?
1. Yes 是
2. No 否

DA032 Now I have some questions about your eyesight. Do you usually wear glasses or corrective lenses? 下面问您一些关于视力的问题。您通常是否戴眼镜（包括矫正视力镜片）?
1. Yes 是
2. Legally blind 失明 → Skip to DA038_W4 跳至 DA038_W4
3. No 否

4. Sometimes 偶尔

DA033 How good is your eyesight for seeing things at a distance, like recognizing a friend from across the street (with glasses or corrective lenses if you wear them)? Would you say your eyesight for seeing things at a distance is excellent, very good, good, fair, or poor? 您看远处的东西怎么样？比如说能不能隔着马路认出朋友（包括戴着眼镜）。是极好，很好，好，一般还是不好？（访员注意：经常戴眼镜者询问戴眼镜的视力）

1. Excellent 极好
2. Very good 很好
3. Good 好
4. Fair 一般
5. Poor 不好

997 Don't know 不知道

DA034 How good is your eyesight for seeing things up close, like reading ordinary newspaper print (with glasses or corrective lenses if you wear them)? Would you say your eyesight for seeing things up close is excellent, very good, good, fair, or poor? 您看近处的东西怎么样？比如说戴着眼镜能不能看报纸？是极好，很好，好，一般还是不好？（访员注意：经常戴眼镜者询问戴眼镜的视力）

1. Excellent 极好
2. Very good 很好
3. Good 好
4. Fair 一般
5. Poor 不好

997 Don't know 不知道

PROCEDURE 程序：

If XRType = NEWIW, ask DA035 如果是新的受访者，询问 DA035

If XRType = REIW and R reported in last IW that had cataract surgery on one eye (ZcataractNUM = 1), ask DA035_W2_1 如果是回访受访者，上期访问回答一只眼睛做过白内障手术（ZcataractNUM = 1），询问 DA035_W2_1

If XRType = REIW and R did not report in last IW that had cataract surgery (Zcataract ≠ 1), ask DA035_W2_2 如果是回访受访者，上期访问回答没有做过白内障手术，询问 DA035_W2_2

DA035 Have you ever had cataract surgery? 您是否做过白内障手术？

1. Yes 是 → Skip to DA036 跳至 DA036
2. No 否 → Skip to procedure before DA037 跳至 DA037 前的程序

DA035_W2_1 Have you had another cataract surgery since [ZIWTime] other than what you told us about then? 上次访问记录显示您一只眼睛做过白内障，（加载上次访问日期）之后您的另一只眼睛是否做过白内障手术？

1. Yes 是 → Skip to procedure before DA037 跳至 DA037 前的程序
2. No 否 → Skip to procedure before DA037 跳至 DA037 前的程序

DA035_W2_2 Have you had cataract surgery since [ZIWTime]? （加载上次访问日期）自上次访问以来，您是否做过白内障手术?

1. Yes 是
2. No 否 → Skip to procedure before DA037 跳至 DA037 前的程序

DA036 Have you had cataract surgery on both eyes or just one? 白内障手术是做的一只眼还是两只眼?

1. One eye only 一只眼
2. Both eyes 两只眼

PROCEDURE 程序:

If XRType = NEWIW or XRType = REIW and R did not report in last IW that had glaucoma (Zglaucoma ≠ 1), ask DA037 如果是新的受访者或者是上期回答没有得过青光眼的回访受访者（Zglaucoma ≠ 1），询问 DA037

If XRType = REIW and R reported in last IW that had glaucoma (Zglaucoma = 1), ask DA037_W2 如果是上期回答得过青光眼的回访受访者（Zglaucoma = 1）询问 DA037_W2

DA037 Has a doctor/nurse/paramedical/doctor of traditional Chinese medicine doctor ever treated you for glaucoma? 是否有医生诊断过您得了青光眼?

1. Yes 是 → Skip to DA038_W4 跳至 DA038_W4
2. No 否 → Skip to DA038_W4 跳至 DA038_W4

DA037_W2 You told us you had glaucoma, has a doctor/nurse/paramedical/ doctor of traditional Chinese medicine doctor ever treated you for glaucoma relapses since [ZIWTime]? [加载上次访问时间] 上一次的访问记录显示您患有青光眼，之后您的青光眼是否复发?

1. Yes 是
2. No 否
3. Never had glaucoma 一直没有青光眼

DA038_W4 Now I have some questions about your hearing. Do you ever wear a hearing aid? 现在有几个关于听力的问题。您是否通常戴助听器?

1. Yes 是
2. No 否

DA039 Is your hearing very good, good, fair, poor, or very poor (with a hearing aid if you normally use it and without if you normally don't)? Would you say your hearing is excellent, very good, good, fair, or poor? 您的听力如何?（如果您经常戴助听器，那么戴助听器时听力如何? 如果您不经常戴助听器，那么不戴助听器时听力如何?）是极好，很好，好，一般还是不好?

1. Excellent 极好
2. Very good 很好
3. Good 好
4. Fair 一般
5. Poor 不好

997 Don’t know 不知道

PROCEDURE 程序：

If XRType = NEWIW or XRType = REIW that did not lost all teeth in last IW (Ztooth ≠ 1), ask DA040 如果是新的受访者或上期回答牙齿未掉光的回访受访者（Ztooth ≠ 1），询问 DA040

DA040 Have you lost all of your teeth? 您的牙齿是否已经掉光?

1. Yes 是
2. No 否

DA040_W4_1 Do you wear dentures? 您是否佩戴假牙?

1. Yes 是
2. No 否

DA040_W4_2 Have you experienced difficulty chewing solid foods (for example, apples)? (IW: ask the ability to chew with dentures for denture wearers) 有没有因为牙的原因导致无法咀嚼偏硬的食物（如苹果等)?（访员注意：通常戴假牙者询问戴假牙时候的咀嚼能力）

1. Yes 是
2. No 否
3. Not Applicable 不适用

DA041_W4 Are you often troubled with any body pains? 您是否经常因为疼痛而难受？是完全没有、有一点、有一些、比较多、还是非常多?

[IWER: The pain in all parts of R’s body 访员注意：这里询问的是身体所有部位的疼痛情况]

1. None 完全没有 → Skip to DA045 跳至 DA045
2. A little 有一点
3. Somewhat 有一些
4. Quite a bit 比较多
5. Very 非常多

DA042 On what part of your body do you feel pain? Please list all parts of body you are currently feeling pain. 身体哪些部位感到疼痛？请列出所有部位。

1. Head (Headache) 头
2. Shoulder 肩膀
3. Arm 胳膊
4. Wrist 手腕
5. Fingers 手指
6. Chest 胸
7. Stomach (Stomachache) 胃
8. Back 背
9. Waist 腰
10. Buttocks 臀部
11. Leg 腿

12. Knees 膝盖
13. Ankle 脚踝
14. Toes 脚趾
15. Neck 脖子
16. Other, please specify 其它部位，请注明 _____ **(DA042_1)**

DA042_W2_1 Are you taking measures to reduce the pain? (Check all that apply) 您是否采取以下措施以减轻疼痛?（可多选）

1. Taking Chinese traditional medicine 服用中药
2. Taking Western modern medicine 服用西药
3. Acupuncture treatment 针灸疗法
4. Professional massage therapy 推拿按摩
5. Other, please specify 其他 _____ **(DA042_W2_1_1)**
6. None 以上都没有

DA045 Are there any other medical diseases or conditions that are important to your health now that we have not talked about? 是否还有其他严重的疾病或身体不适我没问到?

1. Yes 是
2. No 否 → Skip DA046 跳过 DA046

DA046 What illness is that? 这些疾病是什么? _____

DA048 How would you evaluate your health during childhood, up to and including age 15? Excellent, very good, good,fair, poor? 您 15 岁之前（包括 15 岁）的身体状况怎么样? 极好，很好，好，一般，不好?

1. Excellent 极好
2. Very Good 很好
3. Good 好
4. Fair 一般
5. Poor 不好

997 Don't know 不知道

PART II Lifestyle and Health Behaviors 第二部分：生活方式和健康行为

DA049 During the past month, how many hours of actual sleep did you get at night (average hours for one night)? (This may be shorter than the number of hours you spend in bed.) 过去一个月内，您平均每天晚上真正睡着的时间大约是几小时?（可能短于您在床上躺着的时间）
_____ 0...24 hours 小时

DA050 During the past month, how long did you take a nap after lunch? 过去一个月内，您通常午睡多长时间? _____ Minutes 分钟
[IWER：If R didn't take a nap, please record for 0 访员注意：如果受访者不午睡，请记录为 0]

[INTRO: We would like to know how much time you usually spend on physical activities every week 下面有一些问题是有关您通常每周花了多少时间做这些活动]

CAPI:

Ask DA051 for each type of physical activity, including: 针对以后每种类型的体力活动类型，依次询问 DA051，具体体力活动类型包括：

1、Vigorous-intensity activity (Vigorous activities can cause shortness of breath. Examples of vigorous-intensity activities include carrying heavy stuff, digging, hoeing, aerobic workout, bicycling at a fast speed, riding a cargo bike/motorcycle, etc.) 非常消耗体力的激烈活动（激烈的活动会让你呼吸急促，比如搬运重物、挖地、耕作、有氧运动、快速骑车、骑车载货等）；

2、Moderate activity (Moderate activities can make you breathe faster than usual. Examples of moderate activities include carrying light stuff, bicycling at a normal speed, mopping, Tai-Chi, and speed walking. 中等强度的体力活动（中等体力的活动让您的呼吸比平时快一些，比如搬运轻便的东西、常规速度骑自行车、拖地、打太极拳、疾走）；

3、Mild activities such as walking (walking from one place to another place at a workplace or home, and taking a walk for leisure, sports, exercise or entertainment) 轻度体力活动如走路（走路包括工作或者在家的时候从一个地方走到另一个地方，以及其他您为了休闲、运动、锻炼或娱乐而散步）。

If DA051[i] = 1, ask DA052 to DA051_1 for each type of physical activity. 如果 DA051[i] = 1，针对受访者回答持续做的每一项体力活动类型，依次询问 DA052 至 DA051_1

DA051 Please recall the [preload the type of physical activity] that you have taken part in for at least 10 minutes every time in a week. Do you usually take this type of activity for at least 10 minutes every week? 下面请回忆下您通常每周做的 [加载体力活动类型]，只需回忆您每次运动了至少十分钟的活动，您通常每周有没有至少持续做这种类型的活动十分钟？

1. Yes 是
2. No 否 → Start the next type of physical activity or skip to DA056 开始下一项体力活动循环或跳至 DA056

DA052 How many days a week do you take part in [preload the type of physical activity] for at least 10 minutes? 您通常每周有多少天做 [加载体力活动类型] 至少十分钟？ ______ 1...7 days 天

DA053 During the days you take part in [preload the type of physical activity], how long do you do [preload the type of physical activity] every day? 在做 [加载体力活动类型] 的这些天里，您一天花多少时间做 [加载体力活动类型]？

1. <2 hours 小时
2. ≥ 2 hours 小时 → Skip to DA055 跳至 DA055

DA054 During the days you take part in [preload the type of physical activity], how long do you do [preload the type of physical activity] every day? 在做 [加载体力活动类型] 的这些天里，您一天花多少时间做 [加载体力活动类型]？

1. < 30 minutes 分钟 → Skip to DA051_1 跳至 DA051_1
2. ≥ 30 minutes 分钟 → Skip to DA051_1 跳至 DA051_1

DA055 During the days you take part in [preload the type of physical activity], how long do you do [preload the type of physical activity] every day? 在做 [加载体力活动类型] 的这些天里，您一天花多少时间做 [加载体力活动类型]?

1. < 4 hours 小时
2. ≥ 4 hours 小时

DA051_1 What's the purpose for doing these physical activities, for entertainment, job demand or exercise in doing these physical activities? 是因为工作需要、娱乐活动、体育锻炼还是其他?

1. Job demands 工作需要
2. Entertainments 娱乐
3. Exercise 体育锻炼
4. Other 其他

DA056 Have you done any of these activities in the last month? (Check all that apply) 您过去一个月是否进行了下列社交活动?（可多选）

1. Interacted with friends 串门、跟朋友交往
2. Played Ma-jong, played chess, played cards, or went to community club 打麻将、下棋、打牌、去社区活动室
3. Provided help to family, friends, or neighbors who do not live with you 向与您不住在一起的亲人、朋友或者邻居提供帮助
4. Went to a sport, social, or other kind of club 跳舞、健身、练气功等
5. Took part in a community-related organization 参加社团组织活动
6. Done voluntary or charity work 志愿者活动或者慈善活动
7. Cared for a sick or disabled adult who does not live with you 照顾与您不住在一起的病人或残疾人
8. Attended an educational or training course 上学或者参加培训课程
9. Stock investment 炒股（基金及其他金融证券）
10. Used the Internet 上网
11. Other 其他社交活动
12. None of these 以上均没有

PROCEDURE 程序:

If DA056 = 10, ask DA056_W3 如果 DA056 = 10，询问 DA056_W3

DA056_W3 Which types of devices do you use to access the Internet? (check all that apply) 请问您使用以下哪些工具上网?（可多选）

1. Desktop computer 台式电脑
2. Laptop computer 笔记本电脑
3. Tablet computer (such as IPAD) 平板电脑（如 IPAD）

4. Cellphone 手机
5. Other devices, please specify 其他设备，请注明 _____ （**DA056_W3_1**）

PROCEDURE 程序：
Ask the frequency of each social activity selected in DA056 in the last month in DA057 在 DA057 中循环询问 DA056 中选择的过去一个月参加的每一项社交活动的频率

DA057 How often in the last month [did/have][you] do [preload DA056]? Almost daily, almost every week, or not regularly? 过去一个月，您每隔多长时间会做 [加载DA056]？差不多每天，差不多每周或不经常？
1. Almost daily 差不多每天
2. Almost every week 差不多每周
3. Not regularly 不经常

PROCEDURE 程序：
If DA056 = 10, ask DA056_W4_1 - DA056_W4_4 如果 DA056 = 10，询问 DA056_W4_1 - DA056_W4_4

DA056_W4_1 What do you usually do on the Internet? (check all that apply) 请问您上网一般做什么？（可多选）
1. Chat 聊天
2. Watch news 看新闻
3. Watch videos 看视频
4. Play games 玩游戏
5. Financial management 理财
6. Others, please specify 其他，请注明 _____ （**DA056_W4_1_1**）

DA056_W4_2 Do you use mobile payments, such as Alipay and WeChat pay? 您是否会用手机支付，如支付宝、微信钱包等？
1. Yes 会
2. No 不会

DA056_W4_3 Do you use WeChat? 您是否使用微信？
1. Yes 使用
2. No 不使用 → Skip to branchpoint before DA059 跳至 DA059 前的程序

DA056_W4_4 Do you post WeChat moments? 您发不发微信朋友圈？
1. Yes 发
2. No 不发

[INTRO: Next, I would like to ask whether you have had the habit of smoking cigarettes/smoking a pipe/chewing tobacco, now or in the past. By smoking we mean smoking more than 100 cigarettes in your life 下面我想知道您现在或者过去是否有抽烟、用烟管吸烟或咀嚼烟草的习惯（吸烟指的是一生吸烟 100 支以上）]

DA059 BRANCHPOINT:
If XRType = REIW and R reported ever smoked (Zsmoke = 1), skip to DA061_W4 如果回访受访者吸过烟，跳至 DA061_W4
If XRType = REIW and R did not report ever smoked (Zsmoke ≠ 1), or XRType = NEWIW, ask DA059 如果是从未吸过烟的回访受访者或新的受访者，询问 DA059

DA059 Have you ever chewed tobacco, smoked a pipe, smoked self-rolled cigarettes, or smoked cigarettes/cigars? 您吸过烟吗?（包括香烟、旱烟、用烟管吸烟或咀嚼烟草）
1. Yes 是
2. No 否 → Skip to DA067 跳至 DA067

DA061 Do you still have the habit or have you totally quit? 您现在还在吸烟还是戒烟了?
1. Still have 仍然抽烟 → Skip to DA060 跳至 DA060
2. Quit 戒烟 → Skip to DA060 跳至 DA060

DA061_W4 Our records from your last interview show that you have ever smoked, Do you still have the habit or have you totally quit? 上一次的访问记录显示您曾经吸烟，您现在还在吸烟还是戒烟了?
1. Still have 仍然抽烟
2. Quit 戒烟
3. Never smoked 从未吸过烟 → Skip to DA067 跳至 DA067

DA060 Which products did/do you normally use? 吸烟时，一般抽什么烟?
1. Smoking a pipe 用烟管吸烟（烟袋、旱烟）
2. Smoking self-rolled cigarettes 自己卷烟抽
3. Filtered cigarette 带滤咀香烟
4. Unfiltered cigarette 不带滤咀香烟
5. Cigar 雪茄
6. Water cigarettes 水烟

PROCEDURE 程序:
If R stopped smoking (DA061 = 2 or DA061_W4 = 2), ask DA062 如果 DA061 = 2 戒烟或 DA061_W4 = 2 戒烟，询问 DA062

DA062 At what age did you totally quit smoking? 您这次成功戒烟是在多少岁或那一年?
_____ 1...120 (**DA062_1**) Age 岁 or 或 _____ 1900...2018 (**DA062_2**) Year 年
[IWER: Mark the year using four digits 访员注意: 用 4 位数表示年]

PROCEDURE 程序:
If DA060 = 3, 4, ask DA063 如果 DA060 = 3, 4，询问 DA063

DA063 In one day about how many cigarettes do/did you consume [preload: now/before totally quitting]? 您 [加载：现在/戒烟前] 平均一天抽多少支香烟? _____ Cigarettes 支

PROCEDURE 程序:

If XRType = REIW that has been asked when started smoking, skip DA065 如果回访受访者之前被问到过开始吸烟的年龄 ZDA065 ≠ ., 跳过 DA065

DA065 At what age did you start to smoke on a regular basis? 您是多少岁开始经常抽烟的?
_____ 1...120 (**DA065_1**) Age 岁 Or 或 _____ 1900...2018 (**DA065_2**) Year 年
[IWER: Mark the year using four digits 访员注意: 用 4 位数表示年]

DA067 Did you drink any alcoholic beverages, such as beer, wine, or liquor in the past year? How often? 在过去的一年，您喝酒吗，包括啤酒、葡萄酒、米酒、黄酒或白酒等? 喝酒频率如何?

1. Drink more than once a month 喝酒，每月超过一次
2. Drink but less than once a month 喝酒，但每月少于一次 → Skip to procedure before DA069 跳至 DA069 前的程序
3. None of these 什么都不喝 → Skip to procedure before DA069 跳至 DA069 前的程序

DA068 What type of alcoholic beverages did you drink? Liquor, wine, or beer? (check all that apply) 您平时喝哪种酒? 烈性酒、葡萄酒、啤酒还是其他?（可多选）

1. Liquor, including white liquor, whisky, and others 烈性酒，包括白酒、威士忌和其他酒 → Skip to procedure before DA071 跳至 DA071 前的程序
2. Beer 啤酒 → Skip to procedure before DA071 跳至 DA071 前的程序
3. Wine or rice wine 葡萄酒、米酒或黄酒 → Skip to procedure before DA071 跳至 DA071 前的程序

PROCEDURE 程序:

If XRType = REIW that has never been asked whether drinking alcoholic beverages in the past, ask DA069 如果回访受访者之前没有被问过以前是否喝酒 ZDA069 = ., 询问 DA069
If XRType = REIW that only never had a drink and used to drink a little (ZDA069 = 1 or ZDA069 = 2), skip to DA081. 如果回访受访者之前回答过以前不喝酒或很少喝酒 ZDA069 = 1 或 ZDA069 = 2, 跳至 DA081
If XRType = REIW that used to drink (ZDA069 = 3), skip to the procedure before DA070 如果回访受访者之前回答过以前喝酒 ZDA069 = 3, 跳至 DA070 前的程序

DA069 Did you ever drink alcoholic beverages in the past? How often? 您以前喝酒吗? 喝酒频率如何?

1. I never had a drink. 我从不或极少喝酒 → Skip to DA081 跳至 DA081
2. I used to drink less than once a month. 我很少喝酒，每月少于一次 → Skip to DA081 跳至 DA081
3. I used to drink more than once a month. 我以前喝酒，每月超过一次

PROCEDURE 程序:

If XRType = REIW that has been asked when quitting alcoholic beverages in the past, skip DA070 如果回访受访者之前喝酒 ZDA069 = 3 且被问过何时戒酒的 ZDA070 ≠ ., 跳过 DA070

DA070 When did you quit or reduce drinking? 请问您什么时候戒酒或减少喝酒次数的?
_____ 1900...2018 (**DA070_1**) Year 年 or Age 或年龄: _____1...120 (**DA070_2**) Years 岁
[IWER: Record year in 4 digits 访员注意:年请用 4 位数表示]

**PROCEDURE** 程序:
If XRType = REIW that has been asked when started drinking, skip DA071 如果回访受访者之前被问到过开始饮酒的年龄 ZDA071 ≠ ., 跳过 DA071

DA071 When did you start drinking? 请问您什么时候开始饮酒?
_____ 1900...2018 (**DA071_1**) Year 年 or Age 或年龄 _____ 1...120 (**DA071_2**) Years 岁
[IWER: Record year in 4 digits. 访员注意:年请用 4 位数表示]

[INTRO: Now, I am going to ask you how often and how much you drank during the past year. Please tell me how often you drank per month, and how much you drank at a time on average. I will repeat the questions for different types of alcoholic beverages 现在我想了解过去一年您喝酒的情况。请回答您平均每个月喝多少次酒,一次大概喝多少。我会针对不同的酒类,分别提问]

**PROCEDURE** 程序:
If DA068 = 1, ask DA072 如果 DA068 = 1, 询问 DA072

DA072 How often did you drink liquor, including white liquor, whisky, and others per month in the last year? 过去一年内您平均一个月喝几次烈性酒,包括白酒、威士忌?
1. Once a month 每月一次
2. 2-3 times a month 每月 2-3 次
3. Once a week 每周一次
4. 2-3 times a week 每周 2-3 次
5. 4-6 times a week 每周 4-6 次
6. Once a day 每天一次
7. Twice a day 一天两次
8. More than twice a day 一天超过两次

DA073 The last time you drank liquor last year, how many liang of liquor did you drink? (1 liang = 50 ml) 请问您过去一年内最近一次喝烈性酒, 喝了多少两? (1 两 = 50 毫升) _____ Liang 两

**PROCEDURE** 程序:
If DA068 = 2, ask DA074 如果 DA068 = 2, 询问 DA074

DA074 How many times per month did you drink beer in the last year? 过去一年内您平均一个月喝几次啤酒?
1. Once a month 每月一次
2. 2-3 times a month 每月 2-3 次
3. Once a week 每周一次
4. 2-3 times a week 每周 2-3 次

5. 4-6 times a week 每周 4-6 次
6. Once a day 每天一次
7. Twice a day 一天两次
8. More than twice a day 一天超过两次

DA075 The last time you drank beer last year, how many bottles of beer did you drink? (1 bottle = 2.5 mugs, 1 mug = 220 ml) 请问您过去一年内最近一次喝啤酒，喝了多少瓶?（1 瓶 = 2.5 杯，1 杯 = 220 毫升）

_____ (**DA075_1**) 0...120 Bottles 瓶 or 或者 _____ (**DA075_2**) 0...300 Mugs 杯

PROCEDURE 程序：

If DA068 = 3, ask DA076 如果 DA068 = 3，询问 DA076

DA076 How often did you drink wine or rice wine per month in the last year? 过去一年内您平均一个月喝几次葡萄酒、米酒或黄酒?

1. Once a month 每月一次
2. 2-3 times a month 每月 2-3 次
3. Once a week 每周一次
4. 2-3 times a week 每周 2-3 次
5. 4-6 times a week 每周 4-6 次
6. Once a day 每天一次
7. Twice a day 一天两次
8. More than twice a day 一天超过两次

DA077 The last time you drank it last year, how many liang of wine did you drink? (1 liang = 50 ml) 请问您过去一年内最近一次喝葡萄酒、米酒或黄酒，喝了多少两?（1 两 = 50 毫升）

_____ 0.00...100.00 Liang 两

**INTERVIEWER CHECK AGE OF RESPONDENT?** 访员请检查受访者的年龄。	**1.** < 65 **YEAR** 岁 → **COLUMN A A** 栏<br>**2.** 65 – 69 **YEAR** 岁 → **COLUMN B B** 栏<br>**3.** 70 – 74 **YEAR** 岁 → **COLUMN C C** 栏<br>**4.** 75 – 79 **YEAR** 岁 → **COLUMN D D** 栏<br>**5.** 80 – 84 **YEAR** 岁 → **COLUMN E E** 栏				**6.** 85 – 89 **YEAR** 岁 → **COLUMN F F** 栏<br>**7.** 90 – 94 **YEAR** 岁 → **COLUMN G G** 栏<br>**8.** 95 – 99 **YEAR** 岁 → **COLUMN H H** 栏<br>**9.** ≥ 100 **YEAR** 岁 → **COLUMN I I** 栏			
	A	B	C	D	E	F	G	H	I
AGE 年龄	75 years 岁	80 years 岁	85 years 岁	90 years 岁	95 years 岁	100 years 岁	105 years 岁	110 years 岁	115 years 岁
**DA081** Suppose there are 5 steps, where the lowest step represents the smallest chance and the highest step represents the highest chance, on what step do you think is your chance in reaching the age of [...]? 下面我们想问一下您主观预期寿命的一个问题，这个问题反映了您对自身健康状况的预期。假定有五个级别，最低一级代表可能性最小，最高一级代表可能性最大，您设想您活到这个年龄的可能性有多大。1 Almost impossible 几乎不可能 2 Not very likely 不太可能 3 Maybe 有可能 4 Very likely 很可能 5 Almost certain 简直一定 997 Don't know 不知道	1<br>2<br>3<br>4<br>5	1<br>2<br>3<br>4<br>5	1<br>2<br>3<br>4<br>5	1<br>2<br>3<br>4<br>5	1<br>2<br>3<br>4<br>5	1<br>2<br>3<br>4<br>5	1<br>2<br>3<br>4<br>5	1<br>2<br>3<br>4<br>5	1<br>2<br>3<br>4<br>5

DB Functional Limitations and Helpers 身体功能障碍以及辅助者

PROCEDURE 程序：

If R is younger than 50 (year of birth is after 1968) and if DA002 = 1, 2 and DA005[i] = 2 and Zdisability[i] = 2 and DA007[i] = 2 and DA008[i] = 2 and Zdisease[i] = 2 and DA041_W4 = 1, skip DB001 - DB015 如果受访者年龄小于 50 岁（出生年份在 1968 年之后），并且自我评价健康状况良好、没有残疾、疼痛和慢性病，跳过 DB001 - DB015

[We need to understand difficulties people may have with various activities because of a health or physical problem. Please tell me whether you have difficulty performing any of the following tasks on a regular basis. Exclude any difficulties that you expect to last less than three months 人们可能会由于健康或身体的原因而在多种活动中有困难。请说说您每天做事的时候是否遇到了这些困难（排除那些你预计在三个月内能解决的问题）。每道题目的选项都是一样的，包括没有困难，有困难但仍可以完成，有困难且需要帮助，无法完成。请选择合适的答案]

DB001 Do you have any difficulty with running or jogging about 1 Km? 您现在跑或慢跑 1 公里，有困难吗?

1. No, I don't have any difficulty 没有困难 → Skip to DB004 跳至 DB004
2. I have difficulty but can still do it 有困难但仍可以完成
3. Yes, I have difficulty and need help 有困难，需要帮助
4. I can not do it 无法完成

DB002 Do you have difficulty with walking 1 km? 您现在走 1 公里，有困难吗?

1. No, I don't have any difficulty 没有困难 → Skip DB003 跳过 DB003
2. I have difficulty but can still do it 有困难但仍可以完成
3. Yes, I have difficulty and need help 有困难，需要帮助
4. I can not do it 无法完成

DB003 Do you have difficulty with walking 100 metres? 您走 100 米，有困难吗?

1. No, I don't have any difficulty 没有困难
2. I have difficulty but can still do it 有困难但仍可以完成
3. Yes, I have difficulty and need help 有困难，需要帮助
4. I can not do it 无法完成

DB004 Do you have difficulty with getting up from a chair after sitting for a long period? 您在椅子上坐时间久了再站起来，有困难吗?

1. No, I don't have any difficulty 没有困难
2. I have difficulty but can still do it 有困难但仍可以完成
3. Yes, I have difficulty and need help 有困难，需要帮助
4. I can not do it 无法完成

DB005 Do you have difficulty with climbing several flights of stairs without resting? 您连续不停地爬几层楼，有困难吗?

1. No, I don't have any difficulty 没有困难
2. I have difficulty but can still do it 有困难但仍可以完成
3. Yes, I have difficulty and need help 有困难，需要帮助
4. I can not do it 无法完成

DB006 Do you have difficulty with stooping, kneeling, or crouching? 弯腰、屈膝或者下蹲，您有困难吗?

1. No, I don't have any difficulty 没有困难
2. I have difficulty but can still do it 有困难但仍可以完成
3. Yes, I have difficulty and need help 有困难，需要帮助
4. I can not do it 无法完成

DB007 Do you have difficulty with reaching or extending your arms above shoulder level? (he/she is regarded as not having difficulty only if he/she can extend both of his/her arms, otherwise he/she is regarded as having difficulty.) 您把手臂沿着肩向上伸展，有困难吗?（两个手都没困难才算没困难，否则算有困难）

1. No, I don't have any difficulty 没有困难
2. I have difficulty but can still do it 有困难但仍可以完成
3. Yes, I have difficulty and need help 有困难，需要帮助
4. I can not do it 无法完成

DB008 Do you have difficulty with lifting or carrying weights over 10 jin, like a heavy bag of groceries? 您提 10 斤重的东西，有困难吗?（注意是市斤）

1. No, I don't have any difficulty 没有困难
2. I have difficulty but can still do it 有困难但仍可以完成
3. Yes, I have difficulty and need help 有困难，需要帮助
4. I can not do it 无法完成

DB009 Do you have difficulty with picking up a small coin from a table? 您从桌上拿起一小枚硬币，有困难吗?

1. No, I don't have any difficulty 没有困难
2. I have difficulty but can still do it 有困难但仍可以完成
3. Yes, I have difficulty and need help 有困难，需要帮助
4. I can not do it 无法完成

PROCEDURE 程序：
If (DB001 = 1 & DB004 = 1 ... DB009 = 1), then skip to DB016 如果受访者在 DB001 ~ DB009 没有任何困难，跳至 DB016

[INTRO: Here are a few more everyday activities. Please tell me if you have any difficulties with these because of a physical, mental, emotional or memory problem. Again, exclude any that you expect to last less than three months 下面我们想了解一下您日常生活的情况。请问您目前是否因为身体、精神、情感或者记忆方面的原因导致完成下面我们提到的一些日常行为有困难。我们指的“困难”不包括那些预计三个月内能够解决的困难]

DB010 Because of health and memory problems, do you have any difficulty with dressing? Dressing includes taking clothes out from a closet, putting them on, buttoning up, and fastening a belt. 请问您是否因为健康和记忆的原因，自己穿衣服有困难？穿衣服包括从衣橱中拿出衣服，穿上衣服，扣上钮扣，系上腰带。

1. No, I don't have any difficulty 没有困难 → Skip to DB011 跳至 DB011
2. I have difficulty but can still do it 有困难但仍可以完成
3. Yes, I have difficulty and need help 有困难，需要帮助
4. I can not do it 无法完成

DB010_W2 Does anyone ever help you dress? 穿衣服的时候是否有人帮助你？

1. Yes 有
2. No 没有

DB011 Because of health and memory problems, do you have any difficulty with bathing or showering? 请问您是否因为健康和记忆的原因，洗澡有困难？

1. No, I don't have any difficulty 没有困难 → Skip to DB012 跳至 DB012
2. I have difficulty but can still do it 有困难但仍可以完成
3. Yes, I have difficulty and need help 有困难，需要帮助
4. I can not do it 无法完成

DB011_W2 Does anyone ever help you bathe? 洗澡的时候是否有人帮助你？

1. Yes 有
2. No 没有

DB012 Because of health and memory problems, do you have any difficulty with eating, such as cutting up your food? (Definition: By eating, we mean eating food by oneself when it is ready) 请问您是否因为健康和记忆的原因，自己吃饭有困难，比如自己夹菜？（定义：当饭菜准备好以后，自己吃饭定义为用餐。）

1. No, I don't have any difficulty 没有困难 → Skip to DB013 跳至 DB013
2. I have difficulty but can still do it 有困难但仍可以完成
3. Yes, I have difficulty and need help 有困难，需要帮助
4. I can not do it 无法完成

DB012_W2 Does anyone ever help you eat? 吃饭的时候是否有人帮助你？

1. Yes 有
2. No 没有

DB013 Do you have any difficulty with getting into or out of bed? 您起床、下床有没有困难？

1. No, I don't have any difficulty 没有困难 → Skip to DB014 跳至 DB014
2. I have difficulty but can still do it 有困难但仍可以完成
3. Yes, I have difficulty and need help 有困难，需要帮助
4. I can not do it 无法完成

DB013_W2 Does anyone ever help you get in or out of bed? 起床、下床是否有人帮你?

1. Yes 有
2. No 没有

DB014 Because of health and memory problems, do you have any difficulties with using the toilet, including getting up and down? 请问您是否因为健康和记忆的原因，上厕所有困难，包括蹲下、站起?

1. No, I don't have any difficulty 没有困难 → Skip to DB015 跳至 DB015
2. I have difficulty but can still do it 有困难但仍可以完成
3. Yes, I have difficulty and need help 有困难，需要帮助
4. I can not do it 无法完成

DB014_W2 Does anyone ever help you use the toilet? 上厕所是否有人帮你?

1. Yes 有
2. No 没有

DB015 Because of health and memory problems, do you have any difficulties with controlling urination and defecation? If you use a catheter (conduit) or a pouch by yourself, then you are not considered to have difficulties. 请问您是否因为健康和记忆的原因，控制大小便有困难?（自己能够使用导尿管或者尿袋算能够控制自理）

1. No, I don't have any difficulty 没有困难
2. I have difficulty but can still do it 有困难但仍可以完成
3. Yes, I have difficulty and need help 有困难，需要帮助
4. I can not do it 无法完成

DB016 Because of health and memory problems, do you have any difficulties with doing household chores? (Definition: By doing household chores, we mean house cleaning, doing dishes, making the bed, and arranging the house) 请问您是否因为健康和记忆的原因，做家务活的时候有困难?（定义：做家务，我们指的是房屋清洁，洗碗盘，整理被褥和房间摆设）
[IWER: If R cannot mop the floor, but can scrub, or R cannot fold heavy bedding, but is able to do light ones, then mark (3) 访员注意：如果受访者不能拖地，但是可以擦洗桌子，或者受访者不能整理重的被褥，但是可以整理一些轻便的，请选择（3）]

1. No, I don't have any difficulty 没有困难 → skip to DB017 跳至 DB017
2. I have difficulty but can still do it 有困难但仍可以完成
3. Yes, I have difficulty and need help 有困难，需要帮助
4. I can not do it 无法完成

DB016_W2 Does anyone help you do household chores? 做家务的时候是否有人帮助你?

1. Yes 有
2. No 没有

DB017 Because of health and memory problems, do you have any difficulties with preparing hot meals? (Definition: By preparing hot meals, we mean preparing ingredients, cooking,

and serving food) 请问您是否因为健康和记忆的原因，做饭有困难？（定义：做饭我们定义为准备原材料，做饭菜，端上餐桌）

[IWER: If another person prepares ingredients or if R can cook rice, but is not able to prepare side dishes, then mark (3) 访员注意：如果由于健康原因，受访者需要别人帮忙洗菜切菜，或者受访者只能自己煮米饭但不能做菜，也就是说，由于健康原因受访者只能完成做饭的一些简单的动作，那么选择（3）]

1. No, I don't have any difficulty 没有困难 → skip to DB018 跳至 DB018
2. I have difficulty but can still do it 有困难但仍可以完成
3. Yes, I have difficulty and need help 有困难，需要帮助
4. I can not do it 无法完成

DB017_W2 Does anyone help you prepare hot meals? 做饭的时候是否有人帮助你?

1. Yes 有
2. No 没有

DB018 Because of health and memory problems, do you have any difficulties with shopping for groceries? By shopping, we mean deciding what to buy and paying for it. 请问您是否因为健康和记忆的原因，自己去商店买食品杂货有困难？我们这里说的买东西是指决定买什么和付钱。

1. No, I don't have any difficulty 没有困难 → skip to DB035 跳至 DB035
2. I have difficulty but can still do it 有困难但仍可以完成
3. Yes, I have difficulty and need help 有困难，需要帮助
4. I can not do it 无法完成

DB018_W2 Does anyone help you shop for groceries? 是否有人帮助你去商店买食品杂货等?

1. Yes 有
2. No 没有

DB035 Because of health and memory problems, do you have any difficulties with making phone calls? 请问您是否因为健康和记忆的原因，拨打电话有困难?

1. No, I don't have any difficulty 没有困难 → skip to DB020 跳至 DB020
2. I have difficulty but can still do it 有困难但仍可以完成
3. Yes, I have difficulty and need help 有困难，需要帮助
4. I can not do it 无法完成
5. Not relevant to me (no phone) 不适用，家里没有电话 → skip to DB020 跳至 DB020

DB035_W2 Does anyone help you make telephone calls? 打电话的时候是否有人帮助你?

1. Yes 有
2. No 没有

DB020 Because of health and memory problems, do you have any difficulties with taking medications? By taking medications, we mean taking the right portion of medication right on time. 请问您是否因为健康和记忆的原因，自己吃药有困难？吃药是指能记得什么时间吃和吃多少。

1. No, I don't have any difficulty 没有困难 → skip to DB019 跳至 DB019
2. I have difficulty but can still do it 有困难但仍可以完成
3. Yes, I have difficulty and need help 有困难，需要帮助
4. I can not do it 无法完成

DB020_W2 Does anyone help you take medications? 吃药的时候是否有人帮助你?
1. Yes 有
2. No 没有

DB019 Because of health and memory problems, do you have any difficulties with managing your money, such as paying your bills, keeping track of expenses, or managing assets? 请问您是否因为健康和记忆的原因，管钱有困难，比如支付账单、记录支出项目、管理财物?
1. No, I don't have any difficulty 没有困难 → skip DB019_W2 跳过 DB019_W2
2. I have difficulty but can still do it 有困难但仍可以完成
3. Yes, I have difficulty and need help 有困难，需要帮助
4. I can not do it 无法完成

DB019_W2 Does anyone help you manage your money? 是否有人帮助你管钱?
1. Yes 有
2. No 没有

PROCEDURE 程序：
If DB010_W2 = 1 or DB011_W2 = 1 or DB012_W2 = 1 or DB013_W2 = 1 or DB014_W2 = 1 or DB016_W2 = 1 or DB017_W2 = 1 or DB018_W2 = 1 or DB035_W2 = 1 or DB020_W2 = 1 or DB019_W2 = 1, ask DB022_W3_1; otherwise, skip to DB029

DB022_W3_1 Who most often helps you with [make sure we ask this only once for all these activities; do not ask for each problem separately] (dressing, bathing, eating, getting out of bed, using the toilet, controlling urination and defecation, doing chores, preparing hot meals, shopping, managing money, making phone calls, taking medications) (check all that apply)? 请问在以上（穿衣、洗澡、吃饭、起床、入厕、家务、做饭、购物、打电话、吃药、管钱等）困难中，都有谁帮助您?（多选题）
1. Spouse 配偶
2. Father, Mother, Father-in-law, Mother-in-law 父母、岳父母、公公、婆婆
3. Children, Children's spouses, Grandson, Granddaughter 子女、儿媳/女婿、孙子女/外孙子女
4. Sibling, Brother-in-law, Sister-in-law, Sibling of spouse, Children of sibling, Brother-in-law of spouse, Sister-in-law of spouse, Children of brother-in-law, Children of sister-in-law 兄弟姐妹及其配偶、子女，您配偶的兄弟姐妹及其配偶、子女
5. Other relative 其他亲属
6. Paid helper (such as nanny) 雇佣人员（如保姆），共 ______ **(DB022_W3_1_1)** 位
7. Volunteer 志愿者或者志愿机构人员
8. Employee(s) of facility 养老院人员

9. Community 社区提供的帮助
10. Other, please specify 其他人员，请注明 _____ (**DB022_W4_1**)

[IWER: "employee(s) of facility" appears on list only for an R currently living in a nursing home or who was living in a nursing home or hospice when he/she died 访员注意：养老院人员选项仅针对住在养老院或在养老院去世的受访者]

PROCEDURE 程序：
If DB022_W3_1 = 2, ask DB023_W3_1 如果 DB022_W3_1 = 2, ask DB023_W3_1

DB023_W3_1 Among father, mother, father-in-law, mother-in-law, who help you? (check all that apply) 在父母、岳父母、公公、婆婆中，帮助您的是哪几位？（可多选）

1. Father 父亲
2. Mother 母亲
3. Father-in-law 岳父/公公
4. Mother-in-law 岳母/婆婆

PROCEDURE 程序：
If DB022_W3_1 = 3, ask DB023_W3_2

DB023_W3_2 For the children, children-in-law, grandson, granddaughter who helped you, which children' s family are they from? (check all that apply) 帮助您的子女、儿媳/女婿、孙子女/外孙子女，是以下哪个子女家的？（可多选）

1-25 [Preload children's name] [加载子女姓名]
26 None of the above 以上都没有

PROCEDURE 程序：
For each helper from every child's family, repeat the question DB023_W3_3 对来自每个子女家的帮助者，循环询问 DB023_W3_3

DB023_W3_3 For the family members of [Preload child's name], who help you in person? (check all that apply) [加载子女姓名] 家的哪些人亲自帮助您（可多选）

1 [Preload child's name] himself/herself [加载子女姓名] 本人
2 [Preload child's name] his/her spouse [加载子女姓名] 的配偶
3 [Preload child's name] his/her children. How many helped you in person? [加载子女姓名] 的孩子，即您的（外）孙子女，亲自帮助您的 [加载子女姓名] 的孩子有 _____ 个 (**DB023_W3_3_1**)

PROCEDURE 程序：
If DB022_W3_1 = 4, ask DB023_W3_4 如果 DB022_W3_1 = 4, 询问 DB023_W3_4

DB023_W3_4 For the siblings, spouse and children of siblings, siblings of your spouse, spouse and children of siblings of your spouse who helped you, which siblings' family are they from? 帮助您的兄弟姐妹及其配偶、子女，您配偶的兄弟姐妹及其配偶、子女，是以下哪个兄弟姐妹家的？（多选题）

1-15 [Preload siblings' name] [加载兄弟姐妹的姓名]
16-30 [Preload spouse's siblings' name] [加载配偶的兄弟姐妹的姓名]
99 None of the above 以上都没有

PROCEDURE 程序：
For each helper from every sibling, siblings of spouse, repeat the question DB023_W3_5 对来自每个兄弟姐妹、配偶兄弟姐妹家的帮助者，循环询问 DB023_W3_5

DB023_W3_5 For the family members of [Preload name of siblings, siblings of spouse], who help you in person? (check all that apply) [加载兄弟姐妹姓名/配偶兄弟姐妹的姓名] 家的哪些人亲自帮助您?（可多选）
1 [Preload siblings' (of spouse) name] himself/herself [加载（配偶的）兄弟姐妹姓名] 本人
2 [Preload siblings' (of spouse) name] his/her spouse [加载（配偶的）兄弟姐妹姓名] 的配偶
3 For the children from [Preload siblings' (of spouse) name], how many children from [Preload siblings' (of spouse) name] help you in person? [加载（配偶的）兄弟姐妹姓名] 的孩子, 亲自帮助您的 [加载（配偶的）兄弟姐妹姓名] 的孩子有 _____ 个 **(DB023_W3_5_1)**

PROCEDURE 程序：
If DB022_W3_1 = 5, ask DB023_W3_6 如果 DB022_W3_1 = 5, 询问 DB023_W3_6

DB023_W3_6 The number of the relatives who help you in person 亲自为您提供帮助的其他亲属共有 _____ 位，What's their relationship with you 都是您什么人？ _____ **(DB023_W3_6_1)**

PROCEDURE 程序：
If DB022_W3_1 = 10, ask DB023_W3_7 如果 DB022_W3_1 = 10, 询问 DB023_W3_7

DB023_W3_7 The number of the others who help you in person 亲自为您提供帮助的其他人共有 _____ 位, What's that person's relationship with you 都是您什么人? _____ **(DB023_W3_7_1)**

PROCEDURE 程序：
If the number of helpers larger than 7, ask DB023_W3_9 如果帮助者类型大于 7 个（即选择下列这些变量的数量大于 7：DB022_W3_1 = 1，DB022_W3_1 = 5，DB022_W3_1 = 6, DB022_W3_1 = 7, DB022_W3_1 = 8, DB022_W3_1 = 9, DB022_W3_1 = 10, DB023_W3_1 = 1, DB023_W3_1 = 2, DB023_W3_1 = 3, DB023_W3_1 = 4, DB023_W3_3[i] = 1, DB023_W3_3[i] = 2，DB023_W3_3[i] = 3，DB023_W3_5[i] = 1，DB023_W3_5[i] = 2，DB023_W3_5[i] = 3），询问 DB023_W3_9

DB023_W3_9 From all the helpers list below, please select the most important 7 helpers for you 在下面所列的所有帮助者中，请选择帮助您最多的 7 类人。
Name of all helpers from DB022_W3_1 - DB023_W3_5 按顺序列出 DB022_W3_1 - DB023_W3_5 选择的所有帮助者

PROCEDURE 程序：
For each helper chosen in DB022_W3_1 -DB023_W3_5, ask DB023 - DB025 对 DB022_W3_1 - DB023_W3_5 选择的每一类提供帮助的人，循环提问 DB023 - DB025

DB023 During the last month, on about how many days did [helper] help you? 在过去一个月内，[帮助者] 帮助了您多少天？ _____ 1...31 Days 天

DB024 On the days [helper] helps you, about how many hours per day is that? 在 [帮助者] 帮助您的那些天，他/她大概每天花多少小时帮助您？ _____ 1...24 Hours 小时
[IWER: less than an hour, mark 1 访员注意：少于一个小时请记为 1]

DB025 Is he/she living in your home? 他/她在照顾您的时候，是否和您住在一起？
1. Yes 是
2. No 否

DB029 Do you use the following auxiliary? (Check all that apply) 你使用以下辅助工具么？（可多选）
1. Walking stick 拐杖
2. Travel device 代步器
3. Manual wheelchair 手动轮椅
4. Electric Wheelchair 电动轮椅
5. Catheter, urine collection bag 导尿管，导尿袋
6. Toilet Series 便携式座便器
7. None of the above 以上都没有

DB030 Suppose that in the future, you needed help with basic daily activities like eating or dressing. Do you have relatives or friends (besides your spouse/partner) who would be willing and able to help you over a long period of time? 如果以后您在日常生活方面需要照顾，比如吃饭，穿衣，有亲人或朋友能长期照顾您吗？
1. Yes 是
2. No 否 → Skip to DB036_W4 跳至 DB036_W4

DB031 What is the relationship to you of that person or those persons? （Check all that apply） 他/她是您的什么人？（可多选）
1. Spouse 配偶
2. Father, Mother, Father-in-law, Mother-in-law 父母、岳父母、公公、婆婆
3. Children, Children's spouses, Grandson, Granddaughter 子女、儿媳/女婿、孙子女/外孙子女
4. Sibling, Brother-in-law, Sister-in-law, Sibling of spouse, Children of sibling, Brother-in-law of spouse, Sister-in-law of spouse, Children of brother-in-law, Children of sister-in-law 兄弟姐妹及其配偶、子女，您配偶的兄弟姐妹及其配偶、子女
5. Other relative 其他亲属
6. Paid helper (such as nanny) 雇佣人员（如保姆）

7. Volunteer 志愿者或者志愿机构人员
8. Employee(s) of facility 养老院人员
9. Community 社区提供的帮助
10. Other, please specify 其他人员，请注明 ______ **(DB031_1)**

> **PROCEDURE** 程序：
> If DB031 = 2, ask DB031_W3_1

DB031_W3_1 Father, mother, father-in-law, mother-in-law, who will help you in future? 在父母、岳父母、公公、婆婆中，以后会帮助您的是哪些?（可多选）

1. Father 父亲
2. Mother 母亲
3. Father-in-law 岳父/公公
4. Mother-in-law 岳母/婆婆

> **PROCEDURE** 程序：
> If DB031 = 3, ask DB031_W3_2 如果 DB031 = 3， 询问 DB031_W3_2

DB031_W3_2 For the children, children-in-law, grandchildren who will help you in future, which children's family are they from? 以后会帮助您的子女、儿媳/女婿、孙子女/外孙子女，是以下哪个子女家的?（可多选）

1-25 [Preload children's name] [加载子女姓名]
26 None of the above 以上都没有

> **PROCEDURE** 程序：
> If DB031 = 4, ask DB031_W3_4 如果DB031 = 4, 询问 DB031_W3_4

DB031_W3_4 For the siblings, spouse and children of siblings, spouse's siblings, spouse and children of spouse's siblings who will help you in future, which children's family are they from? 以后会帮助您的兄弟姐妹及其配偶、子女，您配偶的兄弟姐妹及其配偶、子女，是以下哪个兄弟姐妹家的?（多选题）

1-15 [Preload siblings' name] [加载兄弟姐妹的姓名]
16-30 [Preload siblings' name of spouse] [加载配偶的兄弟姐妹的姓名]
99 None of the above 以上都没有

> **PROCEDURE** 程序：
> If DB031 = 5, ask DB031_W3_6 如果 DB031 = 5, 询问 DB031_W3_6

DB031_W3_6 The number of other relatives who will help you in person in future 以后亲自为您提供帮助的其他亲属共有 ______ 位

> **PROCEDURE** 程序：
> If DB031 = 10, ask DB031_W3_7 如果 DB031 = 10, 询问 DB031_W3_7

DB031_W3_7 The number of others who will help you in person in future 以后亲自为您提供帮助的其他人共有 ______ 位

[INTRO: We would like to know whether your health problems limit your ability to work 下面我们想知道，您有没有因为身体原因影响到您的工作能力。]

DB036_W4 Do you think the following description fits you: I cannot work because of my disability or health problems 您看我这句话是否符合您的情况：因为残疾或健康原因，我无法正常工作或劳动。

1. I am unable to work 我完全无法正常工作或劳动 → Skip to DB032 跳至 DB032
2. I cannot work long hours 我不能长时间工作或劳动
3. I don't have any problem 我做起来没有问题

DB037_W4 Do you think the following description fits you: I cannot do housework because of my disability or health problems. 您看我这句话是否符合您的情况：因为残疾或健康原因，我无法正常做家务。

1. I am unable to do housework 我完全无法正常做家务
2. I cannot do housework for an extended period of time 我不能长时间做家务
3. I don't have any problem 我做起来没有问题

DB032 How often did the respondent receive assistance in answering this section 受访者填写该部分问卷时是否求助?

[IWER: If it is answered by a proxy, please record the respondent's reaction 访员注意：如果是协助回答，请记录受访者的反应]

1. Never 从不
2. A few times 有一些时候
3. Most or all of the time 大多数时候
4. The section was completed by a proxy respondent（the respondent is absent）受访者不在场，完全请人代填 → Skip to DB033 跳至 DB033

DB033 What is your relationship to R? 您和受访者是什么关系?

[IWER: What is the proxy's relationship to R? If unknown, please ask the proxy 访员注意：代填问卷的人和受访者是什么关系。如果不清楚，请问代填者]

1. Spouse 配偶
2. Mother 母亲
3. Father 父亲
4. Mother-in-law 岳母/婆婆
5. Father-in-law 岳父公公
6. Sibling 兄弟姐妹
7. Brother-in-law, sister-in-law 姐夫妹夫/嫂子弟媳
8. Child 孩子
9. Spouse of child 孩子的配偶

10. Grandchild 孙子女
11. Other relative 其他亲戚
12. Helper or other non-relative 帮忙的人或者其他非亲属

DB034 What is the main reason for proxy（the respondent is absent)？受访者不在场，完全请人代填的主要原因是什么?

1. The respondent has serious physical handicaps 受访者有严重身体障碍
2. The respondent has serious mental handicaps 受访者有严重精神障碍
3. The respondent has declined this interview. 受访者拒访
4. Other 其他 ______ **(DB034_1)**

DC Cognition and Depression 认知和抑郁

SECTION INTRO Introducing Respondent Interview 介绍受访者问卷

[INTRO: Next I will be asking you to complete a series of memory, thinking, and other tasks. When we are done, I will ask one of your informant to also complete a short questionnaire. If we should come to any question that you don't want to answer, just let me know and I will go on to the next question 下面我将会请您完成一系列与记忆和思考相关的小任务。我还需要了解您情况的知情人一起完成一个简短的问卷。在采访过程中，如果我们问到了一些让您感到为难或不想回答的问题，您可以告诉我，我们会跳过这些题目]

PROCEDURE 程序：

Respondent aged 60 years and older should done all tests in DC 60 岁及以上（Age>=60）的受访者回答 DC 模块所有的问题；

Respondent aged less than 60 years should only done the tests listed below 60 岁以下（Age<60）的受访者仅回答下述问题：

DC001_W4、DC002_W4、DC003_W4、DC005_W4、DC006_W4、DC004、DC014_W4、DC014_W4_1、DC014_W4_2、DC014_W4_3、DC014_W4_4、DC014_W4_5、DC024、DC024_W4 in SECTION MMSE 简易精神状态检查部分的上述题目

All items in SECTION WR 字词回忆部分的所有题目

All items in SECTION CESD 抑郁量表部分的所有题目

All items in SECTION SAT 满意度部分的所有题目

All items in SECTION DR 延迟回忆部分的所有题目

All items in SECTION NS 数列题部分的所有题目

SECTION MMSE Mini Mental State Exam 简易精神状态检查

[INTRO: First I would like to ask you some questions to check your memory and concentration. Some of them may be easy and some may be hard 访员指导语：首先我将会问你一些问题以检查你的记忆力和注意力。其中有些问题简单，有些问题比较难]

DC001_W4 What is the year? 今年是哪一年？

1 = Correct 正确; 5 = Error 错误; 97 = Not assessed 未评估

DC002_W4 What is the season of the year? 现在是什么季节？

1 = Correct 正确; 5 = Error 错误; 97 = Not assessed 未评估

DC003_W4 What is the date? 今天是这个月的几号？

[IWER: Lunar date is correct,not allowed to check the calendar 访员注意：阴历日期也正确，不允许查手机或者日历]

1 = Correct 正确; 5 = Error 错误; 97 = Not assessed 未评估

DC005_W4 What is the day of the week? 今天是星期几?

1 = Correct 正确; 5 = Error 错误; 97 = Not assessed 未评估

DC006_W4 What is the month? 现在是几月份?

[IWER: Lunar month is correct,not allowed to check the calendar 访员注意：阴历月份也正确，不允许查手机或者日历]

1 = Correct 正确; 5 = Error 错误; 97 = Not assessed 未评估

DC007_W4 What state are we in? 我们现在在哪个省?

1 = Correct 正确; 5 = Error 错误; 97 = Not assessed 未评估

DC008_W4 What county are we in? 我们现在在哪个城市（或州、或地区、或县、或岛屿）?

1 = Correct 正确; 5 = Error 错误; 97 = Not assessed 未评估

DC009_W4 What city or town are we in? 我们现在在哪个区（镇/村）?

1 = Correct 正确; 5 = Error 错误; 97 = Not assessed 未评估

DC010_W4 What floor of the building are we on? 我们现在是在第几层楼?

1 = Correct 正确; 5 = Error 错误; 97 = Not assessed 未评估

DC012_W4 What is this address? (If institutionalized, what is the name of the institution?) 这个地方叫什么?

[IWER: Ask the name of the hospital if interviewed in hospital, ask the name of the building or street if interviewed in community 访员注意：如果是在医院，则问医院名称；如果是在居委会，则问建筑物名称或者街道名称]

1 = Correct 正确; 5 = Error 错误; 97 = Not assessed 未评估

DC004 What is the month? 您觉得自己现在的记忆力怎么样？是极好、很好、好、一般还是不好?

1 = Excellent 极好; 2 = Very Good 很好; 3 = Good 好; 4 =Fair 一般; 5 = Poor 不好

[INTRO: I am going to name three objects. After I have said them, I want you to repeat them. Remember what they are because I am going to ask you to name them again in a few minutes. Ball, flag, tree. Please repeat the names for me 访员指导语：现在我要说三样东西的名称，在我讲完之后，请您重复说一遍，请您记住这三样东西，因为等一下要再问您的。皮球、国旗、树木。请您为我重复一遍这三样东西的名称]

DC013_W4_1 Interviewer please record words which are correctly recalled 请访员记录下受访者回答正确的物品。

1. Ball 皮球
2. Flag 国旗
3. Tree 树木
4. None 均不正确

97 Not assessed 未评估

PROCEDURE 程序：
If all 3 are recalled correctly (DC013_W4_1s1 = 1 and DC013_W4_1s2 = 1 and DC013_W4_1s3 = 1), skip to DC014_W4, otherwise, go on to DC013_W4_2 如果三个物品名称均回忆正确（即 DC013_W4_1s1 = 1 并且 DC013_W4_1s2 = 1 并且 DC013_W4_1s3 = 1），跳至 DC014_W4；其他情况下，跳至 DC013_W4_2

[INTRO: I am going to read the three objects again. After I have said them, I want you to repeat them. Ball, Flag, Tree. Please repeat the names for me 访员指导语：现在我将再次重复一遍三样东西的名称。在我讲完之后，请您重复说一遍。皮球、国旗、树木。请您为我重复一遍这三样东西的名称]

DC013_W4_2 Interviewer please record words which are correctly recalled 请访员记录下受访者回答正确的物品。
1. Ball 皮球
2. Flag 国旗
3. Tree 树木
4. None 均不正确
97. Not assessed 未评估

PROCEDURE 程序：
If all 3 are recalled correctly (DC013_W4_2s1 = 1 and DC013_W4_2s2 = 1 and DC013_W4_2s3 = 1), skip to DC014_W4, otherwise, go on to DC013_W4_3 如果三个物品名称均回忆正确（即 DC013_W4_2s1 = 1 并且 DC013_W4_2s2 = 1 并且 DC013_W4_2s3 = 1），跳至 DC014_W4；其他情况下，跳至 DC013_W4_3

[INTRO: I am going to read the three objects again. After I have said them, I want you to repeat them. Ball, Flag, Tree. Please repeat the names for me 访员指导语：现在我将再次重复一遍三样东西的名称。在我讲完之后，请您重复说一遍。皮球、国旗、树木。请您为我重复一遍这三样东西的名称]

DC013_W4_3 Interviewer please record words which are correctly recalled 请访员记录下受访者回答正确的物品。
1. Ball 皮球
2. Flag 国旗
3. Tree 树木
4. None 均不正确
97. Not assessed 未评估

PROCEDURE 程序：
If all 3 are recalled correctly (DC013_W4_3s1 = 1 and DC013_W4_3s2 = 1 and DC013_W4_3s3 = 1), skip to DC014_W4, otherwise, go on to DC013_W4_4 如果三个物品名称均回忆正确（即 DC013_W4_3s1 = 1 并且 DC013_W4_3s2 = 1 并且 DC013_W4_3s3 = 1），跳至 DC014_W4；其他情况下，跳至 DC013_W4_4

[INTRO: I am going to read the three objects again. After I have said them, I want you to repeat them. Ball, Flag, Tree. Please repeat the names for me 访员指导语：现在我将再次重复一遍三样东西的名称。在我讲完之后，请您重复说一遍。皮球、国旗、树木。请您为我重复一遍这三样东西的名称]

DC013_W4_4 Interviewer please record words which are correctly recalled 请访员记录下受访者回答正确的物品。

1. Ball 皮球
2. Flag 国旗
3. Tree 树木
4. None 均不正确
97. Not assessed 未评估

PROCEDURE 程序：

If all 3 are recalled correctly (DC013_W4_4s1 = 1 and DC013_W4_4s2 = 1 and DC013_W4_4s3 = 1), skip to DC014_W4, otherwise, go on to DC013_W4_5 如果三个物品名称均回忆正确（即 DC013_W4_4s1 = 1 并且 DC013_W4_4s2 = 1 并且 DC013_W4_4s3 = 1），跳至 DC014_W4；其他情况下，跳至 DC013_W4_5

[INTRO: I am going to read the three objects again. After I have said them, I want you to repeat them. Ball, Flag, Tree. Please repeat the names for me 访员指导语：现在我将再次重复一遍三样东西的名称。在我讲完之后，请您重复说一遍。皮球、国旗、树木。请您为我重复一遍这三样东西的名称]

DC013_W4_5 Interviewer please record words which are correctly recalled 请访员记录下受访者回答正确的物品。

1. Ball 皮球
2. Flag 国旗
3. Tree 树木
4. None 均不正确
97. Not assessed 未评估

[INTRO: please calculate 100 minus 7, and keep minus 7 continuously, tell me each answer you get from minus 7, until I say stop 访员指导语：请您算一算 100 减去 7，然后从所得的数目再减去 7，如此一直计算下去，请您将每减一个 7 后的答案告诉我，直到我说“停”为止]

[IWER: After read the introduction, do not give any others instructions during the test, and do not remind the subject what should be done. No extra instructions except saying “continue” 访员注意：指导语读完后，受访者在计算过程中，请不要再向受访者发出任何指令，也不能提醒受访者应该怎么做。请记住不能给任何额外提示，只能说“继续”]

DC014_W4 100 minus 7, and keep minus 7 continuously for five times 100 减 7, 连续减 5 次

DC014_W4_1 Record answer from respondent 记录答案 ______ **(DC014_W4_1_1)**

97. DK 不知道 → Skip to DC015_W4 跳至 DC015_W4
98. RF 拒绝回答 → Skip to DC015_W4 跳至 DC015_W4

DC014_W4_2 Record answer from respondent 记录答案：______ **(DC014_W4_2_1)**

97. DK 不知道 → Skip to DC015_W4 跳至 DC015_W4
98. RF 拒绝回答 → Skip to DC015_W4 跳至 DC015_W4

DC014_W4_3 Record answer from respondent 记录答案: ______ (**DC014_W4_3_1**)
97. DK 不知道 → Skip to DC015_W4 跳至 DC015_W4
98. RF 拒绝回答 → Skip to DC015_W4 跳至 DC015_W4

DC014_W4_4 Record answer from respondent 记录答案: ______ (**DC014_W4_4_1**)
97. DK 不知道 → Skip to DC015_W4 跳至 DC015_W4
98. RF 拒绝回答 → Skip to DC015_W4 跳至 DC015_W4

DC014_W4_5 Record answer from respondent 记录答案: ______ (**DC014_W4_5_1**)
97. DK 不知道
98. RF 拒绝回答

DC024 [IWER: Please Indicate whether the respondent used paper and pencil or any other aid during Used paper, pen or other aid when completing the number subtraction 访员注意: 受访者在回答这些算术题时，是否用了纸、笔或其他辅助工具?]
1. Used aid 用了辅助工具
2. Did not use aid 没用辅助工具

PROCEDURE 程序:
If (DC013_W4_1s97 = 1, and DC013_W4_2s97 = 1, and DC013_W4_3s97 = 1, and DC013_W4_4s97 = 1, and DC013_W4_5s97 = 1), or DC013_W4_5s4 = 1, then skip to DC016_W4 如果 (DC013_W4_1s97 = 1, 或 DC013_W4_2s97 = 1, 或 DC013_W4_3s97 = 1, 或 DC013_W4_4s97 = 1, 或 DC013_W4_5s97 = 1), 或 DC013_W4_5s4 = 1, 跳至 DC016_W4

DC015_W4 What were the three objects I asked you to remember? 现在请您告诉我, 刚才我要您记住的三样东西是什么?
1. Ball 皮球
2. Flag 国旗
3. Tree 树木
4. None 均不正确
97. Not assessed 未评估

[PROGRAMMER: Display a picture of a watch on this page 此页面出现手表的图片]

DC016_W4 [Interviwer, point to the watch picture on screen to respondent and ask 访员将平板屏幕面向受访者，指向屏幕显示的图片，并问] What is this called? “请问这是什么?”
1 = Correct 正确; 5 = Error 错误; 97 = Not assessed 未评估

[PROGRAMMER: Display a picture of a pencil on this page 此页面出现铅笔的图片]

DC017_W4 [Interviwer, point to the pencil picture on screen to respondent and ask 访员将平板屏幕面向受访者，指向屏幕显示的图片，并问] What is this called? “请问这是什么?”
1 = Correct 正确; 5 = Error 错误; 97 = Not assessed 未评估

DC018_W4 I would like you to repeat a phrase after me. The phrase is: “No if’s and’s or but’s” 现在请您重复一遍我说的这句话。这句话是“四十四只石狮子”。

[IWER: Allow only one attempt to repeat the phrase 访员注意：只允许受访者重复一次这句话

IWER: You can repeat the phrase up to five times if the respondent is struggling to hear the phrase 访员注意：如果受访者难已听清这句话的话，你可以重复最多五次

IWER: You cannot repeat it if respondent already attempted the phrase 访员注意：如果受访者已经开始尝试重复这句话，请不要再向受访者重复]

1 = Correct 正确；5 = Error 错误；97 = Not assessed 未评估

[PROGRAMMER: The following sentence will be displayed on a single page, occupy the whole screen 下面这句话单独显示在一页，占据屏幕的整个页面]

DC019_W4 Interviwer show the page to respondent, which says “Close your eyes”, ask respondent to “Read the words on this page, then do what it says.” 访员将写有“闭上您的眼睛”大字的屏幕面向受访者，请受访者照着这张卡片所写的去做。

1. Participant closed eyes; correct 受访者闭上了眼睛；正确
2. Correct, Examiner read the phrase aloud 正确，访员大声朗读出卡片上的句子后受访者闭上了眼睛
5. Error/Omission (e.g. participant did not close eyes) 错误/遗漏（例如：受访者没有闭上眼睛）
97. Not assessed 未评估

[INTRO: I am going to give you a piece of paper. When I do, take the paper in your right hand, fold the paper in half with both hands, and put the paper down on your left lap 访员指导语：现在我将会给您一张纸。请您用右手拿起纸，双手将纸对折并把折好的纸放在左腿上]

[IWER: You can read this instruction only once. Do not repeat instructions unless respindent didn’t hear the instruction and do not coach 访员注意：你只能为受访者解释一次这个指令。除非受访者没听到指令也不给出相应动作，否则请勿重复解释指令]

DC020_W4 Hand 手

1. Respondent takes the paper with her/his right hand, correct 受访者用右手拿起纸，正确
5. Respondent does not take the paper with the right hand score, error 受访者没有用右手拿起纸，错误
97. Not assessed 未评估

DC021_W4 Fold 折纸

1. Respondent folds the paper with both hands, correct 受访者用双手折纸，正确
5. Respondent does not fold the paper with both hands, error 受访者没有用双手折纸，错误
97. Not assessed 未评估

DC022_W4 Leg 腿

1. Respondent places the paper in her/his left lab, correct 受访者将折好的纸放在左腿上，正确
5. The participate does not place the paper in her/his left lap, error 受访者没有将折好的纸放在左腿上，错误
97. Not assessed 未评估

DC023_W4 Write any complete sentence on that piece of paper for me 请受访者写一句完整的、有意义的句子。

[IWER: Make sure to take photo of the paper, upload the photo onto system, check and score correctness of sentence 访员注意：请将纸拍照上传，检查并记录句子的正确程度（必须有主语、动词且有意义算正确）]

1 = Correct 正确; 5 = Error 错误; 97 = Not assessed 未评估

[PROGRAMMER: Here add camera function for interviewers to take photo 此处增加拍照]

DC024_W4 Here is a drawing. Please copy the drawing on this paper 这里有一幅图。请您按照图的样子画在这里。

[IWER: Score correct if A) There are two five-sided figures which intersect to form a four-sided figure and B) All angles in the five sided figure much be preserved 访员注意：如果受访者所复制的图形满足 A) 两个四边相交的五边形 B) 五边内的所有角都是完整的，则算作正确]

1 = Correct 正确; 5 = Error 错误; 97 = Not assessed 未评估

[PROGRAMMER: Here add camera function for interviewers to take photo 此处增加拍照]

SECTION HT HRS Telephone Interview for Cognitive Status (TICS) 认知状况电话访问量表

[INTRO: Now I'm going to ask you for the names of some people and things 现在我要问您一些人和事物的名字]

DC025_W4 What do people usually use to cut paper? 人们平时用来剪纸的东西叫什么?

1. Scissors or shears only 剪刀/剪子
5. Not correct 错误
8. DK 不知道
97. RF 拒绝回答

DC026_W4 What do you call the kind of prickly plant that grows in the desert? 哪种长在沙漠里的植物是多刺的?

1. Cactus or name of kind of cactus 仙人掌/仙人球
5. Not correct 错误

8. DK 不知道
97. RF 拒绝回答
99. Other 其他，please specify 具体注明 _____ （**DC026_W4_1**）

DC027_W4 Who is the President of the United States right now? 现在中国的国家主席是谁?
1. Last name (XI) correct 习近平/习大大
5. Not correct 错误
8. DK 不知道
97. RF 拒绝回答

SECTION WR Word Recall 字词回忆

WR101_INTRO I am going to show you ten printed words. Read each word out loud as I show it to you. Later I will ask you to recall all ten words. Is this clear? 现在我将向您展示十个词语。请您将十个词语大声朗读出来。接着我会请您回忆这十个词语。您明白了么?
[IWER: Probe as needed for understanding of task 访员注意：请检查受访者是否明白如何进行这项测试]
1. Yes 是 → skip DC028_W4_1 跳过 DC028_W4_1
2. No 否

**PROCEDURE** 程序：<br>If respondent aged 60 years and above who refused this task, skip to RF101_Intro 如果 60 岁及以上的受访者拒绝完成这项任务，则跳至 RF101_Intro<br>If respondent aged less than 60 years who refused this task, skip to SECTION CESD 如果 60 岁以下的受访者拒绝完成这项任务，则跳至 SECTION CESD 部分

DC028_W4_1 Please record reasons for refusal? 请记录受访者为什么无法完成此项测试?
1. Refused or unwilling to to 拒绝或不愿意进行此项测试
2. Cannot speak in whole life 终生无法说话
3. Cannot speak when getting old 进入老年阶段后开始无法说话
4. Deaf or poor hearing 耳聋或听力不好
5. Other 其他，please specify 请注明 _____ (**DC028_W4_1_1**）

**PROCEDURE** 程序：<br>After DC028_W4_1, skip to RF101_Intro 回答完成 DC028_W4_1 后跳至 RF101_Intro

[IWER: Show wordlist at a slow, steady rate, approximately one word every two seconds 访员注意：以缓慢、稳定的速度向受访者展示词表。频率掌握在约每两秒一个词]

[IWER: If R indicates he/she cannot read: 访员注意：如果受访者表示他/她无法阅读词表:
TELL THE R: I will read the words for you and you repeat them after me 告诉受访者，你将会为其阅读词表，请该受访者在你读完后重复

Read the words out loud as you show the wordlist in a slow, steady rate, approximately one word every seconds 以缓慢、稳定的速度，边展示词表边大声朗读词表内容。频率掌握在约每两秒一个词
R should repeat the word after you read it our loud, before moving to the next word 在换到下一组词语前，你应该大声朗读完，并请受访者重复该词]

[PROGRAMMER:
Word sequence: Butter, Arm, Shore, Letter, Queen, Cabin, Pole, Ticket, Grass, Engine 词组顺序：奶油、胳膊、海滨、信件、王后、木屋、扁担、票、草、发动机
Display one word at a time in landscape manner, the word should occupy the whole screen 每页面仅横向显示一个词，词语需占满整个屏幕]

DC028_W4 Now please tell me the words you can recall. 现在请告诉我您可以想起来的词语。
[IWER: PERMIT as much time as R wishes – up to about 2 minutes 访员注意：受访者需要多长时间就给多长时间，最多可以有两分钟
Please select words that is being correctly recalled by respondent (Select all that apply) 请选择受访者成功回忆起的词语（多选）]

1. Butter 奶油
2. Arm 胳膊
3. Shore 海滨
4. Letter 信件
5. Queen 王后
6. Cabin 木屋
7. Pole 扁担
8. Ticket 票
9. Grass 草
10. Engine 发动机
11. None 没有回忆起任何词语
12. RF 拒绝回忆 → Skip to DC031_W4 跳至 DC031_W4

[INTRO: I am going to show you the same list of words in a different order. Read each word out loud as I show it to you. Later I will ask you to recall all ten words. Are you ready? 现在我将以一个不同的顺序来向您展示和刚才相同的一系列词语。请您将十个词语大声朗读出来。接着我会请您回忆这十个词语。准备好了么？]

[IWER: Show wordlist at a slow, steady rate, approximately one word every two seconds 访员注意：以缓慢、稳定的速度向受访者展示词表 1 – 测试 2。频率掌握在约每两秒一个词]

[IWER: If R indicates he/she cannot read: 访员注意：如果受访者表示他/她无法阅读词表:
TELL THE R: I will read the words for you and you repeat them after me 告诉受访者，你将会为其阅读词表，请该受访者在你读完后重复
Read the words out loud as you show the wordlist in a slow, steady rate, approximately one word every seconds 以缓慢、稳定的速度，边展示词表边大声朗读词表内容。频率掌握在约每两秒一个词

R should repeat the word after you read it our loud, before moving to the next word 在换到下一组词语前，你应该大声朗读完，并请受访者重复该词]

[PROGRAMMER:

Word sequence: Pole, Letter, Butter, Queen, Arm, Shore, Grass, Cabin, Ticket, Engine 词组顺序：扁担、信件、奶油、王后、胳膊、海滨、草、木屋、票、发动机

Display one word at a time in landscape manner, the word should occupy the whole screen 每页面仅横向显示一个词，词语需占满整个屏幕]

DC029_W4 Now please tell me the words you can recall 现在请告诉我您可以想起来的词语。

[IWER: PERMIT as much time as R wishes – up to about 2 minutes 访员注意：受访者需要多长时间就给多长时间，最多可以有两分钟

Please select words that is being correctly recalled by respondent (Select all that apply) 请选择受访者成功回忆起的词语（多选）]

1. Pole 扁担
2. Letter 信件
3. Butter 奶油
4. Queen 王后
5. Arm 胳膊
6. Shore 海滨
7. Grass 草
8. Cabin 木屋
9. Ticket 票
10. Engine 发动机
11. None 没有回忆起任何词语
12. RF 拒绝回忆 → Skip to DC031_W4 跳至 DC031_W4

[INTRO: I am going to show you the same list of words in a different order. Read each word out loud as I show it to you. Later I will ask you to recall all ten words. Are you ready? 现在我将以一个不同的顺序来向您展示和刚才相同的一系列词语。请您将十个词语大声朗读出来。接着我会请您回忆这十个词语。准备好了么？]

[IWER: Show wordlist at a slow, steady rate, approximately one word every two seconds 访员注意：以缓慢、稳定的速度向受访者展示词表 1 – 测试 3。频率掌握在约每两秒一个词]

[IWER: If R indicates he/she cannot read: 访员注意：如果受访者表示他/她无法阅读词表:

TELL THE R: I will read the words for you and you repeat them after me 告诉受访者，你将会为其阅读词表，请该受访者在你读完后重复

Read the words out loud as you show the wordlist in a slow, steady rate, approximately one word every seconds 以缓慢、稳定的速度，边展示词表边大声朗读词表内容。频率掌握在约每两秒一个词

R should repeat the word after you read it our loud, before moving to the next word 在换到下一组词语前，你应该大声朗读完，并请受访者重复该词]

[PROGRAMMER:

Word sequence: Shore, Letter, Arm, Cabin, Pole, Ticket, Engine, Grass, Butter, Queen 词组顺序海滨、信件、胳膊、木屋、扁担、票、发动机、草、奶油、王后
Display one word at a time in landscape manner, the word should occupy the whole screen 每页面仅横向显示一个词，词语需占满整个屏幕]

DC030_W4 Now please tell me the words you can recall. 现在请告诉我您可以想起来的词语。
[IWER: PERMIT as much time as R wishes – up to about 2 minutes 访员注意：受访者需要多长时间就给多长时间，最多可以有两分钟
Please select words that is being correctly recalled by respondent(Select all that apply) 请选择受访者成功回忆起的词语（多选）]

1. Shore 海滨
2. Letter 信件
3. Arm 胳膊
4. Cabin 木屋
5. Pole 扁担
6. Ticket 票
7. Engine 发动机
8. Grass 草
9. Butter 奶油
10. Queen 王后
11. None 没有回忆起任何词语
12. RF 拒绝回忆

DC031_W4 [IWER: Indicate whether any of the following apply to the administation of the word lists (select all that apply) 访员注意：请注明在整个过程中是否有发生下列情况（多选）]

1. Administered verbally 口头完成测评
2. An interrution occured during administration of list 当进行词表时有被迫中断
3. Respondent had difficulty reading the words 受访者读词有困难
4. No issues occured 以上情况均未发生

SECTION RF Retrieval Fluency 口语流畅性：“动物类别”

RF INTRODUCTION ANIMAL NAMING RF 介绍动物类别

[This test can not only assess the degree of impairment of oral expression, but also measure semantic memory and language skills. Tell R “I will tell you a certain category, please say names belonging to this category as many as you can, the sooner the better. For example: when I say ‘types of clothing’, you can say shirts, ties or hats and so on. Can you think of other names in the category of clothing?” 此项测试既可测评口语表达能力受损的程度，也可测评语义记忆和语言能力。对受访者说：“我将告诉您某种类别，请说出所有您知道属于这一类别的名称，越快越好。比如：我说‘衣物的种类’，您可以说衬衫、领带或帽子等。您还能想出衣物种类中的其它名称吗?”

Wait for the respondent to say two names. If the respondent succed, then announce the answer is correct and start the test. If the respondent says the wrong name or responds incorrectly, please correct him/her and repeat the instruction. If the respondent cannot answer, repeat the instruction. If the interviewee apparently cannot understand the guideline, stop the task and ask why 等待受访者说出两个名称。如果受访者能成功地做到，就说答案正确，并开始测试。如果受访者说了一个错误的名称或者回答不恰当，就纠正受访者的回答并重复指导语。如果受访者仍然无法回答，就再重复一次指导语。如果受访者显然还是不能理解指导语，就停止此任务，并询问为什么会这样

After making sure that the respondent understand the test and say two names correctly, please say “Now I want to see how many different animals you can name. You will have 60 seconds. When I say, “Begin”, say the animal names as fast as you can.” 在你知道受访者已明白此任务应如何进行并且也说出了两个正确的衣物类名称后，请接着说：“现在我们来看看您能说出多少种动物类别。您有 60 秒的时间。当我说开始的时候，请您快速说出您能想到的所有动物类别，越快越好。”]

[IWER: Get ready to time 60 seconds. Repeat instructions if necessary. If the respondent stopped before the time was up, they can be encouraged to try to figure out more animal names. If the respondent stop for more than 15 secons, repeat the instrucion “I want you to name as many animal names as you can”. Even though the instruction is repeated during the test, the time should mot be prolonged 访员注意：准备好计时 60 秒。如果需要，可以向受访者重复指令。如果受访者在时间未到时停了下来，就鼓励受访者尽量想出更多的动物名称。如果受访者沉默了 15 秒，就重复基本的指导语（“我要您说出所有您能想到的动物名称。”）即使在测试过程中重复了指导语，但测试时间仍不可延长]

[IWER: The scores are number of animals the respondet name every 15 seconds. As long as they do not belong to minerals and plants can be regarded as animals. Only real animals (including the dragon) are considered the correct answers, non-real animals (such as phoenix, unicorn, etc.) cannot be counted. Please exclude the names of duplicates, the nick-names of owners for animals, the names of animals of the same species of different colors, sex or cubs. Specificly, count categories of animals (E.G., dog), as well as specific types (E.G., collie, terrier) as correct 访员注意：受访者的分数是每 15 秒钟其所说的正确动物名称数的总和。只要不属于矿物和植物都可算是动物。只有真实存在的动物（包括龙）可视为正确答案，非真实存在的动物（如凤凰、麒麟等）不计入正确答案。但不包括重复的名称、主人为动物起的名字、种类相同但颜色不同的动物以及某种动物的公、母或幼兽的名称。具体而言，以下名称均可得分：回答种类的名称（如：狗）或任何该种类范围内的伴生物种名称（如：小猎犬、达克斯猎犬）]

[IWER: Please record the interview process, to facilitate the recording of all animal names after the test.After the test is finshed, please make a positive and negative judgement on the names of the animals listed, and enter the correct number on to tablet 访员注意：访问过程请录音，方便此项测试结束后记下所有的动物名称。测试结束后，请你对所列出的动物名称进行正误的评判，然后将正确的回答个数输入平板内]

[IWER: Start timing: press [START TIMING] as soon as you tell the R to begin 访员注意：请在告诉受访者开始后马上按下 [开始计时]]

RF101_Intro Are you ready? (PAUSE.) Begin 您准备好了么?（暂停）开始!

1. 是 → skip DC039_W4_1 跳过 DC039_W4_1
2. 否

DC039_W4_1 Please record reasons for not complete this test 请记录受访者为什么无法完成此项测试?

1. Refuse or unwilling to do 拒绝或不愿意进行此项测试
2. Cannot speak in whole life 终生无法说话
3. Cannot speak after getting old 进入老年阶段后开始无法说话
4. Deaf or poor hearing 耳聋或听力不好
5. Other 其他，please speify 请注明 _____ **(DC039_W4_1_1)**

**PROCEDURE** 程序：<br>After DC039_W4_1 skip to SECTION CSI-D 回答完成 DC039_W4_1 后跳至 SECTION CSI-D

[PROGRAMMER: DON'T KNOW/DK and REFUSE/RF are not recorded for retrieval fluency test 请勿将“不知道”或“拒答”记录在口语流畅性测试内]

[IWER: Every time when respondent speak out a name, interviewer should press ENTER. If the name is correct, interviewer make a mark on paper. After the 60 second end, record all answers on system 访员注意：受访者受访者每说出一个动物名称，访员按一下“记录”键，在 60 秒测试结束后，将所有的答案计入纸质问卷，并拍照上传至系统]

DC032_W4 System record the total number of animal names during 0-15 seconds 记录 0-15 秒说出的动物总数：_____

DC033_W4 The amount of correct names during 0-15 seconds0-15 秒正确的个数：_____

DC034_W4 System record the total number of animal names during 16-30 seconds 记录 16-30 秒说出的动物总数：_____

DC035_W4 The amount of correct names during 16-30 seconds16-30 秒正确的个数：_____

DC036_W4 System record the total number of animal names during 31-45 seconds 记录 31-45 秒说出的动物总数：_____

DC037_W4 The amount of correct names during 31-45 seconds31-45 秒正确的个数：_____

DC038_W4 System record the total number of animal names during 46-60 seconds 记录 46-60 秒说出的动物总数：_____

DC039_W4 The amount of correct names during 46-60 seconds46-60 秒正确的个数：_____

SECTION CSI-D Community Screening Instrument for Dementia Interviewee Part 简明社区痴呆筛查量表-认知功能部分

DC042_W4 [IWER: Point to your elbow and say 访员指向自己的肘部问：] What do we call this? 我们把这个叫做什么？

1. Correct 正确
5. Error 错误
97. Cannot answer 不能回答

DC043_W4 What do you do with a hammer? 锤子一般用来做什么？

[IWER: Acceptable responses are: “pound”, “to drive a nail into something”, “to pound something” , “to hit something with” 访员注意：答案只要是钉钉子或者类似意思就算对]

1. Correct 正确
5. Error 错误
97. Cannot answer 不能回答

DC044_W4 Where is the local market/ local store? 您这附近最近的市场/商店在哪里？

[IWER: “Can you be more specific” if the Respondent uses vague answer 访员注意：如果受访者的答案不明确，访员可以适当提醒：“请问您可以说得更具体些么？”]

1. Clearly indicate the route local market/ local store 明确指出如何走到市场/商场的路线
2. Address of local market/ local store 市场/商场的地址
3. Name of local market/ local store 市场/商店名字
4. A vague answer 一个很不清楚的回答
5. DK 不知道
97. Cannot answer 不能回答

DC045_W4 Please point first to the window and then to the door 请先指一下窗户再指一下门。

1. R point to the window and then the door 受访者指了一下窗户后指一下门
2. R just point to window – there is no door nearby 受访者仅仅指了窗户– 附近没有门
3. R just point to door – there is no window nearby 受访者仅仅指了门– 附近没有窗户
4. Not applied 不适用
5. Incorrect action 错误动作
97. Cannot answer 不能回答

SECTION CESD Depression 抑郁量表

[INTRO: The 10 items below refer to how you have felt and behaved during the last week. Every item has the same selective answers including rarely or none of the time, some, occasionally, and most or all of the time. Choose the appropriate response 访员指导语：下面 10 道问题是有关您上周的感觉及行为，每道题目的答案都是一样的，包括很少或者根本没有，不太多，有时或者说有一半的时间还是大多数的时间，请您选择合适的答案]

DC009 I was bothered by things that don’t usually bother me 我因一些小事而烦恼。

1. Rarely or none of the time 很少或者根本没有（<1 day 天）
2. Some or a little of the time 不太多（1-2 days 天）
3. Occasionally or a moderate amount of the time 有时或者说有一半的时间（3-4 days 天）
4. Most or all of the time 大多数的时间（5-7 days 天）
8. DK 不知道
9. RF 拒绝回答

DC010 I had trouble keeping my mind on what I was doing 我在做事时很难集中精力。

1. Rarely or none of the time 很少或者根本没有（<1 day 天）
2. Some or a little of the time 不太多（1-2 days 天）
3. Occasionally or a moderate amount of the time 有时或者说有一半的时间（3-4 days 天）
4. Most or all of the time 大多数的时间（5-7 days 天）
8. DK 不知道
9. RF 拒绝回答

DC011 I felt depressed 我感到情绪低落。

1. Rarely or none of the time 很少或者根本没有（<1 day 天）
2. Some or a little of the time 不太多（1-2 days 天）
3. Occasionally or a moderate amount of the time 有时或者说有一半的时间（3-4 days 天）
4. Most or all of the time 大多数的时间（5-7 days 天）
8. DK 不知道
9. RF 拒绝回答

DC012 I felt everything I did was an effort 我觉得做任何事都很费劲。

1. Rarely or none of the time 很少或者根本没有（<1 day 天）
2. Some or a little of the time 不太多（1-2 days 天）
3. Occasionally or a moderate amount of the time 有时或者说有一半的时间（3-4 days 天）
4. Most or all of the time 大多数的时间（5-7 days 天）
8. DK 不知道
9. RF 拒绝回答

DC013 I felt hopeful about the future 我对未来充满希望。

1. Rarely or none of the time 很少或者根本没有（<1 day 天）
2. Some or a little of the time 不太多（1-2 days 天）
3. Occasionally or a moderate amount of the time 有时或者说有一半的时间（3-4 days 天）
4. Most or all of the time 大多数的时间（5-7 days 天）

8. DK 不知道
9. RF 拒绝回答

DC014 I felt fearful 我感到害怕。
1. Rarely or none of the time 很少或者根本没有（<1 day 天）
2. Some or a little of the time 不太多（1-2 days 天）
3. Occasionally or a moderate amount of the time 有时或者说有一半的时间（3-4 days 天）
4. Most or all of the time 大多数的时间（5-7 days 天）
8. DK 不知道
9. RF 拒绝回答

DC015 My sleep was restless 我的睡眠不好。
1. Rarely or none of the time 很少或者根本没有（<1 day 天）
2. Some or a little of the time 不太多（1-2 days 天）
3. Occasionally or a moderate amount of the time 有时或者说有一半的时间（3-4 days 天）
4. Most or all of the time 大多数的时间（5-7 days 天）
8. DK 不知道
9. RF 拒绝回答

DC016 I was happy 我很愉快。
1. Rarely or none of the time 很少或者根本没有（<1 day 天）
2. Some or a little of the time 不太多（1-2 days 天）
3. Occasionally or a moderate amount of the time 有时或者说有一半的时间（3-4 days 天）
4. Most or all of the time 大多数的时间（5-7 days 天）
8. DK 不知道
9. RF 拒绝回答

DC017 I felt lonely 我感到孤独。
1. Rarely or none of the time 很少或者根本没有（<1 day 天）
2. Some or a little of the time 不太多（1-2 days 天）
3. Occasionally or a moderate amount of the time 有时或者说有一半的时间（3-4 days 天）
4. Most or all of the time 大多数的时间（5-7 days 天）
8. DK 不知道
9. RF 拒绝回答

DC018 I could not get ”going” 我觉得我无法继续我的生活。
1. Rarely or none of the time 很少或者根本没有（<1 day 天）
2. Some or a little of the time 不太多（1-2 days 天）

3. Occasionally or a moderate amount of the time 有时或者说有一半的时间（3-4 days 天）
4. Most or all of the time 大多数的时间（5-7 days 天）
8. DK 不知道
9. RF 拒绝回答

SECTION SAT Satisfaction 满意度部分

DC028 Please think about your life-as-a-whole. How satisfied are you with it? Are you completely satisfied, very satisfied, somewhat satisfied, not very satisfied, or not at all satisfied? 总体来看，您对自己的生活是否感到满意？是极其满意，非常满意，比较满意，不太满意还是一点也不满意？

1. Completely satisfied 极其满意
2. Very satisfied 非常满意
3. Somewhat satisfied 比较满意
4. Not very satisfied 不太满意
5. Not at all satisfied 一点也不满意

DC042_W3 How satisfied are you with your health? 您对您的健康满意吗？是极其满意，非常满意，比较满意，不太满意还是一点也不满意？

1. Completely satisfied 极其满意
2. Very satisfied 非常满意
3. Somewhat satisfied 比较满意
4. Not very satisfied 不太满意
5. Not at all satisfied 一点也不满意

PROCEDURE 程序：
If BE001 = 1, 2, ask DC043_W3

DC043_W3 How satisfied are you with your marriage (relationship with spouse)? 您对您的婚姻满意吗？也就是说您对您和您配偶的关系满意吗？是极其满意，非常满意，比较满意，不太满意还是一点也不满意？

1. Completely satisfied 极其满意
2. Very satisfied 非常满意
3. Somewhat satisfied 比较满意
4. Not very satisfied 不太满意
5. Not at all satisfied 一点也不满意
6. No spouse now 现在没有配偶

DC044_W3 How satisfied are you with your relationship with children? 您对您和您子女的关系满意吗？是极其满意，非常满意，比较满意，不太满意还是一点也不满意？

[IWE: Only for respndents who have living offspring 访员注意：仅对当前有存活子女的受访者询问此问题]

1. Completely satisfied 极其满意
2. Very satisfied 非常满意
3. Somewhat satisfied 比较满意
4. Not very satisfied 不太满意
5. Not at all satisfied 一点也不满意
6. No child now 现在没有子女

DC046_W4 How satisfied are you with the air quality this year? Are you completely satisfied, very satisfied, somewhat satisfied, not very satisfied, or not at all satisfied? 您对今年的空气质量是否感到满意？是极其满意，非常满意，比较满意，不太满意还是一点也不满意？

1. Completely satisfied 极其满意
2. Very satisfied 非常满意
3. Somewhat satisfied 比较满意
4. Not very satisfied 不太满意
5. Not at all satisfied 一点也不满意

SECTION DR Delayed Recall 延迟回忆

PROCEDURE 程序:

If respondent refuse to do WR101_INTRO, or DC028_W4s12 = 1, skip to SECTION NS 如果受访者拒绝 WR101_INTROL，或者 DC028_W4s12 = 1, 跳至下一模块 SECTION NS

[INTRO: A few minutes ago I asked you to learn a list of ten words which you read one at a time from cards. Now I want you to try to recall as many of those 10 words as you can. OK, now tell me as many of those ten words as you can remember 访员指导语：几分钟前我请您读了十张卡片上的词语。现在我想请您尽量尝试去回忆这十个词。好，现在请您告诉我您还记得这十个词里的哪几个词？越多越好]

[IWER: PERMIT as much time as R wishes – up to about 2 minutes 访员注意：受访者需要多长时间就给多长时间，最多可以有两分钟]

DC047_W4 Please select words that is being correctly recalled by respondent 请选择受访者成功回忆起的词语。

1. Shore 海滨
2. Letter 信件
3. Arm 胳膊
4. Cabin 木屋
5. Pole 扁担
6. Ticket 票
7. Engine 发动机
8. Grass 草
9. Butter 奶油
10. Queen 王后

11. None word being recalled 没有回忆起任何词语
12. Refuse to recall 拒绝回忆

SECTION NS Number Series 数列题

[IWER: The following procedure requires the interviewer and the respondent to look at screen together, and following instructions. Respondent is allowed to use pencil and paper to calculate, but other aids such as a calculator will not be allowed to use 访员注意：以下测试过程请受访者共同看电脑屏幕，并直接按提示进行操作。受访者可以使用铅笔和纸作为辅助工具，但不得使用计算器等其他辅助工具]

Number Series First Example 数字序列例一

INTRO: Next I'm going to show you several numbers on the screen. There will be a blank number in the series with a "?" mark. Please look at the numbers from left to right and try to find their pattern. Based on this pattern, tell me what number goes in the blank. Sometimes the blank will be at the end of the series, and sometimes the blank will be in the beginning or in the middle. Now can we start with an example to illustrate 访员指导语：接下来，电脑屏幕上会显示的一行数字，其中有一位是空缺的，这个空缺的数字使用"?"标记。请从左往右仔细观察这组数字，试着找出这组数字的规律，并按照这个规律填出空缺处应有的数字。空缺有时候出现在这组数字的开头，有时候出现在中间，有时候出现在结尾。下面让我们用一个具体的例题来解释这个数字游戏

Please look at this group of numbers, what number should go into the blank? 现在您看到以下一组数字，这个空缺的数字应该填几呢?

3	4	5	?

DC029_W4_1 Record answer from respondent: 记录答案：3，4，5，_____ **(DC029_W3_1)**

6. R doesn't understand instructions 受访者没有理解指令
7. R can't write / no paper / pen available 受访者无法书写 → Skip to END OF SECTION NS 跳至 本模块结尾处
8. DK 不知道
9. RF 拒绝回答

[IWER 访员注意:

If R does not give the Correct response (6) then say: the answer we were looking for is 6. Then ask R "do you understand the direction for this test?", in order to check that the R understand the task 如果答案是不正确的，或者没有回答，这时访员说："这里应该填 6"。这时，问受访者："您理解这个测试的意思吗?"，来判断受访者是否理解了这个测试的规律

If the respondent says he or she does not konw the answer, record DK.Do no record a "DK"response as "R doesn't understand instructions" 如果受访者表示其不知道答案，记录"不知道"。如果受访者表示没有理解指令，请勿记录"不知道"]

Number Series Second Example 数字序列例二

Next, show R the second example on the card and say: Let's try another one. What number goes in the blank based on the pattern of numbers? 接下来，向受访者出示卡上的第二个例子，并且问："让我们来试试另一个：这个图上空缺的地方应该填几呢?"

7	6	?	4

DC030_W4_1 Record answer from respondent: 记录答案：7，6，_____，4 (**DC030_W3_1**)

6. R doesn't understand instructions 受访者没有理解指令
8. DK 不知道
9. RF 拒绝回答

[IWER 访员注意:

If R does not give the Correct response (5) then say: the answer we were looking for is 5 如果答案是不正确的，或者没有回答，这时访员说："这里应该填 5"

If the respondent sys he or she does not konw the answer, record DK.Do no record a "DK"response as "R doesn't understand instructions" 如果受访者表示其不知道答案，记录"不知道"。如果受访者表示没有理解指令，请勿记录"不知道"

]

DC031_W4_0 [IWER: Now, ask respondent "do you understand the directions for this task?" To see if respondent actually konw the rule in this task 访员注意：这时，问受访者："您理解这个测试的意思吗？"，来判断受访者是否理解了这个测试的规律]

1. Continue 继续
5. R seems confused or does not understand task 受访者似乎很困惑，或者不能理解指令
8. DK 不知道 → Skip to END OF SECTION NS 跳至 本模块结尾处
9. RF 拒绝回答 → Skip to END OF SECTION NS 跳至 本模块结尾处

[INTRO: I am now going to show you six more questions like the one you just did. Sometimes the blank will be at the end of the series, and sometimes it may be at the beginning or in the middle. You may be asked a question with more than one blank in the sequence. The numbers might increase, like 1, 2, 3, or decrease, like 3, 2, 1. Some of the problems may be easy but others may be hard. Just do the best you can 访员指导语：从现在开始，您将看到电脑屏幕上会出现刚才类似的问题。空白数字有时会出现在数字序列的结尾，有时会出现在数字序列的中间。而有些问题中可能会出现多个空白数。数字序列可能是递增的，比如 1、2、3；也有可能是递减的，比如 3、2、1。这些问题的难度有所差异。有些可能比较简单，而有些可能比较难。请尽您所能进行回答
It is more important to answer the item correctly than to answer quickly, so take a little time to think before answering. It is okay if you do not know the answer because some of the items are intended to be very difficult. You can go on to the next item at any time. Are you ready to begin? 正确地回答这些问题比迅速地给出答案更加重要，所以在回答之前请仔细考虑。由于本项测试中有些题目非常难，所以对某些题目如果您不知道答案的话没有关系。您可以在任何时候选择放弃某道题而进入下一题。您准备好了就可以继续]

[IWER: Permit as much time as R wishes for each question. If the respondent says he or she does not know the answer, record DK. Do not record A "Don't Know" response as "R doesn't

understand instructions.” If the R has not given an answer after about a minute, ask: would you just like to go on to the next quesion? 访员注意：如果受访者表示其不知道答案，记录“不知道”。如果受访者表示没有理解指令，请勿记录“不知道”。如果在题目出现一分钟后受访者仍未给出答案，访员可以询问：“您想跳到下一个题目么?”]

[IWER: Do not give any hit and do not tell R whether his/her answer is correct or not 访员注意：请不要给出其他提示，不要告诉受访者正确的答案或者告诉受访者其答案是否正确]

PROCEDURE 程序：

All respondents answer DC031_W3_1, DC031_W3_2, DC031_W3_3 所有的受访者回答以下题目 DC031_W3_1，DC031_W3_2，DC031_W3_3

DC031_W3_1 Number Series 数列题

7	8	?	10

[IWER: Correct response 正确答案是：9]

DC031_W4_1 Record answer from respondent: 记录答案：7，8，_____ (**DC031_W3_1**)，10

[IWER: If the respondent says not know the answer, record DK., do not record a “don’t know” response as “unable to do” 访员注意：如果受访者表示不知道答案，记录“不知道”。如果受访者表示“不知道该怎么做”，请勿记录“不知道”]

7. R unable to do 受访者不知道该怎么做 → Skip to END OF SECTION NS 跳至本模块结尾处
8. DK 不知道
9. RF 拒绝回答

DC031_W3_2 Number Series 数列题

8	?	12	14

[IWER: Correct response 正确答案是：10]

DC031_W4_2 Record answer from respondent: 记录答案：8，_____ (**DC031_W3_2**)，12，14

[IWER: If the respondent says not know the answer, record DK 访员注意：如果受访者表示不知道答案，记录“不知道”]

8. DK 不知道
9. RF 拒绝回答

DC031_W3_3 Number Series 数列题

18	10	6	?	3

[IWER: Correct response 正确答案是：4]

DC031_W4_3 Record answer from respondent: 记录答案：18，10，6，_____ (**DC031_W3_3**)，3

[IWER: If the respondent says not know the answer, record DK 访员注意：如果受访者表示不知道答案，记录“不知道”]

8. DK 不知道
9. RF 拒绝回答

PROCEDURE 程序：

If respondent answered none of the STARTING BLOCK correctly, continue on to DC032_W3_1, DC032_W3_2, DC032_W3_3 如果受访者在 STARTING BLOCK 中回答正确的题目数为 0，那么回答以下题目 DC032_W3_1，DC032_W3_2，DC032_W3_3

DC032_W3_1 Number Series 数列题

1	2	3	?

[IWER: Correct response 正确答案是：4]

DC032_W4_1 Record answer from respondent: 记录答案：1，2，3，______ (**DC032_W3_1**)

[IWER: If the respondent says not know the answer, record DK 访员注意：如果受访者表示不知道答案，记录“不知道”]

8. DK 不知道
9. RF 拒绝回答

DC032_W3_2 Number Series 数列题

6	5	4	?

[IWER: Correct response 正确答案是：3]

DC032_W4_2 Record answer from respondent: 记录答案：6，5，4，______ (**DC032_W3_2**)

[IWER: If the respondent says not know the answer, record DK 访员注意：如果受访者表示不知道答案，记录“不知道”]

8. DK 不知道
9. RF 拒绝回答

DC032_W3_3 Number Series 数列题

12	?	16	18

[IWER: Correct response 正确答案是：14]

DC032_W4_3 Record answer from respondent: 记录答案：12，______ (**DC032_W3_3**)，16，18

[IWER: If the respondent says not know the answer, record DK 访员注意：如果受访者表示不知道答案，记录“不知道”]

8. DK 不知道
9. RF 拒绝回答

PROCEDURE 程序：

After finishing DC032_W3_1, DC032_W3_2, DC032_W3_3, Skip to END OF SECTION NS 回答完 DC032_W3_1，DC032_W3_2，DC032_W3_3 后，跳至 本模块结尾处

If respondent got one answer correctly in the STARTING BLOCK, continue on to DC033_W3_1, DC033_W3_2, DC033_W3_3 如果受访者在 STARTING BLOCK 中回答正确的题目数为 1，那么回答以下题目 DC033_W3_1，DC033_W3_2，DC033_W3_3

DC033_W3_1 Number Series 数列题

5	?	3	2

[IWER: Correct response 正确答案是：4]

DC033_W4_1 Record answer from respondent: 记录答案：5，_____ (**DC033_W3_1**)，3，2

[IWER: If the respondent says not know the answer, record DK 访员注意：如果受访者表示不知道答案，记录“不知道”]

8. DK 不知道
9. RF 拒绝回答

DC033_W3_2 Number Series 数列题

4	7	10	?

[IWER: Correct response 正确答案是：13]

DC033_W4_2 Record answer from respondent: 记录答案：4，7，10，_____ (**DC033_W3_2**)

[IWER: If the respondent says not know the answer, record DK 访员注意：如果受访者表示不知道答案，记录“不知道”]

8. DK 不知道
9. RF 拒绝回答

DC033_W3_3 Number Series 数列题

?	4	6	8

[IWER: Correct response 正确答案是：2]

DC033_W4_3 Record answer from respondent: 记录答案：_____ (**DC033_W3_3**)，4，6，8

[IWER: If the respondent says not know the answer, record DK 访员注意：如果受访者表示不知道答案，记录“不知道”]

8. DK 不知道
9. RF 拒绝回答

PROCEDURE 程序：

After finshing DC033_W3_1, DC033_W3_2, DC033_W3_3, Skip to END OF SECTION NS 回答完 DC033_W3_1，DC033_W3_2，DC033_W3_3 后，跳至 本模块结尾处

If respondent got two answers correctly in the STARTING BLOCK, continue on to DC034_W3_1, DC034_W3_2, DC034_W3_3 如果受访者在 STARTING BLOCK 中回答正确的题目数为 2，那么回答以下题目 DC034_W3_1，DC034_W3_2，DC034_W3_3

DC034_W3_1 Number Series 数列题

1	3	3	5	7	7	?

[IWER: Correct response 正确答案是：9]

DC034_W4_1 Record answer from respondent: 记录答案：1, 3, 3, 5, 7, 7, ______ (**DC034_W3_1**)

[IWER: If the respondent says not know the answer, record DK 访员注意：如果受访者表示不知道答案，记录“不知道”]

8. DK 不知道
9. RF 拒绝回答

DC034_W3_2 Number Series 数列题

3	?	8	12	17

[IWER: Correct response 正确答案是：5]

DC034_W4_2 Record answer from respondent: 记录答案：3, ______ (**DC034_W3_2**), 8, 12, 17

[IWER: If the respondent says not know the answer, record DK 访员注意：如果受访者表示不知道答案，记录“不知道”]

8. DK 不知道
9. RF 拒绝回答

DC034_W3_3 Number Series 数列题

17	?	12	8

[IWER: Correct response 正确答案是：15]

DC034_W4_3 Record answer from respondent: 记录答案：17, ______ (**DC034_W3_3**), 12, 8

[IWER: If the respondent says not know the answer, record DK 访员注意：如果受访者表示不知道答案，记录“不知道”]

8. DK 不知道
9. RF 拒绝回答

PROCEDURE 程序：

After finishing DC034_W3_1, DC034_W3_2, DC034_W3_3, Skip to END OF SECTION NS回答完DC034_W3_1，DC034_W3_2，DC034_W3_3 后，跳至 本模块结尾处

If respondent got all three answers correctly in the STARTING BLOCK, continue on to DC035_W3_1, DC035_W3_2, DC035_W3_3 如果受访者在 STARTING BLOCK 中回答正确的题目数为 3，那么回答以下题目 DC035_W3_1，DC035_W3_2，DC035_W3_3

DC035_W3_1 Number Series 数列题

10	?	3	1

[IWER: Correct response 正确答案是：6]

DC035_W4_1 Record answer from respondent: 记录答案：10， _____ (**DC035_W3_1**)， 3， 1
[IWER:If the respondent says not know the answer, record DK 访员注意：如果受访者表示不知道答案，记录“不知道”]
8. DK 不知道
9. RF 拒绝回答

DC035_W3_2 Number Series 数列题

18	17	15	?	8

[IWER: Correct response 正确答案是：12]

DC035_W4_2 Record answer from respondent: 记录答案：18， 17， 15， _____ (**DC035_W3_2**)， 8
[IWER:If the respondent says not know the answer, record DK 访员注意：如果受访者表示不知道答案，记录“不知道”]
8. DK 不知道
9. RF 拒绝回答

DC035_W3_3 Number Series 数列题

3	3	4	6	6	7	?	?

[IWER: Correct response 正确答案是：9， 9]

DC035_W4_3 Record answer from respondent: 记录答案：3, 3, 4, 6, 6, 7, _____ (**DC035_W3_3_1**), _____ (**DC035_W3_3_2**)
[IWER:If the respondent says not know the answer, record DK 访员注意：如果受访者表示不知道答案，记录“不知道”]
8. DK 不知道
9. RF 拒绝回答

[END OF SECTION NS 本模块结束]

SECTION WRE Wordlist Recognition 词组辨识

PROCEDURE 程序：
If respondent refuse to do WR101_INTRO, or DC028_W4s12 = 1, skip to DD 如果受访者拒绝 WR101_INTROL， 或者 DC028_W4s12 = 1。跳至下一模块 DD

WRE_Intro [INTRO: Now I am going to show you a set of words printed on cards. Some of the words are from the list you saw earlier and some are words I haven’t shown you before. I want you to tell me which words are from the list you saw earlier. Do you have any questions? Are you ready? 现在我会给您展示一些词卡。有些词是您刚刚见过的，有些词我没有给您看过。我想请您告诉我，哪些词是您刚才见过的。您有什么问题么？准备好了么？]

1. Yes 是
2. No 否 → Skip to DC –Interview Observation 跳至 DC 部分访员观察

[PROGRAMMER: Display one word at a time in landscape manner, the word should occupy the whole screen 每页面仅横向显示一个词，词语需占满整个屏幕]

[IWER: Show next word. If R is unable to read, say the word "Church" out loud while still showing the card 访员注意：展示下一张词卡，如果受访者无法阅读，请在展示词卡【教堂】的同时大声朗读出词卡上的词语]

DC048_W4 Is this one of the words you saw earlier? 这个词您刚才见过么?
1 = Yes 是; 5 = No 否; 8 = DK 不知道

[IWER: Show next word. If R is unable to read, say the word "Coffee" out loud while still showing the card 访员注意：展示下一张词卡，如果受访者无法阅读，请在展示词卡【咖啡】的同时大声朗读出词卡上的词语]

DC049_W4 Is this one of the words you saw earlier? 这个词您刚才见过么?
1 = Yes 是; 5 = No 否; 8 = DK 不知道

[IWER: Show next word. If R is unable to read, say the word "Butter" out loud while still showing the card 访员注意：展示下一张词卡，如果受访者无法阅读，请在展示词卡【奶油】的同时大声朗读出词卡上的词语]

DC050_W4 Is this one of the words you saw earlier? 这个词您刚才见过么?
1 = Yes 是; 5 = No 否; 8 = DK 不知道

[IWER: Show next word. If R is unable to read, say the word "Dollar" out loud while still showing the card 访员注意：展示下一张词卡，如果受访者无法阅读，请在展示词卡【金钱】的同时大声朗读出词卡上的词语]

DC051_W4 Is this one of the words you saw earlier? 这个词您刚才见过么?
1 = Yes 是; 5 = No 否; 8 = DK 不知道

[IWER: Show next word. If R is unable to read, say the word "Arm" out loud while still showing the card 访员注意：展示下一张词卡，如果受访者无法阅读，请在展示词卡【胳膊】的同时大声朗读出词卡上的词语]

DC052_W4 Is this one of the words you saw earlier? 这个词您刚才见过么?
1 = Yes 是; 5 = No 否; 8 = DK 不知道

[IWER: Show next word. If R is unable to read, say the word "Shore" out loud while still showing the card 访员注意：展示下一张词卡，如果受访者无法阅读，请在展示词卡【海滨】的同时大声朗读出词卡上的词语]

DC053_W4 Is this one of the words you saw earlier? 这个词您刚才见过么?
1 = Yes 是; 5 = No 否; 8 = DK 不知道

[IWER: Show next word. If R is unable to read, say the word “Five” out loud while still showing the card 访员注意：展示下一张词卡，如果受访者无法阅读，请在展示词卡【五】的同时大声朗读出词卡上的词语]

DC054_W4 Is this one of the words you saw earlier? 这个词您刚才见过么?
1 = Yes 是; 5 = No 否; 8 = DK 不知道

[IWER: Show next word. If R is unable to read, say the word “Letter” out loud while still showing the card 访员注意：展示下一张词卡，如果受访者无法阅读，请在展示词卡【信件】的同时大声朗读出词卡上的词语]

DC055_W4 Is this one of the words you saw earlier? 这个词您刚才见过么?
1 = Yes 是; 5 = No 否; 8 = DK 不知道

[IWER: Show next word. If R is unable to read, say the word “Hotel” out loud while still showing the card 访员注意：展示下一张词卡，如果受访者无法阅读，请在展示词卡【宾馆】的同时大声朗读出词卡上的词语]

DC056_W4 Is this one of the words you saw earlier? 这个词您刚才见过么?
1 = Yes 是; 5 = No 否; 8 = DK 不知道

[IWER: Show next word. If R is unable to read, say the word “Mountain” out loud while still showing the card 访员注意：展示下一张词卡，如果受访者无法阅读，请在展示词卡【大山】的同时大声朗读出词卡上的词语]

DC057_W4 Is this one of the words you saw earlier? 这个词您刚才见过么?
1 = Yes 是; 5 = No 否; 8 = DK 不知道

[IWER: Show next word. If R is unable to read, say the word “Queen” out loud while still showing the card 访员注意：展示下一张词卡，如果受访者无法阅读，请在展示词卡【王后】的同时大声朗读出词卡上的词语]

DC058_W4 Is this one of the words you saw earlier? 这个词您刚才见过么?
1 = Yes 是; 5 = No 否; 8 = DK 不知道

[IWER: Show next word. If R is unable to read, say the word “Cabin” out loud while still showing the card 访员注意：展示下一张词卡，如果受访者无法阅读，请在展示词卡【木屋】的同时大声朗读出词卡上的词语]

DC059_W4 Is this one of the words you saw earlier? 这个词您刚才见过么?
1 = Yes 是; 5 = No 否; 8 = DK 不知道

[IWER: Show next word. If R is unable to read, say the word “Slipper” out loud while still showing the card 访员注意：展示下一张词卡，如果受访者无法阅读，请在展示词卡【拖鞋】的同时大声朗读出词卡上的词语]

DC060_W4 Is this one of the words you saw earlier? 这个词您刚才见过么?
1 = Yes 是; 5 = No 否; 8 = DK 不知道

[IWER: Show next word. If R is unable to read, say the word “Pole” out loud while still showing the card 访员注意：展示下一张词卡，如果受访者无法阅读，请在展示词卡【扁担】的同时大声朗读出词卡上的词语]

DC061_W4 Is this one of the words you saw earlier? 这个词您刚才见过么?
1 = Yes 是; 5 = No 否; 8 = DK 不知道

[IWER: Show next word. If R is unable to read, say the word “Village” out loud while still showing the card 访员注意：展示下一张词卡，如果受访者无法阅读，请在展示词卡【村庄】的同时大声朗读出词卡上的词语]

DC062_W4 Is this one of the words you saw earlier? 这个词您刚才见过么?
1 = Yes 是; 5 = No 否; 8 = DK 不知道

[IWER: Show next word. If R is unable to read, say the word “String” out loud while still showing the card 访员注意：展示下一张词卡，如果受访者无法阅读，请在展示词卡【绳子】的同时大声朗读出词卡上的词语]

DC063_W4 Is this one of the words you saw earlier? 这个词您刚才见过么?
1 = Yes 是; 5 = No 否; 8 = DK 不知道

[IWER: Show next word. If R is unable to read, say the word “Ticket” out loud while still showing the card 访员注意：展示下一张词卡，如果受访者无法阅读，请在展示词卡【票】的同时大声朗读出词卡上的词语]

DC064_W4 Is this one of the words you saw earlier? 这个词您刚才见过么?
1 = Yes 是; 5 = No 否; 8 = DK 不知道

[IWER: Show next word. If R is unable to read, say the word “Troops” out loud while still showing the card 访员注意：展示下一张词卡，如果受访者无法阅读，请在展示词卡【军队】的同时大声朗读出词卡上的词语]

DC065_W4 Is this one of the words you saw earlier? 这个词您刚才见过么?
1 = Yes 是; 5 = No 否; 8 = DK 不知道

[IWER: Show next word. If R is unable to read, say the word “Grass” out loud while still showing the card 访员注意：展示下一张词卡，如果受访者无法阅读，请在展示词卡【草】的同时大声朗读出词卡上的词语]

DC066_W4 Is this one of the words you saw earlier? 这个词您刚才见过么?
1 = Yes 是; 5 = No 否; 8 = DK 不知道

[IWER: Show next word. If R is unable to read, say the word "Engine" out loud while still showing the card 访员注意：展示下一张词卡，如果受访者无法阅读，请在展示词卡【发动机】的同时大声朗读出词卡上的词语]

DC067_W4 Is this one of the words you saw earlier? 这个词您刚才见过么?
1 = Yes 是; 5 = No 否; 8 = DK 不知道

END_DC Interview Observation DC 部分访员观察

DC068_W4 Does any of the following happen during the interview? 受访者访问过程中，有没有以下情况发生?

1. Poor eyesight 受访者视力不好
2. Poor hearing withour hearing-aid 受访者听力不好，未戴助听器
3. Wearing hearing-aid 受访者戴助听器
4. Shaking hands 受访者手抖，影响了某些测试
5. The interview is interrupted by some stuff or noises 访问过程中受到其他事务或噪音的干扰
6. The quality of the interview is doubted due to some emotional problems of R 受访者本身情绪问题，导致问卷质量不高
7. Others, please specify 其他，请注明 _____ （**Other_ Interviewee2**）
8. None 以上都没有

DC069_W4 What kind of language is used during the interview? 访问过程中使用的语言?

1. Mandarin 普通话
2. Local dialect 当地方言
3. Other, please specify 其他方言，请注明 _____ （**LANGUAGE_OTHER**）

DC070_W4 Whether hired local translators during interview because of language barrier? 访问过程中是否因为语言不通，使用了当地的翻译?

1. Yes 使用了当地居民帮忙翻译
2. No 未使用翻译

This page intentionally left blank

DD Informants Information 知情人信息收集

**PROCEDURE** 程序：
If respondent aged less than 60, skip this section 60 岁以下的受访者跳过此部分

INTRO_INFSELECT [INTRO: We would also like you to nominate a person, relative or friend, who can complete a short interview of about twenty minutes about you. This person should be someone who knows you well and interacts with you frequently. This person will receive $25 as a token of appreciation for his/her interview 访员指导语：我们想请您推荐一个人，他/她可以是您的亲人或者朋友，我们会请他/她参与一个和您有关的小访问，大约需要十分钟。这个人需要非常了解您，经常和您互动交流
We would like you to nominate up to three people in order of preference that you would like us to contact. We will contact the second or third person only if we are unable to complete the interview with your first nominee 我们想请您推荐最多三个人，顺序可以按照您希望我们联系的顺序。只有当我们无法联系上您的首位被推荐人时，才会联系第二位和第三位被推荐人]

1. Continue 继续
2. Respondent doesn't have any informant to give [END INTERVIEW] 受访者没有推荐任何知情人 → END 不进行知情人访问

INF1_Name What is the first person's name? 您推荐的第一个人的名字是？ ______

INF1_RTR What is this person's relationship to you? 第一位被推荐人和您是什么关系？

1. Spouse/Partner 配偶/伴侣
2. Child 孩子
3. Grandchild 孙子/女
4. Sibling 兄弟姐妹
5. Parent 父母
6. Friend 朋友
7. Guardian 监护人
8. Neighbor 邻居
9. Other 其他，please specify 请说明 ______ **(INF1_RTR_OTHER)**

NOTICE1 [IWER: Does the respondent have a second informant nominee to give? 访员注意：受访者是否有第二位知情人要推荐？]

1. Yes 是 → Skip to INF2_Name 跳至 INF2_Name
2. No 否 → Skip to INFORMANT SELECTION 跳至 INFORMANT SELECTION

INF2_Name What is the second person's name? 您推荐的第二个人的名字是？ ______

INF2_RTR What is this person's relationship to you? 第二位被推荐人和您是什么关系？

1. Spouse/Partner 配偶/伴侣
2. Child 孩子

3. Grandchild 孙子/女
4. Sibling 兄弟姐妹
5. Parent 父母
6. Friend 朋友
7. Guardian 监护人
8. Neighbor 邻居
9. Other 其他，please specify 请说明 _____ **(INF2_RTR_OTHER)**

NOTICE2 [IWER:Does the respondent have a third informant nominee to give? 访员注意：受访者是否有第三位知情人要推荐？]

1. Yes 是 → Skip to INF3_Name 跳至 INF3_Name
2. No 否 → Skip to INFORMANT SELECTION 跳至 INFORMANT SELECTION

INF3_Name What is the third person's name? 您推荐的第三个人的名字是? _____

INF3_RTR What is this person's relationship to you? 第三位被推荐人和您是什么关系?

1. Spouse/Partner 配偶/伴侣
2. Child 孩子
3. Grandchild 孙子/女
4. Sibling 兄弟姐妹
5. Parent 父母
6. Friend 朋友
7. Guardian 监护人
8. Neighbor 邻居
9. Other 其他，please specify 请说明 _____ **(INF3_RTR_OTHER)**

[IWER: Suspend blaise and contact informant nominee to schedule the interview 访员注意：在"添加联系人"处，记录能够联系到该知情人的联系电话和地址等信息，预约知情人访问]

INFORMANT_SELECTION – 知情人选择 [IWER: Who is your selected informant 访员注意：谁是您选中的知情人？]

1. INF1 [加载 INF1_Name] → Skip to VOL_STMT_IF 跳至 VOL_STMT_IF
2. INF2 [加载 INF2_Name] → Skip to INF1_NOIWREASON 跳至 INF1_NOIWREASON
3. INF3 [加载 INF3_Name] → Skip to INF1_NOIWREASON 跳至 INF1_NOIWREASON

97. Other informant 其他知情人 → Skip to INFOTHER_REASON 跳至 INFOTHER_REASON

INF1_NOIWREASON [IWER: Why was INFORMANT 1 not selected for the interviwe? 访员注意：为什么知情人 1 [加载 INF1_Name] 没有被选为参加访问的知情人？]

1. Refused 知情人 1 拒绝参加访问
2. No contact 未联系上
3. Health reasons 知情人 1 的健康原因
4. Not kowledgeable about R 对受访者不了解

5. Other 其他，please specify 请说明 _____ **(INF1_NOIWREASON_OTHER)**

PROCEDURE 程序：

If INFORMANT_SELECTION = 3, then skip to INF2_NOIWEASON 如果 INFORMANT_SELECTION = 3，则询问 INF2_NOIWREASON

INF2_NOIWREASON IWER: Why was INFORMANT 2 not selected for the interviwe? 访员注意：为什么知情人 2 没有被选为参加访问的知情人？

1. Refused 知情人 2 拒绝参加访问
2. No contact 未联系上
3. Health reasons 知情人 2 的健康原因
4. Not kowledgeable about R 对受访者不了解
5. Other, specify 其他，请说明： _____ **(INF2_NOIWREASON_OTHER)**

PROCEDURE 程序：

If INFORMANT_SELECTION = 97, then skip to INFOTHER_REASON. 如果 INFORMANT_SELECTION = 97，则询问 INFOTHER_REASON

INFOTHER_REASON IWER: Why was another informant selected for the interviwe? 访员注意：为什么选择了其他知情人参加访问？

1. Other 其他，please specify 请说明 _____ **(INFOTHER_REASON_Other)**
2. None of the original informant nominees worked out 受访者提供的知情人候选人均无法参加访问

INFOTHER_Name The name of the informant is 该知情人的姓名 _____

SECTION INTRO IF Introducing Informant Interview 介绍知情人问卷

VOL_STMT_IF [INTRO: Before we begin, I want you to know that this interview is completely voluntary. If we should come to any question that you don't want to answer, just let me know and I will go on to the next question. Your identity as a participant and any personally identifying information you provide will be kept confidential 访员指导语：在正式开始之前我想先请您了解，参与本次采访是完全自愿的。在采访过程中，如果我们问到了一些让您感到为难或不想回答的问题，您可以告诉我，我们会跳过这些题目。您在整个过程中提供的所有私人信息都会被严格地保密]

[INTRO: Thank you for agreeing to participate in this study. For this interview I will be asking you a series of thing about [preload R name], including his/her ability to complete memory, thinking, and other tasks 感谢您愿意参加本次研究项目。在此次采访中，我将询问您关于 [加载受访者姓名] 的一些事情，包括他/她完成日常活动、参与社交和认知活动的情况等]

SECTION DEMOGRAPHICS_IF Demographics of Informant 知情人基本信息

DD001_W4 What is your age? 您今年多大了？ _____ 18...110

DD002_W4 [IWER: indicate informant gender 访员注意：记录知情人的性别]

1. Male 男性
2. Female 女性

DD003_W4 What is the highest grade of school or year of college you completed? 您的最高学历?

1. No formal education 未受过教育（文盲）
2. Did not finish primary school 未读完小学，但能够读、写
3. Sishu/home school 私塾
4. Elementary school 小学毕业
5. Middle schoo 初中毕业
6. High school 高中毕业
7. Vocational school 中专（包括中等师范、职高）毕业
8. Two-/Three-Year College/Associate degree 大专毕业
9. Four-Year College/Bachelor's degree 本科毕业
10. Master's degree 硕士毕业
11. Doctoral degree/Ph.D. 博士毕业

DD004_W4 What is your relationship with [RESPONDENT NAME]? 您与 [加载受访者姓名] 的关系?

1. Spouse/Partner 配偶 → Skip to DD005_W4 跳至 DD005_W4
2. Child 子女 → Skip to DD006_W4 跳至 DD006_W4
3. Grandchild 孙子女 → Skip to DD005_W4 跳至 DD005_W4
4. Sibling 兄弟姐妹 → Skip to DD006_W4 跳至 DD006_W4
5. Parent 父母 → Skip to DD006_W4 跳至 DD006_W4
6. Friend 朋友 → Skip to DD005_W4 跳至 DD005_W4
7. Guardian 监护人 → Skip to DD005_W4 跳至 DD005_W4
8. Neighbor 邻居 → Skip to DD005_W4 跳至 DD005_W4
9. Other 其他，please specify 请说明 _____ **(DD004_W4_1)** → Skip to DD005_W4 跳至 DD005_W4

**PROCEDURE** 程序：<br>Assign DD005_W4 = 10 if DD004_W4 = 2 (Child), 4 (Sibling), 5 (Parent) 如果 DD004_W4 = 2 子女，4 兄弟姐妹，5 父母，DD005_W4 自动记录 10

DD005_W4 How many years have you known [RESPONDENT NAME]? 您与 [加载受访者姓名] 认识多久了？ _____ 1...100（可填写小数）

DD006_W4 On average in the past year how often did you see [RESPONDENT NAME]? 过去一年您与 [加载受访者姓名] 平均多久见一次?

1. Lives with respondent 与受访者住在一起
2. Daily 每天
3. Several times/week 几次/周
4. Once a week 每周一次
5. One-three times a month1-3 次/月
6. LEess than once a month<1 次/月
7. Never 从未见过
8. Other 其他，please specify 请注明 ______ **(DD006_W4_1)**

DD007_W4 Are you a caregiver for [Respondent]? 您是 [加载受访者姓名] 的照料者吗?

1 Yes 是
2 No 否

DD008_W4 Has [Respondent] been diagnosed with stroke? [加载受访者姓名] 是否被诊断为中风?

1. Yes 是
2. No 否
9. DK 不知道

DD009_W4 Has [Respondent] been diagnosed with Parkinson's disease? [加载受访者姓名] 是否被诊断为帕金森综合症?

1. Yes 是
2. No 否
9. DK 不知道

DD010_W4 Has [Respondent] been diagnosed with Alzheimer's disease? [加载受访者姓名] 是否被诊断为阿尔兹海默病?

1. Yes 是
2. No 否
9. DK 不知道

DD011_W4 Has [Respondent] been diagnosed with memory problems? [加载受访者姓名] 是否被诊断为有记忆力问题?

1. Yes 是
2. No 否
9. DK 不知道

SECTION JORM IQCODE The Jorm Informant Quesionnaire on Cognitive Decline in the Elderly 老年人认知功能减退知情者问卷

[INTRO: We want you to remember what [R NAME] was like [DD005_W4 / 10 if DD005_W4 > 10] years ago and to compare it with what (he/she) is like now. DD005_W4 / Ten years ago was in

[current year – DD005_W4 / 10 if DD005_W4 > 10] 访员指导语：我希望您能记起 [加载受访者姓名] [加载 DD005_W4, 如果DD005_W4 > 10 则为 10] 年前的情形, 来和他/她现在的情形比较。[DD005_W4, 或者 10 年] 前是指 [当前年份 – DD005_W4, 如果 DD005_W4 > 10 则为 10]。

First, we want to confirm [R NAME]'s memory and intelligence with you, regarding his/her memory on Daily rountines and past situations. Please note the importance of comparing (his/her) present performance with [DD005_W4 / 10 if DD005_W4 > 10] years ago. So if [DD005_W4 / 10 if DD005_W4 > 10] years ago [R NAME] always forgot where (he/she) had left things, and (he/she) still does, then this would be considered "Not much changed" 我首先我要请教您 [加载受访者姓名] 记忆力方面的情形，包括他/她对现在的日常生活和以前所发生的事情的记忆力。请记住，我们主要是比较 [加载受访者姓名] 现在和他/她 [加载 DD005_W4, 如果 DD005_W4 > 10 则为 10] 前的情况。所以，假如他/她在 [加载 DD005_W4，或 10 如果 DD005_W4 > 10] 前就常常忘记东西放在哪里，而现在仍然如此，就请您回答"没有什么变化"。For DD005_W4 = DK or RF, display 10 years. 如果 DD005_W4 = "不知道"或者"拒绝回答"，显示 10 年]

DD012_W4 Compared with [DD005_W4 / 10 if DD005_W4 > 10] years ago, how is [R NAME] at recognizing the face of family members and friends? 同 [加载 DD005_W4 或 10 如果 DD005_W4 > 10] 年前比，[加载受访者姓名] 现在认得出家人和熟人的面孔?

1. Much better 好多了
2. Improved 好一点
3. Not much changed 没什么变化
4. Gotten worse 差一点
5. Much worse 差多了
6. Does not apply; R doesn't do activity 不适用
8. DK 不知道
9. RF 拒绝回答

DD013_W4 Compared with [DD005_W4 / 10 if DD005_W4 > 10] yearsago, how is [R NAME] at recognizing the name of family members and friends? 同 [加载 DD005_W4 或 10 如果 DD005_W4 > 10] 年前比，[加载受访者姓名] 现在记得家人和熟朋友的名字?

1. Much better 好多了
2. Improved 好一点
3. Not much changed 没什么变化
4. Gotten worse 差一点
5. Much worse 差多了
6. Does not apply; R doesn't do activity 不适用
8. DK 不知道
9. RF 拒绝回答

DD014_W4 Compared with [DD005_W4 / 10 if DD005_W4 > 10] years ago, how is [R NAME] at remembering things about family and friends, such as occupations, birthdays, and addresses? 同 [加载 DD005_W4 或 10 如果 DD005_W4 > 10] 年前比，[加载受访者姓名] 现在记得家人和熟朋友的职业、生日、住址?

1. Much better 好多了
2. Improved 好一点
3. Not much changed 没什么变化
4. Gotten worse 差一点
5. Much worse 差多了
6. Does not apply; R doesn't do activity 不适用
8. DK 不知道
9. RF 拒绝回答

DD015_W4 Compared with [DD005_W4 / 10 if DD005_W4 > 10] years ago how is [R NAME] at Remembering things that have happened recently? 同 [加载 DD005_W4 或 10 如果 DD005_W4 > 10] 年前比，[加载受访者姓名] 认得最近发生的事情?

1. Much better 好多了
2. Improved 好一点
3. Not much changed 没什么变化
4. Gotten worse 差一点
5. Much worse 差多了
6. Does not apply; R doesn't do activity 不适用
8. DK 不知道
9. RF 拒绝回答

DD016_W4 Compared with [DD005_W4 / 10 if DD005_W4 > 10] years ago how is [R NAME] at recalling conversations a few days later? 同 [加载 DD005_W4 或 10 如果 DD005_W4 > 10] 年前比，[加载受访者姓名] 认得前几天谈话的内容?

1. Much better 好多了
2. Improved 好一点
3. Not much changed 没什么变化
4. Gotten worse 差一点
5. Much worse 差多了
6. Does not apply; R doesn't do activity 不适用
8. DK 不知道
9. RF 拒绝回答

DD017_W4 Compared with [DD005_W4 / 10 if DD005_W4 > 10] years ago how is [R NAME] at forgetting what was about to say in the middle of conversations? 同 [加载 DD005_W4 或 10 如果 DD005_W4 > 10] 年前比，[加载受访者姓名] 说话到一半就忘记了要说什么?

1. Much better 好多了
2. Improved 好一点
3. Not much changed 没什么变化
4. Gotten worse 差一点
5. Much worse 差多了
6. Does not apply; R doesn't do activity 不适用

8. DK 不知道
9. RF 拒绝回答

DD018_W4 Compared with [DD005_W4 / 10 if DD005_W4 > 10] years ago how is [R NAME] at remembering [his/her] address and telephone number? 同 [加载 DD005_W4 或 10 如果 DD005_W4 > 10] 年前比，[加载受访者姓名] 记得住址和电话?

1. Much better 好多了
2. Improved 好一点
3. Not much changed 没什么变化
4. Gotten worse 差一点
5. Much worse 差多了
6. Does not apply; R doesn't do activity 不适用
8. DK 不知道
9. RF 拒绝回答

DD019_W4 Compared with [DD005_W4 / 10 if DD005_W4 > 10] years ago how is [R NAME] at remembering what day and month it is? 同 [加载 DD005_W4 或 10 如果 DD005_W4 > 10] 年前比，[加载受访者姓名] 记得今天是星期几、是几月份?

1. Much better 好多了
2. Improved 好一点
3. Not much changed 没什么变化
4. Gotten worse 差一点
5. Much worse 差多了
6. Does not apply; R doesn't do activity 不适用
8. DK 不知道
9. RF 拒绝回答

DD020_W4 Compared with [DD005_W4 / 10 if DD005_W4 > 10] years ago how is [R NAME] at remembering where things are usually kept? 同 [加载 DD005_W4 或 10 如果 DD005_W4 > 10] 年前比，[加载受访者姓名] 记得东西经常是放在什么地方?

1. Much better 好多了
2. Improved 好一点
3. Not much changed 没什么变化
4. Gotten worse 差一点
5. Much worse 差多了
6. Does not apply; R doesn't do activity 不适用
8. DK 不知道
9. RF 拒绝回答

DD021_W4 Compared with [DD005_W4 / 10 if DD005_W4 > 10] years ago how is [R NAME] at remembering where to find things which have been put in a different place from usual? 同 [加载 DD005_W4 或 10 如果 DD005_W4 > 10] 年前比，[加载受访者姓名] 东西未归回原位，仍能找到?

1. Much better 好多了
2. Improved 好一点
3. Not much changed 没什么变化
4. Gotten worse 差一点
5. Much worse 差多了
6. Does not apply; R doesn't do activity 不适用
8. DK 不知道
9. RF 拒绝回答

DD022_W4 Compared with [DD005_W4 / 10 if DD005_W4 > 10] years ago how is [R NAME] at adapting changes in Daily life? 同 [加载 DD005_W4 或 10 如果 DD005_W4 > 10] 年前比, [加载受访者姓名] 能适应日常生活的一些改变?

1. Much better 好多了
2. Improved 好一点
3. Not much changed 没什么变化
4. Gotten worse 差一点
5. Much worse 差多了
6. Does not apply; R doesn't do activity 不适用
8. DK 不知道
9. RF 拒绝回答

DD023_W4 Compared with [DD005_W4 / 10 if DD005_W4 > 10] years ago how is [R NAME] at knowing how to work familiar machines around the house? 同 [加载 DD005_W4 或 10 如果 DD005_W4 > 10] 年前比，[加载受访者姓名] 使用家常用具的能力（如电视机、铁锤等）？

1. Much better 好多了
2. Improved 好一点
3. Not much changed 没什么变化
4. Gotten worse 差一点
5. Much worse 差多了
6. Does not apply; R doesn't do activity 不适用
8. DK 不知道
9. RF 拒绝回答

DD024_W4 Compared with [DD005_W4 / 10 if DD005_W4 > 10] years ago how is [R NAME] at learning to use a new gadget or machine around house? 同 [加载 DD005_W4 或 10 如果 DD005_W4 > 10] 年前比，[加载受访者姓名] 会学习使用新的家常用具?

1. Much better 好多了
2. Improved 好一点
3. Not much changed 没什么变化
4. Gotten worse 差一点
5. Much worse 差多了
6. Does not apply; R doesn't do activity 不适用

8. DK 不知道
9. RF 拒绝回答

DD025_W4 Compared with [DD005_W4 / 10 if DD005_W4 > 10] years ago how is [R NAME] at learning new things in general? 同 [加载 DD005_W4 或 10 如果 DD005_W4 > 10] 年前比，[加载受访者姓名] 会学习新东西的能力?

1. Much better 好多了
2. Improved 好一点
3. Not much changed 没什么变化
4. Gotten worse 差一点
5. Much worse 差多了
6. Does not apply; R doesn't do activity 不适用
8. DK 不知道
9. RF 拒绝回答

DD026_W4 Compared with [DD005_W4 / 10 if DD005_W4 > 10] years ago how is [R NAME] at remembering childhood or adolescent memories? 同 [加载 DD005_W4 或 10 如果 DD005_W4 > 10] 年前比，[加载受访者姓名] 能记住年青及童年往事?

1. Much better 好多了
2. Improved 好一点
3. Not much changed 没什么变化
4. Gotten worse 差一点
5. Much worse 差多了
6. Does not apply; R doesn't do activity 不适用
8. DK 不知道
9. RF 拒绝回答

DD027_W4 Compared with [DD005_W4 / 10 if DD005_W4 > 10] years ago how is [R NAME] at remembering things learned when he/she was young? 同 [加载 DD005_W4 或 10 如果 DD005_W4 > 10] 年前比，[加载受访者姓名] 能记住年轻时所学的东西?

1. Much better 好多了
2. Improved 好一点
3. Not much changed 没什么变化
4. Gotten worse 差一点
5. Much worse 差多了
6. Does not apply; R doesn't do activity 不适用
8. DK 不知道
9. RF 拒绝回答

DD028_W4 Compared with [DD005_W4 / 10 if DD005_W4 > 10] years ago how is [R NAME] at recognizing words which were not commonly used? 同 [加载 DD005_W4 或 10 如果 DD005_W4 > 10] 年前比，[加载受访者姓名] 懂一些不常用的字?

1. Much better 好多了
2. Improved 好一点
3. Not much changed 没什么变化
4. Gotten worse 差一点
5. Much worse 差多了
6. Does not apply; R doesn't do activity 不适用
8. DK 不知道
9. RF 拒绝回答

DD029_W4 Compared with [DD005_W4 / 10 if DD005_W4 > 10] years ago how is [R NAME] at following articles in magazines? 同 [加载 DD005_W4 或 10 如果 DD005_W4 > 10] 年前比, [加载受访者姓名] 懂报刊杂志上的文章?

1. Much better 好多了
2. Improved 好一点
3. Not much changed 没什么变化
4. Gotten worse 差一点
5. Much worse 差多了
6. Does not apply; R doesn't do activity 不适用
8. DK 不知道
9. RF 拒绝回答

DD030_W4 Compared with [DD005_W4 / 10 if DD005_W4 > 10] years ago how is [R NAME] at following a story in a book or on TV? 同 [加载 DD005_W4 或 10 如果 DD005_W4 > 10] 年前比, [加载受访者姓名] 懂电视上和书本中讲的故事?

1. Much better 好多了
2. Improved 好一点
3. Not much changed 没什么变化
4. Gotten worse 差一点
5. Much worse 差多了
6. Does not apply; R doesn't do activity 不适用
8. DK 不知道
9. RF 拒绝回答

DD031_W4 Compared with [DD005_W4 / 10 if DD005_W4 > 10] years ago how is [R NAME] at writting letters? 同 [加载 DD005_W4 或 10 如果 DD005_W4 > 10] 年前比, [加载受访者姓名] 写信表达的能力?

1. Much better 好多了
2. Improved 好一点
3. Not much changed 没什么变化
4. Gotten worse 差一点
5. Much worse 差多了
6. Does not apply; R doesn't do activity 不适用

8. DK 不知道
9. RF 拒绝回答

DD032_W4 Compared with [DD005_W4 / 10 if DD005_W4 > 10] years ago how is [R NAME] at knowing some important historical events? 同 [加载 DD005_W4 或 10 如果 DD005_W4 > 10] 年前比，[加载受访者姓名] 知道一些重要的历史事件?

1. Much better 好多了
2. Improved 好一点
3. Not much changed 没什么变化
4. Gotten worse 差一点
5. Much worse 差多了
6. Does not apply; R doesn't do activity 不适用
8. DK 不知道
9. RF 拒绝回答

DD033_W4 Compared with [DD005_W4 / 10 if DD005_W4 > 10] years ago how is [R NAME] at making decisions on everyday matters? 同 [加载 DD005_W4 或 10 如果 DD005_W4 > 10] 年前比，[加载受访者姓名] 对日常事务自己会做决定?

1. Much better 好多了
2. Improved 好一点
3. Not much changed 没什么变化
4. Gotten worse 差一点
5. Much worse 差多了
6. Does not apply; R doesn't do activity 不适用
8. DK 不知道
9. RF 拒绝回答

DD034_W4 Compared with [DD005_W4 / 10 if DD005_W4 > 10] years ago how is [R NAME] at handling money for shopping? 同 [加载 DD005_W4 或 10 如果 DD005_W4 > 10] 年前比，[加载受访者姓名] 会使用钱买东西?

1. Much better 好多了
2. Improved 好一点
3. Not much changed 没什么变化
4. Gotten worse 差一点
5. Much worse 差多了
6. Does not apply; R doesn't do activity 不适用
8. DK 不知道
9. RF 拒绝回答

DD035_W4 Compared with [DD005_W4 / 10 if DD005_W4 > 10] years ago how is [R NAME] at handling financial matters; for example, the pension, or dealing with the bank? 同 [加载 DD005_W4 或 10 如果 DD005_W4 > 10] 年前比，[加载受访者姓名] 处理财务的能力（如退休金，到银行）?

1. Much better 好多了
2. Improved 好一点
3. Not much changed 没什么变化
4. Gotten worse 差一点
5. Much worse 差多了
6. Does not apply; R doesn't do activity 不适用
8. DK 不知道
9. RF 拒绝回答

DD036_W4 Compared with [DD005_W4 / 10 if DD005_W4 > 10] years ago how is [R NAME] at handling other everyday arithmetic problems; for example, knowing how much food to buy, knowing how long between visits from family or friends? 同 [加载 DD005_W4 或 10 如果 DD005_W4 > 10] 年前比，[加载受访者姓名] 处理日常生活上的数字问题，如：知道买多少食物；知道朋友或家人上一次来访后已经有多久了？

1. Much better 好多了
2. Improved 好一点
3. Not much changed 没什么变化
4. Gotten worse 差一点
5. Much worse 差多了
6. Does not apply; R doesn't do activity 不适用
8. DK 不知道
9. RF 拒绝回答

DD037_W4 Compared with [DD005_W4 / 10 if DD005_W4 > 10] years ago how is [R NAME] at using his/her intelligence to understand what's going on and to reason things through? 同 [加载 DD005_W4 或 10 如果 DD005_W4 > 10] 年前比，[加载受访者姓名] 了解发生了什么事，并能想出适当的处理方式？

1. Much better 好多了
2. Improved 好一点
3. Not much changed 没什么变化
4. Gotten worse 差一点
5. Much worse 差多了
6. Does not apply; R doesn't do activity 不适用
8. DK 不知道
9. RF 拒绝回答

SECTION BLESSED Blessed Dementia Scale Part II Blessed 痴呆量表第二部分

[INTRO: Next I am going to ask you how well [R NAME] does with different activities 访员指导语：下面我会问您有关 [加载受访者姓名] 平时做不同活动的情况]

[INTRO: Please tell me the number you see on the screen that represents the correct answer 访员指导语：请您选择在本题下方显示的答案中最符合问题答案的选项]

DD039_W4 Regarding eating, would you say [R NAME] feeds (himself/herself) without assistance, with minor assistance, with much assistance, or has to be fed? 关于吃饭的问题，您认为 [加载受访者姓名] 可以在没有帮助下自己吃饭，还是有少量帮助、或大量帮助的情况下自己吃饭？或者他/她需要别人喂食？

1. Feeds self without assistance 无需帮助，自己吃饭
2. Feeds self with monor assistance 在少量帮助的情况下自己吃饭
3. Feeds self with much assistance 在大量帮助的情况下自己吃饭
4. Has to be fed 需要别人喂食
8. DK 不知道
9. RF 拒绝回答

[INTRO: Please tell me the number you see on the screen that represents the correct answer 访员指导语：请您选择在本题下方显示的答案中最符合问题答案的选项]

DD040_W4 Regarding using the toilet, would you say [R NAME] can clean and care for (himself/herself) at a toilet, has occasional incontinence or needs to be reminded, has frequent incontinence or needs much assistance, or has little or no control? 关于上厕所的问题，您认为 [加载受访者姓名] 可以自己上厕所并清理好自己，还是偶尔失禁、需要被提醒，还是经常失禁、需要大量帮助，还是几乎没有任何控制能力？

1. Clean, cares for self at toilet 可以自己上厕所并清理好自己
2. Occasional incontinence, or needs to be reminded 偶尔失禁，需要被提醒
3. Frequent incontinence, or needs much assistance 经常失禁，需要大量帮助
4. Little or no control 几乎没有任何控制能力
8. DK 不知道
9. RF 拒绝回答

[INTRO: Please tell me the number you see on the screen that represents the correct answer 访员指导语：请您选择在本题下方显示的答案中最符合问题答案的选项]

DD041_W4 Regarding dressing, would you say [R NAME] is able to get dressed Unaided, occasionally misplaces buttons, etc., and requires minor help, gets dressed with the wrong sequences, forgets items and requires much assistance, or is Unable to dress? 关于穿衣服的问题，您认为 [加载受访者姓名] 可以在没有帮助的情况下自己穿衣服？还是需要少量帮助，比如偶尔扣错扣子？还是需要大量帮助，比如穿衣服时顺序错乱、忘记穿某件衣物？还是完全无法穿衣？

1. Unaided 可以在没有帮助的情况下自己穿衣
2. Occasionally misplaces buttons, etc., requires minor help 需要少量帮助，比如偶尔扣错扣子
3. Wrong sequences, forgets items, requeres much assistance 需要大量帮助，比如穿衣服时顺序错乱、忘记穿某件衣物

4. Unable to dress 完全无法穿衣
8. DK 不知道
9. RF 拒绝回答

SECTION CSI-D Community Screening Instrument for Dementia Informant Part 简明社区痴呆筛查量表知情人部分

DD042_W4 Comparing with his/her condition a few years ago, would you say [R NAME]'s ability to speak is significantly worse off recently? 与几年之前比较，[加载受访者姓名] 近一段时间的办事说话能力有没有比较明显的变差了?

1. No 没有
2. Yes 有
8. DK 不知道
9. RF 拒绝回答

DD043_W4 Comparing with his/her condition a few years ago, would you say [R NAME]'s ability to think and understand is significantly worse off recently? 与几年之前比较，[加载受访者姓名] 近一段时间的思维能力和理解能力变差了?

1. No 没有
2. Yes 有
8. DK 不知道
9. RF 拒绝回答

DD044_W4 Comparing with his/her condition a few years ago, would you say [R NAME] always forgot where he/she put things recently? 与几年之前比较，[加载受访者姓名] 近一段时间经常忘记自己把东西放到哪儿了?

1. No 没有
2. Yes 有
8. DK 不知道
9. RF 拒绝回答

DD045_W4 Comparing with his/her condition a few years ago, would you say [R NAME] always forgot what happened the day before yesterday recently? 与几年之前比较，[加载受访者姓名] 近一段时间会忘记前天发生的事情吗?

1. No 没有
2. Yes 有
8. DK 不知道
9. RF 拒绝回答

DD046_W4 Comparing with his/her condition a few years ago, would you say [R NAME] sometimes could not recognize the current location? 与几年之前比较，[加载受访者姓名] 有时会忘了自己在哪里?

1. No 没有
2. Yes 有
8. DK 不知道
9. RF 拒绝回答

DD047_W4 Comparing with his/her condition a few years ago, would you say [R NAME] had difficulty dressing (for example, wrong button, could not dress or dress in the wrong sequence)? 与几年之前比较，[加载受访者姓名] 穿衣服有困难吗？（如扣错纽扣、不会穿衣服、穿衣服顺序搞错等）

[INTRO: If the difficulty is caused by physical disability, then choose “No” 访员注意：如果是身体残疾的原因所致，则选择“没有”]

1. No 没有
2. Yes 有
8. DK 不知道
9. RF 拒绝回答

END_DD Interview Observation DD 部分访员观察

[INTRO: Thank you for completing the main interview 访问结束，感谢您的参与！]

DD048_W4 Whether one of the following situations happened during the interview? 知情人访问过程中，有没有以下情况发生？

1. The informant hesitated on the questions regarding cognition and daily routines, because the respondent was on the scene 因受访者在场，知情人在回答受访者认知或日常活动的能力下降时略有犹豫
2. The quality of informant interview was questionable due to his/her cognition problems 因知情人本人的认知方面问题，导致知情人问卷质量不高
3. The quality of informant interview was low due to his/her emotional problem 因知情人本身情绪问题，导致问卷质量不高
4. The interview was interrupted by noises or other stuff 访问过程中受到其他事务或噪音的干扰
5. Other 其他，please specify 请注明 ______ **(DD048_W4_1)**
6. None of the above happed 以上都没有

DD049_W4 Language used during interview? 访问过程中使用的语言？

1. Mandarin 普通话
2. Local dialect 当地方言
3. Other, please specify 其他方言，请注明：______ **(DD049_W4_1)**

DD050_W4 Whether hired local translators during interview because of language barrier? 访问过程中是否因为语言不通，使用了当地的翻译？

1. Yes 使用了当地居民帮忙翻译

2. No 未使用翻译

DD051_W4 Telephone interview or not? 知情人模块是否通过电话访问的形式进行?

1. Yes 是
2. No 否

This page intentionally left blank

E Health Care and Insurance 医疗保健与保险

PART I Medical Insurance 医疗保险

[INTRO: Now we would like to know about health insurance or benefits that you might have 现在我们想了解一下您享受的健康保险或福利]

EA001_W4 Are you the policy holder/primary beneficiary of any of the types of health insurance listed below? (circle all that apply) 您本人目前是否参加了以下医疗保险？（可多选）

1. Urban employee medical insurance (yi-bao) 城镇职工医疗保险（医保）
2. Urban and rural resident medical insurance (integrated urban resident medical insurance and new rural cooperative medical insurance) 城乡居民医疗保险（合并城镇居民和新型农村合作医疗保险）
3. Urban resident medical insurance 城镇居民医疗保险
4. New rural cooperative medical insurance (he-zuo-yi-liao) 新型农村合作医疗保险（合作医疗）
5. Government medical insurance 公费医疗
6. Medical aid 医疗救助
7. Private medical insurance: purchased by work unit 商业医疗保险: 单位购买
8. Private medical insurance: purchased by individual 商业医疗保险: 个人购买
9. Urban non-employed persons's health insurance 城镇无业居民大病医疗保险
10. Long-term care insurance 长期护理保险
11. Other medical insurance 其他医疗保险，specify 请注明 ______ (**EA001_1**)
12. No insurance 没有保险 → Skip to EA009 跳至 EA009

[Soft Check: If pick 12, cannot pick any other, "you chose no insurance and a specific type of insurance, this is not possible" 如果选 12，不能选其他项，"您不能既选择没有保险，又选择有某一种保险"，请核实

Cannot pick 2, 3, 4 at the same time, cannot pick 1 and 5 at the same time, cannot pick (2 or 3 or 4) and (1 or 5) at the same time, "You choose contradictory insurances, please check" 不能同时选择"2 城乡居民医疗保险"、"3 城镇居民医疗保险"和"4 新型农村合作医疗"；不能同时选择"1 城镇职工医疗保险"和"5 公费医疗"；不能同时选择"3 城镇居民医疗保险"和"1 城镇职工医疗保险"。"选择的冲突的保险类型"，请核实]

[F1:（1）"城镇职工医疗保险"指依法对城镇职工的基本医疗权利给予保障的社会医疗保险制度。

（2）"城乡居民医疗保险"指一些地区率先将城镇居民医疗保险与新型农村合作医疗合并，推行统一的城乡居民医疗保险制度。

（3）"城镇居民医疗保险"指 2007 年 7 月国务院决定在全国 79 个城市实行城镇居民基本医疗保险试点。参保范围：城镇非从业居民，包括：不属于城镇职工基本医疗保险制度覆盖范围的中小学阶段的学生（包括职业高中、中专、技校学生）、少年儿童和其他非从业城镇居民。

（4）"新型农村合作医疗保险"指由政府组织、引导、支持，农民自愿参加，个人、集体和政府多方筹资，以大病统筹为主的农民医疗互助共济制度。采取个人缴费、集体扶持和政府资助的方式筹集资金。

（5）"公费医疗"指国家为保障国家工作人员身体健康而实行的一项社会保障制度。其享受对象

主要是各级国家机关、党派、团体以及文化、教育、科研、卫生、体育等事业单位的工作人员和离退休人员，二等乙级以上革命伤残军人以及在校大学生等。
(6)“医疗救助”指在政府的支持下，依靠社会力量建立的旨在向困难群体提供某些或全部基本医疗服务的制度。
(7)“商业医疗保险”是相对于社会保险而言的。商业医疗保险由商业保险公司经办，以营利为目的，企业或职工自愿参加。
(8)“其他医疗保险”如中国职工保险互助会《在职职工重大疾病互助保障计划》和《生育保险》。
(9)“医疗救助”在部分区县试点，救助标准是由各区县根据自身经济情况制定，导致救助标准有高有低。主要三种，一是提供社会医疗救助金，给救助对象以经济补偿；二是给医疗机构一定的经济补贴，使后者直接减免救助对象的部分医疗费；三是由社会医疗救助机构举办专门医疗机构，免费为救助对象提供医疗服务。
(10)“医疗保险”是指基本医疗保险，是为补偿疾病所带来的医疗费用的一种保险。职工因疾病、负伤、生育时，由社会或企业提供必要的医疗服务或物质帮助的社会保险。如中国的公费医疗、劳保医疗。
(11) 城镇无业居民大病医疗保险参保范围是具有本市非农业户籍，男满 16 周岁不满 60 周岁，女满 16 周岁不满 50 周岁，未纳入城镇职工基本医疗保险覆盖范围的居民。筹资标准为每人每年 700 元，其中个人缴纳 600 元、财政补助 100 元。
(12) 长期护理保险主要是为长期失能人员的基本生活照料和与其基本生活密切相关的医疗护理提供资金或服务保障的社会保险制度。部分地方也称其为失能人员医疗照护保险、基本照护保险、长期医疗护理保险、长期照护保险等]

PROCEDURE 程序：

If EA001_W4 = 1 – 11, skip to Procedure before EA002 如果选择了 1-11 任一种医疗保险，跳至 EA002 前面的程序

If EA001_W4 = 7, 8, skip EA002 如果参保的是商业保险，跳过 EA002

EA002 Do you have supplemental insurance to this plan? （e.g., critical illness insurance, etc.)
你有没有参加补充医疗保险？（例如大病医疗等）

1. Yes 有
2. No 无

[F1：“补充医疗保险”指由于国家的基本医疗保险只能满足参保人的基本医疗需求，超过基本医疗保险范围之外的医疗需求可以通过补充医疗保险予以补充。是相对于基本医疗保险而言的，包括企业补充医疗保险、商业医疗保险、社会互助和社区医疗保险等多种形式]

PROCEDURE 程序：

For each circled type of insurance (1-11), ask the following questions EA003_W4 - EA008
对以上选择的 1-11 的保险类型，循环提问以下问题 EA003_W4 - EA008

EA003_W4 Where did you set up your insurance account/policy? 您的保险是在哪里办的？

1. This county [Load the name of current living county] [加载现在一般居住地所在的省市县]
2. (If it is not in this county) the place of your hukou （如户口不在本县/市）户籍所在地
3. Other (Province/City/County) 其它省/市/县 ______ (**EA003_W4_1**)

EA008 When did this benefit begin? 您是什么时候参加这份保险的?

_____ 1900...2018 (**EA008_1**) Year 年 _____ 0...12 (**EA008_2**) Month 月

[IWER: Please estimate the year if respondents cannot recall the time clearly 访员注意: 若受访者不清楚参保时间，请大概估计出参保年份

Mark the year using four digits. Take down the month as its actual number. For example, write January as “1” not “01”, December as “12”. If do not remember month, fill “0” 访员注意: 用 4 位数表示年，按照实际的月份填写月。例：1 月写作“1”，而不是“01”，12 月写作“12”。如果记不住月份，请填入“0”]

PROCEDURE 程序:

If EA001_W4 ≠ 12, skip to PROCEDURE before EC001 如果受访者参加过医疗保险,跳至 EC001 前的程序

EA009 What is your main reason for not having health insurance? (circle all that apply) 您目前没有参加任何医疗保险的主要原因是什么?（可多选)

1. I do not need it 不需要
2. Cannot afford it 认为保险费太贵了
3. Do not know where or from whom to get it 不知道该去哪办
4. Do not trust the institutions that offer health insurance 不相信健康保险机构
5. Do not have suitable programs for me to buy 没有合适的保险项目
6. Never thought of it 从没有想过这个问题
7. Others 其他原因, _____ (**EA009_1**)

EB001_W4 Are you the policy holder/primary beneficiary of any of the types of health insurance listed below BEFORE? (circle all that apply) 您本人以前是否参加过以下医疗保险?（可多选）

1. Urban employee medical insurance (yi-bao) 城镇职工医疗保险（医保）
2. Urban and rural resident medical insurance (integrated urban resident medical insurance and new rural cooperative medical insurance) 城乡居民医疗保险（合并城镇居民和新型农村合作医疗保险）
3. Urban resident medical insurance 城镇居民医疗保险
4. New rural cooperative medical insurance (he-zuo-yi-liao) 新型农村合作医疗保险（合作医疗）
5. Government medical insurance 公费医疗
6. Medical aid 医疗救助
7. Private medical Insurance: Purchased by employer 商业医疗保险: 单位购买
8. Private medical Insurance: Purchased by Individual 商业医疗保险: 个人购买
9. Urban non-employed persons’s health insurance 城镇无业居民大病医疗保险
10. Long-term care insurance 长期护理险
11. Other medical insurance 其他医疗保险，please specify 请注明 ______ (**EB001_W4_1**)
12. None of the above 以上都没有

PROCEDURE 程序:

If EB001_W4 = 1 – 11, ask EB003 and EB004_W4 以前参保 EB001_W4 的是 1-11 类别的保险,依次分别询问 EB003 和 EB004_W4

EB003 When did you quit this insurance? 请问您什么时候退出这种保险的?

_____ 1900...2013 (**EB003_1**) Year 年 _____ 0...12 (**EB003_2**) Month 月

[IWER: Mark the year using four digits. Take down the month as its actual number. For example, write January as "1" not "01", December as "12". If do not remember month, fill "0" 访员注意: 用 4 位数表示年, 按照实际的月份填写月。例: 1 月写作 "1", 而不是 "01" ,12 月写作 "12"。如果记不住月份, 请填入 "0"]

EB004_W4 Why did you quit this insurance? 请问您为什么会退出这种保险 [加载退出的保险类型]?

1. Employer no longer exists 单位不存在了
2. Insurance no longer provided locally 本地不再提供这种保险
3. I resigned/ was fired from the employer 我从单位辞职/被辞退了
4. I didn't want to participate in 自己不想参加了
5. My family did not want me to participate in 家人不愿意给参加了
6. Premium was too expensive 因为保费太贵了
7. Other 其它, please specify 请注明 _____ (**EB004_W4_1**)

PART II Health Care Costs and Utilization 医疗成本与使用情况

[IWER: Please do not allow proxy to answer Part II 访员注意: "医疗成本与使用情况" 这部分不允许请别人代为回答]

PROCEDURE 程序:

For XRType = NEWIW, answer EC001 新的受访者回答 EC001

For XRType = REIW, answer EC001_W4 回访受访者回答 EC001_W4

EC001 When did you take the last physical examination? (Not including CHARLS physical examination) 您最近一次常规体检是什么时候? (注意: 不包括 CHARLS 体检)

1. _____ 1900...2018 (**EC001_1**) Year 年 _____ 0...12 (**EC001_2**) Month 月
2. Have never take physical examination yet 一辈子都没有参加过体检

[IWER: Mark the year using four digits. Take down the month as its actual number. For example, write January as "1" not "01", December as "12". If do not remember month, fill "0" 访员注意: 用 4 位数表示年, 按照实际的月份填写月。例: 1 月写作 "1", 而不是 "01" ,12 月写作 "12"。如果记不住月份, 请填入 "0"]

EC001_W4 When did you take the last physical examination since [ZIWTIME]? (Not including CHARLS physical examination) 自上次访问以来 [加载上次访问时间], 您最近一次常规体检是什么时候? 注意: 不包括 CHARLS 体检

1. _____ 1900...2018 (**EC001_W4_1**) Year 年 _____ 0...12 (**EC001_W4_2**) Month 月

2. Have never take any other physical examination since last survey 自上次访问以来没有参加过常规体检

[IWER: Mark the year using four digits. Take down the month as its actual number. For example, write January as “1” not “01”, December as “12”. If do not remember month, fill “0” 访员注意: 用 4 位数表示年，按照实际的月份填写月。例：1 月写作“1”，而不是“01”,12 月写作“12”。如果记不住月份，请填入“0”]

EC001_W3_1 Which item do you take in this physical examination? 这次常规体检，您检查了哪些项目?

1. Physical examination 体格检查
2. Routine blood test 血常规
3. Routine urine test 尿常规
4. Liver function test 肝功能
5. Kidney function test 肾功能
6. Lipids profile test 血脂三项
7. Blood glucose test 空腹血糖
8. Surgical 外科
9. Internal medicine 内科
10. Five sense organ test 五官科
11. Electrocardiogram 心电图
12. B-type ultrasonic 腹部 B 超
13. Chest fluoroscopy 胸部透视
14. Male or female specialist 男女专科
15. Other 其他, please specify 请注明 ______ **(EC001_W3_1_1**)

[INTRO: The next questions pertain to medical facilities or medical providers you may have visited for outpatient care during the past 1 month (excluding hospitalization) 下面我们要问问您过去一个月，到门诊看病或者接受治疗的情况，不包括住院]

ED001 In the last month have you visited a public hospital, private hospital, public health center, clinic, or health worker’s or doctor’s practice, or been visited by a health worker or doctor for outpatient care? (Not including physical examination) 过去一个月里，您是否去医疗机构看过门诊或者接受过上门医疗服务？(不包括做体检)

1. Yes 是
2. No 否 → Skip to EE003 跳至 EE003

ED004_W4 Which types of medical facilities have you visited in the last 4 weeks for outpatient treatment?（circle all that apply）过去一个月，您去过哪些医疗机构接受门诊治疗?（可多选）

1. General hospital (Not including traditional chinese medicine hospital) 综合医院（即全科医院，不包括中医院）
2. Specialized hospital (Not including traditional chinese medicine hospital) 专科医院（不包括中医院）

3. Chinese medicine hospital 中医院
4. Community healthcare center 社区卫生服务中心
5. Township hospital 乡镇卫生院
6. Health care post 卫生服务站
7. Village clinic/Private clinic 村诊所/私人诊所
8. Nursing home 养老机构
9. Other 其他

PROCEDURE 程序：
For each item 1-8 checked in ED004_W4, ask ED005 对 ED004_W4 选择的 1-8 的每一种医疗机构，提问 ED005

ED005 How many times did you visit/been visited by [preload ED004_W4 answer] during the last month? 过去一个月中，您去 [加载 ED004_W4的答案] 医疗机构看过几次门诊? ______ Times 次

PROCEDURE 程序：
If sum(ED005) > 1, then ask ED005_W4 and ED006_W4; otherwise, skip to ED008_W4 如果 ED005 中的次数总和 > 1, 则问 ED005_W4 和 ED006_W4；否则，就跳至 ED008_W4

ED005_W4 Did you visit the [preload ED004_W4 answer] medical facitiliy for the same disease during the last month? 过去一个月中，您去 [加载 ED004_W4的答案] 医疗机构看的都是同一种病吗?
1. Yes 是
2. No 否

ED006_W4 How much did all the visits to [preload ED004_W4 answer] spent during the last month? (Include self-paid part and reimbursement part) 您过去一个月去 [加载ED004_W4的答案] 看病的总费用大概是多少? （包括自付和报销部分的总费用）
[IWER: If possible, please check the list of hospital chargescost 访员注意：如果可能，查看一下医院收费明细单]
1. Total cost 总费用 ______ **(ED006_W4_1)** Yuan 元
   [Soft Check: upper bound: 30,000 上限：30,000]
2. Don't know 不知道
   [Brackets: 50/100/200/500/1000]

ED007 Out-of-pocket part 自付费用
1. Out-of-pocket part 其中自己花了多少钱? ______ **(ED007_1)** Yuan 元
   [Soft Check: ED007_1 ≤ ED006_W4_1, else remind "Self-paid part cannot be more than total cost" 否则提示"自己支付的费用不可能多于总费用"]
2. Didn't pay anything 没有付任何钱
3. Don't know 不知道
   [Brackets: 50/100/200/500/1000]

[INTRO: Now I'd like to ask you some questions about your most recent visit to a health care provider in the last month 下面我想了解一下您过去一个月最近一次就诊的情况]

ED008_W4 Which health care provider did you visit most recently during the past month? 过去一个月，您最近一次去了哪种医疗机构就诊?

1. Preload the health care providers in ED004_W4 加载 ED004_W4 填的医疗机构

ED009 Is this facility public or private? 这家机构是公立的还是私立的?

1. Public 公立的
2. Private 私立的

PROCEDURE 程序:
If ED008_W4 = 1 – 3, ask ED010 如果 ED008_W4 = 1 – 3，询问 ED010

ED010 What's the level of this facility? 这家医疗机构是什么级别的?

1. County/district 县/市/区级
2. Regional/city 地/市
3. Provincial/affiliated to a ministry 省/部属
4. Military 军队
5. Others 其他，please specify 请注明 ______ **(ED010_1)**
6. Not applicable 不适用

ED012 Did the provider visit you at home? 这次是不是医护人员上门服务?

1. Yes 是 → kip to ED023_W4，跳至 ED023_W4
2. No 否

ED013 How many kilometers is it from the medical facility to your residence? 从您家到这家医疗机构有几公里? ______ Km 公里
[Soft Check: upper limit 上限: 3000]

ED017 What was the purpose of your visit? (circle all that apply) 您上次去这家医疗机构的目的是什么? (可多选)

1. Immunization 免疫接种
2. Consultation 咨询
3. Medical check-up 体检
4. Treatment of illness 看病
5. Other 其他

PROCEDURE 程序:
If ED017 = 4, then ask ED018 —ED020, else ask ED023_W4 如果 ED017 答案中包含 4 "看病"，继续问 ED018 —ED020，否则询问 ED023_W4

ED018 Could you tell me the disease name? 您能告诉我您得了什么病吗? ______

ED019 Was the visit a first visit or a follow-up visit for the symptom? 这次看病是第一次就诊还是复诊?

1. First 第一次就诊
2. Follow-up 复诊

ED020 Was the visit for ordinary outpatient service or an emergency? 这次看病是普通门诊还是急诊?

1. Ordinary 普通门诊
2. Emergency 急诊

ED023_W4 What was the total cost of this visit (including both treatment and medication cost, which includes the purchase of prescription drugs from this medical facility or other pharmacies)? (Includes out-of-pocket part and reimbursement part) 这次就诊（包括药费和诊疗费，药费包括在这家医疗机构或其他药店购买医生开的处方药）总共花了多少钱?（包括自付和报销部分的总花费）

1. _____ (**ED023_W4_1**) Yuan 元
   [Soft Check: upper limit 上限: 30,000]
2. Don't know 不知道
   [Brackets: 25/50/120/400/1200]

ED024 How much did you pay out of pocket, after reimbursement from insurance? 这次就诊所花的费用里，您自己支付了多少?

1. _____ (**ED024_1**) Yuan 元
   [Soft Check: upper limit: 30,000 上限：30,000
   ED024_1 must be no more than 不能大于 ED023_W4_1, else "Pay out of pocket cannot be more than total cost" 否则提示"自己支付的费用不可能多于总费用"，请核实]
2. Did not pay anything 没有付任何钱
3. Don't know 不知道
   [Brackets: 15/30/100/300/1000]

ED028_W4 What insurance did you use or will you use? (circle all that apply) 这次就诊的费用（包含诊疗费和药费）您使用或将使用哪种医疗保险报销?（可多选）

1-11 Load the medical insurance type filled in EA001_W4 加载 EA001_W4 填的医保类型
12 Reimbursed by R's union 单位报销
13 No insurance 没有保险
14 Not revelent to R 不适用

ED029 Did you give any "red envelopes" to the doctors for this visit? 您最近一次就诊有没有给医生送红包?

1. Yes 是
2. No 否

**PROCEDURE** 程序：<br>If ED008_W4 = 8 (nursing home), ask ED030_W4 如果 ED008_W4 =8 养老机构，询问 ED030_W4

ED030_W4 Can you be reimbursed for the nursing and rehabilitation cost in the nursing home? 您在养老机构就诊的护理费用和康复费用是否可以报销?

1. Nursing expenses can be reimbursed 护理费用可以报销
2. Rehabilitation expenses can be reimbursed 康复费用可以报销
3. Both nursing and rehabilitation expenses can be reimbursed 护理费用和康复费用都可以报销
4. None can be reimbursed 都不能报销

[INTRO: The following questions pertain to hospitalization (inpatient care) that you have had during the past year 下面我们想了解一下您过去一年接受住院治疗的情况]

EE003 Have you received inpatient care in the past year? 过去一年内，您住过院吗?

1. Yes 是
2. No 否 → Skip to EF001_W4 跳至 EF001_W4

EE004 How many times have you received inpatient care during the past year? 过去一年，您接受过几次住院治疗? ______ Times 次

PROCEDURE 程序:

If EE004 = 1, skip to PROCEDURE before EE007 如果 EE004 = 1，跳至 EE007 前的程序

EE004_W4 Did you receive the several inpatient care for the same reason? 几次住院都是因为同一种原因吗?

1. Yes 是
2. No 否

EE005_W4 What was the total medical cost for all the inpatient care you received during the past year? (Include out-of-pocket part and reimbursement part. Only include fees paid to the hospital, including ward fees but excluding wages paid to a hired nurse, transportation costs, and accommodation costs for yourself or family members) 过去一年住院的总费用大概是多少? 包括自付和报销部分的总费用。只包括付给医院的费用，不包括陪护的工资、自己或家人的交通费和住宿费，但包括医院病房费。

1. Total cost 总费用 ______ (**EE005_W4_1**) Yuan 元
   [Soft Check: upper limit 上限: 300,000]
2. Don't know 不知道
   [Brackets: 1500/3000/7000/15000/30000]

EE006 Out-of-pocket part 自付费用

1. Out-of-pock part 其中自己花了多少钱 ______ Yuan 元
   [Soft Check: EE006_1 ≤ EE005_W4_1, else " Out-of-pocket part cannot be more than total cost" 否则提示"自己支付的费用不可能多于总费用"]
2. Didn't pay anything 没有付任何钱
3. Don't know 不知道
   [Brackets: 600/1500/4000/8000/18000]

[INTRO: We want details about the last hospitalization you had in the past year 我们想了解您在过去一年中，最后一次住院的详细情况]

PROCEDURE 程序：
If ED001 = 1 ask EE007 如果 ED001 = 1 询问 EE007

EE007 Is this the same facility as mentioned in ED008_W4 for outpatient care? 这家医院是过去一个月看门诊的 [ED008_W4] 那家吗?

1. Yes 是 → Skip to EE016 跳至 EE016
2. No 否

PROCEDURE 程序：
If EE007 = 2 or ED001 = 2 ask EE008_W4 - EE013 如果 EE007 = 2 或者 ED001 = 2 询问 EE008_W4 - EE013

EE008_W4 What is the type of health service facility which you visited for last inpatient care (hospital admissions) for your most recent hospitalization in the past year? 过去一年中，您最后一次接受住院治疗所在的医疗机构的类型是什么?

1. General hospital (Not including traditional chinese medicine hospital) 综合医院（即全科医院，不包括中医院）
2. Specialized hospital (Not including traditional chinese medicine hospital) 专科医院（不包括中医院）
3. Chinese Medicine Hospital 中医院
4. Community Healthcare Center 社区卫生服务中心
5. Township Hospital 乡镇卫生院
6. Health care post 卫生服务站
7. Nursing home 养老机构
8. Other 其他

EE009 Is this facility public or private? 这家医疗机构是公立的还是私立的?

1. Public 公立的
2. Private 私立的

PROCEDURE 程序：
If EE008_W4 = 1 – 3, ask EE010 如果 EE008_W4 = 1 – 3，询问 EE010

EE010 What's the administrative level of this facility? 这家医疗机构是什么级别的?

1. County/district 县/市/区级
2. Regional/city 地/市
3. Provincial/affiliated to a ministry 省/部属
4. Military 军队
5. Others 其他

EE012_W4 What is the location of this facility? 这家医疗机构的具体的地址是什么?

1. This county [加载现在一般居住地所在的省市县]
2. Other 其它省/市/县 _____ (**EE012_W4_1**)

EE013 How many kilometers is it from the medical facility to your residence? 从您家到这家医疗机构有几公里? _____ Km 公里
[Soft Check: upper limit 上限: 3000]

EE016 How many nights were you hospitalized there? 您在那里住了几晚? _____ Nights 个晚上
[Soft Check: upper limit 上限: 40]

EE017 What was the starting date of your hospital stay? 您从哪一天开始住院?
_____ 1900...2018 (**EE017_1**) Year 年 _____ 0...12 (**EE017_2**) Month 月 _____ 0...31 (**EE017_3**) Day 日
[IWER: Mark the year using four digits. Take down the month as its actual number. For example, write January as "1" not "01", December as "12". If do not remember month and day, fill "0" 访员注意: 用 4 位数表示年, 按照实际的月份填写月。例: 1 月写作 "1", 而不是 "01", 12 月写作 "12"。如果记不住月份和日期, 请填入 "0"]

EE018 What was your date of exit? 您哪天出院?
1. _____ 1900...2018 (**EE018_1**) Year 年 _____ 0...12 (**EE018_2**) Month 月 _____ 0...31 (**EE018_3**) Day 日
2. Still there 仍然在住院

[Soft Check: Date of exit should be not before starting date, "exit date is before starting date, please ask R again", also exit date should be within 1 year of today, else "exit date is one year ago, please verify the revision" 出院日期应该比住院日期要晚, 否则提示 "出院日期早于住院日期, 请再问一次受访者", 出院日期应该在从今天往前数的一年之内, 否则提示 "出院日期在一年前", 请核实修改]

EE019 Why were you hospitalized? 您住院的原因是什么?
1. Sickness 生病
2. Accident 意外 → Skip to PROCEDURE before EE024_W4 跳至 EE024_W4 前的程序
3. Violence 暴力 → Skip to PROCEDURE before EE024_W4 跳至 EE024_W4 前的程序
4. Other 其他 → Skip to PROCEDURE before EE024_W4 跳至 EE024_W4 前的程序

EE020 Could you tell me the name of the disease? 您能告诉我们您是得了什么病住院的吗? _____

EE024_W4 What was the total medical cost of hospitalization? (Only include the fees paid to the hospital, excluding the wage of hired nurse, the fare or rent, but including the ward fees) 这次住院一共花了多少钱? (只包括付给医院的费用, 不包括陪护的工资、自己或家人的交通费和住宿费, 但包括医院病房费)
1. _____ (**EE024_W4_1**) Yuan 元
   [Soft Check: upper limit 上限: 100,000]
2. Don't know 不知道
   [Brackets: 700/1500/3500/8000/15000]

EE027 How much did you or will you eventually pay out of pocket for the total costs of hospitalization? 这次住院，您最后自己总共付了（将要付）多少钱?

1. _____ (**EE027_1**) Yuan 元
   [Soft Check: upper limit: 100,000 上限：100,000
   EE027_1 must be no more than 不应该大于 EE024_W4_1，else "Pay out of pocket cannot be more than total cost" 否则提示"自己支付的费用不可能多于总费用"]
2. Didn't pay anything. 没有付任何钱
3. Don't know 不知道
   [Brackets: 400/800/2000/5000/10000]

EE031_W4 What kind of medical insurance did you use or will you use to reimburse your costs? (circle all that apply) 您使用或将使用哪种医疗保险报销?（可多选）

1-11 Load the medical insurance type filled in EA001_W4 加载 EA001_W4 填的医保类型
12 Reimbursed by Employer 单位报销
13 No insurance 没有保险
14 Not revelent to R 不适用

EE032 Did you pay any "Red Envelopes" to the doctors for this visit? 这次住院期间有没有给医生送红包?

1. Yes 是
2. No 否

PROCEDURE 程序：
If EE008_W4 =7, ask EE033_W4 如果 EE008_W4 =7 养老机构，询问 EE033_W4]

EE033_W4 Can you be reimbursed for the cost of nursing and rehabilitation at the nursing home? 您在养老机构住院的护理费用和康复费用是否可以报销?

1. Nursing expenses can be reimbursed 护理费用可以报销
2. Rehabilitation expenses can be reimbursed 康复费用可以报销
3. Both nursing and rehabilitation expenses can be reimbursed 护理费用和康复费用都可以报销
4. None can be reimbursed 都不能报销

[INTRO: Now we'd like to know whether you have treated yourself during the past month 下面我们想了解在过去一个月，您进行自我治疗的情况]

EF001_W4 Did you take any purchased medicine during the past month? (Not including prescription medications) 过去一个月, 你是否自己买药吃 (注意: 此处不包括凭处方取药的情况)?
[IWER: Taking any medicine delivered by others or stored by oneself is also counted 访员注意：服用别人送的药或自己存的药也算自己买药吃]

1. Yes 是
2. No 否 → Skip to EH001_W4 跳至 EH001_W4

EF002_W4 What is the approximate total cost for purchased medicine during the last month? (Include out-of-pocket part and reimbursement part) 过去一个月，自己买药的花费大概是多少？（包括自付和报销部分的总花费）

1. _____ (**EF002_W4_1**) Yuan 元
   [Soft Check: upper limits: 2,000 上限检查：2,000]
2. Don't know 不知道
   [Brackets: 10/30/100/200/300]

**PROCEDURE** 程序：<br>If EF002_W4 = 0, ask EH001_W4 如果 EF002_W4 = 0，询问 EH001_W4

EF003 How much did you pay out-of-pocket? 除了报销的部分，您自己支付了多少？

1. _____ (**EF003_1**) Yuan 元
   [Soft Check: upper limits: 2,000 上限：2,000
   EF003_1 should be no more than EF002_W4_1 EF003_1 不应该大于 EF002_W4_1，否则提示"自己支付的费用不可能多于总费用"，请核实]
2. Didn't pay anything. 没有付任何钱
3. Don't know 不知道
   [Brackets: 10/30/100/200/300]

EF005_W4 What insurance did you use or will you use to reimburse your costs ? (circle all that apply) 您使用或将使用哪种医疗保险报销？（可多选）

1-11 Load the medical insurance type filled in EA001_W4 加载 EA001_W4 填的医保类型
12 Reimbursed by R's union 单位报销
13 No insurance 没有保险
14 Not revelent to R 不适用

EH001_W4 Have you ever received the benefits of long-term care insurance? 您是否享受过长期护理保险的待遇？

1. Yes 是
2. No 否 → Skip to EH005_W4 跳至 EH005_W4

EH002_W4 What services did long-term care insurance pay or partially pay for? (circle all that apply) 长期护理保险帮您支付或部分支付了哪些服务的费用？(可多选)

1. Basic life care, such as bathing and turning over 基本生活照料，如洗澡、翻身
2. Common clinical care, such as nasal feeding and catheterization 常用临床护理，如鼻饲、导尿
3. Risk prevention guidance, such as fall prevention guidance 风险防范指导，如防跌倒指导
4. Functional maintenance or rehabilitation training, such as eating training, passive joint activity 功能维护或康复训练，如进食训练、关节被动活动
5. Other 其他，please specify 具体说明 _____ (**EH002_W4_1**)

EH003_W4 In the past month, your total expenditure on long-term care insurance services (including leasing and purchasing corresponding equipment) was 过去一个月，您购买长期护理险服务（包括租赁、购买相应设备）的总支出 ______ **(EH003_W4_1)** Yuan 元，of which 其中，long-term care insurance paid 长期护理保险支付 ______ **(EH003_W4_2)** Yuan 元

EH004_W4 How much does long-term care insurance help you pay for long-term care services? 长期护理保险对您支付长期护理服务费用有多大帮助?

1. Extremely helpful 极其有帮助
2. Very helpful 非常有帮助
3. Somewhat helpful 比较有帮助
4. Somewhat unhelpful 不太有帮助
5. Not helpful at all 一点也没有帮助

PROCEDURE 程序:

If R aged 60 and above, ask EH005_W4, otherwise skip to EH007_W3 60 岁及以上的人回答 EH005_W4，否则跳至 EH007_W3

EH005_W4 Have you ever receive the following home and community care services? (circle all that apply) 您是否享受了以下居家和社区养老服务?（可多选）

1. Day care centers, nursing homes, senior dining tables, etc 日间照料中心、托老所、老年餐桌等养老服务中心
2. Regular physical examination 定期体检
3. Onsite visits 上门巡诊
4. Family beds 家庭病床
5. Community nursing 社区护理
6. Health management 健康管理
7. Entertainment 娱乐活动
8. Other 其他，please specify 请注明 ______ **(EH005_W4_1)**
9. None of the above 以上均没有 → Skip to EH007_W3 跳至 EH007_W3

EH006_W4 Did you have the subsidies of home and community care services? 您是否享受了居家和社区养老服务的补贴?

1. Yes 是
2. No 否
3. Don't know 不知道

EH007_W3 Are you satisfied with the quality, cost and convenience of local medical services? Choose from vary satisfied, somewhat satisfied, neutral, somewhat dissatisfied and very dissatisfied. 您对本地医疗服务的质量、成本和方便程度满意吗? 是非常满意、比较满意、一般、比较不满意、还是一点也不满意?

1. Very satisfied 非常满意
2. Somewhat satisfied 比较满意
3. Neutral 一般

4. Somewhat dissatisfied 比较不满意
5. Very dissatisfied 一点也不满意

EH008_W4 Have you ever receive the paid family doctor services? 您是否享受了有偿的家庭医生签约服务?

1. Yes 是
2. No 否

EF006 How often did the respondent receive assistance in answering section E-Health care and insurance? 受访者填写该部分问卷时是否求助?

[IWER: If it is answered by a proxy, please record the reaction of respondent 访员注意：如果是协助回答，请记录受访者的反应]

1. Never 从未 → End of healthcare module 结束本模块
2. A few times 偶尔几次 → End of healthcare module 结束本模块
3. Most or all of the time 大多数 → End of healthcare module 结束本模块
4. The section was completed by a proxy respondent（the respondent is absent）受访者不在场，完全请人代填

EF007 What is your relationship to R? 您和受访者是什么关系?

[IWER: What is the proxy's relationship to R? If unknown, please ask the proxy 访员注意：代填问卷的人和受访者是什么关系。如果不清楚，请问代填者]

1. Spouse 配偶
2. Mother 母亲
3. Father 父亲
4. Mother-in-law 岳母/婆婆
5. Father-in-law 岳父/公公
6. Sibling 兄弟姐妹
7. Brother-in-law, sister-in-law 姐夫妹夫/嫂子弟媳
8. Child 孩子
9. Spouse of child 孩子的配偶
10. Grandchild 孙子女
11. Other relative 其他亲戚
12. Helper or other non-relative 帮忙的人或者其他非亲属

EF008 What is the main reason for proxy（the respondent is absent)? 受访者不在场，完全请人代填的主要原因是什么?

1. The respondent has serious physical handicaps 受访者有严重身体障碍
2. The respondent has serious mental handicaps, 受访者有严重精神障碍
3. The respondent has rejected this interview 受访者拒访
4. Other 其他，please specify 请注明 ______ **(EF008_1)**

This page intentionally left blank

F　Work and Retirement 工作和退休

Preloaded Variables from the Last Wave's Interview: 根据上一轮调查形成的加载变量

**ZF1** = 1	Not working in the last IW<br>上一轮调查时，当时无工作，曾有工作
**ZF1** = 2	Never worked in the last IW<br>上一轮调查时，从未工作
**ZF1** = 3	Working in the last IW<br>上一轮调查时，就业中
**ZF4** = 1	Self-employed agricultural work in the last IW<br>上一轮调查时，农业自雇
**ZF5** = 1	Only at self-employed agricultural work in the last IW<br>上一轮调查时，仅农业自雇
**ZF5** = 2	Had jobs other than self-employed agricultural in the last IW<br>上一轮调查时，有除农业自雇以外其他工作
**ZF7** = 1	Nonfarm employed in the last IW<br>上一轮调查时，非农受雇
**ZF7** = 2	Nonfarm self-employed in the last IW<br>上一轮调查时，个体私营
**ZF7** = 3	Unpaid help for family business in the last IW<br>上一轮调查时，无偿家庭帮工
**ZF7** = 4	Agricultural employed in the last IW<br>上一轮调查时，农业受雇
**ZF11** = 1	Employed in the last IW<br>上一轮调查时，受雇
**ZF11** = 2	Self-employed nonfarm in the last IW<br>上一轮调查时，非农自雇或无偿家庭帮工
**ZF13** = 1	Missing work status in the last IW<br>上一轮调查时，工作信息缺失，需要补询
**ZF14**	Name of the employer in the last IW<br>老受访者上一轮调查时的雇主名称
**ZF15**	Name of the self-employment business in the last IW<br>老受访者上一轮调查时公司或生意的名称
**ZF16**	Name of the family business without getting paid in the last IW<br>老受访者上一轮调查时不拿工资帮工的公司或生意的名称
**ZF17** = 1	Retirement processed in the last IW<br>上一轮调查时，已退休
**ZF18** = 1	Internal retirement processed in the last IW<br>上一轮调查时，已内退
**ZF19** = 1	Receding processed in the last IW<br>上一轮调查时，已退职
**ZF20** = 1	Internal retirement processed, but not yet retirement in the last IW<br>上一轮调查时，内退未退休

**ZF21** = 1	No (internal) retirement or receding processed in the last IW<br>上一轮调查时，未办理退休/退职/内退
**ZF25_1** = 1	Old R had completed retirment or receding procedures in the last IW, but the processing employer/office is missing.<br>上一轮调查时，老受访者办理了退休/退职，但是办理的单位地址缺失
**ZF25_2** = 1	Old R had completed receding procedures in the last IW, but the time is missing.<br>上一轮调查时，老受访者办理了退职，但是办理的时间缺失
**ZF25_3** = 1	Old R had completed receding procedures in the last IW, but the wage is missing.<br>上一轮调查时，老受访者办理了退职，但是退职前月工资缺失
**ZF25_4** = 1	Old R had completed retirement or early retirement.<br>上一轮调查时，老受访者办理了 [正式/提前] 退休
**ZF25_5** = 1	Old R had completed retirement or early retirement, but the time is missing.<br>上一轮调查时，老受访者办理了 [正式/提前] 退休，但是办理的时间缺失
**ZF25_6** = 1	Old R had completed retirement or early retirement, but the wage is missing.<br>上一轮调查时，老受访者办理了 [正式/提前] 退休，但是退休前月工资缺失
**ZF25_8** = 1	Old R had completed internal retirement.<br>上一轮调查时，老受访者办理了内退
**ZF25_9** = 1	Old R had completed internal retirement, but the time is missing.<br>上一轮调查时，老受访者办理了内退，但是办理的时间缺失
**ZF25_10** = 1	Old R had completed internal retirement, but the wage prior to internal retirement is missing.<br>上一轮调查时，老受访者办理了内退，但是内退前的工资缺失
**ZF25_11** = 1	Old R had completed internal retirement, but the wage at the time of internal retirement is missing.<br>上一轮调查时，老受访者办理了内退，但是内退时的工资缺失

FA Work Status 工作概况

[INTRO: Now we will ask you some questions about your work and retirement. (IWER: Work is defined as activities for earning a livelihood. If the products or services created in an activity contribute to R's livelihood, even if no income is earned, the activity should be defined as work. For example, agricultural and individual business are both considered as work) 引语：下面，我们将会询问您的工作和退休的情况。（访员注意，工作定义为以生计为目的的活动。即使活动并不带来任何货币收入，只要活动中创造的产品和服务对维持生计有显著作用，这个活动就是工作。干农活，干个体，都是工作）]

FC008 Did you engage in agricultural work for at least 10 days in the past year for your own household? Agricultural work includes farming, forestry, fishing, animal production and selling agricultral products produced by your own household. 过去一年，您有没有为自家干过农活、从事农业活动，并且至少 10 天以上？干农活指的就是从事农业生产经营活动，包括种地、管理果树、采集农林产品、养鱼、打鱼、养牲畜以及去市场销售自家生产的农产品等。

1. Yes 是
2. No 否

FC001 Did you work for other farmers/employers and get paid for at least ten days in the past year? 过去一年，您有没有为其他农户或雇主干农活挣钱，并且至少 10 天以上？

1. Yes 是
2. No 否

FA002_W4 Not including agricultural work, did you work for at least one hour last week in paid work, individual business or family business without getting paid? 除去与务农有关的工作，上周您有没有工作至少一个小时？这样的工作包括挣工资打工、从事个体与私营生意、或不拿工资为家庭经营帮工等。

1. Yes 是 → Skip to FC019_W4 BRANCHPOINT 跳至 FC019_W4 BRANCHPOINT
2. No 否

[IWER: If R has a business that involves both agricultural and non-agricultural work, please classify it as agricultural self-employed work. 访员注意：如果受访者拥有一个农业与非农业混业经营的生意，请将其归于农业自雇工作]

FA003 Are you currently engaged in any non-agricultural work but are on vacation, on sick or other leave, or in job training? 您是否有上一题问的非农工作，上周没干过（或干足一个小时），但是目前正处在临时放假、休病假、或其他假期中，或者正在在职培训？

1. Yes 是
2. No 否 → Skip to FA007_W4 BRANCHPOINT 跳至 FA007_W4 BRANCHPOINT

FA004 In which month and year did you start your leave or training? 您是什么时候开始休假或参加培训的？

______ 1900...2018 (**FA004_1**) year 年 ______ 0...12 (**FA004_2**) month 月

[IWER：Mark a year using four digits. Record a month using the calendar number. For

example, January as “1” instead of “01” and December as “12”. If R does not recall the month, fill in with “0”. 访员注意: 用 4 位数表示年，按照实际的月份填写月。例：1 月写作 “1”，而不是 “01”，12 月写作 “12”。如果记不住月份，请填入 “0”]

FA005 Do you expect to get back to this job at a definite time in the future or within 6 months? 您能够在确定的时间或者 6 个月以内，回到原来的工作么?

1. Yes 是 → Skip to FC019_W4 BRANCHPOINT 跳至 FC019_W4 BRANCHPOINT
2. No 否

FA006 Do you still receive any salaries or incomes from this work? 这个单位是否仍然给您发工资? 或者您是否仍然能从休假的工作中取得工作收入?

1. Yes 是 → Skip to FC019_W4 BRANCHPOINT 跳至 FC019_W4 BRANCHPOINT
2. No 否

FA007_W4 BRANCHPOINT 无非农工作者:

- If (FC001 = 1), skip to FC019_W4 BRANCHPOINT. 有农业受雇工作 (FC001 = 1) 跳至 FC019_W4 BRANCHPOINT
- If (FC001 = 2) & (FC008 = 1 | ZF1 = 1 | ZF1 = 3), Skip to FA010_W4_1 BRANCHPOINT. 仅农业自雇或本轮无工作上轮已知曾工作过的回访受访者 (FC001 = 2) & (FC008 = 1 | ZF1 = 1 | ZF1 = 3) 跳至 FA010_W4_1 BRANCHPOINT
- Otherwise, Skip to FA007. 无工作的新受访者及上一轮工作状态为 “从未工作” 的回访受访者跳至 FA007

FA007 Have you ever worked for at least three months during your lifetime? Work includes agricultural work, paid work, self-employed business, and unpaid help for family business. 您这辈子是否曾经工作了至少三个月的时间?（工作包括务农、挣工资打工、从事个体、私营生意或不拿工资为家庭经营帮工等）

1. Yes 是 → Skip to FA010_W4_1 BRANCHPOINT 跳至 FA010_W4_1 BRANCHPOINT
2. No 否

FA008 Work includes all kinds of labor activities other than housework, whether you earn a wage or not. Are you sure that you never worked for at least three months during your lifetime? 我们所说的工作指除家务劳动以外的所有干活的劳动，不管是不是有工资。您确定这辈子没有工作过至少三个月的时间?

1. Yes, never worked before 是的，从未工作过
2. No, ever worked. 不是，以前工作过 → Skip to FA010_W4_1 BRANCHPOINT 跳至 FA010_W4_1 BRANCHPOINT

FA009 What is the main reason for you not to work in your lifetime? 请问您不工作的主要原因是什么?

1. Disability (physical or psychological) 残疾，这里包括生理和心理上的残疾
2. Housekeeping 操持家务
3. No need to work because of family affluence 家庭富裕，不需要工作

4. Taking care of siblings 照看兄弟姐妹
5. Other, please specify 其他，请注明 _____ (**FA009_1**)

PROCEDURE 程序:
Skip to FA010_W4_1 BRANCHPOINT 跳至 FA010_W4_1 BRANCHPOINT

FC019_W4 BRANCHPOINT:
- Engaged in agricultural employed, agricultural self-employed, and non-agricultural work (FC008 = 1 & FC001 = 1 & (FA002_W4 = 1 | FA005 = 1 | FA006 = 1)), Skip to FC020_W4_a 同时从事农业自雇，农业受雇，以及非农工作 (FC008 = 1 & FC001 = 1 & (FA002_W4 = 1 | FA005 = 1 | FA006 = 1)) 跳至 FC020_W4_a
- Engaged in agricultural employed, agricultural self-employed, but not non-agricultural work (FC008 = 1 & FC001 = 1 & !(FA002_W4 = 1 | FA005 = 1 | FA006 = 1)), Skip to FC019_W4_b 同时从事农业自雇，农业受雇，且不从事非农工作 (FC008 = 1 & FC001 = 1 & !(FA002_W4 = 1 | FA005 = 1 | FA006 = 1)) 跳至 FC019_W4_b
- Engaged in agricultural self-employed, non-agricultural work, but not agricultural employed (FC008 = 1 & FC001 = 2 & (FA002_W4 = 1 | FA005 = 1 | FA006 = 1)), Skip to FC019_W4_c 同时从事农业自雇，非农工作，且不从事农业受雇 (FC008 = 1 & FC001 = 2 & (FA002_W4 = 1 | FA005 = 1 | FA006 = 1)) 跳至 FC019_W4_c
- Engaged in agricultural employed, non-agricultural work, but not agricultural self-employed (FC008 = 2 & FC001 = 1 & (FA002_W4 = 1 | FA005 = 1 | FA006 = 1)), Skip to FC020_W4_d 同时从事农业受雇，非农工作，且不从事农业自雇 (FC008 = 2 & FC001 = 1 & (FA002_W4 = 1 | FA005 = 1 | FA006 = 1)) 跳至 FC020_W4_d
- Engaged in agricultural employed, but not agricultural self-employed and non-agricultural work (FC008 = 2 & FC001 = 1 & !(FA002_W4 = 1 | FA005 = 1 | FA006 = 1)), Skip to FC019_W4_e 从事农业受雇，且不从事农业自雇和非农工作 (FC008 = 2 & FC001 = 1 & !(FA002_W4 = 1 | FA005 = 1 | FA006 = 1)) 跳至 FC019_W4_e
- Engaged in non-agricultural work, but not agricultural self-employed and agricultural employed (FC008 = 2 & FC001 = 2 & (FA002_W4 = 1 | FA005 = 1 | FA006 = 1)), Skip to FC019_W4_f 从事非农工作，且不从事农业自雇和农业受雇 (FC008 = 2 & FC001 = 2 & (FA002_W4 = 1 | FA005 = 1 | FA006 = 1)) 跳至 FC019_W4_f

[INTRO: We will now ask about your main job. Your main job is the one you currently spend most of the time at work. Short-term jobs of the same type are considered as one job 引语：我们之后将会着重询问您的主要工作。您的主要工作就是您工作时间最长的那份工作。同类型的零工，加总起来算作一份工作]

FC020_W4_a Except agricultural work for your household, what is your main job? A job can be paid agricultural work or non-agricultural work. Is this job paid agricultural work, paid non-agricultral work, individual business, or unpaid help for family business? 除去给自家干农活，您目前的主要工作是什么？这里要考虑的工作包括刚才您所说的给其他农户干农活挣钱，也包括所有的非农业工作。那么，这份主要工作具体是农活挣工资打工，非农活打工，从事个体或者私营生意，还是不拿工资为家庭经营帮工？

[IWER: Short-term jobs are temporary jobs for one employer or a variety of employers, for example, working for different agricultural households. The name of employers can be coded as “not the same household” or “not the same firm”. The workplace can be coded as the location of most employers 访员注意：同类型的零工，指的是雇主不固定且类型相同的零散工作，例如：给不同的农户割麦子，做小时工、日结临时工等。此类工作的雇主单位名称应记为“不固定居民户”或“不固定企业”等。雇主单位类型、行业、所有制等信息应记为多数单位共同的情况。雇主单位所在地为这些雇主单位的基本分布区域]

1. Non-agricultural employed 非农受雇
2. Non-agricultural self-employed 从事个体或者私营经济活动
3. Unpaid help for family business 不拿工资为家庭经营活动帮工
4. Agricultural employed 农业受雇

PROCEDURE 程序:
Skip to FA010_W4_1 BRANCHPOINT 跳至 FA010_W4_1 BRANCHPOINT

[INTRO: We will only ask about your main job. The main job refers to the one you currently spend most of the time at work. Short-term jobs of the same type are considered as one job 引语：我们之后将会只着重询问您的主要工作。您的主要工作就是您工作时间最长的那份农活打工工作。同类型的零散农活，加总起来算作一份工作]

FC019_W4_b Except agricultural work for your household, are you currently engeged in more than one paid agricultral job? 不考虑给自家干，您目前挣工资打工的农活是否不只一份？譬如说给不同的雇主干不同类型的农活。

1. Yes, at least two jobs 是，至少两份
2. No, only one job 否，只有一份

PROCEDURE 程序:
Skip to FA010_W4_1 BRANCHPOINT 跳至 FA010_W4_1 BRANCHPOINT

FC019_W4_c Short-term jobs of the same type are considered as one job. Except agricultural work for your household, are you currently engeged in more than one non-agricultural job? 同类型的零工，加总起来算作一份工作。不考虑给自家干农活，您目前的非农业工作是否不只一份?

1. Yes, at least two jobs 是，至少两份 → Skip to FC020_W4_c 跳至 FC020_W4_c
2. No, only one job 否，只有一份 → Skip to FC021_W4_c 跳至 FC021_W4_c

[IWER: Short-term jobs are temporary jobs for one employer or a variety of employers, for example, working for different agricultural households. The name of employers can be coded as “not the same household” or “not the same enterprise”. The workplace can be coded as the general location of R’s employers 访员注意：同类型的零工，指的是雇主不固定且类型相同的零散工作，例如：给不同的农户割麦子，做小时工、日结临时工等。此类工作的雇主单位名称应记为“不固定居民户”或“不固定企业”等。雇主单位类型、行业、所有制等信息应记为多数单位共同的情况。雇主单位所在地为这些雇主单位的基本分布区域]

[INTRO: We will only ask about your main job. The main job refers to the one you currently spend most of the time at work. Short-term jobs of the same type are considered as one job 引语：我们之后将会只着重询问您的主要工作。您的主要工作就是您工作时间最长的那份工作]

FC020_W4_c Except agricultural work for your household, what is your main job? Is this job paid work, individual business, or unpaid help for family business? 不考虑给自家干农活，您目前的主要非农业工作是什么？这份主要工作具体是挣工资打工，从事个体或者私营生意，还是不拿工资为家庭经营帮工？

1. Non-agricultural employed 非农受雇
2. Non-agricultural self-employed 从事个体或者私营经济活动
3. Unpaid help for family business 不拿工资为家庭经营活动帮工

FC021_W4_c How do you describe your current job? Do you earn a wage or do you run your own business or do unpaid work for family business? 您这份非农业工作是挣工资打工，从事个体或者私营生意还是不拿工资为家庭经营帮工？

1. Non-agricultural employed 非农受雇
2. Non-agricultural self-employed 从事个体或者私营经济活动
3. Unpaid help for family business 不拿工资为家庭经营活动帮工

PROCEDURE 程序：

Skip to FA010_W4_1 BRANCHPOINT 跳至 FA010_W4_1 BRANCHPOINT

[INTRO: We will only ask about your main job. The main job refers to the one you currently spend most of the time at work. Short-term jobs of the same type are considered as one job 引语：我们之后将会只着重询问您的主要工作。您的主要工作就是您工作时间最长的那份工作。同类型的零工，加总起来算作一份工作]

FC020_W4_d What is your current main job? A job can be paid agricultural work or non-agricultural work. Is this job paid agricultural work, paid non-agricultral work, individual business, or unpaid help for family business? 您目前的主要工作是什么？这里须考虑的工作包括刚才您所说的给其他农户干农活挣钱，也包括所有的非农业工作。那么，这份主要工作具体是农活挣工资打工，非农活打工，从事个体或者私营生意，还是不拿工资为家庭经营帮工？ [IWER: Short-term jobs are temporary jobs for one employer or a variety of employers, for example, working for different agricultural households. The name of employers can be coded as “not the same household” or “not the same enterprise”. The workplace can be coded as the general location of R’s employers 访员注意：同类型的零工，指的是雇主不固定且类型相同的零散工作，例如：给不同的农户割麦子，做小时工、日结临时工等。此类工作的雇主单位名称应记为“不固定居民户”或“不固定企业”等。雇主单位类型、行业、所有制等信息应记为多数单位共同的情况。雇主单位所在地为这些雇主单位的基本分布区域]

1. Non-agricultural employed 非农受雇
2. Non-agricultural self-employed 从事个体或者私营经济活动
3. Unpaid help for family business 不拿工资为家庭经营活动帮工
4. Agricultural employed 农业受雇

PROCEDURE 程序:
Skip to FA010_W4_1 BRANCHPOINT 跳至 FA010_W4_1 BRANCHPOINT

[INTRO: We will only ask about your main job. The main job refers to the one you currently spend most of the time at work. Short-term jobs of the same type are considered as one job 引语: 我们之后将会只着重询问您的主要工作。您的主要工作就是您工作时间最长的那份农活打工工作。同类型的零散农活, 加总起来算作一份工作]

FC019_W4_e Are you currently engeged in more than one paid agricultural job? 您目前挣工资打工的农活是否不只一份? 譬如说给不同的雇主干不同类型的农活。
1. Yes, at least two jobs 是, 至少两份
2. No, only one job 否, 只有一份

PROCEDURE 程序:
Skip to FA010_W4_1 BRANCHPOINT 跳至 FA010_W4_1 BRANCHPOINT

FC019_W4_f Short-term jobs of the same type are considered as one job. Are you currently engeged in more than one non-agricultural job? 同类型的零工, 加总起来算作一份工作。您目前的非农业工作是否不只一份?
1. Yes, at least two jobs 是, 至少两份 → Skip to FC020_W4_f 跳至 FC020_W4_f
2. No, only one job 否, 只有一份 → Skip to FC021_W4_f 跳至 FC021_W4_f

[IWER: Short-term jobs are temporary jobs for one employer or a variety of employers, for example, working for different agricultural households. The name of employers can be coded as "not the same household" or "not the same enterprise". The workplace can be coded as the general location of R's employers 访员注意: 同类型的零工, 指的是雇主不固定且类型相同的零散工作, 例如: 给不同的农户割麦子, 做小时工、日结临时工等。此类工作的雇主单位名称应记为"不固定居民户"或"不固定企业"等。雇主单位类型、行业、所有制等信息应记为多数单位共同的情况。雇主单位所在地为这些雇主单位的基本分布区域]

[INTRO: We will only ask about your main job. The main job refers to the one you currently spend most of the time at work. Short-term jobs of the same type are considered as one job 引语: 我们之后将会只着重询问您的主要工作。您的主要工作就是您工作时间最长的那份工作]

FC020_W4_f What is your main non-agricultural job? Is this job paid work, individual business or unpaid help for family business? 您目前的主要非农业工作是什么? 这份主要工作具体是挣工资打工, 从事个体或者私营生意, 还是不拿工资为家庭经营帮工?
1. Non-agricultural employed 非农受雇
2. Non-agricultural self-employed 从事个体或者私营经济活动
3. Unpaid help for family business 不拿工资为家庭经营活动帮工

FC021_W4_f How do you describe your current job? Do you earn a wage or do you run your own business or do unpaid work for family business? 您这份非农业工作是挣工资打工, 从事个体或者私营生意还是不拿工资为家庭经营帮工?

1. Non-agricultural employed 非农受雇
2. Non-agricultural self-employed 从事个体或者私营经济活动
3. Unpaid help for family business 不拿工资为家庭经营活动帮工

FA010_W4_1 BRANCHPOINT:
Generate variables of work status 生成本轮工作状态变量:

- XF1 = 1 if currently not working 若现无工作 (FC008 = 2 & FC001 = 2 & (FA003 = 2 | FA006 = 2) & !(FA008 = 1))
- XF1 = 2 if never works 若从未工作 (FA008 = 1)
- XF1 = 3 if working 若就业中 (FC008 = 1 | FC001 = 1 | FA002_W4 = 1 | FA005 = 1 | FA006 = 1)
- XF4 = 1 if self-employed agricultural 若农业自雇 (FC008 = 1)
- XF5 = 1 if self-employed agricultural is the only job 若仅农业自雇 (FC008 = 1 & ! (FC001 = 1 | FA002_W4 = 1 | FA005 = 1 | FA006 = 1))
- XF5 = 2 if work other than self-employed agricultural 若有除农业自雇以外其他工作 (FC001 = 1 | FA002_W4 = 1 | FA005 = 1 | FA006 = 1)

Generate variables of main job types 生成本轮主要工作类型变量:

- XF7 = 1 if nonfarm employed 若非农受雇 (FC020_W4_a = 1 | FC020_W4_c = 1 | FC020_W4_d = 1 | FC020_W4_f = 1 | FC021_W4_c = 1 | FC021_W4_f = 1)
- XF7 = 2 if nonfarm self-employed 若个体私营 (FC020_W4_a = 2 | FC020_W4_c = 2 | FC020_W4_d = 2 | FC020_W4_f = 2 | FC021_W4_c = 2 | FC021_W4_f = 2)
- XF7 = 3 if unpaid help for family business 若家庭帮工 (FC020_W4_a = 3 | FC020_W4_c = 3 | FC020_W4_d = 3 | FC020_W4_f = 3 | FC021_W4_c = 3 | FC021_W4_f = 3)
- XF7=4 if agricultural employed 若农业受雇 (FC020_W4_a = 4 | FC020_W4_d = 4 | (FC019_W4_b = 1 | FC019_W4_b = 2 | FC019_W4_e = 1 | FC019_W4_e = 2))
- XF11 = 1 if employed 若受雇 (XF7 = 1 | XF7 = 4)
- XF11 = 2 if self-employed nonfarm 若非农自雇及帮工 (XF7 = 2 | XF7 = 3)

BRANCHPOINT 跳转:

- For old R missing work status in the last IW 上轮工作缺失的旧受访者 (XRTYPE = REIW & ZF13 = 1), Skip to FA006_W3_1 跳至 FA006_W3_1
- For new R 新受访者 (XRTYPE = NEWIW), Skip to FA_END_W4 BRANCHPOINT 跳至 FA_END_W4 BRANCHPOINT
- Otherwise 上轮工作未缺失旧受访者 Skip to FA010_W4_2 BRANCHPOINT 跳至 FA010_W4_2 BRANCHPOINT

Update Missing Work Status in the Last Visit 上轮工作状态补询子模块

[INTRO: Due to incomplete answers on your work information in the last visit, we will ask you some questions about your job at that time 引语：由于上次访问时我们没能完整记录您的工作情况，下面，我们将会询问一些您当时的工作情况]

FA006_W3_1 Did you engage in agricultural work (including farming, forestry, fishing, and animal production for your own household or others) in the last IW? 在 [加载上一轮调查时间 ZIWTime]，您有没有从事农业生产经营活动? 农业生产经营活动包括为自家或其他农户种地、管理果树、采集农林产品、养鱼、打鱼、养牲畜以及去市场销售自家生产的农产品等

1. Yes 有
2. No 没有 → Skip to FA006_W3_3 跳至 FA006_W3_3

FA006_W3_2 What agricultural work did you engage in the last IW? (Select all that apply) 您当时从事的农业生产经营活动是?（可多选）

1. Household agricultural work 自家农业生产活动
2. Agricultural employed 为其他农户/农场打工

FA006_W3_3 Did you engage in non-agricultural work in the last IW? We consider any of the following activities to be at work: earn a wage, run your own business and unpaid work for family business. Work does not include doing your own housework or doing activities without pay, such as volunteer work. 在 [加载上一轮调查时间 ZIWTime]，您有没有从事非农工作? 非农工作包括挣工资工作、从事个体、私营经济活动或不拿工资为家庭经营活动帮工等，但不包括家务劳动、义务的志愿劳动。

1. Yes 有
2. No 没有 → Skip FA006_W3_4 跳过 FA006_W3_4

FA006_W3_4 What non-agricultural work did you do in the last IW? 您当时主要从事的非农工作是?

1. Employed 挣工资工作
2. Self-employed 从事个体、私营经济活动
3. Unpaid help for family business 不拿工资为家庭经营活动帮工

PROCEDURE 程序: Update preloaded work variables from the last IW 更新上轮工作状态变量 **XZF**

- XZF1 = 1 if then not working 若当时无工作，曾有工作 (FA006_W3_1 = 2 & FA006_W3_3 = 2)
- XZF1 = 2 if never works 若从未工作 (不更新)
- XZF1 = 3 if working 若就业中 (FA006_W3_1 = 1 | FA006_W3_3 = 1)
- XZF4 = 1 if self-employed agricultural 若农业自雇 (FA006_W3_2s1 = 1)
- XZF5 = 1 if self-employed agricultural is the only job 若仅农业自雇 (FA006_W3_2s1 = 1 & !(FA006_W3_2s2 = 1) & FA006_W3_3 = 2)
- XZF5 = 2 if work other than self-employed agricultural 若其他工作非仅农业自雇 (FA006_W3_2s2 = 2 | FA006_W3_3 = 1)
- XZF7 = 1 if nonfarm employed 若非农受雇 (FA006_W3_4 = 1)
- XZF7 = 2 if nonfarm self-employed 若个体私营 (FA006_W3_4 = 2)
- XZF7 = 3 if unpaid help for family business 若无偿家庭帮工 (FA006_W3_4 = 3)
- XZF7 = 4 if agricultural employed 若农业受雇 (FA006_W3_2s2 = 2)
- XZF11 = 1 if employed 若受雇 (FA006_W3_2s2 = 2 | FA006_W3_4 = 1)
- XZF11 = 2 if self-employed nonfarm 若非农自雇及无偿家庭帮工 (FA006_W3_4 = 2 | FA006_W3_4 = 3)
- XZF13 = 0 work status in the last IW updated 就业状态信息已不缺失

Job Switch 两轮工作状态转换确认子模块

FA010_W4_2 BRANCHPOINT:

- Not working → Working 上轮无工作 → 本轮就业 (XZF1 = 1 & XF1 = 3), Skip to FA010_W4_1 跳至 FA010_W4_1
- Working → Not working 上轮就业 → 本轮无工作 (XZF1 = 3 & XF1 = 1), Skip to FA011_W4_1 跳至 FA011_W4_1
- Self-employed agricultural only → Other jobs → 本轮其他工作 (XZF5 = 1 & XF5 = 2), Skip to FA012_W4_1 跳至 FA012_W4_1
- Other jobs → Self-employed agricultural only 上轮其他工作 → 本轮仅农业自雇 (XZF5 = 2 & XF5 = 1), Skip to FA013_W4_1 跳至 FA013_W4_1
- Employed → Nonfarm self-employed 上轮受雇 → 本轮非农自雇 (XZF11 = 1 & XF11 = 2), Skip to FA014_W4_1 跳至 FA014_W4_1
- Nonfarm self-employed → Employed 上轮非农自雇 → 本轮受雇 (XZF11 = 2 & XF11 = 1), Skip to FA015_W4_1 跳至 FA015_W4_1
- Otherwise 其他受访者, Skip to FA_END_W4 BRANCHPOINT 跳至 FA_END_W4 BRANCHPOINT

FA010_W4_1 In the last IW you told us you did not work, so between the last IW and now, when did you start working? 上一次访问时您曾告诉我们，在 [加载上一轮调查时间 ZIWTime] 您当时没有工作，那么从那时起到现在，您是什么时候开始工作的？

1. _____ (**FA010_W4_1_1**) year 年 _____ (**FA010_W4_1_2**) month 月
2. I was working 那时我有工作 → Skip to FA006_W3_1 跳至 FA006_W3_1

[Hard Check: FA010_W4_1_1 ≥ ZIWTime
IWER: Job switch happened later than the last IW. If the respondent can not remember the time, please code as –1 访员注意：工作转换时间应晚于上一轮调查时间。如果受访者无法记起具体年月，请填 –1]

FA010_W4_3 What was the reason for you to start to work? 您开始工作的主要原因是什么？

1. Economic reasons, need more income 经济原因，需要更多收入
2. Bad health status in the past 之前自身健康状况不理想
3. Caring family members in the past 之前在照料家庭成员
4. Personal development in the past, e.g. short leave, study, go abroad, traveling etc. 之前在进行个人发展，如短期休息、上学、出国、长途旅游等
5. Others, please specifiy 其他，请说明 _____ **(FA010_W4_3_1)**

FA010_W4_4 Please describe your first job after you started to work recently. 请您描述一下结束无工作状态时从事的那一份工作（的类型）：

1. Non-agricultral employoed 非农受雇
2. Non-agricultral self-employed 从事个体或者私营经济活动
3. Unpaid nonfarm family business 不拿工资为家庭经营活动帮工
4. Agricultral employed 农业受雇
5. Agricultral self-employed 农业自雇

PROCEDURE 程序：
Skip to FA_END_W4 BRANCHPOINT 跳至 FA_END_W4 BRANCHPOINT

FA011_W4_1 In the last IW you told us you were working. Work refers to agricultural work, paid work, individual business, unpaid help for family business. When did you end that work? 上一次访问时您曾告诉我们，在 [加载上一轮调查时间 ZIWTime] 您当时有工作，这里所指的工作包括干农活、挣工资打工、从事个体或者私营经济活动、不拿工资为家庭经营活动帮工。那么从那时起到现在，您是什么时候停止工作的？

1. _____ **(FA011_W4_1_1)** year 年 _____ **(FA011_W4_1_2)** month 月 → Skip to 跳至 FA011_W4_2
2. I did not work at that time 那时我没有工作 → Update variable 更新变量 XZF1 = 1
   [Hard Check: FA011_W4_1_1 ≥ ZIWTime
   IWER: Job switch happened later than the last IW. If the respondent can not remember the time, please code as –1. 访员注意：工作转换时间应晚于上一轮调查时间。如果受访者无法记起具体年月，请填 –1]

FA011_W4_1_1 When did you end your last job? 那么请问您是什么时候停止最近/最后一份工作的？

_____ **(FA011_W4_1_1_1)** year 年 _____ **(FA011_W4_1_1_2)** month 月
[IWER: If the respondent can not remember the time, please code as –1 访员注意：如果受访者无法记起具体年月，请填 –1]

FA011_W4_2 Please describe your last job. 请您描述一下最近那一份工作（的类型）：

1. Non-agricultral employoed 非农受雇
2. Non-agricultral self-employed 从事个体或者私营经济活动
3. Unpaid nonfarm family business 为家庭经营活动帮工
4. Agricultral employed 农业受雇
5. Agricultral self-employed 农业自雇

FA011_W4_3 What was the reason for you to stop doing this job? 您停止工作的主要原因是什么?

1. Forced to leave, e.g. being fired 被动原因失去最近一份工作，如被单位辞退、店面被拆、土地被征用等
2. Health reason 自身健康状况
3. Caring for family members 照料家庭成员
4. Retirement 到了退休年龄而离开工作岗位
5. Others, please specifiy 其他，请说明 _____ (**FA011_W4_3_1**)

FA011_W4_4 Upon leaving your last job, did you receive any severance package other than retirement allowance? (For example, lump-sum payment, condolence payment, etc.) 最近一份工作结束时，除去退休工资，您是否还得到了其他补偿？如买断工龄，伤残津贴等。

1. Yes 是
2. No 否 → Skip to FA_END_W4 BRANCHPOINT 跳至 FA_END_W4 BRANCHPOINT

[IWER: The "lump-sum payment" refers to the onetime payment that state-owned companies offered to its workers upon terminating contract with them in the 1980s and 1990s, when the companies were going through privatization and laying off redundant employees. The specific amount of the payment would be bargained by both sides based on the previous position, wage level, etc. of the employee, as well as the situation of the company 访员注意:"买断工龄"指改革开放初期我国一些国有企业在改革过程中安置富余人员的一种办法，即参照员工在企业的工作年限、工资水平、工作岗位等条件，结合企业的实际情况，经企业与员工双方协商，报有关部门批准，由企业一次性支付给员工一定数额的货币，从而解除企业和富余员工之间的劳动关系，把员工推向社会的一种形式]

FA011_W4_5 What is the severance pay? 您得到了多少补偿? _____ (**FA011_W4_5_1**) Yuan 元; The pay is based on what? 补偿原因/依据是? _____ (**FA011_W4_5_2**) For lump-sum payment, what was the length of your service used for its calculation? 如果是因为买断工龄，请问是按照多少年工龄算的? _____ 0...120 (**FA011_W4_5_3**) Year 年

FA011_W4_bracket [IWER: If R is unwilling to answer or does not remember, ask unfolding bracket questions here 访员注意：如果受访者不愿回答或者忘记了或者填写 0，在此处分级展开提问] 1000 /2000 /5000 /10,000 /20,000 Yuan 元

**PROCEDURE** 程序:
Skip to FA_END_W4 BRANCHPOINT 跳至 FA_END_W4 BRANCHPOINT

FA012_W4_1 In the last IW you told us your work is household agricultural work. Between that time and now, when did you start your other new job(s)? 上一次访问时您曾告诉我们，在 [加载上一轮调查时间 ZIWTime] 您的工作只是给自家干农活，那么从那时起到现在，您是什么时候有了其他工作的?

1. _____ (**FA012_W4_1_1**) year 年 _____ (**FA012_W4_1_2**) month 月
2. I did not do household agricultural work at that time. 那时我并不在家干农活或并不只是在家干农活 → Skip to FA006_W3_1 跳至 FA006_W3_1
   [Hard Check: FA012_W4_1_1 ≥ ZIWTime
   IWER: Job switch happened later than the last IW. If the respondent can not remember the time, please code as –1 访员注意：工作转换时间应晚于上一轮调查时间。如果受访者无法记起具体年月，请填 –1]

FA012_W4_2 Not including household agricultural work, when did you start your current main job? 除了干自家农活以外，您什么时候开始有了我们刚才谈到的这份（主要）工作?
_____ (**FA012_W4_2_1**) year 年 _____ (**FA012_W4_2_2**) month 月
[Hard Check: FA012_W4_2_1 ≥ FA012_W4_1_1
IWER: The start time of your current/most recent job should not be earlier than the job switch time. If the respondent can not remember the time, please code as –1 访员注意：最近工作开始时间应不早于工作开始转换时间。如果受访者无法记起具体年月，请填 –1]

FA012_W4_3 What is the reason for switching from household agricultural work only to other work? 您从只给自家干农活，转变到有了其他的工作，主要原因是什么?

1. Forced to switch, e.g. having lost farm land 被动原因，如土地被征用
2. Switch job for higher income 收入更高，主动转换工作
3. Health reason 自身健康因素，工作强度更适合自己的身体条件
4. Caring for family members 与照料家庭成员有关
5. Moving, e.g. leaving rural villages 住处搬迁，自主离开农村
6. Others, please specifiy 其他，请说明 _____ (**FA012_W4_3_1**)

PROCEDURE 程序:
Skip to FA_END_W4 BRANCHPOINT 跳至 FA_END_W4 BRANCHPOINT

FA013_W4_1 In the last IW you told us you were working, besides doing household agricultural work. Between that time and now, when did you end other work? 上一次访问时您曾告诉我们，在 [加载上一轮调查时间 ZIWTime] 您还从事给自家干农活以外其他的工作，那么从那时起到现在，您什么时候停止了所有不是自家务农的工作?

1. _____ (**FA013_W4_1_1**) year 年 _____ (**FA013_W4_1_2**) month 月
2. I had no other job then. 那时我并没有其他的工作 Skip to FA006_W3_1 跳至 FA006_W3_1
   [Hard Check: FA013_W4_1_1 ≥ ZIWTime
   IWER: Job switch happened later than the last IW. If the respondent can not remember the time, please code as –1 访员注意：工作转换时间应晚于上一轮调查时间。如果受访者无法记起具体年月，请填 –1]

FA013_W4_2 When did you start doing household agricultural work only? 请问您是什么时候开始只在自家务农?
_____ (**FA013_W4_2_1**) year 年 _____ (**FA013_W4_2_2**) month 月
[Hard Check: FA013_W4_2_1 ≥ FA013_W4_1_1

IWER: The starting time of doing household agricultural work only should not be earlier than the job switching time. If the respondent can not remember the time, please code as –1 访员注意：开始务农时间应不早于工作开始转换时间。如果受访者无法记起具体年月，请填 –1]

FA013_W4_3 What is your reason for switching from having other jobs to household agricultural work only? 您从当时有其他的工作，转变到只给自家干农活，主要原因是什么？

1. Forced to leave the last job, e.g. being fired 被动原因失去最后一份工作，如被单位辞退、店面被拆等
2. Switch job for higher income 收入更高，主动转换工作
3. Health reason 自身健康因素，工作强度更适合自己的身体条件
4. Caring for family members 与照料家庭成员有关
5. Moving, e.g. leaving rural villages 住处搬迁，自主从城镇返回农村
6. Retirment 到了退休年龄而离开工作岗位
7. Others, please specifiy 其他，请说明 _____ (**FA013_W4_3_1**)

PROCEDURE 程序:
Skip to FA_END_W4 BRANCHPOINT 跳至 FA_END_W4 BRANCHPOINT

FA014_W4_1 In the last IW you told us you worked largely for some other employers. Between that time and now, when did you end this kind of employed work? 上一次访问时您曾告诉我们，在 [加载上一轮调查时间 ZIWTime] 您大部分时间在为其他单位或雇主工作，那么从那时起到现在，您的工作什么时候不再以这样的工作为主？

1. _____ (**FA014_W4_1_1**) year 年 _____ (**FA014_W4_1_2**) month 月
2. I didn't work for some other employers at that time. 那时我并没有为其他单位或雇主工作 → Skip to FA006_W3_1 跳至 FA006_W3_1
   [Hard Check: FA014_W4_1_1 ≥ ZIWTime
   IWER: Job switch happened later than the last IW. If the respondent can not remember the time, please code as –1 访员注意：工作转换时间应晚于上一轮调查时间。如果受访者无法记起具体年月，请填 –1]

FA014_W4_2 When did you start working largely with your current business, or for family business without pay? 请问您是什么时候开始大部分时间在从事现在的个体/私营生意，或无偿为家庭经营帮工？

_____ (**FA014_W4_2_1**) year 年 _____ (**FA014_W4_2_2**) month 月

[Hard Check: FA014_W4_2_1 ≥ FA014_W4_1_1

IWER: Individual/helper work should not start earlier than the job switching time. If the respondent can not remember the time, please code as –1 访员注意：自雇/帮工工作开始时间应不早于工作转换开始时间。如果受访者无法记起具体年月，请填 –1]

FA014_W4_3 What is your reason for switching from wage earning jobs to individual business or family business unpaid work? 您的主要工作从当时从事挣工资的工作（受雇），转变到个体/私营生意/或无偿为家庭经营帮工，主要原因是什么？

1. Inherit business or get business from relatives 继承接班或从亲属处取得生意控制权（无偿取得，不包含购入）
2. Forced to leave, e.g. being fired 被动原因失去受雇工作，如被单位辞退等
3. Higher income in self-employed business, or due to family business reasons 自雇收入更高，或家庭经营活动需要，而主动转换工作
4. Health reasons 自身健康因素，工作强度更适合自己的身体条件
5. Caring for family members 与照料家庭成员有关
6. Retirement 到了退休年龄而离开工作岗位
7. Others, please specify 其他，请说明 _____ **(FA014_W4_3_1)**

PROCEDURE 程序:

If FA014_W4_3 $\neq$ 1, Skip to FA_END_W4 BRANCHPOINT 若 FA014_W4_3 $\neq$ 1 继承接班，跳至 FA_END_W4 BRANCHPOINT

FA014_W4_4 From which relative did you get the individual business? 您现在的个体/私营生意从哪位亲属处取得?

1. Parents 父母
2. Parents in law 配偶父母
3. Spouse 配偶
4. Siblings 兄弟姐妹
5. Children (preload names of children) 子女（此处加载所有 [受访者的姓名] 的子女姓名）_____ **(FA014_W4_4_1)**
6. Relatives, please specify the relationship 其他亲属,请说明亲属关系 _____ **(FA014_W4_4_2)**

PROCEDURE 程序:

Skip to FA_END_W4 BRANCHPOINT 跳至 FA_END_W4 BRANCHPOINT

FA015_W4_1 In the last IW you told us you worked largely for individual business or family business unpaid work. Between that time and now, when did you end that job? 上一次访问时您曾告诉我们，在 [加载上一轮调查时间 ZIWTime] 您大部分时间在从事个体/私营生意/也包括无偿为家庭经营帮工，那么从那时起到现在，您的工作什么时候不再以这样的工作为主?

1. _____ **(FA015_W4_1_1)** year 年 _____ **(FA015_W4_1_2)** month 月
2. I did not have a non-farm self-employed job then. 那时我并没有非农业的自雇工作
   Skip to FA006_W3_1 跳至 FA006_W3_1
   [Hard Check: FA015_W4_1_1 $\geq$ ZIWTime
   IWER: Job switch happened later than the last IW. If the respondent can not remember the time, please code as –1 访员注意：工作转换时间应晚于上一轮调查时间。如果受访者无法记起具体年月，请填 –1]

FA015_W4_2 When did you start your current paid work? 请问您是什么时候开始大部分时间在从事现在的挣工资受雇工作?

_____ **(FA015_W4_2_1)** year 年 _____ **(FA015_W4_2_2)** month 月
[Hard Check: FA015_W4_2_1 $\geq$ FA015_W4_1_1

IWER: The start time of this employed job should not be earlier than the job switching time. If the respondent can not remember the time, please code as −1 访员注意：受雇工作开始时间应不早于工作转换开始时间。如果受访者无法记起具体年月，请填 −1]

FA015_W4_3 What is the reason for switching from individual business or unpaid work for family business to this wage earning employed job? 您的主要工作从当时从事个体/私营生意/或无偿为家庭经营帮工，转换到现在挣工资受雇工作，主要原因是什么？

1. Inherited by children or transfered to relatives 子女继承接班或移交给亲属（无偿赠与，不包含出售）
2. Forced to leave, e.g. being fired 被动原因失去自雇工作，如店面被拆等
3. Higher income 收入更高，主动转换工作
4. Health reasons 自身健康因素，工作强度更适合自己的身体条件
5. Caring for family members 与照料家庭成员有关
6. Family or economic conflicts 家庭矛盾或家庭经济纠纷
7. Others, please specify 其他，请说明 _____ **(FA015_W4_3_1)**

PROCEDURE 程序:

If FA015_W4_3 $\neq$ 1, Skip to FA_END_W4 BRANCHPOINT 若 FA015_W4_3 $\neq$ 1 亲属继承接班，跳至 FA_END_W4 BRANCHPOINT

FA015_W4_4 Which relative did you transfer the business to? 您当时的个体/私营生意移交给了哪位亲属？

1. Parents 父母
2. Parents in law 配偶父母
3. Spouse 配偶
4. Siblings 兄弟姐妹
5. Children (preload names of children) 子女（此处加载所有 [受访者的姓名] 的子女姓名) _____ **(FA015_W4_4_1)**
6. Spouse of children (preload names of children) 子女配偶（此处加载所有 [受访者的姓名] 的子女姓名） _____ **(FA015_W4_4_2)**
7. Grandchildren (preload names of children who are the transferees' parents) 孙子女（此处加载所有 [受访者的姓名] 的子女姓名） _____ **(FA015_W4_4_3)**
8. Relatives, please specify the relationship 其他亲属，请说明亲属关系，请说明 _____ **(FA015_W4_4_4)**

FA_END_W4 BRANCHPOINT 工作类型分支:

- Never worked 现无或从未工作 (XF1 = 1 | XF1 = 2), Skip to FK002 跳至 FK002
- Agricultral self-employed 农业自雇 (XF4 = 1), Skip to FC022_W4 跳至 FC022_W4
- Employed but not agricultral self-employed 受雇且无农业自雇 (XF11 = 1 & ! XF4 = 1), Skip to FD001 跳至 FD001
- Non-farm self-employed but not agricultral self-employed (otherwise) 非农自雇且无农业自雇 Skip to FH001 跳至 FH001

FC Self-Employed Agricultural Work 农业自雇

[INTRO: Now we will ask you some questions about your work on household agricultural work in the past year 引语：现在我们将向您询问你过去一年为自家干的农活。]

FC022_W4 Which type of agricultural work are you engaged in? e.g. growing crops, growing fruits and raising animals. 请问您主要从事哪种类型的农业工作？例如：谷物种植、牲畜养殖、管理果树等 _____

FC023_W4 What kind of work do you mainly do? 请问您主要做什么工作？

1. Manual labor 简单体力劳动
2. Management 经营管理
3. Machine operation 机械操作
4. Others, pleas specify 其他，请注明 _____ **(FC023_W4_1)**

FC009 How many months did you work on [farming, forestry, animal production, and fishing] for your own household in the past year? 过去一年中，您有几个月为自家从事 [包括种地、管理果树、采集农林产品等种植业和林业经营活动] 或 [饲养牲畜等畜牧业经营活动] 或 [养殖水产品等渔业经营活动]？ _____ 0...12 months 月

FC010 How many days did you work for your own household per week on average during a normal month in the past year? 过去一年中，在您为自家从事 [种地、管理果树、采集农林产品等种植业及林业经营活动] 或 [饲养牲畜等畜牧业经营活动] 或 [养殖水产品等渔业经营活动] 的月份里，您一般每周干几天？ _____ 0...7 days 天

FC011 How many hours did you usually work for your own household during a normal work day in the past year? 过去一年中，在您为自家从事 [种地、管理果树、采集农林产品等种植业及林业经营活动] 或 [饲养牲畜等畜牧业经营活动] 或 [养殖水产品等渔业经营活动] 的日子里，您一般每天要干几个小时？ _____ 0...24 Hours 小时

[Soft Check: FC011 > 16 is unreasonable]

FC012 Where is your workplace most of the time? 大多数时间里，您干农活的地点是？ [preload sampling community ID]

1. Same as the permanent address 目前居住地的同个村/社区：[加载现居住地 BB001_W3]
2. Other village/community in the permanent address's county/city/district 居住地 BB001_W3 所在县/市/区的其他村/社区
   _____ **(FC012_1)** township 乡/镇/街道
   _____ **(FC012_2)** village/community 村/社区
3. Other 其他：
   _____ **(FC012_3)** province/city/county/district 省/市/县
   _____ **(FC012_4)** township/village/community 县/乡/镇/街道/村/社区

[IWER: Fill in with "others" if unknown to R 访员注意：如果记不清详细的地址名称，请在相应输入框中填入"其他"]

FC012_W3 How satisfied are you with your job? 请问您对干自家农活这份工作满意吗?

1. Completely satisfied 极其满意
2. Very satisfied 非常满意
3. Some what satisfied 比较满意
4. Not very satisfied 不太满意
5. Not at all satisfied 一点也不满意

FC013 How many days of work did you miss in the past year due to health problems? 过去一年您有多少天因为健康原因干不了农活? _____ 0...366 days 天
[IWER: Mark 0 if R didn't miss any work days 访员注意：如果没有，请注明"0"]

FC024_W4 Do you employ other people in your household agricultural work? How many people are employed? 请问您的农活雇人吗？一般雇了多少人? _____ 0...9999 人

PROCEDURE 程序:

- If no other job 若无其他工作 (XF5 = 1), Skip to FM_BEGINNING_W4 BRANCHPOINT 跳至 FM_BEGINNING_W4 BRANCHPOINT
- If employed 受雇工作 (XF11 = 1), Skip to FD001 跳至 FD001
- If nonfarm self-employed 非农自雇及无偿家庭帮工 (XF11 = 2), Skip to FH001 跳至 FH001

FD Employed 受雇

[INTRO: Now we will ask you some information about that wage earning job. We need to confirm whether you are a dispatched worker by a company or some individual 引语：现在我们将向您询问刚才说到的那份主要的挣工资工作。首先，我们要确定一下您是不是派遣职工，或受雇于个人]

FD001 Do you receive wages from your current workplace, or from a dispatch/contract company, or some individual? 您的工资是（非个体）工作单位发放、还是要通过某个派遣单位拿、还是向个人领取?

1. Place of work 工作单位
2. Labor dispatch company 劳务派遣单位
3. Individual 个人

[IWER: "Workplace" is where labor activities take place. "Dispatch company" is where workers establish work contracts. "Individual" refers to a boss of small business or a farm household 访员注意："工作单位"是指受访者从事劳动的（非个体）单位。"派遣单位"是指劳动者签订合同，或者人事所在的单位，比如劳务输出公司。"个人"指诸如包工头、个体老板、农户等情形]

[CAPI: For dispatched/contract workers (FD001 = 2), note: the next few questions pertain to the situation at your current workplace, but not to the company that dispatched/contracted you out; for those hired by individuals (FD001 = 3), note: the next few questions pertain to the situation at your current workplace, but not to the individual that paid you wages 对派遣工人

(FD001 = 2)，请提示：下面几个关于单位类型、名称和行业的问题是询问您为之工作的单位（或个人），而不是派遣单位。对个体雇员 (FD001 = 3)，请提示：如果您不是给发工资的人（包工头）工作，下面所有关于单位类型、名称和行业的问题是询问您为之工作的单位（或个人），不是给您发工资的人]

FD002 What is the type of your workplace/employer? 您的工作单位/雇主属于哪种类型？（请访员读出所有选项）

1. Government 政府部门
2. Public institution 事业单位
3. NGO 非营利机构，比如社团、协会、学会等
4. Firm 企业
5. Individual firm 个体户
6. Farmer 农户
7. Individual household 居民户
8. Other 其他，请说明 _____ **(FD002_1)**

FD003 What is the name of your workplace/employer? Please state specifically the name of your company or institution. 您的工作单位/雇主是什么名称？（请标明详细的单位名称）
Name of the workplace 工作单位名称：_____ **(FD003_1)**（例 1：北京大学餐饮服务中心；例 2：北京华信服装服饰有限责任公司）
Name of the department 所在部门名称：_____ **(FD003_2)**（例 1：农园餐厅；例 2：纺纱生产车间）
[IWER: Write down the name of the household head if R works for a family 访员注意：如果雇主是家庭，工作单位请写户主的姓名]

PROCEDURE 程序:

- If REIW R's employer in the last IW not missing 上轮受雇单位名称未缺失 (XZF14 ≠ null), Skip to FD003_W4_1 跳至 FD003_W4_1
- If REIW R was employed and the employer in the last IW is missing 上轮受雇且工作单位名称缺失 (XZF11 = 1 & XZF14 = null), Skip to FD003_W4_2 跳至 FD003_W4_2
- Otherwise NEWIW 及 REIW 上轮非受雇 Skip to FD004 跳至 FD004

FD003_W4_1 For the interviewer, check whether the names of the workplace at the last wave and this wave are the same. 访员请自行比对本轮单位名称 **[加载变量 FD003_1]** 与上轮单位名称 **[加载变量XZF14]**，两者是否系同一单位？同类的零散受雇工作视为同一单位

1. Yes 是 → Skip to FD004 跳至 FD004
2. No 否

FD003_W4_2 [If XZF14 ≠ null: in the last interview [ZIWTime] you told us the name of your workplace is XZF14, is your current workplace same as the one in the last interview [ZIWTime]? 若 XZF14 ≠ null → 上一次访问时您曾告诉我们上一轮 [加载上一轮调查时间 ZIWTime] 您的工作单位是 [加载变量XZF14]，] 您的现工作单位和 [加载上一轮调查时间 ZIWTime] 时的工作单位，是一样的吗？

1. Yes 是 → The interview determines whether the name of R's employer should be modified in FD003_1 访员自行判断是否修改本轮单位名称记录 FD003_1，then Skip to FD004 然后跳至 FD004
2. No 否

FD003_W4_3 What is the reason for your change to the current workplace/employer? 请问您的主要工作换成了现单位/雇主，主要原因是什么？

1. Involuntary switch: business failure (including decrease in working hours), lay-off, bankruptcy, and etc. 非自愿，原单位不景气（包括工时减少，变成非主要工作）、裁员、停业、倒闭等
2. For higher income 经济原因，收入和福利更高
3. Health reasons 自身健康，工作强度更适合自己的身体条件
4. Voluntary switch: personal interest, relaxation, better relationship with current colleagues. 因除经济、强度外的工作因素作出的自愿选择，更适合自己的兴趣、心情更轻松、同事关系更融洽等
5. Family reasons: moving, children's education 住所原因，搬迁或对配偶、父母、子女就读学校等有利
6. Retirement 到退休年龄办理退休，离开原单位
7. Others, please specify 其他，请说明 _____ **(FD003_W4_3_1)**

FD004 Where is your workplace located? Where is your workplace located? 您工作单位/雇主的地点是？(preload sampling community ID)

1. Same as the permanent address 目前居住地的同个村/社区：[加载现居住地 BB001_W3]
2. Other village/community in the permanent address's county/city/district 居住地 BB001_W3 所在县/市/区的其他村/社区
   _____ **(FD004_1)** township 乡/镇/街道
   _____ **(FD004_2)** village/community 村/社区
3. Other 其他：
   _____ **(FD004_3)** province/city/county/district 省/市/县
   _____ **(FD004_4)** township/village/community 县/乡/镇/街道/村/社区
4. Abroad 国外

[IWER: Fill in with "others" if unknown to R 访员注意：如果记不清详细的地址名称，请在相应输入框中填入"其他"]

PROCEDURE 程序：

If R is working for an individual farmer or resident household (FD002 = 6, 7), Skip to FD011 如果受访者为农户、居民户工作（FD002 = 6, 7），跳至 FD011

FD005 What industry does you workplace belong to—that is, what does your workplace make or do? 您的工作单位/雇主主要是做什么的，也就是，单位/雇主制造什么产品或者从事什么活动？

_____ （例 1：为校内提供餐饮服务；例 2：制造纱质布料）

[IWER: Type of business 访员注意：产业或行业类型。注意：回答中请不要用逗号]

PROCEDURE 程序：
- If employed by government 如果受访者是政府雇员（FD002 = 1），跳至 FD006
- If employed by a public institution 如果受访者是事业单位（FD002 = 2），跳至 FD009
- If employed by a firm 如果受访者是企业员工（FD002 = 4），跳至 FD010
- Otherwise 如果受访者是为其他单位工作（非营利机构、个体户、其他雇主）（FD002 = 3, 5, 8），跳至 FD011

FD006 Are you a civil servant? 您是公务员吗?
1. Yes 是
2. No 否

FD007 Are you a formal employee of an establishment? 您是正式编制内的员工吗?
1. Yes 是
2. No 否

PROCEDURE 程序:
Skip to FD011 跳至 FD011

FD009 Are you a formal employee of an establishment? 您是正式编制内的员工吗?
1. Yes 是
2. No 否

PROCEDURE 程序:
Skip to FD011 跳至 FD011

FD010 What is the ownership type of your workplace/employer? 您单位/雇主的所有制类型是?
1. 100% State owned firm 国有企业
2. State-controlled firm 国有控股企业
3. 100% Collective-owned firm 集体所有制企业
4. Collective-controlled firm 集体控股企业
5. 100% Private firm 私营/个体
6. Private-controlled firm 私人控股企业
7. 100% foreign-owned 外商独资
8. Joint venture 中外合资
9. Other joint-ownership 其他联营企业
10. Other 其他，请注明 _____ **(FD010_1)**

FD011 When did you start working for this employer? 您什么时候开始在这个单位/雇主工作的?
如果之间曾经中断在此单位/雇主的工作，请告诉我们您这一次在此单位工作是从什么时候开始的? _____ 1900...2018 **(FD011_1)** Year 年 _____ 0...12 **(FD011_2)** Month 月
[IWER: Mark a year using four digits. Record a month using the calendar number. For example, January as “1” instead of “01” and December as “12”. If R does not recall the month, fill in with “0” 访员注意: 用 4 位数表示年，按照实际的月份填写月。例：1 月写作“1”，而不是“01”，12 月写作“12”。如果记不住月份，请填入“0”]

[Soft Check: Consistency of employment start date. Make Sure that the individual was at least a minimum age when he/she started working for this employer (e.g., prompt to check if under 16). Specifically (FD011_1+FD011_2/12)-(CV009_a+CV009_b/12)<16 prompts a soft check]

FD012 What sort of work do you do? 您自己具体从事什么工作? _____ **(FD012_1)**（例 1：餐厅面点厨师；例 2：生产线绕线工人）Do you have any professional certificate? 是否有相关的职业资格证书? _____ **(FD012_2)**（例 1：有高级中式烹调师证；例 2：没有职业资格证书）
[IWER: Ask about the specific work that R does 访员注意：请详细填写具体工作内容。注意回答中请不要用逗号]

FD013 What is your current position? 这份工作中您担任的职务是什么?
1. Clerk/worker 普通职工（无职务）
2. Team Leader 组长或股长
3. Section Chief 科长
4. Director of a division 处长
5. Director-General of a bureau and above 局长及以上
6. Village Leader 村干部
7. Township Leader 乡镇干部
8. Division manager 单位部门经理
9. Overall/General manager 单位总经理
10. Other, 其他，请注明 _____ **(FD013_1)**

FD014 What is your current professional/technical level? 您有专业/技术职称吗? 如果有，您目前的专业/技术职称是什么?
1. Technician 技术员
2. Primary level 初级职称
3. Intermediate level 中级职称
4. Advanced level 高级职称
5. No professional/technical level 无职称

[IWER: A professional/technical level is referred to an occupation rank evaluated professionally 访员注意：“专业/技术职称”指专业技术人员的专业技术与学识水平和工作能力的等级称号]

FD015 Are you in a position to supervise others? 您是否管理别人?
1. Yes 是
2. No 否 → Skip to FD020 跳至 FD020

FD016 How many people are there under your supervision? 您管着多少人?
1. 1~5 people 1~5 人
2. 6~10 people 6~10 人
3. 11~15 people 11~15 人
4. 16~30 people 16~30 人

5. 31~99 people 31~99 人
6. More than 100 people 超过 100 人

[CAPI: For dispatched worker (FD001 = 2), note: the questions on labor contract and social insurances are related to your dispatch company. For those hired by individuals (FD001 = 3), note: the questions on labor contract and social insurances are related to the person who paid you wages. 对派遣职工 (FD001 = 2)，请提示：下面关于劳动合同和社会保险的问题是关于您派遣单位的情况。对个体雇员 (FD001 = 3)，请提示：下面关于劳动合同和社会保险的问题和给您发工资的人（包工头）有关]

FD020 Did you receive a labor contract (or employment contract) in written form from your current workplace (or labor dispatch company)? 您与单位/雇主签了书面的劳动合同吗?
1. Yes 是
2. No 否 → Skip to FD024 跳至 FD024

FD021 What is the agreed period of employment (labor contract period)? 您的劳动合同的期限是多长?
1. Defined period 固定期限 _____ 0...100 (**FD021_1**) Years 年 _____ 0...11 (**FD021_2**) months 月
   [IWER: If R does not recall the months, please fill in with “0” 访员注意：如果记不住多少个月，请填“0”]
2. Not defined 无固定期限 → Skip to FD024 跳至 FD024
3. Same as the term of the project 以完成一定工作任务为期限的劳动合同

FD022 Has the current employment contract ever been renewed? 现在的劳动合同是续签过的吗?
1. Yes 是
2. No 否 → Skip to FD024 跳至 FD024

FD023 How many times has the contract been renewed? 您的劳动合同是第几次续签? _____ 1...50 times 次

FD024 How long do you expect to work at your current workplace? 您预期还会在这工作（单位）干多久?
1. Less than one year 不到 1 年
2. One to two years 1-2 年
3. Two to three years 2-3 年 → Skip FD025 跳过 FD025
4. More than three years 超过 3 年 → Skip FD025 跳过 FD025

FD025 Why do you expect so? 你为什么这么觉得呢?
1. Because the pre-defined contract period will soon expire 因为之前签的合同快到期了
2. Because the verbally agreed contract period will soon expire (although there’s no written contract) 尽管没有书面合同，但因为之前约定的时间快到了

3. Because I expect no contract renewal will be agreed upon 因为估计雇主会让我辞职
4. Because the current job/project will be completed 因为目前的工作/项目即将结束
5. Because the person I am substituting/replacing will return 因为我顶替的那个人要回来了
6. Because I can only work during certain seasons 因为我只能在某些时间段工作
7. Because I want to look for a job that better suits my job aptitude, abilities, and preferences 因为我想找一份更适合我的工作
8. Because I will reach the retirement age as set by regulations/practice 因为我快到退休年龄了
9. Because of family care responsibilities, poor health, etc. 因为家庭或健康方面的原因
10. Other 其他

FD029 Except for national/public holidays, how many days of paid vacation do you have this year at your current workplace? 除国家法定节假日以外，今年您可以享受几天的带薪休假? ______ 0...366 days 天
[IWER: Mark 0 if there is no paid vacation 访员注意：如果没有带薪休假，请标明"0"]
[Soft Check for a reasonable range: If FD029 > 30, prompt for verification]

FD030 How many days of work did you miss at this current job in the past year due to health problems? 在过去的一年中, 您由于健康原因, 在这个单位/雇主请假多少天? ______ 0...366 days 天
[IWER: Mark 0 if the R didn't miss work days 访员注意：如果没有不在职的情况，请标明"0"]

PROCEDURE 程序:
If FD030 = 0, skip FD031 如果 FD030 = 0，跳过 FD031

FD031 In these days, how many are fully paid? 在这些天里, 有多少天是不扣工资或奖金的? ______ 0...366 days 天
[Soft Check for reasonable range: If FD031 > FD030, prompt for verification]

FG003_W4 Does your employer provide pension insurance, health insurance, unemployment insurance, worker's injury insurance, maternity insurance, and housing provident fund? (Select all that apply) 您的雇主是否提供社会保险和住房公积金? 也就是五险一金，包括养老保险、医疗保险、失业保险、工伤保险、生育保险和住房公积金，或三险一金，包括养老保险、医疗保险、失业保险和住房公积金?（可多选）

1. Pension insurance 养老保险
2. Health insurance 医疗保险
3. Unemployment insurance 失业保险
4. Worker's injury insurance 工伤保险
5. Maternity insurance 生育保险
6. Housing provident fund 住房公积金
7. None 没有 → Skip to FD034_W4 跳至 FD034_W4

FG010_W4 On what income base is the contribution to the above-mentioned social insurance determined? 请问您每月缴纳社会保险费和住房公积金（五险一金）时是根据多少工资（缴费基数）计算的?

1. ______ Yuan/month 元/月 **(FG010_W4_1)**
2. 60% of local average income 当地职工月平均工资的 60%（即缴费基数下限）

997. Don't know 不知道

FD034_W4 Does your employer purchase supplementary health insurance for you? 请问雇主单位给您购买了补充性商业医疗保险吗?

1. Yes 有
2. No 没有

[IWER: Supplementary health insurance is for out-patient use, which is different from the accident insurance 访员注意: 补充性商业医疗保险主要用以报销门诊，须与下面的意外伤害险区分开来。]

FD032_W3 Does your employer purchase workplace accident insurance for you? 请问雇主单位给您买了人身意外伤害保险了吗?

1. Yes 有
2. No 没有 → Skip to FE001 跳至 FE001

FD033_W3 How much does the insurance pay at the most if you encounter a severe accident at your workplace (e.g. death) ? 如果您发生了意外伤害，最高能拿到多少钱的赔偿? ______ Yuan 元

FE Questions About Labor Supply 劳动力供给

[CAPI: For dispatched/contract workers (FD001 = 2), note for FE001: the next few questions on labor supply pertain to the situation at your current workplace, but not to the company that dispatched/contracted you out; for those hired by individuals (FD001 = 3), note for FE001: the next few questions on labor supply pertain to the situation at your current workplace, but not to the individual that paid you wages 对派遣工人 (FD001 = 2)，对 FE001 提示：下面几个关于劳动力供给的问题是指您工作单位或雇主的情况，而不是派遣单位。对个体雇员 (FD001 = 3)，对 FE001 提示：如果您不是给发工资的人（包工头）工作，下面几个关于劳动力供给的问题是是询问您为之工作的单位（或个人），不是给您发工资的人]

FE001 Including paid vacations and sick leave fully paid, how many months did you work in the past year? 过去一年中，这份工作您一共干了几个月？带工资的假期和不扣工资的病假都算作工作时间。______ 1...12 months 月

[IWER: Working month is at least "1". Each month needs to be considered if R ever worked 访员注意：这里考虑自然月，至少填一。如果当月工作了一天以上，就视为当月在工作]

FE002 How many days a week did you work on average in the past year? 过去一年中，这份工作您一般每周干几天？ _____ 0...7 days 天
[IWER: If R's working days are not regular, working days in a week can be imputed by total working days in a year divided by working months and then dividied by four 访员注意：对于工作时间不规律的受访者，每周工作天数可以用过去一年总工作天数除以上题的月份数再除以一月的周数 4]

FE003 How many hours did you work per day on average in the past year, excluding meal breaks but including any paid or unpaid overtime? 过去一年中，这份工作您一般每天干多少个小时？工作时间不包括午休时间，但包括加班时间。这里不管是否有报酬，都算做工作时间 _____ 0...24 Hours 小时
[Soft Check: Verify if number of hours per day is unreasonable, e.g., FE003 > 16]

FF Questions About Wages 受雇工作的工资问题

[CAPI: For dispatched/contract workers (FD001 = 2), note for FF001 - FG002: the next few questions on wages are related to your dispatch company; for those hired by individuals (FD001 = 3), note for FF001 - FG002: the next few questions on wages are related to the person who paid you wages. Benefits from the workplace should not be included 对派遣工人 (FD001 = 2), 对 FF001 - FG002 均提示：下面几个问题是关于您从派遣单位和工作单位获得工资和福利的情况。在询问工资时，请您除奖金外不要考虑单位的福利。对个体雇员 (FD001 = 3)，对 FF001 - FG002 均提示：下面几个问题是关于您从派遣单位和工作单位获得工资和福利的情况。如果您不是给发工资的人（包工头）工作，在询问工资时，请您除奖金外不要考虑单位的福利]

FF001 How is your wage paid mainly? Is it regularly paid, contract-based, performance-based, or other? If it is regularly paid, please tell me how often you receive your wages. Do you have a yearly contract, monthly, weekly, daily, or hourly? Please select one. 您的工资主要是怎么支付的？是定期支付、按项目支付、按绩效支付还是其他方式？如果是定期支付，请回答您是多久领一次工资？您是否按年、月、周、日还是小时领取工资？请选择一个

1. Yearly wages 年薪
2. Monthly wages 月薪 → Skip to FF004_W4 跳至 FF004_W4
3. Weekly wages 周薪 → Skip to FF006_W4 跳至 FF006_W4
4. Daily wages 日薪 → Skip to FF008 跳至 FF008
5. Hourly wages 小时工资 → Skip to FF010 跳至 FF010
6. Contract-based 按项目 → Skip to FF012_W4 跳至 FF012_W4
7. Performance-based 按绩效 → Skip to FF012_W4 跳至 FF012_W4
8. Other 其他，请注明 _____ **(FF001_W4_1)** → Skip to FF012_W4 跳至 FF012_W4

FF002_W4 What is your salary including bonuses in the past year? 把奖金等各种收入都算在内，您过去一年从单位/雇主拿到多少钱？ _____ **(FF002_W4_a)** Yuan 元，Bonuses 其中，奖金有多少？ _____ **(FF002_W4_b)** Yuan 元，这里的奖金请包括工作中获得的小费、红包、和礼品。如果单位拖欠工资奖金，请告诉我们您应得的工资和奖金

[Soft Check: Prompt for clarification if under a low threshold, e.g. under 1200 Yuan annual. Specifically, prompt to clarify if FF002_W4_a$<$ 1200.]
[Hard Check: FF002_W4_a ≥ FF002_W4_b]

FF002_W4_bracket [IWER: If R is unwilling to answer or does not remember, ask unfolding bracket questions here 访员注意：如果受访者不愿回答或者忘记了或者填写 0，在此处分级展开提问] What is your salary from that workplace in the past year, including all bonuses? 把奖金等各种收入都算在内，您过去一年从单位拿到多少钱？10,000 /30,000 /50,000 /100,000 /200,000 Yuan 元

FF002_W4_1 Are personal income tax, social insurance contributions, housing fund contributions and other fees already deducted from this salary? 这些工资奖金收入中，有没有扣除个人所得税、各类保险、住房公积金或其他杂费？

1. Yes 有
2. No 没有

997. Don't know 不知道

FF002_W4_2 What is the total amount of these personal income tax, social insurance contributions, housing fund contributions and other fees? 这些收入中被扣除的，或是对应于这些收入应缴付的个人所得税、各类保险、住房公积金或其他杂费一共是多少元？ ______ **(FF002_W4_2_1)** Yuan 元

PROCEDURE 程序：
Skip to FF014 跳至 FF014

FF004_W4 What is your salary including bonuses last month? 把奖金等各种收入都算在内，您上个月从单位/雇主拿到多少钱？ ______ **(FF004_W4_a)** Yuan 元，Bonuses 其中，奖金有多少？ ______ **(FF004_W4_b)** Yuan 元，这里的奖金请包括工作中获得的小费、红包、和礼品。如果单位拖欠工资奖金，请告诉我们您应得的工资和奖金
[Soft Check: Prompt for clarification if under a low threshold, e.g. under 100 Yuan per month. Specifically, prompt to clarify if FF004_W4_a $<$ 100]
[Hard Check: FF004_W4_a ≥ FF004_W4_b]

FF004_W4_bracket [IWER: If R is unwilling to answer or does not remember, ask unfolding bracket questions here 访员注意：如果受访者不愿回答或者忘记了或者填写 0，在此处分级展开提问] What is your monthly salary from that workplace, including all bonuses? 把奖金等各种收入都算在内的总额，及奖金总额，分别是多少？500 /1,000 /2,500 /5,000 /10,000 Yuan 元

FF004_W4_1 Are personal income tax, social insurance contributions, housing fund contributions and other fees already deducted from this salary? 这些工资奖金收入中，有没有扣除个人所得税、各类保险、住房公积金或其他杂费？

1. Yes 有

2. No 没有
997. Don’t know 不知道

FF004_W4_2 What is the total amount of these personal income tax, social insurance contributions, housing fund contributions and other fees? 这些收入中被扣除的,或是对应于这些收入应缴付的个人所得税、各类保险、住房公积金或其他杂费一共是多少元? _____**(FF004_W4_2_1)** Yuan 元

**PROCEDURE** 程序:
Skip to FF014 跳至 FF014

FF006_W4 What is your salary including bonuses last week? 把奖金等各种收入都算在内，您上周从单位/雇主拿到多少钱? _____ Yuan 元，这里的奖金请包括工作中获得的小费、红包、和礼品。如果单位拖欠工资奖金，请告诉我们您应得的工资和奖金
[Soft Check: Prompt for clarification if under a low threshold, e.g. under 25 Yuan per week. Specifically, prompt to clarify if FF006_W4 < 25]

FF006_W4_bracket [IWER: If R is unwilling to answer or does not remember, ask unfolding bracket questions here 访员注意：如果受访者不愿回答或者忘记了或者填写 0，在此处分级展开提问] 把奖金等各种收入都算在内，您上周从单位拿到多少钱? 20 /50 /100 /200 /500 Yuan 元

**PROCEDURE** 程序:
Skip to FF012_W4 跳至 FF012_W4

FF008 In general, what is your daily wage? 您一般每天挣多少钱? _____ Yuan 元
[Soft Check: Prompt for clarification if under a low threshold, e.g. under 5 Yuan per day. Specifically, prompt to clarify if FF008 < 5]

FF009 [IWER: If R is unwilling to answer or does not remember, ask unfolding bracket questions here 访员注意：如果受访者不愿回答或者忘记了或者填写 0，在此处分级展开提问] 20 /50 /100 /200 /500 Yuan 元

**PROCEDURE** 程序:
Skip to FF012_W4 跳至 FF012_W4

FF010 What is your hourly wage? 您每小时的工资是多少? _____ Yuan 元
[Soft Check: Prompt for clarification if under a low threshold, e.g. under 1 Yuan per hour. Specifically, prompt to clarify if FF010 < 1]

FF010_bracket [IWER: If R is unwilling to answer or does not remember, ask unfolding bracket questions here 访员注意：如果受访者不愿回答或者忘记了或者填写 0，在此处分级展开提问] 10 /30 /50 /100 /200 Yuan 元

FF012_W4 How much were you paid including bonuses last month from this job? 把奖金等各种收入都算在内, 从这份工作您上个月拿到多少钱? _____ (**FF012_W4_a**) Yuan 元，Bonuses 其中，奖金有多少? _____ (**FF012_W4_b**) Yuan 元，这里的奖金请包括工作中获得的小费、红包、和礼品。如果单位拖欠工资奖金，请告诉我们您应得的工资和奖金
[Soft Check: Prompt for clarification if under a low threshold, e.g. under 100 Yuan per month. Specifically, prompt to clarify if FF012_W4 $<$ 100]
[Hard Check: FF012_W4_a $\geq$ FF012_W4_b]

FF012_W4_bracket [IWER: If R is unwilling to answer or does not remember, ask unfolding bracket questions here 访员注意：如果受访者不愿回答或者忘记了或者填写 0，在此处分级展开提问] 把奖金等各种收入都算在内，您上个月从单位拿到多少钱? 500 /1,000 /2,500 /5,000 /10,000 Yuan 元

FF012_W4_1 Are personal income tax, social insurance contributions, housing fund contributions and other fees already deducted from this salary? 这些工资奖金收入中，有没有扣除个人所得税、各类保险、住房公积金或其他杂费?

1. Yes 有
2. No 没有

997. Don’t know 不知道

FF012_W4_2 What is the total amount of these personal income tax, social insurance contributions, housing fund contributions and other fees? 这些收入中被扣除的,或是对应于这些收入应缴付的个人所得税、各类保险、住房公积金或其他杂费一共是多少元? _____ (**FF002_W4_2_1**) Yuan 元

FF014 What is the amount of all your other bonuses (not paid regularly as your wages) received in the past year? 在扣除社会保险和个人所得税后，过去一年中，您从单位/雇主拿到的其他所有奖金一共有多少钱? 这里指的是不和薪酬一起按期支付的奖金，比如年终奖、过节费等。_____ Yuan 元
[Soft Check: Prompt on bonuses and monthly earnings if bonuses is more than five times monthly net income]

FG Fringe Benefits 单位福利

FG001 Please answer yes if any of the following benefits are provided by your current workplace/employer. (Select all that apply) 下面我们将列举一些福利。请对这份工作回答您单位/雇主的福利情况以及您是否享受以下福利? (可多选)

1. Free lunch 免费午餐
2. Free breakfast 免费早餐
3. Free dinner 免费晚餐
4. Meal allowance 餐费补贴
5. Transportation allowance 交通费补贴

6. Free housing 免费住宿
7. Housing allowance 住房补贴
8. Company car 单位配车
9. Company shuttle bus 单位班车
10. Other allowance, please specify 包括实物折合的其他补贴，请注明 _____ **(FG001_1)**
11. None 没有

PROCEDURE 程序:
For each checked answer of FG001, ask FG002 对上题的每一个答案，都分别提问 FG002

FG002 What is the monetary value of each allowance you receive per month? 每个月这种补助有多少，或者值多少钱? _____ Yuan 元

PROCEDURE 程序:
Skip to FJ_BEGINNING_W4 BRANCHPOINT 跳至 FJ_BEGINNING_W4 BRANCHPOINT

FH Non-Farm Self-Employed and Unpaid Work for Family Business 非农自雇和为家庭经营活动帮工

[INTRO: Now we will ask you some questions about your individual business or helper job 引语：现在我们将向您询问刚才说到的那份个体/私营/帮工的主要工作]

FH001 How many months did you work in the past year? 过去一年中，您工作了几个月? _____
0...12 months 月

FH002 How many days did you work per week on average in the past year? 过去一年中，您一般每周工作几天? _____ 0...7 days 天

FH003 How many hours did you work per day on average in the past year, excluding meal breaks but including any paid or unpaid overtime on a normal work month? 过去一年中，您一般每天工作多少个小时? 不包括午餐和其他休息时间，但是包括加班时间，不管是否有报酬 _____ 0...24 hours 小时
[Soft Check: Verify if number of hours per day is unreasonable, e.g., FH003 > 16]

FH004 How many days of work did you miss in the past year due to health problems? 过去一年中，您由于健康原因，有多少天没有干活? _____ 0...366 days 天
[IWER: Mark 0 if you didn't miss any work days 访员注意：如果没有，请注明“0”]

PROCEDURE 程序:
If R is a family business helper (XF7 = 3), Skip to FH012 如果受访者的工作是家庭帮工 (XF7 = 3)，跳至 FH012

FH005 What is the name of your company or workplace? 您的企业或者生意的名称是?（请标明详细的单位名称）_____ （例 1：北京华信服装服饰有限责任公司；例 2：丽丽服装店）
[IWER: If there is more than one company, ask about the main one. Mark 0 if there is no name 访员注意：如果在几个企业工作或者做了多项生意，问主要工作的企业或者主要从事的生意。如果没有名称，请标明“0”]

PROCEDURE 程序:
- If REIW R's business in the last IW not missing, 上轮个体/私营单位名称未缺失 (XZF15 ≠ null), Skip to FH005_W4_1 跳至 FH005_W4_1
- If REIW R was self-employed and the business in the last IW is missing 上轮个体/私营且单位名称缺失 (XZF7=2 & XZF15 = null), Skip to FH005_W4_2 跳至 FH005_W4_2
- Otherwise NEWIW 及 REIW 上轮非个体/私营 (else), Skip to FH006 跳至 FH006

FH005_W4_1 For the interviewer, check whether the names of the businesses at the last wave and this wave are the same. 访员请自行比对本轮生意名称 **[加载变量 FH005]** 与上轮生意名称 **[加载变量XZF15]**，两者是否系同一单位？同类的零散非农自雇工作视为同一单位
1. Yes 是 → Skip to FH006 跳至 FH006
2. No 否

FH005_W4_2 [If XZF15 ≠ null: in the last interview [ZIWTime] you told us the name of your business is XZF15, is your current business same as the one in the last interview [ZIWTime]? 若 XZF15 ≠ null → 上一次访问时您曾告诉我们上一轮 [加载上一轮调查时间 ZIWTime] 您的生意是 [加载变量XZF15]，] 您现在的生意和 [加载上一轮调查时间 ZIWTime] 时的生意，是一样的吗?
1. Yes 是 → The interview determines whether the name of R's business should be modified in FH005 访员自行判断是否修改本轮生意名称记录 FH005, then Skip to FH006 然后跳至 FH006
2. No 否

FH005_W4_3 What is the reason for the business change? 请问您（主要）生意发生变化的主要原因是什么?
1. For higher income 经济原因，新生意更赚钱、或更花时间等
2. Unanticipated business failure 非正常经营原因，原生意破产、倒闭等
3. External problems, e.g. government restrictions 外部原因，如原生意场所被拆除或受到限制、政府政策的限制
4. Health reasons 自身健康，工作强度更适合自己的身体条件
5. Personal interest 因除经济、强度外的因素作出的自愿选择，更适合自己的兴趣、心情更轻松等
6. Housing or location considerations 住所原因，搬迁或对配偶、父母、子女就读学校等有利
7. Others, please specify 其他，请说明 _____ **(FH005_W4_3_1)**

FH006 Where is your company or workplace located? 您的公司/生意的地址是?

1. Same as the permanent address 目前居住地的同个村/社区: [加载现居住地 BB001_W3]
2. Other village/community in the permanent address's county/city/district 居住地 BB001_W3 所在县/市/区的其他村/社区
   _____ (**FH006_1**) township 乡/镇/街道
   _____ (**FH006_2**) village/community 村/社区
3. Other 其他:
   _____ (**FH006_3**) province/city/county/district 省/市/县
   _____ (**FH006_4**) township/village/community 县/乡/镇/街道/村/社区
4. Abroad 国外

[IWER: Fill in with "others" if unknown to R 访员注意: 如果记不清详细的地址名称, 请在相应输入框中填入"其他"]

FH007 What industry does your business beong to? that is, what does your business do or make? 您主要做什么生意, 即生产什么产品或者从事什么经营活动? _____ (例 1: 制造纱质布料; 例 2: 销售服装)
[IWER: Type of business 产业或行业类型, 注意回答中请不要用逗号]

FH008 When did you start working at the current company or workplace? 您什么时候开始经营这个企业/这项生意? _____ 1900...2018 (**FH008_1**) Year 年 _____ 0...12 (**FH008_2**) Month 月
[IWER: Mark the year using four digits. Take down the month as its actual number. For example, write January as "1" not "01", December as "12". If do not remember month, fill in with "0" 访员注意: 用 4 位数表示年, 按照实际的月份填写月。例: 1 月写作"1", 而不是"01", 12 月写作"12"。如果记不住月份, 请填入'0']
[A check similar to the one for FD011. (FD011_1 + FD011_2 /12)- (CV009_a + CV009_b/12) < 16 or (FD011_1 + FD011_2 /12)- (CV009_a + CV009_b/12) < FB001_1 or FH008_1 < FB001_2]

FH009_W4 How many people are employed in your company/workplace? 请问您的公司/生意一般雇了多少人? _____ 0...9999 人

PROCEDURE 程序:
Skip to FJ_BEGINNING_W4 BRANCHPOINT 跳至 FJ_BEGINNING_W4 BRANCHPOINT

FH012 What is the name of company or workplace that you help without getting paid? 您无偿帮忙的企业或者生意的名称是? _____ (例 1: 北京华信服装服饰有限责任公司; 例 2: 丽丽服装店)
[IWER:Mark 0 if there is no name 访员注意: 如果没有名称, 请标明"0"]

PROCEDURE 程序:

- If REIW R's helping business in the last IW not missing 上轮无偿家庭帮工单位名称未缺失 (XZF16 ≠ null), Skip to FH012_W4_1 跳至 FH012_W4_1
- If REIW R help a family business without getting paid and the helping business in the last IW is missing 上轮无偿家庭帮工且单位名称缺失 (XZF7=3 & XZF16 = null), Skip to FH012_W4_2 跳至 FH012_W4_2
- Otherwise NEWIW 及 REIW 上轮非无偿家庭帮工, Skip to FH013 跳至 FH013

FH012_W4_1 For the interviewer, check whether the names of the businesses at the last wave and this wave are the same. 访员请自行比对本轮帮工单位名称 **[加载变量 FH012]** 与上轮帮工单位名称 **[加载变量 XZF16]**，两者是否系同一单位?

1. Yes 是 → Skip to FH013 跳至 FH013
2. No 否

FH012_W4_2 [If XZF16 ≠ null: in the last interview [ZIWTime] you told us the name of your helping business is XZF16, is your current helping business same as the one in the last interview [ZIWTime] 若 XZF16 ≠ null → 上一次访问时您曾告诉我们上一轮 [加载上一轮调查时间 ZIWTime] 您的帮工单位是 [加载变量XZF16]，] 您现在的帮工单位和 [加载上一轮调查时间 ZIWTime] 时的帮工单位，是一样的吗?

1. Yes 是 → The interview determines whether the name of R's employed should be modified in FH012 访员自行判断是否修改本轮单位名称记录 FH012, then Skip to FH013 然后跳至 FH013
2. No 否

FH012_W4_3 请问您（主要）帮工单位发生变化的主要原因是什么?

1. For higher income 经济原因，新帮工单位生意更好、或更花时间等
2. Unanticipated business failure 非正常经营原因，原帮工单位破产、倒闭等
3. External problems, e.g. government restrictions 外部原因，如原帮工单位场所被拆除或受到限制、政府政策的限制
4. Health reasons 自身健康，工作强度更适合自己的身体条件
5. Personal interest 因除经济、强度外的因素作出的自愿选择，更适合自己或家庭成员的兴趣、心情更轻松等
6. Housing or location considerations 住所原因，搬迁或对配偶、父母、子女就读学校等有利
7. Others, please specify 其他，请说明 _____ **(FH012_W4_3_1)**

FH013 Where is this company or workplace loacted? 这个公司/生意的地址是?

1. Same as the permanent address 目前居住地的同个村/社区: [加载现居住地 BB001_W3]
2. Other village/community in the permanent address's county/city/district 居住地 BB001_W3 所在县/市/区的其他村/社区
   _____ **(FH013_1)** township 乡/镇/街道
   _____ **(FH013_2)** village/community 村/社区

3. Other 其他:
   ______ (**FH013_3**) province/city/county/district 省/市/县
   ______ (**FH013_4**) township/village/community 县/乡/镇/街道/村/社区
4. Abroad 国外

[IWER: Fill in with “others” if unknown to R 访员注意：如果记不清详细的地址名称，请在相应输入框中填入“其他”]

FH014 What industry does you helping business belong to—that is, what does this company do or make? 他们主要做什么生意，即生产什么产品或者从事什么经营活动? ______ （例 1：制造纱质布料；例 2：销售服装）
[IWER: Type of business 产业或行业类型，注意回答中请不要用逗号]

FH015 What sort of work did you do? 您具体从事什么工作? ______ （例 1：会计；例 2：帮助店主销售服装）
[IWER: Ask the specific work 访员注意：请详细填写具体工作内容，注意回答中请不要用逗号]

FH018 When did you start working at the current company or workplace? 您什么时候开始在这个企业/生意帮忙的? ______ 1900...2018 (**FH018_1**) Year 年 ______ 0...12 (**FH018_2**) Month 月

FJ Side Job (Employed or Self-employed) 非主要职业（受雇或自雇）

FJ_BEGINNING_W4 BRANCHPOINT:
If R doesn't have a side job 如果受访者没有非主要工作 (FC019_W4_b = 2 | FC019_W4_c = 2 | FC019_W4_e = 2 | FC019_W4_f = 2)，Skip to FM_BEGINNING_W4 BRANCHPOINT 跳至 FM_BEGINNING_W4 BRANCHPOINT

FJ001_W4 How many jobs do you currently engage in, excluding your main job and household agricultral work? 除了刚才问到的主要工作（以及给自家干农活），您目前还从事其他几份工作? ______ 1...20 份
[IWER: We know R has a few jobs from the beginning of this section. These jobs may include agricultrual employed and non-agricultrual employed. If R asserts having no more side job at this point, check and correct FC008, FC001, FA002_W4, FC019_W4 访员注意：通过工作模块起始部分的回答，我们已知悉受访者从事多份工作，包括农业受雇和非农业工作。如果受访者此处声称并无其他非主要工作，请返回检查模块最开始的 FC008、FC001、FA002_W4、FC019_W4（若曾问及）等各题]

FJ002_W4 How many hours a week do you work on average at your side job(s), not considering your main job? 除了刚才问到的主要工作（以及给自家干农活），平均来说，过去一年中，您每周在其他工作上总共工作多少个小时? ______ 0...168 Hours per week 小时/周

FJ003_W4 What is the average monthly income or wage that you get from side job(s) other than your main job? 除了刚才问到的主要工作（以及给自家干农活），平均来说，过去一年中，您其他工作每月的总收入，也就是所有工资加奖金加福利，是? _____ Yuan per month 元/月

FJ003_bracket [IWER: If R is unwilling to answer or does not remember, ask unfolding bracket questions here 访员注意：如果受访者不愿回答，此处分级展开提问] 500 /1,000 /2,500 /5,000 /10,000 Yuan 元

PROCEDURE 程序:
Skip to FM_BEGINNING_W4 BRANCHPOINT 跳至 FM_BEGINNING_W4 BRANCHPOINT

FK Unemployment and Job Search Activities 失业，求职经历

FK002 Did you search for a new job during the past month? 过去一个月您是否找过工作?

1. Yes 是
2. No 否

FM054 Are you currently engaged in any pastime activitity and still get some incomes from doing it? 您目前是否从事小的消遣性的工作但是也有收入?

1. Yes 是
2. No 否 → Skip to FK_END_W4 BRANCHPOINT 跳至 FK_END_W4 BRANCHPOINT

[IWER: "Pastime work" refers to an activity not for the purpose of earnings, and also not for helping family business either 访员注意："消遣性工作"指不是以挣钱为目的的活动，以及不再为家庭经营活动帮工的工作]

FM055 What kind of pastime work are you engaged in? 您从事哪种消遣性的工作? _____
[IWER: Ask the specific work 访员注意：请详细填写具体工作内容，注意回答中请不要用逗号]

FM056 When did you start this work? 您从什么时候开始做这项工作的?_____ 1900...2018 (**FM056_1**) year 年 _____ 0...12 (**FM056_2**) month 月
[IWER: Mark the year using four digits. Take down the month as its actual number. For example, write January as "1" not "01", December as "12". If do not remember month, fill '0' 访员注意: 用 4 位数表示年，按照实际的月份填写月。例：1 月写作"1"，而不是"01"，12 月写作"12"。如果记不住月份，请填入"0"]

FM057 How many days per week do you usually work for your pastime job? 您一般每周做几天? An average of 平均每周 _____ 0...7 days per week 天

FM058 How many hours per week do you usually work at your pastime job? 您一般每周做几个小时? An average of 平均每周 _____ 0.00...168.00 hours per week 小时

FM059 What is your monthly income from the pastime work? 您每月能通过消遣性工作挣多少钱? _____ Yuan 元

[IWER: Mark 0 if there is no net income, and mark 999997 if running a deficit 访员注意：如果没有净收入填 0，如果每月亏损填 999997]

FM059_bracket [IWER: If R is unwilling to answer or does not remember, ask unfolding bracket questions here 访员注意：如果受访者不愿回答或者忘记了或者填写 0，在此处分级展开提问]

500 /1,000 /2,000 /3,500 /5,000 Yuan 元

FK_END_W4 BRANCHPOINT:
- If new R is working 新受访者且工作过 (XRTYPE = NEWIW & XF1 = 1)，Skip to FL001 跳至 FL001
- If old R ever worked 旧受访者且工作过 (XRTYPE = REIW & XF1 = 1)，Skip to FM_BEGINNING_W4 BRANCHPOINT 跳至 FM_BEGINNING_W4 BRANCHPOINT
- If R never works 从未工作 (XF1 = 2)，Skip to FN002_W4 跳至 FN002_W4

FL Last Job 最近一份工作

[INTRO：The next questions are about the last main job you had, which could be agricultural, earning a wage, running your own business or working for unpaid help for family business. It does not include doing your own housework or doing activities without pay, such as voluntary work. If you have more than one job, we are interested in the job at which you spent most of the time. We're also more interested in your situation near the termination of this job 引语：下面的问题是关于您最近一份工作的情况，可以是务农、挣工资工作、从事个体、私营经济活动、或者不拿工资为家庭经营活动帮工，但不包括家务劳动或者志愿者劳动等没有报酬的工作。如果您之前同时从事不止一份工作，我们想了解您的主要工作，即占用您时间最多的那份工作]

FL001 Were you engaged in paid work for someone else (including unpaid work for family business), self-employed business, or household agricultural work? 您最近一份工作是为别人工作，还是自己从事非农业个体或者私营经济活动，或者务农？为别人工作包括不拿工资为家庭经营活动帮工。

1. Employed 受雇，包括非农打工和农业打工等
2. Self-employed 非农自雇，例如从事非农个体或者非农私营经济活动等
3. Unpaid help for family business 不拿工资为非农家庭经营活动帮工
4. Self-employed agricultural 农业自雇，即自主进行的农业生产经营活动，包括为自家或自家生意种地、管理果树、采集农林产品、养鱼、打鱼、养牲畜以及去市场销售自家生产的农产品等；不包括农业打工

FL002 In which year and month did you start this job? 您从哪年哪月开始做这份工作的？ _____ 1900...2018 (**FL002_1**) Year 年 _____ 0...12 (**FL002_2**) Month 月

[IWER: Mark the year using four digits. Take down the month as its actual number. For example, write January as "1" not "01", December as "12". If do not remember month, fill in with "0" 访员注意: 用 4 位数表示年，按照实际的月份填写月。例：1 月写作 "1"，而不

是“01”, 12 月写作“12”。如果记不住月份，请填入“0”]
[Soft Check: Prompt for Verification/Clarification if the Respondent was Less than 16 at time of starting this job, e.g. Prompt if (FL002_1 + FL002_2 /12) - (CV009_a + CV009_b/12) < 16, Replace 16 in the above check with FB001. FL002_1 < FB001_2 or FL002_1 < (CV009_a + FB001_1)]

FL003 In which year and month did you stop this job? 您什么时候停止做这份工作的? ______ 1900...2018 **(FL003_1)** Year 年 ______ 0...12 **(FL003_2)** Month 月
[IWER: Mark the year using four digits. Take down the month as its actual number. For example, write January as “1” not “01”, December as “12”. If do not remember month, fill in with “0” 访员注意: 用 4 位数表示年，按照实际的月份填写月。例：1 月写作“1”，而不是“01”, 12 月写作“12”。如果记不住月份，请填入“0”]
[Soft Check: Prompt for Verification/clarification if End Date is Before the Start Date, e.g. FL003_1 < FL002_1]

FL004 Where was the job located? 工作地点在哪里?
1. Same as the permanent address 目前居住地的同个村/社区: [加载现居住地 BB001_W3]
2. Other village/community in the permanent address's county/city/district 居住地 BB001_W3 所在县/市/区的其他村/社区
   ______ **(FL004_1)** township 乡/镇/街道
   ______ **(FL004_2)** village/community 村/社区
3. Other 其他:
   ______ **(FL004_3)** province/city/county/district 省/市/县
   ______ **(FL004_4)** township/village/community 县/乡/镇/街道/村/社区
4. Abroad 国外

[IWER: Fill in with “others” if unknown to R 访员注意：如果记不清详细的地址名称，请在相应输入框中填入“其他”]

PROCEDURE 程序:
For agricultral work (FL001 = 4), Skip to FL004_W4_1 如果FL001 = 4，跳至 FL004_W4_1
For non-agricultural work (FL001 = 1, 2, 3), ask FL005 to FL008 如果是非农工作（FL001 = 1/2/3），询问 FL005 至 FL008

FL004_W4_1 Which type of agricultural work are you engaged in? e.g. growing crops, growing fruits and raising animals. 请问您主要从事哪种类型的农业工作? ______ 例如：谷物种植、牲畜养殖、管理果树等

FL004_W4_2 What kind of work do you mainly do? 请问您主要做什么工作?
1. Manual labor 简单体力劳动
2. Management 经营管理
3. Machine operation 机械操作
4. Others, pleas specify 其他，请注明 ______ **(FL004_W4_2_1)**

**PROCEDURE** 程序:<br>If FL001 = 4, Skip to FL020_W4 如果 FL001 = 4, 跳至 FL020_W4

FL005 What was the name of your workplace/employer? Please state specifically the name of your company or business. 您的工作单位或者雇主是什么名称?(请详细标明名称) Name of the workplace 工作单位名称: _____ **(FL005_1)** (例 1: 北京大学餐饮服务中心; 例 2: 北京华信服装服饰有限责任公司), Name of the department 所在部门名称: _____ **(FL005_2)** (例 1: 农园餐厅; 例 2: 纺纱生产车间)

FL006 What industry did the workplace belong to, that is, what did the workplace make or do? 该单位主要是做什么的, 也就是, 他们制造什么产品或者从事什么活动? _____ (例 1: 为校内提供餐饮服务; 例 2: 制造纱质布料)
[IWER: Type of business 访员注意: 产业或行业类型, 注意回答中请不要用逗号]

FL007 Is this workplace still in business? 这个单位还存在吗?
1. Yes 是
2. No 否

FL008 In general, how many hours per week did you work? 您当时一般每周工作几个小时? 做这份工作 _____ 0...168 Hours per week 小时/周
[Soft Check: Prompt for Verification if FL008 > 80]

**PROCEDURE** 程序:<br>• If FL001 = 1 Skip to FL009 最后一份工作是受雇 (FL001 = 1) 跳至 FL009<br>• If FL001 = 2 Skip to FL012_W4 最后一份工作是个体/私营 (FL001 = 2) 跳至 FL012_W4<br>• If FL001 = 3 Skip to FL020_W4 最后一份工作是帮工 (FL001 = 3) 跳至 FL020_W4

FL012_W4 In general, how many people did the business employ? 请问您的公司/生意正常时一般雇多少人? _____ 0...9999 人

**PROCEDURE** 程序:<br>Skip to FL020_W4 跳至 FL020_W4

FL009 What were your monthly wages, bonuses, and allowance from this job, before you stopped this job? 包括工资、奖金和补贴在内, 您停止做这份工作前, 平均每月总共挣多少钱? _____ Yuan 元
[IWER: Mark 0 if there is no net income, and mark 999997 if running a deficit 访员注意: 如果没有净收入请标注 0, 如果是亏损请标注 999997]
[Soft Check: Prompt for Verification if FL009 < 100]

FL009_bracket [IWER: If R is unwilling to answer or does not remember, ask unfolding bracket questions here 访员注意: 如果受访者不愿回答或者忘记了或者填写 0, 在此处分级展开提问]
500 /1,000 /2,500 /5,000 /10,000 Yuan 元

FL011 What was the yearly amount of other bonuses not paid regularly as wages? 您从单位拿到的其他所有奖金每年一共有多少钱？奖金指的是不和薪酬一起按期支付，比如年终奖等 _____ Yuan 元

FL011_bracket [IWER: If R is unwilling to answer or does not remember, ask unfolding bracket questions here 访员注意：如果受访者不愿回答或者忘记了或者填写 0，在此处分级展开提问]
500 /1,000 /2,500 /5,000 /10,000 Yuan 元

FL013 Were you a formal/contract/temporary employee or a hourly paid worker? 您是正式员工、合同制员工、临时工，还是小时工？

1. Formal employee 正式员工
2. Contract employee 合同制员工
3. Temporary employee 临时工
4. Hourly paid worker 小时工

FL014 Did you work for the government, public institution, firm, NGO, individual farmer or a resident household? 您的工作单位/雇主属于哪种类型？（请访员读出所有选项）

1. Government 政府部门
2. Public institution 事业单位 → Skip to FL017 跳至 FL017
3. NGO 非营利机构，例如社团、协会、学会等 → Skip to FL017 跳至 FL017
4. Firm 企业 Skip to → FL016 跳至 FL016
5. Individual firm 个体户 → Skip to FL017 跳至 FL017
6. Individual farmer 农户 → Skip to FL017 跳至 FL017
7. Individual household 居民户 → Skip to FL017 跳至 FL017
8. Other 其他，请注明 _____ **(FL014_1)** → Skip to FL017 跳至 FL017

FL015 Were you a civil servant? 您是公务员吗？

1. Yes 是
2. No 否

PROCEDURE 程序:
Skip to FL017 跳至 FL017

FL016 What was the ownership type of the business? 您的企业的所有制类型是？

1. 100% State owned firm 国有企业
2. State-controlled firm 国有控股企业
3. 100% Collective-owned firm 集体所有制企业
4. Collective-controlled firm 集体控股企业
5. 100% Private firm 私营/个体
6. Private-controlled firm 私人控股企业
7. 100% foreign-owned 外商独资
8. Joint venture 中外合资
9. Other joint-ownership 其他联营企业

10. Other, please specify 其他，请注明 _____ **(FL016_1)**

FL017 What sort of work did you do? 您自己具体从事什么工作? _____ **(FL017_1)**（例 1：餐厅面点厨师；例 2：生产线绕线工人）Do you have any professional certificate? 是否有相关的职业资格证书? _____ **(FL017_2)**（例 1：有高级中式烹调师证；例 2：没有职业资格证书）
[IWER: Ask about the specific work 访员注意：请详细填写具体工作内容，注意回答中请不要用逗号]

FL020_W4 Why did you stop working? 您为什么现在不工作了，而离开了当时这个工作单位？或者不继续您的生意或务农了，或者不再为家庭经营活动帮工了呢?

1. Forced to leave 被动原因失去最近一份工作，如被单位辞退、店面被拆、土地被征用等
2. Health reasons 自身健康状况
3. Caring for family members 照料家庭成员
4. Retirement 到了退休年龄而离开工作岗位
5. Others, please specify 其他，请说明 _____ **(FL020_W4_1)**

FL021 Upon leaving your last job, did you receive any severance package other than retirement allowance? (For example, lump-sum payment, condolence payment, etc.) 上一份工作结束时，除去退休工资，您是否还得到了其他补偿？如买断工龄，伤残津贴等。

1. Yes 是
2. No 否 → 跳至 FM_BEGINNING_W4 BRANCHPOINT

[IWER: The "lump-sum payment" refers to the onetime payment that state-owned companies offered to its workers upon terminating contract with them in the 1980s and 1990s, when the companies were going through privatization and laying off redundant employees. The specific amount of the payment would be bargained by both sides based on the previous position, wage level, etc. of the employee, as well as the situation of the company 访员注意："买断工龄"指改革开放初期我国一些国有企业在改革过程中安置富余人员的一种办法，即参照员工在企业的工作年限、工资水平、工作岗位等条件，结合企业的实际情况，经企业与员工双方协商，报有关部门批准，由企业一次性支付给员工一定数额的货币，从而解除企业和富余员工之间的劳动关系，把员工推向社会的一种形式]

FL022_W4 What is the severance pay? 您得到了多少补偿? _____ **(FL022_W4_1)** Yuan 元；The pay is based on what? 补偿原因/依据是? _____ **(FL022_W4_2)** If you received a lump-sum payment, what was the length of your service used for its calculation? 如果是因为买断工龄，请问是按照多少年工龄算的? _____ 0...120 **(FL022_W4_3)** Year 年

FL022_bracket [IWER: If R is unwilling to answer or does not remember, ask unfolding bracket questions here 访员注意：如果受访者不愿回答或者忘记了或者填写 0，在此处分级展开提问]
1000 /2000 /5000 /10,000 /20,000 Yuan 元

FM Retirement 退休与退职

FM_BEGINNING_W4 BRANCHPOINT:
If (XF5 = 2), Skip to FK000_W3_1 有除农业自雇以外其他工作 (XF5 = 2), 跳至 FK000_W3_1
If ((XF1 = 1 & FK002 = 1) | XF5 = 1), Skip to FK000_W3_2 如果受访者无工作且在找工作或仅农业自雇 ((XF1 = 1 & FK002 = 1) | XF5 = 1), 跳至 FK000_W3_2
If (XF1 = 1 & FK002 = 2), Skip to FM000_W4 BRANCHPOINT 如果受访者无工作且未在找工作 (XF1 = 1 & FK002 = 2), 跳至 FM000_W4 BRANCHPOINT

FK000_W3_1 How satisfied are you with your job? 请问您对（主要）工作满意吗?
1. Completely satisfied 极其满意
2. Very satisfied 非常满意
3. Some what satisfied 比较满意
4. Not very satisfied 不太满意
5. Not at all satisfied 一点也不满意

FK000_W3_2 At what age do you plan to stop working, i.e. stop earning incomes or working for family business without pay or engaging in any other work more serious than pasttime work? 您计划在多大年龄时停止工作，即停止从事一切以挣钱为目的的活动，也不再为家庭经营活动帮工，将来也不打算从事比消遣性工作更劳累的工作?______ 1...120 Years old 岁
[IWER: “pastime” refers to any work that is not mainly for earning incomes or working for family business, please ask for an approximation. “0” if plan to keep working as long as being physically capable 访员注意：“消遣性工作”指不是以挣钱为目的的活动，以及不再为家庭经营活动帮工的工作，请告知大概年龄。如果您计划只要健康允许，就一直工作，请标明“0”]

FM000_W4 BRANCHPOINT :
If (XRTYPE = NEWIW | XZF21 = 1), Skip to FB011_W4 新受访者 + 上轮未办理退休/退职/内退 (XRTYPE = NEWIW | XZF21 = 1) , 跳至 FB011_W4

FM000_W4 Last time you told us that [preload ZIWTime] you had already processed [preload one of the following types: normal retirement/ internal retirement/ receding]. Is this record correct? (Retirement refers to the pension-guaranteed retirement from government, public institutions, and enterprises, and also eligible by individuals from the informal sector who have contributed to the elemental social pension insurance. Having started to receive pensions from the Urban Residence Insurance, the New Agricultural Insurance, and the Insurance for Urban and Rural Residents is not considered as retirement) 上一次访问时您曾告诉我们，在 [加载上一轮调查时间 ZIWTime] 您当时已经办理了 [退休 XZF17 = 1][内退 XZF18 = 1][退职 XZF19 = 1]，请问我们的记录是对的吗?（退休是指从政府部门、事业单位、和企业单位退休，以及参加了基本养老保险的灵活就业人员所办理的退休。开始领取城居保、新农保、城乡居民养老保险金，不算是办理了退休手续）
1. Yes 是

2. No 否 → [If having retired 若退休 XZF17 = 1, update variables 更新变量 XZF17 = 0, XZF21 = 1][If having processed internal retirement 若内退 XZF18 = 1, update variables 更新变量 XZF18 = 0, XZF20 = 0, XZF21 = 1][If having processed receding 若退职 XZF19 = 1, update variables 更新变量 XZF19 = 0, XZF21 = 1]

FM001_W4_1 BRANCHPOINT :

1. Update missing information of old R's retirement 旧受访者退休信息缺失补询
   - If in the last wave XZF17 = 1, check XZF25_1 XZF25_5 XZF25_6; if XZF25_1 = 1, ask FM004, if XZF25_5 = 1, ask FM014, if XZF25_6 = 1, ask FM016. INTRO: You informed us in the last wave that you have completed the retirement procedures, and we would like to confirm some answers. 如果旧受访者上一轮调查时已经办理了 [正式/提前] 退休（XZF17 = 1），依次检查 XZF25_1 XZF25_5 XZF25_6，如果 XZF25_1 = 1，则询问 FM004，如果 XZF25_5 = 1，则询问 FM014，如果 XZF25_6 = 1，则询问 FM016。询问引语：您上轮告知我们您已经办理了退休手续，有些问题我们希望再确认一下
   - If in the last wave XZF18 = 1, check XZF25_1, XZF25_9, XZF25_10, XZF25_11; if XZF25_1 = 1, ask FM004, if XZF25_9 = 1, ask FM025, if XZF25_10 = 1, ask FM027, if XZF25_11 = 1, ask FM028. INTRO: You informed us in the last wave that you have completed the internal retirement procedures, and we would like to confirm some answers. 如果旧受访者上一轮调查时已经办理了内退（XZF18 = 1），依次检查 XZF25_1, XZF25_9，XZF25_10，XZF25_11，如果 XZF25_1 = 1，则询问 FM004，如果 XZF25_9 = 1，则询问 FM025，如果 XZF25_10 = 1，则询问 FM027，如果 XZF25_11 = 1，则询问 FM028。询问引语：您上轮告知我们您已经办理了内退手续，有些问题我们希望再确认一下
   - If in the last wave XZF19 = 1, check XZF25_1, XZF25_2, XZF25_3; if XZF25_1 = 1, ask FM004, if XZF25_2 = 1, ask FM005, if XZF25_3 = 1, ask FM007. INTRO: You informed us in the last wave that you have completed the receding process, and we would like to confirm some answers 如果旧受访者上一轮调查时已经办理了退职（XZF19 = 1），依次检查 XZF25_1，XZF25_2，XZF25_3，如果 XZF25_1 = 1，则询问 FM004，如果 XZF25_2 = 1，则询问 FM005，如果 XZF25_3 = 1，则询问 FM007。询问引语：您上轮告知我们您已经办理了退职手续，有些问题我们希望再确认一下
2. PROCEDURE 跳转
   - If (XZF20 = 1), Skip to FM037_W2 上轮内退未退休 (XZF20 = 1)，跳至 FM037_W2
   - If (XZF21 = 1), Skip to FB011_W4 上轮未办理退休/退职/内退 (XZF21 = 1)，跳至 FB011_W4
   - Others 其他，Skip to FN002_W4 跳至 FN002_W4

FB011_W4 Have you completed retirement procedures（including early retirement）or internal retirement（Note: Retirement refers to the pension-guaranteed retirement from government, public institutions, and enterprises, and also eligible by individuals from the informal sector who have contributed to the elemental social pension insurance. Having started to receive pensions from the Urban Residence Insurance, the New Agricultural Insurance, and the Insurance for Urban and Rural Residents is not considered as retirement.）? 下面是关于您退休手续的一些问题。您是否已经办理了退休手续，包括提前退休，或

内退？提醒您一下，退休是指从政府部门、事业单位、和企业单位退休，以及参加了基本养老保险的灵活就业人员所办理的退休。开始领取城居保、新农保、城乡居民养老保险金，不算是办理了退休手续。

1. Yes 是 → Skip to FM001 跳至 FM001
2. No 否

FB012 Have you completed the receding procedures? 您是否办理了退职手续?

1. Yes 是
2. No 否 → Skip to FN002_W4 跳至 FN002_W4

[IWER: “receding” refers to the case where the employee is not eligible to retire in terms of age or length of work years, but has lost the ability to work due to illness or disabilities and hence needs to withdraw from his/her previous position. According to the law the employee shall receive certain amount of compensation in this scenario 访员注意：“退职”指职工因病残完全丧失劳动能力，但在年龄、工龄或个人缴费年限方面又不具备退休条件的，经医院证明并经劳动鉴定委员会确认、组织批准后退出生产或工作岗位，并按国家有关规定给予一定的物质帮助和被补偿，进行休养]

FM001 Which of the following is the employer/office that processed your [preload: retirement /receding] 给您办理 [退休/退职] 手续的单位是下面的哪一个?

[IWER: If R has no employer (no answer to FD003 or FL005), please choose “(3) None of the above” 访员注意：如果此受访者没有工作单位 (FD003 缺失 & FL005 缺失)，请选择“(3) 以上都不是”，并继续]

1. Current employer 现在的工作单位：[加载 FD003] → Skip to FM005 跳至 FM005
2. Last employer 最近一份工作的单位 [对新受访者，加载FL005，对旧受访者，加载变量 XZF14] → Skip to FM005 跳至 FM005
3. None of the above 以上都不是

FM002 What is the name of the employer that processed your [preload: retirement /receding]? 给您办理 [退休/退职] 手续的单位的名称是什么? ______

FM003 What was the type of your employer [preload: retirement /receding]? 您办理 [退休/退职] 的单位是哪种类型的?

1. Government 政府部门
2. Public institution 事业单位
3. NGO 非营利机构，如社团、协会、学会等
4. Firm 企业
5. Individual firm 个体户
6. Farmer 农户
7. Individual household 居民户
8. Other 其他

FM004 Where is this employer located? 您办理 [退休/退职] 的单位的地址是?

1. Same as the permanent address 目前居住地的同个村/社区：[加载现居住地 BB001_W3]

2. Other village/community in the permanent address's county/city/district 居住地 BB001_W3 所在县/市/区的其他村/社区
   ______ **(FM004_1)** township 乡/镇/街道
   ______ **(FM004_2)** village/community 村/社区
3. Other 其他:
   ______ **(FM004_3)** province/city/county/district 省/市/县
   ______ **(FM004_4)** township/village/community 县/乡/镇/街道/村/社区
4. Abroad 国外

[IWER: Fill in with "others" if unknown to R 访员注意：如果记不清详细的地址名称，请在相应输入框中填入"其他"]

PROCEDURE 程序:
If (FB011_W4 = 1), Skip to FM011 如果是退休受访者 (FB011_W4 = 1)，跳至 FM011

FM005 In what month and year did you recede from your position? 您是在哪年哪月办理的退职? ______ **(FM005_1)** 1900...2018 year 年 ______ **(FM005_2)** 0...12 month 月
[IWER: Mark a year using four digits. Record a month using the calendar number. For example, January as "1" instead of "01" and December as "12". If R does not recall the month, fill in with "0" 访员注意: 用 4 位数表示年，按照实际的月份填写月。例：1 月写作"1"，而不是"01"，12 月写作"12"。如果记不住月份，请填入'0']

FM006 What was the main reason you receded from your position? 您办退职的主要原因是?
1. Unable to work anymore due to health condition but not eligible to retire 身体原因不能继续工作，而又不符合退休条件
2. With length of eligible work years less than three, paused working more than a year due to illness or injuries unrelated to work 连续工龄不满三年，因病或非因工负伤而停止工作的时间满一年
3. Was diagnosed with a severe chronic disease which rendered work impossible within six months of employment 录用后在六个月以内，发现原来有严重慢性疾病，不能坚持工作的
4. Receded from position voluntarily 自愿退职
5. Reached retirement age, but length of eligible work years not long enough 到了退休年龄，但工龄不够
6. Other 其他

FM007 Before your receding, what was your monthly salary? 您退职前的总工资是每月多少钱?
______ Yuan/month 元/月 (including basic wages, bonuses, and etc. , 包括基本工资和奖金等)

FM007_bracket [IWER: If R is unwilling to answer or does not remember, ask unfolding bracket questions here 访员注意：如果受访者不愿回答或者忘记了或者填写 0，在此处分级展开提问]
500 /1,000 /2,000 /3,500 /5,000 Yuan 元

FM008 Upon receding, did you receive any compensation? 您是否得到了退职补偿?
1. Yes 是
2. No 否 → Skip to FM042 跳至 FM042

FM009 How much was the compensation? 您得到了多少补偿? ______ Yuan 元

FM009_bracket [IWER: If R is unwilling to answer or does not remember, ask unfolding bracket questions here 访员注意：如果受访者不愿回答或者忘记了或者填写 0，在此处分级展开提问]
500 /1,000 /2,000 /3,500 /5,000 Yuan 元

PROCEDURE 程序:
Skip to FM042 跳至 FM042

FM011 Was your retirement normal retirement, early retirement, or internal retirement initially, followed by normal retirement? 您办的是正常退休, 提前退休还是先内退然后办的正式退休或者将来再办正式退休?
1. Normal retirement 正常退休
2. Early retirement 提前退休
3. Internal retirement first, then normal retirement 先内退然后又正式退休
4. Internal retirement, but not yet normal retirement 内退但目前还没有办理正式退休

FM012 Did you retire as a worker or as a cadre? 您 [退休/内退] 时的身份是干部还是工人?
1. Worker 工人
2. Cadre 干部

PROCEDURE 程序:
If FM011 = 3 or FM011 = 4 , Skip to FM025 如果 FM011 = 3, 4， 跳至 FM025

FM014 In what month and year did you take [preload: normal/early] retirement? 您办理 [正式/提前] 退休手续是在哪年哪月?______ 1900...2018 (**FM014_1**) year 年 ______ 0...12 (**FM014_2**) month 月
[IWER: Mark a year using four digits. Record a month using the calendar number. For example, January as “1” instead of “01” and December as “12”. If R does not recall the month, fill in with “0” 访员注意: 用 4 位数表示年，按照实际的月份填写月。例：1 月写作“1”，而不是“01”, 12 月写作“12”。如果记不住月份，请填入‘0’]
[Soft Check: Prompt for Verification/Correction if Age of Early Retirement is Young, e.g. Prompt if ((FM014_1 + FM014_2 /12) - (CV009_a + CV009_b/12) < 45 & CV004 = 2) | ((FM014_1 + FM014_1/12) - (CV009_a + CV009_b/12) < 50 & CV004 = 1)]

PROCEDURE 程序:
Skip to FM016 if FM011 = 1 (normal retirement). 如果 FM011 = 1 正常退休，跳至 FM016

FM015 What was the main reason you processed early retirement? 您办提前退休的主要原因是?

1. I have work experience of 30 years, which is enough for early retirement. 我的工龄达到了 30 年，可以提前退休
2. My work had high risk and high intensity, for which I was eligible for early retirement. 单位是高风险、高强度的工作，允许提前退休
3. My employer was restructured/bankrupt, and hence I was offered early retirement 我的工作单位要破产、已经破产或者正在重组因此允许提前退休
4. Due to poor health 健康原因
5. Due to family reason 家庭原因
6. Other 其他

FM016 Before your retirement, including bonuses and subsidies, et. al, what was your total monthly salary? 您退休前的工资, 包括奖金和各种补贴每月多少钱? _____ Yuan/month 元/月

FM016_bracket [IWER: If R is unwilling to answer or does not remember, ask unfolding bracket questions here 访员注意：如果受访者不愿回答或者忘记了或者填写 0，在此处分级展开提问]
500 /1,000 /2,500 /5,000 /10,000 Yuan 元

**PROCEDURE** 程序:
Skip to FM036 跳至 FM036

FM025 In what month and year did you take internal retirement? 您是在哪年哪月办理的内退?
_____ 1900...2018 (**FM025_1**) year 年 _____ 0...12 (**FM025_2**) month 月
[IWER: Mark a year using four digits. Record a month using the calendar number. For example, January as "1" instead of "01" and December as "12". If R does not recall the month, fill in with "0" 访员注意: 用 4 位数表示年，按照实际的月份填写月。例：1 月写作"1"，而不是"01"，12 月写作"12"。如果记不住月份，请填入"0"]
[Soft Check: Prompt for Verification/Correction if Age of Retirement is Young, e.g. Prompt if (FM030_1 + FM030_2/12) - (CV009_a + CV009_b/12) < 45 & CV004 = 2) | ((FM030_1 + FM030_2/12) - (CV009_a + CV009_b/12) < 50 & CV004 = 1)]

FM026 What was the main reason you processed internal retirement? 您办理内退的主要原因是?

1. 5 years less than the legal retirement age 距法定退休年龄不足 5 年。
2. My employer was restructured/bankrupt 我的工作单位要破产、已经破产或者正在重组
3. Due to poor health 健康原因
4. Due to family reason 家庭原因
5. Other 其他

FM027 Before your retirement, what was your total monthly salary, including basic wage, bonus, etc.? 您内退前上班时的工资、奖金全部加起来是平均每月多少钱? _____ Yuan/month 元/月

FM027_bracket [IWER: If R is unwilling to answer or does not remember, ask unfolding bracket questions here 访员注意：如果受访者不愿回答或者忘记了或者填写 0，在此处分级展开提问]
500 /1,000 /2,000 /3,500 /5,000 Yuan 元

FM028 How much was the internal retirement wage (everything included) when you processed internal retirement? 您内退的时候，工资、奖金全部加起来每个月平均能拿到多少？
______ 元/月。

FM028_bracket [IWER: If R is unwilling to answer or does not remember, ask unfolding bracket questions here 访员注意：如果受访者不愿回答或者忘记了或者填写 0，在此处分级展开提问]
500 /1,000 /2,000 /3,500 /5,000 Yuan 元

**PROCEDURE** 程序:
If (FM011 = 4), Skip to FM037 如果受访者内退但未退休 (FM011 = 4)，跳至 FM037

FM030 In what year and month did you complete the normal retirement procedures 您在哪年哪月办理的正式退休手续？ ______ 1900...2018 (**FM030_1**) year 年 ______ 0...12 (**FM030_2**) month 月
[IWER: Mark a year using four digits. Record a month using the calendar number. For example, January as “1” instead of “01” and December as “12”. If R does not recall the month, fill in with “0” 访员注意: 用 4 位数表示年，按照实际的月份填写月。例：1 月写作“1”，而不是“01”, 12 月写作“12”。如果记不住月份，请填入‘0’]
[Soft Check: Prompt for Verification/Correction if Age of Retirement is Young, e.g. Prompt if ((FM030_1 + FM030_2 /12) - (CV009_a+CV009_b/12) < 45 & CV004 = 2) | ((FM030_1 + FM030_2/12) - (CV009_a + CV009_b/12) < 50 & CV004 = 1)]

FM036 How many years of eligible service or social insurance contributions did you have at the time of normal retirement? 您正式退休时计算了多少年工龄/社会保险缴费年限或视同缴费年限？ ______ 0.00...100.00 Years 年

**PROCEDURE** 程序:
Skip to FM042 跳至 FM042

FM037_W2 Have you completed normal retirement procedures? 您是否已经办理正式退休手续？
1. Yes 是 → Skip to FM030 跳至 FM030
2. No 否 → Skip to FN002_W4 跳至 FN002_W4

FM037 In what month and year are you going to process normal retirement? 您将会在哪年哪月办理正式退休手续？ ______ 2018...2050 (**FM037_1**) year 年 ______ 0...12 (**FM037_2**) month 月
[IWER: Mark a year using four digits. Record a month using the calendar number. For example, January as “1” instead of “01” and December as “12”. If R does not recall the month, fill in with “0” 访员注意: 用 4 位数表示年，按照实际的月份填写月。例：1 月写作“1”，而不是“01”, 12 月写作“12”。如果记不住月份，请填入“0”]

[Soft Check: Verify if Age of Respondent will be outside the legal retirement range, e.g., Prompt for verification if ((FM037_1 + FM037_2/12) - (CV009_a + CV009_b/12) < 50 | (FM037_1 + FM037_2/12) - (CV009_a + CV009_b/12) > 55) & CV004 = 2) | (FM037_1 + FM037_2 /12) - (CV009_a + CV009_b/12) < 55 | (FM037_1 + FM037_2/12) - (CV009_a + CV009_b/12) > 60) & CV004 = 1)]

FM040 How many years of eligible service or social insurance contributions will you have at the time of retirement? 您正式退休时预计将计算多少年工龄/社会保险缴费年限或视同缴费年限? ______ 0.00...100.00 Years 年

FM041 How many years of eligible service or social insurance contributions do you currently have? 您现在已经有多少年工龄了/社会保险缴费年限或视同缴费年限? ______ 0.00...100.00 Years 年
[Hard Check: FM040 > FM041]

FM042 Did you have a spouse when you processed [preload: normal retirement / early retirement / internal retirement / receding]? 您办理 [正式退休/提前退休/内退/退职（注：从 FM011 处加载。如果 FM037_W2 = 1，则此处加载“正式退休”。下同）] 手续时是否有配偶?
1. Yes 是
2. No 否

FM043 How was your health at the time of your [preload: normal retirement / early retirement / internal retirement / receding]? Was it excellent, very good, good, fair or poor? 您 [正式退休/提前退休/内退/退职] 时您的身体状况如何? 是极好、很好、好、一般还是不好?
1. Excellent 极好
2. Very good 很好
3. Good 好
4. Fair 一般
5. Poor 不好

PROCEDURE 程序:
Skip to FM047 if FM042 = 2 如果 FM042 = 2，跳至 FM047

FM044 Had your spouse already processed retirement when you processed [Preload: normal retirement / early retirement / internal retirement / receding]? 您办理 [正式退休/提前退休/内退/退职] 时配偶是否已经办理了退休手续?
1. Yes 是
2. No 否

FM045 What kind of economic activities was your spouse engaged in at the time of your [preload: retirement / receding]? 您 [退休/退职] 时配偶在从事哪种经济活动?
1. Employed 受雇于某公司或个人，领取工资
2. Ran own business 从事个体或者私营经济活动

3. Not working, but looking for a job 没有工作但是在找工作
4. Not working and not looking for a job，or only doing housework 没有工作也没有找工作, 或仅仅在家作家务
5. Farming 务农

FM046 How was your spouse's health at the time of your [preload: normal retirement / early retirement / internal retirement / receding]? Was it excellent, very good, good, fair or poor? 您 [正式退休/提前退休/内退/退职] 时您配偶的身体状况如何? 是极好、很好、好、一般还是不好?
1. Excellent 极好
2. Very good 很好
3. Good 好
4. Fair 一般
5. Poor 不好

FM047 Was your father alive at the time of your [preload: normal retirement / early retirement / internal retirement / receding]? 您 [正式退休/提前退休/内退/退职] 时，您父亲还健在吗?
1. Yes 是
2. No 否 → Skip to FM049 跳至 FM049

FM048 How about the health of your father at the time of your [preload: normal retirement / early retirement / internal retirement / receding]? Was it excellent, very good, good, fair or poor? 您 [正式退休/提前退休/内退/退职] 时您父亲的身体状况如何? 是极好、很好、好、一般还是不好?
1. Excellent 极好
2. Very good 很好
3. Good 好
4. Fair 一般
5. Poor 不好

FM049 Was your mother alive at the time of your [preload: normal retirement / early retirement / internal retirement / receding]? 您 [正式退休/提前退休/内退/退职] 时，您母亲还健在吗?
1. Yes 是
2. No 否 → Skip to FM051 跳至 FM051

FM050 How about the health of your mother at the time of your [preload: normal retirement / early retirement / internal retirement / receding]? Was it excellent, very good, good, fair or poor? 您 [正式退休/提前退休/内退/退职] 时您母亲的身体状况如何? 是极好、很好、好、一般还是不好?
1. Excellent 极好
2. Very good 很好
3. Good 好

4. Fair 一般
5. Poor 不好

FM051 How many grandchildren below age 6 did you have at the time of your [preload: normal retirement / early retirement / internal retirement / receding]? 您 [正式退休/提前退休/内退/退职] 时有几个六岁以下的孙子女或者外孙子女? ______ 0...50 persons 人
[IWER: If none, fill in with "0" 访员注意：如果没有请填"0"]

PROCEDURE 程序:
For a new R with a job (XRTYPE = NEIW & XF1 = 3), Skip to FM053 如果是新受访者且现有工作 (XRTYPE = NEIW & XF1 = 3)，跳至 FM053

FM052 Did you work after you processed [preload: regular retirement / early retirement / internal retirement / receding position], including agricultural, paid work, running your own business or working for family business, but excluding housework and volunteering. 在您办理了 [正式退休/提前退休/内退/退职] 之后，您是否工作过? 务农、挣工资工作、从事个体、私营经济活动或不拿工资为家庭经营活动帮工都算是工作，但不包括家务劳动、义务的志愿劳动。
1. Yes 是
2. No 否 → Skip to FN002_W4 跳至 FN002_W4

FM053 After you processed [preload: normal retirement / early retirement / internal retirement / receding], how long had you waited before you started to work again? 在您办理了 [正式退休/提前退休/内退/退职] 之后，您过了多长时间又开始工作的? ______ 0.00...100.00 Years 年

This page intentionally left blank

FN　Pension 养老金

[引语: 下面我们将问一些有关您的养老保险的问题，这些问题对于评估现有的养老政策以及制定将来的养老政策有着重要意义]

[F1：（1）“政府机关和事业单位退休金”指受访者从政府和事业单位领取的退休金。目前，我国大部分政府机构和事业单位，都还没有实行社会养老保险制度，仍实行退休养老制度。这一制度下，一般不需要个人在退休前缴纳养老保险金。（2）“职工基本养老保险”是指受访者参加社会基本养老保险制度，达到退休年龄并符合领取条件后，领取到社会养老保险金。（3）“补充养老保险（年金）”包括职业年金和企业年金；企业年金是指企业及其职工在依法参加基本养老保险的基础上，自愿建立的补充养老保险制度。现在一些机关和事业单位也开始建立了职业年金。（4）“城乡居民社会养老保险”指为推进城乡一体化，某些地区将城镇居民社会养老保险和新型农村社会养老保险统筹安排、合并实施，称为“城乡居民社会养老保障制度”。（5）“城镇居民养老保险”指通过建立城镇居民养老保险个人账户，实行个人缴费和财政补贴相结合的筹资模式，为目前未享受定期养老待遇或其他相关定期养老待遇的城镇居民提供的养老保险。（6）“新型农村社会养老保险”（新农保）采取个人缴费、集体补助和政府补贴相结合，其中中央财政将对地方进行补助，并且会直接补贴到农民头上。(7)“征地养老保险”，也叫做失地农民养老保险，是指因土地被政府征用而享有的一种养老保险。有一些不需要缴费到达年龄就可以领取，需要缴费的在新农保实行以后已经合并。(8)“人寿保险”是一种以人的生死为保险对象的保险。是被保险人在保险责任期内生存或死亡，由保险人根据契约规定给付保险金的一种保险。（9）“商业养老保险（人寿保险除外）”，是指受访者直接向商业保险公司投保，定期缴纳保险费，从合同约定年龄开始持续、定期地领取养老金的养老保险]

PART 1　Pension for Public Servants, Public Institution Employees, and Basic Pension for Enterprise Employees 政府机关、事业单位养老保险（退休金）及职工基本养老保险

FN002_W4 Do you currently receive, expect to receive, or contribute to the pension for public servants, or pension for public institution employees, or basic pension for enterprise employees? 您是否正在领取，预计将来可以领取或目前正在缴费政府机关退休金、事业单位退休金或是职工基本养老保险?

1. Yes 是，I currently participate in or receive benefits 我现在正在参加或领取
2. No 否，I do not participate in or receive benefit 我现在既没有参加也没有领取 → Skip to FN030_W4 跳至 FN030_W4

FN002_W4_a Which pension(s) do you currently receive, expect to receive benefits from, or contribute to? (Check all that apply) 您现在正在领取，预计将来可以领取或目前正在缴费的是以下哪种养老保险?（可多选）

1. Pension for public servants 政府机关或公务员的退休金
2. Pension for public institution employees 事业编制职工退休金
3. Basic pension for enterprise employees 职工基本养老保险
4. Unknown 不知道类别

PROCEDURE 程序:

For each choice chosen in FN002_W4_a ask FN002_W4_b - FN014_W4_c in a loop 对于 FN002_W4_a 选中的每个选项，循环询问 FN002_W4_b - FN014_W4_c

To generate [Name of Pension] in a loop, if FN002_W4_a = 1, 2, 3, then [Name of Pension] is the corresponding choice 循环中的 [保险名称] 生成：如果 FN002_W4_a = 1, 2, 3，则 [保险名称] 为对应的选项

if FN002_W4_a = 4, then [Name of Pension] is “pension for public servants, or public institution employees, or basic pension for enterprise employees” 如果 FN002_W4_a = 4，则 [保险名称] 为“政府机关、事业单位或职工基本养老保险”

FN002_W4_b Do you currently receive benefits from [Name of Pension]? 您现在正在领取 [保险名称] 吗?

1. Yes 是的，I do 我正在领取
2. No 不, I haven’t started to receive any benefits 我还没有开始领取 → Skip to FN007_W4 跳至 FN007_W4

FN003_W2 In what month and year did you start to receive benefits from [Name of Pension]? 您从哪年哪月开始领取 [保险名称]? _____ 1900...2018 (**FN003_W2_1**) Year 年 _____ 0...12 (**FN003_W2_2**) Month 月

[IWER: Year is in four digits. Month is in its actual value. For example, January is “1” not “01”, December is “12”. If R does not remember the month, fill “0” 访员注意：年份填写范围 [1900-2018]，用 4 位数表示年，按照实际的月份填写月，例：1 月写作“1”，而不是“01”，12 月写作“12”。如果记不住月份和日期，请填入“0”]

FN005_W2 How much pension benefits do you receive from [Name of Pension] each month (including subsidies?) 您现在每月从 [保险名称] 领多少退休金/养老金 (包括各种补贴)? _____ Yuan 元

[IWER: Use “-1” for unknown, ask unfolding bracket questions for “-1” or “0 Yuan” 访员注意：允许填写“1”表示不知道，填写“1”或回答 0 元时，将分级展开提问]

[Soft Check: Verify if monthly benefits are low or high, e.g., prompt if FN005_W2 < 200 yuan/month]

FN005_W2_bracket [IWER: If R is unwilling to answer, does not remember, or the answer is 0 Yuan, ask unfolding bracket questions 访员注意：如果受访者不愿回答，忘记了，或者回答 0 元时，在此处分级展开提问] 1500 /2,000 /2,500 /3,000 /4,000 yuan 元

FN006_W4 Where do you currently receive benefits from [Name of Pension]? 目前在哪领取 [保险名称]?

1. The same as current residence BB001_W3 和当前一般居住地 BB001_W3 一样
2. County/city/district of current residence BB001_W3 当前一般居住地 BB001_W3 所在县/市/区的 _____ (**FN006_W4_1**) other townships/subdistricts 其他乡/镇/街道，_____ (**FN006_W4_2**) villages/neighborhoods 村/社区

3. Other 其它: _____ **(FN006_W4_3)** province_city_county/city/district 省 _ 市 _ 县/市/区, _____ **(FN006_W4_4)** township/subdistrict/village/neighborhood 乡/镇/街道/村/社区

997. Unknown 不知道

PROCEDURE 程序:
Skip to FN014_W4_a 跳至 FN014_W4_a

FN007_W4 Where do you currently contribute to [Name of Pension]? 目前在哪里缴费 [保险名称]?

1. The same as current residence BB001_W3 和当前一般居住地 BB001_W3 一样
2. County/city/district of current residence BB001_W3 当前一般居住地 BB001_W3 所在县/市/区的 _____ **(FN007_W4_1)** other townships/subdistricts 其他乡/镇/街道, _____ **(FN007_W4_2)** villages/neighborhoods 村/社区
3. Other 其它: _____ **(FN007_W4_3)** province_city_county/city/district 省 _ 市 _ 县/市/区, _____ **(FN007_W4_4)** township/subdistrict/village/neighborhood 乡/镇/街道/村/社区

997. Unknown 不知道

FN008_W4 Do you participate this pension plan by yourself or through your company? 对于[保险名称], 您目前是自己上社保还是通过单位上社保?

1. By Myself 自己上
2. Through my employer 通过单位上 → Skip to FN012_W2 跳至 FN012_W2

FN104_W4 Do you receive 4050 subsidies? 您有 4050 补贴吗?

[IWER: The 4050 group refers to unemployed females over 40 years old, unemployed males over 50, and the severely disabled of the same age. Because of no work, they need government support. Thus, the government implements a series of social protection policies, including social insurance subsidies that target the 4050 group 访员注意: 4050 人员是指满 40 周岁的失业女性和满 50 周岁的失业男性, 以及同样年龄的重度残疾人。这些人由于没有工作, 更需要政府的照拂。因此国家对 4050 人员采取了一系列的保障政策, 其中就包括给予这部分人员社保补贴]

1. Yes 有
2. No 没有 → Skip to FN012_W2 跳至 FN012_W2

997. Unknown 不知道 → Skip to FN012_W2 跳至 FN012_W2

FN105_W4 In what month and year did you start to receive 4050 subsidies? 您从哪年哪月开始有 4050 补贴? _____ 1900...2018 **(FN105_W4_1)** Year 年 _____ 0...12 **(FN105_W4_2)** Month 月

[IWER: Year is in four digits. Month is in its actual value. For example, January is “1” not “01”, December is “12”. If R does not remember the month, fill “0” 访员注意: 用 4 位数表示年, 按照实际的月份填写月。例: 1 月写作 “1”, 而不是 “01”, 12 月写作 “12”。如果记不住月份, 请填入 “0”]

FN106_W4 What is the share or the amount of the pension subsidy? 养老保险补贴的比例是多少或补贴多少钱? _____ 0.00...100.00 (**FN106_W4_1**) percent 比例 Or 或 _____ (**FN106_W4_2**) Yuan per month 元/月
[CAPI：Allow unknown cases，denote as -1 编程注意：允许不知道的情况，如果不知道填-1]

FN107_W4 What is the share or the amount of the medical insurance subsidy? 医疗保险补贴的比例是多少或补贴多少钱? _____ 0.00...100.00 (**FN107_W4_1**) percent 比例 Or 或 _____ (**FN107_W4_2**) Yuan per month 元/月
[CAPI：Allow unknown cases, denote as -1 编程注意：允许不知道的情况，如果不知道填-1]

PROCEDURE 程序:
FN104_W4 - FN107_W4 appear once in the loop FN104_W4 - FN107_W4 在循环中只出现一次

FN012_W2 Do you or your employer need to contribute to [Name of Pension] from which you expect to receive benefits or you currently contribute to? 您预计将来可以领取或目前正在缴费的 [保险名称] 需要您个人或单位缴费吗?
1. Yes 需要
2. No 不需要 → Skip to FN017_W2 跳至 FN017_W2

PROCEDURE 程序:
If FN008_W4 = 1 and FN012_W2 = 1, then ask FN013_W2 如果 FN008_W4 = 1 并且 FN012_W2 = 1，询问 FN013_W2
If FN008_W4 = 2 and FN012_W2 = 1, then ask FN013_W2- FN014_W2_bracket 如果 FN008_W4 = 2 并且 FN012_W2 = 1，询问 FN013_W2 - FN014_W2_bracket

FN013_W2 For [Name of Pension], how much do you currently contribute by yourself? 对于[保险名称]，您个人现在缴纳的保险费是多少? _____ (**FN013_W2_1**) Yuan per month 元/月 Or 或_____ 0.00...100.00 (**FN013_W2_2**) percent of wage 工资的比例
[IWER: Use "-1" for unknown, ask unfolding bracket questions for "-1" or "0 Yuan" 访员注意：允许填写 "1" 表示不知道，填写 "1" 或回答 0 元时，将分级展开提问]

FN013_W2_bracket [IWER: If R is unwilling to answer or does not remember, ask unfolding bracket questions here 访员注意：如果受访者不愿回答，忘记了，在此处分级展开提问] 50 /200 /300 /500 /750 yuan 元

FN014_W2 For [Name of Pension], how much does your employer currently contribute? 对于[保险名称]，单位现在为您缴纳的保险费是多少? _____ (**FN014_W2_1**) Yuan per month 元/月 Or 或 _____ 0.00...100.00 (**FN014_W2_2**) percent of wage 工资的比例
[IWER: Use "-1" for unknown, ask unfolding bracket questions for "-1" or "0 Yuan" 访员注意：允许填写 "1" 表示不知道，填写 "1" 或回答 0 元时，将分级展开提问]

FN014_W2_bracket [IWER: If R is unwilling to answer or does not remember, ask unfolding bracket questions here 访员注意：如果受访者不愿回答，忘记了，在此处分级展开提问] 50 /100 /200 /400 /700 yuan 元

FN017_W2 For [Name of Pension], how many years of contribution history have you established so far in this province/city? [Whether or not working for the same employer, countable working history/social insurance contribution years or equivalent contribution years should be counted.] 对于 [保险名称]，到现在为止您在本省/市可以用来计算养老金或退休金的工龄/社会保险缴费年限或视同缴费年限一共有多少年？[不管雇主是否相同，只要能够累积计算工龄/社会保险缴费年限或视同缴费年限，都应算进去。] ______ 0.00...100.00 Years 年

FN017_W3 For [Name of Pension], how many years of contribution history have you established so far in other provinces/cities? [Whether or not working for the same employer, countable working history/social insurance contribution years or equivalent contribution years should be counted] 对于 [保险名称]，到现在为止您在其他省/市可以用来计算养老金或退休金的工龄/社会保险缴费年限或视同缴费年限一共有多少年？[不管雇主是否相同，只要能够累积计算工龄/社会保险缴费年限或视同缴费年限，都应算进去] ______ 0.00...100.00 Years 年

FN019_W4 Can you receive pension benefits from [Name of Pension] in the future? 您以后可以从 [保险名称] 中领取养老金吗？

1. Yes 是的，我可以
2. No 不，我不可以 → Skip to FN014_W4_a 跳至 FN014_W4_a

997. Unknown 不知道 → Skip to FN014_W4_a 跳至 FN014_W4_a

FN020_W4 At what age do you expect to receive benefits from [Name of Pension]? 您预期在多大年龄开始领 [保险名称]? ______ 45...120 Years old 岁

FN021_W2 How much do you expect to receive from [Name of Pension]? It can be in the percentage of your wage before retirement or the amount of monthly benefit 您预期从 [保险名称] 中领多少钱的养老金？可以是退休前工资的百分比，也可以是每月多少钱。______ （**FN021_W2_1**）Yuan per month 元/月 Or 或 ______ 0.00..100.00（**FN021_W2_2**）percent of wage before retirement of final pay % 退休前工资
[IWER: Use "-1" for unknown, ask unfolding bracket questions for "-1" or "0 Yuan" 访员注意：允许填写"1"表示不知道，填写"1"或回答 0 元时，将分级展开提问]

FN021_W2_bracket [IWER: If R is unwilling to answer, does not remember, or the answer is 0 Yuan, ask unfolding bracket questions 访员注意：如果受访者不愿回答，忘记了，或者回答 0 元时，在此处分级展开提问] 1,000 /1,500 /2,000 /3,000 /4,500 Yuan 元

FN014_W4_a When you enrolled in [Name of Pension], do you need to make up the contribution? 您在加入 [保险名称] 时，是不是需要补缴一笔钱？
[IWER: The make-up contribution to pension programs for public institutions and enterprises refers to the lump sum or multiple payments that R wants to pay in the following cases: R predicts insufficient contribution history at retirement and needs to make up the previous contributions, or R hopes to receive more benefits in the future 访员注意：参加企事业养老保险补缴费用是指受访者预计在退休时缴费年限不够领取养老金，需要补缴之前的费用；或者希望未来领取更多的养老金而一次性或分多次缴纳的一笔费用]

1. Yes 是
2. No 否 → Skip to FN030_W4 跳至 FN030_W4

FN014_W4_b For [Name of Pension], how much do you need to make up the contribution overall? 对于 [保险名称]，您总共需要补缴多少钱?
1. _____ (**FN014_W4_b_1**) Yuan 元
2. _____ (**FN014_W4_b_2**) 10,000 Yuan 元

[IWER: Use "-1" for unknown, ask unfolding bracket questions for "-1" or "0 Yuan" 访员注意：允许填写 "1" 表示不知道，填写 "1" 或回答 0 元时，将分级展开提问]

FN014_W4_b_bracket [IWER: If R is unwilling to answer, does not remember, or the answer is 0 Yuan, ask unfolding bracket questions 访员注意：如果受访者不愿回答，忘记了，或者回答 0 元时，在此处分级展开提问] 1,000/3,000 /5,000 /8,000 /10,000 Yuan 元

FN014_W4_c For [Name of Pension], when did you make up the contribution? 对于 [保险名称]，您是何时补缴的? _____ 1900...2018 (**FN014_W4_c_1**) Year 年 _____ 0...12 (**FN014_W4_c_2**) Month 月

[IWER: Year is in four digits. Month is in its actual value. For example, January is "1" not "01", December is "12". If R does not remember month, fill "0" 访员注意: 用 4 位数表示年，按照实际的月份填写月。例：1 月写作 "1"，而不是 "01"，12 月写作 "12"。如果记不住月份，请填入 "0"]

PART 2 Supplementary Pension Insurance (Annuity) 补充养老保险（年金）

FN030_W4 Do you currently receive, or expect to receive or contribute to supplementary pension insurance of government and public institutions (Annuity), or Enterprises (Enterprise annuity)? 您是否正在领取，或预计将来可以领取或正在缴费政府机关事业单位补充养老保险（年金）或者企业补充养老保险（企业年金）?
1. Yes 是，I currently participate in or receive pension benefits 我现在正在参加或领取
2. No 否，I currently do not participate in or receive pension benefits 我现在既没有参加也没有领取 → Skip to FN058_W4 跳至 FN058_W4

FN030_W4_a Which supplementary pension do you currently receive, or expect to receive or contribute to? 您现在正在领取，或预计将来可以领取或正在缴费的是以下哪种补充养老保险?
1. Supplementary Pension for public servants (occupational annuity for public servants) 政府机关或公务员的补充养老保险金（政府机关职工年金）
2. Supplementary Pension for public institution employees (occupational annuity for public institution employees) 事业编制职工补充养老保险金（事业单位职工年金）
3. supplementary pension for enterprise employees (enterprise annuity) 企业职工补充养老保险 (企业年金)
4. Unknown 不知道类型

PROCEDURE 程序:

For each choice chosen in FN030_W4_a, ask FN031_W4 - FN037_W2 对于 FN030_W4_a 选中的每个选项，询问 FN031_W4 - FN037_W2

To generate [Name of Pension]: If FN030_W4_a = 1, 2, 3, then [Name of Pension] is the corresponding choice, if FN030_W4_a = 4, then [Name of Pension] is "Supplementary Pension for public servants, public institution employees, or for enterprise employees" 询问中的 [保险名称] 生成：如果 FN030_W4_a = 1, 2, 3，则 [保险名称] 为对应的选项，如果 FN030_W4_a = 4，则 [保险名称] 为"政府机关，事业单位或者企业补充养老保险"

FN031_W4 Do you currently receive benefits from [Name of Pension]? 您现在正在领取 [保险名称] 吗?

1. Yes 是的，I do 我正在领取
2. No 不，I have not started to receive any benefits 我还没有开始领取

FN032_W4 Which type does your [Name of Pension] belong to? 您的 [保险名称] 属于哪种类型?
[IWER: DB Retirement Pension Plan: The pension benefit is pre-determined and the contribution is determined by the benefit accordingly 收益确定性退休金计划：预先确定员工领取的养老金水平，在此基础上确定缴费金额。DC Retirement Pension Plan: The contribution is pre-determined, and the pension benefit depends on the management of the pension funds 缴费确定性退休金计划：预先确定缴费的金额，员工领取的退休金根据养老基金管理情况而定]

1. Defined Benefit (DB) Retirement Pension 收益确定型退休金
2. Defined Contribution (DC) Retirement Pension 缴费确定型退休金

997. Unknown type 不知道类型

PROCEDURE 程序:

If FN031_W4 = 1, Skip to FN041_W2 如果 FN031_W4 = 1，跳至 FN041_W2

FN034_W4 For [Name of Pension], where do you currently contribute? 您的 [保险名称]，目前在哪里缴费?

1. The same as current residence BB001_W3 和当前一般居住地 BB001_W3 一样
2. County/city/district of current residence BB001_W3 当前一般居住地 BB001_W3 所在县/市/区的 _____ (**FN034_W4_1**) other townships/subdistricts 其他乡/镇/街道，_____ (**FN034_W4_2**) villages/neighborhoods 村/社区
3. Other 其它: _____ (**FN034_W4_3**) province_city_county/city/district 省 _ 市 _ 县/市/区，_____ (**FN034_W4_4**) township/subdistrict/village/neighborhood 乡/镇/街道/村/社区

997. Unknown 不知道

FN033_W2 For how many years have you enrolled in [Name of Pension]? [Including years working for different employers but enrolling in the same pension program] 您参加 [保险名称] 多少年了? [包括受雇不同雇主但享受同一计划的年度] _____ 0.00...100.00 Years 年

FN035_W4_a Does [Name of Pension] that you expect to receive benefit from or that you contribute to require contributions from you or your employer? 您预计将来可以领取或目前正在缴费的 [保险名称] 需要您个人或单位缴费吗?

1. Yes 需要
2. No 不需要 → Skip to FN038_W2 跳至 FN038_W2

FN035_W4_b For [Name of Pension], how much does your employer currently contribute? 对于这份 [保险名称]，单位现在为您缴纳的保险费是多少？ _____ (**FN035_W4_b_1**) Yuan per month 元/月 Or 或 _____ 0.00...100.00 (**FN035_W4_b_2**) percent of wage 工资的比例
[IWER: Use "-1" for unknown, ask unfolding bracket questions for "-1" or "0 Yuan" 访员注意：允许填写"1"表示不知道，填写"1"或回答 0 元时，将分级展开提问]

FN035_W4_b_bracket [IWER: If R is unwilling to answer, does not remember, or the answer is 0 Yuan, ask unfolding bracket questions 访员注意：如果受访者不愿回答，忘记了，或者回答 0 元时，在此处分级展开提问] 500 /1,000 /2,000 /3,500 /5,000 Yuan 元

FN035_W4_c For [Name of Pension], how much do you currently pay by yourself? 对于这份 [保险名称]，您个人现在缴纳的保险费是多少？ _____ (**FN035_W4_c_1**) Yuan per month 元/月 Or 或 _____ 0.00...100.00 (**FN035_W4_c_2**) percent of wage 工资的比例
[IWER: If R is unwilling to answer, does not remember, or the answer is 0 Yuan, ask unfolding bracket questions 访员注意：允许填写 "-1" 表示不知道，填写 "-1" 或回答 0 元时，将分级展开提问]

FN035_W4_c_bracket [IWER: If R is unwilling to answer or does not remember, ask unfolding bracket questions here 访员注意：如果受访者不愿回答，忘记了，或者回答 0 元时，在此处分级展开提问] 500 /1,000 /2,000 /3,500 /5,000 yuan 元

FN038_W2 What is the earliest age at which you could leave this employer and start to receive benefits from [Name of Pension]? 您最早可以在多大年纪时离开这家单位，并开始领取 [保险名称]? _____ 45...120 Years old 岁

FN039_W4 How much do you expect to receive from [Name of Pension]? It can be in the percentage of your wage before retirement or it can be the amount of monthly benefit 您预期从这份 [保险名称] 领多少钱的养老金？可以是退休前工资的百分比，也可以是每月多少钱 _____（**FN039_W4_1**）Yuan per month 元/月 Or 或 _____ 0.00..100.00（**FN039_W4_2**）of wage before retirement % 退休前工资
[IWER: Use "-1" for unknown, ask unfolding bracket questions for "-1" or "0 Yuan" 访员注意：允许填写"1"表示不知道，填写"1"或回答 0 元时，将分级展开提问]

FN039_W4_bracket [IWER: If R is unwilling to answer, does not remember, or the answer is 0 Yuan, ask unfolding bracket questions 访员注意：如果受访者不愿回答，忘记了，或者回答 0 元时，在此处分级展开提问] 500 /1,000 /2,000 /3,500 /5,000 Yuan 元

PROCEDURE 程序:

If FN032_W4 = 2, 997, Skip to FN037_W2 如果 FN032_W4 = 2, 997， 跳至 FN037_W2
If FN032_W4 = 1, Skip to FN058_W4 如果 FN032_W4 = 1， 跳至 FN058_W4

FN041_W2 In what month and year did you start to receive pension benefits from [Name of Pension]? 您是从哪年哪月开始领这份 [保险名称] 的? _____ 1900...2018 (**FN041_W2_1**) Year 年 _____ 0...12 (**FN041_W2_2**) Month 月
[IWER: Year is in four digits. Month is in its actual value. For example, January is "1" not "01", December is "12". If R does not remember the month, fill "0" 访员注意: 用 4 位数表示年，按照实际的月份填写月。例：1 月写作 "1"，而不是 "01"，12 月写作 "12"。如果记不住月份，请填入 "0"]

FN042_W2 How much is your benefit from [Name of Pension] each month? 您每月从这份 [保险名称] 领多少钱? _____ Yuan per month 元/月
[IWER: Use "-1" for unknown, ask unfolding bracket questions for "1" or "0 Yuan" 访员注意：允许填写 "1" 表示不知道，填写 "1" 或回答 0 元时，将分级展开提问]

FN042_W2_bracket [IWER: If R is unwilling to answer, does not remember, or the answer is 0 Yuan, ask unfolding bracket questions 访员注意：如果受访者不愿回答，忘记了，或者回答 0 元时，在此处分级展开提问] 500 /1,000 /2,000 /3,500 /5,000 Yuan 元

FN042_W4 Where do you receive benefits from [Name of Pension]? 您目前在哪里领取这份 [保险名称]?

1. The same as current residence BB001_W3 和当前一般居住地 BB001_W3 一样
2. County/city/district of current residence BB001_W3 当前一般居住地 BB001_W3 所在县/市/区的 _____ (**FN042_W4_1**) other townships/subdistricts 其他乡/镇/街道, _____ (**FN042_W4_2**)villages/neighborhoods 村/社区
3. Other 其它: _____ (**FN042_W4_3**) province_city_county/city/district 省 _ 市 _ 县/市/区, _____ (**FN042_W4_4**) township/subdistrict/village/neighborhood 乡/镇/街道/村/社区

997. Unknown 不知道

PROCEDURE 程序:
If FN032_W4 = 2, 997, ask FN037_W2 如果 FN032_W4 = 2, 997, 询问 FN037_W2
If FN032_W4 = 1, Skip to FN058_W4 如果 FN032_W4 = 1, 跳至 FN058_W4

FN037_W2 Have you ever checked your account balance of [Name of Pension]? 您有没有查看过您的 [保险名称] 的账户余额?

1. Yes 有, _____ (**FN037_W2_1**) Yuan 元, in 在 _____ 1900...2018 (**FN037_W2_2**) Year 年 ___0...12 (**FN037_W2_3**) Month 月
   [IWER: Year is in four digits. Month is in its actual value. For example, January is "1" not "01", December is "12". If R does not remember the month, fill "0" 访员注意: 用 4 位数表示年，按照实际的月份填写月。例：1 月写作 "1"，而不是 "01"，12 月写作 "12"。如果记不住月份，请填入 "0"]
2. No 没有

PART 3 Urban and Rural Resident Pension, New Rural Resident Pension and Urban Resident Pension 城乡居民养老保险、新型农村养老保险及城镇居民养老保险

FN058_W4 Do you currently receive, or expect to receive or contribute to Urban and Rural Resident Pension, New Rural Resident Pension, and Urban Resident Pension? 您是否正在领取，或预计将来可以领取或正在缴费以下居民养老保险，居民养老保险包括城乡居民养老保险、新型农村养老保险以及城镇居民养老保险?

1. Yes 是，I do 我现在正在参加或领取 → Skip to FN058_W4_a 跳至 FN058_W4_a
2. No 否，I do not participate or receive any benefit 我现在既没有参加也没有领取

FN069_W4 Are you eligible for Urban and Rural Resident Pension, New Rural Resident Pension, and Urban Resident Pension? In other words, your hukou-registered village or neighborhood implemented the three pension programs above, and you can participate if you are willing to. 您是否有资格领取，或预计将来可以领取或正在缴费以下居民养老保险，居民养老保险包括城乡居民养老保险、新型农村养老保险以及城镇居民养老保险，也就是说你户口所在地的村或社区开展了上述三种居民养老保险，并且如果你愿意就可以参加?

1. Yes 有资格 → Skip to FN057_W4_4 跳至 FN057_W4_4
2. No 没有资格

997. I do not know 不知道我有没有资格 → Skip to FN057_W4_4 跳至 FN057_W4_4

FN069_W4_a The reason for ineligibility 没有资格的原因

1. Not available in my Hukou registration area 我户口所在地没有开展
2. I have participated in government and public institution pension or basic pension for enterprise workers 我已经参加了政府机关事业单位养老保险或职工基本养老保险
3. Other 其他，please specify 请注明 ______ **(FN069_W4_a_1)**

**PROCEDURE** 程序:<br>Skip to FN103_W4 跳至 FN103_W4

FN057_W4_4 According to the age on your ID, have you reached the age requirement to receive the following pensions for residents? Pensions for residents include Urban and Rural Resident Pension, New Rural Resident Pension, and Urban Resident Pension 您现在身份证上的年龄已经到领取这几种居民养老保险的年龄了吗? 居民养老保险包括城乡居民养老保险、新型农村养老保险以及城镇居民养老保险

1. Yes 是
2. No 否 → Skip to FN057_W3_6 跳至 FN057_W3_6

FN057_W4_5 What is the reason that you do not receive any benefits? 您现在不领取的原因?

1. did not pay the insurance premium 我没有缴费
2. I am not in my hukou place 我不在户口所在地
3. Do not know where to receive 我不知道去哪里领

4. Poor health, cannot go and receive 我身体原因无法去领
5. Other 其他，please specify 请注明 _____ **(FN057_W4_5_1)**

PROCEDURE 程序:
Skip to FN103_W4 跳至 FN103_W4

FN057_W3_6 What is the reason that you do not pay the premium? 您现在没有缴纳的原因?
1. No money 我没有钱
2. I do not satisfy with the benefit, it is not worthwhile to participate 我对待遇水平不满意，缴费参加该保险是不划算的。
3. Low benefit level, does not make any differences to my life 待遇水平低，对我的生活没有什么意义
4. It is not convenient to apply and to pay 申请办理或缴费不方便
5. The design of the payments is not reasonable 缴费设计不合理
6. I do not have the local hukou 我没有本地户口
7. I have already enrolled in other pension plans, and cannot double enroll 我已经参加了其他社会养老保险项目，不能重复参加
8. Others 其他，please specify 请注明 _____ **(FN057_W3_6_1)**

PROCEDURE 程序:
Skip to FN103_W4 跳至 FN103_W4

FN058_W4_a Which pension program do you currently receive benefit from, expect to receive benefit from, or contribute to? 您现在正在领取，预计将来可以领取或目前正在缴费的是以下哪种养老保险?
1. Urban and Rural Resident Pension 城乡居民养老保险
2. New Rural Resident Pension 新型农村养老保险
3. Urban Resident Pension 城镇居民养老保险
4. I do not know the type 不知道种类

PROCEDURE 程序:
For each choice chosen in FN058_W4_a, ask FN058_W4_b - FN072_W4_b 对于 FN058_W4_a 选中的每个选项，询问 FN058_W4_b - FN072_W4_b
To generate [Name of Pension], if FN058_W4_a = 1, 2, 3, then [Name of Pension] is the corresponding choice 询问中的 [保险名称] 生成：如果 FN058_W4_a = 1, 2, 3，则 [保险名称] 为对应的选项
If FN058_W4_a = 4, then [Name of Pension] is "Urban and Rural Resident Pension, New Rural Resident Pension, and Urban Resident Pension" 如果 FN058_W4_a = 4，则 [保险名称] 为"城乡居民养老保险，新型农村养老保险或城镇居民养老保险"

FN058_W4_b Do you currently receive pension benefits from [Name of Pension]? 您现在正在领取 [保险名称] 吗?
1. Yes 是的，I do 我正在领取 → Skip to FN067_W2 跳至 FN067_W2

2. No 不，I have not started to recieve any benefits 我还没有开始领取

FN061_W4 For how many years have you contributed to or participated in other ways in [Name of Pension]? 请问您给这个 [保险名称] 缴费或以其它方式参加多少年了?_____ 0.00...100.00 Year 年

FN059_W2 Where did you enroll in [Name of Pension]? 您在哪儿参加 [保险名称] 的?
1. The same as current residence BB001_W3 和当前一般居住地 BB001_W3 一样
2. County/city/district of current residence BB001_W3 当前一般居住地 BB001_W3 所在县/市/区的 _____ **(FN059_W4_1)** other townships/subdistricts 其他乡/镇/街道，_____ **(FN059_W4_2)** villages/neighborhoods 村/社区
3. Other 其它: _____ **(FN059_W4_3)** province_city_county/city/district 省 _ 市 _ 县/市/区, _____ **(FN059_W4_4)** township/subdistrict/village/neighborhood 乡/镇/街道/村/社区

997. Unknown 不知道

FN062_W4 Does [Name of Pension] that you expect to receive benefit from or you currently contribute to require contributions? Contributions include individual contributions and government subsidies 您预计将来可以领取或目前正在缴费的 [保险名称] 需要缴费吗? 缴费包括个人缴费和政府补贴
1. Yes 是
2. No 否 → Skip to FN065_W2 跳至 FN065_W2

FN062_W4_a How much does the government subsidize your [Name of Pension]? 政府给您这份 [保险名称] 补贴多少?_____ **(FN062_W4_a_1)** Yuan per month 元/月 Or 或 _____ **(FN062_W4_a_2)** Yuan per year 元/年
[IWER: Use "-1" for unknown, ask unfolding bracket questions for "-1" or "0 Yuan" 访员注意：允许填写"1"表示不知道，填写"1"或回答 0 元时，将分级展开提问]

FN062_W4_a_bracket [IWER: If R is unwilling to answer or does not remember, ask unfolding bracket questions here 访员注意：如果受访者不愿回答，忘记了，在此处分级展开提问] 5 /10 /15 /20 /30 Yuan 元

FN062_W4_b For your [Name of Pension], how much is your current individual contribution? 对于您的 [保险名称]，您个人现在缴纳的保险费是多少? _____ **(FN062_W4_b_1)** Yuan per month 元/月 Or 或 _____ **(FN062_W4_b_2)** Yuan per year 元/年
[IWER: Use "-1" for unknown, ask unfolding bracket questions for "-1" or "0 Yuan" 访员注意：允许填写"1"表示不知道，填写"1"或回答 0 元时，将分级展开提问]

FN062_W4_b_bracket [IWER: If R is unwilling to answer, does not remember, or the answer is 0 Yuan, ask unfolding bracket questions 访员注意：如果受访者不愿回答，忘记了，或者回答 0 元时，在此处分级展开提问] 10 /50 /100 /200 /500 Yuan 元

FN065_W2 When do you expect to receive pension benefits from [Name of Pension] ? 您预期在多大年龄享用 [保险名称]? At age 在 _____ 45...120 (**FN065_W2_1**) years old 岁 or in 或在 _____ 0.00...100.00 (**FN065_W2_2**) years 年之后

[Soft Check: Prompt or Verify if FN065_w2_1 < 50 or FN065_w2_2 > 60]

FN066_W2 How much do you expect to receive from [Name of Pension]? 您预期从这份 [保险名称] 中自己能领多少钱?_____ (**FN066_W2_1**) Yuan per month 元/月 Or 或 _____ (**FN066_W2_2**) Yuan 元（Lump sum amount 一次结清）

[IWER: Use "-1" for unknown, ask unfolding bracket questions for "-1" or "0 Yuan" 访员注意：允许填写 "1" 表示不知道，填写 "1" 或回答 0 元时，将分级展开提问]

[Soft Check: Prompt or Verify if These Benefits are Low or High, e.g., FN066_w2_1 < 100 | FN066_w2_1 > 5000 per month.]

FN066_W2_bracket [IWER: If R is unwilling to answer, does not remember, or the answer is 0 Yuan, ask unfolding bracket questions 访员注意：如果受访者不愿回答，忘记了，或者回答 0 元时，在此处分级展开提问] 55 /60 /75 /100 /400 Yuan 元

FN063_W4 For those who currently contribute to or participate in other ways in pension programs for residents, have you ever checked your account balance of [Name of Pension]? 对于目前正在缴费或以其它方式参加居民养老保险的人，您有没有查看过您 [保险名称] 的账户余额?

1. Yes 有，_____ （**FN063_W4_1**）Yuan 元 in _____ 1900...2018 （**FN063_W4_2**）Year 年 _____ 0...12（**FN063_W4_3**）Month 月

   [IWER：Year is in four digits. Month is in its actual value. For example, January is "1" not "01", December is "12". If R does not remember the month, fill "0" 访员注意：用 4 位数表示年，按照实际的月份填写月。例：1 月写作 "1"，而不是 "01"，12 月写作 "12"。如果记不住月份，请填入 "0"]
2. No 没有

PROCEDURE 程序:

Skip to FN072_W3 跳至 FN072_W3

FN067_W2 In what month and year did you start to receive benefits from [Name of Pension]? 您是从哪年哪月开始领取 [保险名称]? _____ 1900...2018 (**FN067_W2_1**) Year 年 _____ 0...12 (**FN067_W2_2**) Month 月

[IWER：Year is in four digits. Month is in its actual value. For example, January is "1" not "01", December is "12". If R does not remember the month, fill "0" 访员注意：用 4 位数表示年，按照实际的月份填写月。例：1 月写作 "1"，而不是 "01"，12 月写作 "12"。如果记不住月份，请填入 "0"]

FN068_W2 How much do you currently receive from [Name of Pension] each month? 您现在每月从 [保险名称] 中领多少钱? _____ Yuan per month 元/月

[IWER: Use "-1" for unknown, ask unfolding bracket questions for "-1" or "0 Yuan" 访员注意：允许填写 "-1" 表示不知道，填写 "-1" 或回答 0 元时，将分级展开提问]

FN068_W2_bracket [IWER: If R is unwilling to answer or does not remember, ask unfolding bracket questions here 访员注意：如果受访者不愿回答，忘记了，或者回答 0 元时，在此处分级展开提问] 55 /60 /75 /85 /100 Yuan 元

FN070_W4 Where do you receive the pension from [Name of Pension]? 您目前在哪儿领取 [保险名称]?

1. The same as current residence BB001_W3 和当前一般居住地 BB001_W3 一样
2. County/city/district of current residence BB001_W3 当前一般居住地 BB001_W3 所在县/市/区的 _____ (**FN070_W4_1**) other townships/subdistricts 其他乡/镇/街道，_____ (**FN070_W4_2**) villages/neighborhoods 村/社区
3. Other 其它：_____ (**FN070_W4_3**) province_city_county/city/district 省 _ 市 _ 县/市/区, _____ (**FN070_W4_4**) township/subdistrict/village/neighborhood 乡/镇/街道/村/社区

997. Unknown 不知道

FN072_W3 Do you need to pay make-up contributions when you enroll in [Name of Pension]? 您在加入 [保险名称] 时，是不是需要补缴一笔钱?
[IWER: The make-up contribution for (new pension for rural residents) refers to the lump-sum payment or multiple payments that the R wants to pay in the following cases: R predicts a less-than-15 year contribution history by the age of 60, or has never participated and needs to make up previous contributions, or R hopes to receive more pensions in the future 访员注意：参加（新农保）补缴费用是指受访者预计在 60 岁时不够 15 年的缴费年限；或者此前一直没有参加，需要补缴之前的费用；或者希望未来领取更多的养老金而一次性或分多次缴纳的一笔费用]

1. Yes 是
2. No 否 → Skip to FN103_W4 跳至 FN103_W4

997. Unknown 不知道 → Skip to FN103_W4 跳至 FN103_W4
999. Refuse to answer 拒绝回答 → Skip to FN103_W4 跳至 FN103_W4

FN072_W3_1 For [Name of Pension], how much do you make up for? 对于 [保险名称]，您总共需要补缴多少钱?

1. _____ (**FN072_W3_1_1**) Yuan 元
2. 或 _____ (**FN072_W3_1_2**) 10,000Yuan 万元

[IWER: Use “-1” for unknown, ask unfolding bracket questions for “-1” or “0 Yuan” 访员注意：允许填写“1”表示不知道，填写“1”或回答 0 元时，将分级展开提问]

FN072_W3_1_bracket [IWER: If R is unwilling to answer, does not remember, or the answer is 0 Yuan, ask unfolding bracket questions 访员注意：如果受访者不愿回答，忘记了，或者回答 0 元时，在此处分级展开提问] 1,000/3,000 /5,000 /8,000 /10,000 Yuan 元

FN072_W4_b For [Name of Pension], when did you make up the contribution? 对于 [保险名称]，您什么时候补缴的? _____ 1900...2018 (**FN072_W4_b_1**) Year 年 ___ 0...12 (**FN072_W4_b_2**) Month 月
[IWER: Year is in four digits. Month is in its actual value. For example, January is “1” not

"01", December is "12". If R does not remember the month, fill "0" 访员注意：用 4 位数表示年，按照实际的月份填写月。例：1 月写作"1"，而不是"01"，12 月写作"12"。如果记不住月份，请填入"0"]

FN103_W4 Have you ever participated in the Rural Social Pension Insurance (old rural pension)? 您是否曾经加入过农村社会养老保险（老农保）?
[IWER: "Rural pension insurance" refers to the Rural Social Pension Insurance. At the end of 1992, the Ministry of Civil Affairs promulgated "Basic scheme of county-level rural social pension insurance" confirmed that the rural social pension was implemented at the county level 访员注意："农村养老保险"是指农村社会养老保险。1992 年民政部颁布了《县级农村社会养老保险基本方案》，确定以县为基本单位开展农村社会养老保险]

1. Yes 是
2. No 否

PART 4 Pension for Land-Expropriated Farmers 征地养老保险 (失地农民养老保险/被征地农民养老保险)

FN079_W2_3 Do you currently receive or expect to receive or contribute to the pension for land-expropriated farmers? 您是否正在领取或将来可以领取或目前正在缴费征地养老保险?

1. Currently enroll 参加了保险，have not received any benefits 还未领取养老保险金
2. Currently receive benefits 正在领取征地养老保险金 → Skip to FN079_W2_10 跳至 FN079_W2_10
3. No 没有参加或领取征地养老保险 → Skip to FN073_W4 跳至 FN073_W4

FN079_W4 Where did you participate in the pension? 您在哪儿参加的?

1. The same as current residence BB001_W3 和当前一般居住地 BB001_W3 一样
2. County/city/district of current residence BB001_W3 当前一般居住地 BB001_W3 所在县/市/区的 _____ (**FN079_W4_1**) other townships/subdistricts 其他乡/镇/街道，_____ (**FN079_W4_2**) villages/neighborhoods 村/社区
3. Other 其它：_____ (**FN079_W4_3**) province_city_county/city/district 省 _ 市 _ 县/市/区，_____ (**FN079_W4_4**) township/subdistrict/village/neighborhood 乡/镇/街道/村/社区

997. Unknown 不知道

FN079_W2_4 Does your pension for land-expropriated farmers require individual contributions? 您的征地养老保险是否需要个人缴费?

1. Yes 是
2. No 否 → Skip to FN079_W2_8 跳至 FN079_W2_8

FN079_W2_6 How much do you need to pay? 要缴纳多少钱? _____ (**FN079_W2_6_1**) Yuan per month 元/月 or 或 _____ (**FN079_W2_6_2**) Yuan per year 元/年
[IWER: Use "-1" for unknown, ask unfolding bracket questions for "-1" or "0 Yuan" 访员注意：允许填写"1"表示不知道，填写"1"或回答 0 元时，将分级展开提问]

FN079_W2_6_bracket [IWER: If R is unwilling to answer, does not remember, or the answer is 0 Yuan, ask unfolding bracket questions 访员注意：如果受访者不愿回答，忘记了，或者回答 0 元时，在此处分级展开提问] 100 /500 /800 /1,000 /1,500 Yuan/month 元/月

FN079_W2_8 When do you expect to receive this pension? 您预期在什么时候享用该保险? At age 在 _____ 45...120 (**FN079_W2_8_1**) years old 岁 or in 或在 _____ 0.00...100.00 (**FN079_W2_8_2**) years 年之后

FN079_W2_9 How much do you expect to receive? 您预期自己能领多少钱?_____ (**FN079_W2_9_1**) Yuan per month 元/月 Or 或 _____ (**FN079_W2_9_2**) Yuan 元（Lump sum amount 一次结清）

[IWER: Use "-1" for unknown, ask unfolding bracket questions for "-1" or "0 Yuan" 访员注意：允许填写"1"表示不知道，填写"1"或回答 0 元时，将分级展开提问]

FN079_W2_9_bracket [IWER: If R is unwilling to answer, does not remember, or the answer is 0 Yuan, ask unfolding bracket questions 访员注意：如果受访者不愿回答，忘记了，或者回答 0 元时，在此处分级展开提问] 100 /500 /1,000 /1,500 /2,000 Yuan/month 元/月

PROCEDURE 程序:

Skip to FN073_W4 跳至 FN073_W4

FN079_W2_10 In what month and year did you start to receive this pension? 您是从哪年哪月开始领取征地养老保险金的? _____ 1900...2018 (**FN079_W2_10_1**) Year 年 _____ 0...12 (**FN079_W2_10_2**) Month 月

[IWER: Year is in four digits. Month is in its actual value. For example, January is "1" not "01", December is "12". If R does not remember the month, fill "0" 访员注意: 用 4 位数表示年，按照实际的月份填写月。例：1 月写作"1"，而不是"01"，12 月写作"12"。如果记不住月份，请填入"0"]

FN079_W2_11 How much do you currently receive each month? 您现在每月领多少钱? _____ Yuan per month 元/月

[IWER: Use "-1" for unknown, ask unfolding bracket questions for "-1" or "0 Yuan" 访员注意：允许填写"1"表示不知道，填写"1"或回答 0 元时，将分级展开提问]

FN079_W2_11_bracket [IWER: If R is unwilling to answer, does not remember, or the answer is 0 Yuan, ask unfolding bracket questions 访员注意：如果受访者不愿回答，忘记了，或者回答 0 元时，在此处分级展开提问] 10 /50 /100 /500 /1,000 Yuan/month 元/月

FN080_W4 Where do you currently receive the benefits? 您目前在哪儿领取?

1. The same as current residence BB001_W3 和当前一般居住地 BB001_W3 一样
2. County/city/district of current residence BB001_W3 当前一般居住地 BB001_W3 所在县/市/区的 _____ (**FN080_W4_1**) other townships/subdistricts 其他乡/镇/街道, _____ (**FN080_W4_2**) villages/neighborhoods 村/社区
3. Other 其它: _____ (**FN080_W4_3**) province_city_county/city/district 省 _ 市 _ 县/市/区, _____ (**FN080_W4_4**) township/subdistrict/village/neighborhood 乡/镇/街道/村/社区

997. Unknown 不知道

PART 5 Life Insurance 人寿保险

FN073_W4 Do you currently receive or expect to receive or contribute to any life insurance?
您是否正在领取或将来可以领取或目前正在缴费人寿保险?
1. Yes 是, 参加或领取
2. No 否 → Skip to FN043_W4 跳至 FN043_W4

FN056_W2_3 What kind of life insurance did you buy? 你购买的人寿保险是哪一种?
[IWER: If R has multiple life insurances policies, record the most important one 访员注意：如果受访者有多种人寿保险，在此处记录最主要的一种]
1. Term life insurance 定期寿险
2. Whole life insurance 终身寿险
3. Pure endowment insurance 生存保险
4. Endowment insurance 生死两全保险
5. Other 其它，please specify 请填写具体名称 ______ **(FN056_W2_3_1)**

FN056_W2_7 have you ever received a life insurance payout? 你是否从人寿保险中领取过赔付金?
1. Yes 是 → Skip to FN077_W4 跳至 FN077_W4
2. No 否

FN074_W4 Where did you participate in this insurance? 您在哪儿参加的这份保险?
1. The same as current residence BB001_W3 和当前一般居住地 BB001_W3 一样
2. County/city/district of current residence BB001_W3 当前一般居住地 BB001_W3 所在县/市/区的 ______ **(FN074_W4_1)** other townships/subdistricts 其他乡/镇/街道，______ **(FN074_W4_2)** villages/neighborhoods 村/社区
3. Other 其它: ______ **(FN074_W4_3)** province_city_county/city/district 省 _ 市 _ 县/市/区, ______ **(FN074_W4_4)** township/subdistrict/village/neighborhood 乡/镇/街道/村/社区

997. Unknown 不知道

FN075_W4 For how many years have you contributed to or participated in other ways in this pension? 请问您给这个养老保险缴费或以其它方式参加多少年了？ ______ 0.00..100.00 Year(s) 年

FN056_W2_5 How to pay the insurance premium? 保险费的缴纳方式是?
1. Monthly 月交
2. Quaterly 季交
3. Biannually 半年交
4. Annually 年交

FN056_W2_6 How much is the payment amount? 缴费金额是多少?
1. If pay it monthly 如果月交，______ **(FN056_W2_6_1)** Yuan/Month 元/月
2. If pay it quarterly 如果季交，______ **(FN056_W2_6_2)** Yuan/Quarter 元/季

3. If pay it biannually 如果半年交，_____ (**FN056_W2_6_3**) Yuan/Half a year 元/半年
4. If pay it annually 如果年交，_____ (**FN056_W2_6_4**) Yuan/Year 元/年

FN076_W4 In the future, can you receive pension benefits for yourself from the life insurance mentioned above 您自己以后可以从上述人寿保险中领取养老金吗?

1. Yes 是的，我可以
2. No 不，我不可以 → Skip to FN043_W4 跳至 FN043_W4

FN076_W4_a When do you expect to claim life insurance benefits? 您预期在什么时候享用该保险? At age 在 _____ 45...120 (**FN076_W4_a_1**) years old 岁 or in 或在 _____ 0.00...100.00 (**FN076_W4_a_2**) years 年之后

FN056_W2_9 How much do you expect to receive? 你预计将来从这份人寿保险中领取多少钱? _____ (**FN056_W2_9_1**) Yuan/Month 元/月 Or 或 _____ (**FN056_W2_9_2**) Yuan 元（Lump sum amount 一次结清）

[IWER: Use "-1" for unknown, ask unfolding bracket questions for "-1" or "0 Yuan" 访员注意：允许填写"1"表示不知道，填写"1"或回答 0 元时，将分级展开提问]

FN056_W2_9_bracket [IWER: If R is unwilling to answer, does not remember, or the answer is 0 Yuan, ask unfolding bracket questions 访员注意：如果受访者不愿回答，忘记了，或者回答 0 元时，在此处分级展开提问] 1,000/2,000 /3,000 /6,000 /10,000 Yuan 元

PROCEDURE 程序:
Skip to FN043_W4 跳至 FN043_W4

FN077_W4 In what month and year did you start to receive benefits from life insurance? 您是从哪年哪月领取这份人寿保险的? _____ 1900...2018 (**FN077_W4_1**) Year 年 _____ 0...12 (**FN077_W4_2**) Month 月

[IWER：Year is in four digits. Month is in its actual value. For example, January is "1" not "01", December is "12". If R does not remember the month, fill "0" 访员注意：用 4 位数表示年，按照实际的月份填写月。例：1 月写作"1"，而不是"01"，12 月写作"12"。如果记不住月份，请填入"0"]

FN056_W2_8 How much did you get? 领取过多少钱? _____ Yuan 元

[IWER: Use "-1" for unknown, ask unfolding bracket questions for "-1" or "0 Yuan" 访员注意：允许填写"1"表示不知道，填写"1"或回答 0 元时，将分级展开提问]

FN056_W2_8_bracket [IWER: If R is unwilling to answer, does not remember, or the answer is 0 Yuan, ask unfolding bracket questions 访员注意：如果受访者不愿回答，忘记了，或者回答 0 元时，在此处分级展开提问] 1,000/3,000 /5,000 /8,000 /10,000 Yuan 元

FN078_W4 From where do you receive these life insurance benefits? 您目前在哪儿领取这份人寿保险?

1. The same as current residence BB001_W3 和当前一般居住地 BB001_W3 一样

2. County/city/district of current residence BB001_W3 当前一般居住地 BB001_W3 所在县/市/区的 _____ (**FN078_W4_1**) other townships/subdistricts 其他乡/镇/街道, _____ (**FN078_W4_2**) villages/neighborhoods 村/社区
3. Other 其它: _____ (**FN078_W4_3**) province_city_county/city/district 省 _ 市 _ 县/市/区, _____ (**FN078_W4_4**) township/subdistrict/village/neighborhood 乡/镇/街道/村/社区

997. Unknown 不知道

PART 6 Commercial Pension Insurance（Exclude Life Insurance）商业养老保险（人寿保险除外）

FN043_W4 Do you currently receive or expect to receive or contribute to any commercial pension insurance? 您是否正在领取或预计将来可以领取或正在缴费商业养老保险?

1. Yes, I purchased 参加了
2. Yes, I'm receivng the benefits 现在正在领取 → Skip to FN055_W2 跳至 FN055_W2
3. Neither 没有参加也没有领取 → Skip to FN083_W2 跳至 FN083_W2

FN044_W2 Where did you participate in this insurance? 您在哪儿参加的这份保险?

1. The same as current residence BB001_W3 和当前一般居住地 BB001_W3 一样
2. County/city/district of current residence BB001_W3 当前一般居住地 BB001_W3 所在县/市/区的 _____ (**FN044_W4_1**) other townships/subdistricts 其他乡/镇/街道, _____ (**FN044_W4_2**) villages/neighborhoods 村/社区
3. Other 其它: _____ (**FN044_W4_3**) province_city_county/city/district 省 _ 市 _ 县/市/区, _____ (**FN044_W4_4**) township/subdistrict/village/neighborhood 乡/镇/街道/村/社区

997. Unknown 不知道

FN047_W2 How do you contribute to the commercial pension? 以什么样的形式交纳保险费?

1. By annually 按年
2. By lump sum payment 一次性缴费 → Skip to FN046_W4 跳至 FN046_W4

FN045_W4 For how many years have you contributed to or participated in other ways in this pension? 请问您给这个养老保险缴费或以其它方式参加多少年了? _____ 0.00..100.00 Year(s) 年

FN048_W2 How much premium do you pay every year? 您每年需要交纳多少保险费? _____ Yuan/year 元/年
[IWER: Use "-1" for unknown, ask unfolding bracket questions for "-1" or "0 Yuan" 访员注意：允许填写"1"表示不知道，填写"1"或回答 0 元时，将分级展开提问]

FN048_W2_bracket [IWER: If R is unwilling to answer, does not remember, or the answer is 0 Yuan, ask unfolding bracket questions 访员注意：如果受访者不愿回答，忘记了，或者回答 0 元时，在此处分级展开提问] 3,000 /6,000 /10,000 /20,000 /30,000 Yuan per year 元/年

FN049_W2 How many years do you need to pay? 您需要缴几年保险费? _____ 0.00..100.00 Years 年
[Soft Check: Prompt for verification if greater than a legal maximum]

PROCEDURE 程序:
Skip to FN051_W4 跳至 FN051_W4

FN046_W4 When did you start paying for the commercial pension? 您何时开始给这商业养老保险缴费? _____ 1900...2018 (**FN046_W4_1**) Year 年 _____ 0...12 (**FN046_W4_2**) Month 月
[IWER: Year is in four digits. Month is in its actual value. For example, January is "1" not "01", December is "12". If R does not remember the month, fill "0" 访员注意: 用 4 位数表示年, 按照实际的月份填写月。例: 1 月写作"1", 而不是"01", 12 月写作"12"。如果记不住月份, 请填入"0"]

FN050_W2 How much premium do you need to pay in total? 您一共需要交纳多少保险费? _____ Yuan 元
[IWER: Use "-1" for unknown, ask unfolding bracket questions for "-1" or "0 Yuan" 访员注意: 允许填写"1"表示不知道, 填写"1"或回答 0 元时, 将分级展开提问]

FN050_W2_bracket [IWER: If R is unwilling to answer, does not remember, or the answer is 0 Yuan, ask unfolding bracket questions 访员注意: 如果受访者不愿回答, 忘记了, 或者回答 0 元时, 在此处分级展开提问] 1,000 /2,000 /5,000 /10,000 /20,000 Yuan 元

FN051_W4 When do you expect to claim this insurance benefit? 您预期在什么时候享用该保险? At age 在 _____ 45...120 (**FN051_W4_1**) years old 岁 or in 或在 _____ 0.00...100.00 (**FN051_W4_2**) years 年之后

FN051_W2 How do you receive the pension? 保险费的领取方式?
1. 1.A lump-sum payout 一次性领取 → Skip to FN054_W4 跳至 FN054_W4
2. Annually 年领
3. Monthly 月领 → Skip to FN053_W2 跳至 FN053_W2

FN052_W4 How much benefit do you expect to receive? 您预期每年能领取多少保险费? _____ Yuan/year 元/年
[IWER: Use "-1" for unknown, ask unfolding bracket questions for "-1" or "0 Yuan" 访员注意: 允许填写"1"表示不知道, 填写"1"或回答 0 元时, 将分级展开提问]
[Soft Check: Prompt for verification if low or high, e.g. FN052_w4 < 1200 per year or FN052_w4 > 60000 per year]

FN052_W4_bracket [IWER: If R is unwilling to answer, does not remember, or the answer is 0 Yuan, ask unfolding bracket questions 访员注意: 如果受访者不愿回答, 忘记了, 或者回答 0 元时, 在此处分级展开提问] 1,200 /6,000 /12,000 /24,000 /36,000 Yuan 元

PROCEDURE 程序:
Skip to FN083_W2 跳至 FN083_W2

FN053_W2 How much do you expect to receive each month in the future? 您预期以后每月能领取多少养老金? _____ Yuan/month 元/月
[IWER: Use "-1" for unknown, ask unfolding bracket questions for "-1" or "0 Yuan" 访员注意:允许填写"1"表示不知道,填写"1"或回答 0 元时,将分级展开提问]
[Soft Check: Prompt for verification if low or high, e.g. FN053_w2 < 100 per month or FN053_w2 >5000 per year]

FN053_W2_bracket [IWER: If R is unwilling to answer, does not remember, or the answer is 0 Yuan, ask unfolding bracket questions 访员注意:如果受访者不愿回答,忘记了,或者回答 0 元时,在此处分级展开提问] 500 /1,000 /2,000 /3,500 /5,000 Yuan 元

PROCEDURE 程序:
Skip to FN083_W2 跳至 FN083_W2

FN054_W4 How much lump sum benefit do you expect to receive? 您预期一次性领取的养老金总额能达到多少? _____ Yuan 元
[IWER: Use "-1" for unknown, ask unfolding bracket questions for "-1" or "0 Yuan" 访员注意:允许填写"1"表示不知道,填写"1"或回答 0 元时,将分级展开提问]

FN054_W4_bracket [IWER: If R is unwilling to answer, does not remember, or the answer is 0 Yuan, ask unfolding bracket questions 访员注意:如果受访者不愿回答,忘记了,或者回答 0 元时,在此处分级展开提问] 1,000 /5,000 /10,000 /50,000 /100,000 Yuan 元

PROCEDURE 程序:
Skip to FN083_W2 跳至 FN083_W2

FN055_W2 In what month and year did you start to receive commercial pension benefits? 您是从哪年哪月开始领商业养老金的? _____ 1900...2018 (**FN055_W2_1**) Year 年 _____ 0...12 (**FN055_W2_2**) Month 月
[IWER: Year is in four digits. Month is in its actual value. For example, January is "1" not "01", December is "12". If R does not remember the month, fill "0" 访员注意: 用 4 位数表示年,按照实际的月份填写月。例:1 月写作"1",而不是"01",12 月写作"12"。如果记不住月份,请填入"0"]

FN056_W2 What is your monthly benefit? 您每月领多少钱? _____ Yuan per month 元/月
[IWER: Use "-1" for unknown, ask unfolding bracket questions for "-1" or "0 Yuan" 访员注意:允许填写"1"表示不知道,填写"1"或回答 0 元时,将分级展开提问]

FN056_W2_bracket [IWER: If R is unwilling to answer, does not remember, or the answer is 0 Yuan, ask unfolding bracket questions 访员注意:如果受访者不愿回答,忘记了,或者回答 0 元时,在此处分级展开提问] 500 /1,000 /2,000 /3,500 /5,000 Yuan 元

FN056_W4 Where do you currently receive benefits from this commercial insurance? 您目前在哪儿领取这份商业养老保险?

1. The same as current residence BB001_W3 和当前一般居住地 BB001_W3 一样
2. County/city/district of current residence BB001_W3 当前一般居住地 BB001_W3 所在县/市/区的 _____ (**FN056_W4_1**) other townships/subdistricts 其他乡/镇/街道, _____ (**FN056_W4_2**) villages/neighborhoods 村/社区
3. Other 其它: _____ (**FN056_W4_3**) province_city_county/city/district 省 _ 市 _ 县/市/区, _____ (**FN056_W4_4**) township/subdistrict/village/neighborhood 乡/镇/街道/村/社区

997. Unknown 不知道

PART 7 Other Pension 其他养老保险

FN083_W2 In addition to the aforementioned pension programs, do you currently receive or expect to receive or contribute to any other pension? 除了上述几种养老保险之外，您是否正在领取或预计可以领取或目前正在缴费其他养老保险金?

1. Yes 是，participate in 正在参加其他养老保险项目/正在缴费，目前还未领取养老金
2. Yes 是，receive 参加了其他养老保险项目，现在在领取养老金
3. No 否，没有参加其他养老保险项目

PROCEDURE 程序:

If FN083_W2 = 3 and (FN002_W4 = 1 or FN030_W4 = 1 or FN058_W4 = 1 or FN079_W2_3 = 1, 2 or FN073_W4 = 1 or FN043_W4 = 1, 2), Skip to FN097_W2 如果 FN083_W2 = 3 并且 (FN002_W4 = 1 或 FN030_W4 = 1 或 FN058_W4 = 1 或 FN079_W2_3 = 1, 2 或 FN073_W4 = 1 或 FN043_W4 = 1, 2), 跳至 FN097_W2

If FN083_W2 = 3 and FN002_W4 = 2 and FN030_W4 = 2 and FN058_W4 = 2 and FN079_W2_3 = 3 and FN073_W4 = 2 and FN043_W4 = 3 , Skip to FN099_W4 如果 FN083_W2 = 3 且 FN002_W4 = 2 且 FN030_W4 = 2 且 FN058_W4 = 2 且 FN079_W2_3 = 3 且 FN073_W4 = 2 且 FN043_W4 = 3 , 跳至 FN099_W4

FN084_W2 What is the name of the program? 这个养老保险的名称是 _____

[IWER: If R participates/receives multiple other types of pensions, choose the most important one to answer 访员注意：如果受访者回答正在参加/领取多份其他养老保险，请其选取自己认为最主要的一份回答]

FN084_W4 Do you currently receive benefits from this pension program? 您现在正在领取这份养老保险吗?

1. Yes 是的，我正在领取 → Skip to FN095_W2 跳至 FN095_W2
2. No 不，我还没有开始领取

FN085_W2 Where did you participate in the pension program? 您在哪儿参加的这份保险项目?

1. The same as current residence BB001_W3 和当前一般居住地 BB001_W3 一样

2. County/city/district of current residence BB001_W3 当前一般居住地 BB001_W3 所在县/市/区的 _____ (**FN085_W4_1**) other townships/subdistricts 其他乡/镇/街道, _____ (**FN085_W4_2**) villages/neighborhoods 村/社区
3. Other 其它: _____ (**FN085_W4_3**) province_city_county/city/district 省 _ 市 _ 县/市/区, _____ (**FN085_W4_4**) township/subdistrict/village/neighborhood 乡/镇/街道/村/社区

997. Unknown 不知道

FN087_W2 Did you need to pay the premium? 您参加的这份养老保险需要缴费吗?
1. Yes 需要
2. No 不需要 → Skip to FN093_W2 跳至 FN093_W2

FN089_W2 How do you contribute to the pension? 以什么样的形式交纳保险费?
1. Annually or Monthly 按年或按月
2. Lump sum payment 一次性缴费 → Skip to FN086_W4 跳至 FN086_W4

FN090_W4 How many years have you participated in this pension 请问您给这个养老保险缴费或以其它方式参加多少年了? _____ 0.00...100.00 Year(s) 年

FN091_W2 How many years do you need to pay? 您需要缴几年保险费? _____ 0.00...100.00 Years 年
[Soft Check: Prompt for verification if greater than a legal maximum]

FN090_W2 On an annual basis, how much do you pay every year? 按年算的话, 您每年需要交纳多少保险费? _____ Yuan/year 元/年
[IWER: Use "-1" for unknown, ask unfolding bracket questions for "-1" or "0 Yuan" 访员注意: 允许填写 "1" 表示不知道, 填写 "1" 或回答 0 元时, 将分级展开提问]

FN090_W2_bracket [IWER: If R is unwilling to answer, does not remember, or the answer is 0 Yuan, ask unfolding bracket questions 访员注意: 如果受访者不愿回答, 忘记了, 或者回答 0 元时, 在此处分级展开提问] 500 /1,000 /2,000 /3,000 /5,000 Yuan 元

**PROCEDURE** 程序:
Skip to FN093_W2 跳至 FN093_W2

FN086_W4 When did you start paying this pension? 您何时开始给这份养老保险缴费? _____ 1900...2018 (**FN086_W4_1**) Year 年 _____ 0...12 (**FN086_W4_2**) Month 月
[IWER: Year is in four digits. Month is in its actual value. For example, January is "1" not "01", December is "12". If R does not remember the month, fill "0" 访员注意: 用 4 位数表示年, 按照实际的月份填写月。例: 1 月写作 "1", 而不是 "01", 12 月写作 "12"。如果记不住月份, 请填入 "0"]

FN092_W4 How much premium do you need to pay in total? 您一次性一共缴费多少? _____ Yuan 元
[IWER: Use "-1" for unknown, ask unfolding bracket questions for "-1" or "0 Yuan" 访员注意: 允许填写 "1" 表示不知道, 填写 "1" 或回答 0 元时, 将分级展开提问]

FN092_W4_bracket [IWER: If R is unwilling to answer, does not remember, or the answer is 0 Yuan, ask unfolding bracket questions 访员注意：如果受访者不愿回答，忘记了，或者回答 0 元时，在此处分级展开提问] 1,000 /2,000 /5,000 /10,000 /20,000 Yuan 元

FN093_W2 When do you expect to receive pension 您预期在多大年龄享用该保险？At age 在_____ (**FN093_W2_1**) 45...120 岁 or in 或在 _____ (**FN093_W2_2**) years 年之后

FN094_W2 How much do you expect to receive? 您预期自己能领多少钱？_____ (**FN094_W2_1**) Yuan per month 元/月 Or 或 _____ (**FN094_W2_2**) Yuan 元（Lump sum amount 一次结清）

**PROCEDURE** 程序:
Skip to FN097_W2 跳至 FN097_W2

FN095_W2 In what month and year did you start to receive this pension benefits? 您是从哪年哪月开始领取这份养老金或者退休金的？_____ 1900...2018 (**FN095_W2_1**) year 年 _____ 0...12 (**FN095_W2_2**) month 月
[IWER: Year is in four digits. Month is in its actual value. For example, January is “1” not “01”, December is “12”. If R does not remember the month, fill “0” 访员注意：用 4 位数表示年，按照实际的月份填写月。例：1 月写作“1”，而不是“01”，12 月写作“12”。如果记不住月份，请填入“0”]

FN096_W2 How much do you currently receive each month? 您现在每月领多少钱？_____ Yuan per month 元/月
[IWER: Use “-1” for unknown, ask unfolding bracket questions for “-1” or “0 Yuan” 访员注意：允许填写“1”表示不知道，填写“1”或回答 0 元时，将分级展开提问]

FN096_W2_bracket [IWER: If R is unwilling to answer, does not remember, or the answer is 0 Yuan, ask unfolding bracket questions 访员注意：如果受访者不愿回答，忘记了，或者回答 0 元时，在此处分级展开提问] 500 /1,000 /2,000 /3,500 /5,000 Yuan 元

FN096_W4 Where do you currently receive this pension? 您目前在哪儿领取这份养老保险？

1. The same as current residence BB001_W3 和当前一般居住地 BB001_W3 一样
2. County/city/district of current residence BB001_W3 当前一般居住地 BB001_W3 所在县/市/区的 _____ (**FN096_W4_1**) other townships/subdistricts 其他乡/镇/街道，_____ (**FN096_W4_2**) villages/neighborhoods 村/社区
3. Other 其它：_____ (**FN096_W4_3**) province_city_county/city/district 省 _ 市 _ 县/市/区，_____ (**FN096_W4_4**) township/subdistrict/village/neighborhood 乡/镇/街道/村/社区

997. Unknown 不知道

**PROCEDURE** 程序:
Skip to FN097_W2 跳至 FN097_W2

[Ask R who do not enroll in any pension 以上一种养老保险都没有的受访者回答]

FN099_W4 Do you currently receive, or expect to receive or contribute to the following pensions? (Choose all that apply) 是否正在领取，预计将来可以领取或目前正在缴费以下养老保险（多选）?

[Hard Check: If the response is that R receives and enrolls the following pensions, option 1-13, IWER should check and confirm. If it was missing previously, go back and fill in the information 如果受访者回答正在领取和参保以下的养老保险，选项 1-13，访员应该核查并确认。如果是之前的信息没有填写，应回到前面相应的部分把信息补全]

1. Pension for public servants 政府机关或公务员的退休金
2. Pension for public institution employees 事业单位退休金
3. Basic pension for enterprise employees 职工基本养老保险
4. Supplementary pension for public servants (Annuity) 政府机关或公务员的补充养老保险（年金）
5. Supplementary pension for public institution employees (Annuity) 事业编制职工的补充养老保险（年金）
6. Supplementary pension for enterprise employees (Enterprise Annuity) 企业职工补充养老保险（企业年金）
7. Urban and Rural Resident Pension 城乡居民养老保险
8. New Rural Resident Pension 新型农村养老保险
9. Urban Resident Pension 城镇居民养老保险
10. Pension for land-expropriated farmers 征地养老保险
11. Life insurance 人寿保险
12. Commercial pension insurance (Exclude life insurance) 商业养老保险（人寿保险除外）
13. Other pension programs 其他养老保险
14. Never claimed or enrolled in any pension program 一种养老保险都没有

997. Unknown 不知道
999. Refuse to answer 拒绝回答

PROCEDURE 程序:

If FN099_W4 = 14, 997, 999, ask FN100_W4 - FN102_W4 如果 FN099_W4 = 14, 997, 999，询问 FN100_W4 - FN102_W4

FN099_W4 = 14, 997, 999 cannot be chosen together with other options in FN099_W4

FN099_W4 = 14, 997, 999 不能和 FN099_W4 的其他选项一起选

FN100_W4 Have you ever had any of the following pensions? (choose all that apply) 是否曾经有过以下养老保险（多选）?

1. Pension for public servants 政府机关或公务员的退休金
2. Pension for public institution employees 事业单位退休金
3. Basic pension for enterprise employees 职工基本养老保险
4. Supplementary pension for public servants (Annuity) 政府机关或公务员的补充养老保险（年金）
5. Supplementary pension for public institution employees (Annuity) 事业编制职工的补充养老保险（年金）

6. Supplementary pension for enterprise employees (Enterprise Annuity) 企业职工补充养老保险（企业年金）
7. Urban and Rural Resident Pension 城乡居民养老保险
8. New Rural Resident Pension 新型农村养老保险
9. Urban Resident Pension 城镇居民养老保险
10. Pension for land-expropriated farmers 征地养老保险
11. Life insurance 人寿保险
12. Commercial pension insurance (Exclude life insurance) 商业养老保险（人寿保险除外）
13. Pension insurance for rural residents (Old rural pension) 农村养老保险（老农保）
14. Other pension programs, please specify 其他养老保险, 请注明 _____ **(FN100_W4_1)**
15. Never claimed or enrolled in any pension program 从来都没有领取或参保过任何养老保险

997. Unknown 不知道
999. Refuse to answer 拒绝回答

PROCEDURE 程序:

If FN100_W4 = 1, 2, 3, 4, 5, 6, 7, 8, 9, 10, 11, 12, 13, 14, then ask FN101_W4 - FN102_W4 in loops 如果 FN100_W4 = 1, 2, 3, 4, 5, 6, 7, 8, 9, 10, 11, 12, 13, 14, 则循环询问 FN101_W4 - FN102_W4

To generate [Name of Pension] in a loop: if FN100_W4 = 1, 2, 3, 4, 5, 6, 7, 8, 9, 10, 11, 12, 13, 14, then [Name of Pension] is the corresponding option 循环中的 [保险名称] 生成：如果 FN100_W4 = 1, 2, 3, 4, 5, 6, 7, 8, 9, 10, 11, 12, 13, 14, 则 [保险名称] 为对应的选项

If FN100_W4 = 15, 997, 999, then Skip to FN097_W2 如果 FN100_W4 = 15, 997, 999, 则跳至 FN097_W2

FN101_W4 When did you stop participating in [Name of Pension]? 何时停止这份 [保险名称]?

_____ 1900...2018 **(FN101_W4_1)** Year 年 _____ 0...12 **(FN101_W4_2)** Month 月

[IWER: Year is in four digits. Month is in its actual value. For example, January is "1" not "01", December is "12". If R does not remember the month, fill "0" 访员注意：用 4 位数表示年，按照实际的月份填写月。例：1 月写作 "1"，而不是 "01"，12 月写作 "12"。如果记不住月份，请填入 "0"]

FN102_W4 Why did you stop participating in [Name of Pension]? 为什么停止这份 [保险名称]?

_____

[Ask all R 所有人回答]

FN097_W2 If you are too old to work, what would be your financial resource? 如果您将来老了干不动工作了，您认为生活来源主要将是什么?

[IWER: "Commercial insurance" refers to the life insurance that R directly purchases from the commercial insurance company, pays premium regularly, and starts to receive benefits continuously and regularly at the contracted age 访员注意："商业养老保险" 是指受访者直接向商业保险公司投保，定期缴纳保险费，从合同约定年龄开始持续、定期地领取养老金的人寿保险，能有效地满足客户的养老需要]

1. Children 子女 → Skip to FN098_W2 跳至 FN098_W2
2. Savings 储蓄 → End this section 结束本部分
3. Pension 养老金或退休金 → End this section 结束本部分
4. Commercial pension insurance 商业养老保险 → End this section 结束本部分
5. Other 其他，please specify 请注明 ______ **(FN097_W2_1)** → End this section 结束本部分

FN098_W2 Which child(ren)? (choose all that apply) 是哪个/哪些子女?（可多选）

1-N. Preload the list of children 加载子女列表

This page intentionally left blank

G&H Income, Expenditures and Assets 收入、支出与资产

G2 Household Income and Expenditures 家户收入与支出

[IWER: Part 1_1 is asked of the main respondent and spouse respectively. Other parts in this section is asked of the family financial respondent. Do not allow a proxy respondent to answer the entire section Part 1_1 由主要受访者及其配偶分别回答，其余部分由家庭财务受访者回答。本部分不允许完全请别人代答]

PART 1 Household Wage Income and Individual-based Transfers 家户工资收入和个人获得的转移收入

Part 1_1: Main Respondent and Spouse's Wage Income and Individual-based Transfers 主要受访者及其配偶的工资收入和个人获得的转移收入

[IWER: Please conduct Part 1_1 when the main respondent and spouse are at home. Don't allow a proxy to complete the part 访员注意：当主要受访者及其配偶在场时，提问 Part 1_1 问卷。这部分不允许请人完全代填]

GA001 Did you receive any wage and bonus income in the past year? 过去一年，您有没有领工资，包括奖金、各种补贴，不包括退休工资？这里的工资来自所有的工作。

1. Yes 有
2. No 没有 → Skip to GA003_W4 跳至 GA003_W4

GA002 How much did you receive last year? 一共领了多少钱？ ______ Yuan 元

[Soft Check: $> 240,000$]

[IWER: If R is unwilling to answer or does not remember, ask unfolding bracket questions 访员注意：如果受访者不愿回答或者忘记了，展开提问]

GA002_bracket [CAPI: If Respondent is unwilling to answer, does not remember, or the input value is 0, please ask unfolding bracket questions here 如果受访者不愿回答或者忘记了或者填了 0 元，在此处分级展开提问] 5,000/10,000/30,000/50,000/100,000 Yuan 元

GA002_W2_1 Does the above mentioned wage exclude any insurance, income tax, public housing funds and other fees? 上面提到的工资有没有扣除各类保险、所得税、住房公积金或其他杂费？

1. Yes 有
2. No 没有

997. Do not know 不知道
999. Refuse to answer 拒绝回答 → Skip to GA003_W4 跳至 GA003_W4

GA002_W2_2 What is the total amount of your insurance, income tax, public housing funds and other fees? 您被扣除的/上交的个人所得税、各类保险、住房公积金或其他杂费一共是多少元?

1. ______ (**GA002_W2_2a**) Yuan/Year 元/年
2. ______ (**GA002_W2_2b**) Yuan/Month 元/月
3. About 或相当于工资的 ______ (**GA002_W2_2c**) % of wage
4. No 没有

997. Do not know 不知道
999. Refuse to answer 拒绝回答

[IWER: Ask unfolding bracket questions for option 1,2,3 if respondent forget the value 访员注意：对于选项 1，2，3，如果受访者忘记了，展开提问]

PROCEDURE 程序:

If GA002_W2_2 = 1, but GA002_W2_2a = 0 or missing, ask the unfolding bracket questions 如果 GA002_W2_2 = 1，但 GA002_W2_2a = 0 或缺失，那么分级展开提问

If GA002_W2_2 = 2, but GA002_W2_2b = 0 or missing, ask the unfolding bracket questions 如果 GA002_W2_2 = 2，但 GA002_W2_2b = 0 或缺失，那么分级展开提问

If GA002_W2_2 = 3, but GA002_W2_2c = 0 or missing, ask the unfolding bracket questions 如果 GA002_W2_2 = 3，但 GA002_W2_2c = 0 或缺失，那么分级展开提问

If GA002_W2_2 = 997, ask the unfolding questions 如果 GA002_W2_2 = 997，那么分级展开提问

GA002_W2_2_bracket What is the total amount of personal income tax, insurance, public housing funds and other fees? 个人所得税、各类保险、住房公积金或其他杂费一共是多少元/月？300/500/1,000/2,000/3,000 Yuan/Month 元/月

GA003_W4 Did you receive any of the following types of income transfers in the past year? (check all that apply) 过去一年，您有没有领到下列转移支付收入？(可多选)

[F1: Medical Aid: Medical Aid for Serious Diseases refers to additional cash assistance outside of Medical Insurance Scope for urban and rural residents who are living difficultly caused by a major illness. Medical Aid play as final support outside the basic medical insurance F1：医疗救助：重特大疾病的医疗救助是指给予患有重大疾病并造成医疗和家庭生活困难的城乡居民，在医疗保险之外一定金额的现金救助。医疗救助主要是发挥在基本医疗保险以外的兜底作用]

1. Pensions (including wages from governments, public institutions and firms, supplemental pension of the firms,and income from such programs as rural pension insurance, Urban residents’ pension and commercial pension insurance, new rural social pension insurance) 退休金或养老金, 包括政府机关和事业单位退休金，企业职工基本养老保险，企业补充养老保险，农村/城乡/城镇居民养老保险，商业养老保险，人寿保险，征地养老保险等，领了 ______ (**GA003_W4_1**) Yuan 元
2. Unemployment compensation 失业补助，领了 ______ (**GA003_W4_2**) Yuan 元
3. Pension voucher 养老卡/券，领了 ______ (**GA003_W4_3**) Yuan 元

4. Pension subsidy for the oldest old 高龄老人养老补助, 领了 _____ (**GA003_W4_4**) Yuan 元
5. Workers' compensation from Industrial Accident Compensation Insurance includes wage-replacement benefits, disability benefits, and survivors' benefits 工伤保险金包括误工补贴、伤残补助等，领了 _____ (**GA003_W4_5**) Yuan 元
6. Elderly family planning subsidies 独生子女老年补助, 领了 _____ (**GA003_W4_6**) Yuan 元
7. Medical aid 医疗救助，领了 _____ (**GA003_W4_7**)Yuan 元
8. Other government subsidies 政府给个人的其他补助，please specify 请注明 _____ (**GA003_W4_8_1**)，领了 _____ (**GA003_W4_8**) Yuan 元
9. Other social income transfer sources 社会给个人的其他转移支付收入，如社会捐助等，please specify 请注明 _____ (**GA003_W4_9_1**)，领了 _____ (**GA003_W4_9**) Yuan 元
10. None of the above 以上均没有 → Skip to GA009_W4 BRANCHPOINT 跳至 GA009_W4 BRANCHPOINT

PROCEDURE 程序:

If GA003_W4 = 3, ask GA004_W4_3_1 如果 GA003_W4 = 3，即受访者领取了养老卡/券，询问 GA004_W4_3_1

GA004_W4_3_1 How do you use the voucher? (check all that apply) 您怎样使用养老卡/券的?（可多选）

1. Life care 生活照料
2. Domestic service 家政服务
3. Rehabilitation service 康复服务
4. Buy food 养老餐桌
5. Buy medicine 购买药品
6. Buy life items 购买生活物品
7. Emergency 紧急救护
8. Other 其他，please specify 请注明 _____ (**GA004_W4_3_2**)

PROCEDURE 程序:

If GA003_W4 = 1 and GA003_W4_1 = 0 / missing, Respondent received pension but did not answer how much received, or input value is 0, ask GA004_W4_1_bracket 如果 GA003_W4 = 1 且 GA003_W4_1 = 0 或缺失，即领取了退休金或养老金，但是没回答多少钱或者领取了 0 元，提问 GA004_W4_1_bracket

GA004_W4_1_bracket How much pension have you received last year? 过去一年，退休金/养老金一共领了多少钱?

400 /600 /1,000 /3,000 /20,000 Yuan 元/年

GA009_W4 BRANCHPOINT:

If XRType = REIW, Skip to GA009_W4_1, ask GA009_W4_1 to GA009_W4_5 如果 XRType = REIW，即回访受访者，跳至 GA009_W4_1，回答 GA009_W4_1 到 GA009_W4_5

If XRType = NEWIW, Skip to GA011_W4_1, ask GA011_W4_1 to GA011_W4_5 如果 XRType = NEWIW，即新受访者，跳至 GA011_W4_1，回答 GA011_W4_1 到 GA011_W4_5

GA009_W4_1 Have you ever inherited anything since 2013? 从 2013 年以来，您是否继承过遗产？
1. Yes 是，几次？ _____ (**GA009_W4_1_1**) times 次
2. No 否 → Skip to GA000_W4 跳至 GA000_W4

999. Refuse to answer 拒绝回答 → Skip to GA000_W4 跳至 GA000_W4

PROCEDURE 程序：
If GA009_W4_1 = 1 and GA009_W4_1_1 = 1, ask GA009_W4_2 and GA009_W4_3 如果 GA009_W4_1 = 1 且 GA009_W4_1_1 = 1，询问 GA009_W4_2 和 GA009_W4_3

GA009_W4_2 How much have you inherited? The original value when inherited was 您继承的遗产值多少钱？按继承时的价值来算 _____ (**GA009_W4_2_1**) Yuan 元，When did the inheritance occur? 哪一年继承的遗产？ _____ (**GA009_W4_2_2**) Year 年
[Soft Check: GA009_W4_2_1 > 200,000]

GA009_W4_3 From whom you inherited? 您从谁那里继承了遗产？
1. Parents 父母
2. Parents-in-law 岳父母
3. Children 子女
4. Relatives 亲戚
5. Others 其它

PROCEDURE 程序：
If GA009_W4_1 = 1 and GA009_W4_1_1 > 1 or missing, ask GA009_W4_4 and GA009_W4_5 如果 GA009_W4_1 = 1 且 GA009_W4_1_1 > 1 或者缺失，询问 GA009_W4_4 和 GA009_W4_5

GA009_W4_4 How much in total have you inherited? The original value when inherited was 您继承的遗产总共值多少钱？按继承时的价值来算 _____ (**GA009_W4_4_1**) Yuan 元
The amount for largest inheritance was 继承的最大的一笔遗产值多少钱？_____ (**GA009_W4_4_2**) Yuan 元
When did the largest inheritance occur? 继承数目最大那次是哪一年？_____ (**GA009_W4_4_3**) Year 年
[Soft Check: GA009_W4_4_2 > 200,000]

GA009_W4_5 The largest inheritance was inerited from whom? 继承数目最大的那次，您从谁那里继承了遗产？
1. Parents 父母
2. Parents-in-law 岳父母
3. Children 子女
4. Relatives 亲戚
5. Others 其它

PROCEDURE 程序：
If new R, ask GA011_W4_1 to GA011_W4_5 如果是新受访者，询问 GA011_W4_1 至 GA011_W4_5

GA011_W4_1 Have you ever inherited anything? 您是否继承过遗产?

1. Yes 是，几次? _____ (**GA011_W4_1_1**) Times 次
2. No 否 → Skip to GA000_W4 跳至 GA000_W4

999. Refuse to answer 拒绝回答 → Skip to GA000_W4 跳至 GA000_W4

PROCEDURE 程序:
If GA011_W4_1 = 1 and GA011_W4_1_1 = 1, ask GA011_W4_2 and GA011_W4_3 如果 GA011_W4_1 = 1 且 GA011_W4_1_1 = 1，询问 GA011_W4_2 和 GA011_W4_3

GA011_W4_2 How much have you inherited? The original value when inherited was 您继承的遗产值多少钱? 按继承时的价值来算 _____ (**GA011_W4_2_1**) Yuan 元，When did the inheritance occur? 哪一年继承的遗产? _____ (**GA011_W4_2_2**) Year 年
[Soft Check: GA011_W4_2_1 > 200, 000]

GA011_W4_3 From whom you inherite? 您从谁那里继承了遗产?

1. Parents 父母
2. Parents-in-law 岳父母
3. Children 子女
4. Relatives 亲戚
5. Others 其它

PROCEDURE 程序:
If GA011_W4_1 = 1, and GA011_W4_1_1 > 1 or missing, ask GA011_W4_4 and GA011_W4_5 如果 GA011_W4_1 = 1 且 GA011_W4_1_1 > 1 或者缺失，询问 GA011_W4_4 和 GA011_W4_5

GA011_W4_4 How much in total have you inherited? The original value when inherited was 您继承的遗产总共值多少钱? 按继承时的价值来算 _____ (**GA011_W4_4_1**) Yuan 元
The amount for largest inheritance was 继承的最大的一笔遗产值多少钱?_____ (**GA011_W4_4_2**) Yuan 元
When did the largest inheritance occur? 继承数目最大那次是哪一年?_____ (**GA011_W4_4_3**) Year 年
[Soft Check: GA011_W4_4_2 > 200, 000]

GA011_W4_5 The largest inheritance was inerited from whom? 继承数目最大的那次，您从谁那里继承了遗产?

1. Parents 父母
2. Parents-in-law 岳父母
3. Children 子女
4. Relatives 亲戚
5. Others 其它

Part 1_2: Other Household Member's Wage Income and Individual-based transfers 其他家户成员的工资收入和个人获得的转移收入

GA000_W4 Please pick one member from the [Preload all household family members] who would be most familiar with the financial condition of the household? 以下哪个家户成员是家庭财务受访者？财务受访者须熟悉家庭的经济状况
[加载家户成员名单]

[IWER: Make sure no others are at present 访员注意：请确保没有其他人在场]

[INTRO: We'd like to ask you some questions regarding the incomes of OTHER members in your household. Your answers will be kept strictly confidential and will only be used for academic research 下面我们想问问您家其他家户成员的收入情况。我们将会对您的回答严格保密，并且仅用于学术研究]

GA005 BRANCHPOINT
Preload the other household members from Family Module. Ask GA005 to GA012_W4_5 to all other household members (excluding Main Respondent and the spouse of Main Respondent). If there are no other household members, Skip to GB001_W4 加载其他家户成员名单。其他家户成员名单要从家庭模块获得。对于每一个家户成员（不包括主要受访者及其配偶），分别询问 GA005 至 GA012_W4_5。如果没有其他家户成员，跳至 GB001_W4

GA005 Did [preload other household member's name] receive any wage and bonus income in the past year? 过去一年，[加载其他家户成员名字] 有没有领工资，包括奖金，不包括退休工资？这里的工资来自所有的工作。

1. Yes 有
2. No 没有 → Skip to GA007_W4 跳至 GA007_W4

997. Do not know 不知道 → Skip to GA007_W4 跳至 GA007_W4
999. Refuse to answer 拒绝回答 → Skip to GA007_W4 跳至 GA007_W4

GA006_W4 How much did [preload other household member's name] receive last year? 一共领了多少钱？ ______ Yuan 元
[Soft Check: > 240,000]
[IWER: If R is unwilling to answer or does not remember, ask unfolding bracket questions 访员注意：如果受访者不愿回答或者忘记了，展开提问]

GA006_bracket [CAPI: If Respondent is unwilling to answer, does not remember, or the input value is 0, please ask unfolding bracket questions here 如果受访者不愿回答或者忘记了或者填了 0 元，在此处分级展开提问] How much did [preload other household member's name] receive last year? 过去一年领了多少钱？
5,000/10,000/30,000/50,000/100,000 Yuan 元

GA006_W4_1 Does the above mentioned wage that [preload other household member's name] earned excludes any insurance, income tax, public housing funds and other fees? 上面提到的 [加载其他家户成员名字] 的工资有没有扣除各类保险、所得税、住房公积金或其他杂费？

1. Yes 有
2. No 没有

997. Do not know 不知道
999. Refuse to answer 拒绝回答 → Skip to GA007_W4 跳到 GA007_W4

GA006_W4_2 What is the total amount of [preload other household member's name]'s insurance, income tax, public housing funds and other fees? [加载其他家户成员名字] 被扣除的/上交的个人所得税、各类保险、住房公积金或其他杂费一共是多少元?

1. _____ (**GA006_W4_2a**) Yuan/Year 元/年
2. _____ (**GA006_W4_2b**) Yuan/Month 元/月
3. About 或相当于工资的 _____ (**GA006_W4_2c**) % of wage
4. No 没有

997. Do not know 不知道
999. Refuse to answer 拒绝回答

PROCEDURE 程序:

If GA006_W4_2 = 1, but GA006_W4_2a = 0 / missing, ask the unfolding bracket questions 如果 GA006_W4_2 = 1, 但 GA006_W4_2a = 0 或缺失, 那么分级展开提问
If GA006_W4_2 = 2, but GA006_W4_2b = 0 / missing, ask the unfolding bracket questions 如果 GA006_W4_2 = 2, 但 GA006_W4_2b = 0 或缺失, 那么分级展开提问
If GA006_W4_2 = 3, but GA006_W4_2c = 0 / missing, ask the unfolding bracket questions 如果 GA006_W4_2 = 3, 但 GA006_W4_2c = 0 或缺失, 那么分级展开提问
If GA006_W4_2 = 997, ask the unfolding questions 如果 GA006_W4_2 = 997, 那么分级展开提问

GA006_W4_2_bracket What is the total amount of [preload other household member's name]'s insurance, income tax, public housing funds and other fees? 个人所得税、各类保险、住房公积金或其他杂费一共是多少元/月?
300/500/1,000/2,000/3,000 Yuan/Month 元/月

GA007_W4 Did [preload other household member's name] receive any of the following types of individual transfer income in the past year? (check all that apply) 过去一年, [加载其他家户成员名字] 有没有领到下列转移支付收入? (可多选)

1. Pensions (including wages from governments, public institutions and firms, supplemental pension of the firms,and income from such programs as rural pension insurance, Urban residents' pension and commercial pension insurance, new rural social pension insurance) 退休金或养老金, 包括政府机关和事业单位退休金, 企业职工基本养老保险, 企业补充养老保险, 农村/城乡/城镇居民养老保险, 商业养老保险, 人寿保险, 征地养老保险等, 领了 _____ (**GA007_W4_1**) Yuan 元
   [IWER: If R is unwilling to answer or does not remember, ask unfolding bracket questions here 访员注意: 如果受访者忘记了, 展开提问]
2. Unemployment compensation 失业补助, 领了 _____ (**GA007_W4_2**) Yuan 元
3. Pension voucher 养老卡/券, 领了 _____ (**GA007_W4_3**) Yuan 元
4. Pension subsidy for the oldest old 高龄老人养老补助, 领了 _____ (**GA007_W4_4**) Yuan 元

5. Workers' compensation from Industrial Accident Compensation Insurance includes wage-replacement benefits, disability benefits, and survivors' benefits 工伤保险金包括误工补贴、伤残补助等，领了 _____ （**GA007_W4_5**）Yuan 元
6. Elderly family planning subsidies 独生子女老年补助, 领了 _____（**GA007_W4_6**）Yuan 元
7. Medical aid 医疗救助，领了 _____ （**GA007_W4_7**）Yuan 元
   [F1: Medical Aid: Medical Aid for Serious Diseases refers to additional cash assistance outside of Medical Insurance Scope for urban and rural residents who are living difficultly caused by a major illness. Medical Aid play as final support outside the basic medical insurance F1：医疗救助：重特大疾病的医疗救助是指给予患有重大疾病并造成医疗和家庭生活困难的城乡居民，在医疗保险之外一定金额的现金救助。医疗救助主要是发挥在基本医疗保险以外的兜底作用]
8. Other government subsidies 政府给个人的其他补助，please specify 请注明 _____ (**GA007_W4_8_1**)，领了 _____ （**GA007_W4_8**）Yuan 元
9. Other social income sources 社会给个人的其他转移支付收入，please specify 请注明 _____ （**GA007_W4_9_1**），领了 _____ （**GA007_W4_9**）Yuan 元
10. None of the above 以上均没有 → Skip to GA012_W4_1 跳至 GA012_W4_1

PROCEDURE 程序:
If GA007_W4 = 1 and GA007_W4_1 = 0 / missing, which means respondent has received pension but did not answer how much received, or the input value is 0 Yuan, ask GA008_W4_1_bracket 如果 GA007_W4 = 1 且 GA007_W4_1 = 0 或缺失，即领取了退休金或养老金，但是没回答多少钱或者填了 0 元，询问 GA008_W4_1_bracket

GA008_W4_1_bracket How much pension have [preload other household member's name] received last year? 过去一年，退休金/养老金一共领了多少钱?
400/600/1,000/3,000/20,000 Yuan/Year 元/年

GA012_W4_1 Have [preload other household member's name] ever inherited anything? [加载其他家户成员名字] 是否继承过遗产?
1. Yes 是，几次? _____ (**GA012_W4_1_1**) times 次
2. No 否 → Skip to next household member 跳至下一个家户成员

997. Do not know 不知道 → Skip to next household member 跳至下一个家户成员
999. Refuse to answer 拒绝回答 → Skip to next household member 跳至下一个家户成员

PROCEDURE 程序:
If GA012_W4_1 = 1 and GA012_W4_1_1 = 1, ask GA012_W4_2 and GA012_W4_3 如果 GA012_W4_1 = 1 且 GA012_W4_1_1 = 1，询问 GA012_W4_2 和 GA012_W4_3

GA012_W4_2 How much in total have [preload other household member's name] inherited? The original value when inherited was [加载其他家户成员名字] 继承的遗产值多少钱? 按继承时的价值来算 _____ (**GA012_W4_2_1**) Yuan 元，When did the inheritance occur? 哪一年继承的遗产? _____ (**GA012_W4_2_2**) Year 年
[Soft Check: GA012_W4_2_1 > 200,000]

GA012_W4_3 From whom [preload other household member's name] inherited? [加载其他家户成员名字] 从谁那里继承了遗产?

1. Parents of [preload other household member's name] [加载其他家户成员名字] 的父母
2. Parents-in-law of [preload other household member's name] [加载其他家户成员名字] 的岳父母
3. Children of [preload other household member's name] [加载其他家户成员名字] 的子女
4. Relatives of [preload other household member's name] [加载其他家户成员名字] 的亲戚
5. Others 其它

997. Do not Know 不知道

PROCEDURE 程序:
If GA012_W4_1 = 1 and GA012_W4_1_1 > 1 or missing, ask GA012_W4_4 and GA012_W4_5
如果 GA012_W4_1 = 1 且 GA012_W4_1_1 > 1 或者缺失，询问 GA012_W4_4 和 GA012_W4_5

GA012_W4_4 How much in total have [preload other household member's name] inherited? The original value when inherited was [加载其他家户成员名字] 继承的遗产总共值多少钱? 按继承时的价值来算 _____ (**GA012_W4_4_1**) Yuan 元
The amount for largest inheritance was 继承的最大的一笔遗产值多少钱?_____ (**GA012_W4_4_2**) Yuan 元
When did the largest inheritance occur? 继承数目最大那次是哪一年?_____ (**GA012_W4_4_3**) Year 年
[Soft Check: GA012_W4_4_2 > 200, 000]

GA012_W4_5 The largest inheritance of [preload other household member's name] was inerited from whom? 继承数目最大的那次，[加载其他家户成员名字] 从谁那里继承了遗产?

1. Parents of [preload other household member's name] [加载其他家户成员名字] 的父母
2. Parents-in-law of [preload other household member's name] [加载其他家户成员名字] 的岳父母
3. Children of [preload other household member's name] [加载其他家户成员名字] 的子女
4. Relatives of [preload other household member's name] [加载其他家户成员名字] 的亲戚
5. Others 其它

997. Do not know 不知道

PART 2 Household Agricultural Income and Expenditure 家户农业收入与支出

[INTRO: Next we will ask some questions about agricultural income and expenditure in your household 下面，我们将问一些有关您家农业收入与支出的问题]

GB001_W4 In the past year, did your household members, including you, your spouse, and other household members, engage in agricultural activities (including cropping, forestry, livestock,and fish), or selling agricultural products you produced at market? 过去一年, 您家的家户成员, 包括您、您的配偶和其他家户成员, 有没有从事种地、管理果树、采集农林产品、养鱼、打鱼、养牲畜等农业活动，或者去市场销售自家生产的农产品?

1. Yes 有
2. No 没有 → Skip to GC001 跳至 GC001

GB002_W4 Who engaged in agricultural work in the past year? (check all that apply) 过去一年，从事这些农业活动的家户成员有哪些？(可多选)

[preload all household member name, include the MainR and Spouse] [加载家户成员名单, 包括主要受访者和配偶]

Crops and Forestry Products 农林产品

GB003 Did your household engage in cropping or forestry last year? 您家过去一年是否从事了种植业或林业，包括种植花木、蔬菜和各类农作物，种植蘑菇、木耳等林下作物，种植茶叶?

1. Yes 是
2. No 否 → Skip to GB007 跳至 GB007

GB004 When was the most recent harvest? 最近的一次收成是在什么时候? _____ 2009...2018 (**GB004_1**) Year 年 _____ 0...12 (**GB004_2**) Month 月

[IWER: Mark the year using four digits. Take down the month as its actual number. For example, write January as "1" not "01", December as "12". If do not remember month, fill "0" 访员注意: 用 4 位数表示年，按照实际的月份填写月。例：1 月写作“1”，而不是“01”，12 月写作“12”。如果记不住月份，请填入“0”]

GB005 What is the total value of all crops and forestry products produced in the past year? 过去一年, 您家生产的农产品和林产品加起来一共值多少钱? _____ (**GB005_1**) Yuan 元, Among it, what is the value of the crops and forestry products that is home consumed? 其中, 自家消费的值多少钱? _____ (**GB005_2**) Yuan 元 or percentage 或占多少百分比? _____ (**GB005_3**) %

[Soft Check: GB005_1 > 75000]

[IWER: If R is unwilling to answer or does not remember, ask unfolding bracket questions 访员注意：如果受访者不愿回答或者忘记了，展开提问]

GB005_bracket [CAPI: If Respondent is unwilling to answer/does not remember, or the input value is 0 for GB005_1, please ask unfolding bracket questions here 对于问题 GB005_1，如果受访者不愿回答或者忘记了或者填了 0 元，在此处分级展开提问] What is the total value of all crops and forestry products? 加起来一共值多少钱?

1,000/3,000/5,000/7,000/10,000 Yuan 元

GB005_W2_bracket [CAPI：If Respondent is unwilling to answer/does not remember, or the input value is 0 for GB005_2 and GB005_3, please ask unfolding bracket questions here 对于问题 GB005_2 和 GB005_3, 如果受访者都不愿回答或者忘记了或者填了 0 元，在此处分级展开提问] What is the total value of all crops and forestry products for household consumption? 自家消费的值多少钱?
1,000/3,000/5,000/7,000/10,000 Yuan 元

GB006 What was the total cost of producing crops (including vegetables and Chinese herbs) and forestry products in the past year? (including Seeds (including home-used seeds), Fertilizer, Organic fertilizer, Pesticide, Plastic sheets, Hiring labor (including with machine or animals), Land rents, Rents (excluding land rents), Irrigation, Fuel, Transportation, Processing, Marketing (including packaging, management fee)) 过去一年，您家为了农业生产和林业生产, 总共投入了多少钱? 投入包括种子（含自家留种的价值）、化肥、农家肥、农药、塑料薄膜、雇工费（包括其使用的机器和役畜）、土地租金、除地租以外的其他租金（例如：租用收割机）、灌溉费、燃料、运输费、加工费、市场费用（包括包装费, 管理费等）_____ Yuan 元

[soft check: 50,000 yuan]
[IWER: if R is unwilling to answer or does not remember, ask unfolding bracket questions 访员注意：如果受访者不愿回答或者忘记了，展开提问]

GB006_bracket [CAPI：If Respondent is unwilling to answer, does not remember, or the input value is 0, please ask unfolding bracket questions here 如果受访者不愿回答或者忘记了或者填了 0 元，在此处分级展开提问:] 300/600/1,000/2000/5,000 Yuan 元

Livestock and Fisheries 牲畜和水产品

GB007 Has your family raised any livestock or fish in the past year? (including chicken, duck, cattle, pig, sheep, etc.) 您家过去一年养过牲畜或者水产品吗？牲畜包括家禽、家畜如鸡、鸭、牛、猪、羊等都算

1. Yes 是
2. No 否 → Skip to GC001 跳至 GC001

GB008 What is the current value of all livestock (including chicken, duck, cattle, pig, sheep, etc.) and aquatic life? 现在，您家所有的家禽、家畜和水产品加起来一共值多少钱？家禽、家畜包括鸡、鸭、牛、猪、羊等，水产品包括鱼等 _____ Yuan 元
[Soft Check: $> 100,000$]
[IWER: If R is unwilling to answer or does not remember, ask unfolding bracket questions 访员注意：如果受访者不愿回答或者忘记了，展开提问]

GB008_bracket [CAPI: If Respondent is unwilling to answer, does not remember, or the input value is 0, please ask unfolding bracket questions here 如果受访者不愿回答或者忘记了或者填了 0 元，在此处分级展开提问] 500/1,500/3,000 /4,500 /9,000 Yuan 元

GB009 What were the value of all livestock and aquatic life you have at this time last year? 去年这个时候，您家拥有的牲畜及水产品一共值多少钱？ _____ Yuan 元
[Soft Check: > 100, 000]
[IWER: If R is unwilling to answer or does not remember, ask unfolding bracket questions 访员注意：如果受访者不愿回答或者忘记了，展开提问]

GB009_bracket [CAPI: If Respondent is unwilling to answer, does not remember, or the input value is 0, please ask unfolding bracket questions here 如果受访者不愿回答或者忘记了或者填了 0 元，在此处分级展开提问] 500 /1,500 /2,500 /4,000 /8,000 Yuan 元

GB010 How much did you spend on purchasing new livestock and aquatic life in the past year? 过去一年，你们家买牲畜及水产品一共花了多少钱？ _____ Yuan 元
[Soft Check: > 50, 000]

GB011 What was the value of all livestock and aquatic life that were sold or consumed in the past year? 过去一年，你们家卖出去的和自家消费的牲畜及水产品加起来一共值多少钱？不包括副产品 _____ (**GB011_1**) Yuan 元，Among it, what is the amount or percent consumed at your home? 其中，自家消费的值多少钱？ _____ (**GB011_2**) Yuan 元或占多少百分比？ _____ (**GB011_3**) %
[Soft Check: > 100, 000]
[IWER: If R is unwilling to answer or does not remember, ask unfolding bracket questions 访员注意：如果受访者不愿回答或者忘记了，展开提问]

GB011_bracket [CAPI: For GB011_1, if Respondent is unwilling to answer, does not remember, or the input value is 0, please ask unfolding bracket questions here 对于问题 GB011_1，如果受访者不愿回答或者忘记了或者填了 0 元，在此处分级展开提问] What was the value of all livestock and aquatic life? 加起来一共值多少钱？
200 /900 /1,500 /2,500 /5,000 Yuan 元

GB011_w2_bracket [CAPI: For GB011_2 and GB011_3, if Respondent is unwilling to answer, does not remember, or the input value is 0, please ask unfolding bracket questions here 对于问题 GB011_2 和 GB011_3, 如果受访者都不愿回答或者忘记了或者填了 0 元, 在此处分级展开提问] What is the total value of all livestock and aquatic life for household consumption 其中，自家消费的值多少钱？
200 /900 /1,500 /2,500 /5,000 Yuan 元

GB012 What was the value of all livestock byproducts produced (including the self consumption value) in the past year, including milk, wool (including cashmere, sheep or goat skin), and eggs? 过去一年，你们家养的牲畜生产出来的副产品，比如鸡蛋、牛奶、羊毛、羊绒、羊皮，加起来一共值多少钱，包括所有卖出去的和自家消费的？ _____ (**GB012_1**) Yuan 元，Among it, what is the amount or percent consumed at your home? 其中，自家消费的值多少钱？ _____ (**GB012_2**) Yuan 元或占多少百分比？ _____ (**GB012_3**) %
[Soft Check: > 50, 000]
[IWER: If R is unwilling to answer or does not remember, ask unfolding bracket questions 访员注意：如果受访者不愿回答或者忘记了，展开提问]

GB012_bracket [CAPI: For GB012_1, if Respondent is unwilling to answer, does not remember, or the input value is 0, please ask unfolding bracket questions here 对于问题 GB012_1，如果受访者不愿回答或者忘记了或者填了 0 元，在此处分级展开提问] What was the value of all livestock byproducts produced (including the self consumption value) in the past year? 一共值多少钱，包括所有卖出去的和自家消费的？
100 /200 /300 /500 /1,000 Yuan 元

GB012_w2_bracket [CAPI: For GB012_2 and GB012_3, If Respondent is unwilling to answer, does not remember, or the input value is 0, please ask unfolding bracket questions here 对于问题 GB012_2 和 GB012_3，如果受访者都不愿回答或者忘记了或者填了 0 元，在此处分级展开提问] What was the total value for household consumption? 其中，自家消费的值多少钱？
100 /200 /300 /500 /1,000 Yuan 元

GB013 In the past year, how much did you spend on raising these livestock and aquatic products, including feeding fees, medical expenses, grazing fees, barn fence fees, and labor costs, etc.? 过去一年，养这些牲畜及水产品一共花了多少钱，包括喂养费、医药费、放牧费、畜舍栅栏费、雇工费等？ _____ Yuan 元
[Soft Check: $> 50,000$]

PART 3 Self-Employed Activities 个体经营或开办私营企业

GC001 Did your household members engage in any self-employed activities last year? 过去一年，您家是否有家户成员从事某些个体经营或开办私营企业？
1. Yes 是
2. No 否 → Skip to GD001_W4_1 跳至 GD001_W4_1

GC002 How many types of activities did your household members participate in the past year? 过去一年，您家家户成员从事几项个体经营活动或开办几家私营企业？ _____

PROCEDURE 程序：
Ask all these GC002 activities in GC006_W4 在 GC006_W4 中询问这 GC002 项活动

GC006_W4 Please briefly describe the self employed activities that your household member engaged, such as location/business type, etc. 请简要描述一下这几项个体经营活动和私营企业？可以是个体经营的地点，经营的项目等
1. _____ **(GC006_W4_1)**
2. _____ **(GC006_W4_2)**
3. _____ **(GC006_W4_3)**
...
GC002 _____ **(GC006_W4_i)**

PROCEDURE 程序:
Please ask GC003 - GC005 for all self employed activities mentioned above. The description loaded from GC006_W4 针对每一项个体经营活动或私营企业，询问 GC003 - GC005。其中个体经营活动描述的加载来自 GC006_W4

GC003 Who engaged in this self-employment businesse [Preload Self Employeed Activity *i*] in the past year? (check all that apply) 过去一年，从事 [加载第 *i* 项个体经营的描述] 这项个体经营或私营企业的家户成员有哪些？(可多选)
[Preload the list of all household members 加载所有家户成员名单]

GC004 [Preload Self Employeed Activity *i*] which types of activities? [加载第 *i* 项个体经营的描述] 这项活动属于什么行业？

1. Services (cooking, sewing, private clinic etc.) 服务业（烹饪、缝纫、私人诊所等）
2. Transportation 交通运输
3. Construction 建筑业
4. Mining 采掘
5. Processing production 加工生产业
6. Business 商业
7. Others 其他，please specify 请注明 _____ **(GC004_1)**

GC005 Not including fixed capital costs, what is your best estimate of the net income earned from this activity [Preload Self Employeed Activity *i*] by your household members last year? If the activity was conducted jointly with non-household members, report only the net income earned by household members. Remember to consider the following types of costs: energy, housing or equipment rental, raw materials, transportation, marketing, wages, taxes or fees 不包括固定资本成本，您能否精确估计一下您家过去一年从 [加载第 *i* 项个体经营的描述] 这项活动净赚多少钱？如果此项经营活动有非家户成员参与，只计算家户成员的净收入。别忘了考虑下列各种成本：能源、住房和设备租用费、原材料、交通费、营销、工资、税收和杂费 _____ Yuan 元
[Soft Check: $> 500,000$]
[IWER: If R is unwilling to answer or does not remember, ask unfolding bracket questions 访员注意：如果受访者不愿回答或者忘记了，展开提问]

GC005_bracket [CAPI: If Respondent is unwilling to answer, does not remember, or the input value is 0, please ask unfolding bracket questions here 如果受访者不愿回答或者忘记了或者填了 0 元，在此处分级展开提问] 5,000 /10,000 /50,000 /100,000 /200,000 Yuan 元

PART 4 Household Public Transfer Income 家户公共转移支付收入

[INTRO: We ask the public transfers received by the households (with household as the unit). Public transfers have characteristic of welfare, such as Wubaohu Subsidy and Tekunhu Subsidy gived by government. 这部分询问家户从公共机构所得的各种转移支付收入。公共转移支付具有福利支出的性质，如政府给五保户和特困户的补助金等。这里询问的是以家户为单位获得的转移支付]

GD001_W4_1 Does your household receive Dibao assistance? 您家是低保户吗?

1. Yes 是
2. No 不是 → Skip to GD003_W4_1 跳至 GD003_W4_1

999. Refuse to answer 拒绝回答 → Skip to GD003_W4_1 跳至 GD003_W4_1

GD001_W4_2 When did your household become a Dibaohu? 哪一年成为低保户? _____ Year 年

GD001_W4_3 How much did you receive in the past year? 过去一年，您家领了多少低保户补助? _____ Yuan 元

GD003_W4_1 Is your household a registered Pinkunhu? 您家是建档立卡贫困户吗?

1. Yes 是
2. No 不是 → Skip to GD002_W4 跳至 GD002_W4

997. Do not know 不知道 → Skip to GD002_W4 跳至 GD002_W4
999. Refuse to answer 拒绝回答 → Skip to GD002_W4 跳至 GD002_W4

GD003_W4_2 When did you become a registered Pinkunhu? 哪一年成为建档立卡贫困户? _____ Year 年

GD002_W4 Did your household receive any of the following government subsidies in the past year? (check all that apply) 您家过去一年有没有收到下列政府补助，社会捐助或者补偿?（可多选）

1. Reforestation 退耕还林: how much? 有多少? _____ (**GD002_W4_1**) Yuan 元
2. Agricultural subsidies 农业补助: how much? 有多少? _____ (**GD002_W4_2**) Yuan 元
3. Wubaohu (targets low-income, blind, disabled, aged persons, and young persons that have no means to support themselves) 五保户补助金，用来补助那些无法自力更生的低收入者、盲人、身体残疾者、老人和小孩: how much? 有多少? _____ (**GD002_W4_3**) Yuan 元
4. Pinkunhu subsidy, including registered Pinkunhu and non-registered Pinkunhu 贫困户补助，包括建档立卡和非建档立卡贫困户补助: how much? 有多少? _____ (**GD002_W4_4**) Yuan 元
5. Work injury subsidies to the immediate family members 工伤人员供养直系亲属抚恤金 how much? 有多少? _____ (**GD002_W4_5**) Yuan 元
6. Emergency or disaster relief (jiujikuan, jiuzaikuan) last year? 重大灾害后，政府的捐助和补助，包括救济金、赈灾款等: how much? 加上实物救助的价值一共有多少? _____ (**GD002_W4_6**) Yuan 元
7. Social donations and subsidies, including food, clothes, school bags, crowdfunding medical expenses, and physical assistance 社会捐助和补助，包括食品、衣服，书包和众筹医疗费等: how much? 加上实物救助的价值一共有多少? _____ (**GD002_W4_7**) Yuan 元
8. Compensation for land seizure last? 征地补偿金: how much? 有多少? _____ (**GD002_W4_8**) Yuan 元

9. Compensation to pulling down your house or apartment last year? 住房拆迁补偿:how much? 有多少? _____(**GD002_W4_9**) Yuan 元
10. Other 其他补助, please specify 请注明_____(**GD002_W4_other**): how much? 有多少? _____ (**GD002_W4_10**) Yuan 元
11. None 没有收到任何政府社会的补助和捐助 → Skip to GD004_W4 跳至 GD004_W4

[Soft Check: > 20,000 for each item 对每项进行检查]

GD004_W4 Does your household receive any claims from Productive insurance in past year? How much? (input 0 if not received). Productive Insurance include Agricultural insurance claims, etc. 您家过去一年有没有得到生产性保险赔付？得到多少？（如果没有，填 0）生产性保险赔付包括农业保险赔付等 _____ Yuan 元

PART 5 Household Living Expenditure 家户生活支出

[IWER: Please read aloud "This section is about your household living expenditure, including all your household members [preloaded names of household members] living expenditure" 访员注意：请大声念出"本部分是所有家户成员的生活支出，包括 [加载所有家户成员名单]"]

GE000_W4 Generally, how much does your family spend a month? Including rent, food, clothing, communication expenses, water and electricity costs, fuel costs, service expenditures, entertainment expenditures, daily necessities and medical expenses. 平均而言，您家一个月花费多少钱？包括房租、食物、衣服、通讯支、水电费、燃料费、服务支出、娱乐支出、日用品和医疗支出等 _____ Yuan 元

GE001 We wish to know your family food expenditure for the last week. Are you the primary person who purchases food for the household? 我们想知道您家最近一周的食品支出，您负责为家里购买食品吗?

1. Yes 是 → Skip to GE004 跳至 GE004
2. No 否

GE002 Who is the primary person purchasing food for the household? 谁负责为您家购买食品?

1-25. Other household member list 其他家户成员列表
26-50. Other children [List all the children who are not in the household member list] 其他子女 [列出所有不在家户成员名单中的子女并从中选取]
51. Nanny 保姆
52. Neighbor 邻居
53. Other 其他
97. Main Respondent 主要受访者
98. Spouse of the main respondent 主要受访者配偶

[IWER: If possible, the primary person who purchases food for the household should answer the questions about expenditures GE004 - GE008 访员注意：如果可能的话，由负责为该家庭购买食品的人来回答 GE004 - GE008]

GE004 In the past week, how many people usually ate meals together in your household (not including guests)? 最近一周，不包括客人, 您家里一般有几口人吃饭? _____ Persons 人
[Soft Check: > 10]

GE005 Last week how many meals did you provide to guests? 最近一周，您家的客人在您家吃了几顿饭，按人次计算? _____ Meals 人次?
[Soft Check: > 100]

[INTRO: The next questions are about your household living expenditure for all household members, including the expenditure happened outside, such as accommodation and meals fees 引语：下面问题是关于您家的生活支出的。这些支出包括所有家户成员的生活支出。只要是家户成员，其在外的支出也须包括在内，如住宿费和伙食费等]

GE006_W4 In the past week, how much did your household spend on food (excluding eating out expenditure, alcohol, cigarettes, cigars and tobacco expenditure)? 最近一周，您家花了多少钱购买食品，不包括摆酒、办酒席、外出就餐、购买香烟、酒水等? _____ Yuan 元
[Soft Check: > 6,000]

GE006_W2 Does your household produce agricultural products yourself (including plants, meat, eggs, aquatic lives, oil, vegetables and fruits, cigaretts and wine, drinks and milk products, produced food, seasonings, etc.)? 您家自己生产农产品吗, 包括粮食作物、肉类、蛋类、水产品、油、蔬菜水果、烟酒、饮料及乳制品、加工食品、调料等?

1. Yes 生产
2. No 不生产 → Skip to GE007_W4 跳至 GE007_W4

GE006_W2_1 In the past week, what was the market value of the food that members of the household consumed that you grew yourselves? 最近一周，您家里人消费的自家生产的农产品在市场上卖的话值多少钱? _____ Yuan 元

GE007_W4 Among it, how much did your household spend on eating out? 最近一周，您家花了多少钱外出就餐，不包括摆酒和办酒席? _____ Yuan 元
[Soft Check: > 3,000]

GE008 Among it, how much did your household spend on alcohol, Cigarettes, cigars and tobacco? 最近一周，您家花了多少钱购买香烟、酒水等? _____ Yuan 元
[Soft Check: > 3,000]

GE009 Please tell me the expenditure on the following items last month for your household 下面我们想了解您家过去一个月在以下各项消费中的支出。
[IWER: Fill in 0 if no corresponding expenditure; missing if the respondent cannot recall the expenditure 访员注意: 没有相应项支出用 0 元表示，记不清则请受访者估计一个数字，如果实在无法估计，该支出用缺失表示]

1. Communication fees, including post, internet usage, telephone and cell phone usage 邮电、通讯支出，包括电话、手机、上网、邮寄等 _____ (**GE009_1**) Yuan 元

2. Utilities: Water and electricity 水费、电费_____ (**GE009_2**) Yuan 元
3. Fuels, including gas, coal, etc. 燃料费，包括煤炭、煤制品、柴草、木炭、天然气、液化气等 _____ (**GE009_3**) Yuan 元
4. Expenses for babysitters, housekeepers and servants 保姆、小时工、佣人等的支出 _____ (**GE009_4**) Yuan 元
5. Local Transportation 在当地的交通费 _____ (**GE009_5**) Yuan 元
6. Daily necessities, including toiletries, household items, kitchen supplies, decorative items, etc. 日用品，包括洗漱用品、家居用品、厨卫用品、装饰用品等 _____ (**GE009_6**) Yuan 元
7. Entertainment, including expenses for books, newspapers, VCCs, DVDs, cinema tickets and bars 文化娱乐支出, 包括书报杂志、光盘、影剧票、歌舞厅和网吧 _____ (**GE009_7**) Yuan 元

[Soft Check: > 5,000 for each category]

PROCEDURE 程序:
If GE009 = 4, babysisters, housekeepers and servants, ask GE009_W4_4 如果 GE009 = 4, 即家里雇佣保姆/小时工/佣人，则询问 GE009_W4_4
Otherwise, Skip to GE010 否则跳至 GE010

GE009_W4_4 Will the babysitters/hourly worker/servant employed by your household take care of the following people? (check all that apply) 您家雇佣的保姆/小时工/佣人会照顾以下人员吗?（可多选）

1. The elders in the household 家户里的老人
2. the children in the household 家户里的小孩
3. Others 其他，please specify 请注明 _____ (**GE009_W4_4_1**)

GE010 In the last year how much did your household spend on the following items? 下面我们想了解您家过去一年在以下各项消费中的支出。

[IWER: Fill in 0 if no corresponding expenditure; fill in missing if the respondent cannot recall the expenditure 访员注意：没有相应项支出用 0 元表示，记不清则请受访者估计一个数字，如果实在无法估计，该支出用缺失表示]

1. Clothing and bedding 衣着消费 _____ (**GE010_1**) Yuan 元
2. Long distance traveling expenses 家庭的旅游支出 _____ (**GE010_2**) Yuan 元
3. Heating(centrally heated) 家庭的取暖费支出，指集中供暖 _____ (**GE010_3**) Yuan 元
4. Furniture, consumption of durable goods and electronics, includes refrigerator, washing machine, TV, computers and expensive instruments like pianio. 家具、耐用消费品及电器的支出,包括电冰箱、洗衣机、电视、电脑和高档乐器如钢琴等 _____ (**GE010_4**) Yuan 元
5. Education and training(including tuition, training fees, etc.) 教育和培训支出，包括学杂费、培训费等 _____ (**GE010_5**) Yuan 元
6. Direct and Indirect Medical expenses. Note: Indirect medical expenses refer to transportation expenses, nutrition expenses, family expenses, etc. incurred due

to medical treatment. It does not include the part already paid by Medicare 医疗支出，包括直接或间接。注：间接医疗支出，指因为医疗而产生的交通费、营养费、家人陪护花费等。不包括医保已经赔付部分。 _____ **(GE010_6)** Yuan 元

7. Fitness expenditures 保健费用，包括健身锻炼及产品器械、保健品等 _____ **(GE010_7)** Yuan 元
8. Beauty (including make-ups, facials, massages, etc.) 美容支出，包括化妆品、美容护理、按摩等 _____ **(GE010_8)** Yuan 元
9. Automobiles 购买汽车 _____ **(GE010_9)** Yuan 元
10. Purchase, Maintenance and repair (of transportation vehicles, appliances, communication products, etc.) 各种交通工具，如自行车、电动自行车等（不包括汽车），和通讯工具，如电话、手机等，的购买、维修及配件费用 _____ **(GE010_10)** Yuan 元
11. Property management fees (including parking fee) 物业费,包括车位费 _____ **(GE010_11)** Yuan 元
12. Taxes and fees turned over to the government 上交给政府相关部门的税费和杂费，不包括所得税 _____ **(GE010_12)** Yuan 元
13. Donations to the society (including cash, and items like food, clothing, etc.) 社会捐助支出，包括现金，食品、衣服等 _____ **(GE010_13)** Yuan 元
14. The rent of the house or bed, including the accommodation expenses for household members, such as the campus dormitory fee, excluding the hotel fee for tourism 房子或者床位的租金,包括家户成员在外的住宿费,如住校宿舍费,不包括旅游的宾馆费 _____ **(GE010_W4_14)** Yuan 元
15. Expenses for setting and holding banquets 摆酒和办酒席的支出 _____ **(GE010_W4_15)** Yuan 元

[Soft Check: $>100,000$ for each item]

GE011 How often did the respondent receive assistance in answering section Household income and expenditure? 受访者填写该部分问卷时是否求助?

[IWER: If it is answered by a proxy, please record the respondent's reaction 访员注意：如果是协助回答，请记录受访者的反应]

1. Never 从未
2. A few times 偶尔几次
3. Most or all of the time 大多数时间

HA Household Assets 家户资产

[IWER: This section is asked of the family financial respondent. Do not allow a proxy respondent to answer the entire section 访员注意：这部分要问家庭财务受访者，不允许完全请别人代答。仅计算主要受访者及其配偶的资产。如果资产为多人所有，仅计算主要受访者和配偶所有部分]

PART 1 Land 土地

[INTRO: The following questions pertain to your land 下面是关于土地的问题]

HA054 Does [preload main respondent's name] and [preload main respondent's spouse's name] (if any) have any collective distributing or rent cultivated land, forest land, pasture and/or pond? (Choose all that apply) [加载主要受访者名字] 和 [加载主要受访者配偶的名字]（如果有的话）是否有集体分配的耕地、林地、牧场或水塘，或者从别人那里租用了耕地、林地、牧场或水塘？（可多选）

1. Cultivated land 耕地
2. Forest land 林地
3. Pasture 牧场
4. Pond 水塘
5. None 没有 → Skip to HA064 跳至HA064

**PROCEDURE** 程序: According to all options choosed in HA054, ask HA070_W4 - HA063 in loop 针对 HA054 的所有选择，循环询问 HA070_W4 - HA063

HA070_W4 Have the right of [Load the chosed option in HA054] been confirmed by government? [加载 HA054 的答案] 有没有进行确权？

1. Yes 有
2. No 没有 → Skip to HA055 跳至 HA055

997. Do not Know 不知道 → Skip to HA055 跳至 HA055
999. Refuse to answer 拒绝回答 → Skip to HA055 跳至 HA055

HA071_W4 When the right of [Load the chosed option in HA054] been confirmed? 哪一年对 [加载 HA054 的答案] 进行确权？ ______ Year 年

HA055 How many mu of [preload answer from HA054] do you and your spouse have? [加载主要受访者名字] 和 [加载主要受访者配偶的名字]（如果有的话）从集体分配到的 [加载 HA054 的答案] 有多少亩？ ______ Mu 亩

[IWER: Only those assigned to the Main Respondent and the spouse are recorded here, and those assigned to other household members should not be included 访员注意：这里只记录受访者和其配偶分配到的，其他家户成员分配到的要剥离出去]

[Soft Check: > 50]

HA056 How many mu of them are irrigable? 其中可灌溉面积是多少亩？ ______ Mu 亩

[Hard Check: > HA055]

HA057 What is the rent per mu per year you would get if you rent out all your [preload answer from HA054]? 如果出租的话，每亩 [加载 HA054 的答案] 每年的租金会是多少？ ______ Yuan per mu per year 元每年每亩

[Soft Check: < 10, > 4000]

[IWER: If R is unwilling to answer or does not remember, ask unfolding bracket questions 访员注意：如果受访者不愿回答或者忘记了，展开提问]

HA057_W4_bracket [CAPI: If Respondent is unwilling to answer or does not remember, please ask unfolding bracket questions here 如果受访者不愿回答或者忘记了，在此处分级展开提问。注意，这里填 0 不需要展开] 50/100/300/600/1,000 yuan 元

**PROCEDURE** 程序:<br>If HA057 = 0, ask HA057_W4_1 如果 HA057 = 0，则询问 HA057_W4_1<br>Otherwise, Skip to HA058 否则跳至 HA058

HA057_W4_1 Why the rent is 0? 租金为 0 的原因是什么?

1. The profit is low 土地收益不高
2. No one rents 没人租
3. Other 其他，please specify 请注明 _____ (**HA057_W4_2**)

HA058 Do [preload main respondent's name] and [preload main respondent's spouse's name] (if any) rent out any of [preload answer from HA054] in the past year? 过去一年 [加载主要受访者名字] 和 [加载主要受访者配偶的名字]（如果有的话）是否将 [加载 HA054 的答案] 出租给了其他人?

1. Yes 是
2. No 否 → Skip to HA061 跳至 HA061

HA059 How much mu [preload answer from HA054] did [preload main respondent's name] and [preload main respondent's spouse's name] (if any) rent out the past year? 过去一年 [加载主要受访者名字] 和 [加载主要受访者配偶的名字]（如果有的话）出租了多少亩 [加载 HA054 的答案]? _____ Mu 亩

[Soft Check: > HA055]

HA060 How much rental income did [preload main respondent's name] and [preload main respondent's spouse's name] (if any) earn from [preload answer from HA054] in the past year? 过去一年 [加载主要受访者名字] 和 [加载主要受访者配偶的名字]（如果有的话）出租 [加载 HA054 的答案] 收到的租金是多少? _____ Yuan 元

HA061 Did [preload main respondent's name] and [preload main respondent's spouse's name] (if any) rent in any [preload answer from HA054] from others (including the collective) in the past year? 过去一年 [加载主要受访者名字] 和 [加载主要受访者配偶的名字]（如果有的话）是否从别人，包括集体，那租用了 [加载 HA054 的答案]?

1. Yes 是
2. No 否 → Skip to HA064 跳至 HA064

HA062 How much mu [preload answer from HA054] did [preload main respondent's name] and [preload main respondent's spouse's name] (if any) rent at the past year? 过去一年 [加载主要受访者名字] 和 [加载主要受访者配偶的名字]（如果有的话）租用了多少亩 [加载 HA054 的答案]? _____ Mu 亩

[Soft Check: > 100]

HA063 How much rent did [preload main respondent's name] and [preload main respondent's spouse's name] (if any) pay for [preload answer from HA054]? 过去一年 [加载主要受访者名字] 和 [加载主要受访者配偶的名字]（如果有的话）租用 [加载 HA054 的答案] 付出的租金是多少？ _____ Yuan 元
[Soft Check: $> 20,000$]

HA064 How much rental income did [preload main respondent's name] and [preload main respondent's spouse's name] (if any) earn for any other household assets other than housing or land? (trees, use of fixed capital, durables, or livestock)? 除了出租房屋或者土地，过去一年 [加载主要受访者名字] 和 [加载主要受访者配偶的名字]（如果有的话）从出租其他家庭资产，如树木、固定资本的使用、耐用品或者牲畜，收取了多少租金？

1. Yes 有出租 _____ **(HA064_1)** Yuan 元
2. Not applicable 没有出租

997. Do not know 不知道
999. Refuse to answer 拒绝回答

PART 2 Equipments, Consumption Durables, and Valuables 家用设备、耐用消费品和其他贵重物品

HA065 Do [preload main respondent's name] and [preload main respondent's spouse's name] (if any) own the following assets? (Choose all that apply) Only consider the part that owned if the asset is shared with others [加载主要受访者名字] 和 [加载主要受访者配偶的名字]（如果有的话）有下列物品吗？仅考虑 [加载主要受访者名字] 和 [加载主要受访者配偶的名字]（如果有的话）所拥有那部分产权的价值（可多选）
For each asset owned by main respondent and his/her spouse] what is the asset' s current value? 针对受访者和其配偶有的每一项物品提问：当前值多少？

1. Automobile 汽车 _____ **(HA065_1)**
2. Electric Bicycle 电动自行车 _____ **(HA065_2)**
3. Motorcycle 摩托车 _____ **(HA065_3)**
4. Refrigerator 电冰箱、冰柜 _____ **(HA065_4)**
5. Washing machine 洗衣机 _____ **(HA065_5)**
6. TV 电视机 _____ **(HA065_6)**
7. Computer and pad 电脑和平板 _____ **(HA065_7)**
8. Stereo system 音响 _____ **(HA065_8)**
9. Video camera 摄像机 _____ **(HA065_9)**
10. Camera 照相机 _____ **(HA065_10)**
11. Air conditioner 空调 _____ **(HA065_11)**
12. Mobile phone 手机 _____ **(HA065_12)**
13. Furniture 值钱家具 _____ **(HA065_13)**
14. Music instrument 高档乐器 _____ **(HA065_14)**
15. Valuable decorations, ornaments 昂贵的装饰、物品 _____ **(HA065_15)**
16. Treasures and precious metal (such as gold) 珠宝和贵重金属,如黄金等 _____ **(HA065_16)**

17. Antiques, valuable paintings and calligraphic work, and other artistic work 古董、字画及其他收藏品 _____ **(HA065_17)**
18. Air Purifier 空气净化器 _____ **(HA065_18)**
19. None 没有以上物品

[Soft Check: $< 3,000, > 500,000$ for automobile, and $< 100, > 30,000$ for all other items]

HA066_W4 Do [preload main respondent's name] and [preload main respondent's spouse's name] (if any) own the following fixed capital assets? How much are the assets worth? Only consider the part that owned if the asset is shared with others (check all that apply)[加载主要受访者名字] 和 [加载主要受访者配偶的名字]（如果有的话）有下列农用固定资产吗？现在值多少钱？仅考虑 [加载主要受访者名字] 和 [加载主要受访者配偶的名字]（如果有的话）所拥有那部分产权的价值（可多选）

1. Tractor 拖拉机，current value 现在值 _____ **(HA066_W4_1)** Yuan 元
   [Soft Check: $< 1000, > 30,000$]
2. Thresher 脱粒机，包括打稻机，current value 现在值 _____ **(HA066_W4_2)** Yuan 元
   [Soft Check: $< 100, > 10,000$]
3. Harvester 收割机，current value 现在值 _____ **(HA066_W4_3)** Yuan 元
   [Soft Check: $< 100, > 10,000$]
4. Water pump 抽水机，包括水泵，current value 现在值 _____ **(HA066_W4_4)** Yuan 元
   [Soft Check: $< 100, > 10,000$]
5. Processing equipment 加工机械, current value 现在值 _____ **(HA066_W4_5)** Yuan 元
   [Soft Check: $< 100, > 10,000$]
6. Seeder 播种机，current value 现在值 _____ **(HA066_W4_6)** Yuan 元
   [Soft Check: $< 100, > 10,000$]
7. Agricultural aircraft 农用飞机，current value 现在值 _____ **(HA066_W4_7)** 10,000 Yuan 万元
   [Soft Check: $< 1, > 1,000$]
8. None 没有以上固定资产

HA067_W4 What is the current value of other fixed capital assets for [preload main respondent's name] and [preload main respondent's spouse's name] (if any) used in household production or self-employed activities? Including business property, such as shops, factories and warehouses [加载主要受访者名字] 和 [加载主要受访者配偶的名字]（如果有的话）用于家庭生产、个体经营或开办私营企业的非农用固定资产现在值多少钱？包括经营性房产，如店铺，厂房和仓库。_____ Yuan 元

[IWER: Be sure to ask about fixed capital assets used in all self-employment activities, do not count assets already reported above 访员注意：务必要询问用于所有家庭生产、个体经营或开办私营企业活动的固定资产，不包括前面问过了的固定资产]

HA068 Do [preload main respondent's name] and [preload main respondent's spouse's name] (if any) have any other durable or fixed assets worth 500 yuan or more? 除了刚才提到的耐用品和固定资产，[加载主要受访者名字] 和 [加载主要受访者配偶的名字]（如果有的话）有其他 500 元及以上的其他耐用品或固定资产吗？

1. Yes 有 How much are the assets worth? 现在值多少? _____ (**HA068_1**) Yuan 元
   [Hard Check: > 500] [Soft Check: $> 50,000$]
2. No 没有

997. Do not know 不知道
999. Refuse to answer 拒绝回答

HA076 How often did the respondent receive assistance in answering section Household assets? 受访者填写该部分问卷时是否求助?
[IWER: If it is answered by a proxy, please record the respondent's reaction 访员注意：如果是协助回答，请记录受访者的反应]

1. Never 从未
2. A few times 偶尔几次
3. Most or all of the time 大多数时间

HB Individual Assets 个人资产

[IWER: Please conduct sections HB and HC when the main respondent and his/her spouse are at home. Don't allow a proxy to complete the entire sections 访员注意：对主要受访者和其配偶，分别询问 HC 和 HD 部分问卷。这部分不允许请人完全代填]

PART 1 Financial Assets 金融资产

[INTRO: The following questions pertain to your financial asset 下面是关于金融资产的问题]

[IWER: make sure others are not present, IWER read following instructions: the following questions pertain to your financial asset, the answers to these questions will be kept strictly confidential and will be used for research purposes only 访员注意：请确保没有其他人在场。请调查员念"下面是关于您的金融资产问题。我们不是探究您的隐私，而是用于判断储蓄等金融资产是否影响您的养老决策。我们将会对您的回答严格保密，并且仅用于学术研究"]

HC001 How much cash is held by you and your spouse at home? 您和您爱人现在在家里有多少现金，包括随身携带以及放在家里的? _____ Yuan 元
[Soft Check: $> 50,000, < 100$]
[IWER: If R is unwilling to answer or does not remember, ask unfolding bracket questions 访员注意：如果受访者不愿回答或者忘记了，展开提问]

HC002 [CAPI: If Respondent is unwilling to answer, does not remember, or the input value is 0, please ask unfolding bracket questions here 如果受访者不愿回答或者忘记了或者填了 0 元，在此处分级展开提问] 500 /1,000 /2,000 /5,000 /10,000 Yuan 元

[CAPI: Prompt for HC003_W4 - HC017_W4, add IWER: for electronic money, deposit, bonds, stocks, and funds, only include assets legally in his/her name 对 HC003_W4 - HC017_W4 添加访员注意：对于电子货币，存款、政府债券、股票和基金，只计算法定意义上在受访者名下的财产]

HC003_W4 How much electronic money do you have currently, including the money in the WeChat Wallet and Alipay's balance, etc? Exclude Yu'ebao and other products that generate revenue 您现在有多少电子货币，包括微信钱包以及支付宝余额里的钱等？不包括余额宝等会产生收益的产品 _____ Yuan 元

[Soft Check: $> 1,000$]

[IWER: If R is unwilling to answer or does not remember, ask unfolding bracket questions 访员注意：如果受访者不愿回答或者忘记了，展开提问]

HC004_W4 [CAPI: If Respondent is unwilling to answer, does not remember, or the input value is 0, please ask unfolding bracket questions here 如果受访者不愿回答或者忘记了或者填了 0 元，在此处分级展开提问] 10/50/200/1,000/5,000 Yuan 元

HC005 What is the total amount of deposits you are currently holding in financial institutions (eg: bank, credit union)? 您现在在金融机构，如银行，信用社等，存了多少钱？_____ Yuan 元

[Soft Check: $> 500,000, < 100$]

[IWER: If R is unwilling to answer or does not remember, ask unfolding bracket questions 访员注意：如果受访者不愿回答或者忘记了，展开提问]

HC006 [CAPI: If Respondent is unwilling to answer, does not remember, or the input value is 0, please ask unfolding bracket questions here 如果受访者不愿回答或者忘记了或者填了 0 元，在此处分级展开提问] 2,000/10,000/50,000/100,000/500,000 Yuan 元

HC007 Do you have any government bonds (e.g. Treasury bills, corporate bonds) in your name? 您有（记在您名下的）债券，如国库券，企业债券吗？

1. Yes 有
2. No 没有 → Skip to HC010 跳至 HC010

999. Refuse to answer 拒绝回答 → Skip to HC010 跳至 HC010

HC008 What is the total face value of government bonds (e.g. Treasury bills, corporate bonds) that you are currently holding? 您现有的所有债券，如国库券，企业债券等，总共面值多少？_____ Yuan 元

[Soft Check: $> 50,000$]

[IWER: If R is unwilling to answer or does not remember, ask unfolding bracket questions 访员注意：如果受访者不愿回答或者忘记了，展开提问]

HC009 [CAPI: If Respondent is unwilling to answer, does not remember, or the input value is 0, please ask unfolding bracket questions here 如果受访者不愿回答或者忘记了或者填了 0 元，在此处分级展开提问] 10,000 /50,000 /100,000 /200,000 /500,000 Yuan 元

HC010 Do you hold any stocks (in your name) currently? 您现在持有（记在您名下的）股票吗？

1. Yes 有
2. No 没有 → Skip to HC015 跳至 HC015

999. Refuse to answer 拒绝回答 → Skip to HC015 跳至 HC015

HC013 What is the present market value of all the stocks you are currently holding? 您现在持有的股票当前价值多少? ______ Yuan 元

[Soft Check: $> 200,000$]

[IWER: If R is unwilling to answer or does not remember, ask unfolding bracket questions 访员注意：如果受访者不愿回答或者忘记了，展开提问]

HC014 [CAPI: If Respondent is unwilling to answer, does not remember, or the input value is 0, please ask unfolding bracket questions here 如果受访者不愿回答或者忘记了或者填了 0 元，在此处分级展开提问] 10,000 /50,000 /100,000 /200,000 /500,000 Yuan 元

HC015 Do you hold any funds in your name currently? 您现在持有（记在您名下的）基金吗?

1. Yes 有
2. No 没有 → Skip to HC016_W4 跳至 HC016_W4

999. Refuse to answer 拒绝回答 → Skip to HC016_W4 跳至 HC016_W4

HC018 What is the present market value of all the mutual funds you are currently holding? 您现在持有的基金当前价值多少? ______ Yuan 元

[Soft Check: $> 200,000$]

[IWER: If R is unwilling to answer or does not remember, ask unfolding bracket questions 访员注意：如果受访者不愿回答或者忘记了，展开提问]

HC019 [CAPI: If Respondent is unwilling to answer, does not remember, or the input value is 0, please ask unfolding bracket questions here 如果受访者不愿回答或者忘记了或者填了 0 元，在此处分级展开提问] 10,000 /50,000 /100,000 /200,000 /500,000 Yuan 元

HC016_W4 Do you hold any other (in your name) wealth management products currently in addition to deposits, bonds, stocks, and funds? Including Yu'ebao, P2P, income insurance, etc. 除了存款、债券、股票和基金，您现在持有其他（记在您名下的）理财产品吗？包括余额宝、P2P、收益类保险等。

1. Yes 有
2. No 没有 → Skip to PROCEDURE before HC020 跳至 HC020 前面的程序

999. Refuse to answer 拒绝回答 → Skip to PROCEDURE before HC020 跳至 HC020 前面的程序

HC017_W4 What is the present market value of all the wealth management products you are currently holding? 您所持有的其他理财产品当前价值多少? ______ Yuan 元

[Soft Check: $> 200,000$]

[IWER: If R is unwilling to answer or does not remember, ask unfolding bracket questions 访员注意：如果受访者不愿回答或者忘记了，展开提问]

HC017_W4_bracket [CAPI: If Respondent is unwilling to answer, does not remember, or the input value is 0, please ask unfolding bracket questions here 如果受访者不愿回答或者忘记了或者填了 0 元，在此处分级展开提问] 10,000 /50,000 /100,000 /200,000 /500,000 Yuan 元

PROCEDURE 程序:
if HC005 = 0 and HC003_W4 = 0 and HC007 = 2 and HC010 = 2 and HC015 = 2 and HC016_W4 = 2, then skip HC020 如果 HC005 = 0，且 HC003_W4 = 0，且 HC007，HC010，HC015，HC016_W4 答案都为 2，则跳过 HC020

HC020 What percentage of the deposits, bonds, stocks, funds, and other wealth management products held in your name is fully controlled by you and not your spouse? (%) 您名下的存款、政府债券、股票，基金和其他理财产品有百分之多少是你自己的? ______ 0...100 %
[Hard Check: $\geq 0, \leq 100$]

HC021 Do you have any other deposits, bonds, stocks, or funds that belong to you but not in your or your spouse's name? 您是否还有其他存款、政府债券、股票和基金没有记在您或您爱人的名下?
1. Yes 是
2. No 否 → Skip to HC027 跳至 HC027

999. Refuse to answer 拒绝回答 → Skip to HC027 跳至 HC027

HC022 What is the value of such assets? 这些资产的总价值是多少? ______ yuan 元
[Soft Check: $> 200,000$]

HC027 Do you have public housing fund? 您有住房公积金吗?
1. Yes 有
2. No 没有 → Skip to HC030 跳至 HC030

999. Refuse to answer 拒绝回答 → Skip to HC030 跳至 HC030

HC028 What is the total amount of money in your public housing fund? 您现在住房公积金账户上一共有多少钱? ______ Yuan 元
[Soft Check: $> 100,000$]
[IWER: If R is unwilling to answer or does not remember, ask unfolding bracket questions 访员注意：如果受访者不愿回答或者忘记了，展开提问]

HC029 [CAPI: If Respondent is unwilling to answer, does not remember, or the input value is 0, please ask unfolding bracket questions here 如果受访者不愿回答或者忘记了或者填了 0 元，在此处分级展开提问] 5,000/10,000/50,000/100,000/200,000 Yuan 元

HC030 Have you contributed any Jizikuan to your employer or other emploers and have not got it back? (Jizikuan is a type of fund that employers collect from workers for investment or building apartments, etc.)? 您有没有交给自己单位或其他单位的集资款还未返还?
1. Yes 有
2. No 没有 → Skip to HC033 跳至 HC033

999. Refuse to answer 拒绝回答 → Skip to HC033 跳至 HC033

HC031 What is the amount of your jizikuan? 您的集资款总金额是多少? _____ Yuan 元
[Soft Check: $> 200,000$]
[IWER: If R is unwilling to answer or does not remember, ask unfolding bracket questions 访员注意：如果受访者不愿回答或者忘记了，展开提问]

HC032 [CAPI: If Respondent is unwilling to answer, does not remember, or the input value is 0, please ask unfolding bracket questions here 如果受访者不愿回答或者忘记了或者填了 0 元，在此处分级展开提问] 5,000/10,000/50,000/100,000/200,000 Yuan 元

HC033 Do you have any unpaid salary that your employer still owes you? 目前，有单位拖欠您的工资吗?
1. Yes 有
2. No 没有 → Skip to HC039_W3 跳至 HC039_W3
999. Refuse to answer 拒绝回答 → Skip to HC039_W3 跳至 HC039_W3

HC034 What is the amount of your unpaid salary? 一共拖欠您多少工资? _____ Yuan 元
[Soft Check: $> 100,000$]
[IWER: If R is unwilling to answer or does not remember, ask unfolding bracket questions 访员注意：如果受访者不愿回答或者忘记了，展开提问]

HC035 [CAPI: If Respondent is unwilling to answer, does not remember, or the input value is 0, please ask unfolding bracket questions here 如果受访者不愿回答或者忘记了或者填了 0 元，在此处分级展开提问] 5,000/10,000 /50,000 /100,000 /200,000 Yuan 元

HC039_W3 Have you lent to other families or individuals and not been repaid by them? 除了前面提到的未返还的集资款和拖欠的工资，现在还有没有人或单位欠您的钱没还的?
1. Yes 有
2. No 没有 → Skip HC040_W3 and HC041_W3 跳过 HC040_W3 和 HC041_W3
999. Refuse to answer 拒绝回答 → Skip HC040_W3 and HC041_W3 跳过 HC040_W3 和 HC041_W3

HC040_W3 What is the total amount of the loans? 他们一共还欠你多少钱? _____ Yuan 元
[Soft Check: $> 500,000$]
[IWER: If R is unwilling to answer or does not remember, ask unfolding bracket questions 访员注意：如果受访者不愿回答或者忘记了，展开提问]

HC041_W3 [CAPI: If Respondent is unwilling to answer, does not remember, or the input value is 0, please ask unfolding bracket questions here 如果受访者不愿回答或者忘记了或者填了 0 元，在此处分级展开提问] 5,000/10,000/50,000/100,000/200,000 Yuan 元

PART 2 Debts 债务

[INTRO: The following questions pertain to your debt 下面是关于您债务的问题]

HD001 What is the total amount of loans that you haven't repaid yet (not including mortage and credit card balance)? Please fill in 0 if there is no loan 您尚未还清的贷款总额是多少？不包括房贷不包括信用卡。如果没有，请填 0。_____ Yuan 元
[Soft Check: $> 50,000$]
[IWER: If R is unwilling to answer or does not remember, ask unfolding bracket questions 访员注意：如果受访者不愿回答或者忘记了，展开提问]

HD002 [CAPI: If Respondent is unwilling to answer or does not remember, please ask unfolding bracket questions here 如果受访者不愿回答或者忘记了，在此处分级展开提问]
5,000/10,000/50,000/100,000/500,000 Yuan 元

HD003 Do you have any credit card? A credit card includes Jingdong Baitiao and Huabei. If so, what is the amount of your credit card balance? If not, please fill in 0 您有信用卡吗？信用卡包括京东白条和花呗。有的话，现在您的信用卡所欠金额是多少？如果没有，请填 0。_____ Yuan 元
[Soft Check: $> 50,000$]
[IWER: If R is unwilling to answer or does not remember, ask unfolding bracket questions 访员注意：如果受访者不愿回答或者忘记了，展开提问]

HD004 [CAPI: If Respondent is unwilling to answer or does not remember, please ask unfolding bracket questions here 如果受访者不愿回答或者忘记了，在此处分级展开提问]
500/1,000/5,000/10,000/50,000 Yuan 元

HD004_W3 What is the total amount of money you owe to other families, individuals, or employers? Exclude the loans and credit card debt mentioned above. Please fill in 0 if you owe no money 您还欠其他家庭、个人或单位的钱总数是多少？不包括前面提到的贷款及信用卡欠款等，如果没有，请填 0。_____ Yuan 元
[Soft Check: $> 50,000$]
[IWER: If R is unwilling to answer or does not remember, ask unfolding bracket questions 访员注意：如果受访者不愿回答或者忘记了，展开提问]

HD004_W3_1 [CAPI: If Respondent is unwilling to answer or does not remember, please ask unfolding bracket questions here 如果受访者不愿回答或者忘记了，在此处分级展开提问]
500/1,000/5,000/10,000/50,000 Yuan 元

HD006_W4 Has someone tried to defraud you in the past year? There is no need to have lost property 过去一年，是否有人试图对您进行诈骗？不需要有财物损失。

1. Yes 有
2. No 没有 → Skip to HD005_W4 跳至 HD005_W4

999. Refuse to answer 拒绝回答 → Skip to HD005_W4 跳至 HD005_W4

HD007_W4 What is the type of the fraud? 对您进行诈骗的类型是？

1. Telecommunications fraud 电信电话网络诈骗

2. Fundraising event fraud 集资诈骗
3. Fraudulent pyramid scheme 传销诈骗
4. Sales fraud 销售诈骗
5. Other 其他，please specify 请注明 _____ **(HD007_W4_1)**

HD008_W4 What is the amount of money you lost? If no loss, fill in 0 Yuan 损失了多少钱？如果没有损失，填 0 _____ Yuan 元

HD005_W4 Have you experienced fraud in the past with property loss? Not including that you experienced in the past year 以前，您是否有遭遇诈骗的经历？有财物损失，不包括过去一年。

1. Yes 有，in total 一共有 _____ **(HD005_W4_0)** times 次
2. No 没有 → Skip to HD012 跳至 HD012

999. Refuse to answer 拒绝回答 → Skip to HD012 跳至 HD012

HD005_W4_1 If ever experienced, what is the amount of money you lost? Consider only that with the largest number of losses 有的话，损失了多少钱？仅考虑损失数目最大那次 _____ Yuan 元

HD005_W4_2 Which year? Consider only that with the largest number of losses 哪一年？仅考虑损失数目最大那次 _____ 年

HD012 How often did the respondent receive assistance in answering section H ASSETS? 受访者填写该部分问卷时是否求助？

[IWER: If it is answered by a proxy, please record the respondent's reaction. 访员注意：如果是协助回答，请记录受访者的反应]

1. Never 从未
2. A few times 偶尔几次
3. Most or all of the time 大多数时间

HA&I House Property and Housing Characteristics 房产和住房情况

Preloaded Variables from the Last Wave's Interview: 根据上一轮调查形成的加载变量

**ZLocation_*i*_**	Location of ith house in last IW<br>上轮调查时第 *i* 个房子的地址
**ZSize_*i*_**	Size of ith house in last IW<br>上轮调查时第 *i* 个房子的面积
**ZTimeBuy_*i*_**	Time of purchase of ith house in last IW<br>上轮调查时第 *i* 个房子的购买时间
**ZTimePrice_*i*_**	Purchase price of ith house in last IW<br>上轮调查时第 *i* 个房子的购买价格
**ZValueLastTime_*i*_**	Market value of ith house in last IW<br>上轮调查时第 *i* 个房子的市场价值
**ZHA000_W4_1_*i*_**	Whether i^{th} house have ownership information, 1 for yes, 0 for no<br>上轮调查时第 *i* 个房子是否有产权信息，1 为有，0 为没有
**ZHA000_W4_2_*i*_**	String expression for i^{th} house's ownership information in last IW<br>上轮调查时第 *i* 个房子产权信息的字符串表达
**Z_N**	Totol number of house properties preloaded for the household<br>家户加载的总房产数

HA House Property 房产

[IWER: Ask financial respondent to answer questions in this section. Do not allow others to completely answer the entire section 访员注意：这部分要询问家庭财务受访者，不允许完全请别人代答]

[INTRO: The following questions pertain to the house properties of [preload main respondent's name] and [preload main respondent's spouse's name] (if any) 下面是关于 [加载主要受访者名字] 和 [加载主要受访者配偶名字](如果有的话) 有产权的房子的问题]

HA000_W4_0 BRANCHPOINT:

Skip to HA000_W4_0 if this is a new household 如果是新的受访户，跳至 HA000_W4_0

Skip to HA000_W4_1 BRANCHPOINT if previously interviewed 如果是老受访户，跳至 HA000_W4_1 BRANCHPOINT

HA000_W4_0 How many house properties are in [preload main respondent's name] and [preload main respondent's spouse's name] (if any)? [加载主要受访者名字] 和 [主要受访者配偶名字](如果有的话) 名下有几处房产? ______ houses 处

[IWER: A property under somebody's name is that [load main respondent's name] or [load main respondent's spouse's name] (if any) is on the property right certificate. Fill in 0 if they have no property 访员注意：名下的房产是指房产证上有 [加载主要受访者名字] 或 [加载主要受访者配偶名字](如果有的话) 的名字。没有房产，填 0]

HA000_W4_1 BRANCHPOINT :
Skip to HA024_W4 BRANCHPOINT if this is a new household 如果该家户是新受访家户，跳至 HA024_W4 BRANCHPOINT

[CAPI: Preload information about all the house properties recorded in the last wave, i.e. the houses with property rights. For each of the house properties, ask loop questions from HA000_W4 to HA022_W4 Bracket. Skip to HA023_W4 when the loop ends 加载家户上一轮所有房产的信息，即有产权的房子，对于每一处，询问 HA000_W4 - HA022_W4 Bracket 的循环 (循环序号从 1 开始)。循环结束之后，跳至 HA023_W4]

[INTRO: Now we would like to know more about the house(s) you mentioned on the last interview 现在我们想了解一下上次访问时您提到的房子的情况]

HA000_W4 The interviewer confirms the following table to the respondent. If correct, it does not need to be modified, if not, modify it directly in the table, noting that the time of purchase, the purchase price and the market value in [ZIWTime] cannot be modified 访员向受访者确认下面表中内容，如果正确，不需修改，如果错误，直接在表中修改，注意购买时间，购买价格和 [ZIWTime] 访问时价值不可修改。
[CAPI: The location and size of the house can be directly modified. But the time of purchase, the purchase price and the market value in [ZIWTTime] cannot be modified 房子地址，房子面积都可以直接修改，购买时间，购买价格和 [ZIWTime] 访问时价值不可修改]
[IWER: Do not check the "Do not know this house" box just because the house doesn't exist this year (e.g. it was demolished), or because the house is not owned by main respondent and spouse this year (due to sale or other reasons) 访员注意：不要仅仅因为以下原因勾选不认识这个房子选项：今年房子不存在了（比如被拆），或者房子今年不属于主要受访者和配偶所有（出售或者其他原因导致）]

1.

房子地址	房子面积	购买时间	购买价格	[ZIWTime] 访问时价值
(ZLocation_*i*_)	(ZSize_*i*_)	(ZTimeBuy_*i*_)	(ZPriceBuy_*i*_)	(ZValueLastTime_*i*_)

2. Do not know this house 不认识这个房子 → Skip to the next house property (if any) 跳至下一处房产（如果有的话）

PROCEDURE 程序:
Preload house property information from the previous interview 加载房子之前访问的产权信息
Skip to HA000_W4_2 if house property information is blank, e.g. ZHA000_W4_1 = 0 如果产权信息为空，即 ZHA000_W4_1 = 0，那么跳至 HA000_W4_2
If not (ZHA000_W4_1 = 1), Skip to HA000_W4_1 如果产权信息不为空，即 ZHA000_W4_1 = 1，请跳至 HA000_W4_1

HA000_W4_1 In [ZIWTime], the ownership structure of [preload house information] is like this [preload house property information in the previous interview ZHA000_W4_2], correct? [ZIWTime] 的时候，[加载房子信息] 的产权结构是这样的，对吗？[加载房子之前的产权信息，ZHA000_W4_2]

1. Yes 是 → Skip to HA001_W4_0 跳至 HA001_W4_0
2. No 否
999. Refuse to answer 拒绝回答

HA000_W4_2 In [ZIWTime], what is the ownership structure of [preload house information] (check all that apply) [ZIWTime] 的时候，[加载房子信息] 的产权是怎么样的？（可多选）

[IWER: Check “Other”, specify the reason, and fill in 100 (%) for the percentage if the house is not related to the main respondent and his/her spouse, or to other relatives or friends at that time (e.g. renting from unrelated people) 访员注意：如果那时此房子和主要受访者及其配偶，以及其他亲戚朋友没有任何关系（比如租住在没有亲属关系的人家），那么勾选“其他”，填明原因，百分比填 100（%）]

1. [Preload the name(s) of the main respondent and his/her spouse (if any)] [加载主要受访者和其配偶名字，如果有的话]，占 ______ (**HA000_W4_2_1**) %
2. [Preload main repsondent’s name]’s children, son-in-law, daughter-in-law [加载主要受访者名字] 的子女，女婿，儿媳
3. [Preload main repsondent’s name]’s siblings [加载主要受访者名字] 的兄弟姐妹，占 ______ (**HA000_W4_2_3**) %
4. [Preload main repsondent’s name]’s parents, parents-in-law [加载主要受访者名字] 的父母，岳父母
5. [Preload main repsondent’s name]’s grandchildren [加载主要受访者名字] 的孙子女、外孙子女
6. [Preload main repsondent’s name]’s other relatives [加载主要受访者名字] 的其他亲戚，占 ______ (**HA000_W4_2_6**) %
7. [Preload main repsondent’s name]’s friend(s) [加载主要受访者名字] 的朋友，占 ______ (**HA000_W4_2_7**) %
8. Other 其他, please specify 请注明 ______ (**HA000_W4_2_9**), 占 ______ (**HA000_W4_2_8**) %
9. No property right certificate. But [Preload the name(s) of the main respondent and his/her spouse (if any)] has(ve) the rights to use and inherit, but no right to sell 没有产权证，但 [加载主要受访者和其配偶名字，如果有的话] 有使用权、继承权，没有出售权

999. Refuse to answer 拒绝回答

PROCEDURE 程序:

Ask HA000_W4_3 and HA000_W4_4 if child(ren), son-in-law or daughter-in-law has property rights of the house (HA000_W4_2 = 2) 如果子女，女婿或儿媳对该房子有产权 (HA000_W4_2 = 2)，那么提问 HA000_W4_3 和 HA000_W4_4

HA000_W4_3 Which children or spouses of children? (check all that apply) 是哪些子女或子女的配偶？（可多选）

[Preload the list of children 加载子女列表]

PROCEDURE 程序:

For each child selected in HA000_W4_3, ask HA000_W4_4 对 HA000_W4_3 中选择的每个子女，循环提问 HA000_W4_4

HA000_W4_4 The share of [preload names of children in HA000_W4_3] or spouse in house property rights is? [加载 HA000_W4_3 中的子女名字] 或其配偶占 [加载房子信息] 产权的比重是 ______ %

PROCEDURE 程序:
Ask HA000_W4_5 and HA000_W4_6 if parents or parents-in-law own property rights to the house (HA000_W4_2 = 4) 如果父母、岳父母对该房子有产权 (HA000_W4_2 = 4)，那么提问 HA000_W4_5 和 HA000_W4_6

HA000_W4_5 Which parents or parents-in-law? (check all that apply) 是哪个/几个父母或者岳父母?（可多选）
[Preload list of parents/parents-in-law 加载父母及岳父母列表]

PROCEDURE 程序:
For each parent selected in HA000_W4_5, ask HA000_W4_6 对 HA000_W4_5 中选择的每个父母或岳父母，循环提问 HA000_W4_6

HA000_W4_6 The share of [preload name of parents / parents-in-law in HA000_W4_5] in house property is? [加载 HA000_W4_5 中的父母或岳父母名字] 占 [加载房子信息] 产权的比重是 ______ %

PROCEDURE 程序:
Ask HA000_W4_7 and HA000_W4_8 if grandchildren have property rights of the house (HA000_W4_2 = 5) 如果孙子女、外孙子女对该房子有产权 (HA000_W4_2 = 5)，那么提问 HA000_W4_7 和 HA000_W4_8

HA000_W4_7 These grandchildren are children of which of your children? (check all that apply) 孙子女/外孙子女是哪些子女的孩子?（可多选）
[Preload the list of children 加载子女列表]

PROCEDURE 程序:
For each child selected in HA000_W4_7, ask HA000_W4_8 对 HA000_W4_7 中选择的每个子女，循环提问 HA000_W4_8

HA000_W4_8 The share of [preload names of children in HA000_W4_7]'s child in house property is? [加载 HA000_W4_7 中的子女名字] 的孩子占 [加载房子信息] 产权的比重是 ______ %

HA001_W4_0 Has the ownership structure of [preload house information] changed now compared to when it was [ZIWTime]? 与 [ZIWTime] 的时候相比，现在 [加载房子信息] 的产权结构发生变化了吗?
1. Yes, changed 有变化
2. No, no change 没有变化
997. Do not know 不知道
999. Refuse to answer 拒绝回答

PROCEDURE 程序:

If ZHA000_W4_1 = 0 and HA000_W4_2 ≠ 999 and HA001_W4_0 = 2, or ZHA000_W4_1 = 1 and HA001_W4_0 = 2, 999 and HA000_W4_2 ≠ 999 and HA001_W4_0 = 2, i.e. if the respondent answered the detailed ownership structure and there is no change until now, then Skip to HA004_W4 BRANCHPOINT 如果 ZHA000_W4_1 = 0 且 HA000_W4_2 ≠ 999 且 HA001_W4_0 = 2，或 ZHA000_W4_1 = 1 且 HA001_W4_0 = 2, 999 且 HA000_W4_2 ≠ 999 且 HA001_W4_0 = 2，即回答了之前的详细产权信息且现在产权没有变化，那么跳至 HA004_W4 BRANCHPOINT

Otherwise, ask HA001_W4_1 其他情况，询问 HA001_W4_1

HA001_W4_1 What is the ownership structure of [preload house information] now (check all that apply)? 现在，[加载房子信息] 的产权是怎么样的？（可多选）

[IWER: Check "Other", specify the reason, and fill in 100% if the house was demolished or sold to someone you Do not know 访员注意：如果房子被拆，被出售给不认识的人，那么勾选"其他"，注明原因，占比写 100%]

1. [Preload the name(s) of the main respondent and his/her spouse (if any)] [加载主要受访者和其配偶名字，如果有的话]，占 _____ (**HA001_W4_1_1**) %
2. [Preload the name(s) of the main respondent and his/her spouse (if any)] [加载主要受访者名字] 的子女，女婿，儿媳
3. [Preload main repsondent's name]'s siblings [加载主要受访者名字] 的兄弟姐妹，占 _____ (**HA001_W4_1_3**) %
4. [Preload main repsondent's name]'s parents, parents-in-law [加载主要受访者名字] 的父母，岳父母
5. [Preload main repsondent's name]'s grandchildren [加载主要受访者名字] 的孙子女、外孙子女
6. [Preload main repsondent's name]'s other relatives [加载主要受访者名字] 的其他亲戚，占 _____ (**HA001_W4_1_6**) %
7. [Preload main repsondent's name]'s friend(s) [加载主要受访者名字] 的朋友，占 _____ (**HA001_W4_1_7**) %
8. Other 其他, please specify 请注明 _____ (**HA001_W4_1_9**), 占 _____ (**HA001_W4_1_8**) %
9. No property right certificate. But [preload the name(s) of the main respondent and his/her spouse (if any)] has(ve) the rights to use and inherit, but no right to sell 没有产权证，但 [加载主要受访者和其配偶名字，如果有的话] 有使用权、继承权，没有出售权

997. Do not know 不知道
999. Refuse to answer 拒绝回答

PROCEDURE 程序:

Ask HA001_W4_2 and HA001_W4_3 if child(ren), or child(ren)-in-law has property right to the house (HA001_W4_1 = 2) 如果子女，女婿或儿媳对该房子有产权 (HA001_W4_1 = 2)，则询问 HA001_W4_2 和 HA001_W4_3

HA001_W4_2 Which children or spouses of children? (check all that apply) 是哪些子女或子女的配偶?（可多选）
[Preload the list of children 加载子女列表]

PROCEDURE 程序:
For each child selected in HA001_W4_2, ask HA001_W4_3 对 HA001_W4_2 中选择的每个子女，循环提问 HA001_W4_3

HA001_W4_3 The share of [preload name of children in HA001_W4_2] or spouse in house property rights is? [加载 HA001_W4_2 中的子女名字] 或其配偶占）占 [加载房子信息] 产权的比重是 ______ %

PROCEDURE 程序:
Ask HA001_W4_4 and HA001_W4_5 if parents or parents-in-law have property rights to the house (HA001_W4_1 = 4) 如果父母、岳父母对该房子有产权 (HA001_W4_1 = 4)，则询问 HA001_W4_4 和 HA001_W4_5

HA001_W4_4 Which parents / parents-in-law? (check all that apply) 是哪个/几个父母或者岳父母?（可多选）
[Preload list of parents/parents-in-law 加载父母及岳父母列表]

PROCEDURE 程序:
For each parent selected in HA001_W4_4, ask HA001_W4_5 对 HA001_W4_4 中选择的每个父母或岳父母，循环提问 HA001_W4_5

HA001_W4_5 The share of [preload names of parents / parents-in-law in HA001_W4_4] in house property is? [加载 HA001_W4_4 中的父母岳父母名字] 占 [加载房子信息] 产权的比重是 ______ %

PROCEDURE 程序:
Ask HA001_W4_6 and HA001_W4_7 if grandchildren own property rights to the house (HA001_W4_1 = 5) 如果孙子女、外孙子女对该房子有产权 (HA001_W4_1 = 5)，则询问 HA001_W4_6 和 HA001_W4_7

HA001_W4_6 These grandchildren of children of which of your children? (check all that apply) 孙子女/外孙子女是哪些子女的孩子?（可多选）
[Preload the list of children 加载子女列表]

PROCEDURE 程序:
For each child selected in HA001_W4_6, ask HA001_W4_7 对 HA001_W4_6 中选择的每个子女，循环提问 HA001_W4_7

HA001_W4_7 The share of [preload children's names in HA001_W4_6]'s child in house property is? [加载 HA001_W4_6 中的子女名字] 的孩子占 [加载房子信息] 产权的比重是 ______ %

HA004_W4 BRANCHPOINT :

Ask questions from HA004_W4 to HA007_W4 Bracket if HA001_W4_1 = 1, 9, or (HA000_W4_2 = 1, 9) and HA001_W4_0 = 2, i.e., the main respondent or his/her spouse owns property right to the house currently 如果 HA001_W4_1= 1, 9，或（HA000_W4_2 = 1, 9）且 HA001_W4_0 = 2，即现在主要受访者或其配偶有此房子的产权，则询问 HA004_W4 至 HA007_W4 Bracket

Ask questions from HA010_W4 to HA022_W4 Bracket if ZHA000_W4_1 = 1 and HA000_W4_1 = 1 and HA001_W4_0 = 1 and (HA001_W4_1 ≠ 1, 9), or ZHA000_W4_1 = 1 and (HA000_W4_1 = 2, 999) and (HA000_W4_2 = 1, 9) and HA001_W4_0 = 1 and (HA001_W4_1 ≠ 1, 9), or ZHA000_W4_1 = 0 and (HA000_W4_2 = 1, 9) and HA001_W4_0 = 1 and (HA001_W4_1 ≠ 1, 9), i.e., the main respondent or his/her spouse previously owned property right to the house, but now does not 如果 ZHA000_W4_1 = 1 且 HA000_W4_1 = 1 且 HA001_W4_0 = 1 且 HA001_W4_1 ≠ 1, 9), 或 ZHA000_W4_1 = 1 且(HA000_W4_1 = 2, 999) 且（HA000_W4_2 = 1, 9）且 HA001_W4_0 = 1 且（HA001_W4_1 ≠ 1, 9），或 ZHA000_W4_1 = 0 且（HA000_W4_2 = 1, 9）且 HA001_W4_0 = 1 且（HA001_W4_1 ≠ 1, 9），即此前主要受访者或其配偶有此房子产权，但现在没有，则询问 HA010_W4 至 HA022_W4 Bracket

In other cases, end this loop and Skip to the next house property, if any 其他情况，结束本循环，跳至下一处房产，如果有的话

HA004_W4 What is the current market value of the house [preload house info]? That is, how much would the house sell for if it were sold now 现在，[加载房子信息] 的房子的市场价值是多少？即如果现在出售，房子能卖多少钱 Total Price 总价格 ______ **(HA004_W4_1)** 10,000 Yuan 万元 or Unit Price 单位价格 ______ **(HA004_W4_2)** 1,000 Yuan/square meter 千元/平方米

[IWER: If R is unwilling to answer or does not remember, ask unfolding bracket questions 访员注意：如果受访者不愿回答或者忘记了，展开提问]

HA004_W4 Bracket [CAPI: If Respondent is unwilling to answer, does not remember, or the input value is 0, please ask unfolding bracket questions here 如果受访者不愿回答或者忘记了或者填了 0 元，在此处分级展开提问]

Unit Price 单位价格 1,000/3,000/5,000/8,000/15,000 Yuan/Square Meter 元/平方米

HA005_W4 Is the house [preload house info] for rent? [加载房子信息] 的房子是否出租?

1. Yes 是
2. No 否 → Skip to HA007_W4 跳至 HA007_W4

999. Refuse to answer 拒绝回答 → Skip to HA007_W4 跳至 HA007_W4

HA006_W4 What is the monthly rent of the house [preload house information]? [加载房子信息] 的房子每月的租金是多少? ______ Yuan/Month 元/月

[IWER: If R is unwilling to answer or does not remember, ask unfolding bracket questions 访员注意：如果受访者不愿回答或者忘记了，展开提问]

HA006_W4 Bracket [CAPI: If Respondent is unwilling to answer, does not remember, or the input value is 0, please ask unfolding bracket questions here 如果受访者不愿回答或者忘记了或者填了 0 元，在此处分级展开提问]
Monthly rent 每月租金 500/1,000/2,000/5,000/8,000 Yuan/Month 元/月

HA007_W4 What is the market rent for the house [load house info]? [加载房子信息] 房子的市场租金是多少？ _____ Yuan/month 元/月
[IWER: If R is unwilling to answer or does not remember, ask unfolding bracket questions 访员注意：如果受访者不愿回答或者忘记了，展开提问]

HA007_W4 Bracket [CAPI: If Respondent is unwilling to answer, does not remember, or the input value is 0, please ask unfolding bracket questions here 如果受访者不愿回答或者忘记了或者填了 0 元，在此处分级展开提问]
Market rent 市场租金 500/1,000/2,000/5,000/8,000 Yuan/month 元/月

[CAPI: End the loop and Skip to the next house property, if any 结束本循环，跳至下一所房产，如果有的话]

HA010_W4 What is the reason why the house [load house info] does not belong to [preload name of main respondent] and [preload name of main respondent's spouse] (if any)? [加载房子信息] 的房子不属于 [加载主要受访者名字] 和 [加载主要受访者配偶名字](如果有的话) 的原因是什么？

1. Sale 出售
2. Demolished 被拆迁
3. Grants 赠予
4. Other 其他

PROCEDURE 程序：
Ask questions from HA011_W4 to HA012_W4 Bracket if the house is sold (HA010_W4 = 1)
如果房子出售了（HA010_W4 = 1），则询问 HA011_W4 至 HA012_W4 Bracket

HA011_W4 When was the house [preload house information] sold? 何时出售 [加载房子信息] 的房子？ _____ (**HA011_W4_1**) Year 年 _____ (**HA011_W4_2**) Month 月

HA012_W4 What is the net income received from the sale of the house after deducting related expenses? 扣除相关费用，出售该住宅后获得的净收入是多少？ _____ 10,000 Yuan 万元
[IWER: If R is unwilling to answer or does not remember, ask unfolding bracket questions 访员注意：如果受访者不愿回答或者忘记了，展开提问]

HA012_W4 Bracket [CAPI: If Respondent is unwilling to answer, does not remember, or the input value is 0, please ask unfolding bracket questions here 如果受访者不愿回答或者忘记了或者填了 0 元，在此处分级展开提问]
The net income 净收入 20,000/50,000/100,000/200,000/500,000 Yuan 元

[CAPI: End the loop and Skip to the next house property, if any 结束本循环, 跳至下一所房产，如果有的话]

PROCEDURE 程序:
Ask questions from HA013_W4 to HA015_W4 Bracket if the house is demolished (HA010_W4 = 2) 如果房子被拆迁了（HA010_W4 = 2），则询问 HA013_W4 至 HA015_W4 Bracket

HA013_W4 Did your family receive any compensation for the demolition of the house [preload house info]? [加载房子信息] 的房子被拆迁，您家是否得到拆迁赔偿款?

1. Yes 是
2. No 否 → End the loop and Skip to the next property, if any 结束本循环, 跳至下一所房产，如果有的话

999. Refuse to answer 拒绝回答 → End the loop and Skip to the next property, if any 结束本循环, 跳至下一所房产，如果有的话

HA014_W4 When did your family receive compensation for the demolition of the house? 您家何时获得了拆迁补偿款? ______ (**HA014_W4_1**) Year 年 ______ (**HA014_W4_2**) Month 月

HA015_W4 What was the total amount of money your family received for the demolition? 您家获得的拆迁补偿款的总额是多少? ______ 10,000 Yuan 万元
[IWER: If R is unwilling to answer or does not remember, ask unfolding bracket questions 访员注意：如果受访者不愿回答或者忘记了，展开提问]

HA015_W4 Bracket [CAPI: If Respondent is unwilling to answer, does not remember, or the input value is 0, please ask unfolding bracket questions here 如果受访者不愿回答或者忘记了或者填了 0 元，在此处分级展开提问]
Total amount of money 总价格 20,000/50,000/100,000/200,000/500,000 Yuan 元

[CAPI: End the loop and Skip to the next house property, if any 结束本循环, 跳至下一所房产，如果有的话]

PROCEDURE 程序:
Ask questions from HA016_W4 to HA019_W4 Bracket if the house is gifted out (HA010_W4 = 3) 如果房子赠予出去（HA010_W4 = 3），则询问 HA016_W4 至 HA019_W4 Bracket

HA016_W4 To whom was the house [preload house information] gifted? (check all that apply) [加载房子信息] 的房子赠予给了谁?（可多选）

1. [Preload name of the main respondent]'s children, son-in-law, daughter-in-law [加载主要受访者名字] 的子女，女婿，儿媳
2. [Preload name of the main respondent]'s sibling(s) [加载主要受访者名字] 的兄弟姐妹
3. [Preload name of the main respondent]'s parents, or parents-in-law [加载主要受访者名字] 的父母，岳父母

4. [Preload name of the main respondent]'s grandchildren [加载主要受访者名字] 的孙子女、外孙子女
5. [Preload name of the main respondent]'s other relatives [加载主要受访者名字] 的其他亲戚
6. [Preload name of the main respondent]'s friends [加载主要受访者名字] 的朋友
7. Other 其他，please specify 请注明 _____ **(HA016_W4_1)**

PROCEDURE 程序:
Ask HA016_W4_2 if the gift was made to a child, son-in-law or daughter-in-law (HA016_W4 = 1) 如果赠予了子女，女婿或儿媳（HA016_W4 = 1），则询问 HA016_W4_2

HA016_W4_2 Which child(ren) / child(ren)'s spouse(s)? (check all that apply) 是哪些子女/哪些子女的配偶?（可多选）
[Preload the list of children 加载子女列表]

PROCEDURE 程序:
Ask HA016_W4_3 if the gift was made to parents or parents-in-law (HA016_W4 = 3) 如果赠予了父母，岳父母（HA016_W4 = 3），则询问 HA016_W4_3

HA016_W4_3 Which parents / parents-in-law? (check all that apply) 是哪个/些父母, 岳父母?（可多选）
[Preload list of parents/parents-in-law 加载父母及岳父母列表]

PROCEDURE 程序:
Ask HA016_W4_4 if the gift was made to grandchildren (HA016_W4 = 4) 如果赠予了孙子女、外孙子女（HA016_W4 = 4），则询问 HA016_W4_4

HA016_W4_4 These grandchildren are children of which of your children? (check all that apply) 是哪些子女的孩子?（可多选）
[Preload the list of children 加载子女列表]

HA017_W4 When was the house [preload house information] gifted to someone else? [加载房子信息] 的房子什么时候赠予他人的? _____ **(HA017_W4_1)** Year 年 _____ **(HA017_W4_2)** Month 月

HA018_W4 Did the person who acquired the house property offer your family any compensation in cash or in kind? 获得房产的人有没有给您家现金或者实物的补偿?

1. Yes 是
2. No 否 → End the loop, Skip to the next property, if any 结束本循环, 跳至下一所房产, 如果有的话

999. Refuse to answer 拒绝回答 → End the loop, Skip to the next property, if any 结束本循环, 跳至下一所房产，如果有的话

HA019_W4 What is the total amount of compensation to your family? In-kind included 算下来，给您家的补偿总额是多少？包括实物 _____ 1,000 Yuan 千元
[IWER: If R is unwilling to answer or does not remember, ask unfolding bracket questions 访员注意：如果受访者不愿回答或者忘记了，展开提问]

HA019_W4 Bracket [CAPI: If Respondent is unwilling to answer or does not remember, please ask unfolding bracket questions here 如果受访者不愿回答或者忘记了，在此处分级展开提问。注意，填 0 在这道题不需要展开]
The total amount of compensation 总价格 5,000/10,000/50,000/100,000/200,000 Yuan 元

[CAPI: End the loop and Skip to the next house property, if any 结束本循环，跳至下一所房产，如果有的话]

PROCEDURE 程序:
Ask questions from HA020_W4 to HA022_W4 Bracket if the house is handled in other ways (HA010_W4 = 4) 如果房子处置的其他方式（HA010_W4 = 4），则询问 HA020_W4 至 HA022_W4 Bracket

HA020_W4 You said that the house [preload house info] is not owned by [preload main respondent name] and [preload his/her spouse] (if any), then how is it handled? 您说 [加载房子信息] 的房子不属于 [加载主要受访者名字] 和 [加载主要受访者配偶名字]（如果有的话）了，那么具体的处置方式是什么？ _____

HA021_W4 When was it [Preload house information] disposed of? [加载房子信息] 的房子什么时候处置的？ _____ (**HA021_W4_1**) Year 年 _____ (**HA021_W4_2**) Month 月

HA022_W4 What is the net income from handling of the house [load house info], in-kind included? [加载房子信息] 的房子处置后的净收入？包括实物 _____ 10,000 Yuan 万元
[IWER: If R is unwilling to answer or does not remember, ask unfolding bracket questions 访员注意：如果受访者不愿回答或者忘记了，展开提问]

HA022_W4 Bracket [CAPI: If Respondent is unwilling to answer, does not remember, or the input value is 0, please ask unfolding bracket questions here 如果受访者不愿回答或者忘记了或者填了 0 元，在此处分级展开提问]
20,000/50,000/100,000/200,000/500,000 Yuan 元

[CAPI: End the loop and Skip to the next house property, if any 结束本循环，跳至下一所房产，如果有的话]

HA023_W4 Is there any other house property owned by [load name of main respondent] and [load name of main respondent's spouse] (if any) in addition to the properties listed below? fill in 0 if not [Preload properties that the main respondent and his/her spouse own property rights] 除了下面列出的房产，[加载主要受访者名字] 和 [加载主要受访者配偶名字]（如果有的话）还有其他房产吗？没有填 0。_____ 处
[加载主要受访者和其配偶有产权的房产]

HA024_W4 BRANCHPOINT :

If it is a new respondent and the number of house properties is not 0, *i* starts at 1 and ends at the number of properties recorded in HA000_W4_0. Skip to HA054_W3 when the loop ends 如果是新受访者且房产数目不为 0，则 *i* 从 1 开始到 HA000_W4_0 记录的房产数为止。循环结束后，跳至 HA054_W3

If the respondent has previously been asked, *i* starts at 1 and ends at the number of properties recorded in HA023_W4. Skip to HA054_W3 when the loop ends 如果是老受访者且新得房产数目不为 0，则 *i* 从 1 开始，到 HA023_W4 记录的房产数为止。循环结束后，跳至 HA054_W3

Skip to HA054_W3 if the number of newly acquired house properties is 0 如果新得房产数目为 0，那么跳至 HA054_W3

HA024_W4[*i*] The *i*th new house you mentioned is located in 您说的第 *i* 个新房子位于
______ (**HA024_W4_1**[*i*]) province-city-county/district 省-市-县/区,
______ (**HA024_W4_2**[*i*]) county/district-town/village/street-village/community 县/区-乡/镇/街道-村/社区,
______ (**HA024_W4_4**[*i*]) No./Building 号/几栋几号

HA025_W4[*i*] Is the house [preload house property information] located in a rural or urban area? [加载房产信息] 的房产是位于农村还是城市?

1. City or town central areas 城或镇中心区
2. Town or semi-rural areas 城乡或镇乡结合区
3. Rural areas 农村
4. Special regions 特殊区域

HA026_W4[*i*] What type of house is the property [preload property information]? [加载房产信息] 的房产所属的房子类型?

1. Unit buildings 单元楼
2. Detached house 独栋
3. Row housing 联排
4. Courtyard house 四合院
5. Compound 大杂院
6. Work shed 工棚
7. Irregular construction 不规则建筑
8. Other 其他，please specify 请注明 ______ (**HA026_W4_1**[*i*])

HA027_W4[*i*] Does the house [preload house property information] belong exclusively to [preload name of main respondent] and [preload name of his/her spouse] (if any)? [加载房产信息] 的房产是完全属于 [加载主要受访者名字] 和 [加载主要受访者配偶名字]（如果有的话)?

1. Yes 是 → Skip to HA035_W4 跳至 HA035_W4
2. No 否

HA028_W4[*i*] What is the ownership structure of the house now [preload house information] (check all that apply)? 现在，[加载房子信息] 的产权是怎么样的?（可多选）

1. [Preload the name(s) of the main respondent and his/her spouse (if any)] [加载主要受访者和其配偶名字，如果有的话]，占 _____ (**HA028_W4_1**[*i*]) %
2. [Preload main repsondent's name]'s children, son-in-law, daughter-in-law [加载主要受访者名字] 的子女，女婿，儿媳
3. [Preload main repsondent's name]'s siblings [加载主要受访者名字] 的兄弟姐妹，占 _____ (**HA028_W4_3**[*i*]) %
4. [Preload main repsondent's name]'s parents, parents-in-law [加载主要受访者名字] 的父母，岳父母
5. [Preload main repsondent's name]'s grandchildren [加载主要受访者名字] 的孙子女、外孙子女
6. [Preload main repsondent's name]'s other relatives [加载主要受访者名字] 的其他亲戚，占 _____ (**HA028_W4_6**[*i*]) %
7. [Preload main repsondent's name]'s friend(s) [加载主要受访者名字] 的朋友，占 _____ (**HA028_W4_7**[*i*]) %
8. Other 其他, please specify 请注明 _____ (**HA028_W4_9**[*i*]), 占 _____ (**HA028_W4_8**[*i*]) %
9. No property right certificate. But [preload the name(s) of the main respondent and his/her spouse (if any)] have the rights to use and inherit, but no right to sell 没有产权证，但 [加载主要受访者和其配偶名字，如果有的话] 有使用权、继承权，没有出售权

999. Refuse to answer 拒绝回答

PROCEDURE 程序:

Ask HA029_W4 and HA030_W4 if child(ren), son-in-law or daughter-in-law own property right to the house (HA028_W4 = 2) 如果子女，女婿或儿媳对该房子有产权（HA028_W4 = 2），则询问 HA029_W4 和 HA030_W4

HA029_W4[*i*] Which children / spouses of children? (check all that apply) 是哪些子女/子女的配偶?（可多选）

[Preload the list of children 加载子女列表]

PROCEDURE 程序:

For each child selected in HA029_W4[*i*], ask HA030_W4[*i*] 对 HA029_W4[*i*] 中选择的每个子女，循环提问 HA030_W4[*i*]

HA030_W4[*i*] The share of [preload names of children in HA029_W4[*i*]] or spouse in house property rights is? [加载 HA029_W4[*i*] 中的子女名字]/配偶占 [加载房子信息] 产权的比重是 _____ %

PROCEDURE 程序:

Ask HA031_W4 and HA032_W4 if parents or parents-in-law own property rights to the house (HA028_W4 = 4) 如果父母、岳父母对该房子有产权(HA028_W4 = 4), 则询问 HA031_W4 和 HA032_W4

HA031_W4[*i*] Which parents / parents-in-law? (check all that apply) 是哪个/几个父母或者岳父母？（可多选）
[Preload list of parents/parents-in-law 加载父母及岳父母列表]

PROCEDURE 程序:
For each parent selected in HA031_W4[*i*], ask HA032_W4[*i*] 对 HA031_W4[*i*] 中选择的每个父母或岳父母，循环提问 HA032_W4[*i*]

HA032_W4[*i*] The share of [preload names of parents/parents-in-law in HA031_W4[*i*]] in house property is? [加载 HA031_W4[*i*] 中的父母岳父母名字] 占 [加载房子信息] 产权的比重是 _____ %

PROCEDURE 程序:
Ask HA033_W4 and HA034_W4 if grandchildren own property rights to the house (HA028_W4 = 5) 如果孙子女、外孙子女对该房子有产权（HA028_W4 = 5），则询问 HA033_W4 和 HA034_W4

HA033_W4[*i*] These grandchildren are children of which of your children? (check all that apply) 孙子女/外孙子女是哪些子女的孩子？（可多选）
[Preload the list of children 加载子女列表]

PROCEDURE 程序:
For each child selected in HA033_W4[*i*], ask HA034_W4[*i*] 对 HA033_W4[*i*] 中选择的每个子女，循环提问 HA034_W4[*i*]

HA034_W4[*i*] The share of [preload children's names in HA033_W4[*i*]]'s child in house property is [加载 HA033_W4[*i*] 中的子女名字] 的孩子占 [加载房子信息] 产权的比重是 _____ %

HA035_W4[*i*] [Preload house information] What is the floor area of the house? [加载房子信息] 房子的建筑面积是多大？ _____ Square meter(s) 平方米

HA036_W4[*i*] What is the market value of the house [preload house information] now? That is, how much would the house sell for if it were sold now 现在，[加载房子信息] 的房子的市场价值是多少？即如果现在出售，房子能卖多少钱 Total price 总价格 _____ (**HA036_W4_1**[*i*]) 10,000 Yuan 万元 or unit price 或者单位价格 _____ (**HA036_W4_2**[*i*]) 1,000 Yuan/Square metre 千元/平方米
[IWER: If R is unwilling to answer or does not remember, ask unfolding bracket questions 访员注意：如果受访者不愿回答或者忘记了，展开提问]

HA036_W4[*i*] **Bracket** [CAPI: If Respondent is unwilling to answer, does not remember, or the input value is 0, please ask unfolding bracket questions here 如果受访者不愿回答或者忘记了或者填了 0 元，在此处分级展开提问]
unit price 单位价格 1,000/3,000/5,000/8,000/15,000 Yuan/Square metre 元/平方米

HA037_W4[*i*] What year was the house [preload house information] acquired or built? [加载房子信息] 的房子是哪一年取得或建成的? _____ Year 年

HA038_W4[*i*] How much did it cost to acquire or build the house [preload house information]? 为了取得或建成 [加载房子信息] 的房子，当时花了多少钱? _____ 10,000 Yuan 万元

HA039_W4[*i*] Is the house [preload house information] for rent? [加载房子信息] 的房子是否出租?

1. Yes 是
2. No 否 → Skip to HA041_W4 跳至 HA041_W4

999. Refuse to answer 拒绝回答 → Skip to HA041_W4 跳至 HA041_W4

HA040_W4[*i*] What is the monthly rent of the house [preload house information]? [加载房子信息] 的房子每月的租金是多少? _____ Yuan/month 元/月
[IWER: If R is unwilling to answer or does not remember, ask unfolding bracket questions 访员注意：如果受访者不愿回答或者忘记了，展开提问]

HA040_W4[*i*] **Bracket** [CAPI: If Respondent is unwilling to answer, does not remember, or the input value is 0, please ask unfolding bracket questions here 如果受访者不愿回答或者忘记了或者填了 0 元，在此处分级展开提问]
The monthly rent 每月租金 500/1,000/2,000/5,000/8,000 Yuan/month 元/月

HA041_W4[*i*] What is the market rent of the house [preload house information]? [加载房子信息] 房子的市场租金是多少? _____ Yuan/Month 元/月
[IWER: If R is unwilling to answer or does not remember, ask unfolding bracket questions 访员注意：如果受访者不愿回答或者忘记了，展开提问]

HA041_W4[*i*] **Bracket** [CAPI: If Respondent is unwilling to answer, does not remember, or the input value is 0, please ask unfolding bracket questions here 如果受访者不愿回答或者忘记了或者填了 0 元，在此处分级展开提问]
The market rent 市场租金 500/1,000/2,000/5,000/8,000 Yuan/Month 元/月

[CAPI: End the loop and Skip to the next house property, if any]

HA054_W3 Do you or other household members take mortage to purchase your and your spouse's houses now? 您家是否正在用银行按揭贷款来购买您的这些房子?
[IWER: Ask questions for all house properties owned by the respondent and his/her spouse, excluding houses with paid off mortgage 针对受访者和其配偶拥有的所有房产提问，已还清贷款不算]

1. Yes 是
2. No 否 → End section HA Housing 结束房产模块

999. Refuse to answer 拒绝回答 → End section HA Housing 结束房产模块

HA055_W3 What is the outstanding amount of the loans? What is the outstanding amount of the loans including the principal and interest? 连本金带利息，还有多少贷款没还清? ______ 10,000 Yuan 万元

HA056_W3 How much is the unpaid interest? 其中应付利息是 ______ 10,000 Yuan 万元

HA057_W3 What is the monthly mortgage payment? 每月要还多少贷款? ______ 10,000 Yuan 万元

[Soft Check: $> 20,000$]

I Housing Characteristics 住房情况

**PROCEDURE** 程序:<br>Only main respondent answer I000_W4 - I026 仅主要受访者回答 I000_W4 - I026

I000_W4 Which of the following houses are you currently living in? 以下哪个房子是您正在居住的?

[CAPI: The serial number of house in this question is assigned by loaded house serial number and then assigned by new house seiral number 此题房子的序号，首先按照加载的旧房子的序号排列，紧接着按照新房子的序号排列]

1-25. [Preload list of house properties] [加载产权房列表] → Skip to I002 跳至 I002

99. None of the above 以上都没有

I000_W4_1 The house you are living in is located at 您正在居住的房子位于

______ **(I000_W4_1_1)** province-city-county/district 省-市-县/区,

______ **(I000_W4_1_2)** town-village/street office-neighborhood association 镇-村/街道办事处-居委会,

______ **(I000_W4_1_4)** No./Building 号/几栋几号

I000_W4_2 Is the house that you are living in in rural or urban area? 正在居住的房子是位于农村还是城市?

1. City or town central areas 城或镇中心区
2. Town or semi-rural areas 城乡或镇乡结合区
3. Rural areas 农村
4. Special regions 特殊区域

I000_W4_3 What type of building does the house you are living in belong to? 正在居住的房子所属建筑的类型?

1. Unit buildings 单元楼
2. Detached house 独栋
3. Row housing 联排
4. Courtyard house 四合院
5. Compound 大杂院
6. Work shed 工棚
7. Irregular construction 不规则建筑
8. Other 其他，please specify 请注明 ______ **(I000_W4_3_1)**

I000_W4_4 What is the ownership of the house you are living in? (check all that apply) 正在居住的房子的产权是怎么样的?（可多选）

[IWER: Check “Other”, specify the reason, and fill in 100 (%) in the percentages if the house is not related to the main respondent and his/her spouse, or to other relatives or friends at that time (e.g. renting from unrelated people) 访员注意：如果那时此房子和主要受访者及其配偶，以及其他亲戚朋友没有任何关系（比如租住在没有亲属关系的人家），那么勾选“其他”，填明原因，百分比填 100（%）]

1. [Preload the name(s) of the main respondent and his/her spouse (if any)] [加载主要受访者和其配偶名字（如果有的话）]，占 _____ **(I000_W4_4_1)** %
2. [Preload main repsondent's name]'s children, son-in-law, daughter-in-law [加载主要受访者名字] 的子女，女婿，儿媳
3. [Preload main repsondent's name]'s siblings [加载主要受访者名字] 的兄弟姐妹，占 _____ **(I000_W4_4_3)** %
4. [Preload main repsondent's name]'s parents, parents-in-law [加载主要受访者名字] 的父母，岳父母
5. [Preload main repsondent's name]'s grandchildren [加载主要受访者名字] 的孙子女、外孙子女
6. [Preload main repsondent's name]'s other relatives [加载主要受访者名字] 的其他亲戚，占_____ **(I000_W4_4_6)** %
7. [Preload main repsondent's name]'s friend(s) [加载主要受访者名字] 的朋友，占 _____ **(I000_W4_4_7)** %
8. Other 其他，please specify 请注明 _____ **(I000_W4_4_9)**，占 _____ **(I000_W4_4_8)** %
9. No property right certificate. But [preload the name(s) of the main respondent and his/her spouse (if any)] has(ve) the rights to use and inherit, but no right to sell 没有产权证，但 [加载主要受访者和其配偶名字（如果有的话）] 有使用权、继承权，没有出售权

999. Refuse to answer 拒绝回答

PROCEDURE 程序:

Ask I000_W4_5 and I000_W4_6 if child(ren), son-in-law or daughter-in-law owns property right to the house (I000_W4_4 = 2) 如果子女，女婿或儿媳对该房子有产权（I000_W4_4 = 2），则询问 I000_W4_5 和 I000_W4_6

I000_W4_5 Which children/spouses of children? (check all that apply) 是哪些子女/子女的配偶?（可多选）

[Preload the list of children 加载子女列表]

PROCEDURE 程序:

For each child selected in I000_W4_5, ask I000_W4_6 对 I000_W4_5 中选择的每个子女，循环提问 I000_W4_6

I000_W4_6 The share of [preload names of children in I000_W4_5] and spouse in house property rights is? [加载 I000_W4_5 中的子女名字] 和配偶占 [加载房子信息] 产权的比重是 _____ %

PROCEDURE 程序:

Ask I000_W4_7 and I000_W4_8 if parents or parents-in-law own property rights to the house (I000_W4_4 = 4) 如果父母、岳父母对该房子有产权(I000_W4_4 = 4), 则询问 I000_W4_7 和 I000_W4_8

I000_W4_7 Which parents/parents-in-law? (check all that apply) 是哪个/几个父母或者岳父母?(可多选)

[Preload list of parents/parents-in-law 加载父母及岳父母列表]

PROCEDURE 程序:

For each parent selected in 000_W4_7, ask I000_W4_8 对 000_W4_7 中选择的每个父母或岳父母，循环提问 I000_W4_8

I000_W4_8 The share of [preload names of parents/parents-in-law in I000_W4_7] in house property is? [加载 I000_W4_7 中的父母岳父母名字] 占 [加载房子信息] 产权的比重是 _____ %

PROCEDURE 程序:

Ask I000_W4_9 and I000_W4_10 if grandchildren own property rights to the house (I000_W4_4 = 5) 如果孙子女、外孙子女对该房子有产权（I000_W4_4 = 5），则询问 I000_W4_9 和 I000_W4_10

I000_W4_9 Which of your sons' or daughters' are these grandchildren? (check all that apply) 孙子女/外孙子女是哪些子女的孩子？（可多选）

[Preload the list of children 加载子女列表]

PROCEDURE 程序:

For each child selected in I000_W4_9, ask I000_W4_10 对 I000_W4_9 中选择的每个子女，循环提问 I000_W4_10

I000_W4_10 The share of [preload child(ren)'s name(s) in I000_W4_9]'s child in house property is? [加载 I000_W4_9 中的子女名字] 的孩子占 [加载房子信息] 产权的比重是 _____ %

PROCEDURE 程序:

Skip to the I000_W4_12 if the main respondent or his/her spouse owns property right to the house where they live in (I000_W4_4 = 1, 9) 如果主要受访者或配偶对居住地房子有产权（I000_W4_4 = 1, 9），则跳至 I000_W4_12

Ask I000_W4_11 then Skip to I000_W4_13 if the main respondent or his/her spouse owns no property right to the house where they live in (I000_W4_4 ≠ 1, 9) 如果主要受访者或配偶对居住地房子没有产权（I000_W4_4 ≠ 1, 9），询问 I000_W4_11 后跳至 I000_W4_13

I000_W4_11 What is the usable area of the house you are living in? 正在居住的房子的使用面积是多少？ _____ Square metres 平方米

I000_W4_12 What is the floor area of the house you are living in? 正在居住的房子的建筑面积是多少？ _____ Square meters 平方米

I000_W4_15 What is the market value of the house now? That is, how much the house would sell for if it were sold now 现在，房子的市场价值是多少？即如果现在出售，房子能卖多少钱，Total price 总价格 _____ （**I000_W4_15_1**）10,000 Yuan 万元 or unit price 或者单位价格 _____ （**I000_W4_15_2**）1,000 Yuan/Square meter 千元/平方米

[IWER: If R is unwilling to answer or does not remember, ask unfolding bracket questions 访员注意：如果受访者不愿回答或者忘记了，展开提问]

I000_W4_15 Bracket [CAPI: If Respondent is unwilling to answer, does not remember, or the input value is 0, please ask unfolding bracket questions here 如果受访者不愿回答或者忘记了或者填了 0 元，在此处分级展开提问]
unit price 单位价格 1,000/3,000/5,000/8,000/15,000 1,000 Yuan/Square meter 千元/平方米

I000_W4_16 Which year was the house acquired or built? 房子是哪一年取得或建成的? _____ Year 年

I000_W4_17 How much money was spent to acquire or build this house? 为了取得或建成这所房子，当时花了多少钱? _____ 10,000 Yuan 万元

I000_W4_13 What is the monthly rent for the house? 房子每月的租金是多少? _____ Yuan/Month 元/月
[IWER: If don’t have to pay for the rent, fill 0. If R is unwilling to answer or does not remember, ask unfolding bracket questions 访员注意：如果不用付租金，填 0。如果受访者不愿回答或者忘记了，展开提问]

I000_W4_13 Bracket [CAPI: If Respondent is unwilling to answer, does not remember, or the input value is 0, please ask unfolding bracket questions here 如果受访者不愿回答或者忘记了或者填了 0 元，在此处分级展开提问]
The monthly rent 每月租金 500/1,000/2,000/5,000/8,000 Yuan/Month 元/月

I000_W4_14 What is the market rent for the house? 房子的市场租金是多少? _____ Yuan/Month 元/月
[IWER: If R is unwilling to answer or does not remember, ask unfolding bracket questions 访员注意：如果受访者不愿回答或者忘记了，展开提问]

I000_W4_14 Bracket [CAPI: If Respondent is unwilling to answer, does not remember, or the input value is 0, please ask unfolding bracket questions here 如果受访者不愿回答或者忘记了或者填了 0 元，在此处分级展开提问]
The market rent 市场租金 500/1,000/2,000/5,000/8,000 Yuan/Month 元/月

I002 What is the total area of the homestead of the house you are living in? (Homestead is the land allocated by rural collective economic organizations to farmers for building houses and small gardens to meet their living needs.) 您正在居住的房子的宅基地的面积有多大?（宅基地是指农村集体经济组织为保障农户生活需要而拨给农户建造房屋及小庭院使用的土地）
_____ m^2 平方米
[Soft Check: $< 10, > 1000$]
[IWER: If no entry/not applicable, please fill in 0 如果没有此项，请填写 0]

I003 Is your residence used for business as well? 您正在居住的房子兼作生产经营用房吗?
1. Yes 是
2. No 否

I004 What type of structure is this building? 您正在居住的房子是什么建筑结构?

1. Concrete and steel/Bricks and wood 钢筋混凝土或砖木结构
2. Adobe 土坯房/土房
3. Wood/Thatched 木草屋/茅草屋
4. Cave dwelling 窑洞
5. Mongolian yurt/Woolen felt/Tent 蒙古包/毡房/帐篷
6. Stone 石头房
7. Other 其它结构，please specify 请注明 ______ **(I004_1)**

I005 When was this house built? 您正在居住的房子是什么时候建成的? ______ Year 年

> **PROCEDURE** 程序:
> Ask I005_1 if I005 is missing, i.e. not sure when the house was built 如果 I005 缺失，即不确定房子建造时间，那么回答 I005_1
> Otherwise, skip to I006 其他情况，跳至 I006

I005_1 If R is unclear about year, please choose among following items 如果不清楚具体年份，从以下选项中选择:

1. 0 – 5 years 0 – 5 年
2. 5 – 10 years 5 – 10 年
3. 10 – 20 years 10 – 20 年
4. 20 – 30 years 20 – 30 年
5. 30 – 40 years 30 – 40 年
6. More than 40 years 40 年以上

I006 Is the building one story or multi-level building? 您正在居住的房子所在建筑是平房还是楼房?

1. One-story building 平房
2. Common multi-story building 一般楼房 → Skip to I008 跳至 I008
3. Self-contained multi-story building 独门独户楼房 → Skip to I009 跳至 I009

I007 Is the story independent or compound? 是独立的平房，还是大杂院?

1. Independent story 独立的平房
2. Compound 大杂院

> **PROCEDURE** 程序:
> Skip to I010_W4 跳至 I010_W4

I008 Which story is this building on? 该住房在第几层? ______ Storey 层

> **PROCEDURE** 程序:
> If I008 > 1, ask I009 如果 I008 > 1，询问 I009
> Otherwise, skip to I010_W4 否则跳至 I010_W4

I009 Does it have elevator? 有电梯吗?
1. Yes 是
2. No 否

I010_W4 Are there any handicapped facilities (e.g., non-stair ramp)? 住宅是否有无障碍通道?(例如：没有台阶的斜坡等)
1. Yes 是
2. No 否
3. No steps on level ground, no need for handicapped facilities 平地无台阶，不需要无障碍通道

**PROCEDURE** 程序:
If I010_W4 = 2, ask I011 如果 I010_W4 = 2，询问 I011

I011 How many steps had to be climbed to get to the main entrance of the household's flat? 从外面回来要爬多少个阶梯才能到家门口?
[IWER: Do not count steps if an elevator is available 访员注意：可以坐电梯而不用爬的阶梯忽略不计]
1. 0 step 0 个
2. 1 to 5 steps 1 – 5 个阶梯
3. 6 to 15 steps 6 – 15 个阶梯
4. 16 to 25 steps 16 – 25 个阶梯
5. More than 25 steps 25 个阶梯以上

I012 How many bedrooms, living rooms, bathrooms, and kitchens are there in your residence? 正在居住的房子有 _____ **(I012_1)** bedrooms 室 _____ **(I012_2)** living rooms 厅 _____ **(I012_3)** toilets 卫生间（厕所） _____ **(I012_4)** kitchens 厨房 _____ **(I012_5)** balcony 阳台
[Soft Check: > 20]

**PROCEDURE** 程序:
Ask I013 if no toilets in the answer to I012 如果 I012 答案中没有卫生间，则提问 I013

I013 How far is the nearest toilet to your house? 离您正在居住的房子最近的厕所多远? _____ Meters 米
[Soft Check: > 500]

I014 What is the type of toilet? Is it with or without a seat? If the respondent has both, select "toilet with a seat" 厕所是什么样的? 是蹲坑式还是坐式? 如果两者都有，选坐式
1. Toilet without a seat 蹲坑式
2. Toilet with a seat 坐式 → Skip to I016 跳至 I016

I015 Is the toilet flushable? 厕所能冲水吗?
1. Yes 是
2. No 否

I016 Does your residence have electricity? 您正在居住的房子是否有电?
1. Yes 是
2. No 否

I017 Does your residence have running water? 是否有自来水?
1. Yes 是
2. No 否

I018 Is there in-house shower or bath facility? What type? 住房内有无洗澡设施? 是什么样的?
1. Concentration supply of hot warter 统一供热水
2. Water heater installed by the household 家庭自装热水器
3. No 无

I019 Does your residence have coal gas or natural gas supply? 是否有管道煤气或天然气?
1. Yes 是
2. No 否

I020 Does your residence have heating? (does not include AC with heating or self-made heating) 是否带供暖设施（不包括土暖气和可制暖的空调)?
1. Yes 是
2. No 否 → Skip to I022_W4 跳至 I022_W4

I021_W4 What is the main heating energy source? 供暖所用的主要能源是什么?
1. Solar 太阳能
2. Coal 煤炭、蜂窝煤
3. Natural gas 管道天然气或煤气
4. Liquefied Petroleum Gas 液化石油气
5. Electric 电
6. Crop residue/Wood burning 秸秆、柴火
7. Other 其他，please specify 请注明 ______ **(I021_1)**
8. Concentration heating 统一供暖

I022_W4 What is the main source of cooking fuel? 如果做饭的话，做饭用的主要燃料是什么?
1. Coal 煤炭、蜂窝煤
2. Natural gas 管道天然气或煤气
3. Marsh gas 沼气
4. Liquefied Petroleum Gas 液化石油气
5. Electric 电
6. Crop residue/Wood burning 秸秆、柴火
7. Other 其他，please specify 请注明 ______ **(I022_1)**
8. Do not cook 不做饭

I023 Does your residence have a telephone connection? 您正在居住的房子装电话了吗?

1. Yes 是
2. No 否

I024 Does your residence have broad-band internet connection? 您正在居住的房子可以宽带上网吗?

1. Yes 是
2. No 否

I027_W3 Does your residence have an air cleaner in your home? 您正在居住的房子里是否有空气净化器?

1. Yes 是
2. No 否

I025 [Interviewer records it 访员自己记录] How clear and tidy is in this household? 这户人家的室内整洁度如何?

1. Excellent 非常整洁
2. Very clear 很整洁
3. Clear 整洁
4. Fair 一般
5. Poor 不整洁
6. Not applicable 不适用

I026 [Interviewer records it 访员自己记录] How is the temperature in this household? 这户人家的室内温度如何?

1. Very hot 很热
2. Hot 比较热
3. Bearable 还可以
4. Cold 比较冷
5. Very cold 很冷
6. Not applicable 不适用
