# Supplementary material for: The impact of pension insurance types on the health of older adults in China: a study based on the 2018 CHARLS data
Source: Front Public Health. 2023 Jun 2;11:1180024. doi: 10.3389/fpubh.2023.1180024 (PMC10272461; doi:10.3389/fpubh.2023.1180024)
Supplement: Supplementary file 1 [file Data_Sheet_1.ZIP › CHARLS_2018_Users_Guide.pdf]

# China Health and Retirement Longitudinal Study

# Wave 4 User's Guide


Yaohui Zhao

John Strauss

Xinxin Chen

Yafeng Wang

Jinquan Gong

Qinqin Meng

Gewei Wang

Huali Wang



September 2020


How to cite this document: Zhao, Yaohui, John Strauss, Xinxin Chen, Yafeng Wang, Jinquan Gong, Qinqin Meng, Gewei Wang, Huali Wang. (2020). China Health and Retirement Longitudinal Study Wave 4 User's Guide, *National School of Development*, *Peking University*.


National School of Development

Peking University

*This page intentionally left blank*

# How to Cite the CHARLS Data

当你使用 CHARLS 数据开展研究时，在介绍数据的部分，请引用对 CHARLS 数据进行介绍的权威论文，这样可以佐证数据的权威性。

When you write a paper using the CHARLS data, in data description part, making proper citations of authoritative papers can enhance the credibility of your research.

1. 介绍基线抽样方法时，请引用：
   When describing sampling method, you may cite:
   - Zhao, Yaohui, John Strauss, Gonghuan Yang, John Giles, Peifeng (Perry) Hu, Yisong Hu, Xiaoyan Lei, Man Liu, Albert Park, James P. Smith, Yafeng Wang. (2013). China Health and Retirement Longitudinal Study: 2011-2012 National Baseline User's Guide, *National School of Development, Peking University*.
   - Zhao, Yaohui, John Strauss, Xinxin Chen, Yafeng Wang, Jinquan Gong, Qinqin Meng, Gewei Wang, Huali Wang. (2020). China Health and Retirement Longitudinal Study Wave 4 User's Guide, *National School of Development, Peking University*.
   - Zhao, Yaohui, Yisong Hu, James P Smith, John Strauss, Gonghuan Yang. (2014). Cohort Profile: The China Health and Retirement Longitudinal Study (CHARLS), *International Journal of Epidemiology*, 43 (1): 61–68.
2. 介绍体检数据时，请引用：
   When describing anthropometric measures, please cite:
   - Zhao, Yaohui, Yisong Hu, James P Smith, John Strauss, Gonghuan Yang. (2014). Cohort Profile: The China Health and Retirement Longitudinal Study (CHARLS), *International Journal of Epidemiology*, 43 (1): 61–68.
3. 介绍血样数据时，请引用：
   When describing blood biomarker data, please cite:
   - Chen, Xinxin, Eileen Crimmins, Perry Hu, Jung Ki Kim, Qinqin Meng, John Strauss, Yafeng Wang, Yuan Zhang, Yaohui Zhao, 2019, Venous Blood-based Biomarkers in the China Health and Retirement Longitudinal Study: Rationale, Design, and Results of the 2015 Wave, *American Journal of Epidemiology*, 188(11): 1871–1877
4. 介绍跨期数据时，请引用：
   When describing panel data, please cite:
   - Zhao, Yaohui, John Strauss, Xinxin Chen, Yafeng Wang, Jinquan Gong, Qinqin Meng, Gewei Wang, Huali Wang. (2020). China Health and Retirement Longitudinal Study Wave 4 User's Guide, *National School of Development, Peking University*.

*This page intentionally left blank*

# Preface

This document describes the overall process, including the design, implementation, and data release, of the China Health and Retirement Longitudinal Study (CHARLS) national survey of wave four in 2018. This manual aims to enhance the user's understanding and application of the survey data.

CHARLS is a longitudinal survey that aims to be representative of the residents in mainland China aged 45 and older, with no upper age limit. It attempts to set up a high-quality public micro-database, which can provide a wide range of information from socioeconomic status to health conditions, to serve the needs of scientific research on the elderly.

To ensure the adoption of best practices and international comparability or results, CHARLS is harmonized with leading international research studies in the Health and Retirement Study (HRS) model. The national baseline survey was conducted in 2011-12, with wave 2 in 2013, wave 3 in 2015, and wave 4 in 2018. In order to ensure sample representativeness, the CHARLS baseline survey covered 150 countries/districts, 450 villages/urban communities, across the country, involving 17,708 individuals in 10,257 households, reflecting the mid-aged and older Chinese population collectively.

All data collected in CHARLS are maintained at the Institute of Social Science Survey of Peking University, Beijing, China. The first three waves of CHARLS data plus the Life History wave have all been released publicly, on the CHARLS website (http://www.charls.pku.edu.cn/en). By the end of 2019, more than 37,000 users have registered and downloaded the data, including about 10% from outside of China. Publications based on CHARLS have grown rapidly. Using online search, we found about 2,000 articles, of which 749 are English journal articles, 49 are international university theses, 31 are English language book chapters, and 56 are English working papers or reports.

*This page intentionally left blank*

# Acknowledgements

The China Health and Retirement Longitudinal Study (CHARLS) is an enormous project that required the efforts of many people. We want to express our gratitude to the CHARLS research team, the field team, and every respondent.

CHARLS project is a collaborative effort of many scholars at home and abroad. The Principal Investigators are Professor Yaohui Zhao, National School of Development (China Center for Economic Research) at Peking University, Professor John Strauss from the University of Southern California, and Professor Gonghuan Yang at the Chinese Academy of Medical Sciences/Peking Union Medical University. Dr. John Giles of the World Bank, Dr. Perry Hu of the University of California at Los Angeles, Dr. Qingyue Meng of Peking University, Professor Albert Park of the Hong Kong University of Science and Technology, and Professor Xiaoyan Lei are co-Principal Directors. Other key members of the research team included Yan Shen of Peking University, Xinzheng Shi of Tsinghua University, and Xiaoyu Wu of the Central University of Finance and Economics.

The CHARLS fieldwork in wave 4 was administered by Dr. Xinxin Chen who led a team of field supervisors, including Yuan Jia, Yongjie Wang, Qianyu Hu, and Xiaoli Zhao to recruit, train and manage a team of 564 field interviewers who worked hard in the field to achieve the high quality we see in the data. Dr. Yafeng Wang led a team, including Qinqin Meng, Jinquan Gong, Gewei Wang, Hai Bo, Bo Hou, Xiaoyu Wang and Hongyan Zhou to design the questionnaire, examine the paradata created during the fieldwork to assist quality control, clean the data and create sampling weights. Chuan Chen led the programmers, Yinxia Zhao and Sanming Ge to programm the questionnaire into CAPI and provided the technical support for the training and filedwork. Na Song managed all the logistics issues for the fieldwork, and Haiyu Jin managed the CHARLS accounts, payments to interviewers and subcontractors. Lu Chen, Ting Dai, Liqin Ding, Chenxi Kong, Zhirui Shi, Liu Tian, Yulei Weng, Jiahui Xu, and Yuan Zhang supplemented and proofread all English translations of the household questionnaire. More than a dozen students participated in post-survey data checking, cleaning, recalling respondents, coding open-ended questions, and preparing data for public release.

The China Health and Retirement Longitudinal Study (CHARLS) has received critical support from both home and abroad. The National Natural Science Foundation of China (grant number 70773002, 70910107022, 71130002, and 71450001), Behavioral and Social Research division of the National Institute on Aging of the National Institutes of

Health in the United States (grant numbers 1R21AG031372, 1R01AG037031, R03TW008358, R03AG049144, R01AG053228), the World Bank Group (contract number 7145915, 7159234, 7172961), Chinese medical board (contract number 13-154, 16-249), and Peking University all provided critical financial support for our project.

CHARLS received valuable support from many individuals and organizations at home and abroad. Our Chinese advisory committee members include Justin Lin, Qiren Zhou, Yang Yao of Peking University, Xuejin Zuo and Feng Wang of Fudan University, Peng Du of Renmin University, and Fang Cai of the Chinese Academy of Social Sciences. Our international advisory committee members include Dr. James P. Smith (Chair) of the RAND Corporation, David Wise, Lisa Berkmen, and David Bloom of Harvard University, David Weir and Robert Willis of the University of Michigan, Arie Kapteyn and Jinkook Lee of the University of Southern California, James Banks of the University of Manchester, Axel Borsch-Supan of Max Planck Institute. We thank many Peking University officials, especially former President Jianhua Lin, and Professor Qiang Li, Director of the Institute of Social Science Survey. We received valuable support from Qide Han, former Vice Chairman of the Standing Committee of the National People's Congress.

The survey could not have taken place without the understanding and support of all households participated in the CHARLS project. The data provided not only lays the foundation for academic studies on the Chinese aging problem but also throws light on the future development of the social welfare system for our government. We extend our sincerest thanks.

# Contents


How to Cite the CHARLS Data i

Preface iii

Acknowledgements v

1 General Introduction to CHARLS 1
- 1.1 Background and Significance . . . 1
- 1.2 Ethical Approval . . . 2
- 1.3 Organization of this Document . . . 2

2 Sampling 5
- 2.1 Baseline Sampling . . . 5
- 2.2 Refreshment Samples . . . 6
- 2.3 Proxies . . . 6

3 Survey Content 9
- 3.1 Survey Content - Overview . . . 9
- 3.2 CHARLS HCAP . . . 10

4 Fieldwork and Response 13

5 Coding 15

6 Weighting 17

7 Dataset Information 19

8 Appendices 21
- 8.1 Details of Household Survey Content in Waves 2 to 4 . . . 21
  - 8.1.1 Cover Screen and Exit Interview . . . 21
  - 8.1.2 Demographic Background . . . 21
  - 8.1.3 Family Relationships . . . 21
  - 8.1.4 Health Status: Self-reports . . . 22
  - 8.1.5 Health Status: Biomarkers . . . 24
  - 8.1.6 Health Care Utilization and Insurance . . . 25
  - 8.1.7 Work, Retirement and Pensions . . . 25
  - 8.1.8 Income, Expenditure and Assets . . . 25

8.1.9 Housing Characteristics . . . . . . . . . . . . . . . . . . . . . . . 26
8.2 CHARLS Cognition Questions . . . . . . . . . . . . . . . . . . . . . . . 26
8.2.1 Standard CHARLS Cognition Questions . . . . . . . . . . . . . . . 27
8.2.2 CHARLS HCAP Cognition Questions . . . . . . . . . . . . . . . . 28
8.3 Field Procedures . . . . . . . . . . . . . . . . . . . . . . . . . . . . . 30
8.3.1 Questionnaire Design . . . . . . . . . . . . . . . . . . . . . . . . 31
8.3.2 Construction of the CAPI System . . . . . . . . . . . . . . . . . . 31
8.3.3 Interviewer Recruitment and Training . . . . . . . . . . . . . . . . 31
8.3.4 Field Organization . . . . . . . . . . . . . . . . . . . . . . . . . . 32
8.3.5 Languages Used in the Field . . . . . . . . . . . . . . . . . . . . . 33
8.3.6 Quality Control in the Field . . . . . . . . . . . . . . . . . . . . . 33
8.4 Tables and Figures . . . . . . . . . . . . . . . . . . . . . . . . . . . . 34

**References** **39**

# 1 General Introduction to CHARLS

## 1.1 Background and Significance

China has the largest aging population in the world, and also one of the highest aging rates in the world today. It is projected that the proportion of those aged 65 or over will increase from 7% of the population in 2010 to 26% in 2050. The old-age support ratio (defined as the number of prime-age adults aged 15 to 64 divided by the number of adults aged 65 or above) will drop from about 9.9:1 in 2010 to 2.3:1 in 2050 (United Nations, 2019).

Related to the aging process, China has been undergoing a rapid health transition in which the nature of health problems changes from infectious diseases, which affect mainly the young, to chronic diseases affecting the elderly (Yang et al., 2013). Moreover, China is undergoing the aging process at much lower income levels than was the experience in industrial countries. Compared to most other countries with Health and Retirement Studies, China is much more rural, with lower levels of schooling among the elderly, lower levels of public services available, and enhanced importance of the family for social security. How to deal with problems of support for the well-being of the elderly is one of the most significant challenges to the fast booming Chinese society in the decades to come.

In response to this challenge, the Chinese government has taken robust actions to solve the problem. In recent decades, a series of social safety nets have been put into place. Such policies include the Minimum Living Standard Guarantee System, the New Co-operative Medical Insurance System, the Urban and Rural Resident Medical Insurance System, the New Rural Social Pension Program, the Urban Resident Social Pension Program, etc. Although some of these policies are not specifically designed for the elderly, the aged population is undoubtedly one of the most important beneficiary groups. Similar to many other policies, they are initiated by the central government, but the local governments maintain certain autonomy in the process of implementation. The local governments may decide on the schedule for pilot tests and promotion, and they may have different implementation plans. CHARLS is measuring the existence of these social safety nets at both the household and community levels and will allow analysis that hopefully will provide a more scientific basis for the government to further revise and amend the existing policies.

Prior to the CHARLS's baseline survey of 2011-12, scientific studies of China's aging-

related issues were at an early stage, the greatest obstacle being a lack of sufficient micro, longitudinal data. The existing data tended to be small scale in parts of China, not collecting the breadth of data necessary for good social scientific analysis of the health of the older population. For instance, there exist some health data sets that centered on health measures, with indicators of socioeconomic status largely neglected; on the other hand, data sets collected by social science scholars tend to be insufficient in health-related measures. Since the welfare of the elderly is closely associated with their health and socioeconomic status, and also because health and socioeconomic levels are themselves interrelated, micro-data that is of extensive coverage and high accuracy is highly needed for research on Chinese aging problems. CHARLS is an attempt to fill this gap.

CHARLS is the first nationally representative survey of the older population that enables the study of the health of the older population in China patterned after US Health and Retirement Study (HRS) and related aging surveys around the world (e.g., the English Longitudinal Survey of Aging, ELSA, and the Survey of Health, Aging and Retirement in Europe, SHARE, Japanese Study of Aging and Retirement (JSTAR), the Longitudinal Aging Survey of India (LASI), the Indonesia Family Life Survey (IFLS) and the Korean Longitudinal Survey of Aging (KLoSA), etc.).

## 1.2 Ethical Approval

Ethical approval for all the CHARLS waves was granted from the Institutional Review Board at Peking University. The IRB approval number for the main household survey, including anthropometrics, is `IRB00001052-11015`; the IRB approval number for biomarker collection, was `IRB00001052-11014`.

During the fieldwork, each respondent who agreed to participate in the survey was asked to sign two copies of the informed consent, and one copy was kept in the CHARLS office, which was also scanned and saved in PDF format. Four separate consents were obtained: one for the main fieldwork, one for the non-blood biomarkers and one for the taking of the blood samples, and another for storage of blood for future analyses.

## 1.3 Organization of this Document

Section 2 of this manual documents the sample design, focusing on the baseline sampling procedures, the refresher samples, and proxy interviews. In section 3, we introduce the contents of the survey, including a brief description of the survey content, and especially CHARLS HCAP added in wave 4. Section 4 describes how the fieldwork

was organized and how we tracked the respondents. Section 5 discusses occupation and sector coding, section 6 describes how sampling weights were constructed, and section 7 provides some basic information of the dataset.

The Appendices describe the details of the survey content by modules and HCAP tests, and provide a detailed discussion of the survey process, from questionnaire design, pretests, interviewer training, field procedures, and post-field activities to prepare for public data release.

*This page intentionally left blank*

# 2 Sampling

The CHARLS national baseline survey was conducted in 28 provinces, 150 countries / districts, 450 villages/urban communities across the country in 2011-2012, with wave 2 in 2013, wave 3 in 2015, and wave 4 in 2018. The CHARLS sample is representative of people aged 45 and over, living in households; institutionalized elderly are not sampled, but wave 1 respondents who later move to an institution are followed.

## 2.1 Baseline Sampling

The CHARLS's baseline survey includes one person per household aged 45 years of age or older and their spouse, totaling 17,708 individuals, living in 10,257 households in 450 villages/urban communities (Zhao et al., 2013, 2014a). A stratified (by per capita GDP of urban districts and rural counties) multi-stage (county/district-village/community-household) PPS random sampling strategy was adopted.

At the first stage, all county-level units were sorted (stratified) by region, within the region by urban district or rural county, and by GDP per capita (Tibet was the only province not included). The region was a categorical variable based on the NBS division of provinces. After this sorting (stratification), 150 counties or urban districts were chosen with probability proportional to population size (Zhao et al., 2013). For each county-level unit, 3 PSUs (villages and urban neighborhoods) are randomly chosen with probability proportional to population (Zhao et al., 2013). Hence CHARLS is nationally representative and representative of both rural and urban areas within China. Counties and districts in 28 provinces are included in the CHARLS sample.

In light of the outdated household listings at the village/community level due to population migration, CHARLS designed a mapping/listing software (Charls-GIS) that makes use of Google-earth map images to list all dwelling units in all residential buildings to create sampling frames.

In each sampled household, a short screening form was used to identify whether the household had a member meeting the age eligibility requirements. If a household had persons older than 45 and meeting the residence criterion, one of them was randomly selected. If the chosen person was 45 or older, then he/she became the main respondent, and his or her spouse was interviewed.

After applying sampling weights created using the sampling procedure, the CHARLS baseline sample demographics mimics very closely that of the population census in

2010 (Zhao et al., 2014a).

## 2.2 Refreshment Samples

CHARLS is a study of people aged 45 and over. As the study progresses, the sample respondents get older, leaving the youngest ages unrepresented unless new sample members are recruited to fill the gap. Those aged 45-46 at any given wave will be 47-48 by the next wave. Therefore a refreshment sample of 45-46-year-olds will be needed if the sample is to fully represent those aged 45+.

CHARLS reserved the refreshment samples in the baseline survey. If a household had persons older than 40, we randomly selected one of them. If the chosen person is 45 or older, then he/she becomes a main respondent and also interviewed his or her spouse. If the chosen person is between ages 40 and 44 he/she is reserved as a refresher sample for future rounds of survey. In wave 2, respondents who were aged 43-44 in wave 1 (plus their spouses) were added from the refresher sample, the same for waves 3 and 4 in 2015 and 2018, out of those aged 41-42 and 40 in wave 1.

Counting refresher samples and age-eligible respondents who failed to be found in the baseline but successfully contacted in the follow-up waves, the total number of individuals (main respondents plus spouses) has increased from 17,708 in wave 1 to 19,817 in wave 4 (Table 1).

Table 1 describes the age/sex composition of the CHARLS baseline sample. We have data on 17,708 individuals, of which 52.1% are female. While most of the respondents are the younger old, 40% are aged 60 years and older.

## 2.3 Proxies

A proxy interview was pursued under some special circumstances when the respondent could not complete the survey, for example, when the respondent was physically or cognitively impaired, in a hospital, or couldn't be tracked by the field teams during the fieldwork period. Some of those who refused to take part in person but someone else could do the interview on their behalf could also have a proxy interview. Before the proxy interview, the interviewer was asked to identify a proxy informant who knew the respondent well and could provide enough information about him. In most cases, a close family member, such as a spouse or child, assumed this role.

In order to limit the use of proxies during interviews, the interviewer was required to call a designated central office staff in the Beijing project office to apply for a proxy

code. The office staff checked by asking some specific reasons for the proxy request. If the request was approved, the interviewer received a proxy code and proceeded to the "complete substitution" mode. Then The CAPI system automatically switched to the substitution mode before entering the first module. Compared with the normal mode, the proxy respondent was asked only a subset of questions. In waves 4, the overall proxy rate was 8.1%.

# 3 Survey Content

## 3.1 Survey Content - Overview

The core survey consists of the following sections: (0) cover-screen, (b) demographics, (c) family structure/transfer, (d) health, (e) health insurance and health care utilization, (f) work, retirement and pension, (g&h) Income, expenditures and assets, (l) housing characteristics and the community and policy modules. The HCAP modules are in the section of health, which was added in wave 4. Table 2 summarizes the main questionnaire contents in Wave 4.

In addition to the wealth of individual social, economic and behavioral data, CHARLS is characterized by the rich information on the respondent's health. The section of health begins with the self-reports, including the respondent's self-assessment of general health, doctors diagnoses of a set of chronic diseases, eyesight, hearing and dental health, hedonic well-being, activities of daily living (ADLs), instrumental activities of daily living (IADLs), and physical functioning. Sections on depressive symptoms and cognition follow. Starting in Wave 3 we introduced an adaptive number series test of fluid intelligence, patterned on the HRS number series test. Furthermore, information is collected on several health behaviors. These include detailed information on smoking, drinking, and physical activities.

Following ELSA and the HRS, biomarkers, blood and non-blood, were collected in some waves but not all. Non-blood biomarkers such as anthropometrics and blood pressure were collected in Waves 1, 2 and 3 (for details of non-blood biomarkers collecting procedures, please see Zhao et al. (2013)). Besides non-blood biomarkers, the blood biomarkers were collected in Waves 1 and 3, to harmonize with HRS and other aging surveys, which collect blood every other wave. The first blood collection of CHARLS was conducted in the baseline survey from 2011 to 2012, and we collected blood samples for 11,847 individuals. The second follow-up wave of blood collection was done in Wave 3 (2015), and we collected blood samples for 13,013 individuals. Details of blood collection and analysis are described in Chen et al. (2019).

CHARLS used preloads in designing the questionnaire in CAPI in the three follow-up waves, 2-4. Starting in Wave 4 we converted all questions into logic graphs to show the routing patterns for each possible answer from both cross-section and preloaded variables, which helped to avoid programming errors.

Details of the content in each module are described in appendix 8.1.

## 3.2 CHARLS HCAP

Cognitive health is an important component of old-age health. The disease burden of dementia is among the highest of all chronic conditions. In order to measure dementia, in wave 4 of CHARLS, we added the Harmonized Cognitive and Dementia Assessment in China, or CHARLS HCAP, for respondents aged 60 and over, that can be used to conduct analyses on Alzheimer's disease and related dementias (ADRD). The CHARLS HCAP has been closely harmonized with the Health and Retirement Study (HRS) HCAP and those in Mexico, England, India and South Africa. The CHARLS HCAP is the first nationally representative sample in China that measures respondent tests and informant interviews that can help assess the dementia status of respondents. It also measures in detail the help that respondents received and from whom.

As a first step, we conducted a validation study in 2017 on a small sample aged 65 and above (825 CHARLS respondents and 766 subjects from hospitals in six provinces in China) and administered the full set of HRS HCAP tests on the respondents and their informants, and at the same time held an independent physician assessment using the clinical dementia rating (CDR). The physicians were all experienced in conducting CDRs, who were additionally trained by our physician. Details of the validation study are described in the paper by Meng et al. (2019).

From these data, a statistical model was built to use interview tests to predict dementia and CIND. As a result of this study, in Wave 4 the following tests were selected for the respondents, they are: the Mini-Mental State Exam (MMSE) (section MMSE), the Health and Retirement Study (HRS) - telephone interview for cognitive status (TICS) questions not in MMSE (section HT); the CERAD version of immediate word recall (section WR) and delayed word recall (section DR); animal naming (section RF); word list recognition (section WRE) and the brief community screening instrument for dementia CSI-D (section CSI-D for respondents). An informant interview to a person who knew the respondent well was also administered, in which we used the Jorm IQ-CODE (section JORM); the Blessed Part 2 (section BLESSED), and the CSI-D (section CSI-D for informants) informant interview (please refer to the appendix for details of the HCAP tests in wave 4). In Wave 4, some 11,021 respondents aged 60 and older, and their informants were part of the wave 4 CHARLS HCAP. The response rate was 99% out of all wave 4 respondents aged 60 and over.

We strongly recommend to users not to try to calculate individual respondent assessments of dementia or MCI at this time. We are working on creating such assessments

utilizing the validation sample, which we plan to release when ready, and they are expected to be done in early 2021, hopefully. Without the validation sample, including doctor diagnosis, it is impossible to assess the dementia or MCI status. Now we are in the process of analyzing the associations between the CDR assessment and the tests we gave as part of the wave 4 HCAP in order to derive prediction equations for dementia. We will use these predictive equations to make assessments with the wave 4 HCAP (which does not include CDR assessments).

# 4 Fieldwork and Response

The successful completion of this project requires implementing a set of core activities: questionnaire designing, CAPI programming, field staff recruitment and training, fieldwork organization, and quality control. Detailed descriptions of these activities are described in Appendix 8.3. Here, we briefly describe how we tracked respondents in Waves 2 to 4.

Respondents and spouses are tracked if they exit the original household. Main respondents and spouses in the baseline survey are followed throughout the life of CHARLS, or until they die. If the main respondent or spouse re-marries, the new spouse is interviewed so long as they are still married to the baseline respondent at the time of the specific wave. Exit interviews were conducted in waves 2 to 4 on respondents who died between waves, including verbal autopsies using the 2012 version from the World Health Organization.

For respondents surveyed in the baseline wave, more than 90% of them were re-contacted in each of the follow-up waves. In addition, we did not give up households or individuals we could not find in the baseline and contacted them in follow-up waves, so are respondents who failed to respond in any one or more waves.

CHARLS aims to interview every respondent face to face, no matter where they moved to. To achieve this, we borrowed tracking procedures from the very successful Indonesia Family Life Survey (IFLS, PI Strauss) and innovated by computerizing the procedure so that samples that moved can be quickly transferred to the team in the destination area. From the previous wave, we have contact information such as the current land and cell telephone numbers for each respondent. We also have the name, address and telephone numbers of relatives or friends who would likely know where they were, should they move. We use this information if we do not find a respondent where we found them previously.

Through these efforts, we obtained high rates of follow-up of our respondents. The response rate for the baseline survey was 80.5% (94% in rural areas and 69% in urban areas, lower in urban areas as is common in most surveys undertaken in developing countries (Zhao et al., 2013)). A description of the sample and response rates in waves 2, 3 and 4 are provided in Table 3.

The response rate of the tracked sample (panel sample) remains at higher than 86% in any of the follow-up waves. Specifically, among those households which were in-

terviewed in the baseline survey, about 88% of them completed at least one module in Wave 2 in 2013 (92% in rural areas and 83% in urban areas). In Waves 3 and 4, about 87% and 86% of the tracked households completed at least one module (Table 3). The success rates are high compared to many HRS-type surveys.

# 5 Coding

The coding procedures that were used successfully in Wave 3 continued in wave 4 to code the occupation and industry in the work module (questions of FC022_W4, FD005, FD012_1, FH007, FH014, FH015, FL006, and FL017_1), to code the disease in the health care module (ED018) and the cause of death in the module of verbal autopsy (VAS42 and VAS43)

Take the occupation and industry coding as an example; in the work module, occupation and industry were open-ended questions. By doing so, the interviewer did not have to instantaneously classify the occupation and industry, which would be very difficult for them to do well on site. Instead, we developed a web-based coding system. After inputting the work module data in the system, the coder then did the coding, supervised by a manager.

Figure 1 illustrates the coding procedures. Usually, there were two coders working on the same data simultaneously and independently. After the group finished coding the same entries of occupation or industry, their results were matched by the system automatically. If results from the two coders matched, this entry was deemed successfully coded. If the two results differ, this entry goes to the third coder. If the results from the third coder match any of the 2 previous results, this entry was coded successfully.

We provide in-time feedback to the field interviewer if his/her recorded answers to the open-ended questions cannot be coded successfully. Our feedback staff would contact the interviewer in the field to get enough information. In some cases, even after the feedback, the coders might still fail to code the entry. In Wave 4, about 96% entries were coded successfully in total.

When coding occupations, we use the standard Chinese GB 6-digit classification coding system, and added a few categories for some occupations which cannot be properly coded. All industries received 2-digits coding.

*This page intentionally left blank*

# 6 Weighting

Weights are recommended when making inferences at the national level in order to minimize bias resulting from different non-response rates among key sub-groups. Based on sampling weights for the baseline wave data (Zhao et al., 2013), we construct cross-sectional sample weights directly from the sampling probabilities for households and individuals in Wave 4, taking account of the death and divorce. We do not provide panel weights from wave 3, since panel weights depend on analysis purpose, users may use the panel of all waves, or only a subset of waves. Users can construct panel weights according to any appropriate sample arrition adjustment method.

We provide two sets of cross-sectional household weights, one with corrections for non-response (`HH_weight_ad1`) and one without (`HH_weight`). We provide two sets of cross-sectional individual weights, one with corrections for household and individual non-response corrections (`INDV_weight_ad2`), and one without any non-response corrections (`INDV_weight`).

Weights are calculated for the main respondent and his/her spouse only, including proxy and partial interviews. All other non-sample individuals that were interviewed, such as wrong baseline respondents (interviewer went to the wrong address, for example, which is confirmed in follow-up waves) receive no weights. The variable `crosssection` in the `Sample_Infor` dataset can be used to identify the cross-sectional sample.

*This page intentionally left blank*

# 7 Dataset Information

In this released version (versionID: 20200914) we released *twelve* main datasets, the associated *two* datasets (sample information and cross-sectional weights). The PSU information is the same as previous waves. The following table provides detailed information about these *fourteen* datasets.

| **Module in Questionnaire** | **Dataset** | **Information** |
|---|---|---|
| B. Demographic Background | Demographic_Background | Demographic information for main respondent and spouse |
| C. Family (C1) | Family_Information | Information of household and family members |
| C. Family (C2) | Family_Transfer | Transfer among family members |
| D. Health Status and Functioning | Health_Status_and_Functioning | Health behaviour and Status |
| DC. Cognition and Depression | Cognition | Cognition and HCAP |
| DD. Insider | Insider | Informant part of HCAP |
| E. Health Care and Insurance | Health_Care_and_Insurance | Health care utilization, health care costs and medical insurance |
| F. Work and Retirement | Work_Retirement | Work status and retirement |
| FN. Pension | Pension | Pension |
| G&H. Income, Expenditures and Assets (G2, HA) | Household_Income | Household income, expenditure and assets |
| G&H. Income, Expenditures and Assets (G2, HB) | Individual_Income | Individual income and assets |
| HA&I. House Property and Housing Characteristics | Housing | House properties and characteristics of house currently living in |
| | Weights.dta | Cross-sectional weights |
| | Sample_Info.dta | Responsed Samples, whether cross-sectional, whether died, and interview date |

All the data sets are stored in Stata 14 format, users can also find the summary information of variables from the released codebook.

The IDs (ID, householdID and communityID) can be matched with there counterparts in the previous waves. Users need to adjust the householdID and ID in the baseline wave, as noted in the release note of wave 2.

*This page intentionally left blank*

# 8 Appendices

## 8.1 Details of Household Survey Content in Waves 2 to 4

The core survey of CHARLS consists of the following sections:

### 8.1.1 Cover Screen and Exit Interview

In addition to having a screen for age-eligible members for a refresher sample, the cover screen collects information on whether the respondent in the previous wave is still alive, the change of his/her marital status between the previous wave and the current wave, and the current marital status. In case of a split from divorce, a new household is created. If the main respondent or spouse re-marries, the new spouse is interviewed as well.

For persons who have died, we have an exit module, similar to HRS. In the exit module, we get information on various conditions just prior to death and information on the cause of death. For the cause of death, we have implemented the World Health Organization's verbal autopsy (VA) questions since wave 2.

### 8.1.2 Demographic Background

This section collects personal information of both main respondents and spouses since the last wave, with some preload checking of data collected previously (i.e., sex, birth date, age, education). The main contents of the questionnaire include the respondents' birth date and place, resident status, some limited migration history, hukou status, place of hukou and any hukou changes, educational background, and marital status. To those divorced or widowed respondents, we also ask basic personal information about their ex-spouses.

### 8.1.3 Family Relationships

In this section, we confirm lists of household members, adding or reducing if there is change. Then, we gather detailed information about all immediate family members (parents, children, and siblings, by person), regardless of whether they are co-resident. This allows researchers to understand the characteristics of the entire set of persons who are potential support givers to the respondent. Information is collected on the parent/child's or sibling's age, whether they are still alive and, if not, when they died, educational details, occupation, whether the person owned a house, and in the case of parents and children, whether the parent/child was biological, a step

or adoptive-parent/child. Incomes are asked of all children and siblings. In addition, we ask information about other household members, excluding the main respondents, their spouses, and all immediate family members. Finally, detailed questions are asked about financial and time transfers to and from each non-co-resident parent, child or sibling. If a respondent is unable to provide a specific value, unfolding brackets are used to reduce the number of unknown responses.

#### 8.1.4 Health Status: Self-reports

This section measures self-reports of health with a biomarker section at the end. We start with the respondent rating health on a scale of very good, good, fair, poor, and very poor. This is followed by questions asking about new diagnoses by doctors of a set of chronic diseases and the timing of diagnoses of specific conditions. Where relevant, we also ask about current medications and treatments for each specific condition. Questions about eyesight, hearing and dental health are asked and then questions on hedonic well-being. We follow this subsection with a section to obtain information on activities of daily living (ADLs), instrumental activities of daily living (IADLs), and physical functioning. A section on depressive symptoms follows, using the 10 question version of the CES-D.

For those under 60, not taking the HCAP tests, there is then a short cognition section. We begin with a reduced form of the Telephone Interview for Cognitive Status, TICS (Brandt, Spencer, and Folstein, 1988), using the same structure as HRS. This includes recognition of date: month, day, year, season (we allow using lunar calendar in addition to Gregorian calendar), day of the week, how the respondent rates their own memory on an excellent, very good, good, fair, poor scale, and serial subtraction of 7s from 100 (up to five times). We also ask the respondent to redraw a picture of overlapping pentagons. In addition, for those respondents not participating in HCAP, we use the HRS version of the immediate and delayed word recall (Ofstedal, Fisher, and Herzog, 2005), using 10 nouns, with approximately 5 minutes between the immediate and delayed answers. As in HRS, we do not read out the words a second time before the delayed recall. Starting in wave 3 we introduced an adaptive number series test of fluid intelligence, patterned on the HRS number series test, but modified and tested to be more appropriate in low numeracy populations (Fisher et al., 2013; Prindle and McArdle, 2013; Strauss et al., 2018).

For those at the age of 60 and above, the respondents and their informants took the HCAP tests. On the basis of the CHARLS validation study, 7 tests were chosen to be in

CHARLS HCAP starting in wave 4. These are the MMSE, remaining questions from the HRS version of TICS that are not in MMSE, the CERAD immediate and delayed word recalls, animal naming, CERAD word list recognition and CSI-D. These are considered by the HRS HCAP team to be among the most essential tests they use. We also have an informant interview with a person who knows the respondent well. For the informant interview, we use the Blessed Dementia Scale, Part 2, the CSI-D informant interview, and the Jorm IQCODE.

The MMSE (Folstein, Folstein, and McHugh, 1975; Crum et al., 1993) is very widely used as part of a diagnosis of dementia. It has been widely used in China (e.g., Katzman et al., 1988; Yi and Vaupel, 2002). The HRS HCAP version of the Telephone Interview for Cognitive Status (TICS) (De Jager, Budge and Clarke, 2003) consists of items in TICS that are not in MMSE. These include questions that assess naming (including what do people usually use to cut paper? what do you call the kind of prickly plant that grows in the desert?) and knowledge (who is the President of China right now?). We use the CERAD version of Immediate and delayed word recall (Fillenbaum et al., 2008). The subjects were given three trials of learning and immediate recall. They were asked to recall as many words as possible after 5-minute and 30-minute intervals. In each trial, the subjects were re-read the list of words and asked to recall as many as possible, allowing for learning. Also, the word recognition test was administered to measure the effect of memory retrieval. The Community Screening Instrument for Dementia (CSI-D) (Chan et al., 2003; Prince et al., 2011) developed by the 10/66 studies consists of seven cognitive test items, such as questions describing the use of a hammer and naming an elbow, and delayed recall of three words. The total score ranges from 0 to 9 with higher scores indicating better cognitive function.

The informant survey is a very important part of HRS HCAP, especially for respondents who are unable to complete all of the tests. The informant survey also helps in judging whether the cognition problems have been worsening over time or have been stable. Given the low levels of education of the current Chinese elderly, especially women, the informant survey helps to determine whether cognition is always low for a respondent because perhaps of low schooling or whether recent and long-run worsening has occurred. We use three informant surveys in order to assess changes in cognitive status. The Jorm Informant Questionnaire on Cognitive Decline in the Elderly (IQCODE) (Jorm and Jacomb, 1989; Jorm, 1994; Wang et al., 2006) is used in the HRS HCAP and is administered to one person who knows the respondent well. It is an essential component of HCAP, especially for respondents who are unable or unwilling to complete all

of the neurocognitive tests. The informant survey also helps in determining whether cognitive problems worsened over time or have been stable, which is useful for helping to classify respondents as having MCI or dementia, or having stable but low cognition. The IQCODE assesses change in cognitive functioning for over ten years. We use the 26-item version of the IQCODE. Cognitive changes are scored on a 5-point scale, with 1 indicating "much improved", 3 "not much change", and 5 "much worse". We use part II of the Blessed dementia scale (BDS) (Erkinjuntti et al., 1988; Lam et al., 1997) to evaluate how well the elderly individual does with three different basic living activities (eating, using the toilet, and dressing) based on the interview of a close informant. The ability for each item is rated from 1 (fully independent) - 4 (entirely dependent). The Community Screening Instrument for Dementia (CSI-D) (Chan et al., 2003; Prince et al., 2011), which is the informant part of the brief CSI-D used by the 10/66 studies, consists of 6 questions assessing changes in the subject's daily functioning. The items include worsening of the ability to speak, worsening of ability to think and understand, often forgetting where she/he had put things, always forgetting what happened the day before yesterday, sometimes not being able to recognize the current location, and difficulty in dressing.

In addition to self-reported health outcome variables, information on several health behaviors is collected. This includes detailed information on smoking, drinking, and physical activities.

### 8.1.5 Health Status: Biomarkers

We follow HRS, ELSA and other aging studies in taking measurements on detailed biomarkers: blood and non-blood. Non-blood biomarkers such as anthropometrics and blood pressure were collected in waves 1, 2, 3. After Wave 3, we reverted to collecting biomarkers in every other wave, to harmonize with HRS and other aging surveys. In CHARLS, we collect data on height, lower leg and upper arm lengths (useful to get measures related to height not contaminated by shrinkage), waist circumference, blood pressure (measured 3 times), grip strength (measured by a dynamometer two times for each hand), lung capacity measured by a peak flow meter, and do a timed sit to stand (5 times starting from a full sit position on a common, plastic stool). We also conduct balance tests, the same used in HRS, and a timed walk at normal speed for 2.5 meters, again following HRS.

In waves 1 and 3, we collected whole blood samples. The blood collection and local hospital analysis are funded by complementary grants within China. For a detailed

description of the process of blood collection, the methods for blood-based bioassays and descriptive results of wave 3, please refer to the paper by Chen et al. (2019).

Sampling weights that correct for non-response is provided in the released biomarker data. For blood and separately non-blood biomarkers we create similar sample weights (Zhao et al., 2014b).

### 8.1.6 Health Care Utilization and Insurance

Indicators of curative and preventive health care utilization and health insurance coverage are collected in this module. A separate section on health insurance is asked to collect details of current and past coverage and whether coverage was lost. Health care utilization of outpatient care for the last one month is asked, with details about the last visit. Inpatient utilization over the past 1 year is asked, with details about the last visit. The questions include how much was total cost, what was out of pocket cost, whether insurance was used, if others helped pay for the care, if so whom, and how far respondents traveled.

### 8.1.7 Work, Retirement and Pensions

This section records current job status and collects detailed data including labor supply, wages and fringe benefits, including social insurance programs received through the employer. If the person has side jobs, some limited information is collected about those jobs. For people not working, information on their last job is collected. Self-employment data are collected, including household agricultural unpaid work.

We collect detailed retirement information, distinguishing between nominal retirement (pensionable) and actual retirement (withdraw from the labor market), and ask detailed questions about pensions, social security, and job-related health insurance. These questions cover the different pension systems that exist in China, such as the New Rural Social Pension Insurance, the Urban and Other Residents Pension, firm basic pensions, and pensions for government workers. We record whether counties have integrated the rural health insurance program with the urban resident program as they have been supposed to since 2016. Ordinary pensioners are distinguished in this section from "revolutionary" retirees, who get special dispensations.

### 8.1.8 Income, Expenditure and Assets

This section collects Information on wage and pubic transfer income of the main respondent and his/her spouse. In addition, the questionnaire is also designed to ob-

tain income information on household-level businesses, including as a separate section, farming, and household-level public transfer income. Also, complete household expenditure data are collected, including the value of home-produced food consumption. This part of the questionnaire is answered by the household member who is most knowledgeable about these matters. For family non-farm enterprises, estimated profits are obtained on each separate family business. Crop and livestock (and fishery) revenues and expenses are asked separately. The collection of expenditures, including auto-consumption of food, provides a very useful measure of household income. It is far less beset with measurement error than are typical measures of income, and it is a better measure of long-run resources because households tend to smooth their consumption over time, relative to their income.

This section further measures assets at both household and individual levels. Both the household value of assets is asked about and how much is owned by the main respondent or spouse. The assets are divided into the following types: housing, land, household equipment, consumer durables, and financial assets. Details on the house and land are collected, whether it is rented or owned. Details are asked for about how the house was purchased, if it was. Details are also asked about the physical characteristics of the house. For houses that are built, the financial and time costs of building them are collected so that a measure of current value can be calculated. The land is not officially privately owned in China, but information on land used is collected to try to assess the use-value of such land (including trees). Household equipment and consumer durables are asked about (self-employment assets are gotten in the work section). Financial assets of the main respondent and spouse, and separately other household members, are asked. Information on current debts is also collected.

### 8.1.9 Housing Characteristics

This section collects house characteristics. We include questions such as what type of building the house is, how many floors it has, whether it is handicapped accessible and whether it has facilities like toilet, electricity, running water, telephone, internet, air cleaner, etc. Tidiness and the temperature inside the house are included as well.

## 8.2 CHARLS Cognition Questions

CHARLS has several measures of cognition in all the waves. In wave 4, two sets of cognition questions were used. The standard CHARLS cognition questions were used for respondents under age 60. The CHARLS HCAP questions, which included the stan-

dard CHARLS cognition questions, were used for those 60 and over.

### 8.2.1 Standard CHARLS Cognition Questions

In both Pilot waves and in the first three national waves of CHARLS we began with a reduced form of the Telephone Interview for Cognitive Status, TICS (Brandt, Spencer, and Folstein, 1988), using the same structure as HRS. This includes recognition of date: month, day, year, season (we allow using lunar calendar in addition to Gregorian calendar), day of the week, how the respondent rates their own memory on an excellent, very good, good, fair, poor scale, and serial subtraction of 7s from 100 (up to five times). We also ask the respondent to redraw a picture of overlapping pentagons. In addition, we use the HRS version of the CERAD immediate and delayed word recall (Ofstedal, Fisher, and Herzog, 2005), using 10 nouns, with approximately 5 minutes between the immediate and delayed answers. We do not read out the words a second time before the delayed recall. Starting in wave 3 we introduced an adaptive number series test of fluid intelligence, patterned on the HRS number series test, but modified and tested to be more appropriate in low numeracy populations (Fisher et al., 2013; Prindle and McArdle, 2013).

CHARLS has considerable experience in fielding cognition tests in the Chinese context. This includes handling issues related to different languages and dialects. In general, we rely on interviewers who speak the local language or dialect to translate the questions at the spot. When we recruit interviewers, we prioritize those with dialectic or language skills. Almost all of our students are college students. There are 594 universities or colleges in Beijing and every province is well represented in students, so we have had no difficulty recruiting students from places of our survey, even those from Xinjiang or Tibet (we have a Tibetan county in Sichuan province in our sample). In rare cases where the dialect is an issue, for example, in Zhejiang province, where neighboring townships may have different dialects, we seek help from young people in the household or the community. Many of the choices are pre-printed on show cards. If a respondent is literate, then he/she can choose an answer from the show cards. The great majority (92%) of Chinese are Han and use the same written language. Some of the large ethnic minorities have adopted the Han written language too, such as Manchurian, Hui and Zhuang. Therefore we estimate that less than 4% of the Chinese population uses a different written language. We translate and print sets of show cards in Tibetan, Uigur, and Mongolian languages and this has proven to be adequate. For example, the word recall test has been shown to be linked in expected ways with social interactions, ed-

ucation and other socio-economic indicators (Smith, Tian, and Zhao, 2013; Lei et al., 2012, 2014b). Our CES-D depressive symptom questions list words that are possibly at issue because of dialects and language. CHARLS research using these questions have demonstrated very good validity and normal associations with socioeconomic indicators (Lei et al., 2014a).

### 8.2.2 CHARLS HCAP Cognition Questions

The tests included in the CHARLS HCAP study have been chosen based on analyses by the HRS team (Weir, McCammon, and Langa, 2014; Crimmins et al., 2011) of how well as a group they predict doctor assessed dementia using the HRS ADAMS data, the Religious Orders Study (ROS) and the Memory and Aging Project (MAP) data (Bennett et al., 2012b,a); the latter two studies referred to as the Rush studies.

The validation study was implemented in two stages. First, we administered all of the HRS HCAP instruments to the validation respondents. Other tests that had proved useful in China but were not part of the HRS HCAP battery were considered, for example, tests that were part of the China 10/66 or the Chinese Mental Health Survey but not in HCAP and a measure of depressive symptoms (CES-D 10). That is, for respondents, we used key components of HRS-HCAP, including: Mini-Mental State Exam (MMSE), telephone interview for cognitive status (TICS), the Consortium to Establish a Registry for Alzheimer's Disease (CERAD) version of immediate and delayed word recall, word recognition, animal naming, symbol digit modalities test, digit span backwards test, immediate and delayed logical memory, immediate constructional praxis, number series, Trail making test – part A and B, and the 10/66 dementia community screening instrument for dementia (CSI-D). And the symbol cancellation test substituted for the letter cancellation test since few Chinese elderly would know the Roman alphabet. For respondents with no dementia, they were able to answer all of the tests, which took a little over 1 hour. For those with dementia, they answered many of the tests, so their time required was much less.

In addition, we used a short (30 minute) informant questionnaire used in HRS HCAP (the Jorm IQCODE) for one person who knows the respondent well. This is a very important part of HRS HCAP, especially for respondents who are unable to complete all of the tests. The Jorm IQCODE informant interview reveals factors such as whether respondents' cognitive capabilities have deteriorated over time or have been stable (a distinction between dementia or CIND and being normal but possibly at low cognition levels), or whether respondents need help in their daily activities, now and in

the past (a distinction between dementia and CIND). While other informant questionnaires could be used, such as the AD8, Jorm IQCODE covers more domains and has been tested against AD8 by HRS staff (with correlation about .72). Importantly, using Jorm IQCODE for the informant interview provides better harmonization with the HRS and other HCAPs. An IQCODE version exists that has been validated in low education elderly populations in China (Fuh et al., 1995; Wang et al., 2006). CHARLS staff administered the HCAP tests and informant interviews in 2017.

In wave 4, since we collected the cognition data as part of a regular wave, we could not spend an entire hour plus on only cognition, so we used key parts of HRS HCAP tests. The CHARLS HCAP tests in wave 4 were chosen based on the analysis of the validation study. Tests were preferred if they had fewer respondents with missing scores. And regressions were run on to predict physician-diagnosed dementia with scores of different tests as covariates. Tests that did better in the prediction of dementia were placed higher in the list.

In wave 4, the following key parts of the HRS HCAP tests were used. For the respondents, the tests included the Mini-Mental State Exam (MMSE), the Health and Retirement Study (HRS) - telephone interview for cognitive status (TICS) questions not in MMSE; the CERAD version of immediate word recall and delayed word recall; animal naming; word list recognition and the brief community screening instrument for dementia CSI-D. For the informant, the tests included the Jorm IQCODE, the Blessed Part 2, and the CSI-D for informant interview.

The MMSE (Folstein, Folstein, and McHugh, 1975) is widely used to assess overall cognition, including in China (Li et al., 1988, 1989; Zhang et al., 1990). The items on orientation (year, date, day of the week), serially subtracting 7 from 100, and the drawing of overlapping pentagons are part of the regular CHARLS cognition battery. However, the rest of the MMSE items are new to CHARLS-HCAP. Based on results from the validation study (Meng et al., 2019), we used the Chinse version of MMSE in wave 4.

The HRS-HCAP version of the TICS (de Jager, Budge, and Clarke, 2003) consists of items in TICS that are not in MMSE. These include questions that assess naming (what do people usually use to cut paper? what do you call the kind of prickly plant that grows in the desert?) and knowledge (who is the President of China right now?).

The CSI-D (Chan et al., 2003; Prince et al., 2011), developed by the 10/66 studies, consists of seven cognitive test items including questions describing the use of a hammer, naming an elbow, pointing to the window and then to the door, locating the nearest

store, orientation to season, orientation to day of the week, and delayed recall of three words. The total score ranges from 0 to 9 with higher scores indicating better cognitive function.

We used the CERAD version of immediate and delayed word recall (Fillenbaum et al., 2008). The respondents were given three trials of learning and free immediate recall. They were asked to recall as many words as possible after five-minute and thirty-minute intervals. We calculated the number of exact words. In each trial, the respondents were re-read the list of words and asked to recall as many as possible, allowing for learning. Also, the word recognition test was administered to measure the effect of memory retrieval.

In the informant interview, we used three sets of tests to assess changes in respondents' cognitive status. First, the Jorm Informant Questionnaire on Cognitive Decline in the Elderly (IQCODE) (Jorm, 1994; Jorm and Jacomb, 1989; Wang et al., 2006) was used in the HRS-HCAP and was administered to one person who knew the respondent well. It is an essential component of the HRS-HCAP, especially for respondents who are unable or unwilling to complete all of the tests. The informant interview also helps in determining whether cognitive problems worsened over time or have been stable, which is useful for helping to classify respondents as having MCI or dementia or having stable but low cognition. The IQCODE assesses change in cognitive functioning over 10 years. We used the 26-item version of the IQCODE. Cognitive changes were scored on a 5- point scale. The ratings were then averaged over the questions. Second, we used part II of the Blessed dementia scale (BDS) (Erkinjuntti et al., 1988; Lam et al., 1997) to evaluate how well the elderly individual does with three different basic living activities (eating, using the toilet, and dressing) based on the interview of a close informant. Finally, the informant part of CSI-D (Chan et al., 2003; Prince et al., 2011) was used by the 10/66 studies and consisted of six questions assessing changes in the respondent's daily functioning. The items include worsening of the ability to speak, worsening of ability to think and understand, often forgetting where she/he had put things, always forgetting what happened the day before yesterday, sometimes not being able to recognize the current location, and difficulty in dressing.

## 8.3 Field Procedures

This Appendix discusses the field procedures and the steps leading up to Wave 4 of the CHARLS national survey. It usually takes one year to prepare the fieldwork of each wave. The CHARLS research team started revising the questionnaire in August 2017

and organized several pilots to test and improve the questionnaire and the survey procedure. In March and April of 2018, we conducted several formal pretests before finalizing the questionnaire using CAPI. From the experience of the pretests, the research team revised the questionnaires and procedures for the field survey. After that, the project team started to recruit interviewers and prepare training materials. Six classes of training took place at Peking University from February to July in 2018. Finally, 564 persons passed training examinations and went to the field. Most of the fieldwork was completed by the end of September of 2018; the last interviews took place at the end of March in 2019.

### 8.3.1 Questionnaire Design

Because Wave 4 is a longitudinal survey, which has complicated rules about preloading answers from previous waves and probing when detecting a change in status between waves, the research team worked very hard to streamline the questionnaire. We painstakingly drew electronic flow charts of each questionnaire module to sort out the logic of each question. In the process, we corrected previous CAPI errors and logic mistakes that existed in earlier waves.

In addition, we added a dementia screening module (HCAP) to CHARLS of all respondents aged 60 and older and their informants. We used the validated instruments from the tests for Harmonized Cognitive Assessment Protocol (HCAP), which was conducted in 2017. In the process of producing the final questionnaire, seven formal pretests took place in 24 communities in Beijing. The questionnaires were finalized following these pretests.

### 8.3.2 Construction of the CAPI System

In order to meet the requirements of the resurvey, a substantial amount of programming was carried out, particularly to accommodate the needs of follow-up procedure of the newly age-eligible respondents and non-response sample in the previous waves. We moved to tablets from laptops in Wave 4 in 2018, which facilitated collecting GPS, photos and voice recordings. Importantly, the brand new system based on tablet ran much faster than the old system.

### 8.3.3 Interviewer Recruitment and Training

In order to facilitate recruiting more than 500 interviewers in a limited time, we designed a web-based recruitment system where job applicants filled in relevant infor-

mation online, and initial screening was conducted. The positions were advertised at CHARLS Wechat public account, some help-wanted websites, both at national websites and provincial sites and at the university Bulletin Board System (BBS). In many cases, we relied on colleagues in local universities to recruit their students as interviewers. The criteria used in selecting interviewers were their stated work attitudes, any previous field experience, and communication skills. We targeted interviewers who can speak local dialects.

Most of our interviewers are undergraduate and graduate students from universities in Beijing, which has been true for all waves. In Wave 4, more than one-third of the interviewers enrolled in a formal course at Peking University. This course provided comprehensive training to students on survey methodology and gave academic credits. The rest of the interviewers received an 8-day intensive training on weekends. CHARLS training ensures that students familiarize themselves with the CAPI system, grasp the content of the questionnaire, develop interview skills, and understand the fieldwork protocol as well as their respective responsibilities. The training includes live interviews with convenience samples and rehearsals in both rural villages and urban communities in Beijing and nearby, observed by supervisors. We choose only those who passed exams and performed well in rehearsals. In addition, team-building activities are organized. The protocol of interviewer recruitment and training was almost the same in Waves 2 and 3.

### 8.3.4 Field Organization

At each wave, all interviews were personal face-to-face CAPI interviews. The protocol of field organization in Wave 4 was almost the same as that in Waves 2 and 3. In Wave 4, 564 interviewers were organized into 76 teams. Mostly, a team was responsible for interviewing all respondents in 6 villages/communities in 2 counties, including those who had moved from elsewhere into the area. Two people of each team played the role of an advance unit that updated the contact information of respondents so that movers could be identified early on and cases reassigned. They also organized the logistics for the team and conducted community-level and county-level surveys. The rest of the team consisted of a team leader and interviewers who were in charge of carrying out all the follow-up procedures and conducting individual interviews.

During the fieldwork, each team was assigned a supervisor who had experience in fieldwork. The field supervisors report to the Field Director at the CHARLS headquarters. Each supervisor manages 2-5 teams, and the CHARLS headquarters responded to all

problems that could not be solved by the supervisors.

#### 8.3.5 Languages Used in the Field

The great majority (92%) of Chinese are Han and use the same written language. Some of the large ethnic minorities have adopted the Han written language, too, such as Manchurian, Hui and Zhuang. Therefore we estimate that less than 4% of the Chinese population use a different written language. Despite having the same written language, various dialects are used in different parts of China.

To minimize communication difficulties, when we recruit interviewers, we prioritize those with dialectic or language skills. All of our students are college students. In Wave 4 there are 564 student interviewers from universities or colleges throughout China, and every province is well represented in students, so we have had no difficulty recruiting students from places of our survey, even those from Xinjiang or Tibet (we have a Tibetan county in Sichuan province in our sample). In rare cases in which none of the interviewers speak the respondent's dialect, or if a minority language other than Mandarin is needed, another staff or a local resident who speaks that dialect or language is used as a translator.

Many of the choices of the questionnaire are pre-printed on show cards. If a respondent is literate, then he/she can choose an answer from the show cards. We translate and printed sets of show cards in Tibetan, Uyghur, and Mongolian languages, and this has proven to be adequate.

#### 8.3.6 Quality Control in the Field

Quality control measures that previously worked well continued to be employed in Wave 4, including quality control sessions at the time of training and self-checklist upon completion. Conventional measures were also used to produce an accurate assessment of the data quality, such as data checking and audio recording playback (Zhao et al., 2013).

The use of Computer-Assisted Personal Interview (CAPI) dramatically increases our ability to catch and correct errors made by the interviewers in the field. While they are still in the field, CAPI informs the interviewer immediately when a section has been improperly skipped, was incomplete, or was found to take too little time. We train interviewers in these procedures. A self-check list is also provided upon completion of interviews to ensure all required tasks were performed. Restrictive passwords are used and only issued by authorized persons to limit the use of proxies during interviews.

Other data checking measures used include comparing respondent photos between different waves to ensure that the same persons are interviewed, checking audio recordings, and short phone interviews with respondents. The CAPI system allows the teams to send the data back through the internet to the head office at the end of each day, uploading the data onto a secure website, which allows for checking in real-time. We check the first interview of each interviewer, plus 10% of subsequent interviews on average. For those whose interview is inferior to their peers, their following interviews are checked more often. Usually, the interviewer will get the feedback from the quality control team within 48 hours after they finish the interview and send back the required data for quality control. By doing so, some errors are corrected before the team leaves for the next village/community.

In Wave 4, 77 members of the quality control team provided detailed feedback to interviewers on a day-to-day basis. Such communication proved to be a useful means to supplement the pre-field training and complement the in-field supervision.

## 8.4 Tables and Figures

**Table 1: Number and Age/sex Structure of Individuals: 2011 - 2018**

| Age Group | Baseline, 2011 | | | Wave 2, 2013 | | | Wave 3, 2015 | | | Wave 4, 2018 | | |
|---|---|---|---|---|---|---|---|---|---|---|---|---|
| | Total | Male | Female | Total | Male | Female | Total | Male | Female | Total | Male | Female |
| -44 | 483 | 74 | 409 | 435 | 76 | 359 | 718 | 137 | 581 | 255 | 31 | 224 |
| 45-49 | 3,575 | 1,643 | 1,932 | 3,153 | 1,398 | 1,755 | 3,175 | 1,503 | 1,672 | 1,960 | 820 | 1,140 |
| 50-54 | 2,707 | 1,310 | 1,397 | 2,827 | 1,348 | 1,479 | 3,551 | 1,694 | 1,857 | 3,500 | 1,664 | 1,836 |
| 55-59 | 3,520 | 1,721 | 1,799 | 3,406 | 1,655 | 1,751 | 3,095 | 1,532 | 1,563 | 3,045 | 1,429 | 1,616 |
| 60-64 | 2,823 | 1,432 | 1,391 | 3,152 | 1,581 | 1,571 | 3,594 | 1,723 | 1,871 | 3,375 | 1,665 | 1,710 |
| 65-69 | 1,836 | 928 | 908 | 2,084 | 1,051 | 1,033 | 2,537 | 1,297 | 1,240 | 3,162 | 1,512 | 1,650 |
| 70-74 | 1,291 | 681 | 610 | 1,466 | 756 | 710 | 1,679 | 823 | 856 | 1,996 | 1,002 | 994 |
| 75-79 | 850 | 427 | 423 | 981 | 511 | 470 | 1,083 | 577 | 506 | 1,330 | 656 | 674 |
| 80+ | 612 | 260 | 352 | 750 | 331 | 419 | 841 | 368 | 473 | 1,193 | 561 | 632 |
| Obs. | 17,697 | 8,476 | 9,221 | 18,254 | 8,707 | 9,547 | 20,273 | 9,654 | 10,619 | 19,816 | 9,340 | 10,476 |

There are 11 individuals in 2011, 10 individuals in 2013, and 11 individuals in 2015 lacking age information.

**Table 2: Summary of Data Collected in Household Questionnaire in Wave 4**

| | |
|---|---|
| **Demographic information**<br>Birthdate and birthplace<br>Residence and migration<br>Hukou information<br>Education<br>Marital status and history | **Work, retirement and pension**<br>Current job status<br>Detailed information on the current main job<br>Unemployment and job search activities<br>Retirement<br>Pension |
| **Household members (excluding respondents)**<br>Sex, birth date, marital status<br>Relationship with the main respondent<br>Hukou information<br>Education | **Income, expenditures and assets**<br>Household income and expenditures<br>Household assets<br>Individual income and assets |
| **Family**<br>All parents, children and siblings<br>Demographics<br>Education<br>Occupation<br>For deceased: time of death<br>Interactions of each family member: Time spent caring for parents Visits from children<br>Two-way financial exchanges with parents and children | **Housing characteristics**<br>Construction materials<br>Home facilities<br>Cleanliness |
| **Health status and functioning**<br>Self-reported general health<br>Doctor diagnosed chronic and infectious disease<br>Eye, hearing, oral health, pain accidents, fall, fracture Lifestyle and life behavior including sleep, physical activity, social connectedness, diet, smoking and drinking<br>Functional limitations and helpers<br>Cognition (including a number series test)<br>Depression | **CHARLS HCAP (aged 60+)**<br>Interview of the Informant: Jorm IQCODE, e Blessed Part 2 and CSI-D<br>Interview of the respondent: Mini MMSE, HRS TICS, CERAD version of immediate word recall, delayed word recall, animal naming, word list recognition and CSI-D |
| **Health care and insurance**<br>Current and past medical insurance<br>Health care utilization: outpatient and inpatient care<br>Health care costs and payment methods | **Interviewer observation**<br>Interference during interviews<br>Attitude and comprehensive ability of the respondent |

**Table 3: Response Rates: 2011-2018**

| | | Baseline | Wave 2, 2013 | | Wave 3, 2015 | | Wave 4, 2018 | |
|---|---|---|---|---|---|---|---|---|
| | | 2011[a] | Cross Section[b] | Panel[c] | Cross Section[b] | Panel[c] | Cross Section[b] | Panel[c] |
| Response Rate (%) | Total | 80.51 | 82.63 | 88.30 | 82.13 | 87.15 | 83.84 | 86.46 |
| | Rural | 94.15 | 91.74 | 92.18 | 91.32 | 93.13 | 91.40 | 92.79 |
| | Urban | 68.63 | 72.20 | 82.61 | 71.64 | 78.45 | 74.55 | 77.24 |
| No. of Households | Total | 10,257 | 10,629 | 9,022 | 11,797 | 8,715 | 10,524 | 8,288 |
| | Rural | 6,033 | 6,340 | 5,547 | 6,993 | 5,483 | 6,456 | 5,226 |
| | Urban | 4,224 | 4,289 | 3,475 | 4,804 | 3,232 | 4,068 | 3,062 |
| No. of Respondents | Total | 17,708 | 18,264 | 15,196 | 20,284 | 14,522 | 17,970 | 13,567 |
| | Rural | 10,537 | 10,950 | 9,439 | 12,075 | 9,200 | 11,017 | 8,622 |
| | Urban | 7,171 | 7,314 | 5,757 | 8,209 | 5,322 | 6,953 | 4,945 |

[a] The response rate in the baseline is computed as the number of households that completed at least one main module divided by the number of implied age-eligible households.

[b] The cross-sectional response rate in the follow-up waves (2013-2018) is computed in the same way as that in the baseline.

[c] The panel response rate in the follow-up waves (2013-2018) is computed as the number of respondents which were interviewed in the baseline and completed at least one main module in the current wave divided by the number of respondents that were interviewed in the baseline. Since CHARLS not only tracks the response samples in previous waves, but also include the non-response samples and refresh samples, so the cross-sectional sample is not necessarily the same as the panel sample.

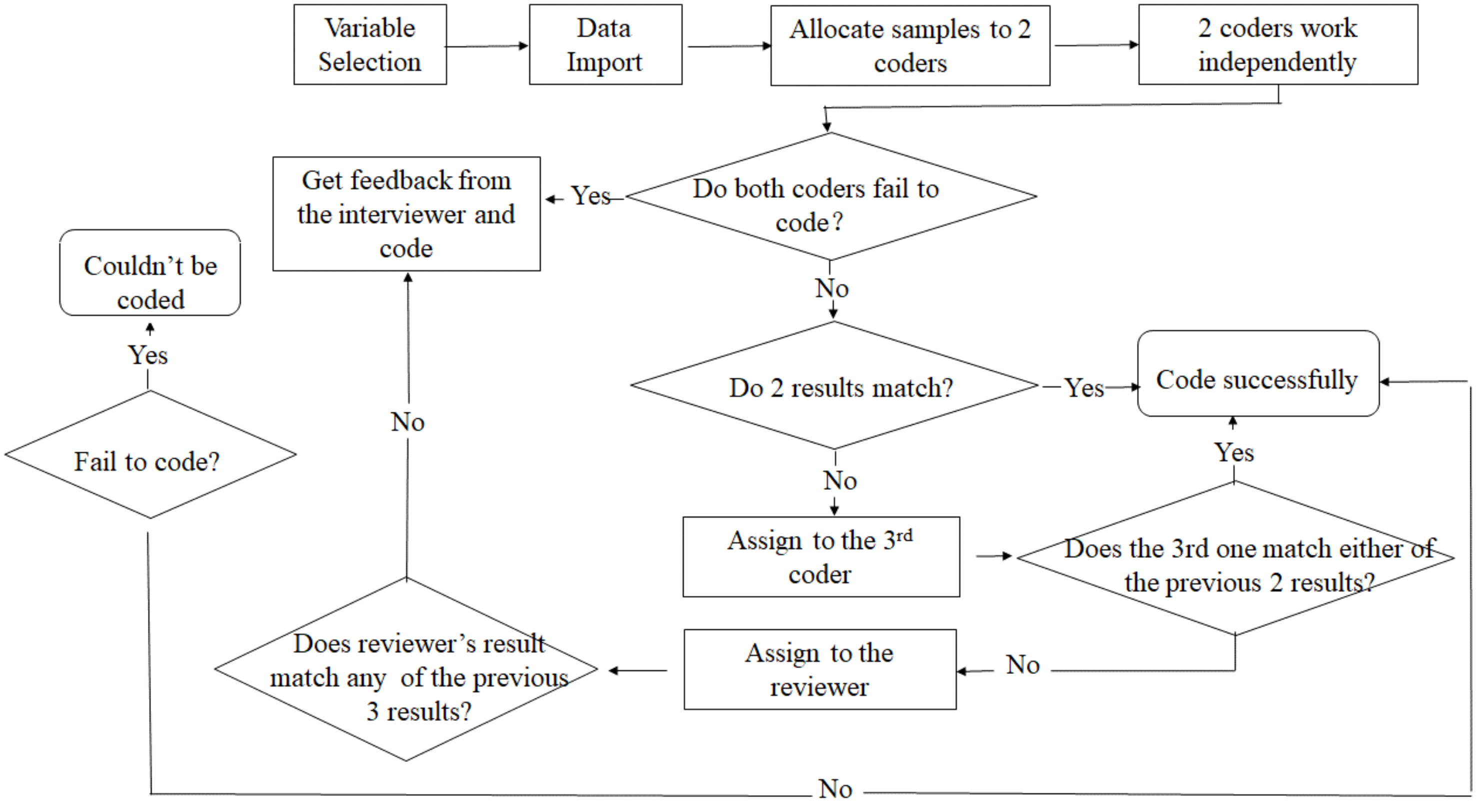


**Figure 1: The Flowchart of Coding**

# References

Bennett, D. A., J. A. Schneider, Z. Arvanitakis, and R. S. Wilson. 2012a. "Overview and Findings from the Religious Orders Study." *Curr Alzheimer Res* 9 (6):628–45.

Bennett, David, Julie Schneider, Aron Buchman, Lisa Barnes, Patricia Boyle, and Robert Wilson. 2012b. "Overview and Findings from the Rush Memory and Aging Project." *Current Alzheimer Research* 9 (6):646–663.

Brandt, Jason, Miriam Spencer, and Marshal Folstein. 1988. "The telephone interview for cognitive status." *J Neuropsychiatry Neuropsychol Behav Neurol* 1 (2):111–117.

Chan, T. S., L. C. Lam, H. F. Chiu, and M. Prince. 2003. "Validity and applicability of the Chinese version of community screening instrument for dementia." *Dement Geriatr Cogn Disord* 15 (1):10–8.

Chen, Xinxin, Eileen Crimmins, Peifeng Hu, Jung Ki Kim, Qinqin Meng, John Strauss, Yafeng Wang, Junxia Zeng, Yuan Zhang, and Yaohui Zhao. 2019. "Venous Blood-Based Biomarkers in the China Health and Retirement Longitudinal Study: Rationale, Design, and Results From the 2015 Wave." *American Journal of Epidemiology* 188 (11):1871–1877.

Crimmins, Eileen M, Jung Ki Kim, Kenneth M Langa, and David R Weir. 2011. "Assessment of cognition using surveys and neuropsychological assessment: the Health and Retirement Study and the Aging, Demographics, and Memory Study." *Journals of Gerontology Series B: Psychological Sciences and Social Sciences* 66 (suppl_1):i162–i171.

Crum, R. M., J. C. Anthony, S. S. Bassett, and M. F. Folstein. 1993. "Population-based norms for the Mini-Mental State Examination by age and educational level." *Jama* 269 (18):2386–91.

de Jager, C. A., M. M. Budge, and R. Clarke. 2003. "Utility of TICS-M for the assessment of cognitive function in older adults." *Int J Geriatr Psychiatry* 18 (4):318–24.

Erkinjuntti, T, Laura Hokkanen, R Sulkava, and J Palo. 1988. "The Blessed Dementia Scale as a screening test for dementia." *J International Journal of Geriatric Psychiatry* 3 (4):267–273.

Fillenbaum, G. G., G. van Belle, J. C. Morris, R. C. Mohs, S. S. Mirra, P. C. Davis, P. N. Tariot, J. M. Silverman, C. M. Clark, K. A. Welsh-Bohmer, and A. Heyman. 2008. "Consortium to Establish a Registry for Alzheimer's Disease (CERAD): the first twenty years." *Alzheimers Dement* 4 (2):96–109.

Fisher, GG, JJ McArdle, RJ McCammon, Amanda Sonnega, and DR Weir. 2013. "New measures of fluid intelligence in the HRS (HRS Documentation Report DR-027)." Report, Survey Research Center, University of Michigan.

Folstein, M. F., S. E. Folstein, and P. R. McHugh. 1975. ""Mini-mental state". A practical method for grading the cognitive state of patients for the clinician." *J Psychiatr Res* 12 (3):189–98.

Fuh, JL, EL Teng, KN Lin, EB Larson, SJ Wang, CY Liu, P Chou, BIT Kuo, and HC Liu. 1995. "The Informant Questionnaire on Cognitive Decline in the Elderly (IQCODE) as a screening tool for dementia for a predominantly illiterate Chinese population." *J Neurology* 45 (1):92–96.

Jorm, A. F. and P. A. Jacomb. 1989. "The Informant Questionnaire on Cognitive Decline in the Elderly (IQCODE): socio-demographic correlates, reliability, validity and some norms." *Psychol Med* 19 (4):1015–22.

Jorm, AF. 1994. "A short form of the Informant Questionnaire on Cognitive Decline in the Elderly (IQCODE): development and cross-validation." *J Psychological medicine* 24 (1):145–153.

Katzman, R., M. Y. Zhang, Qu Ouang Ya, Z. Y. Wang, W. T. Liu, E. Yu, S. C. Wong, D. P. Salmon, and I. Grant. 1988. "A Chinese version of the Mini-Mental State Examination; impact of illiteracy in a Shanghai dementia survey." *J Clin Epidemiol* 41 (10):971–8.

Lam, L. C., H. F. Chiu, S. W. Li, W. F. Chan, C. K. Chan, M. Wong, and K. O. Ng. 1997. "Screening for dementia: a preliminary study on the validity of the Chinese version of the Blessed-Roth Dementia Scale." *Int Psychogeriatr* 9 (1):39–46.

Lei, X., Y. Hu, J. J. McArdle, J. P. Smith, and Y. Zhao. 2012. "Gender Differences in Cognition among Older Adults in China." *J Hum Resour* 47 (4):951–971.

Lei, X., X. Sun, J. Strauss, P. Zhang, and Y. Zhao. 2014a. "Depressive symptoms and SES among the mid-aged and elderly in China: evidence from the China Health and Retirement Longitudinal Study national baseline." *Soc Sci Med* 120:224–32.

Lei, Xiaoyan, James P Smith, Xiaoting Sun, and Yaohui Zhao. 2014b. "Gender differences in cognition in China and reasons for change over time: evidence from CHARLS." *Journal of the Economics of Ageing* 4:46–55.

Li, G., Y. Shen, C. Chen, S. Li, W. Zhang, and M. Liu. 1989. "Test of mini-mental state examination in different population." *Chinese Mental Health Journal* 3 (4):148–151.

Li, G, Y Shen, C Chen, S Li, Y Zhao, M Liu, and Q Wang. 1988. “Study on the brief testing for dementia: testing MMSE among urban elderly.” *Chin Ment Health J* 2 (1):13–18.

Meng, Qinqin, Huali Wang, John Strauss, Kenneth M. Langa, Xinxin Chen, Mingwei Wang, Qiumin Qu, Wei Chen, Weihong Kuang, Nan Zhang, Tao Li, Yafeng Wang, and Yaohui Zhao. 2019. “Validation of neuropsychological tests for the China Health and Retirement Longitudinal Study Harmonized Cognitive Assessment Protocol.” *International Psychogeriatrics* 31 (12):1709–1719.

Ofstedal, MB, GG Fisher, and AR Herzog. 2005. “Documentation of cognitive functioning measures in the health and retirement study.” Report, University of Michigan.

Prince, M., D. Acosta, C. P. Ferri, M. Guerra, Y. Huang, K. S. Jacob, J. J. Llibre Rodriguez, A. Salas, A. L. Sosa, J. D. Williams, and K. S. Hall. 2011. “A brief dementia screener suitable for use by non-specialists in resource poor settings–the cross-cultural derivation and validation of the brief Community Screening Instrument for Dementia.” *Int J Geriatr Psychiatry* 26 (9):899–907.

Prindle, John and John McArdle. 2013. “Number series abilities in the Mexico and Indonesia Sample.” Report, Department of Psychology, University of Southern California.

Smith, James P, Meng Tian, and Yaohui Zhao. 2013. “Community effects on elderly health: evidence from CHARLS National Baseline.” *The journal of the economics of ageing* 1:50–59.

Strauss, John, Firman Witoelar, Qinqin Meng, Xinxin Chen, Zhao Yaohui, Bondan Sikoki, and Yafeng Wang. 2018. “Cognition and SES Relationships Among the Mid-Aged and Elderly: A Comparison of China and Indonesia.” *NBER Working Paper* (24583).

United Nations, Population Division, Department of Economic and Social Affairs. 2019. “World Population Prospects: The 2019 Revision.” .

Wang, Heng, Xin-qing Zhang, Zhe Tang, Jing-sheng Zhou, and Hou-liang Sun. 2006. “Application of informant questionnaire on cognitive decline in the elderly in the screening of cognitive impairment in the elderly.” *Chinese Journal of Geriatrics* 25 (05):386–388.

Weir, R., D., L. Ryan McCammon, and K. Langa. 2014. “Cognitive Test Selection for

the Global Aging Studies Protocol." Report, Survey Research Center, University of Michigan.

Yang, G., Y. Wang, Y. Zeng, G. F. Gao, X. Liang, M. Zhou, X. Wan, S. Yu, Y. Jiang, M. Naghavi, T. Vos, H. Wang, A. D. Lopez, and C. J. Murray. 2013. "Rapid health transition in China, 1990-2010: findings from the Global Burden of Disease Study 2010." *Lancet* 381 (9882):1987–2015.

Yi, Zeng and James W Vaupel. 2002. "Functional capacity and self–evaluation of health and life of oldest old in China." *Journal of Social Issues* 58 (4):733–748.

Zhang, M. Y., R. Katzman, D. Salmon, H. Jin, G. J. Cai, Z. Y. Wang, G. Y. Qu, I. Grant, E. Yu, P. Levy, and et al. 1990. "The prevalence of dementia and Alzheimer's disease in Shanghai, China: impact of age, gender, and education." *Ann Neurol* 27 (4):428–37.

Zhao, Yaohui, Yisong Hu, James P Smith, John Strauss, and Gonghuan Yang. 2014a. "Cohort Profile: The China Health and Retirement Longitudinal Study (CHARLS)." *Int J Epidemiol* 43 (1):61–8.

Zhao, Yaohui, Crimmins E. M., Peifeng (Perry) Hu, Yisong Hu, Tao Ge, Jung Ki Kim, John Strauss, Gonghuan Yang, Xiangjun Yin, and Yafeng Wang. 2014b. "China Health and Retirement Longitudinal Study 2011–2012 National Baseline Blood Data User's Guide." Tech. rep., National School of Development, Peking University.

Zhao, Yaohui, John Strauss, Gonghuan Yang, John Giles, Peifeng (Perry) Hu, Yisong Hu, Xiaoyan Lei, Man Liu, Albert Park, James P. Smith, and Yafeng Wang. 2013. "China Health and Retirement Longitudinal Study: 2011-2012 National Baseline User's Guide." Tech. rep., National School of Development, Peking University.
